# Dynamics of single-nuclei transcriptomic profiling of adipose tissue from diverse anatomical locations during mouse aging process

**DOI:** 10.1038/s41598-024-66918-w

**Published:** 2024-07-12

**Authors:** Yujie Wu, Ying Sun, Long Chen, Xingyan Tong, Can Liu, Lu Lu, Rui Zhang, Siyuan Wang, Ziyu Chen, Jiaman Zhang, Ziyin Han, Bo Zeng, Mingzhou Li, Long Jin

**Affiliations:** 1 Swine and Poultry Breeding Industry, College of Animal Science and Technology, Sichuan Agricultural University, Chengdu, 611130 China; 2 Department of Geriatics, Sichuan Provincial People’s Hospital, School of Medicine, University of Electronic Science and Technology of China, Chengdu, 611130 China

**Keywords:** Molecular biology, Transcriptomics

## Abstract

Adipose tissue plays critical roles in an individual’s aging process. In this research, we use single-nucleus RNA sequencing to create highly detailed transcriptional maps of subcutaneous adipose tissue and visceral adipose tissue in young and aged mice. We comprehensively identify the various cell types within the white adipose tissue of mice, our study has elucidated seven distinct cell types within this tissue. Further analyses focus on adipocytes, fibro-adipogenic progenitors, and immune cells, revealing age-related declines in the synthetic metabolic activity of adipocytes, diminished immune regulation, and reduced maturation or proliferation of fibroblasts in undifferentiated adipocytes. We confirm the presence of distinct subpopulations of adipocytes, highlighting decreases in adipogenesis subgroups due to aging. Additionally, we uncover a reduction in immune cell subpopulations, driven by age-associated immune system dysregulation. Furthermore, pseudo-time analyses indicate that Adipocyte1 represents the 'nascent' phase of adipocyte development, while Adipocyte2 represents the 'mature' phase. We use cell–cell interaction to explore the age-dependent complexities of the interactions between FAPs and adipocytes, and observed increased expression of the inflammation-related Retn-Tlr4 interaction in older mice, while the anti-inflammatory Angpt1-Tek interaction was only detected in young mice. These transcriptional profiles serve as a valuable resource for understanding the functional genomics underlying metabolic disorders associated with aging in human adipose tissue.

## Introduction

Adipose tissue is versatile, serving as an energy reservoir, protective insulator, and crucial endocrine communicator, interacting with other tissues to regulate systemic metabolism^1^. Adipose tissue is categorized into two primary types: brown adipose tissue (BAT) and white adipose tissue (WAT). White adipose tissue (WAT) is broadly categorized into subcutaneous adipose tissue (SAT) and visceral adipose tissue (VAT)^2^. SAT is located beneath the skin, distinct from VAT, which is found around internal organs^3^. Inguinal and gonadal fat pads in mice are often considered representative of SAT and VAT, respectively, due to their accessibility, prevalence among white adipose depots, and ease of identification.

Aging is closely linked to metabolic decline^4^, which is associated with a range of lipid metabolism-related conditions, including diabetes mellitus^5^, hyperlipidemia^6^, and atherosclerosis^7^. Fibro-adipogenic progenitors (FAPs) make up a diverse group of stem cells, preadipocytes, and fibroblasts^8^. The initial transition of mesenchymal stem cells (MSCs) into the adipogenic realm marks the beginning of adipogenesis—a process that still requires in-depth exploration, especially in the context of transcriptional regulation during adipose tissue aging^9^. Recent studies highlight the pivotal role of adipose tissue’s immune cells in metabolism, with aging in adipose tissue causing WAT hypoxia, which in turn initiates inflammation and metabolic irregularities^10,11^. A comprehensive understanding of changes in WAT cell types during aging is crucial to unravel the cellular and molecular mechanisms behind aging-related comorbidities.

Recent advancements in single-cell transcriptomics have opened doors to understanding cellular diversity and functional states at an individual cell level. These methods offer remarkable reproducibility and sensitivity^12^. However, due to the challenges posed by large lipid-filled adipocytes and the limited availability of single-cell resolution characterization of WAT, a new approach has been adopted—using single-nucleus sequencing (snRNA-seq) on isolated nuclei from adipocytes to conduct transcriptional profiling^13^. snRNA-seq offers a comprehensive view of WAT cell types in their natural environment, bypassing the potential loss of essential cell types associated with manual tissue isolation procedures^14^. Analyzing adipocytes, the primary functional cell type, presents unique challenges due to their substantial size and delicacy^15^, which can result in a lack of adipocyte data in snRNA-seq datasets^16^.

In this study, we employed snRNA-seq to shed light on the cell types present in inguinal and gonadal adipose tissue in mice. This approach, involving different age groups, provided a unique perspective to explore the intricate cellular architecture and variances in adipose tissue across diverse anatomical regions in mice. Furthermore, it offered profound insights into the complex interplay of cellular mechanisms governing the regulation of white adipose tissue as it ages.

## Results

### Single-nucleus RNA sequencing uncovers the full spectrum of cell types in mouse SAT and VAT

**Figure 1:**
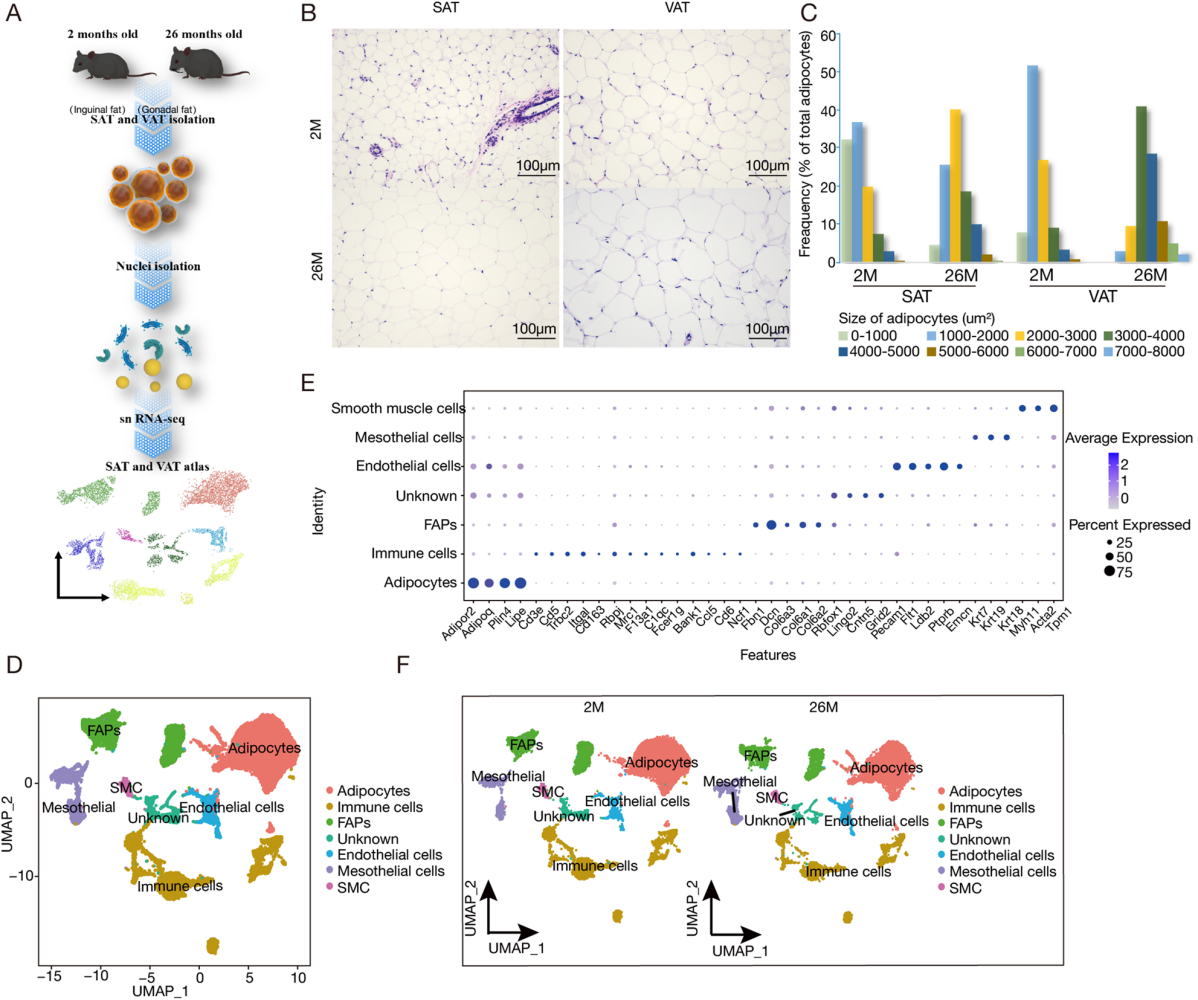
snRNA-Seq of young and old white adipose tissue. (**A**) Workflow Overview: We conducted single-nucleus RNA sequencing (snRNA-seq) on nuclei isolated from combined subcutaneous (inguinal) and visceral (gonadal) adipose tissues of both young (2 months old) and old (26 months old) mice (n = 2 for each age group). (**B**) Representative sections of white adipose tissue (WAT) stained with Hematoxylin and Eosin (H&E), including SAT and VAT from both young and aged mice (scale bars indicate 100 μm). The figure on the right shows the analysis of the size and frequency distribution of adipocytes, for each site and age group, we randomly selected three tissue sections and measured the area of 241 adipocytes, which are depicted in this figure. (**C**) Adipocyte size distribution in young vs. aged mice (**D**) Uniform manifold approximation and projection (UMAP) representation of WAT cell types, covering both SAT and VAT. The embedding uesd the 1000 most variable genes and the initial 15 harmonized principal components. Clusters were delineated according to this UMAP representation. (**E**) Representation of normalized gene expression values in a bubble diagram, depicting the adipose lineage, endothelial cells, immune cells, mesothelial cells, and smooth muscle cells. (**F**) UMAP representations of cell types within SAT and VAT in 2-month-old and 26-month-old specimens.

To comprehensively profile WAT across ages, we used single-nucleus RNA sequencing (snRNA-seq) on SAT and VAT adipose tissues from young (2-month-old) and aged (26-month-old) female C57BL/6 J mice. Firstly, we used hematoxylin and eosin (H&E) staining to elucidate age-dependent changes in adipocytes. As expected, both SAT and VAT from 26-month-old mice exhibited larger adipose areas compared to their 2-month-old counterparts (Figs. 1B,C, S1E). These observations underscore that aging leads to a substantial expansion in the area of both SAT and VAT in mice.

Then, we specifically collected subcutaneous (inguinal) and visceral (gonadal) adipose tissues from two mice within each age group for snRNA-Seq analysis (Fig. 1A). Following stringent quality filtration, we combined these eight datasets into a consolidated dataset for further analysis. Subsequent integrative analysis of the cell cycle revealed harmonized data across G1, G2M, and S phases (Fig. S1A,B). The integrated and clustered whole WAT sample yielded seven distinct clusters (Fig. 1D) within the transcriptional profiles of 35,611 nuclei (Table S1). These clusters included adipocytes (expressing *Adipor2*, *Adipoq*, and *Plin4*), immune cells (expressing *Trbc2*, *Rbpj*, and *Bank1*), preadipocytes (expressing *Fbn1*, *Dcn*, and *Col6a1*), endothelial cells (expressing *Pecam1*, *Flt1*, and *Ptpr*), mesothelial cells (expressing *Krt7*, *Krt19*, and *Krt18*), smooth muscle cells (expressing *Myh11*, *Acta2*, and *Tpm1*), and one cluster that remained unidentified following differential gene expression and pathway analyses, annotated as UNKNOWN (Fig. 1E). Upon clustering at two age intervals with respect to SAT and VAT (Figs. 1F, S1C,D), it was observed that aging did not lead to the disappearance of cell types, but differences were evident between the two.

### Age-linked alterations in specific cell types

**Figure 2:**
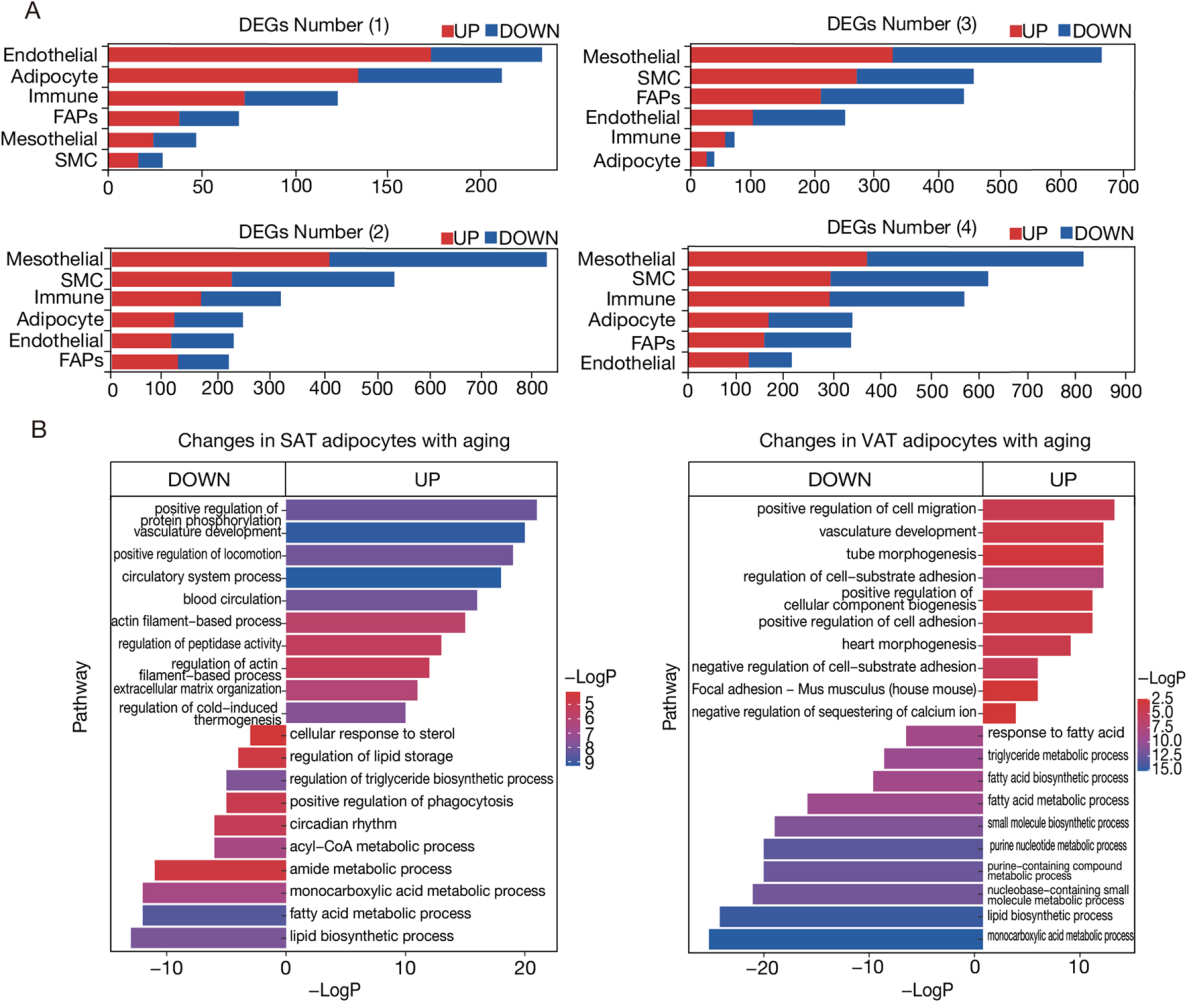
Aging leads to a decline in adipocyte synthesis and metabolic capacity. (**A**) This investigation encompassed four specific comparisons: (1) Aging patterns within SAT tissues (referred to as “SAT Aging”), (2) Aging patterns within VAT tissues (“VAT Aging”), (3) comparisons between SAT and VAT tissues at the young age of 26 months, and (4) comparisons between SAT and VAT tissues at the elderly age of 2 months. Under these four comparisons, we analyzed the number of differential genes. (**B**) Adipocytes in SAT and VAT underwent KEGG and GO enrichment analyses at both 2 and 26 months of age, respectively.

By integrating and clustering the samples of WAT, we determined the proportions of nuclei for each distinct cell type (Fig. S2A). Remarkably, while aging introduced variations, no specific cell type completely disappeared across the age spectrum. To uncover the variations among cell types in adipose tissues at different aging stages, we initiated a functional-specific enrichment analysis of the cell populations we obtained. (Figs. 2A, S2D–J, Table S2).

Further transcriptomic analysis revealed molecular distinctions between SAT and VAT during the aging process (Fig. S2B), At 26 months of age, SAT displayed downregulation in 63 genes, while VAT showed downregulation in 114 genes. Upon a comprehensive assessment of the relationship between age and anatomical sites, we observed that the correlation between age and specific sites was notably stronger than that between SAT and VAT. This robust evidence underscores the prominent influence of aging on adipose tissue alterations compared to intrinsic variations within tissues (Fig. S2C).

Next, we conducted a functional enrichment analysis to map the age-related variation of SAT and VAT. Interestingly, aging adipocytes in SAT exhibited upregulation of genes associated with circulatory and vascular system development. In contrast, pathways linked to fatty acid metabolism and lipid biosynthesis displayed a downward trend at 26 months of age. Additionally, VAT adipocytes upregulated genes controlling cellular matrix adhesion and positive regulation of cell migration while downregulating those governing monocarboxylic acid metabolism, lipid biosynthesis, and purine nucleotide metabolism (Fig. 2B). The immune cell compartment offered further insights into the aging process. SAT immune cells at 26 months exhibited increased regulation of myeloid differentiation, type II interferon production, and inflammatory response, while pathways associated with adaptive responses and lymphocyte value-addition lagged behind. VAT, in contrast, amplified T-cell and B-cell activation but restrained its regulation of the inflammatory response (Fig. S2D). This dichotomy in cellular responses extended to the preadipocyte (FAPs) population. In SAT, the regulation of angiogenesis increased while positive T-cell selection and B-cell value-addition decreased at 26 months. Concurrently, VAT preadipocytes showcased increased activity in energy-coupled transmembrane transport pathways but a reduced emphasis on intercellular adhesion and prominent organization (Fig. S2F–H).

### Aging causes changes in the states of mesothelial cell and endothelial cell populations

To understand how mesothelial and endothelial cells in adipose tissue change during aging, we analyzed these cells in detail. Endothelial cells are key for blood vessel formation in fat tissue^17^, while mesothelial cells can become fat cells, affecting fat tissue growth^18^. Our study focused on how these cell types change and function differently as they age.

**Figure 3:**
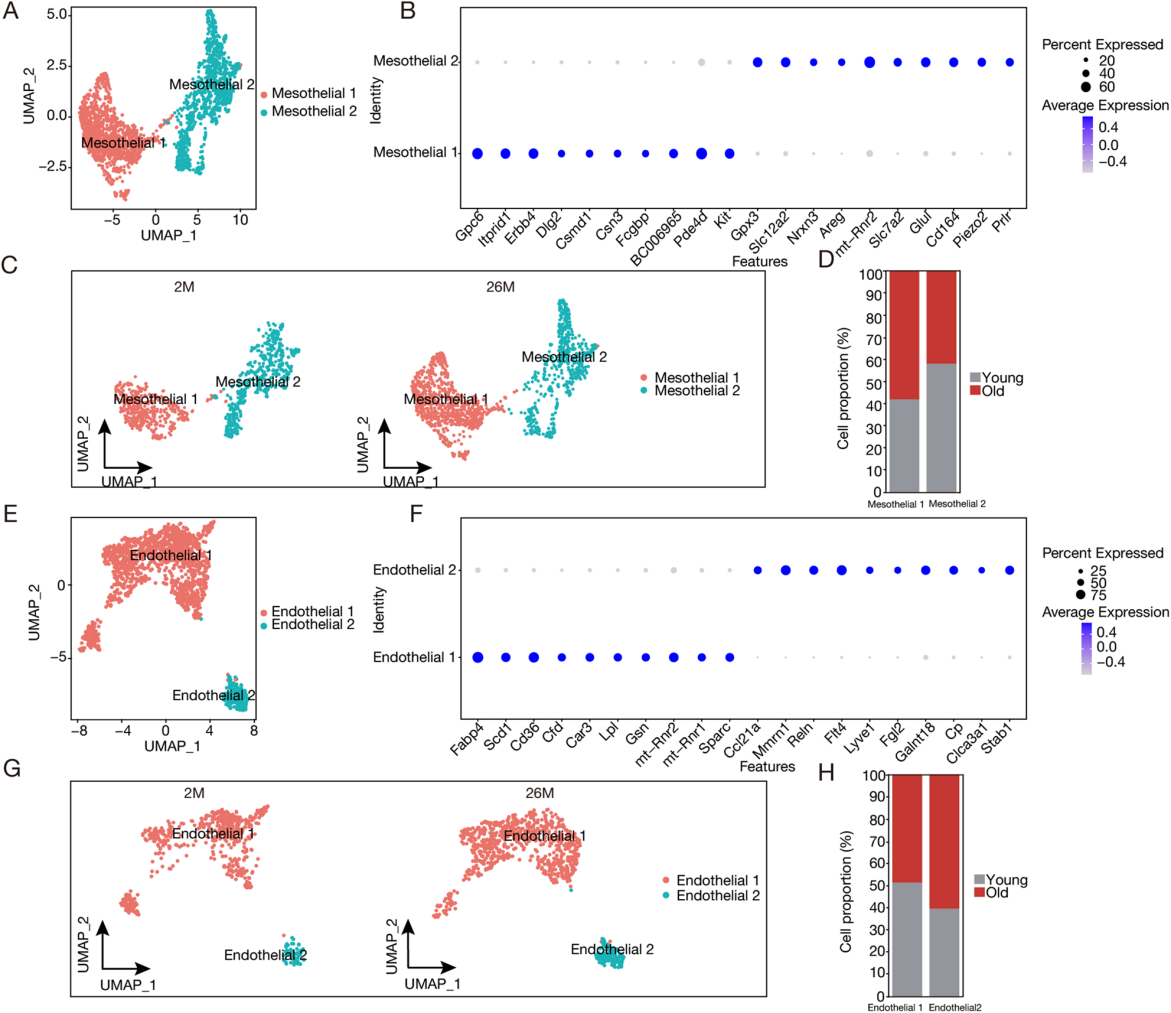
Aging Changes the mesothelial cell and endothelial cell proportion in white adipose tissue. (**A**) Uniform manifold approximation and projection (UMAP) visualization of mesothelial subpopulations. (**B**) Representation of normalized gene expression values as a bubble diagram, illustrating Mesothelial1 and Mesothelial2. (**C**) UMAP representations of cellular subtypes within mesothelial cell populations in 2-month-old and 26-month-old mice. (**D**) Chart of mesothelial cell subgroup proportions during aging. (**E**) UMAP depiction of endothelial subpopulations. (**F**) Bubble diagram displaying normalized gene expression values for Endothelial1 and Endothelial2. (**G**) UMAP representations of cellular subtypes within endothelial cell populations in 2-month-old and 26-month-old mice. (**H**) Chart of endothelial cell subgroup proportions during aging.

Following the re-clustering analysis of mesothelial cell nuclei (n = 2441), we divided them into two distinct subpopulations (Fig. 3A,C, Table S3-1). A deep examination of differential gene expression and pathways enabled us to annotate one subpopulation as “Mesothelial1” characterized by the expression of genes including *Gpc6*, *Erbb4*, and *Pde4d* (n = 1277). These genes play roles in receptor-linked protein signaling pathways, influencing various biological processes, including cell differentiation, proliferation, and signal transduction. We identified the other subpopulation as “Mesothelial2” (n = 1134), which primarily expresses genes like *Cd164*, *Slc7a2*, and *Areg*, implicated in cytoplasmic translation, RNA splicing, messenger RNA synthesis, and regulation of the extracellular matrix and supramolecular fiber organization (Fig. 3B). In addition, we compared mesothelial subpopulation cell numbers in 2-month-old versus 26-month-old mice, observing an elevation in the proportion of Mesothelial1 cells and a reduction in Mesothelial2 cells alongside aging (Fig. 3D). We identified a higher number of mesothelial cell subpopulations in VAT relative to SAT (Fig. S3A–D).

Ultimately, upon an extensive re-clustering of 1509 endothelial cell nuclei, we segregated them into two unique subpopulations (Fig. 3E,G and Table S3-2). Incorporating differential gene expression and pathway analyses, we identified a subpopulation we named “Endothelial1” (n = 1326), which expresses genes such as *Fabp4*, *Scd1*, and *Cd36*, all of which are active in lipid biosynthesis. Conversely, the “Endothelial2” subpopulation was identified by the expression of *Ccl21a*, *Mmrn1*, and *Flt4* (n = 183), which are crucial for lymphatic and vascular development (Fig. 3F). Intriguingly, by comparing the endothelial subpopulation distribution in 2-month-old versus 26-month-old mice, we observed that with increasing age, Endothelial1 increases while Endothelial2 decreases (Fig. 3H). Additionally, compared to SAT, we identified a higher number of Endothelial2 subpopulations in VAT (Fig. S3C,D).

### Age-driven modulations in immune cell subpopulations

Aging profoundly influences the immune landscape, inducing alterations in the distribution and functionality of diverse immune cell subpopulations. Comprehending these variations is critical for elucidating the age-associated vulnerabilities and therapeutic targets. SAT and VAT have emerged as critical dysfunction nodes during aging, contributing notably to pathophysiological shifts^19^. Delving into the intricacies of immune cell malfunctions within these adipose tissues may enrich our understanding of age-associated immune-metabolic deviations.

**Figure 4:**
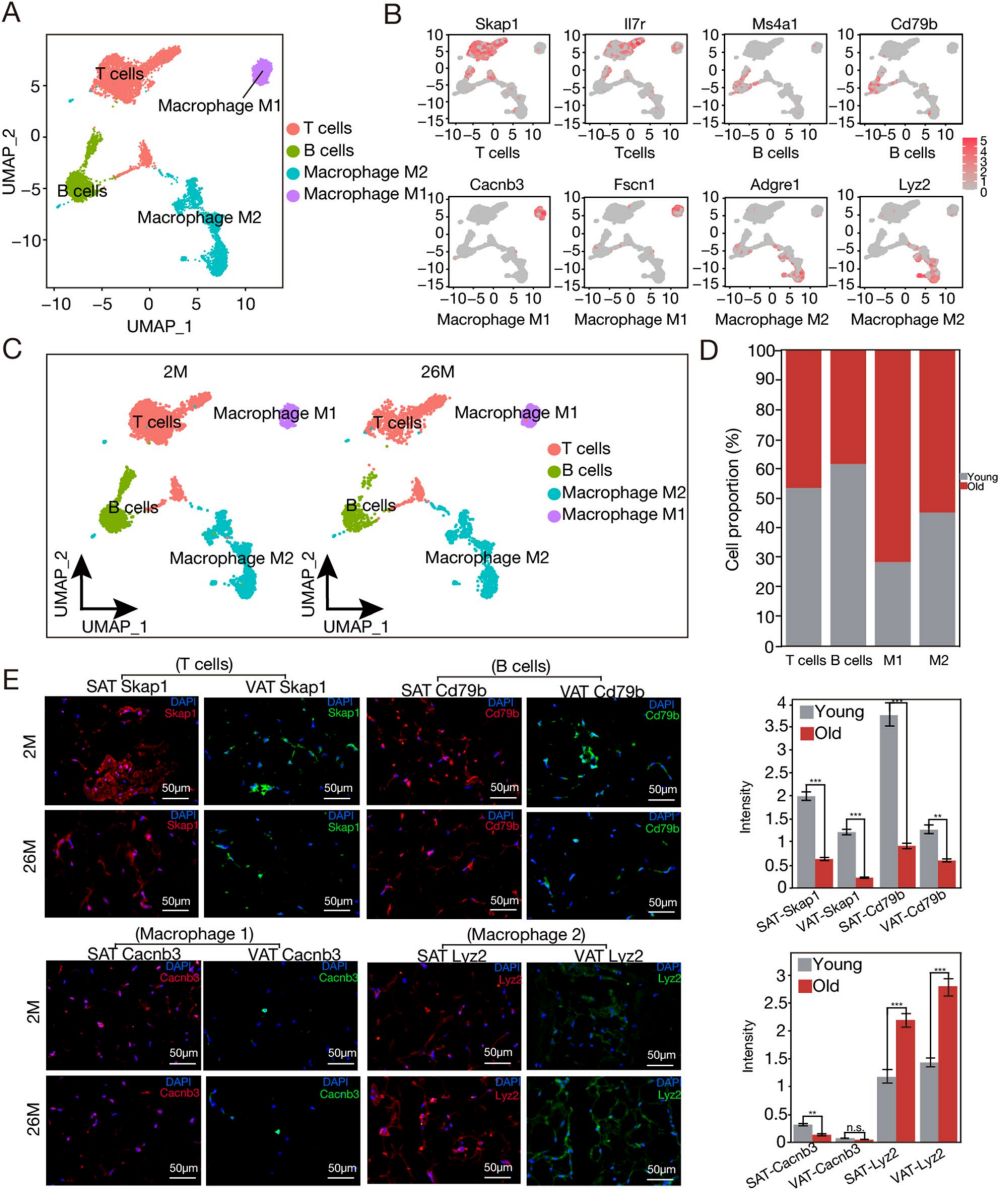
Aging results in a reduction of immune cell subtypes. (**A**) Uniform manifold approximation and projection (UMAP) of immune subpopulations. The embedding depends on the 1000 most variable genes, and the first 16 harmonized principal components present. Cluster characterization was executed based on the UMAP embedding. (**B**) Identification of marker genes within immune subpopulations. Representation of an analysis of B cells, T cells, M1 macrophages, and M2 macrophages. (**C**) UMAP depiction of cellular subtypes within immune cell populations in specimens at 2 months and 26 months. (**D**) Chart of immune cell subgroup proportions during aging. (**E**) Immunofluorescence staining-based identification of distinct immune cell subsets. *Cd79b* acted as a proxy for B cells, *Skap1* indicated T cells, *Cacnb3* represented M1 macrophages, and *Lyz2* signified M2 macrophages. Within this characterization, SAT was indicated in red, while VAT was denoted in green. Comparative analyses were performed based on fluorescence intensity and the abundance of these markers (scale bars represent 50 μm). Right figure shows the intensity of immunofluorescence.

In our examination, immune cells in adipose tissues were methodically clustered. From an initial assembly of immune cell-origin nuclei (n = 8850), re-clustering identified four prominent subpopulations (Table S4). Via a comprehensive differential expression and pathway assessment, we were able to annotate these clusters: two were designated as macrophage subtypes known as M1 (marked by genes including *Cacnb3* and *Fscn1*, n = 779) and M2 (expressing *Adgre1*, *Lyz2*, and *Ccl6*, n = 2208), while the other two subtypes denoted T cells (*Skap1* and *ll7r*, n = 4126) and B cells (*Ms4a1* and *Cd79b*, n = 1737) (Fig. 4A,B). In the older cohort, the proportion of M1 increased from 6.1 to 15.4%. The proportion of M2 increased from 23.4 to 28.5% (Fig. 4C,D). Clearly, both B and T cell proportions dwindled alongside advancing age (Fig. 4E). A corresponding exploration comparing SAT and VAT uncovered inherent differences in immune cell composition between these fat reservoirs. VAT exhibited a preponderance of M2 macrophages and a paucity of M1 macrophages compared to SAT (Fig. S4A). Corroborating this, both B and T cell fractions were substantially diminished in VAT compared to SAT (Fig. S4A-B). Subsequently, immunofluorescence was used for further validation. *Skap1* was identified as a marker for T cells^20^, *Cd79b* for B cells^21^, *Cacnb3* for M1 macrophages, and *Lyz2* for M2 macrophages (Fig. 4E).

### Age-associated diminution in FAPs subpopulations

Given the intrinsic capacity of fibro-adipogenic progenitors (FAPs) to differentiate into adipocytes and their pivotal role in lipid biosynthesis and metabolism, we conducted a refined re-clustering analysis of FAPs, aimed at elucidating their nuanced functionalities within adipose tissue dynamics during aging.

**Figure 5:**
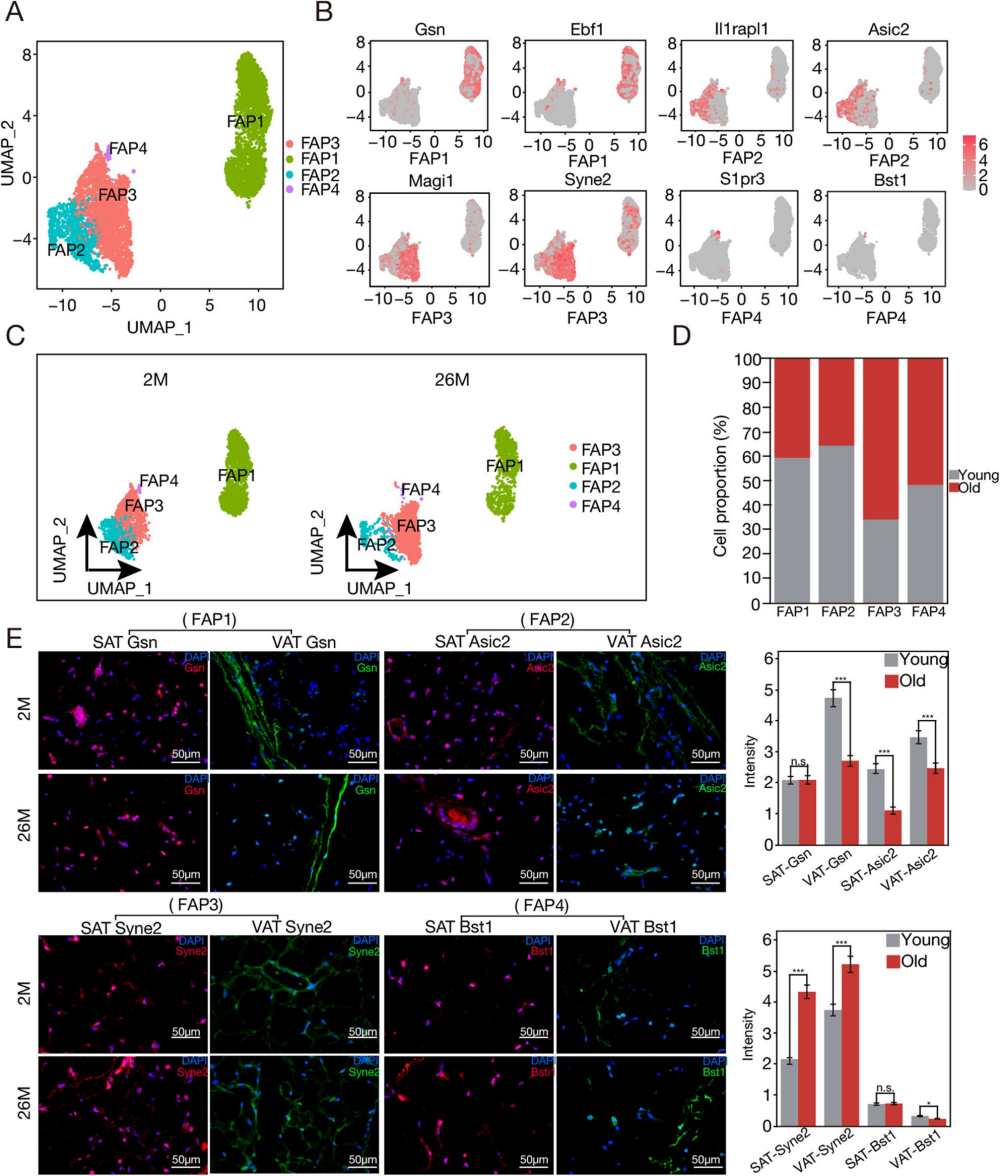
Aging leads to a change in FAPs subpopulations. (**A**) Uniform Manifold Approximation and Projection (UMAP) representation of fibro-adipogenic progenitor (FAP) subpopulations. The embedding is established based on the 1000 most variable genes and the primary 16 harmonized principal components. Clustering was performed according to the UMAP embedding. (**B**) Definition of marker genes within FAP Subpopulations. A detailed examination of FAP1, FAP2, FAP3, and FAP4. (**C**) UMAP illustrations of cellular subtypes within FAP populations in 2-month-old and 26-month-old specimens. (**D**) Chart of FAPs subgroup proportions during aging. (**E**) Using immunofluorescence staining, distinct subpopulations of FAPs were identified. *Gsn* was indicative of FAP1, *Asic2* represented FAP2, *Syne2* identified FAP3, and *Bst1* signified FAP4. Within this framework, SAT was identified in red, while VAT was defined in green. Analyses were conducted according to both fluorescence intensity and the relative abundance of these markers (scale bars represent 50 μm). Right figure shows the quantification of immunofluorescence.

The reclassification of FAPs (n = 6024) produced four discrete subsets (Table S5). Initial characterization of these cohorts was achieved via differential gene expression and pathway assessments, resulting in their annotation as FAP1 through FAP4. FAP1 is identified by the expression of preadipocyte/adipose stem cell identifiers (including *Gsn* and *Ebf1*, n = 2498). FAP2 exhibits advanced preadipocyte characteristics (exemplified by the expression of *II1rapI1* and *Asic2*, n = 1090), suggesting that FAP2 indicates bona fide preadipocytes. FAP3 encompasses cells expressing genes linked to fibrosis and extracellular matrix accumulation (indicated by expressions of genes such as *Magi1* and *Syne2*, n = 2336). Finally, FAP4 comprises a substantial number of cells involved in adaptive immunity (marked by the expression of genes including *S1pr3* and *Bst1*, n = 100) (Fig. 5A,B). Intriguingly, when contrasting the preadipocyte subpopulation compositions across the 2-month and 26-month time points, we identified that aging prompted an augmented relative number of FAP3. Conversely, the proportions of FAP1 and FAP2 underwent clear reductions, while the ratio of FAP4 remained generally stable (Figs. 5C,D, S5A,B). Furthermore, when investigating the preadipocyte distribution over both SAT and VAT locales, distinctions in adiposity conditions became clear. Notably, within VAT, there was a pronounced increase in the proportions of FAP2, and FAP3 compared to their SAT counterparts (Fig. S5A). Subsequently, immunofluorescence was used for further validation. SAT was marked in red, while VAT was identified in green, consistent with the snRNA-seq findings (Fig. 5E).

### Mouse SAT and VAT harbor diverse adipose tissue subpopulations

In fat tissue, fat cells have important jobs like storing energy, releasing hormones, controlling inflammation and the immune system, and keeping the body’s structure stable. As we get older, these cells start to change in how they store energy and work. To better understand these changes as mice age, we studied fat cells closely and looked at how they adapt during the aging process.

**Figure 6:**
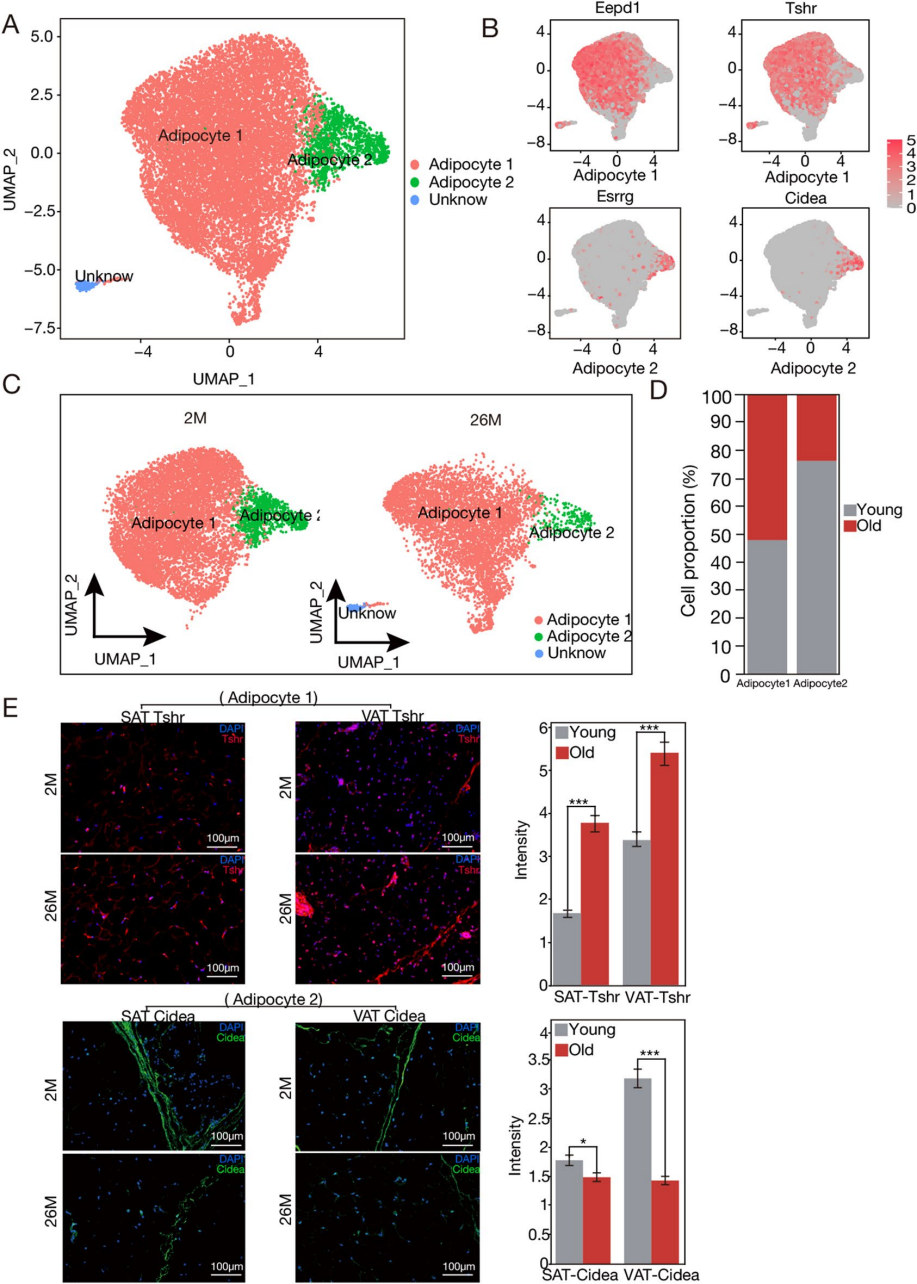
Adipocyte subpopulations in white adipose tissue are changed by aging. (**A**) Uniform manifold approximation and projection (UMAP) visualization of adipocyte subpopulations. The embedding is based on the 1000 most variable genes and the first 16 harmonized principal components. Clustering was conducted using the UMAP embedding. (**B**) Description of marker genes within adipocyte subpopulations. Detailed examination of Adipocyte1 and Adipocyte2 subpopulations. (**C**) UMAP illustrations of cellular subtypes within adipocyte populations in 2-month-old and 26-month-old specimens. (**D**) Chart of adipocytes subgroup proportions during aging. (**E**) Adipocyte subgroups Adipocyte1 and Adipocyte2 were identified through immunofluorescence staining. *Tshr* (identified in red) represents Adipocyte1, whereas *Cidea* (marked in green) represents Adipocyte2. Comparative analysis was performed according to the fluorescence intensity and abundance of the markers. The left image displays *Tshr* immunofluorescence in SAT and VAT, while the right image illustrates *Cidea* immunofluorescence in SAT and VAT (scale bars represent 100 μm). Right figure shows the quantification of immunofluorescence.

Adipocytes constituted the first large population of nuclei (n = 14,524), which were divided into two distinct subpopulations (Table S6), all of which expressed significantly elevated levels of classical adipocyte markers, including *Adipor2*, *Adipoq*, and *Plin4* compared to the remaining nuclei (Fig. 6A,B). These subpopulations were uncovered via meticulous differential gene expression and pathway exploration. The “Adipocyte1” cohort, defined by factors such as *Eepd1* and *Tshr* (n = 13,318), was linked to RNA editing, DNA transcription, and mRNA metabolism, and appears in the nascent phase of adipogenesis. At this juncture, essential molecular activities such as DNA replication, transcription, and translation become pivotal, orchestrating foundational steps for adipogenesis, thus, we define 'Adipocyte1' as nascent. Contrastingly, the “Adipocyte2” cluster mirrors mature adipocytes (n = 1163). Their transcriptomic landscape, enriched in genes pivotal to lipid biosynthesis, the TCA cycle, glucagon responsiveness, and adipocyte-specific transcription regulators (e.g., *Cidea*, *Acacb*, *Ppara*), identifying them as glucagon-sensitive entities adept at de novo adipogenesis, hence, we define ‘Adipocyte2’ as mature. When comparing the compositions of SAT and VAT adipocytes across different ages, it can be observed that the proportion of Adipocyte1 increases with age, while the proportion of Adipocyte2 decreases as age progresses (Figs. 6C,D, S6A,B).

To corroborate the age-associated diversity within distinct adipocyte subsets, we performed staining of SAT and VAT in mice. Followed by quantification of the relative abundance of thyrotropin hormone receptor (*Tshr*) and cell death-inducing DFFA-like effector a (*Cidea*) using immunofluorescence microscopy. Our snRNA-seq data indicate active transcription of the *Tshr* gene in adipocytes, suggesting a potential role for *Tshr* in these cells. Further experiments are necessary to precisely define *Tshr* protein’s localization and function. Research implies *Tshr’s* involvement in triglyceride synthesis regulation via glycerophosphoryl transferase 3^22^, highlighting its significant impact on adipocyte growth, differentiation, and various secretory functions, thereby playing a crucial role in fatty acid synthesis. *Cidea*, localized on lipid droplet surfaces, the endoplasmic reticulum, and the nucleus, has been found to interact with AMPK’s beta subunit, enabling its ubiquitination and degradation. In *Cidea* knockout mice, AMPK protein levels and enzymatic activity were elevated, promoting fatty acid beta-oxidation. Therefore, *Cidea* plays a critical role in lipolysis. *Tshr* and *Cidea* were distinct markers for Adipocyte1 and Adipocyte2 subpopulations, respectively, in our research. Corresponding to snRNA-seq observations, SAT and VAT from aged mice generally displayed limited relative fluorescence signals for both *Tshr* and *Cidea*. Conversely, young mice exhibited high relative fluorescence signals for both markers within SAT and VAT (Fig. 6E).

### Predicting the transcriptional trajectories among adipocyte subgroups in vivo during aging using snRNA-seq

**Figure 7:**
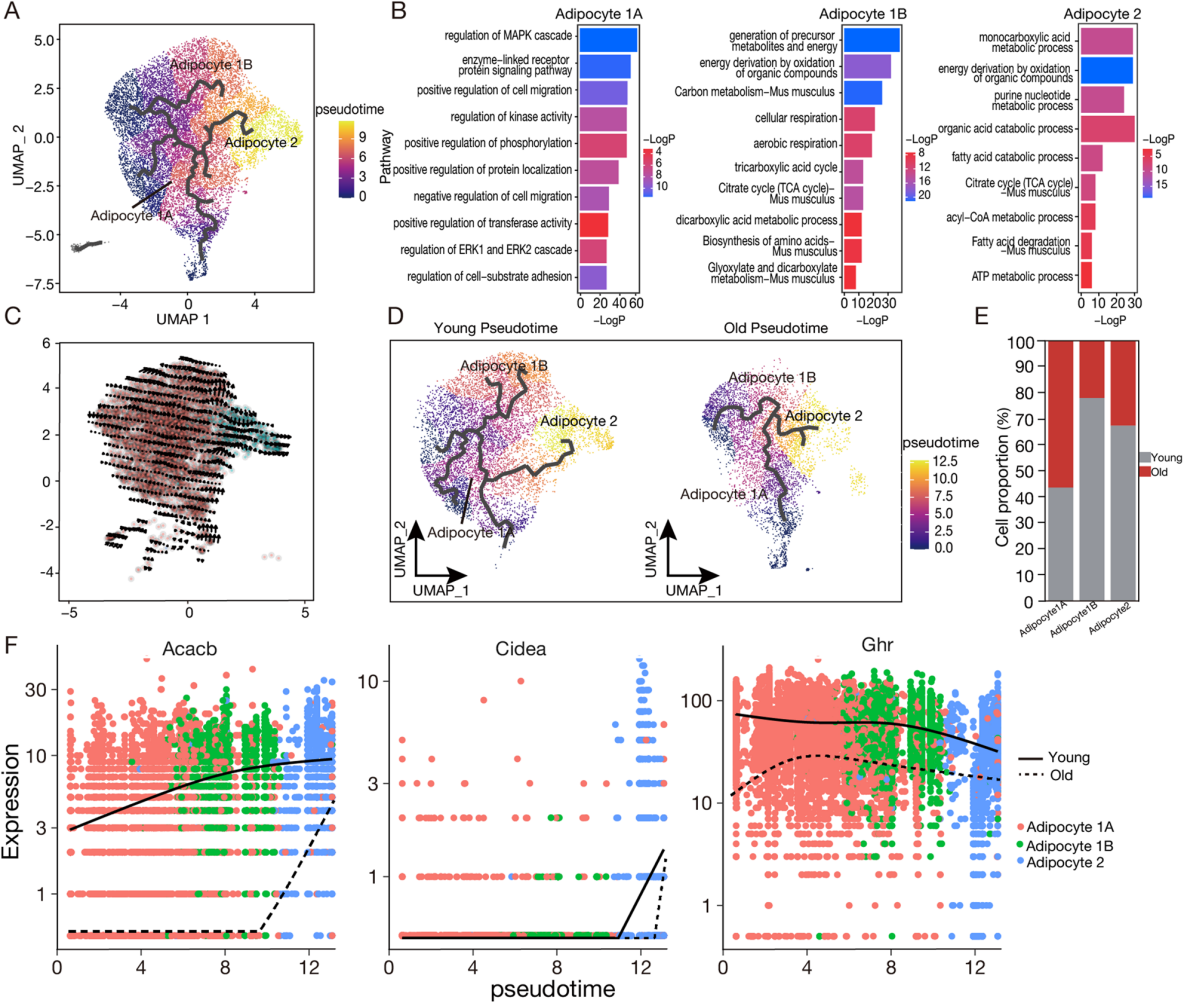
Reconstruction of adipogenesis in vivo. (**A**) UMAP visualization of the pseudotemporal trajectory of adipocyte subpopulations. (**B**) Functional enrichment analysis of Adipocyte1-A, Adipocyte1-B, and Adipocyte2. (**C**) UMAP representation of RNA velocity across adipocyte subpopulations. (**D**) UMAP trajectories of adipocyte subpopulations from young and old mice. The left panel depicts young mice, while the right represents old mice. (**E**) The quantity of Adipocyte1-A, Adipocyte1-B, and Adipocyte2 in 2-month-old and 26-month-old mice. (**F**) Gene expression trajectories for *Acacb*, *Cidea*, and *Ghr* across adipocyte subpopulations in young and old mice. The plots display individual expression levels within three identified adipocyte subtypes (Adipocyte 1A, Adipocyte 1B, and Adipocyte2) over pseudotime. Solid lines represent the expression trend for young mice, while dashed lines indicate the trend for old mice.

Due to the presence of 13,318 nuclei in Adipocyte1, we wanted to further categorize the cells based on their function. Therefore, we re-clustered Adipocyte1 and annotated the clusters as Adipocyte1A and Adipocyte1B (Figs. 7A, S7A). Further functional enrichment analysis revealed that Adipocyte1A is mainly involved in early adipocyte protein transcription and translation (Fig. S7B), while Adipocyte1B is primarily involved in the formation of intermediates.

To investigate the transcriptional regulatory pathways within these adipocyte subpopulations, we used snRNA-seq data for pseudotime trajectory and RNA velocity analyses^23^. Using Monocle, we identified three pseudotime trajectories: two transitioning from Adipocyte1 to Adipocyte2, with gene enrichment analysis showing their involvement in early adipocyte protein synthesis, labeled as Adipocyte1A, and one representing a self-transition within Adipocyte1, involved in precursor formation, intermediate metabolism, and fatty acid synthesis intermediates, labeled as Adipocyte1B (Fig. 7A,B). Based on pseudotime trajectories, we confirmed Adipocyte1’s capacity to transition to Adipocyte2, and from these results, we inferred that Adipocyte1B represents an intermediate state transitioning from Adipocyte1 to Adipocyte2. RNA velocity landscapes revealed transcriptional directionality from Adipocyte1 to Adipocyte2, with Adipocyte2 exhibiting the most significant RNA velocity characteristics (Fig. 7C).

To gain deeper insights into the transcriptional trajectory alterations within adipocyte subpopulations during the aging process, we conducted pseudotime trajectory analysis of adipocytes from young and old mice (Fig. 7D, Table S7-1, 2). Notably, in 26-month-old mice, the pseudotime trajectory of Adipocyte1-B disappeared, alongside a significant reduction in its cell population (Fig. 7E), confirming the profound impact of aging on the differentiation of adipocyte subgroups. Additionally, we analyzed the developmental trajectories of young and old mice individually and observed that Adipocyte2 in both age groups exhibited high expression of specific module genes (Module6 in young mice Adipocyte2 and Module1 in old mice Adipocyte2) (Fig. S7C,D). Further exploration of the dominant genes within these highly expressed modules, including the top 20 genes mentioned in the text, revealed that key genes associated with adipose synthesis and metabolism, such as *Cidea*, *Ghr*, and *Acacb*, show higher expressions in young mice compared to old mice (Fig. 7F).

### Ligand–receptor analysis reveals the interactions between FAPs and adipocytes across different age groups

Given the inherent capability of FAPs to undergo adipogenic transformation, our aim is to elucidate the impact of aging on the interplay between FAPs and adipocytes. We employed advanced computational methods to assess the ligand–receptor (L–R) interaction activity across these cellular subpopulations.

**Figure 8:**
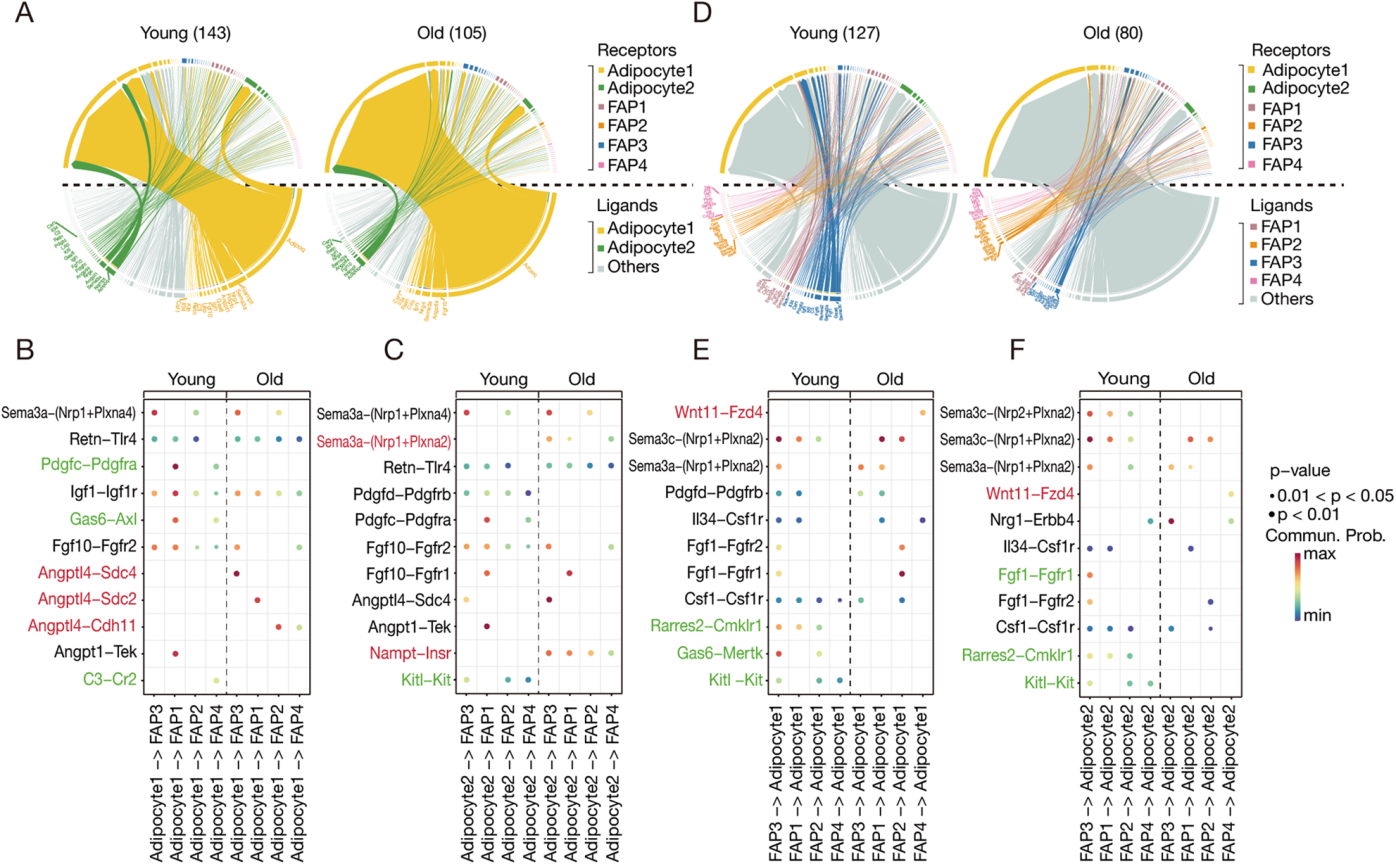
Investigation of Ligand–receptor (L–R) interactions across subgroups of adipocytes, FAPs, and other cell types in 2-month-old and 26-month-old mice. (**A**) Chord diagrams displaying intercellular L–R interactions in adipocyte subpopulations and FAPs in young (left) and old (right) mice. Ligands differentially expressed by adipocytes are below the dashed line, while receptors overexpressed in the receiving cells (consisting of both adipocytes and FAPs) are situated above. Communications from adipocytes (L) to FAPs (R) are indicated in green and yellow, with other interactions presented in gray. (**B**) Bubble charts define normalized interaction scores between adipocytes and FAPs in both young and old states. These interaction scores are determined by the levels of ligand expression in Adipocyte1 and receptor expression within the receiving cells. Columns represent receiving cell types, while rows signify predicted L–R pairs. The color scale denotes interaction strength, with higher scores marking stronger cellular interaction. Green L–R pairs exist only in young mice, whereas red pairs are unique to old mice. **(C)** Bubble charts define normalized interaction scores between adipocytes and FAPs in both young and old states. These interaction scores are determined by the levels of ligand expression in Adipocyte2 and receptor expression within the receiving cells. (**D**) Chord diagrams in young (left) and old (right) adipose tissues showing intercellular L–R interactions. Ligands differentially expressed by receiving FAPs are below the dashed line, whereas receptors overexpressed in adipocytes are located above. Communications from FAPs (L) to adipocytes (R) are highlighted, while other interactions are indicated in gray. (**E**) Bubble charts illustrating the interaction scores between ligands in FAPs and their receptors in adipocyte1. (**F**) Bubble charts illustrating the interaction scores between ligands in FAPs and their receptors in adipocytes2.

Our integrative analysis identified 270 potential ligand–receptor (L–R) interaction pairs between adipocytes and FAPs in young mice, and 185 such pairs in aged mice. This suggests a decline in intercellular communication during aging (Fig. S8A). In young mice, adipocytes overexpressed ligands such as *Nampt*, *Sema3a*, *Angpt1*, and *Fgf10*, while in the aged mice, there was a proclivity towards *Adipoq* expression. This expression alteration resulted in a significant reduction in L–R interactions in the aged mice, particularly influencing the impact of adipocytes on FAPs (Fig. 8A). Further dissection of the communication between adipocytes and FAPs identified 11 specific L–R pairs that exhibited tighter associations in either the young or aged mice (Fig. 8B). Notably, interactions like Pdgfc–Pdgfra and Gas6–Axl were exclusive to young mice, while Angptl4–Sdc4, Angptl4–Sdc2, and Angptl4–Cdh11 manifested only in aged mice. A similar communication pattern was identified between Adipocyte2 and FAPs, with young mice exhibiting a greater abundance of shared L–R pairs (Fig. 8B,C). Examining the communication from FAPs to adipocytes, the young mice demonstrated 127 interaction pairs, as opposed to 85 in aged mice, further confirming the detrimental impact of aging on cellular communication (Fig. 8D). In the young, FAPs were engaged in extensive cross-talk with both Adipocyte1 and Adipocyte2 subpopulations, facilitated by interactions like *Rarres2*, *Gas6* with *Cmklr1*, *Mertk*, and others (Fig. 8E,F). Such interactions were reduced or absent in the aged cohort Collectively, our findings highlight a significant reduction in the communication between Adipocytes and FAPs during the aging process. Employing a consistent approach, we investigated the interplay between adipocytes and immune cells, mesothelial cells, and endothelial cells. Our findings signify that aging declines the interactions between them, suggesting that aging may have detrimental influences on the crosstalk between adipocytes and other cellular entities (Fig. S8B–F).

## Discussion

In our study, we used single-nucleus RNA sequencing (snRNA-seq) to clarify the transcriptomic landscape of both SAT and VAT in young and old mice at an unprecedented single-nucleus resolution. This approach enabled the generation of a comprehensive cellular atlas of SAT and VAT, allowing for a complete investigation into the cellular diversity and adiposity variations within specific regions of the mouse body. Furthermore, it allowed us to disentangle the cellular mechanisms governing the age-associated regulation of white adipose tissue in mice.

Intriguingly, our observations in aged mice revealed an upregulation of T cell activation and B cell activation in VAT, alongside a downregulation in inflammatory response modulation. This phenomenon has been previously elucidated in studies demonstrating an increase in mature B2 cells and plasma IgG levels in VAT during aging^24^. Concurrently, aged VAT had heightened *Pou2af1* expression, a B cell-specific nuclear cofactor. *Pou2af1* removal in mice improved metabolic parameters, stunted the growth of B2 cells in VAT, and reduced circulating IgG2c and pro-inflammatory cytokines^24^. Additionally, aged VAT experienced an expansion of various T cell populations, notably CD8+ and CD4+ T cells, with Tregs increasing along with age^25^. This leads us to speculate that the multifaceted interplay of immune cells in VAT maintains immune landscape homeostasis throughout aging, while the overall immune function exhibits an age-associated decline. In our investigation, we observed an increasing trend in the proportions of both M1 and M2 macrophages with age. Maintaining a stable balance between M1 and M2 macrophages in the immune cell environment is crucial; M1 macrophages primarily mediate pro-inflammatory responses, while M2 macrophages are mainly involved in anti-inflammatory responses^26^. However, our research showed that the increase in M1 proportion exceeded that of M2, suggesting an enhanced inflammatory response in immune cells with aging. a shift in this balance can readily precipitate a state of inflammation within the organism. These studies emphasized that aging skews the adipocyte-macrophage axis towards a pro-inflammatory approach^27^. Consolidating our insights, aging exerts a dampening effect on immune cell functionalities, characterized by a subdued non-specific immune transcriptomic profile in macrophages and a compromised adaptive immune transcriptomic profile throughout B and T cells. Consequently, this results in a systemic weakening of the innate and adaptive immune defenses.

In addition, our study aligns with previous research on SAT and VAT, indicating that SAT lipogenesis surpasses VAT^28^. Elevated lipolysis and reduced lipogenesis in VAT lead to higher release of lipid metabolites^29^, while SAT promotes lipid storage due to enhanced lipogenesis^30^. We identified two adipocyte subpopulations: nascent adipocytes (Adipocyte1) and mature adipocytes (Adipocyte2). Aging increases Adipocyte1 and decreases Adipocyte2, suggesting reduced mature adipocyte formation and impaired maturation of immature adipocytes, accompanied by decreased metabolic pathway expression, particularly lipid anabolism. This shift may contribute to the reduced adipogenic potential observed in senescent mice. For the first time, we used *Tshr* and *Cidea* as biomarkers in aging research, with *Tshr* identified on adipocyte membranes^22^ and *Cidea* linked to fatty acid metabolism^31^. Aging also significantly expands SAT and VAT areas^32^. Existing research shows that adipocyte hypertrophy causes hypoxia in WAT, triggering inflammation and metabolic dysfunctions^33^. We propose that adipocyte size contributes to inflammation-related metabolic abnormalities in SAT and VAT, supported by data showing aging influences pro-inflammatory molecule secretion, aggravating metabolic diseases^34^.

Notably, in old mice, cell differentiation trajectories associated with precursor metabolism and energy generation appear diminished. It is well-known that such cell differentiation is closely tied to the generation of metabolic intermediates^35^, playing a central role in various metabolic pathways, such as glycolysis, the tricarboxylic acid cycle (also known as TCA or the citric acid cycle), and fatty acid metabolism^36^. Reducing these intermediates during aging may adversely affect the biosynthesis and metabolism of mature adipocytes. Through in-depth analysis of highly expressed module genes in Adipocyte2, we identified that aging appears to result in a decrease in gene expression levels related to fat synthesis metabolism. This includes genes such as *Cidea*, tied to energy expenditure and thermogenesis^37^, *Ghr* is involved in regulating lipid metabolism, which includes processes like fat breakdown and synthesis^38^, and *Acacb*, which plays a regulatory role in fatty acid oxidation^39^. These findings from the pseudotime analysis align well with the conclusions drawn in Fig. 5, providing strong support for the profound impact of aging on the differentiation trajectories of adipocyte subpopulations.

Finally, through comprehensive receptor–ligand (R–L) interaction analyses, we uncover the detrimental impact of aging on the cellular crosstalk between Adipocytes and FAPs. In Adipocyte1-FAPs interactions, pivotal ligand–receptor pairs such as Pdgfc–Pdgfra^40^ are crucial in cellular growth, differentiation, and migration. Conversely, ANGPTL4^41^ engages multiple receptors-SDC4, SDC2, and CDH11-to influence cell adhesion and lipolysis. These findings highlight that in young mice, interactions between Adipocyte1 and FAPs are mutually beneficial, fostering FAP-to-Adipocyte1 conversion or facilitating Adipocyte1 maturation. In aged mice, the dynamics shift towards detrimental outcomes, potentially due to age-related pathologies or physiological alterations, characterized by an enhanced interaction between Angptl4 and its diverse receptors. Mirroring these trends, Adipocyte2-FAPs interactions are similar. In older mice, the pro-inflammatory Retn-Tlr4 interactions are upregulated^42^, while anti-inflammatory Angpt1-Tek pairs are present mainly in young mice^43^. The FAPs-Adipocytes crosstalk reveals that aging undermines communication between these cell types, specifically in anti-inflammatory responses and tissue repair. Collectively, our data validate the adverse effects of aging on adipose tissue from the perspective of cellular communication.

## Materials and methods

### Study approval

The aim of this study was to explore the transcriptional differences in the adipocytes of young and old mice as they age. All animal experiments received approval from the Institutional Animal Care and Use Committee of the College of Animal Science and Technology, Sichuan Agricultural University, China, with the permit number DKY-2021202050. This study’s animal experiments adhered to the ARRIVE guidelines for reporting animal experiments and involved no legal or ethical issues. All methods were performed in accordance with the relevant guidelines and regulations.

### Mice

C57BL/6J mice were fed a sterile, full-nutrition pellet feed (Synergy Bioscience, feed number 1010002). Purified water, autoclaved after bottling, was provided ad libitum and changed 2–3 times per week, with individual water bottles assigned to each duplex cage. Mice were housed under standard laboratory conditions with a 12-h light/dark cycle and ad libitum access to food and water. All subjects were ensured to be Specific-Pathogen-Free (SPF) grade, excluding any disease effects. We selected female mice because males usually weigh more than females, and there’s often an inverse relationship between body weight and lifespan, especially in males, suggesting that male mortality is more affected by body weight. Also, male survival rates more across locations and groups, indicating that females are more resilient to environmental factors affecting survival^44^. Our experimental procedures, approved by the Institutional Animal Care and Use Committee at Sichuan Agricultural University under permit number DKY-2021202050. Adhered strictly to the ARRIVE guidelines, to maintain objectivity and reproducibility, we implemented randomization and blinding, with independent researchers conducting the random selection and group assignment of mice. A double-blind approach was also employed to prevent bias from experiment operators and data analysts.

### Nuclei isolation sorting from WAT (SAT and VAT)

WAT, including both SAT and VAT was isolated from female C57BL/6J mice at 2 months and 26 months of age. This resulted in a sample pool comprising four SAT specimens, and four VAT specimens, with two replicates from each age category. This strategy generated eight distinct sets of snRNA-Seq sequencing data. The harvested tissues were rinsed with pre-chilled PBSE, a PBS buffer supplemented with 2 mM EGTA. Subsequent nuclear extraction was conducted using the GEXSCOPE® Nucleus Separation Solution (manufactured by Singleron Biotechnologies, Nanjing, China), adhering to the protocol provided by the manufacturer. The resulting extracted nuclei were resuspended in PBSE to achieve a concentration of 106 nuclei per 400 μL, followed by filtration through a 40 μm cell filter, and counting assisted by Trypan blue. Enriched nuclei suspended in phosphate-buffered saline with EGTA (PBSE) were stained with DAPI (diluted 1:1000, Thermo Fisher Scientific, Cat# D1306) to tag the DNA. Under a fluorescence microscope, we identified single nuclei based on their distinct blue fluorescence, ensuring that only DAPI-positive singlets were analyzed. DAPI was used to verify single nuclei, and this process was critical for the accuracy of our subsequent single-nucleus RNA sequencing (snRNA-Seq), as it excluded any potential doublets or clusters.

### Single-nucleus RNA-sequencing

The single-nucleus suspension was corrected to a concentration of 3 to 4 × 105 nuclei/mL in PBS. This suspension was placed onto a microfluidic chip, using the GEXSCOPE® Single Nucleus RNA-seq Kit (Singleron Biotechnologies). Following the manufacturer’s guidelines (Singleron Biotechnologies), we constructed snRNA-seq libraries. These libraries were sequenced on an Illumina HiSeq × 10 platform, employing a 150 bp paired-end read protocol. The same sequencing approach was used for both SAT and VAT. A detailed procedure is outlined below.

Initially, the library was quantified using a Qubit dsDNA HS Assay Kit for double-stranded DNA fluorescence quantification, following the manufacturer’s instructions. Libraries were pooled, occupying a lane as a unit, with each library equilibrated to a 2 nM concentration according to molar concentration. A dilution sheet was prepared, cross-referencing all samples involved in each lane. Sample numbers were examined to prevent any errors. A new 96-well plate was used as a dilution plate. Using a Hamilton automated system and nuclease-free water, we added 2 μL of each sample to attain homogenization. Subsequently, according to the respective data volume proportions, all homogenized samples from the same lane were combined in a new 1.5 mL centrifuge tube. Fresh 0.2 N NaOH and 400 mM Tris–HCl were prepared to achieve a 0.25 nM PhiX concentration. Once the library was included proportionally, samples were vortexed to mix and centrifuged at 280×*g* for 1 min, before being placed on ice for later use. Finally, the sequencing flow cell was mounted, and the parameter configuration was established. We selected the NovaSeq Xp workflow, inputting the experimental name, cycle number, read length, and storage path. Following a software-based review of the information and an automatic machine parameter check, sequencing began after confirmation of the operational parameters.

### Histology

The size of adipocytes was measured using the image analysis software ImageJ, which analyzed the hematoxylin and eosin (H&E) stained slides marked with adipocytes. For each sample, three different fields of view were randomly selected for the measurement to ensure the results were representative and covered different areas of the adipose tissue. The selection of each field of view aimed to capture uniformly distributed adipocytes, allowing for an accurate reflection of the variation in adipocyte size within the tissue. Adipose tissue was embedded into Tissue Tek O.C.T. compound (Sakura, 4583), frozen in 96% ethanol atop dry ice, and stored at − 80 °C. The frozen O.C.T.-embedded adipose tissue blocks were fixed for 24 h at 4 °C using 4% paraformaldehyde dissolved in 1 × PBS (pH 7.4) before being embedded in paraffin. Subsequently, the paraffin-embedded adipose tissue blocks were sectioned at a thickness of 5 um using an HM 355S Automatic Microtome (Axlab) and arranged on Superfrost Plus adhesion slides, ready for histological staining. We have prepared four HE-stained histological sections from four different organizations: SAT Young, SAT Old, VAT Young, VAT Old. For each section, we measured the area of 20 adipocytes and calculated their average area.

### Single-nucleus RNA-sequencing analysis

In our snRNA-seq analysis, for young SAT samples, we loaded approximately 7861 nuclei (SAT 1) and 8074 nuclei (SAT 2) onto the microfluidic chip, with average reads per cell of 63,552 and 56,149, median UMIs of 1196 and 1185, and gene counts of 770 and 793, respectively, achieving sequencing saturations of about 63.53%. For old SAT samples, 6158 nuclei (SAT 1) and 9497 nuclei (SAT 2) were loaded, with average reads per cell of 75,878 and 58,361, median UMIs of 935 and 1058, and gene counts of 613 and 665, respectively, with saturation levels of 66.15% and 37.04%. In young VAT, 7344 nuclei (VAT 1) and 17,148 nuclei (VAT 2) were processed, with average reads per cell of 81,286 and 27,007, median UMIs of 1441 and 1844, and gene counts of 699 and 1153, respectively, and saturations of 65.79% and 52.77%. For old VAT, 7928 nuclei (VAT 1) and 11,341 nuclei (VAT 2) were sequenced, with average reads per cell of 61,912 and 44,745, median UMIs of 1126 and 1037, and gene counts of 704, reaching saturation levels of 67.09% and 66.87%, respectively. In our study, we filtered nuclei based on the following criteria: more than 500 genes per nucleus (nFeature_RNA > 500), more than 1000 total counts per nucleus (nCount_RNA > 1000), and less than 15% mitochondrial gene content (percent.mt < 15) to ensure data quality. After quality control in our single-cell sequencing study, the number of nuclei was as follows: SAT 1 young had 4191 nuclei, SAT 2 young also had 4191 nuclei, SAT 1 old had 2128 nuclei, and SAT 2 old had 9497 nuclei. For VAT, VAT 1 young had 2570 nuclei, VAT 2 young had 10,637 nuclei, VAT 1 old had 3861 nuclei, and VAT 2 old had 4144 nuclei.

In our single-nucleus RNA sequencing (snRNA-seq) study, we used Celescope from Singleron for read alignment with the Mus musculus GRCm39 reference genome. To address batch effects, we employed the Harmony tool^45^ for batch correction, ensuring data integrity and minimal batch effect, as evidenced by the consistent cell cycle phase distribution across G1, G2M, and S phases in our integrative analysis. For cell cycle phase estimation, we used Seurat’s^46^ CellCycleScoring function leveraging well-characterized cell cycle markers, and normalized data using Seurat’s ScaleData function for accurate phase attribution.

We performed clustering using the ‘runUMAP’ function^47^, selecting 30 dimensions based on PCA’s ElbowPlot to capture data variance effectively, with a resolution of 0.1 determined by the ‘clustree’ method to optimally define clusters. Cell clusters were separated into various cell types, identified through the expression of established marker genes. Cell‐type‐specific gene signatures were revealed using Seurat’s “FindAllMarkers” function^48^. To determine cell‐type‐specific genes, we applied thresholds: a minimum log2 fold change (FC) ≥ 1 and at least 25% presence in one of the populations. Marker gene expression profiles were depicted using Seurat’s “DotPlot” function.

Differential gene expression analysis was conducted using the ‘FindMarkers’ function^49^ within Seurat (log2FC > 1, *P* < 0.05). To ensure the robustness of our findings, trajectory analysis was performed using Monocle3 and Velocity^50^, allowing us to observe dynamic gene expression changes and predict future cell states.

Lastly, CellChat was utilized for cell–cell communication analysis, inputting our snRNA-seq data to analyze interaction patterns among adipocyte cell types. CellChat’s^51^ network construction and analysis functions facilitated a comprehensive understanding of the communication dynamics and regulatory networks, shedding light on the complex interactions governing adipose tissue aging and differentiation.

### Immunofluorescence microscopy of mature adipocytes

#### Tissue paraffin embedding

In our immunofluorescence experiments, we used the following primary antibodies: *Cd79b* from Thermo Fisher (11-0793-42) at 0.5–1 µg/mL, *Skap1* (Thermo Fisher, cat# PA5-85456, 1:1000), *Lyz2* (Thermo Fisher, cat# PA5-16668, 1:2000), *Gsn* (EIAab, cat# P0372Rb-h, 1:500), *Asic2* (Thermo Fisher, cat# PA5-143911, 1:1000), *Syne2* (Thermo Fisher, cat# MA5-18075, 1:1000), *Cacnb3* (GeneTex, cat# GTX47704, 1:1000), and *Bst1* (Thermo Fisher, cat# 17-1579-42, 5 µL/0.125 µg/test). For *Tshr* and *Cidea*, we used Thermo Fisher antibodies (cat# PA5-116082 at 1:1000, and cat# PA5-19908 at 2.5–5 µg/mL, respectively).

Adipose tissue sections underwent a series of treatments for optimal immunostaining. The sections were dewaxed and rehydrated via a sequence of solvents: two xylene baths (15 min each), followed by two absolute ethanol rinses (5 min each), followed by descending alcohol concentrations of 85% and 75% (5 min each), and finally rinsed in distilled water. For antigen retrieval, sections were immersed in citric acid antigen repair buffer (pH 6.0) within a repair box. They were then subjected to intermittent microwave heating, cycling between medium and low power until boiling, for a duration of 10 min. Care was taken to limit excessive buffer evaporation, ensuring that sections remained hydrated throughout the process. After the sections cooled naturally, they were washed three times in PBS (pH 7.4), with each wash lasting 5 min on a shaker. The tissue sections were blocked and incubated with a primary antibody, prepared at an appropriate dilution in PBS, overnight at 4 °C in a humidified chamber to prevent evaporation. After the treatment with the antibody, sections were rinsed three times in PBS (pH 7.4) on a shaker, for 5 min per wash. They were then treated with a fluorescently labeled antibody for 50 min at room temperature in the dark. For nuclear counterstaining, sections underwent three additional washes in PBS (pH 7.4) for 5 min each on a shaker. After the tissue sections were slightly air-dried, DAPI staining solution (1:1000 dilution, Thermo Fisher Scientific, Cat# D1306) was applied to facilitate nuclear counterstaining. The sections were incubated at room temperature for 10 min to allow thorough staining. Subsequently, they were washed three times with PBS to remove excess stain, gently air-dried, and then mounted with an anti-fade mounting medium to preserve fluorescence. High-resolution imaging was performed using a Nikon upright fluorescence microscope to capture the distinct blue fluorescence of DAPI-stained nuclei, and representative images were systematically documented.

### Supplementary Information

Supplementary Information.

## Data Availability

The single-nucleus RNAseq dataset generated in this study are available at Gene Expression Omnibus (GEO) database (GSE241718, https://www.ncbi.nlm.nih.gov/geo/query/acc.cgi?acc=GSE241718, the secure token is mdstkmisnfmpzoh.). Sources of public datasets analyzed during the current study are (GSE241275, https://www.ncbi.nlm.nih.gov/geo/query/acc.cgi?acc=GSE241275, the secure token is ehytegqyzvsfnej.). The appearance of two separate submissions is because the data were uploaded in two stages. However, all data are from the same batch of samples and mice, sequenced on the same platform.
