# Supplementary material for: Dynamics of single-nuclei transcriptomic profiling of adipose tissue from diverse anatomical locations during mouse aging process
Source: Sci Rep. 2024 Jul 12;14:16093. doi: 10.1038/s41598-024-66918-w (PMC11245496; doi:10.1038/s41598-024-66918-w)
Supplement: Supplementary file 1 — Supplementary Information. [file 41598_2024_66918_MOESM1_ESM.pdf]

Supplementary information for

Dynamics of single-nuclei transcriptomic profiling of adipose tissue from diverse anatomical locations during mouse aging process

Yujie Wu, Ying Sun, Long Chen, Xingyan Tong, Can Liu, Lu Lu, Rui Zhang, Siyuan Wang, Ziyu Chen, Jiaman Zhang, Ziyin Han, Bo zeng, Mingzhou Li,*, Long Jin,*

Corresponding author. Email: longjin@sicau.edu.cn (L. Jin); mingzhou.li@sicau.edu.cn (M. Li);

Figure S1

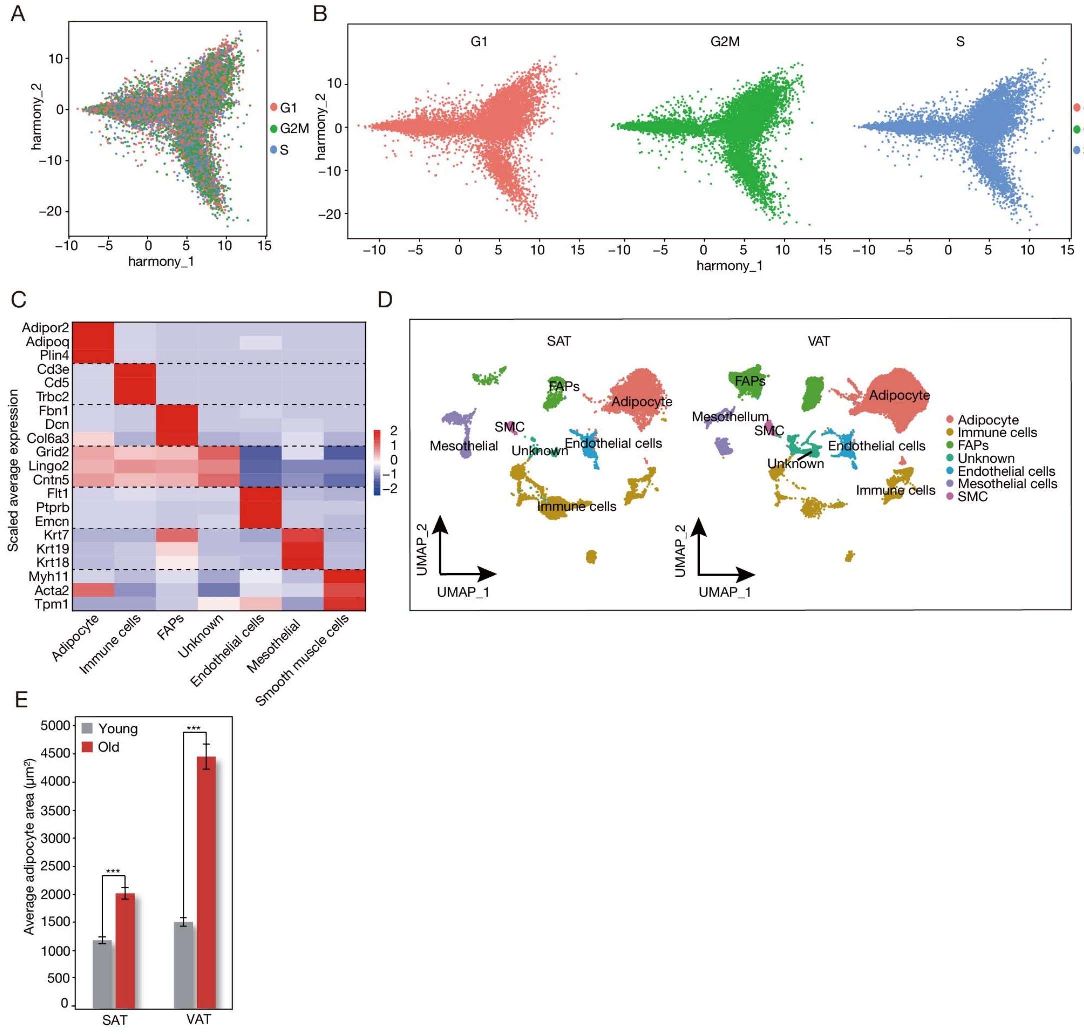

Figure S1: Cell cycle plots in single-nucleus RNA analysis, marker gene charts after clustering, and UMAP comparison between SAT and VAT locations.(A) Analysis of the entire cell cycle process.**(B)** G1, G2/M, and S phases of the cell cycle.**(C)** Enrichment maps of seven cluster marker genes identified through clustering analysis.**(D)** UMAP representations of SAT and VAT adipose tissues compared across two age intervals.**(E)**To reduce potential bias introduced by manual selection, we identified and selected 20 adipocytes from each section subjected to H&E staining at each age for area determination. We then calculated the average area for subsequent size comparison, this figure shows a comparative analysis of adipocyte area in SAT and VAT between 2-month-old and 26-month-old mice.

Figure S2

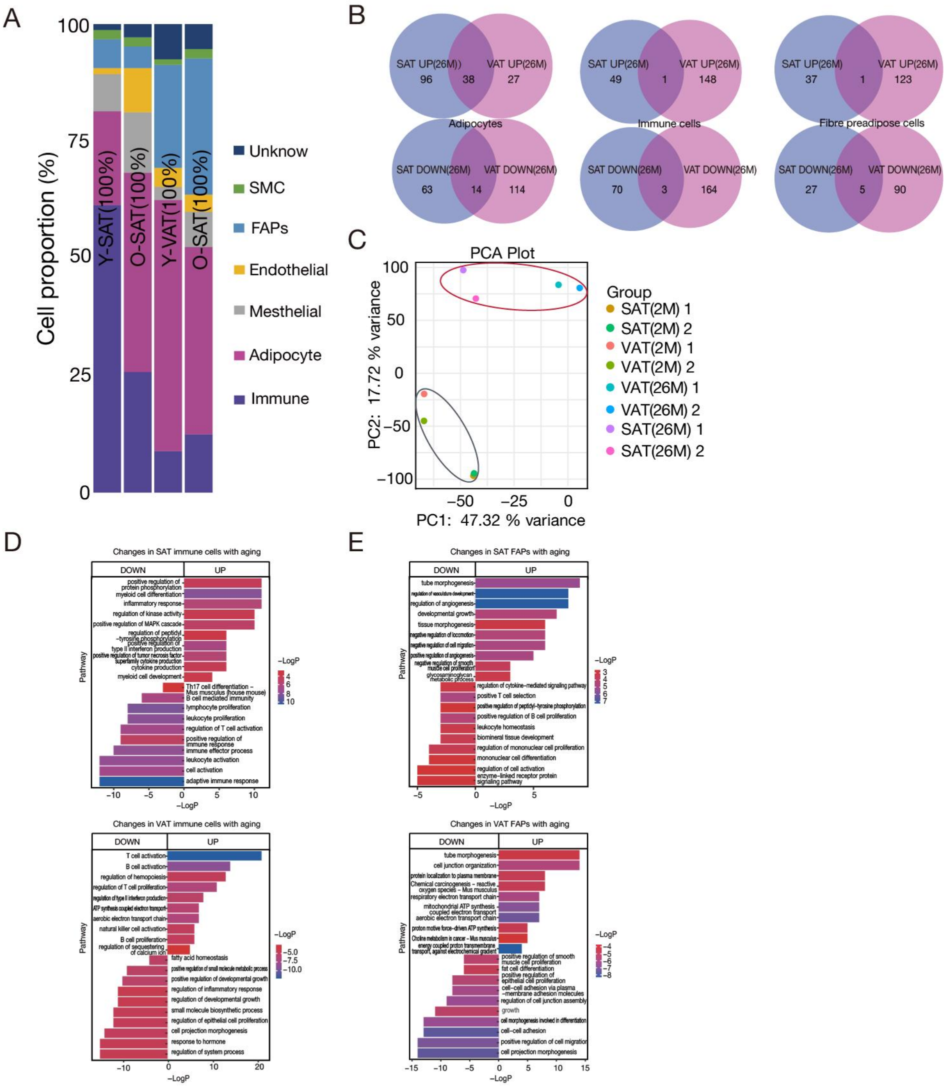
A
B
C
D
E
Cell proportion (%)
Y-SAT(100%)
O-SAT(100%)
Y-VAT(100%)
O-SAT(100%)
Unknow
SMC
FAPs
Endothelial
Mesthelial
Adipocyte
Immune
SAT UP(26M)
VAT UP(26M)
SAT DOWN(26M)
VAT DOWN(26M)
Adipocytes
Immune cells
Fibre preadipose cells
PCA Plot
PC1: 47.32 % variance
PC2: 17.72 % variance
Group
SAT(2M) 1
SAT(2M) 2
VAT(2M) 1
VAT(2M) 2
VAT(26M) 1
VAT(26M) 2
SAT(26M) 1
SAT(26M) 2
Changes in SAT immune cells with aging
Changes in VAT immune cells with aging
Changes in SAT FAPs with aging
Changes in VAT FAPs with aging
DOWN
UP
Pathway
−LogP

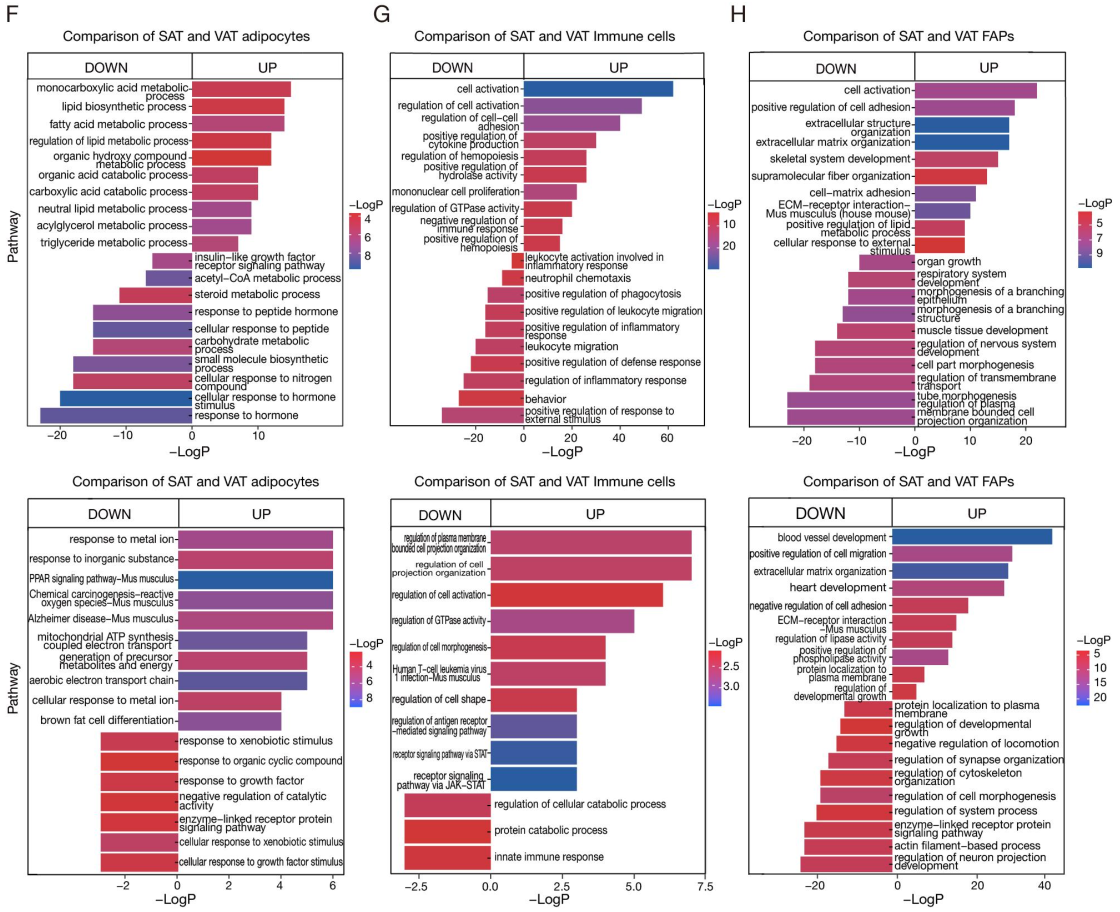
F
Comparison of SAT and VAT adipocytes
DOWN
UP
monocarboxylic acid metabolic process
lipid biosynthetic process
fatty acid metabolic process
regulation of lipid metabolic process
organic hydroxy compound metabolic process
organic acid catabolic process
carboxylic acid catabolic process
neutral lipid metabolic process
acylglycerol metabolic process
triglyceride metabolic process
insulin−like growth factor receptor signaling pathway
acetyl−CoA metabolic process
steroid metabolic process
response to peptide hormone
cellular response to peptide
carbohydrate metabolic process
small molecule biosynthetic process
cellular response to nitrogen compound
cellular response to hormone stimulus
response to hormone
Pathway
−LogP
G
Comparison of SAT and VAT Immune cells
cell activation
regulation of cell activation
regulation of cell−cell adhesion
positive regulation of cytokine production
regulation of hemopoiesis
positive regulation of hydrolase activity
mononuclear cell proliferation
regulation of GTPase activity
negative regulation of immune response
positive regulation of hemopoiesis
leukocyte activation involved in inflammatory response
neutrophil chemotaxis
positive regulation of phagocytosis
positive regulation of leukocyte migration
positive regulation of inflammatory response
leukocyte migration
positive regulation of defense response
regulation of inflammatory response
behavior
positive regulation of response to external stimulus
H
Comparison of SAT and VAT FAPs
cell activation
positive regulation of cell adhesion
extracellular structure organization
extracellular matrix organization
skeletal system development
supramolecular fiber organization
cell−matrix adhesion
ECM−receptor interaction−Mus musculus (house mouse)
positive regulation of lipid metabolic process
cellular response to external stimulus
organ growth
respiratory system development
morphogenesis of a branching epithelium
morphogenesis of a branching structure
muscle tissue development
regulation of nervous system development
cell part morphogenesis
regulation of transmembrane transport
tube morphogenesis
regulation of plasma membrane bounded cell projection organization
Comparison of SAT and VAT adipocytes
response to metal ion
response to inorganic substance
PPAR signaling pathway−Mus musculus
Chemical carcinogenesis−reactive oxygen species−Mus musculus
Alzheimer disease−Mus musculus
mitochondrial ATP synthesis coupled electron transport
generation of precursor metabolites and energy
aerobic electron transport chain
cellular response to metal ion
brown fat cell differentiation
response to xenobiotic stimulus
response to organic cyclic compound
response to growth factor
negative regulation of catalytic activity
enzyme−linked receptor protein signaling pathway
cellular response to xenobiotic stimulus
cellular response to growth factor stimulus
Comparison of SAT and VAT Immune cells
regulation of plasma membrane bounded cell projection organization
regulation of cell projection organization
regulation of cell activation
regulation of GTPase activity
regulation of cell morphogenesis
Human T−cell leukemia virus 1 infection−Mus musculus
regulation of cell shape
regulation of antigen receptor−mediated signaling pathway
receptor signaling pathway via STAT
receptor signaling pathway via JAK−STAT
regulation of cellular catabolic process
protein catabolic process
innate immune response
Comparison of SAT and VAT FAPs
blood vessel development
positive regulation of cell migration
extracellular matrix organization
heart development
negative regulation of cell adhesion
ECM−receptor interaction−Mus musculus
regulation of lipase activity
positive regulation of phospholipase activity
protein localization to plasma membrane
regulation of developmental growth
protein localization to plasma membrane
regulation of developmental growth
negative regulation of locomotion
regulation of synapse organization
regulation of cytoskeleton organization
regulation of cell morphogenesis
regulation of system process
enzyme−linked receptor protein signaling pathway
actin filament−based process
regulation of neuron projection development

I

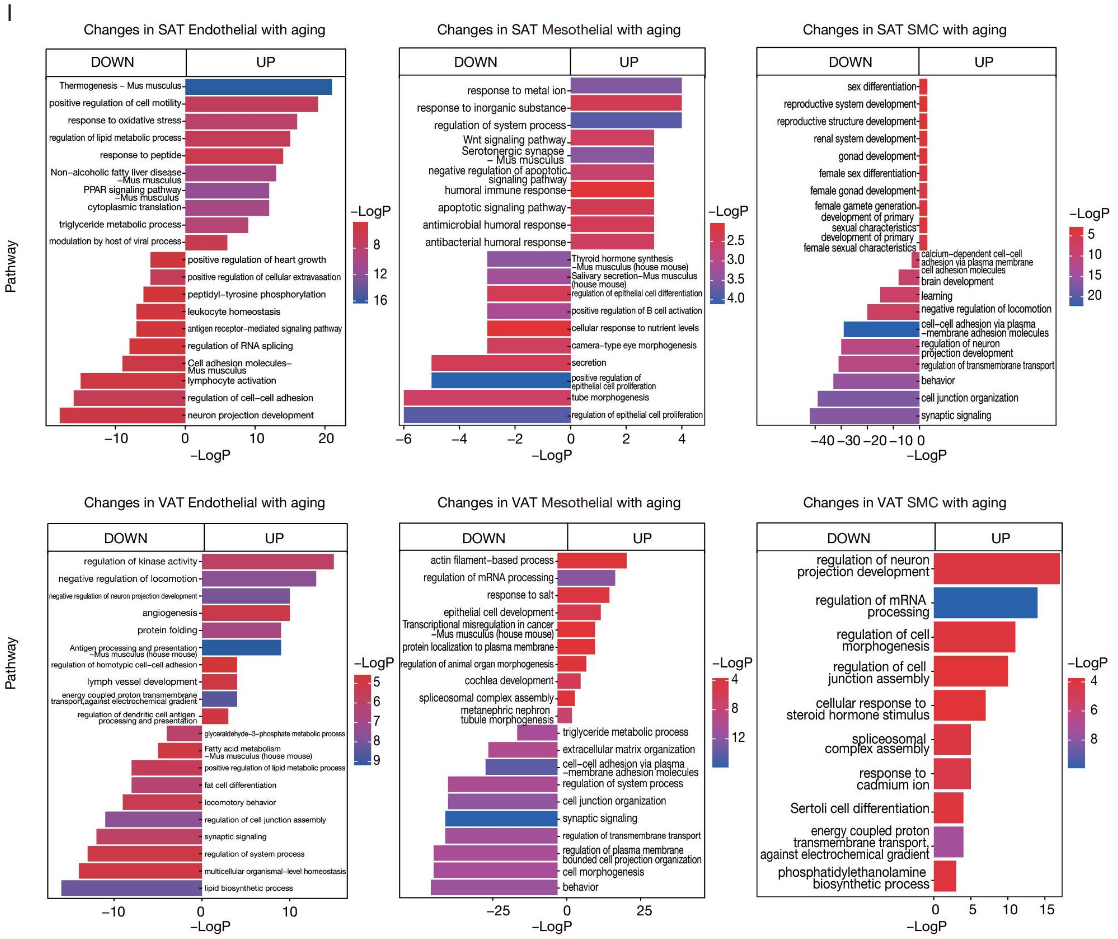
Changes in SAT Endothelial with aging
DOWN
UP
Thermogenesis – Mus musculus
positive regulation of cell motility
response to oxidative stress
regulation of lipid metabolic process
response to peptide
Non−alcoholic fatty liver disease −Mus musculus
PPAR signaling pathway −Mus musculus
cytoplasmic translation
triglyceride metabolic process
modulation by host of viral process
positive regulation of heart growth
positive regulation of cellular extravasation
peptidyl−tyrosine phosphorylation
leukocyte homeostasis
antigen receptor−mediated signaling pathway
regulation of RNA splicing
Cell adhesion molecules− Mus musculus
lymphocyte activation
regulation of cell−cell adhesion
neuron projection development
−LogP
Pathway
Changes in SAT Mesothelial with aging
response to metal ion
response to inorganic substance
regulation of system process
Wnt signaling pathway
Serotonergic synapse − Mus musculus
negative regulation of apoptotic signaling pathway
humoral immune response
apoptotic signaling pathway
antimicrobial humoral response
antibacterial humoral response
Thyroid hormone synthesis −Mus musculus (house mouse)
Salivary secretion−Mus musculus (house mouse)
regulation of epithelial cell differentiation
positive regulation of B cell activation
cellular response to nutrient levels
camera−type eye morphogenesis
secretion
positive regulation of epithelial cell proliferation
tube morphogenesis
regulation of epithelial cell proliferation
Changes in SAT SMC with aging
sex differentiation
reproductive system development
reproductive structure development
renal system development
gonad development
female sex differentiation
female gonad development
female gamete generation
development of primary sexual characteristics
development of primary female sexual characteristics
calcium−dependent cell−cell adhesion via plasma membrane cell adhesion molecules
brain development
learning
negative regulation of locomotion
cell−cell adhesion via plasma −membrane adhesion molecules
regulation of neuron projection development
regulation of transmembrane transport
behavior
cell junction organization
synaptic signaling
Changes in VAT Endothelial with aging
regulation of kinase activity
negative regulation of locomotion
negative regulation of neuron projection development
angiogenesis
protein folding
Antigen processing and presentation −Mus musculus (house mouse)
regulation of homotypic cell−cell adhesion
lymph vessel development
energy coupled proton transmembrane transport,against electrochemical gradient
regulation of dendritic cell antigen processing and presentation
glyceraldehyde−3−phosphate metabolic process
Fatty acid metabolism −Mus musculus (house mouse)
positive regulation of lipid metabolic process
fat cell differentiation
locomotory behavior
regulation of cell junction assembly
synaptic signaling
regulation of system process
multicellular organismal−level homeostasis
lipid biosynthetic process
Changes in VAT Mesothelial with aging
actin filament−based process
regulation of mRNA processing
response to salt
epithelial cell development
Transcriptional misregulation in cancer −Mus musculus (house mouse)
protein localization to plasma membrane
regulation of animal organ morphogenesis
cochlea development
spliceosomal complex assembly
metanephric nephron tubule morphogenesis
triglyceride metabolic process
extracellular matrix organization
cell−cell adhesion via plasma −membrane adhesion molecules
regulation of system process
cell junction organization
synaptic signaling
regulation of transmembrane transport
regulation of plasma membrane bounded cell projection organization
cell morphogenesis
behavior
Changes in VAT SMC with aging
regulation of neuron projection development
regulation of mRNA processing
regulation of cell morphogenesis
regulation of cell junction assembly
cellular response to steroid hormone stimulus
spliceosomal complex assembly
response to cadmium ion
Sertoli cell differentiation
energy coupled proton transmembrane transport, against electrochemical gradient
phosphatidylethanolamine biosynthetic process

J

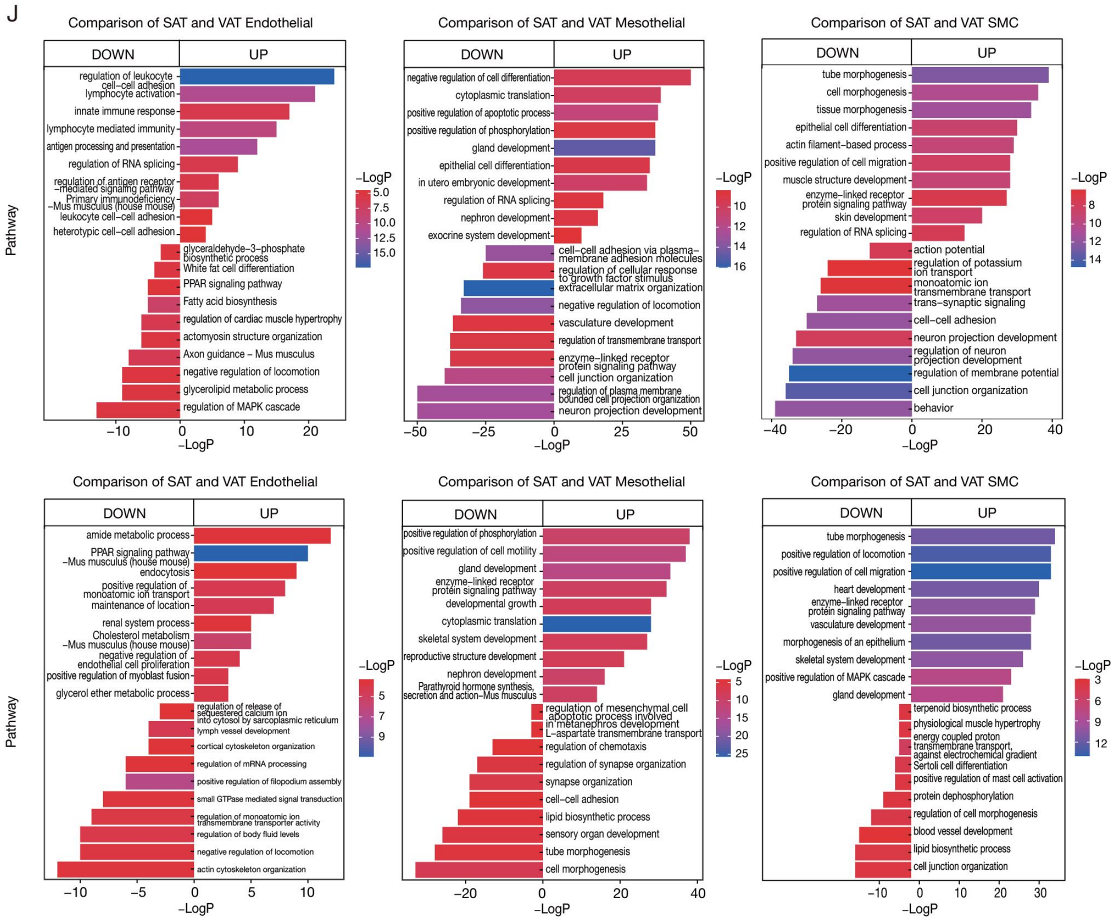

Figure S2. KEGG and GO analysis of 6 clusters. (A) Proportional representation of nuclei across the seven identified clusters of cells. **(B)** Genes exhibiting up-regulation and down-regulation between SAT and VAT adipocytes in 26-month-old mice. **(C)** PCA plots illustrating SAT and VAT data points. **(D)** Immune cells in SAT and VAT were subjected to KEGG and GO enrichment analyses at both 2 and 26 months of age, respectively. **(E)** fibro/adipogenic progenitors (FAPs) in SAT and VAT were subjected to KEGG and GO enrichment analyses at both 2 and 26 months of age, respectively. **(F)** KEGG and GO enrichment analyses conducted on adipocytes from SAT and VAT adipose tissues at two ages, 2 months and 26 months. **(G)** KEGG and GO enrichment analyses conducted on immune cells from SAT and VAT adipose tissues at two ages, 2 months and 26 months. **(H)** KEGG and GO enrichment analyses conducted on FAPs from SAT and VAT adipose tissues at two ages, 2 months and 26 months. **(I)** Mesothelial cells and endothelial cells within SAT and VAT adipose tissues subjected to KEGG and GO enrichment analyses at 2 and 26 months of age. **(J)** KEGG and GO enrichment analyses conducted on mesothelial cells and endothelial cells from SAT and VAT adipose tissues at two ages, 2 months and 26 months.

Figure S3

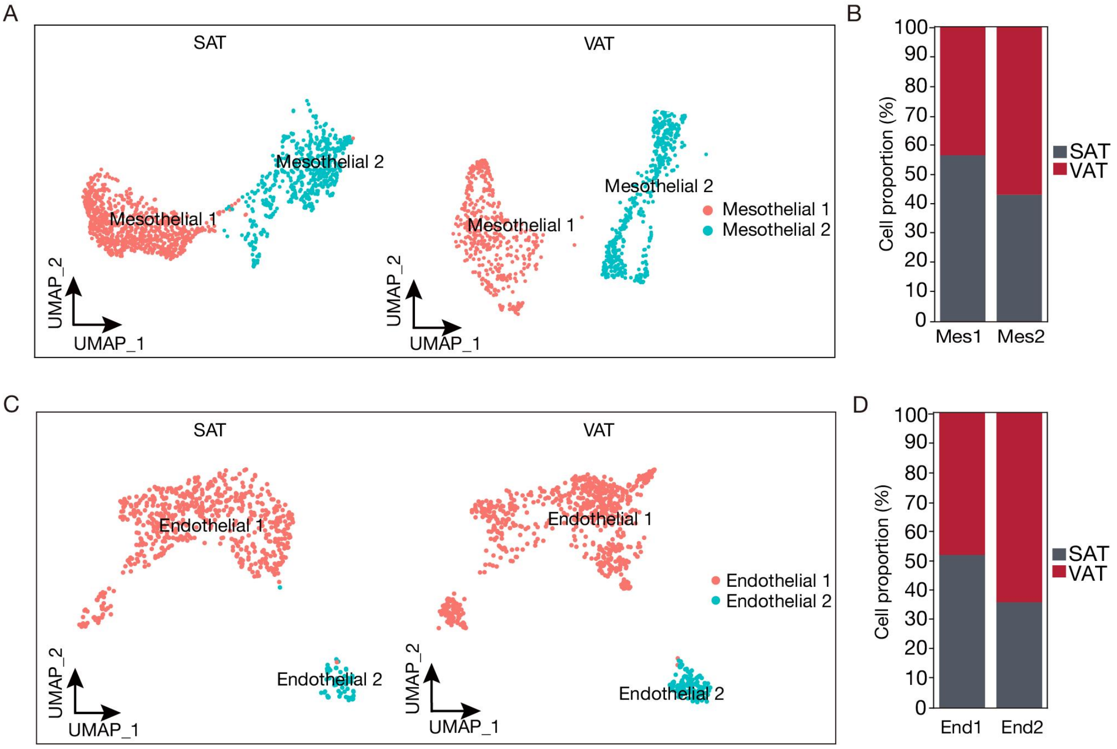

Figure S3. Comparison of endothelial and mesenchymal cell subgroups in adipose tissue between SAT and VAT locations. **(A)** UMAP illustrations comparing mesothelial subgroups across SAT and VAT adipose tissues over two ages. **(B)** Mesothelial cell subgroup proportion chart for SAT and VAT. **(C)** UMAP illustrations comparing endothelial cell subgroups across SAT and VAT adipose tissues over two ages. **(D)** Endothelial cell subgroup proportion chart for SAT and VAT.

Figure S4

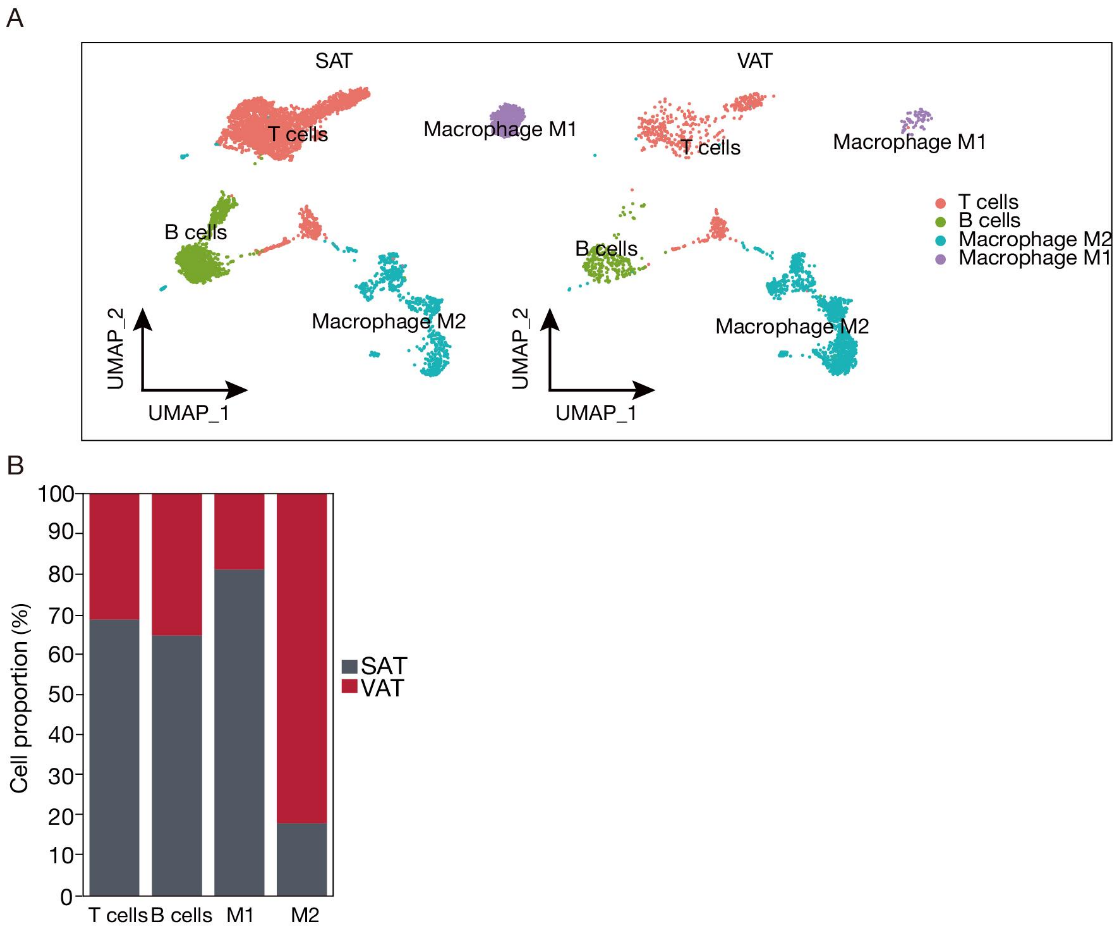

Figure S4. Comparison of immune cell subgroups in adipose tissue between SAT and VAT locations. (A) UMAP illustrations comparing immune cell subgroups between SAT and VAT adipose tissues over two ages. **(B)** Immune subgroup proportion chart for SAT and VAT.

Figure S5

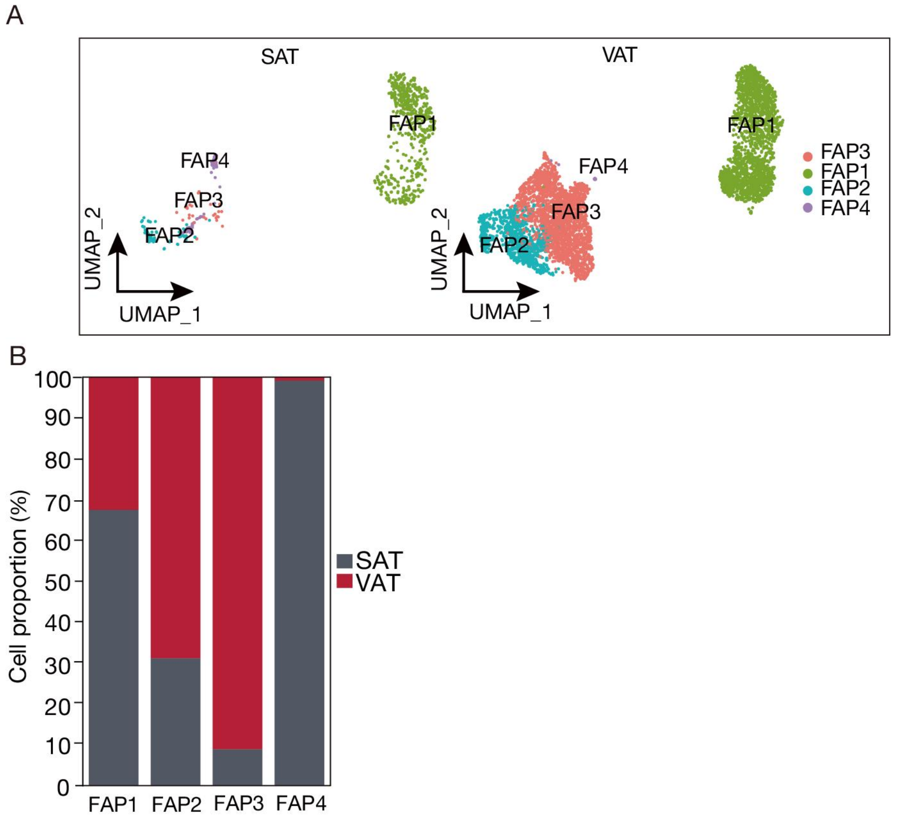

Figure S5. Comparison of FAPs subgroups in adipose tissue between SAT and VAT locations. **(A)** UMAP illustrations comparing FAP subgroups between SAT and VAT adipose tissues across two ages. **(B)** FAPs subgroup proportion chart for SAT and VAT.

Figure S6

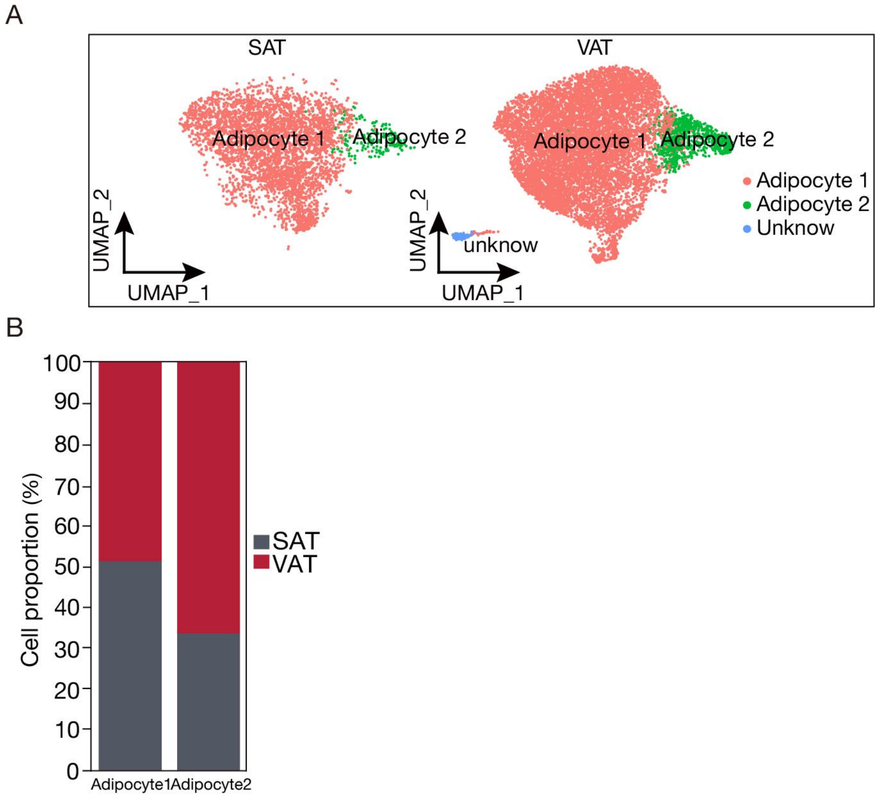

Figure S6. Comparison of adipocyte subgroups in adipose tissue between SAT and VAT locations. (A) UMAP illustrations comparing adipocyte subgroups between SAT and VAT adipose tissues over two ages. **(B)** Adipocytes subgroup proportion chart for SAT and VAT.

Figure S7

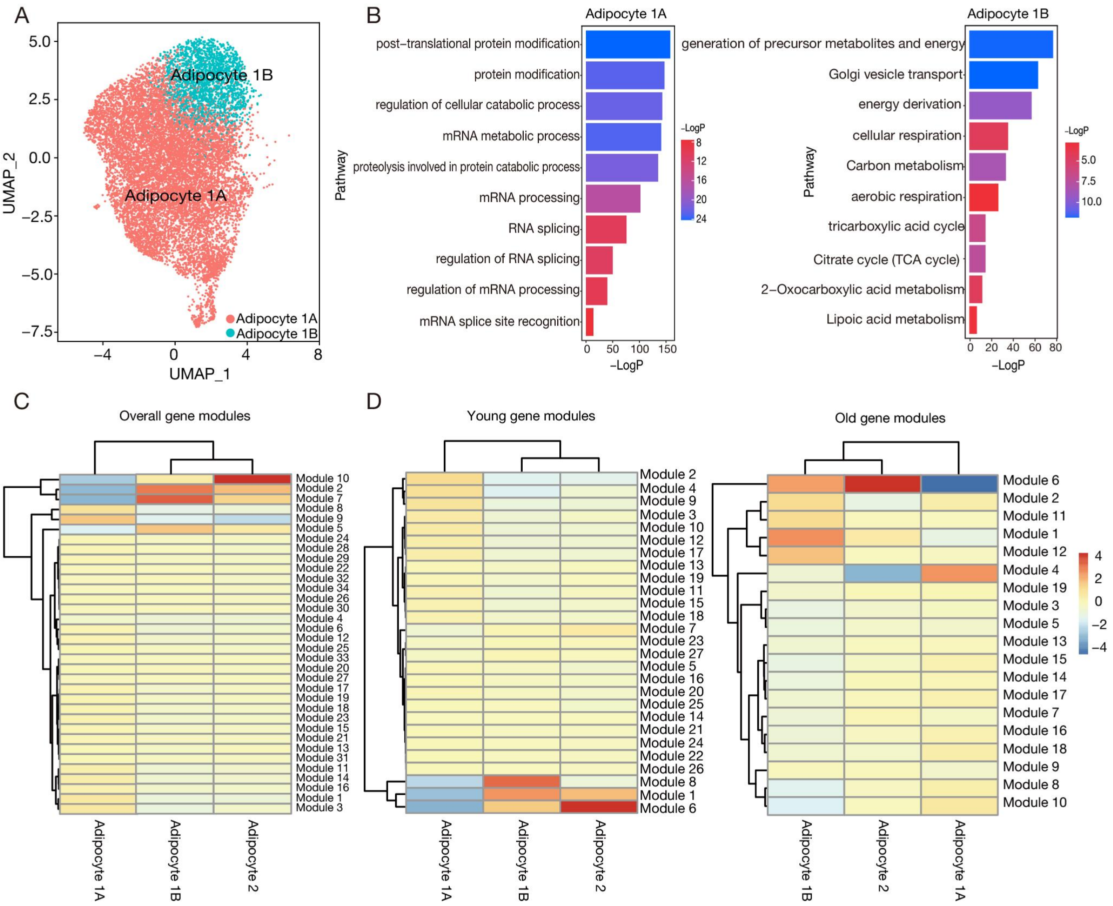

Figure S7. Validation and functional analysis of pseudotime differentiation trajectories in Adipocyte1 and Module genes in pseudo-temporal analysis of young and old mice. (A) Re-clustering was performed on Adipocyte1, annotating them as Adipocyte1A and Adipocyte1B. **(B)** This figure presents the enrichment analysis after re-clustering Adipocyte1. **(C)** Total gene module expression levels. **(D)** Module analysis performed to determine gene modules experiencing changes in the developmental trajectory of mouse adipocytes at 2 months and 26 months of age. Nineteen modules were presented in the 26-month-old mice, and 27 modules were presented in the 2-month-old mice, accompanied by expression intensities at each developmental stage. The color scale represents the normalized level of module gene expression.

Figure S8

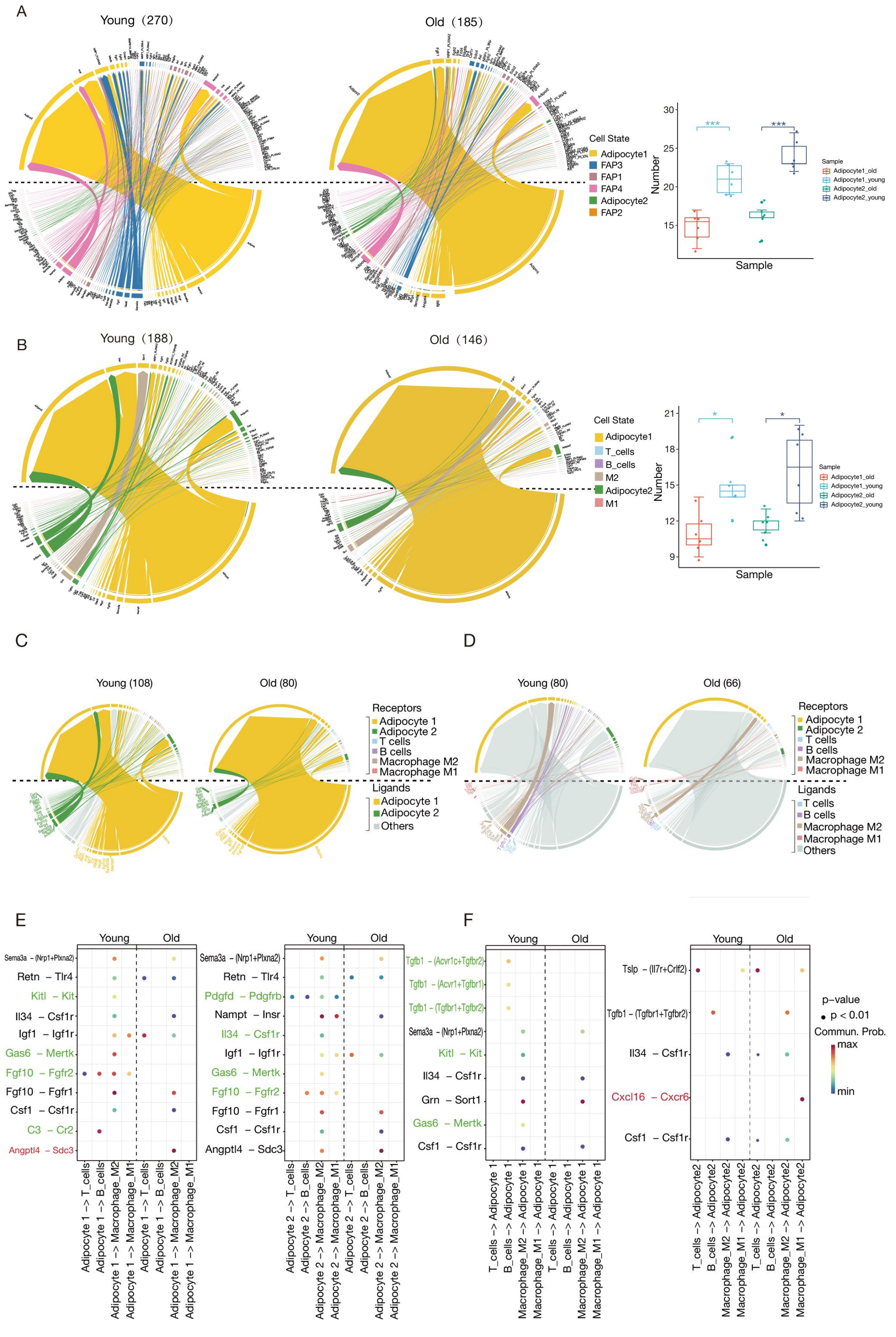
A
Young（270）
Old（185）
Cell State
Adipocyte1
FAP3
FAP1
FAP4
Adipocyte2
FAP2
Number
Sample
Adipocyte1_old
Adipocyte1_young
Adipocyte2_old
Adipocyte2_young
B
Young（188）
Old（146）
Cell State
Adipocyte1
T_cells
B_cells
M2
Adipocyte2
M1
C
Young (108)
Old (80)
Receptors
Adipocyte 1
Adipocyte 2
T cells
B cells
Macrophage M2
Macrophage M1
Ligands
Adipocyte 1
Adipocyte 2
Others
D
Young (80)
Old (66)
Receptors
Adipocyte 1
Adipocyte 2
T cells
B cells
Macrophage M2
Macrophage M1
Ligands
T cells
B cells
Macrophage M2
Macrophage M1
Others
E
Young
Old
Sema3a – (Nrp1+Plxna2)
Retn – Tlr4
Kitl – Kit
Il34 – Csf1r
Igf1 – Igf1r
Gas6 – Mertk
Fgf10 – Fgfr2
Fgf10 – Fgfr1
Csf1 – Csf1r
C3 – Cr2
Angptl4 – Sdc3
Adipocyte 1 –> T_cells
Adipocyte 1 –> B_cells
Adipocyte 1 –> Macrophage_M2
Adipocyte 1 –> Macrophage_M1
Pdgfd – Pdgfrb
Nampt – Insr
Adipocyte 2 –> T_cells
Adipocyte 2 –> B_cells
Adipocyte 2 –> Macrophage_M2
Adipocyte 2 –> Macrophage_M1
F
Tgfb1 – (Acvr1c+Tgfbr2)
Tgfb1 – (Acvr1+Tgfbr1)
Tgfb1 – (Tgfbr1+Tgfbr2)
Grn – Sort1
T_cells –> Adipocyte 1
B_cells –> Adipocyte 1
Macrophage_M2 –> Adipocyte 1
Macrophage_M1 –> Adipocyte 1
Tslp – (Il7r+Crlf2)
Tgfb1 – (Tgfbr1+Tgfbr2)
Il34 – Csf1r
Cxcl16 – Cxcr6
Csf1 – Csf1r
T_cells –> Adipocyte2
B_cells –> Adipocyte2
Macrophage_M2 –> Adipocyte2
Macrophage_M1 –> Adipocyte2
p–value
p < 0.01
Commun. Prob.
max
min

G

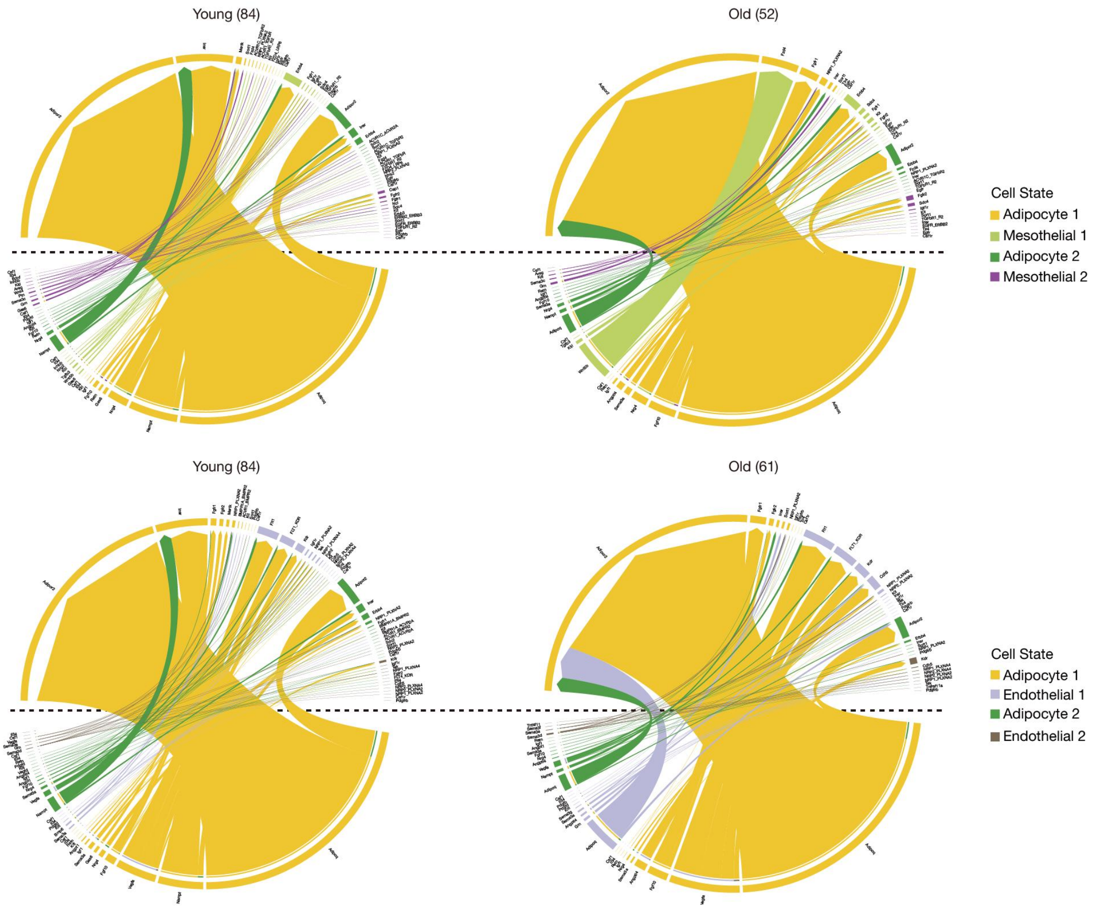

H

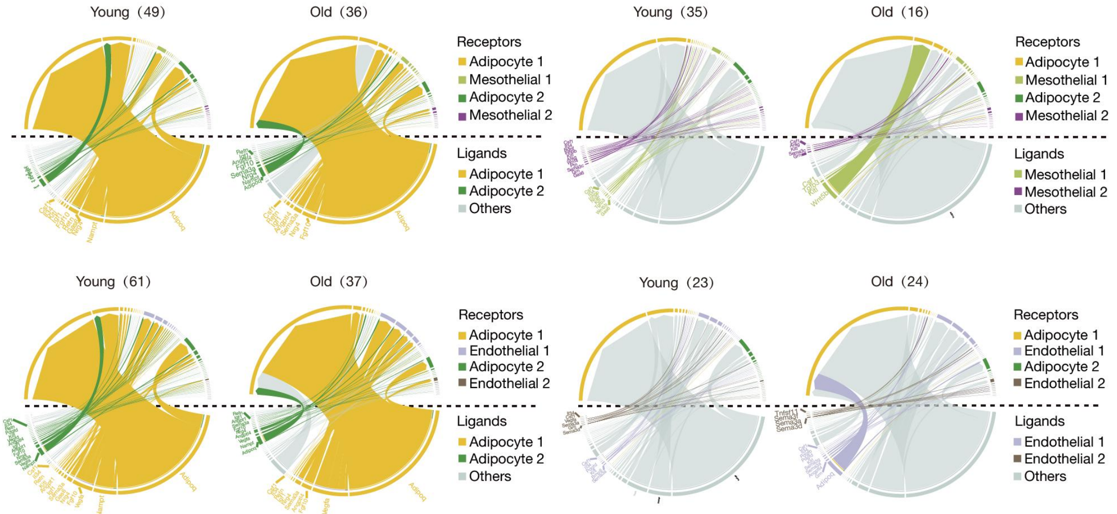

Figure S8. CCI analysis of adipocytes with immune cells, endothelial cells, and mesothelial cells. (A) Chord diagrams illustrating the interplay between adipocyte subpopulations and FAP subgroups,The right figure represents the statistical analysis of the significance of interactions between adipocytes and FAPs (fibro-adipogenic progenitors), showing a decrease in these interactions with aging. The right image is the saliency analysis of the interaction between adipocytes as ligands and receptors with FAPs. **(B)** Chord diagrams illustrating the interplay between adipocyte subpopulations and immune subgroups. The right image is the saliency

analysis of the interaction between adipocytes as ligands and receptors with immune cells. **(C)** Chord diagrams displaying intercellular L-R interactions in adipocyte subpopulations and immune cells in young (left) and old (right) mice. Differentially expressed ligands in adipocytes are below the dashed line, while receptors overexpressed in receiving cells (consisting of both adipocytes and immune cells) are located above. Communications from adipocytes (L) to FAPs (R) are identified in green and yellow, with other interactions indicated in gray. **(D)** Bubble charts illustrating normalized interaction scores between Adipocyte1 and immune cells in both young and old states. These interaction scores are calculated based on the level of ligand expression in Adipocyte1 and receptor expression in the receiving cells. Columns represent receiving cell types, while the rows signify predicted L-R pairs. Color scale indicates interaction strength, with higher scores indicating stronger cellular interaction. Red L-R pairs exist solely in young mice, while green pairs are unique to old mice. **(E)** Heatmaps of interaction scores demonstrating the interplay between ligands in Adipocyte2 and their receptors in FAPs. **(F)** Chord diagrams in young (left) and old (right) adipose tissues showing intercellular L-R interactions. Differentially expressed ligands in receiving immune cells are below the dashed line, while receptors overexpressed in adipocytes are located above. Communications from immune cells (L) to adipocytes (R) are prominently highlighted, while other interactions are indicated in gray. **(G)** Chord diagrams illustrating the interplay between adipocyte subpopulations and mesothelial/endothelial subgroups. **(H)** Chord diagrams illustrating intercellular L-R interactions in adipocyte subpopulations and mesothelial/endothelial cells in young (left) and old (right) mice.

Table S1-The total number of nuclei for sn-RNA sequencing

sample: Identifier for the sample being analyzed.
nCount RNA: The total number of RNA molecules counted in the sample.
nFeature: The number of distinct RNA features detected.
percent.mt: The percentage of RNA features derived from mitochondrial genes.
clusters: The assigned cluster number for the sample.
classifications: 'Singlet' indicates a single nucleus was identified.
celltype: The type of cell identified in the sample

sample	nCount	nFeature	percent.mt	clusters	classifications_	celltype
SAT_2M	1531	948	0.5867014	4	Singlet	Immune_cells
SAT_2M	1863	1052	1.0712373	0	Singlet	Adipocyte
SAT_2M	1296	782	0.231303	4	Singlet	Immune_cells
SAT_2M	1323	867	1.509434	4	Singlet	Immune_cells
SAT_2M	2118	1154	4.8493409	1	Singlet	Immune_cells
SAT_2M	1217	806	0.3270646	1	Singlet	Immune_cells
SAT_2M	2404	1265	1.038206	0	Singlet	Adipocyte
SAT_2M	1669	878	12.874252	9	Singlet	Mesothelial
SAT_2M	1538	977	2.3316062	5	Singlet	Immune_cells
SAT_2M	2145	1334	0.7834101	4	Singlet	Immune_cells
SAT_2M	2662	1456	0.5615874	0	Singlet	Adipocyte
SAT_2M	1902	1110	8.7526205	1	Singlet	Immune_cells
SAT_2M	1217	765	1.2305168	3	Singlet	FAPs
SAT_2M	1176	791	1.4358108	1	Singlet	Immune_cells
SAT_2M	1549	966	0.4513217	1	Singlet	Immune_cells
SAT_2M	3832	1937	10.09653	9	Singlet	Mesothelial
SAT_2M	1370	812	9.1836735	1	Singlet	Immune_cells
SAT_2M	1030	675	1.4520813	5	Singlet	Immune_cells
SAT_2M	2223	1371	0	5	Singlet	Immune_cells
SAT_2M	1451	889	5.4183813	4	Singlet	Immune_cells
SAT_2M	2535	1394	1.3752456	3	Singlet	FAPs
SAT_2M	2005	1140	2.4390244	1	Singlet	Immune_cells
SAT_2M	2118	1244	2.9190207	1	Singlet	Immune_cells
SAT_2M	1785	1014	6.6443328	4	Singlet	Immune_cells
SAT_2M	1651	998	3.3836858	1	Singlet	Immune_cells
SAT_2M	2010	1126	0.2483855	0	Singlet	Adipocyte
SAT_2M	1280	843	1.0911925	4	Singlet	Immune_cells
SAT_2M	1476	936	0.5398111	1	Singlet	Immune_cells
SAT_2M	1960	1132	4.1158537	1	Singlet	Immune_cells
SAT_2M	3054	1597	0.3583062	0	Singlet	Adipocyte
SAT_2M	1255	782	2.9968454	1	Singlet	Immune_cells
SAT_2M	1532	938	2.0833333	1	Singlet	Immune_cells
SAT_2M	1161	744	2.3156089	5	Singlet	Immune_cells
SAT_2M	1825	1027	1.474604	10	Singlet	Immune_cells
SAT_2M	1258	791	1.5079365	1	Singlet	Immune_cells
SAT_2M	1630	1031	0.244798	3	Singlet	FAPs
SAT_2M	1443	839	4.7487956	2	Singlet	FAPs
SAT_2M	1825	1086	3.7704918	1	Singlet	Immune_cells
SAT_2M	1317	828	2.5679758	4	Singlet	Immune_cells
SAT_2M	1411	909	0.4189944	0	Singlet	Adipocyte
SAT_2M	1097	738	2.8105168	7	Singlet	Endothelial_cells

SAT_2M	1987	1198	2.1042084	1	Singlet	Immune_cells
SAT_2M	2234	1354	0.4474273	11	Singlet	Smooth_muscle_cells
SAT_2M	5334	2449	6.4008984	9	Singlet	Mesothelial
SAT_2M	1514	861	4.5335085	10	Singlet	Immune_cells
SAT_2M	1088	732	1.8298262	4	Singlet	Immune_cells
SAT_2M	1090	708	0.8174387	4	Singlet	Immune_cells
SAT_2M	1718	1016	0.5803831	1	Singlet	Immune_cells
SAT_2M	1399	797	8.4939329	1	Singlet	Immune_cells
SAT_2M	1192	739	12.541806	5	Singlet	Immune_cells
SAT_2M	3726	1842	0.294275	0	Singlet	Adipocyte
SAT_2M	3489	1770	0.1430615	0	Singlet	Adipocyte
SAT_2M	1971	1105	1.5688259	1	Singlet	Immune_cells
SAT_2M	1112	717	10.286225	1	Singlet	Immune_cells
SAT_2M	1005	694	0.3944773	4	Singlet	Immune_cells
SAT_2M	1068	714	2.334267	1	Singlet	Immune_cells
SAT_2M	1159	729	0.5977797	0	Singlet	Adipocyte
SAT_2M	2147	1249	1.0175763	0	Singlet	Adipocyte
SAT_2M	1646	1037	5.1359517	1	Singlet	Immune_cells
SAT_2M	2523	1419	0.2761341	5	Singlet	Immune_cells
SAT_2M	1478	1012	1.3449899	4	Singlet	Immune_cells
SAT_2M	1225	818	6.1788618	1	Singlet	Immune_cells
SAT_2M	1797	1126	0.3320421	3	Singlet	FAPs
SAT_2M	1729	1010	2.4137931	3	Singlet	FAPs
SAT_2M	1831	1122	0.1629549	3	Singlet	FAPs
SAT_2M	1883	1150	2.0634921	1	Singlet	Immune_cells
SAT_2M	2417	1315	1.0699588	1	Singlet	Immune_cells
SAT_2M	2107	1171	4.0265277	1	Singlet	Immune_cells
SAT_2M	1546	901	1.674179	10	Singlet	Immune_cells
SAT_2M	1609	1052	0.6798517	4	Singlet	Immune_cells
SAT_2M	1253	772	8.9171975	1	Singlet	Immune_cells
SAT_2M	1282	767	5.7275542	1	Singlet	Immune_cells
SAT_2M	1277	769	7.788162	4	Singlet	Immune_cells
SAT_2M	1639	960	9.787234	4	Singlet	Immune_cells
SAT_2M	1269	778	1.0172144	0	Singlet	Adipocyte
SAT_2M	1415	900	0.2807018	4	Singlet	Immune_cells
SAT_2M	1609	1009	0.0619963	8	Singlet	Mesothelial
SAT_2M	1919	1117	8.4155844	4	Singlet	Immune_cells
SAT_2M	1781	1096	4.4667783	5	Singlet	Immune_cells
SAT_2M	1584	1056	0.1255493	4	Singlet	Immune_cells
SAT_2M	1892	1166	0.1578117	0	Singlet	Adipocyte
SAT_2M	1109	715	0.990099	5	Singlet	Immune_cells
SAT_2M	1064	706	0	0	Singlet	Adipocyte
SAT_2M	1359	779	9.3567251	1	Singlet	Immune_cells
SAT_2M	1483	947	0.8091706	4	Singlet	Immune_cells
SAT_2M	1505	905	1.6556291	7	Singlet	Endothelial_cells
SAT_2M	1620	900	13.131936	1	Singlet	Immune_cells
SAT_2M	2304	1338	3.9775184	3	Singlet	FAPs
SAT_2M	2439	1271	0.7352941	0	Singlet	Adipocyte
SAT_2M	1792	1082	0.2212389	0	Singlet	Adipocyte
SAT_2M	1381	951	0.9386282	4	Singlet	Immune_cells
SAT_2M	2025	1125	6.5356265	1	Singlet	Immune_cells

SAT_2M	1391	774	8.2437276	0	Singlet	Adipocyte
SAT_2M	1347	793	10.682493	1	Singlet	Immune_cells
SAT_2M	1945	1128	6.2980031	4	Singlet	Immune_cells
SAT_2M	2961	1508	0.1686341	0	Singlet	Adipocyte
SAT_2M	2320	1322	6.048906	5	Singlet	Immune_cells
SAT_2M	1134	773	1.2334802	4	Singlet	Immune_cells
SAT_2M	2195	1174	0.2723559	0	Singlet	Adipocyte
SAT_2M	1406	886	1.2031139	0	Singlet	Adipocyte
SAT_2M	2008	1100	2.0347395	5	Singlet	Immune_cells
SAT_2M	1294	752	9.4208494	1	Singlet	Immune_cells
SAT_2M	1336	841	7.0789866	1	Singlet	Immune_cells
SAT_2M	1705	1005	4.4950379	5	Singlet	Immune_cells
SAT_2M	1031	709	4.0501446	3	Singlet	FAPs
SAT_2M	1930	1167	0.4130098	0	Singlet	Adipocyte
SAT_2M	1299	846	4.765565	1	Singlet	Immune_cells
SAT_2M	1491	877	0.8026756	0	Singlet	Adipocyte
SAT_2M	1223	813	4	4	Singlet	Immune_cells
SAT_2M	6181	2172	1.3565891	10	Singlet	Immune_cells
SAT_2M	1295	830	0.4604758	1	Singlet	Immune_cells
SAT_2M	1506	919	4.7682119	1	Singlet	Immune_cells
SAT_2M	1375	889	0.1440922	0	Singlet	Adipocyte
SAT_2M	1145	757	0.0867303	1	Singlet	Immune_cells
SAT_2M	1303	856	0.1525553	8	Singlet	Mesothelial
SAT_2M	1780	1017	8.4404695	1	Singlet	Immune_cells
SAT_2M	1284	815	3.7267081	1	Singlet	Immune_cells
SAT_2M	2340	1368	0.383959	11	Singlet	Smooth_muscle_cells
SAT_2M	1003	674	0.1980198	0	Singlet	Adipocyte
SAT_2M	2129	1229	11.261682	9	Singlet	Mesothelial
SAT_2M	1193	789	0.5	1	Singlet	Immune_cells
SAT_2M	1087	737	3.3913841	4	Singlet	Immune_cells
SAT_2M	1199	777	2.7385892	3	Singlet	FAPs
SAT_2M	1125	728	1.2378426	3	Singlet	FAPs
SAT_2M	1297	813	0.2307692	4	Singlet	Immune_cells
SAT_2M	1807	1057	0.6615215	0	Singlet	Adipocyte
SAT_2M	1166	786	2.9838022	1	Singlet	Immune_cells
SAT_2M	1773	1056	5.3872054	1	Singlet	Immune_cells
SAT_2M	2148	1226	1.0697674	10	Singlet	Immune_cells
SAT_2M	1295	814	3.3742331	1	Singlet	Immune_cells
SAT_2M	1272	824	5.7632399	4	Singlet	Immune_cells
SAT_2M	1343	787	12.509252	1	Singlet	Immune_cells
SAT_2M	1289	804	0.7716049	4	Singlet	Immune_cells
SAT_2M	1056	689	7.6850984	1	Singlet	Immune_cells
SAT_2M	1178	745	0.0844595	1	Singlet	Immune_cells
SAT_2M	3300	1792	5.5186972	5	Singlet	Immune_cells
SAT_2M	2938	1601	8.5142469	4	Singlet	Immune_cells
SAT_2M	1559	967	0.1914486	4	Singlet	Immune_cells
SAT_2M	1426	971	0.209351	7	Singlet	Endothelial_cells
SAT_2M	1706	994	1.7554125	4	Singlet	Immune_cells
SAT_2M	2820	1493	0.0352983	0	Singlet	Adipocyte
SAT_2M	1069	691	0.5591799	3	Singlet	FAPs
SAT_2M	1677	972	5.9630292	1	Singlet	Immune_cells

SAT_2M	1674	1040	0.4753417	0	Singlet	Adipocyte
SAT_2M	1655	1054	1.9173158	1	Singlet	Immune_cells
SAT_2M	3432	1724	9.0724048	9	Singlet	Mesothelial
SAT_2M	1589	950	0.3757044	0	Singlet	Adipocyte
SAT_2M	1669	1070	0.8338297	5	Singlet	Immune_cells
SAT_2M	1174	767	0.2533784	4	Singlet	Immune_cells
SAT_2M	1100	714	0.0908265	1	Singlet	Immune_cells
SAT_2M	1833	1074	0.2162162	0	Singlet	Adipocyte
SAT_2M	1508	874	5.5555556	1	Singlet	Immune_cells
SAT_2M	1397	887	1.2108262	4	Singlet	Immune_cells
SAT_2M	3284	1630	0.2425713	0	Singlet	Adipocyte
SAT_2M	1902	1121	5.987395	1	Singlet	Immune_cells
SAT_2M	1349	855	1.0294118	6	Singlet	Neurons
SAT_2M	1693	963	10.67217	1	Singlet	Immune_cells
SAT_2M	1042	697	2.6819923	1	Singlet	Immune_cells
SAT_2M	1317	806	2.5583145	1	Singlet	Immune_cells
SAT_2M	1599	995	0.4361371	3	Singlet	FAPs
SAT_2M	1221	793	2.7777778	9	Singlet	Mesothelial
SAT_2M	1247	791	0.3187251	4	Singlet	Immune_cells
SAT_2M	3859	1871	3.0232558	0	Singlet	Adipocyte
SAT_2M	2609	1433	3.2146958	4	Singlet	Immune_cells
SAT_2M	1092	750	0.6398537	4	Singlet	Immune_cells
SAT_2M	1347	854	4.9926579	1	Singlet	Immune_cells
SAT_2M	1451	840	7.3287671	1	Singlet	Immune_cells
SAT_2M	3309	1577	2.5339367	3	Singlet	FAPs
SAT_2M	5207	2360	6.7725273	9	Singlet	Mesothelial
SAT_2M	1352	827	0.7374631	0	Singlet	Adipocyte
SAT_2M	2431	1391	0.4498978	0	Singlet	Adipocyte
SAT_2M	4498	2180	1.7559458	9	Singlet	Mesothelial
SAT_2M	1394	906	4.5681656	1	Singlet	Immune_cells
SAT_2M	1239	790	0.4830918	4	Singlet	Immune_cells
SAT_2M	1231	770	2.6699029	2	Singlet	FAPs
SAT_2M	2973	1576	0.6688963	0	Singlet	Adipocyte
SAT_2M	6871	2883	4.6920395	9	Singlet	Mesothelial
SAT_2M	1031	732	0.3868472	4	Singlet	Immune_cells
SAT_2M	3126	1589	0.7019783	0	Singlet	Adipocyte
SAT_2M	3878	2046	0.3581479	9	Singlet	Mesothelial
SAT_2M	1650	1044	0.6027728	8	Singlet	Mesothelial
SAT_2M	1273	808	1.1755486	1	Singlet	Immune_cells
SAT_2M	1126	739	0.4413063	4	Singlet	Immune_cells
SAT_2M	1507	910	1.9205298	4	Singlet	Immune_cells
SAT_2M	1820	1113	3.2876712	8	Singlet	Mesothelial
SAT_2M	1428	926	0.2084781	2	Singlet	FAPs
SAT_2M	1240	865	0.32	0	Singlet	Adipocyte
SAT_2M	1217	826	0.5700326	5	Singlet	Immune_cells
SAT_2M	3329	1706	0.1796945	8	Singlet	Mesothelial
SAT_2M	1351	848	0.2177068	0	Singlet	Adipocyte
SAT_2M	1968	1139	3.4954407	1	Singlet	Immune_cells
SAT_2M	1502	917	2.3793787	1	Singlet	Immune_cells
SAT_2M	1415	949	0.5625879	4	Singlet	Immune_cells
SAT_2M	2581	1454	0.1539646	0	Singlet	Adipocyte

SAT_2M	1219	774	0.6525285	1	Singlet	Immune_cells
SAT_2M	1204	752	1.3266998	0	Singlet	Adipocyte
SAT_2M	1284	810	1.4717273	0	Singlet	Adipocyte
SAT_2M	2044	1176	5.8479532	1	Singlet	Immune_cells
SAT_2M	1406	857	4.7282992	5	Singlet	Immune_cells
SAT_2M	1622	1040	1.8971848	9	Singlet	Mesothelial
SAT_2M	1581	1000	2.5769956	1	Singlet	Immune_cells
SAT_2M	1537	950	7.3882048	4	Singlet	Immune_cells
SAT_2M	1762	1041	4.1406693	1	Singlet	Immune_cells
SAT_2M	1307	906	2.1276596	1	Singlet	Immune_cells
SAT_2M	1012	691	0.2952756	3	Singlet	FAPs
SAT_2M	1832	1081	3.8147139	1	Singlet	Immune_cells
SAT_2M	1066	720	2.0579981	4	Singlet	Immune_cells
SAT_2M	1463	885	4.8233696	4	Singlet	Immune_cells
SAT_2M	1113	701	0.3571429	2	Singlet	FAPs
SAT_2M	1375	879	0.1448226	1	Singlet	Immune_cells
SAT_2M	1017	679	1.2720157	5	Singlet	Immune_cells
SAT_2M	1435	883	0.4861111	1	Singlet	Immune_cells
SAT_2M	1039	758	0.7611798	1	Singlet	Immune_cells
SAT_2M	2163	1174	6.7188219	0	Singlet	Adipocyte
SAT_2M	2307	1235	2.5108225	0	Singlet	Adipocyte
SAT_2M	1849	1190	3.0645161	4	Singlet	Immune_cells
SAT_2M	1210	753	2.7138158	4	Singlet	Immune_cells
SAT_2M	1297	811	0.3843198	1	Singlet	Immune_cells
SAT_2M	1291	870	0.1544402	4	Singlet	Immune_cells
SAT_2M	1443	875	1.3793103	4	Singlet	Immune_cells
SAT_2M	1032	643	5.3191489	2	Singlet	FAPs
SAT_2M	3145	1661	1.2330066	0	Singlet	Adipocyte
SAT_2M	1856	1142	4.3571813	1	Singlet	Immune_cells
SAT_2M	1140	755	1.6608392	1	Singlet	Immune_cells
SAT_2M	1731	1037	3.4005764	0	Singlet	Adipocyte
SAT_2M	1012	594	2.2571148	10	Singlet	Immune_cells
SAT_2M	2017	1239	0.148075	8	Singlet	Mesothelial
SAT_2M	1469	882	10.794297	1	Singlet	Immune_cells
SAT_2M	1333	881	0.2998501	1	Singlet	Immune_cells
SAT_2M	1482	954	0.4024145	4	Singlet	Immune_cells
SAT_2M	1444	892	0.7596685	0	Singlet	Adipocyte
SAT_2M	1655	990	0.1806141	2	Singlet	FAPs
SAT_2M	3583	1820	0.9460211	0	Singlet	Adipocyte
SAT_2M	1664	1019	0.7751938	0	Singlet	Adipocyte
SAT_2M	1924	1113	2.8940568	1	Singlet	Immune_cells
SAT_2M	1320	776	2.5738077	0	Singlet	Adipocyte
SAT_2M	1880	1120	0.4253057	4	Singlet	Immune_cells
SAT_2M	3385	1709	0.5306604	3	Singlet	FAPs
SAT_2M	1393	861	9.5204009	1	Singlet	Immune_cells
SAT_2M	2937	1437	12.317115	9	Singlet	Mesothelial
SAT_2M	2104	1267	2.1306818	4	Singlet	Immune_cells
SAT_2M	1358	835	1.3225569	0	Singlet	Adipocyte
SAT_2M	1237	793	1.1226945	1	Singlet	Immune_cells
SAT_2M	1040	677	1.1472275	1	Singlet	Immune_cells
SAT_2M	4425	1750	1.9183029	10	Singlet	Immune_cells

SAT_2M	1608	1034	1.8610422	1	Singlet	Immune_cells
SAT_2M	1627	1011	2.7658267	4	Singlet	Immune_cells
SAT_2M	1647	971	0.7874016	0	Singlet	Adipocyte
SAT_2M	1659	1049	0.2402402	4	Singlet	Immune_cells
SAT_2M	1245	788	0.7171315	4	Singlet	Immune_cells
SAT_2M	1418	909	1.2658228	4	Singlet	Immune_cells
SAT_2M	1662	1021	0.9580838	4	Singlet	Immune_cells
SAT_2M	2625	1483	0.5311077	0	Singlet	Adipocyte
SAT_2M	1400	898	0.9957326	4	Singlet	Immune_cells
SAT_2M	1279	853	1.5588465	11	Singlet	Smooth_muscle_cells
SAT_2M	1555	980	4.4117647	1	Singlet	Immune_cells
SAT_2M	1347	777	0.1477105	1	Singlet	Immune_cells
SAT_2M	2303	1281	2.5917927	10	Singlet	Immune_cells
SAT_2M	2455	1268	2.1553477	0	Singlet	Adipocyte
SAT_2M	1647	996	1.0253317	1	Singlet	Immune_cells
SAT_2M	2196	1289	2.8959276	1	Singlet	Immune_cells
SAT_2M	2200	1231	7.1169538	1	Singlet	Immune_cells
SAT_2M	1242	853	0.2392345	4	Singlet	Immune_cells
SAT_2M	3457	1844	0.547235	11	Singlet	Smooth_muscle_cells
SAT_2M	1086	732	5.1771117	1	Singlet	Immune_cells
SAT_2M	1266	798	0.4720692	1	Singlet	Immune_cells
SAT_2M	1442	921	0.2061856	1	Singlet	Immune_cells
SAT_2M	1388	920	1.2160229	4	Singlet	Immune_cells
SAT_2M	1636	1040	3.6407767	1	Singlet	Immune_cells
SAT_2M	2205	1229	0.1810774	0	Singlet	Adipocyte
SAT_2M	1767	1004	2.1934758	0	Singlet	Adipocyte
SAT_2M	2766	1569	8.9305005	4	Singlet	Immune_cells
SAT_2M	1325	839	5.7938299	1	Singlet	Immune_cells
SAT_2M	1245	780	0.5608974	0	Singlet	Adipocyte
SAT_2M	7694	3262	12.18531	9	Singlet	Mesothelial
SAT_2M	1421	877	2.6499303	1	Singlet	Immune_cells
SAT_2M	1236	772	4.0355125	1	Singlet	Immune_cells
SAT_2M	1213	787	1.7170891	9	Singlet	Mesothelial
SAT_2M	1070	707	1.5668203	1	Singlet	Immune_cells
SAT_2M	1288	875	1.2355212	1	Singlet	Immune_cells
SAT_2M	1499	979	2.2546419	9	Singlet	Mesothelial
SAT_2M	1506	868	5.2840159	1	Singlet	Immune_cells
SAT_2M	1157	739	1.9742489	5	Singlet	Immune_cells
SAT_2M	1113	721	3.9320822	1	Singlet	Immune_cells
SAT_2M	1952	1030	4.7546012	1	Singlet	Immune_cells
SAT_2M	1810	1026	0.5506608	0	Singlet	Adipocyte
SAT_2M	2303	1349	0.0866176	3	Singlet	FAPs
SAT_2M	1637	944	1.459854	0	Singlet	Adipocyte
SAT_2M	3358	1869	1.0989011	9	Singlet	Mesothelial
SAT_2M	1307	850	2.1292776	11	Singlet	Smooth_muscle_cells
SAT_2M	1975	1190	2.7300303	1	Singlet	Immune_cells
SAT_2M	1686	1035	2.4778761	1	Singlet	Immune_cells
SAT_2M	1833	1095	0.1078167	0	Singlet	Adipocyte
SAT_2M	1006	696	0.9803922	6	Singlet	Neurons
SAT_2M	4640	2035	0.8165019	0	Singlet	Adipocyte
SAT_2M	1018	671	0.2938296	1	Singlet	Immune_cells

SAT_2M	2790	1452	0.8950949	0	Singlet	Adipocyte
SAT_2M	1019	669	0.8789063	0	Singlet	Adipocyte
SAT_2M	1197	810	2.0678246	1	Singlet	Immune_cells
SAT_2M	3824	1869	4.6226169	9	Singlet	Mesothelial
SAT_2M	1449	813	2.4725275	0	Singlet	Adipocyte
SAT_2M	1960	1083	11.788618	4	Singlet	Immune_cells
SAT_2M	1159	726	8.5197935	5	Singlet	Immune_cells
SAT_2M	1867	1138	3.4777956	1	Singlet	Immune_cells
SAT_2M	1299	811	0.690184	4	Singlet	Immune_cells
SAT_2M	2323	1257	5.9151307	1	Singlet	Immune_cells
SAT_2M	3348	1885	0.5970149	5	Singlet	Immune_cells
SAT_2M	5069	2313	0.8451258	2	Singlet	FAPs
SAT_2M	1086	726	0.2744739	0	Singlet	Adipocyte
SAT_2M	1106	677	5.7502246	10	Singlet	Immune_cells
SAT_2M	1420	886	0.5594406	1	Singlet	Immune_cells
SAT_2M	1090	752	0.7319305	1	Singlet	Immune_cells
SAT_2M	1324	898	0.2996255	4	Singlet	Immune_cells
SAT_2M	1217	738	0.1642036	0	Singlet	Adipocyte
SAT_2M	1786	1089	5.4038997	1	Singlet	Immune_cells
SAT_2M	1281	820	3.7267081	1	Singlet	Immune_cells
SAT_2M	1535	902	11.860013	4	Singlet	Immune_cells
SAT_2M	2545	1414	3.7211124	5	Singlet	Immune_cells
SAT_2M	1723	1062	5.5523424	1	Singlet	Immune_cells
SAT_2M	1270	852	0.2351097	1	Singlet	Immune_cells
SAT_2M	1242	808	2.4859663	1	Singlet	Immune_cells
SAT_2M	1484	873	0.134499	0	Singlet	Adipocyte
SAT_2M	8726	2692	1.9784995	10	Singlet	Immune_cells
SAT_2M	1427	895	0.2777778	0	Singlet	Adipocyte
SAT_2M	1223	794	0.0814996	4	Singlet	Immune_cells
SAT_2M	2090	1207	2.9957204	0	Singlet	Adipocyte
SAT_2M	2237	1277	9.6888889	9	Singlet	Mesothelial
SAT_2M	1767	1049	7.9706049	1	Singlet	Immune_cells
SAT_2M	3473	1739	0.3156385	0	Singlet	Adipocyte
SAT_2M	1190	763	3.2554257	3	Singlet	FAPs
SAT_2M	1248	768	4.24	1	Singlet	Immune_cells
SAT_2M	1863	1070	4.4919786	1	Singlet	Immune_cells
SAT_2M	5930	2763	5.8229552	9	Singlet	Mesothelial
SAT_2M	2160	1256	3.0009234	1	Singlet	Immune_cells
SAT_2M	2460	1335	1.4592623	0	Singlet	Adipocyte
SAT_2M	2077	1137	2.401537	0	Singlet	Adipocyte
SAT_2M	2489	1543	4.2433947	4	Singlet	Immune_cells
SAT_2M	1412	942	0.5649718	4	Singlet	Immune_cells
SAT_2M	3097	1524	1.8333869	10	Singlet	Immune_cells
SAT_2M	1056	727	1.2275732	4	Singlet	Immune_cells
SAT_2M	1083	714	0.3686636	4	Singlet	Immune_cells
SAT_2M	1263	735	5.6872038	1	Singlet	Immune_cells
SAT_2M	1779	1056	4.6498599	1	Singlet	Immune_cells
SAT_2M	1628	989	2.0158827	1	Singlet	Immune_cells
SAT_2M	1694	1023	0.5896226	4	Singlet	Immune_cells
SAT_2M	1003	674	0	4	Singlet	Immune_cells
SAT_2M	1220	800	5.4693878	1	Singlet	Immune_cells

SAT_2M	1677	1063	2.4390244	4	Singlet	Immune_cells
SAT_2M	1612	960	1.8552876	1	Singlet	Immune_cells
SAT_2M	3127	1570	0.1909004	0	Singlet	Adipocyte
SAT_2M	1368	853	1.9536903	0	Singlet	Adipocyte
SAT_2M	1286	864	0.3093581	5	Singlet	Immune_cells
SAT_2M	15519	5208	9.6346797	9	Singlet	Mesothelial
SAT_2M	2864	1461	10.19485	4	Singlet	Immune_cells
SAT_2M	1490	931	1.9385027	4	Singlet	Immune_cells
SAT_2M	1837	1180	0.3252033	0	Singlet	Adipocyte
SAT_2M	1285	819	1.0054138	0	Singlet	Adipocyte
SAT_2M	1496	951	0.9271523	0	Singlet	Adipocyte
SAT_2M	1694	1089	0.1763668	5	Singlet	Immune_cells
SAT_2M	1212	803	0.9009009	5	Singlet	Immune_cells
SAT_2M	1238	770	0.7246377	1	Singlet	Immune_cells
SAT_2M	1244	795	3.6888532	1	Singlet	Immune_cells
SAT_2M	1259	783	0.8675079	0	Singlet	Adipocyte
SAT_2M	1075	735	0.462963	4	Singlet	Immune_cells
SAT_2M	1068	706	0.1865672	4	Singlet	Immune_cells
SAT_2M	1583	935	4.3588124	8	Singlet	Mesothelial
SAT_2M	2336	1313	1.4925373	0	Singlet	Adipocyte
SAT_2M	1136	722	5.7591623	1	Singlet	Immune_cells
SAT_2M	1309	838	0.3810976	5	Singlet	Immune_cells
SAT_2M	1493	977	1.2040134	4	Singlet	Immune_cells
SAT_2M	1201	785	10.13289	1	Singlet	Immune_cells
SAT_2M	1417	886	0.4225352	1	Singlet	Immune_cells
SAT_2M	1702	1079	0.234192	4	Singlet	Immune_cells
SAT_2M	3619	1754	4.1563446	1	Singlet	Immune_cells
SAT_2M	1035	703	0.7707129	4	Singlet	Immune_cells
SAT_2M	1783	1098	0.5030743	4	Singlet	Immune_cells
SAT_2M	1459	911	8.1395349	1	Singlet	Immune_cells
SAT_2M	1796	1003	7.8420467	1	Singlet	Immune_cells
SAT_2M	1085	730	2.8233151	4	Singlet	Immune_cells
SAT_2M	3600	1712	4.8766971	10	Singlet	Immune_cells
SAT_2M	1182	762	2.10615	4	Singlet	Immune_cells
SAT_2M	1634	987	8.7697929	1	Singlet	Immune_cells
SAT_2M	1409	844	6.3165366	1	Singlet	Immune_cells
SAT_2M	1743	1061	1.6561965	5	Singlet	Immune_cells
SAT_2M	1763	1067	1.1293055	0	Singlet	Adipocyte
SAT_2M	1826	1149	0.3825137	9	Singlet	Mesothelial
SAT_2M	1319	777	4.4663134	1	Singlet	Immune_cells
SAT_2M	1261	844	2.8391167	4	Singlet	Immune_cells
SAT_2M	1183	778	0.084317	4	Singlet	Immune_cells
SAT_2M	1827	1064	8.4514722	4	Singlet	Immune_cells
SAT_2M	1466	831	7.0556309	1	Singlet	Immune_cells
SAT_2M	8231	2445	2.0990051	10	Singlet	Immune_cells
SAT_2M	1979	1142	6.3476071	1	Singlet	Immune_cells
SAT_2M	1165	759	0.3412969	0	Singlet	Adipocyte
SAT_2M	2045	1165	6.7284252	4	Singlet	Immune_cells
SAT_2M	2326	1294	3.2175032	0	Singlet	Adipocyte
SAT_2M	1192	763	0.2502085	1	Singlet	Immune_cells
SAT_2M	1815	1062	0.219901	3	Singlet	FAPs

SAT_2M	1392	881	3.8020086	3	Singlet	FAPs
SAT_2M	1825	1079	0.4352557	0	Singlet	Adipocyte
SAT_2M	4144	1828	1.9937545	10	Singlet	Immune_cells
SAT_2M	3717	1766	0.993022	0	Singlet	Adipocyte
SAT_2M	1429	933	0.4884857	0	Singlet	Adipocyte
SAT_2M	1057	650	12.45283	9	Singlet	Mesothelial
SAT_2M	1309	788	1.0678871	4	Singlet	Immune_cells
SAT_2M	1157	751	0.0859845	1	Singlet	Immune_cells
SAT_2M	1400	894	7.8947368	1	Singlet	Immune_cells
SAT_2M	1234	784	5.4119548	4	Singlet	Immune_cells
SAT_2M	1263	767	7.9527559	1	Singlet	Immune_cells
SAT_2M	1105	732	8.4532374	1	Singlet	Immune_cells
SAT_2M	1025	652	1.2670565	0	Singlet	Adipocyte
SAT_2M	1962	1160	0.8109478	0	Singlet	Adipocyte
SAT_2M	2128	1230	0.8415147	0	Singlet	Adipocyte
SAT_2M	1793	1105	2.729805	4	Singlet	Immune_cells
SAT_2M	1596	850	5.6320401	9	Singlet	Mesothelial
SAT_2M	1282	823	3.0373832	1	Singlet	Immune_cells
SAT_2M	1394	885	0.3563792	4	Singlet	Immune_cells
SAT_2M	1558	923	2.1766965	1	Singlet	Immune_cells
SAT_2M	1007	685	1.7786561	8	Singlet	Mesothelial
SAT_2M	1253	795	4.0669856	4	Singlet	Immune_cells
SAT_2M	1105	723	1.0820559	5	Singlet	Immune_cells
SAT_2M	1256	824	0.1582278	5	Singlet	Immune_cells
SAT_2M	3793	1914	1.7100763	9	Singlet	Mesothelial
SAT_2M	1391	885	3.0692363	1	Singlet	Immune_cells
SAT_2M	1695	1013	3.4685479	5	Singlet	Immune_cells
SAT_2M	1335	840	0.6726457	0	Singlet	Adipocyte
SAT_2M	1246	843	1.9857029	1	Singlet	Immune_cells
SAT_2M	1726	1010	1.0368664	3	Singlet	FAPs
SAT_2M	1422	1053	1.0238908	6	Singlet	Neurons
SAT_2M	1606	1032	0.1863354	5	Singlet	Immune_cells
SAT_2M	1247	797	0.6389776	0	Singlet	Adipocyte
SAT_2M	1361	781	0.8810573	1	Singlet	Immune_cells
SAT_2M	1095	702	0.8211679	10	Singlet	Immune_cells
SAT_2M	1785	1069	1.8404908	0	Singlet	Adipocyte
SAT_2M	1222	772	1.9543974	1	Singlet	Immune_cells
SAT_2M	1341	861	0.296077	1	Singlet	Immune_cells
SAT_2M	1016	649	0.686948	1	Singlet	Immune_cells
SAT_2M	1404	882	4.9715909	1	Singlet	Immune_cells
SAT_2M	1287	856	2.7131783	4	Singlet	Immune_cells
SAT_2M	1646	973	0.4244997	1	Singlet	Immune_cells
SAT_2M	1730	1060	2.6528258	3	Singlet	FAPs
SAT_2M	2884	1477	1.8307427	3	Singlet	FAPs
SAT_2M	1719	1052	0.4062681	4	Singlet	Immune_cells
SAT_2M	6012	2846	3.1068284	9	Singlet	Mesothelial
SAT_2M	1095	801	0.2659574	4	Singlet	Immune_cells
SAT_2M	1100	719	0.1813237	4	Singlet	Immune_cells
SAT_2M	1151	731	2.4242424	0	Singlet	Adipocyte
SAT_2M	1004	624	0.29615	4	Singlet	Immune_cells
SAT_2M	1116	701	0.6233304	1	Singlet	Immune_cells

SAT_2M	2523	1422	0.4748714	3	Singlet	FAPs
SAT_2M	1325	763	1.3544018	10	Singlet	Immune_cells
SAT_2M	1293	848	4.3143297	1	Singlet	Immune_cells
SAT_2M	1087	709	1.3686131	4	Singlet	Immune_cells
SAT_2M	1306	877	1.9832189	4	Singlet	Immune_cells
SAT_2M	1053	723	0.5617978	1	Singlet	Immune_cells
SAT_2M	2411	1323	0.0413565	8	Singlet	Mesothelial
SAT_2M	2968	1451	0.7733692	0	Singlet	Adipocyte
SAT_2M	3823	1879	0.4702194	11	Singlet	Smooth_muscle_cells
SAT_2M	1100	732	3.8949275	1	Singlet	Immune_cells
SAT_2M	1924	1150	0.6195147	1	Singlet	Immune_cells
SAT_2M	1213	809	0.5733006	5	Singlet	Immune_cells
SAT_2M	1320	858	2.3255814	1	Singlet	Immune_cells
SAT_2M	1934	1080	4.7227926	0	Singlet	Adipocyte
SAT_2M	1191	749	0.9205021	0	Singlet	Adipocyte
SAT_2M	1797	1073	2.8872848	2	Singlet	FAPs
SAT_2M	1892	1154	0.5789474	0	Singlet	Adipocyte
SAT_2M	1427	814	8.4958217	1	Singlet	Immune_cells
SAT_2M	1887	1131	1.9545695	0	Singlet	Adipocyte
SAT_2M	1229	722	9.1720779	10	Singlet	Immune_cells
SAT_2M	2886	1608	0.3791796	0	Singlet	Adipocyte
SAT_2M	1270	808	4.7058824	6	Singlet	Neurons
SAT_2M	1915	1212	0.8831169	4	Singlet	Immune_cells
SAT_2M	1371	869	1.9650655	1	Singlet	Immune_cells
SAT_2M	1011	658	3.2576505	1	Singlet	Immune_cells
SAT_2M	1304	813	0.3825555	1	Singlet	Immune_cells
SAT_2M	3232	1658	11.066461	9	Singlet	Mesothelial
SAT_2M	2172	1288	0.2751032	0	Singlet	Adipocyte
SAT_2M	1267	869	0.7836991	1	Singlet	Immune_cells
SAT_2M	1369	878	0.2184996	1	Singlet	Immune_cells
SAT_2M	1265	793	4.4952681	1	Singlet	Immune_cells
SAT_2M	1005	669	0.7928642	1	Singlet	Immune_cells
SAT_2M	1321	834	7.5882795	1	Singlet	Immune_cells
SAT_2M	1013	695	1.5701668	4	Singlet	Immune_cells
SAT_2M	1371	831	4.3572985	4	Singlet	Immune_cells
SAT_2M	1263	835	0.5507474	1	Singlet	Immune_cells
SAT_2M	1667	1083	0.7751938	5	Singlet	Immune_cells
SAT_2M	1484	932	0.7372654	0	Singlet	Adipocyte
SAT_2M	1522	955	0.7203667	0	Singlet	Adipocyte
SAT_2M	1033	672	0.1936108	4	Singlet	Immune_cells
SAT_2M	4790	2017	3.2554257	10	Singlet	Immune_cells
SAT_2M	2331	1397	3.5912783	1	Singlet	Immune_cells
SAT_2M	1543	959	0.1936733	4	Singlet	Immune_cells
SAT_2M	1960	1338	0.2024292	4	Singlet	Immune_cells
SAT_2M	2911	1551	4.5953361	1	Singlet	Immune_cells
SAT_2M	4731	2168	11.825464	9	Singlet	Mesothelial
SAT_2M	3489	1657	8.8386728	0	Singlet	Adipocyte
SAT_2M	1661	911	6.25	9	Singlet	Mesothelial
SAT_2M	1071	712	3.8033395	1	Singlet	Immune_cells
SAT_2M	1128	772	0.530504	1	Singlet	Immune_cells
SAT_2M	1305	859	1.606733	1	Singlet	Immune_cells

SAT_2M	1035	707	0.1924928	1	Singlet	Immune_cells
SAT_2M	1351	822	2.5868441	10	Singlet	Immune_cells
SAT_2M	2186	1203	10.255242	4	Singlet	Immune_cells
SAT_2M	1513	960	2.2442244	4	Singlet	Immune_cells
SAT_2M	1705	1088	0.4672897	5	Singlet	Immune_cells
SAT_2M	1221	741	5.400982	5	Singlet	Immune_cells
SAT_2M	1801	1076	2.4916944	4	Singlet	Immune_cells
SAT_2M	1499	851	2.1150033	0	Singlet	Adipocyte
SAT_2M	1077	717	0.1831502	4	Singlet	Immune_cells
SAT_2M	1105	720	3.060306	4	Singlet	Immune_cells
SAT_2M	1286	819	5.1162791	1	Singlet	Immune_cells
SAT_2M	1914	1064	4.8462741	0	Singlet	Adipocyte
SAT_2M	1553	933	6.3665595	1	Singlet	Immune_cells
SAT_2M	1737	1035	7.466973	9	Singlet	Mesothelial
SAT_2M	1316	872	0.8345979	0	Singlet	Adipocyte
SAT_2M	1411	934	0.2111189	0	Singlet	Adipocyte
SAT_2M	1831	1125	0.1084599	0	Singlet	Adipocyte
SAT_2M	1490	949	3.1417112	1	Singlet	Immune_cells
SAT_2M	1744	1030	4	4	Singlet	Immune_cells
SAT_2M	1656	998	7.2158749	4	Singlet	Immune_cells
SAT_2M	1654	986	3.3816425	1	Singlet	Immune_cells
SAT_2M	1970	1166	0.2026342	0	Singlet	Adipocyte
SAT_2M	1494	911	4.5393858	1	Singlet	Immune_cells
SAT_2M	1865	1146	9.1586794	1	Singlet	Immune_cells
SAT_2M	1403	880	0.2828854	0	Singlet	Adipocyte
SAT_2M	1263	809	5.6082149	1	Singlet	Immune_cells
SAT_2M	1292	802	4.6707504	9	Singlet	Mesothelial
SAT_2M	3758	1676	1.9123506	10	Singlet	Immune_cells
SAT_2M	1229	768	0.2435065	0	Singlet	Adipocyte
SAT_2M	1039	660	0.6724304	10	Singlet	Immune_cells
SAT_2M	1471	798	9.4493542	9	Singlet	Mesothelial
SAT_2M	1255	801	0.9538951	4	Singlet	Immune_cells
SAT_2M	1630	994	4.642639	4	Singlet	Immune_cells
SAT_2M	1984	1243	0.35	3	Singlet	FAPs
SAT_2M	3328	1709	1.9507803	9	Singlet	Mesothelial
SAT_2M	1847	1130	1.2372243	0	Singlet	Adipocyte
SAT_2M	2312	1323	0.4312204	0	Singlet	Adipocyte
SAT_2M	2877	1512	0.7612457	0	Singlet	Adipocyte
SAT_2M	1566	990	0.5069708	0	Singlet	Adipocyte
SAT_2M	1039	665	0.7670182	4	Singlet	Immune_cells
SAT_2M	1249	808	2.8776978	4	Singlet	Immune_cells
SAT_2M	1028	723	0.5785921	4	Singlet	Immune_cells
SAT_2M	1638	1043	0.1215805	3	Singlet	FAPs
SAT_2M	1178	822	1.090604	4	Singlet	Immune_cells
SAT_2M	1474	893	3.2542373	1	Singlet	Immune_cells
SAT_2M	2289	1328	0.562527	0	Singlet	Adipocyte
SAT_2M	2261	1284	1.1941619	4	Singlet	Immune_cells
SAT_2M	2720	1488	4.0719002	8	Singlet	Mesothelial
SAT_2M	1341	836	0.8166295	0	Singlet	Adipocyte
SAT_2M	1103	778	1.2544803	5	Singlet	Immune_cells
SAT_2M	1345	898	3.4048853	8	Singlet	Mesothelial

SAT_2M	1812	980	3.1798246	10	Singlet	Immune_cells
SAT_2M	1579	880	1.6970459	9	Singlet	Mesothelial
SAT_2M	1070	737	1.0185185	1	Singlet	Immune_cells
SAT_2M	1341	831	0	3	Singlet	FAPs
SAT_2M	2602	1338	2.2553517	0	Singlet	Adipocyte
SAT_2M	1349	829	0.0735835	1	Singlet	Immune_cells
SAT_2M	1092	712	0.7312614	4	Singlet	Immune_cells
SAT_2M	1646	1055	0.8474576	1	Singlet	Immune_cells
SAT_2M	1227	736	0.1605136	0	Singlet	Adipocyte
SAT_2M	1711	970	10.37296	1	Singlet	Immune_cells
SAT_2M	1468	917	2.0380435	1	Singlet	Immune_cells
SAT_2M	3081	1345	4.7619048	10	Singlet	Immune_cells
SAT_2M	1109	745	1.4427412	7	Singlet	Endothelial_cells
SAT_2M	1594	989	3.5514019	1	Singlet	Immune_cells
SAT_2M	1055	722	0.2822201	4	Singlet	Immune_cells
SAT_2M	1556	990	0.4467135	4	Singlet	Immune_cells
SAT_2M	1359	849	10.499266	1	Singlet	Immune_cells
SAT_2M	1153	788	3.0276817	4	Singlet	Immune_cells
SAT_2M	1839	1044	1.5159718	5	Singlet	Immune_cells
SAT_2M	1394	897	0.2135231	4	Singlet	Immune_cells
SAT_2M	2522	1411	0.7089405	0	Singlet	Adipocyte
SAT_2M	2136	1230	0.7434944	0	Singlet	Adipocyte
SAT_2M	4792	1734	1.8549396	10	Singlet	Immune_cells
SAT_2M	2058	1252	6.0562016	4	Singlet	Immune_cells
SAT_2M	1658	1026	0.8438819	4	Singlet	Immune_cells
SAT_2M	1517	980	1.0512484	5	Singlet	Immune_cells
SAT_2M	1224	767	8.9649552	1	Singlet	Immune_cells
SAT_2M	2155	1262	0.6928406	0	Singlet	Adipocyte
SAT_2M	1686	1058	0.2366864	4	Singlet	Immune_cells
SAT_2M	1059	716	1.8885741	1	Singlet	Immune_cells
SAT_2M	2978	1505	5.6540649	0	Singlet	Adipocyte
SAT_2M	3578	1926	4.6901173	8	Singlet	Mesothelial
SAT_2M	1500	900	0	0	Singlet	Adipocyte
SAT_2M	2572	1474	1.3894249	0	Singlet	Adipocyte
SAT_2M	1098	685	4.3557169	8	Singlet	Mesothelial
SAT_2M	1307	789	0.6859756	0	Singlet	Adipocyte
SAT_2M	1363	838	7.6079005	1	Singlet	Immune_cells
SAT_2M	1273	834	3.299293	4	Singlet	Immune_cells
SAT_2M	1222	814	0.7352941	1	Singlet	Immune_cells
SAT_2M	1243	763	0.8012821	0	Singlet	Adipocyte
SAT_2M	1617	1012	0.8039579	1	Singlet	Immune_cells
SAT_2M	1755	1031	0.5116543	1	Singlet	Immune_cells
SAT_2M	2070	1079	12.757794	1	Singlet	Immune_cells
SAT_2M	1333	897	7.5598802	1	Singlet	Immune_cells
SAT_2M	1599	964	4.9782203	5	Singlet	Immune_cells
SAT_2M	3195	1569	2.9338327	0	Singlet	Adipocyte
SAT_2M	1011	681	0.1966568	4	Singlet	Immune_cells
SAT_2M	1276	832	0.933126	0	Singlet	Adipocyte
SAT_2M	2009	1217	0.4952947	2	Singlet	FAPs
SAT_2M	1001	683	1.8774704	5	Singlet	Immune_cells
SAT_2M	1691	1122	0.1176471	8	Singlet	Mesothelial

SAT_2M	1265	801	0.1577287	0	Singlet	Adipocyte
SAT_2M	1176	730	6.2711864	1	Singlet	Immune_cells
SAT_2M	1581	941	1.0062893	0	Singlet	Adipocyte
SAT_2M	2713	1375	7.0640177	1	Singlet	Immune_cells
SAT_2M	1394	829	8.7857143	1	Singlet	Immune_cells
SAT_2M	1650	852	1.443175	0	Singlet	Adipocyte
SAT_2M	1077	682	2.1256932	1	Singlet	Immune_cells
SAT_2M	1761	1092	1.5254237	1	Singlet	Immune_cells
SAT_2M	1378	855	3.7572254	1	Singlet	Immune_cells
SAT_2M	3055	1608	0	0	Singlet	Adipocyte
SAT_2M	2306	1279	0.1728608	0	Singlet	Adipocyte
SAT_2M	1021	606	8.1213307	0	Singlet	Adipocyte
SAT_2M	2319	1133	0.4729149	0	Singlet	Adipocyte
SAT_2M	1083	700	2.3941068	10	Singlet	Immune_cells
SAT_2M	1819	1129	3.5145524	4	Singlet	Immune_cells
SAT_2M	1660	1057	0.5415162	0	Singlet	Adipocyte
SAT_2M	1143	767	0.5181347	1	Singlet	Immune_cells
SAT_2M	1714	830	6.504065	4	Singlet	Immune_cells
SAT_2M	2835	1569	12.565628	4	Singlet	Immune_cells
SAT_2M	1914	858	7.7725613	0	Singlet	Adipocyte
SAT_2M	1103	678	0.8115419	1	Singlet	Immune_cells
SAT_2M	1653	1009	1.0830325	0	Singlet	Adipocyte
SAT_2M	1435	773	1.2517385	4	Singlet	Immune_cells
SAT_2M	1273	820	1.4855356	1	Singlet	Immune_cells
SAT_2M	1311	830	4.9279757	4	Singlet	Immune_cells
SAT_2M	1304	902	0.3055768	11	Singlet	Smooth_muscle_cells
SAT_2M	1968	1247	3.3924051	4	Singlet	Immune_cells
SAT_2M	3268	1580	0.5159332	0	Singlet	Adipocyte
SAT_2M	3324	1668	0.448833	0	Singlet	Adipocyte
SAT_2M	1059	578	0.2816901	4	Singlet	Immune_cells
SAT_2M	2776	1555	0.9673952	11	Singlet	Smooth_muscle_cells
SAT_2M	1483	894	0.4578156	6	Singlet	Neurons
SAT_2M	1513	1061	0.4611331	5	Singlet	Immune_cells
SAT_2M	1097	740	2.4412297	7	Singlet	Endothelial_cells
SAT_2M	1207	749	1.7269737	0	Singlet	Adipocyte
SAT_2M	1223	812	2.0308692	4	Singlet	Immune_cells
SAT_2M	1007	682	0.6916996	1	Singlet	Immune_cells
SAT_2M	1221	763	0.3273322	9	Singlet	Mesothelial
SAT_2M	1355	840	4.2584435	1	Singlet	Immune_cells
SAT_2M	1927	1005	1.1387164	4	Singlet	Immune_cells
SAT_2M	2934	1232	6.6326531	3	Singlet	FAPs
SAT_2M	1257	849	1.9077901	1	Singlet	Immune_cells
SAT_2M	1555	973	2.5657473	4	Singlet	Immune_cells
SAT_2M	1121	614	0	4	Singlet	Immune_cells
SAT_2M	1831	1075	1.5267176	1	Singlet	Immune_cells
SAT_2M	1611	1002	4.07911	4	Singlet	Immune_cells
SAT_2M	1886	1102	0.6342495	0	Singlet	Adipocyte
SAT_2M	1274	875	1.015625	9	Singlet	Mesothelial
SAT_2M	1216	811	0.2457002	3	Singlet	FAPs
SAT_2M	1198	720	9.9166667	1	Singlet	Immune_cells
SAT_2M	1423	834	0.6306938	0	Singlet	Adipocyte

SAT_2M	1057	730	3.8570085	4	Singlet	Immune_cells
SAT_2M	1617	955	3.6307692	1	Singlet	Immune_cells
SAT_2M	1120	725	0.9760426	4	Singlet	Immune_cells
SAT_2M	1342	876	1.9985196	0	Singlet	Adipocyte
SAT_2M	1075	700	2.3234201	5	Singlet	Immune_cells
SAT_2M	1462	939	0.3394433	8	Singlet	Mesothelial
SAT_2M	2195	1250	1.3593113	8	Singlet	Mesothelial
SAT_2M	1960	1168	5.8943089	1	Singlet	Immune_cells
SAT_2M	2440	1435	3.3932952	3	Singlet	FAPs
SAT_2M	1077	735	1.9408503	5	Singlet	Immune_cells
SAT_2M	1208	749	1.3147083	0	Singlet	Adipocyte
SAT_2M	1384	878	3.4507549	2	Singlet	FAPs
SAT_2M	1083	757	0.5338078	6	Singlet	Neurons
SAT_2M	1982	1225	1.207851	1	Singlet	Immune_cells
SAT_2M	1029	760	0.3656307	6	Singlet	Neurons
SAT_2M	1006	701	5.3571429	5	Singlet	Immune_cells
SAT_2M	1866	1068	1.5532941	10	Singlet	Immune_cells
SAT_2M	2113	1204	5.7601511	1	Singlet	Immune_cells
SAT_2M	6335	2543	1.1037528	10	Singlet	Immune_cells
SAT_2M	1579	1019	0.5047319	7	Singlet	Endothelial_cells
SAT_2M	1235	819	3.4733441	1	Singlet	Immune_cells
SAT_2M	3542	1790	1.3235708	10	Singlet	Immune_cells
SAT_2M	1521	942	0.3267974	0	Singlet	Adipocyte
SAT_2M	1454	860	9.1780822	1	Singlet	Immune_cells
SAT_2M	2566	1379	1.5558149	8	Singlet	Mesothelial
SAT_2M	2891	1627	0.1727713	0	Singlet	Adipocyte
SAT_2M	1298	875	4.058193	7	Singlet	Endothelial_cells
SAT_2M	1711	1076	3.8998836	4	Singlet	Immune_cells
SAT_2M	4937	2049	3.7560582	10	Singlet	Immune_cells
SAT_2M	6469	2825	9.2255477	9	Singlet	Mesothelial
SAT_2M	1066	708	4.5751634	1	Singlet	Immune_cells
SAT_2M	1120	752	1.2488849	4	Singlet	Immune_cells
SAT_2M	1758	913	8.2386364	10	Singlet	Immune_cells
SAT_2M	2882	1749	0.6187693	4	Singlet	Immune_cells
SAT_2M	1171	794	0.5947324	4	Singlet	Immune_cells
SAT_2M	1077	735	3.0164534	3	Singlet	FAPs
SAT_2M	1237	778	7.073955	1	Singlet	Immune_cells
SAT_2M	1359	785	10.433505	5	Singlet	Immune_cells
SAT_2M	1382	814	10.878963	1	Singlet	Immune_cells
SAT_2M	1053	646	8.7984863	5	Singlet	Immune_cells
SAT_2M	1499	865	12.2	1	Singlet	Immune_cells
SAT_2M	2105	1210	7.4196597	1	Singlet	Immune_cells
SAT_2M	1557	983	0.8945687	4	Singlet	Immune_cells
SAT_2M	2482	1314	3.9373242	10	Singlet	Immune_cells
SAT_2M	1735	1023	3.5714286	1	Singlet	Immune_cells
SAT_2M	2909	1556	1.5395142	0	Singlet	Adipocyte
SAT_2M	1152	766	0.2597403	3	Singlet	FAPs
SAT_2M	1934	1108	0.1029866	0	Singlet	Adipocyte
SAT_2M	1859	1202	2.6273458	4	Singlet	Immune_cells
SAT_2M	2556	1174	10.234375	5	Singlet	Immune_cells
SAT_2M	1536	974	6.2946139	4	Singlet	Immune_cells

SAT_2M	1810	1107	3.3076075	8	Singlet	Mesothelial
SAT_2M	1193	745	5.5045872	5	Singlet	Immune_cells
SAT_2M	1333	803	9.588015	1	Singlet	Immune_cells
SAT_2M	1714	1089	1.216686	1	Singlet	Immune_cells
SAT_2M	1515	868	2.9644269	5	Singlet	Immune_cells
SAT_2M	1004	695	0.29615	1	Singlet	Immune_cells
SAT_2M	2399	1358	1.7040732	0	Singlet	Adipocyte
SAT_2M	1212	749	0.0823723	1	Singlet	Immune_cells
SAT_2M	1900	1152	0.4707113	5	Singlet	Immune_cells
SAT_2M	1782	1115	0.280112	4	Singlet	Immune_cells
SAT_2M	1602	1001	1.3715711	8	Singlet	Mesothelial
SAT_2M	1055	699	1.7907634	0	Singlet	Adipocyte
SAT_2M	1876	1183	1.1116993	5	Singlet	Immune_cells
SAT_2M	1862	1114	3.9079229	4	Singlet	Immune_cells
SAT_2M	3280	1661	0.4254026	0	Singlet	Adipocyte
SAT_2M	1212	757	7.0204082	4	Singlet	Immune_cells
SAT_2M	1390	820	4.9356223	1	Singlet	Immune_cells
SAT_2M	1781	1068	2.3542601	1	Singlet	Immune_cells
SAT_2M	1218	749	1.7959184	0	Singlet	Adipocyte
SAT_2M	1038	655	0.3838772	1	Singlet	Immune_cells
SAT_2M	1250	836	5.1958433	4	Singlet	Immune_cells
SAT_2M	1311	816	1.754386	0	Singlet	Adipocyte
SAT_2M	1602	983	4.1358025	1	Singlet	Immune_cells
SAT_2M	1446	892	4.3358568	1	Singlet	Immune_cells
SAT_2M	4095	2057	5.211885	4	Singlet	Immune_cells
SAT_2M	2109	1223	10.287872	5	Singlet	Immune_cells
SAT_2M	1339	841	5.2945563	1	Singlet	Immune_cells
SAT_2M	2869	1478	1.181376	0	Singlet	Adipocyte
SAT_2M	2211	1307	4.6973803	11	Singlet	Smooth_muscle_cells
SAT_2M	1169	743	0.6820119	0	Singlet	Adipocyte
SAT_2M	1302	923	0.4562738	1	Singlet	Immune_cells
SAT_2M	1219	706	2.5326797	1	Singlet	Immune_cells
SAT_2M	1782	1110	0.2238388	4	Singlet	Immune_cells
SAT_2M	2122	1146	2.1146617	10	Singlet	Immune_cells
SAT_2M	3058	1587	0.3911343	3	Singlet	FAPs
SAT_2M	1157	742	0.4280822	0	Singlet	Adipocyte
SAT_2M	1364	833	3.1318281	10	Singlet	Immune_cells
SAT_2M	1576	1006	4.3588124	1	Singlet	Immune_cells
SAT_2M	1390	920	0.3584229	4	Singlet	Immune_cells
SAT_2M	1350	836	1.0316875	1	Singlet	Immune_cells
SAT_2M	2914	1654	0.1366587	0	Singlet	Adipocyte
SAT_2M	1221	822	4.2518397	5	Singlet	Immune_cells
SAT_2M	2202	1180	6.6966292	0	Singlet	Adipocyte
SAT_2M	2284	1321	0.435161	4	Singlet	Immune_cells
SAT_2M	2801	1490	1.2433393	0	Singlet	Adipocyte
SAT_2M	1341	834	0.297619	4	Singlet	Immune_cells
SAT_2M	1864	1114	0.6393181	4	Singlet	Immune_cells
SAT_2M	2389	1330	11.389237	1	Singlet	Immune_cells
SAT_2M	4431	1953	3.0146232	0	Singlet	Adipocyte
SAT_2M	3403	1815	7.9532164	4	Singlet	Immune_cells
SAT_2M	2077	1183	6.6891242	1	Singlet	Immune_cells

SAT_2M	1779	1197	0.4466778	11	Singlet	Smooth_muscle_cells
SAT_2M	1168	779	2.3829787	1	Singlet	Immune_cells
SAT_2M	1492	985	0.5996003	3	Singlet	FAPs
SAT_2M	1256	819	3.4920635	0	Singlet	Adipocyte
SAT_2M	1802	1110	0.1102536	6	Singlet	Neurons
SAT_2M	2060	1241	0.4819277	2	Singlet	FAPs
SAT_2M	1361	879	0.2915452	1	Singlet	Immune_cells
SAT_2M	1718	1063	1.9174898	5	Singlet	Immune_cells
SAT_2M	1862	1074	8.3511777	1	Singlet	Immune_cells
SAT_2M	1326	894	0.7524454	1	Singlet	Immune_cells
SAT_2M	1189	704	1.4214047	4	Singlet	Immune_cells
SAT_2M	3021	1634	6.3057115	4	Singlet	Immune_cells
SAT_2M	1415	857	5.2521008	1	Singlet	Immune_cells
SAT_2M	1683	1016	0.4739336	0	Singlet	Adipocyte
SAT_2M	1317	848	1.5921152	1	Singlet	Immune_cells
SAT_2M	1205	841	0.6578947	4	Singlet	Immune_cells
SAT_2M	4211	1899	2.7711985	10	Singlet	Immune_cells
SAT_2M	1170	705	0.4248088	0	Singlet	Adipocyte
SAT_2M	3091	1561	3.8994521	0	Singlet	Adipocyte
SAT_2M	1283	817	0.2327386	1	Singlet	Immune_cells
SAT_2M	2011	1145	1.1892963	4	Singlet	Immune_cells
SAT_2M	3049	1512	0.3274394	3	Singlet	FAPs
SAT_2M	1648	903	2.4213075	0	Singlet	Adipocyte
SAT_2M	1430	928	1.4675052	4	Singlet	Immune_cells
SAT_2M	1524	821	2.1596859	1	Singlet	Immune_cells
SAT_2M	3309	1555	5.6342272	0	Singlet	Adipocyte
SAT_2M	1028	682	0.6796117	4	Singlet	Immune_cells
SAT_2M	2006	1121	3.1730293	1	Singlet	Immune_cells
SAT_2M	4284	2067	1.4225746	0	Singlet	Adipocyte
SAT_2M	1899	1064	4.2430592	4	Singlet	Immune_cells
SAT_2M	1456	893	6.7854695	1	Singlet	Immune_cells
SAT_2M	1285	733	0.3098373	4	Singlet	Immune_cells
SAT_2M	1001	675	0.5946482	0	Singlet	Adipocyte
SAT_2M	3447	1855	0.0868056	5	Singlet	Immune_cells
SAT_2M	2764	1320	1.2274368	0	Singlet	Adipocyte
SAT_2M	2330	1331	0.1280956	8	Singlet	Mesothelial
SAT_2M	1674	998	5.4198928	1	Singlet	Immune_cells
SAT_2M	1221	720	4.3300654	1	Singlet	Immune_cells
SAT_2M	2914	1422	3.0748206	4	Singlet	Immune_cells
SAT_2M	2000	1333	0.4457652	4	Singlet	Immune_cells
SAT_2M	2674	1411	0.5959032	0	Singlet	Adipocyte
SAT_2M	1061	635	3.1073446	0	Singlet	Adipocyte
SAT_2M	1770	1104	3.9753639	1	Singlet	Immune_cells
SAT_2M	1127	720	0.0883392	1	Singlet	Immune_cells
SAT_2M	1262	777	8.4518167	1	Singlet	Immune_cells
SAT_2M	1423	910	1.4583333	4	Singlet	Immune_cells
SAT_2M	2400	1341	3.1950207	1	Singlet	Immune_cells
SAT_2M	1105	726	1.534296	4	Singlet	Immune_cells
SAT_2M	1479	965	2.4932615	7	Singlet	Endothelial_cells
SAT_2M	1645	1019	2.4271845	4	Singlet	Immune_cells
SAT_2M	1006	699	0.6910168	4	Singlet	Immune_cells

SAT_2M	2030	1222	1.8654885	0	Singlet	Adipocyte
SAT_2M	1618	932	5.6103576	1	Singlet	Immune_cells
SAT_2M	1052	608	0.5644403	4	Singlet	Immune_cells
SAT_2M	2937	1447	12.267028	0	Singlet	Adipocyte
SAT_2M	1129	644	0.4405286	0	Singlet	Adipocyte
SAT_2M	3298	1630	1.1164756	0	Singlet	Adipocyte
SAT_2M	2071	1258	2.5012025	4	Singlet	Immune_cells
SAT_2M	1005	582	0.4970179	1	Singlet	Immune_cells
SAT_2M	1305	789	3.0280091	4	Singlet	Immune_cells
SAT_2M	1005	622	0.6951341	0	Singlet	Adipocyte
SAT_2M	1692	1042	4.8320566	5	Singlet	Immune_cells
SAT_2M	1247	831	2.6294821	1	Singlet	Immune_cells
SAT_2M	1200	673	4.0429043	1	Singlet	Immune_cells
SAT_2M	1036	589	1.0587103	1	Singlet	Immune_cells
SAT_2M	1197	720	1.6680567	11	Singlet	Smooth_muscle_cells
SAT_2M	1013	561	1.9723866	1	Singlet	Immune_cells
SAT_2M	1237	730	3.1375704	1	Singlet	Immune_cells
SAT_2M	3558	1900	5.5243971	9	Singlet	Mesothelial
SAT_2M	1630	1067	3.3618582	11	Singlet	Smooth_muscle_cells
SAT_2M	2746	1623	1.1982571	2	Singlet	FAPs
SAT_2M	1253	701	14.920635	1	Singlet	Immune_cells
SAT_2M	2965	1545	1.5151515	0	Singlet	Adipocyte
SAT_2M	1471	925	0.6101695	0	Singlet	Adipocyte
SAT_2M	2199	1299	4.8570132	4	Singlet	Immune_cells
SAT_2M	2007	1154	0.2485089	5	Singlet	Immune_cells
SAT_2M	3344	1754	0.9259259	0	Singlet	Adipocyte
SAT_2M	1134	734	0.7887818	4	Singlet	Immune_cells
SAT_2M	1244	825	0.5608974	4	Singlet	Immune_cells
SAT_2M	8366	3149	4.1298639	9	Singlet	Mesothelial
SAT_2M	1137	800	0.8756567	5	Singlet	Immune_cells
SAT_2M	1243	690	6.1501597	10	Singlet	Immune_cells
SAT_2M	1699	997	0.4105572	0	Singlet	Adipocyte
SAT_2M	1600	1060	3.8557214	1	Singlet	Immune_cells
SAT_2M	1222	776	0.5668016	5	Singlet	Immune_cells
SAT_2M	2834	1552	0.2455279	0	Singlet	Adipocyte
SAT_2M	1068	762	0.3710575	5	Singlet	Immune_cells
SAT_2M	1004	657	1.3779528	2	Singlet	FAPs
SAT_2M	1146	810	0.6076389	4	Singlet	Immune_cells
SAT_2M	1036	684	2.3121387	5	Singlet	Immune_cells
SAT_2M	1556	953	0.2557545	1	Singlet	Immune_cells
SAT_2M	1039	659	1.2416428	4	Singlet	Immune_cells
SAT_2M	1175	796	2.0321761	0	Singlet	Adipocyte
SAT_2M	1798	1111	2.0487265	1	Singlet	Immune_cells
SAT_2M	1987	1150	0.6036217	0	Singlet	Adipocyte
SAT_2M	1134	766	0	1	Singlet	Immune_cells
SAT_2M	1581	939	1.5141956	4	Singlet	Immune_cells
SAT_2M	1624	965	0.1842752	1	Singlet	Immune_cells
SAT_2M	2241	1179	9.8265896	1	Singlet	Immune_cells
SAT_2M	1128	733	0.4413063	1	Singlet	Immune_cells
SAT_2M	1316	825	0.6792453	0	Singlet	Adipocyte
SAT_2M	1108	768	0.0894454	5	Singlet	Immune_cells

SAT_2M	2983	1606	4.8317228	1	Singlet	Immune_cells
SAT_2M	1148	674	10.947003	1	Singlet	Immune_cells
SAT_2M	1785	1158	0.3899721	4	Singlet	Immune_cells
SAT_2M	1283	815	0.7698229	4	Singlet	Immune_cells
SAT_2M	1245	788	0.6384677	1	Singlet	Immune_cells
SAT_2M	1347	827	0.7358352	0	Singlet	Adipocyte
SAT_2M	1038	706	2.2900763	1	Singlet	Immune_cells
SAT_2M	1888	1209	1.6384778	9	Singlet	Mesothelial
SAT_2M	2109	1198	0.0945626	0	Singlet	Adipocyte
SAT_2M	1122	798	0.2620087	4	Singlet	Immune_cells
SAT_2M	6907	3065	12.613784	4	Singlet	Immune_cells
SAT_2M	1283	791	0.6225681	1	Singlet	Immune_cells
SAT_2M	1050	703	0.6616257	3	Singlet	FAPs
SAT_2M	1011	692	0.6896552	4	Singlet	Immune_cells
SAT_2M	1647	1090	0.2421308	8	Singlet	Mesothelial
SAT_2M	1339	801	9.9925429	1	Singlet	Immune_cells
SAT_2M	1512	947	0.7910349	1	Singlet	Immune_cells
SAT_2M	1409	848	2.8995757	1	Singlet	Immune_cells
SAT_2M	5996	2418	1.9147519	10	Singlet	Immune_cells
SAT_2M	1068	722	1.0261194	4	Singlet	Immune_cells
SAT_2M	1506	876	7.2703239	1	Singlet	Immune_cells
SAT_2M	1031	683	0.0965251	4	Singlet	Immune_cells
SAT_2M	1224	752	0.5672609	4	Singlet	Immune_cells
SAT_2M	3076	1564	0.5832793	0	Singlet	Adipocyte
SAT_2M	2221	1271	0.8952551	0	Singlet	Adipocyte
SAT_2M	2471	1381	0.6048387	0	Singlet	Adipocyte
SAT_2M	1448	910	1.4452856	1	Singlet	Immune_cells
SAT_2M	1497	936	3.5262808	4	Singlet	Immune_cells
SAT_2M	2288	1204	1.4372822	3	Singlet	FAPs
SAT_2M	2027	1168	2.1151008	0	Singlet	Adipocyte
SAT_2M	1124	784	0.6134969	1	Singlet	Immune_cells
SAT_2M	1166	749	0.1710864	0	Singlet	Adipocyte
SAT_2M	1232	697	5.0929669	0	Singlet	Adipocyte
SAT_2M	1511	905	3.5596572	1	Singlet	Immune_cells
SAT_2M	1542	962	1.8087855	0	Singlet	Adipocyte
SAT_2M	1588	972	0	0	Singlet	Adipocyte
SAT_2M	1410	936	0.7768362	4	Singlet	Immune_cells
SAT_2M	2440	1445	4.6644845	8	Singlet	Mesothelial
SAT_2M	1808	1116	0.6615215	1	Singlet	Immune_cells
SAT_2M	1341	792	1.6344725	3	Singlet	FAPs
SAT_2M	1426	933	1.396648	4	Singlet	Immune_cells
SAT_2M	1288	808	3.8491147	1	Singlet	Immune_cells
SAT_2M	1514	1009	1.8372703	4	Singlet	Immune_cells
SAT_2M	2631	1456	7.4829932	10	Singlet	Immune_cells
SAT_2M	1172	800	0.1703578	11	Singlet	Smooth_muscle_cells
SAT_2M	1067	645	6.1452514	1	Singlet	Immune_cells
SAT_2M	1618	1027	3.331277	1	Singlet	Immune_cells
SAT_2M	2095	1228	3.4269396	4	Singlet	Immune_cells
SAT_2M	1296	791	0.1540832	1	Singlet	Immune_cells
SAT_2M	1787	987	5.5122494	10	Singlet	Immune_cells
SAT_2M	1313	848	1.9726859	1	Singlet	Immune_cells

SAT_2M	2542	1161	2.7766914	10	Singlet	Immune_cells
SAT_2M	1080	708	3.2407407	3	Singlet	FAPs
SAT_2M	1492	958	0.266489	1	Singlet	Immune_cells
SAT_2M	1887	1075	5.0211416	1	Singlet	Immune_cells
SAT_2M	1245	803	0.7165605	4	Singlet	Immune_cells
SAT_2M	1464	933	3.2719836	4	Singlet	Immune_cells
SAT_2M	1411	913	2.6798307	1	Singlet	Immune_cells
SAT_2M	1227	803	0.6477733	0	Singlet	Adipocyte
SAT_2M	1022	688	3.7937743	0	Singlet	Adipocyte
SAT_2M	1380	851	2.894356	1	Singlet	Immune_cells
SAT_2M	3349	1675	0.3267974	0	Singlet	Adipocyte
SAT_2M	2304	1338	0.5181347	4	Singlet	Immune_cells
SAT_2M	1292	821	1.7705928	0	Singlet	Adipocyte
SAT_2M	6571	2646	8.4143378	9	Singlet	Mesothelial
SAT_2M	1418	880	3.2913165	1	Singlet	Immune_cells
SAT_2M	2659	1448	6.2453254	5	Singlet	Immune_cells
SAT_2M	2133	1236	0.2802429	0	Singlet	Adipocyte
SAT_2M	1507	930	2.8985507	0	Singlet	Adipocyte
SAT_2M	1447	875	1.1732229	0	Singlet	Adipocyte
SAT_2M	2188	1297	0.4988662	0	Singlet	Adipocyte
SAT_2M	1045	726	0.0956938	4	Singlet	Immune_cells
SAT_2M	1304	784	10.022953	1	Singlet	Immune_cells
SAT_2M	1886	1099	3.7645811	1	Singlet	Immune_cells
SAT_2M	2891	1508	4.283247	10	Singlet	Immune_cells
SAT_2M	2241	1157	0.8900757	10	Singlet	Immune_cells
SAT_2M	1334	803	0.5219985	4	Singlet	Immune_cells
SAT_2M	1848	994	11.764706	1	Singlet	Immune_cells
SAT_2M	1182	835	1.937658	1	Singlet	Immune_cells
SAT_2M	1843	1164	0.3243243	0	Singlet	Adipocyte
SAT_2M	1713	1106	0.8705746	2	Singlet	FAPs
SAT_2M	1955	1140	0.4081633	0	Singlet	Adipocyte
SAT_2M	6681	2853	8.77429	9	Singlet	Mesothelial
SAT_2M	1221	777	6.1324612	1	Singlet	Immune_cells
SAT_2M	1358	831	0.5856515	6	Singlet	Neurons
SAT_2M	2333	1330	2.48394	8	Singlet	Mesothelial
SAT_2M	1229	754	2.8455285	1	Singlet	Immune_cells
SAT_2M	1451	918	0.2745367	11	Singlet	Smooth_muscle_cells
SAT_2M	1708	970	6.0676779	3	Singlet	FAPs
SAT_2M	1667	1032	0.1196888	4	Singlet	Immune_cells
SAT_2M	1215	770	2.4549918	1	Singlet	Immune_cells
SAT_2M	1190	810	0.2508361	5	Singlet	Immune_cells
SAT_2M	1162	780	0.2575107	8	Singlet	Mesothelial
SAT_2M	1133	740	0.1762115	4	Singlet	Immune_cells
SAT_2M	2855	1373	3.5364146	2	Singlet	FAPs
SAT_2M	1067	737	0.8395522	5	Singlet	Immune_cells
SAT_2M	1379	800	0.6521739	0	Singlet	Adipocyte
SAT_2M	1349	882	0.5865103	1	Singlet	Immune_cells
SAT_2M	1457	891	4.4429255	5	Singlet	Immune_cells
SAT_2M	1241	770	1.7642342	4	Singlet	Immune_cells
SAT_2M	1748	992	2.6106697	0	Singlet	Adipocyte
SAT_2M	1455	911	3.7568306	3	Singlet	FAPs

SAT_2M	1403	911	0.9978617	4	Singlet	Immune_cells
SAT_2M	1474	912	2.300406	1	Singlet	Immune_cells
SAT_2M	2189	1238	4.9590537	0	Singlet	Adipocyte
SAT_2M	1811	1119	0.4412576	0	Singlet	Adipocyte
SAT_2M	1491	966	0.8037508	4	Singlet	Immune_cells
SAT_2M	1464	924	0.2043597	4	Singlet	Immune_cells
SAT_2M	1513	937	3.1496063	1	Singlet	Immune_cells
SAT_2M	1012	705	0.5847953	6	Singlet	Neurons
SAT_2M	3290	1596	1.8507282	10	Singlet	Immune_cells
SAT_2M	1423	899	2.5245442	1	Singlet	Immune_cells
SAT_2M	2212	1292	2.8378378	8	Singlet	Mesothelial
SAT_2M	2446	1299	10.938776	9	Singlet	Mesothelial
SAT_2M	1964	1165	0.8117707	3	Singlet	FAPs
SAT_2M	1485	892	0.3344482	4	Singlet	Immune_cells
SAT_2M	2292	1276	1.1759582	0	Singlet	Adipocyte
SAT_2M	1087	720	2.7372263	4	Singlet	Immune_cells
SAT_2M	2748	1446	3.5119479	3	Singlet	FAPs
SAT_2M	1717	1079	0.5235602	1	Singlet	Immune_cells
SAT_2M	1211	756	1.3147083	0	Singlet	Adipocyte
SAT_2M	1734	970	13.333333	1	Singlet	Immune_cells
SAT_2M	1147	788	3.0408341	5	Singlet	Immune_cells
SAT_2M	1902	1085	2.7792344	4	Singlet	Immune_cells
SAT_2M	1308	836	1.217656	1	Singlet	Immune_cells
SAT_2M	1317	852	7.1321321	5	Singlet	Immune_cells
SAT_2M	1857	1100	3.2637774	1	Singlet	Immune_cells
SAT_2M	1774	1175	0.8356546	4	Singlet	Immune_cells
SAT_2M	1227	750	0.8906883	1	Singlet	Immune_cells
SAT_2M	1848	1118	1.0248112	3	Singlet	FAPs
SAT_2M	1686	982	4.4404973	0	Singlet	Adipocyte
SAT_2M	1948	1139	1.9989749	4	Singlet	Immune_cells
SAT_2M	3708	1607	2.5020178	10	Singlet	Immune_cells
SAT_2M	2517	1417	0.5527043	0	Singlet	Adipocyte
SAT_2M	1328	791	6.4467766	1	Singlet	Immune_cells
SAT_2M	1460	853	1.5037594	0	Singlet	Adipocyte
SAT_2M	2131	1268	6.0465116	1	Singlet	Immune_cells
SAT_2M	2354	1283	4.5685279	0	Singlet	Adipocyte
SAT_2M	1276	767	4.1860465	1	Singlet	Immune_cells
SAT_2M	1004	648	4.6812749	1	Singlet	Immune_cells
SAT_2M	1404	876	0.9964413	4	Singlet	Immune_cells
SAT_2M	1410	758	4.8192771	5	Singlet	Immune_cells
SAT_2M	3074	1403	1.6866688	10	Singlet	Immune_cells
SAT_2M	1661	962	6.0587882	1	Singlet	Immune_cells
SAT_2M	1858	1058	4.887218	1	Singlet	Immune_cells
SAT_2M	1223	801	1.0551948	0	Singlet	Adipocyte
SAT_2M	1914	1112	0.9399478	0	Singlet	Adipocyte
SAT_2M	2935	1489	2.7541653	0	Singlet	Adipocyte
SAT_2M	1235	794	7.5	1	Singlet	Immune_cells
SAT_2M	1437	900	0.9722222	1	Singlet	Immune_cells
SAT_2M	1283	828	0.1547988	8	Singlet	Mesothelial
SAT_2M	1194	779	0.3338898	3	Singlet	FAPs
SAT_2M	1439	855	4.0858726	4	Singlet	Immune_cells

SAT_2M	1297	792	5.5895865	1	Singlet	Immune_cells
SAT_2M	1973	1198	6.703629	8	Singlet	Mesothelial
SAT_2M	1160	700	2.1551724	0	Singlet	Adipocyte
SAT_2M	1301	846	1.1494253	4	Singlet	Immune_cells
SAT_2M	1024	644	7.7073171	9	Singlet	Mesothelial
SAT_2M	1095	762	0.3633061	5	Singlet	Immune_cells
SAT_2M	1054	725	1.2287335	1	Singlet	Immune_cells
SAT_2M	3533	1885	5.7344633	5	Singlet	Immune_cells
SAT_2M	1032	709	1.1594203	3	Singlet	FAPs
SAT_2M	2134	1212	0.4659832	0	Singlet	Adipocyte
SAT_2M	1403	862	0.5661713	1	Singlet	Immune_cells
SAT_2M	1331	787	6.0015004	1	Singlet	Immune_cells
SAT_2M	1967	1192	2.5316456	3	Singlet	FAPs
SAT_2M	1208	769	0.0823723	0	Singlet	Adipocyte
SAT_2M	1959	1090	9.3272171	1	Singlet	Immune_cells
SAT_2M	1441	901	7.967033	1	Singlet	Immune_cells
SAT_2M	2094	1169	3.3333333	2	Singlet	FAPs
SAT_2M	1028	690	0.9661836	0	Singlet	Adipocyte
SAT_2M	1770	1114	6.1304837	1	Singlet	Immune_cells
SAT_2M	3496	1566	3.7913341	10	Singlet	Immune_cells
SAT_2M	1301	790	10.276074	9	Singlet	Mesothelial
SAT_2M	2931	1607	2.4539877	7	Singlet	Endothelial_cells
SAT_2M	1664	1037	1.497006	2	Singlet	FAPs
SAT_2M	2336	1352	1.9165247	1	Singlet	Immune_cells
SAT_2M	1286	838	2.1671827	1	Singlet	Immune_cells
SAT_2M	19351	5345	7.7605779	9	Singlet	Mesothelial
SAT_2M	1918	1101	4.6354167	9	Singlet	Mesothelial
SAT_2M	2396	1241	4.4185077	5	Singlet	Immune_cells
SAT_2M	1848	1046	1.0242588	0	Singlet	Adipocyte
SAT_2M	1423	837	8.0815179	9	Singlet	Mesothelial
SAT_2M	1228	772	0.9724473	1	Singlet	Immune_cells
SAT_2M	1218	812	3.1122031	1	Singlet	Immune_cells
SAT_2M	3419	1688	0.1751825	0	Singlet	Adipocyte
SAT_2M	1239	814	0.32	0	Singlet	Adipocyte
SAT_2M	2402	1455	1.8272425	7	Singlet	Endothelial_cells
SAT_2M	1016	712	1.7681729	1	Singlet	Immune_cells
SAT_2M	1500	967	0.5315615	4	Singlet	Immune_cells
SAT_2M	2280	1357	0.8304196	1	Singlet	Immune_cells
SAT_2M	3518	1830	0.7361268	0	Singlet	Adipocyte
SAT_2M	1614	1054	0.9264978	5	Singlet	Immune_cells
SAT_2M	1268	803	9.1549296	1	Singlet	Immune_cells
SAT_2M	1113	721	0.8035714	0	Singlet	Adipocyte
SAT_2M	1230	809	4.0387722	1	Singlet	Immune_cells
SAT_2M	1267	823	0.4698512	0	Singlet	Adipocyte
SAT_2M	1593	1032	0.8739076	4	Singlet	Immune_cells
SAT_2M	2556	1450	5.1831645	1	Singlet	Immune_cells
SAT_2M	1119	735	3.75	4	Singlet	Immune_cells
SAT_2M	1266	788	0.6299213	0	Singlet	Adipocyte
SAT_2M	1596	979	0.8104738	3	Singlet	FAPs
SAT_2M	1646	965	6.9174757	1	Singlet	Immune_cells
SAT_2M	1048	692	2.6590693	1	Singlet	Immune_cells

SAT_2M	1295	871	0.2302379	1	Singlet	Immune_cells
SAT_2M	1194	781	1.5780731	1	Singlet	Immune_cells
SAT_2M	1068	660	4.53284	0	Singlet	Adipocyte
SAT_2M	1224	847	0.4065041	5	Singlet	Immune_cells
SAT_2M	1292	762	0.4622496	1	Singlet	Immune_cells
SAT_2M	1196	734	0	4	Singlet	Immune_cells
SAT_2M	1085	723	0.4591368	4	Singlet	Immune_cells
SAT_2M	1453	913	3.6351166	5	Singlet	Immune_cells
SAT_2M	1294	792	0.308642	4	Singlet	Immune_cells
SAT_2M	1551	1004	5.3376206	1	Singlet	Immune_cells
SAT_2M	1735	998	2.2413793	1	Singlet	Immune_cells
SAT_2M	2019	1137	4.5767717	0	Singlet	Adipocyte
SAT_2M	1053	676	4.2573321	1	Singlet	Immune_cells
SAT_2M	1586	909	3.0094044	1	Singlet	Immune_cells
SAT_2M	1033	691	6.8965517	4	Singlet	Immune_cells
SAT_2M	1065	680	0.1867414	1	Singlet	Immune_cells
SAT_2M	1481	923	0.7397445	4	Singlet	Immune_cells
SAT_2M	1390	844	1.0043042	0	Singlet	Adipocyte
SAT_2M	1133	733	0.2643172	4	Singlet	Immune_cells
SAT_2M	2894	1637	0.2068252	3	Singlet	FAPs
SAT_2M	1394	804	9.4084105	1	Singlet	Immune_cells
SAT_2M	2009	1272	2.8051181	6	Singlet	Neurons
SAT_2M	2251	1305	0.3101462	11	Singlet	Smooth_muscle_cells
SAT_2M	3040	1537	0.0327761	11	Singlet	Smooth_muscle_cells
SAT_2M	2245	1092	5.8274021	10	Singlet	Immune_cells
SAT_2M	1067	698	4.1977612	1	Singlet	Immune_cells
SAT_2M	1398	885	0.4270463	3	Singlet	FAPs
SAT_2M	2021	1208	3.458498	1	Singlet	Immune_cells
SAT_2M	1328	844	1.7306245	1	Singlet	Immune_cells
SAT_2M	1127	731	0.9708738	1	Singlet	Immune_cells
SAT_2M	4029	1908	8.4552442	9	Singlet	Mesothelial
SAT_2M	1245	800	0.7980846	4	Singlet	Immune_cells
SAT_2M	1574	990	1.8999367	4	Singlet	Immune_cells
SAT_2M	1751	1134	1.5384615	9	Singlet	Mesothelial
SAT_2M	1351	917	0.9544787	7	Singlet	Endothelial_cells
SAT_2M	1719	1058	6.8945539	1	Singlet	Immune_cells
SAT_2M	1174	796	0.7601351	4	Singlet	Immune_cells
SAT_2M	2075	1171	0.3360538	0	Singlet	Adipocyte
SAT_2M	1492	944	0.596817	0	Singlet	Adipocyte
SAT_2M	1932	1087	2.4729521	1	Singlet	Immune_cells
SAT_2M	1703	1025	1.9894675	1	Singlet	Immune_cells
SAT_2M	6155	2849	6.610499	4	Singlet	Immune_cells
SAT_2M	2590	1360	7.0134875	5	Singlet	Immune_cells
SAT_2M	1834	1162	0.1627781	1	Singlet	Immune_cells
SAT_2M	1442	991	1.5172414	11	Singlet	Smooth_muscle_cells
SAT_2M	1502	966	1.7857143	1	Singlet	Immune_cells
SAT_2M	1244	852	5.3642914	1	Singlet	Immune_cells
SAT_2M	1120	740	0.177305	1	Singlet	Immune_cells
SAT_2M	2467	1276	10.741792	1	Singlet	Immune_cells
SAT_2M	1794	1117	4.6744574	4	Singlet	Immune_cells
SAT_2M	3891	1999	12.752881	4	Singlet	Immune_cells

SAT_2M	2034	1096	4.766537	1	Singlet	Immune_cells
SAT_2M	1946	1198	3.3333333	9	Singlet	Mesothelial
SAT_2M	5554	2756	2.8941219	9	Singlet	Mesothelial
SAT_2M	1341	889	0.5200594	1	Singlet	Immune_cells
SAT_2M	1876	1061	9.7014925	9	Singlet	Mesothelial
SAT_2M	2388	1284	8.7301587	1	Singlet	Immune_cells
SAT_2M	1167	717	1.4554795	4	Singlet	Immune_cells
SAT_2M	1233	733	11.588331	4	Singlet	Immune_cells
SAT_2M	1388	900	0.5025126	4	Singlet	Immune_cells
SAT_2M	1690	1019	10.691081	4	Singlet	Immune_cells
SAT_2M	1428	894	0.0697837	3	Singlet	FAPs
SAT_2M	1040	751	0.4789272	3	Singlet	FAPs
SAT_2M	7772	3411	12.498394	4	Singlet	Immune_cells
SAT_2M	1489	876	0.3340013	1	Singlet	Immune_cells
SAT_2M	1387	920	2.3689878	3	Singlet	FAPs
SAT_2M	1709	1095	0.4672897	4	Singlet	Immune_cells
SAT_2M	1333	909	2.0895522	7	Singlet	Endothelial_cells
SAT_2M	1693	1045	2.4160283	1	Singlet	Immune_cells
SAT_2M	1095	739	2.1857924	2	Singlet	FAPs
SAT_2M	1328	854	4.9475262	1	Singlet	Immune_cells
SAT_2M	1200	786	3.3305579	4	Singlet	Immune_cells
SAT_2M	1465	952	1.2960437	4	Singlet	Immune_cells
SAT_2M	2792	1523	0.2850018	0	Singlet	Adipocyte
SAT_2M	2024	1165	0.147929	0	Singlet	Adipocyte
SAT_2M	1378	852	2.8880866	1	Singlet	Immune_cells
SAT_2M	1224	800	0	1	Singlet	Immune_cells
SAT_2M	1823	1130	1.5803815	5	Singlet	Immune_cells
SAT_2M	1435	898	3.0991736	1	Singlet	Immune_cells
SAT_2M	2450	1309	0.0812348	0	Singlet	Adipocyte
SAT_2M	1384	860	2.8860029	1	Singlet	Immune_cells
SAT_2M	1249	769	7.154213	1	Singlet	Immune_cells
SAT_2M	1414	814	10.570825	1	Singlet	Immune_cells
SAT_2M	1387	847	3.0150754	3	Singlet	FAPs
SAT_2M	3578	1795	1.2820513	0	Singlet	Adipocyte
SAT_2M	2273	1347	6.7603161	1	Singlet	Immune_cells
SAT_2M	2576	1312	2.2033243	0	Singlet	Adipocyte
SAT_2M	4541	2362	2.7204914	9	Singlet	Mesothelial
SAT_2M	2085	1212	9.7142857	9	Singlet	Mesothelial
SAT_2M	1317	926	0.6828528	4	Singlet	Immune_cells
SAT_2M	1120	701	0.8007117	0	Singlet	Adipocyte
SAT_2M	1675	986	4.4073853	1	Singlet	Immune_cells
SAT_2M	1360	820	0.2197802	4	Singlet	Immune_cells
SAT_2M	5624	2689	4.7246892	9	Singlet	Mesothelial
SAT_2M	1559	981	3.9692702	1	Singlet	Immune_cells
SAT_2M	1223	838	0.324412	5	Singlet	Immune_cells
SAT_2M	2297	1277	1.0841284	0	Singlet	Adipocyte
SAT_2M	1722	965	9.8901099	1	Singlet	Immune_cells
SAT_2M	1201	814	0.5780347	1	Singlet	Immune_cells
SAT_2M	1234	712	8.3870968	1	Singlet	Immune_cells
SAT_2M	2301	1326	4.6400694	5	Singlet	Immune_cells
SAT_2M	1454	886	7.0596299	1	Singlet	Immune_cells

SAT_2M	1935	972	0.9292721	10	Singlet	Immune_cells
SAT_2M	1836	1099	2.8757461	1	Singlet	Immune_cells
SAT_2M	3764	1879	0.5291005	11	Singlet	Smooth_muscle_cells
SAT_2M	1223	790	0.5709625	1	Singlet	Immune_cells
SAT_2M	1058	753	0.2824859	1	Singlet	Immune_cells
SAT_2M	2167	1241	0.2754821	0	Singlet	Adipocyte
SAT_2M	1222	766	0.4088307	1	Singlet	Immune_cells
SAT_2M	2918	1487	6.2585499	9	Singlet	Mesothelial
SAT_2M	1782	1049	4.262479	1	Singlet	Immune_cells
SAT_2M	1002	673	2.3738872	0	Singlet	Adipocyte
SAT_2M	2442	1449	5.4878049	4	Singlet	Immune_cells
SAT_2M	1239	764	9.9113618	4	Singlet	Immune_cells
SAT_2M	1875	1077	4.250797	1	Singlet	Immune_cells
SAT_2M	1222	820	2.0424837	4	Singlet	Immune_cells
SAT_2M	1440	950	0.9715475	1	Singlet	Immune_cells
SAT_2M	1757	1105	3.2386364	4	Singlet	Immune_cells
SAT_2M	4106	1941	0.2431907	0	Singlet	Adipocyte
SAT_2M	1517	826	4.1967213	5	Singlet	Immune_cells
SAT_2M	1740	988	0.7995431	0	Singlet	Adipocyte
SAT_2M	1498	975	2.1319121	4	Singlet	Immune_cells
SAT_2M	1016	674	0.2918288	0	Singlet	Adipocyte
SAT_2M	2543	1358	4.8254217	5	Singlet	Immune_cells
SAT_2M	4356	1694	5.8661778	10	Singlet	Immune_cells
SAT_2M	1321	861	0.0750751	1	Singlet	Immune_cells
SAT_2M	1039	678	0.9587728	4	Singlet	Immune_cells
SAT_2M	1287	863	2.0849421	1	Singlet	Immune_cells
SAT_2M	1280	848	2.7993779	11	Singlet	Smooth_muscle_cells
SAT_2M	2660	1484	1.8024784	8	Singlet	Mesothelial
SAT_2M	2606	1386	7.9310345	1	Singlet	Immune_cells
SAT_2M	1263	829	4.6420142	1	Singlet	Immune_cells
SAT_2M	1492	844	14.390406	1	Singlet	Immune_cells
SAT_2M	1475	928	0.3369272	4	Singlet	Immune_cells
SAT_2M	1639	991	2.189781	1	Singlet	Immune_cells
SAT_2M	1229	760	8.0161943	1	Singlet	Immune_cells
SAT_2M	1005	702	0.4911591	6	Singlet	Neurons
SAT_2M	1214	726	7.3130649	5	Singlet	Immune_cells
SAT_2M	1106	771	4.5822102	1	Singlet	Immune_cells
SAT_2M	3042	1767	0.3927987	8	Singlet	Mesothelial
SAT_2M	1192	819	0.5794702	0	Singlet	Adipocyte
SAT_2M	1401	851	8.5592011	1	Singlet	Immune_cells
SAT_2M	1276	785	0.859375	0	Singlet	Adipocyte
SAT_2M	1350	841	0.5895357	1	Singlet	Immune_cells
SAT_2M	1187	694	0.6672227	1	Singlet	Immune_cells
SAT_2M	2101	1219	0.3311258	0	Singlet	Adipocyte
SAT_2M	1524	954	2.6902887	1	Singlet	Immune_cells
SAT_2M	1439	839	0.6925208	0	Singlet	Adipocyte
SAT_2M	2822	1515	8.5572843	1	Singlet	Immune_cells
SAT_2M	1523	1031	0.5204945	6	Singlet	Neurons
SAT_2M	2785	1566	0.5372493	0	Singlet	Adipocyte
SAT_2M	1324	875	6.4006024	1	Singlet	Immune_cells
SAT_2M	1275	758	11.18061	1	Singlet	Immune_cells

SAT_2M	2499	1369	1.3142174	0	Singlet	Adipocyte
SAT_2M	1304	793	0.8403361	1	Singlet	Immune_cells
SAT_2M	1064	718	0.5612722	0	Singlet	Adipocyte
SAT_2M	1958	1195	2.3350254	1	Singlet	Immune_cells
SAT_2M	1150	686	6.3203463	3	Singlet	FAPs
SAT_2M	1898	1117	0.5249344	4	Singlet	Immune_cells
SAT_2M	1405	885	6.01983	0	Singlet	Adipocyte
SAT_2M	1346	830	4.6460177	0	Singlet	Adipocyte
SAT_2M	1439	896	0.9715475	1	Singlet	Immune_cells
SAT_2M	1082	700	0.1829826	0	Singlet	Adipocyte
SAT_2M	1552	927	0.4490058	11	Singlet	Smooth_muscle_cells
SAT_2M	3955	1787	4.8016174	10	Singlet	Immune_cells
SAT_2M	1215	783	4.0749796	1	Singlet	Immune_cells
SAT_2M	1127	793	2.4626209	1	Singlet	Immune_cells
SAT_2M	1073	688	0.8372093	0	Singlet	Adipocyte
SAT_2M	1137	764	0.3496504	4	Singlet	Immune_cells
SAT_2M	1499	907	3.3244681	1	Singlet	Immune_cells
SAT_2M	1651	986	3.081571	1	Singlet	Immune_cells
SAT_2M	2230	1464	0.3125	4	Singlet	Immune_cells
SAT_2M	1219	813	1.6299919	4	Singlet	Immune_cells
SAT_2M	2175	1119	6.3273728	3	Singlet	FAPs
SAT_2M	2588	1397	1.2736395	1	Singlet	Immune_cells
SAT_2M	1400	886	0.1424501	0	Singlet	Adipocyte
SAT_2M	3024	1582	0.2303389	0	Singlet	Adipocyte
SAT_2M	1601	915	9.9564406	1	Singlet	Immune_cells
SAT_2M	1396	875	0.5698006	3	Singlet	FAPs
SAT_2M	1392	825	4.4412607	1	Singlet	Immune_cells
SAT_2M	1502	976	1.3262599	4	Singlet	Immune_cells
SAT_2M	1203	798	0.4115226	1	Singlet	Immune_cells
SAT_2M	1345	906	0.5939124	1	Singlet	Immune_cells
SAT_2M	2185	1293	1.7743403	5	Singlet	Immune_cells
SAT_2M	1140	773	2.6019081	1	Singlet	Immune_cells
SAT_2M	3252	1663	0.8277131	0	Singlet	Adipocyte
SAT_2M	2688	1438	0.3335804	0	Singlet	Adipocyte
SAT_2M	1529	967	2.1442495	1	Singlet	Immune_cells
SAT_2M	2633	1359	11.516748	1	Singlet	Immune_cells
SAT_2M	1845	1133	5.4054054	4	Singlet	Immune_cells
SAT_2M	2212	1150	5.32972	10	Singlet	Immune_cells
SAT_2M	1377	804	10.224801	1	Singlet	Immune_cells
SAT_2M	1141	748	0.3496504	1	Singlet	Immune_cells
SAT_2M	2265	1272	7.5297226	1	Singlet	Immune_cells
SAT_2M	1495	935	0.3342246	1	Singlet	Immune_cells
SAT_2M	1383	926	0.1444043	4	Singlet	Immune_cells
SAT_2M	1883	1109	5.6733828	9	Singlet	Mesothelial
SAT_2M	6786	2716	1.5004413	10	Singlet	Immune_cells
SAT_2M	1148	771	0.4344049	1	Singlet	Immune_cells
SAT_2M	1295	813	2.9141104	3	Singlet	FAPs
SAT_2M	1279	766	9.5015576	1	Singlet	Immune_cells
SAT_2M	1139	688	4.9694856	1	Singlet	Immune_cells
SAT_2M	1915	1201	2.4517475	2	Singlet	FAPs
SAT_2M	2000	1116	1.746507	5	Singlet	Immune_cells

SAT_2M	3588	1861	1.3915948	5	Singlet	Immune_cells
SAT_2M	2237	1292	0.1782531	0	Singlet	Adipocyte
SAT_2M	1266	803	0	4	Singlet	Immune_cells
SAT_2M	1313	826	8.973384	1	Singlet	Immune_cells
SAT_2M	2016	1146	2.4066798	0	Singlet	Adipocyte
SAT_2M	1577	903	7.7264091	1	Singlet	Immune_cells
SAT_2M	1436	933	0.6954103	3	Singlet	FAPs
SAT_2M	2064	1096	3.6319613	3	Singlet	FAPs
SAT_2M	2262	1315	2.553941	9	Singlet	Mesothelial
SAT_2M	1306	858	0.4562738	4	Singlet	Immune_cells
SAT_2M	1421	839	0.4908836	1	Singlet	Immune_cells
SAT_2M	1815	1155	0.9356081	4	Singlet	Immune_cells
SAT_2M	1566	962	0.5092298	1	Singlet	Immune_cells
SAT_2M	1852	1125	1.6693592	4	Singlet	Immune_cells
SAT_2M	1478	873	3.0384875	0	Singlet	Adipocyte
SAT_2M	1648	1022	4.6553809	5	Singlet	Immune_cells
SAT_2M	2080	1196	0.3350886	0	Singlet	Adipocyte
SAT_2M	1556	772	8.8518281	10	Singlet	Immune_cells
SAT_2M	2347	1345	0.2969877	8	Singlet	Mesothelial
SAT_2M	1125	761	4.8330404	1	Singlet	Immune_cells
SAT_2M	1016	692	0.3883495	9	Singlet	Mesothelial
SAT_2M	2223	1216	3.1305903	0	Singlet	Adipocyte
SAT_2M	1081	694	0.5484461	1	Singlet	Immune_cells
SAT_2M	1180	764	0.7614213	3	Singlet	FAPs
SAT_2M	1187	777	0.5867561	1	Singlet	Immune_cells
SAT_2M	1308	859	3.5034273	1	Singlet	Immune_cells
SAT_2M	1621	1010	2.2727273	4	Singlet	Immune_cells
SAT_2M	4038	1866	0.3201182	0	Singlet	Adipocyte
SAT_2M	1034	711	0.0963391	1	Singlet	Immune_cells
SAT_2M	1423	901	0	3	Singlet	FAPs
SAT_2M	1413	871	2.8975265	4	Singlet	Immune_cells
SAT_2M	2164	1191	2.8965517	0	Singlet	Adipocyte
SAT_2M	2196	1275	0.3631412	0	Singlet	Adipocyte
SAT_2M	1449	889	1.1716058	0	Singlet	Adipocyte
SAT_2M	1681	994	1.2389381	5	Singlet	Immune_cells
SAT_2M	1588	1011	0.0625391	6	Singlet	Neurons
SAT_2M	1020	672	1.6456922	0	Singlet	Adipocyte
SAT_2M	1266	821	3.3044847	1	Singlet	Immune_cells
SAT_2M	1525	905	2.8013029	1	Singlet	Immune_cells
SAT_2M	1387	880	4.7925608	4	Singlet	Immune_cells
SAT_2M	1684	1028	0.2357101	0	Singlet	Adipocyte
SAT_2M	5651	2394	0.7937908	10	Singlet	Immune_cells
SAT_2M	1012	684	1.8700787	1	Singlet	Immune_cells
SAT_2M	1761	1090	4.5377198	4	Singlet	Immune_cells
SAT_2M	1182	787	0.926706	4	Singlet	Immune_cells
SAT_2M	1149	792	0.3457217	4	Singlet	Immune_cells
SAT_2M	2188	1257	3.4655723	4	Singlet	Immune_cells
SAT_2M	2046	1288	0.78125	4	Singlet	Immune_cells
SAT_2M	1422	887	0.9090909	7	Singlet	Endothelial_cells
SAT_2M	1848	1139	6.3140853	1	Singlet	Immune_cells
SAT_2M	1532	1014	1.0430248	4	Singlet	Immune_cells

SAT_2M	1243	817	5.2208835	1	Singlet	Immune_cells
SAT_2M	3931	1729	4.3390003	10	Singlet	Immune_cells
SAT_2M	2973	1711	0.1341382	3	Singlet	FAPs
SAT_2M	1335	871	5.754858	1	Singlet	Immune_cells
SAT_2M	1118	661	8.2887701	9	Singlet	Mesothelial
SAT_2M	1029	630	6.122449	0	Singlet	Adipocyte
SAT_2M	2123	1216	0.8423023	0	Singlet	Adipocyte
SAT_2M	1174	795	1.5293118	4	Singlet	Immune_cells
SAT_2M	1243	835	0.0798722	4	Singlet	Immune_cells
SAT_2M	2747	1490	2.2037572	0	Singlet	Adipocyte
SAT_2M	3859	1817	0.232438	0	Singlet	Adipocyte
SAT_2M	3396	1678	0.5284792	0	Singlet	Adipocyte
SAT_2M	1966	1156	0.2534212	0	Singlet	Adipocyte
SAT_2M	1208	800	1.1541632	1	Singlet	Immune_cells
SAT_2M	1236	848	0.3980892	6	Singlet	Neurons
SAT_2M	1609	986	0.3097893	0	Singlet	Adipocyte
SAT_2M	1321	833	0.4521477	0	Singlet	Adipocyte
SAT_2M	2857	1250	3.9496679	10	Singlet	Immune_cells
SAT_2M	2578	1387	1.5063731	0	Singlet	Adipocyte
SAT_2M	1253	823	4.9363057	1	Singlet	Immune_cells
SAT_2M	1441	960	0.6219765	4	Singlet	Immune_cells
SAT_2M	1735	1116	0.5178366	1	Singlet	Immune_cells
SAT_2M	1033	674	0.3872217	6	Singlet	Neurons
SAT_2M	1128	738	0.8833922	4	Singlet	Immune_cells
SAT_2M	2453	1346	1.4985824	0	Singlet	Adipocyte
SAT_2M	1007	662	1.0912698	4	Singlet	Immune_cells
SAT_2M	1278	844	4.140625	1	Singlet	Immune_cells
SAT_2M	4854	2415	10.441932	4	Singlet	Immune_cells
SAT_2M	4802	2205	11.060291	9	Singlet	Mesothelial
SAT_2M	1298	845	3.5222052	1	Singlet	Immune_cells
SAT_2M	1263	841	0.3943218	1	Singlet	Immune_cells
SAT_2M	1752	1102	0.2844141	5	Singlet	Immune_cells
SAT_2M	1006	631	3.5749752	5	Singlet	Immune_cells
SAT_2M	4403	2112	0.2486438	0	Singlet	Adipocyte
SAT_2M	1263	798	5.5248619	1	Singlet	Immune_cells
SAT_2M	1600	1002	3.6204744	10	Singlet	Immune_cells
SAT_2M	1618	1026	1.1117974	4	Singlet	Immune_cells
SAT_2M	1247	767	0.5586592	1	Singlet	Immune_cells
SAT_2M	1399	844	2.2824536	1	Singlet	Immune_cells
SAT_2M	1636	1012	3.8344492	8	Singlet	Mesothelial
SAT_2M	2829	1425	9.5875925	9	Singlet	Mesothelial
SAT_2M	1470	897	6.6530889	4	Singlet	Immune_cells
SAT_2M	1535	956	1.0403121	1	Singlet	Immune_cells
SAT_2M	2136	1310	7.8285182	4	Singlet	Immune_cells
SAT_2M	2149	1246	1.4883721	10	Singlet	Immune_cells
SAT_2M	1626	1034	1.9619865	1	Singlet	Immune_cells
SAT_2M	1230	768	5.7676686	1	Singlet	Immune_cells
SAT_2M	1361	826	6.2408223	1	Singlet	Immune_cells
SAT_2M	2598	1516	0.5760369	2	Singlet	FAPs
SAT_2M	1672	1045	3.641791	1	Singlet	Immune_cells
SAT_2M	4388	2142	1.4068527	2	Singlet	FAPs

SAT_2M	1377	861	5.2098408	1	Singlet	Immune_cells
SAT_2M	3842	1651	1.4025974	10	Singlet	Immune_cells
SAT_2M	1333	844	2.2488756	7	Singlet	Endothelial_cells
SAT_2M	1445	946	1.2405238	4	Singlet	Immune_cells
SAT_2M	4056	1829	0.4174853	10	Singlet	Immune_cells
SAT_2M	1119	723	2.6785714	4	Singlet	Immune_cells
SAT_2M	1229	808	0.2411576	1	Singlet	Immune_cells
SAT_2M	1961	1206	0.4058853	0	Singlet	Adipocyte
SAT_2M	1694	1019	1.2309496	1	Singlet	Immune_cells
SAT_2M	1191	779	6.0251046	1	Singlet	Immune_cells
SAT_2M	1420	882	3.0133146	1	Singlet	Immune_cells
SAT_2M	5481	2632	6.0280459	9	Singlet	Mesothelial
SAT_2M	1812	1068	1.4332966	1	Singlet	Immune_cells
SAT_2M	1605	969	1.0552452	0	Singlet	Adipocyte
SAT_2M	1716	1056	3.372093	4	Singlet	Immune_cells
SAT_2M	1623	1041	0.1839362	0	Singlet	Adipocyte
SAT_2M	1307	852	0.2286585	4	Singlet	Immune_cells
SAT_2M	1027	687	2.526725	4	Singlet	Immune_cells
SAT_2M	1394	794	8.6366881	3	Singlet	FAPs
SAT_2M	1090	714	0.9140768	4	Singlet	Immune_cells
SAT_2M	1818	1099	3.5557987	4	Singlet	Immune_cells
SAT_2M	1007	701	1.2820513	4	Singlet	Immune_cells
SAT_2M	1536	920	0.3246753	3	Singlet	FAPs
SAT_2M	1068	695	1.1173184	6	Singlet	Neurons
SAT_2M	1787	1059	8.2681564	0	Singlet	Adipocyte
SAT_2M	1221	705	10.956664	0	Singlet	Adipocyte
SAT_2M	1157	818	0.0856164	11	Singlet	Smooth_muscle_cells
SAT_2M	1543	942	8.7741935	1	Singlet	Immune_cells
SAT_2M	1617	1030	0.1227747	8	Singlet	Mesothelial
SAT_2M	1008	701	0.1982161	1	Singlet	Immune_cells
SAT_2M	1465	896	1.8329939	1	Singlet	Immune_cells
SAT_2M	1122	750	0.1776199	3	Singlet	FAPs
SAT_2M	1428	966	2.5783972	1	Singlet	Immune_cells
SAT_2M	1025	736	1.352657	11	Singlet	Smooth_muscle_cells
SAT_2M	1050	716	1.994302	11	Singlet	Smooth_muscle_cells
SAT_2M	1816	1157	3.8398245	2	Singlet	FAPs
SAT_2M	1121	755	0.3552398	0	Singlet	Adipocyte
SAT_2M	1258	769	0.2377179	0	Singlet	Adipocyte
SAT_2M	1848	1040	9.7297297	9	Singlet	Mesothelial
SAT_2M	1334	855	0.1492537	0	Singlet	Adipocyte
SAT_2M	1618	1046	0.6790123	5	Singlet	Immune_cells
SAT_2M	1292	878	0.6955178	4	Singlet	Immune_cells
SAT_2M	1251	800	4.7770701	1	Singlet	Immune_cells
SAT_2M	1515	901	1.2483574	4	Singlet	Immune_cells
SAT_2M	1472	905	5.0709939	1	Singlet	Immune_cells
SAT_2M	4028	1925	0.3711952	0	Singlet	Adipocyte
SAT_2M	1338	906	1.2620638	4	Singlet	Immune_cells
SAT_2M	2540	1305	2.5933202	5	Singlet	Immune_cells
SAT_2M	1574	1023	1.0139417	4	Singlet	Immune_cells
SAT_2M	2012	1109	7.7799802	9	Singlet	Mesothelial
SAT_2M	1232	774	3.4789644	1	Singlet	Immune_cells

SAT_2M	1143	769	0.2620087	8	Singlet	Mesothelial
SAT_2M	1266	852	4.3273013	4	Singlet	Immune_cells
SAT_2M	2423	1372	2.5777414	6	Singlet	Neurons
SAT_2M	1232	836	2.0242915	1	Singlet	Immune_cells
SAT_2M	1355	797	8.4621045	9	Singlet	Mesothelial
SAT_2M	6960	2327	1.5338303	10	Singlet	Immune_cells
SAT_2M	1441	887	2.4187975	1	Singlet	Immune_cells
SAT_2M	1009	703	1.7786561	11	Singlet	Smooth_muscle_cells
SAT_2M	2545	1399	0.3525264	0	Singlet	Adipocyte
SAT_2M	1926	1145	0.6185567	0	Singlet	Adipocyte
SAT_2M	2325	1361	4.5064378	4	Singlet	Immune_cells
SAT_2M	1025	681	2.3346304	1	Singlet	Immune_cells
SAT_2M	1330	806	0.9737828	4	Singlet	Immune_cells
SAT_2M	1114	763	0.2683363	2	Singlet	FAPs
SAT_2M	1596	949	9.3241552	1	Singlet	Immune_cells
SAT_2M	1282	848	4.7360248	1	Singlet	Immune_cells
SAT_2M	1387	883	6.6044508	1	Singlet	Immune_cells
SAT_2M	1222	802	5.9494703	1	Singlet	Immune_cells
SAT_2M	2339	1329	2.7268854	1	Singlet	Immune_cells
SAT_2M	1429	890	2.3743017	1	Singlet	Immune_cells
SAT_2M	4795	2260	4.1172801	4	Singlet	Immune_cells
SAT_2M	2110	1167	6.3297119	1	Singlet	Immune_cells
SAT_2M	1275	827	2.1875	9	Singlet	Mesothelial
SAT_2M	2303	1258	0.216544	3	Singlet	FAPs
SAT_2M	1389	893	0.3589375	8	Singlet	Mesothelial
SAT_2M	5293	2509	5.1847662	9	Singlet	Mesothelial
SAT_2M	1049	690	0.1906578	0	Singlet	Adipocyte
SAT_2M	1562	955	0.8939974	1	Singlet	Immune_cells
SAT_2M	1704	1048	1.2850467	0	Singlet	Adipocyte
SAT_2M	4212	1866	2.3387668	10	Singlet	Immune_cells
SAT_2M	1242	784	4.5746388	1	Singlet	Immune_cells
SAT_2M	3021	1616	0.330033	0	Singlet	Adipocyte
SAT_2M	2465	1378	0.645682	0	Singlet	Adipocyte
SAT_2M	1959	1128	3.9634146	0	Singlet	Adipocyte
SAT_2M	1016	672	0.5876592	4	Singlet	Immune_cells
SAT_2M	1266	857	0.8661417	1	Singlet	Immune_cells
SAT_2M	1673	968	7.8571429	1	Singlet	Immune_cells
SAT_2M	1218	801	0.9787928	0	Singlet	Adipocyte
SAT_2M	4169	2134	1.3161043	11	Singlet	Smooth_muscle_cells
SAT_2M	2542	1463	0.7442225	11	Singlet	Smooth_muscle_cells
SAT_2M	1313	844	2.8809704	1	Singlet	Immune_cells
SAT_2M	2247	1060	9.7604259	3	Singlet	FAPs
SAT_2M	1183	797	0.1683502	0	Singlet	Adipocyte
SAT_2M	1047	687	3.8058991	4	Singlet	Immune_cells
SAT_2M	2037	1233	1.1741683	1	Singlet	Immune_cells
SAT_2M	1057	725	0.7532957	5	Singlet	Immune_cells
SAT_2M	1254	827	0.2384738	4	Singlet	Immune_cells
SAT_2M	1349	821	8.4008843	1	Singlet	Immune_cells
SAT_2M	1444	1009	1.0951403	8	Singlet	Mesothelial
SAT_2M	2513	1434	3.6139793	1	Singlet	Immune_cells
SAT_2M	1596	980	3.320802	1	Singlet	Immune_cells

SAT_2M	1546	909	0.064433	10	Singlet	Immune_cells
SAT_2M	1253	807	2.4603175	8	Singlet	Mesothelial
SAT_2M	1619	1005	0.0613497	4	Singlet	Immune_cells
SAT_2M	1225	799	3.493095	1	Singlet	Immune_cells
SAT_2M	3169	1642	0.4094488	11	Singlet	Smooth_muscle_cells
SAT_2M	1338	864	1.7176998	1	Singlet	Immune_cells
SAT_2M	1910	1140	0.3636364	0	Singlet	Adipocyte
SAT_2M	1017	693	6.1643836	4	Singlet	Immune_cells
SAT_2M	1476	825	8.7897228	1	Singlet	Immune_cells
SAT_2M	1857	1033	7.1734475	1	Singlet	Immune_cells
SAT_2M	1366	840	0.5847953	1	Singlet	Immune_cells
SAT_2M	1748	1088	0.1136364	11	Singlet	Smooth_muscle_cells
SAT_2M	1492	1017	0.7323569	1	Singlet	Immune_cells
SAT_2M	1575	909	7.3371284	1	Singlet	Immune_cells
SAT_2M	2387	1371	0.8347245	0	Singlet	Adipocyte
SAT_2M	3002	1586	8.2141669	4	Singlet	Immune_cells
SAT_2M	4251	2152	5.4720526	8	Singlet	Mesothelial
SAT_2M	1868	1042	6.1497326	1	Singlet	Immune_cells
SAT_2M	1243	780	1.3632719	4	Singlet	Immune_cells
SAT_2M	4249	2031	0.4691532	0	Singlet	Adipocyte
SAT_2M	2142	1228	0.3242242	0	Singlet	Adipocyte
SAT_2M	1343	903	1.4880952	1	Singlet	Immune_cells
SAT_2M	1871	1191	1.3800425	11	Singlet	Smooth_muscle_cells
SAT_2M	2368	1285	1.137321	0	Singlet	Adipocyte
SAT_2M	2218	1219	6.1911171	4	Singlet	Immune_cells
SAT_2M	1908	1180	1.7259414	4	Singlet	Immune_cells
SAT_2M	2730	1537	0.2550091	0	Singlet	Adipocyte
SAT_2M	1302	757	1.0695187	7	Singlet	Endothelial_cells
SAT_2M	4548	2045	1.0309278	10	Singlet	Immune_cells
SAT_2M	1550	938	5.3949904	1	Singlet	Immune_cells
SAT_2M	2275	1295	7.3011734	5	Singlet	Immune_cells
SAT_2M	1630	1050	2.490887	0	Singlet	Adipocyte
SAT_2M	1165	720	2.5706941	0	Singlet	Adipocyte
SAT_2M	1035	655	0.6743738	4	Singlet	Immune_cells
SAT_2M	1213	801	2.2094926	5	Singlet	Immune_cells
SAT_2M	1627	971	1.3431013	0	Singlet	Adipocyte
SAT_2M	1713	990	8.1049563	1	Singlet	Immune_cells
SAT_2M	1256	774	1.9669552	3	Singlet	FAPs
SAT_2M	1490	951	3.1375167	1	Singlet	Immune_cells
SAT_2M	1396	857	0.21398	0	Singlet	Adipocyte
SAT_2M	1615	1008	0.3705991	11	Singlet	Smooth_muscle_cells
SAT_2M	4460	1908	0.8950548	10	Singlet	Immune_cells
SAT_2M	1745	1029	0.3957038	0	Singlet	Adipocyte
SAT_2M	1420	934	1.0445682	3	Singlet	FAPs
SAT_2M	1065	699	0	11	Singlet	Smooth_muscle_cells
SAT_2M	1331	861	0.2987304	5	Singlet	Immune_cells
SAT_2M	5857	2539	0.1360313	0	Singlet	Adipocyte
SAT_2M	2298	1289	0.7826087	3	Singlet	FAPs
SAT_2M	1664	944	9.760479	1	Singlet	Immune_cells
SAT_2M	2010	1241	0.8919723	9	Singlet	Mesothelial
SAT_2M	1392	861	5.3724928	1	Singlet	Immune_cells

SAT_2M	1592	1023	1.0598504	1	Singlet	Immune_cells
SAT_2M	3573	1769	6.6945607	9	Singlet	Mesothelial
SAT_2M	3379	1631	4.5172719	1	Singlet	Immune_cells
SAT_2M	1549	949	0.9633911	4	Singlet	Immune_cells
SAT_2M	1338	849	1.1185682	1	Singlet	Immune_cells
SAT_2M	3029	1617	3.5985474	1	Singlet	Immune_cells
SAT_2M	1532	949	3.1838856	1	Singlet	Immune_cells
SAT_2M	1023	687	5.4966249	1	Singlet	Immune_cells
SAT_2M	4932	2111	6.6343042	9	Singlet	Mesothelial
SAT_2M	2558	1409	0.2341007	0	Singlet	Adipocyte
SAT_2M	2090	1261	0.2847651	4	Singlet	Immune_cells
SAT_2M	1297	830	0.5347594	0	Singlet	Adipocyte
SAT_2M	2452	1290	8.7912088	1	Singlet	Immune_cells
SAT_2M	1661	973	0.5988024	4	Singlet	Immune_cells
SAT_2M	1848	1042	5.9331176	4	Singlet	Immune_cells
SAT_2M	1231	832	0.7258065	4	Singlet	Immune_cells
SAT_2M	1520	874	6.9599475	8	Singlet	Mesothelial
SAT_2M	1112	793	0.2688172	1	Singlet	Immune_cells
SAT_2M	2172	1227	7.7838828	1	Singlet	Immune_cells
SAT_2M	2312	1352	0.0859107	6	Singlet	Neurons
SAT_2M	1640	980	1.0309278	0	Singlet	Adipocyte
SAT_2M	1460	976	1.0217984	1	Singlet	Immune_cells
SAT_2M	1082	649	8.0406654	0	Singlet	Adipocyte
SAT_2M	1200	790	2.2350993	1	Singlet	Immune_cells
SAT_2M	1991	1172	3.8825286	1	Singlet	Immune_cells
SAT_2M	1718	1077	0.6391633	1	Singlet	Immune_cells
SAT_2M	1543	858	0.1899937	6	Singlet	Neurons
SAT_2M	1884	1051	2.065678	10	Singlet	Immune_cells
SAT_2M	2001	1227	0.5979073	11	Singlet	Smooth_muscle_cells
SAT_2M	1470	859	6.4276049	6	Singlet	Neurons
SAT_2M	1532	975	1.4267185	0	Singlet	Adipocyte
SAT_2M	1586	949	5.6532663	4	Singlet	Immune_cells
SAT_2M	1781	1017	3.7535014	1	Singlet	Immune_cells
SAT_2M	5495	2722	5.7179161	9	Singlet	Mesothelial
SAT_2M	1457	907	0.8898015	4	Singlet	Immune_cells
SAT_2M	1329	847	0.0749064	1	Singlet	Immune_cells
SAT_2M	1554	994	0.2570694	4	Singlet	Immune_cells
SAT_2M	1081	702	1.3799448	4	Singlet	Immune_cells
SAT_2M	6189	2448	3.5979348	10	Singlet	Immune_cells
SAT_2M	1769	1051	0.2820079	4	Singlet	Immune_cells
SAT_2M	1123	759	0.4436557	4	Singlet	Immune_cells
SAT_2M	1008	645	1.1846002	1	Singlet	Immune_cells
SAT_2M	1416	867	12.350035	1	Singlet	Immune_cells
SAT_2M	1962	1169	0	8	Singlet	Mesothelial
SAT_2M	1283	831	0.3881988	0	Singlet	Adipocyte
SAT_2M	2856	1596	5.7759221	4	Singlet	Immune_cells
SAT_2M	1474	971	3.4552846	4	Singlet	Immune_cells
SAT_2M	1868	938	8.5026738	0	Singlet	Adipocyte
SAT_2M	1448	834	5.5785124	1	Singlet	Immune_cells
SAT_2M	1369	829	4.8762737	1	Singlet	Immune_cells
SAT_2M	1275	764	2.1840874	5	Singlet	Immune_cells

SAT_2M	2301	1405	2.3326134	4	Singlet	Immune_cells
SAT_2M	2207	1236	6.4050519	1	Singlet	Immune_cells
SAT_2M	1295	820	0.5384615	0	Singlet	Adipocyte
SAT_2M	1960	1190	2.5980642	1	Singlet	Immune_cells
SAT_2M	1697	976	3.0570253	1	Singlet	Immune_cells
SAT_2M	3341	1477	2.7446301	10	Singlet	Immune_cells
SAT_2M	1043	671	4.9760766	4	Singlet	Immune_cells
SAT_2M	1552	847	8.7820513	5	Singlet	Immune_cells
SAT_2M	1445	871	0.4118051	0	Singlet	Adipocyte
SAT_2M	1574	915	2.0189274	3	Singlet	FAPs
SAT_2M	4009	2067	5.2945563	5	Singlet	Immune_cells
SAT_2M	1260	842	0.8695652	1	Singlet	Immune_cells
SAT_2M	1307	824	8.3079268	1	Singlet	Immune_cells
SAT_2M	2332	1357	0.2997859	4	Singlet	Immune_cells
SAT_2M	1450	875	2.3415978	1	Singlet	Immune_cells
SAT_2M	1233	796	0.5649718	1	Singlet	Immune_cells
SAT_2M	1594	968	7.4328545	4	Singlet	Immune_cells
SAT_2M	3025	1380	7.4695624	10	Singlet	Immune_cells
SAT_2M	1944	1151	6.6052227	1	Singlet	Immune_cells
SAT_2M	1542	966	1.0981912	1	Singlet	Immune_cells
SAT_2M	1386	830	8.5858586	1	Singlet	Immune_cells
SAT_2M	1093	708	5.7534247	8	Singlet	Mesothelial
SAT_2M	2288	1254	13.141862	1	Singlet	Immune_cells
SAT_2M	2147	1306	0.4618938	4	Singlet	Immune_cells
SAT_2M	1228	763	11.941511	5	Singlet	Immune_cells
SAT_2M	2230	1184	8.0197133	10	Singlet	Immune_cells
SAT_2M	1371	765	8.8064047	0	Singlet	Adipocyte
SAT_2M	1120	794	1.7761989	7	Singlet	Endothelial_cells
SAT_2M	1820	1088	2.6790596	5	Singlet	Immune_cells
SAT_2M	1217	789	3.5977105	4	Singlet	Immune_cells
SAT_2M	1670	1055	2.202381	1	Singlet	Immune_cells
SAT_2M	4250	1950	0.2579737	0	Singlet	Adipocyte
SAT_2M	1102	723	0.4524887	4	Singlet	Immune_cells
SAT_2M	1536	935	0.5844156	4	Singlet	Immune_cells
SAT_2M	1065	645	0.4655493	3	Singlet	FAPs
SAT_2M	2067	1240	0.3381643	0	Singlet	Adipocyte
SAT_2M	3263	1662	0.4881025	0	Singlet	Adipocyte
SAT_2M	1461	981	2.503383	4	Singlet	Immune_cells
SAT_2M	1297	785	4.2177914	1	Singlet	Immune_cells
SAT_2M	1176	771	0.508044	4	Singlet	Immune_cells
SAT_2M	1081	774	2.7598896	1	Singlet	Immune_cells
SAT_2M	1151	762	0.952381	4	Singlet	Immune_cells
SAT_2M	1621	1004	5.4301403	1	Singlet	Immune_cells
SAT_2M	6457	2923	4.5644438	9	Singlet	Mesothelial
SAT_2M	1193	837	0.4173623	11	Singlet	Smooth_muscle_cells
SAT_2M	2671	1435	0.1120657	0	Singlet	Adipocyte
SAT_2M	3295	1185	0.9399636	10	Singlet	Immune_cells
SAT_2M	1396	816	10.6201	0	Singlet	Adipocyte
SAT_2M	1449	906	3.024055	4	Singlet	Immune_cells
SAT_2M	1122	729	2.2222222	1	Singlet	Immune_cells
SAT_2M	1451	936	0.2061856	1	Singlet	Immune_cells

SAT_2M	1840	1027	4.7593294	0	Singlet	Adipocyte
SAT_2M	1178	781	0.5932203	9	Singlet	Mesothelial
SAT_2M	1512	968	0.9198423	1	Singlet	Immune_cells
SAT_2M	1954	1161	6.6428206	5	Singlet	Immune_cells
SAT_2M	1191	754	0.1663894	0	Singlet	Adipocyte
SAT_2M	1798	1073	0.4994451	2	Singlet	FAPs
SAT_2M	1626	1013	0.9107468	0	Singlet	Adipocyte
SAT_2M	1789	973	5.5586437	1	Singlet	Immune_cells
SAT_2M	1918	1172	1.7027864	6	Singlet	Neurons
SAT_2M	1556	851	3.2133676	10	Singlet	Immune_cells
SAT_2M	1031	676	1.0617761	4	Singlet	Immune_cells
SAT_2M	1358	847	4.981685	1	Singlet	Immune_cells
SAT_2M	1112	684	2.8699552	0	Singlet	Adipocyte
SAT_2M	1308	852	3.6557502	1	Singlet	Immune_cells
SAT_2M	2646	1431	3.2015066	1	Singlet	Immune_cells
SAT_2M	1692	1000	1.3545347	1	Singlet	Immune_cells
SAT_2M	1278	808	4.9960968	1	Singlet	Immune_cells
SAT_2M	1408	883	2.6969482	1	Singlet	Immune_cells
SAT_2M	1042	657	0.6666667	0	Singlet	Adipocyte
SAT_2M	3199	1690	2.3371767	8	Singlet	Mesothelial
SAT_2M	1282	797	5.982906	1	Singlet	Immune_cells
SAT_2M	1146	743	0.7765315	4	Singlet	Immune_cells
SAT_2M	1568	972	1.0094637	1	Singlet	Immune_cells
SAT_2M	1182	828	0.6728343	5	Singlet	Immune_cells
SAT_2M	1756	1098	0.2832861	1	Singlet	Immune_cells
SAT_2M	2029	1199	3.5907526	10	Singlet	Immune_cells
SAT_2M	2610	1477	0.6097561	0	Singlet	Adipocyte
SAT_2M	1139	669	2.0087336	3	Singlet	FAPs
SAT_2M	1520	944	1.5758372	4	Singlet	Immune_cells
SAT_2M	1449	817	6.2715369	1	Singlet	Immune_cells
SAT_2M	1671	1020	7.2152654	1	Singlet	Immune_cells
SAT_2M	1124	769	0.3536693	0	Singlet	Adipocyte
SAT_2M	1163	813	0.5097706	1	Singlet	Immune_cells
SAT_2M	2109	1198	0.8045433	3	Singlet	FAPs
SAT_2M	2163	1309	3.5961272	4	Singlet	Immune_cells
SAT_2M	1852	1161	3.7176724	5	Singlet	Immune_cells
SAT_2M	1686	1053	1.1764706	1	Singlet	Immune_cells
SAT_2M	1324	848	0.4524887	1	Singlet	Immune_cells
SAT_2M	2167	1286	0.5960569	4	Singlet	Immune_cells
SAT_2M	2206	1231	0.4972875	0	Singlet	Adipocyte
SAT_2M	2470	1397	0.4437273	8	Singlet	Mesothelial
SAT_2M	1820	1035	6.1235648	4	Singlet	Immune_cells
SAT_2M	1598	957	8.0524345	1	Singlet	Immune_cells
SAT_2M	1752	1105	0.3977273	1	Singlet	Immune_cells
SAT_2M	2224	1353	0.5829596	0	Singlet	Adipocyte
SAT_2M	1177	780	0.58873	0	Singlet	Adipocyte
SAT_2M	1068	706	6.4007421	1	Singlet	Immune_cells
SAT_2M	1453	930	0.7544582	4	Singlet	Immune_cells
SAT_2M	1415	927	4.4086774	5	Singlet	Immune_cells
SAT_2M	6245	2439	1.1653895	10	Singlet	Immune_cells
SAT_2M	1353	882	5.2475979	4	Singlet	Immune_cells

SAT_2M	1292	834	1.006192	4	Singlet	Immune_cells
SAT_2M	4337	2086	0.2065168	0	Singlet	Adipocyte
SAT_2M	1163	725	0.1713796	0	Singlet	Adipocyte
SAT_2M	2108	1214	0.8506616	0	Singlet	Adipocyte
SAT_2M	1157	697	1.8134715	1	Singlet	Immune_cells
SAT_2M	1296	848	0.6163328	4	Singlet	Immune_cells
SAT_2M	1282	778	1.1609907	1	Singlet	Immune_cells
SAT_2M	1294	791	0.3076923	0	Singlet	Adipocyte
SAT_2M	2195	1228	0.4083485	0	Singlet	Adipocyte
SAT_2M	1085	735	4.5413261	1	Singlet	Immune_cells
SAT_2M	1497	928	1.3990673	1	Singlet	Immune_cells
SAT_2M	1462	942	4.2205582	8	Singlet	Mesothelial
SAT_2M	1123	676	0.530504	6	Singlet	Neurons
SAT_2M	1041	679	7.2380952	1	Singlet	Immune_cells
SAT_2M	1475	965	3.3624748	8	Singlet	Mesothelial
SAT_2M	1063	682	0.9380863	4	Singlet	Immune_cells
SAT_2M	1267	820	6.0534591	4	Singlet	Immune_cells
SAT_2M	1016	639	2.1632252	7	Singlet	Endothelial_cells
SAT_2M	1270	813	1.5540016	4	Singlet	Immune_cells
SAT_2M	2064	1180	4.6859903	1	Singlet	Immune_cells
SAT_2M	1819	1171	1.3691128	4	Singlet	Immune_cells
SAT_2M	1137	741	0.1748252	4	Singlet	Immune_cells
SAT_2M	1436	938	0.3477051	4	Singlet	Immune_cells
SAT_2M	1170	756	0.170068	3	Singlet	FAPs
SAT_2M	1705	1068	3.9204213	1	Singlet	Immune_cells
SAT_2M	1363	847	0.0732064	1	Singlet	Immune_cells
SAT_2M	1664	983	1.7899761	1	Singlet	Immune_cells
SAT_2M	1212	795	2.707137	1	Singlet	Immune_cells
SAT_2M	1288	795	4.0989946	1	Singlet	Immune_cells
SAT_2M	1092	726	1.9195612	4	Singlet	Immune_cells
SAT_2M	1414	887	0	0	Singlet	Adipocyte
SAT_2M	1591	960	2.5529265	1	Singlet	Immune_cells
SAT_2M	1951	1172	1.3306039	1	Singlet	Immune_cells
SAT_2M	1633	996	0.304878	3	Singlet	FAPs
SAT_2M	1266	754	4.570528	1	Singlet	Immune_cells
SAT_2M	1225	869	0.4022526	1	Singlet	Immune_cells
SAT_2M	1670	1055	0.5369928	4	Singlet	Immune_cells
SAT_2M	1136	784	0.7901668	3	Singlet	FAPs
SAT_2M	2062	1247	2.4154589	1	Singlet	Immune_cells
SAT_2M	1009	620	0.4945598	8	Singlet	Mesothelial
SAT_2M	1208	755	0.0825083	4	Singlet	Immune_cells
SAT_2M	4302	2044	1.4143288	10	Singlet	Immune_cells
SAT_2M	1062	645	6.1567164	1	Singlet	Immune_cells
SAT_2M	1216	767	9.0612245	1	Singlet	Immune_cells
SAT_2M	2151	1213	4.0816327	1	Singlet	Immune_cells
SAT_2M	1220	709	14.367347	1	Singlet	Immune_cells
SAT_2M	1642	1039	1.9382193	1	Singlet	Immune_cells
SAT_2M	3281	1649	5.4506699	8	Singlet	Mesothelial
SAT_2M	2149	1272	3.6228518	4	Singlet	Immune_cells
SAT_2M	3118	1584	6.474359	1	Singlet	Immune_cells
SAT_2M	1176	743	2.2015241	8	Singlet	Mesothelial

SAT_2M	1988	1152	0.1002506	0	Singlet	Adipocyte
SAT_2M	1324	802	4.4360902	1	Singlet	Immune_cells
SAT_2M	4144	2137	9.2266924	4	Singlet	Immune_cells
SAT_2M	1391	887	0.6446991	0	Singlet	Adipocyte
SAT_2M	1540	958	0.9038089	1	Singlet	Immune_cells
SAT_2M	5559	2337	0.3403798	0	Singlet	Adipocyte
SAT_2M	1403	819	1.9914651	4	Singlet	Immune_cells
SAT_2M	1589	970	5.8197747	1	Singlet	Immune_cells
SAT_2M	1437	959	1.1126565	1	Singlet	Immune_cells
SAT_2M	2092	1114	4.148784	0	Singlet	Adipocyte
SAT_2M	1380	851	0.216763	5	Singlet	Immune_cells
SAT_2M	2102	1129	3.1783681	10	Singlet	Immune_cells
SAT_2M	1342	861	1.6332591	1	Singlet	Immune_cells
SAT_2M	2166	1224	0.6868132	0	Singlet	Adipocyte
SAT_2M	1101	723	0.8035714	4	Singlet	Immune_cells
SAT_2M	1348	790	5.1035503	1	Singlet	Immune_cells
SAT_2M	1207	838	0.410509	0	Singlet	Adipocyte
SAT_2M	3706	1781	0.1613337	0	Singlet	Adipocyte
SAT_2M	1028	664	1.9138756	5	Singlet	Immune_cells
SAT_2M	1044	661	0.7662835	1	Singlet	Immune_cells
SAT_2M	1423	927	0.3508772	0	Singlet	Adipocyte
SAT_2M	1304	866	0.0765697	5	Singlet	Immune_cells
SAT_2M	1023	580	6.4453125	5	Singlet	Immune_cells
SAT_2M	1179	740	0.7607777	4	Singlet	Immune_cells
SAT_2M	2324	1415	0.1710864	5	Singlet	Immune_cells
SAT_2M	1306	759	10.738766	1	Singlet	Immune_cells
SAT_2M	1369	868	7.8717201	1	Singlet	Immune_cells
SAT_2M	2153	1299	3.3890436	1	Singlet	Immune_cells
SAT_2M	1817	1197	0.1641138	4	Singlet	Immune_cells
SAT_2M	3382	1475	0.7674144	10	Singlet	Immune_cells
SAT_2M	2218	1263	0.6714414	0	Singlet	Adipocyte
SAT_2M	1263	793	2.4486572	1	Singlet	Immune_cells
SAT_2M	3787	1723	3.1603898	2	Singlet	FAPs
SAT_2M	1245	749	9.2295345	1	Singlet	Immune_cells
SAT_2M	1183	838	0.2529511	3	Singlet	FAPs
SAT_2M	1459	899	3.4836066	1	Singlet	Immune_cells
SAT_2M	1254	817	1.1867089	1	Singlet	Immune_cells
SAT_2M	1791	1125	0.7777778	0	Singlet	Adipocyte
SAT_2M	1252	811	0.1593626	0	Singlet	Adipocyte
SAT_2M	1672	976	3.6352801	1	Singlet	Immune_cells
SAT_2M	2038	1225	3.6728697	1	Singlet	Immune_cells
SAT_2M	1353	850	2.9325513	1	Singlet	Immune_cells
SAT_2M	1245	835	0.1598721	8	Singlet	Mesothelial
SAT_2M	1120	777	0.7104796	3	Singlet	FAPs
SAT_2M	1817	1053	3.4520548	1	Singlet	Immune_cells
SAT_2M	1966	1152	5.906108	4	Singlet	Immune_cells
SAT_2M	2050	1188	2.5304136	1	Singlet	Immune_cells
SAT_2M	1080	765	1.559633	1	Singlet	Immune_cells
SAT_2M	1727	1021	0.4039238	0	Singlet	Adipocyte
SAT_2M	1764	1085	1.8018018	4	Singlet	Immune_cells
SAT_2M	1759	1051	0.5665722	4	Singlet	Immune_cells

SAT_2M	2434	1345	0.7374027	0	Singlet	Adipocyte
SAT_2M	1555	933	1.5286624	0	Singlet	Adipocyte
SAT_2M	1062	760	0.1874414	5	Singlet	Immune_cells
SAT_2M	1416	937	0.4210526	5	Singlet	Immune_cells
SAT_2M	1308	854	3.4351145	4	Singlet	Immune_cells
SAT_2M	1269	772	5.0235479	1	Singlet	Immune_cells
SAT_2M	1100	740	0	1	Singlet	Immune_cells
SAT_2M	1079	690	1.0128913	4	Singlet	Immune_cells
SAT_2M	2327	1353	2.8718388	4	Singlet	Immune_cells
SAT_2M	2147	1161	3.5266821	10	Singlet	Immune_cells
SAT_2M	1417	872	2.5334272	4	Singlet	Immune_cells
SAT_2M	2753	1458	1.8076645	0	Singlet	Adipocyte
SAT_2M	1084	696	5.5860806	1	Singlet	Immune_cells
SAT_2M	1282	790	5.5598456	1	Singlet	Immune_cells
SAT_2M	2630	1428	12.168309	4	Singlet	Immune_cells
SAT_2M	1166	760	0.1712329	2	Singlet	FAPs
SAT_2M	1013	676	0.0986193	1	Singlet	Immune_cells
SAT_2M	2221	1296	2.1152115	4	Singlet	Immune_cells
SAT_2M	1334	870	1.2724551	1	Singlet	Immune_cells
SAT_2M	2350	1307	0.2538071	0	Singlet	Adipocyte
SAT_2M	2633	1479	5.026455	5	Singlet	Immune_cells
SAT_2M	1362	761	7.6302274	0	Singlet	Adipocyte
SAT_2M	2229	1321	0.2238138	8	Singlet	Mesothelial
SAT_2M	1099	723	4.0797824	4	Singlet	Immune_cells
SAT_2M	1039	664	0.2887392	0	Singlet	Adipocyte
SAT_2M	1520	1058	0.3913894	4	Singlet	Immune_cells
SAT_2M	1097	726	0.5449591	4	Singlet	Immune_cells
SAT_2M	1405	898	1.910828	3	Singlet	FAPs
SAT_2M	2890	1530	8.1034483	1	Singlet	Immune_cells
SAT_2M	1454	860	5.4869685	1	Singlet	Immune_cells
SAT_2M	1317	837	0.1515152	1	Singlet	Immune_cells
SAT_2M	1004	711	0.8823529	6	Singlet	Neurons
SAT_2M	1092	711	7.4954296	5	Singlet	Immune_cells
SAT_2M	1047	692	1.3346044	4	Singlet	Immune_cells
SAT_2M	1860	1106	0.1607717	0	Singlet	Adipocyte
SAT_2M	1631	1032	5.0214329	1	Singlet	Immune_cells
SAT_2M	1486	912	0.5354752	0	Singlet	Adipocyte
SAT_2M	1315	827	6.3732929	1	Singlet	Immune_cells
SAT_2M	1439	946	0.2770083	4	Singlet	Immune_cells
SAT_2M	1100	756	1.4492754	1	Singlet	Immune_cells
SAT_2M	1530	916	5.8708415	3	Singlet	FAPs
SAT_2M	6230	2501	1.2810248	10	Singlet	Immune_cells
SAT_2M	3574	1915	0.1111729	4	Singlet	Immune_cells
SAT_2M	2359	1225	1.1849344	0	Singlet	Adipocyte
SAT_2M	2502	1405	0.3577107	0	Singlet	Adipocyte
SAT_2M	1458	946	4.2436687	7	Singlet	Endothelial_cells
SAT_2M	1608	1014	0.3095975	0	Singlet	Adipocyte
SAT_2M	1029	696	2.7079304	1	Singlet	Immune_cells
SAT_2M	1103	755	0.2680965	5	Singlet	Immune_cells
SAT_2M	1098	781	0.5449591	5	Singlet	Immune_cells
SAT_2M	1498	914	0.7338225	0	Singlet	Adipocyte

SAT_2M	2750	1449	2.2133527	10	Singlet	Immune_cells
SAT_2M	1549	987	2.7634961	1	Singlet	Immune_cells
SAT_2M	1125	771	2.1276596	9	Singlet	Mesothelial
SAT_2M	1321	811	3.1626506	4	Singlet	Immune_cells
SAT_2M	2319	1290	0.7311828	0	Singlet	Adipocyte
SAT_2M	1132	742	0.6167401	4	Singlet	Immune_cells
SAT_2M	2170	1261	0.3205128	0	Singlet	Adipocyte
SAT_2M	2279	1150	3.6355672	5	Singlet	Immune_cells
SAT_2M	1210	815	1.8976898	11	Singlet	Smooth_muscle_cells
SAT_2M	2847	1442	1.997897	10	Singlet	Immune_cells
SAT_2M	1402	841	0.212917	1	Singlet	Immune_cells
SAT_2M	1499	896	4.5816733	0	Singlet	Adipocyte
SAT_2M	1512	947	1.7786561	0	Singlet	Adipocyte
SAT_2M	2097	1193	0.2376426	4	Singlet	Immune_cells
SAT_2M	1946	1215	0.6646217	4	Singlet	Immune_cells
SAT_2M	1485	924	12.358804	5	Singlet	Immune_cells
SAT_2M	1486	894	7.3627845	1	Singlet	Immune_cells
SAT_2M	2829	1477	0.7054674	3	Singlet	FAPs
SAT_2M	2080	1192	0.2876318	0	Singlet	Adipocyte
SAT_2M	1774	1071	2.2446689	0	Singlet	Adipocyte
SAT_2M	1532	979	2.0182292	1	Singlet	Immune_cells
SAT_2M	1462	960	0.3376097	4	Singlet	Immune_cells
SAT_2M	1100	721	3.3273381	3	Singlet	FAPs
SAT_2M	2471	1358	6.0532688	4	Singlet	Immune_cells
SAT_2M	2089	1213	1.1927481	0	Singlet	Adipocyte
SAT_2M	1406	874	1.9787986	4	Singlet	Immune_cells
SAT_2M	1419	871	6.7558058	1	Singlet	Immune_cells
SAT_2M	1313	849	0.6079027	1	Singlet	Immune_cells
SAT_2M	1452	915	0.6177076	1	Singlet	Immune_cells
SAT_2M	1497	1016	0.464499	4	Singlet	Immune_cells
SAT_2M	1467	879	3.8565629	4	Singlet	Immune_cells
SAT_2M	1184	776	2.2689076	1	Singlet	Immune_cells
SAT_2M	1271	851	0.862069	2	Singlet	FAPs
SAT_2M	1422	919	5.087108	1	Singlet	Immune_cells
SAT_2M	1112	718	0.4476276	4	Singlet	Immune_cells
SAT_2M	1515	933	4.2077581	5	Singlet	Immune_cells
SAT_2M	1333	938	1.1869436	5	Singlet	Immune_cells
SAT_2M	1730	1044	1.3848817	3	Singlet	FAPs
SAT_2M	1865	1124	2.5572722	4	Singlet	Immune_cells
SAT_2M	1144	698	5.9947871	0	Singlet	Adipocyte
SAT_2M	2716	1349	4.8144065	10	Singlet	Immune_cells
SAT_2M	1979	1108	5.0728277	4	Singlet	Immune_cells
SAT_2M	1267	806	2.679275	4	Singlet	Immune_cells
SAT_2M	1512	940	3.2343234	1	Singlet	Immune_cells
SAT_2M	1245	765	6.4748201	1	Singlet	Immune_cells
SAT_2M	1239	704	13.043478	1	Singlet	Immune_cells
SAT_2M	1666	1039	1.5550239	4	Singlet	Immune_cells
SAT_2M	1805	1000	3.0973451	3	Singlet	FAPs
SAT_2M	1382	881	1.7844397	4	Singlet	Immune_cells
SAT_2M	3265	1642	0.2134146	0	Singlet	Adipocyte
SAT_2M	1353	828	3.7555228	4	Singlet	Immune_cells

SAT_2M	1454	925	0.1375516	4	Singlet	Immune_cells
SAT_2M	1157	736	4.2241379	2	Singlet	FAPs
SAT_2M	1611	931	4.8387097	1	Singlet	Immune_cells
SAT_2M	1633	954	9.4454601	1	Singlet	Immune_cells
SAT_2M	1089	717	0.5459509	1	Singlet	Immune_cells
SAT_2M	1751	1065	1.5384615	1	Singlet	Immune_cells
SAT_2M	1534	964	5.0847458	1	Singlet	Immune_cells
SAT_2M	1282	837	3.6604361	4	Singlet	Immune_cells
SAT_2M	1445	885	0.6194081	0	Singlet	Adipocyte
SAT_2M	2016	1192	3.5061728	4	Singlet	Immune_cells
SAT_2M	1626	963	0.7334963	0	Singlet	Adipocyte
SAT_2M	1008	665	0	1	Singlet	Immune_cells
SAT_2M	3699	1899	0.7808293	2	Singlet	FAPs
SAT_2M	1228	800	4.291498	1	Singlet	Immune_cells
SAT_2M	1307	795	2.9029794	0	Singlet	Adipocyte
SAT_2M	1098	778	0.4528986	7	Singlet	Endothelial_cells
SAT_2M	1361	868	1.3899049	1	Singlet	Immune_cells
SAT_2M	2233	1374	5.57041	8	Singlet	Mesothelial
SAT_2M	3157	1621	1.290932	0	Singlet	Adipocyte
SAT_2M	1269	789	1.3333333	10	Singlet	Immune_cells
SAT_2M	1865	1073	1.1235955	4	Singlet	Immune_cells
SAT_2M	1286	778	0.620155	4	Singlet	Immune_cells
SAT_2M	1791	1119	3.6707453	1	Singlet	Immune_cells
SAT_2M	1383	868	3.0042918	4	Singlet	Immune_cells
SAT_2M	1263	808	4.3341214	4	Singlet	Immune_cells
SAT_2M	2337	1340	0.5106383	0	Singlet	Adipocyte
SAT_2M	1032	678	0	1	Singlet	Immune_cells
SAT_2M	1409	953	0.210084	8	Singlet	Mesothelial
SAT_2M	1581	993	0.9410289	0	Singlet	Adipocyte
SAT_2M	1667	1083	0.7789095	5	Singlet	Immune_cells
SAT_2M	1324	811	10.248681	4	Singlet	Immune_cells
SAT_2M	1961	1132	4.7352342	1	Singlet	Immune_cells
SAT_2M	2208	1378	0.3155996	8	Singlet	Mesothelial
SAT_2M	1084	695	0.1833181	0	Singlet	Adipocyte
SAT_2M	1952	1082	0.9615385	9	Singlet	Mesothelial
SAT_2M	2016	1148	1.9782394	7	Singlet	Endothelial_cells
SAT_2M	2535	1450	0.2749411	0	Singlet	Adipocyte
SAT_2M	2182	1241	1.9186843	4	Singlet	Immune_cells
SAT_2M	1318	845	3.1698113	1	Singlet	Immune_cells
SAT_2M	2692	1499	0.9214891	0	Singlet	Adipocyte
SAT_2M	1413	968	0.2111189	5	Singlet	Immune_cells
SAT_2M	2386	1275	10.208333	5	Singlet	Immune_cells
SAT_2M	1511	927	4.743083	1	Singlet	Immune_cells
SAT_2M	1525	941	0.3267974	4	Singlet	Immune_cells
SAT_2M	2196	1232	0.7262823	6	Singlet	Neurons
SAT_2M	2292	1321	0.6533101	10	Singlet	Immune_cells
SAT_2M	1464	954	1.2837838	1	Singlet	Immune_cells
SAT_2M	1772	1099	3.1584884	1	Singlet	Immune_cells
SAT_2M	2831	1714	0.2103049	4	Singlet	Immune_cells
SAT_2M	3993	1841	1.4246438	5	Singlet	Immune_cells
SAT_2M	1398	861	0.3558719	1	Singlet	Immune_cells

SAT_2M	2111	1295	0.8486563	5	Singlet	Immune_cells
SAT_2M	3449	1713	0.3468208	0	Singlet	Adipocyte
SAT_2M	1187	805	0.4194631	11	Singlet	Smooth_muscle_cells
SAT_2M	1925	1109	6.5939772	1	Singlet	Immune_cells
SAT_2M	2545	1469	2.0272904	5	Singlet	Immune_cells
SAT_2M	1012	658	0.1966568	4	Singlet	Immune_cells
SAT_2M	2108	1127	5.6105611	9	Singlet	Mesothelial
SAT_2M	1315	790	2.193646	1	Singlet	Immune_cells
SAT_2M	5070	2091	1.0234206	10	Singlet	Immune_cells
SAT_2M	1181	810	0.5076142	4	Singlet	Immune_cells
SAT_2M	5103	2305	10.64371	9	Singlet	Mesothelial
SAT_2M	4946	1859	1.9785988	10	Singlet	Immune_cells
SAT_2M	2169	1224	0.0915751	0	Singlet	Adipocyte
SAT_2M	1163	769	0.0859107	0	Singlet	Adipocyte
SAT_2M	1250	773	0.3968254	0	Singlet	Adipocyte
SAT_2M	1717	1015	3.7101449	1	Singlet	Immune_cells
SAT_2M	1855	1129	7.0475174	1	Singlet	Immune_cells
SAT_2M	2181	1185	3.9215686	3	Singlet	FAPs
SAT_2M	1555	892	9.8392283	1	Singlet	Immune_cells
SAT_2M	1165	742	1.4542344	1	Singlet	Immune_cells
SAT_2M	1498	883	6.5649867	1	Singlet	Immune_cells
SAT_2M	1808	1081	6.0154525	1	Singlet	Immune_cells
SAT_2M	2130	1257	2.9990628	8	Singlet	Mesothelial
SAT_2M	1816	1135	2.247807	4	Singlet	Immune_cells
SAT_2M	1834	1020	0.595883	0	Singlet	Adipocyte
SAT_2M	1219	804	1.3854931	4	Singlet	Immune_cells
SAT_2M	1046	696	0.8538899	3	Singlet	FAPs
SAT_2M	3137	1685	0.6977482	0	Singlet	Adipocyte
SAT_2M	2058	1194	1.3982642	0	Singlet	Adipocyte
SAT_2M	2790	1475	0.7142857	2	Singlet	FAPs
SAT_2M	1708	970	9.2657343	1	Singlet	Immune_cells
SAT_2M	1632	973	0.1219512	4	Singlet	Immune_cells
SAT_2M	1272	791	9.0766823	1	Singlet	Immune_cells
SAT_2M	1538	1001	0.6443299	8	Singlet	Mesothelial
SAT_2M	2480	1373	14.903846	5	Singlet	Immune_cells
SAT_2M	2781	1447	0.7899461	0	Singlet	Adipocyte
SAT_2M	1559	941	0.7042254	4	Singlet	Immune_cells
SAT_2M	1605	973	4.4776119	1	Singlet	Immune_cells
SAT_2M	1087	688	0.091659	4	Singlet	Immune_cells
SAT_2M	1635	963	2.3114355	4	Singlet	Immune_cells
SAT_2M	2471	1335	3.1880549	0	Singlet	Adipocyte
SAT_2M	2758	1464	0.1082251	0	Singlet	Adipocyte
SAT_2M	2650	1402	0.6012777	0	Singlet	Adipocyte
SAT_2M	9785	3699	8.1097623	9	Singlet	Mesothelial
SAT_2M	1314	803	2.4297646	4	Singlet	Immune_cells
SAT_2M	1490	954	3.3400134	5	Singlet	Immune_cells
SAT_2M	1360	889	1.9061584	1	Singlet	Immune_cells
SAT_2M	1543	980	1.1605416	3	Singlet	FAPs
SAT_2M	1865	1147	1.5491453	4	Singlet	Immune_cells
SAT_2M	1827	1070	5.0328228	1	Singlet	Immune_cells
SAT_2M	1362	840	8.5043988	1	Singlet	Immune_cells

SAT_2M	1802	1145	6.0454798	1	Singlet	Immune_cells
SAT_2M	1120	743	0.530504	0	Singlet	Adipocyte
SAT_2M	1307	820	2.972561	1	Singlet	Immune_cells
SAT_2M	5055	2037	1.0073079	2	Singlet	FAPs
SAT_2M	3229	1491	1.7906761	10	Singlet	Immune_cells
SAT_2M	1438	998	0.5536332	4	Singlet	Immune_cells
SAT_2M	1910	1054	5.4364872	1	Singlet	Immune_cells
SAT_2M	4366	1933	2.0576132	5	Singlet	Immune_cells
SAT_2M	1038	690	2.2051774	4	Singlet	Immune_cells
SAT_2M	2287	1377	0.8278867	3	Singlet	FAPs
SAT_2M	1574	986	2.528445	1	Singlet	Immune_cells
SAT_2M	2184	1287	0	8	Singlet	Mesothelial
SAT_2M	7010	3050	11.065224	4	Singlet	Immune_cells
SAT_2M	2506	1432	7.0461783	9	Singlet	Mesothelial
SAT_2M	2105	1103	14.042553	1	Singlet	Immune_cells
SAT_2M	3615	1779	0.8008837	11	Singlet	Smooth_muscle_cells
SAT_2M	1232	872	0.798722	0	Singlet	Adipocyte
SAT_2M	1570	1007	0.0632511	2	Singlet	FAPs
SAT_2M	1714	1115	1.5125073	1	Singlet	Immune_cells
SAT_2M	1181	730	0.3358522	1	Singlet	Immune_cells
SAT_2M	1056	692	2.1698113	1	Singlet	Immune_cells
SAT_2M	1739	1024	9.1065292	1	Singlet	Immune_cells
SAT_2M	2320	1257	1.1177988	0	Singlet	Adipocyte
SAT_2M	1606	1017	3.9130435	1	Singlet	Immune_cells
SAT_2M	1517	952	0.7905138	0	Singlet	Adipocyte
SAT_2M	1680	1069	4.682869	1	Singlet	Immune_cells
SAT_2M	1404	894	1.5602837	4	Singlet	Immune_cells
SAT_2M	1355	785	0.6617647	4	Singlet	Immune_cells
SAT_2M	1043	694	0.0956023	1	Singlet	Immune_cells
SAT_2M	1478	930	1.4874915	4	Singlet	Immune_cells
SAT_2M	1212	854	1.8837019	1	Singlet	Immune_cells
SAT_2M	1537	974	4.6722907	1	Singlet	Immune_cells
SAT_2M	1108	765	6.7736185	6	Singlet	Neurons
SAT_2M	1664	972	0.059988	1	Singlet	Immune_cells
SAT_2M	1481	950	5.0301811	1	Singlet	Immune_cells
SAT_2M	3112	1588	0.3526771	0	Singlet	Adipocyte
SAT_2M	1341	843	4.6130952	4	Singlet	Immune_cells
SAT_2M	4178	1993	9.436216	5	Singlet	Immune_cells
SAT_2M	1491	900	1.6722408	4	Singlet	Immune_cells
SAT_2M	1533	861	9.7529259	1	Singlet	Immune_cells
SAT_2M	1029	654	1.2536162	0	Singlet	Adipocyte
SAT_2M	1766	1051	1.4140271	0	Singlet	Adipocyte
SAT_2M	1126	766	1.6769638	1	Singlet	Immune_cells
SAT_2M	1420	919	0.3479471	4	Singlet	Immune_cells
SAT_2M	1148	705	0.5172414	1	Singlet	Immune_cells
SAT_2M	1106	751	0.0902527	1	Singlet	Immune_cells
SAT_2M	1441	879	8.005521	4	Singlet	Immune_cells
SAT_2M	2963	1582	1.4765101	0	Singlet	Adipocyte
SAT_2M	3393	1870	1.3811343	9	Singlet	Mesothelial
SAT_2M	2561	1455	0.2329193	0	Singlet	Adipocyte
SAT_2M	1170	737	3.1489362	3	Singlet	FAPs

SAT_2M	1228	796	0.973236	1	Singlet	Immune_cells
SAT_2M	4313	1971	1.8514233	2	Singlet	FAPs
SAT_2M	1212	739	4.3513957	0	Singlet	Adipocyte
SAT_2M	1304	857	0.304414	1	Singlet	Immune_cells
SAT_2M	1645	1073	0.5373134	4	Singlet	Immune_cells
SAT_2M	7670	3183	3.6704412	9	Singlet	Mesothelial
SAT_2M	1530	977	0.5208333	4	Singlet	Immune_cells
SAT_2M	1287	767	1.7857143	0	Singlet	Adipocyte
SAT_2M	1226	775	0.4068348	4	Singlet	Immune_cells
SAT_2M	1232	805	6.5164924	4	Singlet	Immune_cells
SAT_2M	2067	1196	8.011583	1	Singlet	Immune_cells
SAT_2M	1091	726	0.0909091	3	Singlet	FAPs
SAT_2M	1371	881	2.7636364	1	Singlet	Immune_cells
SAT_2M	1390	764	6.09319	9	Singlet	Mesothelial
SAT_2M	1774	1033	0.9009009	0	Singlet	Adipocyte
SAT_2M	1038	700	3.4058657	4	Singlet	Immune_cells
SAT_2M	1241	800	5.6134723	1	Singlet	Immune_cells
SAT_2M	1022	688	0.2929688	4	Singlet	Immune_cells
SAT_2M	1199	770	4.9084859	1	Singlet	Immune_cells
SAT_2M	1800	1075	1.1086475	0	Singlet	Adipocyte
SAT_2M	1381	904	0.3610108	4	Singlet	Immune_cells
SAT_2M	1846	1067	4.6511628	1	Singlet	Immune_cells
SAT_2M	1876	1095	0.4257584	4	Singlet	Immune_cells
SAT_2M	5444	2110	2.0913594	10	Singlet	Immune_cells
SAT_2M	1332	875	2.827381	1	Singlet	Immune_cells
SAT_2M	1088	750	1.2658228	4	Singlet	Immune_cells
SAT_2M	7083	2488	2.6104134	10	Singlet	Immune_cells
SAT_2M	1554	949	1.5296367	0	Singlet	Adipocyte
SAT_2M	1431	952	2.1557719	1	Singlet	Immune_cells
SAT_2M	4161	1989	0.1197318	0	Singlet	Adipocyte
SAT_2M	1229	844	0.5681818	4	Singlet	Immune_cells
SAT_2M	3656	1539	1.7472017	10	Singlet	Immune_cells
SAT_2M	1076	718	3.6044362	4	Singlet	Immune_cells
SAT_2M	1750	1053	3.8176638	1	Singlet	Immune_cells
SAT_2M	1715	981	12.398137	1	Singlet	Immune_cells
SAT_2M	3399	1716	2.170088	0	Singlet	Adipocyte
SAT_2M	1579	1032	0.1893939	5	Singlet	Immune_cells
SAT_2M	1688	1012	2.6035503	1	Singlet	Immune_cells
SAT_2M	1594	972	3.1269543	4	Singlet	Immune_cells
SAT_2M	1254	895	0.9531374	8	Singlet	Mesothelial
SAT_2M	1285	817	0.7763975	1	Singlet	Immune_cells
SAT_2M	1713	1106	0.6376812	1	Singlet	Immune_cells
SAT_2M	1834	1191	0.1085776	4	Singlet	Immune_cells
SAT_2M	1292	780	7.9414032	1	Singlet	Immune_cells
SAT_2M	1601	1057	2.4968789	1	Singlet	Immune_cells
SAT_2M	1198	763	0.5823627	0	Singlet	Adipocyte
SAT_2M	5947	2687	7.5478028	9	Singlet	Mesothelial
SAT_2M	1117	792	0.9743136	4	Singlet	Immune_cells
SAT_2M	1898	1147	2.0472441	10	Singlet	Immune_cells
SAT_2M	4717	2030	1.3744978	10	Singlet	Immune_cells
SAT_2M	2726	1408	0.5486467	0	Singlet	Adipocyte

SAT_2M	1267	839	0.5520505	4	Singlet	Immune_cells
SAT_2M	1664	1018	0.059952	1	Singlet	Immune_cells
SAT_2M	1469	958	1.5582656	4	Singlet	Immune_cells
SAT_2M	1001	658	2.2862823	3	Singlet	FAPs
SAT_2M	3785	1880	0.2637131	2	Singlet	FAPs
SAT_2M	2267	1275	0.437254	0	Singlet	Adipocyte
SAT_2M	2623	1386	1.1039208	0	Singlet	Adipocyte
SAT_2M	1196	811	0.4177109	4	Singlet	Immune_cells
SAT_2M	1152	755	0.3460208	4	Singlet	Immune_cells
SAT_2M	1902	1189	4.1535226	4	Singlet	Immune_cells
SAT_2M	1746	1129	3.0821918	1	Singlet	Immune_cells
SAT_2M	1765	1029	7.6314302	0	Singlet	Adipocyte
SAT_2M	1253	861	0.3930818	4	Singlet	Immune_cells
SAT_2M	2497	1463	1.8769968	8	Singlet	Mesothelial
SAT_2M	1982	1172	0.1005025	0	Singlet	Adipocyte
SAT_2M	1823	1142	0.2189382	5	Singlet	Immune_cells
SAT_2M	1266	832	2.6025237	1	Singlet	Immune_cells
SAT_2M	1564	946	2.4234694	1	Singlet	Immune_cells
SAT_2M	1292	861	4.0123457	1	Singlet	Immune_cells
SAT_2M	2558	1424	0.1169591	0	Singlet	Adipocyte
SAT_2M	1368	917	0.2909091	1	Singlet	Immune_cells
SAT_2M	1522	917	9.102816	1	Singlet	Immune_cells
SAT_2M	1432	915	3.5986159	4	Singlet	Immune_cells
SAT_2M	1107	721	5.2867384	1	Singlet	Immune_cells
SAT_2M	1600	994	2.1091811	5	Singlet	Immune_cells
SAT_2M	1874	1118	5.9136921	1	Singlet	Immune_cells
SAT_2M	2130	1207	3.3566434	0	Singlet	Adipocyte
SAT_2M	3179	1656	2.256346	8	Singlet	Mesothelial
SAT_2M	1290	804	9.2449923	1	Singlet	Immune_cells
SAT_2M	1132	730	0.7943513	5	Singlet	Immune_cells
SAT_2M	3665	1795	0.7070982	0	Singlet	Adipocyte
SAT_2M	1034	676	8.2851638	1	Singlet	Immune_cells
SAT_2M	1006	678	0.3956479	4	Singlet	Immune_cells
SAT_2M	3141	1612	0.9847522	0	Singlet	Adipocyte
SAT_2M	1339	846	3.4149963	1	Singlet	Immune_cells
SAT_2M	2860	1463	9.9930119	1	Singlet	Immune_cells
SAT_2M	1627	988	3.4292713	1	Singlet	Immune_cells
SAT_2M	1322	883	1.7950636	1	Singlet	Immune_cells
SAT_2M	1521	937	1.4332248	6	Singlet	Neurons
SAT_2M	2102	1283	0.2843602	0	Singlet	Adipocyte
SAT_2M	1491	918	4.0053405	1	Singlet	Immune_cells
SAT_2M	1197	797	2.7477102	10	Singlet	Immune_cells
SAT_2M	1538	992	2.8976175	1	Singlet	Immune_cells
SAT_2M	2383	1389	0.8343763	8	Singlet	Mesothelial
SAT_2M	2320	1319	3.5698925	4	Singlet	Immune_cells
SAT_2M	1497	892	0.998004	1	Singlet	Immune_cells
SAT_2M	1089	728	0.2734731	4	Singlet	Immune_cells
SAT_2M	2150	1159	1.5806602	3	Singlet	FAPs
SAT_2M	1411	872	0.5657709	1	Singlet	Immune_cells
SAT_2M	1377	858	4.765343	1	Singlet	Immune_cells
SAT_2M	1644	957	2.4242424	5	Singlet	Immune_cells

SAT_2M	3200	1749	0.5902454	8	Singlet	Mesothelial
SAT_2M	2583	1344	0.8104979	3	Singlet	FAPs
SAT_2M	1328	853	2.5583145	1	Singlet	Immune_cells
SAT_2M	1573	1020	1.8975332	5	Singlet	Immune_cells
SAT_2M	1280	815	0.5460218	4	Singlet	Immune_cells
SAT_2M	1283	882	0.5417957	4	Singlet	Immune_cells
SAT_2M	1281	790	4.040404	1	Singlet	Immune_cells
SAT_2M	1766	1076	1.1267606	7	Singlet	Endothelial_cells
SAT_2M	1273	841	0.5451713	1	Singlet	Immune_cells
SAT_2M	7313	3012	3.1275608	9	Singlet	Mesothelial
SAT_2M	1264	772	13.779528	4	Singlet	Immune_cells
SAT_2M	1036	708	1.4354067	4	Singlet	Immune_cells
SAT_2M	1880	1098	0.2119767	1	Singlet	Immune_cells
SAT_2M	1015	629	0.3929273	1	Singlet	Immune_cells
SAT_2M	1413	923	1.8361582	0	Singlet	Adipocyte
SAT_2M	1318	766	9.1597275	1	Singlet	Immune_cells
SAT_2M	1216	799	1.5548282	4	Singlet	Immune_cells
SAT_2M	1043	668	0.4784689	1	Singlet	Immune_cells
SAT_2M	1702	1036	0.5284792	0	Singlet	Adipocyte
SAT_2M	1705	1043	0.2912056	0	Singlet	Adipocyte
SAT_2M	2785	1414	1.3953488	0	Singlet	Adipocyte
SAT_2M	1296	799	0.3849115	4	Singlet	Immune_cells
SAT_2M	1927	1129	0.9264025	0	Singlet	Adipocyte
SAT_2M	1346	779	9.5484826	0	Singlet	Adipocyte
SAT_2M	1127	747	0.3542958	1	Singlet	Immune_cells
SAT_2M	1310	823	0.9863429	1	Singlet	Immune_cells
SAT_2M	1161	816	1.2908778	5	Singlet	Immune_cells
SAT_2M	1265	815	1.336478	0	Singlet	Adipocyte
SAT_2M	2887	1556	2.4922118	9	Singlet	Mesothelial
SAT_2M	1301	783	8.9723926	1	Singlet	Immune_cells
SAT_2M	1819	1191	0.9304871	11	Singlet	Smooth_muscle_cells
SAT_2M	1336	843	2.2338049	0	Singlet	Adipocyte
SAT_2M	1353	871	2.7900147	1	Singlet	Immune_cells
SAT_2M	1362	864	1.2426901	1	Singlet	Immune_cells
SAT_2M	1981	1121	6.3819095	1	Singlet	Immune_cells
SAT_2M	1199	808	0.4163197	7	Singlet	Endothelial_cells
SAT_2M	1082	731	0.0922509	4	Singlet	Immune_cells
SAT_2M	2035	1180	3.1357178	1	Singlet	Immune_cells
SAT_2M	1175	787	1.1854361	1	Singlet	Immune_cells
SAT_2M	1244	840	0.3187251	3	Singlet	FAPs
SAT_2M	1198	788	0.4139073	0	Singlet	Adipocyte
SAT_2M	1154	788	2.7562446	1	Singlet	Immune_cells
SAT_2M	5307	2417	4.5300752	9	Singlet	Mesothelial
SAT_2M	1672	979	5.9311981	1	Singlet	Immune_cells
SAT_2M	2383	1326	0.3753128	0	Singlet	Adipocyte
SAT_2M	1681	1070	0.1184834	8	Singlet	Mesothelial
SAT_2M	2108	1257	5.0614948	1	Singlet	Immune_cells
SAT_2M	1509	1007	0.5948447	4	Singlet	Immune_cells
SAT_2M	3661	1832	0.0544366	0	Singlet	Adipocyte
SAT_2M	1021	658	1.5655577	0	Singlet	Adipocyte
SAT_2M	1979	1163	0.1006036	0	Singlet	Adipocyte

SAT_2M	1716	1033	0.1737116	0	Singlet	Adipocyte
SAT_2M	1246	772	3.2721468	1	Singlet	Immune_cells
SAT_2M	2236	1331	0.6690455	1	Singlet	Immune_cells
SAT_2M	1178	742	0.5071851	8	Singlet	Mesothelial
SAT_2M	1969	1075	1.0080645	1	Singlet	Immune_cells
SAT_2M	1721	1034	5.561993	1	Singlet	Immune_cells
SAT_2M	1423	935	1.054111	4	Singlet	Immune_cells
SAT_2M	1847	1040	9.3783494	1	Singlet	Immune_cells
SAT_2M	1360	833	4.4787078	1	Singlet	Immune_cells
SAT_2M	1538	984	0.3878474	1	Singlet	Immune_cells
SAT_2M	1765	1047	0.1128032	0	Singlet	Adipocyte
SAT_2M	2487	1425	0.3996803	6	Singlet	Neurons
SAT_2M	1280	893	1.7107309	4	Singlet	Immune_cells
SAT_2M	2240	1288	5.1601423	5	Singlet	Immune_cells
SAT_2M	1552	991	0.8365508	3	Singlet	FAPs
SAT_2M	2154	1300	0.1848429	4	Singlet	Immune_cells
SAT_2M	1166	737	1.283148	4	Singlet	Immune_cells
SAT_2M	1348	892	4.804139	4	Singlet	Immune_cells
SAT_2M	1449	811	12.57732	1	Singlet	Immune_cells
SAT_2M	1576	993	0.6979695	1	Singlet	Immune_cells
SAT_2M	3236	1725	1.1405672	10	Singlet	Immune_cells
SAT_2M	1397	903	0.3579098	4	Singlet	Immune_cells
SAT_2M	1686	1016	2.7202839	0	Singlet	Adipocyte
SAT_2M	1426	860	1.958042	5	Singlet	Immune_cells
SAT_2M	1307	809	5.1948052	1	Singlet	Immune_cells
SAT_2M	2102	1163	0.3796868	8	Singlet	Mesothelial
SAT_2M	1519	997	0.3931848	4	Singlet	Immune_cells
SAT_2M	3148	1619	0.6647673	0	Singlet	Adipocyte
SAT_2M	1092	723	0.8189263	1	Singlet	Immune_cells
SAT_2M	1830	1108	0.6528836	11	Singlet	Smooth_muscle_cells
SAT_2M	1383	814	1.373825	0	Singlet	Adipocyte
SAT_2M	2400	1465	0.1244297	4	Singlet	Immune_cells
SAT_2M	1016	686	0.5865103	3	Singlet	FAPs
SAT_2M	1221	726	2.5326797	0	Singlet	Adipocyte
SAT_2M	7676	3431	7.2644574	9	Singlet	Mesothelial
SAT_2M	2262	1315	2.1126761	0	Singlet	Adipocyte
SAT_2M	1837	1089	4.076087	4	Singlet	Immune_cells
SAT_2M	1227	845	0.242915	3	Singlet	FAPs
SAT_2M	2191	1266	4.5454545	9	Singlet	Mesothelial
SAT_2M	2363	1347	0.1688476	0	Singlet	Adipocyte
SAT_2M	3245	1719	0.122737	0	Singlet	Adipocyte
SAT_2M	10869	4334	7.0496324	9	Singlet	Mesothelial
SAT_2M	1008	715	3.0632411	11	Singlet	Smooth_muscle_cells
SAT_2M	1637	1068	1.8159806	5	Singlet	Immune_cells
SAT_2M	2061	1136	12.015504	9	Singlet	Mesothelial
SAT_2M	1527	949	1.0430248	1	Singlet	Immune_cells
SAT_2M	3439	1703	4.2608696	0	Singlet	Adipocyte
SAT_2M	1232	825	0.6472492	0	Singlet	Adipocyte
SAT_2M	3500	1839	11.126961	8	Singlet	Mesothelial
SAT_2M	2737	1581	0.5830904	1	Singlet	Immune_cells
SAT_2M	1950	1086	3.4693878	1	Singlet	Immune_cells

SAT_2M	2625	1497	6.6996574	1	Singlet	Immune_cells
SAT_2M	1311	851	0.3799392	0	Singlet	Adipocyte
SAT_2M	1620	1021	7.3230769	1	Singlet	Immune_cells
SAT_2M	1382	986	0.4307251	4	Singlet	Immune_cells
SAT_2M	1559	943	0.1274697	0	Singlet	Adipocyte
SAT_2M	1172	775	0.6779661	3	Singlet	FAPs
SAT_2M	1333	852	0.4484305	4	Singlet	Immune_cells
SAT_2M	3061	1684	0.4874878	0	Singlet	Adipocyte
SAT_2M	2302	1285	4.529767	0	Singlet	Adipocyte
SAT_2M	1641	987	2.1726011	1	Singlet	Immune_cells
SAT_2M	1300	831	0.6097561	0	Singlet	Adipocyte
SAT_2M	1377	929	2.0999276	1	Singlet	Immune_cells
SAT_2M	1399	900	6.2588905	1	Singlet	Immune_cells
SAT_2M	2795	1651	0.1428571	5	Singlet	Immune_cells
SAT_2M	1546	952	7.4550129	4	Singlet	Immune_cells
SAT_2M	1239	766	3.935743	1	Singlet	Immune_cells
SAT_2M	1245	840	2.1445592	4	Singlet	Immune_cells
SAT_2M	1844	1075	4.5479155	4	Singlet	Immune_cells
SAT_2M	1895	1087	7.0526316	9	Singlet	Mesothelial
SAT_2M	1223	727	1.8744906	1	Singlet	Immune_cells
SAT_2M	1428	900	0.6968641	0	Singlet	Adipocyte
SAT_2M	1746	1110	0.1712329	4	Singlet	Immune_cells
SAT_2M	1635	1033	2.1315469	4	Singlet	Immune_cells
SAT_2M	1682	1022	1.7730496	4	Singlet	Immune_cells
SAT_2M	1014	680	1.4778325	8	Singlet	Mesothelial
SAT_2M	1516	959	2.8928337	3	Singlet	FAPs
SAT_2M	1457	984	2.739726	1	Singlet	Immune_cells
SAT_2M	1290	832	3.2457496	1	Singlet	Immune_cells
SAT_2M	1568	971	1.7721519	1	Singlet	Immune_cells
SAT_2M	1911	1155	2.9796132	1	Singlet	Immune_cells
SAT_2M	1873	1155	0.532198	4	Singlet	Immune_cells
SAT_2M	1948	1098	5.0025523	1	Singlet	Immune_cells
SAT_2M	1158	757	0.2581756	11	Singlet	Smooth_muscle_cells
SAT_2M	1337	834	1.2639405	1	Singlet	Immune_cells
SAT_2M	2165	1223	5.649977	9	Singlet	Mesothelial
SAT_2M	2272	1331	2.7204914	9	Singlet	Mesothelial
SAT_2M	1811	1130	0.8820287	4	Singlet	Immune_cells
SAT_2M	1482	959	0.0668003	0	Singlet	Adipocyte
SAT_2M	1090	714	1.0938924	1	Singlet	Immune_cells
SAT_2M	1534	888	7.5471698	1	Singlet	Immune_cells
SAT_2M	2114	1255	3.5781544	2	Singlet	FAPs
SAT_2M	1965	1144	7.5339026	5	Singlet	Immune_cells
SAT_2M	1075	723	0.1853568	4	Singlet	Immune_cells
SAT_2M	1553	956	0.1284522	4	Singlet	Immune_cells
SAT_2M	1361	897	1.3225569	1	Singlet	Immune_cells
SAT_2M	1398	867	6.3662375	1	Singlet	Immune_cells
SAT_2M	1029	729	1.7424976	1	Singlet	Immune_cells
SAT_2M	1171	778	1.6183986	4	Singlet	Immune_cells
SAT_2M	1787	1067	2.5626741	1	Singlet	Immune_cells
SAT_2M	1190	833	1.1666667	8	Singlet	Mesothelial
SAT_2M	1516	939	4.91481	3	Singlet	FAPs

SAT_2M	1503	865	8.8430851	1	Singlet	Immune_cells
SAT_2M	1735	1072	3.2664756	0	Singlet	Adipocyte
SAT_2M	11669	4206	2.533379	2	Singlet	FAPs
SAT_2M	1261	731	0.8702532	3	Singlet	FAPs
SAT_2M	1318	803	2.1953066	0	Singlet	Adipocyte
SAT_2M	2888	1385	1.6218081	10	Singlet	Immune_cells
SAT_2M	1085	645	1.7431193	7	Singlet	Endothelial_cells
SAT_2M	1290	820	2.7006173	1	Singlet	Immune_cells
SAT_2M	1467	1012	1.5509103	4	Singlet	Immune_cells
SAT_2M	1364	857	0.5763689	0	Singlet	Adipocyte
SAT_2M	5268	2225	2.1722705	10	Singlet	Immune_cells
SAT_2M	7244	2860	6.717137	4	Singlet	Immune_cells
SAT_2M	1847	1048	1.5616586	5	Singlet	Immune_cells
SAT_2M	1438	934	1.8030513	4	Singlet	Immune_cells
SAT_2M	1484	916	0.6729475	1	Singlet	Immune_cells
SAT_2M	1223	860	1.5359741	1	Singlet	Immune_cells
SAT_2M	1080	693	2.2201665	1	Singlet	Immune_cells
SAT_2M	1477	941	0.1348618	8	Singlet	Mesothelial
SAT_2M	2469	1359	3.266129	1	Singlet	Immune_cells
SAT_2M	1634	994	4.0928528	1	Singlet	Immune_cells
SAT_2M	1029	735	0.4789272	1	Singlet	Immune_cells
SAT_2M	1273	816	0.3888025	0	Singlet	Adipocyte
SAT_2M	3785	2044	6.1084781	4	Singlet	Immune_cells
SAT_2M	1852	1080	10.580021	1	Singlet	Immune_cells
SAT_2M	1280	775	8.5271318	1	Singlet	Immune_cells
SAT_2M	1748	1083	0.6837607	2	Singlet	FAPs
SAT_2M	2647	1504	2.2820801	1	Singlet	Immune_cells
SAT_2M	1354	852	0.2941176	0	Singlet	Adipocyte
SAT_2M	1064	703	6.2383613	9	Singlet	Mesothelial
SAT_2M	1073	736	0.3693444	4	Singlet	Immune_cells
SAT_2M	1952	1189	1.9928462	4	Singlet	Immune_cells
SAT_2M	2550	1499	6.0179758	9	Singlet	Mesothelial
SAT_2M	1263	800	1.6600791	4	Singlet	Immune_cells
SAT_2M	1252	801	9.1917591	1	Singlet	Immune_cells
SAT_2M	1163	749	0.5136986	4	Singlet	Immune_cells
SAT_2M	1320	913	0.9803922	11	Singlet	Smooth_muscle_cells
SAT_2M	1389	838	4.0143369	1	Singlet	Immune_cells
SAT_2M	2668	1363	3.4444028	0	Singlet	Adipocyte
SAT_2M	1270	751	1.2588513	0	Singlet	Adipocyte
SAT_2M	1299	828	6.0582822	10	Singlet	Immune_cells
SAT_2M	1057	692	0.6578947	0	Singlet	Adipocyte
SAT_2M	1015	683	1.373896	4	Singlet	Immune_cells
SAT_2M	1052	674	0.4748338	0	Singlet	Adipocyte
SAT_2M	1614	1034	1.6019717	7	Singlet	Endothelial_cells
SAT_2M	2534	1365	0.1573564	0	Singlet	Adipocyte
SAT_2M	1159	760	2.4158758	1	Singlet	Immune_cells
SAT_2M	1443	829	0.3422313	4	Singlet	Immune_cells
SAT_2M	2882	1492	4.9792531	5	Singlet	Immune_cells
SAT_2M	1959	1126	0	0	Singlet	Adipocyte
SAT_2M	1231	783	1.0526316	1	Singlet	Immune_cells
SAT_2M	1259	775	0.1582278	1	Singlet	Immune_cells

SAT_2M	1551	913	0.5787781	2	Singlet	FAPs
SAT_2M	1136	789	0.2631579	4	Singlet	Immune_cells
SAT_2M	1998	990	1.2961117	10	Singlet	Immune_cells
SAT_2M	1255	846	4.2925278	4	Singlet	Immune_cells
SAT_2M	1230	796	0.4862237	0	Singlet	Adipocyte
SAT_2M	2033	1211	0.3929273	4	Singlet	Immune_cells
SAT_2M	1106	708	0.1798561	1	Singlet	Immune_cells
SAT_2M	2022	1230	5.1282051	1	Singlet	Immune_cells
SAT_2M	1377	934	2.8673835	11	Singlet	Smooth_muscle_cells
SAT_2M	2562	1325	2.8793774	10	Singlet	Immune_cells
SAT_2M	1426	843	11.629526	1	Singlet	Immune_cells
SAT_2M	2212	1294	1.8410418	1	Singlet	Immune_cells
SAT_2M	2038	1208	0.8308895	1	Singlet	Immune_cells
SAT_2M	1132	756	0.877193	7	Singlet	Endothelial_cells
SAT_2M	2779	1521	8.1003584	1	Singlet	Immune_cells
SAT_2M	1343	873	0.1480385	0	Singlet	Adipocyte
SAT_2M	2158	1224	3.8781163	0	Singlet	Adipocyte
SAT_2M	1910	1267	0.2075765	4	Singlet	Immune_cells
SAT_2M	1514	912	3.0880421	1	Singlet	Immune_cells
SAT_2M	1204	771	1.3234078	1	Singlet	Immune_cells
SAT_2M	1476	913	6.2795409	1	Singlet	Immune_cells
SAT_2M	2200	1302	3.851382	1	Singlet	Immune_cells
SAT_2M	2302	1300	1.7764298	0	Singlet	Adipocyte
SAT_2M	1168	728	2.5553663	3	Singlet	FAPs
SAT_2M	1107	714	1.440144	5	Singlet	Immune_cells
SAT_2M	1247	816	0.4803843	1	Singlet	Immune_cells
SAT_2M	3203	1680	3.1434796	4	Singlet	Immune_cells
SAT_2M	1298	820	7.296467	4	Singlet	Immune_cells
SAT_2M	1744	1103	5.2451539	8	Singlet	Mesothelial
SAT_2M	1359	895	3.4331629	1	Singlet	Immune_cells
SAT_2M	2392	1316	4.704413	1	Singlet	Immune_cells
SAT_2M	1677	1047	0.9501188	3	Singlet	FAPs
SAT_2M	1252	778	8.3794466	4	Singlet	Immune_cells
SAT_2M	3845	1855	0.5450298	0	Singlet	Adipocyte
SAT_2M	1434	929	2.0876827	11	Singlet	Smooth_muscle_cells
SAT_2M	1606	972	5.4692356	1	Singlet	Immune_cells
SAT_2M	1483	875	1.138647	0	Singlet	Adipocyte
SAT_2M	2820	1531	1.6572638	0	Singlet	Adipocyte
SAT_2M	1607	1028	5.0216987	4	Singlet	Immune_cells
SAT_2M	3361	1816	0.2368967	5	Singlet	Immune_cells
SAT_2M	1115	793	0.969163	4	Singlet	Immune_cells
SAT_2M	5810	2326	1.647503	0	Singlet	Adipocyte
SAT_2M	1251	782	4.2264753	4	Singlet	Immune_cells
SAT_2M	1508	884	4.7051027	4	Singlet	Immune_cells
SAT_2M	3451	1715	1.2124711	0	Singlet	Adipocyte
SAT_2M	3181	1650	3.9510818	1	Singlet	Immune_cells
SAT_2M	1523	963	2.3963731	6	Singlet	Neurons
SAT_2M	2407	1263	6.8680182	5	Singlet	Immune_cells
SAT_2M	1037	675	3.3589251	11	Singlet	Smooth_muscle_cells
SAT_2M	1540	982	0.9721322	0	Singlet	Adipocyte
SAT_2M	2010	1173	2.8741328	5	Singlet	Immune_cells

SAT_2M	1058	686	5.7601511	5	Singlet	Immune_cells
SAT_2M	4024	1967	2.5793651	10	Singlet	Immune_cells
SAT_2M	1444	884	0.137836	1	Singlet	Immune_cells
SAT_2M	1998	1094	7.089366	1	Singlet	Immune_cells
SAT_2M	1365	835	0.8035062	0	Singlet	Adipocyte
SAT_2M	1409	903	1.5602837	8	Singlet	Mesothelial
SAT_2M	1227	781	4.3938161	2	Singlet	FAPs
SAT_2M	5883	2530	0.1697793	0	Singlet	Adipocyte
SAT_2M	1540	951	9.9354839	4	Singlet	Immune_cells
SAT_2M	1764	1041	0.3393665	0	Singlet	Adipocyte
SAT_2M	1079	698	3.9704524	1	Singlet	Immune_cells
SAT_2M	1097	715	0.7226739	4	Singlet	Immune_cells
SAT_2M	1202	835	1.0726073	6	Singlet	Neurons
SAT_2M	1942	1102	3.3898305	0	Singlet	Adipocyte
SAT_2M	1261	828	0.390625	4	Singlet	Immune_cells
SAT_2M	1590	993	5.0062578	1	Singlet	Immune_cells
SAT_2M	2141	1110	3.6864209	0	Singlet	Adipocyte
SAT_2M	1243	719	0.6415397	3	Singlet	FAPs
SAT_2M	1229	734	8.2792208	1	Singlet	Immune_cells
SAT_2M	1361	865	0.5102041	4	Singlet	Immune_cells
SAT_2M	1353	911	1.5418502	4	Singlet	Immune_cells
SAT_2M	2261	1225	0.5298013	10	Singlet	Immune_cells
SAT_2M	2363	1374	0.0842815	5	Singlet	Immune_cells
SAT_2M	1122	682	7.2888889	5	Singlet	Immune_cells
SAT_2M	1302	838	0.1531394	8	Singlet	Mesothelial
SAT_2M	1287	814	1.3157895	3	Singlet	FAPs
SAT_2M	1334	814	0.2240478	1	Singlet	Immune_cells
SAT_2M	1075	712	1.843318	4	Singlet	Immune_cells
SAT_2M	1374	904	0.1440922	4	Singlet	Immune_cells
SAT_2M	1325	825	2.2607385	0	Singlet	Adipocyte
SAT_2M	3455	1736	0.1445087	3	Singlet	FAPs
SAT_2M	1691	1056	2.3557126	4	Singlet	Immune_cells
SAT_2M	1071	778	2.0072993	8	Singlet	Mesothelial
SAT_2M	1113	749	0.2688172	1	Singlet	Immune_cells
SAT_2M	2888	1571	1.6179002	5	Singlet	Immune_cells
SAT_2M	2935	1483	4.047619	10	Singlet	Immune_cells
SAT_2M	1716	1067	0.6956522	8	Singlet	Mesothelial
SAT_2M	1405	830	4.0311174	1	Singlet	Immune_cells
SAT_2M	2511	1486	0.5564388	3	Singlet	FAPs
SAT_2M	4433	2215	7.2698627	4	Singlet	Immune_cells
SAT_2M	1940	1175	2.4640657	8	Singlet	Mesothelial
SAT_2M	3144	1261	0.6660324	10	Singlet	Immune_cells
SAT_2M	1525	1001	0.1306336	0	Singlet	Adipocyte
SAT_2M	5125	2087	2.0463847	10	Singlet	Immune_cells
SAT_2M	1491	1006	0.8660893	0	Singlet	Adipocyte
SAT_2M	1669	929	0.8328376	0	Singlet	Adipocyte
SAT_2M	1767	1058	1.2935883	1	Singlet	Immune_cells
SAT_2M	3180	1698	1.4433637	1	Singlet	Immune_cells
SAT_2M	1268	749	4.015748	0	Singlet	Adipocyte
SAT_2M	1274	819	0.6987578	1	Singlet	Immune_cells
SAT_2M	1012	693	0.4906771	1	Singlet	Immune_cells

SAT_2M	1485	977	2.4617432	1	Singlet	Immune_cells
SAT_2M	1854	1071	0.915455	0	Singlet	Adipocyte
SAT_2M	2687	1456	1.038961	3	Singlet	FAPs
SAT_2M	1391	835	5.0251256	1	Singlet	Immune_cells
SAT_2M	3190	1856	3.319762	9	Singlet	Mesothelial
SAT_2M	1123	764	0.3552398	1	Singlet	Immune_cells
SAT_2M	1531	812	2.2193211	10	Singlet	Immune_cells
SAT_2M	1311	884	0.6828528	4	Singlet	Immune_cells
SAT_2M	5028	2028	0.6558029	10	Singlet	Immune_cells
SAT_2M	1265	806	0.5494505	1	Singlet	Immune_cells
SAT_2M	1376	881	0.7919366	0	Singlet	Adipocyte
SAT_2M	1303	791	0.6891271	1	Singlet	Immune_cells
SAT_2M	1368	828	8.490566	1	Singlet	Immune_cells
SAT_2M	1024	684	0.6789525	1	Singlet	Immune_cells
SAT_2M	1003	637	0.0995025	1	Singlet	Immune_cells
SAT_2M	1796	1033	1.9422863	7	Singlet	Endothelial_cells
SAT_2M	1273	809	0.1566171	1	Singlet	Immune_cells
SAT_2M	1835	1199	0.2164502	4	Singlet	Immune_cells
SAT_2M	1255	801	3.0134814	1	Singlet	Immune_cells
SAT_2M	1267	759	0.9419152	2	Singlet	FAPs
SAT_2M	1012	640	0.7889546	0	Singlet	Adipocyte
SAT_2M	1747	1067	2.1664766	1	Singlet	Immune_cells
SAT_2M	2479	1470	0.7237636	8	Singlet	Mesothelial
SAT_2M	4801	2070	7.722649	0	Singlet	Adipocyte
SAT_2M	1738	1014	4.5819015	4	Singlet	Immune_cells
SAT_2M	1705	1067	5.5911474	7	Singlet	Endothelial_cells
SAT_2M	1613	987	3.2756489	1	Singlet	Immune_cells
SAT_2M	2159	1239	10.119595	5	Singlet	Immune_cells
SAT_2M	2347	1245	7.1701315	1	Singlet	Immune_cells
SAT_2M	1020	688	0.195122	4	Singlet	Immune_cells
SAT_2M	2110	1254	4.6588235	1	Singlet	Immune_cells
SAT_2M	1153	772	0.6039689	1	Singlet	Immune_cells
SAT_2M	2288	1338	0.04363	0	Singlet	Adipocyte
SAT_2M	1483	881	8.7306917	1	Singlet	Immune_cells
SAT_2M	1929	1140	2.1705426	1	Singlet	Immune_cells
SAT_2M	1446	883	1.2413793	1	Singlet	Immune_cells
SAT_2M	3229	1623	3.7916153	0	Singlet	Adipocyte
SAT_2M	1221	826	0.2452984	4	Singlet	Immune_cells
SAT_2M	1537	891	4.150454	1	Singlet	Immune_cells
SAT_2M	1513	976	5.0524934	1	Singlet	Immune_cells
SAT_2M	1026	742	0.7714561	7	Singlet	Endothelial_cells
SAT_2M	7129	3111	8.4441955	4	Singlet	Immune_cells
SAT_2M	1365	800	10.407569	1	Singlet	Immune_cells
SAT_2M	4837	2369	5.6553148	9	Singlet	Mesothelial
SAT_2M	1031	709	0.192123	4	Singlet	Immune_cells
SAT_2M	1923	1015	3.1104199	0	Singlet	Adipocyte
SAT_2M	1645	991	0.9691096	1	Singlet	Immune_cells
SAT_2M	3172	1711	0.0313578	11	Singlet	Smooth_muscle_cells
SAT_2M	1340	829	0.4454343	4	Singlet	Immune_cells
SAT_2M	2161	1131	1.8441678	0	Singlet	Adipocyte
SAT_2M	1372	846	6.1046512	1	Singlet	Immune_cells

SAT_2M	2417	1249	1.3630731	10 Singlet	Immune_cells
SAT_2M	1716	980	6.1592098	1 Singlet	Immune_cells
SAT_2M	1421	910	0.0701262	4 Singlet	Immune_cells
SAT_2M	1062	703	0.656045	0 Singlet	Adipocyte
SAT_2M	5460	2437	9.0859332	9 Singlet	Mesothelial
SAT_2M	1639	1080	2.3142509	4 Singlet	Immune_cells
SAT_2M	2443	1367	0.1631987	0 Singlet	Adipocyte
SAT_2M	1295	820	7.1865443	1 Singlet	Immune_cells
SAT_2M	1620	971	4.1794714	4 Singlet	Immune_cells
SAT_2M	1547	927	6.4951768	1 Singlet	Immune_cells
SAT_2M	2361	1366	0.3764115	0 Singlet	Adipocyte
SAT_2M	1345	786	5.8475204	4 Singlet	Immune_cells
SAT_2M	1470	931	5.5555556	1 Singlet	Immune_cells
SAT_2M	2048	1161	0.3900536	0 Singlet	Adipocyte
SAT_2M	6703	2478	1.8562519	10 Singlet	Immune_cells
SAT_2M	2915	1541	2.6712329	1 Singlet	Immune_cells
SAT_2M	1972	1229	1.3144591	1 Singlet	Immune_cells
SAT_2M	1530	905	1.0437052	8 Singlet	Mesothelial
SAT_2M	1282	776	4.034135	1 Singlet	Immune_cells
SAT_2M	4296	2186	11.275758	9 Singlet	Mesothelial
SAT_2M	1511	926	3.3663366	4 Singlet	Immune_cells
SAT_2M	1520	913	3.6769534	1 Singlet	Immune_cells
SAT_2M	1226	791	3.2599837	1 Singlet	Immune_cells
SAT_2M	3333	1660	0.1793722	3 Singlet	FAPs
SAT_2M	1477	874	0.8097166	3 Singlet	FAPs
SAT_2M	1913	1157	0.729927	1 Singlet	Immune_cells
SAT_2M	2324	1262	2.016302	0 Singlet	Adipocyte
SAT_2M	1001	665	6.0636183	4 Singlet	Immune_cells
SAT_2M	1613	879	12.677798	1 Singlet	Immune_cells
SAT_2M	7798	3408	4.0578597	9 Singlet	Mesothelial
SAT_2M	1383	855	0.7225434	1 Singlet	Immune_cells
SAT_2M	1098	693	7.5523203	1 Singlet	Immune_cells
SAT_2M	1036	672	2.7911453	1 Singlet	Immune_cells
SAT_2M	1693	1050	0.4112808	0 Singlet	Adipocyte
SAT_2M	2980	1545	9.5110516	4 Singlet	Immune_cells
SAT_2M	1349	896	0.1470588	8 Singlet	Mesothelial
SAT_2M	1484	818	9.9062918	9 Singlet	Mesothelial
SAT_2M	1630	1064	3.3027523	1 Singlet	Immune_cells
SAT_2M	1781	1078	4.2553191	4 Singlet	Immune_cells
SAT_2M	7128	2744	3.7415919	10 Singlet	Immune_cells
SAT_2M	1306	847	1.065449	0 Singlet	Adipocyte
SAT_2M	1817	1082	2.1953897	5 Singlet	Immune_cells
SAT_2M	1330	891	0.3748126	8 Singlet	Mesothelial
SAT_2M	2574	1317	12.48068	1 Singlet	Immune_cells
SAT_2M	1170	757	0.6762468	3 Singlet	FAPs
SAT_2M	1632	1008	1.5834348	1 Singlet	Immune_cells
SAT_2M	1659	969	6.3359235	0 Singlet	Adipocyte
SAT_2M	1330	758	11.002994	1 Singlet	Immune_cells
SAT_2M	3298	1765	0.5142166	0 Singlet	Adipocyte
SAT_2M	1845	1060	0.9179266	1 Singlet	Immune_cells
SAT_2M	1970	1162	0.8080808	4 Singlet	Immune_cells

SAT_2M	1085	740	0.3673095	1	Singlet	Immune_cells
SAT_2M	3299	1660	5.4347826	0	Singlet	Adipocyte
SAT_2M	2097	1268	8.0965909	5	Singlet	Immune_cells
SAT_2M	1174	782	1.0143702	4	Singlet	Immune_cells
SAT_2M	1264	821	1.8882769	4	Singlet	Immune_cells
SAT_2M	1682	899	2.7893175	3	Singlet	FAPs
SAT_2M	1175	789	3.3898305	1	Singlet	Immune_cells
SAT_2M	1185	746	1.9343987	0	Singlet	Adipocyte
SAT_2M	1699	1010	0.7025761	3	Singlet	FAPs
SAT_2M	1808	1122	8.3838941	4	Singlet	Immune_cells
SAT_2M	1130	751	3.7069726	4	Singlet	Immune_cells
SAT_2M	2835	1426	4.1240747	10	Singlet	Immune_cells
SAT_2M	1238	759	0.8032129	1	Singlet	Immune_cells
SAT_2M	1054	698	0.8466604	4	Singlet	Immune_cells
SAT_2M	2541	1501	4.8311076	8	Singlet	Mesothelial
SAT_2M	1632	926	3.4756098	1	Singlet	Immune_cells
SAT_2M	1035	709	0.6730769	7	Singlet	Endothelial_cells
SAT_2M	1486	918	2.41773	1	Singlet	Immune_cells
SAT_2M	3021	1625	0.4618938	0	Singlet	Adipocyte
SAT_2M	1423	924	0.2102313	1	Singlet	Immune_cells
SAT_2M	1393	862	8.5714286	4	Singlet	Immune_cells
SAT_2M	1247	817	1.1217949	4	Singlet	Immune_cells
SAT_2M	1080	799	0.7373272	4	Singlet	Immune_cells
SAT_2M	4371	2018	1.7832647	2	Singlet	FAPs
SAT_2M	1343	918	0.6686478	4	Singlet	Immune_cells
SAT_2M	2553	1333	0.8987886	5	Singlet	Immune_cells
SAT_2M	4931	1858	0.7696982	10	Singlet	Immune_cells
SAT_2M	1046	710	0.4743833	5	Singlet	Immune_cells
SAT_2M	1892	1138	6.4873418	5	Singlet	Immune_cells
SAT_2M	1226	787	6.8292683	1	Singlet	Immune_cells
SAT_2M	1903	1106	2.6743576	1	Singlet	Immune_cells
SAT_2M	3063	1585	1.33507	3	Singlet	FAPs
SAT_2M	2764	1517	0.2506266	0	Singlet	Adipocyte
SAT_2M	1236	764	4.4141252	4	Singlet	Immune_cells
SAT_2M	12096	4127	11.184428	4	Singlet	Immune_cells
SAT_2M	1736	1023	0.2302821	1	Singlet	Immune_cells
SAT_2M	1552	927	2.1208226	1	Singlet	Immune_cells
SAT_2M	2039	1273	0.9794319	1	Singlet	Immune_cells
SAT_2M	5360	2029	1.2849162	10	Singlet	Immune_cells
SAT_2M	2235	1251	0.2678571	0	Singlet	Adipocyte
SAT_2M	1014	706	0.2944063	4	Singlet	Immune_cells
SAT_2M	1308	833	3.5714286	5	Singlet	Immune_cells
SAT_2M	1590	996	3.9448967	1	Singlet	Immune_cells
SAT_2M	1521	928	6.5003283	1	Singlet	Immune_cells
SAT_2M	3351	1638	12.555787	9	Singlet	Mesothelial
SAT_2M	1407	893	1.0623229	1	Singlet	Immune_cells
SAT_2M	1114	729	1.1669659	5	Singlet	Immune_cells
SAT_2M	1452	938	0.2061856	5	Singlet	Immune_cells
SAT_2M	2203	1188	8.6386251	5	Singlet	Immune_cells
SAT_2M	1538	940	1.8134715	1	Singlet	Immune_cells
SAT_2M	1223	722	0.4878049	3	Singlet	FAPs

SAT_2M	1866	1114	0.1599147	0	Singlet	Adipocyte
SAT_2M	1319	778	2.4187453	3	Singlet	FAPs
SAT_2M	3060	1603	9.517601	5	Singlet	Immune_cells
SAT_2M	1883	1084	0.2649709	0	Singlet	Adipocyte
SAT_2M	1624	1041	0.0613121	8	Singlet	Mesothelial
SAT_2M	2203	1274	5.6535504	4	Singlet	Immune_cells
SAT_2M	1665	986	4.9013748	9	Singlet	Mesothelial
SAT_2M	1311	824	1.5921152	1	Singlet	Immune_cells
SAT_2M	1385	854	0.8639309	1	Singlet	Immune_cells
SAT_2M	1045	706	2.0972355	0	Singlet	Adipocyte
SAT_2M	2446	1456	0.2442997	11	Singlet	Smooth_muscle_cells
SAT_2M	1175	783	2.4576271	9	Singlet	Mesothelial
SAT_2M	2795	1478	1.460114	0	Singlet	Adipocyte
SAT_2M	2125	1275	4.4600939	5	Singlet	Immune_cells
SAT_2M	1318	810	3.5391566	1	Singlet	Immune_cells
SAT_2M	1353	846	1.6212233	1	Singlet	Immune_cells
SAT_2M	1485	910	7.7436582	1	Singlet	Immune_cells
SAT_2M	2217	1268	1.0341727	0	Singlet	Adipocyte
SAT_2M	2004	1163	0.7936508	0	Singlet	Adipocyte
SAT_2M	3451	1789	1.0124385	5	Singlet	Immune_cells
SAT_2M	2806	1496	4.1888534	2	Singlet	FAPs
SAT_2M	2216	1353	2.374552	0	Singlet	Adipocyte
SAT_2M	1263	870	0.3162055	8	Singlet	Mesothelial
SAT_2M	1154	767	1.2942192	1	Singlet	Immune_cells
SAT_2M	1945	1086	2.6046987	0	Singlet	Adipocyte
SAT_2M	1707	1058	0	7	Singlet	Endothelial_cells
SAT_2M	1467	1036	0.2713704	8	Singlet	Mesothelial
SAT_2M	1153	764	0.7805724	4	Singlet	Immune_cells
SAT_2M	2058	1241	5.2606178	1	Singlet	Immune_cells
SAT_2M	1644	960	13.680387	9	Singlet	Mesothelial
SAT_2M	1019	671	3.7717602	0	Singlet	Adipocyte
SAT_2M	1984	1205	0.2008032	0	Singlet	Adipocyte
SAT_2M	4776	2141	4.3933054	9	Singlet	Mesothelial
SAT_2M	2086	1210	0.5243089	0	Singlet	Adipocyte
SAT_2M	1931	1175	4.0721649	1	Singlet	Immune_cells
SAT_2M	2175	1210	2.4736601	0	Singlet	Adipocyte
SAT_2M	1062	729	0.1876173	11	Singlet	Smooth_muscle_cells
SAT_2M	1162	754	2.7467811	1	Singlet	Immune_cells
SAT_2M	1273	815	0.5451713	0	Singlet	Adipocyte
SAT_2M	3929	1928	8.7430309	4	Singlet	Immune_cells
SAT_2M	1552	962	5.984556	1	Singlet	Immune_cells
SAT_2M	1196	805	0.3333333	1	Singlet	Immune_cells
SAT_2M	5675	2691	4.1380525	9	Singlet	Mesothelial
SAT_2M	3329	1703	1.1954573	0	Singlet	Adipocyte
SAT_2M	4971	2456	2.1669342	11	Singlet	Smooth_muscle_cells
SAT_2M	3306	1754	2.109069	11	Singlet	Smooth_muscle_cells
SAT_2M	1261	820	0.2371542	1	Singlet	Immune_cells
SAT_2M	1370	835	10.618182	4	Singlet	Immune_cells
SAT_2M	2053	1108	0.3406326	8	Singlet	Mesothelial
SAT_2M	1783	1098	5.7713652	1	Singlet	Immune_cells
SAT_2M	1106	711	0.1796945	5	Singlet	Immune_cells

SAT_2M	1001	689	0.3929273	4	Singlet	Immune_cells
SAT_2M	3031	1603	0.461285	3	Singlet	FAPs
SAT_2M	2624	1405	0.6056018	0	Singlet	Adipocyte
SAT_2M	1401	945	2.1126761	1	Singlet	Immune_cells
SAT_2M	1367	838	0.5098325	0	Singlet	Adipocyte
SAT_2M	3474	1876	5.520414	8	Singlet	Mesothelial
SAT_2M	1240	764	2.7375201	4	Singlet	Immune_cells
SAT_2M	1468	878	0.2717391	1	Singlet	Immune_cells
SAT_2M	2244	1194	5.4270463	4	Singlet	Immune_cells
SAT_2M	1652	1010	1.1390887	0	Singlet	Adipocyte
SAT_2M	1223	780	7.4979625	1	Singlet	Immune_cells
SAT_2M	1332	881	0.2237136	4	Singlet	Immune_cells
SAT_2M	1913	1166	0.9880395	0	Singlet	Adipocyte
SAT_2M	1516	909	2.8852459	4	Singlet	Immune_cells
SAT_2M	2716	1576	0.7328692	11	Singlet	Smooth_muscle_cells
SAT_2M	2211	1249	0.2242152	6	Singlet	Neurons
SAT_2M	1987	1143	8.1256231	1	Singlet	Immune_cells
SAT_2M	1565	903	2.0382166	5	Singlet	Immune_cells
SAT_2M	2040	1219	1.2195122	0	Singlet	Adipocyte
SAT_2M	1341	822	4.0029652	1	Singlet	Immune_cells
SAT_2M	2379	1282	1.6270338	1	Singlet	Immune_cells
SAT_2M	3571	1463	2.1818182	10	Singlet	Immune_cells
SAT_2M	1659	1010	3.2432432	4	Singlet	Immune_cells
SAT_2M	1868	1085	0.3725386	0	Singlet	Adipocyte
SAT_2M	2062	1209	3.52657	1	Singlet	Immune_cells
SAT_2M	1219	746	1.1409943	0	Singlet	Adipocyte
SAT_2M	1484	870	2.020202	2	Singlet	FAPs
SAT_2M	1024	685	0.2901354	1	Singlet	Immune_cells
SAT_2M	1251	798	5.6573705	1	Singlet	Immune_cells
SAT_2M	2067	1191	0.574988	0	Singlet	Adipocyte
SAT_2M	1564	949	2.3536896	1	Singlet	Immune_cells
SAT_2M	2700	1409	0.2583979	0	Singlet	Adipocyte
SAT_2M	1384	897	1.294964	4	Singlet	Immune_cells
SAT_2M	7760	3265	3.2540193	9	Singlet	Mesothelial
SAT_2M	2262	1171	3.2541777	10	Singlet	Immune_cells
SAT_2M	2809	1585	0.7097232	11	Singlet	Smooth_muscle_cells
SAT_2M	1516	920	2.6281209	1	Singlet	Immune_cells
SAT_2M	2974	1628	0.7380074	3	Singlet	FAPs
SAT_2M	1558	993	1.342711	4	Singlet	Immune_cells
SAT_2M	1052	709	2.1800948	1	Singlet	Immune_cells
SAT_2M	1428	872	0.9769714	1	Singlet	Immune_cells
SAT_2M	1257	820	5.1221434	1	Singlet	Immune_cells
SAT_2M	1146	771	1.1304348	4	Singlet	Immune_cells
SAT_2M	2346	1233	5.6050955	1	Singlet	Immune_cells
SAT_2M	1126	772	0.5291005	4	Singlet	Immune_cells
SAT_2M	1420	877	9.0653549	8	Singlet	Mesothelial
SAT_2M	1392	878	5.8823529	4	Singlet	Immune_cells
SAT_2M	1638	1032	1.8248175	4	Singlet	Immune_cells
SAT_2M	1372	879	0.4354136	4	Singlet	Immune_cells
SAT_2M	3610	1590	3.8153166	10	Singlet	Immune_cells
SAT_2M	1218	711	7.704918	1	Singlet	Immune_cells

SAT_2M	1509	979	0.1986755	4	Singlet	Immune_cells
SAT_2M	3825	1876	0.1560874	0	Singlet	Adipocyte
SAT_2M	1467	925	6.3902107	11	Singlet	Smooth_muscle_cells
SAT_2M	1835	1125	1.4673913	4	Singlet	Immune_cells
SAT_2M	1884	1069	0.2631579	0	Singlet	Adipocyte
SAT_2M	1250	768	1.5139442	0	Singlet	Adipocyte
SAT_2M	1363	865	2.489019	1	Singlet	Immune_cells
SAT_2M	1154	759	0.4314064	0	Singlet	Adipocyte
SAT_2M	1426	838	9.623431	1	Singlet	Immune_cells
SAT_2M	1214	789	5.2631579	1	Singlet	Immune_cells
SAT_2M	2065	1172	0.3866602	0	Singlet	Adipocyte
SAT_2M	1011	687	0.3933137	4	Singlet	Immune_cells
SAT_2M	2375	1307	0.0419287	0	Singlet	Adipocyte
SAT_2M	1288	769	0.0775194	4	Singlet	Immune_cells
SAT_2M	2148	1238	1.7674419	4	Singlet	Immune_cells
SAT_2M	2454	1261	7.9075426	10	Singlet	Immune_cells
SAT_2M	1881	1159	5.076679	1	Singlet	Immune_cells
SAT_2M	1729	1012	2.9936672	5	Singlet	Immune_cells
SAT_2M	1487	900	1.8084394	0	Singlet	Adipocyte
SAT_2M	1235	757	4.7504026	1	Singlet	Immune_cells
SAT_2M	1736	1059	0.5163511	0	Singlet	Adipocyte
SAT_2M	1882	1135	5.2436441	4	Singlet	Immune_cells
SAT_2M	1487	898	2.1390374	1	Singlet	Immune_cells
SAT_2M	1550	964	0.2559181	4	Singlet	Immune_cells
SAT_2M	1274	762	0.0782473	4	Singlet	Immune_cells
SAT_2M	3538	1922	0.1408848	5	Singlet	Immune_cells
SAT_2M	1594	977	0.9968847	0	Singlet	Adipocyte
SAT_2M	1824	1093	0.6553796	4	Singlet	Immune_cells
SAT_2M	1538	908	0.1296176	0	Singlet	Adipocyte
SAT_2M	1057	734	0.656045	6	Singlet	Neurons
SAT_2M	1181	753	1.3422819	1	Singlet	Immune_cells
SAT_2M	1946	1065	2.6060296	10	Singlet	Immune_cells
SAT_2M	1055	713	0.1886792	4	Singlet	Immune_cells
SAT_2M	1683	1021	9.0425532	1	Singlet	Immune_cells
SAT_2M	2291	1238	7.8627281	1	Singlet	Immune_cells
SAT_2M	1878	960	13.716108	1	Singlet	Immune_cells
SAT_2M	1587	1049	0.6870706	4	Singlet	Immune_cells
SAT_2M	1425	892	0.1386963	4	Singlet	Immune_cells
SAT_2M	1707	1009	0.93622	0	Singlet	Adipocyte
SAT_2M	1516	894	5.4641211	0	Singlet	Adipocyte
SAT_2M	1628	909	7.2171254	0	Singlet	Adipocyte
SAT_2M	1035	616	3.7681159	0	Singlet	Adipocyte
SAT_2M	1224	776	4.5602606	1	Singlet	Immune_cells
SAT_2M	1535	954	2.8589994	1	Singlet	Immune_cells
SAT_2M	1610	886	0.9305211	9	Singlet	Mesothelial
SAT_2M	5857	2688	1.533481	9	Singlet	Mesothelial
SAT_2M	3343	1854	0.506103	9	Singlet	Mesothelial
SAT_2M	1207	777	0.0825083	1	Singlet	Immune_cells
SAT_2M	1028	729	2.1174206	5	Singlet	Immune_cells
SAT_2M	1368	784	2.189781	0	Singlet	Adipocyte
SAT_2M	1210	775	0.5733006	0	Singlet	Adipocyte

SAT_2M	1899	1158	0.2103049	11	Singlet	Smooth_muscle_cells
SAT_2M	2303	1282	0.5632582	0	Singlet	Adipocyte
SAT_2M	5475	1614	1.075857	10	Singlet	Immune_cells
SAT_2M	4075	1927	0.8576329	10	Singlet	Immune_cells
SAT_2M	3394	1774	0.2054593	0	Singlet	Adipocyte
SAT_2M	5328	2196	2.4340011	10	Singlet	Immune_cells
SAT_2M	3630	1817	0.2743484	3	Singlet	FAPs
SAT_2M	1931	1085	8.0061983	1	Singlet	Immune_cells
SAT_2M	1212	787	1.5599343	0	Singlet	Adipocyte
SAT_2M	1844	1089	1.8388318	1	Singlet	Immune_cells
SAT_2M	2123	1247	0.5164319	5	Singlet	Immune_cells
SAT_2M	1491	948	2.1361816	1	Singlet	Immune_cells
SAT_2M	2192	1293	4.7792444	1	Singlet	Immune_cells
SAT_2M	1431	890	5.4166667	1	Singlet	Immune_cells
SAT_2M	4577	2269	0.0654165	5	Singlet	Immune_cells
SAT_2M	1557	863	11.139565	7	Singlet	Endothelial_cells
SAT_2M	1216	846	2.8571429	5	Singlet	Immune_cells
SAT_2M	1095	779	1.5440509	11	Singlet	Smooth_muscle_cells
SAT_2M	2130	1247	3.0444965	4	Singlet	Immune_cells
SAT_2M	1370	822	0.3628447	0	Singlet	Adipocyte
SAT_2M	1238	748	0.7228916	3	Singlet	FAPs
SAT_2M	1695	1062	3.8732394	1	Singlet	Immune_cells
SAT_2M	1632	965	0.794621	3	Singlet	FAPs
SAT_2M	3592	1562	6.4812239	10	Singlet	Immune_cells
SAT_2M	2203	1287	1.1312217	0	Singlet	Adipocyte
SAT_2M	3015	1583	1.8874172	1	Singlet	Immune_cells
SAT_2M	2055	1216	0.4356244	0	Singlet	Adipocyte
SAT_2M	1504	894	12.060968	1	Singlet	Immune_cells
SAT_2M	1260	820	1.5810277	4	Singlet	Immune_cells
SAT_2M	1053	744	1.8570102	4	Singlet	Immune_cells
SAT_2M	2148	1316	0.1395349	2	Singlet	FAPs
SAT_2M	2234	1280	0.0446429	8	Singlet	Mesothelial
SAT_2M	1006	691	1.4851485	1	Singlet	Immune_cells
SAT_2M	1663	1039	0.0596303	0	Singlet	Adipocyte
SAT_2M	3855	2004	7.4696304	9	Singlet	Mesothelial
SAT_2M	1916	1189	4.2730589	1	Singlet	Immune_cells
SAT_2M	1212	783	4.7502048	1	Singlet	Immune_cells
SAT_2M	2171	1213	9.2916283	4	Singlet	Immune_cells
SAT_2M	1826	1074	0.1094092	5	Singlet	Immune_cells
SAT_2M	1523	941	6.2704115	1	Singlet	Immune_cells
SAT_2M	1795	1013	4.6008869	7	Singlet	Endothelial_cells
SAT_2M	1964	1254	4.6216353	5	Singlet	Immune_cells
SAT_2M	1637	1054	0.968523	3	Singlet	FAPs
SAT_2M	1223	667	0.7287449	6	Singlet	Neurons
SAT_2M	2210	1335	3.0234657	4	Singlet	Immune_cells
SAT_2M	2549	1494	1.3586957	6	Singlet	Neurons
SAT_2M	1459	918	0.5453306	1	Singlet	Immune_cells
SAT_2M	1925	1111	3.3678756	1	Singlet	Immune_cells
SAT_2M	2240	1382	1.8666667	5	Singlet	Immune_cells
SAT_2M	1124	739	0.8888889	5	Singlet	Immune_cells
SAT_2M	1280	839	1.4751553	1	Singlet	Immune_cells

SAT_2M	2011	1180	2.026693	3	Singlet	FAPs
SAT_2M	1777	1017	7.2869955	3	Singlet	FAPs
SAT_2M	2243	1235	0.6219458	0	Singlet	Adipocyte
SAT_2M	1231	792	1.1363636	1	Singlet	Immune_cells
SAT_2M	1581	970	3.2683847	11	Singlet	Smooth_muscle_cells
SAT_2M	1145	765	0.6097561	1	Singlet	Immune_cells
SAT_2M	1443	919	1.5214385	1	Singlet	Immune_cells
SAT_2M	1240	812	4.9719326	1	Singlet	Immune_cells
SAT_2M	1071	697	2.1435228	0	Singlet	Adipocyte
SAT_2M	1432	903	5.409154	1	Singlet	Immune_cells
SAT_2M	2022	1256	0.4926108	7	Singlet	Endothelial_cells
SAT_2M	1354	858	6.5585851	1	Singlet	Immune_cells
SAT_2M	1760	1081	0.1132503	4	Singlet	Immune_cells
SAT_2M	1801	1074	9.1463415	1	Singlet	Immune_cells
SAT_2M	1101	758	1.9004525	4	Singlet	Immune_cells
SAT_2M	1627	1009	6.6217045	1	Singlet	Immune_cells
SAT_2M	1161	782	3.3419023	1	Singlet	Immune_cells
SAT_2M	2283	1308	2.5696864	1	Singlet	Immune_cells
SAT_2M	1844	1086	0.1622499	0	Singlet	Adipocyte
SAT_2M	7017	2815	7.7338641	9	Singlet	Mesothelial
SAT_2M	1037	686	0.481232	1	Singlet	Immune_cells
SAT_2M	1199	745	7.3272273	1	Singlet	Immune_cells
SAT_2M	2589	1481	0.3457549	0	Singlet	Adipocyte
SAT_2M	1422	896	0.6271777	4	Singlet	Immune_cells
SAT_2M	1241	824	2.3961661	4	Singlet	Immune_cells
SAT_2M	1999	1206	0.8928571	0	Singlet	Adipocyte
SAT_2M	1208	789	0.8203445	4	Singlet	Immune_cells
SAT_2M	1494	917	1.4046823	0	Singlet	Adipocyte
SAT_2M	2184	1254	0.6843066	0	Singlet	Adipocyte
SAT_2M	1656	989	4.736211	1	Singlet	Immune_cells
SAT_2M	1231	808	0.0808407	4	Singlet	Immune_cells
SAT_2M	1083	742	1.0999083	4	Singlet	Immune_cells
SAT_2M	1536	1006	0.8414239	5	Singlet	Immune_cells
SAT_2M	1315	868	0.531511	2	Singlet	FAPs
SAT_2M	1375	853	0.1447178	1	Singlet	Immune_cells
SAT_2M	1273	807	1.4139827	1	Singlet	Immune_cells
SAT_2M	2433	1180	9.6959737	10	Singlet	Immune_cells
SAT_2M	1365	886	0.8029197	0	Singlet	Adipocyte
SAT_2M	1793	1041	0.6677796	0	Singlet	Adipocyte
SAT_2M	1590	966	0	1	Singlet	Immune_cells
SAT_2M	3129	1667	0.4141446	0	Singlet	Adipocyte
SAT_2M	4593	2265	12.538027	4	Singlet	Immune_cells
SAT_2M	1871	1178	0.3733333	4	Singlet	Immune_cells
SAT_2M	1174	813	0.3392706	4	Singlet	Immune_cells
SAT_2M	1086	703	1.1948529	0	Singlet	Adipocyte
SAT_2M	2442	1160	5.9232026	10	Singlet	Immune_cells
SAT_2M	1370	867	0.7976795	0	Singlet	Adipocyte
SAT_2M	1485	970	0.7343124	11	Singlet	Smooth_muscle_cells
SAT_2M	2551	1509	5.3104256	9	Singlet	Mesothelial
SAT_2M	2905	1595	0.0343761	8	Singlet	Mesothelial
SAT_2M	1585	1037	0.4380476	4	Singlet	Immune_cells

SAT_2M	1252	836	0.3167063	4	Singlet	Immune_cells
SAT_2M	1229	835	3.4677419	4	Singlet	Immune_cells
SAT_2M	1294	805	2.0061728	4	Singlet	Immune_cells
SAT_2M	2671	1594	2.0096762	9	Singlet	Mesothelial
SAT_2M	1820	1177	0.5455537	0	Singlet	Adipocyte
SAT_2M	4377	2223	4.3903549	4	Singlet	Immune_cells
SAT_2M	2096	1204	0.3320683	0	Singlet	Adipocyte
SAT_2M	1862	1177	0.5356186	4	Singlet	Immune_cells
SAT_2M	1382	908	3.030303	5	Singlet	Immune_cells
SAT_2M	1853	1133	0.3745318	0	Singlet	Adipocyte
SAT_2M	1588	934	0.2512563	1	Singlet	Immune_cells
SAT_2M	3253	1552	1.563458	10	Singlet	Immune_cells
SAT_2M	1392	875	3.9341917	3	Singlet	FAPs
SAT_2M	1378	830	6.6618392	1	Singlet	Immune_cells
SAT_2M	1230	799	0.8116883	9	Singlet	Mesothelial
SAT_2M	1217	786	1.7944535	1	Singlet	Immune_cells
SAT_2M	1607	1040	0.1856436	4	Singlet	Immune_cells
SAT_2M	1777	1087	1.1198208	0	Singlet	Adipocyte
SAT_2M	1082	739	8.5086917	3	Singlet	FAPs
SAT_2M	1015	636	0.6889764	0	Singlet	Adipocyte
SAT_2M	1914	1185	0.6767309	4	Singlet	Immune_cells
SAT_2M	1179	715	3.7288136	5	Singlet	Immune_cells
SAT_2M	1217	800	3.755102	9	Singlet	Mesothelial
SAT_2M	1341	851	6.9784707	1	Singlet	Immune_cells
SAT_2M	2729	1530	0.5845817	7	Singlet	Endothelial_cells
SAT_2M	2065	1247	0.4343629	0	Singlet	Adipocyte
SAT_2M	3371	1763	0.5325444	0	Singlet	Adipocyte
SAT_2M	2283	1250	5.0958188	1	Singlet	Immune_cells
SAT_2M	1871	1216	0.1600854	4	Singlet	Immune_cells
SAT_2M	1030	692	1.2536162	4	Singlet	Immune_cells
SAT_2M	1624	993	1.8326206	0	Singlet	Adipocyte
SAT_2M	1051	681	0.095057	4	Singlet	Immune_cells
SAT_2M	2057	1210	0.0485437	8	Singlet	Mesothelial
SAT_2M	4897	2267	0.650671	2	Singlet	FAPs
SAT_2M	1530	922	0.5204945	1	Singlet	Immune_cells
SAT_2M	1176	794	0.4222973	4	Singlet	Immune_cells
SAT_2M	1286	837	0.3866976	1	Singlet	Immune_cells
SAT_2M	3461	1741	0.3159104	0	Singlet	Adipocyte
SAT_2M	1459	953	4.9897471	1	Singlet	Immune_cells
SAT_2M	3460	1744	0.3741007	0	Singlet	Adipocyte
SAT_2M	6591	2904	7.927846	9	Singlet	Mesothelial
SAT_2M	1395	812	4.7686833	9	Singlet	Mesothelial
SAT_2M	3045	1583	0.3595946	8	Singlet	Mesothelial
SAT_2M	2530	1457	1.7619421	6	Singlet	Neurons
SAT_2M	2347	1379	0.2541296	0	Singlet	Adipocyte
SAT_2M	1252	746	1.9904459	0	Singlet	Adipocyte
SAT_2M	1700	972	7.9859072	1	Singlet	Immune_cells
SAT_2M	1077	734	3.7002775	4	Singlet	Immune_cells
SAT_2M	1227	764	10.008071	1	Singlet	Immune_cells
SAT_2M	1008	634	0.4945598	0	Singlet	Adipocyte
SAT_2M	1643	984	1.2091898	3	Singlet	FAPs

SAT_2M	1703	1016	0.877193	0	Singlet	Adipocyte
SAT_2M	2468	1331	2.9435484	9	Singlet	Mesothelial
SAT_2M	1155	755	0.1716738	1	Singlet	Immune_cells
SAT_2M	1441	915	0.8934708	4	Singlet	Immune_cells
SAT_2M	1113	719	0.8035714	1	Singlet	Immune_cells
SAT_2M	4572	1938	9.5935315	0	Singlet	Adipocyte
SAT_2M	3076	1651	0.064704	0	Singlet	Adipocyte
SAT_2M	1321	830	5.7185854	1	Singlet	Immune_cells
SAT_2M	2283	1297	4.317488	1	Singlet	Immune_cells
SAT_2M	1795	1058	4.8199446	1	Singlet	Immune_cells
SAT_2M	11173	3842	8.7042002	9	Singlet	Mesothelial
SAT_2M	1419	820	9.4736842	1	Singlet	Immune_cells
SAT_2M	5604	2100	1.0509441	10	Singlet	Immune_cells
SAT_2M	1468	890	4.2090971	1	Singlet	Immune_cells
SAT_2M	1216	717	13.078798	1	Singlet	Immune_cells
SAT_2M	1630	1026	0.18226	4	Singlet	Immune_cells
SAT_2M	1347	842	1.8518519	1	Singlet	Immune_cells
SAT_2M	2357	1361	5.0042409	9	Singlet	Mesothelial
SAT_2M	1265	795	0.3921569	1	Singlet	Immune_cells
SAT_2M	2297	1368	0.1300954	5	Singlet	Immune_cells
SAT_2M	1310	854	2.7987897	1	Singlet	Immune_cells
SAT_2M	1150	770	0.8561644	0	Singlet	Adipocyte
SAT_2M	1445	937	4.4704264	4	Singlet	Immune_cells
SAT_2M	1914	1108	5.0962038	8	Singlet	Mesothelial
SAT_2M	1920	1159	3.9937759	1	Singlet	Immune_cells
SAT_2M	1277	846	1.399689	4	Singlet	Immune_cells
SAT_2M	1037	690	2.7911453	1	Singlet	Immune_cells
SAT_2M	1124	720	0.0888099	4	Singlet	Immune_cells
SAT_2M	2178	1404	0.2284148	4	Singlet	Immune_cells
SAT_2M	1289	946	0.7621951	6	Singlet	Neurons
SAT_2M	2561	1353	0.4674718	0	Singlet	Adipocyte
SAT_2M	1275	786	4.4600939	1	Singlet	Immune_cells
SAT_2M	1090	742	0.0909091	2	Singlet	FAPs
SAT_2M	2872	1725	5.5536272	4	Singlet	Immune_cells
SAT_2M	1860	1097	6.387547	1	Singlet	Immune_cells
SAT_2M	2247	1176	3.7316748	10	Singlet	Immune_cells
SAT_2M	1477	917	0.4026846	0	Singlet	Adipocyte
SAT_2M	1471	954	4.0650407	4	Singlet	Immune_cells
SAT_2M	3899	1944	0.6398771	0	Singlet	Adipocyte
SAT_2M	1242	763	6.5060241	1	Singlet	Immune_cells
SAT_2M	1679	1041	0.9478673	1	Singlet	Immune_cells
SAT_2M	2084	1137	9.8659004	1	Singlet	Immune_cells
SAT_2M	1059	709	0.6610009	4	Singlet	Immune_cells
SAT_2M	1618	1033	0.617284	1	Singlet	Immune_cells
SAT_2M	1241	815	0.0805802	4	Singlet	Immune_cells
SAT_2M	3308	1586	1.5379976	10	Singlet	Immune_cells
SAT_2M	1246	809	7.1770335	11	Singlet	Smooth_muscle_cells
SAT_2M	1235	775	1.5310234	0	Singlet	Adipocyte
SAT_2M	1697	969	5.8201058	1	Singlet	Immune_cells
SAT_2M	1440	940	0.2773925	4	Singlet	Immune_cells
SAT_2M	2511	1318	0.0793966	8	Singlet	Mesothelial

SAT_2M	1301	851	0.228833	3	Singlet	FAPs
SAT_2M	1506	976	0.6596306	1	Singlet	Immune_cells
SAT_2M	1498	902	5.1162791	3	Singlet	FAPs
SAT_2M	1086	684	2.9384757	1	Singlet	Immune_cells
SAT_2M	1927	1158	3.5105834	1	Singlet	Immune_cells
SAT_2M	1789	1224	0.8328706	4	Singlet	Immune_cells
SAT_2M	1807	1142	4.5755237	1	Singlet	Immune_cells
SAT_2M	1816	1111	0.6037322	0	Singlet	Adipocyte
SAT_2M	1806	1183	2.092511	4	Singlet	Immune_cells
SAT_2M	1380	837	4.6209386	1	Singlet	Immune_cells
SAT_2M	2435	1405	0.5716619	0	Singlet	Adipocyte
SAT_2M	4303	2014	2.2495362	0	Singlet	Adipocyte
SAT_2M	1488	906	9.0545939	1	Singlet	Immune_cells
SAT_2M	1472	912	0.807537	0	Singlet	Adipocyte
SAT_2M	1672	1011	0.5360334	0	Singlet	Adipocyte
SAT_2M	1229	768	0	4	Singlet	Immune_cells
SAT_2M	2049	1198	1.3113162	0	Singlet	Adipocyte
SAT_2M	1006	669	0.5934718	1	Singlet	Immune_cells
SAT_2M	1409	940	0.3523608	8	Singlet	Mesothelial
SAT_2M	1310	829	6.231003	1	Singlet	Immune_cells
SAT_2M	1477	969	3.3085753	1	Singlet	Immune_cells
SAT_2M	1233	929	0.7003891	6	Singlet	Neurons
SAT_2M	1300	771	5.5981595	1	Singlet	Immune_cells
SAT_2M	1514	938	12.352168	5	Singlet	Immune_cells
SAT_2M	1297	819	2.8439662	1	Singlet	Immune_cells
SAT_2M	1238	811	0.882825	1	Singlet	Immune_cells
SAT_2M	1897	1168	1.1560694	0	Singlet	Adipocyte
SAT_2M	1136	799	0.1751313	2	Singlet	FAPs
SAT_2M	1042	710	0.8563273	4	Singlet	Immune_cells
SAT_2M	1552	1013	0.6422608	8	Singlet	Mesothelial
SAT_2M	1698	963	10.076157	1	Singlet	Immune_cells
SAT_2M	1566	844	6.7049808	0	Singlet	Adipocyte
SAT_2M	1236	799	3.0645161	1	Singlet	Immune_cells
SAT_2M	1973	1163	0.353714	8	Singlet	Mesothelial
SAT_2M	2005	1215	0.3964321	4	Singlet	Immune_cells
SAT_2M	1493	954	0.9352037	1	Singlet	Immune_cells
SAT_2M	1424	907	0.3501401	1	Singlet	Immune_cells
SAT_2M	1321	798	7.3906486	1	Singlet	Immune_cells
SAT_2M	2030	1229	0.246063	5	Singlet	Immune_cells
SAT_2M	1418	863	1.5460295	1	Singlet	Immune_cells
SAT_2M	1755	1096	6.5834279	1	Singlet	Immune_cells
SAT_2M	2123	1311	0.6539	5	Singlet	Immune_cells
SAT_2M	2547	1405	6.0297572	1	Singlet	Immune_cells
SAT_2M	1292	804	3.0139104	1	Singlet	Immune_cells
SAT_2M	1505	956	7.8444298	1	Singlet	Immune_cells
SAT_2M	2583	1418	3.3500193	0	Singlet	Adipocyte
SAT_2M	1345	843	0.8862629	1	Singlet	Immune_cells
SAT_2M	1075	721	0.1851852	1	Singlet	Immune_cells
SAT_2M	1337	858	1.1160714	4	Singlet	Immune_cells
SAT_2M	3971	1964	0.4003002	0	Singlet	Adipocyte
SAT_2M	1328	787	1.9461078	5	Singlet	Immune_cells

SAT_2M	1554	977	1.5404365	3	Singlet	FAPs
SAT_2M	2587	1415	0.2312139	0	Singlet	Adipocyte
SAT_2M	1905	1156	0.4175365	1	Singlet	Immune_cells
SAT_2M	1311	883	0.7587253	4	Singlet	Immune_cells
SAT_2M	1389	932	4.7959914	1	Singlet	Immune_cells
SAT_2M	1418	900	0.7703081	1	Singlet	Immune_cells
SAT_2M	1125	797	0.4428698	4	Singlet	Immune_cells
SAT_2M	1354	831	9.0707965	1	Singlet	Immune_cells
SAT_2M	2186	1323	2.5932666	4	Singlet	Immune_cells
SAT_2M	1363	795	11.670314	1	Singlet	Immune_cells
SAT_2M	1083	707	0.5529954	4	Singlet	Immune_cells
SAT_2M	2451	1374	1.9934906	10	Singlet	Immune_cells
SAT_2M	1110	745	0.4468275	0	Singlet	Adipocyte
SAT_2M	2219	1240	0.1345895	0	Singlet	Adipocyte
SAT_2M	2547	1392	0.1174168	0	Singlet	Adipocyte
SAT_2M	1082	715	0.3676471	1	Singlet	Immune_cells
SAT_2M	1437	934	0.3467406	0	Singlet	Adipocyte
SAT_2M	3167	1688	3.7118591	8	Singlet	Mesothelial
SAT_2M	1873	924	6.8617021	10	Singlet	Immune_cells
SAT_2M	1016	683	3.4246575	1	Singlet	Immune_cells
SAT_2M	1901	1218	0.4195071	11	Singlet	Smooth_muscle_cells
SAT_2M	1672	1035	0.4169148	4	Singlet	Immune_cells
SAT_2M	1678	992	2.1403092	1	Singlet	Immune_cells
SAT_2M	2247	1238	0.4	0	Singlet	Adipocyte
SAT_2M	1180	813	0.4219409	4	Singlet	Immune_cells
SAT_2M	1365	776	2.5491624	10	Singlet	Immune_cells
SAT_2M	1792	1172	3.0353201	4	Singlet	Immune_cells
SAT_2M	1625	935	0.122399	2	Singlet	FAPs
SAT_2M	1232	857	0.4842615	8	Singlet	Mesothelial
SAT_2M	1644	1000	6.2085594	1	Singlet	Immune_cells
SAT_2M	1370	931	0.6535948	8	Singlet	Mesothelial
SAT_2M	1290	839	0.2307692	8	Singlet	Mesothelial
SAT_2M	1537	1014	0.130039	4	Singlet	Immune_cells
SAT_2M	1227	815	2.6785714	1	Singlet	Immune_cells
SAT_2M	1213	751	1.2274959	0	Singlet	Adipocyte
SAT_2M	2321	1460	0.0428266	8	Singlet	Mesothelial
SAT_2M	1644	1038	2.9714979	4	Singlet	Immune_cells
SAT_2M	1720	1017	0.4624277	3	Singlet	FAPs
SAT_2M	4302	2001	0.7649513	10	Singlet	Immune_cells
SAT_2M	1216	765	0.5700326	1	Singlet	Immune_cells
SAT_2M	2063	1187	3.5800677	1	Singlet	Immune_cells
SAT_2M	1298	823	0.2304147	0	Singlet	Adipocyte
SAT_2M	1271	812	0.7014809	1	Singlet	Immune_cells
SAT_2M	2240	1226	8.7789661	1	Singlet	Immune_cells
SAT_2M	1041	630	1.5355086	0	Singlet	Adipocyte
SAT_2M	3568	1834	1.2273361	2	Singlet	FAPs
SAT_2M	2861	1619	4.2582897	9	Singlet	Mesothelial
SAT_2M	1054	689	0.1895735	1	Singlet	Immune_cells
SAT_2M	1923	1189	0.2575992	11	Singlet	Smooth_muscle_cells
SAT_2M	1550	953	4.8293625	1	Singlet	Immune_cells
SAT_2M	1076	674	6.2037037	0	Singlet	Adipocyte

SAT_2M	2365	1391	3.7489469	8	Singlet	Mesothelial
SAT_2M	1821	1082	0.5452563	3	Singlet	FAPs
SAT_2M	1165	772	0.2570694	1	Singlet	Immune_cells
SAT_2M	1036	699	1.4450867	4	Singlet	Immune_cells
SAT_2M	4678	2092	2.5630073	10	Singlet	Immune_cells
SAT_2M	1738	1076	5.8486239	1	Singlet	Immune_cells
SAT_2M	1676	950	2.1454112	1	Singlet	Immune_cells
SAT_2M	1145	745	0.8680556	4	Singlet	Immune_cells
SAT_2M	1466	922	0.4078858	5	Singlet	Immune_cells
SAT_2M	2482	1310	0.2009646	0	Singlet	Adipocyte
SAT_2M	1255	765	1.9032514	0	Singlet	Adipocyte
SAT_2M	1096	757	2.1798365	1	Singlet	Immune_cells
SAT_2M	1201	808	0.4149378	7	Singlet	Endothelial_cells
SAT_2M	1946	1230	0.4601227	4	Singlet	Immune_cells
SAT_2M	2702	1395	9.0439276	9	Singlet	Mesothelial
SAT_2M	2080	1236	1.9645424	4	Singlet	Immune_cells
SAT_2M	1010	618	4.0275049	7	Singlet	Endothelial_cells
SAT_2M	1804	1103	0.2759382	0	Singlet	Adipocyte
SAT_2M	1955	1206	1.1178862	0	Singlet	Adipocyte
SAT_2M	2586	1458	5.0788765	2	Singlet	FAPs
SAT_2M	2908	1508	0.4796163	0	Singlet	Adipocyte
SAT_2M	1036	665	0.8628955	0	Singlet	Adipocyte
SAT_2M	4274	2318	8.9911256	9	Singlet	Mesothelial
SAT_2M	1004	666	0.1980198	4	Singlet	Immune_cells
SAT_2M	1311	848	0.3023432	1	Singlet	Immune_cells
SAT_2M	2212	1276	3.1981982	0	Singlet	Adipocyte
SAT_2M	1750	947	10	9	Singlet	Mesothelial
SAT_2M	1489	932	4.736491	1	Singlet	Immune_cells
SAT_2M	1814	1106	0.8237232	0	Singlet	Adipocyte
SAT_2M	1329	887	0.4477612	1	Singlet	Immune_cells
SAT_2M	2605	1349	0.4980843	0	Singlet	Adipocyte
SAT_2M	1449	886	3.2391454	1	Singlet	Immune_cells
SAT_2M	2168	1329	0.5064457	11	Singlet	Smooth_muscle_cells
SAT_2M	1547	1001	2.0539153	4	Singlet	Immune_cells
SAT_2M	1188	819	7.3701843	4	Singlet	Immune_cells
SAT_2M	1392	882	0.5017921	0	Singlet	Adipocyte
SAT_2M	1541	931	5.7679844	1	Singlet	Immune_cells
SAT_2M	1146	771	0.3478261	1	Singlet	Immune_cells
SAT_2M	7101	2879	1.6451069	9	Singlet	Mesothelial
SAT_2M	1724	1079	3.1070196	1	Singlet	Immune_cells
SAT_2M	3254	1536	3.1183124	3	Singlet	FAPs
SAT_2M	1883	1179	0.1584786	0	Singlet	Adipocyte
SAT_2M	2013	1171	1.8802573	0	Singlet	Adipocyte
SAT_2M	1597	976	1.7478152	1	Singlet	Immune_cells
SAT_2M	1826	1097	0.436205	8	Singlet	Mesothelial
SAT_2M	1257	808	5.621536	9	Singlet	Mesothelial
SAT_2M	1584	914	5.7898049	0	Singlet	Adipocyte
SAT_2M	1190	745	9.0606816	1	Singlet	Immune_cells
SAT_2M	1907	1174	4.3251694	1	Singlet	Immune_cells
SAT_2M	1500	976	0.5984043	0	Singlet	Adipocyte
SAT_2M	1244	854	2.3961661	0	Singlet	Adipocyte

SAT_2M	5219	2160	2.8702641	10	Singlet	Immune_cells
SAT_2M	1935	1052	3.9134912	0	Singlet	Adipocyte
SAT_2M	1232	776	4.6116505	1	Singlet	Immune_cells
SAT_2M	1125	733	1.4171833	0	Singlet	Adipocyte
SAT_2M	1270	835	9.8668755	5	Singlet	Immune_cells
SAT_2M	1055	709	0.6585136	1	Singlet	Immune_cells
SAT_2M	2433	1342	2.49693	9	Singlet	Mesothelial
SAT_2M	1353	821	3.3923304	1	Singlet	Immune_cells
SAT_2M	1533	982	0.4545455	7	Singlet	Endothelial_cells
SAT_2M	1366	872	3.7790698	1	Singlet	Immune_cells
SAT_2M	1950	1185	0.3061224	4	Singlet	Immune_cells
SAT_2M	1098	774	0.9963768	0	Singlet	Adipocyte
SAT_2M	2118	1312	0.3762935	4	Singlet	Immune_cells
SAT_2M	1075	717	0.0925926	4	Singlet	Immune_cells
SAT_2M	1645	1027	3.688029	1	Singlet	Immune_cells
SAT_2M	2188	1315	2.3266423	1	Singlet	Immune_cells
SAT_2M	2047	1132	4.0389294	1	Singlet	Immune_cells
SAT_2M	1196	784	1.8318068	1	Singlet	Immune_cells
SAT_2M	2164	1216	0.2762431	0	Singlet	Adipocyte
SAT_2M	5006	2373	8.4130782	9	Singlet	Mesothelial
SAT_2M	1171	789	0.5937235	4	Singlet	Immune_cells
SAT_2M	1343	875	1.3323464	1	Singlet	Immune_cells
SAT_2M	3529	1884	0.9047215	3	Singlet	FAPs
SAT_2M	1162	794	5.097706	1	Singlet	Immune_cells
SAT_2M	2246	1257	0.2220249	3	Singlet	FAPs
SAT_2M	1092	767	0.3629764	1	Singlet	Immune_cells
SAT_2M	2444	1363	0.5291005	0	Singlet	Adipocyte
SAT_2M	1633	980	1.8270402	0	Singlet	Adipocyte
SAT_2M	1464	969	1.2951602	3	Singlet	FAPs
SAT_2M	1258	837	3.083004	1	Singlet	Immune_cells
SAT_2M	2715	1493	12.298091	1	Singlet	Immune_cells
SAT_2M	2031	1222	5.3071253	4	Singlet	Immune_cells
SAT_2M	1869	1093	1.2253596	0	Singlet	Adipocyte
SAT_2M	2421	1412	0.3292181	8	Singlet	Mesothelial
SAT_2M	1992	1240	1.6024036	1	Singlet	Immune_cells
SAT_2M	1038	748	0.094162	6	Singlet	Neurons
SAT_2M	1354	874	4.797048	8	Singlet	Mesothelial
SAT_2M	1262	801	0.4724409	0	Singlet	Adipocyte
SAT_2M	1421	885	1.396648	0	Singlet	Adipocyte
SAT_2M	1429	885	0.069735	0	Singlet	Adipocyte
SAT_2M	1367	913	2.5566107	1	Singlet	Immune_cells
SAT_2M	1071	693	0.8379888	1	Singlet	Immune_cells
SAT_2M	1992	1105	0.2497503	0	Singlet	Adipocyte
SAT_2M	1450	869	1.3764625	1	Singlet	Immune_cells
SAT_2M	4835	2186	0.267986	2	Singlet	FAPs
SAT_2M	1353	856	2.8085735	1	Singlet	Immune_cells
SAT_2M	1758	1064	6.1398522	1	Singlet	Immune_cells
SAT_2M	1692	1004	0.8833922	0	Singlet	Adipocyte
SAT_2M	1064	676	2.9990628	3	Singlet	FAPs
SAT_2M	2050	1048	1.9970774	5	Singlet	Immune_cells
SAT_2M	1687	1028	0.7083825	0	Singlet	Adipocyte

SAT_2M	1964	1081	5.2631579	10	Singlet	Immune_cells
SAT_2M	1142	777	4.0905135	5	Singlet	Immune_cells
SAT_2M	1319	809	12.773998	7	Singlet	Endothelial_cells
SAT_2M	1255	895	3.1570639	4	Singlet	Immune_cells
SAT_2M	2097	1254	1.52019	4	Singlet	Immune_cells
SAT_2M	4246	1813	1.6	10	Singlet	Immune_cells
SAT_2M	1141	784	4.1083916	11	Singlet	Smooth_muscle_cells
SAT_2M	1261	832	0.9478673	1	Singlet	Immune_cells
SAT_2M	1105	763	0.5415162	1	Singlet	Immune_cells
SAT_2M	2101	1322	5.7156353	1	Singlet	Immune_cells
SAT_2M	1370	849	6.095791	1	Singlet	Immune_cells
SAT_2M	1023	688	4.2759961	1	Singlet	Immune_cells
SAT_2M	1118	759	0.5347594	1	Singlet	Immune_cells
SAT_2M	1821	1094	4.8061169	1	Singlet	Immune_cells
SAT_2M	1071	758	3.7104072	6	Singlet	Neurons
SAT_2M	1068	670	0.4681648	0	Singlet	Adipocyte
SAT_2M	1306	849	1.6030534	1	Singlet	Immune_cells
SAT_2M	1292	812	0.4640371	4	Singlet	Immune_cells
SAT_2M	1331	852	0.5223881	0	Singlet	Adipocyte
SAT_2M	1365	914	0.2177068	3	Singlet	FAPs
SAT_2M	1525	988	0.1959504	1	Singlet	Immune_cells
SAT_2M	1992	1211	0.0501253	4	Singlet	Immune_cells
SAT_2M	4116	1715	2.3188406	10	Singlet	Immune_cells
SAT_2M	1152	765	7.5862069	1	Singlet	Immune_cells
SAT_2M	1468	950	2.3696682	1	Singlet	Immune_cells
SAT_2M	1271	824	0.6284368	1	Singlet	Immune_cells
SAT_2M	1498	941	8.8489687	4	Singlet	Immune_cells
SAT_2M	1468	986	2.300406	4	Singlet	Immune_cells
SAT_2M	1368	944	1.0122921	5	Singlet	Immune_cells
SAT_2M	1565	922	5.393401	1	Singlet	Immune_cells
SAT_2M	3627	1841	2.0363236	0	Singlet	Adipocyte
SAT_2M	1257	796	4.6862589	4	Singlet	Immune_cells
SAT_2M	2823	1507	0.4232804	2	Singlet	FAPs
SAT_2M	1166	764	4.2662116	1	Singlet	Immune_cells
SAT_2M	1923	1163	0.7261411	1	Singlet	Immune_cells
SAT_2M	1582	986	0.2523659	1	Singlet	Immune_cells
SAT_2M	1019	594	0.9765625	10	Singlet	Immune_cells
SAT_2M	6379	3039	10.338346	9	Singlet	Mesothelial
SAT_2M	1417	875	0.4926108	1	Singlet	Immune_cells
SAT_2M	2734	1423	2.374863	0	Singlet	Adipocyte
SAT_2M	1644	1084	3.3918837	4	Singlet	Immune_cells
SAT_2M	2887	1536	1.8301105	10	Singlet	Immune_cells
SAT_2M	1346	881	4.818384	4	Singlet	Immune_cells
SAT_2M	1985	1219	1.0531595	5	Singlet	Immune_cells
SAT_2M	1490	973	0.4	2	Singlet	FAPs
SAT_2M	1297	921	0.3837299	11	Singlet	Smooth_muscle_cells
SAT_2M	2761	1407	5.4972875	5	Singlet	Immune_cells
SAT_2M	1260	843	0.555115	1	Singlet	Immune_cells
SAT_2M	2903	1691	0.4459691	2	Singlet	FAPs
SAT_2M	1271	857	1.8010963	1	Singlet	Immune_cells
SAT_2M	2100	1362	0.1886792	5	Singlet	Immune_cells

SAT_2M	1360	860	1.0241405	1	Singlet	Immune_cells
SAT_2M	1310	817	5.6990881	1	Singlet	Immune_cells
SAT_2M	1069	712	0.9337068	0	Singlet	Adipocyte
SAT_2M	3099	1610	0.4501608	8	Singlet	Mesothelial
SAT_2M	1064	731	1.8691589	1	Singlet	Immune_cells
SAT_2M	1348	807	10.265879	1	Singlet	Immune_cells
SAT_2M	2083	1225	0.2868069	0	Singlet	Adipocyte
SAT_2M	1442	951	0.621118	4	Singlet	Immune_cells
SAT_2M	2169	1241	7.1886447	5	Singlet	Immune_cells
SAT_2M	1193	756	3.153527	9	Singlet	Mesothelial
SAT_2M	1416	897	0.5621926	1	Singlet	Immune_cells
SAT_2M	1570	1002	0.3162555	3	Singlet	FAPs
SAT_2M	1257	852	1.340694	11	Singlet	Smooth_muscle_cells
SAT_2M	1059	675	0.7476636	1	Singlet	Immune_cells
SAT_2M	1429	952	0.6289308	3	Singlet	FAPs
SAT_2M	1280	810	2.8838659	1	Singlet	Immune_cells
SAT_2M	2576	1484	2.473908	5	Singlet	Immune_cells
SAT_2M	2394	1333	2.9142381	0	Singlet	Adipocyte
SAT_2M	1903	1115	0.5238345	0	Singlet	Adipocyte
SAT_2M	1346	870	3.1875463	1	Singlet	Immune_cells
SAT_2M	1196	740	2.3313905	5	Singlet	Immune_cells
SAT_2M	1294	803	4.6332046	1	Singlet	Immune_cells
SAT_2M	1309	899	3.5794183	1	Singlet	Immune_cells
SAT_2M	1097	711	0.2717391	8	Singlet	Mesothelial
SAT_2M	2693	1426	3.2198372	0	Singlet	Adipocyte
SAT_2M	3447	1872	8.6491177	4	Singlet	Immune_cells
SAT_2M	1404	832	2.9871977	1	Singlet	Immune_cells
SAT_2M	1253	846	4.5346062	1	Singlet	Immune_cells
SAT_2M	1824	1228	0.2726281	4	Singlet	Immune_cells
SAT_2M	1176	772	0	5	Singlet	Immune_cells
SAT_2M	3753	1934	3.826734	8	Singlet	Mesothelial
SAT_2M	1620	974	0.4907975	0	Singlet	Adipocyte
SAT_2M	1060	712	0.3766478	4	Singlet	Immune_cells
SAT_2M	3754	1787	3.4775684	8	Singlet	Mesothelial
SAT_2M	1602	1072	1.3027295	11	Singlet	Smooth_muscle_cells
SAT_2M	1084	709	3.6900369	7	Singlet	Endothelial_cells
SAT_2M	4217	2127	3.6363636	9	Singlet	Mesothelial
SAT_2M	1672	979	5.6052475	1	Singlet	Immune_cells
SAT_2M	1656	1060	3.5371703	4	Singlet	Immune_cells
SAT_2M	1204	810	0.4950495	1	Singlet	Immune_cells
SAT_2M	3478	1784	0.2583238	8	Singlet	Mesothelial
SAT_2M	1827	1079	4.5602606	0	Singlet	Adipocyte
SAT_2M	1079	709	0.7373272	4	Singlet	Immune_cells
SAT_2M	1377	862	0.7896626	0	Singlet	Adipocyte
SAT_2M	1415	833	7.2382291	1	Singlet	Immune_cells
SAT_2M	1305	839	0.0763359	5	Singlet	Immune_cells
SAT_2M	1503	920	1.259947	3	Singlet	FAPs
SAT_2M	1529	972	1.8892508	3	Singlet	FAPs
SAT_2M	1240	843	1.2779553	4	Singlet	Immune_cells
SAT_2M	1238	789	1.369863	1	Singlet	Immune_cells
SAT_2M	1380	837	9.221902	1	Singlet	Immune_cells

SAT_2M	1832	1031	1.7905589	10	Singlet	Immune_cells
SAT_2M	1014	672	0.1968504	3	Singlet	FAPs
SAT_2M	1690	1123	0.4129794	11	Singlet	Smooth_muscle_cells
SAT_2M	1360	832	4.9671293	1	Singlet	Immune_cells
SAT_2M	1247	810	2.0766773	1	Singlet	Immune_cells
SAT_2M	1022	678	2.3460411	1	Singlet	Immune_cells
SAT_2M	1940	1062	2.7277406	3	Singlet	FAPs
SAT_2M	1386	834	8.3870968	1	Singlet	Immune_cells
SAT_2M	1289	814	1.843318	1	Singlet	Immune_cells
SAT_2M	1323	828	6.511976	1	Singlet	Immune_cells
SAT_2M	1949	1210	0.3070624	5	Singlet	Immune_cells
SAT_2M	1397	932	0.1428571	3	Singlet	FAPs
SAT_2M	1289	863	4.5107034	4	Singlet	Immune_cells
SAT_2M	1427	929	2.5139665	1	Singlet	Immune_cells
SAT_2M	2047	1148	12.237933	4	Singlet	Immune_cells
SAT_2M	1893	1081	3.0095037	1	Singlet	Immune_cells
SAT_2M	1231	827	1.6155089	7	Singlet	Endothelial_cells
SAT_2M	1176	722	6.9373942	1	Singlet	Immune_cells
SAT_2M	1281	896	0.6196747	4	Singlet	Immune_cells
SAT_2M	1169	797	3.4100597	1	Singlet	Immune_cells
SAT_2M	3814	1863	0.1306506	0	Singlet	Adipocyte
SAT_2M	3126	1682	5.1036683	1	Singlet	Immune_cells
SAT_2M	3755	1512	5.7233704	10	Singlet	Immune_cells
SAT_2M	1387	831	0.6479482	4	Singlet	Immune_cells
SAT_2M	1450	975	1.0295127	9	Singlet	Mesothelial
SAT_2M	1599	1031	0.436409	5	Singlet	Immune_cells
SAT_2M	1225	769	0.648824	2	Singlet	FAPs
SAT_2M	1793	1082	0.4444444	4	Singlet	Immune_cells
SAT_2M	2022	1227	1.2357884	1	Singlet	Immune_cells
SAT_2M	3818	1907	0.1822917	8	Singlet	Mesothelial
SAT_2M	1049	615	6.6476733	5	Singlet	Immune_cells
SAT_2M	1171	777	0.0848896	1	Singlet	Immune_cells
SAT_2M	1253	773	5.1751592	1	Singlet	Immune_cells
SAT_2M	3354	1727	1.960202	0	Singlet	Adipocyte
SAT_2M	1699	1023	0.4692082	4	Singlet	Immune_cells
SAT_2M	1329	921	0.1497006	4	Singlet	Immune_cells
SAT_2M	1793	1060	1.1660189	0	Singlet	Adipocyte
SAT_2M	4367	1963	0.4105839	0	Singlet	Adipocyte
SAT_2M	1052	684	4.3273754	4	Singlet	Immune_cells
SAT_2M	1266	780	2.2798742	5	Singlet	Immune_cells
SAT_2M	1571	931	9.0793651	1	Singlet	Immune_cells
SAT_2M	1266	768	6.8396226	1	Singlet	Immune_cells
SAT_2M	1740	1090	2.0011435	0	Singlet	Adipocyte
SAT_2M	1716	944	6.0500291	5	Singlet	Immune_cells
SAT_2M	1291	805	6.3320463	1	Singlet	Immune_cells
SAT_2M	3187	1645	0.3436426	0	Singlet	Adipocyte
SAT_2M	1507	862	2.3178808	4	Singlet	Immune_cells
SAT_2M	2473	1354	0.0403388	0	Singlet	Adipocyte
SAT_2M	1742	1114	0.5154639	4	Singlet	Immune_cells
SAT_2M	1422	898	5.3997195	9	Singlet	Mesothelial
SAT_2M	2226	1303	3.3647376	5	Singlet	Immune_cells

SAT_2M	1743	917	3.0892449	5	Singlet	Immune_cells
SAT_2M	5034	2453	3.2322031	9	Singlet	Mesothelial
SAT_2M	2186	1229	1.7343679	0	Singlet	Adipocyte
SAT_2M	1260	830	0.0790514	3	Singlet	FAPs
SAT_2M	2345	1216	0.8085106	10	Singlet	Immune_cells
SAT_2M	2532	1376	7.8470032	1	Singlet	Immune_cells
SAT_2M	2014	1228	0.3463632	1	Singlet	Immune_cells
SAT_2M	1625	932	1.4760148	0	Singlet	Adipocyte
SAT_2M	1706	994	4.3911007	1	Singlet	Immune_cells
SAT_2M	1554	920	2.946829	1	Singlet	Immune_cells
SAT_2M	1297	802	7.3133179	4	Singlet	Immune_cells
SAT_2M	3497	1545	1.3417071	10	Singlet	Immune_cells
SAT_2M	2151	1193	0.4627487	0	Singlet	Adipocyte
SAT_2M	1974	1124	5.5050505	1	Singlet	Immune_cells
SAT_2M	1340	819	5.3452116	5	Singlet	Immune_cells
SAT_2M	2127	1235	3.8371549	1	Singlet	Immune_cells
SAT_2M	2012	1201	1.4814815	4	Singlet	Immune_cells
SAT_2M	2477	1352	8.3299799	1	Singlet	Immune_cells
SAT_2M	5737	2641	4.0826963	9	Singlet	Mesothelial
SAT_2M	2697	1451	0.7366483	0	Singlet	Adipocyte
SAT_2M	2293	1323	1.9113814	1	Singlet	Immune_cells
SAT_2M	1297	822	0.8455035	7	Singlet	Endothelial_cells
SAT_2M	4531	2111	0.3523453	2	Singlet	FAPs
SAT_2M	1404	876	3.1205674	5	Singlet	Immune_cells
SAT_2M	1385	846	5.4597701	1	Singlet	Immune_cells
SAT_2M	1130	726	2.7312775	1	Singlet	Immune_cells
SAT_2M	1477	1000	0.6044325	8	Singlet	Mesothelial
SAT_2M	1947	1068	5.3196931	1	Singlet	Immune_cells
SAT_2M	1219	759	8.7204564	4	Singlet	Immune_cells
SAT_2M	2095	1276	0.0947418	5	Singlet	Immune_cells
SAT_2M	1519	972	0.3942181	7	Singlet	Endothelial_cells
SAT_2M	2122	1295	2.1626704	4	Singlet	Immune_cells
SAT_2M	1407	904	0.8498584	4	Singlet	Immune_cells
SAT_2M	1558	978	0.255102	0	Singlet	Adipocyte
SAT_2M	1547	995	1.414791	0	Singlet	Adipocyte
SAT_2M	1367	926	0.0727273	5	Singlet	Immune_cells
SAT_2M	1092	729	1.7319964	1	Singlet	Immune_cells
SAT_2M	2795	1502	1.1035956	0	Singlet	Adipocyte
SAT_2M	2571	1419	0.1159644	0	Singlet	Adipocyte
SAT_2M	2580	1415	2.4361949	9	Singlet	Mesothelial
SAT_2M	1212	800	0.247117	4	Singlet	Immune_cells
SAT_2M	1276	806	0.3897116	0	Singlet	Adipocyte
SAT_2M	1198	773	0.4979253	3	Singlet	FAPs
SAT_2M	1630	1039	0.670323	1	Singlet	Immune_cells
SAT_2M	1184	753	0.5892256	1	Singlet	Immune_cells
SAT_2M	2263	1258	1.5432099	5	Singlet	Immune_cells
SAT_2M	1760	884	0.3335186	6	Singlet	Neurons
SAT_2M	1247	812	3.8338658	1	Singlet	Immune_cells
SAT_2M	1274	807	3.6050157	1	Singlet	Immune_cells
SAT_2M	2973	1526	0.2010724	0	Singlet	Adipocyte
SAT_2M	1466	907	0.1352265	1	Singlet	Immune_cells

SAT_2M	2129	1214	5.6153486	4	Singlet	Immune_cells
SAT_2M	1181	805	1.5088013	3	Singlet	FAPs
SAT_2M	1281	757	1.5576324	0	Singlet	Adipocyte
SAT_2M	1238	792	1.1281225	4	Singlet	Immune_cells
SAT_2M	1857	1100	0.7498661	3	Singlet	FAPs
SAT_2M	1489	902	3.0120482	1	Singlet	Immune_cells
SAT_2M	1030	700	1.8322083	1	Singlet	Immune_cells
SAT_2M	1788	1105	0.4997224	0	Singlet	Adipocyte
SAT_2M	1530	1006	4.4299674	1	Singlet	Immune_cells
SAT_2M	1286	849	0.3861004	4	Singlet	Immune_cells
SAT_2M	1066	637	1.5917603	10	Singlet	Immune_cells
SAT_2M	1449	916	6.6620879	1	Singlet	Immune_cells
SAT_2M	1820	1161	0.4378763	8	Singlet	Mesothelial
SAT_2M	1344	831	7.9646018	4	Singlet	Immune_cells
SAT_2M	1502	875	4.9071618	1	Singlet	Immune_cells
SAT_2M	1653	1028	0.1201923	1	Singlet	Immune_cells
SAT_2M	1191	805	1.6736402	1	Singlet	Immune_cells
SAT_2M	1801	1065	0.7174393	0	Singlet	Adipocyte
SAT_2M	1501	899	9.6560847	1	Singlet	Immune_cells
SAT_2M	1535	1003	1.0322581	4	Singlet	Immune_cells
SAT_2M	1812	1085	5.7142857	1	Singlet	Immune_cells
SAT_2M	1335	878	0.0745156	8	Singlet	Mesothelial
SAT_2M	9360	3062	0.6932594	10	Singlet	Immune_cells
SAT_2M	1995	1089	2.1489255	1	Singlet	Immune_cells
SAT_2M	4251	2154	10.372278	4	Singlet	Immune_cells
SAT_2M	1443	890	0.6219765	4	Singlet	Immune_cells
SAT_2M	3507	1662	0.6260672	0	Singlet	Adipocyte
SAT_2M	2546	1160	4.7824383	10	Singlet	Immune_cells
SAT_2M	1094	690	0.1814882	4	Singlet	Immune_cells
SAT_2M	2656	1538	4.5454545	8	Singlet	Mesothelial
SAT_2M	1680	1038	1.4218009	0	Singlet	Adipocyte
SAT_2M	2404	1420	1.5295577	3	Singlet	FAPs
SAT_2M	1415	836	6.135402	1	Singlet	Immune_cells
SAT_2M	1300	813	2.6656512	0	Singlet	Adipocyte
SAT_2M	2433	1297	7.6260763	9	Singlet	Mesothelial
SAT_2M	1189	737	1.1725293	3	Singlet	FAPs
SAT_2M	2144	1244	4.8837209	4	Singlet	Immune_cells
SAT_2M	1693	988	0.4708652	0	Singlet	Adipocyte
SAT_2M	1196	811	0.6568145	8	Singlet	Mesothelial
SAT_2M	1367	872	4.0116703	1	Singlet	Immune_cells
SAT_2M	2318	1297	0.4282655	0	Singlet	Adipocyte
SAT_2M	1367	809	7.0751276	1	Singlet	Immune_cells
SAT_2M	1480	978	0.6734007	1	Singlet	Immune_cells
SAT_2M	2106	1158	1.7510648	10	Singlet	Immune_cells
SAT_2M	2010	1158	1.5888779	5	Singlet	Immune_cells
SAT_2M	1731	1007	0.4608295	0	Singlet	Adipocyte
SAT_2M	1635	953	6.2843197	1	Singlet	Immune_cells
SAT_2M	2172	1288	1.7931034	5	Singlet	Immune_cells
SAT_2M	1130	765	0.7042254	1	Singlet	Immune_cells
SAT_2M	1950	1101	1.6871166	4	Singlet	Immune_cells
SAT_2M	1564	880	5.2329292	9	Singlet	Mesothelial

SAT_2M	2312	1187	4.9180328	5	Singlet	Immune_cells
SAT_2M	1133	697	0.1755926	7	Singlet	Endothelial_cells
SAT_2M	7423	2934	5.3140097	9	Singlet	Mesothelial
SAT_2M	1363	856	2.0437956	1	Singlet	Immune_cells
SAT_2M	1188	755	0.5042017	0	Singlet	Adipocyte
SAT_2M	3070	1714	0.4209845	8	Singlet	Mesothelial
SAT_2M	1329	869	1.0502626	4	Singlet	Immune_cells
SAT_2M	2103	1196	6.3920455	1	Singlet	Immune_cells
SAT_2M	1685	1094	0.2955083	11	Singlet	Smooth_muscle_cells
SAT_2M	1619	985	0.0610128	8	Singlet	Mesothelial
SAT_2M	1924	1224	0.9849663	4	Singlet	Immune_cells
SAT_2M	1709	1099	2.5043681	5	Singlet	Immune_cells
SAT_2M	1224	800	2.2727273	4	Singlet	Immune_cells
SAT_2M	3010	1584	0.5632869	0	Singlet	Adipocyte
SAT_2M	1388	881	0.2867384	3	Singlet	FAPs
SAT_2M	3866	1974	7.1613237	9	Singlet	Mesothelial
SAT_2M	1301	779	0.8396947	10	Singlet	Immune_cells
SAT_2M	1073	757	0.4651163	8	Singlet	Mesothelial
SAT_2M	1262	812	3.7095501	1	Singlet	Immune_cells
SAT_2M	1469	895	0.135318	4	Singlet	Immune_cells
SAT_2M	1262	866	0.7069914	4	Singlet	Immune_cells
SAT_2M	1706	1021	3.9250146	4	Singlet	Immune_cells
SAT_2M	1256	821	2.1428571	4	Singlet	Immune_cells
SAT_2M	1830	1028	3.4858388	10	Singlet	Immune_cells
SAT_2M	1293	838	0.1540832	4	Singlet	Immune_cells
SAT_2M	1080	653	0.6445672	6	Singlet	Neurons
SAT_2M	1515	972	1.0437052	4	Singlet	Immune_cells
SAT_2M	4184	2100	7.8281623	9	Singlet	Mesothelial
SAT_2M	4276	2025	0.2561714	2	Singlet	FAPs
SAT_2M	1759	1062	0.2828054	3	Singlet	FAPs
SAT_2M	1484	921	0.6044325	1	Singlet	Immune_cells
SAT_2M	1315	826	3.9453718	4	Singlet	Immune_cells
SAT_2M	1842	1126	0.2166847	0	Singlet	Adipocyte
SAT_2M	1007	689	1.5857284	5	Singlet	Immune_cells
SAT_2M	1607	1032	0.0620347	3	Singlet	FAPs
SAT_2M	1251	755	8.2071713	1	Singlet	Immune_cells
SAT_2M	2382	1378	0.6260434	5	Singlet	Immune_cells
SAT_2M	1235	806	1.7741935	1	Singlet	Immune_cells
SAT_2M	1073	736	0.3710575	4	Singlet	Immune_cells
SAT_2M	1069	737	0.5591799	4	Singlet	Immune_cells
SAT_2M	1285	802	5.1044084	1	Singlet	Immune_cells
SAT_2M	1856	1071	3.707684	1	Singlet	Immune_cells
SAT_2M	2538	1506	0.3532182	5	Singlet	Immune_cells
SAT_2M	1388	788	6.8149211	9	Singlet	Mesothelial
SAT_2M	2422	1310	0.2886598	0	Singlet	Adipocyte
SAT_2M	1051	651	1.9830028	5	Singlet	Immune_cells
SAT_2M	1164	774	0.1715266	5	Singlet	Immune_cells
SAT_2M	2065	1151	9.4202899	1	Singlet	Immune_cells
SAT_2M	1595	1018	1.2476606	1	Singlet	Immune_cells
SAT_2M	1405	875	5.2631579	1	Singlet	Immune_cells
SAT_2M	1487	928	3.9597315	1	Singlet	Immune_cells

SAT_2M	2278	1356	0.7870573	0	Singlet	Adipocyte
SAT_2M	1654	928	3.0722892	10	Singlet	Immune_cells
SAT_2M	1321	835	0.6772009	7	Singlet	Endothelial_cells
SAT_2M	1710	1059	3.3858727	1	Singlet	Immune_cells
SAT_2M	1234	756	5.6542811	1	Singlet	Immune_cells
SAT_2M	2668	1526	0.2985075	9	Singlet	Mesothelial
SAT_2M	1413	856	3.810868	8	Singlet	Mesothelial
SAT_2M	1402	937	0.0712251	4	Singlet	Immune_cells
SAT_2M	1673	1003	0.0594177	1	Singlet	Immune_cells
SAT_2M	1132	739	0.1762115	1	Singlet	Immune_cells
SAT_2M	8387	2892	2.9642857	10	Singlet	Immune_cells
SAT_2M	1361	841	4.1025641	1	Singlet	Immune_cells
SAT_2M	1109	742	0.4492363	5	Singlet	Immune_cells
SAT_2M	3089	1620	2.7490298	8	Singlet	Mesothelial
SAT_2M	1521	940	6.0327869	1	Singlet	Immune_cells
SAT_2M	2199	1333	2.7589326	0	Singlet	Adipocyte
SAT_2M	1291	790	7.0216049	1	Singlet	Immune_cells
SAT_2M	1094	773	0.6369427	4	Singlet	Immune_cells
SAT_2M	1311	785	1.2898331	4	Singlet	Immune_cells
SAT_2M	2626	1536	5.1711027	9	Singlet	Mesothelial
SAT_2M	1938	1141	0.462963	0	Singlet	Adipocyte
SAT_2M	1576	1016	0.4413619	11	Singlet	Smooth_muscle_cells
SAT_2M	1452	843	2.2633745	10	Singlet	Immune_cells
SAT_2M	1077	675	0.2770083	0	Singlet	Adipocyte
SAT_2M	1499	1015	0.0665336	4	Singlet	Immune_cells
SAT_2M	1416	937	0.7042254	4	Singlet	Immune_cells
SAT_2M	1439	943	4.2273042	3	Singlet	FAPs
SAT_2M	1072	693	0.2793296	4	Singlet	Immune_cells
SAT_2M	2136	1286	2.6523965	1	Singlet	Immune_cells
SAT_2M	2952	1710	0.6761325	11	Singlet	Smooth_muscle_cells
SAT_2M	1038	675	0.3846154	4	Singlet	Immune_cells
SAT_2M	1244	862	0	8	Singlet	Mesothelial
SAT_2M	2202	1290	0.4984141	4	Singlet	Immune_cells
SAT_2M	1070	749	0.6523765	9	Singlet	Mesothelial
SAT_2M	2042	1164	12.115291	1	Singlet	Immune_cells
SAT_2M	1043	710	2.2878932	1	Singlet	Immune_cells
SAT_2M	1685	1065	0.4719764	5	Singlet	Immune_cells
SAT_2M	1042	751	0.1908397	4	Singlet	Immune_cells
SAT_2M	2017	1221	3.7567968	5	Singlet	Immune_cells
SAT_2M	2573	1491	3.4522886	9	Singlet	Mesothelial
SAT_2M	1988	1176	0.6490265	0	Singlet	Adipocyte
SAT_2M	1178	816	0.0845309	5	Singlet	Immune_cells
SAT_2M	1212	728	8.3127572	0	Singlet	Adipocyte
SAT_2M	1253	773	1.2678288	1	Singlet	Immune_cells
SAT_2M	1371	893	0.2904866	3	Singlet	FAPs
SAT_2M	1245	748	10.09539	1	Singlet	Immune_cells
SAT_2M	2599	1398	1.1119632	0	Singlet	Adipocyte
SAT_2M	1488	829	12.173913	0	Singlet	Adipocyte
SAT_2M	1261	782	8.458498	1	Singlet	Immune_cells
SAT_2M	2753	1580	0.9067827	11	Singlet	Smooth_muscle_cells
SAT_2M	1442	894	0.1380262	5	Singlet	Immune_cells

SAT_2M	1811	1068	0.0547345	0	Singlet	Adipocyte
SAT_2M	1779	1108	0.8389262	1	Singlet	Immune_cells
SAT_2M	2161	1252	2.2038567	1	Singlet	Immune_cells
SAT_2M	2143	1212	4.0968343	1	Singlet	Immune_cells
SAT_2M	1453	889	1.303155	7	Singlet	Endothelial_cells
SAT_2M	3819	2011	0.1043841	8	Singlet	Mesothelial
SAT_2M	1277	845	2.4199844	1	Singlet	Immune_cells
SAT_2M	5868	2730	3.6078965	9	Singlet	Mesothelial
SAT_2M	1387	922	1.3639627	11	Singlet	Smooth_muscle_cells
SAT_2M	1293	817	0.385505	1	Singlet	Immune_cells
SAT_2M	1434	804	8.5476025	5	Singlet	Immune_cells
SAT_2M	1610	1016	2.3529412	4	Singlet	Immune_cells
SAT_2M	1390	865	0.215208	4	Singlet	Immune_cells
SAT_2M	1298	772	9.7542243	1	Singlet	Immune_cells
SAT_2M	1921	1179	6.7392431	5	Singlet	Immune_cells
SAT_2M	1255	790	1.8181818	1	Singlet	Immune_cells
SAT_2M	1558	999	1.5364917	1	Singlet	Immune_cells
SAT_2M	1315	783	3.4848485	4	Singlet	Immune_cells
SAT_2M	3889	1911	8.7134803	3	Singlet	FAPs
SAT_2M	2227	1224	1.2053571	0	Singlet	Adipocyte
SAT_2M	1701	1072	2.9342723	4	Singlet	Immune_cells
SAT_2M	3210	1604	5.8457711	4	Singlet	Immune_cells
SAT_2M	1288	805	4.3276662	1	Singlet	Immune_cells
SAT_2M	1428	915	0.4895105	1	Singlet	Immune_cells
SAT_2M	1510	969	0.3960396	4	Singlet	Immune_cells
SAT_2M	1181	738	1.0970464	5	Singlet	Immune_cells
SAT_2M	2384	1464	2.5930573	5	Singlet	Immune_cells
SAT_2M	1240	789	4.995971	4	Singlet	Immune_cells
SAT_2M	2039	1178	0.5879471	4	Singlet	Immune_cells
SAT_2M	1776	1100	0.2247191	3	Singlet	FAPs
SAT_2M	2080	1234	3.8332535	4	Singlet	Immune_cells
SAT_2M	1714	1042	0.462963	0	Singlet	Adipocyte
SAT_2M	1343	802	6.547619	9	Singlet	Mesothelial
SAT_2M	1221	781	6.3621533	4	Singlet	Immune_cells
SAT_2M	1214	754	0	0	Singlet	Adipocyte
SAT_2M	3657	1800	0.2452316	0	Singlet	Adipocyte
SAT_2M	1419	840	2.5174825	0	Singlet	Adipocyte
SAT_2M	2852	1750	0.2782609	4	Singlet	Immune_cells
SAT_2M	6043	2842	2.5586002	9	Singlet	Mesothelial
SAT_2M	1961	1246	0.305499	1	Singlet	Immune_cells
SAT_2M	1323	810	0.0751315	0	Singlet	Adipocyte
SAT_2M	1813	1136	0.2751789	5	Singlet	Immune_cells
SAT_2M	1463	855	2.9231815	0	Singlet	Adipocyte
SAT_2M	1088	746	1.1872146	4	Singlet	Immune_cells
SAT_2M	1403	882	0.7102273	4	Singlet	Immune_cells
SAT_2M	2526	1402	2.8853755	1	Singlet	Immune_cells
SAT_2M	1483	894	1.6823688	8	Singlet	Mesothelial
SAT_2M	2231	1227	0.2231147	3	Singlet	FAPs
SAT_2M	3115	1470	0.9309791	3	Singlet	FAPs
SAT_2M	1049	695	1.7061611	4	Singlet	Immune_cells
SAT_2M	5458	2290	1.7547066	10	Singlet	Immune_cells

SAT_2M	1232	788	1.2067578	0	Singlet	Adipocyte
SAT_2M	1086	700	2.5617566	4	Singlet	Immune_cells
SAT_2M	1183	763	1.6020236	8	Singlet	Mesothelial
SAT_2M	1304	795	3.1369549	0	Singlet	Adipocyte
SAT_2M	1971	1123	1.8218623	1	Singlet	Immune_cells
SAT_2M	2240	1273	0.2228164	1	Singlet	Immune_cells
SAT_2M	6628	2767	4.1227806	9	Singlet	Mesothelial
SAT_2M	1242	726	4.3338684	1	Singlet	Immune_cells
SAT_2M	2828	1485	0.0704225	0	Singlet	Adipocyte
SAT_2M	1730	973	0.9792627	3	Singlet	FAPs
SAT_2M	1421	907	0.4189944	0	Singlet	Adipocyte
SAT_2M	1567	985	1.9047619	0	Singlet	Adipocyte
SAT_2M	1572	987	0.0635728	4	Singlet	Immune_cells
SAT_2M	1200	771	1.1599006	4	Singlet	Immune_cells
SAT_2M	1377	909	3.9158811	1	Singlet	Immune_cells
SAT_2M	1298	849	0.6891271	4	Singlet	Immune_cells
SAT_2M	1262	826	0.7120253	11	Singlet	Smooth_muscle_cells
SAT_2M	2078	1325	0.6190476	4	Singlet	Immune_cells
SAT_2M	1807	1037	6.4088398	1	Singlet	Immune_cells
SAT_2M	1069	771	0.7187781	6	Singlet	Neurons
SAT_2M	1509	968	0.5952381	4	Singlet	Immune_cells
SAT_2M	1516	919	0.3929273	4	Singlet	Immune_cells
SAT_2M	1884	1182	0.3176284	1	Singlet	Immune_cells
SAT_2M	2387	1375	4.317144	5	Singlet	Immune_cells
SAT_2M	1396	849	0.8571429	0	Singlet	Adipocyte
SAT_2M	1014	645	6.6666667	9	Singlet	Mesothelial
SAT_2M	2404	1404	0.6614303	0	Singlet	Adipocyte
SAT_2M	1082	738	0.5469462	1	Singlet	Immune_cells
SAT_2M	1249	834	2.4720893	1	Singlet	Immune_cells
SAT_2M	1185	750	2.9411765	3	Singlet	FAPs
SAT_2M	1149	768	0.0868056	4	Singlet	Immune_cells
SAT_2M	1488	905	3.1522468	1	Singlet	Immune_cells
SAT_2M	3566	1972	7.877095	9	Singlet	Mesothelial
SAT_2M	1127	756	1.4159292	1	Singlet	Immune_cells
SAT_2M	2184	1262	2.1917808	4	Singlet	Immune_cells
SAT_2M	1813	1080	0.7709251	1	Singlet	Immune_cells
SAT_2M	2939	1532	1.4280857	10	Singlet	Immune_cells
SAT_2M	2542	1260	2.1594032	10	Singlet	Immune_cells
SAT_2M	1264	798	0.3152088	4	Singlet	Immune_cells
SAT_2M	1343	846	0.5956813	4	Singlet	Immune_cells
SAT_2M	1199	813	0.8257638	3	Singlet	FAPs
SAT_2M	2051	1187	6.3346228	5	Singlet	Immune_cells
SAT_2M	3077	1666	0	0	Singlet	Adipocyte
SAT_2M	1336	896	2.9147982	1	Singlet	Immune_cells
SAT_2M	1581	1049	0.7528231	6	Singlet	Neurons
SAT_2M	1083	710	2.5664528	1	Singlet	Immune_cells
SAT_2M	1443	865	7.3153899	1	Singlet	Immune_cells
SAT_2M	2033	1240	2.5477707	4	Singlet	Immune_cells
SAT_2M	1273	812	6.0344828	1	Singlet	Immune_cells
SAT_2M	1559	935	5.1592357	1	Singlet	Immune_cells
SAT_2M	8646	3605	9.5463465	9	Singlet	Mesothelial

SAT_2M	1847	1167	0.7567568	1	Singlet	Immune_cells
SAT_2M	1304	845	0.608365	1	Singlet	Immune_cells
SAT_2M	1349	845	0.5173688	4	Singlet	Immune_cells
SAT_2M	1739	1019	1.0303377	0	Singlet	Adipocyte
SAT_2M	1374	925	2.5974026	4	Singlet	Immune_cells
SAT_2M	1249	794	0.2394254	4	Singlet	Immune_cells
SAT_2M	1207	745	0.2477291	4	Singlet	Immune_cells
SAT_2M	1302	852	3.7519142	6	Singlet	Neurons
SAT_2M	1207	769	7.5409836	4	Singlet	Immune_cells
SAT_2M	1405	902	3.7588652	1	Singlet	Immune_cells
SAT_2M	5048	2461	1.6422636	9	Singlet	Mesothelial
SAT_2M	3434	1603	2.6422764	10	Singlet	Immune_cells
SAT_2M	1455	944	6.7715458	4	Singlet	Immune_cells
SAT_2M	1489	952	5.941255	1	Singlet	Immune_cells
SAT_2M	3851	1872	1.7357513	10	Singlet	Immune_cells
SAT_2M	1278	819	6.1032864	5	Singlet	Immune_cells
SAT_2M	1626	983	9.9325567	4	Singlet	Immune_cells
SAT_2M	1370	949	0.1458789	8	Singlet	Mesothelial
SAT_2M	3191	1636	0.2502346	0	Singlet	Adipocyte
SAT_2M	1557	991	0.3196931	4	Singlet	Immune_cells
SAT_2M	2396	1333	6.6888243	1	Singlet	Immune_cells
SAT_2M	1500	995	1.6600266	1	Singlet	Immune_cells
SAT_2M	1007	679	0.4940711	0	Singlet	Adipocyte
SAT_2M	1276	810	5.4644809	1	Singlet	Immune_cells
SAT_2M	1103	735	6.8778281	1	Singlet	Immune_cells
SAT_2M	1136	751	0.6134969	1	Singlet	Immune_cells
SAT_2M	1023	668	1.5579357	0	Singlet	Adipocyte
SAT_2M	8604	3435	8.3739272	9	Singlet	Mesothelial
SAT_2M	1583	989	1.3240858	0	Singlet	Adipocyte
SAT_2M	1494	975	0.4676019	5	Singlet	Immune_cells
SAT_2M	1209	797	1.4026403	4	Singlet	Immune_cells
SAT_2M	1709	1027	5.6428156	1	Singlet	Immune_cells
SAT_2M	3736	1886	0.8006405	11	Singlet	Smooth_muscle_cells
SAT_2M	1611	1039	0.5552128	5	Singlet	Immune_cells
SAT_2M	1612	1087	1.0995724	6	Singlet	Neurons
SAT_2M	1346	850	4.8255382	1	Singlet	Immune_cells
SAT_2M	1745	1065	0.5704507	0	Singlet	Adipocyte
SAT_2M	2443	1323	3.1096563	8	Singlet	Mesothelial
SAT_2M	1522	877	3.8688525	3	Singlet	FAPs
SAT_2M	1069	737	2.1455224	4	Singlet	Immune_cells
SAT_2M	1234	767	4.1062802	1	Singlet	Immune_cells
SAT_2M	7310	3145	4.9043716	9	Singlet	Mesothelial
SAT_2M	1262	815	1.9762846	10	Singlet	Immune_cells
SAT_2M	2636	1455	0.3775009	8	Singlet	Mesothelial
SAT_2M	4364	1980	0.4108651	0	Singlet	Adipocyte
SAT_2M	1057	719	0.8482564	4	Singlet	Immune_cells
SAT_2M	2691	1535	3.7352071	1	Singlet	Immune_cells
SAT_2M	1393	942	2.4113475	1	Singlet	Immune_cells
SAT_2M	1426	945	1.048218	11	Singlet	Smooth_muscle_cells
SAT_2M	1111	629	0.4492363	0	Singlet	Adipocyte
SAT_2M	3210	1634	0.1550388	0	Singlet	Adipocyte

SAT_2M	1785	1108	0.8928571	0	Singlet	Adipocyte
SAT_2M	1447	863	0.4814305	0	Singlet	Adipocyte
SAT_2M	1325	820	0.9730539	4	Singlet	Immune_cells
SAT_2M	1508	939	8.1740277	1	Singlet	Immune_cells
SAT_2M	3319	1890	1.7122259	8	Singlet	Mesothelial
SAT_2M	1602	1030	0.4361371	4	Singlet	Immune_cells
SAT_2M	1261	822	6.71406	1	Singlet	Immune_cells
SAT_2M	1531	946	2.7308192	1	Singlet	Immune_cells
SAT_2M	1666	1037	1.0143198	6	Singlet	Neurons
SAT_2M	1649	1006	0.4221954	0	Singlet	Adipocyte
SAT_2M	1535	951	0.843608	0	Singlet	Adipocyte
SAT_2M	2688	1505	3.9157739	1	Singlet	Immune_cells
SAT_2M	1016	658	4.0354331	0	Singlet	Adipocyte
SAT_2M	1572	982	2.7989822	4	Singlet	Immune_cells
SAT_2M	1005	632	0.4945598	0	Singlet	Adipocyte
SAT_2M	1629	1017	4.1514042	4	Singlet	Immune_cells
SAT_2M	1348	830	0.2956393	1	Singlet	Immune_cells
SAT_2M	1268	803	9.4043887	1	Singlet	Immune_cells
SAT_2M	1215	820	1.965602	4	Singlet	Immune_cells
SAT_2M	1272	782	6.2745098	1	Singlet	Immune_cells
SAT_2M	2536	1391	0.0785238	8	Singlet	Mesothelial
SAT_2M	1422	889	0.209937	1	Singlet	Immune_cells
SAT_2M	1299	814	9.984639	1	Singlet	Immune_cells
SAT_2M	1217	804	0.4088307	0	Singlet	Adipocyte
SAT_2M	1627	978	0.7955936	0	Singlet	Adipocyte
SAT_2M	1299	879	1.2987013	4	Singlet	Immune_cells
SAT_2M	1696	1037	0.7611241	1	Singlet	Immune_cells
SAT_2M	1355	869	1.025641	0	Singlet	Adipocyte
SAT_2M	1271	805	8.2877248	9	Singlet	Mesothelial
SAT_2M	2503	1332	0.7949126	0	Singlet	Adipocyte
SAT_2M	1382	834	5.1079137	1	Singlet	Immune_cells
SAT_2M	1848	1083	0.7555316	0	Singlet	Adipocyte
SAT_2M	1751	969	14.058054	1	Singlet	Immune_cells
SAT_2M	4302	2083	0.7866728	0	Singlet	Adipocyte
SAT_2M	4017	1852	2.5074479	3	Singlet	FAPs
SAT_2M	2012	1248	5.109127	8	Singlet	Mesothelial
SAT_2M	1104	797	0.7207207	4	Singlet	Immune_cells
SAT_2M	4227	1817	3.9433294	10	Singlet	Immune_cells
SAT_2M	1094	757	5.4545455	8	Singlet	Mesothelial
SAT_2M	1507	914	2.0475561	0	Singlet	Adipocyte
SAT_2M	2087	1164	4.2583732	5	Singlet	Immune_cells
SAT_2M	2214	1244	5.718145	3	Singlet	FAPs
SAT_2M	1217	772	8.1566069	1	Singlet	Immune_cells
SAT_2M	1146	768	0.4310345	2	Singlet	FAPs
SAT_2M	2286	1359	0.5228758	0	Singlet	Adipocyte
SAT_2M	3499	1825	1.5104018	0	Singlet	Adipocyte
SAT_2M	1020	689	1.7526777	1	Singlet	Immune_cells
SAT_2M	1004	635	4.7666336	3	Singlet	FAPs
SAT_2M	1312	805	7.5056861	1	Singlet	Immune_cells
SAT_2M	1196	802	0.910596	4	Singlet	Immune_cells
SAT_2M	1434	897	9.1412742	1	Singlet	Immune_cells

SAT_2M	1333	833	5.4640719	1	Singlet	Immune_cells
SAT_2M	2023	1060	8.9478859	0	Singlet	Adipocyte
SAT_2M	2094	1232	1.8562589	7	Singlet	Endothelial_cells
SAT_2M	2100	1266	1.657197	11	Singlet	Smooth_muscle_cells
SAT_2M	1218	808	2.4509804	1	Singlet	Immune_cells
SAT_2M	1030	709	0.3861004	5	Singlet	Immune_cells
SAT_2M	2041	1180	4.7828209	1	Singlet	Immune_cells
SAT_2M	1165	738	0.5128205	0	Singlet	Adipocyte
SAT_2M	2118	1240	4.2313117	5	Singlet	Immune_cells
SAT_2M	1399	918	0.4996431	1	Singlet	Immune_cells
SAT_2M	1113	702	0.3577818	0	Singlet	Adipocyte
SAT_2M	3679	1902	8.1936182	9	Singlet	Mesothelial
SAT_2M	1234	805	0.886382	1	Singlet	Immune_cells
SAT_2M	1723	1147	2.0677771	5	Singlet	Immune_cells
SAT_2M	2215	1236	0.8082622	0	Singlet	Adipocyte
SAT_2M	1135	770	3.2513181	4	Singlet	Immune_cells
SAT_2M	1118	762	0.2666667	11	Singlet	Smooth_muscle_cells
SAT_2M	1423	887	8.345021	1	Singlet	Immune_cells
SAT_2M	1263	808	0.3159558	0	Singlet	Adipocyte
SAT_2M	1559	1010	0.3184713	4	Singlet	Immune_cells
SAT_2M	1801	1047	0.7743363	0	Singlet	Adipocyte
SAT_2M	1071	686	0.8356546	1	Singlet	Immune_cells
SAT_2M	1773	1080	0.2812148	3	Singlet	FAPs
SAT_2M	1639	1040	2.0109689	4	Singlet	Immune_cells
SAT_2M	2125	1285	3.3739456	5	Singlet	Immune_cells
SAT_2M	1703	1000	3.9719626	1	Singlet	Immune_cells
SAT_2M	1335	890	1.264881	4	Singlet	Immune_cells
SAT_2M	1911	1098	2.1387585	1	Singlet	Immune_cells
SAT_2M	1363	841	2.6315789	1	Singlet	Immune_cells
SAT_2M	1430	896	0.6276151	1	Singlet	Immune_cells
SAT_2M	3248	1661	6.9653268	9	Singlet	Mesothelial
SAT_2M	3533	1850	0.5644934	0	Singlet	Adipocyte
SAT_2M	1476	836	6.7476383	0	Singlet	Adipocyte
SAT_2M	2828	1577	2.9608742	9	Singlet	Mesothelial
SAT_2M	1983	1186	0.952381	4	Singlet	Immune_cells
SAT_2M	1328	853	0.5259204	1	Singlet	Immune_cells
SAT_2M	1636	994	1.9452888	1	Singlet	Immune_cells
SAT_2M	1108	663	5.4954955	3	Singlet	FAPs
SAT_2M	1458	837	5	0	Singlet	Adipocyte
SAT_2M	1514	951	1.7015707	1	Singlet	Immune_cells
SAT_2M	1782	991	2.1324355	3	Singlet	FAPs
SAT_2M	1183	755	0.5025126	0	Singlet	Adipocyte
SAT_2M	1903	1064	8.3246073	1	Singlet	Immune_cells
SAT_2M	1142	691	2.2707424	0	Singlet	Adipocyte
SAT_2M	1968	1191	5.8467742	1	Singlet	Immune_cells
SAT_2M	1092	698	0.4549591	0	Singlet	Adipocyte
SAT_2M	1448	890	2.2587269	0	Singlet	Adipocyte
SAT_2M	2427	1464	0.2050861	6	Singlet	Neurons
SAT_2M	1357	846	0.2919708	4	Singlet	Immune_cells
SAT_2M	1522	993	0.5225343	1	Singlet	Immune_cells
SAT_2M	2769	1474	0.5755396	0	Singlet	Adipocyte

SAT_2M	1659	1030	0.359928	0	Singlet	Adipocyte
SAT_2M	1748	1018	1.9351167	0	Singlet	Adipocyte
SAT_2M	2583	1468	0.7716049	2	Singlet	FAPs
SAT_2M	1845	1183	0.2699784	11	Singlet	Smooth_muscle_cells
SAT_2M	2566	1387	3.4589973	0	Singlet	Adipocyte
SAT_2M	2731	1354	4.3034282	1	Singlet	Immune_cells
SAT_2M	1328	833	2.8528529	1	Singlet	Immune_cells
SAT_2M	1031	598	1.5473888	1	Singlet	Immune_cells
SAT_2M	3156	1702	1.5161087	1	Singlet	Immune_cells
SAT_2M	2249	1217	5.1065719	1	Singlet	Immune_cells
SAT_2M	1487	930	0.2	1	Singlet	Immune_cells
SAT_2M	1399	849	5.2142857	1	Singlet	Immune_cells
SAT_2M	1231	796	2.1018593	11	Singlet	Smooth_muscle_cells
SAT_2M	2374	1400	2.9362416	8	Singlet	Mesothelial
SAT_2M	1828	1119	2.568306	1	Singlet	Immune_cells
SAT_2M	1945	1241	2.0460358	9	Singlet	Mesothelial
SAT_2M	1045	736	3.219697	9	Singlet	Mesothelial
SAT_2M	2487	1355	0.4014452	0	Singlet	Adipocyte
SAT_2M	1308	805	3.5060976	1	Singlet	Immune_cells
SAT_2M	1655	1090	0.2403846	8	Singlet	Mesothelial
SAT_2M	1383	889	5.1873199	1	Singlet	Immune_cells
SAT_2M	1409	895	2.1111893	0	Singlet	Adipocyte
SAT_2M	1724	1045	0.9786989	0	Singlet	Adipocyte
SAT_2M	1323	807	4.826546	1	Singlet	Immune_cells
SAT_2M	1837	952	7.4796748	0	Singlet	Adipocyte
SAT_2M	1306	719	11.544343	0	Singlet	Adipocyte
SAT_2M	4030	1817	1.8312299	10	Singlet	Immune_cells
SAT_2M	1121	700	0.5319149	1	Singlet	Immune_cells
SAT_2M	1361	845	1.1730205	0	Singlet	Adipocyte
SAT_2M	1815	1049	1.980198	3	Singlet	FAPs
SAT_2M	1567	970	5.8486968	1	Singlet	Immune_cells
SAT_2M	1804	1130	0.2189382	8	Singlet	Mesothelial
SAT_2M	2312	1376	0.4735256	0	Singlet	Adipocyte
SAT_2M	1150	744	8.7931034	1	Singlet	Immune_cells
SAT_2M	4094	1896	9.1640263	9	Singlet	Mesothelial
SAT_2M	1364	884	1.0248902	9	Singlet	Mesothelial
SAT_2M	1977	1233	0.9547739	4	Singlet	Immune_cells
SAT_2M	1913	1118	5.691906	1	Singlet	Immune_cells
SAT_2M	3257	1633	8.3409997	1	Singlet	Immune_cells
SAT_2M	1102	751	6.8161435	11	Singlet	Smooth_muscle_cells
SAT_2M	1864	1072	7.2153928	4	Singlet	Immune_cells
SAT_2M	3219	1634	1.9832662	0	Singlet	Adipocyte
SAT_2M	1793	1079	0.2214839	1	Singlet	Immune_cells
SAT_2M	1661	984	0.4201681	0	Singlet	Adipocyte
SAT_2M	1709	930	11.826087	1	Singlet	Immune_cells
SAT_2M	1602	987	0.3103662	4	Singlet	Immune_cells
SAT_2M	1350	819	8.9430894	1	Singlet	Immune_cells
SAT_2M	1375	908	4.1756659	4	Singlet	Immune_cells
SAT_2M	1029	625	1.1571842	9	Singlet	Mesothelial
SAT_2M	2196	1258	9.0415913	4	Singlet	Immune_cells
SAT_2M	1103	723	0.1796945	6	Singlet	Neurons

SAT_2M	1472	945	8.3389831	1	Singlet	Immune_cells
SAT_2M	1353	846	0.5147059	1	Singlet	Immune_cells
SAT_2M	1154	755	0.2575107	1	Singlet	Immune_cells
SAT_2M	1143	707	0.9581882	0	Singlet	Adipocyte
SAT_2M	2046	1278	4.4303797	8	Singlet	Mesothelial
SAT_2M	1604	979	3.2178218	0	Singlet	Adipocyte
SAT_2M	2547	1389	0.4308656	0	Singlet	Adipocyte
SAT_2M	1253	686	12.440191	10	Singlet	Immune_cells
SAT_2M	1450	822	0.5483208	1	Singlet	Immune_cells
SAT_2M	1156	754	0.7745267	0	Singlet	Adipocyte
SAT_2M	1103	768	0.8960573	7	Singlet	Endothelial_cells
SAT_2M	1502	973	4.0585496	7	Singlet	Endothelial_cells
SAT_2M	1569	987	0.505689	4	Singlet	Immune_cells
SAT_2M	1488	902	0.2005348	4	Singlet	Immune_cells
SAT_2M	1483	984	0.6028131	8	Singlet	Mesothelial
SAT_2M	1841	1091	0.2162162	0	Singlet	Adipocyte
SAT_2M	1199	828	0.4995837	8	Singlet	Mesothelial
SAT_2M	2471	1275	9.3145161	1	Singlet	Immune_cells
SAT_2M	3590	1902	3.4751182	5	Singlet	Immune_cells
SAT_2M	1023	718	0.7774538	4	Singlet	Immune_cells
SAT_2M	1290	845	1.003861	3	Singlet	FAPs
SAT_2M	1126	725	0.7048458	2	Singlet	FAPs
SAT_2M	1895	1068	6.5893516	1	Singlet	Immune_cells
SAT_2M	1008	685	2.2660099	3	Singlet	FAPs
SAT_2M	2111	1215	0.8470588	3	Singlet	FAPs
SAT_2M	1697	1042	0.2917153	0	Singlet	Adipocyte
SAT_2M	1248	837	2.3125997	1	Singlet	Immune_cells
SAT_2M	1550	890	0.7722008	1	Singlet	Immune_cells
SAT_2M	1701	1023	0.46811	1	Singlet	Immune_cells
SAT_2M	2331	1318	2.2649573	1	Singlet	Immune_cells
SAT_2M	1303	827	4.6529367	1	Singlet	Immune_cells
SAT_2M	1033	650	2.3166023	0	Singlet	Adipocyte
SAT_2M	1142	759	0.5212858	1	Singlet	Immune_cells
SAT_2M	2606	1420	0.1530222	8	Singlet	Mesothelial
SAT_2M	1114	625	10.88314	3	Singlet	FAPs
SAT_2M	1084	688	1.6528926	5	Singlet	Immune_cells
SAT_2M	4160	2304	2.1582734	4	Singlet	Immune_cells
SAT_2M	1428	824	0.349162	10	Singlet	Immune_cells
SAT_2M	1286	804	1.3209013	0	Singlet	Adipocyte
SAT_2M	1301	837	2.607362	4	Singlet	Immune_cells
SAT_2M	1021	690	0.4854369	4	Singlet	Immune_cells
SAT_2M	2401	1331	4.1545492	1	Singlet	Immune_cells
SAT_2M	1436	901	5.7320442	1	Singlet	Immune_cells
SAT_2M	1080	738	1.8450185	4	Singlet	Immune_cells
SAT_2M	1349	906	3.8179148	1	Singlet	Immune_cells
SAT_2M	1429	877	6.6805846	1	Singlet	Immune_cells
SAT_2M	1390	852	2.2158685	1	Singlet	Immune_cells
SAT_2M	1924	1164	5.4780362	1	Singlet	Immune_cells
SAT_2M	1312	833	0.6833713	1	Singlet	Immune_cells
SAT_2M	1721	1074	0.1160093	4	Singlet	Immune_cells
SAT_2M	1337	901	0.7446016	0	Singlet	Adipocyte

SAT_2M	3841	1837	10.585176	9	Singlet	Mesothelial
SAT_2M	1176	759	3.1223629	3	Singlet	FAPs
SAT_2M	2947	1580	1.8817204	0	Singlet	Adipocyte
SAT_2M	1908	1126	0.5227392	1	Singlet	Immune_cells
SAT_2M	1406	852	4.4680851	1	Singlet	Immune_cells
SAT_2M	4249	2035	3.286385	9	Singlet	Mesothelial
SAT_2M	2873	1462	1.9110493	10	Singlet	Immune_cells
SAT_2M	1387	844	0.7168459	0	Singlet	Adipocyte
SAT_2M	3079	1640	1.0349288	0	Singlet	Adipocyte
SAT_2M	1050	685	0.5676443	4	Singlet	Immune_cells
SAT_2M	2570	1231	0.8543689	10	Singlet	Immune_cells
SAT_2M	3931	2094	8.5880273	4	Singlet	Immune_cells
SAT_2M	2476	1486	0.3626108	1	Singlet	Immune_cells
SAT_2M	1254	805	1.5007899	0	Singlet	Adipocyte
SAT_2M	1335	877	0.7440476	0	Singlet	Adipocyte
SAT_2M	3489	1747	1.3409415	0	Singlet	Adipocyte
SAT_2M	1046	715	0.7532957	5	Singlet	Immune_cells
SAT_2M	1383	927	1.218638	1	Singlet	Immune_cells
SAT_2M	1316	854	0.9848485	5	Singlet	Immune_cells
SAT_2M	2470	1379	0.2017756	0	Singlet	Adipocyte
SAT_2M	2581	1430	1.3862149	0	Singlet	Adipocyte
SAT_2M	1034	611	2.8790787	3	Singlet	FAPs
SAT_2M	1060	749	6.1739944	1	Singlet	Immune_cells
SAT_2M	1814	1071	0.933553	0	Singlet	Adipocyte
SAT_2M	1459	873	2.5974026	4	Singlet	Immune_cells
SAT_2M	1142	740	4.4386423	9	Singlet	Mesothelial
SAT_2M	1712	1025	0.5241701	4	Singlet	Immune_cells
SAT_2M	1817	1000	2.6923077	1	Singlet	Immune_cells
SAT_2M	1143	769	0.2595156	0	Singlet	Adipocyte
SAT_2M	1221	813	2.3615635	2	Singlet	FAPs
SAT_2M	1355	865	0.2941176	0	Singlet	Adipocyte
SAT_2M	3525	1858	7.9671109	9	Singlet	Mesothelial
SAT_2M	1235	782	0.8835341	0	Singlet	Adipocyte
SAT_2M	1407	874	8.1502481	1	Singlet	Immune_cells
SAT_2M	3263	1662	0.5187672	0	Singlet	Adipocyte
SAT_2M	1727	1069	0.1154734	8	Singlet	Mesothelial
SAT_2M	1055	695	1.1320755	3	Singlet	FAPs
SAT_2M	1375	859	3.968254	3	Singlet	FAPs
SAT_2M	1498	947	3.3730159	1	Singlet	Immune_cells
SAT_2M	1433	944	0	8	Singlet	Mesothelial
SAT_2M	1181	755	0.4208754	0	Singlet	Adipocyte
SAT_2M	2226	1362	0.4895416	0	Singlet	Adipocyte
SAT_2M	1410	920	0.9873061	3	Singlet	FAPs
SAT_2M	1308	787	1.3740458	0	Singlet	Adipocyte
SAT_2M	2412	1305	1.7384106	0	Singlet	Adipocyte
SAT_2M	2177	1336	2.1431829	1	Singlet	Immune_cells
SAT_2M	2028	1204	1.0314342	1	Singlet	Immune_cells
SAT_2M	3402	1869	9.6385542	9	Singlet	Mesothelial
SAT_2M	1180	772	2.1940928	1	Singlet	Immune_cells
SAT_2M	1637	966	5.0640635	0	Singlet	Adipocyte
SAT_2M	2273	1063	12.648221	10	Singlet	Immune_cells

SAT_2M	1061	704	0.5649718	0	Singlet	Adipocyte
SAT_2M	2172	1204	2.2549471	0	Singlet	Adipocyte
SAT_2M	1130	746	0.8795075	4	Singlet	Immune_cells
SAT_2M	2407	1268	1.8249689	3	Singlet	FAPs
SAT_2M	1028	665	3.7754114	4	Singlet	Immune_cells
SAT_2M	1122	690	1.5017668	10	Singlet	Immune_cells
SAT_2M	1390	869	0.4294918	0	Singlet	Adipocyte
SAT_2M	1277	803	2.5720966	4	Singlet	Immune_cells
SAT_2M	1569	941	4.1348601	7	Singlet	Endothelial_cells
SAT_2M	1384	756	12.849964	1	Singlet	Immune_cells
SAT_2M	1228	787	0.0811688	4	Singlet	Immune_cells
SAT_2M	1243	793	5.9856345	1	Singlet	Immune_cells
SAT_2M	1392	892	0.2148997	11	Singlet	Smooth_muscle_cells
SAT_2M	1826	1087	2.1845986	1	Singlet	Immune_cells
SAT_2M	1593	1056	0.8114856	6	Singlet	Neurons
SAT_2M	1658	966	1.0191847	0	Singlet	Adipocyte
SAT_2M	1445	897	0.5502063	0	Singlet	Adipocyte
SAT_2M	1215	798	1.4742015	1	Singlet	Immune_cells
SAT_2M	1725	1009	1.7877739	0	Singlet	Adipocyte
SAT_2M	1307	844	0.2283105	0	Singlet	Adipocyte
SAT_2M	3978	1753	2.5827482	10	Singlet	Immune_cells
SAT_2M	1117	747	0.6227758	4	Singlet	Immune_cells
SAT_2M	1314	853	0.150263	4	Singlet	Immune_cells
SAT_2M	2361	1323	0.2109705	8	Singlet	Mesothelial
SAT_2M	2386	1291	0.8354219	0	Singlet	Adipocyte
SAT_2M	1274	865	0	1	Singlet	Immune_cells
SAT_2M	1589	1021	7.5282309	4	Singlet	Immune_cells
SAT_2M	1320	827	0.4514673	0	Singlet	Adipocyte
SAT_2M	2655	1445	0.5609574	0	Singlet	Adipocyte
SAT_2M	1073	734	2.8837209	4	Singlet	Immune_cells
SAT_2M	1764	1049	7.4576271	1	Singlet	Immune_cells
SAT_2M	1149	763	2.5108225	11	Singlet	Smooth_muscle_cells
SAT_2M	2830	1579	0.6342495	0	Singlet	Adipocyte
SAT_2M	2022	1181	3.9980257	5	Singlet	Immune_cells
SAT_2M	1688	1036	5.2569403	4	Singlet	Immune_cells
SAT_2M	1037	714	0.3846154	1	Singlet	Immune_cells
SAT_2M	1508	894	0.9920635	8	Singlet	Mesothelial
SAT_2M	1840	1030	5.624662	10	Singlet	Immune_cells
SAT_2M	1704	1123	2.9737609	4	Singlet	Immune_cells
SAT_2M	2486	1362	0.5195843	10	Singlet	Immune_cells
SAT_2M	1723	1044	0.6373117	7	Singlet	Endothelial_cells
SAT_2M	1016	665	1.9607843	7	Singlet	Endothelial_cells
SAT_2M	1796	1132	5.0083472	4	Singlet	Immune_cells
SAT_2M	10662	3011	1.2363023	10	Singlet	Immune_cells
SAT_2M	1702	1079	0.2922268	8	Singlet	Mesothelial
SAT_2M	2499	1465	1.276426	11	Singlet	Smooth_muscle_cells
SAT_2M	1244	822	4.6325879	1	Singlet	Immune_cells
SAT_2M	2045	1235	0.8780488	1	Singlet	Immune_cells
SAT_2M	1402	891	9.787234	1	Singlet	Immune_cells
SAT_2M	1403	889	0.2126152	1	Singlet	Immune_cells
SAT_2M	1466	831	13.473257	1	Singlet	Immune_cells

SAT_2M	1120	641	5.1785714	10	Singlet	Immune_cells
SAT_2M	1283	836	0.621118	3	Singlet	FAPs
SAT_2M	2617	1373	2.0190476	0	Singlet	Adipocyte
SAT_2M	1573	918	12.6109	4	Singlet	Immune_cells
SAT_2M	1781	1103	0.5595971	4	Singlet	Immune_cells
SAT_2M	1972	1115	2.2244692	10	Singlet	Immune_cells
SAT_2M	2242	1317	4.2685638	4	Singlet	Immune_cells
SAT_2M	1079	761	0.8333333	1	Singlet	Immune_cells
SAT_2M	4495	2097	0.2658985	11	Singlet	Smooth_muscle_cells
SAT_2M	1814	1082	2.6908292	1	Singlet	Immune_cells
SAT_2M	5829	2700	1.8334476	9	Singlet	Mesothelial
SAT_2M	1467	1001	1.0183299	9	Singlet	Mesothelial
SAT_2M	1619	1000	1.7209588	1	Singlet	Immune_cells
SAT_2M	1460	929	1.6293279	0	Singlet	Adipocyte
SAT_2M	1474	897	3.5762483	10	Singlet	Immune_cells
SAT_2M	2041	1275	3.6656892	11	Singlet	Smooth_muscle_cells
SAT_2M	1602	976	7.7018634	1	Singlet	Immune_cells
SAT_2M	1398	812	14.051355	1	Singlet	Immune_cells
SAT_2M	2082	1140	6.9311663	1	Singlet	Immune_cells
SAT_2M	1734	1058	0.2296211	0	Singlet	Adipocyte
SAT_2M	2548	1741	0.6982727	6	Singlet	Neurons
SAT_2M	1293	792	0	0	Singlet	Adipocyte
SAT_2M	1516	941	3.0203546	1	Singlet	Immune_cells
SAT_2M	1314	859	0.1510574	8	Singlet	Mesothelial
SAT_2M	1580	945	1.5141956	0	Singlet	Adipocyte
SAT_2M	2409	1313	2.897351	4	Singlet	Immune_cells
SAT_2M	1815	1108	0.9863014	8	Singlet	Mesothelial
SAT_2M	1606	988	2.7278363	7	Singlet	Endothelial_cells
SAT_2M	1425	916	7.5471698	4	Singlet	Immune_cells
SAT_2M	1302	819	0.1530222	1	Singlet	Immune_cells
SAT_2M	1877	1009	5.3780618	5	Singlet	Immune_cells
SAT_2M	1029	687	0.7736944	4	Singlet	Immune_cells
SAT_2M	1162	866	3.0936455	6	Singlet	Neurons
SAT_2M	1620	992	0.3071253	0	Singlet	Adipocyte
SAT_2M	2507	1344	1.2718601	3	Singlet	FAPs
SAT_2M	1443	896	4.4982699	1	Singlet	Immune_cells
SAT_2M	1454	959	0.4103967	0	Singlet	Adipocyte
SAT_2M	1022	696	0.3875969	4	Singlet	Immune_cells
SAT_2M	1738	1013	3.8790645	1	Singlet	Immune_cells
SAT_2M	1114	738	0.1784121	5	Singlet	Immune_cells
SAT_2M	1737	1061	0.9142857	0	Singlet	Adipocyte
SAT_2M	2275	1203	12.472552	4	Singlet	Immune_cells
SAT_2M	4292	1945	1.6042781	0	Singlet	Adipocyte
SAT_2M	2276	1193	5.0854888	1	Singlet	Immune_cells
SAT_2M	3883	1856	0.7958922	0	Singlet	Adipocyte
SAT_2M	1402	857	0.2840909	4	Singlet	Immune_cells
SAT_2M	1271	849	3.5714286	1	Singlet	Immune_cells
SAT_2M	1258	843	4.1897233	1	Singlet	Immune_cells
SAT_2M	2100	1182	0.3789673	0	Singlet	Adipocyte
SAT_2M	4052	2170	6.5747353	4	Singlet	Immune_cells
SAT_2M	1010	638	1.9646365	4	Singlet	Immune_cells

SAT_2M	1120	732	4.1814947	1	Singlet	Immune_cells
SAT_2M	1493	935	9.6064043	4	Singlet	Immune_cells
SAT_2M	1045	704	0.095057	1	Singlet	Immune_cells
SAT_2M	1577	967	1.7056222	1	Singlet	Immune_cells
SAT_2M	1230	787	4.6925566	3	Singlet	FAPs
SAT_2M	1076	732	1.9319227	4	Singlet	Immune_cells
SAT_2M	1655	938	5.632115	1	Singlet	Immune_cells
SAT_2M	1201	808	0.8298755	2	Singlet	FAPs
SAT_2M	3353	1715	0.3861004	0	Singlet	Adipocyte
SAT_2M	1857	1001	4.0728832	0	Singlet	Adipocyte
SAT_2M	1699	995	5.9883721	1	Singlet	Immune_cells
SAT_2M	1801	1077	0.166021	0	Singlet	Adipocyte
SAT_2M	1995	1225	2.3511756	1	Singlet	Immune_cells
SAT_2M	1084	729	3.59447	5	Singlet	Immune_cells
SAT_2M	1145	768	0.3478261	3	Singlet	FAPs
SAT_2M	1715	1109	4.0023202	5	Singlet	Immune_cells
SAT_2M	1343	824	4.6062407	1	Singlet	Immune_cells
SAT_2M	2018	1282	0.3455084	5	Singlet	Immune_cells
SAT_2M	1519	890	10.512484	1	Singlet	Immune_cells
SAT_2M	1889	1102	4.9154334	9	Singlet	Mesothelial
SAT_2M	1048	677	0.8530806	0	Singlet	Adipocyte
SAT_2M	1118	745	5.5258467	1	Singlet	Immune_cells
SAT_2M	2144	1205	2.5605214	1	Singlet	Immune_cells
SAT_2M	1552	931	0.5118362	1	Singlet	Immune_cells
SAT_2M	4183	1734	0.9060563	10	Singlet	Immune_cells
SAT_2M	1439	968	0.2766252	4	Singlet	Immune_cells
SAT_2M	1178	740	6.6892464	1	Singlet	Immune_cells
SAT_2M	1772	1123	1.3498313	1	Singlet	Immune_cells
SAT_2M	1938	1240	2.4729521	8	Singlet	Mesothelial
SAT_2M	2215	1177	4.6292135	0	Singlet	Adipocyte
SAT_2M	2454	1432	0.2436054	8	Singlet	Mesothelial
SAT_2M	2624	1420	7.8787879	1	Singlet	Immune_cells
SAT_2M	1204	823	1.399177	1	Singlet	Immune_cells
SAT_2M	3385	1753	0.2946376	8	Singlet	Mesothelial
SAT_2M	1375	882	0.1451379	5	Singlet	Immune_cells
SAT_2M	1463	931	0.4067797	4	Singlet	Immune_cells
SAT_2M	1231	802	2.9102668	1	Singlet	Immune_cells
SAT_2M	3675	1751	0.1896505	8	Singlet	Mesothelial
SAT_2M	1435	895	0.3479471	0	Singlet	Adipocyte
SAT_2M	1476	941	4.1271989	0	Singlet	Adipocyte
SAT_2M	1463	963	1.0876954	1	Singlet	Immune_cells
SAT_2M	1976	1195	1.0568697	0	Singlet	Adipocyte
SAT_2M	1306	856	0.6880734	4	Singlet	Immune_cells
SAT_2M	1383	855	1.7291066	0	Singlet	Adipocyte
SAT_2M	1684	1019	8.7781732	9	Singlet	Mesothelial
SAT_2M	1248	775	0.3996803	1	Singlet	Immune_cells
SAT_2M	1839	1139	0.4867496	0	Singlet	Adipocyte
SAT_2M	4988	2171	1.8596281	10	Singlet	Immune_cells
SAT_2M	9045	2982	1.2571681	10	Singlet	Immune_cells
SAT_2M	1097	762	4.17802	1	Singlet	Immune_cells
SAT_2M	1334	862	2.2321429	1	Singlet	Immune_cells

SAT_2M	1269	858	0.6259781	4	Singlet	Immune_cells
SAT_2M	1777	1039	7.8991597	1	Singlet	Immune_cells
SAT_2M	1231	822	1.1326861	2	Singlet	FAPs
SAT_2M	2678	1382	4.7282204	0	Singlet	Adipocyte
SAT_2M	2278	1279	0.1313485	0	Singlet	Adipocyte
SAT_2M	2704	1428	3.8063562	3	Singlet	FAPs
SAT_2M	1235	812	5.7211926	1	Singlet	Immune_cells
SAT_2M	2449	1434	0.0813339	0	Singlet	Adipocyte
SAT_2M	1631	1025	0.3660769	0	Singlet	Adipocyte
SAT_2M	1314	817	6.4540623	1	Singlet	Immune_cells
SAT_2M	1276	773	4.4392523	1	Singlet	Immune_cells
SAT_2M	1133	767	0.9683099	0	Singlet	Adipocyte
SAT_2M	2377	1443	0.5035669	8	Singlet	Mesothelial
SAT_2M	1466	900	3.0674847	1	Singlet	Immune_cells
SAT_2M	1373	893	2.6143791	1	Singlet	Immune_cells
SAT_2M	1216	726	10.620915	1	Singlet	Immune_cells
SAT_2M	1014	702	1.6715831	1	Singlet	Immune_cells
SAT_2M	4412	1977	1.8325792	10	Singlet	Immune_cells
SAT_2M	1006	650	1.1904762	0	Singlet	Adipocyte
SAT_2M	2308	1315	1.9006479	3	Singlet	FAPs
SAT_2M	1456	889	7.0499658	1	Singlet	Immune_cells
SAT_2M	1024	679	1.1661808	0	Singlet	Adipocyte
SAT_2M	6579	3049	4.8284239	9	Singlet	Mesothelial
SAT_2M	1393	871	0.5	0	Singlet	Adipocyte
SAT_2M	2592	1416	0.5392912	7	Singlet	Endothelial_cells
SAT_2M	1114	737	0.1793722	1	Singlet	Immune_cells
SAT_2M	2572	1437	0.5791506	0	Singlet	Adipocyte
SAT_2M	1857	1072	0.3755365	0	Singlet	Adipocyte
SAT_2M	1509	923	2.7134348	1	Singlet	Immune_cells
SAT_2M	1052	669	2.173913	9	Singlet	Mesothelial
SAT_2M	3039	1493	1.6721311	10	Singlet	Immune_cells
SAT_2M	1325	856	2.8294862	4	Singlet	Immune_cells
SAT_2M	1126	696	0.1774623	4	Singlet	Immune_cells
SAT_2M	1239	779	8.8729017	1	Singlet	Immune_cells
SAT_2M	1878	1200	1.6419492	4	Singlet	Immune_cells
SAT_2M	1231	782	0.4038772	4	Singlet	Immune_cells
SAT_2M	1502	955	7.6462766	1	Singlet	Immune_cells
SAT_2M	2015	1174	3.8575668	4	Singlet	Immune_cells
SAT_2M	1372	864	7.5090253	1	Singlet	Immune_cells
SAT_2M	1254	784	8.5443038	1	Singlet	Immune_cells
SAT_2M	2768	1582	5.7553957	5	Singlet	Immune_cells
SAT_2M	2618	1431	1.0662605	5	Singlet	Immune_cells
SAT_2M	2538	1415	0.8235294	0	Singlet	Adipocyte
SAT_2M	2839	1292	5.7996485	5	Singlet	Immune_cells
SAT_2M	1959	1071	5.7084608	1	Singlet	Immune_cells
SAT_2M	1252	802	1.1119936	4	Singlet	Immune_cells
SAT_2M	2527	1504	6.855792	4	Singlet	Immune_cells
SAT_2M	1239	800	1.9354839	1	Singlet	Immune_cells
SAT_2M	1673	978	9.6908442	1	Singlet	Immune_cells
SAT_2M	1297	788	0.7698229	1	Singlet	Immune_cells
SAT_2M	2557	1227	2.9664325	10	Singlet	Immune_cells

SAT_2M	1682	1075	4.6745562	1	Singlet	Immune_cells
SAT_2M	1124	677	5.0353357	0	Singlet	Adipocyte
SAT_2M	1912	1145	0.4694836	0	Singlet	Adipocyte
SAT_2M	2032	1217	0.7846984	0	Singlet	Adipocyte
SAT_2M	1362	850	0.727802	0	Singlet	Adipocyte
SAT_2M	1696	1054	2.28739	0	Singlet	Adipocyte
SAT_2M	3117	1658	0.3187759	0	Singlet	Adipocyte
SAT_2M	1549	910	7.1702945	5	Singlet	Immune_cells
SAT_2M	1088	690	0.5484461	0	Singlet	Adipocyte
SAT_2M	2991	1695	0.2333333	5	Singlet	Immune_cells
SAT_2M	1237	812	0.5649718	7	Singlet	Endothelial_cells
SAT_2M	2335	1386	1.0660981	0	Singlet	Adipocyte
SAT_2M	1241	856	1.1235955	4	Singlet	Immune_cells
SAT_2M	1010	719	0.489716	5	Singlet	Immune_cells
SAT_2M	1182	742	1.1804384	0	Singlet	Adipocyte
SAT_2M	3042	1543	0.295082	3	Singlet	FAPs
SAT_2M	2587	1383	0.7310504	0	Singlet	Adipocyte
SAT_2M	1553	997	0.0640615	4	Singlet	Immune_cells
SAT_2M	1305	853	0.8409786	4	Singlet	Immune_cells
SAT_2M	2085	1171	0.9043313	0	Singlet	Adipocyte
SAT_2M	1229	799	4.6079224	1	Singlet	Immune_cells
SAT_2M	1756	1001	8.0225989	5	Singlet	Immune_cells
SAT_2M	2601	1430	8.3237438	1	Singlet	Immune_cells
SAT_2M	1504	845	12.524851	9	Singlet	Mesothelial
SAT_2M	1031	650	0.7722008	9	Singlet	Mesothelial
SAT_2M	1497	893	1.6600266	1	Singlet	Immune_cells
SAT_2M	3483	1703	2.5494128	0	Singlet	Adipocyte
SAT_2M	1987	1162	0.4518072	0	Singlet	Adipocyte
SAT_2M	1583	997	0.3761755	0	Singlet	Adipocyte
SAT_2M	1721	964	2.5507246	3	Singlet	FAPs
SAT_2M	3041	1349	0.9520683	10	Singlet	Immune_cells
SAT_2M	1308	883	3.196347	1	Singlet	Immune_cells
SAT_2M	1164	737	0.7718696	7	Singlet	Endothelial_cells
SAT_2M	1346	840	9.7037037	1	Singlet	Immune_cells
SAT_2M	2368	1271	0.1264223	0	Singlet	Adipocyte
SAT_2M	1418	826	2.5263158	1	Singlet	Immune_cells
SAT_2M	1249	825	0.6309148	6	Singlet	Neurons
SAT_2M	1239	774	3.6173633	1	Singlet	Immune_cells
SAT_2M	1767	1077	3.8961039	1	Singlet	Immune_cells
SAT_2M	1013	673	0.492126	5	Singlet	Immune_cells
SAT_2M	1372	855	0.4338395	4	Singlet	Immune_cells
SAT_2M	3336	1765	4.4179104	2	Singlet	FAPs
SAT_2M	1095	774	3.0520646	1	Singlet	Immune_cells
SAT_2M	2715	1636	3.5701141	8	Singlet	Mesothelial
SAT_2M	1428	946	0.8362369	4	Singlet	Immune_cells
SAT_2M	1248	802	0.8757962	1	Singlet	Immune_cells
SAT_2M	1176	854	0.8210181	6	Singlet	Neurons
SAT_2M	2007	1133	5.5278884	0	Singlet	Adipocyte
SAT_2M	1579	959	1.5180266	1	Singlet	Immune_cells
SAT_2M	2102	1329	1.5617605	4	Singlet	Immune_cells
SAT_2M	1965	1197	2.7918782	9	Singlet	Mesothelial

SAT_2M	1134	806	0.0881057	5	Singlet	Immune_cells
SAT_2M	1509	918	5.5884287	1	Singlet	Immune_cells
SAT_2M	1856	1090	5.8033315	1	Singlet	Immune_cells
SAT_2M	3154	1668	1.1646207	0	Singlet	Adipocyte
SAT_2M	1412	922	0.9797061	5	Singlet	Immune_cells
SAT_2M	4699	2229	6.8621202	9	Singlet	Mesothelial
SAT_2M	1310	818	2.0532319	4	Singlet	Immune_cells
SAT_2M	1027	745	0.5780347	4	Singlet	Immune_cells
SAT_2M	4728	2175	5.2443134	2	Singlet	FAPs
SAT_2M	1262	822	1.7309205	1	Singlet	Immune_cells
SAT_2M	6925	2512	1.7564066	10	Singlet	Immune_cells
SAT_2M	2925	1564	0.2037351	0	Singlet	Adipocyte
SAT_2M	1935	1125	7.9299691	1	Singlet	Immune_cells
SAT_2M	1024	678	0.8789063	5	Singlet	Immune_cells
SAT_2M	1437	880	2.2853186	0	Singlet	Adipocyte
SAT_2M	2521	1440	1.1890606	0	Singlet	Adipocyte
SAT_2M	1306	758	7.0068545	1	Singlet	Immune_cells
SAT_2M	1394	840	5.003574	1	Singlet	Immune_cells
SAT_2M	2613	1539	0.1526135	8	Singlet	Mesothelial
SAT_2M	1531	948	0.5867014	4	Singlet	Immune_cells
SAT_2M	1863	1052	1.0712373	0	Singlet	Adipocyte
SAT_2M	1296	782	0.231303	4	Singlet	Immune_cells
SAT_2M	1323	867	1.509434	4	Singlet	Immune_cells
SAT_2M	2118	1154	4.8493409	1	Singlet	Immune_cells
SAT_2M	1217	806	0.3270646	1	Singlet	Immune_cells
SAT_2M	2404	1265	1.038206	0	Singlet	Adipocyte
SAT_2M	1669	878	12.874252	9	Singlet	Mesothelial
SAT_2M	1538	977	2.3316062	5	Singlet	Immune_cells
SAT_2M	2145	1334	0.7834101	4	Singlet	Immune_cells
SAT_2M	2662	1456	0.5615874	0	Singlet	Adipocyte
SAT_2M	1902	1110	8.7526205	1	Singlet	Immune_cells
SAT_2M	1217	765	1.2305168	3	Singlet	FAPs
SAT_2M	1176	791	1.4358108	1	Singlet	Immune_cells
SAT_2M	1549	966	0.4513217	1	Singlet	Immune_cells
SAT_2M	3832	1937	10.09653	9	Singlet	Mesothelial
SAT_2M	1370	812	9.1836735	1	Singlet	Immune_cells
SAT_2M	1030	675	1.4520813	5	Singlet	Immune_cells
SAT_2M	2223	1371	0	5	Singlet	Immune_cells
SAT_2M	1451	889	5.4183813	4	Singlet	Immune_cells
SAT_2M	2535	1394	1.3752456	3	Singlet	FAPs
SAT_2M	2005	1140	2.4390244	1	Singlet	Immune_cells
SAT_2M	2118	1244	2.9190207	1	Singlet	Immune_cells
SAT_2M	1785	1014	6.6443328	4	Singlet	Immune_cells
SAT_2M	1651	998	3.3836858	1	Singlet	Immune_cells
SAT_2M	2010	1126	0.2483855	0	Singlet	Adipocyte
SAT_2M	1280	843	1.0911925	4	Singlet	Immune_cells
SAT_2M	1476	936	0.5398111	1	Singlet	Immune_cells
SAT_2M	1960	1132	4.1158537	1	Singlet	Immune_cells
SAT_2M	3054	1597	0.3583062	0	Singlet	Adipocyte
SAT_2M	1255	782	2.9968454	1	Singlet	Immune_cells
SAT_2M	1532	938	2.0833333	1	Singlet	Immune_cells

SAT_2M	1161	744	2.3156089	5	Singlet	Immune_cells
SAT_2M	1825	1027	1.474604	10	Singlet	Immune_cells
SAT_2M	1258	791	1.5079365	1	Singlet	Immune_cells
SAT_2M	1630	1031	0.244798	3	Singlet	FAPs
SAT_2M	1443	839	4.7487956	2	Singlet	FAPs
SAT_2M	1825	1086	3.7704918	1	Singlet	Immune_cells
SAT_2M	1317	828	2.5679758	4	Singlet	Immune_cells
SAT_2M	1411	909	0.4189944	0	Singlet	Adipocyte
SAT_2M	1097	738	2.8105168	7	Singlet	Endothelial_cells
SAT_2M	1987	1198	2.1042084	1	Singlet	Immune_cells
SAT_2M	2234	1354	0.4474273	11	Singlet	Smooth_muscle_cells
SAT_2M	5334	2449	6.4008984	9	Singlet	Mesothelial
SAT_2M	1514	861	4.5335085	10	Singlet	Immune_cells
SAT_2M	1088	732	1.8298262	4	Singlet	Immune_cells
SAT_2M	1090	708	0.8174387	4	Singlet	Immune_cells
SAT_2M	1718	1016	0.5803831	1	Singlet	Immune_cells
SAT_2M	1399	797	8.4939329	1	Singlet	Immune_cells
SAT_2M	1192	739	12.541806	5	Singlet	Immune_cells
SAT_2M	3726	1842	0.294275	0	Singlet	Adipocyte
SAT_2M	3489	1770	0.1430615	0	Singlet	Adipocyte
SAT_2M	1971	1105	1.5688259	1	Singlet	Immune_cells
SAT_2M	1112	717	10.286225	1	Singlet	Immune_cells
SAT_2M	1005	694	0.3944773	4	Singlet	Immune_cells
SAT_2M	1068	714	2.334267	1	Singlet	Immune_cells
SAT_2M	1159	729	0.5977797	0	Singlet	Adipocyte
SAT_2M	2147	1249	1.0175763	0	Singlet	Adipocyte
SAT_2M	1646	1037	5.1359517	1	Singlet	Immune_cells
SAT_2M	2523	1419	0.2761341	5	Singlet	Immune_cells
SAT_2M	1478	1012	1.3449899	4	Singlet	Immune_cells
SAT_2M	1225	818	6.1788618	1	Singlet	Immune_cells
SAT_2M	1797	1126	0.3320421	3	Singlet	FAPs
SAT_2M	1729	1010	2.4137931	3	Singlet	FAPs
SAT_2M	1831	1122	0.1629549	3	Singlet	FAPs
SAT_2M	1883	1150	2.0634921	1	Singlet	Immune_cells
SAT_2M	2417	1315	1.0699588	1	Singlet	Immune_cells
SAT_2M	2107	1171	4.0265277	1	Singlet	Immune_cells
SAT_2M	1546	901	1.674179	10	Singlet	Immune_cells
SAT_2M	1609	1052	0.6798517	4	Singlet	Immune_cells
SAT_2M	1253	772	8.9171975	1	Singlet	Immune_cells
SAT_2M	1282	767	5.7275542	1	Singlet	Immune_cells
SAT_2M	1277	769	7.788162	4	Singlet	Immune_cells
SAT_2M	1639	960	9.787234	4	Singlet	Immune_cells
SAT_2M	1269	778	1.0172144	0	Singlet	Adipocyte
SAT_2M	1415	900	0.2807018	4	Singlet	Immune_cells
SAT_2M	1609	1009	0.0619963	8	Singlet	Mesothelial
SAT_2M	1919	1117	8.4155844	4	Singlet	Immune_cells
SAT_2M	1781	1096	4.4667783	5	Singlet	Immune_cells
SAT_2M	1584	1056	0.1255493	4	Singlet	Immune_cells
SAT_2M	1892	1166	0.1578117	0	Singlet	Adipocyte
SAT_2M	1109	715	0.990099	5	Singlet	Immune_cells
SAT_2M	1064	706	0	0	Singlet	Adipocyte

SAT_2M	1359	779	9.3567251	1	Singlet	Immune_cells
SAT_2M	1483	947	0.8091706	4	Singlet	Immune_cells
SAT_2M	1505	905	1.6556291	7	Singlet	Endothelial_cells
SAT_2M	1620	900	13.131936	1	Singlet	Immune_cells
SAT_2M	2304	1338	3.9775184	3	Singlet	FAPs
SAT_2M	2439	1271	0.7352941	0	Singlet	Adipocyte
SAT_2M	1792	1082	0.2212389	0	Singlet	Adipocyte
SAT_2M	1381	951	0.9386282	4	Singlet	Immune_cells
SAT_2M	2025	1125	6.5356265	1	Singlet	Immune_cells
SAT_2M	1391	774	8.2437276	0	Singlet	Adipocyte
SAT_2M	1347	793	10.682493	1	Singlet	Immune_cells
SAT_2M	1945	1128	6.2980031	4	Singlet	Immune_cells
SAT_2M	2961	1508	0.1686341	0	Singlet	Adipocyte
SAT_2M	2320	1322	6.048906	5	Singlet	Immune_cells
SAT_2M	1134	773	1.2334802	4	Singlet	Immune_cells
SAT_2M	2195	1174	0.2723559	0	Singlet	Adipocyte
SAT_2M	1406	886	1.2031139	0	Singlet	Adipocyte
SAT_2M	2008	1100	2.0347395	5	Singlet	Immune_cells
SAT_2M	1294	752	9.4208494	1	Singlet	Immune_cells
SAT_2M	1336	841	7.0789866	1	Singlet	Immune_cells
SAT_2M	1705	1005	4.4950379	5	Singlet	Immune_cells
SAT_2M	1031	709	4.0501446	3	Singlet	FAPs
SAT_2M	1930	1167	0.4130098	0	Singlet	Adipocyte
SAT_2M	1299	846	4.765565	1	Singlet	Immune_cells
SAT_2M	1491	877	0.8026756	0	Singlet	Adipocyte
SAT_2M	1223	813	4	4	Singlet	Immune_cells
SAT_2M	6181	2172	1.3565891	10	Singlet	Immune_cells
SAT_2M	1295	830	0.4604758	1	Singlet	Immune_cells
SAT_2M	1506	919	4.7682119	1	Singlet	Immune_cells
SAT_2M	1375	889	0.1440922	0	Singlet	Adipocyte
SAT_2M	1145	757	0.0867303	1	Singlet	Immune_cells
SAT_2M	1303	856	0.1525553	8	Singlet	Mesothelial
SAT_2M	1780	1017	8.4404695	1	Singlet	Immune_cells
SAT_2M	1284	815	3.7267081	1	Singlet	Immune_cells
SAT_2M	2340	1368	0.383959	11	Singlet	Smooth_muscle_cells
SAT_2M	1003	674	0.1980198	0	Singlet	Adipocyte
SAT_2M	2129	1229	11.261682	9	Singlet	Mesothelial
SAT_2M	1193	789	0.5	1	Singlet	Immune_cells
SAT_2M	1087	737	3.3913841	4	Singlet	Immune_cells
SAT_2M	1199	777	2.7385892	3	Singlet	FAPs
SAT_2M	1125	728	1.2378426	3	Singlet	FAPs
SAT_2M	1297	813	0.2307692	4	Singlet	Immune_cells
SAT_2M	1807	1057	0.6615215	0	Singlet	Adipocyte
SAT_2M	1166	786	2.9838022	1	Singlet	Immune_cells
SAT_2M	1773	1056	5.3872054	1	Singlet	Immune_cells
SAT_2M	2148	1226	1.0697674	10	Singlet	Immune_cells
SAT_2M	1295	814	3.3742331	1	Singlet	Immune_cells
SAT_2M	1272	824	5.7632399	4	Singlet	Immune_cells
SAT_2M	1343	787	12.509252	1	Singlet	Immune_cells
SAT_2M	1289	804	0.7716049	4	Singlet	Immune_cells
SAT_2M	1056	689	7.6850984	1	Singlet	Immune_cells

SAT_2M	1178	745	0.0844595	1	Singlet	Immune_cells
SAT_2M	3300	1792	5.5186972	5	Singlet	Immune_cells
SAT_2M	2938	1601	8.5142469	4	Singlet	Immune_cells
SAT_2M	1559	967	0.1914486	4	Singlet	Immune_cells
SAT_2M	1426	971	0.209351	7	Singlet	Endothelial_cells
SAT_2M	1706	994	1.7554125	4	Singlet	Immune_cells
SAT_2M	2820	1493	0.0352983	0	Singlet	Adipocyte
SAT_2M	1069	691	0.5591799	3	Singlet	FAPs
SAT_2M	1677	972	5.9630292	1	Singlet	Immune_cells
SAT_2M	1674	1040	0.4753417	0	Singlet	Adipocyte
SAT_2M	1655	1054	1.9173158	1	Singlet	Immune_cells
SAT_2M	3432	1724	9.0724048	9	Singlet	Mesothelial
SAT_2M	1589	950	0.3757044	0	Singlet	Adipocyte
SAT_2M	1669	1070	0.8338297	5	Singlet	Immune_cells
SAT_2M	1174	767	0.2533784	4	Singlet	Immune_cells
SAT_2M	1100	714	0.0908265	1	Singlet	Immune_cells
SAT_2M	1833	1074	0.2162162	0	Singlet	Adipocyte
SAT_2M	1508	874	5.5555556	1	Singlet	Immune_cells
SAT_2M	1397	887	1.2108262	4	Singlet	Immune_cells
SAT_2M	3284	1630	0.2425713	0	Singlet	Adipocyte
SAT_2M	1902	1121	5.987395	1	Singlet	Immune_cells
SAT_2M	1349	855	1.0294118	6	Singlet	Neurons
SAT_2M	1693	963	10.67217	1	Singlet	Immune_cells
SAT_2M	1042	697	2.6819923	1	Singlet	Immune_cells
SAT_2M	1317	806	2.5583145	1	Singlet	Immune_cells
SAT_2M	1599	995	0.4361371	3	Singlet	FAPs
SAT_2M	1221	793	2.7777778	9	Singlet	Mesothelial
SAT_2M	1247	791	0.3187251	4	Singlet	Immune_cells
SAT_2M	3859	1871	3.0232558	0	Singlet	Adipocyte
SAT_2M	2609	1433	3.2146958	4	Singlet	Immune_cells
SAT_2M	1092	750	0.6398537	4	Singlet	Immune_cells
SAT_2M	1347	854	4.9926579	1	Singlet	Immune_cells
SAT_2M	1451	840	7.3287671	1	Singlet	Immune_cells
SAT_2M	3309	1577	2.5339367	3	Singlet	FAPs
SAT_2M	5207	2360	6.7725273	9	Singlet	Mesothelial
SAT_2M	1352	827	0.7374631	0	Singlet	Adipocyte
SAT_2M	2431	1391	0.4498978	0	Singlet	Adipocyte
SAT_2M	4498	2180	1.7559458	9	Singlet	Mesothelial
SAT_2M	1394	906	4.5681656	1	Singlet	Immune_cells
SAT_2M	1239	790	0.4830918	4	Singlet	Immune_cells
SAT_2M	1231	770	2.6699029	2	Singlet	FAPs
SAT_2M	2973	1576	0.6688963	0	Singlet	Adipocyte
SAT_2M	6871	2883	4.6920395	9	Singlet	Mesothelial
SAT_2M	1031	732	0.3868472	4	Singlet	Immune_cells
SAT_2M	3126	1589	0.7019783	0	Singlet	Adipocyte
SAT_2M	3878	2046	0.3581479	9	Singlet	Mesothelial
SAT_2M	1650	1044	0.6027728	8	Singlet	Mesothelial
SAT_2M	1273	808	1.1755486	1	Singlet	Immune_cells
SAT_2M	1126	739	0.4413063	4	Singlet	Immune_cells
SAT_2M	1507	910	1.9205298	4	Singlet	Immune_cells
SAT_2M	1820	1113	3.2876712	8	Singlet	Mesothelial

SAT_2M	1428	926	0.2084781	2	Singlet	FAPs
SAT_2M	1240	865	0.32	0	Singlet	Adipocyte
SAT_2M	1217	826	0.5700326	5	Singlet	Immune_cells
SAT_2M	3329	1706	0.1796945	8	Singlet	Mesothelial
SAT_2M	1351	848	0.2177068	0	Singlet	Adipocyte
SAT_2M	1968	1139	3.4954407	1	Singlet	Immune_cells
SAT_2M	1502	917	2.3793787	1	Singlet	Immune_cells
SAT_2M	1415	949	0.5625879	4	Singlet	Immune_cells
SAT_2M	2581	1454	0.1539646	0	Singlet	Adipocyte
SAT_2M	1219	774	0.6525285	1	Singlet	Immune_cells
SAT_2M	1204	752	1.3266998	0	Singlet	Adipocyte
SAT_2M	1284	810	1.4717273	0	Singlet	Adipocyte
SAT_2M	2044	1176	5.8479532	1	Singlet	Immune_cells
SAT_2M	1406	857	4.7282992	5	Singlet	Immune_cells
SAT_2M	1622	1040	1.8971848	9	Singlet	Mesothelial
SAT_2M	1581	1000	2.5769956	1	Singlet	Immune_cells
SAT_2M	1537	950	7.3882048	4	Singlet	Immune_cells
SAT_2M	1762	1041	4.1406693	1	Singlet	Immune_cells
SAT_2M	1307	906	2.1276596	1	Singlet	Immune_cells
SAT_2M	1012	691	0.2952756	3	Singlet	FAPs
SAT_2M	1832	1081	3.8147139	1	Singlet	Immune_cells
SAT_2M	1066	720	2.0579981	4	Singlet	Immune_cells
SAT_2M	1463	885	4.8233696	4	Singlet	Immune_cells
SAT_2M	1113	701	0.3571429	2	Singlet	FAPs
SAT_2M	1375	879	0.1448226	1	Singlet	Immune_cells
SAT_2M	1017	679	1.2720157	5	Singlet	Immune_cells
SAT_2M	1435	883	0.4861111	1	Singlet	Immune_cells
SAT_2M	1039	758	0.7611798	1	Singlet	Immune_cells
SAT_2M	2163	1174	6.7188219	0	Singlet	Adipocyte
SAT_2M	2307	1235	2.5108225	0	Singlet	Adipocyte
SAT_2M	1849	1190	3.0645161	4	Singlet	Immune_cells
SAT_2M	1210	753	2.7138158	4	Singlet	Immune_cells
SAT_2M	1297	811	0.3843198	1	Singlet	Immune_cells
SAT_2M	1291	870	0.1544402	4	Singlet	Immune_cells
SAT_2M	1443	875	1.3793103	4	Singlet	Immune_cells
SAT_2M	1032	643	5.3191489	2	Singlet	FAPs
SAT_2M	3145	1661	1.2330066	0	Singlet	Adipocyte
SAT_2M	1856	1142	4.3571813	1	Singlet	Immune_cells
SAT_2M	1140	755	1.6608392	1	Singlet	Immune_cells
SAT_2M	1731	1037	3.4005764	0	Singlet	Adipocyte
SAT_2M	1012	594	2.2571148	10	Singlet	Immune_cells
SAT_2M	2017	1239	0.148075	8	Singlet	Mesothelial
SAT_2M	1469	882	10.794297	1	Singlet	Immune_cells
SAT_2M	1333	881	0.2998501	1	Singlet	Immune_cells
SAT_2M	1482	954	0.4024145	4	Singlet	Immune_cells
SAT_2M	1444	892	0.7596685	0	Singlet	Adipocyte
SAT_2M	1655	990	0.1806141	2	Singlet	FAPs
SAT_2M	3583	1820	0.9460211	0	Singlet	Adipocyte
SAT_2M	1664	1019	0.7751938	0	Singlet	Adipocyte
SAT_2M	1924	1113	2.8940568	1	Singlet	Immune_cells
SAT_2M	1320	776	2.5738077	0	Singlet	Adipocyte

SAT_2M	1880	1120	0.4253057	4	Singlet	Immune_cells
SAT_2M	3385	1709	0.5306604	3	Singlet	FAPs
SAT_2M	1393	861	9.5204009	1	Singlet	Immune_cells
SAT_2M	2937	1437	12.317115	9	Singlet	Mesothelial
SAT_2M	2104	1267	2.1306818	4	Singlet	Immune_cells
SAT_2M	1358	835	1.3225569	0	Singlet	Adipocyte
SAT_2M	1237	793	1.1226945	1	Singlet	Immune_cells
SAT_2M	1040	677	1.1472275	1	Singlet	Immune_cells
SAT_2M	4425	1750	1.9183029	10	Singlet	Immune_cells
SAT_2M	1608	1034	1.8610422	1	Singlet	Immune_cells
SAT_2M	1627	1011	2.7658267	4	Singlet	Immune_cells
SAT_2M	1647	971	0.7874016	0	Singlet	Adipocyte
SAT_2M	1659	1049	0.2402402	4	Singlet	Immune_cells
SAT_2M	1245	788	0.7171315	4	Singlet	Immune_cells
SAT_2M	1418	909	1.2658228	4	Singlet	Immune_cells
SAT_2M	1662	1021	0.9580838	4	Singlet	Immune_cells
SAT_2M	2625	1483	0.5311077	0	Singlet	Adipocyte
SAT_2M	1400	898	0.9957326	4	Singlet	Immune_cells
SAT_2M	1279	853	1.5588465	11	Singlet	Smooth_muscle_cells
SAT_2M	1555	980	4.4117647	1	Singlet	Immune_cells
SAT_2M	1347	777	0.1477105	1	Singlet	Immune_cells
SAT_2M	2303	1281	2.5917927	10	Singlet	Immune_cells
SAT_2M	2455	1268	2.1553477	0	Singlet	Adipocyte
SAT_2M	1647	996	1.0253317	1	Singlet	Immune_cells
SAT_2M	2196	1289	2.8959276	1	Singlet	Immune_cells
SAT_2M	2200	1231	7.1169538	1	Singlet	Immune_cells
SAT_2M	1242	853	0.2392345	4	Singlet	Immune_cells
SAT_2M	3457	1844	0.547235	11	Singlet	Smooth_muscle_cells
SAT_2M	1086	732	5.1771117	1	Singlet	Immune_cells
SAT_2M	1266	798	0.4720692	1	Singlet	Immune_cells
SAT_2M	1442	921	0.2061856	1	Singlet	Immune_cells
SAT_2M	1388	920	1.2160229	4	Singlet	Immune_cells
SAT_2M	1636	1040	3.6407767	1	Singlet	Immune_cells
SAT_2M	2205	1229	0.1810774	0	Singlet	Adipocyte
SAT_2M	1767	1004	2.1934758	0	Singlet	Adipocyte
SAT_2M	2766	1569	8.9305005	4	Singlet	Immune_cells
SAT_2M	1325	839	5.7938299	1	Singlet	Immune_cells
SAT_2M	1245	780	0.5608974	0	Singlet	Adipocyte
SAT_2M	7694	3262	12.18531	9	Singlet	Mesothelial
SAT_2M	1421	877	2.6499303	1	Singlet	Immune_cells
SAT_2M	1236	772	4.0355125	1	Singlet	Immune_cells
SAT_2M	1213	787	1.7170891	9	Singlet	Mesothelial
SAT_2M	1070	707	1.5668203	1	Singlet	Immune_cells
SAT_2M	1288	875	1.2355212	1	Singlet	Immune_cells
SAT_2M	1499	979	2.2546419	9	Singlet	Mesothelial
SAT_2M	1506	868	5.2840159	1	Singlet	Immune_cells
SAT_2M	1157	739	1.9742489	5	Singlet	Immune_cells
SAT_2M	1113	721	3.9320822	1	Singlet	Immune_cells
SAT_2M	1952	1030	4.7546012	1	Singlet	Immune_cells
SAT_2M	1810	1026	0.5506608	0	Singlet	Adipocyte
SAT_2M	2303	1349	0.0866176	3	Singlet	FAPs

SAT_2M	1637	944	1.459854	0	Singlet	Adipocyte
SAT_2M	3358	1869	1.0989011	9	Singlet	Mesothelial
SAT_2M	1307	850	2.1292776	11	Singlet	Smooth_muscle_cells
SAT_2M	1975	1190	2.7300303	1	Singlet	Immune_cells
SAT_2M	1686	1035	2.4778761	1	Singlet	Immune_cells
SAT_2M	1833	1095	0.1078167	0	Singlet	Adipocyte
SAT_2M	1006	696	0.9803922	6	Singlet	Neurons
SAT_2M	4640	2035	0.8165019	0	Singlet	Adipocyte
SAT_2M	1018	671	0.2938296	1	Singlet	Immune_cells
SAT_2M	2790	1452	0.8950949	0	Singlet	Adipocyte
SAT_2M	1019	669	0.8789063	0	Singlet	Adipocyte
SAT_2M	1197	810	2.0678246	1	Singlet	Immune_cells
SAT_2M	3824	1869	4.6226169	9	Singlet	Mesothelial
SAT_2M	1449	813	2.4725275	0	Singlet	Adipocyte
SAT_2M	1960	1083	11.788618	4	Singlet	Immune_cells
SAT_2M	1159	726	8.5197935	5	Singlet	Immune_cells
SAT_2M	1867	1138	3.4777956	1	Singlet	Immune_cells
SAT_2M	1299	811	0.690184	4	Singlet	Immune_cells
SAT_2M	2323	1257	5.9151307	1	Singlet	Immune_cells
SAT_2M	3348	1885	0.5970149	5	Singlet	Immune_cells
SAT_2M	5069	2313	0.8451258	2	Singlet	FAPs
SAT_2M	1086	726	0.2744739	0	Singlet	Adipocyte
SAT_2M	1106	677	5.7502246	10	Singlet	Immune_cells
SAT_2M	1420	886	0.5594406	1	Singlet	Immune_cells
SAT_2M	1090	752	0.7319305	1	Singlet	Immune_cells
SAT_2M	1324	898	0.2996255	4	Singlet	Immune_cells
SAT_2M	1217	738	0.1642036	0	Singlet	Adipocyte
SAT_2M	1786	1089	5.4038997	1	Singlet	Immune_cells
SAT_2M	1281	820	3.7267081	1	Singlet	Immune_cells
SAT_2M	1535	902	11.860013	4	Singlet	Immune_cells
SAT_2M	2545	1414	3.7211124	5	Singlet	Immune_cells
SAT_2M	1723	1062	5.5523424	1	Singlet	Immune_cells
SAT_2M	1270	852	0.2351097	1	Singlet	Immune_cells
SAT_2M	1242	808	2.4859663	1	Singlet	Immune_cells
SAT_2M	1484	873	0.134499	0	Singlet	Adipocyte
SAT_2M	8726	2692	1.9784995	10	Singlet	Immune_cells
SAT_2M	1427	895	0.2777778	0	Singlet	Adipocyte
SAT_2M	1223	794	0.0814996	4	Singlet	Immune_cells
SAT_2M	2090	1207	2.9957204	0	Singlet	Adipocyte
SAT_2M	2237	1277	9.6888889	9	Singlet	Mesothelial
SAT_2M	1767	1049	7.9706049	1	Singlet	Immune_cells
SAT_2M	3473	1739	0.3156385	0	Singlet	Adipocyte
SAT_2M	1190	763	3.2554257	3	Singlet	FAPs
SAT_2M	1248	768	4.24	1	Singlet	Immune_cells
SAT_2M	1863	1070	4.4919786	1	Singlet	Immune_cells
SAT_2M	5930	2763	5.8229552	9	Singlet	Mesothelial
SAT_2M	2160	1256	3.0009234	1	Singlet	Immune_cells
SAT_2M	2460	1335	1.4592623	0	Singlet	Adipocyte
SAT_2M	2077	1137	2.401537	0	Singlet	Adipocyte
SAT_2M	2489	1543	4.2433947	4	Singlet	Immune_cells
SAT_2M	1412	942	0.5649718	4	Singlet	Immune_cells

SAT_2M	3097	1524	1.8333869	10	Singlet	Immune_cells
SAT_2M	1056	727	1.2275732	4	Singlet	Immune_cells
SAT_2M	1083	714	0.3686636	4	Singlet	Immune_cells
SAT_2M	1263	735	5.6872038	1	Singlet	Immune_cells
SAT_2M	1779	1056	4.6498599	1	Singlet	Immune_cells
SAT_2M	1628	989	2.0158827	1	Singlet	Immune_cells
SAT_2M	1694	1023	0.5896226	4	Singlet	Immune_cells
SAT_2M	1003	674	0	4	Singlet	Immune_cells
SAT_2M	1220	800	5.4693878	1	Singlet	Immune_cells
SAT_2M	1677	1063	2.4390244	4	Singlet	Immune_cells
SAT_2M	1612	960	1.8552876	1	Singlet	Immune_cells
SAT_2M	3127	1570	0.1909004	0	Singlet	Adipocyte
SAT_2M	1368	853	1.9536903	0	Singlet	Adipocyte
SAT_2M	1286	864	0.3093581	5	Singlet	Immune_cells
SAT_2M	15519	5208	9.6346797	9	Singlet	Mesothelial
SAT_2M	2864	1461	10.19485	4	Singlet	Immune_cells
SAT_2M	1490	931	1.9385027	4	Singlet	Immune_cells
SAT_2M	1837	1180	0.3252033	0	Singlet	Adipocyte
SAT_2M	1285	819	1.0054138	0	Singlet	Adipocyte
SAT_2M	1496	951	0.9271523	0	Singlet	Adipocyte
SAT_2M	1694	1089	0.1763668	5	Singlet	Immune_cells
SAT_2M	1212	803	0.9009009	5	Singlet	Immune_cells
SAT_2M	1238	770	0.7246377	1	Singlet	Immune_cells
SAT_2M	1244	795	3.6888532	1	Singlet	Immune_cells
SAT_2M	1259	783	0.8675079	0	Singlet	Adipocyte
SAT_2M	1075	735	0.462963	4	Singlet	Immune_cells
SAT_2M	1068	706	0.1865672	4	Singlet	Immune_cells
SAT_2M	1583	935	4.3588124	8	Singlet	Mesothelial
SAT_2M	2336	1313	1.4925373	0	Singlet	Adipocyte
SAT_2M	1136	722	5.7591623	1	Singlet	Immune_cells
SAT_2M	1309	838	0.3810976	5	Singlet	Immune_cells
SAT_2M	1493	977	1.2040134	4	Singlet	Immune_cells
SAT_2M	1201	785	10.13289	1	Singlet	Immune_cells
SAT_2M	1417	886	0.4225352	1	Singlet	Immune_cells
SAT_2M	1702	1079	0.234192	4	Singlet	Immune_cells
SAT_2M	3619	1754	4.1563446	1	Singlet	Immune_cells
SAT_2M	1035	703	0.7707129	4	Singlet	Immune_cells
SAT_2M	1783	1098	0.5030743	4	Singlet	Immune_cells
SAT_2M	1459	911	8.1395349	1	Singlet	Immune_cells
SAT_2M	1796	1003	7.8420467	1	Singlet	Immune_cells
SAT_2M	1085	730	2.8233151	4	Singlet	Immune_cells
SAT_2M	3600	1712	4.8766971	10	Singlet	Immune_cells
SAT_2M	1182	762	2.10615	4	Singlet	Immune_cells
SAT_2M	1634	987	8.7697929	1	Singlet	Immune_cells
SAT_2M	1409	844	6.3165366	1	Singlet	Immune_cells
SAT_2M	1743	1061	1.6561965	5	Singlet	Immune_cells
SAT_2M	1763	1067	1.1293055	0	Singlet	Adipocyte
SAT_2M	1826	1149	0.3825137	9	Singlet	Mesothelial
SAT_2M	1319	777	4.4663134	1	Singlet	Immune_cells
SAT_2M	1261	844	2.8391167	4	Singlet	Immune_cells
SAT_2M	1183	778	0.084317	4	Singlet	Immune_cells

SAT_2M	1827	1064	8.4514722	4	Singlet	Immune_cells
SAT_2M	1466	831	7.0556309	1	Singlet	Immune_cells
SAT_2M	8231	2445	2.0990051	10	Singlet	Immune_cells
SAT_2M	1979	1142	6.3476071	1	Singlet	Immune_cells
SAT_2M	1165	759	0.3412969	0	Singlet	Adipocyte
SAT_2M	2045	1165	6.7284252	4	Singlet	Immune_cells
SAT_2M	2326	1294	3.2175032	0	Singlet	Adipocyte
SAT_2M	1192	763	0.2502085	1	Singlet	Immune_cells
SAT_2M	1815	1062	0.219901	3	Singlet	FAPs
SAT_2M	1392	881	3.8020086	3	Singlet	FAPs
SAT_2M	1825	1079	0.4352557	0	Singlet	Adipocyte
SAT_2M	4144	1828	1.9937545	10	Singlet	Immune_cells
SAT_2M	3717	1766	0.993022	0	Singlet	Adipocyte
SAT_2M	1429	933	0.4884857	0	Singlet	Adipocyte
SAT_2M	1057	650	12.45283	9	Singlet	Mesothelial
SAT_2M	1309	788	1.0678871	4	Singlet	Immune_cells
SAT_2M	1157	751	0.0859845	1	Singlet	Immune_cells
SAT_2M	1400	894	7.8947368	1	Singlet	Immune_cells
SAT_2M	1234	784	5.4119548	4	Singlet	Immune_cells
SAT_2M	1263	767	7.9527559	1	Singlet	Immune_cells
SAT_2M	1105	732	8.4532374	1	Singlet	Immune_cells
SAT_2M	1025	652	1.2670565	0	Singlet	Adipocyte
SAT_2M	1962	1160	0.8109478	0	Singlet	Adipocyte
SAT_2M	2128	1230	0.8415147	0	Singlet	Adipocyte
SAT_2M	1793	1105	2.729805	4	Singlet	Immune_cells
SAT_2M	1596	850	5.6320401	9	Singlet	Mesothelial
SAT_2M	1282	823	3.0373832	1	Singlet	Immune_cells
SAT_2M	1394	885	0.3563792	4	Singlet	Immune_cells
SAT_2M	1558	923	2.1766965	1	Singlet	Immune_cells
SAT_2M	1007	685	1.7786561	8	Singlet	Mesothelial
SAT_2M	1253	795	4.0669856	4	Singlet	Immune_cells
SAT_2M	1105	723	1.0820559	5	Singlet	Immune_cells
SAT_2M	1256	824	0.1582278	5	Singlet	Immune_cells
SAT_2M	3793	1914	1.7100763	9	Singlet	Mesothelial
SAT_2M	1391	885	3.0692363	1	Singlet	Immune_cells
SAT_2M	1695	1013	3.4685479	5	Singlet	Immune_cells
SAT_2M	1335	840	0.6726457	0	Singlet	Adipocyte
SAT_2M	1246	843	1.9857029	1	Singlet	Immune_cells
SAT_2M	1726	1010	1.0368664	3	Singlet	FAPs
SAT_2M	1422	1053	1.0238908	6	Singlet	Neurons
SAT_2M	1606	1032	0.1863354	5	Singlet	Immune_cells
SAT_2M	1247	797	0.6389776	0	Singlet	Adipocyte
SAT_2M	1361	781	0.8810573	1	Singlet	Immune_cells
SAT_2M	1095	702	0.8211679	10	Singlet	Immune_cells
SAT_2M	1785	1069	1.8404908	0	Singlet	Adipocyte
SAT_2M	1222	772	1.9543974	1	Singlet	Immune_cells
SAT_2M	1341	861	0.296077	1	Singlet	Immune_cells
SAT_2M	1016	649	0.686948	1	Singlet	Immune_cells
SAT_2M	1404	882	4.9715909	1	Singlet	Immune_cells
SAT_2M	1287	856	2.7131783	4	Singlet	Immune_cells
SAT_2M	1646	973	0.4244997	1	Singlet	Immune_cells

SAT_2M	1730	1060	2.6528258	3	Singlet	FAPs
SAT_2M	2884	1477	1.8307427	3	Singlet	FAPs
SAT_2M	1719	1052	0.4062681	4	Singlet	Immune_cells
SAT_2M	6012	2846	3.1068284	9	Singlet	Mesothelial
SAT_2M	1095	801	0.2659574	4	Singlet	Immune_cells
SAT_2M	1100	719	0.1813237	4	Singlet	Immune_cells
SAT_2M	1151	731	2.4242424	0	Singlet	Adipocyte
SAT_2M	1004	624	0.29615	4	Singlet	Immune_cells
SAT_2M	1116	701	0.6233304	1	Singlet	Immune_cells
SAT_2M	2523	1422	0.4748714	3	Singlet	FAPs
SAT_2M	1325	763	1.3544018	10	Singlet	Immune_cells
SAT_2M	1293	848	4.3143297	1	Singlet	Immune_cells
SAT_2M	1087	709	1.3686131	4	Singlet	Immune_cells
SAT_2M	1306	877	1.9832189	4	Singlet	Immune_cells
SAT_2M	1053	723	0.5617978	1	Singlet	Immune_cells
SAT_2M	2411	1323	0.0413565	8	Singlet	Mesothelial
SAT_2M	2968	1451	0.7733692	0	Singlet	Adipocyte
SAT_2M	3823	1879	0.4702194	11	Singlet	Smooth_muscle_cells
SAT_2M	1100	732	3.8949275	1	Singlet	Immune_cells
SAT_2M	1924	1150	0.6195147	1	Singlet	Immune_cells
SAT_2M	1213	809	0.5733006	5	Singlet	Immune_cells
SAT_2M	1320	858	2.3255814	1	Singlet	Immune_cells
SAT_2M	1934	1080	4.7227926	0	Singlet	Adipocyte
SAT_2M	1191	749	0.9205021	0	Singlet	Adipocyte
SAT_2M	1797	1073	2.8872848	2	Singlet	FAPs
SAT_2M	1892	1154	0.5789474	0	Singlet	Adipocyte
SAT_2M	1427	814	8.4958217	1	Singlet	Immune_cells
SAT_2M	1887	1131	1.9545695	0	Singlet	Adipocyte
SAT_2M	1229	722	9.1720779	10	Singlet	Immune_cells
SAT_2M	2886	1608	0.3791796	0	Singlet	Adipocyte
SAT_2M	1270	808	4.7058824	6	Singlet	Neurons
SAT_2M	1915	1212	0.8831169	4	Singlet	Immune_cells
SAT_2M	1371	869	1.9650655	1	Singlet	Immune_cells
SAT_2M	1011	658	3.2576505	1	Singlet	Immune_cells
SAT_2M	1304	813	0.3825555	1	Singlet	Immune_cells
SAT_2M	3232	1658	11.066461	9	Singlet	Mesothelial
SAT_2M	2172	1288	0.2751032	0	Singlet	Adipocyte
SAT_2M	1267	869	0.7836991	1	Singlet	Immune_cells
SAT_2M	1369	878	0.2184996	1	Singlet	Immune_cells
SAT_2M	1265	793	4.4952681	1	Singlet	Immune_cells
SAT_2M	1005	669	0.7928642	1	Singlet	Immune_cells
SAT_2M	1321	834	7.5882795	1	Singlet	Immune_cells
SAT_2M	1013	695	1.5701668	4	Singlet	Immune_cells
SAT_2M	1371	831	4.3572985	4	Singlet	Immune_cells
SAT_2M	1263	835	0.5507474	1	Singlet	Immune_cells
SAT_2M	1667	1083	0.7751938	5	Singlet	Immune_cells
SAT_2M	1484	932	0.7372654	0	Singlet	Adipocyte
SAT_2M	1522	955	0.7203667	0	Singlet	Adipocyte
SAT_2M	1033	672	0.1936108	4	Singlet	Immune_cells
SAT_2M	4790	2017	3.2554257	10	Singlet	Immune_cells
SAT_2M	2331	1397	3.5912783	1	Singlet	Immune_cells

SAT_2M	1543	959	0.1936733	4	Singlet	Immune_cells
SAT_2M	1960	1338	0.2024292	4	Singlet	Immune_cells
SAT_2M	2911	1551	4.5953361	1	Singlet	Immune_cells
SAT_2M	4731	2168	11.825464	9	Singlet	Mesothelial
SAT_2M	3489	1657	8.8386728	0	Singlet	Adipocyte
SAT_2M	1661	911	6.25	9	Singlet	Mesothelial
SAT_2M	1071	712	3.8033395	1	Singlet	Immune_cells
SAT_2M	1128	772	0.530504	1	Singlet	Immune_cells
SAT_2M	1305	859	1.606733	1	Singlet	Immune_cells
SAT_2M	1035	707	0.1924928	1	Singlet	Immune_cells
SAT_2M	1351	822	2.5868441	10	Singlet	Immune_cells
SAT_2M	2186	1203	10.255242	4	Singlet	Immune_cells
SAT_2M	1513	960	2.2442244	4	Singlet	Immune_cells
SAT_2M	1705	1088	0.4672897	5	Singlet	Immune_cells
SAT_2M	1221	741	5.400982	5	Singlet	Immune_cells
SAT_2M	1801	1076	2.4916944	4	Singlet	Immune_cells
SAT_2M	1499	851	2.1150033	0	Singlet	Adipocyte
SAT_2M	1077	717	0.1831502	4	Singlet	Immune_cells
SAT_2M	1105	720	3.060306	4	Singlet	Immune_cells
SAT_2M	1286	819	5.1162791	1	Singlet	Immune_cells
SAT_2M	1914	1064	4.8462741	0	Singlet	Adipocyte
SAT_2M	1553	933	6.3665595	1	Singlet	Immune_cells
SAT_2M	1737	1035	7.466973	9	Singlet	Mesothelial
SAT_2M	1316	872	0.8345979	0	Singlet	Adipocyte
SAT_2M	1411	934	0.2111189	0	Singlet	Adipocyte
SAT_2M	1831	1125	0.1084599	0	Singlet	Adipocyte
SAT_2M	1490	949	3.1417112	1	Singlet	Immune_cells
SAT_2M	1744	1030	4	4	Singlet	Immune_cells
SAT_2M	1656	998	7.2158749	4	Singlet	Immune_cells
SAT_2M	1654	986	3.3816425	1	Singlet	Immune_cells
SAT_2M	1970	1166	0.2026342	0	Singlet	Adipocyte
SAT_2M	1494	911	4.5393858	1	Singlet	Immune_cells
SAT_2M	1865	1146	9.1586794	1	Singlet	Immune_cells
SAT_2M	1403	880	0.2828854	0	Singlet	Adipocyte
SAT_2M	1263	809	5.6082149	1	Singlet	Immune_cells
SAT_2M	1292	802	4.6707504	9	Singlet	Mesothelial
SAT_2M	3758	1676	1.9123506	10	Singlet	Immune_cells
SAT_2M	1229	768	0.2435065	0	Singlet	Adipocyte
SAT_2M	1039	660	0.6724304	10	Singlet	Immune_cells
SAT_2M	1471	798	9.4493542	9	Singlet	Mesothelial
SAT_2M	1255	801	0.9538951	4	Singlet	Immune_cells
SAT_2M	1630	994	4.642639	4	Singlet	Immune_cells
SAT_2M	1984	1243	0.35	3	Singlet	FAPs
SAT_2M	3328	1709	1.9507803	9	Singlet	Mesothelial
SAT_2M	1847	1130	1.2372243	0	Singlet	Adipocyte
SAT_2M	2312	1323	0.4312204	0	Singlet	Adipocyte
SAT_2M	2877	1512	0.7612457	0	Singlet	Adipocyte
SAT_2M	1566	990	0.5069708	0	Singlet	Adipocyte
SAT_2M	1039	665	0.7670182	4	Singlet	Immune_cells
SAT_2M	1249	808	2.8776978	4	Singlet	Immune_cells
SAT_2M	1028	723	0.5785921	4	Singlet	Immune_cells

SAT_2M	1638	1043	0.1215805	3	Singlet	FAPs
SAT_2M	1178	822	1.090604	4	Singlet	Immune_cells
SAT_2M	1474	893	3.2542373	1	Singlet	Immune_cells
SAT_2M	2289	1328	0.562527	0	Singlet	Adipocyte
SAT_2M	2261	1284	1.1941619	4	Singlet	Immune_cells
SAT_2M	2720	1488	4.0719002	8	Singlet	Mesothelial
SAT_2M	1341	836	0.8166295	0	Singlet	Adipocyte
SAT_2M	1103	778	1.2544803	5	Singlet	Immune_cells
SAT_2M	1345	898	3.4048853	8	Singlet	Mesothelial
SAT_2M	1812	980	3.1798246	10	Singlet	Immune_cells
SAT_2M	1579	880	1.6970459	9	Singlet	Mesothelial
SAT_2M	1070	737	1.0185185	1	Singlet	Immune_cells
SAT_2M	1341	831	0	3	Singlet	FAPs
SAT_2M	2602	1338	2.2553517	0	Singlet	Adipocyte
SAT_2M	1349	829	0.0735835	1	Singlet	Immune_cells
SAT_2M	1092	712	0.7312614	4	Singlet	Immune_cells
SAT_2M	1646	1055	0.8474576	1	Singlet	Immune_cells
SAT_2M	1227	736	0.1605136	0	Singlet	Adipocyte
SAT_2M	1711	970	10.37296	1	Singlet	Immune_cells
SAT_2M	1468	917	2.0380435	1	Singlet	Immune_cells
SAT_2M	3081	1345	4.7619048	10	Singlet	Immune_cells
SAT_2M	1109	745	1.4427412	7	Singlet	Endothelial_cells
SAT_2M	1594	989	3.5514019	1	Singlet	Immune_cells
SAT_2M	1055	722	0.2822201	4	Singlet	Immune_cells
SAT_2M	1556	990	0.4467135	4	Singlet	Immune_cells
SAT_2M	1359	849	10.499266	1	Singlet	Immune_cells
SAT_2M	1153	788	3.0276817	4	Singlet	Immune_cells
SAT_2M	1839	1044	1.5159718	5	Singlet	Immune_cells
SAT_2M	1394	897	0.2135231	4	Singlet	Immune_cells
SAT_2M	2522	1411	0.7089405	0	Singlet	Adipocyte
SAT_2M	2136	1230	0.7434944	0	Singlet	Adipocyte
SAT_2M	4792	1734	1.8549396	10	Singlet	Immune_cells
SAT_2M	2058	1252	6.0562016	4	Singlet	Immune_cells
SAT_2M	1658	1026	0.8438819	4	Singlet	Immune_cells
SAT_2M	1517	980	1.0512484	5	Singlet	Immune_cells
SAT_2M	1224	767	8.9649552	1	Singlet	Immune_cells
SAT_2M	2155	1262	0.6928406	0	Singlet	Adipocyte
SAT_2M	1686	1058	0.2366864	4	Singlet	Immune_cells
SAT_2M	1059	716	1.8885741	1	Singlet	Immune_cells
SAT_2M	2978	1505	5.6540649	0	Singlet	Adipocyte
SAT_2M	3578	1926	4.6901173	8	Singlet	Mesothelial
SAT_2M	1500	900	0	0	Singlet	Adipocyte
SAT_2M	2572	1474	1.3894249	0	Singlet	Adipocyte
SAT_2M	1098	685	4.3557169	8	Singlet	Mesothelial
SAT_2M	1307	789	0.6859756	0	Singlet	Adipocyte
SAT_2M	1363	838	7.6079005	1	Singlet	Immune_cells
SAT_2M	1273	834	3.299293	4	Singlet	Immune_cells
SAT_2M	1222	814	0.7352941	1	Singlet	Immune_cells
SAT_2M	1243	763	0.8012821	0	Singlet	Adipocyte
SAT_2M	1617	1012	0.8039579	1	Singlet	Immune_cells
SAT_2M	1755	1031	0.5116543	1	Singlet	Immune_cells

SAT_2M	2070	1079	12.757794	1	Singlet	Immune_cells
SAT_2M	1333	897	7.5598802	1	Singlet	Immune_cells
SAT_2M	1599	964	4.9782203	5	Singlet	Immune_cells
SAT_2M	3195	1569	2.9338327	0	Singlet	Adipocyte
SAT_2M	1011	681	0.1966568	4	Singlet	Immune_cells
SAT_2M	1276	832	0.933126	0	Singlet	Adipocyte
SAT_2M	2009	1217	0.4952947	2	Singlet	FAPs
SAT_2M	1001	683	1.8774704	5	Singlet	Immune_cells
SAT_2M	1691	1122	0.1176471	8	Singlet	Mesothelial
SAT_2M	1265	801	0.1577287	0	Singlet	Adipocyte
SAT_2M	1176	730	6.2711864	1	Singlet	Immune_cells
SAT_2M	1581	941	1.0062893	0	Singlet	Adipocyte
SAT_2M	2713	1375	7.0640177	1	Singlet	Immune_cells
SAT_2M	1394	829	8.7857143	1	Singlet	Immune_cells
SAT_2M	1650	852	1.443175	0	Singlet	Adipocyte
SAT_2M	1077	682	2.1256932	1	Singlet	Immune_cells
SAT_2M	1761	1092	1.5254237	1	Singlet	Immune_cells
SAT_2M	1378	855	3.7572254	1	Singlet	Immune_cells
SAT_2M	3055	1608	0	0	Singlet	Adipocyte
SAT_2M	2306	1279	0.1728608	0	Singlet	Adipocyte
SAT_2M	1021	606	8.1213307	0	Singlet	Adipocyte
SAT_2M	2319	1133	0.4729149	0	Singlet	Adipocyte
SAT_2M	1083	700	2.3941068	10	Singlet	Immune_cells
SAT_2M	1819	1129	3.5145524	4	Singlet	Immune_cells
SAT_2M	1660	1057	0.5415162	0	Singlet	Adipocyte
SAT_2M	1143	767	0.5181347	1	Singlet	Immune_cells
SAT_2M	1714	830	6.504065	4	Singlet	Immune_cells
SAT_2M	2835	1569	12.565628	4	Singlet	Immune_cells
SAT_2M	1914	858	7.7725613	0	Singlet	Adipocyte
SAT_2M	1103	678	0.8115419	1	Singlet	Immune_cells
SAT_2M	1653	1009	1.0830325	0	Singlet	Adipocyte
SAT_2M	1435	773	1.2517385	4	Singlet	Immune_cells
SAT_2M	1273	820	1.4855356	1	Singlet	Immune_cells
SAT_2M	1311	830	4.9279757	4	Singlet	Immune_cells
SAT_2M	1304	902	0.3055768	11	Singlet	Smooth_muscle_cells
SAT_2M	1968	1247	3.3924051	4	Singlet	Immune_cells
SAT_2M	3268	1580	0.5159332	0	Singlet	Adipocyte
SAT_2M	3324	1668	0.448833	0	Singlet	Adipocyte
SAT_2M	1059	578	0.2816901	4	Singlet	Immune_cells
SAT_2M	2776	1555	0.9673952	11	Singlet	Smooth_muscle_cells
SAT_2M	1483	894	0.4578156	6	Singlet	Neurons
SAT_2M	1513	1061	0.4611331	5	Singlet	Immune_cells
SAT_2M	1097	740	2.4412297	7	Singlet	Endothelial_cells
SAT_2M	1207	749	1.7269737	0	Singlet	Adipocyte
SAT_2M	1223	812	2.0308692	4	Singlet	Immune_cells
SAT_2M	1007	682	0.6916996	1	Singlet	Immune_cells
SAT_2M	1221	763	0.3273322	9	Singlet	Mesothelial
SAT_2M	1355	840	4.2584435	1	Singlet	Immune_cells
SAT_2M	1927	1005	1.1387164	4	Singlet	Immune_cells
SAT_2M	2934	1232	6.6326531	3	Singlet	FAPs
SAT_2M	1257	849	1.9077901	1	Singlet	Immune_cells

SAT_2M	1555	973	2.5657473	4	Singlet	Immune_cells
SAT_2M	1121	614	0	4	Singlet	Immune_cells
SAT_2M	1831	1075	1.5267176	1	Singlet	Immune_cells
SAT_2M	1611	1002	4.07911	4	Singlet	Immune_cells
SAT_2M	1886	1102	0.6342495	0	Singlet	Adipocyte
SAT_2M	1274	875	1.015625	9	Singlet	Mesothelial
SAT_2M	1216	811	0.2457002	3	Singlet	FAPs
SAT_2M	1198	720	9.9166667	1	Singlet	Immune_cells
SAT_2M	1423	834	0.6306938	0	Singlet	Adipocyte
SAT_2M	1057	730	3.8570085	4	Singlet	Immune_cells
SAT_2M	1617	955	3.6307692	1	Singlet	Immune_cells
SAT_2M	1120	725	0.9760426	4	Singlet	Immune_cells
SAT_2M	1342	876	1.9985196	0	Singlet	Adipocyte
SAT_2M	1075	700	2.3234201	5	Singlet	Immune_cells
SAT_2M	1462	939	0.3394433	8	Singlet	Mesothelial
SAT_2M	2195	1250	1.3593113	8	Singlet	Mesothelial
SAT_2M	1960	1168	5.8943089	1	Singlet	Immune_cells
SAT_2M	2440	1435	3.3932952	3	Singlet	FAPs
SAT_2M	1077	735	1.9408503	5	Singlet	Immune_cells
SAT_2M	1208	749	1.3147083	0	Singlet	Adipocyte
SAT_2M	1384	878	3.4507549	2	Singlet	FAPs
SAT_2M	1083	757	0.5338078	6	Singlet	Neurons
SAT_2M	1982	1225	1.207851	1	Singlet	Immune_cells
SAT_2M	1029	760	0.3656307	6	Singlet	Neurons
SAT_2M	1006	701	5.3571429	5	Singlet	Immune_cells
SAT_2M	1866	1068	1.5532941	10	Singlet	Immune_cells
SAT_2M	2113	1204	5.7601511	1	Singlet	Immune_cells
SAT_2M	6335	2543	1.1037528	10	Singlet	Immune_cells
SAT_2M	1579	1019	0.5047319	7	Singlet	Endothelial_cells
SAT_2M	1235	819	3.4733441	1	Singlet	Immune_cells
SAT_2M	3542	1790	1.3235708	10	Singlet	Immune_cells
SAT_2M	1521	942	0.3267974	0	Singlet	Adipocyte
SAT_2M	1454	860	9.1780822	1	Singlet	Immune_cells
SAT_2M	2566	1379	1.5558149	8	Singlet	Mesothelial
SAT_2M	2891	1627	0.1727713	0	Singlet	Adipocyte
SAT_2M	1298	875	4.058193	7	Singlet	Endothelial_cells
SAT_2M	1711	1076	3.8998836	4	Singlet	Immune_cells
SAT_2M	4937	2049	3.7560582	10	Singlet	Immune_cells
SAT_2M	6469	2825	9.2255477	9	Singlet	Mesothelial
SAT_2M	1066	708	4.5751634	1	Singlet	Immune_cells
SAT_2M	1120	752	1.2488849	4	Singlet	Immune_cells
SAT_2M	1758	913	8.2386364	10	Singlet	Immune_cells
SAT_2M	2882	1749	0.6187693	4	Singlet	Immune_cells
SAT_2M	1171	794	0.5947324	4	Singlet	Immune_cells
SAT_2M	1077	735	3.0164534	3	Singlet	FAPs
SAT_2M	1237	778	7.073955	1	Singlet	Immune_cells
SAT_2M	1359	785	10.433505	5	Singlet	Immune_cells
SAT_2M	1382	814	10.878963	1	Singlet	Immune_cells
SAT_2M	1053	646	8.7984863	5	Singlet	Immune_cells
SAT_2M	1499	865	12.2	1	Singlet	Immune_cells
SAT_2M	2105	1210	7.4196597	1	Singlet	Immune_cells

SAT_2M	1557	983	0.8945687	4	Singlet	Immune_cells
SAT_2M	2482	1314	3.9373242	10	Singlet	Immune_cells
SAT_2M	1735	1023	3.5714286	1	Singlet	Immune_cells
SAT_2M	2909	1556	1.5395142	0	Singlet	Adipocyte
SAT_2M	1152	766	0.2597403	3	Singlet	FAPs
SAT_2M	1934	1108	0.1029866	0	Singlet	Adipocyte
SAT_2M	1859	1202	2.6273458	4	Singlet	Immune_cells
SAT_2M	2556	1174	10.234375	5	Singlet	Immune_cells
SAT_2M	1536	974	6.2946139	4	Singlet	Immune_cells
SAT_2M	1810	1107	3.3076075	8	Singlet	Mesothelial
SAT_2M	1193	745	5.5045872	5	Singlet	Immune_cells
SAT_2M	1333	803	9.588015	1	Singlet	Immune_cells
SAT_2M	1714	1089	1.216686	1	Singlet	Immune_cells
SAT_2M	1515	868	2.9644269	5	Singlet	Immune_cells
SAT_2M	1004	695	0.29615	1	Singlet	Immune_cells
SAT_2M	2399	1358	1.7040732	0	Singlet	Adipocyte
SAT_2M	1212	749	0.0823723	1	Singlet	Immune_cells
SAT_2M	1900	1152	0.4707113	5	Singlet	Immune_cells
SAT_2M	1782	1115	0.280112	4	Singlet	Immune_cells
SAT_2M	1602	1001	1.3715711	8	Singlet	Mesothelial
SAT_2M	1055	699	1.7907634	0	Singlet	Adipocyte
SAT_2M	1876	1183	1.1116993	5	Singlet	Immune_cells
SAT_2M	1862	1114	3.9079229	4	Singlet	Immune_cells
SAT_2M	3280	1661	0.4254026	0	Singlet	Adipocyte
SAT_2M	1212	757	7.0204082	4	Singlet	Immune_cells
SAT_2M	1390	820	4.9356223	1	Singlet	Immune_cells
SAT_2M	1781	1068	2.3542601	1	Singlet	Immune_cells
SAT_2M	1218	749	1.7959184	0	Singlet	Adipocyte
SAT_2M	1038	655	0.3838772	1	Singlet	Immune_cells
SAT_2M	1250	836	5.1958433	4	Singlet	Immune_cells
SAT_2M	1311	816	1.754386	0	Singlet	Adipocyte
SAT_2M	1602	983	4.1358025	1	Singlet	Immune_cells
SAT_2M	1446	892	4.3358568	1	Singlet	Immune_cells
SAT_2M	4095	2057	5.211885	4	Singlet	Immune_cells
SAT_2M	2109	1223	10.287872	5	Singlet	Immune_cells
SAT_2M	1339	841	5.2945563	1	Singlet	Immune_cells
SAT_2M	2869	1478	1.181376	0	Singlet	Adipocyte
SAT_2M	2211	1307	4.6973803	11	Singlet	Smooth_muscle_cells
SAT_2M	1169	743	0.6820119	0	Singlet	Adipocyte
SAT_2M	1302	923	0.4562738	1	Singlet	Immune_cells
SAT_2M	1219	706	2.5326797	1	Singlet	Immune_cells
SAT_2M	1782	1110	0.2238388	4	Singlet	Immune_cells
SAT_2M	2122	1146	2.1146617	10	Singlet	Immune_cells
SAT_2M	3058	1587	0.3911343	3	Singlet	FAPs
SAT_2M	1157	742	0.4280822	0	Singlet	Adipocyte
SAT_2M	1364	833	3.1318281	10	Singlet	Immune_cells
SAT_2M	1576	1006	4.3588124	1	Singlet	Immune_cells
SAT_2M	1390	920	0.3584229	4	Singlet	Immune_cells
SAT_2M	1350	836	1.0316875	1	Singlet	Immune_cells
SAT_2M	2914	1654	0.1366587	0	Singlet	Adipocyte
SAT_2M	1221	822	4.2518397	5	Singlet	Immune_cells

SAT_2M	2202	1180	6.6966292	0	Singlet	Adipocyte
SAT_2M	2284	1321	0.435161	4	Singlet	Immune_cells
SAT_2M	2801	1490	1.2433393	0	Singlet	Adipocyte
SAT_2M	1341	834	0.297619	4	Singlet	Immune_cells
SAT_2M	1864	1114	0.6393181	4	Singlet	Immune_cells
SAT_2M	2389	1330	11.389237	1	Singlet	Immune_cells
SAT_2M	4431	1953	3.0146232	0	Singlet	Adipocyte
SAT_2M	3403	1815	7.9532164	4	Singlet	Immune_cells
SAT_2M	2077	1183	6.6891242	1	Singlet	Immune_cells
SAT_2M	1779	1197	0.4466778	11	Singlet	Smooth_muscle_cells
SAT_2M	1168	779	2.3829787	1	Singlet	Immune_cells
SAT_2M	1492	985	0.5996003	3	Singlet	FAPs
SAT_2M	1256	819	3.4920635	0	Singlet	Adipocyte
SAT_2M	1802	1110	0.1102536	6	Singlet	Neurons
SAT_2M	2060	1241	0.4819277	2	Singlet	FAPs
SAT_2M	1361	879	0.2915452	1	Singlet	Immune_cells
SAT_2M	1718	1063	1.9174898	5	Singlet	Immune_cells
SAT_2M	1862	1074	8.3511777	1	Singlet	Immune_cells
SAT_2M	1326	894	0.7524454	1	Singlet	Immune_cells
SAT_2M	1189	704	1.4214047	4	Singlet	Immune_cells
SAT_2M	3021	1634	6.3057115	4	Singlet	Immune_cells
SAT_2M	1415	857	5.2521008	1	Singlet	Immune_cells
SAT_2M	1683	1016	0.4739336	0	Singlet	Adipocyte
SAT_2M	1317	848	1.5921152	1	Singlet	Immune_cells
SAT_2M	1205	841	0.6578947	4	Singlet	Immune_cells
SAT_2M	4211	1899	2.7711985	10	Singlet	Immune_cells
SAT_2M	1170	705	0.4248088	0	Singlet	Adipocyte
SAT_2M	3091	1561	3.8994521	0	Singlet	Adipocyte
SAT_2M	1283	817	0.2327386	1	Singlet	Immune_cells
SAT_2M	2011	1145	1.1892963	4	Singlet	Immune_cells
SAT_2M	3049	1512	0.3274394	3	Singlet	FAPs
SAT_2M	1648	903	2.4213075	0	Singlet	Adipocyte
SAT_2M	1430	928	1.4675052	4	Singlet	Immune_cells
SAT_2M	1524	821	2.1596859	1	Singlet	Immune_cells
SAT_2M	3309	1555	5.6342272	0	Singlet	Adipocyte
SAT_2M	1028	682	0.6796117	4	Singlet	Immune_cells
SAT_2M	2006	1121	3.1730293	1	Singlet	Immune_cells
SAT_2M	4284	2067	1.4225746	0	Singlet	Adipocyte
SAT_2M	1899	1064	4.2430592	4	Singlet	Immune_cells
SAT_2M	1456	893	6.7854695	1	Singlet	Immune_cells
SAT_2M	1285	733	0.3098373	4	Singlet	Immune_cells
SAT_2M	1001	675	0.5946482	0	Singlet	Adipocyte
SAT_2M	3447	1855	0.0868056	5	Singlet	Immune_cells
SAT_2M	2764	1320	1.2274368	0	Singlet	Adipocyte
SAT_2M	2330	1331	0.1280956	8	Singlet	Mesothelial
SAT_2M	1674	998	5.4198928	1	Singlet	Immune_cells
SAT_2M	1221	720	4.3300654	1	Singlet	Immune_cells
SAT_2M	2914	1422	3.0748206	4	Singlet	Immune_cells
SAT_2M	2000	1333	0.4457652	4	Singlet	Immune_cells
SAT_2M	2674	1411	0.5959032	0	Singlet	Adipocyte
SAT_2M	1061	635	3.1073446	0	Singlet	Adipocyte

SAT_2M	1770	1104	3.9753639	1	Singlet	Immune_cells
SAT_2M	1127	720	0.0883392	1	Singlet	Immune_cells
SAT_2M	1262	777	8.4518167	1	Singlet	Immune_cells
SAT_2M	1423	910	1.4583333	4	Singlet	Immune_cells
SAT_2M	2400	1341	3.1950207	1	Singlet	Immune_cells
SAT_2M	1105	726	1.534296	4	Singlet	Immune_cells
SAT_2M	1479	965	2.4932615	7	Singlet	Endothelial_cells
SAT_2M	1645	1019	2.4271845	4	Singlet	Immune_cells
SAT_2M	1006	699	0.6910168	4	Singlet	Immune_cells
SAT_2M	2030	1222	1.8654885	0	Singlet	Adipocyte
SAT_2M	1618	932	5.6103576	1	Singlet	Immune_cells
SAT_2M	1052	608	0.5644403	4	Singlet	Immune_cells
SAT_2M	2937	1447	12.267028	0	Singlet	Adipocyte
SAT_2M	1129	644	0.4405286	0	Singlet	Adipocyte
SAT_2M	3298	1630	1.1164756	0	Singlet	Adipocyte
SAT_2M	2071	1258	2.5012025	4	Singlet	Immune_cells
SAT_2M	1005	582	0.4970179	1	Singlet	Immune_cells
SAT_2M	1305	789	3.0280091	4	Singlet	Immune_cells
SAT_2M	1005	622	0.6951341	0	Singlet	Adipocyte
SAT_2M	1692	1042	4.8320566	5	Singlet	Immune_cells
SAT_2M	1247	831	2.6294821	1	Singlet	Immune_cells
SAT_2M	1200	673	4.0429043	1	Singlet	Immune_cells
SAT_2M	1036	589	1.0587103	1	Singlet	Immune_cells
SAT_2M	1197	720	1.6680567	11	Singlet	Smooth_muscle_cells
SAT_2M	1013	561	1.9723866	1	Singlet	Immune_cells
SAT_2M	1237	730	3.1375704	1	Singlet	Immune_cells
SAT_2M	3558	1900	5.5243971	9	Singlet	Mesothelial
SAT_2M	1630	1067	3.3618582	11	Singlet	Smooth_muscle_cells
SAT_2M	2746	1623	1.1982571	2	Singlet	FAPs
SAT_2M	1253	701	14.920635	1	Singlet	Immune_cells
SAT_2M	2965	1545	1.5151515	0	Singlet	Adipocyte
SAT_2M	1471	925	0.6101695	0	Singlet	Adipocyte
SAT_2M	2199	1299	4.8570132	4	Singlet	Immune_cells
SAT_2M	2007	1154	0.2485089	5	Singlet	Immune_cells
SAT_2M	3344	1754	0.9259259	0	Singlet	Adipocyte
SAT_2M	1134	734	0.7887818	4	Singlet	Immune_cells
SAT_2M	1244	825	0.5608974	4	Singlet	Immune_cells
SAT_2M	8366	3149	4.1298639	9	Singlet	Mesothelial
SAT_2M	1137	800	0.8756567	5	Singlet	Immune_cells
SAT_2M	1243	690	6.1501597	10	Singlet	Immune_cells
SAT_2M	1699	997	0.4105572	0	Singlet	Adipocyte
SAT_2M	1600	1060	3.8557214	1	Singlet	Immune_cells
SAT_2M	1222	776	0.5668016	5	Singlet	Immune_cells
SAT_2M	2834	1552	0.2455279	0	Singlet	Adipocyte
SAT_2M	1068	762	0.3710575	5	Singlet	Immune_cells
SAT_2M	1004	657	1.3779528	2	Singlet	FAPs
SAT_2M	1146	810	0.6076389	4	Singlet	Immune_cells
SAT_2M	1036	684	2.3121387	5	Singlet	Immune_cells
SAT_2M	1556	953	0.2557545	1	Singlet	Immune_cells
SAT_2M	1039	659	1.2416428	4	Singlet	Immune_cells
SAT_2M	1175	796	2.0321761	0	Singlet	Adipocyte

SAT_2M	1798	1111	2.0487265	1	Singlet	Immune_cells
SAT_2M	1987	1150	0.6036217	0	Singlet	Adipocyte
SAT_2M	1134	766	0	1	Singlet	Immune_cells
SAT_2M	1581	939	1.5141956	4	Singlet	Immune_cells
SAT_2M	1624	965	0.1842752	1	Singlet	Immune_cells
SAT_2M	2241	1179	9.8265896	1	Singlet	Immune_cells
SAT_2M	1128	733	0.4413063	1	Singlet	Immune_cells
SAT_2M	1316	825	0.6792453	0	Singlet	Adipocyte
SAT_2M	1108	768	0.0894454	5	Singlet	Immune_cells
SAT_2M	2983	1606	4.8317228	1	Singlet	Immune_cells
SAT_2M	1148	674	10.947003	1	Singlet	Immune_cells
SAT_2M	1785	1158	0.3899721	4	Singlet	Immune_cells
SAT_2M	1283	815	0.7698229	4	Singlet	Immune_cells
SAT_2M	1245	788	0.6384677	1	Singlet	Immune_cells
SAT_2M	1347	827	0.7358352	0	Singlet	Adipocyte
SAT_2M	1038	706	2.2900763	1	Singlet	Immune_cells
SAT_2M	1888	1209	1.6384778	9	Singlet	Mesothelial
SAT_2M	2109	1198	0.0945626	0	Singlet	Adipocyte
SAT_2M	1122	798	0.2620087	4	Singlet	Immune_cells
SAT_2M	6907	3065	12.613784	4	Singlet	Immune_cells
SAT_2M	1283	791	0.6225681	1	Singlet	Immune_cells
SAT_2M	1050	703	0.6616257	3	Singlet	FAPs
SAT_2M	1011	692	0.6896552	4	Singlet	Immune_cells
SAT_2M	1647	1090	0.2421308	8	Singlet	Mesothelial
SAT_2M	1339	801	9.9925429	1	Singlet	Immune_cells
SAT_2M	1512	947	0.7910349	1	Singlet	Immune_cells
SAT_2M	1409	848	2.8995757	1	Singlet	Immune_cells
SAT_2M	5996	2418	1.9147519	10	Singlet	Immune_cells
SAT_2M	1068	722	1.0261194	4	Singlet	Immune_cells
SAT_2M	1506	876	7.2703239	1	Singlet	Immune_cells
SAT_2M	1031	683	0.0965251	4	Singlet	Immune_cells
SAT_2M	1224	752	0.5672609	4	Singlet	Immune_cells
SAT_2M	3076	1564	0.5832793	0	Singlet	Adipocyte
SAT_2M	2221	1271	0.8952551	0	Singlet	Adipocyte
SAT_2M	2471	1381	0.6048387	0	Singlet	Adipocyte
SAT_2M	1448	910	1.4452856	1	Singlet	Immune_cells
SAT_2M	1497	936	3.5262808	4	Singlet	Immune_cells
SAT_2M	2288	1204	1.4372822	3	Singlet	FAPs
SAT_2M	2027	1168	2.1151008	0	Singlet	Adipocyte
SAT_2M	1124	784	0.6134969	1	Singlet	Immune_cells
SAT_2M	1166	749	0.1710864	0	Singlet	Adipocyte
SAT_2M	1232	697	5.0929669	0	Singlet	Adipocyte
SAT_2M	1511	905	3.5596572	1	Singlet	Immune_cells
SAT_2M	1542	962	1.8087855	0	Singlet	Adipocyte
SAT_2M	1588	972	0	0	Singlet	Adipocyte
SAT_2M	1410	936	0.7768362	4	Singlet	Immune_cells
SAT_2M	2440	1445	4.6644845	8	Singlet	Mesothelial
SAT_2M	1808	1116	0.6615215	1	Singlet	Immune_cells
SAT_2M	1341	792	1.6344725	3	Singlet	FAPs
SAT_2M	1426	933	1.396648	4	Singlet	Immune_cells
SAT_2M	1288	808	3.8491147	1	Singlet	Immune_cells

SAT_2M	1514	1009	1.8372703	4	Singlet	Immune_cells
SAT_2M	2631	1456	7.4829932	10	Singlet	Immune_cells
SAT_2M	1172	800	0.1703578	11	Singlet	Smooth_muscle_cells
SAT_2M	1067	645	6.1452514	1	Singlet	Immune_cells
SAT_2M	1618	1027	3.331277	1	Singlet	Immune_cells
SAT_2M	2095	1228	3.4269396	4	Singlet	Immune_cells
SAT_2M	1296	791	0.1540832	1	Singlet	Immune_cells
SAT_2M	1787	987	5.5122494	10	Singlet	Immune_cells
SAT_2M	1313	848	1.9726859	1	Singlet	Immune_cells
SAT_2M	2542	1161	2.7766914	10	Singlet	Immune_cells
SAT_2M	1080	708	3.2407407	3	Singlet	FAPs
SAT_2M	1492	958	0.266489	1	Singlet	Immune_cells
SAT_2M	1887	1075	5.0211416	1	Singlet	Immune_cells
SAT_2M	1245	803	0.7165605	4	Singlet	Immune_cells
SAT_2M	1464	933	3.2719836	4	Singlet	Immune_cells
SAT_2M	1411	913	2.6798307	1	Singlet	Immune_cells
SAT_2M	1227	803	0.6477733	0	Singlet	Adipocyte
SAT_2M	1022	688	3.7937743	0	Singlet	Adipocyte
SAT_2M	1380	851	2.894356	1	Singlet	Immune_cells
SAT_2M	3349	1675	0.3267974	0	Singlet	Adipocyte
SAT_2M	2304	1338	0.5181347	4	Singlet	Immune_cells
SAT_2M	1292	821	1.7705928	0	Singlet	Adipocyte
SAT_2M	6571	2646	8.4143378	9	Singlet	Mesothelial
SAT_2M	1418	880	3.2913165	1	Singlet	Immune_cells
SAT_2M	2659	1448	6.2453254	5	Singlet	Immune_cells
SAT_2M	2133	1236	0.2802429	0	Singlet	Adipocyte
SAT_2M	1507	930	2.8985507	0	Singlet	Adipocyte
SAT_2M	1447	875	1.1732229	0	Singlet	Adipocyte
SAT_2M	2188	1297	0.4988662	0	Singlet	Adipocyte
SAT_2M	1045	726	0.0956938	4	Singlet	Immune_cells
SAT_2M	1304	784	10.022953	1	Singlet	Immune_cells
SAT_2M	1886	1099	3.7645811	1	Singlet	Immune_cells
SAT_2M	2891	1508	4.283247	10	Singlet	Immune_cells
SAT_2M	2241	1157	0.8900757	10	Singlet	Immune_cells
SAT_2M	1334	803	0.5219985	4	Singlet	Immune_cells
SAT_2M	1848	994	11.764706	1	Singlet	Immune_cells
SAT_2M	1182	835	1.937658	1	Singlet	Immune_cells
SAT_2M	1843	1164	0.3243243	0	Singlet	Adipocyte
SAT_2M	1713	1106	0.8705746	2	Singlet	FAPs
SAT_2M	1955	1140	0.4081633	0	Singlet	Adipocyte
SAT_2M	6681	2853	8.77429	9	Singlet	Mesothelial
SAT_2M	1221	777	6.1324612	1	Singlet	Immune_cells
SAT_2M	1358	831	0.5856515	6	Singlet	Neurons
SAT_2M	2333	1330	2.48394	8	Singlet	Mesothelial
SAT_2M	1229	754	2.8455285	1	Singlet	Immune_cells
SAT_2M	1451	918	0.2745367	11	Singlet	Smooth_muscle_cells
SAT_2M	1708	970	6.0676779	3	Singlet	FAPs
SAT_2M	1667	1032	0.1196888	4	Singlet	Immune_cells
SAT_2M	1215	770	2.4549918	1	Singlet	Immune_cells
SAT_2M	1190	810	0.2508361	5	Singlet	Immune_cells
SAT_2M	1162	780	0.2575107	8	Singlet	Mesothelial

SAT_2M	1133	740	0.1762115	4	Singlet	Immune_cells
SAT_2M	2855	1373	3.5364146	2	Singlet	FAPs
SAT_2M	1067	737	0.8395522	5	Singlet	Immune_cells
SAT_2M	1379	800	0.6521739	0	Singlet	Adipocyte
SAT_2M	1349	882	0.5865103	1	Singlet	Immune_cells
SAT_2M	1457	891	4.4429255	5	Singlet	Immune_cells
SAT_2M	1241	770	1.7642342	4	Singlet	Immune_cells
SAT_2M	1748	992	2.6106697	0	Singlet	Adipocyte
SAT_2M	1455	911	3.7568306	3	Singlet	FAPs
SAT_2M	1403	911	0.9978617	4	Singlet	Immune_cells
SAT_2M	1474	912	2.300406	1	Singlet	Immune_cells
SAT_2M	2189	1238	4.9590537	0	Singlet	Adipocyte
SAT_2M	1811	1119	0.4412576	0	Singlet	Adipocyte
SAT_2M	1491	966	0.8037508	4	Singlet	Immune_cells
SAT_2M	1464	924	0.2043597	4	Singlet	Immune_cells
SAT_2M	1513	937	3.1496063	1	Singlet	Immune_cells
SAT_2M	1012	705	0.5847953	6	Singlet	Neurons
SAT_2M	3290	1596	1.8507282	10	Singlet	Immune_cells
SAT_2M	1423	899	2.5245442	1	Singlet	Immune_cells
SAT_2M	2212	1292	2.8378378	8	Singlet	Mesothelial
SAT_2M	2446	1299	10.938776	9	Singlet	Mesothelial
SAT_2M	1964	1165	0.8117707	3	Singlet	FAPs
SAT_2M	1485	892	0.3344482	4	Singlet	Immune_cells
SAT_2M	2292	1276	1.1759582	0	Singlet	Adipocyte
SAT_2M	1087	720	2.7372263	4	Singlet	Immune_cells
SAT_2M	2748	1446	3.5119479	3	Singlet	FAPs
SAT_2M	1717	1079	0.5235602	1	Singlet	Immune_cells
SAT_2M	1211	756	1.3147083	0	Singlet	Adipocyte
SAT_2M	1734	970	13.333333	1	Singlet	Immune_cells
SAT_2M	1147	788	3.0408341	5	Singlet	Immune_cells
SAT_2M	1902	1085	2.7792344	4	Singlet	Immune_cells
SAT_2M	1308	836	1.217656	1	Singlet	Immune_cells
SAT_2M	1317	852	7.1321321	5	Singlet	Immune_cells
SAT_2M	1857	1100	3.2637774	1	Singlet	Immune_cells
SAT_2M	1774	1175	0.8356546	4	Singlet	Immune_cells
SAT_2M	1227	750	0.8906883	1	Singlet	Immune_cells
SAT_2M	1848	1118	1.0248112	3	Singlet	FAPs
SAT_2M	1686	982	4.4404973	0	Singlet	Adipocyte
SAT_2M	1948	1139	1.9989749	4	Singlet	Immune_cells
SAT_2M	3708	1607	2.5020178	10	Singlet	Immune_cells
SAT_2M	2517	1417	0.5527043	0	Singlet	Adipocyte
SAT_2M	1328	791	6.4467766	1	Singlet	Immune_cells
SAT_2M	1460	853	1.5037594	0	Singlet	Adipocyte
SAT_2M	2131	1268	6.0465116	1	Singlet	Immune_cells
SAT_2M	2354	1283	4.5685279	0	Singlet	Adipocyte
SAT_2M	1276	767	4.1860465	1	Singlet	Immune_cells
SAT_2M	1004	648	4.6812749	1	Singlet	Immune_cells
SAT_2M	1404	876	0.9964413	4	Singlet	Immune_cells
SAT_2M	1410	758	4.8192771	5	Singlet	Immune_cells
SAT_2M	3074	1403	1.6866688	10	Singlet	Immune_cells
SAT_2M	1661	962	6.0587882	1	Singlet	Immune_cells

SAT_2M	1858	1058	4.887218	1	Singlet	Immune_cells
SAT_2M	1223	801	1.0551948	0	Singlet	Adipocyte
SAT_2M	1914	1112	0.9399478	0	Singlet	Adipocyte
SAT_2M	2935	1489	2.7541653	0	Singlet	Adipocyte
SAT_2M	1235	794	7.5	1	Singlet	Immune_cells
SAT_2M	1437	900	0.9722222	1	Singlet	Immune_cells
SAT_2M	1283	828	0.1547988	8	Singlet	Mesothelial
SAT_2M	1194	779	0.3338898	3	Singlet	FAPs
SAT_2M	1439	855	4.0858726	4	Singlet	Immune_cells
SAT_2M	1297	792	5.5895865	1	Singlet	Immune_cells
SAT_2M	1973	1198	6.703629	8	Singlet	Mesothelial
SAT_2M	1160	700	2.1551724	0	Singlet	Adipocyte
SAT_2M	1301	846	1.1494253	4	Singlet	Immune_cells
SAT_2M	1024	644	7.7073171	9	Singlet	Mesothelial
SAT_2M	1095	762	0.3633061	5	Singlet	Immune_cells
SAT_2M	1054	725	1.2287335	1	Singlet	Immune_cells
SAT_2M	3533	1885	5.7344633	5	Singlet	Immune_cells
SAT_2M	1032	709	1.1594203	3	Singlet	FAPs
SAT_2M	2134	1212	0.4659832	0	Singlet	Adipocyte
SAT_2M	1403	862	0.5661713	1	Singlet	Immune_cells
SAT_2M	1331	787	6.0015004	1	Singlet	Immune_cells
SAT_2M	1967	1192	2.5316456	3	Singlet	FAPs
SAT_2M	1208	769	0.0823723	0	Singlet	Adipocyte
SAT_2M	1959	1090	9.3272171	1	Singlet	Immune_cells
SAT_2M	1441	901	7.967033	1	Singlet	Immune_cells
SAT_2M	2094	1169	3.3333333	2	Singlet	FAPs
SAT_2M	1028	690	0.9661836	0	Singlet	Adipocyte
SAT_2M	1770	1114	6.1304837	1	Singlet	Immune_cells
SAT_2M	3496	1566	3.7913341	10	Singlet	Immune_cells
SAT_2M	1301	790	10.276074	9	Singlet	Mesothelial
SAT_2M	2931	1607	2.4539877	7	Singlet	Endothelial_cells
SAT_2M	1664	1037	1.497006	2	Singlet	FAPs
SAT_2M	2336	1352	1.9165247	1	Singlet	Immune_cells
SAT_2M	1286	838	2.1671827	1	Singlet	Immune_cells
SAT_2M	19351	5345	7.7605779	9	Singlet	Mesothelial
SAT_2M	1918	1101	4.6354167	9	Singlet	Mesothelial
SAT_2M	2396	1241	4.4185077	5	Singlet	Immune_cells
SAT_2M	1848	1046	1.0242588	0	Singlet	Adipocyte
SAT_2M	1423	837	8.0815179	9	Singlet	Mesothelial
SAT_2M	1228	772	0.9724473	1	Singlet	Immune_cells
SAT_2M	1218	812	3.1122031	1	Singlet	Immune_cells
SAT_2M	3419	1688	0.1751825	0	Singlet	Adipocyte
SAT_2M	1239	814	0.32	0	Singlet	Adipocyte
SAT_2M	2402	1455	1.8272425	7	Singlet	Endothelial_cells
SAT_2M	1016	712	1.7681729	1	Singlet	Immune_cells
SAT_2M	1500	967	0.5315615	4	Singlet	Immune_cells
SAT_2M	2280	1357	0.8304196	1	Singlet	Immune_cells
SAT_2M	3518	1830	0.7361268	0	Singlet	Adipocyte
SAT_2M	1614	1054	0.9264978	5	Singlet	Immune_cells
SAT_2M	1268	803	9.1549296	1	Singlet	Immune_cells
SAT_2M	1113	721	0.8035714	0	Singlet	Adipocyte

SAT_2M	1230	809	4.0387722	1	Singlet	Immune_cells
SAT_2M	1267	823	0.4698512	0	Singlet	Adipocyte
SAT_2M	1593	1032	0.8739076	4	Singlet	Immune_cells
SAT_2M	2556	1450	5.1831645	1	Singlet	Immune_cells
SAT_2M	1119	735	3.75	4	Singlet	Immune_cells
SAT_2M	1266	788	0.6299213	0	Singlet	Adipocyte
SAT_2M	1596	979	0.8104738	3	Singlet	FAPs
SAT_2M	1646	965	6.9174757	1	Singlet	Immune_cells
SAT_2M	1048	692	2.6590693	1	Singlet	Immune_cells
SAT_2M	1295	871	0.2302379	1	Singlet	Immune_cells
SAT_2M	1194	781	1.5780731	1	Singlet	Immune_cells
SAT_2M	1068	660	4.53284	0	Singlet	Adipocyte
SAT_2M	1224	847	0.4065041	5	Singlet	Immune_cells
SAT_2M	1292	762	0.4622496	1	Singlet	Immune_cells
SAT_2M	1196	734	0	4	Singlet	Immune_cells
SAT_2M	1085	723	0.4591368	4	Singlet	Immune_cells
SAT_2M	1453	913	3.6351166	5	Singlet	Immune_cells
SAT_2M	1294	792	0.308642	4	Singlet	Immune_cells
SAT_2M	1551	1004	5.3376206	1	Singlet	Immune_cells
SAT_2M	1735	998	2.2413793	1	Singlet	Immune_cells
SAT_2M	2019	1137	4.5767717	0	Singlet	Adipocyte
SAT_2M	1053	676	4.2573321	1	Singlet	Immune_cells
SAT_2M	1586	909	3.0094044	1	Singlet	Immune_cells
SAT_2M	1033	691	6.8965517	4	Singlet	Immune_cells
SAT_2M	1065	680	0.1867414	1	Singlet	Immune_cells
SAT_2M	1481	923	0.7397445	4	Singlet	Immune_cells
SAT_2M	1390	844	1.0043042	0	Singlet	Adipocyte
SAT_2M	1133	733	0.2643172	4	Singlet	Immune_cells
SAT_2M	2894	1637	0.2068252	3	Singlet	FAPs
SAT_2M	1394	804	9.4084105	1	Singlet	Immune_cells
SAT_2M	2009	1272	2.8051181	6	Singlet	Neurons
SAT_2M	2251	1305	0.3101462	11	Singlet	Smooth_muscle_cells
SAT_2M	3040	1537	0.0327761	11	Singlet	Smooth_muscle_cells
SAT_2M	2245	1092	5.8274021	10	Singlet	Immune_cells
SAT_2M	1067	698	4.1977612	1	Singlet	Immune_cells
SAT_2M	1398	885	0.4270463	3	Singlet	FAPs
SAT_2M	2021	1208	3.458498	1	Singlet	Immune_cells
SAT_2M	1328	844	1.7306245	1	Singlet	Immune_cells
SAT_2M	1127	731	0.9708738	1	Singlet	Immune_cells
SAT_2M	4029	1908	8.4552442	9	Singlet	Mesothelial
SAT_2M	1245	800	0.7980846	4	Singlet	Immune_cells
SAT_2M	1574	990	1.8999367	4	Singlet	Immune_cells
SAT_2M	1751	1134	1.5384615	9	Singlet	Mesothelial
SAT_2M	1351	917	0.9544787	7	Singlet	Endothelial_cells
SAT_2M	1719	1058	6.8945539	1	Singlet	Immune_cells
SAT_2M	1174	796	0.7601351	4	Singlet	Immune_cells
SAT_2M	2075	1171	0.3360538	0	Singlet	Adipocyte
SAT_2M	1492	944	0.596817	0	Singlet	Adipocyte
SAT_2M	1932	1087	2.4729521	1	Singlet	Immune_cells
SAT_2M	1703	1025	1.9894675	1	Singlet	Immune_cells
SAT_2M	6155	2849	6.610499	4	Singlet	Immune_cells

SAT_2M	2590	1360	7.0134875	5	Singlet	Immune_cells
SAT_2M	1834	1162	0.1627781	1	Singlet	Immune_cells
SAT_2M	1442	991	1.5172414	11	Singlet	Smooth_muscle_cells
SAT_2M	1502	966	1.7857143	1	Singlet	Immune_cells
SAT_2M	1244	852	5.3642914	1	Singlet	Immune_cells
SAT_2M	1120	740	0.177305	1	Singlet	Immune_cells
SAT_2M	2467	1276	10.741792	1	Singlet	Immune_cells
SAT_2M	1794	1117	4.6744574	4	Singlet	Immune_cells
SAT_2M	3891	1999	12.752881	4	Singlet	Immune_cells
SAT_2M	2034	1096	4.766537	1	Singlet	Immune_cells
SAT_2M	1946	1198	3.3333333	9	Singlet	Mesothelial
SAT_2M	5554	2756	2.8941219	9	Singlet	Mesothelial
SAT_2M	1341	889	0.5200594	1	Singlet	Immune_cells
SAT_2M	1876	1061	9.7014925	9	Singlet	Mesothelial
SAT_2M	2388	1284	8.7301587	1	Singlet	Immune_cells
SAT_2M	1167	717	1.4554795	4	Singlet	Immune_cells
SAT_2M	1233	733	11.588331	4	Singlet	Immune_cells
SAT_2M	1388	900	0.5025126	4	Singlet	Immune_cells
SAT_2M	1690	1019	10.691081	4	Singlet	Immune_cells
SAT_2M	1428	894	0.0697837	3	Singlet	FAPs
SAT_2M	1040	751	0.4789272	3	Singlet	FAPs
SAT_2M	7772	3411	12.498394	4	Singlet	Immune_cells
SAT_2M	1489	876	0.3340013	1	Singlet	Immune_cells
SAT_2M	1387	920	2.3689878	3	Singlet	FAPs
SAT_2M	1709	1095	0.4672897	4	Singlet	Immune_cells
SAT_2M	1333	909	2.0895522	7	Singlet	Endothelial_cells
SAT_2M	1693	1045	2.4160283	1	Singlet	Immune_cells
SAT_2M	1095	739	2.1857924	2	Singlet	FAPs
SAT_2M	1328	854	4.9475262	1	Singlet	Immune_cells
SAT_2M	1200	786	3.3305579	4	Singlet	Immune_cells
SAT_2M	1465	952	1.2960437	4	Singlet	Immune_cells
SAT_2M	2792	1523	0.2850018	0	Singlet	Adipocyte
SAT_2M	2024	1165	0.147929	0	Singlet	Adipocyte
SAT_2M	1378	852	2.8880866	1	Singlet	Immune_cells
SAT_2M	1224	800	0	1	Singlet	Immune_cells
SAT_2M	1823	1130	1.5803815	5	Singlet	Immune_cells
SAT_2M	1435	898	3.0991736	1	Singlet	Immune_cells
SAT_2M	2450	1309	0.0812348	0	Singlet	Adipocyte
SAT_2M	1384	860	2.8860029	1	Singlet	Immune_cells
SAT_2M	1249	769	7.154213	1	Singlet	Immune_cells
SAT_2M	1414	814	10.570825	1	Singlet	Immune_cells
SAT_2M	1387	847	3.0150754	3	Singlet	FAPs
SAT_2M	3578	1795	1.2820513	0	Singlet	Adipocyte
SAT_2M	2273	1347	6.7603161	1	Singlet	Immune_cells
SAT_2M	2576	1312	2.2033243	0	Singlet	Adipocyte
SAT_2M	4541	2362	2.7204914	9	Singlet	Mesothelial
SAT_2M	2085	1212	9.7142857	9	Singlet	Mesothelial
SAT_2M	1317	926	0.6828528	4	Singlet	Immune_cells
SAT_2M	1120	701	0.8007117	0	Singlet	Adipocyte
SAT_2M	1675	986	4.4073853	1	Singlet	Immune_cells
SAT_2M	1360	820	0.2197802	4	Singlet	Immune_cells

SAT_2M	5624	2689	4.7246892	9	Singlet	Mesothelial
SAT_2M	1559	981	3.9692702	1	Singlet	Immune_cells
SAT_2M	1223	838	0.324412	5	Singlet	Immune_cells
SAT_2M	2297	1277	1.0841284	0	Singlet	Adipocyte
SAT_2M	1722	965	9.8901099	1	Singlet	Immune_cells
SAT_2M	1201	814	0.5780347	1	Singlet	Immune_cells
SAT_2M	1234	712	8.3870968	1	Singlet	Immune_cells
SAT_2M	2301	1326	4.6400694	5	Singlet	Immune_cells
SAT_2M	1454	886	7.0596299	1	Singlet	Immune_cells
SAT_2M	1935	972	0.9292721	10	Singlet	Immune_cells
SAT_2M	1836	1099	2.8757461	1	Singlet	Immune_cells
SAT_2M	3764	1879	0.5291005	11	Singlet	Smooth_muscle_cells
SAT_2M	1223	790	0.5709625	1	Singlet	Immune_cells
SAT_2M	1058	753	0.2824859	1	Singlet	Immune_cells
SAT_2M	2167	1241	0.2754821	0	Singlet	Adipocyte
SAT_2M	1222	766	0.4088307	1	Singlet	Immune_cells
SAT_2M	2918	1487	6.2585499	9	Singlet	Mesothelial
SAT_2M	1782	1049	4.262479	1	Singlet	Immune_cells
SAT_2M	1002	673	2.3738872	0	Singlet	Adipocyte
SAT_2M	2442	1449	5.4878049	4	Singlet	Immune_cells
SAT_2M	1239	764	9.9113618	4	Singlet	Immune_cells
SAT_2M	1875	1077	4.250797	1	Singlet	Immune_cells
SAT_2M	1222	820	2.0424837	4	Singlet	Immune_cells
SAT_2M	1440	950	0.9715475	1	Singlet	Immune_cells
SAT_2M	1757	1105	3.2386364	4	Singlet	Immune_cells
SAT_2M	4106	1941	0.2431907	0	Singlet	Adipocyte
SAT_2M	1517	826	4.1967213	5	Singlet	Immune_cells
SAT_2M	1740	988	0.7995431	0	Singlet	Adipocyte
SAT_2M	1498	975	2.1319121	4	Singlet	Immune_cells
SAT_2M	1016	674	0.2918288	0	Singlet	Adipocyte
SAT_2M	2543	1358	4.8254217	5	Singlet	Immune_cells
SAT_2M	4356	1694	5.8661778	10	Singlet	Immune_cells
SAT_2M	1321	861	0.0750751	1	Singlet	Immune_cells
SAT_2M	1039	678	0.9587728	4	Singlet	Immune_cells
SAT_2M	1287	863	2.0849421	1	Singlet	Immune_cells
SAT_2M	1280	848	2.7993779	11	Singlet	Smooth_muscle_cells
SAT_2M	2660	1484	1.8024784	8	Singlet	Mesothelial
SAT_2M	2606	1386	7.9310345	1	Singlet	Immune_cells
SAT_2M	1263	829	4.6420142	1	Singlet	Immune_cells
SAT_2M	1492	844	14.390406	1	Singlet	Immune_cells
SAT_2M	1475	928	0.3369272	4	Singlet	Immune_cells
SAT_2M	1639	991	2.189781	1	Singlet	Immune_cells
SAT_2M	1229	760	8.0161943	1	Singlet	Immune_cells
SAT_2M	1005	702	0.4911591	6	Singlet	Neurons
SAT_2M	1214	726	7.3130649	5	Singlet	Immune_cells
SAT_2M	1106	771	4.5822102	1	Singlet	Immune_cells
SAT_2M	3042	1767	0.3927987	8	Singlet	Mesothelial
SAT_2M	1192	819	0.5794702	0	Singlet	Adipocyte
SAT_2M	1401	851	8.5592011	1	Singlet	Immune_cells
SAT_2M	1276	785	0.859375	0	Singlet	Adipocyte
SAT_2M	1350	841	0.5895357	1	Singlet	Immune_cells

SAT_2M	1187	694	0.6672227	1	Singlet	Immune_cells
SAT_2M	2101	1219	0.3311258	0	Singlet	Adipocyte
SAT_2M	1524	954	2.6902887	1	Singlet	Immune_cells
SAT_2M	1439	839	0.6925208	0	Singlet	Adipocyte
SAT_2M	2822	1515	8.5572843	1	Singlet	Immune_cells
SAT_2M	1523	1031	0.5204945	6	Singlet	Neurons
SAT_2M	2785	1566	0.5372493	0	Singlet	Adipocyte
SAT_2M	1324	875	6.4006024	1	Singlet	Immune_cells
SAT_2M	1275	758	11.18061	1	Singlet	Immune_cells
SAT_2M	2499	1369	1.3142174	0	Singlet	Adipocyte
SAT_2M	1304	793	0.8403361	1	Singlet	Immune_cells
SAT_2M	1064	718	0.5612722	0	Singlet	Adipocyte
SAT_2M	1958	1195	2.3350254	1	Singlet	Immune_cells
SAT_2M	1150	686	6.3203463	3	Singlet	FAPs
SAT_2M	1898	1117	0.5249344	4	Singlet	Immune_cells
SAT_2M	1405	885	6.01983	0	Singlet	Adipocyte
SAT_2M	1346	830	4.6460177	0	Singlet	Adipocyte
SAT_2M	1439	896	0.9715475	1	Singlet	Immune_cells
SAT_2M	1082	700	0.1829826	0	Singlet	Adipocyte
SAT_2M	1552	927	0.4490058	11	Singlet	Smooth_muscle_cells
SAT_2M	3955	1787	4.8016174	10	Singlet	Immune_cells
SAT_2M	1215	783	4.0749796	1	Singlet	Immune_cells
SAT_2M	1127	793	2.4626209	1	Singlet	Immune_cells
SAT_2M	1073	688	0.8372093	0	Singlet	Adipocyte
SAT_2M	1137	764	0.3496504	4	Singlet	Immune_cells
SAT_2M	1499	907	3.3244681	1	Singlet	Immune_cells
SAT_2M	1651	986	3.081571	1	Singlet	Immune_cells
SAT_2M	2230	1464	0.3125	4	Singlet	Immune_cells
SAT_2M	1219	813	1.6299919	4	Singlet	Immune_cells
SAT_2M	2175	1119	6.3273728	3	Singlet	FAPs
SAT_2M	2588	1397	1.2736395	1	Singlet	Immune_cells
SAT_2M	1400	886	0.1424501	0	Singlet	Adipocyte
SAT_2M	3024	1582	0.2303389	0	Singlet	Adipocyte
SAT_2M	1601	915	9.9564406	1	Singlet	Immune_cells
SAT_2M	1396	875	0.5698006	3	Singlet	FAPs
SAT_2M	1392	825	4.4412607	1	Singlet	Immune_cells
SAT_2M	1502	976	1.3262599	4	Singlet	Immune_cells
SAT_2M	1203	798	0.4115226	1	Singlet	Immune_cells
SAT_2M	1345	906	0.5939124	1	Singlet	Immune_cells
SAT_2M	2185	1293	1.7743403	5	Singlet	Immune_cells
SAT_2M	1140	773	2.6019081	1	Singlet	Immune_cells
SAT_2M	3252	1663	0.8277131	0	Singlet	Adipocyte
SAT_2M	2688	1438	0.3335804	0	Singlet	Adipocyte
SAT_2M	1529	967	2.1442495	1	Singlet	Immune_cells
SAT_2M	2633	1359	11.516748	1	Singlet	Immune_cells
SAT_2M	1845	1133	5.4054054	4	Singlet	Immune_cells
SAT_2M	2212	1150	5.32972	10	Singlet	Immune_cells
SAT_2M	1377	804	10.224801	1	Singlet	Immune_cells
SAT_2M	1141	748	0.3496504	1	Singlet	Immune_cells
SAT_2M	2265	1272	7.5297226	1	Singlet	Immune_cells
SAT_2M	1495	935	0.3342246	1	Singlet	Immune_cells

SAT_2M	1383	926	0.1444043	4	Singlet	Immune_cells
SAT_2M	1883	1109	5.6733828	9	Singlet	Mesothelial
SAT_2M	6786	2716	1.5004413	10	Singlet	Immune_cells
SAT_2M	1148	771	0.4344049	1	Singlet	Immune_cells
SAT_2M	1295	813	2.9141104	3	Singlet	FAPs
SAT_2M	1279	766	9.5015576	1	Singlet	Immune_cells
SAT_2M	1139	688	4.9694856	1	Singlet	Immune_cells
SAT_2M	1915	1201	2.4517475	2	Singlet	FAPs
SAT_2M	2000	1116	1.746507	5	Singlet	Immune_cells
SAT_2M	3588	1861	1.3915948	5	Singlet	Immune_cells
SAT_2M	2237	1292	0.1782531	0	Singlet	Adipocyte
SAT_2M	1266	803	0	4	Singlet	Immune_cells
SAT_2M	1313	826	8.973384	1	Singlet	Immune_cells
SAT_2M	2016	1146	2.4066798	0	Singlet	Adipocyte
SAT_2M	1577	903	7.7264091	1	Singlet	Immune_cells
SAT_2M	1436	933	0.6954103	3	Singlet	FAPs
SAT_2M	2064	1096	3.6319613	3	Singlet	FAPs
SAT_2M	2262	1315	2.553941	9	Singlet	Mesothelial
SAT_2M	1306	858	0.4562738	4	Singlet	Immune_cells
SAT_2M	1421	839	0.4908836	1	Singlet	Immune_cells
SAT_2M	1815	1155	0.9356081	4	Singlet	Immune_cells
SAT_2M	1566	962	0.5092298	1	Singlet	Immune_cells
SAT_2M	1852	1125	1.6693592	4	Singlet	Immune_cells
SAT_2M	1478	873	3.0384875	0	Singlet	Adipocyte
SAT_2M	1648	1022	4.6553809	5	Singlet	Immune_cells
SAT_2M	2080	1196	0.3350886	0	Singlet	Adipocyte
SAT_2M	1556	772	8.8518281	10	Singlet	Immune_cells
SAT_2M	2347	1345	0.2969877	8	Singlet	Mesothelial
SAT_2M	1125	761	4.8330404	1	Singlet	Immune_cells
SAT_2M	1016	692	0.3883495	9	Singlet	Mesothelial
SAT_2M	2223	1216	3.1305903	0	Singlet	Adipocyte
SAT_2M	1081	694	0.5484461	1	Singlet	Immune_cells
SAT_2M	1180	764	0.7614213	3	Singlet	FAPs
SAT_2M	1187	777	0.5867561	1	Singlet	Immune_cells
SAT_2M	1308	859	3.5034273	1	Singlet	Immune_cells
SAT_2M	1621	1010	2.2727273	4	Singlet	Immune_cells
SAT_2M	4038	1866	0.3201182	0	Singlet	Adipocyte
SAT_2M	1034	711	0.0963391	1	Singlet	Immune_cells
SAT_2M	1423	901	0	3	Singlet	FAPs
SAT_2M	1413	871	2.8975265	4	Singlet	Immune_cells
SAT_2M	2164	1191	2.8965517	0	Singlet	Adipocyte
SAT_2M	2196	1275	0.3631412	0	Singlet	Adipocyte
SAT_2M	1449	889	1.1716058	0	Singlet	Adipocyte
SAT_2M	1681	994	1.2389381	5	Singlet	Immune_cells
SAT_2M	1588	1011	0.0625391	6	Singlet	Neurons
SAT_2M	1020	672	1.6456922	0	Singlet	Adipocyte
SAT_2M	1266	821	3.3044847	1	Singlet	Immune_cells
SAT_2M	1525	905	2.8013029	1	Singlet	Immune_cells
SAT_2M	1387	880	4.7925608	4	Singlet	Immune_cells
SAT_2M	1684	1028	0.2357101	0	Singlet	Adipocyte
SAT_2M	5651	2394	0.7937908	10	Singlet	Immune_cells

SAT_2M	1012	684	1.8700787	1	Singlet	Immune_cells
SAT_2M	1761	1090	4.5377198	4	Singlet	Immune_cells
SAT_2M	1182	787	0.926706	4	Singlet	Immune_cells
SAT_2M	1149	792	0.3457217	4	Singlet	Immune_cells
SAT_2M	2188	1257	3.4655723	4	Singlet	Immune_cells
SAT_2M	2046	1288	0.78125	4	Singlet	Immune_cells
SAT_2M	1422	887	0.9090909	7	Singlet	Endothelial_cells
SAT_2M	1848	1139	6.3140853	1	Singlet	Immune_cells
SAT_2M	1532	1014	1.0430248	4	Singlet	Immune_cells
SAT_2M	1243	817	5.2208835	1	Singlet	Immune_cells
SAT_2M	3931	1729	4.3390003	10	Singlet	Immune_cells
SAT_2M	2973	1711	0.1341382	3	Singlet	FAPs
SAT_2M	1335	871	5.754858	1	Singlet	Immune_cells
SAT_2M	1118	661	8.2887701	9	Singlet	Mesothelial
SAT_2M	1029	630	6.122449	0	Singlet	Adipocyte
SAT_2M	2123	1216	0.8423023	0	Singlet	Adipocyte
SAT_2M	1174	795	1.5293118	4	Singlet	Immune_cells
SAT_2M	1243	835	0.0798722	4	Singlet	Immune_cells
SAT_2M	2747	1490	2.2037572	0	Singlet	Adipocyte
SAT_2M	3859	1817	0.232438	0	Singlet	Adipocyte
SAT_2M	3396	1678	0.5284792	0	Singlet	Adipocyte
SAT_2M	1966	1156	0.2534212	0	Singlet	Adipocyte
SAT_2M	1208	800	1.1541632	1	Singlet	Immune_cells
SAT_2M	1236	848	0.3980892	6	Singlet	Neurons
SAT_2M	1609	986	0.3097893	0	Singlet	Adipocyte
SAT_2M	1321	833	0.4521477	0	Singlet	Adipocyte
SAT_2M	2857	1250	3.9496679	10	Singlet	Immune_cells
SAT_2M	2578	1387	1.5063731	0	Singlet	Adipocyte
SAT_2M	1253	823	4.9363057	1	Singlet	Immune_cells
SAT_2M	1441	960	0.6219765	4	Singlet	Immune_cells
SAT_2M	1735	1116	0.5178366	1	Singlet	Immune_cells
SAT_2M	1033	674	0.3872217	6	Singlet	Neurons
SAT_2M	1128	738	0.8833922	4	Singlet	Immune_cells
SAT_2M	2453	1346	1.4985824	0	Singlet	Adipocyte
SAT_2M	1007	662	1.0912698	4	Singlet	Immune_cells
SAT_2M	1278	844	4.140625	1	Singlet	Immune_cells
SAT_2M	4854	2415	10.441932	4	Singlet	Immune_cells
SAT_2M	4802	2205	11.060291	9	Singlet	Mesothelial
SAT_2M	1298	845	3.5222052	1	Singlet	Immune_cells
SAT_2M	1263	841	0.3943218	1	Singlet	Immune_cells
SAT_2M	1752	1102	0.2844141	5	Singlet	Immune_cells
SAT_2M	1006	631	3.5749752	5	Singlet	Immune_cells
SAT_2M	4403	2112	0.2486438	0	Singlet	Adipocyte
SAT_2M	1263	798	5.5248619	1	Singlet	Immune_cells
SAT_2M	1600	1002	3.6204744	10	Singlet	Immune_cells
SAT_2M	1618	1026	1.1117974	4	Singlet	Immune_cells
SAT_2M	1247	767	0.5586592	1	Singlet	Immune_cells
SAT_2M	1399	844	2.2824536	1	Singlet	Immune_cells
SAT_2M	1636	1012	3.8344492	8	Singlet	Mesothelial
SAT_2M	2829	1425	9.5875925	9	Singlet	Mesothelial
SAT_2M	1470	897	6.6530889	4	Singlet	Immune_cells

SAT_2M	1535	956	1.0403121	1	Singlet	Immune_cells
SAT_2M	2136	1310	7.8285182	4	Singlet	Immune_cells
SAT_2M	2149	1246	1.4883721	10	Singlet	Immune_cells
SAT_2M	1626	1034	1.9619865	1	Singlet	Immune_cells
SAT_2M	1230	768	5.7676686	1	Singlet	Immune_cells
SAT_2M	1361	826	6.2408223	1	Singlet	Immune_cells
SAT_2M	2598	1516	0.5760369	2	Singlet	FAPs
SAT_2M	1672	1045	3.641791	1	Singlet	Immune_cells
SAT_2M	4388	2142	1.4068527	2	Singlet	FAPs
SAT_2M	1377	861	5.2098408	1	Singlet	Immune_cells
SAT_2M	3842	1651	1.4025974	10	Singlet	Immune_cells
SAT_2M	1333	844	2.2488756	7	Singlet	Endothelial_cells
SAT_2M	1445	946	1.2405238	4	Singlet	Immune_cells
SAT_2M	4056	1829	0.4174853	10	Singlet	Immune_cells
SAT_2M	1119	723	2.6785714	4	Singlet	Immune_cells
SAT_2M	1229	808	0.2411576	1	Singlet	Immune_cells
SAT_2M	1961	1206	0.4058853	0	Singlet	Adipocyte
SAT_2M	1694	1019	1.2309496	1	Singlet	Immune_cells
SAT_2M	1191	779	6.0251046	1	Singlet	Immune_cells
SAT_2M	1420	882	3.0133146	1	Singlet	Immune_cells
SAT_2M	5481	2632	6.0280459	9	Singlet	Mesothelial
SAT_2M	1812	1068	1.4332966	1	Singlet	Immune_cells
SAT_2M	1605	969	1.0552452	0	Singlet	Adipocyte
SAT_2M	1716	1056	3.372093	4	Singlet	Immune_cells
SAT_2M	1623	1041	0.1839362	0	Singlet	Adipocyte
SAT_2M	1307	852	0.2286585	4	Singlet	Immune_cells
SAT_2M	1027	687	2.526725	4	Singlet	Immune_cells
SAT_2M	1394	794	8.6366881	3	Singlet	FAPs
SAT_2M	1090	714	0.9140768	4	Singlet	Immune_cells
SAT_2M	1818	1099	3.5557987	4	Singlet	Immune_cells
SAT_2M	1007	701	1.2820513	4	Singlet	Immune_cells
SAT_2M	1536	920	0.3246753	3	Singlet	FAPs
SAT_2M	1068	695	1.1173184	6	Singlet	Neurons
SAT_2M	1787	1059	8.2681564	0	Singlet	Adipocyte
SAT_2M	1221	705	10.956664	0	Singlet	Adipocyte
SAT_2M	1157	818	0.0856164	11	Singlet	Smooth_muscle_cells
SAT_2M	1543	942	8.7741935	1	Singlet	Immune_cells
SAT_2M	1617	1030	0.1227747	8	Singlet	Mesothelial
SAT_2M	1008	701	0.1982161	1	Singlet	Immune_cells
SAT_2M	1465	896	1.8329939	1	Singlet	Immune_cells
SAT_2M	1122	750	0.1776199	3	Singlet	FAPs
SAT_2M	1428	966	2.5783972	1	Singlet	Immune_cells
SAT_2M	1025	736	1.352657	11	Singlet	Smooth_muscle_cells
SAT_2M	1050	716	1.994302	11	Singlet	Smooth_muscle_cells
SAT_2M	1816	1157	3.8398245	2	Singlet	FAPs
SAT_2M	1121	755	0.3552398	0	Singlet	Adipocyte
SAT_2M	1258	769	0.2377179	0	Singlet	Adipocyte
SAT_2M	1848	1040	9.7297297	9	Singlet	Mesothelial
SAT_2M	1334	855	0.1492537	0	Singlet	Adipocyte
SAT_2M	1618	1046	0.6790123	5	Singlet	Immune_cells
SAT_2M	1292	878	0.6955178	4	Singlet	Immune_cells

SAT_2M	1251	800	4.7770701	1	Singlet	Immune_cells
SAT_2M	1515	901	1.2483574	4	Singlet	Immune_cells
SAT_2M	1472	905	5.0709939	1	Singlet	Immune_cells
SAT_2M	4028	1925	0.3711952	0	Singlet	Adipocyte
SAT_2M	1338	906	1.2620638	4	Singlet	Immune_cells
SAT_2M	2540	1305	2.5933202	5	Singlet	Immune_cells
SAT_2M	1574	1023	1.0139417	4	Singlet	Immune_cells
SAT_2M	2012	1109	7.7799802	9	Singlet	Mesothelial
SAT_2M	1232	774	3.4789644	1	Singlet	Immune_cells
SAT_2M	1143	769	0.2620087	8	Singlet	Mesothelial
SAT_2M	1266	852	4.3273013	4	Singlet	Immune_cells
SAT_2M	2423	1372	2.5777414	6	Singlet	Neurons
SAT_2M	1232	836	2.0242915	1	Singlet	Immune_cells
SAT_2M	1355	797	8.4621045	9	Singlet	Mesothelial
SAT_2M	6960	2327	1.5338303	10	Singlet	Immune_cells
SAT_2M	1441	887	2.4187975	1	Singlet	Immune_cells
SAT_2M	1009	703	1.7786561	11	Singlet	Smooth_muscle_cells
SAT_2M	2545	1399	0.3525264	0	Singlet	Adipocyte
SAT_2M	1926	1145	0.6185567	0	Singlet	Adipocyte
SAT_2M	2325	1361	4.5064378	4	Singlet	Immune_cells
SAT_2M	1025	681	2.3346304	1	Singlet	Immune_cells
SAT_2M	1330	806	0.9737828	4	Singlet	Immune_cells
SAT_2M	1114	763	0.2683363	2	Singlet	FAPs
SAT_2M	1596	949	9.3241552	1	Singlet	Immune_cells
SAT_2M	1282	848	4.7360248	1	Singlet	Immune_cells
SAT_2M	1387	883	6.6044508	1	Singlet	Immune_cells
SAT_2M	1222	802	5.9494703	1	Singlet	Immune_cells
SAT_2M	2339	1329	2.7268854	1	Singlet	Immune_cells
SAT_2M	1429	890	2.3743017	1	Singlet	Immune_cells
SAT_2M	4795	2260	4.1172801	4	Singlet	Immune_cells
SAT_2M	2110	1167	6.3297119	1	Singlet	Immune_cells
SAT_2M	1275	827	2.1875	11	Singlet	Smooth_muscle_cells
SAT_2M	2303	1258	0.216544	3	Singlet	FAPs
SAT_2M	1389	893	0.3589375	8	Singlet	Mesothelial
SAT_2M	5293	2509	5.1847662	9	Singlet	Mesothelial
SAT_2M	1049	690	0.1906578	0	Singlet	Adipocyte
SAT_2M	1562	955	0.8939974	1	Singlet	Immune_cells
SAT_2M	1704	1048	1.2850467	0	Singlet	Adipocyte
SAT_2M	4212	1866	2.3387668	10	Singlet	Immune_cells
SAT_2M	1242	784	4.5746388	1	Singlet	Immune_cells
SAT_2M	3021	1616	0.330033	0	Singlet	Adipocyte
SAT_2M	2465	1378	0.645682	0	Singlet	Adipocyte
SAT_2M	1959	1128	3.9634146	0	Singlet	Adipocyte
SAT_2M	1016	672	0.5876592	4	Singlet	Immune_cells
SAT_2M	1266	857	0.8661417	1	Singlet	Immune_cells
SAT_2M	1673	968	7.8571429	1	Singlet	Immune_cells
SAT_2M	1218	801	0.9787928	0	Singlet	Adipocyte
SAT_2M	4169	2134	1.3161043	11	Singlet	Smooth_muscle_cells
SAT_2M	2542	1463	0.7442225	11	Singlet	Smooth_muscle_cells
SAT_2M	1313	844	2.8809704	1	Singlet	Immune_cells
SAT_2M	2247	1060	9.7604259	3	Singlet	FAPs

SAT_2M	1183	797	0.1683502	0	Singlet	Adipocyte
SAT_2M	1047	687	3.8058991	4	Singlet	Immune_cells
SAT_2M	2037	1233	1.1741683	1	Singlet	Immune_cells
SAT_2M	1057	725	0.7532957	5	Singlet	Immune_cells
SAT_2M	1254	827	0.2384738	4	Singlet	Immune_cells
SAT_2M	1349	821	8.4008843	1	Singlet	Immune_cells
SAT_2M	1444	1009	1.0951403	8	Singlet	Mesothelial
SAT_2M	2513	1434	3.6139793	1	Singlet	Immune_cells
SAT_2M	1596	980	3.320802	1	Singlet	Immune_cells
SAT_2M	1546	909	0.064433	10	Singlet	Immune_cells
SAT_2M	1253	807	2.4603175	8	Singlet	Mesothelial
SAT_2M	1619	1005	0.0613497	4	Singlet	Immune_cells
SAT_2M	1225	799	3.493095	1	Singlet	Immune_cells
SAT_2M	3169	1642	0.4094488	11	Singlet	Smooth_muscle_cells
SAT_2M	1338	864	1.7176998	1	Singlet	Immune_cells
SAT_2M	1910	1140	0.3636364	0	Singlet	Adipocyte
SAT_2M	1017	693	6.1643836	4	Singlet	Immune_cells
SAT_2M	1476	825	8.7897228	1	Singlet	Immune_cells
SAT_2M	1857	1033	7.1734475	1	Singlet	Immune_cells
SAT_2M	1366	840	0.5847953	1	Singlet	Immune_cells
SAT_2M	1748	1088	0.1136364	11	Singlet	Smooth_muscle_cells
SAT_2M	1492	1017	0.7323569	1	Singlet	Immune_cells
SAT_2M	1575	909	7.3371284	1	Singlet	Immune_cells
SAT_2M	2387	1371	0.8347245	0	Singlet	Adipocyte
SAT_2M	3002	1586	8.2141669	4	Singlet	Immune_cells
SAT_2M	4251	2152	5.4720526	8	Singlet	Mesothelial
SAT_2M	1868	1042	6.1497326	1	Singlet	Immune_cells
SAT_2M	1243	780	1.3632719	4	Singlet	Immune_cells
SAT_2M	4249	2031	0.4691532	0	Singlet	Adipocyte
SAT_2M	2142	1228	0.3242242	0	Singlet	Adipocyte
SAT_2M	1343	903	1.4880952	1	Singlet	Immune_cells
SAT_2M	1871	1191	1.3800425	11	Singlet	Smooth_muscle_cells
SAT_2M	2368	1285	1.137321	0	Singlet	Adipocyte
SAT_2M	2218	1219	6.1911171	4	Singlet	Immune_cells
SAT_2M	1908	1180	1.7259414	4	Singlet	Immune_cells
SAT_2M	2730	1537	0.2550091	0	Singlet	Adipocyte
SAT_2M	1302	757	1.0695187	7	Singlet	Endothelial_cells
SAT_2M	4548	2045	1.0309278	10	Singlet	Immune_cells
SAT_2M	1550	938	5.3949904	1	Singlet	Immune_cells
SAT_2M	2275	1295	7.3011734	5	Singlet	Immune_cells
SAT_2M	1630	1050	2.490887	0	Singlet	Adipocyte
SAT_2M	1165	720	2.5706941	0	Singlet	Adipocyte
SAT_2M	1035	655	0.6743738	4	Singlet	Immune_cells
SAT_2M	1213	801	2.2094926	5	Singlet	Immune_cells
SAT_2M	1627	971	1.3431013	0	Singlet	Adipocyte
SAT_2M	1713	990	8.1049563	1	Singlet	Immune_cells
SAT_2M	1256	774	1.9669552	3	Singlet	FAPs
SAT_2M	1490	951	3.1375167	1	Singlet	Immune_cells
SAT_2M	1396	857	0.21398	0	Singlet	Adipocyte
SAT_2M	1615	1008	0.3705991	11	Singlet	Smooth_muscle_cells
SAT_2M	4460	1908	0.8950548	10	Singlet	Immune_cells

SAT_2M	1745	1029	0.3957038	0	Singlet	Adipocyte
SAT_2M	1420	934	1.0445682	3	Singlet	FAPs
SAT_2M	1065	699	0	11	Singlet	Smooth_muscle_cells
SAT_2M	1331	861	0.2987304	5	Singlet	Immune_cells
SAT_2M	5857	2539	0.1360313	0	Singlet	Adipocyte
SAT_2M	2298	1289	0.7826087	3	Singlet	FAPs
SAT_2M	1664	944	9.760479	1	Singlet	Immune_cells
SAT_2M	2010	1241	0.8919723	9	Singlet	Mesothelial
SAT_2M	1392	861	5.3724928	1	Singlet	Immune_cells
SAT_2M	1592	1023	1.0598504	1	Singlet	Immune_cells
SAT_2M	3573	1769	6.6945607	9	Singlet	Mesothelial
SAT_2M	3379	1631	4.5172719	1	Singlet	Immune_cells
SAT_2M	1549	949	0.9633911	4	Singlet	Immune_cells
SAT_2M	1338	849	1.1185682	1	Singlet	Immune_cells
SAT_2M	3029	1617	3.5985474	1	Singlet	Immune_cells
SAT_2M	1532	949	3.1838856	1	Singlet	Immune_cells
SAT_2M	1023	687	5.4966249	1	Singlet	Immune_cells
SAT_2M	4932	2111	6.6343042	9	Singlet	Mesothelial
SAT_2M	2558	1409	0.2341007	0	Singlet	Adipocyte
SAT_2M	2090	1261	0.2847651	4	Singlet	Immune_cells
SAT_2M	1297	830	0.5347594	0	Singlet	Adipocyte
SAT_2M	2452	1290	8.7912088	1	Singlet	Immune_cells
SAT_2M	1661	973	0.5988024	4	Singlet	Immune_cells
SAT_2M	1848	1042	5.9331176	4	Singlet	Immune_cells
SAT_2M	1231	832	0.7258065	4	Singlet	Immune_cells
SAT_2M	1520	874	6.9599475	8	Singlet	Mesothelial
SAT_2M	1112	793	0.2688172	1	Singlet	Immune_cells
SAT_2M	2172	1227	7.7838828	1	Singlet	Immune_cells
SAT_2M	2312	1352	0.0859107	6	Singlet	Neurons
SAT_2M	1640	980	1.0309278	0	Singlet	Adipocyte
SAT_2M	1460	976	1.0217984	1	Singlet	Immune_cells
SAT_2M	1082	649	8.0406654	0	Singlet	Adipocyte
SAT_2M	1200	790	2.2350993	1	Singlet	Immune_cells
SAT_2M	1991	1172	3.8825286	1	Singlet	Immune_cells
SAT_2M	1718	1077	0.6391633	1	Singlet	Immune_cells
SAT_2M	1543	858	0.1899937	6	Singlet	Neurons
SAT_2M	1884	1051	2.065678	10	Singlet	Immune_cells
SAT_2M	2001	1227	0.5979073	11	Singlet	Smooth_muscle_cells
SAT_2M	1470	859	6.4276049	6	Singlet	Neurons
SAT_2M	1532	975	1.4267185	0	Singlet	Adipocyte
SAT_2M	1586	949	5.6532663	4	Singlet	Immune_cells
SAT_2M	1781	1017	3.7535014	1	Singlet	Immune_cells
SAT_2M	5495	2722	5.7179161	9	Singlet	Mesothelial
SAT_2M	1457	907	0.8898015	4	Singlet	Immune_cells
SAT_2M	1329	847	0.0749064	1	Singlet	Immune_cells
SAT_2M	1554	994	0.2570694	4	Singlet	Immune_cells
SAT_2M	1081	702	1.3799448	4	Singlet	Immune_cells
SAT_2M	6189	2448	3.5979348	10	Singlet	Immune_cells
SAT_2M	1769	1051	0.2820079	4	Singlet	Immune_cells
SAT_2M	1123	759	0.4436557	4	Singlet	Immune_cells
SAT_2M	1008	645	1.1846002	1	Singlet	Immune_cells

SAT_2M	1416	867	12.350035	1	Singlet	Immune_cells
SAT_2M	1962	1169	0	8	Singlet	Mesothelial
SAT_2M	1283	831	0.3881988	0	Singlet	Adipocyte
SAT_2M	2856	1596	5.7759221	4	Singlet	Immune_cells
SAT_2M	1474	971	3.4552846	4	Singlet	Immune_cells
SAT_2M	1868	938	8.5026738	0	Singlet	Adipocyte
SAT_2M	1448	834	5.5785124	1	Singlet	Immune_cells
SAT_2M	1369	829	4.8762737	1	Singlet	Immune_cells
SAT_2M	1275	764	2.1840874	5	Singlet	Immune_cells
SAT_2M	2301	1405	2.3326134	4	Singlet	Immune_cells
SAT_2M	2207	1236	6.4050519	1	Singlet	Immune_cells
SAT_2M	1295	820	0.5384615	0	Singlet	Adipocyte
SAT_2M	1960	1190	2.5980642	1	Singlet	Immune_cells
SAT_2M	1697	976	3.0570253	1	Singlet	Immune_cells
SAT_2M	3341	1477	2.7446301	10	Singlet	Immune_cells
SAT_2M	1043	671	4.9760766	4	Singlet	Immune_cells
SAT_2M	1552	847	8.7820513	5	Singlet	Immune_cells
SAT_2M	1445	871	0.4118051	0	Singlet	Adipocyte
SAT_2M	1574	915	2.0189274	3	Singlet	FAPs
SAT_2M	4009	2067	5.2945563	5	Singlet	Immune_cells
SAT_2M	1260	842	0.8695652	1	Singlet	Immune_cells
SAT_2M	1307	824	8.3079268	1	Singlet	Immune_cells
SAT_2M	2332	1357	0.2997859	4	Singlet	Immune_cells
SAT_2M	1450	875	2.3415978	1	Singlet	Immune_cells
SAT_2M	1233	796	0.5649718	1	Singlet	Immune_cells
SAT_2M	1594	968	7.4328545	4	Singlet	Immune_cells
SAT_2M	3025	1380	7.4695624	10	Singlet	Immune_cells
SAT_2M	1944	1151	6.6052227	1	Singlet	Immune_cells
SAT_2M	1542	966	1.0981912	1	Singlet	Immune_cells
SAT_2M	1386	830	8.5858586	1	Singlet	Immune_cells
SAT_2M	1093	708	5.7534247	8	Singlet	Mesothelial
SAT_2M	2288	1254	13.141862	1	Singlet	Immune_cells
SAT_2M	2147	1306	0.4618938	4	Singlet	Immune_cells
SAT_2M	1228	763	11.941511	5	Singlet	Immune_cells
SAT_2M	2230	1184	8.0197133	10	Singlet	Immune_cells
SAT_2M	1371	765	8.8064047	0	Singlet	Adipocyte
SAT_2M	1120	794	1.7761989	7	Singlet	Endothelial_cells
SAT_2M	1820	1088	2.6790596	5	Singlet	Immune_cells
SAT_2M	1217	789	3.5977105	4	Singlet	Immune_cells
SAT_2M	1670	1055	2.202381	1	Singlet	Immune_cells
SAT_2M	4250	1950	0.2579737	0	Singlet	Adipocyte
SAT_2M	1102	723	0.4524887	4	Singlet	Immune_cells
SAT_2M	1536	935	0.5844156	4	Singlet	Immune_cells
SAT_2M	1065	645	0.4655493	3	Singlet	FAPs
SAT_2M	2067	1240	0.3381643	0	Singlet	Adipocyte
SAT_2M	3263	1662	0.4881025	0	Singlet	Adipocyte
SAT_2M	1461	981	2.503383	4	Singlet	Immune_cells
SAT_2M	1297	785	4.2177914	1	Singlet	Immune_cells
SAT_2M	1176	771	0.508044	4	Singlet	Immune_cells
SAT_2M	1081	774	2.7598896	1	Singlet	Immune_cells
SAT_2M	1151	762	0.952381	4	Singlet	Immune_cells

SAT_2M	1621	1004	5.4301403	1	Singlet	Immune_cells
SAT_2M	6457	2923	4.5644438	9	Singlet	Mesothelial
SAT_2M	1193	837	0.4173623	11	Singlet	Smooth_muscle_cells
SAT_2M	2671	1435	0.1120657	0	Singlet	Adipocyte
SAT_2M	3295	1185	0.9399636	10	Singlet	Immune_cells
SAT_2M	1396	816	10.6201	0	Singlet	Adipocyte
SAT_2M	1449	906	3.024055	4	Singlet	Immune_cells
SAT_2M	1122	729	2.2222222	1	Singlet	Immune_cells
SAT_2M	1451	936	0.2061856	1	Singlet	Immune_cells
SAT_2M	1840	1027	4.7593294	0	Singlet	Adipocyte
SAT_2M	1178	781	0.5932203	9	Singlet	Mesothelial
SAT_2M	1512	968	0.9198423	1	Singlet	Immune_cells
SAT_2M	1954	1161	6.6428206	5	Singlet	Immune_cells
SAT_2M	1191	754	0.1663894	0	Singlet	Adipocyte
SAT_2M	1798	1073	0.4994451	2	Singlet	FAPs
SAT_2M	1626	1013	0.9107468	0	Singlet	Adipocyte
SAT_2M	1789	973	5.5586437	1	Singlet	Immune_cells
SAT_2M	1918	1172	1.7027864	6	Singlet	Neurons
SAT_2M	1556	851	3.2133676	10	Singlet	Immune_cells
SAT_2M	1031	676	1.0617761	4	Singlet	Immune_cells
SAT_2M	1358	847	4.981685	1	Singlet	Immune_cells
SAT_2M	1112	684	2.8699552	0	Singlet	Adipocyte
SAT_2M	1308	852	3.6557502	1	Singlet	Immune_cells
SAT_2M	2646	1431	3.2015066	1	Singlet	Immune_cells
SAT_2M	1692	1000	1.3545347	1	Singlet	Immune_cells
SAT_2M	1278	808	4.9960968	1	Singlet	Immune_cells
SAT_2M	1408	883	2.6969482	1	Singlet	Immune_cells
SAT_2M	1042	657	0.6666667	0	Singlet	Adipocyte
SAT_2M	3199	1690	2.3371767	8	Singlet	Mesothelial
SAT_2M	1282	797	5.982906	1	Singlet	Immune_cells
SAT_2M	1146	743	0.7765315	4	Singlet	Immune_cells
SAT_2M	1568	972	1.0094637	1	Singlet	Immune_cells
SAT_2M	1182	828	0.6728343	5	Singlet	Immune_cells
SAT_2M	1756	1098	0.2832861	1	Singlet	Immune_cells
SAT_2M	2029	1199	3.5907526	10	Singlet	Immune_cells
SAT_2M	2610	1477	0.6097561	0	Singlet	Adipocyte
SAT_2M	1139	669	2.0087336	3	Singlet	FAPs
SAT_2M	1520	944	1.5758372	4	Singlet	Immune_cells
SAT_2M	1449	817	6.2715369	1	Singlet	Immune_cells
SAT_2M	1671	1020	7.2152654	1	Singlet	Immune_cells
SAT_2M	1124	769	0.3536693	0	Singlet	Adipocyte
SAT_2M	1163	813	0.5097706	1	Singlet	Immune_cells
SAT_2M	2109	1198	0.8045433	3	Singlet	FAPs
SAT_2M	2163	1309	3.5961272	4	Singlet	Immune_cells
SAT_2M	1852	1161	3.7176724	5	Singlet	Immune_cells
SAT_2M	1686	1053	1.1764706	1	Singlet	Immune_cells
SAT_2M	1324	848	0.4524887	1	Singlet	Immune_cells
SAT_2M	2167	1286	0.5960569	4	Singlet	Immune_cells
SAT_2M	2206	1231	0.4972875	0	Singlet	Adipocyte
SAT_2M	2470	1397	0.4437273	8	Singlet	Mesothelial
SAT_2M	1820	1035	6.1235648	4	Singlet	Immune_cells

SAT_2M	1598	957	8.0524345	1	Singlet	Immune_cells
SAT_2M	1752	1105	0.3977273	1	Singlet	Immune_cells
SAT_2M	2224	1353	0.5829596	0	Singlet	Adipocyte
SAT_2M	1177	780	0.58873	0	Singlet	Adipocyte
SAT_2M	1068	706	6.4007421	1	Singlet	Immune_cells
SAT_2M	1453	930	0.7544582	4	Singlet	Immune_cells
SAT_2M	1415	927	4.4086774	5	Singlet	Immune_cells
SAT_2M	6245	2439	1.1653895	10	Singlet	Immune_cells
SAT_2M	1353	882	5.2475979	4	Singlet	Immune_cells
SAT_2M	1292	834	1.006192	4	Singlet	Immune_cells
SAT_2M	4337	2086	0.2065168	0	Singlet	Adipocyte
SAT_2M	1163	725	0.1713796	0	Singlet	Adipocyte
SAT_2M	2108	1214	0.8506616	0	Singlet	Adipocyte
SAT_2M	1157	697	1.8134715	1	Singlet	Immune_cells
SAT_2M	1296	848	0.6163328	4	Singlet	Immune_cells
SAT_2M	1282	778	1.1609907	1	Singlet	Immune_cells
SAT_2M	1294	791	0.3076923	0	Singlet	Adipocyte
SAT_2M	2195	1228	0.4083485	0	Singlet	Adipocyte
SAT_2M	1085	735	4.5413261	1	Singlet	Immune_cells
SAT_2M	1497	928	1.3990673	1	Singlet	Immune_cells
SAT_2M	1462	942	4.2205582	8	Singlet	Mesothelial
SAT_2M	1123	676	0.530504	6	Singlet	Neurons
SAT_2M	1041	679	7.2380952	1	Singlet	Immune_cells
SAT_2M	1475	965	3.3624748	8	Singlet	Mesothelial
SAT_2M	1063	682	0.9380863	4	Singlet	Immune_cells
SAT_2M	1267	820	6.0534591	4	Singlet	Immune_cells
SAT_2M	1016	639	2.1632252	7	Singlet	Endothelial_cells
SAT_2M	1270	813	1.5540016	4	Singlet	Immune_cells
SAT_2M	2064	1180	4.6859903	1	Singlet	Immune_cells
SAT_2M	1819	1171	1.3691128	4	Singlet	Immune_cells
SAT_2M	1137	741	0.1748252	4	Singlet	Immune_cells
SAT_2M	1436	938	0.3477051	4	Singlet	Immune_cells
SAT_2M	1170	756	0.170068	3	Singlet	FAPs
SAT_2M	1705	1068	3.9204213	1	Singlet	Immune_cells
SAT_2M	1363	847	0.0732064	1	Singlet	Immune_cells
SAT_2M	1664	983	1.7899761	1	Singlet	Immune_cells
SAT_2M	1212	795	2.707137	1	Singlet	Immune_cells
SAT_2M	1288	795	4.0989946	1	Singlet	Immune_cells
SAT_2M	1092	726	1.9195612	4	Singlet	Immune_cells
SAT_2M	1414	887	0	0	Singlet	Adipocyte
SAT_2M	1591	960	2.5529265	1	Singlet	Immune_cells
SAT_2M	1951	1172	1.3306039	1	Singlet	Immune_cells
SAT_2M	1633	996	0.304878	3	Singlet	FAPs
SAT_2M	1266	754	4.570528	1	Singlet	Immune_cells
SAT_2M	1225	869	0.4022526	1	Singlet	Immune_cells
SAT_2M	1670	1055	0.5369928	4	Singlet	Immune_cells
SAT_2M	1136	784	0.7901668	3	Singlet	FAPs
SAT_2M	2062	1247	2.4154589	1	Singlet	Immune_cells
SAT_2M	1009	620	0.4945598	8	Singlet	Mesothelial
SAT_2M	1208	755	0.0825083	4	Singlet	Immune_cells
SAT_2M	4302	2044	1.4143288	10	Singlet	Immune_cells

SAT_2M	1062	645	6.1567164	1	Singlet	Immune_cells
SAT_2M	1216	767	9.0612245	1	Singlet	Immune_cells
SAT_2M	2151	1213	4.0816327	1	Singlet	Immune_cells
SAT_2M	1220	709	14.367347	1	Singlet	Immune_cells
SAT_2M	1642	1039	1.9382193	1	Singlet	Immune_cells
SAT_2M	3281	1649	5.4506699	8	Singlet	Mesothelial
SAT_2M	2149	1272	3.6228518	4	Singlet	Immune_cells
SAT_2M	3118	1584	6.474359	1	Singlet	Immune_cells
SAT_2M	1176	743	2.2015241	8	Singlet	Mesothelial
SAT_2M	1988	1152	0.1002506	0	Singlet	Adipocyte
SAT_2M	1324	802	4.4360902	1	Singlet	Immune_cells
SAT_2M	4144	2137	9.2266924	4	Singlet	Immune_cells
SAT_2M	1391	887	0.6446991	0	Singlet	Adipocyte
SAT_2M	1540	958	0.9038089	1	Singlet	Immune_cells
SAT_2M	5559	2337	0.3403798	0	Singlet	Adipocyte
SAT_2M	1403	819	1.9914651	4	Singlet	Immune_cells
SAT_2M	1589	970	5.8197747	1	Singlet	Immune_cells
SAT_2M	1437	959	1.1126565	1	Singlet	Immune_cells
SAT_2M	2092	1114	4.148784	0	Singlet	Adipocyte
SAT_2M	1380	851	0.216763	5	Singlet	Immune_cells
SAT_2M	2102	1129	3.1783681	10	Singlet	Immune_cells
SAT_2M	1342	861	1.6332591	1	Singlet	Immune_cells
SAT_2M	2166	1224	0.6868132	0	Singlet	Adipocyte
SAT_2M	1101	723	0.8035714	4	Singlet	Immune_cells
SAT_2M	1348	790	5.1035503	1	Singlet	Immune_cells
SAT_2M	1207	838	0.410509	0	Singlet	Adipocyte
SAT_2M	3706	1781	0.1613337	0	Singlet	Adipocyte
SAT_2M	1028	664	1.9138756	5	Singlet	Immune_cells
SAT_2M	1044	661	0.7662835	1	Singlet	Immune_cells
SAT_2M	1423	927	0.3508772	0	Singlet	Adipocyte
SAT_2M	1304	866	0.0765697	5	Singlet	Immune_cells
SAT_2M	1023	580	6.4453125	5	Singlet	Immune_cells
SAT_2M	1179	740	0.7607777	4	Singlet	Immune_cells
SAT_2M	2324	1415	0.1710864	5	Singlet	Immune_cells
SAT_2M	1306	759	10.738766	1	Singlet	Immune_cells
SAT_2M	1369	868	7.8717201	1	Singlet	Immune_cells
SAT_2M	2153	1299	3.3890436	1	Singlet	Immune_cells
SAT_2M	1817	1197	0.1641138	4	Singlet	Immune_cells
SAT_2M	3382	1475	0.7674144	10	Singlet	Immune_cells
SAT_2M	2218	1263	0.6714414	0	Singlet	Adipocyte
SAT_2M	1263	793	2.4486572	1	Singlet	Immune_cells
SAT_2M	3787	1723	3.1603898	2	Singlet	FAPs
SAT_2M	1245	749	9.2295345	1	Singlet	Immune_cells
SAT_2M	1183	838	0.2529511	3	Singlet	FAPs
SAT_2M	1459	899	3.4836066	1	Singlet	Immune_cells
SAT_2M	1254	817	1.1867089	1	Singlet	Immune_cells
SAT_2M	1791	1125	0.7777778	0	Singlet	Adipocyte
SAT_2M	1252	811	0.1593626	0	Singlet	Adipocyte
SAT_2M	1672	976	3.6352801	1	Singlet	Immune_cells
SAT_2M	2038	1225	3.6728697	1	Singlet	Immune_cells
SAT_2M	1353	850	2.9325513	1	Singlet	Immune_cells

SAT_2M	1245	835	0.1598721	8	Singlet	Mesothelial
SAT_2M	1120	777	0.7104796	3	Singlet	FAPs
SAT_2M	1817	1053	3.4520548	1	Singlet	Immune_cells
SAT_2M	1966	1152	5.906108	4	Singlet	Immune_cells
SAT_2M	2050	1188	2.5304136	1	Singlet	Immune_cells
SAT_2M	1080	765	1.559633	1	Singlet	Immune_cells
SAT_2M	1727	1021	0.4039238	0	Singlet	Adipocyte
SAT_2M	1764	1085	1.8018018	4	Singlet	Immune_cells
SAT_2M	1759	1051	0.5665722	4	Singlet	Immune_cells
SAT_2M	2434	1345	0.7374027	0	Singlet	Adipocyte
SAT_2M	1555	933	1.5286624	0	Singlet	Adipocyte
SAT_2M	1062	760	0.1874414	5	Singlet	Immune_cells
SAT_2M	1416	937	0.4210526	5	Singlet	Immune_cells
SAT_2M	1308	854	3.4351145	4	Singlet	Immune_cells
SAT_2M	1269	772	5.0235479	1	Singlet	Immune_cells
SAT_2M	1100	740	0	1	Singlet	Immune_cells
SAT_2M	1079	690	1.0128913	4	Singlet	Immune_cells
SAT_2M	2327	1353	2.8718388	4	Singlet	Immune_cells
SAT_2M	2147	1161	3.5266821	10	Singlet	Immune_cells
SAT_2M	1417	872	2.5334272	4	Singlet	Immune_cells
SAT_2M	2753	1458	1.8076645	0	Singlet	Adipocyte
SAT_2M	1084	696	5.5860806	1	Singlet	Immune_cells
SAT_2M	1282	790	5.5598456	1	Singlet	Immune_cells
SAT_2M	2630	1428	12.168309	4	Singlet	Immune_cells
SAT_2M	1166	760	0.1712329	2	Singlet	FAPs
SAT_2M	1013	676	0.0986193	1	Singlet	Immune_cells
SAT_2M	2221	1296	2.1152115	4	Singlet	Immune_cells
SAT_2M	1334	870	1.2724551	1	Singlet	Immune_cells
SAT_2M	2350	1307	0.2538071	0	Singlet	Adipocyte
SAT_2M	2633	1479	5.026455	5	Singlet	Immune_cells
SAT_2M	1362	761	7.6302274	0	Singlet	Adipocyte
SAT_2M	2229	1321	0.2238138	8	Singlet	Mesothelial
SAT_2M	1099	723	4.0797824	4	Singlet	Immune_cells
SAT_2M	1039	664	0.2887392	0	Singlet	Adipocyte
SAT_2M	1520	1058	0.3913894	4	Singlet	Immune_cells
SAT_2M	1097	726	0.5449591	4	Singlet	Immune_cells
SAT_2M	1405	898	1.910828	3	Singlet	FAPs
SAT_2M	2890	1530	8.1034483	1	Singlet	Immune_cells
SAT_2M	1454	860	5.4869685	1	Singlet	Immune_cells
SAT_2M	1317	837	0.1515152	1	Singlet	Immune_cells
SAT_2M	1004	711	0.8823529	6	Singlet	Neurons
SAT_2M	1092	711	7.4954296	5	Singlet	Immune_cells
SAT_2M	1047	692	1.3346044	4	Singlet	Immune_cells
SAT_2M	1860	1106	0.1607717	0	Singlet	Adipocyte
SAT_2M	1631	1032	5.0214329	1	Singlet	Immune_cells
SAT_2M	1486	912	0.5354752	0	Singlet	Adipocyte
SAT_2M	1315	827	6.3732929	1	Singlet	Immune_cells
SAT_2M	1439	946	0.2770083	4	Singlet	Immune_cells
SAT_2M	1100	756	1.4492754	1	Singlet	Immune_cells
SAT_2M	1530	916	5.8708415	3	Singlet	FAPs
SAT_2M	6230	2501	1.2810248	10	Singlet	Immune_cells

SAT_2M	3574	1915	0.1111729	4	Singlet	Immune_cells
SAT_2M	2359	1225	1.1849344	0	Singlet	Adipocyte
SAT_2M	2502	1405	0.3577107	0	Singlet	Adipocyte
SAT_2M	1458	946	4.2436687	7	Singlet	Endothelial_cells
SAT_2M	1608	1014	0.3095975	0	Singlet	Adipocyte
SAT_2M	1029	696	2.7079304	1	Singlet	Immune_cells
SAT_2M	1103	755	0.2680965	5	Singlet	Immune_cells
SAT_2M	1098	781	0.5449591	5	Singlet	Immune_cells
SAT_2M	1498	914	0.7338225	0	Singlet	Adipocyte
SAT_2M	2750	1449	2.2133527	10	Singlet	Immune_cells
SAT_2M	1549	987	2.7634961	1	Singlet	Immune_cells
SAT_2M	1125	771	2.1276596	9	Singlet	Mesothelial
SAT_2M	1321	811	3.1626506	4	Singlet	Immune_cells
SAT_2M	2319	1290	0.7311828	0	Singlet	Adipocyte
SAT_2M	1132	742	0.6167401	4	Singlet	Immune_cells
SAT_2M	2170	1261	0.3205128	0	Singlet	Adipocyte
SAT_2M	2279	1150	3.6355672	5	Singlet	Immune_cells
SAT_2M	1210	815	1.8976898	11	Singlet	Smooth_muscle_cells
SAT_2M	2847	1442	1.997897	10	Singlet	Immune_cells
SAT_2M	1402	841	0.212917	1	Singlet	Immune_cells
SAT_2M	1499	896	4.5816733	0	Singlet	Adipocyte
SAT_2M	1512	947	1.7786561	0	Singlet	Adipocyte
SAT_2M	2097	1193	0.2376426	4	Singlet	Immune_cells
SAT_2M	1946	1215	0.6646217	4	Singlet	Immune_cells
SAT_2M	1485	924	12.358804	5	Singlet	Immune_cells
SAT_2M	1486	894	7.3627845	1	Singlet	Immune_cells
SAT_2M	2829	1477	0.7054674	3	Singlet	FAPs
SAT_2M	2080	1192	0.2876318	0	Singlet	Adipocyte
SAT_2M	1774	1071	2.2446689	0	Singlet	Adipocyte
SAT_2M	1532	979	2.0182292	1	Singlet	Immune_cells
SAT_2M	1462	960	0.3376097	4	Singlet	Immune_cells
SAT_2M	1100	721	3.3273381	3	Singlet	FAPs
SAT_2M	2471	1358	6.0532688	4	Singlet	Immune_cells
SAT_2M	2089	1213	1.1927481	0	Singlet	Adipocyte
SAT_2M	1406	874	1.9787986	4	Singlet	Immune_cells
SAT_2M	1419	871	6.7558058	1	Singlet	Immune_cells
SAT_2M	1313	849	0.6079027	1	Singlet	Immune_cells
SAT_2M	1452	915	0.6177076	1	Singlet	Immune_cells
SAT_2M	1497	1016	0.464499	4	Singlet	Immune_cells
SAT_2M	1467	879	3.8565629	4	Singlet	Immune_cells
SAT_2M	1184	776	2.2689076	1	Singlet	Immune_cells
SAT_2M	1271	851	0.862069	2	Singlet	FAPs
SAT_2M	1422	919	5.087108	1	Singlet	Immune_cells
SAT_2M	1112	718	0.4476276	4	Singlet	Immune_cells
SAT_2M	1515	933	4.2077581	5	Singlet	Immune_cells
SAT_2M	1333	938	1.1869436	5	Singlet	Immune_cells
SAT_2M	1730	1044	1.3848817	3	Singlet	FAPs
SAT_2M	1865	1124	2.5572722	4	Singlet	Immune_cells
SAT_2M	1144	698	5.9947871	0	Singlet	Adipocyte
SAT_2M	2716	1349	4.8144065	10	Singlet	Immune_cells
SAT_2M	1979	1108	5.0728277	4	Singlet	Immune_cells

SAT_2M	1267	806	2.679275	4	Singlet	Immune_cells
SAT_2M	1512	940	3.2343234	1	Singlet	Immune_cells
SAT_2M	1245	765	6.4748201	1	Singlet	Immune_cells
SAT_2M	1239	704	13.043478	1	Singlet	Immune_cells
SAT_2M	1666	1039	1.5550239	4	Singlet	Immune_cells
SAT_2M	1805	1000	3.0973451	3	Singlet	FAPs
SAT_2M	1382	881	1.7844397	4	Singlet	Immune_cells
SAT_2M	3265	1642	0.2134146	0	Singlet	Adipocyte
SAT_2M	1353	828	3.7555228	4	Singlet	Immune_cells
SAT_2M	1454	925	0.1375516	4	Singlet	Immune_cells
SAT_2M	1157	736	4.2241379	2	Singlet	FAPs
SAT_2M	1611	931	4.8387097	1	Singlet	Immune_cells
SAT_2M	1633	954	9.4454601	1	Singlet	Immune_cells
SAT_2M	1089	717	0.5459509	1	Singlet	Immune_cells
SAT_2M	1751	1065	1.5384615	1	Singlet	Immune_cells
SAT_2M	1534	964	5.0847458	1	Singlet	Immune_cells
SAT_2M	1282	837	3.6604361	4	Singlet	Immune_cells
SAT_2M	1445	885	0.6194081	0	Singlet	Adipocyte
SAT_2M	2016	1192	3.5061728	4	Singlet	Immune_cells
SAT_2M	1626	963	0.7334963	0	Singlet	Adipocyte
SAT_2M	1008	665	0	1	Singlet	Immune_cells
SAT_2M	3699	1899	0.7808293	2	Singlet	FAPs
SAT_2M	1228	800	4.291498	1	Singlet	Immune_cells
SAT_2M	1307	795	2.9029794	0	Singlet	Adipocyte
SAT_2M	1098	778	0.4528986	7	Singlet	Endothelial_cells
SAT_2M	1361	868	1.3899049	1	Singlet	Immune_cells
SAT_2M	2233	1374	5.57041	8	Singlet	Mesothelial
SAT_2M	3157	1621	1.290932	0	Singlet	Adipocyte
SAT_2M	1269	789	1.3333333	10	Singlet	Immune_cells
SAT_2M	1865	1073	1.1235955	4	Singlet	Immune_cells
SAT_2M	1286	778	0.620155	4	Singlet	Immune_cells
SAT_2M	1791	1119	3.6707453	1	Singlet	Immune_cells
SAT_2M	1383	868	3.0042918	4	Singlet	Immune_cells
SAT_2M	1263	808	4.3341214	4	Singlet	Immune_cells
SAT_2M	2337	1340	0.5106383	0	Singlet	Adipocyte
SAT_2M	1032	678	0	1	Singlet	Immune_cells
SAT_2M	1409	953	0.210084	8	Singlet	Mesothelial
SAT_2M	1581	993	0.9410289	0	Singlet	Adipocyte
SAT_2M	1667	1083	0.7789095	5	Singlet	Immune_cells
SAT_2M	1324	811	10.248681	4	Singlet	Immune_cells
SAT_2M	1961	1132	4.7352342	1	Singlet	Immune_cells
SAT_2M	2208	1378	0.3155996	8	Singlet	Mesothelial
SAT_2M	1084	695	0.1833181	0	Singlet	Adipocyte
SAT_2M	1952	1082	0.9615385	9	Singlet	Mesothelial
SAT_2M	2016	1148	1.9782394	7	Singlet	Endothelial_cells
SAT_2M	2535	1450	0.2749411	0	Singlet	Adipocyte
SAT_2M	2182	1241	1.9186843	4	Singlet	Immune_cells
SAT_2M	1318	845	3.1698113	1	Singlet	Immune_cells
SAT_2M	2692	1499	0.9214891	0	Singlet	Adipocyte
SAT_2M	1413	968	0.2111189	5	Singlet	Immune_cells
SAT_2M	2386	1275	10.208333	5	Singlet	Immune_cells

SAT_2M	1511	927	4.743083	1	Singlet	Immune_cells
SAT_2M	1525	941	0.3267974	4	Singlet	Immune_cells
SAT_2M	2196	1232	0.7262823	6	Singlet	Neurons
SAT_2M	2292	1321	0.6533101	10	Singlet	Immune_cells
SAT_2M	1464	954	1.2837838	1	Singlet	Immune_cells
SAT_2M	1772	1099	3.1584884	1	Singlet	Immune_cells
SAT_2M	2831	1714	0.2103049	4	Singlet	Immune_cells
SAT_2M	3993	1841	1.4246438	5	Singlet	Immune_cells
SAT_2M	1398	861	0.3558719	1	Singlet	Immune_cells
SAT_2M	2111	1295	0.8486563	5	Singlet	Immune_cells
SAT_2M	3449	1713	0.3468208	0	Singlet	Adipocyte
SAT_2M	1187	805	0.4194631	11	Singlet	Smooth_muscle_cells
SAT_2M	1925	1109	6.5939772	1	Singlet	Immune_cells
SAT_2M	2545	1469	2.0272904	5	Singlet	Immune_cells
SAT_2M	1012	658	0.1966568	4	Singlet	Immune_cells
SAT_2M	2108	1127	5.6105611	9	Singlet	Mesothelial
SAT_2M	1315	790	2.193646	1	Singlet	Immune_cells
SAT_2M	5070	2091	1.0234206	10	Singlet	Immune_cells
SAT_2M	1181	810	0.5076142	4	Singlet	Immune_cells
SAT_2M	5103	2305	10.64371	9	Singlet	Mesothelial
SAT_2M	4946	1859	1.9785988	10	Singlet	Immune_cells
SAT_2M	2169	1224	0.0915751	0	Singlet	Adipocyte
SAT_2M	1163	769	0.0859107	0	Singlet	Adipocyte
SAT_2M	1250	773	0.3968254	0	Singlet	Adipocyte
SAT_2M	1717	1015	3.7101449	1	Singlet	Immune_cells
SAT_2M	1855	1129	7.0475174	1	Singlet	Immune_cells
SAT_2M	2181	1185	3.9215686	3	Singlet	FAPs
SAT_2M	1555	892	9.8392283	1	Singlet	Immune_cells
SAT_2M	1165	742	1.4542344	1	Singlet	Immune_cells
SAT_2M	1498	883	6.5649867	1	Singlet	Immune_cells
SAT_2M	1808	1081	6.0154525	1	Singlet	Immune_cells
SAT_2M	2130	1257	2.9990628	8	Singlet	Mesothelial
SAT_2M	1816	1135	2.247807	4	Singlet	Immune_cells
SAT_2M	1834	1020	0.595883	0	Singlet	Adipocyte
SAT_2M	1219	804	1.3854931	4	Singlet	Immune_cells
SAT_2M	1046	696	0.8538899	3	Singlet	FAPs
SAT_2M	3137	1685	0.6977482	0	Singlet	Adipocyte
SAT_2M	2058	1194	1.3982642	0	Singlet	Adipocyte
SAT_2M	2790	1475	0.7142857	2	Singlet	FAPs
SAT_2M	1708	970	9.2657343	1	Singlet	Immune_cells
SAT_2M	1632	973	0.1219512	4	Singlet	Immune_cells
SAT_2M	1272	791	9.0766823	1	Singlet	Immune_cells
SAT_2M	1538	1001	0.6443299	8	Singlet	Mesothelial
SAT_2M	2480	1373	14.903846	5	Singlet	Immune_cells
SAT_2M	2781	1447	0.7899461	0	Singlet	Adipocyte
SAT_2M	1559	941	0.7042254	4	Singlet	Immune_cells
SAT_2M	1605	973	4.4776119	1	Singlet	Immune_cells
SAT_2M	1087	688	0.091659	4	Singlet	Immune_cells
SAT_2M	1635	963	2.3114355	4	Singlet	Immune_cells
SAT_2M	2471	1335	3.1880549	0	Singlet	Adipocyte
SAT_2M	2758	1464	0.1082251	0	Singlet	Adipocyte

SAT_2M	2650	1402	0.6012777	0	Singlet	Adipocyte
SAT_2M	9785	3699	8.1097623	9	Singlet	Mesothelial
SAT_2M	1314	803	2.4297646	4	Singlet	Immune_cells
SAT_2M	1490	954	3.3400134	5	Singlet	Immune_cells
SAT_2M	1360	889	1.9061584	1	Singlet	Immune_cells
SAT_2M	1543	980	1.1605416	3	Singlet	FAPs
SAT_2M	1865	1147	1.5491453	4	Singlet	Immune_cells
SAT_2M	1827	1070	5.0328228	1	Singlet	Immune_cells
SAT_2M	1362	840	8.5043988	1	Singlet	Immune_cells
SAT_2M	1802	1145	6.0454798	1	Singlet	Immune_cells
SAT_2M	1120	743	0.530504	0	Singlet	Adipocyte
SAT_2M	1307	820	2.972561	1	Singlet	Immune_cells
SAT_2M	5055	2037	1.0073079	2	Singlet	FAPs
SAT_2M	3229	1491	1.7906761	10	Singlet	Immune_cells
SAT_2M	1438	998	0.5536332	4	Singlet	Immune_cells
SAT_2M	1910	1054	5.4364872	1	Singlet	Immune_cells
SAT_2M	4366	1933	2.0576132	5	Singlet	Immune_cells
SAT_2M	1038	690	2.2051774	4	Singlet	Immune_cells
SAT_2M	2287	1377	0.8278867	3	Singlet	FAPs
SAT_2M	1574	986	2.528445	1	Singlet	Immune_cells
SAT_2M	2184	1287	0	8	Singlet	Mesothelial
SAT_2M	7010	3050	11.065224	4	Singlet	Immune_cells
SAT_2M	2506	1432	7.0461783	9	Singlet	Mesothelial
SAT_2M	2105	1103	14.042553	1	Singlet	Immune_cells
SAT_2M	3615	1779	0.8008837	11	Singlet	Smooth_muscle_cells
SAT_2M	1232	872	0.798722	0	Singlet	Adipocyte
SAT_2M	1570	1007	0.0632511	2	Singlet	FAPs
SAT_2M	1714	1115	1.5125073	1	Singlet	Immune_cells
SAT_2M	1181	730	0.3358522	1	Singlet	Immune_cells
SAT_2M	1056	692	2.1698113	1	Singlet	Immune_cells
SAT_2M	1739	1024	9.1065292	1	Singlet	Immune_cells
SAT_2M	2320	1257	1.1177988	0	Singlet	Adipocyte
SAT_2M	1606	1017	3.9130435	1	Singlet	Immune_cells
SAT_2M	1517	952	0.7905138	0	Singlet	Adipocyte
SAT_2M	1680	1069	4.682869	1	Singlet	Immune_cells
SAT_2M	1404	894	1.5602837	4	Singlet	Immune_cells
SAT_2M	1355	785	0.6617647	4	Singlet	Immune_cells
SAT_2M	1043	694	0.0956023	1	Singlet	Immune_cells
SAT_2M	1478	930	1.4874915	4	Singlet	Immune_cells
SAT_2M	1212	854	1.8837019	1	Singlet	Immune_cells
SAT_2M	1537	974	4.6722907	1	Singlet	Immune_cells
SAT_2M	1108	765	6.7736185	6	Singlet	Neurons
SAT_2M	1664	972	0.059988	1	Singlet	Immune_cells
SAT_2M	1481	950	5.0301811	1	Singlet	Immune_cells
SAT_2M	3112	1588	0.3526771	0	Singlet	Adipocyte
SAT_2M	1341	843	4.6130952	4	Singlet	Immune_cells
SAT_2M	4178	1993	9.436216	5	Singlet	Immune_cells
SAT_2M	1491	900	1.6722408	4	Singlet	Immune_cells
SAT_2M	1533	861	9.7529259	1	Singlet	Immune_cells
SAT_2M	1029	654	1.2536162	0	Singlet	Adipocyte
SAT_2M	1766	1051	1.4140271	0	Singlet	Adipocyte

SAT_2M	1126	766	1.6769638	1	Singlet	Immune_cells
SAT_2M	1420	919	0.3479471	4	Singlet	Immune_cells
SAT_2M	1148	705	0.5172414	1	Singlet	Immune_cells
SAT_2M	1106	751	0.0902527	1	Singlet	Immune_cells
SAT_2M	1441	879	8.005521	4	Singlet	Immune_cells
SAT_2M	2963	1582	1.4765101	0	Singlet	Adipocyte
SAT_2M	3393	1870	1.3811343	9	Singlet	Mesothelial
SAT_2M	2561	1455	0.2329193	0	Singlet	Adipocyte
SAT_2M	1170	737	3.1489362	3	Singlet	FAPs
SAT_2M	1228	796	0.973236	1	Singlet	Immune_cells
SAT_2M	4313	1971	1.8514233	2	Singlet	FAPs
SAT_2M	1212	739	4.3513957	0	Singlet	Adipocyte
SAT_2M	1304	857	0.304414	1	Singlet	Immune_cells
SAT_2M	1645	1073	0.5373134	4	Singlet	Immune_cells
SAT_2M	7670	3183	3.6704412	9	Singlet	Mesothelial
SAT_2M	1530	977	0.5208333	4	Singlet	Immune_cells
SAT_2M	1287	767	1.7857143	0	Singlet	Adipocyte
SAT_2M	1226	775	0.4068348	4	Singlet	Immune_cells
SAT_2M	1232	805	6.5164924	4	Singlet	Immune_cells
SAT_2M	2067	1196	8.011583	1	Singlet	Immune_cells
SAT_2M	1091	726	0.0909091	3	Singlet	FAPs
SAT_2M	1371	881	2.7636364	1	Singlet	Immune_cells
SAT_2M	1390	764	6.09319	9	Singlet	Mesothelial
SAT_2M	1774	1033	0.9009009	0	Singlet	Adipocyte
SAT_2M	1038	700	3.4058657	4	Singlet	Immune_cells
SAT_2M	1241	800	5.6134723	1	Singlet	Immune_cells
SAT_2M	1022	688	0.2929688	4	Singlet	Immune_cells
SAT_2M	1199	770	4.9084859	1	Singlet	Immune_cells
SAT_2M	1800	1075	1.1086475	0	Singlet	Adipocyte
SAT_2M	1381	904	0.3610108	4	Singlet	Immune_cells
SAT_2M	1846	1067	4.6511628	1	Singlet	Immune_cells
SAT_2M	1876	1095	0.4257584	4	Singlet	Immune_cells
SAT_2M	5444	2110	2.0913594	10	Singlet	Immune_cells
SAT_2M	1332	875	2.827381	1	Singlet	Immune_cells
SAT_2M	1088	750	1.2658228	4	Singlet	Immune_cells
SAT_2M	7083	2488	2.6104134	10	Singlet	Immune_cells
SAT_2M	1554	949	1.5296367	0	Singlet	Adipocyte
SAT_2M	1431	952	2.1557719	1	Singlet	Immune_cells
SAT_2M	4161	1989	0.1197318	0	Singlet	Adipocyte
SAT_2M	1229	844	0.5681818	4	Singlet	Immune_cells
SAT_2M	3656	1539	1.7472017	10	Singlet	Immune_cells
SAT_2M	1076	718	3.6044362	4	Singlet	Immune_cells
SAT_2M	1750	1053	3.8176638	1	Singlet	Immune_cells
SAT_2M	1715	981	12.398137	1	Singlet	Immune_cells
SAT_2M	3399	1716	2.170088	0	Singlet	Adipocyte
SAT_2M	1579	1032	0.1893939	5	Singlet	Immune_cells
SAT_2M	1688	1012	2.6035503	1	Singlet	Immune_cells
SAT_2M	1594	972	3.1269543	4	Singlet	Immune_cells
SAT_2M	1254	895	0.9531374	8	Singlet	Mesothelial
SAT_2M	1285	817	0.7763975	1	Singlet	Immune_cells
SAT_2M	1713	1106	0.6376812	1	Singlet	Immune_cells

SAT_2M	1834	1191	0.1085776	4	Singlet	Immune_cells
SAT_2M	1292	780	7.9414032	1	Singlet	Immune_cells
SAT_2M	1601	1057	2.4968789	1	Singlet	Immune_cells
SAT_2M	1198	763	0.5823627	0	Singlet	Adipocyte
SAT_2M	5947	2687	7.5478028	9	Singlet	Mesothelial
SAT_2M	1117	792	0.9743136	4	Singlet	Immune_cells
SAT_2M	1898	1147	2.0472441	10	Singlet	Immune_cells
SAT_2M	4717	2030	1.3744978	10	Singlet	Immune_cells
SAT_2M	2726	1408	0.5486467	0	Singlet	Adipocyte
SAT_2M	1267	839	0.5520505	4	Singlet	Immune_cells
SAT_2M	1664	1018	0.059952	1	Singlet	Immune_cells
SAT_2M	1469	958	1.5582656	4	Singlet	Immune_cells
SAT_2M	1001	658	2.2862823	3	Singlet	FAPs
SAT_2M	3785	1880	0.2637131	2	Singlet	FAPs
SAT_2M	2267	1275	0.437254	0	Singlet	Adipocyte
SAT_2M	2623	1386	1.1039208	0	Singlet	Adipocyte
SAT_2M	1196	811	0.4177109	4	Singlet	Immune_cells
SAT_2M	1152	755	0.3460208	4	Singlet	Immune_cells
SAT_2M	1902	1189	4.1535226	4	Singlet	Immune_cells
SAT_2M	1746	1129	3.0821918	1	Singlet	Immune_cells
SAT_2M	1765	1029	7.6314302	0	Singlet	Adipocyte
SAT_2M	1253	861	0.3930818	4	Singlet	Immune_cells
SAT_2M	2497	1463	1.8769968	8	Singlet	Mesothelial
SAT_2M	1982	1172	0.1005025	0	Singlet	Adipocyte
SAT_2M	1823	1142	0.2189382	5	Singlet	Immune_cells
SAT_2M	1266	832	2.6025237	1	Singlet	Immune_cells
SAT_2M	1564	946	2.4234694	1	Singlet	Immune_cells
SAT_2M	1292	861	4.0123457	1	Singlet	Immune_cells
SAT_2M	2558	1424	0.1169591	0	Singlet	Adipocyte
SAT_2M	1368	917	0.2909091	1	Singlet	Immune_cells
SAT_2M	1522	917	9.102816	1	Singlet	Immune_cells
SAT_2M	1432	915	3.5986159	4	Singlet	Immune_cells
SAT_2M	1107	721	5.2867384	1	Singlet	Immune_cells
SAT_2M	1600	994	2.1091811	5	Singlet	Immune_cells
SAT_2M	1874	1118	5.9136921	1	Singlet	Immune_cells
SAT_2M	2130	1207	3.3566434	0	Singlet	Adipocyte
SAT_2M	3179	1656	2.256346	8	Singlet	Mesothelial
SAT_2M	1290	804	9.2449923	1	Singlet	Immune_cells
SAT_2M	1132	730	0.7943513	5	Singlet	Immune_cells
SAT_2M	3665	1795	0.7070982	0	Singlet	Adipocyte
SAT_2M	1034	676	8.2851638	1	Singlet	Immune_cells
SAT_2M	1006	678	0.3956479	4	Singlet	Immune_cells
SAT_2M	3141	1612	0.9847522	0	Singlet	Adipocyte
SAT_2M	1339	846	3.4149963	1	Singlet	Immune_cells
SAT_2M	2860	1463	9.9930119	1	Singlet	Immune_cells
SAT_2M	1627	988	3.4292713	1	Singlet	Immune_cells
SAT_2M	1322	883	1.7950636	1	Singlet	Immune_cells
SAT_2M	1521	937	1.4332248	6	Singlet	Neurons
SAT_2M	2102	1283	0.2843602	0	Singlet	Adipocyte
SAT_2M	1491	918	4.0053405	1	Singlet	Immune_cells
SAT_2M	1197	797	2.7477102	10	Singlet	Immune_cells

SAT_2M	1538	992	2.8976175	1	Singlet	Immune_cells
SAT_2M	2383	1389	0.8343763	8	Singlet	Mesothelial
SAT_2M	2320	1319	3.5698925	4	Singlet	Immune_cells
SAT_2M	1497	892	0.998004	1	Singlet	Immune_cells
SAT_2M	1089	728	0.2734731	4	Singlet	Immune_cells
SAT_2M	2150	1159	1.5806602	3	Singlet	FAPs
SAT_2M	1411	872	0.5657709	1	Singlet	Immune_cells
SAT_2M	1377	858	4.765343	1	Singlet	Immune_cells
SAT_2M	1644	957	2.4242424	5	Singlet	Immune_cells
SAT_2M	3200	1749	0.5902454	8	Singlet	Mesothelial
SAT_2M	2583	1344	0.8104979	3	Singlet	FAPs
SAT_2M	1328	853	2.5583145	1	Singlet	Immune_cells
SAT_2M	1573	1020	1.8975332	5	Singlet	Immune_cells
SAT_2M	1280	815	0.5460218	4	Singlet	Immune_cells
SAT_2M	1283	882	0.5417957	4	Singlet	Immune_cells
SAT_2M	1281	790	4.040404	1	Singlet	Immune_cells
SAT_2M	1766	1076	1.1267606	7	Singlet	Endothelial_cells
SAT_2M	1273	841	0.5451713	1	Singlet	Immune_cells
SAT_2M	7313	3012	3.1275608	9	Singlet	Mesothelial
SAT_2M	1264	772	13.779528	4	Singlet	Immune_cells
SAT_2M	1036	708	1.4354067	4	Singlet	Immune_cells
SAT_2M	1880	1098	0.2119767	1	Singlet	Immune_cells
SAT_2M	1015	629	0.3929273	1	Singlet	Immune_cells
SAT_2M	1413	923	1.8361582	0	Singlet	Adipocyte
SAT_2M	1318	766	9.1597275	1	Singlet	Immune_cells
SAT_2M	1216	799	1.5548282	4	Singlet	Immune_cells
SAT_2M	1043	668	0.4784689	1	Singlet	Immune_cells
SAT_2M	1702	1036	0.5284792	0	Singlet	Adipocyte
SAT_2M	1705	1043	0.2912056	0	Singlet	Adipocyte
SAT_2M	2785	1414	1.3953488	0	Singlet	Adipocyte
SAT_2M	1296	799	0.3849115	4	Singlet	Immune_cells
SAT_2M	1927	1129	0.9264025	0	Singlet	Adipocyte
SAT_2M	1346	779	9.5484826	0	Singlet	Adipocyte
SAT_2M	1127	747	0.3542958	1	Singlet	Immune_cells
SAT_2M	1310	823	0.9863429	1	Singlet	Immune_cells
SAT_2M	1161	816	1.2908778	5	Singlet	Immune_cells
SAT_2M	1265	815	1.336478	0	Singlet	Adipocyte
SAT_2M	2887	1556	2.4922118	9	Singlet	Mesothelial
SAT_2M	1301	783	8.9723926	1	Singlet	Immune_cells
SAT_2M	1819	1191	0.9304871	11	Singlet	Smooth_muscle_cells
SAT_2M	1336	843	2.2338049	0	Singlet	Adipocyte
SAT_2M	1353	871	2.7900147	1	Singlet	Immune_cells
SAT_2M	1362	864	1.2426901	1	Singlet	Immune_cells
SAT_2M	1981	1121	6.3819095	1	Singlet	Immune_cells
SAT_2M	1199	808	0.4163197	7	Singlet	Endothelial_cells
SAT_2M	1082	731	0.0922509	4	Singlet	Immune_cells
SAT_2M	2035	1180	3.1357178	1	Singlet	Immune_cells
SAT_2M	1175	787	1.1854361	1	Singlet	Immune_cells
SAT_2M	1244	840	0.3187251	3	Singlet	FAPs
SAT_2M	1198	788	0.4139073	0	Singlet	Adipocyte
SAT_2M	1154	788	2.7562446	1	Singlet	Immune_cells

SAT_2M	5307	2417	4.5300752	9	Singlet	Mesothelial
SAT_2M	1672	979	5.9311981	1	Singlet	Immune_cells
SAT_2M	2383	1326	0.3753128	0	Singlet	Adipocyte
SAT_2M	1681	1070	0.1184834	8	Singlet	Mesothelial
SAT_2M	2108	1257	5.0614948	1	Singlet	Immune_cells
SAT_2M	1509	1007	0.5948447	4	Singlet	Immune_cells
SAT_2M	3661	1832	0.0544366	0	Singlet	Adipocyte
SAT_2M	1021	658	1.5655577	0	Singlet	Adipocyte
SAT_2M	1979	1163	0.1006036	0	Singlet	Adipocyte
SAT_2M	1716	1033	0.1737116	0	Singlet	Adipocyte
SAT_2M	1246	772	3.2721468	1	Singlet	Immune_cells
SAT_2M	2236	1331	0.6690455	1	Singlet	Immune_cells
SAT_2M	1178	742	0.5071851	8	Singlet	Mesothelial
SAT_2M	1969	1075	1.0080645	1	Singlet	Immune_cells
SAT_2M	1721	1034	5.561993	1	Singlet	Immune_cells
SAT_2M	1423	935	1.054111	4	Singlet	Immune_cells
SAT_2M	1847	1040	9.3783494	1	Singlet	Immune_cells
SAT_2M	1360	833	4.4787078	1	Singlet	Immune_cells
SAT_2M	1538	984	0.3878474	1	Singlet	Immune_cells
SAT_2M	1765	1047	0.1128032	0	Singlet	Adipocyte
SAT_2M	2487	1425	0.3996803	6	Singlet	Neurons
SAT_2M	1280	893	1.7107309	4	Singlet	Immune_cells
SAT_2M	2240	1288	5.1601423	5	Singlet	Immune_cells
SAT_2M	1552	991	0.8365508	3	Singlet	FAPs
SAT_2M	2154	1300	0.1848429	4	Singlet	Immune_cells
SAT_2M	1166	737	1.283148	4	Singlet	Immune_cells
SAT_2M	1348	892	4.804139	4	Singlet	Immune_cells
SAT_2M	1449	811	12.57732	1	Singlet	Immune_cells
SAT_2M	1576	993	0.6979695	1	Singlet	Immune_cells
SAT_2M	3236	1725	1.1405672	10	Singlet	Immune_cells
SAT_2M	1397	903	0.3579098	4	Singlet	Immune_cells
SAT_2M	1686	1016	2.7202839	0	Singlet	Adipocyte
SAT_2M	1426	860	1.958042	5	Singlet	Immune_cells
SAT_2M	1307	809	5.1948052	1	Singlet	Immune_cells
SAT_2M	2102	1163	0.3796868	8	Singlet	Mesothelial
SAT_2M	1519	997	0.3931848	4	Singlet	Immune_cells
SAT_2M	3148	1619	0.6647673	0	Singlet	Adipocyte
SAT_2M	1092	723	0.8189263	1	Singlet	Immune_cells
SAT_2M	1830	1108	0.6528836	11	Singlet	Smooth_muscle_cells
SAT_2M	1383	814	1.373825	0	Singlet	Adipocyte
SAT_2M	2400	1465	0.1244297	4	Singlet	Immune_cells
SAT_2M	1016	686	0.5865103	3	Singlet	FAPs
SAT_2M	1221	726	2.5326797	0	Singlet	Adipocyte
SAT_2M	7676	3431	7.2644574	9	Singlet	Mesothelial
SAT_2M	2262	1315	2.1126761	0	Singlet	Adipocyte
SAT_2M	1837	1089	4.076087	4	Singlet	Immune_cells
SAT_2M	1227	845	0.242915	3	Singlet	FAPs
SAT_2M	2191	1266	4.5454545	9	Singlet	Mesothelial
SAT_2M	2363	1347	0.1688476	0	Singlet	Adipocyte
SAT_2M	3245	1719	0.122737	0	Singlet	Adipocyte
SAT_2M	10869	4334	7.0496324	9	Singlet	Mesothelial

SAT_2M	1008	715	3.0632411	11	Singlet	Smooth_muscle_cells
SAT_2M	1637	1068	1.8159806	5	Singlet	Immune_cells
SAT_2M	2061	1136	12.015504	9	Singlet	Mesothelial
SAT_2M	1527	949	1.0430248	1	Singlet	Immune_cells
SAT_2M	3439	1703	4.2608696	0	Singlet	Adipocyte
SAT_2M	1232	825	0.6472492	0	Singlet	Adipocyte
SAT_2M	3500	1839	11.126961	9	Singlet	Mesothelial
SAT_2M	2737	1581	0.5830904	1	Singlet	Immune_cells
SAT_2M	1950	1086	3.4693878	1	Singlet	Immune_cells
SAT_2M	2625	1497	6.6996574	1	Singlet	Immune_cells
SAT_2M	1311	851	0.3799392	0	Singlet	Adipocyte
SAT_2M	1620	1021	7.3230769	1	Singlet	Immune_cells
SAT_2M	1382	986	0.4307251	4	Singlet	Immune_cells
SAT_2M	1559	943	0.1274697	0	Singlet	Adipocyte
SAT_2M	1172	775	0.6779661	3	Singlet	FAPs
SAT_2M	1333	852	0.4484305	4	Singlet	Immune_cells
SAT_2M	3061	1684	0.4874878	0	Singlet	Adipocyte
SAT_2M	2302	1285	4.529767	0	Singlet	Adipocyte
SAT_2M	1641	987	2.1726011	1	Singlet	Immune_cells
SAT_2M	1300	831	0.6097561	0	Singlet	Adipocyte
SAT_2M	1377	929	2.0999276	1	Singlet	Immune_cells
SAT_2M	1399	900	6.2588905	1	Singlet	Immune_cells
SAT_2M	2795	1651	0.1428571	5	Singlet	Immune_cells
SAT_2M	1546	952	7.4550129	4	Singlet	Immune_cells
SAT_2M	1239	766	3.935743	1	Singlet	Immune_cells
SAT_2M	1245	840	2.1445592	4	Singlet	Immune_cells
SAT_2M	1844	1075	4.5479155	4	Singlet	Immune_cells
SAT_2M	1895	1087	7.0526316	9	Singlet	Mesothelial
SAT_2M	1223	727	1.8744906	1	Singlet	Immune_cells
SAT_2M	1428	900	0.6968641	0	Singlet	Adipocyte
SAT_2M	1746	1110	0.1712329	4	Singlet	Immune_cells
SAT_2M	1635	1033	2.1315469	4	Singlet	Immune_cells
SAT_2M	1682	1022	1.7730496	4	Singlet	Immune_cells
SAT_2M	1014	680	1.4778325	8	Singlet	Mesothelial
SAT_2M	1516	959	2.8928337	3	Singlet	FAPs
SAT_2M	1457	984	2.739726	1	Singlet	Immune_cells
SAT_2M	1290	832	3.2457496	1	Singlet	Immune_cells
SAT_2M	1568	971	1.7721519	1	Singlet	Immune_cells
SAT_2M	1911	1155	2.9796132	1	Singlet	Immune_cells
SAT_2M	1873	1155	0.532198	4	Singlet	Immune_cells
SAT_2M	1948	1098	5.0025523	1	Singlet	Immune_cells
SAT_2M	1158	757	0.2581756	11	Singlet	Smooth_muscle_cells
SAT_2M	1337	834	1.2639405	1	Singlet	Immune_cells
SAT_2M	2165	1223	5.649977	9	Singlet	Mesothelial
SAT_2M	2272	1331	2.7204914	9	Singlet	Mesothelial
SAT_2M	1811	1130	0.8820287	4	Singlet	Immune_cells
SAT_2M	1482	959	0.0668003	0	Singlet	Adipocyte
SAT_2M	1090	714	1.0938924	1	Singlet	Immune_cells
SAT_2M	1534	888	7.5471698	1	Singlet	Immune_cells
SAT_2M	2114	1255	3.5781544	2	Singlet	FAPs
SAT_2M	1965	1144	7.5339026	5	Singlet	Immune_cells

SAT_2M	1075	723	0.1853568	4	Singlet	Immune_cells
SAT_2M	1553	956	0.1284522	4	Singlet	Immune_cells
SAT_2M	1361	897	1.3225569	1	Singlet	Immune_cells
SAT_2M	1398	867	6.3662375	1	Singlet	Immune_cells
SAT_2M	1029	729	1.7424976	1	Singlet	Immune_cells
SAT_2M	1171	778	1.6183986	4	Singlet	Immune_cells
SAT_2M	1787	1067	2.5626741	1	Singlet	Immune_cells
SAT_2M	1190	833	1.1666667	8	Singlet	Mesothelial
SAT_2M	1516	939	4.91481	3	Singlet	FAPs
SAT_2M	1503	865	8.8430851	1	Singlet	Immune_cells
SAT_2M	1735	1072	3.2664756	0	Singlet	Adipocyte
SAT_2M	11669	4206	2.533379	2	Singlet	FAPs
SAT_2M	1261	731	0.8702532	3	Singlet	FAPs
SAT_2M	1318	803	2.1953066	0	Singlet	Adipocyte
SAT_2M	2888	1385	1.6218081	10	Singlet	Immune_cells
SAT_2M	1085	645	1.7431193	7	Singlet	Endothelial_cells
SAT_2M	1290	820	2.7006173	1	Singlet	Immune_cells
SAT_2M	1467	1012	1.5509103	4	Singlet	Immune_cells
SAT_2M	1364	857	0.5763689	0	Singlet	Adipocyte
SAT_2M	5268	2225	2.1722705	10	Singlet	Immune_cells
SAT_2M	7244	2860	6.717137	4	Singlet	Immune_cells
SAT_2M	1847	1048	1.5616586	5	Singlet	Immune_cells
SAT_2M	1438	934	1.8030513	4	Singlet	Immune_cells
SAT_2M	1484	916	0.6729475	1	Singlet	Immune_cells
SAT_2M	1223	860	1.5359741	1	Singlet	Immune_cells
SAT_2M	1080	693	2.2201665	1	Singlet	Immune_cells
SAT_2M	1477	941	0.1348618	8	Singlet	Mesothelial
SAT_2M	2469	1359	3.266129	1	Singlet	Immune_cells
SAT_2M	1634	994	4.0928528	1	Singlet	Immune_cells
SAT_2M	1029	735	0.4789272	1	Singlet	Immune_cells
SAT_2M	1273	816	0.3888025	0	Singlet	Adipocyte
SAT_2M	3785	2044	6.1084781	4	Singlet	Immune_cells
SAT_2M	1852	1080	10.580021	1	Singlet	Immune_cells
SAT_2M	1280	775	8.5271318	1	Singlet	Immune_cells
SAT_2M	1748	1083	0.6837607	2	Singlet	FAPs
SAT_2M	2647	1504	2.2820801	1	Singlet	Immune_cells
SAT_2M	1354	852	0.2941176	0	Singlet	Adipocyte
SAT_2M	1064	703	6.2383613	9	Singlet	Mesothelial
SAT_2M	1073	736	0.3693444	4	Singlet	Immune_cells
SAT_2M	1952	1189	1.9928462	4	Singlet	Immune_cells
SAT_2M	2550	1499	6.0179758	9	Singlet	Mesothelial
SAT_2M	1263	800	1.6600791	4	Singlet	Immune_cells
SAT_2M	1252	801	9.1917591	1	Singlet	Immune_cells
SAT_2M	1163	749	0.5136986	4	Singlet	Immune_cells
SAT_2M	1320	913	0.9803922	11	Singlet	Smooth_muscle_cells
SAT_2M	1389	838	4.0143369	1	Singlet	Immune_cells
SAT_2M	2668	1363	3.4444028	0	Singlet	Adipocyte
SAT_2M	1270	751	1.2588513	0	Singlet	Adipocyte
SAT_2M	1299	828	6.0582822	10	Singlet	Immune_cells
SAT_2M	1057	692	0.6578947	0	Singlet	Adipocyte
SAT_2M	1015	683	1.373896	4	Singlet	Immune_cells

SAT_2M	1052	674	0.4748338	0	Singlet	Adipocyte
SAT_2M	1614	1034	1.6019717	7	Singlet	Endothelial_cells
SAT_2M	2534	1365	0.1573564	0	Singlet	Adipocyte
SAT_2M	1159	760	2.4158758	1	Singlet	Immune_cells
SAT_2M	1443	829	0.3422313	4	Singlet	Immune_cells
SAT_2M	2882	1492	4.9792531	5	Singlet	Immune_cells
SAT_2M	1959	1126	0	0	Singlet	Adipocyte
SAT_2M	1231	783	1.0526316	1	Singlet	Immune_cells
SAT_2M	1259	775	0.1582278	1	Singlet	Immune_cells
SAT_2M	1551	913	0.5787781	2	Singlet	FAPs
SAT_2M	1136	789	0.2631579	4	Singlet	Immune_cells
SAT_2M	1998	990	1.2961117	10	Singlet	Immune_cells
SAT_2M	1255	846	4.2925278	4	Singlet	Immune_cells
SAT_2M	1230	796	0.4862237	0	Singlet	Adipocyte
SAT_2M	2033	1211	0.3929273	4	Singlet	Immune_cells
SAT_2M	1106	708	0.1798561	1	Singlet	Immune_cells
SAT_2M	2022	1230	5.1282051	1	Singlet	Immune_cells
SAT_2M	1377	934	2.8673835	11	Singlet	Smooth_muscle_cells
SAT_2M	2562	1325	2.8793774	10	Singlet	Immune_cells
SAT_2M	1426	843	11.629526	1	Singlet	Immune_cells
SAT_2M	2212	1294	1.8410418	1	Singlet	Immune_cells
SAT_2M	2038	1208	0.8308895	1	Singlet	Immune_cells
SAT_2M	1132	756	0.877193	7	Singlet	Endothelial_cells
SAT_2M	2779	1521	8.1003584	1	Singlet	Immune_cells
SAT_2M	1343	873	0.1480385	0	Singlet	Adipocyte
SAT_2M	2158	1224	3.8781163	0	Singlet	Adipocyte
SAT_2M	1910	1267	0.2075765	4	Singlet	Immune_cells
SAT_2M	1514	912	3.0880421	1	Singlet	Immune_cells
SAT_2M	1204	771	1.3234078	1	Singlet	Immune_cells
SAT_2M	1476	913	6.2795409	1	Singlet	Immune_cells
SAT_2M	2200	1302	3.851382	1	Singlet	Immune_cells
SAT_2M	2302	1300	1.7764298	0	Singlet	Adipocyte
SAT_2M	1168	728	2.5553663	3	Singlet	FAPs
SAT_2M	1107	714	1.440144	5	Singlet	Immune_cells
SAT_2M	1247	816	0.4803843	1	Singlet	Immune_cells
SAT_2M	3203	1680	3.1434796	4	Singlet	Immune_cells
SAT_2M	1298	820	7.296467	4	Singlet	Immune_cells
SAT_2M	1744	1103	5.2451539	8	Singlet	Mesothelial
SAT_2M	1359	895	3.4331629	1	Singlet	Immune_cells
SAT_2M	2392	1316	4.704413	1	Singlet	Immune_cells
SAT_2M	1677	1047	0.9501188	3	Singlet	FAPs
SAT_2M	1252	778	8.3794466	4	Singlet	Immune_cells
SAT_2M	3845	1855	0.5450298	0	Singlet	Adipocyte
SAT_2M	1434	929	2.0876827	11	Singlet	Smooth_muscle_cells
SAT_2M	1606	972	5.4692356	1	Singlet	Immune_cells
SAT_2M	1483	875	1.138647	0	Singlet	Adipocyte
SAT_2M	2820	1531	1.6572638	0	Singlet	Adipocyte
SAT_2M	1607	1028	5.0216987	4	Singlet	Immune_cells
SAT_2M	3361	1816	0.2368967	5	Singlet	Immune_cells
SAT_2M	1115	793	0.969163	4	Singlet	Immune_cells
SAT_2M	5810	2326	1.647503	0	Singlet	Adipocyte

SAT_2M	1251	782	4.2264753	4	Singlet	Immune_cells
SAT_2M	1508	884	4.7051027	4	Singlet	Immune_cells
SAT_2M	3451	1715	1.2124711	0	Singlet	Adipocyte
SAT_2M	3181	1650	3.9510818	1	Singlet	Immune_cells
SAT_2M	1523	963	2.3963731	6	Singlet	Neurons
SAT_2M	2407	1263	6.8680182	5	Singlet	Immune_cells
SAT_2M	1037	675	3.3589251	11	Singlet	Smooth_muscle_cells
SAT_2M	1540	982	0.9721322	0	Singlet	Adipocyte
SAT_2M	2010	1173	2.8741328	5	Singlet	Immune_cells
SAT_2M	1058	686	5.7601511	5	Singlet	Immune_cells
SAT_2M	4024	1967	2.5793651	10	Singlet	Immune_cells
SAT_2M	1444	884	0.137836	1	Singlet	Immune_cells
SAT_2M	1998	1094	7.089366	1	Singlet	Immune_cells
SAT_2M	1365	835	0.8035062	0	Singlet	Adipocyte
SAT_2M	1409	903	1.5602837	8	Singlet	Mesothelial
SAT_2M	1227	781	4.3938161	2	Singlet	FAPs
SAT_2M	5883	2530	0.1697793	0	Singlet	Adipocyte
SAT_2M	1540	951	9.9354839	4	Singlet	Immune_cells
SAT_2M	1764	1041	0.3393665	0	Singlet	Adipocyte
SAT_2M	1079	698	3.9704524	1	Singlet	Immune_cells
SAT_2M	1097	715	0.7226739	4	Singlet	Immune_cells
SAT_2M	1202	835	1.0726073	6	Singlet	Neurons
SAT_2M	1942	1102	3.3898305	0	Singlet	Adipocyte
SAT_2M	1261	828	0.390625	4	Singlet	Immune_cells
SAT_2M	1590	993	5.0062578	1	Singlet	Immune_cells
SAT_2M	2141	1110	3.6864209	0	Singlet	Adipocyte
SAT_2M	1243	719	0.6415397	3	Singlet	FAPs
SAT_2M	1229	734	8.2792208	1	Singlet	Immune_cells
SAT_2M	1361	865	0.5102041	4	Singlet	Immune_cells
SAT_2M	1353	911	1.5418502	4	Singlet	Immune_cells
SAT_2M	2261	1225	0.5298013	10	Singlet	Immune_cells
SAT_2M	2363	1374	0.0842815	5	Singlet	Immune_cells
SAT_2M	1122	682	7.2888889	5	Singlet	Immune_cells
SAT_2M	1302	838	0.1531394	8	Singlet	Mesothelial
SAT_2M	1287	814	1.3157895	3	Singlet	FAPs
SAT_2M	1334	814	0.2240478	1	Singlet	Immune_cells
SAT_2M	1075	712	1.843318	4	Singlet	Immune_cells
SAT_2M	1374	904	0.1440922	4	Singlet	Immune_cells
SAT_2M	1325	825	2.2607385	0	Singlet	Adipocyte
SAT_2M	3455	1736	0.1445087	3	Singlet	FAPs
SAT_2M	1691	1056	2.3557126	4	Singlet	Immune_cells
SAT_2M	1071	778	2.0072993	8	Singlet	Mesothelial
SAT_2M	1113	749	0.2688172	1	Singlet	Immune_cells
SAT_2M	2888	1571	1.6179002	5	Singlet	Immune_cells
SAT_2M	2935	1483	4.047619	10	Singlet	Immune_cells
SAT_2M	1716	1067	0.6956522	8	Singlet	Mesothelial
SAT_2M	1405	830	4.0311174	1	Singlet	Immune_cells
SAT_2M	2511	1486	0.5564388	3	Singlet	FAPs
SAT_2M	4433	2215	7.2698627	4	Singlet	Immune_cells
SAT_2M	1940	1175	2.4640657	8	Singlet	Mesothelial
SAT_2M	3144	1261	0.6660324	10	Singlet	Immune_cells

SAT_2M	1525	1001	0.1306336	0	Singlet	Adipocyte
SAT_2M	5125	2087	2.0463847	10	Singlet	Immune_cells
SAT_2M	1491	1006	0.8660893	0	Singlet	Adipocyte
SAT_2M	1669	929	0.8328376	0	Singlet	Adipocyte
SAT_2M	1767	1058	1.2935883	1	Singlet	Immune_cells
SAT_2M	3180	1698	1.4433637	1	Singlet	Immune_cells
SAT_2M	1268	749	4.015748	0	Singlet	Adipocyte
SAT_2M	1274	819	0.6987578	1	Singlet	Immune_cells
SAT_2M	1012	693	0.4906771	1	Singlet	Immune_cells
SAT_2M	1485	977	2.4617432	1	Singlet	Immune_cells
SAT_2M	1854	1071	0.915455	0	Singlet	Adipocyte
SAT_2M	2687	1456	1.038961	3	Singlet	FAPs
SAT_2M	1391	835	5.0251256	1	Singlet	Immune_cells
SAT_2M	3190	1856	3.319762	9	Singlet	Mesothelial
SAT_2M	1123	764	0.3552398	1	Singlet	Immune_cells
SAT_2M	1531	812	2.2193211	10	Singlet	Immune_cells
SAT_2M	1311	884	0.6828528	4	Singlet	Immune_cells
SAT_2M	5028	2028	0.6558029	10	Singlet	Immune_cells
SAT_2M	1265	806	0.5494505	1	Singlet	Immune_cells
SAT_2M	1376	881	0.7919366	0	Singlet	Adipocyte
SAT_2M	1303	791	0.6891271	1	Singlet	Immune_cells
SAT_2M	1368	828	8.490566	1	Singlet	Immune_cells
SAT_2M	1024	684	0.6789525	1	Singlet	Immune_cells
SAT_2M	1003	637	0.0995025	1	Singlet	Immune_cells
SAT_2M	1796	1033	1.9422863	7	Singlet	Endothelial_cells
SAT_2M	1273	809	0.1566171	1	Singlet	Immune_cells
SAT_2M	1835	1199	0.2164502	4	Singlet	Immune_cells
SAT_2M	1255	801	3.0134814	1	Singlet	Immune_cells
SAT_2M	1267	759	0.9419152	2	Singlet	FAPs
SAT_2M	1012	640	0.7889546	0	Singlet	Adipocyte
SAT_2M	1747	1067	2.1664766	1	Singlet	Immune_cells
SAT_2M	2479	1470	0.7237636	8	Singlet	Mesothelial
SAT_2M	4801	2070	7.722649	0	Singlet	Adipocyte
SAT_2M	1738	1014	4.5819015	4	Singlet	Immune_cells
SAT_2M	1705	1067	5.5911474	7	Singlet	Endothelial_cells
SAT_2M	1613	987	3.2756489	1	Singlet	Immune_cells
SAT_2M	2159	1239	10.119595	5	Singlet	Immune_cells
SAT_2M	2347	1245	7.1701315	1	Singlet	Immune_cells
SAT_2M	1020	688	0.195122	4	Singlet	Immune_cells
SAT_2M	2110	1254	4.6588235	1	Singlet	Immune_cells
SAT_2M	1153	772	0.6039689	1	Singlet	Immune_cells
SAT_2M	2288	1338	0.04363	0	Singlet	Adipocyte
SAT_2M	1483	881	8.7306917	1	Singlet	Immune_cells
SAT_2M	1929	1140	2.1705426	1	Singlet	Immune_cells
SAT_2M	1446	883	1.2413793	1	Singlet	Immune_cells
SAT_2M	3229	1623	3.7916153	0	Singlet	Adipocyte
SAT_2M	1221	826	0.2452984	4	Singlet	Immune_cells
SAT_2M	1537	891	4.150454	1	Singlet	Immune_cells
SAT_2M	1513	976	5.0524934	1	Singlet	Immune_cells
SAT_2M	1026	742	0.7714561	7	Singlet	Endothelial_cells
SAT_2M	7129	3111	8.4441955	4	Singlet	Immune_cells

SAT_2M	1365	800	10.407569	1	Singlet	Immune_cells
SAT_2M	4837	2369	5.6553148	9	Singlet	Mesothelial
SAT_2M	1031	709	0.192123	4	Singlet	Immune_cells
SAT_2M	1923	1015	3.1104199	0	Singlet	Adipocyte
SAT_2M	1645	991	0.9691096	1	Singlet	Immune_cells
SAT_2M	3172	1711	0.0313578	11	Singlet	Smooth_muscle_cells
SAT_2M	1340	829	0.4454343	4	Singlet	Immune_cells
SAT_2M	2161	1131	1.8441678	0	Singlet	Adipocyte
SAT_2M	1372	846	6.1046512	1	Singlet	Immune_cells
SAT_2M	2417	1249	1.3630731	10	Singlet	Immune_cells
SAT_2M	1716	980	6.1592098	1	Singlet	Immune_cells
SAT_2M	1421	910	0.0701262	4	Singlet	Immune_cells
SAT_2M	1062	703	0.656045	0	Singlet	Adipocyte
SAT_2M	5460	2437	9.0859332	9	Singlet	Mesothelial
SAT_2M	1639	1080	2.3142509	4	Singlet	Immune_cells
SAT_2M	2443	1367	0.1631987	0	Singlet	Adipocyte
SAT_2M	1295	820	7.1865443	1	Singlet	Immune_cells
SAT_2M	1620	971	4.1794714	4	Singlet	Immune_cells
SAT_2M	1547	927	6.4951768	1	Singlet	Immune_cells
SAT_2M	2361	1366	0.3764115	0	Singlet	Adipocyte
SAT_2M	1345	786	5.8475204	4	Singlet	Immune_cells
SAT_2M	1470	931	5.5555556	1	Singlet	Immune_cells
SAT_2M	2048	1161	0.3900536	0	Singlet	Adipocyte
SAT_2M	6703	2478	1.8562519	10	Singlet	Immune_cells
SAT_2M	2915	1541	2.6712329	1	Singlet	Immune_cells
SAT_2M	1972	1229	1.3144591	1	Singlet	Immune_cells
SAT_2M	1530	905	1.0437052	8	Singlet	Mesothelial
SAT_2M	1282	776	4.034135	1	Singlet	Immune_cells
SAT_2M	4296	2186	11.275758	9	Singlet	Mesothelial
SAT_2M	1511	926	3.3663366	4	Singlet	Immune_cells
SAT_2M	1520	913	3.6769534	1	Singlet	Immune_cells
SAT_2M	1226	791	3.2599837	1	Singlet	Immune_cells
SAT_2M	3333	1660	0.1793722	3	Singlet	FAPs
SAT_2M	1477	874	0.8097166	3	Singlet	FAPs
SAT_2M	1913	1157	0.729927	1	Singlet	Immune_cells
SAT_2M	2324	1262	2.016302	0	Singlet	Adipocyte
SAT_2M	1001	665	6.0636183	4	Singlet	Immune_cells
SAT_2M	1613	879	12.677798	1	Singlet	Immune_cells
SAT_2M	7798	3408	4.0578597	9	Singlet	Mesothelial
SAT_2M	1383	855	0.7225434	1	Singlet	Immune_cells
SAT_2M	1098	693	7.5523203	1	Singlet	Immune_cells
SAT_2M	1036	672	2.7911453	1	Singlet	Immune_cells
SAT_2M	1693	1050	0.4112808	0	Singlet	Adipocyte
SAT_2M	2980	1545	9.5110516	4	Singlet	Immune_cells
SAT_2M	1349	896	0.1470588	8	Singlet	Mesothelial
SAT_2M	1484	818	9.9062918	9	Singlet	Mesothelial
SAT_2M	1630	1064	3.3027523	1	Singlet	Immune_cells
SAT_2M	1781	1078	4.2553191	4	Singlet	Immune_cells
SAT_2M	7128	2744	3.7415919	10	Singlet	Immune_cells
SAT_2M	1306	847	1.065449	0	Singlet	Adipocyte
SAT_2M	1817	1082	2.1953897	5	Singlet	Immune_cells

SAT_2M	1330	891	0.3748126	8	Singlet	Mesothelial
SAT_2M	2574	1317	12.48068	1	Singlet	Immune_cells
SAT_2M	1170	757	0.6762468	3	Singlet	FAPs
SAT_2M	1632	1008	1.5834348	1	Singlet	Immune_cells
SAT_2M	1659	969	6.3359235	0	Singlet	Adipocyte
SAT_2M	1330	758	11.002994	1	Singlet	Immune_cells
SAT_2M	3298	1765	0.5142166	0	Singlet	Adipocyte
SAT_2M	1845	1060	0.9179266	1	Singlet	Immune_cells
SAT_2M	1970	1162	0.8080808	4	Singlet	Immune_cells
SAT_2M	1085	740	0.3673095	1	Singlet	Immune_cells
SAT_2M	3299	1660	5.4347826	0	Singlet	Adipocyte
SAT_2M	2097	1268	8.0965909	5	Singlet	Immune_cells
SAT_2M	1174	782	1.0143702	4	Singlet	Immune_cells
SAT_2M	1264	821	1.8882769	4	Singlet	Immune_cells
SAT_2M	1682	899	2.7893175	3	Singlet	FAPs
SAT_2M	1175	789	3.3898305	1	Singlet	Immune_cells
SAT_2M	1185	746	1.9343987	0	Singlet	Adipocyte
SAT_2M	1699	1010	0.7025761	3	Singlet	FAPs
SAT_2M	1808	1122	8.3838941	4	Singlet	Immune_cells
SAT_2M	1130	751	3.7069726	4	Singlet	Immune_cells
SAT_2M	2835	1426	4.1240747	10	Singlet	Immune_cells
SAT_2M	1238	759	0.8032129	1	Singlet	Immune_cells
SAT_2M	1054	698	0.8466604	4	Singlet	Immune_cells
SAT_2M	2541	1501	4.8311076	8	Singlet	Mesothelial
SAT_2M	1632	926	3.4756098	1	Singlet	Immune_cells
SAT_2M	1035	709	0.6730769	7	Singlet	Endothelial_cells
SAT_2M	1486	918	2.41773	1	Singlet	Immune_cells
SAT_2M	3021	1625	0.4618938	0	Singlet	Adipocyte
SAT_2M	1423	924	0.2102313	1	Singlet	Immune_cells
SAT_2M	1393	862	8.5714286	4	Singlet	Immune_cells
SAT_2M	1247	817	1.1217949	4	Singlet	Immune_cells
SAT_2M	1080	799	0.7373272	4	Singlet	Immune_cells
SAT_2M	4371	2018	1.7832647	2	Singlet	FAPs
SAT_2M	1343	918	0.6686478	4	Singlet	Immune_cells
SAT_2M	2553	1333	0.8987886	5	Singlet	Immune_cells
SAT_2M	4931	1858	0.7696982	10	Singlet	Immune_cells
SAT_2M	1046	710	0.4743833	5	Singlet	Immune_cells
SAT_2M	1892	1138	6.4873418	5	Singlet	Immune_cells
SAT_2M	1226	787	6.8292683	1	Singlet	Immune_cells
SAT_2M	1903	1106	2.6743576	1	Singlet	Immune_cells
SAT_2M	3063	1585	1.33507	3	Singlet	FAPs
SAT_2M	2764	1517	0.2506266	0	Singlet	Adipocyte
SAT_2M	1236	764	4.4141252	4	Singlet	Immune_cells
SAT_2M	12096	4127	11.184428	4	Singlet	Immune_cells
SAT_2M	1736	1023	0.2302821	1	Singlet	Immune_cells
SAT_2M	1552	927	2.1208226	1	Singlet	Immune_cells
SAT_2M	2039	1273	0.9794319	1	Singlet	Immune_cells
SAT_2M	5360	2029	1.2849162	10	Singlet	Immune_cells
SAT_2M	2235	1251	0.2678571	0	Singlet	Adipocyte
SAT_2M	1014	706	0.2944063	4	Singlet	Immune_cells
SAT_2M	1308	833	3.5714286	5	Singlet	Immune_cells

SAT_2M	1590	996	3.9448967	1	Singlet	Immune_cells
SAT_2M	1521	928	6.5003283	1	Singlet	Immune_cells
SAT_2M	3351	1638	12.555787	9	Singlet	Mesothelial
SAT_2M	1407	893	1.0623229	1	Singlet	Immune_cells
SAT_2M	1114	729	1.1669659	5	Singlet	Immune_cells
SAT_2M	1452	938	0.2061856	5	Singlet	Immune_cells
SAT_2M	2203	1188	8.6386251	5	Singlet	Immune_cells
SAT_2M	1538	940	1.8134715	1	Singlet	Immune_cells
SAT_2M	1223	722	0.4878049	3	Singlet	FAPs
SAT_2M	1866	1114	0.1599147	0	Singlet	Adipocyte
SAT_2M	1319	778	2.4187453	3	Singlet	FAPs
SAT_2M	3060	1603	9.517601	5	Singlet	Immune_cells
SAT_2M	1883	1084	0.2649709	0	Singlet	Adipocyte
SAT_2M	1624	1041	0.0613121	8	Singlet	Mesothelial
SAT_2M	2203	1274	5.6535504	4	Singlet	Immune_cells
SAT_2M	1665	986	4.9013748	9	Singlet	Mesothelial
SAT_2M	1311	824	1.5921152	1	Singlet	Immune_cells
SAT_2M	1385	854	0.8639309	1	Singlet	Immune_cells
SAT_2M	1045	706	2.0972355	0	Singlet	Adipocyte
SAT_2M	2446	1456	0.2442997	11	Singlet	Smooth_muscle_cells
SAT_2M	1175	783	2.4576271	9	Singlet	Mesothelial
SAT_2M	2795	1478	1.460114	0	Singlet	Adipocyte
SAT_2M	2125	1275	4.4600939	5	Singlet	Immune_cells
SAT_2M	1318	810	3.5391566	1	Singlet	Immune_cells
SAT_2M	1353	846	1.6212233	1	Singlet	Immune_cells
SAT_2M	1485	910	7.7436582	1	Singlet	Immune_cells
SAT_2M	2217	1268	1.0341727	0	Singlet	Adipocyte
SAT_2M	2004	1163	0.7936508	0	Singlet	Adipocyte
SAT_2M	3451	1789	1.0124385	5	Singlet	Immune_cells
SAT_2M	2806	1496	4.1888534	2	Singlet	FAPs
SAT_2M	2216	1353	2.374552	0	Singlet	Adipocyte
SAT_2M	1263	870	0.3162055	8	Singlet	Mesothelial
SAT_2M	1154	767	1.2942192	1	Singlet	Immune_cells
SAT_2M	1945	1086	2.6046987	0	Singlet	Adipocyte
SAT_2M	1707	1058	0	7	Singlet	Endothelial_cells
SAT_2M	1467	1036	0.2713704	8	Singlet	Mesothelial
SAT_2M	1153	764	0.7805724	4	Singlet	Immune_cells
SAT_2M	2058	1241	5.2606178	1	Singlet	Immune_cells
SAT_2M	1644	960	13.680387	9	Singlet	Mesothelial
SAT_2M	1019	671	3.7717602	0	Singlet	Adipocyte
SAT_2M	1984	1205	0.2008032	0	Singlet	Adipocyte
SAT_2M	4776	2141	4.3933054	9	Singlet	Mesothelial
SAT_2M	2086	1210	0.5243089	0	Singlet	Adipocyte
SAT_2M	1931	1175	4.0721649	1	Singlet	Immune_cells
SAT_2M	2175	1210	2.4736601	0	Singlet	Adipocyte
SAT_2M	1062	729	0.1876173	11	Singlet	Smooth_muscle_cells
SAT_2M	1162	754	2.7467811	1	Singlet	Immune_cells
SAT_2M	1273	815	0.5451713	0	Singlet	Adipocyte
SAT_2M	3929	1928	8.7430309	4	Singlet	Immune_cells
SAT_2M	1552	962	5.984556	1	Singlet	Immune_cells
SAT_2M	1196	805	0.3333333	1	Singlet	Immune_cells

SAT_2M	5675	2691	4.1380525	9	Singlet	Mesothelial
SAT_2M	3329	1703	1.1954573	0	Singlet	Adipocyte
SAT_2M	4971	2456	2.1669342	11	Singlet	Smooth_muscle_cells
SAT_2M	3306	1754	2.109069	11	Singlet	Smooth_muscle_cells
SAT_2M	1261	820	0.2371542	1	Singlet	Immune_cells
SAT_2M	1370	835	10.618182	4	Singlet	Immune_cells
SAT_2M	2053	1108	0.3406326	8	Singlet	Mesothelial
SAT_2M	1783	1098	5.7713652	1	Singlet	Immune_cells
SAT_2M	1106	711	0.1796945	5	Singlet	Immune_cells
SAT_2M	1001	689	0.3929273	4	Singlet	Immune_cells
SAT_2M	3031	1603	0.461285	3	Singlet	FAPs
SAT_2M	2624	1405	0.6056018	0	Singlet	Adipocyte
SAT_2M	1401	945	2.1126761	1	Singlet	Immune_cells
SAT_2M	1367	838	0.5098325	0	Singlet	Adipocyte
SAT_2M	3474	1876	5.520414	8	Singlet	Mesothelial
SAT_2M	1240	764	2.7375201	4	Singlet	Immune_cells
SAT_2M	1468	878	0.2717391	1	Singlet	Immune_cells
SAT_2M	2244	1194	5.4270463	4	Singlet	Immune_cells
SAT_2M	1652	1010	1.1390887	0	Singlet	Adipocyte
SAT_2M	1223	780	7.4979625	1	Singlet	Immune_cells
SAT_2M	1332	881	0.2237136	4	Singlet	Immune_cells
SAT_2M	1913	1166	0.9880395	0	Singlet	Adipocyte
SAT_2M	1516	909	2.8852459	4	Singlet	Immune_cells
SAT_2M	2716	1576	0.7328692	11	Singlet	Smooth_muscle_cells
SAT_2M	2211	1249	0.2242152	6	Singlet	Neurons
SAT_2M	1987	1143	8.1256231	1	Singlet	Immune_cells
SAT_2M	1565	903	2.0382166	5	Singlet	Immune_cells
SAT_2M	2040	1219	1.2195122	0	Singlet	Adipocyte
SAT_2M	1341	822	4.0029652	1	Singlet	Immune_cells
SAT_2M	2379	1282	1.6270338	1	Singlet	Immune_cells
SAT_2M	3571	1463	2.1818182	10	Singlet	Immune_cells
SAT_2M	1659	1010	3.2432432	4	Singlet	Immune_cells
SAT_2M	1868	1085	0.3725386	0	Singlet	Adipocyte
SAT_2M	2062	1209	3.52657	1	Singlet	Immune_cells
SAT_2M	1219	746	1.1409943	0	Singlet	Adipocyte
SAT_2M	1484	870	2.020202	2	Singlet	FAPs
SAT_2M	1024	685	0.2901354	1	Singlet	Immune_cells
SAT_2M	1251	798	5.6573705	1	Singlet	Immune_cells
SAT_2M	2067	1191	0.574988	0	Singlet	Adipocyte
SAT_2M	1564	949	2.3536896	1	Singlet	Immune_cells
SAT_2M	2700	1409	0.2583979	0	Singlet	Adipocyte
SAT_2M	1384	897	1.294964	4	Singlet	Immune_cells
SAT_2M	7760	3265	3.2540193	9	Singlet	Mesothelial
SAT_2M	2262	1171	3.2541777	10	Singlet	Immune_cells
SAT_2M	2809	1585	0.7097232	11	Singlet	Smooth_muscle_cells
SAT_2M	1516	920	2.6281209	1	Singlet	Immune_cells
SAT_2M	2974	1628	0.7380074	3	Singlet	FAPs
SAT_2M	1558	993	1.342711	4	Singlet	Immune_cells
SAT_2M	1052	709	2.1800948	1	Singlet	Immune_cells
SAT_2M	1428	872	0.9769714	1	Singlet	Immune_cells
SAT_2M	1257	820	5.1221434	1	Singlet	Immune_cells

SAT_2M	1146	771	1.1304348	4	Singlet	Immune_cells
SAT_2M	2346	1233	5.6050955	1	Singlet	Immune_cells
SAT_2M	1126	772	0.5291005	4	Singlet	Immune_cells
SAT_2M	1420	877	9.0653549	8	Singlet	Mesothelial
SAT_2M	1392	878	5.8823529	4	Singlet	Immune_cells
SAT_2M	1638	1032	1.8248175	4	Singlet	Immune_cells
SAT_2M	1372	879	0.4354136	4	Singlet	Immune_cells
SAT_2M	3610	1590	3.8153166	10	Singlet	Immune_cells
SAT_2M	1218	711	7.704918	1	Singlet	Immune_cells
SAT_2M	1509	979	0.1986755	4	Singlet	Immune_cells
SAT_2M	3825	1876	0.1560874	0	Singlet	Adipocyte
SAT_2M	1467	925	6.3902107	11	Singlet	Smooth_muscle_cells
SAT_2M	1835	1125	1.4673913	4	Singlet	Immune_cells
SAT_2M	1884	1069	0.2631579	0	Singlet	Adipocyte
SAT_2M	1250	768	1.5139442	0	Singlet	Adipocyte
SAT_2M	1363	865	2.489019	1	Singlet	Immune_cells
SAT_2M	1154	759	0.4314064	0	Singlet	Adipocyte
SAT_2M	1426	838	9.623431	1	Singlet	Immune_cells
SAT_2M	1214	789	5.2631579	1	Singlet	Immune_cells
SAT_2M	2065	1172	0.3866602	0	Singlet	Adipocyte
SAT_2M	1011	687	0.3933137	4	Singlet	Immune_cells
SAT_2M	2375	1307	0.0419287	0	Singlet	Adipocyte
SAT_2M	1288	769	0.0775194	4	Singlet	Immune_cells
SAT_2M	2148	1238	1.7674419	4	Singlet	Immune_cells
SAT_2M	2454	1261	7.9075426	10	Singlet	Immune_cells
SAT_2M	1881	1159	5.076679	1	Singlet	Immune_cells
SAT_2M	1729	1012	2.9936672	5	Singlet	Immune_cells
SAT_2M	1487	900	1.8084394	0	Singlet	Adipocyte
SAT_2M	1235	757	4.7504026	1	Singlet	Immune_cells
SAT_2M	1736	1059	0.5163511	0	Singlet	Adipocyte
SAT_2M	1882	1135	5.2436441	4	Singlet	Immune_cells
SAT_2M	1487	898	2.1390374	1	Singlet	Immune_cells
SAT_2M	1550	964	0.2559181	4	Singlet	Immune_cells
SAT_2M	1274	762	0.0782473	4	Singlet	Immune_cells
SAT_2M	3538	1922	0.1408848	5	Singlet	Immune_cells
SAT_2M	1594	977	0.9968847	0	Singlet	Adipocyte
SAT_2M	1824	1093	0.6553796	4	Singlet	Immune_cells
SAT_2M	1538	908	0.1296176	0	Singlet	Adipocyte
SAT_2M	1057	734	0.656045	6	Singlet	Neurons
SAT_2M	1181	753	1.3422819	1	Singlet	Immune_cells
SAT_2M	1946	1065	2.6060296	10	Singlet	Immune_cells
SAT_2M	1055	713	0.1886792	4	Singlet	Immune_cells
SAT_2M	1683	1021	9.0425532	1	Singlet	Immune_cells
SAT_2M	2291	1238	7.8627281	1	Singlet	Immune_cells
SAT_2M	1878	960	13.716108	1	Singlet	Immune_cells
SAT_2M	1587	1049	0.6870706	4	Singlet	Immune_cells
SAT_2M	1425	892	0.1386963	4	Singlet	Immune_cells
SAT_2M	1707	1009	0.93622	0	Singlet	Adipocyte
SAT_2M	1516	894	5.4641211	0	Singlet	Adipocyte
SAT_2M	1628	909	7.2171254	0	Singlet	Adipocyte
SAT_2M	1035	616	3.7681159	0	Singlet	Adipocyte

SAT_2M	1224	776	4.5602606	1	Singlet	Immune_cells
SAT_2M	1535	954	2.8589994	1	Singlet	Immune_cells
SAT_2M	1610	886	0.9305211	9	Singlet	Mesothelial
SAT_2M	5857	2688	1.533481	9	Singlet	Mesothelial
SAT_2M	3343	1854	0.506103	9	Singlet	Mesothelial
SAT_2M	1207	777	0.0825083	1	Singlet	Immune_cells
SAT_2M	1028	729	2.1174206	5	Singlet	Immune_cells
SAT_2M	1368	784	2.189781	0	Singlet	Adipocyte
SAT_2M	1210	775	0.5733006	0	Singlet	Adipocyte
SAT_2M	1899	1158	0.2103049	11	Singlet	Smooth_muscle_cells
SAT_2M	2303	1282	0.5632582	0	Singlet	Adipocyte
SAT_2M	5475	1614	1.075857	10	Singlet	Immune_cells
SAT_2M	4075	1927	0.8576329	10	Singlet	Immune_cells
SAT_2M	3394	1774	0.2054593	0	Singlet	Adipocyte
SAT_2M	5328	2196	2.4340011	10	Singlet	Immune_cells
SAT_2M	3630	1817	0.2743484	3	Singlet	FAPs
SAT_2M	1931	1085	8.0061983	1	Singlet	Immune_cells
SAT_2M	1212	787	1.5599343	0	Singlet	Adipocyte
SAT_2M	1844	1089	1.8388318	1	Singlet	Immune_cells
SAT_2M	2123	1247	0.5164319	5	Singlet	Immune_cells
SAT_2M	1491	948	2.1361816	1	Singlet	Immune_cells
SAT_2M	2192	1293	4.7792444	1	Singlet	Immune_cells
SAT_2M	1431	890	5.4166667	1	Singlet	Immune_cells
SAT_2M	4577	2269	0.0654165	5	Singlet	Immune_cells
SAT_2M	1557	863	11.139565	7	Singlet	Endothelial_cells
SAT_2M	1216	846	2.8571429	5	Singlet	Immune_cells
SAT_2M	1095	779	1.5440509	11	Singlet	Smooth_muscle_cells
SAT_2M	2130	1247	3.0444965	4	Singlet	Immune_cells
SAT_2M	1370	822	0.3628447	0	Singlet	Adipocyte
SAT_2M	1238	748	0.7228916	3	Singlet	FAPs
SAT_2M	1695	1062	3.8732394	1	Singlet	Immune_cells
SAT_2M	1632	965	0.794621	3	Singlet	FAPs
SAT_2M	3592	1562	6.4812239	10	Singlet	Immune_cells
SAT_2M	2203	1287	1.1312217	0	Singlet	Adipocyte
SAT_2M	3015	1583	1.8874172	1	Singlet	Immune_cells
SAT_2M	2055	1216	0.4356244	0	Singlet	Adipocyte
SAT_2M	1504	894	12.060968	1	Singlet	Immune_cells
SAT_2M	1260	820	1.5810277	4	Singlet	Immune_cells
SAT_2M	1053	744	1.8570102	4	Singlet	Immune_cells
SAT_2M	2148	1316	0.1395349	2	Singlet	FAPs
SAT_2M	2234	1280	0.0446429	8	Singlet	Mesothelial
SAT_2M	1006	691	1.4851485	1	Singlet	Immune_cells
SAT_2M	1663	1039	0.0596303	0	Singlet	Adipocyte
SAT_2M	3855	2004	7.4696304	9	Singlet	Mesothelial
SAT_2M	1916	1189	4.2730589	1	Singlet	Immune_cells
SAT_2M	1212	783	4.7502048	1	Singlet	Immune_cells
SAT_2M	2171	1213	9.2916283	4	Singlet	Immune_cells
SAT_2M	1826	1074	0.1094092	5	Singlet	Immune_cells
SAT_2M	1523	941	6.2704115	1	Singlet	Immune_cells
SAT_2M	1795	1013	4.6008869	7	Singlet	Endothelial_cells
SAT_2M	1964	1254	4.6216353	5	Singlet	Immune_cells

SAT_2M	1637	1054	0.968523	3	Singlet	FAPs
SAT_2M	1223	667	0.7287449	6	Singlet	Neurons
SAT_2M	2210	1335	3.0234657	4	Singlet	Immune_cells
SAT_2M	2549	1494	1.3586957	6	Singlet	Neurons
SAT_2M	1459	918	0.5453306	1	Singlet	Immune_cells
SAT_2M	1925	1111	3.3678756	1	Singlet	Immune_cells
SAT_2M	2240	1382	1.8666667	5	Singlet	Immune_cells
SAT_2M	1124	739	0.8888889	5	Singlet	Immune_cells
SAT_2M	1280	839	1.4751553	1	Singlet	Immune_cells
SAT_2M	2011	1180	2.026693	3	Singlet	FAPs
SAT_2M	1777	1017	7.2869955	3	Singlet	FAPs
SAT_2M	2243	1235	0.6219458	0	Singlet	Adipocyte
SAT_2M	1231	792	1.1363636	1	Singlet	Immune_cells
SAT_2M	1581	970	3.2683847	11	Singlet	Smooth_muscle_cells
SAT_2M	1145	765	0.6097561	1	Singlet	Immune_cells
SAT_2M	1443	919	1.5214385	1	Singlet	Immune_cells
SAT_2M	1240	812	4.9719326	1	Singlet	Immune_cells
SAT_2M	1071	697	2.1435228	0	Singlet	Adipocyte
SAT_2M	1432	903	5.409154	1	Singlet	Immune_cells
SAT_2M	2022	1256	0.4926108	7	Singlet	Endothelial_cells
SAT_2M	1354	858	6.5585851	1	Singlet	Immune_cells
SAT_2M	1760	1081	0.1132503	4	Singlet	Immune_cells
SAT_2M	1801	1074	9.1463415	1	Singlet	Immune_cells
SAT_2M	1101	758	1.9004525	4	Singlet	Immune_cells
SAT_2M	1627	1009	6.6217045	1	Singlet	Immune_cells
SAT_2M	1161	782	3.3419023	1	Singlet	Immune_cells
SAT_2M	2283	1308	2.5696864	1	Singlet	Immune_cells
SAT_2M	1844	1086	0.1622499	0	Singlet	Adipocyte
SAT_2M	7017	2815	7.7338641	9	Singlet	Mesothelial
SAT_2M	1037	686	0.481232	1	Singlet	Immune_cells
SAT_2M	1199	745	7.3272273	1	Singlet	Immune_cells
SAT_2M	2589	1481	0.3457549	0	Singlet	Adipocyte
SAT_2M	1422	896	0.6271777	4	Singlet	Immune_cells
SAT_2M	1241	824	2.3961661	4	Singlet	Immune_cells
SAT_2M	1999	1206	0.8928571	0	Singlet	Adipocyte
SAT_2M	1208	789	0.8203445	4	Singlet	Immune_cells
SAT_2M	1494	917	1.4046823	0	Singlet	Adipocyte
SAT_2M	2184	1254	0.6843066	0	Singlet	Adipocyte
SAT_2M	1656	989	4.736211	1	Singlet	Immune_cells
SAT_2M	1231	808	0.0808407	4	Singlet	Immune_cells
SAT_2M	1083	742	1.0999083	4	Singlet	Immune_cells
SAT_2M	1536	1006	0.8414239	5	Singlet	Immune_cells
SAT_2M	1315	868	0.531511	2	Singlet	FAPs
SAT_2M	1375	853	0.1447178	1	Singlet	Immune_cells
SAT_2M	1273	807	1.4139827	1	Singlet	Immune_cells
SAT_2M	2433	1180	9.6959737	10	Singlet	Immune_cells
SAT_2M	1365	886	0.8029197	0	Singlet	Adipocyte
SAT_2M	1793	1041	0.6677796	0	Singlet	Adipocyte
SAT_2M	1590	966	0	1	Singlet	Immune_cells
SAT_2M	3129	1667	0.4141446	0	Singlet	Adipocyte
SAT_2M	4593	2265	12.538027	4	Singlet	Immune_cells

SAT_2M	1871	1178	0.3733333	4	Singlet	Immune_cells
SAT_2M	1174	813	0.3392706	4	Singlet	Immune_cells
SAT_2M	1086	703	1.1948529	0	Singlet	Adipocyte
SAT_2M	2442	1160	5.9232026	10	Singlet	Immune_cells
SAT_2M	1370	867	0.7976795	0	Singlet	Adipocyte
SAT_2M	1485	970	0.7343124	11	Singlet	Smooth_muscle_cells
SAT_2M	2551	1509	5.3104256	9	Singlet	Mesothelial
SAT_2M	2905	1595	0.0343761	8	Singlet	Mesothelial
SAT_2M	1585	1037	0.4380476	4	Singlet	Immune_cells
SAT_2M	1252	836	0.3167063	4	Singlet	Immune_cells
SAT_2M	1229	835	3.4677419	4	Singlet	Immune_cells
SAT_2M	1294	805	2.0061728	4	Singlet	Immune_cells
SAT_2M	2671	1594	2.0096762	9	Singlet	Mesothelial
SAT_2M	1820	1177	0.5455537	0	Singlet	Adipocyte
SAT_2M	4377	2223	4.3903549	4	Singlet	Immune_cells
SAT_2M	2096	1204	0.3320683	0	Singlet	Adipocyte
SAT_2M	1862	1177	0.5356186	4	Singlet	Immune_cells
SAT_2M	1382	908	3.030303	5	Singlet	Immune_cells
SAT_2M	1853	1133	0.3745318	0	Singlet	Adipocyte
SAT_2M	1588	934	0.2512563	1	Singlet	Immune_cells
SAT_2M	3253	1552	1.563458	10	Singlet	Immune_cells
SAT_2M	1392	875	3.9341917	3	Singlet	FAPs
SAT_2M	1378	830	6.6618392	1	Singlet	Immune_cells
SAT_2M	1230	799	0.8116883	9	Singlet	Mesothelial
SAT_2M	1217	786	1.7944535	1	Singlet	Immune_cells
SAT_2M	1607	1040	0.1856436	4	Singlet	Immune_cells
SAT_2M	1777	1087	1.1198208	0	Singlet	Adipocyte
SAT_2M	1082	739	8.5086917	3	Singlet	FAPs
SAT_2M	1015	636	0.6889764	0	Singlet	Adipocyte
SAT_2M	1914	1185	0.6767309	4	Singlet	Immune_cells
SAT_2M	1179	715	3.7288136	5	Singlet	Immune_cells
SAT_2M	1217	800	3.755102	9	Singlet	Mesothelial
SAT_2M	1341	851	6.9784707	1	Singlet	Immune_cells
SAT_2M	2729	1530	0.5845817	7	Singlet	Endothelial_cells
SAT_2M	2065	1247	0.4343629	0	Singlet	Adipocyte
SAT_2M	3371	1763	0.5325444	0	Singlet	Adipocyte
SAT_2M	2283	1250	5.0958188	1	Singlet	Immune_cells
SAT_2M	1871	1216	0.1600854	4	Singlet	Immune_cells
SAT_2M	1030	692	1.2536162	4	Singlet	Immune_cells
SAT_2M	1624	993	1.8326206	0	Singlet	Adipocyte
SAT_2M	1051	681	0.095057	4	Singlet	Immune_cells
SAT_2M	2057	1210	0.0485437	8	Singlet	Mesothelial
SAT_2M	4897	2267	0.650671	2	Singlet	FAPs
SAT_2M	1530	922	0.5204945	1	Singlet	Immune_cells
SAT_2M	1176	794	0.4222973	4	Singlet	Immune_cells
SAT_2M	1286	837	0.3866976	1	Singlet	Immune_cells
SAT_2M	3461	1741	0.3159104	0	Singlet	Adipocyte
SAT_2M	1459	953	4.9897471	1	Singlet	Immune_cells
SAT_2M	3460	1744	0.3741007	0	Singlet	Adipocyte
SAT_2M	6591	2904	7.927846	9	Singlet	Mesothelial
SAT_2M	1395	812	4.7686833	9	Singlet	Mesothelial

SAT_2M	3045	1583	0.3595946	8	Singlet	Mesothelial
SAT_2M	2530	1457	1.7619421	6	Singlet	Neurons
SAT_2M	2347	1379	0.2541296	0	Singlet	Adipocyte
SAT_2M	1252	746	1.9904459	0	Singlet	Adipocyte
SAT_2M	1700	972	7.9859072	1	Singlet	Immune_cells
SAT_2M	1077	734	3.7002775	4	Singlet	Immune_cells
SAT_2M	1227	764	10.008071	1	Singlet	Immune_cells
SAT_2M	1008	634	0.4945598	0	Singlet	Adipocyte
SAT_2M	1643	984	1.2091898	3	Singlet	FAPs
SAT_2M	1703	1016	0.877193	0	Singlet	Adipocyte
SAT_2M	2468	1331	2.9435484	9	Singlet	Mesothelial
SAT_2M	1155	755	0.1716738	1	Singlet	Immune_cells
SAT_2M	1441	915	0.8934708	4	Singlet	Immune_cells
SAT_2M	1113	719	0.8035714	1	Singlet	Immune_cells
SAT_2M	4572	1938	9.5935315	0	Singlet	Adipocyte
SAT_2M	3076	1651	0.064704	0	Singlet	Adipocyte
SAT_2M	1321	830	5.7185854	1	Singlet	Immune_cells
SAT_2M	2283	1297	4.317488	1	Singlet	Immune_cells
SAT_2M	1795	1058	4.8199446	1	Singlet	Immune_cells
SAT_2M	11173	3842	8.7042002	9	Singlet	Mesothelial
SAT_2M	1419	820	9.4736842	1	Singlet	Immune_cells
SAT_2M	5604	2100	1.0509441	10	Singlet	Immune_cells
SAT_2M	1468	890	4.2090971	1	Singlet	Immune_cells
SAT_2M	1216	717	13.078798	1	Singlet	Immune_cells
SAT_2M	1630	1026	0.18226	4	Singlet	Immune_cells
SAT_2M	1347	842	1.8518519	1	Singlet	Immune_cells
SAT_2M	2357	1361	5.0042409	9	Singlet	Mesothelial
SAT_2M	1265	795	0.3921569	1	Singlet	Immune_cells
SAT_2M	2297	1368	0.1300954	5	Singlet	Immune_cells
SAT_2M	1310	854	2.7987897	1	Singlet	Immune_cells
SAT_2M	1150	770	0.8561644	0	Singlet	Adipocyte
SAT_2M	1445	937	4.4704264	4	Singlet	Immune_cells
SAT_2M	1914	1108	5.0962038	8	Singlet	Mesothelial
SAT_2M	1920	1159	3.9937759	1	Singlet	Immune_cells
SAT_2M	1277	846	1.399689	4	Singlet	Immune_cells
SAT_2M	1037	690	2.7911453	1	Singlet	Immune_cells
SAT_2M	1124	720	0.0888099	4	Singlet	Immune_cells
SAT_2M	2178	1404	0.2284148	4	Singlet	Immune_cells
SAT_2M	1289	946	0.7621951	6	Singlet	Neurons
SAT_2M	2561	1353	0.4674718	0	Singlet	Adipocyte
SAT_2M	1275	786	4.4600939	1	Singlet	Immune_cells
SAT_2M	1090	742	0.0909091	2	Singlet	FAPs
SAT_2M	2872	1725	5.5536272	4	Singlet	Immune_cells
SAT_2M	1860	1097	6.387547	1	Singlet	Immune_cells
SAT_2M	2247	1176	3.7316748	10	Singlet	Immune_cells
SAT_2M	1477	917	0.4026846	0	Singlet	Adipocyte
SAT_2M	1471	954	4.0650407	4	Singlet	Immune_cells
SAT_2M	3899	1944	0.6398771	0	Singlet	Adipocyte
SAT_2M	1242	763	6.5060241	1	Singlet	Immune_cells
SAT_2M	1679	1041	0.9478673	1	Singlet	Immune_cells
SAT_2M	2084	1137	9.8659004	1	Singlet	Immune_cells

SAT_2M	1059	709	0.6610009	4	Singlet	Immune_cells
SAT_2M	1618	1033	0.617284	1	Singlet	Immune_cells
SAT_2M	1241	815	0.0805802	4	Singlet	Immune_cells
SAT_2M	3308	1586	1.5379976	10	Singlet	Immune_cells
SAT_2M	1246	809	7.1770335	11	Singlet	Smooth_muscle_cells
SAT_2M	1235	775	1.5310234	0	Singlet	Adipocyte
SAT_2M	1697	969	5.8201058	1	Singlet	Immune_cells
SAT_2M	1440	940	0.2773925	4	Singlet	Immune_cells
SAT_2M	2511	1318	0.0793966	8	Singlet	Mesothelial
SAT_2M	1301	851	0.228833	3	Singlet	FAPs
SAT_2M	1506	976	0.6596306	1	Singlet	Immune_cells
SAT_2M	1498	902	5.1162791	3	Singlet	FAPs
SAT_2M	1086	684	2.9384757	1	Singlet	Immune_cells
SAT_2M	1927	1158	3.5105834	1	Singlet	Immune_cells
SAT_2M	1789	1224	0.8328706	4	Singlet	Immune_cells
SAT_2M	1807	1142	4.5755237	1	Singlet	Immune_cells
SAT_2M	1816	1111	0.6037322	0	Singlet	Adipocyte
SAT_2M	1806	1183	2.092511	4	Singlet	Immune_cells
SAT_2M	1380	837	4.6209386	1	Singlet	Immune_cells
SAT_2M	2435	1405	0.5716619	0	Singlet	Adipocyte
SAT_2M	4303	2014	2.2495362	0	Singlet	Adipocyte
SAT_2M	1488	906	9.0545939	1	Singlet	Immune_cells
SAT_2M	1472	912	0.807537	0	Singlet	Adipocyte
SAT_2M	1672	1011	0.5360334	0	Singlet	Adipocyte
SAT_2M	1229	768	0	4	Singlet	Immune_cells
SAT_2M	2049	1198	1.3113162	0	Singlet	Adipocyte
SAT_2M	1006	669	0.5934718	1	Singlet	Immune_cells
SAT_2M	1409	940	0.3523608	8	Singlet	Mesothelial
SAT_2M	1310	829	6.231003	1	Singlet	Immune_cells
SAT_2M	1477	969	3.3085753	1	Singlet	Immune_cells
SAT_2M	1233	929	0.7003891	6	Singlet	Neurons
SAT_2M	1300	771	5.5981595	1	Singlet	Immune_cells
SAT_2M	1514	938	12.352168	5	Singlet	Immune_cells
SAT_2M	1297	819	2.8439662	1	Singlet	Immune_cells
SAT_2M	1238	811	0.882825	1	Singlet	Immune_cells
SAT_2M	1897	1168	1.1560694	0	Singlet	Adipocyte
SAT_2M	1136	799	0.1751313	2	Singlet	FAPs
SAT_2M	1042	710	0.8563273	4	Singlet	Immune_cells
SAT_2M	1552	1013	0.6422608	8	Singlet	Mesothelial
SAT_2M	1698	963	10.076157	1	Singlet	Immune_cells
SAT_2M	1566	844	6.7049808	0	Singlet	Adipocyte
SAT_2M	1236	799	3.0645161	1	Singlet	Immune_cells
SAT_2M	1973	1163	0.353714	8	Singlet	Mesothelial
SAT_2M	2005	1215	0.3964321	4	Singlet	Immune_cells
SAT_2M	1493	954	0.9352037	1	Singlet	Immune_cells
SAT_2M	1424	907	0.3501401	1	Singlet	Immune_cells
SAT_2M	1321	798	7.3906486	1	Singlet	Immune_cells
SAT_2M	2030	1229	0.246063	5	Singlet	Immune_cells
SAT_2M	1418	863	1.5460295	1	Singlet	Immune_cells
SAT_2M	1755	1096	6.5834279	1	Singlet	Immune_cells
SAT_2M	2123	1311	0.6539	5	Singlet	Immune_cells

SAT_2M	2547	1405	6.0297572	1	Singlet	Immune_cells
SAT_2M	1292	804	3.0139104	1	Singlet	Immune_cells
SAT_2M	1505	956	7.8444298	1	Singlet	Immune_cells
SAT_2M	2583	1418	3.3500193	0	Singlet	Adipocyte
SAT_2M	1345	843	0.8862629	1	Singlet	Immune_cells
SAT_2M	1075	721	0.1851852	1	Singlet	Immune_cells
SAT_2M	1337	858	1.1160714	4	Singlet	Immune_cells
SAT_2M	3971	1964	0.4003002	0	Singlet	Adipocyte
SAT_2M	1328	787	1.9461078	5	Singlet	Immune_cells
SAT_2M	1554	977	1.5404365	3	Singlet	FAPs
SAT_2M	2587	1415	0.2312139	0	Singlet	Adipocyte
SAT_2M	1905	1156	0.4175365	1	Singlet	Immune_cells
SAT_2M	1311	883	0.7587253	4	Singlet	Immune_cells
SAT_2M	1389	932	4.7959914	1	Singlet	Immune_cells
SAT_2M	1418	900	0.7703081	1	Singlet	Immune_cells
SAT_2M	1125	797	0.4428698	4	Singlet	Immune_cells
SAT_2M	1354	831	9.0707965	1	Singlet	Immune_cells
SAT_2M	2186	1323	2.5932666	4	Singlet	Immune_cells
SAT_2M	1363	795	11.670314	1	Singlet	Immune_cells
SAT_2M	1083	707	0.5529954	4	Singlet	Immune_cells
SAT_2M	2451	1374	1.9934906	10	Singlet	Immune_cells
SAT_2M	1110	745	0.4468275	0	Singlet	Adipocyte
SAT_2M	2219	1240	0.1345895	0	Singlet	Adipocyte
SAT_2M	2547	1392	0.1174168	0	Singlet	Adipocyte
SAT_2M	1082	715	0.3676471	1	Singlet	Immune_cells
SAT_2M	1437	934	0.3467406	0	Singlet	Adipocyte
SAT_2M	3167	1688	3.7118591	8	Singlet	Mesothelial
SAT_2M	1873	924	6.8617021	10	Singlet	Immune_cells
SAT_2M	1016	683	3.4246575	1	Singlet	Immune_cells
SAT_2M	1901	1218	0.4195071	11	Singlet	Smooth_muscle_cells
SAT_2M	1672	1035	0.4169148	4	Singlet	Immune_cells
SAT_2M	1678	992	2.1403092	1	Singlet	Immune_cells
SAT_2M	2247	1238	0.4	0	Singlet	Adipocyte
SAT_2M	1180	813	0.4219409	4	Singlet	Immune_cells
SAT_2M	1365	776	2.5491624	10	Singlet	Immune_cells
SAT_2M	1792	1172	3.0353201	4	Singlet	Immune_cells
SAT_2M	1625	935	0.122399	2	Singlet	FAPs
SAT_2M	1232	857	0.4842615	8	Singlet	Mesothelial
SAT_2M	1644	1000	6.2085594	1	Singlet	Immune_cells
SAT_2M	1370	931	0.6535948	8	Singlet	Mesothelial
SAT_2M	1290	839	0.2307692	8	Singlet	Mesothelial
SAT_2M	1537	1014	0.130039	4	Singlet	Immune_cells
SAT_2M	1227	815	2.6785714	1	Singlet	Immune_cells
SAT_2M	1213	751	1.2274959	0	Singlet	Adipocyte
SAT_2M	2321	1460	0.0428266	8	Singlet	Mesothelial
SAT_2M	1644	1038	2.9714979	4	Singlet	Immune_cells
SAT_2M	1720	1017	0.4624277	3	Singlet	FAPs
SAT_2M	4302	2001	0.7649513	10	Singlet	Immune_cells
SAT_2M	1216	765	0.5700326	1	Singlet	Immune_cells
SAT_2M	2063	1187	3.5800677	1	Singlet	Immune_cells
SAT_2M	1298	823	0.2304147	0	Singlet	Adipocyte

SAT_2M	1271	812	0.7014809	1	Singlet	Immune_cells
SAT_2M	2240	1226	8.7789661	1	Singlet	Immune_cells
SAT_2M	1041	630	1.5355086	0	Singlet	Adipocyte
SAT_2M	3568	1834	1.2273361	2	Singlet	FAPs
SAT_2M	2861	1619	4.2582897	9	Singlet	Mesothelial
SAT_2M	1054	689	0.1895735	1	Singlet	Immune_cells
SAT_2M	1923	1189	0.2575992	11	Singlet	Smooth_muscle_cells
SAT_2M	1550	953	4.8293625	1	Singlet	Immune_cells
SAT_2M	1076	674	6.2037037	0	Singlet	Adipocyte
SAT_2M	2365	1391	3.7489469	8	Singlet	Mesothelial
SAT_2M	1821	1082	0.5452563	3	Singlet	FAPs
SAT_2M	1165	772	0.2570694	1	Singlet	Immune_cells
SAT_2M	1036	699	1.4450867	4	Singlet	Immune_cells
SAT_2M	4678	2092	2.5630073	10	Singlet	Immune_cells
SAT_2M	1738	1076	5.8486239	1	Singlet	Immune_cells
SAT_2M	1676	950	2.1454112	1	Singlet	Immune_cells
SAT_2M	1145	745	0.8680556	4	Singlet	Immune_cells
SAT_2M	1466	922	0.4078858	5	Singlet	Immune_cells
SAT_2M	2482	1310	0.2009646	0	Singlet	Adipocyte
SAT_2M	1255	765	1.9032514	0	Singlet	Adipocyte
SAT_2M	1096	757	2.1798365	1	Singlet	Immune_cells
SAT_2M	1201	808	0.4149378	7	Singlet	Endothelial_cells
SAT_2M	1946	1230	0.4601227	4	Singlet	Immune_cells
SAT_2M	2702	1395	9.0439276	9	Singlet	Mesothelial
SAT_2M	2080	1236	1.9645424	4	Singlet	Immune_cells
SAT_2M	1010	618	4.0275049	7	Singlet	Endothelial_cells
SAT_2M	1804	1103	0.2759382	0	Singlet	Adipocyte
SAT_2M	1955	1206	1.1178862	0	Singlet	Adipocyte
SAT_2M	2586	1458	5.0788765	2	Singlet	FAPs
SAT_2M	2908	1508	0.4796163	0	Singlet	Adipocyte
SAT_2M	1036	665	0.8628955	0	Singlet	Adipocyte
SAT_2M	4274	2318	8.9911256	9	Singlet	Mesothelial
SAT_2M	1004	666	0.1980198	4	Singlet	Immune_cells
SAT_2M	1311	848	0.3023432	1	Singlet	Immune_cells
SAT_2M	2212	1276	3.1981982	0	Singlet	Adipocyte
SAT_2M	1750	947	10	9	Singlet	Mesothelial
SAT_2M	1489	932	4.736491	1	Singlet	Immune_cells
SAT_2M	1814	1106	0.8237232	0	Singlet	Adipocyte
SAT_2M	1329	887	0.4477612	1	Singlet	Immune_cells
SAT_2M	2605	1349	0.4980843	0	Singlet	Adipocyte
SAT_2M	1449	886	3.2391454	1	Singlet	Immune_cells
SAT_2M	2168	1329	0.5064457	11	Singlet	Smooth_muscle_cells
SAT_2M	1547	1001	2.0539153	4	Singlet	Immune_cells
SAT_2M	1188	819	7.3701843	4	Singlet	Immune_cells
SAT_2M	1392	882	0.5017921	0	Singlet	Adipocyte
SAT_2M	1541	931	5.7679844	1	Singlet	Immune_cells
SAT_2M	1146	771	0.3478261	1	Singlet	Immune_cells
SAT_2M	7101	2879	1.6451069	9	Singlet	Mesothelial
SAT_2M	1724	1079	3.1070196	1	Singlet	Immune_cells
SAT_2M	3254	1536	3.1183124	3	Singlet	FAPs
SAT_2M	1883	1179	0.1584786	0	Singlet	Adipocyte

SAT_2M	2013	1171	1.8802573	0	Singlet	Adipocyte
SAT_2M	1597	976	1.7478152	1	Singlet	Immune_cells
SAT_2M	1826	1097	0.436205	8	Singlet	Mesothelial
SAT_2M	1257	808	5.621536	9	Singlet	Mesothelial
SAT_2M	1584	914	5.7898049	0	Singlet	Adipocyte
SAT_2M	1190	745	9.0606816	1	Singlet	Immune_cells
SAT_2M	1907	1174	4.3251694	1	Singlet	Immune_cells
SAT_2M	1500	976	0.5984043	0	Singlet	Adipocyte
SAT_2M	1244	854	2.3961661	0	Singlet	Adipocyte
SAT_2M	5219	2160	2.8702641	10	Singlet	Immune_cells
SAT_2M	1935	1052	3.9134912	0	Singlet	Adipocyte
SAT_2M	1232	776	4.6116505	1	Singlet	Immune_cells
SAT_2M	1125	733	1.4171833	0	Singlet	Adipocyte
SAT_2M	1270	835	9.8668755	5	Singlet	Immune_cells
SAT_2M	1055	709	0.6585136	1	Singlet	Immune_cells
SAT_2M	2433	1342	2.49693	9	Singlet	Mesothelial
SAT_2M	1353	821	3.3923304	1	Singlet	Immune_cells
SAT_2M	1533	982	0.4545455	7	Singlet	Endothelial_cells
SAT_2M	1366	872	3.7790698	1	Singlet	Immune_cells
SAT_2M	1950	1185	0.3061224	4	Singlet	Immune_cells
SAT_2M	1098	774	0.9963768	0	Singlet	Adipocyte
SAT_2M	2118	1312	0.3762935	4	Singlet	Immune_cells
SAT_2M	1075	717	0.0925926	4	Singlet	Immune_cells
SAT_2M	1645	1027	3.688029	1	Singlet	Immune_cells
SAT_2M	2188	1315	2.3266423	1	Singlet	Immune_cells
SAT_2M	2047	1132	4.0389294	1	Singlet	Immune_cells
SAT_2M	1196	784	1.8318068	1	Singlet	Immune_cells
SAT_2M	2164	1216	0.2762431	0	Singlet	Adipocyte
SAT_2M	5006	2373	8.4130782	9	Singlet	Mesothelial
SAT_2M	1171	789	0.5937235	4	Singlet	Immune_cells
SAT_2M	1343	875	1.3323464	1	Singlet	Immune_cells
SAT_2M	3529	1884	0.9047215	3	Singlet	FAPs
SAT_2M	1162	794	5.097706	1	Singlet	Immune_cells
SAT_2M	2246	1257	0.2220249	3	Singlet	FAPs
SAT_2M	1092	767	0.3629764	1	Singlet	Immune_cells
SAT_2M	2444	1363	0.5291005	0	Singlet	Adipocyte
SAT_2M	1633	980	1.8270402	0	Singlet	Adipocyte
SAT_2M	1464	969	1.2951602	3	Singlet	FAPs
SAT_2M	1258	837	3.083004	1	Singlet	Immune_cells
SAT_2M	2715	1493	12.298091	1	Singlet	Immune_cells
SAT_2M	2031	1222	5.3071253	4	Singlet	Immune_cells
SAT_2M	1869	1093	1.2253596	0	Singlet	Adipocyte
SAT_2M	2421	1412	0.3292181	8	Singlet	Mesothelial
SAT_2M	1992	1240	1.6024036	1	Singlet	Immune_cells
SAT_2M	1038	748	0.094162	6	Singlet	Neurons
SAT_2M	1354	874	4.797048	8	Singlet	Mesothelial
SAT_2M	1262	801	0.4724409	0	Singlet	Adipocyte
SAT_2M	1421	885	1.396648	0	Singlet	Adipocyte
SAT_2M	1429	885	0.069735	0	Singlet	Adipocyte
SAT_2M	1367	913	2.5566107	1	Singlet	Immune_cells
SAT_2M	1071	693	0.8379888	1	Singlet	Immune_cells

SAT_2M	1992	1105	0.2497503	0	Singlet	Adipocyte
SAT_2M	1450	869	1.3764625	1	Singlet	Immune_cells
SAT_2M	4835	2186	0.267986	2	Singlet	FAPs
SAT_2M	1353	856	2.8085735	1	Singlet	Immune_cells
SAT_2M	1758	1064	6.1398522	1	Singlet	Immune_cells
SAT_2M	1692	1004	0.8833922	0	Singlet	Adipocyte
SAT_2M	1064	676	2.9990628	3	Singlet	FAPs
SAT_2M	2050	1048	1.9970774	5	Singlet	Immune_cells
SAT_2M	1687	1028	0.7083825	0	Singlet	Adipocyte
SAT_2M	1964	1081	5.2631579	10	Singlet	Immune_cells
SAT_2M	1142	777	4.0905135	5	Singlet	Immune_cells
SAT_2M	1319	809	12.773998	7	Singlet	Endothelial_cells
SAT_2M	1255	895	3.1570639	4	Singlet	Immune_cells
SAT_2M	2097	1254	1.52019	4	Singlet	Immune_cells
SAT_2M	4246	1813	1.6	10	Singlet	Immune_cells
SAT_2M	1141	784	4.1083916	11	Singlet	Smooth_muscle_cells
SAT_2M	1261	832	0.9478673	1	Singlet	Immune_cells
SAT_2M	1105	763	0.5415162	1	Singlet	Immune_cells
SAT_2M	2101	1322	5.7156353	1	Singlet	Immune_cells
SAT_2M	1370	849	6.095791	1	Singlet	Immune_cells
SAT_2M	1023	688	4.2759961	1	Singlet	Immune_cells
SAT_2M	1118	759	0.5347594	1	Singlet	Immune_cells
SAT_2M	1821	1094	4.8061169	1	Singlet	Immune_cells
SAT_2M	1071	758	3.7104072	6	Singlet	Neurons
SAT_2M	1068	670	0.4681648	0	Singlet	Adipocyte
SAT_2M	1306	849	1.6030534	1	Singlet	Immune_cells
SAT_2M	1292	812	0.4640371	4	Singlet	Immune_cells
SAT_2M	1331	852	0.5223881	0	Singlet	Adipocyte
SAT_2M	1365	914	0.2177068	3	Singlet	FAPs
SAT_2M	1525	988	0.1959504	1	Singlet	Immune_cells
SAT_2M	1992	1211	0.0501253	4	Singlet	Immune_cells
SAT_2M	4116	1715	2.3188406	10	Singlet	Immune_cells
SAT_2M	1152	765	7.5862069	1	Singlet	Immune_cells
SAT_2M	1468	950	2.3696682	1	Singlet	Immune_cells
SAT_2M	1271	824	0.6284368	1	Singlet	Immune_cells
SAT_2M	1498	941	8.8489687	4	Singlet	Immune_cells
SAT_2M	1468	986	2.300406	4	Singlet	Immune_cells
SAT_2M	1368	944	1.0122921	5	Singlet	Immune_cells
SAT_2M	1565	922	5.393401	1	Singlet	Immune_cells
SAT_2M	3627	1841	2.0363236	0	Singlet	Adipocyte
SAT_2M	1257	796	4.6862589	4	Singlet	Immune_cells
SAT_2M	2823	1507	0.4232804	2	Singlet	FAPs
SAT_2M	1166	764	4.2662116	1	Singlet	Immune_cells
SAT_2M	1923	1163	0.7261411	1	Singlet	Immune_cells
SAT_2M	1582	986	0.2523659	1	Singlet	Immune_cells
SAT_2M	1019	594	0.9765625	10	Singlet	Immune_cells
SAT_2M	6379	3039	10.338346	9	Singlet	Mesothelial
SAT_2M	1417	875	0.4926108	1	Singlet	Immune_cells
SAT_2M	2734	1423	2.374863	0	Singlet	Adipocyte
SAT_2M	1644	1084	3.3918837	4	Singlet	Immune_cells
SAT_2M	2887	1536	1.8301105	10	Singlet	Immune_cells

SAT_2M	1346	881	4.818384	4	Singlet	Immune_cells
SAT_2M	1985	1219	1.0531595	5	Singlet	Immune_cells
SAT_2M	1490	973	0.4	2	Singlet	FAPs
SAT_2M	1297	921	0.3837299	11	Singlet	Smooth_muscle_cells
SAT_2M	2761	1407	5.4972875	5	Singlet	Immune_cells
SAT_2M	1260	843	0.555115	1	Singlet	Immune_cells
SAT_2M	2903	1691	0.4459691	2	Singlet	FAPs
SAT_2M	1271	857	1.8010963	1	Singlet	Immune_cells
SAT_2M	2100	1362	0.1886792	5	Singlet	Immune_cells
SAT_2M	1360	860	1.0241405	1	Singlet	Immune_cells
SAT_2M	1310	817	5.6990881	1	Singlet	Immune_cells
SAT_2M	1069	712	0.9337068	0	Singlet	Adipocyte
SAT_2M	3099	1610	0.4501608	8	Singlet	Mesothelial
SAT_2M	1064	731	1.8691589	1	Singlet	Immune_cells
SAT_2M	1348	807	10.265879	1	Singlet	Immune_cells
SAT_2M	2083	1225	0.2868069	0	Singlet	Adipocyte
SAT_2M	1442	951	0.621118	4	Singlet	Immune_cells
SAT_2M	2169	1241	7.1886447	5	Singlet	Immune_cells
SAT_2M	1193	756	3.153527	9	Singlet	Mesothelial
SAT_2M	1416	897	0.5621926	1	Singlet	Immune_cells
SAT_2M	1570	1002	0.3162555	3	Singlet	FAPs
SAT_2M	1257	852	1.340694	11	Singlet	Smooth_muscle_cells
SAT_2M	1059	675	0.7476636	1	Singlet	Immune_cells
SAT_2M	1429	952	0.6289308	3	Singlet	FAPs
SAT_2M	1280	810	2.8838659	1	Singlet	Immune_cells
SAT_2M	2576	1484	2.473908	5	Singlet	Immune_cells
SAT_2M	2394	1333	2.9142381	0	Singlet	Adipocyte
SAT_2M	1903	1115	0.5238345	0	Singlet	Adipocyte
SAT_2M	1346	870	3.1875463	1	Singlet	Immune_cells
SAT_2M	1196	740	2.3313905	5	Singlet	Immune_cells
SAT_2M	1294	803	4.6332046	1	Singlet	Immune_cells
SAT_2M	1309	899	3.5794183	1	Singlet	Immune_cells
SAT_2M	1097	711	0.2717391	8	Singlet	Mesothelial
SAT_2M	2693	1426	3.2198372	0	Singlet	Adipocyte
SAT_2M	3447	1872	8.6491177	4	Singlet	Immune_cells
SAT_2M	1404	832	2.9871977	1	Singlet	Immune_cells
SAT_2M	1253	846	4.5346062	1	Singlet	Immune_cells
SAT_2M	1824	1228	0.2726281	4	Singlet	Immune_cells
SAT_2M	1176	772	0	5	Singlet	Immune_cells
SAT_2M	3753	1934	3.826734	8	Singlet	Mesothelial
SAT_2M	1620	974	0.4907975	0	Singlet	Adipocyte
SAT_2M	1060	712	0.3766478	4	Singlet	Immune_cells
SAT_2M	3754	1787	3.4775684	8	Singlet	Mesothelial
SAT_2M	1602	1072	1.3027295	11	Singlet	Smooth_muscle_cells
SAT_2M	1084	709	3.6900369	7	Singlet	Endothelial_cells
SAT_2M	4217	2127	3.6363636	9	Singlet	Mesothelial
SAT_2M	1672	979	5.6052475	1	Singlet	Immune_cells
SAT_2M	1656	1060	3.5371703	4	Singlet	Immune_cells
SAT_2M	1204	810	0.4950495	1	Singlet	Immune_cells
SAT_2M	3478	1784	0.2583238	8	Singlet	Mesothelial
SAT_2M	1827	1079	4.5602606	0	Singlet	Adipocyte

SAT_2M	1079	709	0.7373272	4	Singlet	Immune_cells
SAT_2M	1377	862	0.7896626	0	Singlet	Adipocyte
SAT_2M	1415	833	7.2382291	1	Singlet	Immune_cells
SAT_2M	1305	839	0.0763359	5	Singlet	Immune_cells
SAT_2M	1503	920	1.259947	3	Singlet	FAPs
SAT_2M	1529	972	1.8892508	3	Singlet	FAPs
SAT_2M	1240	843	1.2779553	4	Singlet	Immune_cells
SAT_2M	1238	789	1.369863	1	Singlet	Immune_cells
SAT_2M	1380	837	9.221902	1	Singlet	Immune_cells
SAT_2M	1832	1031	1.7905589	10	Singlet	Immune_cells
SAT_2M	1014	672	0.1968504	3	Singlet	FAPs
SAT_2M	1690	1123	0.4129794	11	Singlet	Smooth_muscle_cells
SAT_2M	1360	832	4.9671293	1	Singlet	Immune_cells
SAT_2M	1247	810	2.0766773	1	Singlet	Immune_cells
SAT_2M	1022	678	2.3460411	1	Singlet	Immune_cells
SAT_2M	1940	1062	2.7277406	3	Singlet	FAPs
SAT_2M	1386	834	8.3870968	1	Singlet	Immune_cells
SAT_2M	1289	814	1.843318	1	Singlet	Immune_cells
SAT_2M	1323	828	6.511976	1	Singlet	Immune_cells
SAT_2M	1949	1210	0.3070624	5	Singlet	Immune_cells
SAT_2M	1397	932	0.1428571	3	Singlet	FAPs
SAT_2M	1289	863	4.5107034	4	Singlet	Immune_cells
SAT_2M	1427	929	2.5139665	1	Singlet	Immune_cells
SAT_2M	2047	1148	12.237933	4	Singlet	Immune_cells
SAT_2M	1893	1081	3.0095037	1	Singlet	Immune_cells
SAT_2M	1231	827	1.6155089	7	Singlet	Endothelial_cells
SAT_2M	1176	722	6.9373942	1	Singlet	Immune_cells
SAT_2M	1281	896	0.6196747	4	Singlet	Immune_cells
SAT_2M	1169	797	3.4100597	1	Singlet	Immune_cells
SAT_2M	3814	1863	0.1306506	0	Singlet	Adipocyte
SAT_2M	3126	1682	5.1036683	1	Singlet	Immune_cells
SAT_2M	3755	1512	5.7233704	10	Singlet	Immune_cells
SAT_2M	1387	831	0.6479482	4	Singlet	Immune_cells
SAT_2M	1450	975	1.0295127	9	Singlet	Mesothelial
SAT_2M	1599	1031	0.436409	5	Singlet	Immune_cells
SAT_2M	1225	769	0.648824	2	Singlet	FAPs
SAT_2M	1793	1082	0.4444444	4	Singlet	Immune_cells
SAT_2M	2022	1227	1.2357884	1	Singlet	Immune_cells
SAT_2M	3818	1907	0.1822917	8	Singlet	Mesothelial
SAT_2M	1049	615	6.6476733	5	Singlet	Immune_cells
SAT_2M	1171	777	0.0848896	1	Singlet	Immune_cells
SAT_2M	1253	773	5.1751592	1	Singlet	Immune_cells
SAT_2M	3354	1727	1.960202	0	Singlet	Adipocyte
SAT_2M	1699	1023	0.4692082	4	Singlet	Immune_cells
SAT_2M	1329	921	0.1497006	4	Singlet	Immune_cells
SAT_2M	1793	1060	1.1660189	0	Singlet	Adipocyte
SAT_2M	4367	1963	0.4105839	0	Singlet	Adipocyte
SAT_2M	1052	684	4.3273754	4	Singlet	Immune_cells
SAT_2M	1266	780	2.2798742	5	Singlet	Immune_cells
SAT_2M	1571	931	9.0793651	1	Singlet	Immune_cells
SAT_2M	1266	768	6.8396226	1	Singlet	Immune_cells

SAT_2M	1740	1090	2.0011435	0	Singlet	Adipocyte
SAT_2M	1716	944	6.0500291	5	Singlet	Immune_cells
SAT_2M	1291	805	6.3320463	1	Singlet	Immune_cells
SAT_2M	3187	1645	0.3436426	0	Singlet	Adipocyte
SAT_2M	1507	862	2.3178808	4	Singlet	Immune_cells
SAT_2M	2473	1354	0.0403388	0	Singlet	Adipocyte
SAT_2M	1742	1114	0.5154639	4	Singlet	Immune_cells
SAT_2M	1422	898	5.3997195	9	Singlet	Mesothelial
SAT_2M	2226	1303	3.3647376	5	Singlet	Immune_cells
SAT_2M	1743	917	3.0892449	5	Singlet	Immune_cells
SAT_2M	5034	2453	3.2322031	9	Singlet	Mesothelial
SAT_2M	2186	1229	1.7343679	0	Singlet	Adipocyte
SAT_2M	1260	830	0.0790514	3	Singlet	FAPs
SAT_2M	2345	1216	0.8085106	10	Singlet	Immune_cells
SAT_2M	2532	1376	7.8470032	1	Singlet	Immune_cells
SAT_2M	2014	1228	0.3463632	1	Singlet	Immune_cells
SAT_2M	1625	932	1.4760148	0	Singlet	Adipocyte
SAT_2M	1706	994	4.3911007	1	Singlet	Immune_cells
SAT_2M	1554	920	2.946829	1	Singlet	Immune_cells
SAT_2M	1297	802	7.3133179	4	Singlet	Immune_cells
SAT_2M	3497	1545	1.3417071	10	Singlet	Immune_cells
SAT_2M	2151	1193	0.4627487	0	Singlet	Adipocyte
SAT_2M	1974	1124	5.5050505	1	Singlet	Immune_cells
SAT_2M	1340	819	5.3452116	5	Singlet	Immune_cells
SAT_2M	2127	1235	3.8371549	1	Singlet	Immune_cells
SAT_2M	2012	1201	1.4814815	4	Singlet	Immune_cells
SAT_2M	2477	1352	8.3299799	1	Singlet	Immune_cells
SAT_2M	5737	2641	4.0826963	9	Singlet	Mesothelial
SAT_2M	2697	1451	0.7366483	0	Singlet	Adipocyte
SAT_2M	2293	1323	1.9113814	1	Singlet	Immune_cells
SAT_2M	1297	822	0.8455035	7	Singlet	Endothelial_cells
SAT_2M	4531	2111	0.3523453	2	Singlet	FAPs
SAT_2M	1404	876	3.1205674	5	Singlet	Immune_cells
SAT_2M	1385	846	5.4597701	1	Singlet	Immune_cells
SAT_2M	1130	726	2.7312775	1	Singlet	Immune_cells
SAT_2M	1477	1000	0.6044325	8	Singlet	Mesothelial
SAT_2M	1947	1068	5.3196931	1	Singlet	Immune_cells
SAT_2M	1219	759	8.7204564	4	Singlet	Immune_cells
SAT_2M	2095	1276	0.0947418	5	Singlet	Immune_cells
SAT_2M	1519	972	0.3942181	7	Singlet	Endothelial_cells
SAT_2M	2122	1295	2.1626704	4	Singlet	Immune_cells
SAT_2M	1407	904	0.8498584	4	Singlet	Immune_cells
SAT_2M	1558	978	0.255102	0	Singlet	Adipocyte
SAT_2M	1547	995	1.414791	0	Singlet	Adipocyte
SAT_2M	1367	926	0.0727273	5	Singlet	Immune_cells
SAT_2M	1092	729	1.7319964	1	Singlet	Immune_cells
SAT_2M	2795	1502	1.1035956	0	Singlet	Adipocyte
SAT_2M	2571	1419	0.1159644	0	Singlet	Adipocyte
SAT_2M	2580	1415	2.4361949	9	Singlet	Mesothelial
SAT_2M	1212	800	0.247117	4	Singlet	Immune_cells
SAT_2M	1276	806	0.3897116	0	Singlet	Adipocyte

SAT_2M	1198	773	0.4979253	3	Singlet	FAPs
SAT_2M	1630	1039	0.670323	1	Singlet	Immune_cells
SAT_2M	1184	753	0.5892256	1	Singlet	Immune_cells
SAT_2M	2263	1258	1.5432099	5	Singlet	Immune_cells
SAT_2M	1760	884	0.3335186	6	Singlet	Neurons
SAT_2M	1247	812	3.8338658	1	Singlet	Immune_cells
SAT_2M	1274	807	3.6050157	1	Singlet	Immune_cells
SAT_2M	2973	1526	0.2010724	0	Singlet	Adipocyte
SAT_2M	1466	907	0.1352265	1	Singlet	Immune_cells
SAT_2M	2129	1214	5.6153486	4	Singlet	Immune_cells
SAT_2M	1181	805	1.5088013	3	Singlet	FAPs
SAT_2M	1281	757	1.5576324	0	Singlet	Adipocyte
SAT_2M	1238	792	1.1281225	4	Singlet	Immune_cells
SAT_2M	1857	1100	0.7498661	3	Singlet	FAPs
SAT_2M	1489	902	3.0120482	1	Singlet	Immune_cells
SAT_2M	1030	700	1.8322083	1	Singlet	Immune_cells
SAT_2M	1788	1105	0.4997224	0	Singlet	Adipocyte
SAT_2M	1530	1006	4.4299674	1	Singlet	Immune_cells
SAT_2M	1286	849	0.3861004	4	Singlet	Immune_cells
SAT_2M	1066	637	1.5917603	10	Singlet	Immune_cells
SAT_2M	1449	916	6.6620879	1	Singlet	Immune_cells
SAT_2M	1820	1161	0.4378763	8	Singlet	Mesothelial
SAT_2M	1344	831	7.9646018	4	Singlet	Immune_cells
SAT_2M	1502	875	4.9071618	1	Singlet	Immune_cells
SAT_2M	1653	1028	0.1201923	1	Singlet	Immune_cells
SAT_2M	1191	805	1.6736402	1	Singlet	Immune_cells
SAT_2M	1801	1065	0.7174393	0	Singlet	Adipocyte
SAT_2M	1501	899	9.6560847	1	Singlet	Immune_cells
SAT_2M	1535	1003	1.0322581	4	Singlet	Immune_cells
SAT_2M	1812	1085	5.7142857	1	Singlet	Immune_cells
SAT_2M	1335	878	0.0745156	8	Singlet	Mesothelial
SAT_2M	9360	3062	0.6932594	10	Singlet	Immune_cells
SAT_2M	1995	1089	2.1489255	1	Singlet	Immune_cells
SAT_2M	4251	2154	10.372278	4	Singlet	Immune_cells
SAT_2M	1443	890	0.6219765	4	Singlet	Immune_cells
SAT_2M	3507	1662	0.6260672	0	Singlet	Adipocyte
SAT_2M	2546	1160	4.7824383	10	Singlet	Immune_cells
SAT_2M	1094	690	0.1814882	4	Singlet	Immune_cells
SAT_2M	2656	1538	4.5454545	8	Singlet	Mesothelial
SAT_2M	1680	1038	1.4218009	0	Singlet	Adipocyte
SAT_2M	2404	1420	1.5295577	3	Singlet	FAPs
SAT_2M	1415	836	6.135402	1	Singlet	Immune_cells
SAT_2M	1300	813	2.6656512	0	Singlet	Adipocyte
SAT_2M	2433	1297	7.6260763	9	Singlet	Mesothelial
SAT_2M	1189	737	1.1725293	3	Singlet	FAPs
SAT_2M	2144	1244	4.8837209	4	Singlet	Immune_cells
SAT_2M	1693	988	0.4708652	0	Singlet	Adipocyte
SAT_2M	1196	811	0.6568145	8	Singlet	Mesothelial
SAT_2M	1367	872	4.0116703	1	Singlet	Immune_cells
SAT_2M	2318	1297	0.4282655	0	Singlet	Adipocyte
SAT_2M	1367	809	7.0751276	1	Singlet	Immune_cells

SAT_2M	1480	978	0.6734007	1	Singlet	Immune_cells
SAT_2M	2106	1158	1.7510648	10	Singlet	Immune_cells
SAT_2M	2010	1158	1.5888779	5	Singlet	Immune_cells
SAT_2M	1731	1007	0.4608295	0	Singlet	Adipocyte
SAT_2M	1635	953	6.2843197	1	Singlet	Immune_cells
SAT_2M	2172	1288	1.7931034	5	Singlet	Immune_cells
SAT_2M	1130	765	0.7042254	1	Singlet	Immune_cells
SAT_2M	1950	1101	1.6871166	4	Singlet	Immune_cells
SAT_2M	1564	880	5.2329292	9	Singlet	Mesothelial
SAT_2M	2312	1187	4.9180328	5	Singlet	Immune_cells
SAT_2M	1133	697	0.1755926	7	Singlet	Endothelial_cells
SAT_2M	7423	2934	5.3140097	9	Singlet	Mesothelial
SAT_2M	1363	856	2.0437956	1	Singlet	Immune_cells
SAT_2M	1188	755	0.5042017	0	Singlet	Adipocyte
SAT_2M	3070	1714	0.4209845	8	Singlet	Mesothelial
SAT_2M	1329	869	1.0502626	4	Singlet	Immune_cells
SAT_2M	2103	1196	6.3920455	1	Singlet	Immune_cells
SAT_2M	1685	1094	0.2955083	11	Singlet	Smooth_muscle_cells
SAT_2M	1619	985	0.0610128	8	Singlet	Mesothelial
SAT_2M	1924	1224	0.9849663	4	Singlet	Immune_cells
SAT_2M	1709	1099	2.5043681	5	Singlet	Immune_cells
SAT_2M	1224	800	2.2727273	4	Singlet	Immune_cells
SAT_2M	3010	1584	0.5632869	0	Singlet	Adipocyte
SAT_2M	1388	881	0.2867384	3	Singlet	FAPs
SAT_2M	3866	1974	7.1613237	9	Singlet	Mesothelial
SAT_2M	1301	779	0.8396947	10	Singlet	Immune_cells
SAT_2M	1073	757	0.4651163	8	Singlet	Mesothelial
SAT_2M	1262	812	3.7095501	1	Singlet	Immune_cells
SAT_2M	1469	895	0.135318	4	Singlet	Immune_cells
SAT_2M	1262	866	0.7069914	4	Singlet	Immune_cells
SAT_2M	1706	1021	3.9250146	4	Singlet	Immune_cells
SAT_2M	1256	821	2.1428571	4	Singlet	Immune_cells
SAT_2M	1830	1028	3.4858388	10	Singlet	Immune_cells
SAT_2M	1293	838	0.1540832	4	Singlet	Immune_cells
SAT_2M	1080	653	0.6445672	6	Singlet	Neurons
SAT_2M	1515	972	1.0437052	4	Singlet	Immune_cells
SAT_2M	4184	2100	7.8281623	9	Singlet	Mesothelial
SAT_2M	4276	2025	0.2561714	2	Singlet	FAPs
SAT_2M	1759	1062	0.2828054	3	Singlet	FAPs
SAT_2M	1484	921	0.6044325	1	Singlet	Immune_cells
SAT_2M	1315	826	3.9453718	4	Singlet	Immune_cells
SAT_2M	1842	1126	0.2166847	0	Singlet	Adipocyte
SAT_2M	1007	689	1.5857284	5	Singlet	Immune_cells
SAT_2M	1607	1032	0.0620347	3	Singlet	FAPs
SAT_2M	1251	755	8.2071713	1	Singlet	Immune_cells
SAT_2M	2382	1378	0.6260434	5	Singlet	Immune_cells
SAT_2M	1235	806	1.7741935	1	Singlet	Immune_cells
SAT_2M	1073	736	0.3710575	4	Singlet	Immune_cells
SAT_2M	1069	737	0.5591799	4	Singlet	Immune_cells
SAT_2M	1285	802	5.1044084	1	Singlet	Immune_cells
SAT_2M	1856	1071	3.707684	1	Singlet	Immune_cells

SAT_2M	2538	1506	0.3532182	5	Singlet	Immune_cells
SAT_2M	1388	788	6.8149211	9	Singlet	Mesothelial
SAT_2M	2422	1310	0.2886598	0	Singlet	Adipocyte
SAT_2M	1051	651	1.9830028	5	Singlet	Immune_cells
SAT_2M	1164	774	0.1715266	5	Singlet	Immune_cells
SAT_2M	2065	1151	9.4202899	1	Singlet	Immune_cells
SAT_2M	1595	1018	1.2476606	1	Singlet	Immune_cells
SAT_2M	1405	875	5.2631579	1	Singlet	Immune_cells
SAT_2M	1487	928	3.9597315	1	Singlet	Immune_cells
SAT_2M	2278	1356	0.7870573	0	Singlet	Adipocyte
SAT_2M	1654	928	3.0722892	10	Singlet	Immune_cells
SAT_2M	1321	835	0.6772009	7	Singlet	Endothelial_cells
SAT_2M	1710	1059	3.3858727	1	Singlet	Immune_cells
SAT_2M	1234	756	5.6542811	1	Singlet	Immune_cells
SAT_2M	2668	1526	0.2985075	9	Singlet	Mesothelial
SAT_2M	1413	856	3.810868	8	Singlet	Mesothelial
SAT_2M	1402	937	0.0712251	4	Singlet	Immune_cells
SAT_2M	1673	1003	0.0594177	1	Singlet	Immune_cells
SAT_2M	1132	739	0.1762115	1	Singlet	Immune_cells
SAT_2M	8387	2892	2.9642857	10	Singlet	Immune_cells
SAT_2M	1361	841	4.1025641	1	Singlet	Immune_cells
SAT_2M	1109	742	0.4492363	5	Singlet	Immune_cells
SAT_2M	3089	1620	2.7490298	8	Singlet	Mesothelial
SAT_2M	1521	940	6.0327869	1	Singlet	Immune_cells
SAT_2M	2199	1333	2.7589326	0	Singlet	Adipocyte
SAT_2M	1291	790	7.0216049	1	Singlet	Immune_cells
SAT_2M	1094	773	0.6369427	4	Singlet	Immune_cells
SAT_2M	1311	785	1.2898331	4	Singlet	Immune_cells
SAT_2M	2626	1536	5.1711027	9	Singlet	Mesothelial
SAT_2M	1938	1141	0.462963	0	Singlet	Adipocyte
SAT_2M	1576	1016	0.4413619	11	Singlet	Smooth_muscle_cells
SAT_2M	1452	843	2.2633745	10	Singlet	Immune_cells
SAT_2M	1077	675	0.2770083	0	Singlet	Adipocyte
SAT_2M	1499	1015	0.0665336	4	Singlet	Immune_cells
SAT_2M	1416	937	0.7042254	4	Singlet	Immune_cells
SAT_2M	1439	943	4.2273042	3	Singlet	FAPs
SAT_2M	1072	693	0.2793296	4	Singlet	Immune_cells
SAT_2M	2136	1286	2.6523965	1	Singlet	Immune_cells
SAT_2M	2952	1710	0.6761325	11	Singlet	Smooth_muscle_cells
SAT_2M	1038	675	0.3846154	4	Singlet	Immune_cells
SAT_2M	1244	862	0	8	Singlet	Mesothelial
SAT_2M	2202	1290	0.4984141	4	Singlet	Immune_cells
SAT_2M	1070	749	0.6523765	9	Singlet	Mesothelial
SAT_2M	2042	1164	12.115291	1	Singlet	Immune_cells
SAT_2M	1043	710	2.2878932	1	Singlet	Immune_cells
SAT_2M	1685	1065	0.4719764	5	Singlet	Immune_cells
SAT_2M	1042	751	0.1908397	4	Singlet	Immune_cells
SAT_2M	2017	1221	3.7567968	5	Singlet	Immune_cells
SAT_2M	2573	1491	3.4522886	9	Singlet	Mesothelial
SAT_2M	1988	1176	0.6490265	0	Singlet	Adipocyte
SAT_2M	1178	816	0.0845309	5	Singlet	Immune_cells

SAT_2M	1212	728	8.3127572	0	Singlet	Adipocyte
SAT_2M	1253	773	1.2678288	1	Singlet	Immune_cells
SAT_2M	1371	893	0.2904866	3	Singlet	FAPs
SAT_2M	1245	748	10.09539	1	Singlet	Immune_cells
SAT_2M	2599	1398	1.1119632	0	Singlet	Adipocyte
SAT_2M	1488	829	12.173913	0	Singlet	Adipocyte
SAT_2M	1261	782	8.458498	1	Singlet	Immune_cells
SAT_2M	2753	1580	0.9067827	11	Singlet	Smooth_muscle_cells
SAT_2M	1442	894	0.1380262	5	Singlet	Immune_cells
SAT_2M	1811	1068	0.0547345	0	Singlet	Adipocyte
SAT_2M	1779	1108	0.8389262	1	Singlet	Immune_cells
SAT_2M	2161	1252	2.2038567	1	Singlet	Immune_cells
SAT_2M	2143	1212	4.0968343	1	Singlet	Immune_cells
SAT_2M	1453	889	1.303155	7	Singlet	Endothelial_cells
SAT_2M	3819	2011	0.1043841	8	Singlet	Mesothelial
SAT_2M	1277	845	2.4199844	1	Singlet	Immune_cells
SAT_2M	5868	2730	3.6078965	9	Singlet	Mesothelial
SAT_2M	1387	922	1.3639627	11	Singlet	Smooth_muscle_cells
SAT_2M	1293	817	0.385505	1	Singlet	Immune_cells
SAT_2M	1434	804	8.5476025	5	Singlet	Immune_cells
SAT_2M	1610	1016	2.3529412	4	Singlet	Immune_cells
SAT_2M	1390	865	0.215208	4	Singlet	Immune_cells
SAT_2M	1298	772	9.7542243	1	Singlet	Immune_cells
SAT_2M	1921	1179	6.7392431	5	Singlet	Immune_cells
SAT_2M	1255	790	1.8181818	1	Singlet	Immune_cells
SAT_2M	1558	999	1.5364917	1	Singlet	Immune_cells
SAT_2M	1315	783	3.4848485	4	Singlet	Immune_cells
SAT_2M	3889	1911	8.7134803	3	Singlet	FAPs
SAT_2M	2227	1224	1.2053571	0	Singlet	Adipocyte
SAT_2M	1701	1072	2.9342723	4	Singlet	Immune_cells
SAT_2M	3210	1604	5.8457711	4	Singlet	Immune_cells
SAT_2M	1288	805	4.3276662	1	Singlet	Immune_cells
SAT_2M	1428	915	0.4895105	1	Singlet	Immune_cells
SAT_2M	1510	969	0.3960396	4	Singlet	Immune_cells
SAT_2M	1181	738	1.0970464	5	Singlet	Immune_cells
SAT_2M	2384	1464	2.5930573	5	Singlet	Immune_cells
SAT_2M	1240	789	4.995971	4	Singlet	Immune_cells
SAT_2M	2039	1178	0.5879471	4	Singlet	Immune_cells
SAT_2M	1776	1100	0.2247191	3	Singlet	FAPs
SAT_2M	2080	1234	3.8332535	4	Singlet	Immune_cells
SAT_2M	1714	1042	0.462963	0	Singlet	Adipocyte
SAT_2M	1343	802	6.547619	9	Singlet	Mesothelial
SAT_2M	1221	781	6.3621533	4	Singlet	Immune_cells
SAT_2M	1214	754	0	0	Singlet	Adipocyte
SAT_2M	3657	1800	0.2452316	0	Singlet	Adipocyte
SAT_2M	1419	840	2.5174825	0	Singlet	Adipocyte
SAT_2M	2852	1750	0.2782609	4	Singlet	Immune_cells
SAT_2M	6043	2842	2.5586002	9	Singlet	Mesothelial
SAT_2M	1961	1246	0.305499	1	Singlet	Immune_cells
SAT_2M	1323	810	0.0751315	0	Singlet	Adipocyte
SAT_2M	1813	1136	0.2751789	5	Singlet	Immune_cells

SAT_2M	1463	855	2.9231815	0	Singlet	Adipocyte
SAT_2M	1088	746	1.1872146	4	Singlet	Immune_cells
SAT_2M	1403	882	0.7102273	4	Singlet	Immune_cells
SAT_2M	2526	1402	2.8853755	1	Singlet	Immune_cells
SAT_2M	1483	894	1.6823688	8	Singlet	Mesothelial
SAT_2M	2231	1227	0.2231147	3	Singlet	FAPs
SAT_2M	3115	1470	0.9309791	3	Singlet	FAPs
SAT_2M	1049	695	1.7061611	4	Singlet	Immune_cells
SAT_2M	5458	2290	1.7547066	10	Singlet	Immune_cells
SAT_2M	1232	788	1.2067578	0	Singlet	Adipocyte
SAT_2M	1086	700	2.5617566	4	Singlet	Immune_cells
SAT_2M	1183	763	1.6020236	8	Singlet	Mesothelial
SAT_2M	1304	795	3.1369549	0	Singlet	Adipocyte
SAT_2M	1971	1123	1.8218623	1	Singlet	Immune_cells
SAT_2M	2240	1273	0.2228164	1	Singlet	Immune_cells
SAT_2M	6628	2767	4.1227806	9	Singlet	Mesothelial
SAT_2M	1242	726	4.3338684	1	Singlet	Immune_cells
SAT_2M	2828	1485	0.0704225	0	Singlet	Adipocyte
SAT_2M	1730	973	0.9792627	3	Singlet	FAPs
SAT_2M	1421	907	0.4189944	0	Singlet	Adipocyte
SAT_2M	1567	985	1.9047619	0	Singlet	Adipocyte
SAT_2M	1572	987	0.0635728	4	Singlet	Immune_cells
SAT_2M	1200	771	1.1599006	4	Singlet	Immune_cells
SAT_2M	1377	909	3.9158811	1	Singlet	Immune_cells
SAT_2M	1298	849	0.6891271	4	Singlet	Immune_cells
SAT_2M	1262	826	0.7120253	11	Singlet	Smooth_muscle_cells
SAT_2M	2078	1325	0.6190476	4	Singlet	Immune_cells
SAT_2M	1807	1037	6.4088398	1	Singlet	Immune_cells
SAT_2M	1069	771	0.7187781	6	Singlet	Neurons
SAT_2M	1509	968	0.5952381	4	Singlet	Immune_cells
SAT_2M	1516	919	0.3929273	4	Singlet	Immune_cells
SAT_2M	1884	1182	0.3176284	1	Singlet	Immune_cells
SAT_2M	2387	1375	4.317144	5	Singlet	Immune_cells
SAT_2M	1396	849	0.8571429	0	Singlet	Adipocyte
SAT_2M	1014	645	6.6666667	9	Singlet	Mesothelial
SAT_2M	2404	1404	0.6614303	0	Singlet	Adipocyte
SAT_2M	1082	738	0.5469462	1	Singlet	Immune_cells
SAT_2M	1249	834	2.4720893	1	Singlet	Immune_cells
SAT_2M	1185	750	2.9411765	3	Singlet	FAPs
SAT_2M	1149	768	0.0868056	4	Singlet	Immune_cells
SAT_2M	1488	905	3.1522468	1	Singlet	Immune_cells
SAT_2M	3566	1972	7.877095	9	Singlet	Mesothelial
SAT_2M	1127	756	1.4159292	1	Singlet	Immune_cells
SAT_2M	2184	1262	2.1917808	4	Singlet	Immune_cells
SAT_2M	1813	1080	0.7709251	1	Singlet	Immune_cells
SAT_2M	2939	1532	1.4280857	10	Singlet	Immune_cells
SAT_2M	2542	1260	2.1594032	10	Singlet	Immune_cells
SAT_2M	1264	798	0.3152088	4	Singlet	Immune_cells
SAT_2M	1343	846	0.5956813	4	Singlet	Immune_cells
SAT_2M	1199	813	0.8257638	3	Singlet	FAPs
SAT_2M	2051	1187	6.3346228	5	Singlet	Immune_cells

SAT_2M	3077	1666	0	0	Singlet	Adipocyte
SAT_2M	1336	896	2.9147982	1	Singlet	Immune_cells
SAT_2M	1581	1049	0.7528231	6	Singlet	Neurons
SAT_2M	1083	710	2.5664528	1	Singlet	Immune_cells
SAT_2M	1443	865	7.3153899	1	Singlet	Immune_cells
SAT_2M	2033	1240	2.5477707	4	Singlet	Immune_cells
SAT_2M	1273	812	6.0344828	1	Singlet	Immune_cells
SAT_2M	1559	935	5.1592357	1	Singlet	Immune_cells
SAT_2M	8646	3605	9.5463465	9	Singlet	Mesothelial
SAT_2M	1847	1167	0.7567568	1	Singlet	Immune_cells
SAT_2M	1304	845	0.608365	1	Singlet	Immune_cells
SAT_2M	1349	845	0.5173688	4	Singlet	Immune_cells
SAT_2M	1739	1019	1.0303377	0	Singlet	Adipocyte
SAT_2M	1374	925	2.5974026	4	Singlet	Immune_cells
SAT_2M	1249	794	0.2394254	4	Singlet	Immune_cells
SAT_2M	1207	745	0.2477291	4	Singlet	Immune_cells
SAT_2M	1302	852	3.7519142	6	Singlet	Neurons
SAT_2M	1207	769	7.5409836	4	Singlet	Immune_cells
SAT_2M	1405	902	3.7588652	1	Singlet	Immune_cells
SAT_2M	5048	2461	1.6422636	9	Singlet	Mesothelial
SAT_2M	3434	1603	2.6422764	10	Singlet	Immune_cells
SAT_2M	1455	944	6.7715458	4	Singlet	Immune_cells
SAT_2M	1489	952	5.941255	1	Singlet	Immune_cells
SAT_2M	3851	1872	1.7357513	10	Singlet	Immune_cells
SAT_2M	1278	819	6.1032864	5	Singlet	Immune_cells
SAT_2M	1626	983	9.9325567	4	Singlet	Immune_cells
SAT_2M	1370	949	0.1458789	8	Singlet	Mesothelial
SAT_2M	3191	1636	0.2502346	0	Singlet	Adipocyte
SAT_2M	1557	991	0.3196931	4	Singlet	Immune_cells
SAT_2M	2396	1333	6.6888243	1	Singlet	Immune_cells
SAT_2M	1500	995	1.6600266	1	Singlet	Immune_cells
SAT_2M	1007	679	0.4940711	0	Singlet	Adipocyte
SAT_2M	1276	810	5.4644809	1	Singlet	Immune_cells
SAT_2M	1103	735	6.8778281	1	Singlet	Immune_cells
SAT_2M	1136	751	0.6134969	1	Singlet	Immune_cells
SAT_2M	1023	668	1.5579357	0	Singlet	Adipocyte
SAT_2M	8604	3435	8.3739272	9	Singlet	Mesothelial
SAT_2M	1583	989	1.3240858	0	Singlet	Adipocyte
SAT_2M	1494	975	0.4676019	5	Singlet	Immune_cells
SAT_2M	1209	797	1.4026403	4	Singlet	Immune_cells
SAT_2M	1709	1027	5.6428156	1	Singlet	Immune_cells
SAT_2M	3736	1886	0.8006405	11	Singlet	Smooth_muscle_cells
SAT_2M	1611	1039	0.5552128	5	Singlet	Immune_cells
SAT_2M	1612	1087	1.0995724	6	Singlet	Neurons
SAT_2M	1346	850	4.8255382	1	Singlet	Immune_cells
SAT_2M	1745	1065	0.5704507	0	Singlet	Adipocyte
SAT_2M	2443	1323	3.1096563	8	Singlet	Mesothelial
SAT_2M	1522	877	3.8688525	3	Singlet	FAPs
SAT_2M	1069	737	2.1455224	4	Singlet	Immune_cells
SAT_2M	1234	767	4.1062802	1	Singlet	Immune_cells
SAT_2M	7310	3145	4.9043716	9	Singlet	Mesothelial

SAT_2M	1262	815	1.9762846	10	Singlet	Immune_cells
SAT_2M	2636	1455	0.3775009	8	Singlet	Mesothelial
SAT_2M	4364	1980	0.4108651	0	Singlet	Adipocyte
SAT_2M	1057	719	0.8482564	4	Singlet	Immune_cells
SAT_2M	2691	1535	3.7352071	1	Singlet	Immune_cells
SAT_2M	1393	942	2.4113475	1	Singlet	Immune_cells
SAT_2M	1426	945	1.048218	11	Singlet	Smooth_muscle_cells
SAT_2M	1111	629	0.4492363	0	Singlet	Adipocyte
SAT_2M	3210	1634	0.1550388	0	Singlet	Adipocyte
SAT_2M	1785	1108	0.8928571	0	Singlet	Adipocyte
SAT_2M	1447	863	0.4814305	0	Singlet	Adipocyte
SAT_2M	1325	820	0.9730539	4	Singlet	Immune_cells
SAT_2M	1508	939	8.1740277	1	Singlet	Immune_cells
SAT_2M	3319	1890	1.7122259	8	Singlet	Mesothelial
SAT_2M	1602	1030	0.4361371	4	Singlet	Immune_cells
SAT_2M	1261	822	6.71406	1	Singlet	Immune_cells
SAT_2M	1531	946	2.7308192	1	Singlet	Immune_cells
SAT_2M	1666	1037	1.0143198	6	Singlet	Neurons
SAT_2M	1649	1006	0.4221954	0	Singlet	Adipocyte
SAT_2M	1535	951	0.843608	0	Singlet	Adipocyte
SAT_2M	2688	1505	3.9157739	1	Singlet	Immune_cells
SAT_2M	1016	658	4.0354331	0	Singlet	Adipocyte
SAT_2M	1572	982	2.7989822	4	Singlet	Immune_cells
SAT_2M	1005	632	0.4945598	0	Singlet	Adipocyte
SAT_2M	1629	1017	4.1514042	4	Singlet	Immune_cells
SAT_2M	1348	830	0.2956393	1	Singlet	Immune_cells
SAT_2M	1268	803	9.4043887	1	Singlet	Immune_cells
SAT_2M	1215	820	1.965602	4	Singlet	Immune_cells
SAT_2M	1272	782	6.2745098	1	Singlet	Immune_cells
SAT_2M	2536	1391	0.0785238	8	Singlet	Mesothelial
SAT_2M	1422	889	0.209937	1	Singlet	Immune_cells
SAT_2M	1299	814	9.984639	1	Singlet	Immune_cells
SAT_2M	1217	804	0.4088307	0	Singlet	Adipocyte
SAT_2M	1627	978	0.7955936	0	Singlet	Adipocyte
SAT_2M	1299	879	1.2987013	4	Singlet	Immune_cells
SAT_2M	1696	1037	0.7611241	1	Singlet	Immune_cells
SAT_2M	1355	869	1.025641	0	Singlet	Adipocyte
SAT_2M	1271	805	8.2877248	9	Singlet	Mesothelial
SAT_2M	2503	1332	0.7949126	0	Singlet	Adipocyte
SAT_2M	1382	834	5.1079137	1	Singlet	Immune_cells
SAT_2M	1848	1083	0.7555316	0	Singlet	Adipocyte
SAT_2M	1751	969	14.058054	1	Singlet	Immune_cells
SAT_2M	4302	2083	0.7866728	0	Singlet	Adipocyte
SAT_2M	4017	1852	2.5074479	3	Singlet	FAPs
SAT_2M	2012	1248	5.109127	8	Singlet	Mesothelial
SAT_2M	1104	797	0.7207207	4	Singlet	Immune_cells
SAT_2M	4227	1817	3.9433294	10	Singlet	Immune_cells
SAT_2M	1094	757	5.4545455	8	Singlet	Mesothelial
SAT_2M	1507	914	2.0475561	0	Singlet	Adipocyte
SAT_2M	2087	1164	4.2583732	5	Singlet	Immune_cells
SAT_2M	2214	1244	5.718145	3	Singlet	FAPs

SAT_2M	1217	772	8.1566069	1	Singlet	Immune_cells
SAT_2M	1146	768	0.4310345	2	Singlet	FAPs
SAT_2M	2286	1359	0.5228758	0	Singlet	Adipocyte
SAT_2M	3499	1825	1.5104018	0	Singlet	Adipocyte
SAT_2M	1020	689	1.7526777	1	Singlet	Immune_cells
SAT_2M	1004	635	4.7666336	3	Singlet	FAPs
SAT_2M	1312	805	7.5056861	1	Singlet	Immune_cells
SAT_2M	1196	802	0.910596	4	Singlet	Immune_cells
SAT_2M	1434	897	9.1412742	1	Singlet	Immune_cells
SAT_2M	1333	833	5.4640719	1	Singlet	Immune_cells
SAT_2M	2023	1060	8.9478859	0	Singlet	Adipocyte
SAT_2M	2094	1232	1.8562589	7	Singlet	Endothelial_cells
SAT_2M	2100	1266	1.657197	11	Singlet	Smooth_muscle_cells
SAT_2M	1218	808	2.4509804	1	Singlet	Immune_cells
SAT_2M	1030	709	0.3861004	5	Singlet	Immune_cells
SAT_2M	2041	1180	4.7828209	1	Singlet	Immune_cells
SAT_2M	1165	738	0.5128205	0	Singlet	Adipocyte
SAT_2M	2118	1240	4.2313117	5	Singlet	Immune_cells
SAT_2M	1399	918	0.4996431	1	Singlet	Immune_cells
SAT_2M	1113	702	0.3577818	0	Singlet	Adipocyte
SAT_2M	3679	1902	8.1936182	9	Singlet	Mesothelial
SAT_2M	1234	805	0.886382	1	Singlet	Immune_cells
SAT_2M	1723	1147	2.0677771	5	Singlet	Immune_cells
SAT_2M	2215	1236	0.8082622	0	Singlet	Adipocyte
SAT_2M	1135	770	3.2513181	4	Singlet	Immune_cells
SAT_2M	1118	762	0.2666667	11	Singlet	Smooth_muscle_cells
SAT_2M	1423	887	8.345021	1	Singlet	Immune_cells
SAT_2M	1263	808	0.3159558	0	Singlet	Adipocyte
SAT_2M	1559	1010	0.3184713	4	Singlet	Immune_cells
SAT_2M	1801	1047	0.7743363	0	Singlet	Adipocyte
SAT_2M	1071	686	0.8356546	1	Singlet	Immune_cells
SAT_2M	1773	1080	0.2812148	3	Singlet	FAPs
SAT_2M	1639	1040	2.0109689	4	Singlet	Immune_cells
SAT_2M	2125	1285	3.3739456	5	Singlet	Immune_cells
SAT_2M	1703	1000	3.9719626	1	Singlet	Immune_cells
SAT_2M	1335	890	1.264881	4	Singlet	Immune_cells
SAT_2M	1911	1098	2.1387585	1	Singlet	Immune_cells
SAT_2M	1363	841	2.6315789	1	Singlet	Immune_cells
SAT_2M	1430	896	0.6276151	1	Singlet	Immune_cells
SAT_2M	3248	1661	6.9653268	9	Singlet	Mesothelial
SAT_2M	3533	1850	0.5644934	0	Singlet	Adipocyte
SAT_2M	1476	836	6.7476383	0	Singlet	Adipocyte
SAT_2M	2828	1577	2.9608742	9	Singlet	Mesothelial
SAT_2M	1983	1186	0.952381	4	Singlet	Immune_cells
SAT_2M	1328	853	0.5259204	1	Singlet	Immune_cells
SAT_2M	1636	994	1.9452888	1	Singlet	Immune_cells
SAT_2M	1108	663	5.4954955	3	Singlet	FAPs
SAT_2M	1458	837	5	0	Singlet	Adipocyte
SAT_2M	1514	951	1.7015707	1	Singlet	Immune_cells
SAT_2M	1782	991	2.1324355	3	Singlet	FAPs
SAT_2M	1183	755	0.5025126	0	Singlet	Adipocyte

SAT_2M	1903	1064	8.3246073	1	Singlet	Immune_cells
SAT_2M	1142	691	2.2707424	0	Singlet	Adipocyte
SAT_2M	1968	1191	5.8467742	1	Singlet	Immune_cells
SAT_2M	1092	698	0.4549591	0	Singlet	Adipocyte
SAT_2M	1448	890	2.2587269	0	Singlet	Adipocyte
SAT_2M	2427	1464	0.2050861	6	Singlet	Neurons
SAT_2M	1357	846	0.2919708	4	Singlet	Immune_cells
SAT_2M	1522	993	0.5225343	1	Singlet	Immune_cells
SAT_2M	2769	1474	0.5755396	0	Singlet	Adipocyte
SAT_2M	1659	1030	0.359928	0	Singlet	Adipocyte
SAT_2M	1748	1018	1.9351167	0	Singlet	Adipocyte
SAT_2M	2583	1468	0.7716049	2	Singlet	FAPs
SAT_2M	1845	1183	0.2699784	11	Singlet	Smooth_muscle_cells
SAT_2M	2566	1387	3.4589973	0	Singlet	Adipocyte
SAT_2M	2731	1354	4.3034282	1	Singlet	Immune_cells
SAT_2M	1328	833	2.8528529	1	Singlet	Immune_cells
SAT_2M	1031	598	1.5473888	1	Singlet	Immune_cells
SAT_2M	3156	1702	1.5161087	1	Singlet	Immune_cells
SAT_2M	2249	1217	5.1065719	1	Singlet	Immune_cells
SAT_2M	1487	930	0.2	1	Singlet	Immune_cells
SAT_2M	1399	849	5.2142857	1	Singlet	Immune_cells
SAT_2M	1231	796	2.1018593	11	Singlet	Smooth_muscle_cells
SAT_2M	2374	1400	2.9362416	8	Singlet	Mesothelial
SAT_2M	1828	1119	2.568306	1	Singlet	Immune_cells
SAT_2M	1945	1241	2.0460358	9	Singlet	Mesothelial
SAT_2M	1045	736	3.219697	9	Singlet	Mesothelial
SAT_2M	2487	1355	0.4014452	0	Singlet	Adipocyte
SAT_2M	1308	805	3.5060976	1	Singlet	Immune_cells
SAT_2M	1655	1090	0.2403846	8	Singlet	Mesothelial
SAT_2M	1383	889	5.1873199	1	Singlet	Immune_cells
SAT_2M	1409	895	2.1111893	0	Singlet	Adipocyte
SAT_2M	1724	1045	0.9786989	0	Singlet	Adipocyte
SAT_2M	1323	807	4.826546	1	Singlet	Immune_cells
SAT_2M	1837	952	7.4796748	0	Singlet	Adipocyte
SAT_2M	1306	719	11.544343	0	Singlet	Adipocyte
SAT_2M	4030	1817	1.8312299	10	Singlet	Immune_cells
SAT_2M	1121	700	0.5319149	1	Singlet	Immune_cells
SAT_2M	1361	845	1.1730205	0	Singlet	Adipocyte
SAT_2M	1815	1049	1.980198	3	Singlet	FAPs
SAT_2M	1567	970	5.8486968	1	Singlet	Immune_cells
SAT_2M	1804	1130	0.2189382	8	Singlet	Mesothelial
SAT_2M	2312	1376	0.4735256	0	Singlet	Adipocyte
SAT_2M	1150	744	8.7931034	1	Singlet	Immune_cells
SAT_2M	4094	1896	9.1640263	9	Singlet	Mesothelial
SAT_2M	1364	884	1.0248902	9	Singlet	Mesothelial
SAT_2M	1977	1233	0.9547739	4	Singlet	Immune_cells
SAT_2M	1913	1118	5.691906	1	Singlet	Immune_cells
SAT_2M	3257	1633	8.3409997	1	Singlet	Immune_cells
SAT_2M	1102	751	6.8161435	11	Singlet	Smooth_muscle_cells
SAT_2M	1864	1072	7.2153928	4	Singlet	Immune_cells
SAT_2M	3219	1634	1.9832662	0	Singlet	Adipocyte

SAT_2M	1793	1079	0.2214839	1	Singlet	Immune_cells
SAT_2M	1661	984	0.4201681	0	Singlet	Adipocyte
SAT_2M	1709	930	11.826087	1	Singlet	Immune_cells
SAT_2M	1602	987	0.3103662	4	Singlet	Immune_cells
SAT_2M	1350	819	8.9430894	1	Singlet	Immune_cells
SAT_2M	1375	908	4.1756659	4	Singlet	Immune_cells
SAT_2M	1029	625	1.1571842	9	Singlet	Mesothelial
SAT_2M	2196	1258	9.0415913	4	Singlet	Immune_cells
SAT_2M	1103	723	0.1796945	6	Singlet	Neurons
SAT_2M	1472	945	8.3389831	1	Singlet	Immune_cells
SAT_2M	1353	846	0.5147059	1	Singlet	Immune_cells
SAT_2M	1154	755	0.2575107	1	Singlet	Immune_cells
SAT_2M	1143	707	0.9581882	0	Singlet	Adipocyte
SAT_2M	2046	1278	4.4303797	8	Singlet	Mesothelial
SAT_2M	1604	979	3.2178218	0	Singlet	Adipocyte
SAT_2M	2547	1389	0.4308656	0	Singlet	Adipocyte
SAT_2M	1253	686	12.440191	10	Singlet	Immune_cells
SAT_2M	1450	822	0.5483208	1	Singlet	Immune_cells
SAT_2M	1156	754	0.7745267	0	Singlet	Adipocyte
SAT_2M	1103	768	0.8960573	7	Singlet	Endothelial_cells
SAT_2M	1502	973	4.0585496	7	Singlet	Endothelial_cells
SAT_2M	1569	987	0.505689	4	Singlet	Immune_cells
SAT_2M	1488	902	0.2005348	4	Singlet	Immune_cells
SAT_2M	1483	984	0.6028131	8	Singlet	Mesothelial
SAT_2M	1841	1091	0.2162162	0	Singlet	Adipocyte
SAT_2M	1199	828	0.4995837	8	Singlet	Mesothelial
SAT_2M	2471	1275	9.3145161	1	Singlet	Immune_cells
SAT_2M	3590	1902	3.4751182	5	Singlet	Immune_cells
SAT_2M	1023	718	0.7774538	4	Singlet	Immune_cells
SAT_2M	1290	845	1.003861	3	Singlet	FAPs
SAT_2M	1126	725	0.7048458	2	Singlet	FAPs
SAT_2M	1895	1068	6.5893516	1	Singlet	Immune_cells
SAT_2M	1008	685	2.2660099	3	Singlet	FAPs
SAT_2M	2111	1215	0.8470588	3	Singlet	FAPs
SAT_2M	1697	1042	0.2917153	0	Singlet	Adipocyte
SAT_2M	1248	837	2.3125997	1	Singlet	Immune_cells
SAT_2M	1550	890	0.7722008	1	Singlet	Immune_cells
SAT_2M	1701	1023	0.46811	1	Singlet	Immune_cells
SAT_2M	2331	1318	2.2649573	1	Singlet	Immune_cells
SAT_2M	1303	827	4.6529367	1	Singlet	Immune_cells
SAT_2M	1033	650	2.3166023	0	Singlet	Adipocyte
SAT_2M	1142	759	0.5212858	1	Singlet	Immune_cells
SAT_2M	2606	1420	0.1530222	8	Singlet	Mesothelial
SAT_2M	1114	625	10.88314	3	Singlet	FAPs
SAT_2M	1084	688	1.6528926	5	Singlet	Immune_cells
SAT_2M	4160	2304	2.1582734	4	Singlet	Immune_cells
SAT_2M	1428	824	0.349162	10	Singlet	Immune_cells
SAT_2M	1286	804	1.3209013	0	Singlet	Adipocyte
SAT_2M	1301	837	2.607362	4	Singlet	Immune_cells
SAT_2M	1021	690	0.4854369	4	Singlet	Immune_cells
SAT_2M	2401	1331	4.1545492	1	Singlet	Immune_cells

SAT_2M	1436	901	5.7320442	1	Singlet	Immune_cells
SAT_2M	1080	738	1.8450185	4	Singlet	Immune_cells
SAT_2M	1349	906	3.8179148	1	Singlet	Immune_cells
SAT_2M	1429	877	6.6805846	1	Singlet	Immune_cells
SAT_2M	1390	852	2.2158685	1	Singlet	Immune_cells
SAT_2M	1924	1164	5.4780362	1	Singlet	Immune_cells
SAT_2M	1312	833	0.6833713	1	Singlet	Immune_cells
SAT_2M	1721	1074	0.1160093	4	Singlet	Immune_cells
SAT_2M	1337	901	0.7446016	0	Singlet	Adipocyte
SAT_2M	3841	1837	10.585176	9	Singlet	Mesothelial
SAT_2M	1176	759	3.1223629	3	Singlet	FAPs
SAT_2M	2947	1580	1.8817204	0	Singlet	Adipocyte
SAT_2M	1908	1126	0.5227392	1	Singlet	Immune_cells
SAT_2M	1406	852	4.4680851	1	Singlet	Immune_cells
SAT_2M	4249	2035	3.286385	9	Singlet	Mesothelial
SAT_2M	2873	1462	1.9110493	10	Singlet	Immune_cells
SAT_2M	1387	844	0.7168459	0	Singlet	Adipocyte
SAT_2M	3079	1640	1.0349288	0	Singlet	Adipocyte
SAT_2M	1050	685	0.5676443	4	Singlet	Immune_cells
SAT_2M	2570	1231	0.8543689	10	Singlet	Immune_cells
SAT_2M	3931	2094	8.5880273	4	Singlet	Immune_cells
SAT_2M	2476	1486	0.3626108	1	Singlet	Immune_cells
SAT_2M	1254	805	1.5007899	0	Singlet	Adipocyte
SAT_2M	1335	877	0.7440476	0	Singlet	Adipocyte
SAT_2M	3489	1747	1.3409415	0	Singlet	Adipocyte
SAT_2M	1046	715	0.7532957	5	Singlet	Immune_cells
SAT_2M	1383	927	1.218638	1	Singlet	Immune_cells
SAT_2M	1316	854	0.9848485	5	Singlet	Immune_cells
SAT_2M	2470	1379	0.2017756	0	Singlet	Adipocyte
SAT_2M	2581	1430	1.3862149	0	Singlet	Adipocyte
SAT_2M	1034	611	2.8790787	3	Singlet	FAPs
SAT_2M	1060	749	6.1739944	1	Singlet	Immune_cells
SAT_2M	1814	1071	0.933553	0	Singlet	Adipocyte
SAT_2M	1459	873	2.5974026	4	Singlet	Immune_cells
SAT_2M	1142	740	4.4386423	9	Singlet	Mesothelial
SAT_2M	1712	1025	0.5241701	4	Singlet	Immune_cells
SAT_2M	1817	1000	2.6923077	1	Singlet	Immune_cells
SAT_2M	1143	769	0.2595156	0	Singlet	Adipocyte
SAT_2M	1221	813	2.3615635	2	Singlet	FAPs
SAT_2M	1355	865	0.2941176	0	Singlet	Adipocyte
SAT_2M	3525	1858	7.9671109	9	Singlet	Mesothelial
SAT_2M	1235	782	0.8835341	0	Singlet	Adipocyte
SAT_2M	1407	874	8.1502481	1	Singlet	Immune_cells
SAT_2M	3263	1662	0.5187672	0	Singlet	Adipocyte
SAT_2M	1727	1069	0.1154734	8	Singlet	Mesothelial
SAT_2M	1055	695	1.1320755	3	Singlet	FAPs
SAT_2M	1375	859	3.968254	3	Singlet	FAPs
SAT_2M	1498	947	3.3730159	1	Singlet	Immune_cells
SAT_2M	1433	944	0	8	Singlet	Mesothelial
SAT_2M	1181	755	0.4208754	0	Singlet	Adipocyte
SAT_2M	2226	1362	0.4895416	0	Singlet	Adipocyte

SAT_2M	1410	920	0.9873061	3	Singlet	FAPs
SAT_2M	1308	787	1.3740458	0	Singlet	Adipocyte
SAT_2M	2412	1305	1.7384106	0	Singlet	Adipocyte
SAT_2M	2177	1336	2.1431829	1	Singlet	Immune_cells
SAT_2M	2028	1204	1.0314342	1	Singlet	Immune_cells
SAT_2M	3402	1869	9.6385542	9	Singlet	Mesothelial
SAT_2M	1180	772	2.1940928	1	Singlet	Immune_cells
SAT_2M	1637	966	5.0640635	0	Singlet	Adipocyte
SAT_2M	2273	1063	12.648221	10	Singlet	Immune_cells
SAT_2M	1061	704	0.5649718	0	Singlet	Adipocyte
SAT_2M	2172	1204	2.2549471	0	Singlet	Adipocyte
SAT_2M	1130	746	0.8795075	4	Singlet	Immune_cells
SAT_2M	2407	1268	1.8249689	3	Singlet	FAPs
SAT_2M	1028	665	3.7754114	4	Singlet	Immune_cells
SAT_2M	1122	690	1.5017668	10	Singlet	Immune_cells
SAT_2M	1390	869	0.4294918	0	Singlet	Adipocyte
SAT_2M	1277	803	2.5720966	4	Singlet	Immune_cells
SAT_2M	1569	941	4.1348601	7	Singlet	Endothelial_cells
SAT_2M	1384	756	12.849964	1	Singlet	Immune_cells
SAT_2M	1228	787	0.0811688	4	Singlet	Immune_cells
SAT_2M	1243	793	5.9856345	1	Singlet	Immune_cells
SAT_2M	1392	892	0.2148997	11	Singlet	Smooth_muscle_cells
SAT_2M	1826	1087	2.1845986	1	Singlet	Immune_cells
SAT_2M	1593	1056	0.8114856	6	Singlet	Neurons
SAT_2M	1658	966	1.0191847	0	Singlet	Adipocyte
SAT_2M	1445	897	0.5502063	0	Singlet	Adipocyte
SAT_2M	1215	798	1.4742015	1	Singlet	Immune_cells
SAT_2M	1725	1009	1.7877739	0	Singlet	Adipocyte
SAT_2M	1307	844	0.2283105	0	Singlet	Adipocyte
SAT_2M	3978	1753	2.5827482	10	Singlet	Immune_cells
SAT_2M	1117	747	0.6227758	4	Singlet	Immune_cells
SAT_2M	1314	853	0.150263	4	Singlet	Immune_cells
SAT_2M	2361	1323	0.2109705	8	Singlet	Mesothelial
SAT_2M	2386	1291	0.8354219	0	Singlet	Adipocyte
SAT_2M	1274	865	0	1	Singlet	Immune_cells
SAT_2M	1589	1021	7.5282309	4	Singlet	Immune_cells
SAT_2M	1320	827	0.4514673	0	Singlet	Adipocyte
SAT_2M	2655	1445	0.5609574	0	Singlet	Adipocyte
SAT_2M	1073	734	2.8837209	4	Singlet	Immune_cells
SAT_2M	1764	1049	7.4576271	1	Singlet	Immune_cells
SAT_2M	1149	763	2.5108225	11	Singlet	Smooth_muscle_cells
SAT_2M	2830	1579	0.6342495	0	Singlet	Adipocyte
SAT_2M	2022	1181	3.9980257	5	Singlet	Immune_cells
SAT_2M	1688	1036	5.2569403	4	Singlet	Immune_cells
SAT_2M	1037	714	0.3846154	1	Singlet	Immune_cells
SAT_2M	1508	894	0.9920635	8	Singlet	Mesothelial
SAT_2M	1840	1030	5.624662	10	Singlet	Immune_cells
SAT_2M	1704	1123	2.9737609	4	Singlet	Immune_cells
SAT_2M	2486	1362	0.5195843	10	Singlet	Immune_cells
SAT_2M	1723	1044	0.6373117	7	Singlet	Endothelial_cells
SAT_2M	1016	665	1.9607843	7	Singlet	Endothelial_cells

SAT_2M	1796	1132	5.0083472	4	Singlet	Immune_cells
SAT_2M	10662	3011	1.2363023	10	Singlet	Immune_cells
SAT_2M	1702	1079	0.2922268	8	Singlet	Mesothelial
SAT_2M	2499	1465	1.276426	11	Singlet	Smooth_muscle_cells
SAT_2M	1244	822	4.6325879	1	Singlet	Immune_cells
SAT_2M	2045	1235	0.8780488	1	Singlet	Immune_cells
SAT_2M	1402	891	9.787234	1	Singlet	Immune_cells
SAT_2M	1403	889	0.2126152	1	Singlet	Immune_cells
SAT_2M	1466	831	13.473257	1	Singlet	Immune_cells
SAT_2M	1120	641	5.1785714	10	Singlet	Immune_cells
SAT_2M	1283	836	0.621118	3	Singlet	FAPs
SAT_2M	2617	1373	2.0190476	0	Singlet	Adipocyte
SAT_2M	1573	918	12.6109	4	Singlet	Immune_cells
SAT_2M	1781	1103	0.5595971	4	Singlet	Immune_cells
SAT_2M	1972	1115	2.2244692	10	Singlet	Immune_cells
SAT_2M	2242	1317	4.2685638	4	Singlet	Immune_cells
SAT_2M	1079	761	0.8333333	1	Singlet	Immune_cells
SAT_2M	4495	2097	0.2658985	11	Singlet	Smooth_muscle_cells
SAT_2M	1814	1082	2.6908292	1	Singlet	Immune_cells
SAT_2M	5829	2700	1.8334476	9	Singlet	Mesothelial
SAT_2M	1467	1001	1.0183299	9	Singlet	Mesothelial
SAT_2M	1619	1000	1.7209588	1	Singlet	Immune_cells
SAT_2M	1460	929	1.6293279	0	Singlet	Adipocyte
SAT_2M	1474	897	3.5762483	10	Singlet	Immune_cells
SAT_2M	2041	1275	3.6656892	11	Singlet	Smooth_muscle_cells
SAT_2M	1602	976	7.7018634	1	Singlet	Immune_cells
SAT_2M	1398	812	14.051355	1	Singlet	Immune_cells
SAT_2M	2082	1140	6.9311663	1	Singlet	Immune_cells
SAT_2M	1734	1058	0.2296211	0	Singlet	Adipocyte
SAT_2M	2548	1741	0.6982727	6	Singlet	Neurons
SAT_2M	1293	792	0	0	Singlet	Adipocyte
SAT_2M	1516	941	3.0203546	1	Singlet	Immune_cells
SAT_2M	1314	859	0.1510574	8	Singlet	Mesothelial
SAT_2M	1580	945	1.5141956	0	Singlet	Adipocyte
SAT_2M	2409	1313	2.897351	4	Singlet	Immune_cells
SAT_2M	1815	1108	0.9863014	8	Singlet	Mesothelial
SAT_2M	1606	988	2.7278363	7	Singlet	Endothelial_cells
SAT_2M	1425	916	7.5471698	4	Singlet	Immune_cells
SAT_2M	1302	819	0.1530222	1	Singlet	Immune_cells
SAT_2M	1877	1009	5.3780618	5	Singlet	Immune_cells
SAT_2M	1029	687	0.7736944	4	Singlet	Immune_cells
SAT_2M	1162	866	3.0936455	6	Singlet	Neurons
SAT_2M	1620	992	0.3071253	0	Singlet	Adipocyte
SAT_2M	2507	1344	1.2718601	3	Singlet	FAPs
SAT_2M	1443	896	4.4982699	1	Singlet	Immune_cells
SAT_2M	1454	959	0.4103967	0	Singlet	Adipocyte
SAT_2M	1022	696	0.3875969	4	Singlet	Immune_cells
SAT_2M	1738	1013	3.8790645	1	Singlet	Immune_cells
SAT_2M	1114	738	0.1784121	5	Singlet	Immune_cells
SAT_2M	1737	1061	0.9142857	0	Singlet	Adipocyte
SAT_2M	2275	1203	12.472552	4	Singlet	Immune_cells

SAT_2M	4292	1945	1.6042781	0	Singlet	Adipocyte
SAT_2M	2276	1193	5.0854888	1	Singlet	Immune_cells
SAT_2M	3883	1856	0.7958922	0	Singlet	Adipocyte
SAT_2M	1402	857	0.2840909	4	Singlet	Immune_cells
SAT_2M	1271	849	3.5714286	1	Singlet	Immune_cells
SAT_2M	1258	843	4.1897233	1	Singlet	Immune_cells
SAT_2M	2100	1182	0.3789673	0	Singlet	Adipocyte
SAT_2M	4052	2170	6.5747353	4	Singlet	Immune_cells
SAT_2M	1010	638	1.9646365	4	Singlet	Immune_cells
SAT_2M	1120	732	4.1814947	1	Singlet	Immune_cells
SAT_2M	1493	935	9.6064043	4	Singlet	Immune_cells
SAT_2M	1045	704	0.095057	1	Singlet	Immune_cells
SAT_2M	1577	967	1.7056222	1	Singlet	Immune_cells
SAT_2M	1230	787	4.6925566	3	Singlet	FAPs
SAT_2M	1076	732	1.9319227	4	Singlet	Immune_cells
SAT_2M	1655	938	5.632115	1	Singlet	Immune_cells
SAT_2M	1201	808	0.8298755	2	Singlet	FAPs
SAT_2M	3353	1715	0.3861004	0	Singlet	Adipocyte
SAT_2M	1857	1001	4.0728832	0	Singlet	Adipocyte
SAT_2M	1699	995	5.9883721	1	Singlet	Immune_cells
SAT_2M	1801	1077	0.166021	0	Singlet	Adipocyte
SAT_2M	1995	1225	2.3511756	1	Singlet	Immune_cells
SAT_2M	1084	729	3.59447	5	Singlet	Immune_cells
SAT_2M	1145	768	0.3478261	3	Singlet	FAPs
SAT_2M	1715	1109	4.0023202	5	Singlet	Immune_cells
SAT_2M	1343	824	4.6062407	1	Singlet	Immune_cells
SAT_2M	2018	1282	0.3455084	5	Singlet	Immune_cells
SAT_2M	1519	890	10.512484	1	Singlet	Immune_cells
SAT_2M	1889	1102	4.9154334	9	Singlet	Mesothelial
SAT_2M	1048	677	0.8530806	0	Singlet	Adipocyte
SAT_2M	1118	745	5.5258467	1	Singlet	Immune_cells
SAT_2M	2144	1205	2.5605214	1	Singlet	Immune_cells
SAT_2M	1552	931	0.5118362	1	Singlet	Immune_cells
SAT_2M	4183	1734	0.9060563	10	Singlet	Immune_cells
SAT_2M	1439	968	0.2766252	4	Singlet	Immune_cells
SAT_2M	1178	740	6.6892464	1	Singlet	Immune_cells
SAT_2M	1772	1123	1.3498313	1	Singlet	Immune_cells
SAT_2M	1938	1240	2.4729521	8	Singlet	Mesothelial
SAT_2M	2215	1177	4.6292135	0	Singlet	Adipocyte
SAT_2M	2454	1432	0.2436054	8	Singlet	Mesothelial
SAT_2M	2624	1420	7.8787879	1	Singlet	Immune_cells
SAT_2M	1204	823	1.399177	1	Singlet	Immune_cells
SAT_2M	3385	1753	0.2946376	8	Singlet	Mesothelial
SAT_2M	1375	882	0.1451379	5	Singlet	Immune_cells
SAT_2M	1463	931	0.4067797	4	Singlet	Immune_cells
SAT_2M	1231	802	2.9102668	1	Singlet	Immune_cells
SAT_2M	3675	1751	0.1896505	8	Singlet	Mesothelial
SAT_2M	1435	895	0.3479471	0	Singlet	Adipocyte
SAT_2M	1476	941	4.1271989	0	Singlet	Adipocyte
SAT_2M	1463	963	1.0876954	1	Singlet	Immune_cells
SAT_2M	1976	1195	1.0568697	0	Singlet	Adipocyte

SAT_2M	1306	856	0.6880734	4	Singlet	Immune_cells
SAT_2M	1383	855	1.7291066	0	Singlet	Adipocyte
SAT_2M	1684	1019	8.7781732	9	Singlet	Mesothelial
SAT_2M	1248	775	0.3996803	1	Singlet	Immune_cells
SAT_2M	1839	1139	0.4867496	0	Singlet	Adipocyte
SAT_2M	4988	2171	1.8596281	10	Singlet	Immune_cells
SAT_2M	9045	2982	1.2571681	10	Singlet	Immune_cells
SAT_2M	1097	762	4.17802	1	Singlet	Immune_cells
SAT_2M	1334	862	2.2321429	1	Singlet	Immune_cells
SAT_2M	1269	858	0.6259781	4	Singlet	Immune_cells
SAT_2M	1777	1039	7.8991597	1	Singlet	Immune_cells
SAT_2M	1231	822	1.1326861	2	Singlet	FAPs
SAT_2M	2678	1382	4.7282204	0	Singlet	Adipocyte
SAT_2M	2278	1279	0.1313485	0	Singlet	Adipocyte
SAT_2M	2704	1428	3.8063562	3	Singlet	FAPs
SAT_2M	1235	812	5.7211926	1	Singlet	Immune_cells
SAT_2M	2449	1434	0.0813339	0	Singlet	Adipocyte
SAT_2M	1631	1025	0.3660769	0	Singlet	Adipocyte
SAT_2M	1314	817	6.4540623	1	Singlet	Immune_cells
SAT_2M	1276	773	4.4392523	1	Singlet	Immune_cells
SAT_2M	1133	767	0.9683099	0	Singlet	Adipocyte
SAT_2M	2377	1443	0.5035669	8	Singlet	Mesothelial
SAT_2M	1466	900	3.0674847	1	Singlet	Immune_cells
SAT_2M	1373	893	2.6143791	1	Singlet	Immune_cells
SAT_2M	1216	726	10.620915	1	Singlet	Immune_cells
SAT_2M	1014	702	1.6715831	1	Singlet	Immune_cells
SAT_2M	4412	1977	1.8325792	10	Singlet	Immune_cells
SAT_2M	1006	650	1.1904762	0	Singlet	Adipocyte
SAT_2M	2308	1315	1.9006479	3	Singlet	FAPs
SAT_2M	1456	889	7.0499658	1	Singlet	Immune_cells
SAT_2M	1024	679	1.1661808	0	Singlet	Adipocyte
SAT_2M	6579	3049	4.8284239	9	Singlet	Mesothelial
SAT_2M	1393	871	0.5	0	Singlet	Adipocyte
SAT_2M	2592	1416	0.5392912	7	Singlet	Endothelial_cells
SAT_2M	1114	737	0.1793722	1	Singlet	Immune_cells
SAT_2M	2572	1437	0.5791506	0	Singlet	Adipocyte
SAT_2M	1857	1072	0.3755365	0	Singlet	Adipocyte
SAT_2M	1509	923	2.7134348	1	Singlet	Immune_cells
SAT_2M	1052	669	2.173913	9	Singlet	Mesothelial
SAT_2M	3039	1493	1.6721311	10	Singlet	Immune_cells
SAT_2M	1325	856	2.8294862	4	Singlet	Immune_cells
SAT_2M	1126	696	0.1774623	4	Singlet	Immune_cells
SAT_2M	1239	779	8.8729017	1	Singlet	Immune_cells
SAT_2M	1878	1200	1.6419492	4	Singlet	Immune_cells
SAT_2M	1231	782	0.4038772	4	Singlet	Immune_cells
SAT_2M	1502	955	7.6462766	1	Singlet	Immune_cells
SAT_2M	2015	1174	3.8575668	4	Singlet	Immune_cells
SAT_2M	1372	864	7.5090253	1	Singlet	Immune_cells
SAT_2M	1254	784	8.5443038	1	Singlet	Immune_cells
SAT_2M	2768	1582	5.7553957	5	Singlet	Immune_cells
SAT_2M	2618	1431	1.0662605	5	Singlet	Immune_cells

SAT_2M	2538	1415	0.8235294	0	Singlet	Adipocyte
SAT_2M	2839	1292	5.7996485	5	Singlet	Immune_cells
SAT_2M	1959	1071	5.7084608	1	Singlet	Immune_cells
SAT_2M	1252	802	1.1119936	4	Singlet	Immune_cells
SAT_2M	2527	1504	6.855792	4	Singlet	Immune_cells
SAT_2M	1239	800	1.9354839	1	Singlet	Immune_cells
SAT_2M	1673	978	9.6908442	1	Singlet	Immune_cells
SAT_2M	1297	788	0.7698229	1	Singlet	Immune_cells
SAT_2M	2557	1227	2.9664325	10	Singlet	Immune_cells
SAT_2M	1682	1075	4.6745562	1	Singlet	Immune_cells
SAT_2M	1124	677	5.0353357	0	Singlet	Adipocyte
SAT_2M	1912	1145	0.4694836	0	Singlet	Adipocyte
SAT_2M	2032	1217	0.7846984	0	Singlet	Adipocyte
SAT_2M	1362	850	0.727802	0	Singlet	Adipocyte
SAT_2M	1696	1054	2.28739	0	Singlet	Adipocyte
SAT_2M	3117	1658	0.3187759	0	Singlet	Adipocyte
SAT_2M	1549	910	7.1702945	5	Singlet	Immune_cells
SAT_2M	1088	690	0.5484461	0	Singlet	Adipocyte
SAT_2M	2991	1695	0.2333333	5	Singlet	Immune_cells
SAT_2M	1237	812	0.5649718	7	Singlet	Endothelial_cells
SAT_2M	2335	1386	1.0660981	0	Singlet	Adipocyte
SAT_2M	1241	856	1.1235955	4	Singlet	Immune_cells
SAT_2M	1010	719	0.489716	5	Singlet	Immune_cells
SAT_2M	1182	742	1.1804384	0	Singlet	Adipocyte
SAT_2M	3042	1543	0.295082	3	Singlet	FAPs
SAT_2M	2587	1383	0.7310504	0	Singlet	Adipocyte
SAT_2M	1553	997	0.0640615	4	Singlet	Immune_cells
SAT_2M	1305	853	0.8409786	4	Singlet	Immune_cells
SAT_2M	2085	1171	0.9043313	0	Singlet	Adipocyte
SAT_2M	1229	799	4.6079224	1	Singlet	Immune_cells
SAT_2M	1756	1001	8.0225989	5	Singlet	Immune_cells
SAT_2M	2601	1430	8.3237438	1	Singlet	Immune_cells
SAT_2M	1504	845	12.524851	9	Singlet	Mesothelial
SAT_2M	1031	650	0.7722008	9	Singlet	Mesothelial
SAT_2M	1497	893	1.6600266	1	Singlet	Immune_cells
SAT_2M	3483	1703	2.5494128	0	Singlet	Adipocyte
SAT_2M	1987	1162	0.4518072	0	Singlet	Adipocyte
SAT_2M	1583	997	0.3761755	0	Singlet	Adipocyte
SAT_2M	1721	964	2.5507246	3	Singlet	FAPs
SAT_2M	3041	1349	0.9520683	10	Singlet	Immune_cells
SAT_2M	1308	883	3.196347	1	Singlet	Immune_cells
SAT_2M	1164	737	0.7718696	7	Singlet	Endothelial_cells
SAT_2M	1346	840	9.7037037	1	Singlet	Immune_cells
SAT_2M	2368	1271	0.1264223	0	Singlet	Adipocyte
SAT_2M	1418	826	2.5263158	1	Singlet	Immune_cells
SAT_2M	1249	825	0.6309148	6	Singlet	Neurons
SAT_2M	1239	774	3.6173633	1	Singlet	Immune_cells
SAT_2M	1767	1077	3.8961039	1	Singlet	Immune_cells
SAT_2M	1013	673	0.492126	5	Singlet	Immune_cells
SAT_2M	1372	855	0.4338395	4	Singlet	Immune_cells
SAT_2M	3336	1765	4.4179104	2	Singlet	FAPs

SAT_2M	1095	774	3.0520646	1	Singlet	Immune_cells
SAT_2M	2715	1636	3.5701141	8	Singlet	Mesothelial
SAT_2M	1428	946	0.8362369	4	Singlet	Immune_cells
SAT_2M	1248	802	0.8757962	1	Singlet	Immune_cells
SAT_2M	1176	854	0.8210181	6	Singlet	Neurons
SAT_2M	2007	1133	5.5278884	0	Singlet	Adipocyte
SAT_2M	1579	959	1.5180266	1	Singlet	Immune_cells
SAT_2M	2102	1329	1.5617605	4	Singlet	Immune_cells
SAT_2M	1965	1197	2.7918782	9	Singlet	Mesothelial
SAT_2M	1134	806	0.0881057	5	Singlet	Immune_cells
SAT_2M	1509	918	5.5884287	1	Singlet	Immune_cells
SAT_2M	1856	1090	5.8033315	1	Singlet	Immune_cells
SAT_2M	3154	1668	1.1646207	0	Singlet	Adipocyte
SAT_2M	1412	922	0.9797061	5	Singlet	Immune_cells
SAT_2M	4699	2229	6.8621202	9	Singlet	Mesothelial
SAT_2M	1310	818	2.0532319	4	Singlet	Immune_cells
SAT_2M	1027	745	0.5780347	4	Singlet	Immune_cells
SAT_2M	4728	2175	5.2443134	2	Singlet	FAPs
SAT_2M	1262	822	1.7309205	1	Singlet	Immune_cells
SAT_2M	6925	2512	1.7564066	10	Singlet	Immune_cells
SAT_2M	2925	1564	0.2037351	0	Singlet	Adipocyte
SAT_2M	1935	1125	7.9299691	1	Singlet	Immune_cells
SAT_2M	1024	678	0.8789063	5	Singlet	Immune_cells
SAT_2M	1437	880	2.2853186	0	Singlet	Adipocyte
SAT_2M	2521	1440	1.1890606	0	Singlet	Adipocyte
SAT_2M	1306	758	7.0068545	1	Singlet	Immune_cells
SAT_2M	1394	840	5.003574	1	Singlet	Immune_cells
SAT_2M	2613	1539	0.1526135	8	Singlet	Mesothelial
SAT_26M	1883.7	1192	1.2075214	0	Singlet	Adipocyte
SAT_26M	2878.3	1311	1.2144219	10	Singlet	Immune_cells
SAT_26M	1975.9	1220	3.5175759	1	Singlet	Immune_cells
SAT_26M	1188.5	739	0.4481828	0	Singlet	Adipocyte
SAT_26M	6008.2	2720	5.2941298	9	Singlet	Mesothelial
SAT_26M	1233.5	813	7.4049231	1	Singlet	Immune_cells
SAT_26M	1235.9	777	2.2466076	8	Singlet	Mesothelial
SAT_26M	1255.7	694	2.1676155	10	Singlet	Immune_cells
SAT_26M	1268.1	843	4.7721062	1	Singlet	Immune_cells
SAT_26M	2252.9	1186	5.583768	0	Singlet	Adipocyte
SAT_26M	4117.2	1680	2.3343528	10	Singlet	Immune_cells
SAT_26M	1185.1	962	2.4721801	7	Singlet	Endothelial_cells
SAT_26M	1069.6	788	5.1164723	4	Singlet	Immune_cells
SAT_26M	4829.5	2346	12.134165	9	Singlet	Mesothelial
SAT_26M	1150.8	748	0	0	Singlet	Adipocyte
SAT_26M	3536.7	1841	2.493556	8	Singlet	Mesothelial
SAT_26M	1086.4	749	7.4032883	4	Singlet	Immune_cells
SAT_26M	1855.2	1128	0.0203999	0	Singlet	Adipocyte
SAT_26M	1004.9	665	10.49664	5	Singlet	Immune_cells
SAT_26M	2485	1433	0.1730189	0	Singlet	Adipocyte
SAT_26M	1570.5	1102	3.1518605	11	Singlet	Smooth_muscle_cells
SAT_26M	2258.8	1253	0.0263302	8	Singlet	Mesothelial
SAT_26M	1731.1	995	1.4554321	0	Singlet	Adipocyte

SAT_26M	1234.1	818	4.9042755	1	Singlet	Immune_cells
SAT_26M	2935.7	1521	4.3623914	2	Singlet	FAPs
SAT_26M	1362.8	843	0.4165156	0	Singlet	Adipocyte
SAT_26M	5415.5	1956	0.6446745	10	Singlet	Immune_cells
SAT_26M	1002.4	654	13.372891	1	Singlet	Immune_cells
SAT_26M	1726.3	1050	0.2474342	5	Singlet	Immune_cells
SAT_26M	2459.1	1371	1.6473345	0	Singlet	Adipocyte
SAT_26M	1911.7	1151	2.0997621	0	Singlet	Adipocyte
SAT_26M	6969.5	3206	4.6723708	8	Singlet	Mesothelial
SAT_26M	1229	822	3.8649479	1	Singlet	Immune_cells
SAT_26M	1224.2	959	7.1818462	4	Singlet	Immune_cells
SAT_26M	1224.1	848	0.8478708	11	Singlet	Smooth_muscle_cells
SAT_26M	1379.6	888	5.8965026	8	Singlet	Mesothelial
SAT_26M	1004.1	686	1.8790038	0	Singlet	Adipocyte
SAT_26M	1542.8	955	1.3923159	0	Singlet	Adipocyte
SAT_26M	1347.1	718	5.1828392	10	Singlet	Immune_cells
SAT_26M	1877.1	979	1.7560029	10	Singlet	Immune_cells
SAT_26M	1324.1	634	9.5313295	10	Singlet	Immune_cells
SAT_26M	1127	868	8.7126749	1	Singlet	Immune_cells
SAT_26M	1444.7	1035	8.1571522	4	Singlet	Immune_cells
SAT_26M	1130.1	740	0.8570772	4	Singlet	Immune_cells
SAT_26M	2270.1	1348	3.3792084	1	Singlet	Immune_cells
SAT_26M	1200.4	790	2.4881314	4	Singlet	Immune_cells
SAT_26M	1140.7	752	8.5686832	1	Singlet	Immune_cells
SAT_26M	1283.3	848	0.4244795	0	Singlet	Adipocyte
SAT_26M	1583.7	1263	4.6324862	6	Singlet	Neurons
SAT_26M	1265.7	821	4.6392413	0	Singlet	Adipocyte
SAT_26M	1589.1	995	1.1710661	8	Singlet	Mesothelial
SAT_26M	1081.8	621	4.0924971	10	Singlet	Immune_cells
SAT_26M	1006.1	679	7.7496444	1	Singlet	Immune_cells
SAT_26M	1860.9	1047	3.4247642	0	Singlet	Adipocyte
SAT_26M	1379.7	888	0.8221353	0	Singlet	Adipocyte
SAT_26M	1888.8	1206	12.726228	4	Singlet	Immune_cells
SAT_26M	1724.1	1110	2.7956334	1	Singlet	Immune_cells
SAT_26M	1282.6	845	0.3395063	3	Singlet	FAPs
SAT_26M	1307	794	3.4843249	10	Singlet	Immune_cells
SAT_26M	1321.7	808	4.8286256	0	Singlet	Adipocyte
SAT_26M	3331.6	1763	4.6177342	5	Singlet	Immune_cells
SAT_26M	1071.2	727	0.5497165	0	Singlet	Adipocyte
SAT_26M	1382.2	967	0.1852882	7	Singlet	Endothelial_cells
SAT_26M	1281.1	913	13.256612	6	Singlet	Neurons
SAT_26M	1035	714	1.1521772	0	Singlet	Adipocyte
SAT_26M	2448	1440	4.0790495	9	Singlet	Mesothelial
SAT_26M	1080.6	715	13.708665	4	Singlet	Immune_cells
SAT_26M	3038.2	1573	0.107757	0	Singlet	Adipocyte
SAT_26M	1179.6	822	0.864336	5	Singlet	Immune_cells
SAT_26M	1875.3	1040	7.2316768	0	Singlet	Adipocyte
SAT_26M	1643.6	1061	0	8	Singlet	Mesothelial
SAT_26M	1471.4	867	1.7494987	0	Singlet	Adipocyte
SAT_26M	1682.1	1116	2.2555046	1	Singlet	Immune_cells
SAT_26M	1611.6	1222	2.3190327	6	Singlet	Neurons

SAT_26M	1579.9	1131	5.5123909	8	Singlet	Mesothelial
SAT_26M	2628.7	1564	4.2106766	4	Singlet	Immune_cells
SAT_26M	1247.3	934	5.8726412	4	Singlet	Immune_cells
SAT_26M	4187.9	2088	2.4106904	2	Singlet	FAPs
SAT_26M	1012.1	673	3.7029185	0	Singlet	Adipocyte
SAT_26M	1165.1	793	2.5134021	1	Singlet	Immune_cells
SAT_26M	1388	918	2.7322629	1	Singlet	Immune_cells
SAT_26M	2489.8	1282	12.729318	0	Singlet	Adipocyte
SAT_26M	1528.5	997	2.4001596	8	Singlet	Mesothelial
SAT_26M	1114.2	773	0	4	Singlet	Immune_cells
SAT_26M	1059.7	882	0	6	Singlet	Neurons
SAT_26M	2151.4	1305	1.6002513	8	Singlet	Mesothelial
SAT_26M	2488.2	1366	0.4610215	0	Singlet	Adipocyte
SAT_26M	2694.2	1382	0.0118541	2	Singlet	FAPs
SAT_26M	1058.4	885	2.6157943	4	Singlet	Immune_cells
SAT_26M	2557.7	1370	1.1482258	10	Singlet	Immune_cells
SAT_26M	2110	1276	5.1183452	1	Singlet	Immune_cells
SAT_26M	3074.6	1494	0.4929199	0	Singlet	Adipocyte
SAT_26M	1090.8	723	0	0	Singlet	Adipocyte
SAT_26M	1608.5	967	4.8573675	0	Singlet	Adipocyte
SAT_26M	4979	2588	2.0823429	8	Singlet	Mesothelial
SAT_26M	1632.9	977	0.2122495	3	Singlet	FAPs
SAT_26M	1751.8	1258	5.289683	8	Singlet	Mesothelial
SAT_26M	2691.5	1652	2.4727686	8	Singlet	Mesothelial
SAT_26M	1029.1	702	10.005134	11	Singlet	Smooth_muscle_cells
SAT_26M	1018.6	708	1.0366361	11	Singlet	Smooth_muscle_cells
SAT_26M	1296.4	639	7.2413472	9	Singlet	Mesothelial
SAT_26M	2218.4	1238	2.4321396	0	Singlet	Adipocyte
SAT_26M	3228.1	1647	8.7777294	9	Singlet	Mesothelial
SAT_26M	1442.4	750	7.9523647	9	Singlet	Mesothelial
SAT_26M	1299.9	655	7.6231104	10	Singlet	Immune_cells
SAT_26M	1179	821	4.3484301	7	Singlet	Endothelial_cells
SAT_26M	3570.8	1731	0.1585393	0	Singlet	Adipocyte
SAT_26M	4709.7	1919	2.8118826	10	Singlet	Immune_cells
SAT_26M	2622.1	1373	0.3860527	0	Singlet	Adipocyte
SAT_26M	1396.6	920	5.5114103	1	Singlet	Immune_cells
SAT_26M	2822.1	1351	3.3693935	10	Singlet	Immune_cells
SAT_26M	2954.1	1427	1.9413665	0	Singlet	Adipocyte
SAT_26M	1003.9	640	1.5048952	0	Singlet	Adipocyte
SAT_26M	1096.5	739	0.0691317	8	Singlet	Mesothelial
SAT_26M	1596.4	1063	5.4317173	4	Singlet	Immune_cells
SAT_26M	1752.3	887	5.093954	10	Singlet	Immune_cells
SAT_26M	1223.4	811	5.1725832	1	Singlet	Immune_cells
SAT_26M	1016.1	755	0.0304047	4	Singlet	Immune_cells
SAT_26M	1540.8	918	11.267851	4	Singlet	Immune_cells
SAT_26M	1906.7	1153	0.0403336	8	Singlet	Mesothelial
SAT_26M	1630.1	999	0.8881326	0	Singlet	Adipocyte
SAT_26M	1083.5	731	2.7516609	4	Singlet	Immune_cells
SAT_26M	1821.1	944	5.0081148	7	Singlet	Endothelial_cells
SAT_26M	1336.9	832	3.7348008	8	Singlet	Mesothelial
SAT_26M	1575.5	917	0.1830689	0	Singlet	Adipocyte

SAT_26M	5085.1	1799	1.5747985	10	Singlet	Immune_cells
SAT_26M	2192.8	1098	2.659234	10	Singlet	Immune_cells
SAT_26M	2507.4	1445	1.4063122	1	Singlet	Immune_cells
SAT_26M	1311.1	979	10.952837	4	Singlet	Immune_cells
SAT_26M	1267.7	781	0.01573	0	Singlet	Adipocyte
SAT_26M	1159.1	615	5.2557066	10	Singlet	Immune_cells
SAT_26M	1183	800	8.0835992	8	Singlet	Mesothelial
SAT_26M	1684.3	989	10.30141	9	Singlet	Mesothelial
SAT_26M	2097.9	1231	5.115955	8	Singlet	Mesothelial
SAT_26M	1296.6	850	0.7440965	3	Singlet	FAPs
SAT_26M	1084.8	670	14.209023	4	Singlet	Immune_cells
SAT_26M	1194.6	933	2.5065381	4	Singlet	Immune_cells
SAT_26M	1185.3	731	13.540154	1	Singlet	Immune_cells
SAT_26M	1112.2	692	0.511386	0	Singlet	Adipocyte
SAT_26M	1835.4	1076	0.0132554	0	Singlet	Adipocyte
SAT_26M	1045	603	0.5496122	0	Singlet	Adipocyte
SAT_26M	2075.9	1168	0.9421231	0	Singlet	Adipocyte
SAT_26M	1460.4	820	6.0736137	0	Singlet	Adipocyte
SAT_26M	1363.8	836	0.0110269	0	Singlet	Adipocyte
SAT_26M	1421.1	873	7.248363	4	Singlet	Immune_cells
SAT_26M	1986.6	1098	0.7652718	0	Singlet	Adipocyte
SAT_26M	1367.5	941	0.4181144	4	Singlet	Immune_cells
SAT_26M	2389.4	1334	0.0162503	0	Singlet	Adipocyte
SAT_26M	1093.4	753	3.104581	1	Singlet	Immune_cells
SAT_26M	2907.8	1582	2.1460911	9	Singlet	Mesothelial
SAT_26M	1875.1	1101	0.3440727	0	Singlet	Adipocyte
SAT_26M	1871.8	1222	10.297623	9	Singlet	Mesothelial
SAT_26M	1721	848	5.8579335	10	Singlet	Immune_cells
SAT_26M	2166.9	1282	3.0628396	1	Singlet	Immune_cells
SAT_26M	1128.9	744	0.7097197	0	Singlet	Adipocyte
SAT_26M	1955.8	1122	4.3213316	3	Singlet	FAPs
SAT_26M	2804.4	1408	9.5449921	3	Singlet	FAPs
SAT_26M	1676.1	1054	4.6080216	4	Singlet	Immune_cells
SAT_26M	1131.8	833	0.7203249	6	Singlet	Neurons
SAT_26M	2149.4	1332	0.0315999	8	Singlet	Mesothelial
SAT_26M	1309.8	767	0.1752767	2	Singlet	FAPs
SAT_26M	1544.7	937	0	0	Singlet	Adipocyte
SAT_26M	3210.9	1716	9.3306249	9	Singlet	Mesothelial
SAT_26M	1783.3	1120	2.8769959	7	Singlet	Endothelial_cells
SAT_26M	2999.7	1359	2.8160272	10	Singlet	Immune_cells
SAT_26M	1846.2	1276	1.877454	8	Singlet	Mesothelial
SAT_26M	1321.9	835	5.7210988	1	Singlet	Immune_cells
SAT_26M	2599.3	1282	2.1590037	0	Singlet	Adipocyte
SAT_26M	2027.1	1182	3.0708285	1	Singlet	Immune_cells
SAT_26M	2775.7	1345	1.5090204	10	Singlet	Immune_cells
SAT_26M	1424.3	867	1.5651771	3	Singlet	FAPs
SAT_26M	1120.6	880	8.4514833	6	Singlet	Neurons
SAT_26M	2432.2	1355	0.0406568	0	Singlet	Adipocyte
SAT_26M	1054.2	754	0.5349476	1	Singlet	Immune_cells
SAT_26M	2014.2	1246	10.274092	5	Singlet	Immune_cells
SAT_26M	2426.5	1193	2.7381979	10	Singlet	Immune_cells

SAT_26M	2321.9	1347	6.7079741	8	Singlet	Mesothelial
SAT_26M	3876.9	1830	2.3190704	10	Singlet	Immune_cells
SAT_26M	1587.4	924	0.0219516	0	Singlet	Adipocyte
SAT_26M	1245.9	837	3.5421682	8	Singlet	Mesothelial
SAT_26M	1531.2	948	9.6043577	5	Singlet	Immune_cells
SAT_26M	1269.5	817	0.6168372	0	Singlet	Adipocyte
SAT_26M	2616	1408	0	0	Singlet	Adipocyte
SAT_26M	1006	676	3.5665918	1	Singlet	Immune_cells
SAT_26M	1608.4	1033	5.3743334	1	Singlet	Immune_cells
SAT_26M	1695.8	1068	3.2487391	7	Singlet	Endothelial_cells
SAT_26M	1583.3	1294	0	6	Singlet	Neurons
SAT_26M	1857.9	1131	2.5453783	1	Singlet	Immune_cells
SAT_26M	1657.1	567	8.3216418	7	Singlet	Endothelial_cells
SAT_26M	1040.8	700	0	8	Singlet	Mesothelial
SAT_26M	3651.9	1926	3.4220002	8	Singlet	Mesothelial
SAT_26M	1031.1	777	7.9743129	5	Singlet	Immune_cells
SAT_26M	1019.5	821	0.0610521	6	Singlet	Neurons
SAT_26M	1115.2	743	0.0676615	4	Singlet	Immune_cells
SAT_26M	2312.1	1502	0.8381872	11	Singlet	Smooth_muscle_cells
SAT_26M	1814.2	858	0.6517721	10	Singlet	Immune_cells
SAT_26M	1436.6	834	0.700062	0	Singlet	Adipocyte
SAT_26M	1428.4	955	4.6922607	8	Singlet	Mesothelial
SAT_26M	1247.9	774	0.1370993	0	Singlet	Adipocyte
SAT_26M	2295.5	1154	2.3001057	10	Singlet	Immune_cells
SAT_26M	2317.1	1299	0.4534711	0	Singlet	Adipocyte
SAT_26M	2672.7	1499	0.0348954	8	Singlet	Mesothelial
SAT_26M	1986.8	1133	0.1458397	0	Singlet	Adipocyte
SAT_26M	1507	891	4.1440339	0	Singlet	Adipocyte
SAT_26M	1010.1	674	6.3005548	1	Singlet	Immune_cells
SAT_26M	1392.8	811	1.1754356	0	Singlet	Adipocyte
SAT_26M	1066	689	0.0564064	4	Singlet	Immune_cells
SAT_26M	1638.1	1013	4.8409566	7	Singlet	Endothelial_cells
SAT_26M	1567.1	931	1.389015	3	Singlet	FAPs
SAT_26M	2973.5	1526	2.3195582	8	Singlet	Mesothelial
SAT_26M	2166.2	1293	0.0126252	8	Singlet	Mesothelial
SAT_26M	1575.4	966	2.1063332	1	Singlet	Immune_cells
SAT_26M	4857.8	1852	2.3627997	10	Singlet	Immune_cells
SAT_26M	2137.5	1071	4.4234178	0	Singlet	Adipocyte
SAT_26M	3165	1541	3.0520621	0	Singlet	Adipocyte
SAT_26M	1286.9	848	0.4111163	1	Singlet	Immune_cells
SAT_26M	1036.4	703	2.4357125	4	Singlet	Immune_cells
SAT_26M	1391	951	2.5734012	1	Singlet	Immune_cells
SAT_26M	1785.9	1241	5.1242474	11	Singlet	Smooth_muscle_cells
SAT_26M	1057.3	690	2.3254992	0	Singlet	Adipocyte
SAT_26M	1507.5	1027	1.9457758	0	Singlet	Adipocyte
SAT_26M	1109.8	807	0.6245149	4	Singlet	Immune_cells
SAT_26M	1206.1	765	1.1385972	3	Singlet	FAPs
SAT_26M	12356	3743	0	0	Singlet	Adipocyte
SAT_26M	1107.5	790	2.5497017	11	Singlet	Smooth_muscle_cells
SAT_26M	1311.2	798	14.651759	1	Singlet	Immune_cells
SAT_26M	1013.3	790	5.4738244	1	Singlet	Immune_cells

SAT_26M	1748	1077	9.4931046	0	Singlet	Adipocyte
SAT_26M	2147.6	1327	0	8	Singlet	Mesothelial
SAT_26M	1289.5	784	0	0	Singlet	Adipocyte
SAT_26M	3169.8	1835	1.7016001	8	Singlet	Mesothelial
SAT_26M	1156.5	734	0.6607837	0	Singlet	Adipocyte
SAT_26M	2369.1	1414	4.6531231	1	Singlet	Immune_cells
SAT_26M	2507	1278	7.4543018	0	Singlet	Adipocyte
SAT_26M	1009.5	722	0.0628926	11	Singlet	Smooth_muscle_cells
SAT_26M	1203.6	747	1.0523244	2	Singlet	FAPs
SAT_26M	1892.8	1140	4.2956731	1	Singlet	Immune_cells
SAT_26M	1353.8	896	5.2874685	1	Singlet	Immune_cells
SAT_26M	2848.1	1435	1.4524972	0	Singlet	Adipocyte
SAT_26M	1621.6	1052	4.1587875	8	Singlet	Mesothelial
SAT_26M	2231.8	1273	6.8077474	8	Singlet	Mesothelial
SAT_26M	1109.7	660	1.2763194	10	Singlet	Immune_cells
SAT_26M	1051.9	773	8.3104394	4	Singlet	Immune_cells
SAT_26M	4061.4	2090	12.270182	4	Singlet	Immune_cells
SAT_26M	1333	1021	8.7259566	4	Singlet	Immune_cells
SAT_26M	1104.2	687	3.8524675	2	Singlet	FAPs
SAT_26M	1680.5	1020	4.2917422	9	Singlet	Mesothelial
SAT_26M	1864.8	1173	1.607591	1	Singlet	Immune_cells
SAT_26M	4125.1	1720	1.6255212	10	Singlet	Immune_cells
SAT_26M	2221.9	1424	7.6288077	4	Singlet	Immune_cells
SAT_26M	1062.2	776	6.4388133	4	Singlet	Immune_cells
SAT_26M	5858.9	2970	2.5544508	8	Singlet	Mesothelial
SAT_26M	1312.9	825	3.6850315	0	Singlet	Adipocyte
SAT_26M	1237.9	769	6.7925374	1	Singlet	Immune_cells
SAT_26M	1105.3	790	7.0836843	4	Singlet	Immune_cells
SAT_26M	4332.3	2227	11.121405	5	Singlet	Immune_cells
SAT_26M	1202	773	0.0581464	0	Singlet	Adipocyte
SAT_26M	2964.6	1437	0.5205436	0	Singlet	Adipocyte
SAT_26M	1937.6	1085	0	0	Singlet	Adipocyte
SAT_26M	1860.6	1106	4.9133167	1	Singlet	Immune_cells
SAT_26M	1942.2	1118	6.7004668	4	Singlet	Immune_cells
SAT_26M	4736.7	2122	1.8451421	0	Singlet	Adipocyte
SAT_26M	1240.1	795	2.3880536	1	Singlet	Immune_cells
SAT_26M	1612.1	1081	4.8870669	1	Singlet	Immune_cells
SAT_26M	1581.7	1018	4.4079377	1	Singlet	Immune_cells
SAT_26M	1769.4	1132	13.82525	4	Singlet	Immune_cells
SAT_26M	1846.7	1097	4.9810676	7	Singlet	Endothelial_cells
SAT_26M	1075.8	748	3.8613674	5	Singlet	Immune_cells
SAT_26M	1698.6	1066	6.3593239	8	Singlet	Mesothelial
SAT_26M	1247.1	900	9.8931854	5	Singlet	Immune_cells
SAT_26M	1119.5	688	0.5854077	0	Singlet	Adipocyte
SAT_26M	1475.8	923	2.1270927	7	Singlet	Endothelial_cells
SAT_26M	1017.8	695	8.5415687	1	Singlet	Immune_cells
SAT_26M	1881.5	1041	1.0468582	0	Singlet	Adipocyte
SAT_26M	10191	3408	0.4024106	0	Singlet	Adipocyte
SAT_26M	1777.6	1156	1.1787723	1	Singlet	Immune_cells
SAT_26M	3105.8	1889	2.4280861	8	Singlet	Mesothelial
SAT_26M	1503.1	1058	0.230429	11	Singlet	Smooth_muscle_cells

SAT_26M	1592.7	964	14.469926	10	Singlet	Immune_cells
SAT_26M	1881.9	1153	0.60744	0	Singlet	Adipocyte
SAT_26M	1122.2	708	10.392882	1	Singlet	Immune_cells
SAT_26M	1342.7	832	12.003124	8	Singlet	Mesothelial
SAT_26M	1567.9	679	0.1406925	10	Singlet	Immune_cells
SAT_26M	1921	1071	1.8536191	0	Singlet	Adipocyte
SAT_26M	1272.1	739	7.6985388	0	Singlet	Adipocyte
SAT_26M	1062.7	683	1.3141393	0	Singlet	Adipocyte
SAT_26M	1209	830	4.6108265	1	Singlet	Immune_cells
SAT_26M	2021.6	1251	0	8	Singlet	Mesothelial
SAT_26M	10776	4305	12.053571	9	Singlet	Mesothelial
SAT_26M	1037	731	6.9118934	4	Singlet	Immune_cells
SAT_26M	3014.2	1689	3.0641428	8	Singlet	Mesothelial
SAT_26M	1082.9	709	7.2699283	1	Singlet	Immune_cells
SAT_26M	1113.7	725	8.8077719	7	Singlet	Endothelial_cells
SAT_26M	1655	990	6.8094332	3	Singlet	FAPs
SAT_26M	1266.7	1024	0.0913109	5	Singlet	Immune_cells
SAT_26M	1663.6	1036	3.6766004	7	Singlet	Endothelial_cells
SAT_26M	1438.9	1091	4.5792833	4	Singlet	Immune_cells
SAT_26M	1500.4	932	3.0427406	1	Singlet	Immune_cells
SAT_26M	1174.2	770	4.5179249	1	Singlet	Immune_cells
SAT_26M	8079.1	2234	1.3420943	10	Singlet	Immune_cells
SAT_26M	1405.6	757	13.640217	0	Singlet	Adipocyte
SAT_26M	5281.2	2127	1.3327857	8	Singlet	Mesothelial
SAT_26M	2607.3	1486	11.069078	1	Singlet	Immune_cells
SAT_26M	1074.9	623	8.8238142	1	Singlet	Immune_cells
SAT_26M	1768.8	1066	4.2434766	1	Singlet	Immune_cells
SAT_26M	1445.8	933	0.4595405	8	Singlet	Mesothelial
SAT_26M	1528.2	949	7.1235943	1	Singlet	Immune_cells
SAT_26M	1135.5	679	0.0878418	4	Singlet	Immune_cells
SAT_26M	2049.6	984	10.547496	4	Singlet	Immune_cells
SAT_26M	3346.2	1559	1.8328078	8	Singlet	Mesothelial
SAT_26M	1956.7	1076	14.335815	4	Singlet	Immune_cells
SAT_26M	2357.3	1086	0.2804851	10	Singlet	Immune_cells
SAT_26M	2578.6	1462	0.9772811	0	Singlet	Adipocyte
SAT_26M	1171	675	0	0	Singlet	Adipocyte
SAT_26M	1371.8	794	1.1165656	10	Singlet	Immune_cells
SAT_26M	1057.7	577	9.7143893	10	Singlet	Immune_cells
SAT_26M	1666.1	889	0.1033494	10	Singlet	Immune_cells
SAT_26M	1854.2	1188	1.1053973	1	Singlet	Immune_cells
SAT_26M	1038.7	731	6.8520277	5	Singlet	Immune_cells
SAT_26M	1633.5	984	1.1713766	0	Singlet	Adipocyte
SAT_26M	1620.9	976	0.0589746	0	Singlet	Adipocyte
SAT_26M	1242.9	794	0.0179254	0	Singlet	Adipocyte
SAT_26M	2818.9	1518	0.0423619	0	Singlet	Adipocyte
SAT_26M	1126.4	741	11.998969	4	Singlet	Immune_cells
SAT_26M	1117	745	3.6383833	1	Singlet	Immune_cells
SAT_26M	1143	769	1.0426284	8	Singlet	Mesothelial
SAT_26M	1761.5	1066	4.4849353	1	Singlet	Immune_cells
SAT_26M	1625	1043	0.0636204	0	Singlet	Adipocyte
SAT_26M	1245.1	746	8.1002612	10	Singlet	Immune_cells

SAT_26M	1903.6	1195	5.0065314	1	Singlet	Immune_cells
SAT_26M	3642.3	2146	3.6512209	9	Singlet	Mesothelial
SAT_26M	1357.5	885	0.2378176	0	Singlet	Adipocyte
SAT_26M	1036.7	860	2.6952778	5	Singlet	Immune_cells
SAT_26M	4591.4	1987	0.6774993	0	Singlet	Adipocyte
SAT_26M	2035.7	952	2.236026	10	Singlet	Immune_cells
SAT_26M	1366.7	866	6.7896892	0	Singlet	Adipocyte
SAT_26M	1295.4	887	2.6858814	1	Singlet	Immune_cells
SAT_26M	1665.3	1046	13.013154	1	Singlet	Immune_cells
SAT_26M	1332.4	938	2.1613504	1	Singlet	Immune_cells
SAT_26M	1105.5	689	4.9217948	0	Singlet	Adipocyte
SAT_26M	1640.2	1028	2.3706952	1	Singlet	Immune_cells
SAT_26M	1181.8	749	3.0390489	0	Singlet	Adipocyte
SAT_26M	1676.7	1067	2.1785368	1	Singlet	Immune_cells
SAT_26M	1630.8	1192	5.3779557	9	Singlet	Mesothelial
SAT_26M	1459.2	892	0.1170474	0	Singlet	Adipocyte
SAT_26M	1840.8	1047	0.0662884	0	Singlet	Adipocyte
SAT_26M	4442.3	2135	1.0306741	0	Singlet	Adipocyte
SAT_26M	1478.2	941	5.6620436	1	Singlet	Immune_cells
SAT_26M	1032.5	665	12.887849	4	Singlet	Immune_cells
SAT_26M	1678.8	1008	4.2874018	7	Singlet	Endothelial_cells
SAT_26M	1544.7	986	1.7689236	7	Singlet	Endothelial_cells
SAT_26M	1493.2	967	4.2608406	1	Singlet	Immune_cells
SAT_26M	3100.4	1664	0.0774747	0	Singlet	Adipocyte
SAT_26M	2068.2	1199	6.5093766	4	Singlet	Immune_cells
SAT_26M	1002	609	4.5756309	3	Singlet	FAPs
SAT_26M	1277.9	812	13.386548	4	Singlet	Immune_cells
SAT_26M	1035	723	2.36781	7	Singlet	Endothelial_cells
SAT_26M	3452.3	1523	1.9104976	10	Singlet	Immune_cells
SAT_26M	1464.2	967	2.8729917	1	Singlet	Immune_cells
SAT_26M	1185.4	880	1.1616944	3	Singlet	FAPs
SAT_26M	2440.9	1313	2.6624402	10	Singlet	Immune_cells
SAT_26M	1197.4	784	0.3522264	3	Singlet	FAPs
SAT_26M	1198.9	795	2.0698094	3	Singlet	FAPs
SAT_26M	1621.5	911	8.0633503	5	Singlet	Immune_cells
SAT_26M	1527.3	964	2.6689902	3	Singlet	FAPs
SAT_26M	1150.3	732	3.9103535	7	Singlet	Endothelial_cells
SAT_26M	1052	791	7.2072517	6	Singlet	Neurons
SAT_26M	1043.1	752	7.5486531	11	Singlet	Smooth_muscle_cells
SAT_26M	2475.4	1349	2.7184159	7	Singlet	Endothelial_cells
SAT_26M	1102.2	763	0.1583095	8	Singlet	Mesothelial
SAT_26M	1625.1	1052	3.8811143	4	Singlet	Immune_cells
SAT_26M	1052.9	705	3.2935191	0	Singlet	Adipocyte
SAT_26M	2845.4	1588	1.1003881	0	Singlet	Adipocyte
SAT_26M	1499.5	787	4.1432811	10	Singlet	Immune_cells
SAT_26M	1414.3	903	1.3034132	1	Singlet	Immune_cells
SAT_26M	1986.2	1342	1.1417726	8	Singlet	Mesothelial
SAT_26M	1509.9	973	6.1955622	1	Singlet	Immune_cells
SAT_26M	1060.5	801	6.719468	8	Singlet	Mesothelial
SAT_26M	1068.7	727	4.4229288	0	Singlet	Adipocyte
SAT_26M	2167.1	1189	0.4455934	0	Singlet	Adipocyte

SAT_26M	1082.3	758	2.090375	1	Singlet	Immune_cells
SAT_26M	1420.3	889	3.9869843	1	Singlet	Immune_cells
SAT_26M	1214	980	0.5978955	6	Singlet	Neurons
SAT_26M	4281.1	1841	3.6695141	0	Singlet	Adipocyte
SAT_26M	1164.6	687	8.0000944	0	Singlet	Adipocyte
SAT_26M	1288.9	887	6.1017002	4	Singlet	Immune_cells
SAT_26M	1750.1	1007	0.0159496	0	Singlet	Adipocyte
SAT_26M	2443	1255	4.8030436	0	Singlet	Adipocyte
SAT_26M	1010.5	725	0.0532667	6	Singlet	Neurons
SAT_26M	1339.6	748	0.2496987	8	Singlet	Mesothelial
SAT_26M	1321.5	856	6.3518463	1	Singlet	Immune_cells
SAT_26M	1410.5	743	0.3675276	0	Singlet	Adipocyte
SAT_26M	1098.9	811	11.418951	6	Singlet	Neurons
SAT_26M	1033.7	706	4.4787014	1	Singlet	Immune_cells
SAT_26M	1161	918	1.5618908	6	Singlet	Neurons
SAT_26M	1048.7	625	4.6204829	1	Singlet	Immune_cells
SAT_26M	2496.6	1496	5.5735959	7	Singlet	Endothelial_cells
SAT_26M	1407	862	11.351202	1	Singlet	Immune_cells
SAT_26M	1244.2	702	0.1091677	0	Singlet	Adipocyte
SAT_26M	3451.7	1654	0.402717	0	Singlet	Adipocyte
SAT_26M	1111	689	0.3509922	0	Singlet	Adipocyte
SAT_26M	1197.7	750	9.75481	1	Singlet	Immune_cells
SAT_26M	1865.6	907	0.0201873	0	Singlet	Adipocyte
SAT_26M	1792.5	1022	3.0436449	10	Singlet	Immune_cells
SAT_26M	1356.6	917	4.8353874	4	Singlet	Immune_cells
SAT_26M	3625.3	1769	2.3585845	0	Singlet	Adipocyte
SAT_26M	2797.7	1504	0.0631434	0	Singlet	Adipocyte
SAT_26M	1052.8	677	0.0967392	0	Singlet	Adipocyte
SAT_26M	1505.4	967	5.6940869	1	Singlet	Immune_cells
SAT_26M	1068.9	702	8.3090305	1	Singlet	Immune_cells
SAT_26M	3465.5	1530	2.8242189	10	Singlet	Immune_cells
SAT_26M	1065.4	730	0.0519269	0	Singlet	Adipocyte
SAT_26M	1359.1	869	0.3293565	1	Singlet	Immune_cells
SAT_26M	1574.3	996	1.9616704	9	Singlet	Mesothelial
SAT_26M	1017.9	675	6.8372641	1	Singlet	Immune_cells
SAT_26M	1660.7	1061	0.1855176	0	Singlet	Adipocyte
SAT_26M	1335.3	1038	0	4	Singlet	Immune_cells
SAT_26M	1817	1041	2.7738998	0	Singlet	Adipocyte
SAT_26M	1009.3	623	10.475792	7	Singlet	Endothelial_cells
SAT_26M	2473.7	1467	3.9400955	8	Singlet	Mesothelial
SAT_26M	1360.6	895	3.2736154	1	Singlet	Immune_cells
SAT_26M	1134	709	7.6328015	10	Singlet	Immune_cells
SAT_26M	2507.7	1581	7.123931	4	Singlet	Immune_cells
SAT_26M	2005.7	1200	0.815257	1	Singlet	Immune_cells
SAT_26M	3208.7	1702	14.770063	4	Singlet	Immune_cells
SAT_26M	1251.8	783	2.9665468	0	Singlet	Adipocyte
SAT_26M	5273.3	2214	3.0221596	0	Singlet	Adipocyte
SAT_26M	6282.4	1924	1.4571103	10	Singlet	Immune_cells
SAT_26M	3393.1	1342	2.7838765	10	Singlet	Immune_cells
SAT_26M	1075.5	772	2.2332534	11	Singlet	Smooth_muscle_cells
SAT_26M	1136.6	763	5.0516573	1	Singlet	Immune_cells

SAT_26M	3815.6	1672	1.6875787	10	Singlet	Immune_cells
SAT_26M	1301.4	865	0	8	Singlet	Mesothelial
SAT_26M	1088.1	730	5.3386073	8	Singlet	Mesothelial
SAT_26M	3328.9	1973	1.2785193	8	Singlet	Mesothelial
SAT_26M	2383.7	1406	0.0156369	0	Singlet	Adipocyte
SAT_26M	1342.6	888	3.5843841	3	Singlet	FAPs
SAT_26M	2242.3	1302	6.8679137	9	Singlet	Mesothelial
SAT_26M	1345.8	859	1.9423975	5	Singlet	Immune_cells
SAT_26M	1677.3	931	5.7829476	0	Singlet	Adipocyte
SAT_26M	1435.1	1014	3.4190382	1	Singlet	Immune_cells
SAT_26M	1361.5	886	2.0122533	1	Singlet	Immune_cells
SAT_26M	2619.4	1461	3.7201148	1	Singlet	Immune_cells
SAT_26M	1787.7	1174	8.5247606	1	Singlet	Immune_cells
SAT_26M	1134.2	907	0.2887206	6	Singlet	Neurons
SAT_26M	1658.6	965	0.0424943	0	Singlet	Adipocyte
SAT_26M	1042.4	856	1.020105	6	Singlet	Neurons
SAT_26M	1013.5	622	13.518621	4	Singlet	Immune_cells
SAT_26M	7055.8	2765	4.1889674	2	Singlet	FAPs
SAT_26M	1069.1	730	2.9511005	9	Singlet	Mesothelial
SAT_26M	2692.8	1554	4.0221215	1	Singlet	Immune_cells
SAT_26M	1047.7	735	8.5407177	1	Singlet	Immune_cells
SAT_26M	1103.7	603	5.2414587	10	Singlet	Immune_cells
SAT_26M	1105.5	746	3.1437933	1	Singlet	Immune_cells
SAT_26M	1004.3	802	0.0445597	6	Singlet	Neurons
SAT_26M	1329.5	1091	6.4469656	6	Singlet	Neurons
SAT_26M	1242	751	4.8706406	5	Singlet	Immune_cells
SAT_26M	3292.9	1706	1.8585468	0	Singlet	Adipocyte
SAT_26M	1394.5	935	8.2190048	1	Singlet	Immune_cells
SAT_26M	1109	751	0.0863705	8	Singlet	Mesothelial
SAT_26M	1389	863	11.184981	5	Singlet	Immune_cells
SAT_26M	1330.2	879	9.9382989	5	Singlet	Immune_cells
SAT_26M	2683.5	1298	13.051559	0	Singlet	Adipocyte
SAT_26M	2022.2	1147	10.581352	4	Singlet	Immune_cells
SAT_26M	1165.9	769	4.5795628	0	Singlet	Adipocyte
SAT_26M	9252.5	3769	14.297354	9	Singlet	Mesothelial
SAT_26M	1851.6	1079	0.0568027	0	Singlet	Adipocyte
SAT_26M	3827	1822	0.3756555	0	Singlet	Adipocyte
SAT_26M	1518.4	845	10.084967	0	Singlet	Adipocyte
SAT_26M	1050.7	892	0.4160657	6	Singlet	Neurons
SAT_26M	1088.3	664	6.2116545	0	Singlet	Adipocyte
SAT_26M	3027.9	1514	13.290854	9	Singlet	Mesothelial
SAT_26M	1976.6	1235	1.866974	7	Singlet	Endothelial_cells
SAT_26M	2058.4	1199	12.696883	4	Singlet	Immune_cells
SAT_26M	1640.9	901	0.1374146	0	Singlet	Adipocyte
SAT_26M	1036.7	743	5.9713035	4	Singlet	Immune_cells
SAT_26M	2740.2	1622	10.384471	4	Singlet	Immune_cells
SAT_26M	1010.1	676	4.4150306	8	Singlet	Mesothelial
SAT_26M	1971.1	1061	7.56825	0	Singlet	Adipocyte
SAT_26M	2143.6	1228	0.9960125	0	Singlet	Adipocyte
SAT_26M	1119.1	706	0	0	Singlet	Adipocyte
SAT_26M	1330.1	898	2.4460098	1	Singlet	Immune_cells

SAT_26M	2045.4	1257	3.4209746	1	Singlet	Immune_cells
SAT_26M	1434.3	909	1.6214443	1	Singlet	Immune_cells
SAT_26M	2798.7	1611	3.2056782	1	Singlet	Immune_cells
SAT_26M	2335.5	1044	5.0637843	10	Singlet	Immune_cells
SAT_26M	1133.5	771	5.4231554	1	Singlet	Immune_cells
SAT_26M	1895	1071	0.8112778	0	Singlet	Adipocyte
SAT_26M	2114.9	1204	0.2903548	0	Singlet	Adipocyte
SAT_26M	2702.7	1457	0.238398	0	Singlet	Adipocyte
SAT_26M	2042.6	1230	8.041954	9	Singlet	Mesothelial
SAT_26M	3442.1	1701	3.0216139	0	Singlet	Adipocyte
SAT_26M	1226.7	814	3.6520673	1	Singlet	Immune_cells
SAT_26M	1441.9	861	1.0064865	7	Singlet	Endothelial_cells
SAT_26M	1781.5	1078	1.0912283	0	Singlet	Adipocyte
SAT_26M	1392.2	871	2.8593976	1	Singlet	Immune_cells
SAT_26M	1923.8	1132	0.1905295	0	Singlet	Adipocyte
SAT_26M	1011.2	719	13.138853	4	Singlet	Immune_cells
SAT_26M	3790.4	1948	0.2209978	0	Singlet	Adipocyte
SAT_26M	1405.2	943	4.9674232	8	Singlet	Mesothelial
SAT_26M	1010.4	704	0.4953026	3	Singlet	FAPs
SAT_26M	1099.8	720	1.0973639	0	Singlet	Adipocyte
SAT_26M	2031.7	1139	1.3276811	2	Singlet	FAPs
SAT_26M	2017.8	1105	0.1419194	0	Singlet	Adipocyte
SAT_26M	1075.7	667	7.2139522	8	Singlet	Mesothelial
SAT_26M	1053.6	742	0.5980496	1	Singlet	Immune_cells
SAT_26M	1375.6	903	2.0724027	0	Singlet	Adipocyte
SAT_26M	2231.1	1353	3.4371152	1	Singlet	Immune_cells
SAT_26M	1587	978	2.7278901	0	Singlet	Adipocyte
SAT_26M	2422.1	1210	1.0543849	2	Singlet	FAPs
SAT_26M	1008.1	642	5.8078376	0	Singlet	Adipocyte
SAT_26M	1420.6	733	8.0191645	10	Singlet	Immune_cells
SAT_26M	1151.9	746	1.0581831	0	Singlet	Adipocyte
SAT_26M	1609.6	1014	2.8663992	1	Singlet	Immune_cells
SAT_26M	1410.7	916	1.3496401	5	Singlet	Immune_cells
SAT_26M	1417.5	905	1.882989	0	Singlet	Adipocyte
SAT_26M	3498.5	1851	9.7713246	9	Singlet	Mesothelial
SAT_26M	2567.6	1464	4.1359815	8	Singlet	Mesothelial
SAT_26M	2085.5	1020	5.7965055	10	Singlet	Immune_cells
SAT_26M	1365.5	909	1.301797	1	Singlet	Immune_cells
SAT_26M	3029.7	1985	7.660745	8	Singlet	Mesothelial
SAT_26M	2565.3	1368	0	0	Singlet	Adipocyte
SAT_26M	1818	1149	2.8364869	1	Singlet	Immune_cells
SAT_26M	9504.1	3869	7.7097954	9	Singlet	Mesothelial
SAT_26M	1234.9	990	1.1559628	6	Singlet	Neurons
SAT_26M	1089.1	791	0.0394874	11	Singlet	Smooth_muscle_cells
SAT_26M	1227.2	823	0.0564511	0	Singlet	Adipocyte
SAT_26M	1100.9	788	2.4185078	11	Singlet	Smooth_muscle_cells
SAT_26M	3483.2	1658	3.1126416	10	Singlet	Immune_cells
SAT_26M	1129.4	824	2.0839628	11	Singlet	Smooth_muscle_cells
SAT_26M	3377	1958	5.1349311	8	Singlet	Mesothelial
SAT_26M	1154.8	790	11.326458	1	Singlet	Immune_cells
SAT_26M	1136.1	806	1.0236126	11	Singlet	Smooth_muscle_cells

SAT_26M	1414.1	900	1.4727798	0	Singlet	Adipocyte
SAT_26M	2074.9	1206	4.5991696	8	Singlet	Mesothelial
SAT_26M	1187.8	819	0.260933	11	Singlet	Smooth_muscle_cells
SAT_26M	1149.7	747	3.08129	0	Singlet	Adipocyte
SAT_26M	1251.8	899	1.9287477	1	Singlet	Immune_cells
SAT_26M	1325.8	922	7.9374282	8	Singlet	Mesothelial
SAT_26M	2874.9	1465	0.891463	0	Singlet	Adipocyte
SAT_26M	1019	681	5.3648931	7	Singlet	Endothelial_cells
SAT_26M	1041.4	541	4.3703193	10	Singlet	Immune_cells
SAT_26M	2110.7	1301	1.1265726	7	Singlet	Endothelial_cells
SAT_26M	1614.8	1007	0.7626845	0	Singlet	Adipocyte
SAT_26M	1123.5	707	2.0066554	10	Singlet	Immune_cells
SAT_26M	2523.7	1444	1.8497302	1	Singlet	Immune_cells
SAT_26M	1794.1	946	0.7879434	0	Singlet	Adipocyte
SAT_26M	2648	1351	8.0651144	0	Singlet	Adipocyte
SAT_26M	1089.5	686	0.4224508	0	Singlet	Adipocyte
SAT_26M	3683.6	1749	0.0761395	0	Singlet	Adipocyte
SAT_26M	1688.1	1005	0	0	Singlet	Adipocyte
SAT_26M	4649.4	2193	0.5269865	0	Singlet	Adipocyte
SAT_26M	1659.8	1132	2.4988017	8	Singlet	Mesothelial
SAT_26M	1729.7	1062	5.2587575	1	Singlet	Immune_cells
SAT_26M	2349.7	1273	2.3282181	0	Singlet	Adipocyte
SAT_26M	1340.2	841	1.7792953	0	Singlet	Adipocyte
SAT_26M	1310.3	953	4.2101566	1	Singlet	Immune_cells
SAT_26M	2942	1574	0.7964768	0	Singlet	Adipocyte
SAT_26M	1349	931	0.6920804	4	Singlet	Immune_cells
SAT_26M	1626.3	972	2.2878938	10	Singlet	Immune_cells
SAT_26M	7151.6	3242	6.0688436	8	Singlet	Mesothelial
SAT_26M	1078	763	1.5591419	0	Singlet	Adipocyte
SAT_26M	2422.3	1106	7.1363327	10	Singlet	Immune_cells
SAT_26M	1813.7	1115	4.9363316	0	Singlet	Adipocyte
SAT_26M	1913	1224	4.0196992	1	Singlet	Immune_cells
SAT_26M	1454.2	944	0.7257741	0	Singlet	Adipocyte
SAT_26M	1200.7	743	14.011911	1	Singlet	Immune_cells
SAT_26M	1066.2	739	3.2864882	1	Singlet	Immune_cells
SAT_26M	1560.8	957	1.0185478	0	Singlet	Adipocyte
SAT_26M	1276.6	805	0.0478328	0	Singlet	Adipocyte
SAT_26M	2142	1186	3.9671135	0	Singlet	Adipocyte
SAT_26M	1051.1	769	0.7786413	0	Singlet	Adipocyte
SAT_26M	1344	771	1.8105909	7	Singlet	Endothelial_cells
SAT_26M	1558.2	968	4.6043095	1	Singlet	Immune_cells
SAT_26M	2252.3	1319	5.4376832	8	Singlet	Mesothelial
SAT_26M	1248.7	788	0.1817847	0	Singlet	Adipocyte
SAT_26M	1298.4	963	1.3551988	3	Singlet	FAPs
SAT_26M	1169.4	844	4.4524311	1	Singlet	Immune_cells
SAT_26M	5599.7	2355	0.8594222	2	Singlet	FAPs
SAT_26M	1219.6	800	0.5037686	0	Singlet	Adipocyte
SAT_26M	2232.8	1279	5.45221	0	Singlet	Adipocyte
SAT_26M	1282.8	837	4.3015216	0	Singlet	Adipocyte
SAT_26M	3278.9	1856	2.8565303	8	Singlet	Mesothelial
SAT_26M	1808.3	1000	12.096041	4	Singlet	Immune_cells

SAT_26M	1466.6	980	4.0444919	1	Singlet	Immune_cells
SAT_26M	1803.2	1073	1.7434999	0	Singlet	Adipocyte
SAT_26M	1477.4	1078	2.4582285	4	Singlet	Immune_cells
SAT_26M	1465.6	958	0.896684	0	Singlet	Adipocyte
SAT_26M	1247.4	867	1.3791987	10	Singlet	Immune_cells
SAT_26M	1033.5	724	0.032258	4	Singlet	Immune_cells
SAT_26M	1146.2	718	14.140924	1	Singlet	Immune_cells
SAT_26M	1234.2	895	0.8652605	8	Singlet	Mesothelial
SAT_26M	1261.4	824	7.5184463	1	Singlet	Immune_cells
SAT_26M	4501.7	1547	2.8126765	10	Singlet	Immune_cells
SAT_26M	1691.5	1007	12.358824	4	Singlet	Immune_cells
SAT_26M	1276.8	786	0.7780214	0	Singlet	Adipocyte
SAT_26M	1494.7	981	3.7374401	1	Singlet	Immune_cells
SAT_26M	1316.1	827	1.7103478	3	Singlet	FAPs
SAT_26M	1224.5	829	6.3105989	1	Singlet	Immune_cells
SAT_26M	1376	924	0.6075477	11	Singlet	Smooth_muscle_cells
SAT_26M	1286.4	833	4.3737171	7	Singlet	Endothelial_cells
SAT_26M	1044.2	741	0	8	Singlet	Mesothelial
SAT_26M	2074.1	1227	0.6950724	0	Singlet	Adipocyte
SAT_26M	5139	2584	2.9502462	9	Singlet	Mesothelial
SAT_26M	1483.3	921	4.6316305	1	Singlet	Immune_cells
SAT_26M	1155.9	776	6.4100466	7	Singlet	Endothelial_cells
SAT_26M	1007	661	13.22398	4	Singlet	Immune_cells
SAT_26M	1649.5	1038	2.8991158	1	Singlet	Immune_cells
SAT_26M	1740	1119	3.6030088	1	Singlet	Immune_cells
SAT_26M	4969.1	2639	1.4491663	8	Singlet	Mesothelial
SAT_26M	2116.6	1435	11.962989	9	Singlet	Mesothelial
SAT_26M	1802.3	1091	0.0298816	0	Singlet	Adipocyte
SAT_26M	1005.5	663	0.076183	4	Singlet	Immune_cells
SAT_26M	1354.2	747	1.4209404	10	Singlet	Immune_cells
SAT_26M	1832.6	1032	9.149195	0	Singlet	Adipocyte
SAT_26M	1036	647	2.6280152	3	Singlet	FAPs
SAT_26M	1919.3	1156	0.1232774	8	Singlet	Mesothelial
SAT_26M	2997.6	1579	2.0663523	0	Singlet	Adipocyte
SAT_26M	1013.8	700	1.7810183	0	Singlet	Adipocyte
SAT_26M	1306.3	979	13.297972	1	Singlet	Immune_cells
SAT_26M	3185.4	1763	3.5118147	8	Singlet	Mesothelial
SAT_26M	1514.7	999	3.2049957	1	Singlet	Immune_cells
SAT_26M	1573.1	925	7.838329	7	Singlet	Endothelial_cells
SAT_26M	2646.8	1430	9.646637	4	Singlet	Immune_cells
SAT_26M	1816.1	1084	8.2770932	4	Singlet	Immune_cells
SAT_26M	1786.3	998	0.1189185	0	Singlet	Adipocyte
SAT_26M	1952.9	1093	1.5254053	0	Singlet	Adipocyte
SAT_26M	2119.9	1225	0.0673858	0	Singlet	Adipocyte
SAT_26M	1364.2	946	1.0377351	11	Singlet	Smooth_muscle_cells
SAT_26M	1272.1	882	0	8	Singlet	Mesothelial
SAT_26M	1712.1	996	0.6568736	0	Singlet	Adipocyte
SAT_26M	2312.6	1466	2.6283501	8	Singlet	Mesothelial
SAT_26M	1830.3	1129	2.1644383	1	Singlet	Immune_cells
SAT_26M	1711.8	1101	5.8007942	1	Singlet	Immune_cells
SAT_26M	1291.4	869	2.9280847	1	Singlet	Immune_cells

SAT_26M	1539.2	951	1.8826029	0	Singlet	Adipocyte
SAT_26M	3741.5	1476	0.8138455	10	Singlet	Immune_cells
SAT_26M	10885	4438	7.1182275	9	Singlet	Mesothelial
SAT_26M	1186	770	1.2503843	0	Singlet	Adipocyte
SAT_26M	1330.5	857	0	8	Singlet	Mesothelial
SAT_26M	1341.6	836	5.1063362	7	Singlet	Endothelial_cells
SAT_26M	1090.1	701	8.6150874	1	Singlet	Immune_cells
SAT_26M	4658.1	2028	0.8239613	0	Singlet	Adipocyte
SAT_26M	1034.5	737	0.487955	3	Singlet	FAPs
SAT_26M	4268.6	2580	7.4210298	9	Singlet	Mesothelial
SAT_26M	3744.4	1949	1.4988295	0	Singlet	Adipocyte
SAT_26M	1447.3	953	2.2935721	1	Singlet	Immune_cells
SAT_26M	1196.1	770	1.1135806	0	Singlet	Adipocyte
SAT_26M	1681.7	1078	9.2825802	0	Singlet	Adipocyte
SAT_26M	3379.5	2048	4.5233728	9	Singlet	Mesothelial
SAT_26M	1026.4	688	0.137716	0	Singlet	Adipocyte
SAT_26M	1122.6	652	0	0	Singlet	Adipocyte
SAT_26M	2731.5	1430	1.80993	10	Singlet	Immune_cells
SAT_26M	1374.4	931	4.0166816	1	Singlet	Immune_cells
SAT_26M	2262	1362	2.4455307	8	Singlet	Mesothelial
SAT_26M	1132.7	819	1.2319441	1	Singlet	Immune_cells
SAT_26M	1068.7	658	1.3635296	2	Singlet	FAPs
SAT_26M	1406.7	951	5.3174702	1	Singlet	Immune_cells
SAT_26M	1411.2	897	5.1653247	1	Singlet	Immune_cells
SAT_26M	1687.8	987	1.9260339	2	Singlet	FAPs
SAT_26M	1424.1	950	0.9230672	1	Singlet	Immune_cells
SAT_26M	1617.5	1329	2.6739939	6	Singlet	Neurons
SAT_26M	1252.8	844	0.046753	4	Singlet	Immune_cells
SAT_26M	3265.3	1640	0.0048684	0	Singlet	Adipocyte
SAT_26M	1914.6	1180	1.4910446	1	Singlet	Immune_cells
SAT_26M	1072.4	660	0.9815459	0	Singlet	Adipocyte
SAT_26M	1435.1	903	4.5463319	1	Singlet	Immune_cells
SAT_26M	1310	998	4.8186156	4	Singlet	Immune_cells
SAT_26M	2094.6	1043	7.5996129	0	Singlet	Adipocyte
SAT_26M	1597.6	1085	4.327264	9	Singlet	Mesothelial
SAT_26M	3539	1492	1.9093458	10	Singlet	Immune_cells
SAT_26M	3417.1	1874	0	8	Singlet	Mesothelial
SAT_26M	1219.5	826	0.5462691	7	Singlet	Endothelial_cells
SAT_26M	2631.1	1432	3.0068346	0	Singlet	Adipocyte
SAT_26M	4232.5	2031	0.2052059	0	Singlet	Adipocyte
SAT_26M	3006.5	1564	0.0074207	0	Singlet	Adipocyte
SAT_26M	1679.3	1142	1.431596	1	Singlet	Immune_cells
SAT_26M	4225.4	1689	0.7720237	10	Singlet	Immune_cells
SAT_26M	1037.7	705	6.8480551	1	Singlet	Immune_cells
SAT_26M	1791.5	1105	3.344446	0	Singlet	Adipocyte
SAT_26M	1093.3	745	6.5609534	1	Singlet	Immune_cells
SAT_26M	1300.6	876	6.3636186	1	Singlet	Immune_cells
SAT_26M	1054.4	731	2.8190204	1	Singlet	Immune_cells
SAT_26M	1336.4	875	7.2143275	1	Singlet	Immune_cells
SAT_26M	2182.5	1295	0.1697615	0	Singlet	Adipocyte
SAT_26M	1156.9	700	9.6867242	0	Singlet	Adipocyte

SAT_26M	1552.9	1009	0.5844286	5	Singlet	Immune_cells
SAT_26M	1096.8	703	2.5064477	0	Singlet	Adipocyte
SAT_26M	1118.2	920	1.6056303	6	Singlet	Neurons
SAT_26M	1646.1	1137	2.3510955	0	Singlet	Adipocyte
SAT_26M	1882.5	1043	4.6132497	0	Singlet	Adipocyte
SAT_26M	1039	685	7.1042787	1	Singlet	Immune_cells
SAT_26M	1741.8	1013	3.3084894	10	Singlet	Immune_cells
SAT_26M	1200.4	880	0.4146499	3	Singlet	FAPs
SAT_26M	1109	918	0.4486531	6	Singlet	Neurons
SAT_26M	2352.6	1401	0.064837	0	Singlet	Adipocyte
SAT_26M	1111.4	755	4.1265517	1	Singlet	Immune_cells
SAT_26M	2265.7	1313	0.1263142	0	Singlet	Adipocyte
SAT_26M	1072.2	698	0.455692	0	Singlet	Adipocyte
SAT_26M	1881.2	1172	4.5443236	8	Singlet	Mesothelial
SAT_26M	1826.1	1113	2.281962	1	Singlet	Immune_cells
SAT_26M	1293.4	955	1.191728	1	Singlet	Immune_cells
SAT_26M	1012.5	635	4.2310462	1	Singlet	Immune_cells
SAT_26M	1155.3	772	9.0496446	4	Singlet	Immune_cells
SAT_26M	1193.8	771	5.2649605	1	Singlet	Immune_cells
SAT_26M	1388.7	779	4.8330064	10	Singlet	Immune_cells
SAT_26M	1104.4	753	4.4330337	1	Singlet	Immune_cells
SAT_26M	2386.8	1341	11.146509	7	Singlet	Endothelial_cells
SAT_26M	3628.1	1763	0.1955471	0	Singlet	Adipocyte
SAT_26M	1572.2	903	1.1388975	0	Singlet	Adipocyte
SAT_26M	1953.1	1244	5.5306218	8	Singlet	Mesothelial
SAT_26M	3634.2	2069	4.593731	8	Singlet	Mesothelial
SAT_26M	1748.7	1158	2.0050272	8	Singlet	Mesothelial
SAT_26M	2547.1	1399	0.2212879	0	Singlet	Adipocyte
SAT_26M	2126.6	1330	3.9996532	1	Singlet	Immune_cells
SAT_26M	1026.8	667	0	0	Singlet	Adipocyte
SAT_26M	1132.6	752	0.5906013	0	Singlet	Adipocyte
SAT_26M	1213.7	845	4.9275567	1	Singlet	Immune_cells
SAT_26M	1119.2	798	0.0660876	8	Singlet	Mesothelial
SAT_26M	5819.3	2639	13.128182	9	Singlet	Mesothelial
SAT_26M	1156.3	882	0	6	Singlet	Neurons
SAT_26M	1065.5	687	3.2672797	10	Singlet	Immune_cells
SAT_26M	2461.3	1347	0.6245877	0	Singlet	Adipocyte
SAT_26M	1157.8	786	0.3522873	11	Singlet	Smooth_muscle_cells
SAT_26M	1016.2	671	5.9418407	3	Singlet	FAPs
SAT_26M	4603.4	2181	1.0989824	1	Singlet	Immune_cells
SAT_26M	1921.9	1133	0.76695	0	Singlet	Adipocyte
SAT_26M	1093.4	777	4.6682925	1	Singlet	Immune_cells
SAT_26M	1406.6	944	0.9977076	8	Singlet	Mesothelial
SAT_26M	1055.7	666	0.1653664	0	Singlet	Adipocyte
SAT_26M	3113.2	1526	1.9457081	0	Singlet	Adipocyte
SAT_26M	2441	1437	7.873761	8	Singlet	Mesothelial
SAT_26M	1207.3	759	0.3027908	0	Singlet	Adipocyte
SAT_26M	1783.3	1068	0.7912698	0	Singlet	Adipocyte
SAT_26M	4244.7	1688	2.5734443	10	Singlet	Immune_cells
SAT_26M	1247.5	793	4.9062086	1	Singlet	Immune_cells
SAT_26M	2766.6	1675	2.6754819	8	Singlet	Mesothelial

SAT_26M	1789	1101	0.3204419	0	Singlet	Adipocyte
SAT_26M	1011.2	727	2.8027823	1	Singlet	Immune_cells
SAT_26M	2070.7	1221	4.3555101	1	Singlet	Immune_cells
SAT_26M	1237	826	2.3898652	5	Singlet	Immune_cells
SAT_26M	1693.9	969	5.9526118	0	Singlet	Adipocyte
SAT_26M	3434.7	1780	11.976982	9	Singlet	Mesothelial
SAT_26M	1304.5	891	1.8926699	1	Singlet	Immune_cells
SAT_26M	1423.9	839	12.432816	4	Singlet	Immune_cells
SAT_26M	1180.1	737	3.7517962	0	Singlet	Adipocyte
SAT_26M	1041.4	638	12.282193	1	Singlet	Immune_cells
SAT_26M	1407.7	901	2.3284005	5	Singlet	Immune_cells
SAT_26M	1561.3	1029	3.6849061	8	Singlet	Mesothelial
SAT_26M	1604.8	1092	5.7193018	5	Singlet	Immune_cells
SAT_26M	1201.5	795	0.1664294	0	Singlet	Adipocyte
SAT_26M	3518.4	1820	2.2715218	0	Singlet	Adipocyte
SAT_26M	1172.4	785	8.5595412	1	Singlet	Immune_cells
SAT_26M	2585.1	1372	2.6201976	0	Singlet	Adipocyte
SAT_26M	3824.3	2130	1.1954825	8	Singlet	Mesothelial
SAT_26M	1643.6	980	0.1239316	0	Singlet	Adipocyte
SAT_26M	1396.4	904	6.1307067	1	Singlet	Immune_cells
SAT_26M	1316.6	838	2.9447568	0	Singlet	Adipocyte
SAT_26M	4119.4	1938	1.0392159	0	Singlet	Adipocyte
SAT_26M	1371.3	769	1.2722959	10	Singlet	Immune_cells
SAT_26M	1064.5	678	11.881796	5	Singlet	Immune_cells
SAT_26M	1547.3	1021	5.2892054	1	Singlet	Immune_cells
SAT_26M	1134.6	748	1.2535004	0	Singlet	Adipocyte
SAT_26M	5057.3	2329	1.0390465	9	Singlet	Mesothelial
SAT_26M	1210.2	768	7.0840716	0	Singlet	Adipocyte
SAT_26M	1342.3	1050	1.3200303	4	Singlet	Immune_cells
SAT_26M	1522.8	927	0.3259545	0	Singlet	Adipocyte
SAT_26M	3049.7	1757	5.3713514	8	Singlet	Mesothelial
SAT_26M	3470.8	1655	2.4489889	0	Singlet	Adipocyte
SAT_26M	1011.4	699	5.6150884	8	Singlet	Mesothelial
SAT_26M	1041.9	678	13.871836	1	Singlet	Immune_cells
SAT_26M	3841.9	1851	1.0904717	10	Singlet	Immune_cells
SAT_26M	2495	1381	0.563566	0	Singlet	Adipocyte
SAT_26M	3844	2045	2.5923702	7	Singlet	Endothelial_cells
SAT_26M	4868.4	2089	4.0184346	0	Singlet	Adipocyte
SAT_26M	1552.4	981	0.6094793	1	Singlet	Immune_cells
SAT_26M	1162.2	876	5.5251427	9	Singlet	Mesothelial
SAT_26M	1034.3	702	7.3189425	5	Singlet	Immune_cells
SAT_26M	2550.9	1767	10.212437	9	Singlet	Mesothelial
SAT_26M	1418.1	963	2.1156479	8	Singlet	Mesothelial
SAT_26M	2039.9	1135	0.1542687	0	Singlet	Adipocyte
SAT_26M	1062.1	812	2.0905708	6	Singlet	Neurons
SAT_26M	1164.2	688	6.8153405	0	Singlet	Adipocyte
SAT_26M	1441.8	1000	4.9647476	1	Singlet	Immune_cells
SAT_26M	1010.7	759	9.4680784	6	Singlet	Neurons
SAT_26M	1361.3	847	3.1839546	0	Singlet	Adipocyte
SAT_26M	1404.9	848	3.6217048	0	Singlet	Adipocyte
SAT_26M	5834.8	2659	5.4607309	9	Singlet	Mesothelial

SAT_26M	1693.3	1141	4.7616462	4	Singlet	Immune_cells
SAT_26M	1681.8	1043	0.1986919	0	Singlet	Adipocyte
SAT_26M	2114.6	1396	4.5773345	5	Singlet	Immune_cells
SAT_26M	1854.4	1083	3.8920768	0	Singlet	Adipocyte
SAT_26M	1473.5	912	5.6356175	1	Singlet	Immune_cells
SAT_26M	1399.3	809	2.1106702	10	Singlet	Immune_cells
SAT_26M	1208.4	790	11.744615	4	Singlet	Immune_cells
SAT_26M	2448.1	1459	2.7917795	7	Singlet	Endothelial_cells
SAT_26M	3594.1	1842	3.3230097	2	Singlet	FAPs
SAT_26M	1188.9	883	1.7601181	11	Singlet	Smooth_muscle_cells
SAT_26M	1513.1	919	6.0624379	1	Singlet	Immune_cells
SAT_26M	1329.7	891	5.1442635	1	Singlet	Immune_cells
SAT_26M	3867	1772	9.429798	10	Singlet	Immune_cells
SAT_26M	1019.1	686	13.298403	1	Singlet	Immune_cells
SAT_26M	2128.7	1481	4.6658767	8	Singlet	Mesothelial
SAT_26M	1987.2	1100	4.3227257	0	Singlet	Adipocyte
SAT_26M	1107.7	727	3.0532784	3	Singlet	FAPs
SAT_26M	1530	1013	0.0359919	8	Singlet	Mesothelial
SAT_26M	3454.7	1829	1.739919	0	Singlet	Adipocyte
SAT_26M	7788.6	3146	8.3110869	9	Singlet	Mesothelial
SAT_26M	1271.2	750	3.4051366	7	Singlet	Endothelial_cells
SAT_26M	1266.3	808	11.500754	4	Singlet	Immune_cells
SAT_26M	2635.2	1379	2.4940367	0	Singlet	Adipocyte
SAT_26M	1191.6	747	1.4140843	0	Singlet	Adipocyte
SAT_26M	3657.2	1701	1.1367486	0	Singlet	Adipocyte
SAT_26M	1163.6	718	13.052641	4	Singlet	Immune_cells
SAT_26M	2058.1	1176	0.10662	8	Singlet	Mesothelial
SAT_26M	2384.9	1476	5.5699171	8	Singlet	Mesothelial
SAT_26M	1735.4	1102	1.9152965	1	Singlet	Immune_cells
SAT_26M	1283	801	0.2601187	0	Singlet	Adipocyte
SAT_26M	1057.2	850	0.6316829	6	Singlet	Neurons
SAT_26M	1198.7	746	0.3090703	0	Singlet	Adipocyte
SAT_26M	2964.2	1511	3.5750194	8	Singlet	Mesothelial
SAT_26M	1459.2	889	6.4837729	1	Singlet	Immune_cells
SAT_26M	1125.3	728	6.9742655	1	Singlet	Immune_cells
SAT_26M	1041.6	660	1.995268	10	Singlet	Immune_cells
SAT_26M	2696.4	1437	2.6653221	10	Singlet	Immune_cells
SAT_26M	2394.2	1351	9.6875874	4	Singlet	Immune_cells
SAT_26M	1412	841	0.2488956	0	Singlet	Adipocyte
SAT_26M	2304.6	1474	6.6277362	9	Singlet	Mesothelial
SAT_26M	1137	897	0.370926	7	Singlet	Endothelial_cells
SAT_26M	1944.2	1121	1.6300696	0	Singlet	Adipocyte
SAT_26M	1264.9	791	2.4557467	0	Singlet	Adipocyte
SAT_26M	1059.7	595	6.1063677	10	Singlet	Immune_cells
SAT_26M	1739.1	965	2.2976142	7	Singlet	Endothelial_cells
SAT_26M	1194.8	809	0.38869	9	Singlet	Mesothelial
SAT_26M	1128.7	802	8.4798978	8	Singlet	Mesothelial
SAT_26M	1474.3	965	5.6237273	1	Singlet	Immune_cells
SAT_26M	1784.2	1099	7.5495798	1	Singlet	Immune_cells
SAT_26M	2197.2	1325	6.5455913	8	Singlet	Mesothelial
SAT_26M	1571.8	1023	3.8976915	1	Singlet	Immune_cells

SAT_26M	1103.3	665	2.0876156	10	Singlet	Immune_cells
SAT_26M	3039.5	1449	1.7514163	0	Singlet	Adipocyte
SAT_26M	1810.2	1035	7.2090972	9	Singlet	Mesothelial
SAT_26M	5131.4	2214	12.744743	9	Singlet	Mesothelial
SAT_26M	1408.2	935	0.0786033	4	Singlet	Immune_cells
SAT_26M	1422.6	922	6.0632315	1	Singlet	Immune_cells
SAT_26M	1376.9	830	1.1206559	0	Singlet	Adipocyte
SAT_26M	2001.1	1182	1.2172135	0	Singlet	Adipocyte
SAT_26M	1325.8	868	2.5993227	1	Singlet	Immune_cells
SAT_26M	1085.7	805	7.7275808	1	Singlet	Immune_cells
SAT_26M	1091.1	755	1.0096685	0	Singlet	Adipocyte
SAT_26M	1612.6	894	0.2410307	3	Singlet	FAPs
SAT_26M	1059.6	725	1.1969251	8	Singlet	Mesothelial
SAT_26M	2659.8	1514	0	0	Singlet	Adipocyte
SAT_26M	1219	872	0.2694972	4	Singlet	Immune_cells
SAT_26M	1307.6	823	0.8777949	0	Singlet	Adipocyte
SAT_26M	1129.8	748	4.4580816	1	Singlet	Immune_cells
SAT_26M	2993.3	1452	6.980704	0	Singlet	Adipocyte
SAT_26M	2321.5	1435	3.1714908	8	Singlet	Mesothelial
SAT_26M	2446.1	1268	1.8115522	10	Singlet	Immune_cells
SAT_26M	1484.1	1008	1.8206559	1	Singlet	Immune_cells
SAT_26M	1108.7	866	5.5825649	6	Singlet	Neurons
SAT_26M	1002.7	748	0.0392118	6	Singlet	Neurons
SAT_26M	2923	1671	4.7820445	8	Singlet	Mesothelial
SAT_26M	2329.8	1334	2.8443143	1	Singlet	Immune_cells
SAT_26M	1305.9	1080	1.8086375	6	Singlet	Neurons
SAT_26M	5292.8	2115	2.6531636	10	Singlet	Immune_cells
SAT_26M	1030.1	730	8.5454111	1	Singlet	Immune_cells
SAT_26M	2790.9	1500	12.797554	5	Singlet	Immune_cells
SAT_26M	6930.8	3000	3.2313285	9	Singlet	Mesothelial
SAT_26M	1760.5	1105	1.3197056	1	Singlet	Immune_cells
SAT_26M	1485.5	995	2.9386138	1	Singlet	Immune_cells
SAT_26M	1139.2	714	3.9386676	10	Singlet	Immune_cells
SAT_26M	1339.9	851	0.0945956	0	Singlet	Adipocyte
SAT_26M	3619.2	1765	0.0014201	0	Singlet	Adipocyte
SAT_26M	1501	926	4.9082375	1	Singlet	Immune_cells
SAT_26M	1097	723	0.0282432	0	Singlet	Adipocyte
SAT_26M	1796.7	989	1.2798807	0	Singlet	Adipocyte
SAT_26M	1254.3	865	4.1408917	1	Singlet	Immune_cells
SAT_26M	1609.6	977	0.1193883	0	Singlet	Adipocyte
SAT_26M	1221.2	757	1.6404836	10	Singlet	Immune_cells
SAT_26M	1353.5	801	2.1589772	2	Singlet	FAPs
SAT_26M	2068.9	1186	1.0475615	2	Singlet	FAPs
SAT_26M	1038.4	658	10.250417	0	Singlet	Adipocyte
SAT_26M	2689.3	1362	0.2140444	0	Singlet	Adipocyte
SAT_26M	2546.1	1351	0.2664259	0	Singlet	Adipocyte
SAT_26M	1532.4	1030	7.4770689	4	Singlet	Immune_cells
SAT_26M	2089.1	1153	10.62515	5	Singlet	Immune_cells
SAT_26M	2347.3	1216	0.4258944	0	Singlet	Adipocyte
SAT_26M	1055	730	9.4420947	1	Singlet	Immune_cells
SAT_26M	2306.4	1338	2.299405	8	Singlet	Mesothelial

SAT_26M	1919.5	1069	5.5548915	0	Singlet	Adipocyte
SAT_26M	2841.5	1463	0.5121198	0	Singlet	Adipocyte
SAT_26M	1023.2	873	0	6	Singlet	Neurons
SAT_26M	1151	736	3.9414637	0	Singlet	Adipocyte
SAT_26M	1422.4	873	2.949842	0	Singlet	Adipocyte
SAT_26M	1310.2	943	4.6768612	8	Singlet	Mesothelial
SAT_26M	2297	1355	1.3309818	7	Singlet	Endothelial_cells
SAT_26M	2335	1222	5.904819	0	Singlet	Adipocyte
SAT_26M	1938.4	1115	0.9109797	0	Singlet	Adipocyte
SAT_26M	1063.7	674	0	0	Singlet	Adipocyte
SAT_26M	2641.1	1777	1.2149316	11	Singlet	Smooth_muscle_cells
SAT_26M	1017.4	684	0.2570076	0	Singlet	Adipocyte
SAT_26M	2495.2	1291	10.342529	0	Singlet	Adipocyte
SAT_26M	1230.8	816	4.7204871	1	Singlet	Immune_cells
SAT_26M	2824.8	1422	0.0115906	0	Singlet	Adipocyte
SAT_26M	1276.1	801	0.0475204	0	Singlet	Adipocyte
SAT_26M	2455	1328	10.694102	5	Singlet	Immune_cells
SAT_26M	2839.2	1791	5.5346871	8	Singlet	Mesothelial
SAT_26M	1781.2	1037	0.2956207	0	Singlet	Adipocyte
SAT_26M	1143.5	770	7.2690007	5	Singlet	Immune_cells
SAT_26M	1463.7	1133	0.6119338	6	Singlet	Neurons
SAT_26M	1256.4	823	7.7309651	1	Singlet	Immune_cells
SAT_26M	2595.8	1313	0.4311383	0	Singlet	Adipocyte
SAT_26M	2315.9	1304	0.3213225	0	Singlet	Adipocyte
SAT_26M	1046.7	841	1.2398191	6	Singlet	Neurons
SAT_26M	1593.3	964	0.1840021	0	Singlet	Adipocyte
SAT_26M	2591	1393	0.9344986	0	Singlet	Adipocyte
SAT_26M	1205.8	785	1.2918978	0	Singlet	Adipocyte
SAT_26M	1028.7	659	12.010914	1	Singlet	Immune_cells
SAT_26M	1500.4	827	5.9763843	0	Singlet	Adipocyte
SAT_26M	1007.1	733	0.4030775	3	Singlet	FAPs
SAT_26M	1324.8	864	0.1678021	0	Singlet	Adipocyte
SAT_26M	1063.2	729	4.2030637	1	Singlet	Immune_cells
SAT_26M	1481.6	1006	3.1599203	1	Singlet	Immune_cells
SAT_26M	2442	1321	4.7278791	0	Singlet	Adipocyte
SAT_26M	1716.7	1000	0.0243562	0	Singlet	Adipocyte
SAT_26M	7720.3	3291	14.064283	9	Singlet	Mesothelial
SAT_26M	1224.9	831	5.7693115	1	Singlet	Immune_cells
SAT_26M	1110.1	698	12.221517	4	Singlet	Immune_cells
SAT_26M	2347.1	1398	3.7461543	1	Singlet	Immune_cells
SAT_26M	1230.6	814	6.0869501	0	Singlet	Adipocyte
SAT_26M	1999.1	1179	2.1993062	1	Singlet	Immune_cells
SAT_26M	1885.3	1243	0.8283978	8	Singlet	Mesothelial
SAT_26M	1276.6	811	0.334026	8	Singlet	Mesothelial
SAT_26M	1000.9	714	0.6766942	1	Singlet	Immune_cells
SAT_26M	1819.6	1037	9.9804007	0	Singlet	Adipocyte
SAT_26M	1176.2	738	4.0477971	0	Singlet	Adipocyte
SAT_26M	2133.1	1196	3.1750395	7	Singlet	Endothelial_cells
SAT_26M	2856.8	1438	2.2074258	10	Singlet	Immune_cells
SAT_26M	1759.2	1134	3.231263	7	Singlet	Endothelial_cells
SAT_26M	1064.3	736	4.819142	1	Singlet	Immune_cells

SAT_26M	1548.9	890	0.2162541	0	Singlet	Adipocyte
SAT_26M	1078.6	775	6.1437831	11	Singlet	Smooth_muscle_cells
SAT_26M	2562.1	1482	0	8	Singlet	Mesothelial
SAT_26M	1719.4	1039	1.5218499	0	Singlet	Adipocyte
SAT_26M	4540.8	2263	1.8464165	8	Singlet	Mesothelial
SAT_26M	1591.6	862	1.823964	10	Singlet	Immune_cells
SAT_26M	1002.1	749	12.165471	4	Singlet	Immune_cells
SAT_26M	1187.7	774	4.7213525	0	Singlet	Adipocyte
SAT_26M	1153.2	829	0.1804221	11	Singlet	Smooth_muscle_cells
SAT_26M	2104.8	1151	0.0170378	0	Singlet	Adipocyte
SAT_26M	2118.8	1272	3.6992456	8	Singlet	Mesothelial
SAT_26M	7712.8	3417	8.710433	9	Singlet	Mesothelial
SAT_26M	1567.2	961	0.1292994	8	Singlet	Mesothelial
SAT_26M	1263.5	869	13.746011	1	Singlet	Immune_cells
SAT_26M	1149.9	779	0.1225956	0	Singlet	Adipocyte
SAT_26M	1034.9	686	1.8543517	5	Singlet	Immune_cells
SAT_26M	1106.8	788	8.3392356	4	Singlet	Immune_cells
SAT_26M	3212.1	1737	4.9447925	2	Singlet	FAPs
SAT_26M	2470.1	1497	1.55549	7	Singlet	Endothelial_cells
SAT_26M	7061.9	3155	11.594866	9	Singlet	Mesothelial
SAT_26M	1930.3	1116	1.8929403	0	Singlet	Adipocyte
SAT_26M	1222.1	874	0.4568869	7	Singlet	Endothelial_cells
SAT_26M	1008	711	4.1553313	1	Singlet	Immune_cells
SAT_26M	2331.5	1380	4.7993211	8	Singlet	Mesothelial
SAT_26M	4611.4	1832	3.604538	10	Singlet	Immune_cells
SAT_26M	1588.9	1005	1.7560834	7	Singlet	Endothelial_cells
SAT_26M	1790	994	0.07173	0	Singlet	Adipocyte
SAT_26M	1569.4	945	0	0	Singlet	Adipocyte
SAT_26M	1179.3	740	9.1752678	0	Singlet	Adipocyte
SAT_26M	1255.1	776	1.00854	0	Singlet	Adipocyte
SAT_26M	1452.2	941	1.3455435	1	Singlet	Immune_cells
SAT_26M	1102.9	767	1.6286785	0	Singlet	Adipocyte
SAT_26M	1559.3	953	8.5283745	1	Singlet	Immune_cells
SAT_26M	1275.6	824	6.8297104	8	Singlet	Mesothelial
SAT_26M	1964.7	1175	0.5575235	2	Singlet	FAPs
SAT_26M	2727.4	1621	4.5555689	1	Singlet	Immune_cells
SAT_26M	6526.8	3155	3.7782778	8	Singlet	Mesothelial
SAT_26M	2197.9	1327	0.5743055	2	Singlet	FAPs
SAT_26M	1571.6	1001	10.719992	4	Singlet	Immune_cells
SAT_26M	1436.1	893	13.529248	4	Singlet	Immune_cells
SAT_26M	1026	641	0	0	Singlet	Adipocyte
SAT_26M	2009.7	1173	0.3393618	0	Singlet	Adipocyte
SAT_26M	2532.1	1511	1.2279945	7	Singlet	Endothelial_cells
SAT_26M	1563.5	1074	6.7224658	4	Singlet	Immune_cells
SAT_26M	1177.3	866	10.384554	8	Singlet	Mesothelial
SAT_26M	2126.5	1261	0.8941032	0	Singlet	Adipocyte
SAT_26M	1346.6	866	11.088052	4	Singlet	Immune_cells
SAT_26M	1403.6	922	6.0755614	1	Singlet	Immune_cells
SAT_26M	2201.3	1022	5.7326009	10	Singlet	Immune_cells
SAT_26M	2132.3	1207	1.3725128	0	Singlet	Adipocyte
SAT_26M	2341.3	1396	1.0822253	0	Singlet	Adipocyte

SAT_26M	1055.1	699	7.1549588	1	Singlet	Immune_cells
SAT_26M	2730	1633	0.0505449	8	Singlet	Mesothelial
SAT_26M	1428.1	967	5.6947702	5	Singlet	Immune_cells
SAT_26M	1089.5	742	0.0290061	0	Singlet	Adipocyte
SAT_26M	1562.2	887	8.6746029	1	Singlet	Immune_cells
SAT_26M	1413.7	739	3.9226519	10	Singlet	Immune_cells
SAT_26M	4581.9	2122	0.8582427	0	Singlet	Adipocyte
SAT_26M	4131	1905	3.2074209	2	Singlet	FAPs
SAT_26M	2484.5	1468	4.1530133	7	Singlet	Endothelial_cells
SAT_26M	1296	807	0.3277137	0	Singlet	Adipocyte
SAT_26M	1409.7	918	3.3138207	1	Singlet	Immune_cells
SAT_26M	3608.4	1932	7.5877788	9	Singlet	Mesothelial
SAT_26M	2245.9	1261	1.82318	0	Singlet	Adipocyte
SAT_26M	2552.4	1458	3.5935065	1	Singlet	Immune_cells
SAT_26M	1235.1	939	8.5706314	6	Singlet	Neurons
SAT_26M	1083.6	706	9.3453366	4	Singlet	Immune_cells
SAT_26M	1065.6	815	2.6522168	6	Singlet	Neurons
SAT_26M	2543.2	1463	1.2304086	0	Singlet	Adipocyte
SAT_26M	1528.3	985	4.210622	1	Singlet	Immune_cells
SAT_26M	6438.9	3225	2.5042163	8	Singlet	Mesothelial
SAT_26M	1213.1	769	10.489492	1	Singlet	Immune_cells
SAT_26M	1095.2	740	8.0248074	1	Singlet	Immune_cells
SAT_26M	6617.2	2587	0.0554044	0	Singlet	Adipocyte
SAT_26M	1410.1	828	2.6371443	0	Singlet	Adipocyte
SAT_26M	1182.7	768	0.7409802	0	Singlet	Adipocyte
SAT_26M	1510	1046	0.4298117	8	Singlet	Mesothelial
SAT_26M	1613.6	1000	2.7767722	7	Singlet	Endothelial_cells
SAT_26M	1020.8	705	6.9040308	1	Singlet	Immune_cells
SAT_26M	2181.9	1287	0.2254075	8	Singlet	Mesothelial
SAT_26M	4610	2046	0.2485689	0	Singlet	Adipocyte
SAT_26M	1161.5	745	0.1826505	0	Singlet	Adipocyte
SAT_26M	1953.5	1166	5.0872368	1	Singlet	Immune_cells
SAT_26M	1753.8	1093	5.8987344	1	Singlet	Immune_cells
SAT_26M	1012	596	12.291119	5	Singlet	Immune_cells
SAT_26M	4354.6	2385	5.1880744	8	Singlet	Mesothelial
SAT_26M	6065.6	2241	0.7432849	10	Singlet	Immune_cells
SAT_26M	7769.3	3196	14.056267	4	Singlet	Immune_cells
SAT_26M	1468.8	985	5.0476292	1	Singlet	Immune_cells
SAT_26M	1151	824	2.7914369	5	Singlet	Immune_cells
SAT_26M	1437.8	882	0.0821131	0	Singlet	Adipocyte
SAT_26M	1112.1	760	0	0	Singlet	Adipocyte
SAT_26M	1174.1	826	2.129365	1	Singlet	Immune_cells
SAT_26M	1135.5	777	0.0655645	8	Singlet	Mesothelial
SAT_26M	1223	789	9.57953	4	Singlet	Immune_cells
SAT_26M	1246	802	0.0718281	5	Singlet	Immune_cells
SAT_26M	1516.7	933	8.2692598	1	Singlet	Immune_cells
SAT_26M	1274.4	703	9.028088	0	Singlet	Adipocyte
SAT_26M	1799.5	1050	13.500224	4	Singlet	Immune_cells
SAT_26M	2365.9	1252	0.4343107	0	Singlet	Adipocyte
SAT_26M	1201.1	781	2.6151015	0	Singlet	Adipocyte
SAT_26M	1322.2	858	6.7010897	1	Singlet	Immune_cells

SAT_26M	1189	913	6.4743399	8	Singlet	Mesothelial
SAT_26M	1517.8	756	4.0085856	10	Singlet	Immune_cells
SAT_26M	2749.2	1579	12.143493	4	Singlet	Immune_cells
SAT_26M	2060.4	1309	3.3778693	1	Singlet	Immune_cells
SAT_26M	1139.9	853	0.5958359	6	Singlet	Neurons
SAT_26M	2755.3	1368	0	0	Singlet	Adipocyte
SAT_26M	3380.9	1699	0.8623002	0	Singlet	Adipocyte
SAT_26M	1056.4	760	3.1600604	1	Singlet	Immune_cells
SAT_26M	1042.4	846	2.0888085	6	Singlet	Neurons
SAT_26M	1131	680	11.281418	8	Singlet	Mesothelial
SAT_26M	1358.4	873	1.8741682	8	Singlet	Mesothelial
SAT_26M	1796.7	1099	2.2840793	8	Singlet	Mesothelial
SAT_26M	2076.5	1200	1.240561	0	Singlet	Adipocyte
SAT_26M	1058.7	752	1.1322897	1	Singlet	Immune_cells
SAT_26M	2199.5	1426	3.4374089	9	Singlet	Mesothelial
SAT_26M	2272.7	1323	0	8	Singlet	Mesothelial
SAT_26M	1561	1000	1.3853196	11	Singlet	Smooth_muscle_cells
SAT_26M	11406	4273	11.668946	9	Singlet	Mesothelial
SAT_26M	1262.1	793	0.1792134	0	Singlet	Adipocyte
SAT_26M	1645.6	1071	2.1426275	1	Singlet	Immune_cells
SAT_26M	1231.9	898	8.6287228	7	Singlet	Endothelial_cells
SAT_26M	1804.4	1081	5.2816591	1	Singlet	Immune_cells
SAT_26M	1427.8	950	4.4624452	1	Singlet	Immune_cells
SAT_26M	5662.1	2731	3.8021265	8	Singlet	Mesothelial
SAT_26M	1337.6	877	3.5254108	1	Singlet	Immune_cells
SAT_26M	2389.3	1186	2.3768435	10	Singlet	Immune_cells
SAT_26M	1223.1	831	0.0587112	8	Singlet	Mesothelial
SAT_26M	7510.7	3405	3.6925285	8	Singlet	Mesothelial
SAT_26M	2910.6	1635	2.6307342	9	Singlet	Mesothelial
SAT_26M	3763.2	1868	0.5970739	0	Singlet	Adipocyte
SAT_26M	1072.5	728	12.878275	1	Singlet	Immune_cells
SAT_26M	1244.6	778	0.0472228	8	Singlet	Mesothelial
SAT_26M	1766	1266	3.2721857	11	Singlet	Smooth_muscle_cells
SAT_26M	1026.8	660	10.227551	1	Singlet	Immune_cells
SAT_26M	1130.3	746	14.494169	4	Singlet	Immune_cells
SAT_26M	1100	769	0.2252427	4	Singlet	Immune_cells
SAT_26M	1300.9	835	0.4811722	0	Singlet	Adipocyte
SAT_26M	1590.4	972	0.9715309	0	Singlet	Adipocyte
SAT_26M	1460.2	937	4.7380094	1	Singlet	Immune_cells
SAT_26M	2457.4	1463	4.0139661	8	Singlet	Mesothelial
SAT_26M	2220.9	1171	4.1364428	0	Singlet	Adipocyte
SAT_26M	2811.9	1540	0.1532582	0	Singlet	Adipocyte
SAT_26M	2000.9	1109	5.0376405	3	Singlet	FAPs
SAT_26M	1263.5	840	0.850286	1	Singlet	Immune_cells
SAT_26M	1309.5	835	1.9271446	0	Singlet	Adipocyte
SAT_26M	1236.6	856	1.7859992	1	Singlet	Immune_cells
SAT_26M	1745.9	1019	1.3158852	0	Singlet	Adipocyte
SAT_26M	1297.4	1047	0.9772642	1	Singlet	Immune_cells
SAT_26M	1208.1	759	5.4331871	7	Singlet	Endothelial_cells
SAT_26M	1083.5	737	8.3552038	4	Singlet	Immune_cells
SAT_26M	1009.2	699	10.20753	11	Singlet	Smooth_muscle_cells

SAT_26M	6782.6	2738	1.2758025	0	Singlet	Adipocyte
SAT_26M	2689.4	1548	2.5612072	8	Singlet	Mesothelial
SAT_26M	1142.4	738	7.2819798	1	Singlet	Immune_cells
SAT_26M	1268.2	889	0.0129509	0	Singlet	Adipocyte
SAT_26M	1019.2	737	0.070748	5	Singlet	Immune_cells
SAT_26M	1977.4	1226	3.1057681	1	Singlet	Immune_cells
SAT_26M	5186.7	2484	5.5232314	9	Singlet	Mesothelial
SAT_26M	1091.7	720	0.0591224	8	Singlet	Mesothelial
SAT_26M	1488.4	902	6.0248094	1	Singlet	Immune_cells
SAT_26M	1119.2	753	0	11	Singlet	Smooth_muscle_cells
SAT_26M	1036.7	679	0.3103252	0	Singlet	Adipocyte
SAT_26M	2102.2	1272	0	8	Singlet	Mesothelial
SAT_26M	1977.6	1091	0.9646581	0	Singlet	Adipocyte
SAT_26M	1444.1	893	10.853019	4	Singlet	Immune_cells
SAT_26M	2014.6	1200	4.5842699	1	Singlet	Immune_cells
SAT_26M	1041.2	674	0	0	Singlet	Adipocyte
SAT_26M	2135.7	1279	0.0891095	0	Singlet	Adipocyte
SAT_26M	2267.6	1279	7.1054192	8	Singlet	Mesothelial
SAT_26M	1757.8	1114	1.149037	1	Singlet	Immune_cells
SAT_26M	1517.3	964	0.364822	2	Singlet	FAPs
SAT_26M	1641.5	1028	0.0474483	3	Singlet	FAPs
SAT_26M	2664.3	1559	0	8	Singlet	Mesothelial
SAT_26M	1265.3	760	3.2513173	10	Singlet	Immune_cells
SAT_26M	1123.3	717	10.45609	4	Singlet	Immune_cells
SAT_26M	2436.6	1253	13.667766	1	Singlet	Immune_cells
SAT_26M	3903.1	1512	9.457896	10	Singlet	Immune_cells
SAT_26M	1319.5	822	0.0423501	0	Singlet	Adipocyte
SAT_26M	1132.2	917	0.1467975	6	Singlet	Neurons
SAT_26M	2461.3	1399	3.2321113	7	Singlet	Endothelial_cells
SAT_26M	2119.7	1069	1.4319947	10	Singlet	Immune_cells
SAT_26M	1288.7	833	0.2361947	0	Singlet	Adipocyte
SAT_26M	2778.5	1575	5.3252439	7	Singlet	Endothelial_cells
SAT_26M	1133.3	717	0.830705	3	Singlet	FAPs
SAT_26M	2738.5	1464	1.0906219	0	Singlet	Adipocyte
SAT_26M	1804	954	8.2036598	10	Singlet	Immune_cells
SAT_26M	2169.3	1380	3.9919364	4	Singlet	Immune_cells
SAT_26M	1296.7	853	13.030116	5	Singlet	Immune_cells
SAT_26M	5969.7	2844	3.5621337	8	Singlet	Mesothelial
SAT_26M	2954.5	1715	3.4657067	8	Singlet	Mesothelial
SAT_26M	1942.9	1305	0.2529921	5	Singlet	Immune_cells
SAT_26M	1156	778	0.2819972	0	Singlet	Adipocyte
SAT_26M	2636	1487	0.3840005	0	Singlet	Adipocyte
SAT_26M	1618.3	984	0.1423972	0	Singlet	Adipocyte
SAT_26M	1247.5	746	1.9673112	3	Singlet	FAPs
SAT_26M	1212.3	985	0.1653127	6	Singlet	Neurons
SAT_26M	1821.8	1065	0.4624818	0	Singlet	Adipocyte
SAT_26M	7349.7	3179	1.2974584	8	Singlet	Mesothelial
SAT_26M	1530.8	1025	2.886782	1	Singlet	Immune_cells
SAT_26M	2189.2	1322	4.2023154	1	Singlet	Immune_cells
SAT_26M	2422.8	1321	2.7799482	0	Singlet	Adipocyte
SAT_26M	1453.8	1089	11.712724	6	Singlet	Neurons

SAT_26M	1115.1	836	2.594685	4	Singlet	Immune_cells
SAT_26M	1216.8	746	3.4668713	0	Singlet	Adipocyte
SAT_26M	3046	1295	3.9015867	10	Singlet	Immune_cells
SAT_26M	1092.9	721	8.1947456	1	Singlet	Immune_cells
SAT_26M	1528.3	927	0.1643938	0	Singlet	Adipocyte
SAT_26M	1407.7	947	5.7622073	1	Singlet	Immune_cells
SAT_26M	1981.5	1030	12.22956	0	Singlet	Adipocyte
SAT_26M	4647	1971	4.5428377	10	Singlet	Immune_cells
SAT_26M	1991.9	953	3.2127154	10	Singlet	Immune_cells
SAT_26M	3882.4	1511	1.6741272	10	Singlet	Immune_cells
SAT_26M	1431.4	870	13.804314	1	Singlet	Immune_cells
SAT_26M	1599.3	1217	0.0981675	4	Singlet	Immune_cells
SAT_26M	1155.7	692	1.0113311	0	Singlet	Adipocyte
SAT_26M	1934.4	1202	3.2071475	8	Singlet	Mesothelial
SAT_26M	1270.3	840	6.3326353	6	Singlet	Neurons
SAT_26M	1233.3	928	0	6	Singlet	Neurons
SAT_26M	1356.8	867	2.7972799	1	Singlet	Immune_cells
SAT_26M	2199.2	1311	4.2272351	1	Singlet	Immune_cells
SAT_26M	3755.1	1779	0.2191038	0	Singlet	Adipocyte
SAT_26M	2424.2	1417	3.0599604	1	Singlet	Immune_cells
SAT_26M	2509.4	1277	5.5671537	10	Singlet	Immune_cells
SAT_26M	1790.3	686	2.6348935	4	Singlet	Immune_cells
SAT_26M	1037.3	688	0.3801555	5	Singlet	Immune_cells
SAT_26M	2476.2	1296	3.0785903	0	Singlet	Adipocyte
SAT_26M	1198.3	938	3.9452369	1	Singlet	Immune_cells
SAT_26M	2659.4	1474	0.2119614	0	Singlet	Adipocyte
SAT_26M	2068.2	1339	0.079993	8	Singlet	Mesothelial
SAT_26M	1406.2	798	2.0156353	10	Singlet	Immune_cells
SAT_26M	1544.2	972	1.0730633	0	Singlet	Adipocyte
SAT_26M	1249.3	765	0.2790017	0	Singlet	Adipocyte
SAT_26M	1081.9	671	13.334765	1	Singlet	Immune_cells
SAT_26M	1242.1	942	8.6966608	1	Singlet	Immune_cells
SAT_26M	2526.5	1400	10.649835	4	Singlet	Immune_cells
SAT_26M	1033.7	834	0.7782273	6	Singlet	Neurons
SAT_26M	2186.2	1318	1.5663218	1	Singlet	Immune_cells
SAT_26M	1966.6	1150	0.9188332	0	Singlet	Adipocyte
SAT_26M	3468	2020	3.6626618	4	Singlet	Immune_cells
SAT_26M	1706.2	1071	0.1369243	8	Singlet	Mesothelial
SAT_26M	3748.9	2049	5.1018887	9	Singlet	Mesothelial
SAT_26M	1510	995	0.8482049	8	Singlet	Mesothelial
SAT_26M	1119	687	0.0272488	0	Singlet	Adipocyte
SAT_26M	1331.6	829	1.3874489	5	Singlet	Immune_cells
SAT_26M	1281.1	1070	3.8035361	6	Singlet	Neurons
SAT_26M	1176.6	795	2.2671067	7	Singlet	Endothelial_cells
SAT_26M	2330.2	1305	3.3778943	1	Singlet	Immune_cells
SAT_26M	2769	1445	6.2601619	0	Singlet	Adipocyte
SAT_26M	1015	603	14.031408	1	Singlet	Immune_cells
SAT_26M	1229.3	751	6.5219196	0	Singlet	Adipocyte
SAT_26M	2889.2	1643	4.9041828	8	Singlet	Mesothelial
SAT_26M	1992.6	1198	14.880104	1	Singlet	Immune_cells
SAT_26M	1095.7	727	1.1101751	4	Singlet	Immune_cells

SAT_26M	1164	853	5.0563536	4	Singlet	Immune_cells
SAT_26M	1418.5	809	11.185289	9	Singlet	Mesothelial
SAT_26M	1013	736	7.8668372	1	Singlet	Immune_cells
SAT_26M	1076.1	695	1.3850965	2	Singlet	FAPs
SAT_26M	1133.2	698	0.2498652	0	Singlet	Adipocyte
SAT_26M	1011.1	683	0.6530208	0	Singlet	Adipocyte
SAT_26M	1177	732	0.6854836	0	Singlet	Adipocyte
SAT_26M	1484.2	964	10.093787	1	Singlet	Immune_cells
SAT_26M	2049.9	1188	0.0235153	0	Singlet	Adipocyte
SAT_26M	1791.6	1099	0.0300813	0	Singlet	Adipocyte
SAT_26M	1215.5	861	1.0546632	9	Singlet	Mesothelial
SAT_26M	2459.8	1381	0.8307818	2	Singlet	FAPs
SAT_26M	3361.8	1675	0.028386	0	Singlet	Adipocyte
SAT_26M	1072.1	661	0.0309005	0	Singlet	Adipocyte
SAT_26M	2930.3	1381	3.9277557	10	Singlet	Immune_cells
SAT_26M	1753.2	929	1.7964085	10	Singlet	Immune_cells
SAT_26M	1773.2	1138	0.4693038	8	Singlet	Mesothelial
SAT_26M	2298.6	1321	1.0403446	0	Singlet	Adipocyte
SAT_26M	1183	762	1.6955362	5	Singlet	Immune_cells
SAT_26M	1967.9	1174	3.9863415	1	Singlet	Immune_cells
SAT_26M	4562	2200	8.2589511	9	Singlet	Mesothelial
SAT_26M	2356	1267	5.1926416	0	Singlet	Adipocyte
SAT_26M	1024.3	683	14.073516	1	Singlet	Immune_cells
SAT_26M	1967.8	1165	2.5758454	0	Singlet	Adipocyte
SAT_26M	1763.3	1088	6.096634	1	Singlet	Immune_cells
SAT_26M	1149.7	848	13.844508	1	Singlet	Immune_cells
SAT_26M	1599.3	931	0.1914231	0	Singlet	Adipocyte
SAT_26M	5139.9	2522	11.281067	4	Singlet	Immune_cells
SAT_26M	7487	3274	10.552743	9	Singlet	Mesothelial
SAT_26M	1493.9	960	2.3012956	1	Singlet	Immune_cells
SAT_26M	2810.4	1474	0.1207512	0	Singlet	Adipocyte
SAT_26M	1832.6	1105	0.1727261	8	Singlet	Mesothelial
SAT_26M	2841.9	1552	6.4004068	7	Singlet	Endothelial_cells
SAT_26M	1443.9	929	0.0750575	10	Singlet	Immune_cells
SAT_26M	1617.8	859	1.6954021	10	Singlet	Immune_cells
SAT_26M	1986.8	1017	0.5428329	0	Singlet	Adipocyte
SAT_26M	1051.6	632	3.5132636	4	Singlet	Immune_cells
SAT_26M	2933.1	1560	4.0691468	8	Singlet	Mesothelial
SAT_26M	2901.2	1567	5.2644278	4	Singlet	Immune_cells
SAT_26M	1052	726	6.0478299	1	Singlet	Immune_cells
SAT_26M	1077	723	0.2160031	8	Singlet	Mesothelial
SAT_26M	1101.7	686	4.0401421	5	Singlet	Immune_cells
SAT_26M	1434.1	848	5.2088417	0	Singlet	Adipocyte
SAT_26M	1080.2	683	0.4027531	3	Singlet	FAPs
SAT_26M	2923.4	1668	4.2273272	7	Singlet	Endothelial_cells
SAT_26M	1798.6	1027	0.0219265	0	Singlet	Adipocyte
SAT_26M	1126.9	666	0.0584597	0	Singlet	Adipocyte
SAT_26M	2228.3	1472	7.5890232	8	Singlet	Mesothelial
SAT_26M	1490.5	964	0.1349807	5	Singlet	Immune_cells
SAT_26M	1190.7	735	2.88556	0	Singlet	Adipocyte
SAT_26M	1099.6	855	7.4980178	4	Singlet	Immune_cells

SAT_26M	1911.7	1194	2.1932229	1	Singlet	Immune_cells
SAT_26M	1669.4	1021	0.0449689	0	Singlet	Adipocyte
SAT_26M	1147.7	722	1.0780904	0	Singlet	Adipocyte
SAT_26M	1751.2	1131	1.4917258	1	Singlet	Immune_cells
SAT_26M	1353	800	1.1383777	0	Singlet	Adipocyte
SAT_26M	1064	628	14.261845	4	Singlet	Immune_cells
SAT_26M	1965.9	1205	3.2218055	7	Singlet	Endothelial_cells
SAT_26M	1111	742	7.0704454	1	Singlet	Immune_cells
SAT_26M	1208	762	0.2671066	0	Singlet	Adipocyte
SAT_26M	3225.1	1653	3.1048364	0	Singlet	Adipocyte
SAT_26M	1766.6	1075	3.2716894	1	Singlet	Immune_cells
SAT_26M	1724.6	1105	0.0763274	8	Singlet	Mesothelial
SAT_26M	2364.5	1033	0.7708372	10	Singlet	Immune_cells
SAT_26M	1735.8	1137	1.5551239	2	Singlet	FAPs
SAT_26M	2586	1518	4.2181128	5	Singlet	Immune_cells
SAT_26M	1746.2	1093	3.2124039	1	Singlet	Immune_cells
SAT_26M	1363.7	895	5.5285031	1	Singlet	Immune_cells
SAT_26M	2358.3	1449	6.7988082	1	Singlet	Immune_cells
SAT_26M	1344.4	903	1.0063416	7	Singlet	Endothelial_cells
SAT_26M	1098.6	698	11.666376	1	Singlet	Immune_cells
SAT_26M	1698.2	1103	4.3817431	1	Singlet	Immune_cells
SAT_26M	2980.1	1308	1.8667747	10	Singlet	Immune_cells
SAT_26M	1553.6	939	0.1880248	0	Singlet	Adipocyte
SAT_26M	1952.2	1223	2.4840656	0	Singlet	Adipocyte
SAT_26M	1754.2	1068	6.5898789	3	Singlet	FAPs
SAT_26M	1414.4	891	0.033016	4	Singlet	Immune_cells
SAT_26M	1522.9	1046	5.4731169	1	Singlet	Immune_cells
SAT_26M	2367.2	1093	9.5359109	10	Singlet	Immune_cells
SAT_26M	1102.7	721	0.2332896	5	Singlet	Immune_cells
SAT_26M	1460.9	837	2.0706699	10	Singlet	Immune_cells
SAT_26M	1167.8	762	3.8508713	1	Singlet	Immune_cells
SAT_26M	3728.7	1816	2.4283997	10	Singlet	Immune_cells
SAT_26M	1115.3	728	0.6370357	0	Singlet	Adipocyte
SAT_26M	1355.5	827	14.061318	1	Singlet	Immune_cells
SAT_26M	1275	810	3.0522297	1	Singlet	Immune_cells
SAT_26M	1998.8	1132	0.6571513	0	Singlet	Adipocyte
SAT_26M	1512.4	845	3.5117508	10	Singlet	Immune_cells
SAT_26M	1139.8	809	6.4617508	11	Singlet	Smooth_muscle_cells
SAT_26M	2334.9	1308	4.7185236	7	Singlet	Endothelial_cells
SAT_26M	2395.8	1327	1.6917405	0	Singlet	Adipocyte
SAT_26M	2112.3	1337	0.0136933	8	Singlet	Mesothelial
SAT_26M	2931.4	1646	5.3117944	7	Singlet	Endothelial_cells
SAT_26M	2759.3	1561	0.6424532	1	Singlet	Immune_cells
SAT_26M	1480.6	914	6.739492	1	Singlet	Immune_cells
SAT_26M	2031.2	1045	2.4784863	10	Singlet	Immune_cells
SAT_26M	1138.1	769	0.3520943	0	Singlet	Adipocyte
SAT_26M	1364.9	869	0.0497523	4	Singlet	Immune_cells
SAT_26M	2381.6	1217	0.0294656	0	Singlet	Adipocyte
SAT_26M	1044.6	734	5.9705156	11	Singlet	Smooth_muscle_cells
SAT_26M	1522.3	928	0.3541749	0	Singlet	Adipocyte
SAT_26M	1081.5	690	0	0	Singlet	Adipocyte

SAT_26M	1381.7	865	0.5394071	0	Singlet	Adipocyte
SAT_26M	2048.7	1134	3.0903027	0	Singlet	Adipocyte
SAT_26M	2035.9	1186	1.543841	0	Singlet	Adipocyte
SAT_26M	1023.4	718	0.5059882	2	Singlet	FAPs
SAT_26M	1744.1	804	5.5202664	10	Singlet	Immune_cells
SAT_26M	1624.3	944	1.6242249	0	Singlet	Adipocyte
SAT_26M	1556.7	986	2.3495688	8	Singlet	Mesothelial
SAT_26M	2595.6	1310	6.4906678	10	Singlet	Immune_cells
SAT_26M	1326.3	857	4.1330234	0	Singlet	Adipocyte
SAT_26M	1206.8	758	4.779798	10	Singlet	Immune_cells
SAT_26M	1585.7	990	3.0815297	1	Singlet	Immune_cells
SAT_26M	1679.3	1125	0.4839482	11	Singlet	Smooth_muscle_cells
SAT_26M	1728.3	1082	14.303539	4	Singlet	Immune_cells
SAT_26M	2546.3	1509	2.5201957	9	Singlet	Mesothelial
SAT_26M	1487.6	714	0.0896524	10	Singlet	Immune_cells
SAT_26M	4381.4	2054	0.0847948	0	Singlet	Adipocyte
SAT_26M	1408.9	932	2.146715	5	Singlet	Immune_cells
SAT_26M	1109.9	900	2.7408579	6	Singlet	Neurons
SAT_26M	1137.1	816	7.5395954	4	Singlet	Immune_cells
SAT_26M	1071.9	788	1.0902003	6	Singlet	Neurons
SAT_26M	1182.3	736	14.768764	5	Singlet	Immune_cells
SAT_26M	1228.1	824	4.9522842	1	Singlet	Immune_cells
SAT_26M	1612.2	1039	3.9896698	1	Singlet	Immune_cells
SAT_26M	2304.7	1058	3.3942038	10	Singlet	Immune_cells
SAT_26M	1658.8	977	7.4405283	1	Singlet	Immune_cells
SAT_26M	1222.2	816	2.6510144	1	Singlet	Immune_cells
SAT_26M	1090.2	724	0	8	Singlet	Mesothelial
SAT_26M	1057.4	812	1.0687634	6	Singlet	Neurons
SAT_26M	4906.9	2350	7.2942406	9	Singlet	Mesothelial
SAT_26M	1334.4	858	11.869344	4	Singlet	Immune_cells
SAT_26M	1740.7	1086	0.4779987	0	Singlet	Adipocyte
SAT_26M	1669.8	975	0.1931282	0	Singlet	Adipocyte
SAT_26M	2985.1	1589	0.5900221	0	Singlet	Adipocyte
SAT_26M	1304.4	819	4.6027513	0	Singlet	Adipocyte
SAT_26M	1010.6	699	12.04594	4	Singlet	Immune_cells
SAT_26M	4830.2	2621	2.8513869	8	Singlet	Mesothelial
SAT_26M	5648.7	2585	8.5495265	9	Singlet	Mesothelial
SAT_26M	1859.9	1132	6.4416188	1	Singlet	Immune_cells
SAT_26M	1717.8	1065	7.7402897	1	Singlet	Immune_cells
SAT_26M	1074.6	741	3.191271	1	Singlet	Immune_cells
SAT_26M	1549	1046	4.3859008	1	Singlet	Immune_cells
SAT_26M	1145.8	763	3.9375179	1	Singlet	Immune_cells
SAT_26M	1047.7	661	0.5041834	0	Singlet	Adipocyte
SAT_26M	2220.7	1202	1.8949011	0	Singlet	Adipocyte
SAT_26M	1425.5	907	0	0	Singlet	Adipocyte
SAT_26M	1398.8	851	2.5324154	0	Singlet	Adipocyte
SAT_26M	1385.3	840	0.0469212	0	Singlet	Adipocyte
SAT_26M	1799.6	1104	0.2039435	0	Singlet	Adipocyte
SAT_26M	2312.7	1406	4.3474292	1	Singlet	Immune_cells
SAT_26M	1050.9	745	12.813917	1	Singlet	Immune_cells
SAT_26M	7635.9	3379	3.2792969	8	Singlet	Mesothelial

SAT_26M	1121.6	755	7.6082489	1	Singlet	Immune_cells
SAT_26M	1988.1	1229	5.4170445	9	Singlet	Mesothelial
SAT_26M	2977.5	1730	8.5633581	9	Singlet	Mesothelial
SAT_26M	3721.4	1784	1.8401182	0	Singlet	Adipocyte
SAT_26M	2020.1	1258	1.6634024	8	Singlet	Mesothelial
SAT_26M	4362.1	2266	10.694426	9	Singlet	Mesothelial
SAT_26M	1072.9	770	1.0630745	5	Singlet	Immune_cells
SAT_26M	1340.3	942	3.5154005	1	Singlet	Immune_cells
SAT_26M	1179.4	794	4.2973477	1	Singlet	Immune_cells
SAT_26M	1619.7	1033	2.4446497	1	Singlet	Immune_cells
SAT_26M	2008.5	1088	1.2716262	0	Singlet	Adipocyte
SAT_26M	1652.2	1077	1.3859125	5	Singlet	Immune_cells
SAT_26M	2621.9	1460	3.5144989	8	Singlet	Mesothelial
SAT_26M	1334.6	883	8.520875	1	Singlet	Immune_cells
SAT_26M	1751.7	1162	7.7778073	4	Singlet	Immune_cells
SAT_26M	1357.1	1135	1.3133564	6	Singlet	Neurons
SAT_26M	1524.7	1033	7.5757678	4	Singlet	Immune_cells
SAT_26M	5808.6	2909	7.5407258	9	Singlet	Mesothelial
SAT_26M	1833	1167	3.1714708	1	Singlet	Immune_cells
SAT_26M	1196.7	981	4.5816634	6	Singlet	Neurons
SAT_26M	2834.5	1549	0.1110393	0	Singlet	Adipocyte
SAT_26M	1251.1	779	5.1220884	0	Singlet	Adipocyte
SAT_26M	3398	1836	9.3813261	8	Singlet	Mesothelial
SAT_26M	1709.1	959	0.2819378	0	Singlet	Adipocyte
SAT_26M	2794.2	1685	0.3747505	4	Singlet	Immune_cells
SAT_26M	2546.7	1214	1.3602548	10	Singlet	Immune_cells
SAT_26M	4178	2279	2.1447562	8	Singlet	Mesothelial
SAT_26M	1017.7	650	2.1392892	0	Singlet	Adipocyte
SAT_26M	2300.5	1308	2.3733323	0	Singlet	Adipocyte
SAT_26M	2336.6	1319	1.9652358	8	Singlet	Mesothelial
SAT_26M	1483.1	884	2.4026963	0	Singlet	Adipocyte
SAT_26M	2771.5	1559	1.5146653	8	Singlet	Mesothelial
SAT_26M	3534.4	1757	7.104524	9	Singlet	Mesothelial
SAT_26M	2512.5	1531	1.5367228	1	Singlet	Immune_cells
SAT_26M	1167.9	830	0.9335801	9	Singlet	Mesothelial
SAT_26M	1176.4	703	6.9895297	10	Singlet	Immune_cells
SAT_26M	2516.1	1406	2.4418904	7	Singlet	Endothelial_cells
SAT_26M	1600.6	1033	11.584098	1	Singlet	Immune_cells
SAT_26M	2473.7	1263	2.8361285	0	Singlet	Adipocyte
SAT_26M	1124.7	765	7.1621096	1	Singlet	Immune_cells
SAT_26M	4990.5	1975	0.5645456	10	Singlet	Immune_cells
SAT_26M	5803.5	2890	4.7420342	9	Singlet	Mesothelial
SAT_26M	1279	780	0.2249034	0	Singlet	Adipocyte
SAT_26M	1198.4	853	6.688309	1	Singlet	Immune_cells
SAT_26M	1725.2	1014	0.0324654	0	Singlet	Adipocyte
SAT_26M	1034.1	754	3.3002298	1	Singlet	Immune_cells
SAT_26M	1145.9	701	11.702879	5	Singlet	Immune_cells
SAT_26M	4055	1767	1.0844103	10	Singlet	Immune_cells
SAT_26M	1262.1	795	8.3423794	5	Singlet	Immune_cells
SAT_26M	1038.3	852	0.1252556	4	Singlet	Immune_cells
SAT_26M	1256.3	770	7.7396413	9	Singlet	Mesothelial

SAT_26M	1503.7	994	2.3202899	1	Singlet	Immune_cells
SAT_26M	1141.1	766	5.0384134	1	Singlet	Immune_cells
SAT_26M	1368.1	876	7.2708476	1	Singlet	Immune_cells
SAT_26M	1286.4	789	6.197225	7	Singlet	Endothelial_cells
SAT_26M	1842.4	1243	1.7102164	3	Singlet	FAPs
SAT_26M	6874.3	3340	2.4664875	8	Singlet	Mesothelial
SAT_26M	1082.6	734	3.7411801	4	Singlet	Immune_cells
SAT_26M	2106.5	1268	3.0299214	7	Singlet	Endothelial_cells
SAT_26M	2101.2	1395	2.8863623	2	Singlet	FAPs
SAT_26M	5156.7	2618	2.6425208	8	Singlet	Mesothelial
SAT_26M	1401.2	870	0.0409618	0	Singlet	Adipocyte
SAT_26M	2673.1	1406	0	0	Singlet	Adipocyte
SAT_26M	1878.7	1277	1.8993277	1	Singlet	Immune_cells
SAT_26M	1987.2	1126	4.3954735	0	Singlet	Adipocyte
SAT_26M	1983.4	1155	0.0196044	3	Singlet	FAPs
SAT_26M	1701.4	1030	0.4518633	0	Singlet	Adipocyte
SAT_26M	1725.7	941	0.0445821	0	Singlet	Adipocyte
SAT_26M	3100.2	1418	3.2098821	10	Singlet	Immune_cells
SAT_26M	1068.8	893	0	6	Singlet	Neurons
SAT_26M	1008.3	690	0.0616569	3	Singlet	FAPs
SAT_26M	1002.3	750	0.893824	6	Singlet	Neurons
SAT_26M	1023.8	667	0.0646199	5	Singlet	Immune_cells
SAT_26M	1317.1	1015	1.0662085	6	Singlet	Neurons
SAT_26M	1976.4	1179	2.6544816	8	Singlet	Mesothelial
SAT_26M	3750.7	1884	3.6630679	8	Singlet	Mesothelial
SAT_26M	4777.3	2373	1.0284691	5	Singlet	Immune_cells
SAT_26M	1939.3	1122	1.8809793	0	Singlet	Adipocyte
SAT_26M	1132.1	910	1.5665068	1	Singlet	Immune_cells
SAT_26M	1103.9	770	0.7589741	0	Singlet	Adipocyte
SAT_26M	1070.5	740	9.5772942	5	Singlet	Immune_cells
SAT_26M	1485.4	987	3.5833543	7	Singlet	Endothelial_cells
SAT_26M	1019.3	631	0.2669364	0	Singlet	Adipocyte
SAT_26M	1119.5	750	1.0229384	0	Singlet	Adipocyte
SAT_26M	1860.2	1064	0.5778607	0	Singlet	Adipocyte
SAT_26M	2410.6	1293	0.0754974	0	Singlet	Adipocyte
SAT_26M	1496.9	1054	3.2950018	1	Singlet	Immune_cells
SAT_26M	1381.2	887	1.1475622	7	Singlet	Endothelial_cells
SAT_26M	1223.2	828	14.059099	4	Singlet	Immune_cells
SAT_26M	1561.2	1046	11.208896	9	Singlet	Mesothelial
SAT_26M	1488.2	932	8.5163679	1	Singlet	Immune_cells
SAT_26M	1381.7	956	3.0890301	1	Singlet	Immune_cells
SAT_26M	1212.3	805	0.3332799	0	Singlet	Adipocyte
SAT_26M	2315.3	1390	0.1822302	0	Singlet	Adipocyte
SAT_26M	2800.6	1809	5.0465675	9	Singlet	Mesothelial
SAT_26M	1892.3	1103	3.1479117	1	Singlet	Immune_cells
SAT_26M	1780	1146	2.0669263	1	Singlet	Immune_cells
SAT_26M	1574.2	912	1.3042962	0	Singlet	Adipocyte
SAT_26M	2269.6	1382	2.314138	1	Singlet	Immune_cells
SAT_26M	1252.7	821	1.6068269	1	Singlet	Immune_cells
SAT_26M	1035.8	715	5.5619902	5	Singlet	Immune_cells
SAT_26M	1789.8	1206	0.1593377	11	Singlet	Smooth_muscle_cells

SAT_26M	1985.1	1113	2.6479334	0	Singlet	Adipocyte
SAT_26M	1997	1398	3.5748238	5	Singlet	Immune_cells
SAT_26M	1014.3	710	5.1723631	1	Singlet	Immune_cells
SAT_26M	1522.7	919	5.4756157	0	Singlet	Adipocyte
SAT_26M	1112.4	774	6.8460541	1	Singlet	Immune_cells
SAT_26M	1099.6	715	8.7520155	1	Singlet	Immune_cells
SAT_26M	1469.7	984	2.7499842	11	Singlet	Smooth_muscle_cells
SAT_26M	1321.6	870	9.1023454	1	Singlet	Immune_cells
SAT_26M	2351	1272	0.3123457	0	Singlet	Adipocyte
SAT_26M	1045.3	776	3.8811418	4	Singlet	Immune_cells
SAT_26M	1213.9	820	3.1943201	1	Singlet	Immune_cells
SAT_26M	1500	1020	1.5980104	8	Singlet	Mesothelial
SAT_26M	1168.3	744	2.3487848	0	Singlet	Adipocyte
SAT_26M	3510.4	1984	4.7580444	9	Singlet	Mesothelial
SAT_26M	1381	949	0.0394823	8	Singlet	Mesothelial
SAT_26M	1700.1	1135	0.0598408	11	Singlet	Smooth_muscle_cells
SAT_26M	1216.3	816	8.4863693	4	Singlet	Immune_cells
SAT_26M	1468.8	978	3.6630827	9	Singlet	Mesothelial
SAT_26M	1251.2	865	3.0982843	9	Singlet	Mesothelial
SAT_26M	1656.3	1062	3.7754194	0	Singlet	Adipocyte
SAT_26M	1251.1	794	3.093158	7	Singlet	Endothelial_cells
SAT_26M	2353.9	1452	0.781215	0	Singlet	Adipocyte
SAT_26M	1191.2	813	0.2592974	8	Singlet	Mesothelial
SAT_26M	1270	1115	0.0167174	6	Singlet	Neurons
SAT_26M	2535.2	1266	7.1386796	0	Singlet	Adipocyte
SAT_26M	1405.7	991	1.9585082	5	Singlet	Immune_cells
SAT_26M	3071.7	1740	2.4176236	8	Singlet	Mesothelial
SAT_26M	1190.4	830	2.7600715	1	Singlet	Immune_cells
SAT_26M	1486.4	892	3.4668533	0	Singlet	Adipocyte
SAT_26M	1348.6	801	2.8943163	0	Singlet	Adipocyte
SAT_26M	1750.2	1032	2.8610528	0	Singlet	Adipocyte
SAT_26M	2623.8	1583	3.3328337	1	Singlet	Immune_cells
SAT_26M	2641.5	1256	0.5166068	10	Singlet	Immune_cells
SAT_26M	1309.6	956	3.1425264	8	Singlet	Mesothelial
SAT_26M	1612.3	1038	4.1563827	1	Singlet	Immune_cells
SAT_26M	4321.6	2370	5.4547002	9	Singlet	Mesothelial
SAT_26M	1184.7	745	4.9977446	0	Singlet	Adipocyte
SAT_26M	1811.7	1183	3.0471493	1	Singlet	Immune_cells
SAT_26M	3123.9	1743	0.0278512	0	Singlet	Adipocyte
SAT_26M	1083.9	762	6.517212	4	Singlet	Immune_cells
SAT_26M	1101.2	732	0.1689799	0	Singlet	Adipocyte
SAT_26M	1611.6	1136	1.7959263	1	Singlet	Immune_cells
SAT_26M	1117.6	721	1.1758167	0	Singlet	Adipocyte
SAT_26M	1416.9	878	8.7758361	1	Singlet	Immune_cells
SAT_26M	1440.2	924	0.2075416	3	Singlet	FAPs
SAT_26M	1979.4	1206	4.7056882	9	Singlet	Mesothelial
SAT_26M	1556.9	941	5.300498	5	Singlet	Immune_cells
SAT_26M	3053.8	1427	0.9580696	0	Singlet	Adipocyte
SAT_26M	2470.7	1262	5.1638088	10	Singlet	Immune_cells
SAT_26M	1394.8	891	3.4605564	7	Singlet	Endothelial_cells
SAT_26M	1222	770	4.3464572	0	Singlet	Adipocyte

SAT_26M	2751.1	1362	0	0	Singlet	Adipocyte
SAT_26M	3539.1	1593	2.8182052	10	Singlet	Immune_cells
SAT_26M	2246.8	1197	1.8607699	0	Singlet	Adipocyte
SAT_26M	2199.2	1336	4.3202307	1	Singlet	Immune_cells
SAT_26M	2138.7	1217	4.0223997	2	Singlet	FAPs
SAT_26M	1408.4	954	1.3880368	11	Singlet	Smooth_muscle_cells
SAT_26M	1470.6	967	0.8724489	1	Singlet	Immune_cells
SAT_26M	1087.6	834	7.7430416	6	Singlet	Neurons
SAT_26M	2868.5	1435	3.4795499	0	Singlet	Adipocyte
SAT_26M	1076.2	708	1.5401057	0	Singlet	Adipocyte
SAT_26M	1069.1	905	0.1432075	4	Singlet	Immune_cells
SAT_26M	1016.5	660	0.9163626	0	Singlet	Adipocyte
SAT_26M	1467.7	900	9.3160646	1	Singlet	Immune_cells
SAT_26M	2259.6	1356	4.4606862	1	Singlet	Immune_cells
SAT_26M	2250.7	1353	2.4549605	11	Singlet	Smooth_muscle_cells
SAT_26M	1743.4	826	0.9808704	10	Singlet	Immune_cells
SAT_26M	2700.2	1605	1.4772407	5	Singlet	Immune_cells
SAT_26M	1760.4	1035	9.9248295	0	Singlet	Adipocyte
SAT_26M	1781.5	1164	2.9647154	1	Singlet	Immune_cells
SAT_26M	1001.2	816	0.0550635	6	Singlet	Neurons
SAT_26M	1081.6	684	0.3170411	3	Singlet	FAPs
SAT_26M	1510.2	1059	14.609842	6	Singlet	Neurons
SAT_26M	3846.4	1474	3.3515631	10	Singlet	Immune_cells
SAT_26M	1346.5	1053	2.0137285	4	Singlet	Immune_cells
SAT_26M	1098.7	760	2.4879921	11	Singlet	Smooth_muscle_cells
SAT_26M	1534.3	908	1.0829467	0	Singlet	Adipocyte
SAT_26M	2043.8	1252	0.5008324	1	Singlet	Immune_cells
SAT_26M	1025.3	785	0.8007826	0	Singlet	Adipocyte
SAT_26M	7223.4	1954	2.4892028	10	Singlet	Immune_cells
SAT_26M	1472.3	912	0.510311	7	Singlet	Endothelial_cells
SAT_26M	1062	725	4.0658502	1	Singlet	Immune_cells
SAT_26M	1851.2	1170	3.4212029	1	Singlet	Immune_cells
SAT_26M	2836.3	1556	1.8454036	7	Singlet	Endothelial_cells
SAT_26M	1045.1	803	7.1482172	6	Singlet	Neurons
SAT_26M	1722.5	1096	1.2566956	1	Singlet	Immune_cells
SAT_26M	1951.3	1125	0.2185812	0	Singlet	Adipocyte
SAT_26M	1698.2	1065	2.3532783	9	Singlet	Mesothelial
SAT_26M	1721.6	1173	5.270022	1	Singlet	Immune_cells
SAT_26M	2743	1472	0.5064236	0	Singlet	Adipocyte
SAT_26M	1234.3	884	3.9079643	11	Singlet	Smooth_muscle_cells
SAT_26M	1191.9	769	0.5902412	0	Singlet	Adipocyte
SAT_26M	1560.9	1088	7.470066	9	Singlet	Mesothelial
SAT_26M	1594.6	995	5.4973685	1	Singlet	Immune_cells
SAT_26M	1464.7	945	8.6188244	4	Singlet	Immune_cells
SAT_26M	1857.8	1291	4.1964103	8	Singlet	Mesothelial
SAT_26M	1791	1137	3.0708698	1	Singlet	Immune_cells
SAT_26M	1295.5	860	4.872134	1	Singlet	Immune_cells
SAT_26M	1705	1100	5.6916724	1	Singlet	Immune_cells
SAT_26M	2334.2	1317	2.9437873	0	Singlet	Adipocyte
SAT_26M	1226	742	2.3876873	0	Singlet	Adipocyte
SAT_26M	1168	714	1.2080364	0	Singlet	Adipocyte

SAT_26M	1503.1	964	4.3013192	1	Singlet	Immune_cells
SAT_26M	1217.5	821	1.2510459	8	Singlet	Mesothelial
SAT_26M	1313.6	858	0	0	Singlet	Adipocyte
SAT_26M	3805.2	1566	2.4857376	10	Singlet	Immune_cells
SAT_26M	1252.8	767	3.8970771	0	Singlet	Adipocyte
SAT_26M	1278.9	920	0.0531587	7	Singlet	Endothelial_cells
SAT_26M	1497	976	0.7206744	5	Singlet	Immune_cells
SAT_26M	1009.4	682	7.4160878	1	Singlet	Immune_cells
SAT_26M	6088.1	2908	9.9269944	4	Singlet	Immune_cells
SAT_26M	3235.5	1760	0.6370763	0	Singlet	Adipocyte
SAT_26M	1036.2	656	2.4943829	3	Singlet	FAPs
SAT_26M	1246.7	846	3.7105684	1	Singlet	Immune_cells
SAT_26M	1115.3	797	8.7037184	4	Singlet	Immune_cells
SAT_26M	1714.4	1001	0.5650639	0	Singlet	Adipocyte
SAT_26M	2802.9	1394	1.4588699	0	Singlet	Adipocyte
SAT_26M	1147.1	767	8.7765564	1	Singlet	Immune_cells
SAT_26M	1753.2	988	14.545422	8	Singlet	Mesothelial
SAT_26M	1528.9	950	7.9271401	1	Singlet	Immune_cells
SAT_26M	1263.6	794	6.4727487	0	Singlet	Adipocyte
SAT_26M	1033.6	713	5.6861117	8	Singlet	Mesothelial
SAT_26M	2132.9	1294	13.987767	9	Singlet	Mesothelial
SAT_26M	1288.2	754	10.321755	0	Singlet	Adipocyte
SAT_26M	2600.9	1504	3.664364	8	Singlet	Mesothelial
SAT_26M	1312.6	802	1.3444502	0	Singlet	Adipocyte
SAT_26M	2587.9	1406	2.8331164	8	Singlet	Mesothelial
SAT_26M	2123.8	1274	3.8514705	1	Singlet	Immune_cells
SAT_26M	1299.1	879	5.1435979	1	Singlet	Immune_cells
SAT_26M	1339.8	797	5.0621037	0	Singlet	Adipocyte
SAT_26M	1322.9	769	13.283653	7	Singlet	Endothelial_cells
SAT_26M	2025	1236	6.07043	1	Singlet	Immune_cells
SAT_26M	2327.9	1399	3.587555	8	Singlet	Mesothelial
SAT_26M	1036.6	658	0	4	Singlet	Immune_cells
SAT_26M	2116.4	980	4.1080282	10	Singlet	Immune_cells
SAT_26M	1182.5	795	1.693869	0	Singlet	Adipocyte
SAT_26M	1474.6	863	9.5312636	1	Singlet	Immune_cells
SAT_26M	1261.1	866	4.080153	1	Singlet	Immune_cells
SAT_26M	1041.3	662	2.6586816	0	Singlet	Adipocyte
SAT_26M	1442.7	937	3.820308	1	Singlet	Immune_cells
SAT_26M	1478.2	996	0.9018311	1	Singlet	Immune_cells
SAT_26M	1106.8	816	5.9944443	4	Singlet	Immune_cells
SAT_26M	1170.8	817	2.5639953	0	Singlet	Adipocyte
SAT_26M	1293.2	837	0.2652962	0	Singlet	Adipocyte
SAT_26M	1084.1	758	6.2095084	8	Singlet	Mesothelial
SAT_26M	1210	819	2.1644702	1	Singlet	Immune_cells
SAT_26M	2419.9	1430	9.1707203	9	Singlet	Mesothelial
SAT_26M	2778.7	1541	0.0079165	0	Singlet	Adipocyte
SAT_26M	1231.7	854	1.2768405	1	Singlet	Immune_cells
SAT_26M	1229.1	784	3.5654295	7	Singlet	Endothelial_cells
SAT_26M	4668.4	2216	0	0	Singlet	Adipocyte
SAT_26M	1489	773	4.4251759	10	Singlet	Immune_cells
SAT_26M	2636.4	1302	5.9770264	10	Singlet	Immune_cells

SAT_26M	10044	4366	4.292902	8	Singlet	Mesothelial
SAT_26M	1159.6	732	0.1417698	0	Singlet	Adipocyte
SAT_26M	1123.7	783	5.9188736	1	Singlet	Immune_cells
SAT_26M	1031	650	0.0340878	0	Singlet	Adipocyte
SAT_26M	1194.7	803	7.2664816	1	Singlet	Immune_cells
SAT_26M	1297.8	745	9.1677068	10	Singlet	Immune_cells
SAT_26M	1003.6	709	2.8215936	8	Singlet	Mesothelial
SAT_26M	1569.2	1020	1.9170964	0	Singlet	Adipocyte
SAT_26M	3589.1	1551	1.856338	10	Singlet	Immune_cells
SAT_26M	1049.8	720	0.1211729	4	Singlet	Immune_cells
SAT_26M	1416.5	804	5.8428124	10	Singlet	Immune_cells
SAT_26M	1499.9	955	0.0333723	0	Singlet	Adipocyte
SAT_26M	1218.2	842	0.2447072	1	Singlet	Immune_cells
SAT_26M	1737	1123	0.7062854	8	Singlet	Mesothelial
SAT_26M	1143.4	738	0.3658099	0	Singlet	Adipocyte
SAT_26M	1733.4	1102	4.7465475	0	Singlet	Adipocyte
SAT_26M	2315	1341	1.1240795	7	Singlet	Endothelial_cells
SAT_26M	2430.1	1401	5.357185	0	Singlet	Adipocyte
SAT_26M	2140.2	1218	0.0441139	8	Singlet	Mesothelial
SAT_26M	1710	983	8.1332338	0	Singlet	Adipocyte
SAT_26M	4109.2	1690	1.0709723	10	Singlet	Immune_cells
SAT_26M	2055.3	1220	3.7431976	8	Singlet	Mesothelial
SAT_26M	3196.3	1545	0.8062005	10	Singlet	Immune_cells
SAT_26M	1082.8	767	2.7738561	1	Singlet	Immune_cells
SAT_26M	1135.8	777	0.0450491	11	Singlet	Smooth_muscle_cells
SAT_26M	1212.9	828	2.4361714	1	Singlet	Immune_cells
SAT_26M	1447.3	829	9.4255241	0	Singlet	Adipocyte
SAT_26M	2318.3	1246	0.2798208	2	Singlet	FAPs
SAT_26M	1360.8	798	7.7225386	10	Singlet	Immune_cells
SAT_26M	1049.5	731	1.3827819	1	Singlet	Immune_cells
SAT_26M	2191.6	1379	5.5322086	4	Singlet	Immune_cells
SAT_26M	1340.6	842	0.3108702	0	Singlet	Adipocyte
SAT_26M	1455	939	1.1276149	1	Singlet	Immune_cells
SAT_26M	3095.2	1741	7.7057294	7	Singlet	Endothelial_cells
SAT_26M	1097.8	699	13.468939	4	Singlet	Immune_cells
SAT_26M	1737.6	975	5.9798994	0	Singlet	Adipocyte
SAT_26M	1133.5	771	3.0138685	0	Singlet	Adipocyte
SAT_26M	1940.2	1236	4.6720805	1	Singlet	Immune_cells
SAT_26M	1166.1	779	6.4215779	1	Singlet	Immune_cells
SAT_26M	1786.8	1115	2.0424761	1	Singlet	Immune_cells
SAT_26M	1210.5	802	1.0161967	0	Singlet	Adipocyte
SAT_26M	2669.5	1420	1.5319668	0	Singlet	Adipocyte
SAT_26M	2035.2	1249	5.4491479	1	Singlet	Immune_cells
SAT_26M	1129.4	759	0.0632	0	Singlet	Adipocyte
SAT_26M	1186.4	889	2.1414117	11	Singlet	Smooth_muscle_cells
SAT_26M	1424.3	915	10.645758	1	Singlet	Immune_cells
SAT_26M	1100	702	2.9600787	0	Singlet	Adipocyte
SAT_26M	1056.2	631	14.275294	1	Singlet	Immune_cells
SAT_26M	3064.3	1794	3.3401484	9	Singlet	Mesothelial
SAT_26M	1232.2	771	2.133901	10	Singlet	Immune_cells
SAT_26M	2902.2	1622	1.4978978	0	Singlet	Adipocyte

SAT_26M	2302	1340	1.8382752	1	Singlet	Immune_cells
SAT_26M	2719.5	1484	0.3215612	0	Singlet	Adipocyte
SAT_26M	1263.4	863	1.4106867	1	Singlet	Immune_cells
SAT_26M	3056	1391	1.2484133	10	Singlet	Immune_cells
SAT_26M	1009.5	648	2.101876	0	Singlet	Adipocyte
SAT_26M	1755.7	1176	1.1915718	11	Singlet	Smooth_muscle_cells
SAT_26M	1687.3	1086	2.7857294	1	Singlet	Immune_cells
SAT_26M	1053.8	682	10.674037	5	Singlet	Immune_cells
SAT_26M	1252.3	760	3.8633979	10	Singlet	Immune_cells
SAT_26M	1702.7	865	12.155384	0	Singlet	Adipocyte
SAT_26M	1918.9	1167	2.2752376	0	Singlet	Adipocyte
SAT_26M	2482	1315	1.4251909	0	Singlet	Adipocyte
SAT_26M	2196.4	1301	2.3621531	1	Singlet	Immune_cells
SAT_26M	1313.7	868	2.5669277	5	Singlet	Immune_cells
SAT_26M	1152.3	798	3.8486892	1	Singlet	Immune_cells
SAT_26M	1345.6	891	0.6283846	5	Singlet	Immune_cells
SAT_26M	2853.1	1554	2.741068	0	Singlet	Adipocyte
SAT_26M	1036.8	750	9.1776587	1	Singlet	Immune_cells
SAT_26M	1155	813	4.4178053	4	Singlet	Immune_cells
SAT_26M	5611.2	1997	0.2556853	10	Singlet	Immune_cells
SAT_26M	1580.9	935	1.2699406	10	Singlet	Immune_cells
SAT_26M	1670.4	1035	3.2363322	8	Singlet	Mesothelial
SAT_26M	1534.6	984	1.9948438	1	Singlet	Immune_cells
SAT_26M	1824.7	996	1.5674431	0	Singlet	Adipocyte
SAT_26M	1036	657	0.1525619	0	Singlet	Adipocyte
SAT_26M	7853.3	3632	4.4174101	8	Singlet	Mesothelial
SAT_26M	1903.7	1186	1.2289825	1	Singlet	Immune_cells
SAT_26M	1400.7	805	5.2145421	10	Singlet	Immune_cells
SAT_26M	1970.5	1152	0.0937363	0	Singlet	Adipocyte
SAT_26M	2242.7	1316	2.8440741	5	Singlet	Immune_cells
SAT_26M	1347.2	1021	1.2716675	11	Singlet	Smooth_muscle_cells
SAT_26M	5605.1	2362	0.3134013	0	Singlet	Adipocyte
SAT_26M	1770.2	1057	0.1100035	0	Singlet	Adipocyte
SAT_26M	1528.9	1007	4.5912604	1	Singlet	Immune_cells
SAT_26M	1641.8	1050	1.2038555	0	Singlet	Adipocyte
SAT_26M	5374.1	2341	0.5672748	0	Singlet	Adipocyte
SAT_26M	1239.1	832	2.5637824	1	Singlet	Immune_cells
SAT_26M	1844.6	1210	0.5110769	0	Singlet	Adipocyte
SAT_26M	2468.5	1278	2.6034983	0	Singlet	Adipocyte
SAT_26M	2486.5	1230	5.4725576	0	Singlet	Adipocyte
SAT_26M	2189.4	1228	2.5610808	0	Singlet	Adipocyte
SAT_26M	1122.3	778	0.0495291	8	Singlet	Mesothelial
SAT_26M	1300.4	814	0.0509763	0	Singlet	Adipocyte
SAT_26M	2228	1350	1.8865422	1	Singlet	Immune_cells
SAT_26M	1167.4	823	0.0564837	8	Singlet	Mesothelial
SAT_26M	1448.4	895	0.1081675	0	Singlet	Adipocyte
SAT_26M	3674.2	1660	0.5059717	10	Singlet	Immune_cells
SAT_26M	1130.3	736	5.611856	1	Singlet	Immune_cells
SAT_26M	2903.2	1535	6.1540103	7	Singlet	Endothelial_cells
SAT_26M	2624.3	1414	0.3854578	0	Singlet	Adipocyte
SAT_26M	1898.1	1193	5.0380461	1	Singlet	Immune_cells

SAT_26M	1119.5	736	7.2414309	1	Singlet	Immune_cells
SAT_26M	2446.8	1471	7.4155693	1	Singlet	Immune_cells
SAT_26M	1018.8	762	7.4913556	6	Singlet	Neurons
SAT_26M	1385.8	883	0.6983569	0	Singlet	Adipocyte
SAT_26M	6171.4	2996	8.2490174	9	Singlet	Mesothelial
SAT_26M	1518.5	911	0	0	Singlet	Adipocyte
SAT_26M	4546.9	2142	1.3972181	0	Singlet	Adipocyte
SAT_26M	1903.3	1192	0.0344963	3	Singlet	FAPs
SAT_26M	1011.8	604	2.1730229	0	Singlet	Adipocyte
SAT_26M	1203.9	803	1.738067	1	Singlet	Immune_cells
SAT_26M	2723.8	1558	1.5420677	8	Singlet	Mesothelial
SAT_26M	1291.2	773	12.723561	10	Singlet	Immune_cells
SAT_26M	1101.5	782	3.0325806	9	Singlet	Mesothelial
SAT_26M	1423.5	854	0.5112548	0	Singlet	Adipocyte
SAT_26M	1191.6	741	0.6163418	0	Singlet	Adipocyte
SAT_26M	1400.7	844	9.8497768	1	Singlet	Immune_cells
SAT_26M	1367.5	826	0	0	Singlet	Adipocyte
SAT_26M	2595.4	1407	5.002007	1	Singlet	Immune_cells
SAT_26M	3148.3	1704	2.1372908	0	Singlet	Adipocyte
SAT_26M	1168.4	740	6.0490583	7	Singlet	Endothelial_cells
SAT_26M	1166.5	773	4.0345361	1	Singlet	Immune_cells
SAT_26M	1648.5	922	5.2250135	0	Singlet	Adipocyte
SAT_26M	1617.7	1005	10.407518	9	Singlet	Mesothelial
SAT_26M	1230.3	886	6.7035221	1	Singlet	Immune_cells
SAT_26M	1054.1	844	0.1510737	11	Singlet	Smooth_muscle_cells
SAT_26M	2207.3	1233	1.4167355	0	Singlet	Adipocyte
SAT_26M	3216.4	1348	4.9084738	10	Singlet	Immune_cells
SAT_26M	1052.4	727	13.9999	1	Singlet	Immune_cells
SAT_26M	1569.8	971	8.9364577	7	Singlet	Endothelial_cells
SAT_26M	1670.6	993	3.3164425	8	Singlet	Mesothelial
SAT_26M	5410	1837	0.7212308	10	Singlet	Immune_cells
SAT_26M	1473.2	954	4.0073274	8	Singlet	Mesothelial
SAT_26M	1201.4	842	1.3219975	1	Singlet	Immune_cells
SAT_26M	1904.9	1062	3.7045932	10	Singlet	Immune_cells
SAT_26M	1076.8	662	0	0	Singlet	Adipocyte
SAT_26M	1332.3	779	10.793924	5	Singlet	Immune_cells
SAT_26M	1268.6	905	3.9671425	8	Singlet	Mesothelial
SAT_26M	1461.1	893	3.2820063	7	Singlet	Endothelial_cells
SAT_26M	2153.2	1037	4.9413151	10	Singlet	Immune_cells
SAT_26M	1926.7	1203	3.1042045	8	Singlet	Mesothelial
SAT_26M	1566.6	1029	4.2667689	1	Singlet	Immune_cells
SAT_26M	3316.3	1473	0.6265922	10	Singlet	Immune_cells
SAT_26M	1393	863	0.1577195	0	Singlet	Adipocyte
SAT_26M	1311.9	758	3.5168774	0	Singlet	Adipocyte
SAT_26M	1024.7	744	3.0339669	1	Singlet	Immune_cells
SAT_26M	1939.2	1147	7.9449017	5	Singlet	Immune_cells
SAT_26M	2188	1335	5.8804031	1	Singlet	Immune_cells
SAT_26M	1711.4	1024	3.5893046	8	Singlet	Mesothelial
SAT_26M	3546.8	1715	0.4271175	5	Singlet	Immune_cells
SAT_26M	1033	760	0.1089947	11	Singlet	Smooth_muscle_cells
SAT_26M	1236.6	730	6.6646425	0	Singlet	Adipocyte

SAT_26M	3421.2	1866	1.2172327	8	Singlet	Mesothelial
SAT_26M	1490.9	856	6.5397831	0	Singlet	Adipocyte
SAT_26M	2337.9	1273	2.5400535	3	Singlet	FAPs
SAT_26M	1163.3	660	6.8177234	5	Singlet	Immune_cells
SAT_26M	2724.1	1547	6.2453975	9	Singlet	Mesothelial
SAT_26M	1179.7	709	0.2212032	10	Singlet	Immune_cells
SAT_26M	1484.9	1087	3.313536	8	Singlet	Mesothelial
SAT_26M	1149.4	745	1.5056737	3	Singlet	FAPs
SAT_26M	1310.6	762	3.3508516	0	Singlet	Adipocyte
SAT_26M	1184.1	771	0.7986878	1	Singlet	Immune_cells
SAT_26M	1582.8	1065	0.0962542	0	Singlet	Adipocyte
SAT_26M	2423	1186	5.6723654	10	Singlet	Immune_cells
SAT_26M	1064.9	879	0.0233334	6	Singlet	Neurons
SAT_26M	2585.4	1318	0.1728765	0	Singlet	Adipocyte
SAT_26M	3063.6	1595	0.031362	0	Singlet	Adipocyte
SAT_26M	2624.6	1438	2.408024	5	Singlet	Immune_cells
SAT_26M	2485.2	1100	2.2654187	10	Singlet	Immune_cells
SAT_26M	1039.4	607	0.6906272	3	Singlet	FAPs
SAT_26M	1557.2	980	2.6260152	1	Singlet	Immune_cells
SAT_26M	2647.2	1450	0.0112564	0	Singlet	Adipocyte
SAT_26M	1160.9	760	1.3377882	0	Singlet	Adipocyte
SAT_26M	1163.1	761	0.4363969	0	Singlet	Adipocyte
SAT_26M	2422.2	1364	3.514589	1	Singlet	Immune_cells
SAT_26M	5288	2167	0.0767028	0	Singlet	Adipocyte
SAT_26M	1306.2	852	10.254113	11	Singlet	Smooth_muscle_cells
SAT_26M	1388.1	856	14.236097	4	Singlet	Immune_cells
SAT_26M	2606.5	1354	0.1019786	0	Singlet	Adipocyte
SAT_26M	1804.2	1033	4.2008157	0	Singlet	Adipocyte
SAT_26M	1729.7	1111	0.2782388	8	Singlet	Mesothelial
SAT_26M	2568.7	1531	0	8	Singlet	Mesothelial
SAT_26M	2952.1	1635	3.3155858	8	Singlet	Mesothelial
SAT_26M	1192.5	553	6.7774625	3	Singlet	FAPs
SAT_26M	2190.3	1398	0.8009704	7	Singlet	Endothelial_cells
SAT_26M	1821.5	1224	2.3910652	1	Singlet	Immune_cells
SAT_26M	5265.1	2341	0.4801426	0	Singlet	Adipocyte
SAT_26M	1232	777	0.3508236	10	Singlet	Immune_cells
SAT_26M	3307.4	1714	0.3567146	0	Singlet	Adipocyte
SAT_26M	1496	796	7.0052355	10	Singlet	Immune_cells
SAT_26M	1395.2	1081	5.7898531	6	Singlet	Neurons
SAT_26M	1926.5	1136	6.3855928	10	Singlet	Immune_cells
SAT_26M	2268.7	1243	4.1336524	0	Singlet	Adipocyte
SAT_26M	1299.3	1058	0.1803442	6	Singlet	Neurons
SAT_26M	1338.6	859	5.0260864	0	Singlet	Adipocyte
SAT_26M	3353.6	1686	1.0568022	0	Singlet	Adipocyte
SAT_26M	1957.3	1109	9.3720595	7	Singlet	Endothelial_cells
SAT_26M	2292.5	1265	3.8636542	0	Singlet	Adipocyte
SAT_26M	1294.1	943	2.3567498	1	Singlet	Immune_cells
SAT_26M	1291.9	848	0.7317618	0	Singlet	Adipocyte
SAT_26M	1945.3	1161	5.0457147	1	Singlet	Immune_cells
SAT_26M	5291.7	2378	12.755501	9	Singlet	Mesothelial
SAT_26M	1142.8	732	12.323891	1	Singlet	Immune_cells

SAT_26M	1532.9	992	5.1084016	5	Singlet	Immune_cells
SAT_26M	1884.3	1333	1.0748735	11	Singlet	Smooth_muscle_cells
SAT_26M	2077.2	1065	5.692675	10	Singlet	Immune_cells
SAT_26M	1176.2	948	4.6009979	6	Singlet	Neurons
SAT_26M	4279.8	2051	2.6277092	2	Singlet	FAPs
SAT_26M	2115.1	1241	1.4878477	8	Singlet	Mesothelial
SAT_26M	2983.6	1556	0.0359094	0	Singlet	Adipocyte
SAT_26M	1744.7	1121	3.3818571	1	Singlet	Immune_cells
SAT_26M	1208.5	807	4.6258054	1	Singlet	Immune_cells
SAT_26M	1461.3	924	0.2614145	0	Singlet	Adipocyte
SAT_26M	1856.5	1147	3.8865504	7	Singlet	Endothelial_cells
SAT_26M	1875.6	1089	4.0242112	0	Singlet	Adipocyte
SAT_26M	1510.1	989	6.7517173	0	Singlet	Adipocyte
SAT_26M	1047.6	667	6.1769479	0	Singlet	Adipocyte
SAT_26M	1905	1179	1.2472181	0	Singlet	Adipocyte
SAT_26M	1691.8	978	0.1171242	0	Singlet	Adipocyte
SAT_26M	1122.3	722	0	0	Singlet	Adipocyte
SAT_26M	1434.5	875	0.4352615	0	Singlet	Adipocyte
SAT_26M	2835.9	1692	4.0911788	7	Singlet	Endothelial_cells
SAT_26M	1224.2	755	14.778877	1	Singlet	Immune_cells
SAT_26M	1137.2	802	0.8685309	5	Singlet	Immune_cells
SAT_26M	2596.7	1360	5.6782345	9	Singlet	Mesothelial
SAT_26M	4899.5	2179	6.4752781	8	Singlet	Mesothelial
SAT_26M	1385.4	774	2.7981925	0	Singlet	Adipocyte
SAT_26M	1400	915	0.0877742	8	Singlet	Mesothelial
SAT_26M	1073.9	711	3.7979854	1	Singlet	Immune_cells
SAT_26M	1235.4	781	0.2031067	0	Singlet	Adipocyte
SAT_26M	2040.7	1125	1.1981926	0	Singlet	Adipocyte
SAT_26M	1737.7	1014	1.4433327	0	Singlet	Adipocyte
SAT_26M	1745.2	1078	3.1840131	7	Singlet	Endothelial_cells
SAT_26M	1129.2	832	1.3159494	8	Singlet	Mesothelial
SAT_26M	1156.5	928	0.0436292	6	Singlet	Neurons
SAT_26M	1254.8	866	4.1466687	1	Singlet	Immune_cells
SAT_26M	1324.6	889	6.2252229	5	Singlet	Immune_cells
SAT_26M	1028.2	698	7.494851	4	Singlet	Immune_cells
SAT_26M	2343.3	1143	2.0404053	10	Singlet	Immune_cells
SAT_26M	1705.9	1122	0.0602276	8	Singlet	Mesothelial
SAT_26M	1252.5	729	2.1172124	3	Singlet	FAPs
SAT_26M	1192.7	785	5.3532496	1	Singlet	Immune_cells
SAT_26M	1813.6	1113	5.4123227	1	Singlet	Immune_cells
SAT_26M	2527.8	1243	0.4600624	10	Singlet	Immune_cells
SAT_26M	1302.9	770	2.3471968	7	Singlet	Endothelial_cells
SAT_26M	1021.7	702	6.7353346	1	Singlet	Immune_cells
SAT_26M	2256.8	1164	2.4132849	10	Singlet	Immune_cells
SAT_26M	2665.8	1454	7.7138695	7	Singlet	Endothelial_cells
SAT_26M	4387.2	2249	2.3807488	1	Singlet	Immune_cells
SAT_26M	5076.9	2332	0.6528532	0	Singlet	Adipocyte
SAT_26M	6512.1	2225	0.8275504	10	Singlet	Immune_cells
SAT_26M	3112	1530	0.7019316	0	Singlet	Adipocyte
SAT_26M	2599.2	1256	10.535786	0	Singlet	Adipocyte
SAT_26M	1482.8	868	4.693461	0	Singlet	Adipocyte

SAT_26M	1007.7	719	5.5952966	1	Singlet	Immune_cells
SAT_26M	2022.2	1474	1.9743405	11	Singlet	Smooth_muscle_cells
SAT_26M	2237.6	1194	4.3126047	0	Singlet	Adipocyte
SAT_26M	3469.7	1675	11.013143	0	Singlet	Adipocyte
SAT_26M	1336.2	821	14.600888	4	Singlet	Immune_cells
SAT_26M	2748	1602	3.6803611	1	Singlet	Immune_cells
SAT_26M	2336.6	1222	0.8066564	0	Singlet	Adipocyte
SAT_26M	1027.8	674	3.9700701	1	Singlet	Immune_cells
SAT_26M	1852.2	1087	0.9913464	0	Singlet	Adipocyte
SAT_26M	3120.4	1583	0.5114483	0	Singlet	Adipocyte
SAT_26M	1370.2	984	1.653016	4	Singlet	Immune_cells
SAT_26M	1198	826	2.5186019	1	Singlet	Immune_cells
SAT_26M	1208.9	736	0.0523654	0	Singlet	Adipocyte
SAT_26M	1168.2	743	0.1369608	0	Singlet	Adipocyte
SAT_26M	1137.3	734	1.1609722	0	Singlet	Adipocyte
SAT_26M	1608.9	923	1.517923	0	Singlet	Adipocyte
SAT_26M	1734.1	1109	4.2860397	1	Singlet	Immune_cells
SAT_26M	1260	886	2.8328237	1	Singlet	Immune_cells
SAT_26M	1585.1	920	1.7315411	2	Singlet	FAPs
SAT_26M	1217.8	842	0.3220059	5	Singlet	Immune_cells
SAT_26M	1068	747	6.2134299	1	Singlet	Immune_cells
SAT_26M	1723.7	1143	1.9053803	1	Singlet	Immune_cells
SAT_26M	2426.1	1394	1.8692106	8	Singlet	Mesothelial
SAT_26M	1030.2	626	2.8867531	0	Singlet	Adipocyte
SAT_26M	2593.4	1489	1.53514	2	Singlet	FAPs
SAT_26M	1701.4	963	5.4696739	0	Singlet	Adipocyte
SAT_26M	1163.7	746	7.4960942	0	Singlet	Adipocyte
SAT_26M	2116.3	1142	6.8344044	0	Singlet	Adipocyte
SAT_26M	1693.5	985	9.9053291	4	Singlet	Immune_cells
SAT_26M	1604.2	1002	11.581688	4	Singlet	Immune_cells
SAT_26M	1249.9	771	8.258154	7	Singlet	Endothelial_cells
SAT_26M	1505.2	985	2.2439749	8	Singlet	Mesothelial
SAT_26M	1060	748	3.4231948	1	Singlet	Immune_cells
SAT_26M	2656.6	1138	3.6475744	10	Singlet	Immune_cells
SAT_26M	2017.2	1137	0.5416683	0	Singlet	Adipocyte
SAT_26M	1266.3	893	7.0627979	4	Singlet	Immune_cells
SAT_26M	1021.3	681	8.3657312	1	Singlet	Immune_cells
SAT_26M	1114.5	667	2.7565967	4	Singlet	Immune_cells
SAT_26M	1147	765	5.6093331	1	Singlet	Immune_cells
SAT_26M	5124.1	2606	4.0964166	8	Singlet	Mesothelial
SAT_26M	1821.2	1055	7.0971498	0	Singlet	Adipocyte
SAT_26M	1168.8	853	0.6707281	9	Singlet	Mesothelial
SAT_26M	2067.2	1247	1.6972323	1	Singlet	Immune_cells
SAT_26M	1162.5	777	6.4729408	1	Singlet	Immune_cells
SAT_26M	1819.9	1141	6.0151429	1	Singlet	Immune_cells
SAT_26M	1058.6	885	0.4165205	6	Singlet	Neurons
SAT_26M	1905.8	1105	1.7048698	2	Singlet	FAPs
SAT_26M	4403.5	1838	5.7419915	10	Singlet	Immune_cells
SAT_26M	1559.1	1169	2.3223694	4	Singlet	Immune_cells
SAT_26M	1116.4	714	0.4991524	10	Singlet	Immune_cells
SAT_26M	1183.3	788	0.058229	0	Singlet	Adipocyte

SAT_26M	1093.8	764	0.0689086	8	Singlet	Mesothelial
SAT_26M	2573.1	1246	2.7268056	0	Singlet	Adipocyte
SAT_26M	1268.6	823	5.2311362	1	Singlet	Immune_cells
SAT_26M	1005.1	685	7.817257	1	Singlet	Immune_cells
SAT_26M	1492.9	1246	1.1397217	6	Singlet	Neurons
SAT_26M	2773.8	1565	0.0264644	8	Singlet	Mesothelial
SAT_26M	1347.2	896	2.8027024	3	Singlet	FAPs
SAT_26M	1014.6	689	2.123821	0	Singlet	Adipocyte
SAT_26M	2009.7	1319	4.6584917	5	Singlet	Immune_cells
SAT_26M	1405.6	890	0.0038305	0	Singlet	Adipocyte
SAT_26M	2387.7	1232	0.0515216	0	Singlet	Adipocyte
SAT_26M	2119.4	1270	5.1674876	1	Singlet	Immune_cells
SAT_26M	1932.4	1361	2.4451411	9	Singlet	Mesothelial
SAT_26M	3420.9	1792	11.79581	4	Singlet	Immune_cells
SAT_26M	1014.7	663	1.8567567	9	Singlet	Mesothelial
SAT_26M	2191	1211	0.6179828	0	Singlet	Adipocyte
SAT_26M	2446.6	1124	1.2789958	10	Singlet	Immune_cells
SAT_26M	3083.8	1599	0.345813	0	Singlet	Adipocyte
SAT_26M	1011	668	0.2602004	0	Singlet	Adipocyte
SAT_26M	1400.9	798	4.9589248	10	Singlet	Immune_cells
SAT_26M	1623	897	12.109087	10	Singlet	Immune_cells
SAT_26M	1501.5	924	9.7296898	5	Singlet	Immune_cells
SAT_26M	3553.3	1877	0.8681218	0	Singlet	Adipocyte
SAT_26M	1721.7	960	5.2909268	0	Singlet	Adipocyte
SAT_26M	1080.4	746	4.369838	1	Singlet	Immune_cells
SAT_26M	1317.3	900	2.2207498	0	Singlet	Adipocyte
SAT_26M	1158.3	889	5.1830943	6	Singlet	Neurons
SAT_26M	2031.1	1222	0.4360522	0	Singlet	Adipocyte
SAT_26M	1307.9	853	3.4580234	1	Singlet	Immune_cells
SAT_26M	4967.3	2105	3.1027306	10	Singlet	Immune_cells
SAT_26M	1245.6	861	6.2052283	1	Singlet	Immune_cells
SAT_26M	1186.7	789	0.2895271	5	Singlet	Immune_cells
SAT_26M	1793.1	1102	0.2389538	0	Singlet	Adipocyte
SAT_26M	2517	1388	2.2584809	7	Singlet	Endothelial_cells
SAT_26M	1943	1049	3.8813055	0	Singlet	Adipocyte
SAT_26M	1174.5	766	0.1891568	4	Singlet	Immune_cells
SAT_26M	1013.5	785	0.0960428	4	Singlet	Immune_cells
SAT_26M	5437.8	2707	5.7833701	8	Singlet	Mesothelial
SAT_26M	4057.5	1802	2.7327838	10	Singlet	Immune_cells
SAT_26M	2058.8	1295	2.5644012	1	Singlet	Immune_cells
SAT_26M	1449.2	895	0.1074749	0	Singlet	Adipocyte
SAT_26M	2278.2	1179	0.2471296	10	Singlet	Immune_cells
SAT_26M	1069.6	769	2.6595833	1	Singlet	Immune_cells
SAT_26M	3018.7	1801	3.2508798	8	Singlet	Mesothelial
SAT_26M	1383.6	1017	1.4974502	4	Singlet	Immune_cells
SAT_26M	1968.3	1219	0	8	Singlet	Mesothelial
SAT_26M	1974.7	1155	3.398432	0	Singlet	Adipocyte
SAT_26M	1874.4	1145	4.3313527	1	Singlet	Immune_cells
SAT_26M	1046.1	653	2.6480857	7	Singlet	Endothelial_cells
SAT_26M	1037.9	670	0.5105726	0	Singlet	Adipocyte
SAT_26M	1108.3	723	6.2099063	1	Singlet	Immune_cells

SAT_26M	2320.3	1195	14.978912	5	Singlet	Immune_cells
SAT_26M	2880.5	1388	9.0548245	5	Singlet	Immune_cells
SAT_26M	1380.6	1117	0	4	Singlet	Immune_cells
SAT_26M	1413.7	871	0.3648348	0	Singlet	Adipocyte
SAT_26M	2199.9	1304	3.1765591	1	Singlet	Immune_cells
SAT_26M	1500	872	2.9701863	0	Singlet	Adipocyte
SAT_26M	1761.2	1120	4.5548412	8	Singlet	Mesothelial
SAT_26M	1685.9	1008	0.6070211	2	Singlet	FAPs
SAT_26M	1070	683	0.0210857	3	Singlet	FAPs
SAT_26M	1846.5	1157	2.7197445	8	Singlet	Mesothelial
SAT_26M	2059.6	1149	1.9092276	0	Singlet	Adipocyte
SAT_26M	1135.8	598	0.9138825	10	Singlet	Immune_cells
SAT_26M	1290.9	774	9.07219	10	Singlet	Immune_cells
SAT_26M	3782.5	1676	3.4431033	10	Singlet	Immune_cells
SAT_26M	1050.7	737	0	8	Singlet	Mesothelial
SAT_26M	1017.4	703	13.10171	9	Singlet	Mesothelial
SAT_26M	1662	945	13.618751	4	Singlet	Immune_cells
SAT_26M	1163	787	3.4417129	5	Singlet	Immune_cells
SAT_26M	1161.4	761	0.1616748	0	Singlet	Adipocyte
SAT_26M	1072	688	5.442975	10	Singlet	Immune_cells
SAT_26M	1897.9	1148	3.7811484	1	Singlet	Immune_cells
SAT_26M	1148.3	748	5.5130422	1	Singlet	Immune_cells
SAT_26M	1221.7	858	0.0627802	11	Singlet	Smooth_muscle_cells
SAT_26M	1030.4	667	0.119961	3	Singlet	FAPs
SAT_26M	1163.6	578	8.1145432	10	Singlet	Immune_cells
SAT_26M	2938.4	1631	10.293328	4	Singlet	Immune_cells
SAT_26M	1025.8	713	4.7735989	11	Singlet	Smooth_muscle_cells
SAT_26M	1319.9	901	1.0304106	1	Singlet	Immune_cells
SAT_26M	1191.8	834	0.0645693	8	Singlet	Mesothelial
SAT_26M	1216.7	823	0.0182068	8	Singlet	Mesothelial
SAT_26M	1178.1	757	1.3897157	0	Singlet	Adipocyte
SAT_26M	1218.6	837	2.4635793	8	Singlet	Mesothelial
SAT_26M	2119.1	1145	3.1637697	2	Singlet	FAPs
SAT_26M	3615.5	1614	1.9021673	10	Singlet	Immune_cells
SAT_26M	1209.1	832	3.1242309	1	Singlet	Immune_cells
SAT_26M	1303.3	955	3.3623193	0	Singlet	Adipocyte
SAT_26M	1125.4	633	9.080521	10	Singlet	Immune_cells
SAT_26M	2704.8	1565	3.3414963	9	Singlet	Mesothelial
SAT_26M	1105.2	817	4.9657609	1	Singlet	Immune_cells
SAT_26M	1502.3	970	2.8991622	1	Singlet	Immune_cells
SAT_26M	1263.8	921	9.0381574	4	Singlet	Immune_cells
SAT_26M	2099.6	1258	0.0185796	8	Singlet	Mesothelial
SAT_26M	1675.5	1103	0.2894646	11	Singlet	Smooth_muscle_cells
SAT_26M	1302.4	792	1.3061811	0	Singlet	Adipocyte
SAT_26M	1807.3	1141	8.0758063	1	Singlet	Immune_cells
SAT_26M	1671.3	1017	0	0	Singlet	Adipocyte
SAT_26M	2845.3	1434	7.420068	0	Singlet	Adipocyte
SAT_26M	1772.6	1117	0.5938563	8	Singlet	Mesothelial
SAT_26M	2024.9	992	0.3975984	10	Singlet	Immune_cells
SAT_26M	2579.2	1603	6.1144086	1	Singlet	Immune_cells
SAT_26M	1179.1	890	8.3161098	6	Singlet	Neurons

SAT_26M	2755.9	1536	9.9409423	1	Singlet	Immune_cells
SAT_26M	1224.7	863	13.638252	6	Singlet	Neurons
SAT_26M	2965.9	1709	2.655622	1	Singlet	Immune_cells
SAT_26M	3422.1	1849	7.4557173	8	Singlet	Mesothelial
SAT_26M	1177.1	802	2.5715581	0	Singlet	Adipocyte
SAT_26M	1634.8	934	0.1566446	0	Singlet	Adipocyte
SAT_26M	1745.7	1119	2.776305	4	Singlet	Immune_cells
SAT_26M	1420.7	1007	2.6600293	8	Singlet	Mesothelial
SAT_26M	3208.7	1976	0.6269218	2	Singlet	FAPs
SAT_26M	1967.5	1163	3.2601083	2	Singlet	FAPs
SAT_26M	1123.5	741	0	0	Singlet	Adipocyte
SAT_26M	1825.1	1065	0.120737	0	Singlet	Adipocyte
SAT_26M	1173	735	4.7080687	0	Singlet	Adipocyte
SAT_26M	1072.3	651	2.7668854	0	Singlet	Adipocyte
SAT_26M	1431.4	780	4.0242696	10	Singlet	Immune_cells
SAT_26M	1183.2	795	2.2498763	3	Singlet	FAPs
SAT_26M	1756.6	1058	0.6871777	0	Singlet	Adipocyte
SAT_26M	1828	961	2.2680007	10	Singlet	Immune_cells
SAT_26M	2346.8	1327	2.3447289	1	Singlet	Immune_cells
SAT_26M	2517.3	1325	0.9936667	0	Singlet	Adipocyte
SAT_26M	1180.3	885	1.1080152	6	Singlet	Neurons
SAT_26M	1567.2	829	12.19525	10	Singlet	Immune_cells
SAT_26M	2215	1295	4.6470973	8	Singlet	Mesothelial
SAT_26M	1186.5	773	2.0352573	1	Singlet	Immune_cells
SAT_26M	2828.2	1412	1.3641751	0	Singlet	Adipocyte
SAT_26M	1143.5	784	4.3989265	1	Singlet	Immune_cells
SAT_26M	2796.9	1576	6.7469873	7	Singlet	Endothelial_cells
SAT_26M	2956.6	1318	6.1680988	10	Singlet	Immune_cells
SAT_26M	1011.7	749	0.196714	6	Singlet	Neurons
SAT_26M	1175.8	888	5.8667765	6	Singlet	Neurons
SAT_26M	1178.5	698	8.2280217	10	Singlet	Immune_cells
SAT_26M	1162.1	804	0.8481232	8	Singlet	Mesothelial
SAT_26M	1181.3	792	0.5214074	0	Singlet	Adipocyte
SAT_26M	1125.8	905	0	6	Singlet	Neurons
SAT_26M	2693	1336	0.4142508	10	Singlet	Immune_cells
SAT_26M	1214.3	748	7.7427827	0	Singlet	Adipocyte
SAT_26M	3623.2	1969	10.846157	5	Singlet	Immune_cells
SAT_26M	1662.2	1040	1.6135713	4	Singlet	Immune_cells
SAT_26M	1225	765	0.6776537	0	Singlet	Adipocyte
SAT_26M	1962.1	1139	0.3402664	0	Singlet	Adipocyte
SAT_26M	1211.7	813	1.951152	1	Singlet	Immune_cells
SAT_26M	1064.5	674	1.2934285	0	Singlet	Adipocyte
SAT_26M	1136.4	789	1.8851429	1	Singlet	Immune_cells
SAT_26M	2937.6	1335	4.446702	10	Singlet	Immune_cells
SAT_26M	1029.9	680	0.8801425	0	Singlet	Adipocyte
SAT_26M	1932.7	1118	0.6531524	0	Singlet	Adipocyte
SAT_26M	1229.3	654	0.3210067	10	Singlet	Immune_cells
SAT_26M	1620.4	1084	0.3818621	4	Singlet	Immune_cells
SAT_26M	1623.6	979	2.5178332	10	Singlet	Immune_cells
SAT_26M	1097.3	760	7.9709086	4	Singlet	Immune_cells
SAT_26M	1461.6	929	2.3835233	8	Singlet	Mesothelial

SAT_26M	1133.1	691	8.0458753	10	Singlet	Immune_cells
SAT_26M	2398.6	1575	6.2925728	8	Singlet	Mesothelial
SAT_26M	1484.6	978	6.0335325	1	Singlet	Immune_cells
SAT_26M	1196.9	775	4.7418577	3	Singlet	FAPs
SAT_26M	3472.2	1668	4.8874013	0	Singlet	Adipocyte
SAT_26M	3378.7	1779	6.2826763	9	Singlet	Mesothelial
SAT_26M	1296.5	870	10.208714	4	Singlet	Immune_cells
SAT_26M	1340	885	0.2245404	0	Singlet	Adipocyte
SAT_26M	4085.7	2011	0.9018083	7	Singlet	Endothelial_cells
SAT_26M	1933.2	1291	2.088285	1	Singlet	Immune_cells
SAT_26M	2102	1141	1.4165779	0	Singlet	Adipocyte
SAT_26M	2825.8	1298	2.7025711	10	Singlet	Immune_cells
SAT_26M	1424.8	912	1.9237961	1	Singlet	Immune_cells
SAT_26M	1887.5	1142	0.2165285	0	Singlet	Adipocyte
SAT_26M	1267	949	3.7263904	8	Singlet	Mesothelial
SAT_26M	2144.8	1406	2.3839334	8	Singlet	Mesothelial
SAT_26M	1641.3	1015	3.4692508	1	Singlet	Immune_cells
SAT_26M	2363	1327	0.5770297	0	Singlet	Adipocyte
SAT_26M	1203.8	726	5.5087023	10	Singlet	Immune_cells
SAT_26M	1241.3	865	9.1644044	5	Singlet	Immune_cells
SAT_26M	2718.7	1562	0.0597452	0	Singlet	Adipocyte
SAT_26M	1744.3	1036	6.8311061	5	Singlet	Immune_cells
SAT_26M	1428.1	825	1.7933221	10	Singlet	Immune_cells
SAT_26M	2017.3	1131	4.3874216	4	Singlet	Immune_cells
SAT_26M	1060	733	0.1035538	9	Singlet	Mesothelial
SAT_26M	1294.5	865	3.6836055	1	Singlet	Immune_cells
SAT_26M	2677.2	1501	0.1209417	0	Singlet	Adipocyte
SAT_26M	1370.6	856	2.7639776	0	Singlet	Adipocyte
SAT_26M	1198.7	778	1.4630797	1	Singlet	Immune_cells
SAT_26M	1076.6	703	0.8779139	1	Singlet	Immune_cells
SAT_26M	1112.9	710	7.870071	1	Singlet	Immune_cells
SAT_26M	1225	772	10.762991	4	Singlet	Immune_cells
SAT_26M	1303.4	1073	1.8437052	6	Singlet	Neurons
SAT_26M	2783.3	1537	0.4946798	0	Singlet	Adipocyte
SAT_26M	1560.3	983	5.8808636	1	Singlet	Immune_cells
SAT_26M	6144.9	3009	2.3570291	8	Singlet	Mesothelial
SAT_26M	1103.7	766	5.8269786	4	Singlet	Immune_cells
SAT_26M	1314.7	835	8.5725053	1	Singlet	Immune_cells
SAT_26M	2088.5	1243	1.6247926	0	Singlet	Adipocyte
SAT_26M	2447.2	1323	0.0067086	0	Singlet	Adipocyte
SAT_26M	1487.1	959	4.9054608	1	Singlet	Immune_cells
SAT_26M	1636.7	1065	2.6299177	1	Singlet	Immune_cells
SAT_26M	1993.5	1179	0.0528277	0	Singlet	Adipocyte
SAT_26M	1279.7	972	4.3194633	8	Singlet	Mesothelial
SAT_26M	2682	1315	1.3285791	10	Singlet	Immune_cells
SAT_26M	1151.4	732	1.8493284	0	Singlet	Adipocyte
SAT_26M	1117.9	746	2.1129717	0	Singlet	Adipocyte
SAT_26M	5104	2632	8.2623659	9	Singlet	Mesothelial
SAT_26M	2438.9	1418	0.0721914	8	Singlet	Mesothelial
SAT_26M	1179.1	836	0.2750891	11	Singlet	Smooth_muscle_cells
SAT_26M	1087.7	758	9.0381061	1	Singlet	Immune_cells

SAT_26M	1134.9	694	3.978799	0	Singlet	Adipocyte
SAT_26M	1167.4	825	0.3891557	11	Singlet	Smooth_muscle_cells
SAT_26M	1161.2	763	4.3972766	0	Singlet	Adipocyte
SAT_26M	1460.5	950	2.4481309	1	Singlet	Immune_cells
SAT_26M	2723.2	1509	0.4267924	0	Singlet	Adipocyte
SAT_26M	3152.1	1500	9.4333798	0	Singlet	Adipocyte
SAT_26M	3414.3	1676	1.0595438	0	Singlet	Adipocyte
SAT_26M	1272.5	789	2.4880076	1	Singlet	Immune_cells
SAT_26M	6690.3	3046	8.228755	9	Singlet	Mesothelial
SAT_26M	3165.5	1525	4.2866039	0	Singlet	Adipocyte
SAT_26M	2411.1	1338	0.689681	0	Singlet	Adipocyte
SAT_26M	2436.6	1410	0.0190025	8	Singlet	Mesothelial
SAT_26M	2013.1	1227	0.0771733	8	Singlet	Mesothelial
SAT_26M	1639.4	1067	2.3912057	0	Singlet	Adipocyte
SAT_26M	3275.6	1929	0.7311843	5	Singlet	Immune_cells
SAT_26M	1560.3	1051	0	0	Singlet	Adipocyte
SAT_26M	1562.3	706	10.640668	0	Singlet	Adipocyte
SAT_26M	4365.7	2179	0	0	Singlet	Adipocyte
SAT_26M	3092.3	1707	0.8111323	0	Singlet	Adipocyte
SAT_26M	3589.3	1617	11.985054	7	Singlet	Endothelial_cells
SAT_26M	3406.1	1941	3.0496591	0	Singlet	Adipocyte
SAT_26M	1657.3	1019	2.257853	4	Singlet	Immune_cells
SAT_26M	2389.1	1374	0.8658213	5	Singlet	Immune_cells
SAT_26M	1219	717	6.6933892	7	Singlet	Endothelial_cells
SAT_26M	1987.6	1145	5.4961328	0	Singlet	Adipocyte
SAT_26M	1356.3	844	0.0218469	0	Singlet	Adipocyte
SAT_26M	2173.4	1284	2.0885622	7	Singlet	Endothelial_cells
SAT_26M	1004.9	685	0.2147292	8	Singlet	Mesothelial
SAT_26M	2781	1483	8.6250756	0	Singlet	Adipocyte
SAT_26M	1921.8	1051	2.2316637	10	Singlet	Immune_cells
SAT_26M	1080.4	715	0.0666234	0	Singlet	Adipocyte
SAT_26M	1157.9	803	2.5066343	5	Singlet	Immune_cells
SAT_26M	3459.8	1817	0	8	Singlet	Mesothelial
SAT_26M	1130.9	720	8.6695968	3	Singlet	FAPs
SAT_26M	1640.8	1019	6.6872668	5	Singlet	Immune_cells
SAT_26M	2020.1	1304	1.4204183	3	Singlet	FAPs
SAT_26M	1771.9	943	10.796781	7	Singlet	Endothelial_cells
SAT_26M	1262.9	807	4.534622	0	Singlet	Adipocyte
SAT_26M	3543.8	1780	0.4166997	0	Singlet	Adipocyte
SAT_26M	4862.4	2369	7.7900405	8	Singlet	Mesothelial
SAT_26M	1023.9	622	4.665684	8	Singlet	Mesothelial
SAT_26M	1194.7	814	0.0126997	11	Singlet	Smooth_muscle_cells
SAT_26M	1325.6	793	7.4856913	0	Singlet	Adipocyte
SAT_26M	1151.3	713	0.5019883	5	Singlet	Immune_cells
SAT_26M	1419.9	720	8.9230964	0	Singlet	Adipocyte
SAT_26M	1462.3	980	1.3044642	0	Singlet	Adipocyte
SAT_26M	3518.6	1768	3.124458	10	Singlet	Immune_cells
SAT_26M	1405.4	894	3.7334161	7	Singlet	Endothelial_cells
SAT_26M	1062.2	609	4.2096238	10	Singlet	Immune_cells
SAT_26M	1130.1	828	3.8232914	1	Singlet	Immune_cells
SAT_26M	1248	857	0	0	Singlet	Adipocyte

SAT_26M	1221.8	775	0.0550619	0	Singlet	Adipocyte
SAT_26M	1135.3	758	1.9798213	5	Singlet	Immune_cells
SAT_26M	3700.1	2095	6.2467578	9	Singlet	Mesothelial
SAT_26M	4342.6	1886	10.93013	7	Singlet	Endothelial_cells
SAT_26M	1202.8	763	2.6300184	0	Singlet	Adipocyte
SAT_26M	1399.9	960	1.2356167	7	Singlet	Endothelial_cells
SAT_26M	1775.5	1257	1.9825632	4	Singlet	Immune_cells
SAT_26M	1098.8	689	0.0530721	3	Singlet	FAPs
SAT_26M	3221.2	1666	5.1698898	0	Singlet	Adipocyte
SAT_26M	1268.6	865	1.0486331	0	Singlet	Adipocyte
SAT_26M	2132.3	1347	0.1554891	3	Singlet	FAPs
SAT_26M	1173.2	838	0	8	Singlet	Mesothelial
SAT_26M	1275.4	858	5.2226341	3	Singlet	FAPs
SAT_26M	1764.9	1039	3.6002832	7	Singlet	Endothelial_cells
SAT_26M	3636.5	1866	9.2423251	7	Singlet	Endothelial_cells
SAT_26M	1244.9	701	14.522408	7	Singlet	Endothelial_cells
SAT_26M	1487.9	925	4.2733885	0	Singlet	Adipocyte
SAT_26M	3435.9	1761	0.2327531	0	Singlet	Adipocyte
SAT_26M	1661.2	925	6.3760669	7	Singlet	Endothelial_cells
SAT_26M	1704.8	966	1.8664149	10	Singlet	Immune_cells
SAT_26M	1145.5	535	13.660301	0	Singlet	Adipocyte
SAT_26M	1344.9	941	2.0738844	3	Singlet	FAPs
SAT_26M	1712.9	1028	5.0106356	0	Singlet	Adipocyte
SAT_26M	1068.7	734	0.0220829	8	Singlet	Mesothelial
SAT_26M	1517	1032	0.1164578	0	Singlet	Adipocyte
SAT_26M	1371.4	900	0.161957	0	Singlet	Adipocyte
SAT_26M	1540.4	991	0.1705156	0	Singlet	Adipocyte
SAT_26M	1501.7	963	0.013441	8	Singlet	Mesothelial
SAT_26M	4639.5	2173	10.20915	9	Singlet	Mesothelial
SAT_26M	2929.3	1350	8.8785103	0	Singlet	Adipocyte
SAT_26M	1713.4	1108	1.2945231	3	Singlet	FAPs
SAT_26M	2630.1	1661	0.1443637	5	Singlet	Immune_cells
SAT_26M	2770.8	1679	4.0221926	8	Singlet	Mesothelial
SAT_26M	1534.9	1129	0.1138573	1	Singlet	Immune_cells
SAT_26M	1577.1	983	8.3205454	9	Singlet	Mesothelial
SAT_26M	1280.1	850	8.5024905	4	Singlet	Immune_cells
SAT_26M	5955.3	2525	0.6584094	0	Singlet	Adipocyte
SAT_26M	1121.6	681	9.0144139	7	Singlet	Endothelial_cells
SAT_26M	1645.1	968	5.6864732	10	Singlet	Immune_cells
SAT_26M	1374.5	919	1.3448732	0	Singlet	Adipocyte
SAT_26M	2954.8	1549	1.6218613	0	Singlet	Adipocyte
SAT_26M	1737.3	1122	1.7723436	8	Singlet	Mesothelial
SAT_26M	1953.6	1117	7.3449654	10	Singlet	Immune_cells
SAT_26M	2214.8	1354	4.8573141	7	Singlet	Endothelial_cells
SAT_26M	1712.8	979	0.3568805	0	Singlet	Adipocyte
SAT_26M	1941.7	1118	0.0254109	0	Singlet	Adipocyte
SAT_26M	1495.7	897	7.9213254	7	Singlet	Endothelial_cells
SAT_26M	1061.4	740	1.450307	0	Singlet	Adipocyte
SAT_26M	3409.8	1580	1.3061877	10	Singlet	Immune_cells
SAT_26M	1786.2	1102	0.9225619	0	Singlet	Adipocyte
SAT_26M	1015.5	625	8.44781	3	Singlet	FAPs

SAT_26M	1400	833	0.0560396	0	Singlet	Adipocyte
SAT_26M	1060.5	751	0.134854	0	Singlet	Adipocyte
SAT_26M	1163	797	0.1180406	8	Singlet	Mesothelial
SAT_26M	3079.4	1743	6.3902529	7	Singlet	Endothelial_cells
SAT_26M	2120.2	1209	0	0	Singlet	Adipocyte
SAT_26M	1698.6	1084	1.0735043	0	Singlet	Adipocyte
SAT_26M	4117.6	2104	1.4532962	0	Singlet	Adipocyte
SAT_26M	1701.1	1086	1.6928412	7	Singlet	Endothelial_cells
SAT_26M	1689	1096	0	0	Singlet	Adipocyte
SAT_26M	5269.9	2377	5.2676455	7	Singlet	Endothelial_cells
SAT_26M	2012.8	1272	2.25459	0	Singlet	Adipocyte
SAT_26M	1098.4	711	8.8675471	7	Singlet	Endothelial_cells
SAT_26M	2946.9	1673	0.6983469	0	Singlet	Adipocyte
SAT_26M	1567.6	953	1.9494043	0	Singlet	Adipocyte
SAT_26M	1230.7	760	8.3141703	0	Singlet	Adipocyte
SAT_26M	1309.6	903	1.7220735	6	Singlet	Neurons
SAT_26M	1178.4	896	0.7414686	11	Singlet	Smooth_muscle_cells
SAT_26M	2230.9	1416	0.1049908	0	Singlet	Adipocyte
SAT_26M	3937.4	1898	9.0337772	0	Singlet	Adipocyte
SAT_26M	2205.2	1305	0.1682757	0	Singlet	Adipocyte
SAT_26M	1018.2	720	2.6595978	0	Singlet	Adipocyte
SAT_26M	1921.1	1069	0	0	Singlet	Adipocyte
SAT_26M	1157.3	855	1.1223214	9	Singlet	Mesothelial
SAT_26M	1281.2	872	5.6907735	6	Singlet	Neurons
SAT_26M	1229.1	897	0.0092419	8	Singlet	Mesothelial
SAT_26M	1350.8	830	6.3623359	0	Singlet	Adipocyte
SAT_26M	1293.4	853	7.7204521	0	Singlet	Adipocyte
SAT_26M	1283.8	836	4.7455834	3	Singlet	FAPs
SAT_26M	1109.9	782	1.0208283	11	Singlet	Smooth_muscle_cells
SAT_26M	1986.5	1151	0.0970077	7	Singlet	Endothelial_cells
SAT_26M	1570.9	1058	0.025599	8	Singlet	Mesothelial
SAT_26M	2267.3	1279	1.5176652	0	Singlet	Adipocyte
SAT_26M	1043.4	712	0.0893163	8	Singlet	Mesothelial
SAT_26M	1464.9	1029	0.0621395	6	Singlet	Neurons
SAT_26M	1059	655	1.6970778	3	Singlet	FAPs
SAT_26M	2546.3	1360	6.1644025	7	Singlet	Endothelial_cells
SAT_26M	1625.4	967	1.7144215	2	Singlet	FAPs
SAT_26M	1816.6	1150	1.9627629	0	Singlet	Adipocyte
SAT_26M	1307.3	897	5.029569	1	Singlet	Immune_cells
SAT_26M	1176	777	0	8	Singlet	Mesothelial
SAT_26M	1368.7	815	4.4564116	7	Singlet	Endothelial_cells
SAT_26M	1190	792	0.5359458	0	Singlet	Adipocyte
SAT_26M	2539.7	1427	0	0	Singlet	Adipocyte
SAT_26M	1104.5	760	2.511696	0	Singlet	Adipocyte
SAT_26M	2142	1277	2.7697326	3	Singlet	FAPs
SAT_26M	1193.1	827	0.5262536	6	Singlet	Neurons
SAT_26M	1350.4	840	3.6390046	0	Singlet	Adipocyte
SAT_26M	1550.3	980	0.2091349	0	Singlet	Adipocyte
SAT_26M	1005.6	684	5.9548318	0	Singlet	Adipocyte
SAT_26M	1424	922	1.3758703	0	Singlet	Adipocyte
SAT_26M	1239.2	812	1.2821858	0	Singlet	Adipocyte

SAT_26M	1454.8	836	0	6	Singlet	Neurons
SAT_26M	1262.6	775	0.4373374	10	Singlet	Immune_cells
SAT_26M	1820.8	1030	0.9816828	0	Singlet	Adipocyte
SAT_26M	1399	906	3.9317412	10	Singlet	Immune_cells
SAT_26M	1302.2	749	0.5680572	0	Singlet	Adipocyte
SAT_26M	1486.3	971	4.1572312	0	Singlet	Adipocyte
SAT_26M	1695.9	728	14.521122	0	Singlet	Adipocyte
SAT_26M	1405.3	884	0.3721272	7	Singlet	Endothelial_cells
SAT_26M	1437.9	917	0.7333191	0	Singlet	Adipocyte
SAT_26M	1214.9	864	0.1686629	11	Singlet	Smooth_muscle_cells
SAT_26M	1211.8	808	1.7363485	0	Singlet	Adipocyte
SAT_26M	1917.9	1123	7.5862912	0	Singlet	Adipocyte
SAT_26M	2628.5	1498	5.013272	7	Singlet	Endothelial_cells
SAT_26M	1495.7	903	1.634584	10	Singlet	Immune_cells
SAT_26M	1929	1276	3.4815491	8	Singlet	Mesothelial
SAT_26M	2551.3	1410	2.6477877	0	Singlet	Adipocyte
SAT_26M	1425.8	915	0.0796474	0	Singlet	Adipocyte
SAT_26M	2295.2	1257	0	0	Singlet	Adipocyte
SAT_26M	2481.5	1382	0.4294976	0	Singlet	Adipocyte
SAT_26M	1626.7	997	0.5632585	3	Singlet	FAPs
SAT_26M	2604.3	1604	0.8956896	3	Singlet	FAPs
SAT_26M	3664.9	1703	8.6542996	7	Singlet	Endothelial_cells
SAT_26M	1222.5	802	0.7043244	0	Singlet	Adipocyte
SAT_26M	3260	1764	1.188642	0	Singlet	Adipocyte
SAT_26M	3565.6	1634	13.251495	0	Singlet	Adipocyte
SAT_26M	1507.2	959	6.2281704	5	Singlet	Immune_cells
SAT_26M	2773.7	1334	2.1734885	10	Singlet	Immune_cells
SAT_26M	1429.3	772	9.0080926	0	Singlet	Adipocyte
SAT_26M	2015.8	981	6.6449032	0	Singlet	Adipocyte
SAT_26M	1200.3	776	14.485004	4	Singlet	Immune_cells
SAT_26M	2789.7	1618	8.6994315	7	Singlet	Endothelial_cells
SAT_26M	1310.6	790	4.6220691	0	Singlet	Adipocyte
SAT_26M	2486.4	1395	1.6017792	0	Singlet	Adipocyte
SAT_26M	4350.3	2153	0	0	Singlet	Adipocyte
SAT_26M	1137.4	755	5.7800033	0	Singlet	Adipocyte
SAT_26M	1381.5	862	1.229379	10	Singlet	Immune_cells
SAT_26M	5949.6	2287	6.2751082	7	Singlet	Endothelial_cells
SAT_26M	4822.9	2178	4.3467425	0	Singlet	Adipocyte
SAT_26M	3690.9	1788	0.1565256	0	Singlet	Adipocyte
SAT_26M	1605.7	873	2.2503969	3	Singlet	FAPs
SAT_26M	1228.4	782	3.9505368	0	Singlet	Adipocyte
SAT_26M	1029.5	685	1.4321925	5	Singlet	Immune_cells
SAT_26M	1092.7	711	0.3882626	0	Singlet	Adipocyte
SAT_26M	1532.7	958	3.9571644	0	Singlet	Adipocyte
SAT_26M	1218.2	848	2.7322052	0	Singlet	Adipocyte
SAT_26M	1732.5	1152	2.0657269	0	Singlet	Adipocyte
SAT_26M	2390.4	1339	3.1798385	0	Singlet	Adipocyte
SAT_26M	1407.3	998	0.2256502	3	Singlet	FAPs
SAT_26M	1201.5	876	0.0489421	6	Singlet	Neurons
SAT_26M	1160	850	0.1482773	6	Singlet	Neurons
SAT_26M	1581.2	972	8.594382	3	Singlet	FAPs

SAT_26M	1246.3	770	3.5145138	3	Singlet	FAPs
SAT_26M	2251.8	1173	12.142555	0	Singlet	Adipocyte
SAT_26M	1732.2	980	5.0756521	0	Singlet	Adipocyte
SAT_26M	3520.4	1672	9.6266774	7	Singlet	Endothelial_cells
SAT_26M	1055.9	748	7.3938527	1	Singlet	Immune_cells
SAT_26M	1299.7	813	2.9465536	0	Singlet	Adipocyte
SAT_26M	2605.1	1283	12.716804	7	Singlet	Endothelial_cells
SAT_26M	1832.8	1238	0.3099769	0	Singlet	Adipocyte
SAT_26M	1075.6	726	4.2695482	0	Singlet	Adipocyte
SAT_26M	1213	778	3.8642001	2	Singlet	FAPs
SAT_26M	4604.1	2269	3.3957007	0	Singlet	Adipocyte
SAT_26M	1988.5	1168	0.0879245	0	Singlet	Adipocyte
SAT_26M	1174	789	1.248137	7	Singlet	Endothelial_cells
SAT_26M	2455.9	1461	0.6560879	0	Singlet	Adipocyte
SAT_26M	1216.1	828	0.5491969	0	Singlet	Adipocyte
SAT_26M	1134.4	817	1.2112642	4	Singlet	Immune_cells
SAT_26M	7695.1	3507	3.2858769	9	Singlet	Mesothelial
SAT_26M	2637.6	1363	9.1452432	7	Singlet	Endothelial_cells
SAT_26M	1128.8	747	2.8365739	7	Singlet	Endothelial_cells
SAT_26M	1398.8	1000	0.3019956	4	Singlet	Immune_cells
SAT_26M	2836.7	1565	0.0046371	0	Singlet	Adipocyte
SAT_26M	3584.3	1823	2.5171975	0	Singlet	Adipocyte
SAT_26M	1181.2	849	0.9769486	5	Singlet	Immune_cells
SAT_26M	1006	605	1.9917614	3	Singlet	FAPs
SAT_26M	1875.4	1100	1.7935987	0	Singlet	Adipocyte
SAT_26M	2464.6	1603	4.8691201	4	Singlet	Immune_cells
SAT_26M	1342.3	878	0.8360336	2	Singlet	FAPs
SAT_26M	1003	722	4.7619866	1	Singlet	Immune_cells
SAT_26M	1242.5	848	0.6962081	0	Singlet	Adipocyte
SAT_26M	1079.5	666	1.9621612	0	Singlet	Adipocyte
SAT_26M	1523.2	1007	0.0198132	8	Singlet	Mesothelial
SAT_26M	1397	876	1.1123386	0	Singlet	Adipocyte
SAT_26M	1401.9	927	2.0676422	0	Singlet	Adipocyte
SAT_26M	1008.4	646	0.4052715	0	Singlet	Adipocyte
SAT_26M	2426.4	1446	0.014413	0	Singlet	Adipocyte
SAT_26M	2849.2	1653	1.0807638	0	Singlet	Adipocyte
SAT_26M	1372.8	730	5.8625075	7	Singlet	Endothelial_cells
SAT_26M	7306.5	3305	13.463621	8	Singlet	Mesothelial
SAT_26M	2107.3	1171	9.5283346	0	Singlet	Adipocyte
SAT_26M	1531.6	868	5.5386401	5	Singlet	Immune_cells
SAT_26M	3072.1	1336	2.6117147	10	Singlet	Immune_cells
SAT_26M	2085.2	1012	9.9238818	7	Singlet	Endothelial_cells
SAT_26M	1510.8	748	0.9760469	10	Singlet	Immune_cells
SAT_26M	1125.8	765	0.1971253	0	Singlet	Adipocyte
SAT_26M	1365.9	911	1.971972	5	Singlet	Immune_cells
SAT_26M	1101.3	715	1.3646082	3	Singlet	FAPs
SAT_26M	1015.6	640	0.8553589	3	Singlet	FAPs
SAT_26M	1478.6	883	6.8069406	0	Singlet	Adipocyte
SAT_26M	1323	836	0.000746	0	Singlet	Adipocyte
SAT_26M	1411.9	974	0.0460973	3	Singlet	FAPs
SAT_26M	1139	703	11.390291	4	Singlet	Immune_cells

SAT_26M	4458.3	1875	0	0	Singlet	Adipocyte
SAT_26M	1223.4	981	0.9971855	11	Singlet	Smooth_muscle_cells
SAT_26M	1545.5	982	4.428553	0	Singlet	Adipocyte
SAT_26M	3240.1	1756	0.108925	0	Singlet	Adipocyte
SAT_26M	1012.4	694	1.252107	0	Singlet	Adipocyte
SAT_26M	1668.7	898	14.309222	7	Singlet	Endothelial_cells
SAT_26M	1293.2	921	0.3189992	0	Singlet	Adipocyte
SAT_26M	1252.2	856	0	11	Singlet	Smooth_muscle_cells
SAT_26M	1353.4	950	0.0257936	0	Singlet	Adipocyte
SAT_26M	1099.8	616	1.8073859	7	Singlet	Endothelial_cells
SAT_26M	3875.7	2015	0.2262581	0	Singlet	Adipocyte
SAT_26M	2129.6	1243	2.3273259	7	Singlet	Endothelial_cells
SAT_26M	2547.8	1416	1.1710559	0	Singlet	Adipocyte
SAT_26M	1323.9	905	0.6012564	3	Singlet	FAPs
SAT_26M	3754.1	1858	1.6276092	0	Singlet	Adipocyte
SAT_26M	1368.8	844	0.723264	0	Singlet	Adipocyte
SAT_26M	1884.6	1004	0	6	Singlet	Neurons
SAT_26M	1842.9	1202	2.8842024	3	Singlet	FAPs
SAT_26M	2979.4	1380	1.8616482	0	Singlet	Adipocyte
SAT_26M	1476.9	989	0.6158964	0	Singlet	Adipocyte
SAT_26M	1287.6	748	6.7569493	0	Singlet	Adipocyte
SAT_26M	1892.6	1158	0.8139543	0	Singlet	Adipocyte
SAT_26M	2154.3	1348	0.3302141	0	Singlet	Adipocyte
SAT_26M	1937.8	1026	3.6399853	0	Singlet	Adipocyte
SAT_26M	1048.9	733	2.1277394	0	Singlet	Adipocyte
SAT_26M	5533.7	1964	0.3404264	10	Singlet	Immune_cells
SAT_26M	1412.4	893	8.2411726	5	Singlet	Immune_cells
SAT_26M	1395.9	972	0.2712736	1	Singlet	Immune_cells
SAT_26M	2199	1400	0.0219117	8	Singlet	Mesothelial
SAT_26M	1100.1	671	8.167519	7	Singlet	Endothelial_cells
SAT_26M	2431.3	1331	2.2654178	0	Singlet	Adipocyte
SAT_26M	2433	1480	1.977699	7	Singlet	Endothelial_cells
SAT_26M	1790.4	1039	0.9181397	7	Singlet	Endothelial_cells
SAT_26M	1609.4	1005	6.4185058	0	Singlet	Adipocyte
SAT_26M	1783.5	1094	2.4061048	0	Singlet	Adipocyte
SAT_26M	1814.3	1017	2.2382221	0	Singlet	Adipocyte
SAT_26M	1322	700	1.4557781	10	Singlet	Immune_cells
SAT_26M	2658.7	1574	0	4	Singlet	Immune_cells
SAT_26M	1488.9	927	0.2369482	0	Singlet	Adipocyte
SAT_26M	1629.5	877	13.72856	7	Singlet	Endothelial_cells
SAT_26M	1005.8	648	1.9073983	3	Singlet	FAPs
SAT_26M	1002.8	653	3.5985839	0	Singlet	Adipocyte
SAT_26M	3543.6	1684	9.3807224	0	Singlet	Adipocyte
SAT_26M	2300.6	1248	0.5789981	0	Singlet	Adipocyte
SAT_26M	1022.7	712	0.0354963	6	Singlet	Neurons
SAT_26M	1118.6	698	0.606391	0	Singlet	Adipocyte
SAT_26M	1423	817	11.794448	7	Singlet	Endothelial_cells
SAT_26M	1662.5	1040	0.185866	0	Singlet	Adipocyte
SAT_26M	1056.8	715	1.0212177	0	Singlet	Adipocyte
SAT_26M	2684.6	1512	0	0	Singlet	Adipocyte
SAT_26M	2695.7	1437	7.9394368	0	Singlet	Adipocyte

SAT_26M	1036.2	702	0	9	Singlet	Mesothelial
SAT_26M	1199.1	793	1.2744972	1	Singlet	Immune_cells
SAT_26M	1334.5	846	14.606943	9	Singlet	Mesothelial
SAT_26M	1630.6	992	0	0	Singlet	Adipocyte
SAT_26M	2461.6	1335	11.355963	9	Singlet	Mesothelial
SAT_26M	1314	846	1.8047065	7	Singlet	Endothelial_cells
SAT_26M	2103.8	1455	2.5922521	11	Singlet	Smooth_muscle_cells
SAT_26M	1438.2	941	0.408171	0	Singlet	Adipocyte
SAT_26M	3617.7	1857	11.054814	7	Singlet	Endothelial_cells
SAT_26M	2474.1	1458	0.4615983	0	Singlet	Adipocyte
SAT_26M	4412	2154	0.4602734	0	Singlet	Adipocyte
SAT_26M	2074	1289	0.0001506	0	Singlet	Adipocyte
SAT_26M	6074.3	2808	10.17225	9	Singlet	Mesothelial
SAT_26M	1104.9	772	11.609599	1	Singlet	Immune_cells
SAT_26M	1222.7	786	4.3156301	6	Singlet	Neurons
SAT_26M	3039.7	1627	1.3951208	0	Singlet	Adipocyte
SAT_26M	2077.3	1234	9.9604827	9	Singlet	Mesothelial
SAT_26M	1345.5	843	0	0	Singlet	Adipocyte
SAT_26M	1813.8	1018	12.88861	7	Singlet	Endothelial_cells
SAT_26M	3638.5	1829	0.5668652	0	Singlet	Adipocyte
SAT_26M	2664.8	1418	1.6589021	0	Singlet	Adipocyte
SAT_26M	2870.1	1689	1.9750589	9	Singlet	Mesothelial
SAT_26M	2338.8	1355	3.4555879	7	Singlet	Endothelial_cells
SAT_26M	1150.3	792	3.2209667	5	Singlet	Immune_cells
SAT_26M	1177.5	788	2.0757355	3	Singlet	FAPs
SAT_26M	3764.9	1918	2.0682847	0	Singlet	Adipocyte
SAT_26M	1397	973	0.0088994	0	Singlet	Adipocyte
SAT_26M	1670.9	947	12.494323	9	Singlet	Mesothelial
SAT_26M	1723.6	1030	0.4506602	0	Singlet	Adipocyte
SAT_26M	1444.1	937	0.0342868	3	Singlet	FAPs
SAT_26M	3603	1628	2.8712852	10	Singlet	Immune_cells
SAT_26M	1600	1020	5.1420838	0	Singlet	Adipocyte
SAT_26M	2632.4	1446	1.1574147	0	Singlet	Adipocyte
SAT_26M	1031.1	753	0.0814224	8	Singlet	Mesothelial
SAT_26M	1348.2	800	4.0882607	6	Singlet	Neurons
SAT_26M	1193.3	846	4.2121877	0	Singlet	Adipocyte
SAT_26M	1046.9	714	0.0204575	0	Singlet	Adipocyte
SAT_26M	1163.2	759	1.7487413	7	Singlet	Endothelial_cells
SAT_26M	1261.6	903	1.8017628	7	Singlet	Endothelial_cells
SAT_26M	1160.4	759	0.7700979	3	Singlet	FAPs
SAT_26M	1068.6	790	0.1038993	8	Singlet	Mesothelial
SAT_26M	1340.9	838	5.52854	0	Singlet	Adipocyte
SAT_26M	1303.1	909	0.7799735	4	Singlet	Immune_cells
SAT_26M	1114.7	766	7.466898	0	Singlet	Adipocyte
SAT_26M	2432.7	1409	0.002982	8	Singlet	Mesothelial
SAT_26M	1857.2	976	7.7053691	7	Singlet	Endothelial_cells
SAT_26M	2317.9	1398	4.8361671	0	Singlet	Adipocyte
SAT_26M	2482.9	1367	0.5521553	0	Singlet	Adipocyte
SAT_26M	1446.9	889	10.864747	7	Singlet	Endothelial_cells
SAT_26M	3814.3	1888	0.4542519	0	Singlet	Adipocyte
SAT_26M	1235.2	772	13.70862	5	Singlet	Immune_cells

SAT_26M	1197	742	0.0606208	8	Singlet	Mesothelial
SAT_26M	1600.4	1031	1.215664	0	Singlet	Adipocyte
SAT_26M	1103.1	773	0.9895209	0	Singlet	Adipocyte
SAT_26M	1114.4	798	0.4744202	5	Singlet	Immune_cells
SAT_26M	3464.7	1735	0.060906	0	Singlet	Adipocyte
SAT_26M	1341.7	842	1.0441148	0	Singlet	Adipocyte
SAT_26M	1253.6	843	8.6707454	1	Singlet	Immune_cells
SAT_26M	2435.2	1258	1.2238067	0	Singlet	Adipocyte
SAT_26M	1753.8	1075	4.9766681	7	Singlet	Endothelial_cells
SAT_26M	2702.6	1452	1.7909384	0	Singlet	Adipocyte
SAT_26M	1253.6	776	0	0	Singlet	Adipocyte
SAT_26M	2594.1	1478	2.2194617	5	Singlet	Immune_cells
SAT_26M	1494.4	1065	0.0108401	11	Singlet	Smooth_muscle_cells
SAT_26M	1329	793	0.3697231	10	Singlet	Immune_cells
SAT_26M	1614	945	4.8459676	0	Singlet	Adipocyte
SAT_26M	1192.5	684	6.4178294	0	Singlet	Adipocyte
SAT_26M	3439.8	1796	6.0028738	0	Singlet	Adipocyte
SAT_26M	1226.3	645	5.4583737	0	Singlet	Adipocyte
SAT_26M	1542.5	932	1.3647747	0	Singlet	Adipocyte
SAT_26M	1720.7	1049	6.6080039	0	Singlet	Adipocyte
SAT_26M	1080.4	806	1.7867295	7	Singlet	Endothelial_cells
SAT_26M	1108.3	723	0	0	Singlet	Adipocyte
SAT_26M	1452.1	1018	0.0679603	11	Singlet	Smooth_muscle_cells
SAT_26M	1319.4	980	3.6154456	1	Singlet	Immune_cells
SAT_26M	1212	833	0.799032	0	Singlet	Adipocyte
SAT_26M	4146.5	1974	7.9842788	8	Singlet	Mesothelial
SAT_26M	1055.3	726	0.1214528	3	Singlet	FAPs
SAT_26M	1726	1126	3.1839873	11	Singlet	Smooth_muscle_cells
SAT_26M	1189.8	806	2.5439876	1	Singlet	Immune_cells
SAT_26M	1071.7	809	2.5471274	0	Singlet	Adipocyte
SAT_26M	1153.2	773	9.1292826	1	Singlet	Immune_cells
SAT_26M	1722.6	1077	1.5665174	2	Singlet	FAPs
SAT_26M	1775.5	1079	0.6178992	0	Singlet	Adipocyte
SAT_26M	1014.4	624	1.8953636	3	Singlet	FAPs
SAT_26M	2927.4	1051	13.165509	0	Singlet	Adipocyte
SAT_26M	1302.6	745	0	6	Singlet	Neurons
SAT_26M	1012.8	664	0.1043109	0	Singlet	Adipocyte
SAT_26M	1114.6	808	1.9355971	6	Singlet	Neurons
SAT_26M	1632.7	1108	0.8610496	11	Singlet	Smooth_muscle_cells
SAT_26M	3065.5	1594	0.0057249	0	Singlet	Adipocyte
SAT_26M	1027.4	683	1.040408	0	Singlet	Adipocyte
SAT_26M	1080.5	734	1.1317714	0	Singlet	Adipocyte
SAT_26M	1062.7	704	2.0821462	5	Singlet	Immune_cells
SAT_26M	1005.5	710	0.0828926	5	Singlet	Immune_cells
SAT_26M	2011.3	1148	4.5308899	0	Singlet	Adipocyte
SAT_26M	2495.3	1392	0	0	Singlet	Adipocyte
SAT_26M	1319.7	924	2.5943839	1	Singlet	Immune_cells
SAT_26M	1408	795	7.0054561	0	Singlet	Adipocyte
SAT_26M	1356.6	895	1.6939845	8	Singlet	Mesothelial
SAT_26M	1412.1	875	0.0356864	0	Singlet	Adipocyte
SAT_26M	1117.7	817	0	5	Singlet	Immune_cells

SAT_26M	1178.5	803	1.3643726	0	Singlet	Adipocyte
SAT_26M	1234.9	797	0.0796312	7	Singlet	Endothelial_cells
SAT_26M	2415.3	1480	0	8	Singlet	Mesothelial
SAT_26M	1436.8	971	0.0658637	8	Singlet	Mesothelial
SAT_26M	1005.6	660	2.7048122	5	Singlet	Immune_cells
SAT_26M	1111.4	674	2.6761185	10	Singlet	Immune_cells
SAT_26M	2176.5	1328	0	0	Singlet	Adipocyte
SAT_26M	5008.9	2056	0.7923186	10	Singlet	Immune_cells
SAT_26M	1182.4	707	2.6084509	10	Singlet	Immune_cells
SAT_26M	2296.5	1364	3.1969397	0	Singlet	Adipocyte
SAT_26M	1670.3	1032	0	8	Singlet	Mesothelial
SAT_26M	1579.1	1093	0.7626345	11	Singlet	Smooth_muscle_cells
SAT_26M	1495.9	1027	0.6790079	7	Singlet	Endothelial_cells
SAT_26M	1184.3	781	1.550595	0	Singlet	Adipocyte
SAT_26M	1330.2	890	0.5928557	0	Singlet	Adipocyte
SAT_26M	4434.4	2305	2.3118112	0	Singlet	Adipocyte
SAT_26M	2277.6	1167	3.9223964	10	Singlet	Immune_cells
SAT_26M	4150.9	2178	5.5950236	9	Singlet	Mesothelial
SAT_26M	2258.1	1407	0.1554178	8	Singlet	Mesothelial
SAT_26M	4459.4	2164	0.4872054	0	Singlet	Adipocyte
SAT_26M	1240.1	846	0.5933808	5	Singlet	Immune_cells
SAT_26M	1680	1002	1.8622629	0	Singlet	Adipocyte
SAT_26M	1814.7	1049	0.1326048	0	Singlet	Adipocyte
SAT_26M	1518.3	1004	2.7267969	5	Singlet	Immune_cells
SAT_26M	3751.4	1815	5.2036969	7	Singlet	Endothelial_cells
SAT_26M	3413.4	1421	5.4585275	10	Singlet	Immune_cells
SAT_26M	1680.6	1007	7.5044305	0	Singlet	Adipocyte
SAT_26M	1142.6	740	4.4803012	7	Singlet	Endothelial_cells
SAT_26M	1060.3	794	2.9962946	1	Singlet	Immune_cells
SAT_26M	1346.8	896	0.8204601	1	Singlet	Immune_cells
SAT_26M	1168.1	735	2.416973	5	Singlet	Immune_cells
SAT_26M	1260.8	834	0	0	Singlet	Adipocyte
SAT_26M	7882.2	3543	9.404928	9	Singlet	Mesothelial
SAT_26M	1381.9	903	0.0242988	0	Singlet	Adipocyte
SAT_26M	9103	3819	6.0155926	9	Singlet	Mesothelial
SAT_26M	2103.3	1353	0.0818183	11	Singlet	Smooth_muscle_cells
SAT_26M	5510.3	2675	4.2072223	0	Singlet	Adipocyte
SAT_26M	1025.1	638	0.0633651	0	Singlet	Adipocyte
SAT_26M	2601.6	1426	0.3687585	0	Singlet	Adipocyte
SAT_26M	1180.8	812	0.1644673	0	Singlet	Adipocyte
SAT_26M	1480.2	926	3.2793423	7	Singlet	Endothelial_cells
SAT_26M	1299.3	880	0.2906168	0	Singlet	Adipocyte
SAT_26M	3336	1901	2.7725261	9	Singlet	Mesothelial
SAT_26M	2420.4	1504	0	11	Singlet	Smooth_muscle_cells
SAT_26M	1461.1	969	2.4210671	3	Singlet	FAPs
SAT_26M	4665.8	1814	0	6	Singlet	Neurons
SAT_26M	2278.1	1365	0	8	Singlet	Mesothelial
SAT_26M	1213.1	745	3.7486375	0	Singlet	Adipocyte
SAT_26M	2637.9	1552	5.3771051	7	Singlet	Endothelial_cells
SAT_26M	1225.4	813	0.0204075	0	Singlet	Adipocyte
SAT_26M	2265.1	1189	2.8950792	0	Singlet	Adipocyte

SAT_26M	1181.2	600	9.3351479	0	Singlet	Adipocyte
SAT_26M	1315.8	988	0.9258541	7	Singlet	Endothelial_cells
SAT_26M	2010	1237	2.9235967	7	Singlet	Endothelial_cells
SAT_26M	1826.9	1103	1.6908573	0	Singlet	Adipocyte
SAT_26M	1762.4	1143	1.2402608	0	Singlet	Adipocyte
SAT_26M	1238.1	839	0	0	Singlet	Adipocyte
SAT_26M	8130.1	3114	0.3512618	0	Singlet	Adipocyte
SAT_26M	2730.1	1282	7.4255779	0	Singlet	Adipocyte
SAT_26M	1850.9	1306	7.4560037	1	Singlet	Immune_cells
SAT_26M	1973	1200	1.287855	0	Singlet	Adipocyte
SAT_26M	1626.4	1152	0.0230127	8	Singlet	Mesothelial
SAT_26M	1182.5	804	1.0237121	7	Singlet	Endothelial_cells
SAT_26M	1015.4	646	0.0361123	6	Singlet	Neurons
SAT_26M	1621.1	917	9.5233072	7	Singlet	Endothelial_cells
SAT_26M	2987.1	1660	0.5157008	0	Singlet	Adipocyte
SAT_26M	1951.4	1250	2.4704526	1	Singlet	Immune_cells
SAT_26M	1279.1	816	1.9948393	10	Singlet	Immune_cells
SAT_26M	2257.3	1446	0	8	Singlet	Mesothelial
SAT_26M	3094.9	1756	1.0378476	0	Singlet	Adipocyte
SAT_26M	3799.3	2508	4.8423578	9	Singlet	Mesothelial
SAT_26M	1955.4	1150	8.0153367	7	Singlet	Endothelial_cells
SAT_26M	3721.2	1949	3.1150936	0	Singlet	Adipocyte
SAT_26M	3135	1727	0.3142774	0	Singlet	Adipocyte
SAT_26M	2152.9	1281	0.3693894	0	Singlet	Adipocyte
SAT_26M	1499.1	997	1.7839389	0	Singlet	Adipocyte
SAT_26M	1020.1	716	1.6344628	2	Singlet	FAPs
SAT_26M	1171	705	7.8233033	7	Singlet	Endothelial_cells
SAT_26M	2280.2	1372	0.0831631	0	Singlet	Adipocyte
SAT_26M	1016.6	614	14.484259	1	Singlet	Immune_cells
SAT_26M	1328.3	923	0.1623351	3	Singlet	FAPs
SAT_26M	1081.2	721	3.6777288	5	Singlet	Immune_cells
SAT_26M	1050.6	715	0.160075	0	Singlet	Adipocyte
SAT_26M	1970.2	988	10.247513	0	Singlet	Adipocyte
SAT_26M	1244.3	780	3.4880358	10	Singlet	Immune_cells
SAT_26M	4097.4	2153	0.0942196	0	Singlet	Adipocyte
SAT_26M	1492.5	1046	0.191603	1	Singlet	Immune_cells
SAT_26M	1348.6	833	10.823372	9	Singlet	Mesothelial
SAT_26M	2319.6	1361	6.0219618	4	Singlet	Immune_cells
SAT_26M	3153.3	1694	0.5901803	0	Singlet	Adipocyte
SAT_26M	1125.3	738	0.9504386	0	Singlet	Adipocyte
SAT_26M	1188.6	889	4.1759618	1	Singlet	Immune_cells
SAT_26M	1889.2	1221	0.3810061	0	Singlet	Adipocyte
SAT_26M	1422.9	901	1.2972261	10	Singlet	Immune_cells
SAT_26M	1397.9	939	0.1243206	0	Singlet	Adipocyte
SAT_26M	1072.6	631	8.9619111	7	Singlet	Endothelial_cells
SAT_26M	3082.1	1784	2.6611169	0	Singlet	Adipocyte
SAT_26M	1300.5	860	0.0120579	0	Singlet	Adipocyte
SAT_26M	3830.7	2029	2.4965699	0	Singlet	Adipocyte
SAT_26M	2409.3	1211	5.266522	10	Singlet	Immune_cells
SAT_26M	2024.4	1226	0.0309346	3	Singlet	FAPs
SAT_26M	1220.6	771	2.1767424	0	Singlet	Adipocyte

SAT_26M	2806.9	1608	3.1712362	7	Singlet	Endothelial_cells
SAT_26M	12070	4903	2.5997176	9	Singlet	Mesothelial
SAT_26M	1130.7	815	0.9720216	8	Singlet	Mesothelial
SAT_26M	1710.1	904	0.5898323	10	Singlet	Immune_cells
SAT_26M	1359.2	910	0.3219313	6	Singlet	Neurons
SAT_26M	1119.2	874	4.3717195	1	Singlet	Immune_cells
SAT_26M	2298.8	1361	0	8	Singlet	Mesothelial
SAT_26M	1140.5	792	1.1275921	0	Singlet	Adipocyte
SAT_26M	4883.2	2286	0.021012	0	Singlet	Adipocyte
SAT_26M	3164.6	1595	5.5363714	7	Singlet	Endothelial_cells
SAT_26M	2053.5	1142	12.850688	7	Singlet	Endothelial_cells
SAT_26M	3874	1903	2.4162179	0	Singlet	Adipocyte
SAT_26M	1288.3	839	1.8973248	0	Singlet	Adipocyte
SAT_26M	1807.9	1072	0.2119976	7	Singlet	Endothelial_cells
SAT_26M	1777.2	1081	1.4072214	0	Singlet	Adipocyte
SAT_26M	1185.5	740	0.0396021	8	Singlet	Mesothelial
SAT_26M	1128.7	757	3.8011949	0	Singlet	Adipocyte
SAT_26M	1207.6	658	2.7343246	10	Singlet	Immune_cells
SAT_26M	2689.9	1576	0.5668807	0	Singlet	Adipocyte
SAT_26M	1852.6	1063	0.2347623	0	Singlet	Adipocyte
SAT_26M	2001.8	1294	0	0	Singlet	Adipocyte
SAT_26M	1666.7	959	5.9438336	10	Singlet	Immune_cells
SAT_26M	1126.5	722	2.2887128	0	Singlet	Adipocyte
SAT_26M	1974.5	1203	0.0449735	0	Singlet	Adipocyte
SAT_26M	1885.4	1197	0.150064	0	Singlet	Adipocyte
SAT_26M	1657.3	1035	2.91302	0	Singlet	Adipocyte
SAT_26M	1094.2	715	3.503157	7	Singlet	Endothelial_cells
SAT_26M	1741.3	1120	0.0495328	8	Singlet	Mesothelial
SAT_26M	1513.7	1157	5.4221746	4	Singlet	Immune_cells
SAT_26M	7505.6	3568	4.7691266	9	Singlet	Mesothelial
SAT_26M	1234.5	750	7.8903917	0	Singlet	Adipocyte
SAT_26M	1268.1	856	3.4867241	1	Singlet	Immune_cells
SAT_26M	3342.5	1832	0.2881242	0	Singlet	Adipocyte
SAT_26M	2730.7	1646	0.997187	0	Singlet	Adipocyte
SAT_26M	3145.7	1684	0.0390916	0	Singlet	Adipocyte
SAT_26M	1057.4	693	0.968527	3	Singlet	FAPs
SAT_26M	1121.6	828	5.466203	1	Singlet	Immune_cells
SAT_26M	1054.5	716	1.8839414	6	Singlet	Neurons
SAT_26M	4135.1	1990	0.0921198	0	Singlet	Adipocyte
SAT_26M	1136.6	705	9.1579262	7	Singlet	Endothelial_cells
SAT_26M	1428.1	870	3.454763	0	Singlet	Adipocyte
SAT_26M	1136.3	857	2.7343553	8	Singlet	Mesothelial
SAT_26M	1012.7	717	1.1956248	6	Singlet	Neurons
SAT_26M	1552.2	1028	6.1998838	4	Singlet	Immune_cells
SAT_26M	2512.2	1274	7.2695622	0	Singlet	Adipocyte
SAT_26M	1352.4	845	1.3315947	5	Singlet	Immune_cells
SAT_26M	2180.5	1355	0.2049563	0	Singlet	Adipocyte
SAT_26M	1103	685	5.9273739	7	Singlet	Endothelial_cells
SAT_26M	2103.6	1070	11.597436	0	Singlet	Adipocyte
SAT_26M	1601.1	1033	0.1606594	0	Singlet	Adipocyte
SAT_26M	1165.5	703	1.504975	3	Singlet	FAPs

SAT_26M	1929.3	1050	0.2457201	10	Singlet	Immune_cells
SAT_26M	1175.8	749	4.0474048	7	Singlet	Endothelial_cells
SAT_26M	1108.2	761	0.5455701	3	Singlet	FAPs
SAT_26M	1692.4	1096	0.1048128	8	Singlet	Mesothelial
SAT_26M	1766.4	1010	1.8364612	10	Singlet	Immune_cells
SAT_26M	1450.8	957	2.2032869	0	Singlet	Adipocyte
SAT_26M	1710.8	1098	2.2310984	0	Singlet	Adipocyte
SAT_26M	1170.6	815	0	0	Singlet	Adipocyte
SAT_26M	1001.4	625	1.8233127	10	Singlet	Immune_cells
SAT_26M	2309.2	1098	1.9751755	0	Singlet	Adipocyte
SAT_26M	1390.8	942	0.0551581	8	Singlet	Mesothelial
SAT_26M	1081	604	7.4837359	0	Singlet	Adipocyte
SAT_26M	1134.2	786	4.4420436	4	Singlet	Immune_cells
SAT_26M	1088.3	671	0.0217289	2	Singlet	FAPs
SAT_26M	2398.1	1452	0.0033357	8	Singlet	Mesothelial
SAT_26M	1374.3	870	5.3613326	7	Singlet	Endothelial_cells
SAT_26M	1498.9	823	7.0739569	10	Singlet	Immune_cells
SAT_26M	2402.2	1374	0.7872798	0	Singlet	Adipocyte
SAT_26M	3752.4	1925	0.4811306	0	Singlet	Adipocyte
SAT_26M	1266.3	825	1.5115088	0	Singlet	Adipocyte
SAT_26M	1197.1	778	2.8862043	0	Singlet	Adipocyte
SAT_26M	1072.4	547	8.8449029	9	Singlet	Mesothelial
SAT_26M	3752.1	2182	1.0420778	8	Singlet	Mesothelial
SAT_26M	2811.6	1534	10.298426	0	Singlet	Adipocyte
SAT_26M	2365	1451	2.7735225	4	Singlet	Immune_cells
SAT_26M	1900.9	736	12.381553	0	Singlet	Adipocyte
SAT_26M	1542.2	758	4.9728456	0	Singlet	Adipocyte
SAT_26M	5015.4	2464	0.0030816	0	Singlet	Adipocyte
SAT_26M	2032.2	1150	3.5717536	0	Singlet	Adipocyte
SAT_26M	2570.7	1552	0.0294366	0	Singlet	Adipocyte
SAT_26M	1199.3	831	6.7450566	8	Singlet	Mesothelial
SAT_26M	1050.2	682	7.0850675	5	Singlet	Immune_cells
SAT_26M	1533.3	968	2.4784355	0	Singlet	Adipocyte
SAT_26M	1310.2	844	0.7984583	2	Singlet	FAPs
SAT_26M	1341.9	617	0.5366481	5	Singlet	Immune_cells
SAT_26M	3714.2	1903	0.5964485	0	Singlet	Adipocyte
SAT_26M	1889.3	991	3.4265108	10	Singlet	Immune_cells
SAT_26M	12494	3096	1.0309925	0	Singlet	Adipocyte
SAT_26M	1350	893	0.3679611	7	Singlet	Endothelial_cells
SAT_26M	1755.3	1057	6.7555936	7	Singlet	Endothelial_cells
SAT_26M	2220.9	1447	2.0937077	1	Singlet	Immune_cells
SAT_26M	1276.4	851	0	8	Singlet	Mesothelial
SAT_26M	1202.6	827	2.9346469	1	Singlet	Immune_cells
SAT_26M	2397.4	1428	0.2466823	0	Singlet	Adipocyte
SAT_26M	2803	1158	13.891376	0	Singlet	Adipocyte
SAT_26M	1090.1	687	4.4246748	0	Singlet	Adipocyte
SAT_26M	2177.1	1245	0.2415341	0	Singlet	Adipocyte
SAT_26M	2429.7	1223	11.420242	7	Singlet	Endothelial_cells
SAT_26M	2060.8	1266	0.5013092	0	Singlet	Adipocyte
SAT_26M	1904.3	1136	1.7602516	0	Singlet	Adipocyte
SAT_26M	1859.3	1016	2.4822227	10	Singlet	Immune_cells

SAT_26M	1471.7	1026	0	11	Singlet	Smooth_muscle_cells
SAT_26M	1135.9	754	0.1810307	0	Singlet	Adipocyte
SAT_26M	1357.5	869	3.9877245	0	Singlet	Adipocyte
SAT_26M	1069.9	665	8.8251458	7	Singlet	Endothelial_cells
SAT_26M	1095.2	710	0	2	Singlet	FAPs
SAT_26M	1339	705	10.158657	0	Singlet	Adipocyte
SAT_26M	1291.7	823	0.0398804	0	Singlet	Adipocyte
SAT_26M	1073.6	747	3.2532991	0	Singlet	Adipocyte
SAT_26M	2372.5	1390	1.3977884	0	Singlet	Adipocyte
SAT_26M	2597.8	1345	3.5591121	0	Singlet	Adipocyte
SAT_26M	1147.5	790	0.0526673	3	Singlet	FAPs
SAT_26M	2396.1	1240	12.751016	7	Singlet	Endothelial_cells
SAT_26M	1407.7	978	1.7105894	0	Singlet	Adipocyte
SAT_26M	2271.8	1224	9.9042207	7	Singlet	Endothelial_cells
SAT_26M	1162.5	758	7.1129977	7	Singlet	Endothelial_cells
SAT_26M	2273.7	1346	2.8746882	0	Singlet	Adipocyte
SAT_26M	1750.1	1246	0	8	Singlet	Mesothelial
SAT_26M	1431.3	926	0.0234985	3	Singlet	FAPs
SAT_26M	3122.3	1713	0.2056333	0	Singlet	Adipocyte
SAT_26M	1057	730	0.126448	0	Singlet	Adipocyte
SAT_26M	1543.9	1015	0	8	Singlet	Mesothelial
SAT_26M	1195.3	831	0.1185165	0	Singlet	Adipocyte
SAT_26M	1012.1	699	5.8388174	7	Singlet	Endothelial_cells
SAT_26M	1467	718	6.789768	0	Singlet	Adipocyte
SAT_26M	1008.3	749	1.2186046	1	Singlet	Immune_cells
SAT_26M	2555	1552	0	8	Singlet	Mesothelial
SAT_26M	1531.5	1008	0.7940677	0	Singlet	Adipocyte
SAT_26M	1563.4	954	0.6412746	0	Singlet	Adipocyte
SAT_26M	2072.6	1325	6.6682317	7	Singlet	Endothelial_cells
SAT_26M	1430.1	818	8.4981733	7	Singlet	Endothelial_cells
SAT_26M	3800.1	1933	0.071814	0	Singlet	Adipocyte
SAT_26M	1779.2	1147	2.5466985	0	Singlet	Adipocyte
SAT_26M	1172.8	902	10.351201	1	Singlet	Immune_cells
SAT_26M	1146.7	863	0.1499049	6	Singlet	Neurons
SAT_26M	1043.4	694	5.4645337	10	Singlet	Immune_cells
SAT_26M	3974	1774	13.338336	7	Singlet	Endothelial_cells
SAT_26M	2955	1543	0.0551003	0	Singlet	Adipocyte
SAT_26M	1110.9	732	0.1261529	7	Singlet	Endothelial_cells
SAT_26M	1744.9	1097	0.1432521	0	Singlet	Adipocyte
SAT_26M	1940.4	986	9.4114794	7	Singlet	Endothelial_cells
SAT_26M	2677.5	1470	1.0594829	0	Singlet	Adipocyte
SAT_26M	1809.6	1105	0.3462487	0	Singlet	Adipocyte
SAT_26M	2814.3	1560	3.5870033	0	Singlet	Adipocyte
SAT_26M	1592.4	986	0.0475568	7	Singlet	Endothelial_cells
SAT_26M	1809.8	1029	4.1330189	0	Singlet	Adipocyte
SAT_26M	3515.8	1836	0.201386	0	Singlet	Adipocyte
SAT_26M	1087	709	2.1956978	0	Singlet	Adipocyte
SAT_26M	1873.7	1068	0.0525149	6	Singlet	Neurons
SAT_26M	1212	745	14.751125	7	Singlet	Endothelial_cells
SAT_26M	1471.8	913	0.8612635	7	Singlet	Endothelial_cells
SAT_26M	1119.5	758	13.477229	1	Singlet	Immune_cells

SAT_26M	2739.8	1627	0.9163369	0	Singlet	Adipocyte
SAT_26M	1766.9	1110	0.7117679	5	Singlet	Immune_cells
SAT_26M	1233.5	781	9.075325	5	Singlet	Immune_cells
SAT_26M	1325.5	853	0	6	Singlet	Neurons
SAT_26M	1762.7	1073	11.599178	3	Singlet	FAPs
SAT_26M	1628.3	1134	2.2111693	1	Singlet	Immune_cells
SAT_26M	2038	1338	0.2258718	1	Singlet	Immune_cells
SAT_26M	1536	969	1.1572786	0	Singlet	Adipocyte
SAT_26M	2594.7	1649	0	11	Singlet	Smooth_muscle_cells
SAT_26M	3124.8	1717	1.1814007	0	Singlet	Adipocyte
SAT_26M	1353.9	975	0.5746875	0	Singlet	Adipocyte
SAT_26M	2769.7	1536	0.9054795	0	Singlet	Adipocyte
SAT_26M	1617.7	1025	0.2641611	3	Singlet	FAPs
SAT_26M	2183.8	1306	1.486977	7	Singlet	Endothelial_cells
SAT_26M	1362.3	833	4.9104362	10	Singlet	Immune_cells
SAT_26M	1056.5	797	0	8	Singlet	Mesothelial
SAT_26M	3817.3	2243	0.002984	9	Singlet	Mesothelial
SAT_26M	1278.7	652	0.7085463	10	Singlet	Immune_cells
SAT_26M	1321.5	865	0.058296	8	Singlet	Mesothelial
SAT_26M	1074.7	767	0	0	Singlet	Adipocyte
SAT_26M	3228	1735	2.0490325	5	Singlet	Immune_cells
SAT_26M	1090.8	681	0.2497287	0	Singlet	Adipocyte
SAT_26M	2066.3	1121	10.590782	0	Singlet	Adipocyte
SAT_26M	2319.2	1417	0.0190373	7	Singlet	Endothelial_cells
SAT_26M	2534.4	1469	0.0269802	0	Singlet	Adipocyte
SAT_26M	3681.1	1807	6.8889939	0	Singlet	Adipocyte
SAT_26M	2268.4	1151	0	6	Singlet	Neurons
SAT_26M	2407.3	1561	0	11	Singlet	Smooth_muscle_cells
SAT_26M	1751.8	1036	11.330262	9	Singlet	Mesothelial
SAT_26M	1948.8	1107	2.9334283	0	Singlet	Adipocyte
SAT_26M	1799.5	1108	0	8	Singlet	Mesothelial
SAT_26M	1328.8	828	7.0684502	3	Singlet	FAPs
SAT_26M	1080.5	741	0.0113637	1	Singlet	Immune_cells
SAT_26M	1587.9	1004	0.1063652	0	Singlet	Adipocyte
SAT_26M	4653.4	2326	0.8242873	0	Singlet	Adipocyte
SAT_26M	2234	1176	2.8387288	10	Singlet	Immune_cells
SAT_26M	1522.9	926	1.3490638	5	Singlet	Immune_cells
SAT_26M	2684.1	1517	8.361307	8	Singlet	Mesothelial
SAT_26M	1320.5	863	0.0279437	0	Singlet	Adipocyte
SAT_26M	1752.8	1012	1.9185312	10	Singlet	Immune_cells
SAT_26M	1736.2	1113	7.1558757	7	Singlet	Endothelial_cells
SAT_26M	1146.8	715	7.03684	0	Singlet	Adipocyte
SAT_26M	1901.7	1129	1.1338643	5	Singlet	Immune_cells
SAT_26M	2858.1	1313	11.873851	7	Singlet	Endothelial_cells
SAT_26M	1039.5	665	2.2117134	0	Singlet	Adipocyte
SAT_26M	1099.7	798	0.8101864	5	Singlet	Immune_cells
SAT_26M	4246.8	2362	1.4469663	11	Singlet	Smooth_muscle_cells
SAT_26M	1514.9	977	3.7802587	5	Singlet	Immune_cells
SAT_26M	1023	732	2.141832	0	Singlet	Adipocyte
SAT_26M	1417.3	883	1.9085805	0	Singlet	Adipocyte
SAT_26M	1239.3	910	3.1958698	0	Singlet	Adipocyte

SAT_26M	1149.1	881	1.7633648	5	Singlet	Immune_cells
SAT_26M	2319.5	1422	3.6641059	9	Singlet	Mesothelial
SAT_26M	1348	889	9.5949034	9	Singlet	Mesothelial
SAT_26M	1851.9	1028	6.95307	0	Singlet	Adipocyte
SAT_26M	1238.3	836	2.1700442	5	Singlet	Immune_cells
SAT_26M	3205.5	1771	2.0707492	8	Singlet	Mesothelial
SAT_26M	1919.2	1181	0.0833386	0	Singlet	Adipocyte
SAT_26M	1428.7	876	2.7150752	0	Singlet	Adipocyte
SAT_26M	1392.6	915	0	8	Singlet	Mesothelial
SAT_26M	1249	740	3.9060312	7	Singlet	Endothelial_cells
SAT_26M	1957.3	1285	0	8	Singlet	Mesothelial
SAT_26M	1392	912	1.5700939	0	Singlet	Adipocyte
SAT_26M	2685.4	1605	1.4933622	0	Singlet	Adipocyte
SAT_26M	1429.4	843	6.1332637	0	Singlet	Adipocyte
SAT_26M	1227.7	798	5.8792734	0	Singlet	Adipocyte
SAT_26M	1038.7	639	9.0113083	5	Singlet	Immune_cells
SAT_26M	2227.5	1290	2.9252985	7	Singlet	Endothelial_cells
SAT_26M	2293.1	1441	0.0548037	8	Singlet	Mesothelial
SAT_26M	3192.7	1569	1.7841526	10	Singlet	Immune_cells
SAT_26M	1049	614	1.2262076	10	Singlet	Immune_cells
SAT_26M	1236.7	866	0.151313	0	Singlet	Adipocyte
SAT_26M	1374.5	1013	0.6800641	3	Singlet	FAPs
SAT_26M	1946.5	1231	1.8029926	8	Singlet	Mesothelial
SAT_26M	1379	792	6.9925171	7	Singlet	Endothelial_cells
SAT_26M	1673.8	1104	7.0227815	0	Singlet	Adipocyte
SAT_26M	4591.9	2184	0.3144233	0	Singlet	Adipocyte
SAT_26M	1512.2	989	6.0745662	5	Singlet	Immune_cells
SAT_26M	3901.2	1923	0	0	Singlet	Adipocyte
SAT_26M	2588.2	1505	3.4542403	0	Singlet	Adipocyte
SAT_26M	1561.3	1102	0.2148572	1	Singlet	Immune_cells
SAT_26M	1546.3	1020	9.166933	8	Singlet	Mesothelial
SAT_26M	1767	985	8.2518688	0	Singlet	Adipocyte
SAT_26M	1902.3	1136	1.5495949	1	Singlet	Immune_cells
SAT_26M	2569	1353	0.1511586	0	Singlet	Adipocyte
SAT_26M	2932.5	1591	1.7209258	0	Singlet	Adipocyte
SAT_26M	1550.3	925	3.7467521	0	Singlet	Adipocyte
SAT_26M	4416.8	2184	0.0809108	0	Singlet	Adipocyte
SAT_26M	3088.4	1385	12.571267	7	Singlet	Endothelial_cells
SAT_26M	3934.9	1811	5.2326854	7	Singlet	Endothelial_cells
SAT_26M	6169.5	2687	0.5907265	0	Singlet	Adipocyte
SAT_26M	1968.2	984	0.0677038	5	Singlet	Immune_cells
SAT_26M	1219.3	547	4.1893672	3	Singlet	FAPs
SAT_26M	1490.7	907	3.7633427	7	Singlet	Endothelial_cells
SAT_26M	1260.6	775	2.3818419	6	Singlet	Neurons
SAT_26M	1321.3	879	1.1443929	0	Singlet	Adipocyte
SAT_26M	1108.7	791	1.7963349	1	Singlet	Immune_cells
SAT_26M	1384.7	727	0.1140056	7	Singlet	Endothelial_cells
SAT_26M	2572.1	1584	2.5838981	7	Singlet	Endothelial_cells
SAT_26M	1734.1	1124	2.2408876	1	Singlet	Immune_cells
SAT_26M	2183	1125	0.252757	0	Singlet	Adipocyte
SAT_26M	1143.8	755	0.0525639	0	Singlet	Adipocyte

SAT_26M	1567.5	895	9.3089289	0	Singlet	Adipocyte
SAT_26M	1049.9	711	0.4092863	6	Singlet	Neurons
SAT_26M	1507.6	921	2.0695211	1	Singlet	Immune_cells
SAT_26M	2705.8	1500	4.0355138	0	Singlet	Adipocyte
SAT_26M	1119.6	759	1.4464221	0	Singlet	Adipocyte
SAT_26M	2406.1	1372	12.205862	5	Singlet	Immune_cells
SAT_26M	1654.8	1010	0.0301543	3	Singlet	FAPs
SAT_26M	1689.1	1097	4.6907173	4	Singlet	Immune_cells
SAT_26M	3970.4	2010	0	0	Singlet	Adipocyte
SAT_26M	1335.6	747	0.5577281	0	Singlet	Adipocyte
SAT_26M	1514.2	681	5.4903071	7	Singlet	Endothelial_cells
SAT_26M	1837.9	1161	2.6990534	8	Singlet	Mesothelial
SAT_26M	1195.9	792	2.2275197	1	Singlet	Immune_cells
SAT_26M	1164.4	731	0.4995886	5	Singlet	Immune_cells
SAT_26M	2443.5	1220	0.0969363	10	Singlet	Immune_cells
SAT_26M	1204.4	822	5.2730225	4	Singlet	Immune_cells
SAT_26M	1499.3	877	6.6352772	0	Singlet	Adipocyte
SAT_26M	2880.5	1370	0.0308819	8	Singlet	Mesothelial
SAT_26M	2062.9	910	5.8823444	7	Singlet	Endothelial_cells
SAT_26M	2153.1	1248	0.5401091	0	Singlet	Adipocyte
SAT_26M	1291.6	744	3.9351267	7	Singlet	Endothelial_cells
SAT_26M	2131.1	1174	14.917917	9	Singlet	Mesothelial
SAT_26M	1390.1	891	3.1985545	9	Singlet	Mesothelial
SAT_26M	1122.5	745	9.8901972	7	Singlet	Endothelial_cells
SAT_26M	1204	807	1.5753432	7	Singlet	Endothelial_cells
SAT_26M	2226.3	1046	7.1062975	7	Singlet	Endothelial_cells
SAT_26M	2020.2	1161	1.7806865	0	Singlet	Adipocyte
SAT_26M	1709.1	1093	0.1341923	0	Singlet	Adipocyte
SAT_26M	1179.8	751	1.0740739	0	Singlet	Adipocyte
SAT_26M	2363.9	1162	0	6	Singlet	Neurons
SAT_26M	1194.9	749	2.8447538	7	Singlet	Endothelial_cells
SAT_26M	1452.9	896	0.943217	0	Singlet	Adipocyte
SAT_26M	2449.1	1482	9.4708633	9	Singlet	Mesothelial
SAT_26M	1506.3	978	0.1834157	3	Singlet	FAPs
SAT_26M	2125.8	1137	2.2511843	0	Singlet	Adipocyte
SAT_26M	4198.7	2030	0.0716788	0	Singlet	Adipocyte
SAT_26M	1201.4	801	0.3058349	0	Singlet	Adipocyte
SAT_26M	1283.5	793	1.8978243	0	Singlet	Adipocyte
SAT_26M	5368.4	2659	10.61908	9	Singlet	Mesothelial
SAT_26M	1356.8	852	3.5522465	0	Singlet	Adipocyte
SAT_26M	1766	1012	8.0188789	0	Singlet	Adipocyte
SAT_26M	1499.7	790	3.0541398	3	Singlet	FAPs
SAT_26M	2318.8	1355	0.5682377	0	Singlet	Adipocyte
SAT_26M	5537.9	2661	0.8724634	0	Singlet	Adipocyte
SAT_26M	2000.8	907	12.066677	0	Singlet	Adipocyte
SAT_26M	1718.3	934	2.4273803	10	Singlet	Immune_cells
SAT_26M	1369.8	894	2.7242108	7	Singlet	Endothelial_cells
SAT_26M	10225	4107	2.2590996	8	Singlet	Mesothelial
SAT_26M	1496.6	870	1.3832859	10	Singlet	Immune_cells
SAT_26M	2014.6	1287	0	11	Singlet	Smooth_muscle_cells
SAT_26M	1076.5	748	0	0	Singlet	Adipocyte

SAT_26M	1238.6	968	0.2076493	11 Singlet	Smooth_muscle_cells
SAT_26M	1521.4	985	0.8008215	7 Singlet	Endothelial_cells
SAT_26M	1345	788	0.0244726	8 Singlet	Mesothelial
SAT_26M	1296.9	881	0	5 Singlet	Immune_cells
SAT_26M	2202.1	1397	0.4989364	3 Singlet	FAPs
SAT_26M	1340.6	882	3.2831967	0 Singlet	Adipocyte
SAT_26M	2443	1280	4.8691758	0 Singlet	Adipocyte
SAT_26M	2586.3	1430	1.6115689	0 Singlet	Adipocyte
SAT_26M	1593	1030	4.1035072	7 Singlet	Endothelial_cells
SAT_26M	2035.3	1190	1.3893599	0 Singlet	Adipocyte
SAT_26M	1056.5	784	2.0327741	1 Singlet	Immune_cells
SAT_26M	1085.6	636	7.5551982	0 Singlet	Adipocyte
SAT_26M	1950.3	1189	2.4009197	0 Singlet	Adipocyte
SAT_26M	2848.4	1516	6.8361708	0 Singlet	Adipocyte
SAT_26M	1062.7	655	7.332627	0 Singlet	Adipocyte
SAT_26M	1418.1	884	0.205279	0 Singlet	Adipocyte
SAT_26M	1131.9	792	1.7525341	1 Singlet	Immune_cells
SAT_26M	1736.7	1139	1.0451358	8 Singlet	Mesothelial
SAT_26M	2059	1278	0.4421772	0 Singlet	Adipocyte
SAT_26M	1043.9	746	0.0440817	0 Singlet	Adipocyte
SAT_26M	1534.2	993	2.9759062	3 Singlet	FAPs
SAT_26M	1333.7	789	5.7520061	7 Singlet	Endothelial_cells
SAT_26M	1437	946	0.2035539	3 Singlet	FAPs
SAT_26M	1352.6	832	3.5510518	7 Singlet	Endothelial_cells
SAT_26M	3853.4	1696	12.492718	7 Singlet	Endothelial_cells
SAT_26M	1913.1	1103	1.5391496	0 Singlet	Adipocyte
SAT_26M	1051.9	752	0	6 Singlet	Neurons
SAT_26M	2027.8	1226	0.044334	0 Singlet	Adipocyte
SAT_26M	2376.9	1334	0.6875947	0 Singlet	Adipocyte
SAT_26M	1060.4	716	0.1946789	3 Singlet	FAPs
SAT_26M	1422.2	854	0	0 Singlet	Adipocyte
SAT_26M	1519.7	951	0.7000001	7 Singlet	Endothelial_cells
SAT_26M	1354.3	827	7.7738653	8 Singlet	Mesothelial
SAT_26M	1353.6	763	13.435492	0 Singlet	Adipocyte
SAT_26M	1639.6	954	0.7003604	0 Singlet	Adipocyte
SAT_26M	2218.9	1231	8.359276	0 Singlet	Adipocyte
SAT_26M	1259.4	866	3.5207721	1 Singlet	Immune_cells
SAT_26M	1396.2	806	2.9415551	0 Singlet	Adipocyte
SAT_26M	1736.4	1132	0.0638033	6 Singlet	Neurons
SAT_26M	3469.8	1983	8.7861666	0 Singlet	Adipocyte
SAT_26M	1182.7	790	0.0323347	0 Singlet	Adipocyte
SAT_26M	1423.6	776	11.392204	7 Singlet	Endothelial_cells
SAT_26M	1322.5	782	0	6 Singlet	Neurons
SAT_26M	2247	1377	4.2082466	8 Singlet	Mesothelial
SAT_26M	1322.5	811	1.8626173	0 Singlet	Adipocyte
SAT_26M	1199.6	717	3.4342193	5 Singlet	Immune_cells
SAT_26M	1193	851	0	7 Singlet	Endothelial_cells
SAT_26M	1060.7	594	8.2351068	7 Singlet	Endothelial_cells
SAT_26M	1212.4	684	10.643832	0 Singlet	Adipocyte
SAT_26M	4379	2350	8.1044751	9 Singlet	Mesothelial
SAT_26M	1003.7	745	2.5233047	1 Singlet	Immune_cells

SAT_26M	1236.3	753	4.329151	0	Singlet	Adipocyte
SAT_26M	1564.1	1033	0.0132491	6	Singlet	Neurons
SAT_26M	2919.6	1730	5.5289521	9	Singlet	Mesothelial
SAT_26M	1361.5	841	1.9912374	0	Singlet	Adipocyte
SAT_26M	1162	834	0	0	Singlet	Adipocyte
SAT_26M	1736.7	1088	1.2985591	0	Singlet	Adipocyte
SAT_26M	2395.1	1320	0.003512	8	Singlet	Mesothelial
SAT_26M	1063.3	701	10.053574	1	Singlet	Immune_cells
SAT_26M	1012.1	760	0.074168	1	Singlet	Immune_cells
SAT_26M	2747.9	1583	0	8	Singlet	Mesothelial
SAT_26M	2943.8	1718	3.6061122	7	Singlet	Endothelial_cells
SAT_26M	4320	1933	9.6956756	0	Singlet	Adipocyte
SAT_26M	2838.6	1612	1.1565349	0	Singlet	Adipocyte
SAT_26M	1462.1	1021	1.5173913	9	Singlet	Mesothelial
SAT_26M	1507.1	696	13.097001	0	Singlet	Adipocyte
SAT_26M	1512.9	799	5.5915407	10	Singlet	Immune_cells
SAT_26M	1058.3	670	4.418064	0	Singlet	Adipocyte
SAT_26M	1382.9	945	0	11	Singlet	Smooth_muscle_cells
SAT_26M	1728.2	1139	0	8	Singlet	Mesothelial
SAT_26M	1688.2	966	1.4824701	0	Singlet	Adipocyte
SAT_26M	2794.2	1707	3.4448901	8	Singlet	Mesothelial
SAT_26M	1356.6	895	1.6110372	3	Singlet	FAPs
SAT_26M	3435.3	1930	3.5487018	9	Singlet	Mesothelial
SAT_26M	1007.3	703	0.4522051	0	Singlet	Adipocyte
SAT_26M	1128.5	868	3.071511	5	Singlet	Immune_cells
SAT_26M	1153.7	781	5.0540159	3	Singlet	FAPs
SAT_26M	1318.1	807	5.1920024	0	Singlet	Adipocyte
SAT_26M	1686.4	1042	1.4326071	0	Singlet	Adipocyte
SAT_26M	1243.6	832	0.5900054	0	Singlet	Adipocyte
SAT_26M	1422.4	859	0.202439	0	Singlet	Adipocyte
SAT_26M	1729.5	1025	8.3401113	7	Singlet	Endothelial_cells
SAT_26M	1769.1	1141	0	8	Singlet	Mesothelial
SAT_26M	1160.8	818	3.5204547	1	Singlet	Immune_cells
SAT_26M	2149	1072	6.772116	10	Singlet	Immune_cells
SAT_26M	1012.6	757	0.1833786	0	Singlet	Adipocyte
SAT_26M	1999.8	1282	3.3190335	0	Singlet	Adipocyte
SAT_26M	1029.2	745	0.9854809	1	Singlet	Immune_cells
SAT_26M	3467.2	1881	2.0762493	0	Singlet	Adipocyte
SAT_26M	3214.7	1768	0.1509496	0	Singlet	Adipocyte
SAT_26M	1232.6	907	0.1198747	11	Singlet	Smooth_muscle_cells
SAT_26M	1514.1	948	0.1927196	0	Singlet	Adipocyte
SAT_26M	2003.7	1149	6.6679415	3	Singlet	FAPs
SAT_26M	1280.9	878	4.9287735	9	Singlet	Mesothelial
SAT_26M	4028	1963	0.9868176	0	Singlet	Adipocyte
SAT_26M	1074.2	814	1.2179846	4	Singlet	Immune_cells
SAT_26M	2272.2	1474	0.5126628	1	Singlet	Immune_cells
SAT_26M	1079.8	701	13.29532	1	Singlet	Immune_cells
SAT_26M	1085.8	711	3.6601078	7	Singlet	Endothelial_cells
SAT_26M	3076.1	1628	2.8988123	0	Singlet	Adipocyte
SAT_26M	1387.7	941	7.45219	0	Singlet	Adipocyte
SAT_26M	1195.4	750	1.2854901	0	Singlet	Adipocyte

SAT_26M	1578.1	1020	14.022252	9	Singlet	Mesothelial
SAT_26M	2142	1404	5.5412899	0	Singlet	Adipocyte
SAT_26M	1741	1233	0.1522852	6	Singlet	Neurons
SAT_26M	1307.9	736	4.9075946	0	Singlet	Adipocyte
SAT_26M	1784.3	1083	1.2064912	7	Singlet	Endothelial_cells
SAT_26M	1869	1151	0.0824587	0	Singlet	Adipocyte
SAT_26M	2217.5	1379	1.596078	1	Singlet	Immune_cells
SAT_26M	1224.5	820	0.5239489	0	Singlet	Adipocyte
SAT_26M	1545.9	1101	0.2852483	0	Singlet	Adipocyte
SAT_26M	1525.6	989	2.1488438	10	Singlet	Immune_cells
SAT_26M	1878.5	976	0.9822542	10	Singlet	Immune_cells
SAT_26M	2545.3	1468	2.5307398	7	Singlet	Endothelial_cells
SAT_26M	2758.3	1510	6.5160983	7	Singlet	Endothelial_cells
SAT_26M	2133.8	1217	1.215372	0	Singlet	Adipocyte
SAT_26M	1562.3	1025	0.3476388	0	Singlet	Adipocyte
SAT_26M	1513	910	0.0166213	3	Singlet	FAPs
SAT_26M	1025.8	704	0	8	Singlet	Mesothelial
SAT_26M	1638.2	1024	0	0	Singlet	Adipocyte
SAT_26M	1602	1043	0	6	Singlet	Neurons
SAT_26M	1023.8	709	1.6058458	0	Singlet	Adipocyte
SAT_26M	3117.5	1407	11.515766	7	Singlet	Endothelial_cells
SAT_26M	2036.9	1234	5.74571	7	Singlet	Endothelial_cells
SAT_26M	1951.5	1107	2.1772515	0	Singlet	Adipocyte
SAT_26M	1904.7	806	13.598585	0	Singlet	Adipocyte
SAT_26M	2013.1	1246	0.012712	5	Singlet	Immune_cells
SAT_26M	2140.3	1280	5.2555053	0	Singlet	Adipocyte
SAT_26M	1388.7	774	5.8283352	0	Singlet	Adipocyte
SAT_26M	1255.6	749	2.141881	7	Singlet	Endothelial_cells
SAT_26M	2371.5	1443	7.2733715	8	Singlet	Mesothelial
SAT_26M	1362.9	1055	1.4883809	1	Singlet	Immune_cells
SAT_26M	2443.8	1286	7.2751941	0	Singlet	Adipocyte
SAT_26M	1917.9	1167	3.2605054	0	Singlet	Adipocyte
SAT_26M	1223.5	769	8.5502017	1	Singlet	Immune_cells
SAT_26M	2384.2	1586	1.1485989	11	Singlet	Smooth_muscle_cells
SAT_26M	1017.7	659	5.9857427	3	Singlet	FAPs
SAT_26M	1320.9	872	0.0903703	0	Singlet	Adipocyte
SAT_26M	1361.1	850	10.385193	5	Singlet	Immune_cells
SAT_26M	1165.5	714	11.431427	8	Singlet	Mesothelial
SAT_26M	1866.4	1206	0.1187989	0	Singlet	Adipocyte
SAT_26M	1278.7	758	0.4470156	5	Singlet	Immune_cells
SAT_26M	2829.5	1543	0.0401265	0	Singlet	Adipocyte
SAT_26M	1523.5	1041	1.5393177	1	Singlet	Immune_cells
SAT_26M	1788.7	1085	0.831711	0	Singlet	Adipocyte
SAT_26M	3673	1829	4.9324039	0	Singlet	Adipocyte
SAT_26M	1405	922	0.5902838	0	Singlet	Adipocyte
SAT_26M	1514.7	970	2.2761897	0	Singlet	Adipocyte
SAT_26M	1216.4	811	0	0	Singlet	Adipocyte
SAT_26M	2969.4	1632	11.255757	9	Singlet	Mesothelial
SAT_26M	1386.6	893	3.4445034	8	Singlet	Mesothelial
SAT_26M	2079.5	1309	0	8	Singlet	Mesothelial
SAT_26M	3703.9	1884	1.5280974	0	Singlet	Adipocyte

SAT_26M	2584.1	1516	0.0002124	8	Singlet	Mesothelial
SAT_26M	1908.1	1071	3.4424381	0	Singlet	Adipocyte
SAT_26M	1170.5	842	1.8776442	10	Singlet	Immune_cells
SAT_26M	1676.1	1026	0.4580165	0	Singlet	Adipocyte
SAT_26M	1853.5	1177	0.3939831	0	Singlet	Adipocyte
SAT_26M	2442.6	1358	0.324817	0	Singlet	Adipocyte
SAT_26M	1419.4	915	2.1701974	0	Singlet	Adipocyte
SAT_26M	1254.6	821	2.4879503	0	Singlet	Adipocyte
SAT_26M	1568.4	935	6.4580801	0	Singlet	Adipocyte
SAT_26M	1231.7	684	11.964183	7	Singlet	Endothelial_cells
SAT_26M	1162	738	1.2659585	6	Singlet	Neurons
SAT_26M	1585.3	852	8.9699518	10	Singlet	Immune_cells
SAT_26M	1798.4	1090	2.9818605	0	Singlet	Adipocyte
SAT_26M	1686.4	1055	6.1916869	9	Singlet	Mesothelial
SAT_26M	1437.2	906	4.1266633	7	Singlet	Endothelial_cells
SAT_26M	1617.7	989	1.8230946	0	Singlet	Adipocyte
SAT_26M	1286.6	949	0.1167523	5	Singlet	Immune_cells
SAT_26M	3552.5	1907	1.875071	0	Singlet	Adipocyte
SAT_26M	5157.1	2385	0.1282624	0	Singlet	Adipocyte
SAT_26M	1613.8	1019	2.4481465	0	Singlet	Adipocyte
SAT_26M	1392.3	948	0	8	Singlet	Mesothelial
SAT_26M	3682.1	1972	0.0052169	0	Singlet	Adipocyte
SAT_26M	1276.6	913	0.3451487	0	Singlet	Adipocyte
SAT_26M	1116	831	3.8745777	1	Singlet	Immune_cells
SAT_26M	2048.3	1281	0.1758255	0	Singlet	Adipocyte
SAT_26M	1404.3	918	0.0231977	0	Singlet	Adipocyte
SAT_26M	1662.3	1124	0	8	Singlet	Mesothelial
SAT_26M	1416.7	895	6.1513829	8	Singlet	Mesothelial
SAT_26M	2546.1	1232	14.019185	7	Singlet	Endothelial_cells
SAT_26M	1159.4	664	7.0667981	10	Singlet	Immune_cells
SAT_26M	1185.1	916	4.2374353	4	Singlet	Immune_cells
SAT_26M	1001.6	757	0.913605	11	Singlet	Smooth_muscle_cells
SAT_26M	1591.6	1140	5.7523986	11	Singlet	Smooth_muscle_cells
SAT_26M	1968.7	1107	9.600127	0	Singlet	Adipocyte
SAT_26M	1491.4	1054	3.681157	1	Singlet	Immune_cells
SAT_26M	1368.2	848	2.1874071	0	Singlet	Adipocyte
SAT_26M	2582	1472	1.7963574	0	Singlet	Adipocyte
SAT_26M	1113	738	1.9116742	0	Singlet	Adipocyte
SAT_26M	1078.9	610	10.198168	3	Singlet	FAPs
SAT_26M	1273	906	0.4856302	6	Singlet	Neurons
SAT_26M	1676.5	988	1.6900403	10	Singlet	Immune_cells
SAT_26M	1632.4	1003	3.0193908	0	Singlet	Adipocyte
SAT_26M	3699.2	1816	6.3164211	0	Singlet	Adipocyte
SAT_26M	1070.5	663	4.3601577	7	Singlet	Endothelial_cells
SAT_26M	1989.2	1326	2.8559103	1	Singlet	Immune_cells
SAT_26M	2420.8	1503	0	8	Singlet	Mesothelial
SAT_26M	1249.3	821	8.8801014	8	Singlet	Mesothelial
SAT_26M	2427.4	1277	6.0551372	0	Singlet	Adipocyte
SAT_26M	1239.6	752	1.5642665	0	Singlet	Adipocyte
SAT_26M	2852.2	1485	2.2985076	5	Singlet	Immune_cells
SAT_26M	1027.6	692	5.7609729	7	Singlet	Endothelial_cells

SAT_26M	1512.1	921	0.2303016	0	Singlet	Adipocyte
SAT_26M	1137.9	785	0.2955788	0	Singlet	Adipocyte
SAT_26M	1050.9	721	0	3	Singlet	FAPs
SAT_26M	1156.6	772	3.5179117	0	Singlet	Adipocyte
SAT_26M	4283.6	2197	0.0334658	0	Singlet	Adipocyte
SAT_26M	2204.3	1210	6.4749458	0	Singlet	Adipocyte
SAT_26M	1347.6	822	4.0550288	0	Singlet	Adipocyte
SAT_26M	2043.8	1295	0.6031692	0	Singlet	Adipocyte
SAT_26M	1922	1241	5.6461284	4	Singlet	Immune_cells
SAT_26M	1530.1	896	8.6836821	7	Singlet	Endothelial_cells
SAT_26M	1282.9	800	0.1471621	0	Singlet	Adipocyte
SAT_26M	2507.8	1416	0	8	Singlet	Mesothelial
SAT_26M	1027	679	0.5366045	3	Singlet	FAPs
SAT_26M	1164	765	3.1911659	7	Singlet	Endothelial_cells
SAT_26M	1987.4	1160	3.7902116	0	Singlet	Adipocyte
SAT_26M	1032.9	648	3.3829486	10	Singlet	Immune_cells
SAT_26M	3032.2	1627	1.0059623	0	Singlet	Adipocyte
SAT_26M	1186	874	0.456899	0	Singlet	Adipocyte
SAT_26M	2785.6	1125	5.3441616	0	Singlet	Adipocyte
SAT_26M	2409.8	1265	8.1674253	0	Singlet	Adipocyte
SAT_26M	1732.5	1128	0.8603192	4	Singlet	Immune_cells
SAT_26M	2149.9	1242	0.0310716	0	Singlet	Adipocyte
SAT_26M	2209.2	1052	9.8324108	7	Singlet	Endothelial_cells
SAT_26M	1123.6	770	1.5145326	0	Singlet	Adipocyte
SAT_26M	2954.4	1589	0.8982689	0	Singlet	Adipocyte
SAT_26M	1222.1	745	0	6	Singlet	Neurons
SAT_26M	1520.2	944	7.0089207	8	Singlet	Mesothelial
SAT_26M	1362.7	828	12.405141	0	Singlet	Adipocyte
SAT_26M	1034	693	0.1698433	8	Singlet	Mesothelial
SAT_26M	4281.3	2073	0.0373329	0	Singlet	Adipocyte
SAT_26M	6970.1	3298	5.8069571	9	Singlet	Mesothelial
SAT_26M	1137.4	854	3.1670799	1	Singlet	Immune_cells
SAT_26M	1216.2	839	0.031489	0	Singlet	Adipocyte
SAT_26M	3162.9	1765	4.6058551	0	Singlet	Adipocyte
SAT_26M	1132.8	777	0.7641098	0	Singlet	Adipocyte
SAT_26M	2418.8	1417	5.2092733	0	Singlet	Adipocyte
SAT_26M	1249.7	811	0.1139524	7	Singlet	Endothelial_cells
SAT_26M	1887	1167	7.6916768	7	Singlet	Endothelial_cells
SAT_26M	1221.8	807	7.7291029	7	Singlet	Endothelial_cells
SAT_26M	3497.7	2111	3.1952832	8	Singlet	Mesothelial
SAT_26M	1680	940	4.2212175	0	Singlet	Adipocyte
SAT_26M	1192.5	729	0.3927729	0	Singlet	Adipocyte
SAT_26M	1306.5	876	6.4448342	0	Singlet	Adipocyte
SAT_26M	1327.1	731	8.9573114	7	Singlet	Endothelial_cells
SAT_26M	4184.2	2121	3.7101833	9	Singlet	Mesothelial
SAT_26M	1429.1	810	13.271771	0	Singlet	Adipocyte
SAT_26M	1070.7	634	4.9399281	0	Singlet	Adipocyte
SAT_26M	1792.6	1087	0.9717927	0	Singlet	Adipocyte
SAT_26M	1772.8	1080	0.879474	3	Singlet	FAPs
SAT_26M	1671.7	863	11.640842	7	Singlet	Endothelial_cells
SAT_26M	6981.5	3202	2.7606677	9	Singlet	Mesothelial

SAT_26M	1613.5	992	0.5613754	5	Singlet	Immune_cells
SAT_26M	1451.7	1041	0.0168308	8	Singlet	Mesothelial
SAT_26M	1295.2	831	0.1222084	0	Singlet	Adipocyte
SAT_26M	2029.4	1199	2.8592145	0	Singlet	Adipocyte
SAT_26M	2665.5	1382	10.272201	7	Singlet	Endothelial_cells
SAT_26M	1232.6	915	3.6279954	1	Singlet	Immune_cells
SAT_26M	1352.4	917	0.9208125	3	Singlet	FAPs
SAT_26M	1814.9	1101	0	3	Singlet	FAPs
SAT_26M	2465.6	1314	3.325886	10	Singlet	Immune_cells
SAT_26M	3735.7	1944	1.7219335	0	Singlet	Adipocyte
SAT_26M	3856.2	1634	11.028003	7	Singlet	Endothelial_cells
SAT_26M	5083.1	2372	0.0719571	0	Singlet	Adipocyte
SAT_26M	1235	781	3.7073109	7	Singlet	Endothelial_cells
SAT_26M	2499.3	1320	10.468894	0	Singlet	Adipocyte
SAT_26M	1860.5	1230	0.3897135	5	Singlet	Immune_cells
SAT_26M	1789	1186	2.2304247	5	Singlet	Immune_cells
SAT_26M	1450	915	7.6903384	0	Singlet	Adipocyte
SAT_26M	2554	1466	4.6821192	0	Singlet	Adipocyte
SAT_26M	2468.9	1198	8.9894277	7	Singlet	Endothelial_cells
SAT_26M	1399.6	934	0.3827348	0	Singlet	Adipocyte
SAT_26M	1035.8	731	1.1346901	6	Singlet	Neurons
SAT_26M	1341.2	934	0.1730036	5	Singlet	Immune_cells
SAT_26M	2547.3	1384	6.4915994	0	Singlet	Adipocyte
SAT_26M	2254.6	1335	0.1845222	0	Singlet	Adipocyte
SAT_26M	4249	2146	3.2976832	0	Singlet	Adipocyte
SAT_26M	2661.8	1694	2.4739096	8	Singlet	Mesothelial
SAT_26M	4677.8	2277	0.8347283	5	Singlet	Immune_cells
SAT_26M	1349.5	994	2.4567608	1	Singlet	Immune_cells
SAT_26M	1702.6	1069	1.4231504	0	Singlet	Adipocyte
SAT_26M	1308.5	833	1.3657216	0	Singlet	Adipocyte
SAT_26M	2073.7	1142	10.484407	7	Singlet	Endothelial_cells
SAT_26M	1019.4	651	5.1142962	0	Singlet	Adipocyte
SAT_26M	1160.1	744	1.8396695	0	Singlet	Adipocyte
SAT_26M	1869.9	1029	1.8062789	10	Singlet	Immune_cells
SAT_26M	3715.5	1734	1.4619314	10	Singlet	Immune_cells
SAT_26M	2054.2	1298	3.4094853	0	Singlet	Adipocyte
SAT_26M	2302.4	1455	1.4953576	8	Singlet	Mesothelial
SAT_26M	2778.2	1564	4.8927052	7	Singlet	Endothelial_cells
SAT_26M	1109.5	663	0.1563539	6	Singlet	Neurons
SAT_26M	3322.7	1697	10.534348	0	Singlet	Adipocyte
SAT_26M	3765.8	1907	0.0887083	0	Singlet	Adipocyte
SAT_26M	2072.7	1266	0.3010785	7	Singlet	Endothelial_cells
SAT_26M	3291.7	1838	14.736595	9	Singlet	Mesothelial
SAT_26M	1075	787	1.873402	1	Singlet	Immune_cells
SAT_26M	2031.9	1242	4.8252604	8	Singlet	Mesothelial
SAT_26M	1831.7	875	12.289547	7	Singlet	Endothelial_cells
SAT_26M	1506.2	862	10.643805	0	Singlet	Adipocyte
SAT_26M	1389.1	862	8.1634304	7	Singlet	Endothelial_cells
SAT_26M	2893.8	1622	0.2466405	0	Singlet	Adipocyte
SAT_26M	1898.7	1059	2.2432532	10	Singlet	Immune_cells
SAT_26M	1049.2	746	1.310066	0	Singlet	Adipocyte

SAT_26M	1339	802	0.5605119	5	Singlet	Immune_cells
SAT_26M	1644.3	968	5.6724568	0	Singlet	Adipocyte
SAT_26M	2067.9	1258	1.4451697	5	Singlet	Immune_cells
SAT_26M	2725.6	1455	5.652095	0	Singlet	Adipocyte
SAT_26M	2436	1399	1.6861762	0	Singlet	Adipocyte
SAT_26M	3307.6	1585	1.5741828	10	Singlet	Immune_cells
SAT_26M	1724.5	1079	0.442935	0	Singlet	Adipocyte
SAT_26M	2383.4	1396	0.3470355	0	Singlet	Adipocyte
SAT_26M	1196.7	842	0	5	Singlet	Immune_cells
SAT_26M	1058.4	693	4.5141174	8	Singlet	Mesothelial
SAT_26M	2197.5	1289	6.2637894	8	Singlet	Mesothelial
SAT_26M	3547.5	2030	3.0137275	9	Singlet	Mesothelial
SAT_26M	1853.8	1167	0.2564824	0	Singlet	Adipocyte
SAT_26M	1124.9	836	3.7621663	3	Singlet	FAPs
SAT_26M	2908.5	1538	0.7664722	0	Singlet	Adipocyte
SAT_26M	4465.6	2144	0.9415838	0	Singlet	Adipocyte
SAT_26M	1248	838	6.5306754	9	Singlet	Mesothelial
SAT_26M	1054.4	736	0.2335659	0	Singlet	Adipocyte
SAT_26M	1565.5	969	0.0519433	0	Singlet	Adipocyte
SAT_26M	1228.1	870	2.8083734	1	Singlet	Immune_cells
SAT_26M	1368.8	923	1.2941902	0	Singlet	Adipocyte
SAT_26M	1788.1	903	11.312282	0	Singlet	Adipocyte
SAT_26M	1303.6	981	0.7591599	8	Singlet	Mesothelial
SAT_26M	1604.1	989	5.3895769	8	Singlet	Mesothelial
SAT_26M	1276.2	846	1.5566756	0	Singlet	Adipocyte
SAT_26M	1003.6	645	0.0045314	6	Singlet	Neurons
SAT_26M	3726.1	1976	1.2450061	0	Singlet	Adipocyte
SAT_26M	1102	809	1.9294153	1	Singlet	Immune_cells
SAT_26M	2061.6	1247	0.3545324	0	Singlet	Adipocyte
SAT_26M	1883.6	1268	0.1791423	0	Singlet	Adipocyte
SAT_26M	1339.4	833	0.0273689	0	Singlet	Adipocyte
SAT_26M	1179.2	757	0.7705475	0	Singlet	Adipocyte
SAT_26M	1084.2	883	2.6420601	1	Singlet	Immune_cells
SAT_26M	1035	570	3.836171	5	Singlet	Immune_cells
SAT_26M	1769.2	1059	0.3415015	0	Singlet	Adipocyte
SAT_26M	1071	714	1.4901632	0	Singlet	Adipocyte
SAT_26M	1503.7	974	0.4740074	0	Singlet	Adipocyte
SAT_26M	1087.3	751	0.7550298	3	Singlet	FAPs
SAT_26M	3275.6	1925	2.7743337	5	Singlet	Immune_cells
SAT_26M	1328.6	676	8.8083417	10	Singlet	Immune_cells
SAT_26M	1268.1	871	1.6411434	8	Singlet	Mesothelial
SAT_26M	1030.4	613	12.904543	7	Singlet	Endothelial_cells
SAT_26M	1129.9	758	2.6666134	3	Singlet	FAPs
SAT_26M	1830	1179	0.0467642	8	Singlet	Mesothelial
SAT_26M	1686.4	1120	4.0755781	9	Singlet	Mesothelial
SAT_26M	1945	1112	0.8432633	0	Singlet	Adipocyte
SAT_26M	1726.1	1195	0.1593336	8	Singlet	Mesothelial
SAT_26M	1256.7	831	0.0178788	0	Singlet	Adipocyte
SAT_26M	1875	1038	1.1541994	0	Singlet	Adipocyte
SAT_26M	1648.8	999	0.1642778	0	Singlet	Adipocyte
SAT_26M	1209.8	795	0.425712	0	Singlet	Adipocyte

SAT_26M	1311.7	969	2.3011734	11	Singlet	Smooth_muscle_cells
SAT_26M	1178.6	804	0.0350601	3	Singlet	FAPs
SAT_26M	1014.5	736	0.1444801	6	Singlet	Neurons
SAT_26M	1197.5	733	5.9690917	0	Singlet	Adipocyte
SAT_26M	2165.8	1183	0.189968	0	Singlet	Adipocyte
SAT_26M	1128.8	753	0	0	Singlet	Adipocyte
SAT_26M	1032.2	720	0.5523245	6	Singlet	Neurons
SAT_26M	3161.6	1698	5.173777	0	Singlet	Adipocyte
SAT_26M	1318.2	939	0.0390193	0	Singlet	Adipocyte
SAT_26M	1159.5	757	4.2398147	7	Singlet	Endothelial_cells
SAT_26M	2096.8	1287	0.1662933	5	Singlet	Immune_cells
SAT_26M	1465.4	882	1.2388394	0	Singlet	Adipocyte
SAT_26M	1548.4	986	0.0930806	0	Singlet	Adipocyte
SAT_26M	1184.1	708	3.3037021	3	Singlet	FAPs
SAT_26M	4267.8	2141	0.0533083	0	Singlet	Adipocyte
SAT_26M	2038.9	1097	12.714168	7	Singlet	Endothelial_cells
SAT_26M	1618.3	1192	1.5257933	11	Singlet	Smooth_muscle_cells
SAT_26M	1421.4	929	0.1647168	0	Singlet	Adipocyte
SAT_26M	4628.2	2215	0.3702467	0	Singlet	Adipocyte
SAT_26M	3111.1	1660	1.9602392	0	Singlet	Adipocyte
SAT_26M	2587.7	1389	1.3375815	0	Singlet	Adipocyte
SAT_26M	1438.6	879	0.0388634	0	Singlet	Adipocyte
SAT_26M	2686.2	1434	3.8727348	0	Singlet	Adipocyte
SAT_26M	1212.4	787	1.69102	5	Singlet	Immune_cells
SAT_26M	1029.3	732	0	11	Singlet	Smooth_muscle_cells
SAT_26M	1398.7	867	6.3664661	0	Singlet	Adipocyte
SAT_26M	1167.4	676	7.0389078	6	Singlet	Neurons
SAT_26M	1241.1	779	0.0251938	2	Singlet	FAPs
SAT_26M	1623.9	929	4.5998666	7	Singlet	Endothelial_cells
SAT_26M	1115.4	779	0.4063457	0	Singlet	Adipocyte
SAT_26M	1852.3	1118	0.5011013	0	Singlet	Adipocyte
SAT_26M	1089.8	719	4.2987889	0	Singlet	Adipocyte
SAT_26M	1801.9	1090	3.6689218	7	Singlet	Endothelial_cells
SAT_26M	2165.3	1321	0.0112093	0	Singlet	Adipocyte
SAT_26M	1596.4	1065	2.6547681	0	Singlet	Adipocyte
SAT_26M	1442.3	938	4.0058567	8	Singlet	Mesothelial
SAT_26M	4999.4	2389	12.893682	9	Singlet	Mesothelial
SAT_26M	1748.9	1101	0.4117041	0	Singlet	Adipocyte
SAT_26M	1228.8	645	2.8208275	5	Singlet	Immune_cells
SAT_26M	1680.1	1057	2.3116657	5	Singlet	Immune_cells
SAT_26M	3059.5	1646	0.6007744	0	Singlet	Adipocyte
SAT_26M	2124.9	1229	3.104885	0	Singlet	Adipocyte
SAT_26M	2551.5	1383	3.3535033	0	Singlet	Adipocyte
SAT_26M	1556	1003	2.2191582	0	Singlet	Adipocyte
SAT_26M	1348.2	966	0.7130914	0	Singlet	Adipocyte
SAT_26M	1636.3	1216	4.90864	4	Singlet	Immune_cells
SAT_26M	2594.9	1501	3.4092785	0	Singlet	Adipocyte
SAT_26M	1045.6	719	1.0050463	3	Singlet	FAPs
SAT_26M	1688.3	1006	3.1501002	0	Singlet	Adipocyte
SAT_26M	2250.2	1265	13.565936	9	Singlet	Mesothelial
SAT_26M	2175.5	1270	0.9144279	0	Singlet	Adipocyte

SAT_26M	1274.9	881	0.3593315	0	Singlet	Adipocyte
SAT_26M	1879.4	1258	2.6911208	0	Singlet	Adipocyte
SAT_26M	1357.4	946	0.0995144	6	Singlet	Neurons
SAT_26M	1020.4	728	0	5	Singlet	Immune_cells
SAT_26M	4128.4	1773	10.131568	7	Singlet	Endothelial_cells
SAT_26M	1226	837	1.1160183	9	Singlet	Mesothelial
SAT_26M	2893.2	1499	7.9660144	9	Singlet	Mesothelial
SAT_26M	1030	740	0.1271651	8	Singlet	Mesothelial
SAT_26M	1784.2	1050	1.4049928	10	Singlet	Immune_cells
SAT_26M	1851.4	1179	1.0539798	0	Singlet	Adipocyte
SAT_26M	1360.3	916	3.8797155	3	Singlet	FAPs
SAT_26M	2260.2	1327	0.1161211	0	Singlet	Adipocyte
SAT_26M	3608.4	2112	2.2946764	9	Singlet	Mesothelial
SAT_26M	3985.1	2027	2.012553	0	Singlet	Adipocyte
SAT_26M	4295.4	1884	10.498802	7	Singlet	Endothelial_cells
SAT_26M	1164	861	1.5911294	1	Singlet	Immune_cells
SAT_26M	1401.3	940	2.6775179	0	Singlet	Adipocyte
SAT_26M	1866.7	1142	5.4457538	0	Singlet	Adipocyte
SAT_26M	1285.6	920	0.0064053	11	Singlet	Smooth_muscle_cells
SAT_26M	1339.5	764	7.3994695	7	Singlet	Endothelial_cells
SAT_26M	1020.1	731	3.0256389	1	Singlet	Immune_cells
SAT_26M	1752.8	1229	0.0031003	11	Singlet	Smooth_muscle_cells
SAT_26M	3908.7	1987	9.8902504	7	Singlet	Endothelial_cells
SAT_26M	1075.1	722	2.7685316	3	Singlet	FAPs
SAT_26M	1017.5	706	14.800092	1	Singlet	Immune_cells
SAT_26M	1159.4	822	0.663358	6	Singlet	Neurons
SAT_26M	2072.7	1290	0.4757961	0	Singlet	Adipocyte
SAT_26M	1347.3	904	1.5274189	0	Singlet	Adipocyte
SAT_26M	2058.1	1256	2.6810697	0	Singlet	Adipocyte
SAT_26M	1600.8	1070	1.1456088	7	Singlet	Endothelial_cells
SAT_26M	1146.3	715	11.008103	8	Singlet	Mesothelial
SAT_26M	1352.5	817	1.6616962	0	Singlet	Adipocyte
SAT_26M	1103.7	750	0.2077291	0	Singlet	Adipocyte
SAT_26M	1587.6	1005	5.7080863	5	Singlet	Immune_cells
SAT_26M	1143.3	795	4.5083696	3	Singlet	FAPs
SAT_26M	1989.7	1238	0	0	Singlet	Adipocyte
SAT_26M	2015.4	1188	4.800506	0	Singlet	Adipocyte
SAT_26M	2135.6	1224	0.6219799	0	Singlet	Adipocyte
SAT_26M	2183.2	920	2.1972185	10	Singlet	Immune_cells
SAT_26M	1689	1167	0.1986233	8	Singlet	Mesothelial
SAT_26M	3135.1	1771	1.3430344	8	Singlet	Mesothelial
SAT_26M	1133.3	797	0.2760565	0	Singlet	Adipocyte
SAT_26M	1388.6	845	14.003104	7	Singlet	Endothelial_cells
SAT_26M	2541.1	1404	4.0817783	0	Singlet	Adipocyte
SAT_26M	1065.6	642	6.189762	3	Singlet	FAPs
SAT_26M	2350.9	1332	0.6334344	3	Singlet	FAPs
SAT_26M	2865	1839	0	8	Singlet	Mesothelial
SAT_26M	2724.2	1467	2.3195029	0	Singlet	Adipocyte
SAT_26M	4954	2370	0.7123505	0	Singlet	Adipocyte
SAT_26M	2329.6	1495	0	11	Singlet	Smooth_muscle_cells
SAT_26M	3398.6	1915	4.5569832	7	Singlet	Endothelial_cells

SAT_26M	1409.8	1015	0.3858728	0	Singlet	Adipocyte
SAT_26M	1625.9	1000	2.4303125	0	Singlet	Adipocyte
SAT_26M	6113.9	2888	11.539329	9	Singlet	Mesothelial
SAT_26M	1346.1	891	0.1457831	0	Singlet	Adipocyte
SAT_26M	1546	983	0.3166536	0	Singlet	Adipocyte
SAT_26M	1265.1	877	0.4828938	0	Singlet	Adipocyte
SAT_26M	2748.1	1575	1.6767513	0	Singlet	Adipocyte
SAT_26M	3480.7	1821	1.0942583	0	Singlet	Adipocyte
SAT_26M	1392.4	955	3.2847615	7	Singlet	Endothelial_cells
SAT_26M	1393	911	1.4438925	0	Singlet	Adipocyte
SAT_26M	1966.2	1106	1.7033462	0	Singlet	Adipocyte
SAT_26M	1525.2	965	0.2508463	0	Singlet	Adipocyte
SAT_26M	1417.1	898	2.1920979	0	Singlet	Adipocyte
SAT_26M	1488.9	991	0.0178661	8	Singlet	Mesothelial
SAT_26M	2407.4	1323	1.9464385	0	Singlet	Adipocyte
SAT_26M	1458.2	978	4.6762002	1	Singlet	Immune_cells
SAT_26M	1603.9	966	2.146082	0	Singlet	Adipocyte
SAT_26M	1043.6	779	0.2031184	0	Singlet	Adipocyte
SAT_26M	1578.9	1040	0.7859484	7	Singlet	Endothelial_cells
SAT_26M	1518.7	1042	1.2551805	11	Singlet	Smooth_muscle_cells
SAT_26M	1209.1	609	11.277089	0	Singlet	Adipocyte
SAT_26M	3304.6	1743	5.4901235	0	Singlet	Adipocyte
SAT_26M	1319.4	890	0.1647634	0	Singlet	Adipocyte
SAT_26M	1956.2	1287	0.0851969	8	Singlet	Mesothelial
SAT_26M	1329.6	847	0	0	Singlet	Adipocyte
SAT_26M	1368.8	734	14.926004	0	Singlet	Adipocyte
SAT_26M	1715.6	1146	0.4920531	0	Singlet	Adipocyte
SAT_26M	1060.5	751	0.3882511	3	Singlet	FAPs
SAT_26M	1008.5	719	0.52017	3	Singlet	FAPs
SAT_26M	1704.1	1018	8.142546	0	Singlet	Adipocyte
SAT_26M	1244.7	756	6.0482938	0	Singlet	Adipocyte
SAT_26M	1461.6	1018	0	8	Singlet	Mesothelial
SAT_26M	1338.5	632	11.582716	0	Singlet	Adipocyte
SAT_26M	1122.5	843	2.0251817	1	Singlet	Immune_cells
SAT_26M	4018.4	2038	6.0074556	7	Singlet	Endothelial_cells
SAT_26M	1431.4	819	0.988279	5	Singlet	Immune_cells
SAT_26M	1125.4	736	0.0818341	0	Singlet	Adipocyte
SAT_26M	1031.9	732	1.1535579	3	Singlet	FAPs
SAT_26M	1777.7	1111	3.6688782	7	Singlet	Endothelial_cells
SAT_26M	1251.8	743	2.3478602	0	Singlet	Adipocyte
SAT_26M	1427	940	0.2653314	7	Singlet	Endothelial_cells
SAT_26M	1668.4	898	0.7467206	10	Singlet	Immune_cells
SAT_26M	2162.3	1212	2.6447569	0	Singlet	Adipocyte
SAT_26M	1124	801	6.9844809	8	Singlet	Mesothelial
SAT_26M	2802.7	1482	0.1533437	5	Singlet	Immune_cells
SAT_26M	2505.4	1479	4.1441401	7	Singlet	Endothelial_cells
SAT_26M	2001.6	1179	6.9975954	0	Singlet	Adipocyte
SAT_26M	1684	1049	1.1662517	0	Singlet	Adipocyte
SAT_26M	1573.5	1029	1.9850992	3	Singlet	FAPs
SAT_26M	1230.6	878	3.2248244	5	Singlet	Immune_cells
SAT_26M	1351.8	821	0.6607071	0	Singlet	Adipocyte

SAT_26M	1051.9	722	0.0344159	0	Singlet	Adipocyte
SAT_26M	2709.9	1596	0.0095988	0	Singlet	Adipocyte
SAT_26M	1960.6	1236	0	8	Singlet	Mesothelial
SAT_26M	2179.4	1290	1.3613081	5	Singlet	Immune_cells
SAT_26M	1599.9	1051	0.1099416	0	Singlet	Adipocyte
SAT_26M	2244.2	1446	0.1030667	8	Singlet	Mesothelial
SAT_26M	4364.2	2242	0.2293506	0	Singlet	Adipocyte
SAT_26M	1005.2	692	1.089836	10	Singlet	Immune_cells
SAT_26M	1062.4	776	0.1655008	11	Singlet	Smooth_muscle_cells
SAT_26M	1162.1	806	2.9947352	3	Singlet	FAPs
SAT_26M	1879.3	1014	0.9280805	10	Singlet	Immune_cells
SAT_26M	2749.7	1433	3.8403942	10	Singlet	Immune_cells
SAT_26M	1192.7	781	6.228754	6	Singlet	Neurons
SAT_26M	1581.3	991	0.7899186	0	Singlet	Adipocyte
SAT_26M	2031	1150	2.8057885	0	Singlet	Adipocyte
SAT_26M	1926.1	1075	0.0165117	0	Singlet	Adipocyte
SAT_26M	2014.7	1186	0.1136689	0	Singlet	Adipocyte
SAT_26M	1298.9	865	2.5467238	0	Singlet	Adipocyte
SAT_26M	1467.9	956	0.2286463	0	Singlet	Adipocyte
SAT_26M	1042.3	786	0.4954064	5	Singlet	Immune_cells
SAT_26M	2995.5	1579	6.5154277	0	Singlet	Adipocyte
SAT_26M	6620.8	2790	0.0052603	0	Singlet	Adipocyte
SAT_26M	4678.4	2198	0.0468588	0	Singlet	Adipocyte
SAT_26M	1199.1	804	0.0938784	0	Singlet	Adipocyte
SAT_26M	1295.1	814	5.4761505	6	Singlet	Neurons
SAT_26M	2175.6	1247	4.4769928	0	Singlet	Adipocyte
SAT_26M	1046.5	748	0.3662215	0	Singlet	Adipocyte
SAT_26M	4817.6	2380	1.1547503	0	Singlet	Adipocyte
SAT_26M	3283.4	1475	10.052778	0	Singlet	Adipocyte
SAT_26M	1790.9	1091	0.2877355	0	Singlet	Adipocyte
SAT_26M	1055.2	601	13.375671	7	Singlet	Endothelial_cells
SAT_26M	1099.1	672	4.7173884	0	Singlet	Adipocyte
SAT_26M	2673.5	1551	7.9538776	5	Singlet	Immune_cells
SAT_26M	1626.7	1084	2.1746814	0	Singlet	Adipocyte
SAT_26M	2566.5	1579	1.150974	8	Singlet	Mesothelial
SAT_26M	1670.7	1043	6.2408461	0	Singlet	Adipocyte
SAT_26M	4060.7	1876	4.1900626	0	Singlet	Adipocyte
SAT_26M	1186.8	849	0.3280232	7	Singlet	Endothelial_cells
SAT_26M	1165.6	802	0	0	Singlet	Adipocyte
SAT_26M	1984.7	1320	2.7187921	8	Singlet	Mesothelial
SAT_26M	3648.8	1984	0.4543064	0	Singlet	Adipocyte
SAT_26M	2145.7	1148	4.4204538	0	Singlet	Adipocyte
SAT_26M	1813.8	1133	0.1136893	0	Singlet	Adipocyte
SAT_26M	1188.5	809	0.0233429	0	Singlet	Adipocyte
SAT_26M	1899	1181	1.2697079	0	Singlet	Adipocyte
SAT_26M	1518.7	1041	0.0122494	11	Singlet	Smooth_muscle_cells
SAT_26M	2672.3	1347	9.9487018	7	Singlet	Endothelial_cells
SAT_26M	1943.5	1291	0.7247338	4	Singlet	Immune_cells
SAT_26M	2481.7	1391	11.706713	9	Singlet	Mesothelial
SAT_26M	1684.7	1030	4.3762675	0	Singlet	Adipocyte
SAT_26M	1061.5	748	1.5473943	1	Singlet	Immune_cells

SAT_26M	1272.5	927	0.2224209	11	Singlet	Smooth_muscle_cells
SAT_26M	1850.3	1056	3.1139053	0	Singlet	Adipocyte
SAT_26M	2147	1232	4.694463	0	Singlet	Adipocyte
SAT_26M	2555.6	1452	1.0748612	7	Singlet	Endothelial_cells
SAT_26M	1982	1178	2.5826105	0	Singlet	Adipocyte
SAT_26M	1890.3	1122	0.0196984	0	Singlet	Adipocyte
SAT_26M	1412.5	691	11.4973	0	Singlet	Adipocyte
SAT_26M	1422.5	930	13.053444	0	Singlet	Adipocyte
SAT_26M	1384.8	714	9.8084069	7	Singlet	Endothelial_cells
SAT_26M	1006	705	1.3387421	0	Singlet	Adipocyte
SAT_26M	1199.1	777	12.477861	1	Singlet	Immune_cells
SAT_26M	1901.1	1181	2.0855771	0	Singlet	Adipocyte
SAT_26M	4366.7	2214	2.1496658	0	Singlet	Adipocyte
SAT_26M	1337.9	858	0.0259607	2	Singlet	FAPs
SAT_26M	1012.8	650	1.3454508	9	Singlet	Mesothelial
SAT_26M	1103.1	769	0.5857234	0	Singlet	Adipocyte
SAT_26M	1254.8	549	8.3105149	0	Singlet	Adipocyte
SAT_26M	4993.5	2420	3.8013697	0	Singlet	Adipocyte
SAT_26M	1185.9	808	0.4694046	0	Singlet	Adipocyte
SAT_26M	1564.9	1019	0	0	Singlet	Adipocyte
SAT_26M	2220.4	1265	2.2802552	0	Singlet	Adipocyte
SAT_26M	1063	763	0.1663295	0	Singlet	Adipocyte
SAT_26M	1030.2	650	0.1245773	0	Singlet	Adipocyte
SAT_26M	1444.4	933	0.3794914	0	Singlet	Adipocyte
SAT_26M	2378.8	1396	0.1499808	0	Singlet	Adipocyte
SAT_26M	1037.2	702	1.1321028	7	Singlet	Endothelial_cells
SAT_26M	1669.6	1027	3.0108018	0	Singlet	Adipocyte
SAT_26M	3550.3	2031	0	8	Singlet	Mesothelial
SAT_26M	1151.8	818	1.6027955	8	Singlet	Mesothelial
SAT_26M	1853.8	1127	0.2666787	0	Singlet	Adipocyte
SAT_26M	1813.7	1121	1.3712661	0	Singlet	Adipocyte
SAT_26M	1105.7	785	1.1211687	0	Singlet	Adipocyte
SAT_26M	1239.9	861	1.9272735	1	Singlet	Immune_cells
SAT_26M	1334.7	661	8.2852573	0	Singlet	Adipocyte
SAT_26M	1314.5	840	0.5788433	6	Singlet	Neurons
SAT_26M	2751.7	1480	7.0987816	7	Singlet	Endothelial_cells
SAT_26M	1434.5	922	2.4966257	7	Singlet	Endothelial_cells
SAT_26M	1653.6	1091	3.408054	0	Singlet	Adipocyte
SAT_26M	3766.3	1984	0.1971024	0	Singlet	Adipocyte
SAT_26M	4395.9	2199	0.3915835	0	Singlet	Adipocyte
SAT_26M	2655.9	1440	1.9742751	0	Singlet	Adipocyte
SAT_26M	2279.2	1298	0.0397866	0	Singlet	Adipocyte
SAT_26M	4539.8	2375	3.0479969	8	Singlet	Mesothelial
SAT_26M	1344.3	940	2.4706515	0	Singlet	Adipocyte
SAT_26M	1735.1	1086	0.2820191	0	Singlet	Adipocyte
SAT_26M	2579.2	1487	0.2108666	0	Singlet	Adipocyte
SAT_26M	1664.6	919	4.1700737	10	Singlet	Immune_cells
SAT_26M	4622.7	2369	9.833021	7	Singlet	Endothelial_cells
SAT_26M	1522.1	1005	0.5737608	0	Singlet	Adipocyte
SAT_26M	1193.6	817	0.9891878	0	Singlet	Adipocyte
SAT_26M	1767.6	1212	0.7558281	5	Singlet	Immune_cells

SAT_26M	1614.7	952	0.4060151	0	Singlet	Adipocyte
SAT_26M	1808.5	976	13.848158	7	Singlet	Endothelial_cells
SAT_26M	2889.6	1460	3.723614	0	Singlet	Adipocyte
SAT_26M	1336.3	935	0.0036143	0	Singlet	Adipocyte
SAT_26M	1022.2	827	0.7397384	1	Singlet	Immune_cells
SAT_26M	3922.6	2002	6.4072989	7	Singlet	Endothelial_cells
SAT_26M	1517.7	903	11.225958	0	Singlet	Adipocyte
SAT_26M	2115.3	1360	10.617861	5	Singlet	Immune_cells
SAT_26M	2024.7	1154	4.5412111	0	Singlet	Adipocyte
SAT_26M	1238.2	948	0.096236	0	Singlet	Adipocyte
SAT_26M	1275.9	849	2.8337763	0	Singlet	Adipocyte
SAT_26M	1576.3	954	6.7491757	7	Singlet	Endothelial_cells
SAT_26M	8521.6	3051	8.5658143	7	Singlet	Endothelial_cells
SAT_26M	1474.1	928	0.116836	0	Singlet	Adipocyte
SAT_26M	1002.1	724	0.430799	1	Singlet	Immune_cells
SAT_26M	2130.2	1350	0	8	Singlet	Mesothelial
SAT_26M	1893.8	1110	4.5145108	0	Singlet	Adipocyte
SAT_26M	2077.4	1263	0.0576022	0	Singlet	Adipocyte
SAT_26M	2389.3	1412	2.3899672	0	Singlet	Adipocyte
SAT_26M	4324	2267	0.4961739	0	Singlet	Adipocyte
SAT_26M	2881.4	1795	5.3052942	7	Singlet	Endothelial_cells
SAT_26M	1258.8	837	5.8662382	0	Singlet	Adipocyte
SAT_26M	1481.3	996	0.7216092	8	Singlet	Mesothelial
SAT_26M	2363.3	1400	0.3760877	0	Singlet	Adipocyte
SAT_26M	1160.2	824	0.5989864	0	Singlet	Adipocyte
SAT_26M	1311	835	0.4147028	0	Singlet	Adipocyte
SAT_26M	1962.2	1183	3.2812251	7	Singlet	Endothelial_cells
SAT_26M	1766.5	1127	0.083904	0	Singlet	Adipocyte
SAT_26M	1366.2	875	0.0430056	0	Singlet	Adipocyte
SAT_26M	1795.7	1194	3.6849807	0	Singlet	Adipocyte
SAT_26M	3056.7	1755	7.8309915	5	Singlet	Immune_cells
SAT_26M	3446.5	1928	0	0	Singlet	Adipocyte
SAT_26M	1067.4	738	0.2873644	0	Singlet	Adipocyte
SAT_26M	1162.3	766	0.9277407	7	Singlet	Endothelial_cells
SAT_26M	1281	757	4.7938167	0	Singlet	Adipocyte
SAT_26M	1016.5	706	0.998828	0	Singlet	Adipocyte
SAT_26M	1410	973	0.6979507	0	Singlet	Adipocyte
SAT_26M	3540.4	1863	1.8797243	0	Singlet	Adipocyte
SAT_26M	1059.7	698	4.036384	0	Singlet	Adipocyte
SAT_26M	1325.8	907	0.5328118	0	Singlet	Adipocyte
SAT_26M	1378.7	839	7.568975	7	Singlet	Endothelial_cells
SAT_26M	3051.1	1547	4.5528141	0	Singlet	Adipocyte
SAT_26M	2736.3	1524	2.086694	0	Singlet	Adipocyte
SAT_26M	1085.9	688	0.7610994	0	Singlet	Adipocyte
SAT_26M	2241.1	1353	0	8	Singlet	Mesothelial
SAT_26M	1634.3	1046	2.7202351	0	Singlet	Adipocyte
SAT_26M	3926.5	2104	14.55943	9	Singlet	Mesothelial
SAT_26M	1036.6	646	0.1586555	10	Singlet	Immune_cells
SAT_26M	1267.2	834	12.089488	9	Singlet	Mesothelial
SAT_26M	1395.2	923	0.1775984	0	Singlet	Adipocyte
SAT_26M	1225.3	772	7.8920872	3	Singlet	FAPs

SAT_26M	1950.7	1176	0	0	Singlet	Adipocyte
SAT_26M	4759.3	2163	0.7638725	10	Singlet	Immune_cells
SAT_26M	1667.2	1045	0.7911457	0	Singlet	Adipocyte
SAT_26M	1064.9	536	12.735285	0	Singlet	Adipocyte
SAT_26M	1039.2	744	0.1070248	0	Singlet	Adipocyte
SAT_26M	1637.7	1133	1.7804078	1	Singlet	Immune_cells
SAT_26M	1759.1	1061	0.4622677	0	Singlet	Adipocyte
SAT_26M	1452.2	901	1.6359399	0	Singlet	Adipocyte
SAT_26M	1651.8	1065	0.1417153	2	Singlet	FAPs
SAT_26M	4005.3	1920	7.1657943	0	Singlet	Adipocyte
SAT_26M	3130	1673	0.2478281	0	Singlet	Adipocyte
SAT_26M	2264.3	1387	1.0576755	3	Singlet	FAPs
SAT_26M	1607.2	961	5.739474	0	Singlet	Adipocyte
SAT_26M	4324.5	2313	2.4496389	9	Singlet	Mesothelial
SAT_26M	2614.7	1224	5.5619213	10	Singlet	Immune_cells
SAT_26M	5932.7	2721	0.0054859	0	Singlet	Adipocyte
SAT_26M	1675.8	1024	5.9073261	0	Singlet	Adipocyte
SAT_26M	2071.2	1256	0.1866414	0	Singlet	Adipocyte
SAT_26M	2363.7	1325	2.7034985	0	Singlet	Adipocyte
SAT_26M	2325.8	1195	7.5922934	7	Singlet	Endothelial_cells
SAT_26M	1466.9	909	0.0625166	0	Singlet	Adipocyte
SAT_26M	1596.1	1116	0.008501	0	Singlet	Adipocyte
SAT_26M	2394	1465	0	8	Singlet	Mesothelial
SAT_26M	1330.8	848	1.0280559	0	Singlet	Adipocyte
SAT_26M	3039.8	1633	0	0	Singlet	Adipocyte
SAT_26M	4410.8	2072	11.314324	9	Singlet	Mesothelial
SAT_26M	1258.4	732	6.4273706	7	Singlet	Endothelial_cells
SAT_26M	1109.3	656	11.738057	8	Singlet	Mesothelial
SAT_26M	1680.6	1014	2.333079	5	Singlet	Immune_cells
SAT_26M	3767	1911	4.7204092	0	Singlet	Adipocyte
SAT_26M	6918.3	3509	3.6243476	9	Singlet	Mesothelial
SAT_26M	1360.1	885	0.2180388	0	Singlet	Adipocyte
SAT_26M	1383.2	963	2.4911733	1	Singlet	Immune_cells
SAT_26M	1113.2	776	0.462858	0	Singlet	Adipocyte
SAT_26M	1242.1	773	3.2541246	0	Singlet	Adipocyte
SAT_26M	2524.9	1533	0.3303677	0	Singlet	Adipocyte
SAT_26M	1254.6	874	0.7001169	5	Singlet	Immune_cells
SAT_26M	10690	4436	6.6144225	8	Singlet	Mesothelial
SAT_26M	14365	5309	3.0617765	9	Singlet	Mesothelial
SAT_26M	1945.3	1210	1.7266149	4	Singlet	Immune_cells
SAT_26M	1028.4	731	0	0	Singlet	Adipocyte
SAT_26M	1603.7	1036	0.0142375	0	Singlet	Adipocyte
SAT_26M	3066.7	1472	1.4077404	10	Singlet	Immune_cells
SAT_26M	2142.8	1176	2.694207	0	Singlet	Adipocyte
SAT_26M	1113.8	778	0.534656	1	Singlet	Immune_cells
SAT_26M	1119.5	715	10.396033	9	Singlet	Mesothelial
SAT_26M	1242.1	843	3.320219	1	Singlet	Immune_cells
SAT_26M	1169.9	762	7.3104796	6	Singlet	Neurons
SAT_26M	1907.7	1069	11.203001	5	Singlet	Immune_cells
SAT_26M	4170.1	2176	1.2962701	8	Singlet	Mesothelial
SAT_26M	2892.6	1659	0.9127243	0	Singlet	Adipocyte

SAT_26M	1012.5	718	0.2143074	0	Singlet	Adipocyte
SAT_26M	1157.3	796	0.7363235	1	Singlet	Immune_cells
SAT_26M	1316.8	788	2.9452235	7	Singlet	Endothelial_cells
SAT_26M	3836.3	2269	1.5267374	8	Singlet	Mesothelial
SAT_26M	1024.9	615	0.2960973	0	Singlet	Adipocyte
SAT_26M	1518.6	936	1.6016948	9	Singlet	Mesothelial
SAT_26M	1616.4	926	0.6572832	10	Singlet	Immune_cells
SAT_26M	2639.5	1457	2.0992668	0	Singlet	Adipocyte
SAT_26M	1105.3	722	1.7791829	3	Singlet	FAPs
SAT_26M	1122.8	719	0.134581	0	Singlet	Adipocyte
SAT_26M	3971.3	1893	0.6475486	0	Singlet	Adipocyte
SAT_26M	2313.9	1308	5.0704353	7	Singlet	Endothelial_cells
SAT_26M	1092.9	707	2.1610849	0	Singlet	Adipocyte
SAT_26M	1650.3	1083	3.7691276	0	Singlet	Adipocyte
SAT_26M	1359.9	885	0.1758735	0	Singlet	Adipocyte
SAT_26M	1432.7	952	0.4410218	0	Singlet	Adipocyte
SAT_26M	1150.9	774	0.0975588	0	Singlet	Adipocyte
SAT_26M	1620.1	1046	0	6	Singlet	Neurons
SAT_26M	1449.5	947	2.9748272	0	Singlet	Adipocyte
SAT_26M	1872	929	0.4577275	5	Singlet	Immune_cells
SAT_26M	2748.7	1504	1.5635755	3	Singlet	FAPs
SAT_26M	1027.9	788	1.4661646	1	Singlet	Immune_cells
SAT_26M	1017.2	564	11.952962	7	Singlet	Endothelial_cells
SAT_26M	1222.4	811	4.3623058	3	Singlet	FAPs
SAT_26M	5231.9	2199	1.6344738	0	Singlet	Adipocyte
SAT_26M	1821.3	1126	0.039582	0	Singlet	Adipocyte
SAT_26M	1364	1004	0.8624862	11	Singlet	Smooth_muscle_cells
SAT_26M	1862.1	1013	14.228835	7	Singlet	Endothelial_cells
SAT_26M	2394.8	1122	11.63367	7	Singlet	Endothelial_cells
SAT_26M	1148.1	734	0	8	Singlet	Mesothelial
SAT_26M	3507.4	2039	13.81995	9	Singlet	Mesothelial
SAT_26M	1878.6	1041	8.96174	0	Singlet	Adipocyte
SAT_26M	2611.2	1639	0.9150111	11	Singlet	Smooth_muscle_cells
SAT_26M	1181.2	750	1.464323	7	Singlet	Endothelial_cells
SAT_26M	1469	1109	0.0681033	11	Singlet	Smooth_muscle_cells
SAT_26M	1463.8	972	1.6012875	3	Singlet	FAPs
SAT_26M	1014.7	651	0.9339055	0	Singlet	Adipocyte
SAT_26M	7475.6	2959	8.8826192	7	Singlet	Endothelial_cells
SAT_26M	1469.8	962	7.337741	1	Singlet	Immune_cells
SAT_26M	1544.2	1031	1.1502005	0	Singlet	Adipocyte
SAT_26M	2202.1	1270	4.2050971	0	Singlet	Adipocyte
SAT_26M	1002	671	10.278692	4	Singlet	Immune_cells
SAT_26M	1541.3	961	2.9216473	2	Singlet	FAPs
SAT_26M	1806.4	1213	0.3956781	0	Singlet	Adipocyte
SAT_26M	1972.3	1205	4.0434254	0	Singlet	Adipocyte
SAT_26M	1050.2	571	8.5687928	10	Singlet	Immune_cells
SAT_26M	1888.8	1069	0.5632337	0	Singlet	Adipocyte
SAT_26M	1316.9	922	0.3767252	3	Singlet	FAPs
SAT_26M	1905.6	1303	5.3787778	1	Singlet	Immune_cells
SAT_26M	1762.6	1053	0.7024612	2	Singlet	FAPs
SAT_26M	1259.8	809	0.0098089	0	Singlet	Adipocyte

SAT_26M	2120.5	1214	2.7755378	0	Singlet	Adipocyte
SAT_26M	1012.3	746	0.2540491	0	Singlet	Adipocyte
SAT_26M	1047.1	672	1.4335396	3	Singlet	FAPs
SAT_26M	1456.9	955	0	8	Singlet	Mesothelial
SAT_26M	8278.8	3200	1.1477169	0	Singlet	Adipocyte
SAT_26M	1731.9	1083	1.8517149	0	Singlet	Adipocyte
SAT_26M	1358.3	928	1.2227059	6	Singlet	Neurons
SAT_26M	2481.7	1448	0.0151274	0	Singlet	Adipocyte
SAT_26M	1179.8	803	13.218691	1	Singlet	Immune_cells
SAT_26M	1400.5	806	4.6907519	3	Singlet	FAPs
SAT_26M	2455.7	1334	1.9043354	0	Singlet	Adipocyte
SAT_26M	2613.2	1689	10.21904	11	Singlet	Smooth_muscle_cells
SAT_26M	1048.8	717	2.2159587	5	Singlet	Immune_cells
SAT_26M	1715.9	1044	0.608892	5	Singlet	Immune_cells
SAT_26M	1346.1	912	0	0	Singlet	Adipocyte
SAT_26M	1725.9	1063	3.6542442	0	Singlet	Adipocyte
SAT_26M	1692.8	905	2.6565656	5	Singlet	Immune_cells
SAT_26M	1394.3	867	0.0968762	0	Singlet	Adipocyte
SAT_26M	1326.7	833	3.8551124	3	Singlet	FAPs
SAT_26M	2251.7	1312	1.4498648	8	Singlet	Mesothelial
SAT_26M	3076.3	1627	0.2400281	0	Singlet	Adipocyte
SAT_26M	5060.3	2116	1.3477186	10	Singlet	Immune_cells
SAT_26M	1065.6	677	13.359188	10	Singlet	Immune_cells
SAT_26M	3098.3	1578	7.994316	8	Singlet	Mesothelial
SAT_26M	3165.2	1501	2.7219377	10	Singlet	Immune_cells
SAT_26M	2494.2	1379	11.982238	0	Singlet	Adipocyte
SAT_26M	1408.5	849	0.7988958	0	Singlet	Adipocyte
SAT_26M	1382	858	6.4853968	7	Singlet	Endothelial_cells
SAT_26M	1964.5	1190	0.1193218	0	Singlet	Adipocyte
SAT_26M	1277.8	853	0.2677725	8	Singlet	Mesothelial
SAT_26M	3949.6	1997	0	0	Singlet	Adipocyte
SAT_26M	1102.6	770	7.5824426	1	Singlet	Immune_cells
SAT_26M	1623.8	1076	0.6917255	0	Singlet	Adipocyte
SAT_26M	1097.8	773	6.4648615	1	Singlet	Immune_cells
SAT_26M	1373.1	934	1.6958793	6	Singlet	Neurons
SAT_26M	1233.7	795	2.8410806	7	Singlet	Endothelial_cells
SAT_26M	1767.2	1257	0.3352531	0	Singlet	Adipocyte
SAT_26M	1309.4	855	10.102901	0	Singlet	Adipocyte
SAT_26M	1222.1	872	1.7130271	1	Singlet	Immune_cells
SAT_26M	2123.6	1229	2.719838	0	Singlet	Adipocyte
SAT_26M	1294.1	821	0.0430358	0	Singlet	Adipocyte
SAT_26M	3087.9	1918	0	8	Singlet	Mesothelial
SAT_26M	1811.4	1092	2.2423486	0	Singlet	Adipocyte
SAT_26M	1029.7	595	8.2963196	7	Singlet	Endothelial_cells
SAT_26M	1424.9	919	0.035817	0	Singlet	Adipocyte
SAT_26M	1353.7	823	11.367942	7	Singlet	Endothelial_cells
SAT_26M	1499.3	1056	0.883902	1	Singlet	Immune_cells
SAT_26M	1628.1	941	0.4698046	0	Singlet	Adipocyte
SAT_26M	1286.7	830	2.3746978	7	Singlet	Endothelial_cells
SAT_26M	2470.3	1345	12.012049	0	Singlet	Adipocyte
SAT_26M	2067.1	1254	6.0780475	0	Singlet	Adipocyte

SAT_26M	1410.2	1089	2.6817603	1	Singlet	Immune_cells
SAT_26M	1077.4	783	1.2489964	1	Singlet	Immune_cells
SAT_26M	1479.7	925	3.020656	0	Singlet	Adipocyte
SAT_26M	1840.6	980	10.331996	7	Singlet	Endothelial_cells
SAT_26M	1426.9	935	0.0073286	0	Singlet	Adipocyte
SAT_26M	1088.4	740	0.7793686	8	Singlet	Mesothelial
SAT_26M	1236.8	840	3.2632529	7	Singlet	Endothelial_cells
SAT_26M	2428.2	1319	2.2509637	0	Singlet	Adipocyte
SAT_26M	3218.4	1431	5.5795607	10	Singlet	Immune_cells
SAT_26M	1043.3	746	8.7846648	1	Singlet	Immune_cells
SAT_26M	1378.7	1042	1.0486164	9	Singlet	Mesothelial
SAT_26M	1784.3	1142	0.1394094	8	Singlet	Mesothelial
SAT_26M	3492.8	1795	0	0	Singlet	Adipocyte
SAT_26M	5129.6	2344	0.0335416	0	Singlet	Adipocyte
SAT_26M	1261.7	850	0.3248805	0	Singlet	Adipocyte
SAT_26M	1655.8	986	0.2242045	0	Singlet	Adipocyte
SAT_26M	2186.8	1121	5.0322487	10	Singlet	Immune_cells
SAT_26M	1253.6	831	0.7752755	0	Singlet	Adipocyte
SAT_26M	1478.7	901	11.479258	9	Singlet	Mesothelial
SAT_26M	1665.1	1235	5.948899	1	Singlet	Immune_cells
SAT_26M	1692.6	1123	0	0	Singlet	Adipocyte
SAT_26M	1048.9	712	1.1776307	0	Singlet	Adipocyte
SAT_26M	2006.6	1283	1.5274071	7	Singlet	Endothelial_cells
SAT_26M	1410.9	938	1.1757452	0	Singlet	Adipocyte
SAT_26M	1367.4	881	0.4488791	0	Singlet	Adipocyte
SAT_26M	1611.6	927	2.3842677	0	Singlet	Adipocyte
SAT_26M	1021.8	652	1.435293	3	Singlet	FAPs
SAT_26M	1637	858	13.630224	5	Singlet	Immune_cells
SAT_26M	4615	2103	1.6895158	10	Singlet	Immune_cells
SAT_26M	2529.4	1349	0.9298918	0	Singlet	Adipocyte
SAT_26M	1958	1114	0	2	Singlet	FAPs
SAT_26M	1240	695	9.3090679	0	Singlet	Adipocyte
SAT_26M	2759.6	1631	1.271956	0	Singlet	Adipocyte
SAT_26M	1157.3	801	0.7119501	3	Singlet	FAPs
SAT_26M	1162.4	750	5.1617551	8	Singlet	Mesothelial
SAT_26M	2204.6	1150	7.9119853	9	Singlet	Mesothelial
SAT_26M	1581.4	1110	0.1030117	0	Singlet	Adipocyte
SAT_26M	2706.4	1370	0	8	Singlet	Mesothelial
SAT_26M	1783.4	1087	0.5489298	7	Singlet	Endothelial_cells
SAT_26M	1822.1	1159	2.4939089	8	Singlet	Mesothelial
SAT_26M	2997.8	1518	0.6357525	10	Singlet	Immune_cells
SAT_26M	1000.8	748	1.0378532	1	Singlet	Immune_cells
SAT_26M	1903.1	1322	0.4631753	5	Singlet	Immune_cells
SAT_26M	2107.8	1189	0.8909562	0	Singlet	Adipocyte
SAT_26M	1105.3	716	0.8058085	0	Singlet	Adipocyte
SAT_26M	2991.9	1539	6.3817103	0	Singlet	Adipocyte
SAT_26M	1074.9	718	0	3	Singlet	FAPs
SAT_26M	2245.7	1347	13.897556	7	Singlet	Endothelial_cells
SAT_26M	2353.1	1301	7.5721182	0	Singlet	Adipocyte
SAT_26M	1704.4	1094	0.4812598	5	Singlet	Immune_cells
SAT_26M	1197.9	858	1.6506315	1	Singlet	Immune_cells

SAT_26M	4163.5	2005	0.0909791	0	Singlet	Adipocyte
SAT_26M	1734.2	944	0	6	Singlet	Neurons
SAT_26M	4930.9	2686	3.4432942	9	Singlet	Mesothelial
SAT_26M	1688.4	971	4.4836051	0	Singlet	Adipocyte
SAT_26M	1054.4	823	0.1287393	8	Singlet	Mesothelial
SAT_26M	5823.9	2911	5.653042	9	Singlet	Mesothelial
SAT_26M	2855.1	1615	0.5177871	3	Singlet	FAPs
SAT_26M	2032.5	1339	0	8	Singlet	Mesothelial
SAT_26M	1323.2	822	0.7111884	0	Singlet	Adipocyte
SAT_26M	2865.4	1521	11.21048	7	Singlet	Endothelial_cells
SAT_26M	1295.7	893	0.644887	0	Singlet	Adipocyte
SAT_26M	2011.7	1274	1.9381398	5	Singlet	Immune_cells
SAT_26M	1171.8	678	6.2186726	7	Singlet	Endothelial_cells
SAT_26M	1725.3	1081	11.62438	5	Singlet	Immune_cells
SAT_26M	1725.9	1216	1.9470131	0	Singlet	Adipocyte
SAT_26M	3597.4	1598	0.8005143	10	Singlet	Immune_cells
SAT_26M	2641.7	1466	1.4215468	0	Singlet	Adipocyte
SAT_26M	1139.5	759	6.5374544	0	Singlet	Adipocyte
SAT_26M	2511.6	1502	1.0622918	0	Singlet	Adipocyte
SAT_26M	3325.8	1377	14.919002	0	Singlet	Adipocyte
SAT_26M	1158.7	805	0.6053356	0	Singlet	Adipocyte
SAT_26M	5017.1	2221	0	0	Singlet	Adipocyte
SAT_26M	1477.1	1015	4.831408	0	Singlet	Adipocyte
SAT_26M	2414.4	1415	0.0096446	0	Singlet	Adipocyte
SAT_26M	1246.4	815	8.4730459	0	Singlet	Adipocyte
SAT_26M	2257.2	1104	10.713669	0	Singlet	Adipocyte
SAT_26M	4213.9	2067	1.5827569	0	Singlet	Adipocyte
SAT_26M	2005.4	1242	2.2438879	0	Singlet	Adipocyte
SAT_26M	1664.6	978	4.8832246	0	Singlet	Adipocyte
SAT_26M	1110.8	749	5.0260938	0	Singlet	Adipocyte
SAT_26M	1517.6	974	1.4673923	7	Singlet	Endothelial_cells
SAT_26M	1488.1	1003	1.586894	0	Singlet	Adipocyte
SAT_26M	1547.1	1026	1.293553	0	Singlet	Adipocyte
SAT_26M	1262.3	581	11.793864	0	Singlet	Adipocyte
SAT_26M	3403.1	1767	0	0	Singlet	Adipocyte
SAT_26M	5475.8	2525	5.4720076	10	Singlet	Immune_cells
SAT_26M	1301.7	910	0.6791492	6	Singlet	Neurons
SAT_26M	1807.7	1091	5.8488926	0	Singlet	Adipocyte
SAT_26M	1056.9	739	2.6833248	7	Singlet	Endothelial_cells
SAT_26M	2003	1208	0.984762	3	Singlet	FAPs
SAT_26M	2838.7	1807	4.5780295	8	Singlet	Mesothelial
SAT_26M	3276.6	1804	2.7940456	3	Singlet	FAPs
SAT_26M	1831.1	1122	0.212645	0	Singlet	Adipocyte
SAT_26M	1541.2	865	7.6566673	0	Singlet	Adipocyte
SAT_26M	1251.3	887	5.4463941	4	Singlet	Immune_cells
SAT_26M	1091.6	800	0.5652229	6	Singlet	Neurons
SAT_26M	2131.3	1121	11.972791	0	Singlet	Adipocyte
SAT_26M	1249	858	0.9793597	3	Singlet	FAPs
SAT_26M	1170.7	779	0.2057216	0	Singlet	Adipocyte
SAT_26M	1166.4	830	3.2134613	4	Singlet	Immune_cells
SAT_26M	2726.4	1522	0.7746978	0	Singlet	Adipocyte

SAT_26M	1212.4	794	1.0229555	0	Singlet	Adipocyte
SAT_26M	1396.2	634	10.324141	0	Singlet	Adipocyte
SAT_26M	1082.8	581	4.7431183	5	Singlet	Immune_cells
SAT_26M	1122.8	684	4.0390207	10	Singlet	Immune_cells
SAT_26M	1655.2	948	9.504552	0	Singlet	Adipocyte
SAT_26M	1056.6	623	6.2824469	7	Singlet	Endothelial_cells
SAT_26M	2870.5	1543	2.8784252	10	Singlet	Immune_cells
SAT_26M	2821.4	1355	12.332158	0	Singlet	Adipocyte
SAT_26M	2149.1	1180	1.6691996	0	Singlet	Adipocyte
SAT_26M	1373.1	907	0.6701185	1	Singlet	Immune_cells
SAT_26M	1287.2	945	3.1266035	3	Singlet	FAPs
SAT_26M	1181.6	751	0.5313846	0	Singlet	Adipocyte
SAT_26M	1677	1068	3.4519778	8	Singlet	Mesothelial
SAT_26M	2743.6	1388	12.293486	7	Singlet	Endothelial_cells
SAT_26M	1504.7	923	1.3365249	0	Singlet	Adipocyte
SAT_26M	1596.4	884	11.422192	0	Singlet	Adipocyte
SAT_26M	1123.3	767	0	0	Singlet	Adipocyte
SAT_26M	1346.5	736	13.957219	7	Singlet	Endothelial_cells
SAT_26M	1167.7	727	10.957142	6	Singlet	Neurons
SAT_26M	1782.3	1042	6.6715759	0	Singlet	Adipocyte
SAT_26M	4415.9	2328	0.8058079	0	Singlet	Adipocyte
SAT_26M	1473.2	871	10.50299	7	Singlet	Endothelial_cells
SAT_26M	1012.7	724	0.3203151	6	Singlet	Neurons
SAT_26M	4577.8	2152	0.1057027	0	Singlet	Adipocyte
SAT_26M	2997.6	1413	9.7295154	7	Singlet	Endothelial_cells
SAT_26M	1144.8	723	0.8573282	0	Singlet	Adipocyte
SAT_26M	2250.6	1410	0	9	Singlet	Mesothelial
SAT_26M	2182.4	1285	2.8274867	0	Singlet	Adipocyte
SAT_26M	1742.3	1083	10.47131	4	Singlet	Immune_cells
SAT_26M	1007.4	717	0.8267945	3	Singlet	FAPs
SAT_26M	2695.6	1726	2.6128672	5	Singlet	Immune_cells
SAT_26M	5311	2370	0.8149544	0	Singlet	Adipocyte
SAT_26M	2195.6	1385	1.7888419	9	Singlet	Mesothelial
SAT_26M	1227	865	4.6575895	1	Singlet	Immune_cells
SAT_26M	2063.4	1297	3.6030862	8	Singlet	Mesothelial
SAT_26M	1768.3	1092	10.543592	9	Singlet	Mesothelial
SAT_26M	1255.6	901	3.0655652	11	Singlet	Smooth_muscle_cells
SAT_26M	1042.7	782	2.6302773	1	Singlet	Immune_cells
SAT_26M	1853.5	1164	0.1139783	0	Singlet	Adipocyte
SAT_26M	1714.1	1037	8.636656	0	Singlet	Adipocyte
SAT_26M	6196	3118	1.8955151	9	Singlet	Mesothelial
SAT_26M	2676.1	1489	0.075826	0	Singlet	Adipocyte
SAT_26M	3216.6	1665	0.837703	0	Singlet	Adipocyte
SAT_26M	1630.9	1040	5.3147788	9	Singlet	Mesothelial
SAT_26M	1301.5	923	0	5	Singlet	Immune_cells
SAT_26M	1773.5	1068	1.625778	7	Singlet	Endothelial_cells
SAT_26M	1359.3	675	13.615934	0	Singlet	Adipocyte
SAT_26M	1182.2	816	1.3705993	0	Singlet	Adipocyte
SAT_26M	1323.2	899	0.0575652	0	Singlet	Adipocyte
SAT_26M	1021.1	686	2.7995403	7	Singlet	Endothelial_cells
SAT_26M	3198.9	1799	0.6951703	0	Singlet	Adipocyte

SAT_26M	1143.2	791	2.808618	5	Singlet	Immune_cells
SAT_26M	1971	1140	6.3170268	7	Singlet	Endothelial_cells
SAT_26M	2243.1	1527	2.2140945	8	Singlet	Mesothelial
SAT_26M	1700.5	1041	6.0626169	0	Singlet	Adipocyte
SAT_26M	1520.9	983	2.438312	0	Singlet	Adipocyte
SAT_26M	2701	1556	3.6257278	7	Singlet	Endothelial_cells
SAT_26M	1634.7	1024	0	0	Singlet	Adipocyte
SAT_26M	1328.2	880	2.9616889	0	Singlet	Adipocyte
SAT_26M	1472.2	979	3.2331825	8	Singlet	Mesothelial
SAT_26M	2274	1469	0	5	Singlet	Immune_cells
SAT_26M	1407.5	928	7.6168564	1	Singlet	Immune_cells
SAT_26M	1991.7	1257	0.5994396	0	Singlet	Adipocyte
SAT_26M	1598.5	1151	0	11	Singlet	Smooth_muscle_cells
SAT_26M	2384.7	1265	1.8496361	0	Singlet	Adipocyte
SAT_26M	1475.7	989	0.7492715	7	Singlet	Endothelial_cells
SAT_26M	1680.3	991	6.1934168	0	Singlet	Adipocyte
SAT_26M	2735.9	1552	9.5618898	9	Singlet	Mesothelial
SAT_26M	1309	775	1.3972817	0	Singlet	Adipocyte
SAT_26M	1793.3	1204	0.7768102	9	Singlet	Mesothelial
SAT_26M	1272.6	825	1.1131223	3	Singlet	FAPs
SAT_26M	1391.4	930	0	8	Singlet	Mesothelial
SAT_26M	1580.9	959	6.694033	7	Singlet	Endothelial_cells
SAT_26M	1238.5	865	0.7225	0	Singlet	Adipocyte
SAT_26M	1091	660	11.429211	0	Singlet	Adipocyte
SAT_26M	1906.9	1124	2.1132356	0	Singlet	Adipocyte
SAT_26M	1554.7	1116	0.351544	11	Singlet	Smooth_muscle_cells
SAT_26M	3333.1	1782	1.3065221	0	Singlet	Adipocyte
SAT_26M	2390.2	1331	8.2875243	0	Singlet	Adipocyte
SAT_26M	2830	1598	0.7370736	0	Singlet	Adipocyte
SAT_26M	1064.4	775	0.8122286	6	Singlet	Neurons
SAT_26M	2419.5	1356	0.9486469	0	Singlet	Adipocyte
SAT_26M	4478	2235	0.557791	0	Singlet	Adipocyte
SAT_26M	2195	1271	0.2186929	0	Singlet	Adipocyte
SAT_26M	1977.8	1274	0.1321274	0	Singlet	Adipocyte
SAT_26M	1089.4	774	0.0281369	3	Singlet	FAPs
SAT_26M	1208.3	779	0.0426211	0	Singlet	Adipocyte
SAT_26M	1212.2	820	0	5	Singlet	Immune_cells
SAT_26M	1013.7	785	1.861928	8	Singlet	Mesothelial
SAT_26M	2285.2	1322	4.6941494	0	Singlet	Adipocyte
SAT_26M	1627	913	4.3880402	7	Singlet	Endothelial_cells
SAT_26M	1752.9	1098	1.4967329	11	Singlet	Smooth_muscle_cells
SAT_26M	1432.1	936	0.3148267	7	Singlet	Endothelial_cells
SAT_26M	5571.6	2299	0.0389497	0	Singlet	Adipocyte
SAT_26M	1958.5	1174	3.6465248	0	Singlet	Adipocyte
SAT_26M	3187.7	1625	0.0112998	0	Singlet	Adipocyte
SAT_26M	11846	4798	5.4749559	9	Singlet	Mesothelial
SAT_26M	2750.9	1427	1.1367266	0	Singlet	Adipocyte
SAT_26M	1654	955	1.1087313	0	Singlet	Adipocyte
SAT_26M	2643.5	1468	2.5317609	10	Singlet	Immune_cells
SAT_26M	1305.7	860	0.1178163	0	Singlet	Adipocyte
SAT_26M	1025.1	658	5.2563559	5	Singlet	Immune_cells

SAT_26M	2801.6	1572	2.7392016	0	Singlet	Adipocyte
SAT_26M	3020.9	1758	3.0111414	7	Singlet	Endothelial_cells
SAT_26M	1318.5	809	14.982224	1	Singlet	Immune_cells
SAT_26M	1333.9	967	0.905842	4	Singlet	Immune_cells
SAT_26M	3297.6	1706	6.2517684	7	Singlet	Endothelial_cells
SAT_26M	1131.6	745	5.0155547	0	Singlet	Adipocyte
SAT_26M	1550.6	982	5.4820926	7	Singlet	Endothelial_cells
SAT_26M	1228.9	836	0.1789077	0	Singlet	Adipocyte
SAT_26M	4930.8	2443	2.5987951	0	Singlet	Adipocyte
SAT_26M	1089.6	732	0.2674781	6	Singlet	Neurons
SAT_26M	1350.3	877	0.1642717	0	Singlet	Adipocyte
SAT_26M	1435.6	912	2.7103341	0	Singlet	Adipocyte
SAT_26M	1794.5	1199	3.532468	1	Singlet	Immune_cells
SAT_26M	1068.1	571	5.8364757	7	Singlet	Endothelial_cells
SAT_26M	3827	1957	0.5427323	0	Singlet	Adipocyte
SAT_26M	2697.3	1478	3.8525136	7	Singlet	Endothelial_cells
SAT_26M	1506	974	0.6870881	8	Singlet	Mesothelial
SAT_26M	2629.4	1443	3.2734041	0	Singlet	Adipocyte
SAT_26M	2685.2	1240	12.493107	0	Singlet	Adipocyte
SAT_26M	1043.7	645	6.5347906	0	Singlet	Adipocyte
SAT_26M	1388.1	988	2.0289851	0	Singlet	Adipocyte
SAT_26M	3576.4	1868	0	0	Singlet	Adipocyte
SAT_26M	1236.3	868	0.0748657	0	Singlet	Adipocyte
SAT_26M	1300.2	776	1.1763237	5	Singlet	Immune_cells
SAT_26M	1748.9	1059	9.9193087	7	Singlet	Endothelial_cells
SAT_26M	1413.3	1021	2.4522071	1	Singlet	Immune_cells
SAT_26M	3957.1	2288	1.2810263	8	Singlet	Mesothelial
SAT_26M	3326.5	1812	7.2691733	8	Singlet	Mesothelial
SAT_26M	1606.6	971	0.0976824	0	Singlet	Adipocyte
SAT_26M	1099.6	766	4.4885354	7	Singlet	Endothelial_cells
SAT_26M	1577.7	1077	1.9946511	7	Singlet	Endothelial_cells
SAT_26M	1033.5	638	1.2883345	10	Singlet	Immune_cells
SAT_26M	2086.3	1282	0.309258	5	Singlet	Immune_cells
SAT_26M	1245	858	5.3974066	10	Singlet	Immune_cells
SAT_26M	5572.4	2197	14.146221	7	Singlet	Endothelial_cells
SAT_26M	1015.4	687	1.4606561	0	Singlet	Adipocyte
SAT_26M	1832.1	1085	3.0966661	0	Singlet	Adipocyte
SAT_26M	1569.8	912	1.7682877	10	Singlet	Immune_cells
SAT_26M	1728	1090	0.2317999	0	Singlet	Adipocyte
SAT_26M	1135.6	758	9.2128268	0	Singlet	Adipocyte
SAT_26M	1297.5	746	10.956342	0	Singlet	Adipocyte
SAT_26M	1423	974	0.1523208	0	Singlet	Adipocyte
SAT_26M	1937.8	1343	3.6540447	1	Singlet	Immune_cells
SAT_26M	1433	955	5.7299611	8	Singlet	Mesothelial
SAT_26M	1506	1016	0.115382	0	Singlet	Adipocyte
SAT_26M	1158.9	742	0.581503	0	Singlet	Adipocyte
SAT_26M	2590	1628	2.78933	8	Singlet	Mesothelial
SAT_26M	4244.4	2076	0.1305093	0	Singlet	Adipocyte
SAT_26M	1164.2	795	0.7353022	3	Singlet	FAPs
SAT_26M	1592.2	965	0.8521207	0	Singlet	Adipocyte
SAT_26M	2031.6	1157	4.9090105	7	Singlet	Endothelial_cells

SAT_26M	1789.3	1211	0	5	Singlet	Immune_cells
SAT_26M	1392.1	898	7.3990138	0	Singlet	Adipocyte
SAT_26M	2237.9	1368	1.3840302	0	Singlet	Adipocyte
SAT_26M	1639.1	929	5.1229799	0	Singlet	Adipocyte
SAT_26M	1122	813	3.4006723	0	Singlet	Adipocyte
SAT_26M	1508.5	996	0	5	Singlet	Immune_cells
SAT_26M	1125.4	782	0.0142278	0	Singlet	Adipocyte
SAT_26M	2187.4	1290	4.7338539	0	Singlet	Adipocyte
SAT_26M	2395.2	1388	3.9223845	0	Singlet	Adipocyte
SAT_26M	1811.7	1067	6.8896408	0	Singlet	Adipocyte
SAT_26M	1166.7	826	0.0121666	5	Singlet	Immune_cells
SAT_26M	1638.4	1063	2.4010391	1	Singlet	Immune_cells
SAT_26M	1366	912	3.1455867	0	Singlet	Adipocyte
SAT_26M	3404.8	1663	8.4011505	7	Singlet	Endothelial_cells
SAT_26M	1357.3	873	0.4977561	0	Singlet	Adipocyte
SAT_26M	1160.8	749	0.5728485	0	Singlet	Adipocyte
SAT_26M	1084.1	738	2.3717224	8	Singlet	Mesothelial
SAT_26M	1238.3	839	0.6897734	0	Singlet	Adipocyte
SAT_26M	1611.9	922	7.8780794	10	Singlet	Immune_cells
SAT_26M	4127.5	1901	8.9109558	7	Singlet	Endothelial_cells
SAT_26M	1154.3	788	0.2524249	5	Singlet	Immune_cells
SAT_26M	1135.2	752	13.062573	1	Singlet	Immune_cells
SAT_26M	1091	791	0	5	Singlet	Immune_cells
SAT_26M	1508.9	1010	0.1714914	9	Singlet	Mesothelial
SAT_26M	2256.7	1235	2.4404206	7	Singlet	Endothelial_cells
SAT_26M	1751.1	1086	0.6199903	0	Singlet	Adipocyte
SAT_26M	4330.5	2274	0.7718085	0	Singlet	Adipocyte
SAT_26M	2090.5	1166	7.4828279	10	Singlet	Immune_cells
SAT_26M	1171.3	839	0.2685506	6	Singlet	Neurons
SAT_26M	2765.5	1429	4.4084846	0	Singlet	Adipocyte
SAT_26M	1973.2	1215	0.2352138	0	Singlet	Adipocyte
SAT_26M	1247.3	806	1.4824198	0	Singlet	Adipocyte
SAT_26M	1065.2	722	1.0162993	0	Singlet	Adipocyte
SAT_26M	1086.6	760	0.8504473	0	Singlet	Adipocyte
SAT_26M	2275.3	1512	0.9713554	1	Singlet	Immune_cells
SAT_26M	2355.1	1403	0.6211339	0	Singlet	Adipocyte
SAT_26M	2174.8	1226	1.5040117	0	Singlet	Adipocyte
SAT_26M	1390.8	708	13.433196	0	Singlet	Adipocyte
SAT_26M	1000.3	696	10.015534	5	Singlet	Immune_cells
SAT_26M	1098.6	702	4.140487	10	Singlet	Immune_cells
SAT_26M	2671.5	1484	1.3588984	10	Singlet	Immune_cells
SAT_26M	2600.2	1283	0.4566801	10	Singlet	Immune_cells
SAT_26M	1968.8	1140	0.0226729	0	Singlet	Adipocyte
SAT_26M	1314	841	5.6476964	0	Singlet	Adipocyte
SAT_26M	2625.4	1480	0.5116573	0	Singlet	Adipocyte
SAT_26M	1201.6	726	5.2558352	10	Singlet	Immune_cells
SAT_26M	1735.4	969	6.9223175	7	Singlet	Endothelial_cells
SAT_26M	1430.1	915	0.2776331	0	Singlet	Adipocyte
SAT_26M	2363.1	1304	13.471889	7	Singlet	Endothelial_cells
SAT_26M	2915.5	1536	9.85457	7	Singlet	Endothelial_cells
SAT_26M	2219.8	1386	0	5	Singlet	Immune_cells

SAT_26M	1383.4	908	0	0	Singlet	Adipocyte
SAT_26M	5140.5	2511	1.7251791	0	Singlet	Adipocyte
SAT_26M	1543	1004	0.191662	8	Singlet	Mesothelial
SAT_26M	1412.7	708	9.5799281	0	Singlet	Adipocyte
SAT_26M	3276	1757	1.8131743	0	Singlet	Adipocyte
SAT_26M	1117.7	708	9.5286745	5	Singlet	Immune_cells
SAT_26M	1236.4	806	0.7792463	0	Singlet	Adipocyte
SAT_26M	1187.2	839	4.994793	1	Singlet	Immune_cells
SAT_26M	1917.2	1155	3.0380141	0	Singlet	Adipocyte
SAT_26M	1136.1	619	5.3979687	5	Singlet	Immune_cells
SAT_26M	1798.2	1117	0.4989048	0	Singlet	Adipocyte
SAT_26M	2357.1	1382	2.091223	0	Singlet	Adipocyte
SAT_26M	1572.3	1094	1.5294588	1	Singlet	Immune_cells
SAT_26M	1964.4	1237	0.0204369	0	Singlet	Adipocyte
SAT_26M	1352.5	816	1.4356697	10	Singlet	Immune_cells
SAT_26M	1878.2	1171	0	8	Singlet	Mesothelial
SAT_26M	3969	1941	0.0445113	0	Singlet	Adipocyte
SAT_26M	3937.5	1871	6.4090786	7	Singlet	Endothelial_cells
SAT_26M	1961.9	1252	1.5997114	0	Singlet	Adipocyte
SAT_26M	1009.5	636	4.407931	3	Singlet	FAPs
SAT_26M	4474.1	2248	5.344621	5	Singlet	Immune_cells
SAT_26M	2330.1	1484	3.9993982	9	Singlet	Mesothelial
SAT_26M	1540.3	872	1.4212832	10	Singlet	Immune_cells
SAT_26M	1705.5	1064	0.7401605	0	Singlet	Adipocyte
SAT_26M	1608.5	1009	4.7470864	0	Singlet	Adipocyte
SAT_26M	1844.2	1172	0.3391037	0	Singlet	Adipocyte
SAT_26M	2810.2	1712	5.5825277	8	Singlet	Mesothelial
SAT_26M	2262.5	1339	1.4814539	0	Singlet	Adipocyte
SAT_26M	2140.3	1244	7.2244349	7	Singlet	Endothelial_cells
SAT_26M	1400.5	777	5.3420058	10	Singlet	Immune_cells
SAT_26M	1790.1	1087	1.0614178	0	Singlet	Adipocyte
SAT_26M	1064.8	768	0.5120479	0	Singlet	Adipocyte
SAT_26M	1047.8	683	6.308255	7	Singlet	Endothelial_cells
SAT_26M	2048.5	1159	0	0	Singlet	Adipocyte
SAT_26M	1715.6	1231	2.2102581	11	Singlet	Smooth_muscle_cells
SAT_26M	1003.9	627	2.1299543	5	Singlet	Immune_cells
SAT_26M	1709.6	1061	0.034523	0	Singlet	Adipocyte
SAT_26M	5414.6	2501	0.3579903	0	Singlet	Adipocyte
SAT_26M	1589.5	923	3.3250778	10	Singlet	Immune_cells
SAT_26M	2615.3	1235	11.251326	0	Singlet	Adipocyte
SAT_26M	2630.6	1409	0	0	Singlet	Adipocyte
SAT_26M	1329.8	940	5.7050391	1	Singlet	Immune_cells
SAT_26M	5088.8	2495	5.0106048	0	Singlet	Adipocyte
SAT_26M	1039.3	694	0.2439762	0	Singlet	Adipocyte
SAT_26M	1069.2	681	3.2489818	0	Singlet	Adipocyte
SAT_26M	2313.1	1434	1.1027169	3	Singlet	FAPs
SAT_26M	1418.7	870	5.0712139	0	Singlet	Adipocyte
SAT_26M	1234.6	826	2.9052511	7	Singlet	Endothelial_cells
SAT_26M	1572	917	0.2883429	0	Singlet	Adipocyte
SAT_26M	2053.8	1190	10.801227	0	Singlet	Adipocyte
SAT_26M	2154.9	1197	1.6614258	10	Singlet	Immune_cells

SAT_26M	1076.1	750	0.2387069	9	Singlet	Mesothelial
SAT_26M	1197.5	842	0.2213362	0	Singlet	Adipocyte
SAT_26M	2620.4	1507	0.1190182	0	Singlet	Adipocyte
SAT_26M	2163.5	1314	4.749226	0	Singlet	Adipocyte
SAT_26M	1032.5	694	1.2858657	3	Singlet	FAPs
SAT_26M	3092.8	1556	0	0	Singlet	Adipocyte
SAT_26M	1250.7	576	12.652984	0	Singlet	Adipocyte
SAT_26M	1471.2	706	13.846874	7	Singlet	Endothelial_cells
SAT_26M	1792.9	1087	3.7100755	9	Singlet	Mesothelial
SAT_26M	1313	857	0.0704178	0	Singlet	Adipocyte
SAT_26M	2052.3	1169	0.0181932	0	Singlet	Adipocyte
SAT_26M	1197.5	749	5.8066422	0	Singlet	Adipocyte
SAT_26M	2583.8	1504	5.561445	7	Singlet	Endothelial_cells
SAT_26M	1434.8	792	11.76565	0	Singlet	Adipocyte
SAT_26M	8008.9	3184	1.3169435	0	Singlet	Adipocyte
SAT_26M	1441.5	913	0.1079947	3	Singlet	FAPs
SAT_26M	1421.5	819	3.9536471	3	Singlet	FAPs
SAT_26M	1010	690	1.5085429	0	Singlet	Adipocyte
SAT_26M	1661.2	1168	1.7391327	4	Singlet	Immune_cells
SAT_26M	1114	769	0	8	Singlet	Mesothelial
SAT_26M	1179	626	0.2202006	10	Singlet	Immune_cells
SAT_26M	1050.6	609	6.5359891	7	Singlet	Endothelial_cells
SAT_26M	1809.6	1094	4.296969	0	Singlet	Adipocyte
SAT_26M	1880.4	1225	0.7103645	5	Singlet	Immune_cells
SAT_26M	4142.2	1622	14.521289	7	Singlet	Endothelial_cells
SAT_26M	1115.7	677	12.607968	0	Singlet	Adipocyte
SAT_26M	1699	1065	7.5393484	4	Singlet	Immune_cells
SAT_26M	1649.1	890	1.1715506	10	Singlet	Immune_cells
SAT_26M	2544.6	1530	2.1293307	5	Singlet	Immune_cells
SAT_26M	2955.8	1144	11.564519	0	Singlet	Adipocyte
SAT_26M	1069.3	719	0.9093131	0	Singlet	Adipocyte
SAT_26M	3310	1882	0	5	Singlet	Immune_cells
SAT_26M	6824.2	2809	2.5615155	10	Singlet	Immune_cells
SAT_26M	1486.6	1064	0	5	Singlet	Immune_cells
SAT_26M	1311.8	826	5.7578803	8	Singlet	Mesothelial
SAT_26M	1777.9	1005	11.285138	0	Singlet	Adipocyte
SAT_26M	1477	905	1.6776861	0	Singlet	Adipocyte
SAT_26M	1412.3	763	9.3601191	0	Singlet	Adipocyte
SAT_26M	2222.2	1357	1.0232127	0	Singlet	Adipocyte
SAT_26M	2062.2	1234	3.7908064	9	Singlet	Mesothelial
SAT_26M	1566.8	1102	4.3425453	1	Singlet	Immune_cells
SAT_26M	1628.6	1123	4.6238675	0	Singlet	Adipocyte
SAT_26M	1849.8	1127	0.5087869	0	Singlet	Adipocyte
SAT_26M	1099.4	821	0	11	Singlet	Smooth_muscle_cells
SAT_26M	1143.6	625	1.4549284	0	Singlet	Adipocyte
SAT_26M	1078.4	740	1.4080877	0	Singlet	Adipocyte
SAT_26M	1401.1	927	0.9491703	8	Singlet	Mesothelial
SAT_26M	1984.3	1259	0.5486242	3	Singlet	FAPs
SAT_26M	3760.8	2200	6.072126	8	Singlet	Mesothelial
SAT_26M	1009.8	710	6.2789289	1	Singlet	Immune_cells
SAT_26M	2834	1385	6.5686063	0	Singlet	Adipocyte

SAT_26M	1624.1	943	6.8615467	7	Singlet	Endothelial_cells
SAT_26M	1671.4	988	0.3904787	8	Singlet	Mesothelial
SAT_26M	2884.3	1648	0.3256555	0	Singlet	Adipocyte
SAT_26M	2265.3	1334	0.6728284	7	Singlet	Endothelial_cells
SAT_26M	1159.3	741	1.3914928	7	Singlet	Endothelial_cells
SAT_26M	2913.2	1620	0.1496914	0	Singlet	Adipocyte
SAT_26M	1262.1	931	5.4378849	1	Singlet	Immune_cells
SAT_26M	1518.6	944	9.4761951	0	Singlet	Adipocyte
SAT_26M	1059.6	702	0.329392	3	Singlet	FAPs
SAT_26M	5711.3	2687	0.0264873	0	Singlet	Adipocyte
SAT_26M	1532	981	0.3382096	0	Singlet	Adipocyte
SAT_26M	1602.7	1035	0.0135579	5	Singlet	Immune_cells
SAT_26M	2306.9	1253	10.169209	7	Singlet	Endothelial_cells
SAT_26M	2310.6	1104	11.12023	0	Singlet	Adipocyte
SAT_26M	1730.6	1055	8.1862032	0	Singlet	Adipocyte
SAT_26M	1300.2	776	11.490758	7	Singlet	Endothelial_cells
SAT_26M	1296.4	597	3.8633452	3	Singlet	FAPs
SAT_26M	3633.6	1857	7.5243339	8	Singlet	Mesothelial
SAT_26M	1177.8	717	4.4694405	0	Singlet	Adipocyte
SAT_26M	1467.2	1005	0.0122281	3	Singlet	FAPs
SAT_26M	1698.5	1049	0.6210972	0	Singlet	Adipocyte
SAT_26M	3894	1730	14.484095	7	Singlet	Endothelial_cells
SAT_26M	1056.6	719	0.7483692	6	Singlet	Neurons
SAT_26M	2152.6	1279	0.1103897	0	Singlet	Adipocyte
SAT_26M	1264.7	896	3.5399329	5	Singlet	Immune_cells
SAT_26M	1898.4	1047	1.6937171	3	Singlet	FAPs
SAT_26M	1630.6	1052	3.8833648	7	Singlet	Endothelial_cells
SAT_26M	1281.9	805	5.447709	0	Singlet	Adipocyte
SAT_26M	1407.1	993	7.2640597	8	Singlet	Mesothelial
SAT_26M	4093	2038	0.4748466	0	Singlet	Adipocyte
SAT_26M	2368.4	1087	9.3724259	0	Singlet	Adipocyte
SAT_26M	1061.2	597	0.1839458	9	Singlet	Mesothelial
SAT_26M	1691.1	1150	2.9938219	0	Singlet	Adipocyte
SAT_26M	1164	705	8.9004493	0	Singlet	Adipocyte
SAT_26M	1355.8	907	0.2088816	0	Singlet	Adipocyte
SAT_26M	1020.9	699	3.5226637	5	Singlet	Immune_cells
SAT_26M	1132.9	607	10.956149	7	Singlet	Endothelial_cells
SAT_26M	2656	1579	2.8318132	0	Singlet	Adipocyte
SAT_26M	1186.8	680	9.8768089	0	Singlet	Adipocyte
SAT_26M	1324.4	961	0.009831	0	Singlet	Adipocyte
SAT_26M	1149.3	841	1.5377778	6	Singlet	Neurons
SAT_26M	1439.2	967	0.860487	0	Singlet	Adipocyte
SAT_26M	1946.1	1246	0	0	Singlet	Adipocyte
SAT_26M	1758	1154	7.1789203	1	Singlet	Immune_cells
SAT_26M	1712.1	1178	0	5	Singlet	Immune_cells
SAT_26M	1047.4	707	0.047274	0	Singlet	Adipocyte
SAT_26M	1809.5	1164	0.0015293	8	Singlet	Mesothelial
SAT_26M	2055.2	1279	1.4877894	5	Singlet	Immune_cells
SAT_26M	1902.6	1293	3.624834	9	Singlet	Mesothelial
SAT_26M	1129.8	706	1.3286959	0	Singlet	Adipocyte
SAT_26M	1244.9	773	5.5754907	0	Singlet	Adipocyte

SAT_26M	2570.4	1435	0.0746891	8	Singlet	Mesothelial
SAT_26M	2160.5	1239	0.5536193	0	Singlet	Adipocyte
SAT_26M	2713.7	1171	11.486189	0	Singlet	Adipocyte
SAT_26M	1340.4	983	0.1076728	6	Singlet	Neurons
SAT_26M	1742.1	1056	0.0224257	8	Singlet	Mesothelial
SAT_26M	1446.7	997	0.2095502	0	Singlet	Adipocyte
SAT_26M	1531	915	2.3910832	0	Singlet	Adipocyte
SAT_26M	2843.5	1553	0.0659593	0	Singlet	Adipocyte
SAT_26M	4033.3	2174	1.5054362	8	Singlet	Mesothelial
SAT_26M	2579.9	1417	0.0122334	0	Singlet	Adipocyte
SAT_26M	1189.8	801	3.2818777	0	Singlet	Adipocyte
SAT_26M	2815.9	1445	0.0238681	0	Singlet	Adipocyte
SAT_26M	1157.6	807	2.1024734	8	Singlet	Mesothelial
SAT_26M	5312.7	2363	0.0896545	0	Singlet	Adipocyte
SAT_26M	1307.6	747	8.0251995	7	Singlet	Endothelial_cells
SAT_26M	2368.6	1308	0.0091224	9	Singlet	Mesothelial
SAT_26M	3043.3	1586	1.2892689	0	Singlet	Adipocyte
SAT_26M	3874.5	2159	4.3872066	9	Singlet	Mesothelial
SAT_26M	4331	2043	0.9281228	0	Singlet	Adipocyte
SAT_26M	1700.8	1137	1.9230245	1	Singlet	Immune_cells
SAT_26M	1557.9	997	1.4708345	5	Singlet	Immune_cells
SAT_26M	1461.7	916	6.0076163	8	Singlet	Mesothelial
SAT_26M	1427.7	896	0.9842434	0	Singlet	Adipocyte
SAT_26M	1019.4	694	4.7747818	0	Singlet	Adipocyte
SAT_26M	1472.1	775	13.247535	7	Singlet	Endothelial_cells
SAT_26M	1097	707	1.5374905	3	Singlet	FAPs
SAT_26M	1501.3	902	11.581942	7	Singlet	Endothelial_cells
SAT_26M	3177	1805	2.5428017	9	Singlet	Mesothelial
SAT_26M	1016.1	719	0.5571567	8	Singlet	Mesothelial
SAT_26M	1475	806	6.0844453	0	Singlet	Adipocyte
SAT_26M	2295	1222	10.044008	7	Singlet	Endothelial_cells
SAT_26M	1042	708	5.689374	9	Singlet	Mesothelial
SAT_26M	1888.6	1111	4.8461925	0	Singlet	Adipocyte
SAT_26M	2070.1	1337	0.5766373	0	Singlet	Adipocyte
SAT_26M	1890.5	979	9.8730636	0	Singlet	Adipocyte
SAT_26M	1213.5	795	1.2811103	7	Singlet	Endothelial_cells
SAT_26M	1113.3	735	5.4486294	7	Singlet	Endothelial_cells
SAT_26M	1150.5	829	0	0	Singlet	Adipocyte
SAT_26M	1530	947	7.2668648	9	Singlet	Mesothelial
SAT_26M	1007.7	682	0.088966	8	Singlet	Mesothelial
SAT_26M	2388.8	1295	9.6996384	7	Singlet	Endothelial_cells
SAT_26M	2725.9	1515	6.225732	7	Singlet	Endothelial_cells
SAT_26M	3955.5	2071	1.7483643	0	Singlet	Adipocyte
SAT_26M	2576.5	1339	0.1772497	0	Singlet	Adipocyte
SAT_26M	1139.3	753	5.7063139	7	Singlet	Endothelial_cells
SAT_26M	1021.7	660	2.8456758	5	Singlet	Immune_cells
SAT_26M	1573.3	1032	0.2564648	3	Singlet	FAPs
SAT_26M	1755.5	970	6.5461865	7	Singlet	Endothelial_cells
SAT_26M	1415.9	952	2.0874084	1	Singlet	Immune_cells
SAT_26M	1698.6	936	1.7641817	5	Singlet	Immune_cells
SAT_26M	1074.5	723	0.0764465	0	Singlet	Adipocyte

SAT_26M	3187.3	1624	0.0038107	0	Singlet	Adipocyte
SAT_26M	1066.3	702	0.1727517	0	Singlet	Adipocyte
SAT_26M	1011.8	642	6.431376	7	Singlet	Endothelial_cells
SAT_26M	1243.4	822	4.9530401	5	Singlet	Immune_cells
SAT_26M	1087.8	726	1.584977	6	Singlet	Neurons
SAT_26M	2968.5	1639	1.2917221	0	Singlet	Adipocyte
SAT_26M	2861.1	1775	0.0600287	0	Singlet	Adipocyte
SAT_26M	1985.9	994	12.388117	7	Singlet	Endothelial_cells
SAT_26M	1755.2	1148	4.8745697	4	Singlet	Immune_cells
SAT_26M	1478.9	942	0.2287206	0	Singlet	Adipocyte
SAT_26M	1214.2	751	10.330844	5	Singlet	Immune_cells
SAT_26M	1875.5	1102	0.0269589	0	Singlet	Adipocyte
SAT_26M	2947.6	1585	0.4280218	0	Singlet	Adipocyte
SAT_26M	2580.1	1431	3.1178539	7	Singlet	Endothelial_cells
SAT_26M	1650.6	885	9.5576193	7	Singlet	Endothelial_cells
SAT_26M	1017.2	748	1.7076105	0	Singlet	Adipocyte
SAT_26M	1108.1	784	4.4409462	4	Singlet	Immune_cells
SAT_26M	1388.5	827	0.6517316	0	Singlet	Adipocyte
SAT_26M	1129.1	776	3.0821554	1	Singlet	Immune_cells
SAT_26M	2450.7	1373	2.477279	0	Singlet	Adipocyte
SAT_26M	1918.8	1036	8.3337591	7	Singlet	Endothelial_cells
SAT_26M	4245.2	2230	0.1046358	0	Singlet	Adipocyte
SAT_26M	1616.6	1071	0	8	Singlet	Mesothelial
SAT_26M	1370.8	914	0.0010349	8	Singlet	Mesothelial
SAT_26M	2232.3	1288	7.0093218	0	Singlet	Adipocyte
SAT_26M	1051.2	750	0.0177109	11	Singlet	Smooth_muscle_cells
SAT_26M	3749.9	1859	1.4719959	10	Singlet	Immune_cells
SAT_26M	1177.1	789	0.07272	8	Singlet	Mesothelial
SAT_26M	1188	699	14.980698	7	Singlet	Endothelial_cells
SAT_26M	1221.1	784	0.9797932	2	Singlet	FAPs
SAT_26M	2285.5	1368	0	0	Singlet	Adipocyte
SAT_26M	1413.6	973	0.1455885	0	Singlet	Adipocyte
SAT_26M	1051.8	763	0.7875576	11	Singlet	Smooth_muscle_cells
SAT_26M	4229.3	2140	8.5857078	9	Singlet	Mesothelial
SAT_26M	2010.5	1083	8.3917212	7	Singlet	Endothelial_cells
SAT_26M	1396.2	896	0	0	Singlet	Adipocyte
SAT_26M	1694	899	6.3178878	10	Singlet	Immune_cells
SAT_26M	2454	1405	0.0460836	8	Singlet	Mesothelial
SAT_26M	1454.9	778	7.6768365	0	Singlet	Adipocyte
SAT_26M	1629.2	1021	2.416228	8	Singlet	Mesothelial
SAT_26M	1241	877	1.3501801	1	Singlet	Immune_cells
SAT_26M	4664.9	2112	7.2745446	0	Singlet	Adipocyte
SAT_26M	1638.6	935	0.7884968	10	Singlet	Immune_cells
SAT_26M	1036.6	729	0	8	Singlet	Mesothelial
SAT_26M	1196.9	757	2.9478589	0	Singlet	Adipocyte
SAT_26M	2021.3	1227	0.1479538	0	Singlet	Adipocyte
SAT_26M	2928.8	1463	3.336218	10	Singlet	Immune_cells
SAT_26M	5451.3	1772	9.2391911	0	Singlet	Adipocyte
SAT_26M	3009	1694	0.3408607	0	Singlet	Adipocyte
SAT_26M	1892.2	1153	0.259817	0	Singlet	Adipocyte
SAT_26M	1274.5	780	4.7050826	0	Singlet	Adipocyte

SAT_26M	2083.1	1261	3.6476053	0	Singlet	Adipocyte
SAT_26M	1416	920	0.5398677	2	Singlet	FAPs
SAT_26M	2251.6	1230	4.4611753	0	Singlet	Adipocyte
SAT_26M	1821.5	1109	1.3912654	0	Singlet	Adipocyte
SAT_26M	1237	820	0.0393174	8	Singlet	Mesothelial
SAT_26M	1089.8	724	0.0159782	0	Singlet	Adipocyte
SAT_26M	1697.9	1026	3.9060071	5	Singlet	Immune_cells
SAT_26M	2686.5	1561	3.5037608	0	Singlet	Adipocyte
SAT_26M	3614.3	1922	0.1434721	0	Singlet	Adipocyte
SAT_26M	1131.2	854	0.1694322	6	Singlet	Neurons
SAT_26M	1002.6	723	5.2887321	4	Singlet	Immune_cells
SAT_26M	1650.3	1090	6.0670113	4	Singlet	Immune_cells
SAT_26M	2167.3	1182	3.2675057	7	Singlet	Endothelial_cells
SAT_26M	1648.7	1016	6.2428774	0	Singlet	Adipocyte
SAT_26M	1799.6	1009	0.0848863	0	Singlet	Adipocyte
SAT_26M	2517.2	1500	0.8484944	3	Singlet	FAPs
SAT_26M	2482	1075	6.0332871	10	Singlet	Immune_cells
SAT_26M	1620	1102	0.0813729	0	Singlet	Adipocyte
SAT_26M	10214	4275	1.9388343	9	Singlet	Mesothelial
SAT_26M	2685	1577	0.0565462	11	Singlet	Smooth_muscle_cells
SAT_26M	2804.8	1467	1.5585906	0	Singlet	Adipocyte
SAT_26M	1528.1	1015	4.97777	4	Singlet	Immune_cells
SAT_26M	1857	1109	2.4597441	0	Singlet	Adipocyte
SAT_26M	1263.8	852	7.9478273	1	Singlet	Immune_cells
SAT_26M	3244.4	1558	7.1829781	7	Singlet	Endothelial_cells
SAT_26M	1315.4	905	0.0070699	8	Singlet	Mesothelial
SAT_26M	1363.6	800	12.348009	3	Singlet	FAPs
SAT_26M	1580.6	971	10.721923	4	Singlet	Immune_cells
SAT_26M	1145.3	785	1.6375281	0	Singlet	Adipocyte
SAT_26M	2902	1630	1.6099792	0	Singlet	Adipocyte
SAT_26M	1468	854	1.5227699	2	Singlet	FAPs
SAT_26M	3375.6	1809	1.6325929	0	Singlet	Adipocyte
SAT_26M	1327.7	883	0.8024516	3	Singlet	FAPs
SAT_26M	1629.8	1026	0.0295457	8	Singlet	Mesothelial
SAT_26M	1082.8	733	0.1436469	5	Singlet	Immune_cells
SAT_26M	1720.9	894	13.422428	0	Singlet	Adipocyte
SAT_26M	1544.9	958	0.2027608	0	Singlet	Adipocyte
SAT_26M	2474.2	1473	0.0326616	0	Singlet	Adipocyte
SAT_26M	1175.4	657	10.920098	0	Singlet	Adipocyte
SAT_26M	3678.3	1575	14.389228	7	Singlet	Endothelial_cells
SAT_26M	4083.9	1965	0	0	Singlet	Adipocyte
SAT_26M	1685.3	935	0	6	Singlet	Neurons
SAT_26M	1460.1	1066	4.4962651	1	Singlet	Immune_cells
SAT_26M	1093.4	735	0.1996955	5	Singlet	Immune_cells
SAT_26M	2235.1	1102	10.07607	0	Singlet	Adipocyte
SAT_26M	1380	858	3.2643923	0	Singlet	Adipocyte
SAT_26M	2193.3	1253	1.240386	0	Singlet	Adipocyte
SAT_26M	1204.9	858	0.6121538	6	Singlet	Neurons
SAT_26M	2246.6	1312	4.4847263	8	Singlet	Mesothelial
SAT_26M	2799.9	1603	0.5618158	0	Singlet	Adipocyte
SAT_26M	1272.1	872	0.1370759	0	Singlet	Adipocyte

SAT_26M	2358.8	1264	0.8652946	10	Singlet	Immune_cells
SAT_26M	1871	1054	11.822718	7	Singlet	Endothelial_cells
SAT_26M	3601.5	1588	10.086093	0	Singlet	Adipocyte
SAT_26M	2401.8	1262	7.867068	0	Singlet	Adipocyte
SAT_26M	3141.4	1506	13.185786	9	Singlet	Mesothelial
SAT_26M	2236.8	1378	3.605072	8	Singlet	Mesothelial
SAT_26M	1370.7	922	0	0	Singlet	Adipocyte
SAT_26M	2036.5	1206	1.7076295	7	Singlet	Endothelial_cells
SAT_26M	1717.6	994	2.346506	10	Singlet	Immune_cells
SAT_26M	3369.1	1756	7.3970863	7	Singlet	Endothelial_cells
SAT_26M	1170.1	830	0.0557225	0	Singlet	Adipocyte
SAT_26M	1837.8	968	13.017665	7	Singlet	Endothelial_cells
SAT_26M	1706.2	1045	0.3714495	0	Singlet	Adipocyte
SAT_26M	1466.4	958	0.3994506	0	Singlet	Adipocyte
SAT_26M	1184.7	746	1.5221833	5	Singlet	Immune_cells
SAT_26M	2266	1369	0.1243441	11	Singlet	Smooth_muscle_cells
SAT_26M	1589.5	1040	0.2705548	3	Singlet	FAPs
SAT_26M	4377.2	2159	2.3238275	0	Singlet	Adipocyte
SAT_26M	2222	1255	1.0669775	0	Singlet	Adipocyte
SAT_26M	1071.9	738	2.4966132	5	Singlet	Immune_cells
SAT_26M	1537	943	0.0271219	8	Singlet	Mesothelial
SAT_26M	4530	2294	5.1847836	9	Singlet	Mesothelial
SAT_26M	1149.5	669	11.180009	0	Singlet	Adipocyte
SAT_26M	2516.6	1511	0.0291486	5	Singlet	Immune_cells
SAT_26M	1277.6	886	3.7237796	11	Singlet	Smooth_muscle_cells
SAT_26M	1610.6	906	10.487549	0	Singlet	Adipocyte
SAT_26M	1232.3	797	4.3354234	0	Singlet	Adipocyte
SAT_26M	2707.5	1497	0.1480288	0	Singlet	Adipocyte
SAT_26M	1700.1	1100	0.1661485	8	Singlet	Mesothelial
SAT_26M	1702.4	1211	7.6631309	4	Singlet	Immune_cells
SAT_26M	1171.4	828	0	5	Singlet	Immune_cells
SAT_26M	1486	985	2.4292092	3	Singlet	FAPs
SAT_26M	2041.7	1233	8.9245156	0	Singlet	Adipocyte
SAT_26M	1658.6	984	5.6097043	0	Singlet	Adipocyte
SAT_26M	2471.6	1287	4.9805991	10	Singlet	Immune_cells
SAT_26M	3887.7	2154	0	5	Singlet	Immune_cells
SAT_26M	2057.4	1220	4.0409051	0	Singlet	Adipocyte
SAT_26M	1288.5	817	7.976768	0	Singlet	Adipocyte
SAT_26M	1031.8	700	2.5628463	0	Singlet	Adipocyte
SAT_26M	1450.5	711	12.106847	0	Singlet	Adipocyte
SAT_26M	2285.4	1321	1.4707273	5	Singlet	Immune_cells
SAT_26M	1107.5	820	1.3949533	0	Singlet	Adipocyte
SAT_26M	1529.7	972	2.5281273	0	Singlet	Adipocyte
SAT_26M	2534.8	1432	0.3194147	0	Singlet	Adipocyte
SAT_26M	2328.9	1229	1.681081	10	Singlet	Immune_cells
SAT_26M	2111.7	1228	1.2757557	0	Singlet	Adipocyte
SAT_26M	1051	559	7.5455897	5	Singlet	Immune_cells
SAT_26M	1223.5	794	5.1116493	4	Singlet	Immune_cells
SAT_26M	1389.2	1043	0.3592058	4	Singlet	Immune_cells
SAT_26M	1410.9	739	10.432533	7	Singlet	Endothelial_cells
SAT_26M	1116.8	759	1.0785995	5	Singlet	Immune_cells

SAT_26M	1999.2	1155	0.5644295	0	Singlet	Adipocyte
SAT_26M	3226.8	1577	1.0159435	0	Singlet	Adipocyte
SAT_26M	2145.4	1068	3.731352	10	Singlet	Immune_cells
SAT_26M	1818.3	1081	7.7928295	0	Singlet	Adipocyte
SAT_26M	1202.4	807	0.8136121	6	Singlet	Neurons
SAT_26M	1650.5	1002	2.6189291	0	Singlet	Adipocyte
SAT_26M	2087.7	1184	13.033463	7	Singlet	Endothelial_cells
SAT_26M	1078.7	747	0	0	Singlet	Adipocyte
SAT_26M	1353.4	826	1.0468951	0	Singlet	Adipocyte
SAT_26M	2144.8	1238	5.6180175	8	Singlet	Mesothelial
SAT_26M	1557.2	955	0.3575871	7	Singlet	Endothelial_cells
SAT_26M	2907.5	1645	0	5	Singlet	Immune_cells
SAT_26M	3120.9	1664	0.1309694	0	Singlet	Adipocyte
SAT_26M	2472.7	1266	1.9329614	10	Singlet	Immune_cells
SAT_26M	2035.3	1173	1.6379595	0	Singlet	Adipocyte
SAT_26M	1231.5	837	0.1735177	0	Singlet	Adipocyte
SAT_26M	2300.2	1226	4.9235817	0	Singlet	Adipocyte
SAT_26M	1415.7	847	6.2219446	8	Singlet	Mesothelial
SAT_26M	1850	1280	0.0025206	5	Singlet	Immune_cells
SAT_26M	1684.6	1013	0.4070714	0	Singlet	Adipocyte
SAT_26M	5346.5	2544	0	0	Singlet	Adipocyte
SAT_26M	2143.9	1276	2.7487632	0	Singlet	Adipocyte
SAT_26M	2382.1	1384	8.2596844	8	Singlet	Mesothelial
SAT_26M	2740	1437	0.409119	0	Singlet	Adipocyte
SAT_26M	1067.9	743	0.2473366	3	Singlet	FAPs
SAT_26M	1594.6	990	0.1951928	0	Singlet	Adipocyte
SAT_26M	1086.2	574	13.807676	10	Singlet	Immune_cells
SAT_26M	1094.3	705	5.7476349	0	Singlet	Adipocyte
SAT_26M	2076.7	1465	5.4703201	1	Singlet	Immune_cells
SAT_26M	1158.5	829	1.2967468	4	Singlet	Immune_cells
SAT_26M	2434.8	1379	6.0786993	7	Singlet	Endothelial_cells
SAT_26M	1126.8	769	2.3345775	0	Singlet	Adipocyte
SAT_26M	5103	2274	8.983083	0	Singlet	Adipocyte
SAT_26M	1879.1	1107	0.2435	0	Singlet	Adipocyte
SAT_26M	1441.9	892	0.1100084	0	Singlet	Adipocyte
SAT_26M	2455	1307	1.6723261	0	Singlet	Adipocyte
SAT_26M	1931.1	1071	7.11039	0	Singlet	Adipocyte
SAT_26M	1405.1	956	0	0	Singlet	Adipocyte
SAT_26M	2043.3	1174	7.0934051	0	Singlet	Adipocyte
SAT_26M	5014.8	2421	0.0120803	0	Singlet	Adipocyte
SAT_26M	1153.6	843	2.0762203	1	Singlet	Immune_cells
SAT_26M	1822.9	973	0.6533417	10	Singlet	Immune_cells
SAT_26M	4063.3	2223	6.4280337	9	Singlet	Mesothelial
SAT_26M	1126.7	803	0.1402547	6	Singlet	Neurons
SAT_26M	3677.3	2028	2.0138899	0	Singlet	Adipocyte
SAT_26M	2191.4	1349	0.0624387	0	Singlet	Adipocyte
SAT_26M	1289.5	862	2.6622536	4	Singlet	Immune_cells
SAT_26M	1077.9	734	0.8883136	0	Singlet	Adipocyte
SAT_26M	1372.3	632	9.6020806	0	Singlet	Adipocyte
SAT_26M	3892.4	1887	0.6036076	0	Singlet	Adipocyte
SAT_26M	1219.8	850	0.1067885	5	Singlet	Immune_cells

SAT_26M	1127.5	832	0.5655853	0	Singlet	Adipocyte
SAT_26M	1404.9	937	6.4873093	4	Singlet	Immune_cells
SAT_26M	2326.1	1181	8.9391214	0	Singlet	Adipocyte
SAT_26M	4451.5	2210	5.1455297	9	Singlet	Mesothelial
SAT_26M	1440.1	789	13.906004	7	Singlet	Endothelial_cells
SAT_26M	3703	1903	6.0807514	7	Singlet	Endothelial_cells
SAT_26M	4206.9	2141	2.7461501	0	Singlet	Adipocyte
SAT_26M	1048.1	739	2.4756144	9	Singlet	Mesothelial
SAT_26M	1213.4	815	0	0	Singlet	Adipocyte
SAT_26M	2042.9	1098	4.9166688	7	Singlet	Endothelial_cells
SAT_26M	1101.7	799	4.7759466	1	Singlet	Immune_cells
SAT_26M	2098.1	1122	12.625835	0	Singlet	Adipocyte
SAT_26M	1296.8	873	0.7024265	0	Singlet	Adipocyte
SAT_26M	3675.4	2002	5.292553	9	Singlet	Mesothelial
SAT_26M	3483.6	1746	4.6716552	0	Singlet	Adipocyte
SAT_26M	3129.4	1418	9.7302842	7	Singlet	Endothelial_cells
SAT_26M	2338.8	1391	0.5323808	0	Singlet	Adipocyte
SAT_26M	1981.5	1054	11.659684	7	Singlet	Endothelial_cells
SAT_26M	1092.1	796	2.7841717	4	Singlet	Immune_cells
SAT_26M	1042.2	690	11.5093	4	Singlet	Immune_cells
SAT_26M	1724.6	1126	0.9141176	3	Singlet	FAPs
SAT_26M	1593.8	953	2.7241774	0	Singlet	Adipocyte
SAT_26M	1235.4	853	0.2973377	0	Singlet	Adipocyte
SAT_26M	3076.7	1543	9.0328065	0	Singlet	Adipocyte
SAT_26M	1670.9	857	13.923785	0	Singlet	Adipocyte
SAT_26M	2187.6	1445	0	5	Singlet	Immune_cells
SAT_26M	1379.2	850	1.5442475	3	Singlet	FAPs
SAT_26M	1251.9	806	5.9694315	7	Singlet	Endothelial_cells
SAT_26M	2281.2	1260	4.6461331	0	Singlet	Adipocyte
SAT_26M	1202.8	755	0.5092584	10	Singlet	Immune_cells
SAT_26M	1236.6	791	5.6099055	0	Singlet	Adipocyte
SAT_26M	3995.9	2117	0.8405529	0	Singlet	Adipocyte
SAT_26M	1033.4	703	9.1734674	8	Singlet	Mesothelial
SAT_26M	1255.5	836	0.1805701	0	Singlet	Adipocyte
SAT_26M	1342.1	809	4.9662097	2	Singlet	FAPs
SAT_26M	1896.9	1106	1.7233718	0	Singlet	Adipocyte
SAT_26M	4209.4	2271	0.0091241	11	Singlet	Smooth_muscle_cells
SAT_26M	4290.3	2101	2.3318061	0	Singlet	Adipocyte
SAT_26M	2428.4	1521	2.8314066	1	Singlet	Immune_cells
SAT_26M	1122.9	812	0.0444673	8	Singlet	Mesothelial
SAT_26M	1176.5	790	0	8	Singlet	Mesothelial
SAT_26M	1049.1	811	9.1057739	1	Singlet	Immune_cells
SAT_26M	1374.5	982	0.1613578	0	Singlet	Adipocyte
SAT_26M	1052.2	677	5.0587333	7	Singlet	Endothelial_cells
SAT_26M	1347.4	612	0.9029005	4	Singlet	Immune_cells
SAT_26M	2450	1160	7.8103073	7	Singlet	Endothelial_cells
SAT_26M	1704.5	1041	3.5156659	7	Singlet	Endothelial_cells
SAT_26M	2718.5	1722	0.95421	7	Singlet	Endothelial_cells
SAT_26M	1709.5	1057	3.0462219	0	Singlet	Adipocyte
SAT_26M	1135.8	703	0.0374477	0	Singlet	Adipocyte
SAT_26M	5547.7	2485	0.5849284	0	Singlet	Adipocyte

SAT_26M	2623.6	1087	8.693063	0	Singlet	Adipocyte
SAT_26M	1960.2	1075	8.7834064	7	Singlet	Endothelial_cells
SAT_26M	1289.4	661	7.6813886	0	Singlet	Adipocyte
SAT_26M	1258.9	883	1.3618558	7	Singlet	Endothelial_cells
SAT_26M	1204.3	849	0.0263559	6	Singlet	Neurons
SAT_26M	4082.3	2046	1.7888909	0	Singlet	Adipocyte
SAT_26M	2606	1436	0.6850928	0	Singlet	Adipocyte
SAT_26M	1106.8	791	0.0961542	0	Singlet	Adipocyte
SAT_26M	1718.4	909	7.6502798	7	Singlet	Endothelial_cells
SAT_26M	1323	861	0.2659223	0	Singlet	Adipocyte
SAT_26M	3512.1	1872	2.5243788	0	Singlet	Adipocyte
SAT_26M	1511.7	1059	0.0173104	0	Singlet	Adipocyte
SAT_26M	2160.7	1059	11.080091	7	Singlet	Endothelial_cells
SAT_26M	3617.3	1841	1.3378686	7	Singlet	Endothelial_cells
SAT_26M	1172.5	746	2.8200572	7	Singlet	Endothelial_cells
SAT_26M	1854.7	1153	1.1706112	0	Singlet	Adipocyte
SAT_26M	6299.1	3049	2.9973302	9	Singlet	Mesothelial
SAT_26M	1160	766	2.8625704	3	Singlet	FAPs
SAT_26M	1314.1	777	2.6794655	7	Singlet	Endothelial_cells
SAT_26M	1213.3	842	0	8	Singlet	Mesothelial
SAT_26M	3389.2	1820	0.4580984	0	Singlet	Adipocyte
SAT_26M	1521.1	999	4.9564522	9	Singlet	Mesothelial
SAT_26M	1577.2	1012	4.3453764	7	Singlet	Endothelial_cells
SAT_26M	1266.8	818	4.1569869	7	Singlet	Endothelial_cells
SAT_26M	1467.1	1013	4.5837232	1	Singlet	Immune_cells
SAT_26M	1801.4	1063	1.6007145	0	Singlet	Adipocyte
SAT_26M	1367.9	833	4.4958109	7	Singlet	Endothelial_cells
SAT_26M	1151.6	818	0	8	Singlet	Mesothelial
SAT_26M	1606.2	949	2.4428534	0	Singlet	Adipocyte
SAT_26M	5061.4	2311	5.8197321	0	Singlet	Adipocyte
SAT_26M	1766.7	1149	5.7221142	5	Singlet	Immune_cells
SAT_26M	1417.4	962	0	8	Singlet	Mesothelial
SAT_26M	4231.7	2199	4.4789676	0	Singlet	Adipocyte
SAT_26M	1599	828	12.70862	7	Singlet	Endothelial_cells
SAT_26M	2221.4	1165	0.8823738	10	Singlet	Immune_cells
SAT_26M	1598.3	1107	0.2344231	0	Singlet	Adipocyte
SAT_26M	1220.1	776	5.5184606	5	Singlet	Immune_cells
SAT_26M	3108.8	1505	3.2730956	10	Singlet	Immune_cells
SAT_26M	3388.7	1901	5.3691049	7	Singlet	Endothelial_cells
SAT_26M	2117.8	1350	3.2208606	1	Singlet	Immune_cells
SAT_26M	2020	1171	0.1250873	0	Singlet	Adipocyte
SAT_26M	1422.1	940	0.2300152	0	Singlet	Adipocyte
SAT_26M	2537.9	1371	5.4185135	7	Singlet	Endothelial_cells
SAT_26M	1225.6	864	0.4078923	0	Singlet	Adipocyte
SAT_26M	1146.8	748	0.2242032	0	Singlet	Adipocyte
SAT_26M	1110.4	663	6.5858179	0	Singlet	Adipocyte
SAT_26M	1201.3	874	0.0471356	0	Singlet	Adipocyte
SAT_26M	2364.3	1309	5.6575102	0	Singlet	Adipocyte
SAT_26M	1073.6	771	0.2191393	8	Singlet	Mesothelial
SAT_26M	2984.2	1715	4.632413	0	Singlet	Adipocyte
SAT_26M	1005.7	621	7.0766829	0	Singlet	Adipocyte

SAT_26M	4392.8	2408	1.9388431	8	Singlet	Mesothelial
SAT_26M	1283.7	817	1.7399962	0	Singlet	Adipocyte
SAT_26M	1267.1	793	1.5590663	0	Singlet	Adipocyte
SAT_26M	2075	1265	7.216726	8	Singlet	Mesothelial
SAT_26M	2202.1	1287	3.6209738	0	Singlet	Adipocyte
SAT_26M	1174.9	806	0.0946484	8	Singlet	Mesothelial
SAT_26M	2923.5	1568	0.7083467	0	Singlet	Adipocyte
SAT_26M	1069.7	708	0.1344285	0	Singlet	Adipocyte
SAT_26M	1013.4	760	4.973079	1	Singlet	Immune_cells
SAT_26M	2166	1354	0.3400854	5	Singlet	Immune_cells
SAT_26M	1363.7	969	0.5313438	1	Singlet	Immune_cells
SAT_26M	1696.9	1039	0.0208637	8	Singlet	Mesothelial
SAT_26M	3997.9	1545	8.5774651	0	Singlet	Adipocyte
SAT_26M	4096.2	1718	0.9117945	10	Singlet	Immune_cells
SAT_26M	1011.4	815	0.0190764	5	Singlet	Immune_cells
SAT_26M	3384.6	1784	0.0530457	0	Singlet	Adipocyte
SAT_26M	1339.1	737	6.2473286	3	Singlet	FAPs
SAT_26M	1993.2	953	10.467334	7	Singlet	Endothelial_cells
SAT_26M	1543.4	934	7.1041145	0	Singlet	Adipocyte
SAT_26M	1366.8	891	0.3268124	0	Singlet	Adipocyte
SAT_26M	1095.8	762	5.9442231	2	Singlet	FAPs
SAT_26M	1575.2	899	2.7387314	0	Singlet	Adipocyte
SAT_26M	2089	1146	9.7943271	0	Singlet	Adipocyte
SAT_26M	1629.2	921	0.0399426	6	Singlet	Neurons
SAT_26M	3955.1	2026	0.7551644	0	Singlet	Adipocyte
SAT_26M	2829.3	1577	0	8	Singlet	Mesothelial
SAT_26M	2470.2	1340	3.7093534	7	Singlet	Endothelial_cells
SAT_26M	1064.5	746	1.2997005	0	Singlet	Adipocyte
SAT_26M	1589.4	993	1.8617995	0	Singlet	Adipocyte
SAT_26M	1586.4	867	8.5985617	0	Singlet	Adipocyte
SAT_26M	2151.7	1193	5.2875556	0	Singlet	Adipocyte
SAT_26M	3164.5	1664	0.3123017	0	Singlet	Adipocyte
SAT_26M	1254.5	795	0.2288427	0	Singlet	Adipocyte
SAT_26M	4208.4	2097	0	0	Singlet	Adipocyte
SAT_26M	3898.7	1969	1.2443608	0	Singlet	Adipocyte
SAT_26M	1527.3	934	0.2203504	0	Singlet	Adipocyte
SAT_26M	1520	983	10.862907	6	Singlet	Neurons
SAT_26M	1587.3	978	6.3290559	0	Singlet	Adipocyte
SAT_26M	1836.4	1132	0.3007891	0	Singlet	Adipocyte
SAT_26M	1941.5	1155	0.5411222	0	Singlet	Adipocyte
SAT_26M	1614.2	1127	4.802491	0	Singlet	Adipocyte
SAT_26M	4428.5	2113	0.276641	0	Singlet	Adipocyte
SAT_26M	2048	1114	4.6457714	0	Singlet	Adipocyte
SAT_26M	1558.8	1029	2.7228076	8	Singlet	Mesothelial
SAT_26M	2917	1580	2.7521393	0	Singlet	Adipocyte
SAT_26M	1701.3	1070	0.6525225	0	Singlet	Adipocyte
SAT_26M	1663.6	1074	0	0	Singlet	Adipocyte
SAT_26M	1718.5	1070	0	8	Singlet	Mesothelial
SAT_26M	2206.5	1299	2.1063513	0	Singlet	Adipocyte
SAT_26M	1222.1	709	5.0114642	0	Singlet	Adipocyte
SAT_26M	2215.5	1313	1.2427928	0	Singlet	Adipocyte

SAT_26M	1457	867	3.0793631	10	Singlet	Immune_cells
SAT_26M	1464	1032	0	0	Singlet	Adipocyte
SAT_26M	6508.5	2816	2.0778911	0	Singlet	Adipocyte
SAT_26M	1359	939	0.0994728	0	Singlet	Adipocyte
SAT_26M	1925.9	1308	0.0494355	11	Singlet	Smooth_muscle_cells
SAT_26M	1857.5	1119	5.9950529	7	Singlet	Endothelial_cells
SAT_26M	2266.3	1326	14.125649	7	Singlet	Endothelial_cells
SAT_26M	4569.6	2275	1.0040386	0	Singlet	Adipocyte
SAT_26M	1220.6	768	6.757765	0	Singlet	Adipocyte
SAT_26M	1328.2	883	0.0221011	8	Singlet	Mesothelial
SAT_26M	2593.6	1386	4.9057961	0	Singlet	Adipocyte
SAT_26M	1946.3	1228	0.0688287	8	Singlet	Mesothelial
SAT_26M	8453.4	3054	1.6320814	0	Singlet	Adipocyte
SAT_26M	1632.2	1006	5.952659	0	Singlet	Adipocyte
SAT_26M	1078.6	735	2.540285	0	Singlet	Adipocyte
SAT_26M	1206	721	4.7707478	0	Singlet	Adipocyte
SAT_26M	3583.9	1976	6.5273784	9	Singlet	Mesothelial
SAT_26M	1146.7	710	10.101098	0	Singlet	Adipocyte
SAT_26M	3185.8	1858	1.1186765	7	Singlet	Endothelial_cells
SAT_26M	1411.4	989	1.6916246	11	Singlet	Smooth_muscle_cells
SAT_26M	1284.5	697	2.9081326	5	Singlet	Immune_cells
SAT_26M	1709.1	1077	0.3625201	0	Singlet	Adipocyte
SAT_26M	2658.8	1611	5.8613541	7	Singlet	Endothelial_cells
SAT_26M	7222.6	3719	3.90901	8	Singlet	Mesothelial
SAT_26M	2059.6	1290	2.8427043	0	Singlet	Adipocyte
SAT_26M	1123.8	727	2.3878368	0	Singlet	Adipocyte
SAT_26M	3491.9	1874	0.3416172	0	Singlet	Adipocyte
SAT_26M	1933	1131	3.699766	0	Singlet	Adipocyte
SAT_26M	1642.4	928	5.3521139	10	Singlet	Immune_cells
SAT_26M	1112.5	702	7.6227425	0	Singlet	Adipocyte
SAT_26M	2585.1	1490	3.5388269	0	Singlet	Adipocyte
SAT_26M	2327.4	1363	1.6042537	0	Singlet	Adipocyte
SAT_26M	1036.4	737	0.0948109	8	Singlet	Mesothelial
SAT_26M	1480	1039	4.9767088	1	Singlet	Immune_cells
SAT_26M	2250.1	1274	3.7366581	0	Singlet	Adipocyte
SAT_26M	1327.6	859	1.7737583	0	Singlet	Adipocyte
SAT_26M	1137.6	774	2.6465105	0	Singlet	Adipocyte
SAT_26M	1476.8	917	1.6156631	0	Singlet	Adipocyte
SAT_26M	1027.1	676	1.1831279	0	Singlet	Adipocyte
SAT_26M	1916	1381	0.0532843	11	Singlet	Smooth_muscle_cells
SAT_26M	3750.8	1937	3.5443557	0	Singlet	Adipocyte
SAT_26M	1045.8	723	0.2705369	0	Singlet	Adipocyte
SAT_26M	1000.6	724	0.0296452	0	Singlet	Adipocyte
SAT_26M	2765.5	1521	0.1977396	0	Singlet	Adipocyte
SAT_26M	1635.4	1060	0.054727	8	Singlet	Mesothelial
SAT_26M	1689.5	1150	0.4065492	4	Singlet	Immune_cells
SAT_26M	1540.2	947	0.6957298	0	Singlet	Adipocyte
SAT_26M	1241.3	927	0.2723336	1	Singlet	Immune_cells
SAT_26M	1877.8	1249	1.3209549	7	Singlet	Endothelial_cells
SAT_26M	1534.5	901	3.91647	3	Singlet	FAPs
SAT_26M	1030.6	731	0.1732569	3	Singlet	FAPs

SAT_26M	1395.5	949	0.9806437	9	Singlet	Mesothelial
SAT_26M	5072.2	2370	1.1234192	0	Singlet	Adipocyte
SAT_26M	3913.4	2019	1.7734356	10	Singlet	Immune_cells
SAT_26M	4748.9	2165	0.0730178	0	Singlet	Adipocyte
SAT_26M	2388.5	1395	0.0927024	0	Singlet	Adipocyte
SAT_26M	1976.7	1162	4.0626924	0	Singlet	Adipocyte
SAT_26M	1414.9	860	1.544144	7	Singlet	Endothelial_cells
SAT_26M	2286.4	1322	2.1201532	0	Singlet	Adipocyte
SAT_26M	2284.8	1327	1.5459581	0	Singlet	Adipocyte
SAT_26M	1458.1	833	11.046073	5	Singlet	Immune_cells
SAT_26M	1198	852	0.6502292	8	Singlet	Mesothelial
SAT_26M	1500	930	5.4210725	3	Singlet	FAPs
SAT_26M	1359.7	868	2.8954126	7	Singlet	Endothelial_cells
SAT_26M	1995.8	1133	3.1674658	0	Singlet	Adipocyte
SAT_26M	1036.1	653	0.3106034	9	Singlet	Mesothelial
SAT_26M	3168.6	1900	5.9582388	9	Singlet	Mesothelial
SAT_26M	1550.3	953	5.3945599	0	Singlet	Adipocyte
SAT_26M	1740.3	1035	4.6824281	5	Singlet	Immune_cells
SAT_26M	1730.4	1027	1.275576	0	Singlet	Adipocyte
SAT_26M	2466.6	1394	4.9808999	0	Singlet	Adipocyte
SAT_26M	1327.5	778	12.531669	7	Singlet	Endothelial_cells
SAT_26M	6561.2	2742	0.2231628	0	Singlet	Adipocyte
SAT_26M	3665.9	1833	5.6652668	7	Singlet	Endothelial_cells
SAT_26M	1379.2	928	0.5050072	0	Singlet	Adipocyte
SAT_26M	1044.9	760	1.1447127	5	Singlet	Immune_cells
SAT_26M	1768.1	1072	1.4437215	0	Singlet	Adipocyte
SAT_26M	1353.2	869	0.136079	0	Singlet	Adipocyte
SAT_26M	4381.2	2051	4.4282875	0	Singlet	Adipocyte
SAT_26M	1508.2	964	2.6730577	7	Singlet	Endothelial_cells
SAT_26M	1546	876	11.73228	5	Singlet	Immune_cells
SAT_26M	1061.5	696	0.0206145	0	Singlet	Adipocyte
SAT_26M	1530.5	973	3.8760343	0	Singlet	Adipocyte
SAT_26M	1688.1	944	9.2657233	0	Singlet	Adipocyte
SAT_26M	1131.1	691	0.1730176	8	Singlet	Mesothelial
SAT_26M	1297.7	882	4.2010546	4	Singlet	Immune_cells
SAT_26M	1800.1	1181	2.2004461	0	Singlet	Adipocyte
SAT_26M	3037	1591	0.4222956	0	Singlet	Adipocyte
SAT_26M	1066.4	737	0.6991629	0	Singlet	Adipocyte
SAT_26M	1512.4	727	10.206748	7	Singlet	Endothelial_cells
SAT_26M	3595.2	1874	2.2160557	0	Singlet	Adipocyte
SAT_26M	1487.1	1032	7.9601595	8	Singlet	Mesothelial
SAT_26M	3802.9	1960	1.8262826	0	Singlet	Adipocyte
SAT_26M	1888.4	1109	2.9840138	0	Singlet	Adipocyte
SAT_26M	1423.4	782	3.5759873	7	Singlet	Endothelial_cells
SAT_26M	3198.4	1772	7.7145881	9	Singlet	Mesothelial
SAT_26M	3745.1	1906	7.2272264	7	Singlet	Endothelial_cells
SAT_26M	1053.4	617	8.3821863	10	Singlet	Immune_cells
SAT_26M	1417.6	843	7.0186801	7	Singlet	Endothelial_cells
SAT_26M	1445.8	878	1.5719115	2	Singlet	FAPs
SAT_26M	4559	2098	1.7830738	0	Singlet	Adipocyte
SAT_26M	2622.9	1546	0.11391	0	Singlet	Adipocyte

SAT_26M	1582	1136	1.7634811	11	Singlet	Smooth_muscle_cells
SAT_26M	1821	1133	0	8	Singlet	Mesothelial
SAT_26M	1268.2	929	1.1062392	1	Singlet	Immune_cells
SAT_26M	1703	1068	0.6287355	0	Singlet	Adipocyte
SAT_26M	1215.2	791	0.0314086	0	Singlet	Adipocyte
SAT_26M	2746.5	1458	0.9826872	0	Singlet	Adipocyte
SAT_26M	3173.9	1838	5.5210448	8	Singlet	Mesothelial
SAT_26M	4345.9	2186	0	0	Singlet	Adipocyte
SAT_26M	1010	729	0.2696692	0	Singlet	Adipocyte
SAT_26M	1017.8	671	0.9818661	0	Singlet	Adipocyte
SAT_26M	4076.2	2092	7.444521	0	Singlet	Adipocyte
SAT_26M	2357.8	1149	11.107497	0	Singlet	Adipocyte
SAT_26M	1190.7	796	0.5847344	0	Singlet	Adipocyte
SAT_26M	1633.7	997	2.5296123	0	Singlet	Adipocyte
SAT_26M	1997.8	1160	0.0733239	0	Singlet	Adipocyte
SAT_26M	2448.2	1354	3.5938775	0	Singlet	Adipocyte
SAT_26M	4541.6	1994	10.757215	7	Singlet	Endothelial_cells
SAT_26M	1580.2	907	5.6577176	0	Singlet	Adipocyte
SAT_26M	1025.8	724	0	7	Singlet	Endothelial_cells
SAT_26M	1093.6	723	0.7737117	0	Singlet	Adipocyte
SAT_26M	1345.8	677	13.336395	0	Singlet	Adipocyte
SAT_26M	1361.8	898	2.1277485	0	Singlet	Adipocyte
SAT_26M	2426.8	1485	0.0553428	0	Singlet	Adipocyte
SAT_26M	1015.3	712	0	0	Singlet	Adipocyte
SAT_26M	2216.7	1285	2.8431138	7	Singlet	Endothelial_cells
SAT_26M	1854.5	1056	2.3672388	10	Singlet	Immune_cells
SAT_26M	4379.7	1769	1.8418345	10	Singlet	Immune_cells
SAT_26M	1121.1	695	3.8189718	0	Singlet	Adipocyte
SAT_26M	1258	851	5.7641502	4	Singlet	Immune_cells
SAT_26M	1643.5	1077	2.8130672	0	Singlet	Adipocyte
SAT_26M	1318.5	840	5.0191536	0	Singlet	Adipocyte
SAT_26M	1146.8	747	5.7511188	5	Singlet	Immune_cells
SAT_26M	1579.1	728	10.838819	0	Singlet	Adipocyte
SAT_26M	2175.8	1329	0.2392478	0	Singlet	Adipocyte
SAT_26M	1081.2	668	4.9764099	0	Singlet	Adipocyte
SAT_26M	1869.9	1114	3.6154474	0	Singlet	Adipocyte
SAT_26M	3785.2	1922	0.0964741	0	Singlet	Adipocyte
SAT_26M	4196.8	2056	4.7314428	0	Singlet	Adipocyte
SAT_26M	2022.9	1118	8.2888249	7	Singlet	Endothelial_cells
SAT_26M	2219.8	1422	0	8	Singlet	Mesothelial
SAT_26M	2029.5	1329	0.8916517	0	Singlet	Adipocyte
SAT_26M	2356.3	1277	0.3099231	0	Singlet	Adipocyte
SAT_26M	1646.7	940	8.5347935	10	Singlet	Immune_cells
SAT_26M	1243.8	817	0.1117862	3	Singlet	FAPs
SAT_26M	1176.7	746	0.0132228	6	Singlet	Neurons
SAT_26M	1879.8	1088	4.6001275	0	Singlet	Adipocyte
SAT_26M	1597.2	1030	0.0143374	0	Singlet	Adipocyte
SAT_26M	1447.8	983	0.1570659	0	Singlet	Adipocyte
SAT_26M	1920.8	1278	0.2425915	11	Singlet	Smooth_muscle_cells
SAT_26M	1326.1	863	1.8995265	0	Singlet	Adipocyte
SAT_26M	2723.7	1599	3.8657192	5	Singlet	Immune_cells

SAT_26M	1244.5	800	6.5617922	8	Singlet	Mesothelial
SAT_26M	2538.5	1367	10.16259	7	Singlet	Endothelial_cells
SAT_26M	1339.8	931	0.170196	11	Singlet	Smooth_muscle_cells
SAT_26M	4811.4	2375	0.3398459	0	Singlet	Adipocyte
SAT_26M	2604.5	1613	0.0693333	5	Singlet	Immune_cells
SAT_26M	2811.2	1741	4.1189506	11	Singlet	Smooth_muscle_cells
SAT_26M	1741.1	1037	6.5820449	5	Singlet	Immune_cells
SAT_26M	3139.4	1724	1.7459198	0	Singlet	Adipocyte
SAT_26M	1103.9	679	3.2426492	0	Singlet	Adipocyte
SAT_26M	1082.3	713	6.2586769	0	Singlet	Adipocyte
SAT_26M	2155.7	1248	0.0548511	8	Singlet	Mesothelial
SAT_26M	1108.4	669	8.9097843	0	Singlet	Adipocyte
SAT_26M	1870.6	1135	4.202995	0	Singlet	Adipocyte
SAT_26M	2575.7	1554	0.3534189	0	Singlet	Adipocyte
SAT_26M	1214	785	2.8749625	7	Singlet	Endothelial_cells
SAT_26M	1458.6	1000	1.2224511	0	Singlet	Adipocyte
SAT_26M	1120.6	777	2.5037954	7	Singlet	Endothelial_cells
SAT_26M	4134.5	2048	0.2440464	0	Singlet	Adipocyte
SAT_26M	1092.7	712	4.5429064	6	Singlet	Neurons
SAT_26M	2002.9	1196	0.1996891	0	Singlet	Adipocyte
SAT_26M	5304.9	2556	0.8323786	0	Singlet	Adipocyte
SAT_26M	3708	2228	0.4496834	8	Singlet	Mesothelial
SAT_26M	1187.7	827	1.3188044	0	Singlet	Adipocyte
SAT_26M	1702.9	1079	3.5656127	0	Singlet	Adipocyte
SAT_26M	1112.7	621	5.8859744	10	Singlet	Immune_cells
SAT_26M	1758.3	1195	2.7931073	1	Singlet	Immune_cells
SAT_26M	1026.4	727	0.6358776	6	Singlet	Neurons
SAT_26M	1395.6	902	11.771175	0	Singlet	Adipocyte
SAT_26M	5918.5	2737	6.0279331	9	Singlet	Mesothelial
SAT_26M	1313.5	940	0.1789252	0	Singlet	Adipocyte
SAT_26M	2531.1	1371	1.2862968	0	Singlet	Adipocyte
SAT_26M	1331.8	943	2.119984	3	Singlet	FAPs
SAT_26M	1891.5	1225	2.7659369	9	Singlet	Mesothelial
SAT_26M	1833.8	956	4.857983	10	Singlet	Immune_cells
SAT_26M	2059.2	1267	1.0319084	0	Singlet	Adipocyte
SAT_26M	2702.2	1349	2.2044561	10	Singlet	Immune_cells
SAT_26M	2666.6	1555	0.0857307	0	Singlet	Adipocyte
SAT_26M	3055.7	1783	0.4914755	0	Singlet	Adipocyte
SAT_26M	1228.5	765	1.9463415	7	Singlet	Endothelial_cells
SAT_26M	1157.9	809	1.2929824	7	Singlet	Endothelial_cells
SAT_26M	1294.5	932	2.9060543	1	Singlet	Immune_cells
SAT_26M	1134.5	687	7.647221	0	Singlet	Adipocyte
SAT_26M	2159.2	1113	13.301768	0	Singlet	Adipocyte
SAT_26M	1643.7	1017	6.1466824	0	Singlet	Adipocyte
SAT_26M	1138.4	736	0.8010672	0	Singlet	Adipocyte
SAT_26M	2119.3	1182	4.0416607	7	Singlet	Endothelial_cells
SAT_26M	1843.3	1054	4.0431611	0	Singlet	Adipocyte
SAT_26M	1583.3	887	4.1025375	10	Singlet	Immune_cells
SAT_26M	1766.6	1087	0.6020337	0	Singlet	Adipocyte
SAT_26M	3096.2	1475	1.7417002	10	Singlet	Immune_cells
SAT_26M	1879.3	1073	4.3411409	7	Singlet	Endothelial_cells

SAT_26M	1932.2	1292	0.0354835	8	Singlet	Mesothelial
SAT_26M	2312	1367	0.4547479	0	Singlet	Adipocyte
SAT_26M	1755.4	1082	0.3316178	0	Singlet	Adipocyte
SAT_26M	3041.6	1766	0	8	Singlet	Mesothelial
SAT_26M	1010.9	702	0.2639397	0	Singlet	Adipocyte
SAT_26M	3322.6	1730	0.09442	0	Singlet	Adipocyte
SAT_26M	1732.6	1130	1.1567296	0	Singlet	Adipocyte
SAT_26M	1155.5	754	1.5910938	0	Singlet	Adipocyte
SAT_26M	1756.7	895	1.9186961	10	Singlet	Immune_cells
SAT_26M	4812.5	2198	2.8216834	0	Singlet	Adipocyte
SAT_26M	1489.7	955	2.5017148	7	Singlet	Endothelial_cells
SAT_26M	1148.8	772	0.2317023	0	Singlet	Adipocyte
SAT_26M	1592.3	939	0.4348189	0	Singlet	Adipocyte
SAT_26M	1045.2	780	2.8268923	4	Singlet	Immune_cells
SAT_26M	3301.7	1783	0	0	Singlet	Adipocyte
SAT_26M	1184.9	788	2.4129963	0	Singlet	Adipocyte
SAT_26M	1717.3	761	10.568809	0	Singlet	Adipocyte
SAT_26M	1716.9	1216	0.9630375	11	Singlet	Smooth_muscle_cells
SAT_26M	1280.2	909	0.291256	8	Singlet	Mesothelial
SAT_26M	1005.4	687	0.0116801	2	Singlet	FAPs
SAT_26M	4032.8	2110	0.4314786	0	Singlet	Adipocyte
SAT_26M	1683.2	1128	4.4580727	8	Singlet	Mesothelial
SAT_26M	2069.3	1249	1.4459451	0	Singlet	Adipocyte
SAT_26M	2189.2	1282	4.6352702	0	Singlet	Adipocyte
SAT_26M	1357.4	807	5.9657123	7	Singlet	Endothelial_cells
SAT_26M	1587	1007	1.7921312	10	Singlet	Immune_cells
SAT_26M	1362.8	936	0.5837932	5	Singlet	Immune_cells
SAT_26M	1809.1	1221	1.3808851	9	Singlet	Mesothelial
SAT_26M	2456.3	1266	1.4362523	10	Singlet	Immune_cells
SAT_26M	1322.1	890	0.0928627	0	Singlet	Adipocyte
SAT_26M	3304.4	1794	3.7598392	0	Singlet	Adipocyte
SAT_26M	1182.6	759	3.6473251	8	Singlet	Mesothelial
SAT_26M	3096	1623	0.0908517	0	Singlet	Adipocyte
SAT_26M	3756.1	1918	5.3681333	0	Singlet	Adipocyte
SAT_26M	1178.2	738	4.4684408	0	Singlet	Adipocyte
SAT_26M	1447.7	940	2.6388767	3	Singlet	FAPs
SAT_26M	2549.4	1518	3.5968987	0	Singlet	Adipocyte
SAT_26M	1140.6	808	0.2099507	8	Singlet	Mesothelial
SAT_26M	1522.1	875	11.062733	0	Singlet	Adipocyte
SAT_26M	1562.5	1073	6.9029895	1	Singlet	Immune_cells
SAT_26M	1908.7	1125	4.8321537	0	Singlet	Adipocyte
SAT_26M	1084.4	778	0.3054429	9	Singlet	Mesothelial
SAT_26M	2380.1	1369	0.3885175	0	Singlet	Adipocyte
SAT_26M	1104.9	788	0.5509358	11	Singlet	Smooth_muscle_cells
SAT_26M	2621.8	1465	0.0844987	0	Singlet	Adipocyte
SAT_26M	3618.2	2054	7.29741	9	Singlet	Mesothelial
SAT_26M	1116.7	556	12.338656	5	Singlet	Immune_cells
SAT_26M	1514.2	1125	0.2080475	4	Singlet	Immune_cells
SAT_26M	1845.4	1091	7.3397354	0	Singlet	Adipocyte
SAT_26M	1858.4	1211	2.8924926	7	Singlet	Endothelial_cells
SAT_26M	2596.7	1472	2.9438893	0	Singlet	Adipocyte

SAT_26M	1813.9	1120	2.628191	8	Singlet	Mesothelial
SAT_26M	1305.9	860	5.2084693	1	Singlet	Immune_cells
SAT_26M	1453.5	968	2.0334537	2	Singlet	FAPs
SAT_26M	1542.3	1007	0	8	Singlet	Mesothelial
SAT_26M	3173.5	1714	4.6852746	0	Singlet	Adipocyte
SAT_26M	1494.6	979	0.1259388	5	Singlet	Immune_cells
SAT_26M	1292.2	743	6.5957433	10	Singlet	Immune_cells
SAT_26M	1571.1	1020	6.8007668	5	Singlet	Immune_cells
SAT_26M	1751	1094	0.1145278	8	Singlet	Mesothelial
SAT_26M	1158.5	792	1.332494	5	Singlet	Immune_cells
SAT_26M	5929.1	2726	1.8736055	0	Singlet	Adipocyte
SAT_26M	2019.1	1139	0.5431788	10	Singlet	Immune_cells
SAT_26M	1198.3	818	1.3468504	5	Singlet	Immune_cells
SAT_26M	1034.5	565	6.5561072	10	Singlet	Immune_cells
SAT_26M	1552.1	872	14.958966	9	Singlet	Mesothelial
SAT_26M	1259.5	805	4.0187731	3	Singlet	FAPs
SAT_26M	1779.7	1244	2.20169	5	Singlet	Immune_cells
SAT_26M	2788.8	1512	2.8305245	0	Singlet	Adipocyte
SAT_26M	1601.8	952	5.3912733	0	Singlet	Adipocyte
SAT_26M	1903.1	1222	0.079856	0	Singlet	Adipocyte
SAT_26M	2250	1201	10.847263	7	Singlet	Endothelial_cells
SAT_26M	3573.8	1860	2.7766351	0	Singlet	Adipocyte
SAT_26M	2634.3	1390	6.1203119	0	Singlet	Adipocyte
SAT_26M	1348	756	8.2682008	7	Singlet	Endothelial_cells
SAT_26M	1043.4	776	1.6009868	1	Singlet	Immune_cells
SAT_26M	1159.4	852	2.0598231	1	Singlet	Immune_cells
SAT_26M	1576.8	961	13.679652	7	Singlet	Endothelial_cells
SAT_26M	1160	752	0.2294811	9	Singlet	Mesothelial
SAT_26M	3370.8	1949	9.7266661	9	Singlet	Mesothelial
SAT_26M	1546.2	842	9.1955639	7	Singlet	Endothelial_cells
SAT_26M	1363.4	857	4.1779623	0	Singlet	Adipocyte
SAT_26M	1430.8	947	1.7110029	7	Singlet	Endothelial_cells
SAT_26M	1769	1076	0.8179898	0	Singlet	Adipocyte
SAT_26M	1080.6	793	0.7072914	5	Singlet	Immune_cells
SAT_26M	2778.6	1151	11.187285	0	Singlet	Adipocyte
SAT_26M	1435	912	3.4348112	0	Singlet	Adipocyte
SAT_26M	1903.6	1154	0.018782	0	Singlet	Adipocyte
SAT_26M	1002.2	644	6.9011003	0	Singlet	Adipocyte
SAT_26M	1666.7	1060	1.7683496	4	Singlet	Immune_cells
SAT_26M	3006.2	1830	1.3417782	8	Singlet	Mesothelial
SAT_26M	1572	833	9.8023207	0	Singlet	Adipocyte
SAT_26M	1169.4	774	1.5851265	0	Singlet	Adipocyte
SAT_26M	2754.3	1553	0.1516742	0	Singlet	Adipocyte
SAT_26M	1736.8	1049	0.5700833	0	Singlet	Adipocyte
SAT_26M	1680.8	1037	2.1379754	7	Singlet	Endothelial_cells
SAT_26M	1491.8	909	1.0744021	7	Singlet	Endothelial_cells
SAT_26M	1722.7	1046	0.8919314	0	Singlet	Adipocyte
SAT_26M	1104.3	782	0	0	Singlet	Adipocyte
SAT_26M	1247.6	857	8.5302983	0	Singlet	Adipocyte
SAT_26M	1352.9	823	2.4016652	0	Singlet	Adipocyte
SAT_26M	2391.1	1285	5.5958211	0	Singlet	Adipocyte

SAT_26M	2179.3	1271	0.0464294	0	Singlet	Adipocyte
SAT_26M	3865.4	1969	9.4849445	0	Singlet	Adipocyte
SAT_26M	2207.6	1271	0.0147949	0	Singlet	Adipocyte
SAT_26M	1532.2	975	10.38976	3	Singlet	FAPs
SAT_26M	2181.9	1348	0.2647249	0	Singlet	Adipocyte
SAT_26M	2268.2	1262	2.4690221	10	Singlet	Immune_cells
SAT_26M	1321.5	890	8.0047063	1	Singlet	Immune_cells
SAT_26M	5499.2	2620	2.8098088	0	Singlet	Adipocyte
SAT_26M	1568.4	1051	0	5	Singlet	Immune_cells
SAT_26M	1188.3	787	1.0729511	3	Singlet	FAPs
SAT_26M	2641.9	1609	0	8	Singlet	Mesothelial
SAT_26M	1918.7	1280	1.3656596	8	Singlet	Mesothelial
SAT_26M	1314.6	883	12.27156	1	Singlet	Immune_cells
SAT_26M	1138.3	719	2.9730752	0	Singlet	Adipocyte
SAT_26M	1367	924	0.0001485	8	Singlet	Mesothelial
SAT_26M	1655.4	856	4.908055	0	Singlet	Adipocyte
SAT_26M	2848.7	1601	0.3194058	0	Singlet	Adipocyte
SAT_26M	2013.2	1200	0.2390594	3	Singlet	FAPs
SAT_26M	1002.1	687	1.2922078	3	Singlet	FAPs
SAT_26M	1892.4	1258	0	8	Singlet	Mesothelial
SAT_26M	1287	825	5.0667842	0	Singlet	Adipocyte
SAT_26M	1742.8	1002	3.6643773	0	Singlet	Adipocyte
SAT_26M	2765.8	1549	0.1647694	0	Singlet	Adipocyte
SAT_26M	1535.2	876	11.664844	7	Singlet	Endothelial_cells
SAT_26M	1229.9	773	6.7041407	0	Singlet	Adipocyte
SAT_26M	1401.5	860	0.0484009	7	Singlet	Endothelial_cells
SAT_26M	1187.6	805	0.3205047	6	Singlet	Neurons
SAT_26M	1027.8	737	0.1284523	6	Singlet	Neurons
SAT_26M	1206.8	660	7.7343238	0	Singlet	Adipocyte
SAT_26M	2048.6	1391	0.1340288	11	Singlet	Smooth_muscle_cells
SAT_26M	1018.5	744	0.2247614	8	Singlet	Mesothelial
SAT_26M	2437.1	1350	0.091172	0	Singlet	Adipocyte
SAT_26M	3621.4	1857	3.0687383	0	Singlet	Adipocyte
SAT_26M	1007	692	0.2691038	8	Singlet	Mesothelial
SAT_26M	1828.6	1077	2.757046	2	Singlet	FAPs
SAT_26M	1177.5	762	6.2502917	0	Singlet	Adipocyte
SAT_26M	4912.3	2169	0	0	Singlet	Adipocyte
SAT_26M	1234.7	748	2.0884062	10	Singlet	Immune_cells
SAT_26M	1762	1096	0.2420943	5	Singlet	Immune_cells
SAT_26M	3590	2025	2.208753	0	Singlet	Adipocyte
SAT_26M	1617.9	989	0.5857038	0	Singlet	Adipocyte
SAT_26M	2554.3	1395	0.1195325	0	Singlet	Adipocyte
SAT_26M	1087	734	0.055598	5	Singlet	Immune_cells
SAT_26M	2141.4	1199	0	0	Singlet	Adipocyte
SAT_26M	1094.9	583	8.8337115	0	Singlet	Adipocyte
SAT_26M	1884.6	1240	0.8758068	8	Singlet	Mesothelial
SAT_26M	1835.5	1198	3.5094366	9	Singlet	Mesothelial
SAT_26M	2345.8	1345	3.6121697	7	Singlet	Endothelial_cells
SAT_26M	2585.8	1455	0.0080168	0	Singlet	Adipocyte
SAT_26M	1515.3	915	7.9495196	7	Singlet	Endothelial_cells
SAT_26M	1753	1072	0.6648782	0	Singlet	Adipocyte

SAT_26M	1423.6	858	3.2247752	0	Singlet	Adipocyte
SAT_26M	1977.2	1159	2.1490982	0	Singlet	Adipocyte
SAT_26M	1413.4	914	0.0529458	0	Singlet	Adipocyte
SAT_26M	1040.8	711	0.1069615	0	Singlet	Adipocyte
SAT_26M	1134.9	725	0.2881581	0	Singlet	Adipocyte
SAT_26M	1377.7	874	0.5466752	0	Singlet	Adipocyte
SAT_26M	3549.2	1883	0.279504	0	Singlet	Adipocyte
SAT_26M	2072.8	1296	3.9663965	1	Singlet	Immune_cells
SAT_26M	1540.1	995	0	0	Singlet	Adipocyte
SAT_26M	1060.7	783	1.0410972	1	Singlet	Immune_cells
SAT_26M	1087.9	771	3.2166275	1	Singlet	Immune_cells
SAT_26M	1237.4	750	0.4971872	0	Singlet	Adipocyte
SAT_26M	5382.1	2115	8.5643384	7	Singlet	Endothelial_cells
SAT_26M	1573.5	780	9.8654486	7	Singlet	Endothelial_cells
SAT_26M	1960.3	1284	0.0050861	8	Singlet	Mesothelial
SAT_26M	1849.1	1134	6.142147	0	Singlet	Adipocyte
SAT_26M	3151	1943	6.0996056	8	Singlet	Mesothelial
SAT_26M	2319.9	1409	0.9073691	7	Singlet	Endothelial_cells
SAT_26M	1091.6	676	13.000315	0	Singlet	Adipocyte
SAT_26M	1240.1	790	2.0426147	0	Singlet	Adipocyte
SAT_26M	1318.3	577	12.293166	0	Singlet	Adipocyte
SAT_26M	1630.9	1013	0.0309082	0	Singlet	Adipocyte
SAT_26M	1238.4	757	1.6187555	0	Singlet	Adipocyte
SAT_26M	1056.8	674	0.7453685	3	Singlet	FAPs
SAT_26M	2522.5	1285	11.35465	0	Singlet	Adipocyte
SAT_26M	1810.9	1273	0.256765	1	Singlet	Immune_cells
SAT_26M	1535.5	996	1.6672661	0	Singlet	Adipocyte
SAT_26M	2993.2	1653	0.6535154	0	Singlet	Adipocyte
SAT_26M	2097.3	1240	3.8590181	0	Singlet	Adipocyte
SAT_26M	1090.2	673	1.6330613	7	Singlet	Endothelial_cells
SAT_26M	2274	1312	3.486947	10	Singlet	Immune_cells
SAT_26M	1569.7	922	3.9727498	7	Singlet	Endothelial_cells
SAT_26M	1637.7	1057	2.5211881	0	Singlet	Adipocyte
SAT_26M	1291.1	806	0.870165	5	Singlet	Immune_cells
SAT_26M	1156.9	810	1.1729006	3	Singlet	FAPs
SAT_26M	1140.4	734	0.6694433	10	Singlet	Immune_cells
SAT_26M	1598.7	1143	2.6244255	4	Singlet	Immune_cells
SAT_26M	1584.7	1086	0.1909917	8	Singlet	Mesothelial
SAT_26M	1128.4	805	2.5988769	8	Singlet	Mesothelial
SAT_26M	2759.7	1308	5.4491086	7	Singlet	Endothelial_cells
SAT_26M	1349.7	838	0.8028245	3	Singlet	FAPs
SAT_26M	2487.6	1418	0.1940067	0	Singlet	Adipocyte
SAT_26M	1152.9	699	2.2240576	10	Singlet	Immune_cells
SAT_26M	1754.6	1124	1.2023124	0	Singlet	Adipocyte
SAT_26M	2627.7	1254	13.744022	0	Singlet	Adipocyte
SAT_26M	4048	1860	0.3844515	10	Singlet	Immune_cells
SAT_26M	1066.9	746	0.0296362	0	Singlet	Adipocyte
SAT_26M	2847.7	1412	14.29773	9	Singlet	Mesothelial
SAT_26M	3256	1816	0.286003	0	Singlet	Adipocyte
SAT_26M	1883	1094	3.7161116	7	Singlet	Endothelial_cells
SAT_26M	2291.4	1348	3.7264058	0	Singlet	Adipocyte

SAT_26M	2343.9	1351	2.2301504	0	Singlet	Adipocyte
SAT_26M	1701.2	954	11.376275	7	Singlet	Endothelial_cells
SAT_26M	1215.5	771	7.5218849	7	Singlet	Endothelial_cells
SAT_26M	4127.4	2101	0	0	Singlet	Adipocyte
SAT_26M	1667.9	1060	0	8	Singlet	Mesothelial
SAT_26M	2839.7	1577	0.1007713	0	Singlet	Adipocyte
SAT_26M	1443.7	927	0.446584	0	Singlet	Adipocyte
SAT_26M	2674.6	1494	1.2941513	0	Singlet	Adipocyte
SAT_26M	2109.3	1046	8.1026936	0	Singlet	Adipocyte
SAT_26M	1048.9	769	1.2287331	1	Singlet	Immune_cells
SAT_26M	1337.8	848	0.6454223	10	Singlet	Immune_cells
SAT_26M	1135.1	756	0.0661509	0	Singlet	Adipocyte
SAT_26M	1288.2	865	0.2007931	3	Singlet	FAPs
SAT_26M	1172.3	821	1.1647565	5	Singlet	Immune_cells
SAT_26M	2180.2	1230	0.1394238	0	Singlet	Adipocyte
SAT_26M	1018	733	0	5	Singlet	Immune_cells
SAT_26M	1075	710	1.6332219	0	Singlet	Adipocyte
SAT_26M	3003.8	1529	1.4072766	0	Singlet	Adipocyte
SAT_26M	1670	1075	1.8726916	0	Singlet	Adipocyte
SAT_26M	1887.4	1136	0.0745972	8	Singlet	Mesothelial
SAT_26M	1730.3	955	10.267745	0	Singlet	Adipocyte
SAT_26M	1808.5	1098	0.3266382	0	Singlet	Adipocyte
SAT_26M	5258	2721	0.7450587	8	Singlet	Mesothelial
SAT_26M	1369.6	984	0	8	Singlet	Mesothelial
SAT_26M	1185.2	557	14.149923	0	Singlet	Adipocyte
SAT_26M	1633.8	975	1.0496128	3	Singlet	FAPs
SAT_26M	1022.7	698	2.6812362	0	Singlet	Adipocyte
SAT_26M	2377.2	1361	1.734441	0	Singlet	Adipocyte
SAT_26M	1004.8	715	0	0	Singlet	Adipocyte
SAT_26M	3414	1696	1.0040891	0	Singlet	Adipocyte
SAT_26M	2499.5	1474	0.8453898	0	Singlet	Adipocyte
SAT_26M	2305.1	1107	12.153431	7	Singlet	Endothelial_cells
SAT_26M	1005.2	724	0.6470615	0	Singlet	Adipocyte
SAT_26M	1392.9	871	2.0123237	0	Singlet	Adipocyte
SAT_26M	1499.6	964	5.3434046	9	Singlet	Mesothelial
SAT_26M	1226.2	839	2.1633686	0	Singlet	Adipocyte
SAT_26M	3671.6	2000	0.0185817	0	Singlet	Adipocyte
SAT_26M	2101.5	1260	0	0	Singlet	Adipocyte
SAT_26M	1763.4	1018	2.3839673	0	Singlet	Adipocyte
SAT_26M	1025.1	693	0.6607165	0	Singlet	Adipocyte
SAT_26M	1998.9	1049	5.4492709	10	Singlet	Immune_cells
SAT_26M	1491.3	989	0.1539335	0	Singlet	Adipocyte
SAT_26M	1000.9	654	0.0514382	6	Singlet	Neurons
SAT_26M	4284.2	2053	0	0	Singlet	Adipocyte
SAT_26M	1872.8	1121	0.0511596	0	Singlet	Adipocyte
SAT_26M	4796.6	2498	6.7864664	9	Singlet	Mesothelial
SAT_26M	1939.8	1075	0.8944918	0	Singlet	Adipocyte
SAT_26M	1962.4	1193	0.0242224	0	Singlet	Adipocyte
SAT_26M	2105.2	1136	7.101146	7	Singlet	Endothelial_cells
SAT_26M	1020.8	728	1.5629245	0	Singlet	Adipocyte
SAT_26M	1427.7	882	5.7461509	7	Singlet	Endothelial_cells

SAT_26M	1104	732	8.8639683	0	Singlet	Adipocyte
SAT_26M	3303.5	1562	9.532951	7	Singlet	Endothelial_cells
SAT_26M	2335.2	1324	7.7906714	0	Singlet	Adipocyte
SAT_26M	1911.8	1260	0	9	Singlet	Mesothelial
SAT_26M	1442.1	994	0.3690749	0	Singlet	Adipocyte
SAT_26M	5380.6	2429	0.688241	0	Singlet	Adipocyte
SAT_26M	2944.8	1746	2.8778342	8	Singlet	Mesothelial
SAT_26M	2368.2	1415	0.4669202	0	Singlet	Adipocyte
SAT_26M	2289.3	1264	1.1494094	0	Singlet	Adipocyte
SAT_26M	1167.2	741	4.3324939	0	Singlet	Adipocyte
SAT_26M	1027.3	732	3.5423357	8	Singlet	Mesothelial
SAT_26M	1455.8	909	2.0872626	0	Singlet	Adipocyte
SAT_26M	2505	1384	8.6000475	0	Singlet	Adipocyte
SAT_26M	1278.9	780	5.1825041	0	Singlet	Adipocyte
SAT_26M	3152.1	1594	2.5317878	0	Singlet	Adipocyte
SAT_26M	1372.8	728	8.454837	0	Singlet	Adipocyte
SAT_26M	1135.3	739	3.5569602	8	Singlet	Mesothelial
SAT_26M	1007.9	729	0.7015175	5	Singlet	Immune_cells
SAT_26M	1566.3	990	3.8863357	7	Singlet	Endothelial_cells
SAT_26M	1314.1	912	0.2643581	0	Singlet	Adipocyte
SAT_26M	2308.1	1375	0.4944659	0	Singlet	Adipocyte
SAT_26M	1152.1	708	0.8689092	3	Singlet	FAPs
SAT_26M	1219	837	0.0074748	0	Singlet	Adipocyte
SAT_26M	1028.6	731	0	1	Singlet	Immune_cells
SAT_26M	1083.6	723	2.4709614	9	Singlet	Mesothelial
SAT_26M	1039.8	753	0	8	Singlet	Mesothelial
SAT_26M	1299.5	867	5.3309164	0	Singlet	Adipocyte
SAT_26M	2173.3	1184	0.0912863	0	Singlet	Adipocyte
SAT_26M	1622.6	869	4.1670162	10	Singlet	Immune_cells
SAT_26M	1559.4	908	7.9071685	0	Singlet	Adipocyte
SAT_26M	2396.4	1442	6.3358513	8	Singlet	Mesothelial
SAT_26M	1983.8	1004	8.1322146	0	Singlet	Adipocyte
SAT_26M	1814.3	910	12.242999	0	Singlet	Adipocyte
SAT_26M	4417.2	2285	8.5875996	9	Singlet	Mesothelial
SAT_26M	3317.6	1728	0	0	Singlet	Adipocyte
SAT_26M	1144.8	781	0.213149	3	Singlet	FAPs
SAT_26M	1357.6	673	11.555749	7	Singlet	Endothelial_cells
SAT_26M	1979.6	1248	7.4277477	4	Singlet	Immune_cells
SAT_26M	1936	1186	0.0031001	0	Singlet	Adipocyte
SAT_26M	2561	1456	4.0473193	0	Singlet	Adipocyte
SAT_26M	1657.2	1078	0.0558102	0	Singlet	Adipocyte
SAT_26M	1912.7	1129	1.5239769	0	Singlet	Adipocyte
SAT_26M	1495.6	939	0.5222743	0	Singlet	Adipocyte
SAT_26M	1591.8	792	11.600722	7	Singlet	Endothelial_cells
SAT_26M	3990.9	1924	7.9756514	7	Singlet	Endothelial_cells
SAT_26M	4296.9	1992	0.9861532	0	Singlet	Adipocyte
SAT_26M	1636.3	1018	1.1399055	3	Singlet	FAPs
SAT_26M	1719.4	1066	0.3760636	0	Singlet	Adipocyte
SAT_26M	1023	757	6.6035785	1	Singlet	Immune_cells
SAT_26M	1026.6	718	0.1252479	0	Singlet	Adipocyte
SAT_26M	2050.2	1040	13.336615	7	Singlet	Endothelial_cells

SAT_26M	1211.9	791	0.2094377	7	Singlet	Endothelial_cells
SAT_26M	1266.2	844	3.7472115	5	Singlet	Immune_cells
SAT_26M	1304.1	760	2.8406048	3	Singlet	FAPs
SAT_26M	2563.2	1436	10.585974	7	Singlet	Endothelial_cells
SAT_26M	1328.1	834	6.2057002	5	Singlet	Immune_cells
SAT_26M	1033.3	711	0.0180677	7	Singlet	Endothelial_cells
SAT_26M	1109.7	733	2.7814602	0	Singlet	Adipocyte
SAT_26M	1149.3	747	7.984481	1	Singlet	Immune_cells
SAT_26M	1078.1	776	1.2517452	8	Singlet	Mesothelial
SAT_26M	1240.1	883	0.3085681	0	Singlet	Adipocyte
SAT_26M	1567	983	4.9946648	7	Singlet	Endothelial_cells
SAT_26M	3305.1	1444	8.4529447	7	Singlet	Endothelial_cells
SAT_26M	2517.9	1366	6.6868505	0	Singlet	Adipocyte
SAT_26M	2100.1	1326	3.6887321	4	Singlet	Immune_cells
SAT_26M	1169.8	794	0.0612905	0	Singlet	Adipocyte
SAT_26M	2013.3	1170	0.3139758	0	Singlet	Adipocyte
SAT_26M	1028.4	673	0.0252451	8	Singlet	Mesothelial
SAT_26M	3074.3	1873	3.0866359	8	Singlet	Mesothelial
SAT_26M	1481.8	918	0.523882	0	Singlet	Adipocyte
SAT_26M	2635.4	1565	1.504632	5	Singlet	Immune_cells
SAT_26M	2341.2	1127	1.0046971	10	Singlet	Immune_cells
SAT_26M	1428.6	936	0.0232812	7	Singlet	Endothelial_cells
SAT_26M	1908.8	1124	4.2351099	7	Singlet	Endothelial_cells
SAT_26M	1156.2	691	2.4208043	7	Singlet	Endothelial_cells
SAT_26M	3299.8	1118	14.573149	0	Singlet	Adipocyte
SAT_26M	1997	1149	1.3631058	0	Singlet	Adipocyte
SAT_26M	1685.7	1043	1.9406625	5	Singlet	Immune_cells
SAT_26M	6314.1	2840	0.473328	0	Singlet	Adipocyte
SAT_26M	1436.9	973	1.7041376	1	Singlet	Immune_cells
SAT_26M	1153.6	639	11.525786	7	Singlet	Endothelial_cells
SAT_26M	2928.1	1539	0.0832462	5	Singlet	Immune_cells
SAT_26M	1509.7	977	0.0581683	0	Singlet	Adipocyte
SAT_26M	1097.5	744	1.2432605	0	Singlet	Adipocyte
SAT_26M	1441.3	827	5.227268	10	Singlet	Immune_cells
SAT_26M	1208.1	824	0.2494572	0	Singlet	Adipocyte
SAT_26M	1145	618	3.1036877	0	Singlet	Adipocyte
SAT_26M	6320.3	2765	0.028432	0	Singlet	Adipocyte
SAT_26M	1337.5	962	0.4068113	11	Singlet	Smooth_muscle_cells
SAT_26M	4594.9	2187	0.0782135	0	Singlet	Adipocyte
SAT_26M	1481.1	955	4.4316616	0	Singlet	Adipocyte
SAT_26M	1971.8	1208	1.2772673	5	Singlet	Immune_cells
SAT_26M	2237.6	1405	0.0090194	0	Singlet	Adipocyte
SAT_26M	2371.9	1401	0.0160909	0	Singlet	Adipocyte
SAT_26M	1065.2	776	4.0757469	4	Singlet	Immune_cells
SAT_26M	2705	1502	2.632634	0	Singlet	Adipocyte
SAT_26M	1075.9	805	10.260557	1	Singlet	Immune_cells
SAT_26M	2320	1361	0.6291672	0	Singlet	Adipocyte
SAT_26M	2060.8	1221	1.0986007	0	Singlet	Adipocyte
SAT_26M	1246.4	810	1.7607509	7	Singlet	Endothelial_cells
SAT_26M	1134.9	744	1.740579	0	Singlet	Adipocyte
SAT_26M	1613.6	976	3.1747589	5	Singlet	Immune_cells

SAT_26M	1342.9	918	2.1967024	1 Singlet	Immune_cells
SAT_26M	1036.9	764	3.3986149	1 Singlet	Immune_cells
SAT_26M	1696.5	1026	0.0103921	0 Singlet	Adipocyte
SAT_26M	1105.6	739	3.1544122	0 Singlet	Adipocyte
SAT_26M	3116.9	1557	0.5197003	0 Singlet	Adipocyte
SAT_26M	2089.3	1238	2.5585085	7 Singlet	Endothelial_cells
SAT_26M	1760.4	999	6.5292059	0 Singlet	Adipocyte
SAT_26M	1717.8	1103	0.0070611	0 Singlet	Adipocyte
SAT_26M	2849.6	1371	13.897084	7 Singlet	Endothelial_cells
SAT_26M	1358	872	4.5053021	0 Singlet	Adipocyte
SAT_26M	1093.4	807	4.0099823	1 Singlet	Immune_cells
SAT_26M	1328.5	862	0.0939559	0 Singlet	Adipocyte
SAT_26M	2420.7	1457	0	5 Singlet	Immune_cells
SAT_26M	2151.1	1393	0.2590232	5 Singlet	Immune_cells
SAT_26M	1453.6	963	0.0008669	8 Singlet	Mesothelial
SAT_26M	1253.4	792	7.2360917	5 Singlet	Immune_cells
SAT_26M	1345.2	929	2.0721269	0 Singlet	Adipocyte
SAT_26M	1556.1	1083	1.8469916	5 Singlet	Immune_cells
SAT_26M	1212.8	723	6.7362429	7 Singlet	Endothelial_cells
SAT_26M	1273.9	586	12.742332	0 Singlet	Adipocyte
SAT_26M	2031.3	1257	0	8 Singlet	Mesothelial
SAT_26M	2315.3	1398	1.0566994	0 Singlet	Adipocyte
SAT_26M	1443.6	968	4.7766889	8 Singlet	Mesothelial
SAT_26M	1410.4	901	0.2159911	0 Singlet	Adipocyte
SAT_26M	4315	2049	8.0136474	0 Singlet	Adipocyte
SAT_26M	1629.5	929	4.4917182	10 Singlet	Immune_cells
SAT_26M	2944.9	1537	6.8562361	0 Singlet	Adipocyte
SAT_26M	2120.6	1311	0.007521	0 Singlet	Adipocyte
SAT_26M	2425.8	1195	13.785085	7 Singlet	Endothelial_cells
SAT_26M	1371.6	865	12.581439	9 Singlet	Mesothelial
SAT_26M	2411.6	1431	2.8245477	0 Singlet	Adipocyte
SAT_26M	1146.1	851	0.1605238	0 Singlet	Adipocyte
SAT_26M	1227.1	784	1.5886749	3 Singlet	FAPs
SAT_26M	1009.7	677	0.2259772	0 Singlet	Adipocyte
SAT_26M	1212.9	753	1.6604662	3 Singlet	FAPs
SAT_26M	4552.8	2499	9.8147325	8 Singlet	Mesothelial
SAT_26M	2961.7	1423	9.0066546	7 Singlet	Endothelial_cells
SAT_26M	2952.5	1647	4.7871383	0 Singlet	Adipocyte
SAT_26M	1388	896	1.8351789	7 Singlet	Endothelial_cells
SAT_26M	1883.2	1165	2.9521309	0 Singlet	Adipocyte
SAT_26M	1408.3	906	0	0 Singlet	Adipocyte
SAT_26M	1089.7	718	4.2880475	7 Singlet	Endothelial_cells
SAT_26M	3915.6	2035	0.2565418	0 Singlet	Adipocyte
SAT_26M	1085.1	714	1.5883374	0 Singlet	Adipocyte
SAT_26M	1532.6	976	3.4323863	0 Singlet	Adipocyte
SAT_26M	7291.9	3194	13.94436	9 Singlet	Mesothelial
SAT_26M	1082.3	816	2.2554793	1 Singlet	Immune_cells
SAT_26M	1591.1	1088	4.4581254	4 Singlet	Immune_cells
SAT_26M	1692.9	1075	3.1231798	7 Singlet	Endothelial_cells
SAT_26M	1935.3	1253	0.1407391	0 Singlet	Adipocyte
SAT_26M	4373.6	1989	8.4343034	7 Singlet	Endothelial_cells

SAT_26M	1943.7	1071	1.7235053	0	Singlet	Adipocyte
SAT_26M	3675.3	1950	1.9383168	10	Singlet	Immune_cells
SAT_26M	3130.3	1673	14.389827	9	Singlet	Mesothelial
SAT_26M	3461.8	1879	0.2267065	0	Singlet	Adipocyte
SAT_26M	1165.9	766	0	0	Singlet	Adipocyte
SAT_26M	1429.5	978	0.1751367	0	Singlet	Adipocyte
SAT_26M	2922.2	1689	1.0306575	0	Singlet	Adipocyte
SAT_26M	1157.2	738	0.2105671	0	Singlet	Adipocyte
SAT_26M	1090.4	710	0.2119845	8	Singlet	Mesothelial
SAT_26M	1297.2	669	11.811694	7	Singlet	Endothelial_cells
SAT_26M	3010.7	1633	1.869325	0	Singlet	Adipocyte
SAT_26M	1297.3	868	0.4308279	3	Singlet	FAPs
SAT_26M	1827.4	1174	4.3097158	7	Singlet	Endothelial_cells
SAT_26M	1021.7	714	4.8373322	1	Singlet	Immune_cells
SAT_26M	1226.5	785	0.2914744	0	Singlet	Adipocyte
SAT_26M	3011.5	868	2.7837099	7	Singlet	Endothelial_cells
SAT_26M	4454	2236	1.6110344	0	Singlet	Adipocyte
SAT_26M	1102.3	773	0.5130029	8	Singlet	Mesothelial
SAT_26M	1265.8	886	1.2687813	1	Singlet	Immune_cells
SAT_26M	3352.8	1788	0.0938373	0	Singlet	Adipocyte
SAT_26M	1748	1112	2.7470513	0	Singlet	Adipocyte
SAT_26M	1897.4	1253	3.2553118	4	Singlet	Immune_cells
SAT_26M	3516.5	1780	2.9740077	0	Singlet	Adipocyte
SAT_26M	1397.5	879	0.5504915	0	Singlet	Adipocyte
SAT_26M	5307.5	2473	3.498526	0	Singlet	Adipocyte
SAT_26M	1119.6	791	3.4701505	0	Singlet	Adipocyte
SAT_26M	1375.6	916	1.8233033	0	Singlet	Adipocyte
SAT_26M	1466.1	912	3.1850688	0	Singlet	Adipocyte
SAT_26M	1495.8	866	6.3152388	8	Singlet	Mesothelial
SAT_26M	1274.4	802	1.0438289	0	Singlet	Adipocyte
SAT_26M	4735.8	2334	0	0	Singlet	Adipocyte
SAT_26M	1210.4	764	2.3498586	5	Singlet	Immune_cells
SAT_26M	3596.4	1684	0.7119917	0	Singlet	Adipocyte
SAT_26M	1247.8	847	0.5300924	0	Singlet	Adipocyte
SAT_26M	1044	717	6.1031373	4	Singlet	Immune_cells
SAT_26M	1103.2	716	2.2464031	7	Singlet	Endothelial_cells
SAT_26M	1377.7	861	0.5595088	0	Singlet	Adipocyte
SAT_26M	2315.1	1300	2.6905804	0	Singlet	Adipocyte
SAT_26M	2634.4	1410	1.1927957	0	Singlet	Adipocyte
SAT_26M	1006.2	741	0.6166857	6	Singlet	Neurons
SAT_26M	5074	2490	3.2380888	0	Singlet	Adipocyte
SAT_26M	1675.5	1019	0	0	Singlet	Adipocyte
SAT_26M	2350.1	1381	0	5	Singlet	Immune_cells
SAT_26M	1987.5	1093	9.4200352	0	Singlet	Adipocyte
SAT_26M	1578.5	972	2.4175733	5	Singlet	Immune_cells
SAT_26M	1107.6	768	0.1588136	0	Singlet	Adipocyte
SAT_26M	1045.3	564	12.191571	5	Singlet	Immune_cells
SAT_26M	1425.9	839	0.59653	0	Singlet	Adipocyte
SAT_26M	4131.6	1747	7.5380181	0	Singlet	Adipocyte
SAT_26M	4147.1	1893	3.0125171	10	Singlet	Immune_cells
SAT_26M	2648.2	1031	14.849397	0	Singlet	Adipocyte

SAT_26M	2670.3	1591	1.842309	0	Singlet	Adipocyte
SAT_26M	1791.5	1079	0	0	Singlet	Adipocyte
SAT_26M	1383.6	783	6.9935703	0	Singlet	Adipocyte
SAT_26M	1610.6	977	3.4438835	0	Singlet	Adipocyte
SAT_26M	2659.6	1536	3.3153568	0	Singlet	Adipocyte
SAT_26M	1767.6	1065	11.208628	7	Singlet	Endothelial_cells
SAT_26M	1621.8	1021	4.5058533	7	Singlet	Endothelial_cells
SAT_26M	2897.6	1692	0.1034888	0	Singlet	Adipocyte
SAT_26M	1019.3	728	0.1249319	6	Singlet	Neurons
SAT_26M	1101.9	756	2.3219972	8	Singlet	Mesothelial
SAT_26M	2557.6	1576	0.6162149	0	Singlet	Adipocyte
SAT_26M	1300.7	826	1.2293806	7	Singlet	Endothelial_cells
SAT_26M	4910.3	2181	6.0894767	0	Singlet	Adipocyte
SAT_26M	3540.2	1868	0.5103762	0	Singlet	Adipocyte
SAT_26M	1207.9	848	2.8335779	1	Singlet	Immune_cells
SAT_26M	1353.6	799	3.9214999	0	Singlet	Adipocyte
SAT_26M	2030.8	1204	4.2656632	0	Singlet	Adipocyte
SAT_26M	1346.4	853	1.1308119	0	Singlet	Adipocyte
SAT_26M	1718.2	1106	0.1566696	0	Singlet	Adipocyte
SAT_26M	1375.3	888	4.6126575	0	Singlet	Adipocyte
SAT_26M	1064.3	719	10.534577	1	Singlet	Immune_cells
SAT_26M	2548.9	1440	0.2218908	0	Singlet	Adipocyte
SAT_26M	3331	1739	0.1250594	0	Singlet	Adipocyte
SAT_26M	1868	1155	0.4607054	7	Singlet	Endothelial_cells
SAT_26M	1777.5	1016	3.6295393	0	Singlet	Adipocyte
SAT_26M	2035	1173	12.018915	9	Singlet	Mesothelial
SAT_26M	1161.7	795	0.0114252	0	Singlet	Adipocyte
SAT_26M	1139.9	728	2.3258903	0	Singlet	Adipocyte
SAT_26M	1337.3	877	5.327521	8	Singlet	Mesothelial
SAT_26M	1091.9	799	1.1128615	0	Singlet	Adipocyte
SAT_26M	2396.9	1360	0.3683364	0	Singlet	Adipocyte
SAT_26M	1494.5	908	9.5706938	7	Singlet	Endothelial_cells
SAT_26M	1798.1	1175	0.084654	0	Singlet	Adipocyte
SAT_26M	1037	717	0.2979506	6	Singlet	Neurons
SAT_26M	2010.1	1143	3.9410719	0	Singlet	Adipocyte
SAT_26M	1011.5	663	1.3512182	10	Singlet	Immune_cells
SAT_26M	1706.1	1106	0.6006485	3	Singlet	FAPs
SAT_26M	2030.7	1021	11.943947	7	Singlet	Endothelial_cells
SAT_26M	1873.9	1168	4.8300031	7	Singlet	Endothelial_cells
SAT_26M	1443	988	2.5750297	8	Singlet	Mesothelial
SAT_26M	1549	1042	2.497601	1	Singlet	Immune_cells
SAT_26M	2094.4	1278	0.446296	0	Singlet	Adipocyte
SAT_26M	3132.7	1692	0.0831656	0	Singlet	Adipocyte
SAT_26M	1537	917	1.3451055	0	Singlet	Adipocyte
SAT_26M	1378.4	898	3.1141218	7	Singlet	Endothelial_cells
SAT_26M	1038.3	701	0.2990472	6	Singlet	Neurons
SAT_26M	3328.4	1727	1.0676634	0	Singlet	Adipocyte
SAT_26M	1475.9	980	0.712638	0	Singlet	Adipocyte
SAT_26M	1913.1	1130	3.460239	7	Singlet	Endothelial_cells
SAT_26M	1030.7	734	0.0468445	0	Singlet	Adipocyte
SAT_26M	4885.2	2495	1.1245625	8	Singlet	Mesothelial

SAT_26M	1256.2	807	0.1823768	0	Singlet	Adipocyte
SAT_26M	1617.2	963	0.7646047	0	Singlet	Adipocyte
SAT_26M	1760	985	3.4383925	7	Singlet	Endothelial_cells
SAT_26M	1149.9	845	3.3955071	1	Singlet	Immune_cells
SAT_26M	1316.4	903	7.3731087	4	Singlet	Immune_cells
SAT_26M	2057.2	1211	8.8469763	4	Singlet	Immune_cells
SAT_26M	2126.9	1090	9.8120739	7	Singlet	Endothelial_cells
SAT_26M	1492.1	959	0.0836141	6	Singlet	Neurons
SAT_26M	1875	1102	0.9298712	5	Singlet	Immune_cells
SAT_26M	3349.2	1813	0.0326683	0	Singlet	Adipocyte
SAT_26M	1119	756	10.152996	0	Singlet	Adipocyte
SAT_26M	1815.2	1163	10.433058	4	Singlet	Immune_cells
SAT_26M	8471.8	3440	5.1018008	9	Singlet	Mesothelial
SAT_26M	4241.4	1999	0.4910894	0	Singlet	Adipocyte
SAT_26M	1330.6	837	1.6329093	7	Singlet	Endothelial_cells
SAT_26M	1013.1	674	0.025453	0	Singlet	Adipocyte
SAT_26M	1134.4	734	0.2232517	0	Singlet	Adipocyte
SAT_26M	1567.1	1028	2.2232051	4	Singlet	Immune_cells
SAT_26M	1403.4	807	6.8279239	0	Singlet	Adipocyte
SAT_26M	1129.2	742	0.1210469	7	Singlet	Endothelial_cells
SAT_26M	1229.7	858	1.4324462	10	Singlet	Immune_cells
SAT_26M	1165	752	2.053972	0	Singlet	Adipocyte
SAT_26M	1033.1	742	0	0	Singlet	Adipocyte
SAT_26M	2713.9	1520	1.1327165	7	Singlet	Endothelial_cells
SAT_26M	1435.2	927	4.8745928	0	Singlet	Adipocyte
SAT_26M	5737.4	2483	0.2246143	0	Singlet	Adipocyte
SAT_26M	5682.9	2886	1.3959423	9	Singlet	Mesothelial
SAT_26M	1072	666	4.1932009	0	Singlet	Adipocyte
SAT_26M	2513.6	1503	3.1304636	0	Singlet	Adipocyte
SAT_26M	2093.8	1240	0.0525866	0	Singlet	Adipocyte
SAT_26M	1960.1	1177	3.7767118	7	Singlet	Endothelial_cells
SAT_26M	1287	908	2.6793131	4	Singlet	Immune_cells
SAT_26M	2928.3	1636	3.4893528	0	Singlet	Adipocyte
SAT_26M	1005.3	702	0	8	Singlet	Mesothelial
SAT_26M	1717.5	967	6.4181223	0	Singlet	Adipocyte
SAT_26M	1376.5	945	2.5379588	5	Singlet	Immune_cells
SAT_26M	2483.9	1459	0.1440461	0	Singlet	Adipocyte
SAT_26M	7121	3283	8.0655969	9	Singlet	Mesothelial
SAT_26M	1443.1	716	8.9394423	3	Singlet	FAPs
SAT_26M	1120.1	767	1.1850625	6	Singlet	Neurons
SAT_26M	2786.1	1565	0.0302269	0	Singlet	Adipocyte
SAT_26M	1091.3	753	0.2039497	9	Singlet	Mesothelial
SAT_26M	1687.6	1151	1.8246294	4	Singlet	Immune_cells
SAT_26M	1029.4	719	0.278648	0	Singlet	Adipocyte
SAT_26M	1307.4	774	11.937527	8	Singlet	Mesothelial
SAT_26M	3017.5	1662	2.1615937	0	Singlet	Adipocyte
SAT_26M	1882.9	1209	6.0647317	9	Singlet	Mesothelial
SAT_26M	1142.4	725	0.2173799	6	Singlet	Neurons
SAT_26M	1513.3	948	1.7673264	0	Singlet	Adipocyte
SAT_26M	1239.7	796	6.4839887	7	Singlet	Endothelial_cells
SAT_26M	1767.9	1120	3.2364986	0	Singlet	Adipocyte

SAT_26M	1443.9	888	2.9827344	7	Singlet	Endothelial_cells
SAT_26M	1911	1087	0.0553282	10	Singlet	Immune_cells
SAT_26M	1934.9	1185	6.3151918	0	Singlet	Adipocyte
SAT_26M	1166.3	765	3.7545444	0	Singlet	Adipocyte
SAT_26M	1260.4	853	0.073313	0	Singlet	Adipocyte
SAT_26M	2917	1554	0.3322609	0	Singlet	Adipocyte
SAT_26M	2930.9	1548	6.2587938	0	Singlet	Adipocyte
SAT_26M	1798.1	1111	1.6433339	5	Singlet	Immune_cells
SAT_26M	1551.4	992	0	8	Singlet	Mesothelial
SAT_26M	1740.9	1104	0.1368721	0	Singlet	Adipocyte
SAT_26M	1077.1	676	7.3606853	5	Singlet	Immune_cells
SAT_26M	2101.3	1276	3.3176884	7	Singlet	Endothelial_cells
SAT_26M	4005.1	1960	0.1468673	0	Singlet	Adipocyte
SAT_26M	1204.4	645	0.8248076	3	Singlet	FAPs
SAT_26M	1315.6	897	11.776836	4	Singlet	Immune_cells
SAT_26M	1023.8	687	14.941036	8	Singlet	Mesothelial
SAT_26M	2001	1137	1.5670738	0	Singlet	Adipocyte
SAT_26M	2088.6	1288	0.2341545	0	Singlet	Adipocyte
SAT_26M	2155.6	1095	3.1327591	10	Singlet	Immune_cells
SAT_26M	2101.8	1180	2.1265635	7	Singlet	Endothelial_cells
SAT_26M	4213.2	1741	14.570147	7	Singlet	Endothelial_cells
SAT_26M	1111.6	720	1.4286214	0	Singlet	Adipocyte
SAT_26M	1703.4	1034	0.0042564	0	Singlet	Adipocyte
SAT_26M	3803.9	1913	3.8841827	0	Singlet	Adipocyte
SAT_26M	1557.5	884	0	3	Singlet	FAPs
SAT_26M	2608.6	1695	0.1468993	11	Singlet	Smooth_muscle_cells
SAT_26M	2515.3	1523	0	0	Singlet	Adipocyte
SAT_26M	1204.2	742	0.6736389	5	Singlet	Immune_cells
SAT_26M	1000.8	672	0	0	Singlet	Adipocyte
SAT_26M	4769.4	2290	4.1440522	0	Singlet	Adipocyte
SAT_26M	1030.2	592	8.9151039	7	Singlet	Endothelial_cells
SAT_26M	2102.7	1330	0.0246862	8	Singlet	Mesothelial
SAT_26M	1866.5	1091	5.7126058	0	Singlet	Adipocyte
SAT_26M	1969.8	1249	3.9094236	0	Singlet	Adipocyte
SAT_26M	1824.7	735	13.960322	0	Singlet	Adipocyte
SAT_26M	1274.8	771	0.2271276	0	Singlet	Adipocyte
SAT_26M	1776.5	1064	1.9071783	0	Singlet	Adipocyte
SAT_26M	2001.3	1117	0	2	Singlet	FAPs
SAT_26M	1930.5	1266	0	5	Singlet	Immune_cells
SAT_26M	1313.7	987	0.1539678	6	Singlet	Neurons
SAT_26M	3111.3	1675	3.2733769	0	Singlet	Adipocyte
SAT_26M	1770.1	1151	5.0706934	7	Singlet	Endothelial_cells
SAT_26M	1219.8	807	8.4896262	6	Singlet	Neurons
SAT_26M	1022	757	0.1113264	3	Singlet	FAPs
SAT_26M	1138.1	784	7.5183932	9	Singlet	Mesothelial
SAT_26M	4061	2009	0.3797794	0	Singlet	Adipocyte
SAT_26M	1020.6	701	1.0398763	3	Singlet	FAPs
SAT_26M	1561	1031	1.5282686	0	Singlet	Adipocyte
SAT_26M	2333.8	1287	10.22832	7	Singlet	Endothelial_cells
SAT_26M	2017.1	1167	0.019287	0	Singlet	Adipocyte
SAT_26M	1078.2	794	2.2938575	1	Singlet	Immune_cells

SAT_26M	2948.4	1654	0	8	Singlet	Mesothelial
SAT_26M	2156.1	1329	0	8	Singlet	Mesothelial
SAT_26M	1344.7	876	2.0197246	0	Singlet	Adipocyte
SAT_26M	3169.1	1580	6.9813906	0	Singlet	Adipocyte
SAT_26M	1452.8	863	1.5088929	0	Singlet	Adipocyte
SAT_26M	3365.2	1732	0.4565443	10	Singlet	Immune_cells
SAT_26M	1352.5	870	0	0	Singlet	Adipocyte
SAT_26M	1395.6	820	8.1613748	0	Singlet	Adipocyte
SAT_26M	1331.7	796	6.1363965	3	Singlet	FAPs
SAT_26M	3583.5	1754	8.4663762	0	Singlet	Adipocyte
SAT_26M	1809.8	995	14.44096	7	Singlet	Endothelial_cells
SAT_26M	1285.5	723	11.043973	0	Singlet	Adipocyte
SAT_26M	1113.7	750	0.553931	0	Singlet	Adipocyte
SAT_26M	1368.9	755	3.6598544	10	Singlet	Immune_cells
SAT_26M	1526.5	1028	0	5	Singlet	Immune_cells
SAT_26M	1251.8	674	10.577883	0	Singlet	Adipocyte
SAT_26M	1753.3	1124	0.0091685	0	Singlet	Adipocyte
SAT_26M	1073.5	721	0.1095085	0	Singlet	Adipocyte
SAT_26M	1040.7	627	5.5765271	10	Singlet	Immune_cells
SAT_26M	2443.9	1508	0	5	Singlet	Immune_cells
SAT_26M	1131.3	675	7.1835229	7	Singlet	Endothelial_cells
SAT_26M	1421.4	890	10.336158	1	Singlet	Immune_cells
SAT_26M	1138.7	738	5.5158845	0	Singlet	Adipocyte
SAT_26M	1091.4	768	1.0041538	0	Singlet	Adipocyte
SAT_26M	1928.5	1186	0.2685911	8	Singlet	Mesothelial
SAT_26M	3602.9	1639	5.1873127	10	Singlet	Immune_cells
SAT_26M	4250.6	2228	0.004062	0	Singlet	Adipocyte
SAT_26M	1409.5	851	7.7981316	0	Singlet	Adipocyte
SAT_26M	2706.2	1506	1.442567	0	Singlet	Adipocyte
SAT_26M	1163.4	796	0.7776877	0	Singlet	Adipocyte
SAT_26M	1447.6	929	12.676301	9	Singlet	Mesothelial
SAT_26M	1341.3	765	5.2364727	7	Singlet	Endothelial_cells
SAT_26M	1106	738	1.5596837	0	Singlet	Adipocyte
SAT_26M	4009.5	1980	0.5354385	0	Singlet	Adipocyte
SAT_26M	1105.9	766	0.4880186	0	Singlet	Adipocyte
SAT_26M	5093.7	2017	1.9262119	10	Singlet	Immune_cells
SAT_26M	2268.4	1306	4.0584662	7	Singlet	Endothelial_cells
SAT_26M	1049.3	771	0.9782504	8	Singlet	Mesothelial
SAT_26M	3754	1850	0	0	Singlet	Adipocyte
SAT_26M	1950.9	1287	8.8279038	4	Singlet	Immune_cells
SAT_26M	2287.5	1314	0.7517345	7	Singlet	Endothelial_cells
SAT_26M	1712.7	1000	3.3328129	0	Singlet	Adipocyte
SAT_26M	1302.4	879	0.7502877	0	Singlet	Adipocyte
SAT_26M	1207.9	811	0.0163399	8	Singlet	Mesothelial
SAT_26M	1438.5	967	0.4293057	5	Singlet	Immune_cells
SAT_26M	1094.2	749	2.1827203	0	Singlet	Adipocyte
SAT_26M	1482.8	980	2.4171261	0	Singlet	Adipocyte
SAT_26M	1280.3	876	1.079475	0	Singlet	Adipocyte
SAT_26M	1177	789	0.1796891	6	Singlet	Neurons
SAT_26M	1631.2	1000	7.0765469	5	Singlet	Immune_cells
SAT_26M	2568.6	1489	6.2100319	0	Singlet	Adipocyte

SAT_26M	1191.3	726	0.9774506	0	Singlet	Adipocyte
SAT_26M	1490.7	798	0.821798	0	Singlet	Adipocyte
SAT_26M	2123.7	1249	0.7543627	0	Singlet	Adipocyte
SAT_26M	1149.6	739	0.5423948	7	Singlet	Endothelial_cells
SAT_26M	3340.7	1855	0.028623	0	Singlet	Adipocyte
SAT_26M	1367.2	872	0	8	Singlet	Mesothelial
SAT_26M	4464	2377	13.466311	8	Singlet	Mesothelial
SAT_26M	2570.6	1391	5.7882236	0	Singlet	Adipocyte
SAT_26M	1325.3	729	7.2480569	0	Singlet	Adipocyte
SAT_26M	1588.7	746	14.361425	0	Singlet	Adipocyte
SAT_26M	2253.2	1235	0.8546579	0	Singlet	Adipocyte
SAT_26M	1269	816	3.3563756	0	Singlet	Adipocyte
SAT_26M	1008	718	7.9987265	1	Singlet	Immune_cells
SAT_26M	1091.2	735	6.2871717	5	Singlet	Immune_cells
SAT_26M	1727.2	1084	4.1343958	0	Singlet	Adipocyte
SAT_26M	1176.6	770	6.097376	0	Singlet	Adipocyte
SAT_26M	2965.3	1708	5.4472529	9	Singlet	Mesothelial
SAT_26M	1233.5	791	5.9496463	7	Singlet	Endothelial_cells
SAT_26M	1744.1	1157	1.9409162	8	Singlet	Mesothelial
SAT_26M	2033	1180	0.7110908	0	Singlet	Adipocyte
SAT_26M	3179.7	1941	3.2023279	9	Singlet	Mesothelial
SAT_26M	2791.3	1527	5.3638843	0	Singlet	Adipocyte
SAT_26M	1232	829	2.643061	0	Singlet	Adipocyte
SAT_26M	3919.7	2003	0.1218364	3	Singlet	FAPs
SAT_26M	3315.6	1958	7.8958378	8	Singlet	Mesothelial
SAT_26M	1399.9	868	9.473719	7	Singlet	Endothelial_cells
SAT_26M	1176.5	805	1.7976442	0	Singlet	Adipocyte
SAT_26M	1610.9	994	1.3289286	0	Singlet	Adipocyte
SAT_26M	3005.8	1436	8.83144	7	Singlet	Endothelial_cells
SAT_26M	1142.1	778	0.8041195	0	Singlet	Adipocyte
SAT_26M	1462.5	823	10.148023	7	Singlet	Endothelial_cells
SAT_26M	1053.1	641	2.599832	10	Singlet	Immune_cells
SAT_26M	2030	1280	4.860382	9	Singlet	Mesothelial
SAT_26M	3183.2	1660	8.0669612	7	Singlet	Endothelial_cells
SAT_26M	1213	736	0.1365631	2	Singlet	FAPs
SAT_26M	2475.3	1374	0	8	Singlet	Mesothelial
SAT_26M	1261	788	2.961414	6	Singlet	Neurons
SAT_26M	1339.7	941	0.6265135	0	Singlet	Adipocyte
SAT_26M	1009.9	688	2.9921868	1	Singlet	Immune_cells
SAT_26M	3336.6	2002	2.4364499	9	Singlet	Mesothelial
SAT_26M	1144.2	601	10.853335	7	Singlet	Endothelial_cells
SAT_26M	1205.5	643	12.447879	7	Singlet	Endothelial_cells
SAT_26M	1184.8	769	1.1896425	7	Singlet	Endothelial_cells
SAT_26M	1064.5	737	0.8367125	8	Singlet	Mesothelial
SAT_26M	1191.3	817	4.2237988	0	Singlet	Adipocyte
SAT_26M	1011	628	7.5290326	0	Singlet	Adipocyte
SAT_26M	1007.4	669	0.33673	0	Singlet	Adipocyte
SAT_26M	1696.2	927	10.17802	0	Singlet	Adipocyte
SAT_26M	4317.1	1722	0.0219584	10	Singlet	Immune_cells
SAT_26M	1253.1	847	0.1011647	6	Singlet	Neurons
SAT_26M	1555.2	1008	1.5510322	0	Singlet	Adipocyte

SAT_26M	2957.7	1559	4.8792719	0	Singlet	Adipocyte
SAT_26M	2195.6	1277	1.4029878	0	Singlet	Adipocyte
SAT_26M	1390.8	859	3.4712561	10	Singlet	Immune_cells
SAT_26M	2086.7	1223	0.0130254	0	Singlet	Adipocyte
SAT_26M	3487.5	1735	2.6116058	10	Singlet	Immune_cells
SAT_26M	1583.4	922	10.883131	0	Singlet	Adipocyte
SAT_26M	3326.2	1873	10.302725	7	Singlet	Endothelial_cells
SAT_26M	1947.5	1118	0.2707797	0	Singlet	Adipocyte
SAT_26M	1506.8	834	9.9185566	3	Singlet	FAPs
SAT_26M	1801.5	1027	1.359107	0	Singlet	Adipocyte
SAT_26M	1719.8	1177	3.9620346	1	Singlet	Immune_cells
SAT_26M	2220.4	1349	3.4830819	5	Singlet	Immune_cells
SAT_26M	3396.3	1887	1.1815221	2	Singlet	FAPs
SAT_26M	1385.5	954	0.5455021	0	Singlet	Adipocyte
SAT_26M	1363.2	760	0	6	Singlet	Neurons
SAT_26M	3140.9	1821	0	5	Singlet	Immune_cells
SAT_26M	1254.4	838	2.0724171	0	Singlet	Adipocyte
SAT_26M	1292.5	853	2.772543	5	Singlet	Immune_cells
SAT_26M	2773	1487	4.2518678	0	Singlet	Adipocyte
SAT_26M	1806.9	1127	0.242785	5	Singlet	Immune_cells
SAT_26M	2916.7	1468	1.4244976	10	Singlet	Immune_cells
SAT_26M	1588.1	1013	2.5567022	0	Singlet	Adipocyte
SAT_26M	1445.4	733	10.423347	7	Singlet	Endothelial_cells
SAT_26M	1513.7	902	0.3286242	5	Singlet	Immune_cells
SAT_26M	1112.1	813	1.1818356	0	Singlet	Adipocyte
SAT_26M	1592.9	986	3.6766678	0	Singlet	Adipocyte
SAT_26M	2552	1459	0.8105764	0	Singlet	Adipocyte
SAT_26M	1735.5	1058	1.3027015	0	Singlet	Adipocyte
SAT_26M	1834.4	1095	1.0118577	0	Singlet	Adipocyte
SAT_26M	1189.5	732	2.3686141	1	Singlet	Immune_cells
SAT_26M	2530	1467	0.0120972	0	Singlet	Adipocyte
SAT_26M	1078.4	586	4.1388894	0	Singlet	Adipocyte
SAT_26M	2596.4	1782	1.7235023	1	Singlet	Immune_cells
SAT_26M	2033.4	1178	5.5809358	7	Singlet	Endothelial_cells
SAT_26M	2165.8	1205	8.6947878	0	Singlet	Adipocyte
SAT_26M	1062.8	789	2.6537979	1	Singlet	Immune_cells
SAT_26M	1712.4	1161	0.7349133	8	Singlet	Mesothelial
SAT_26M	1608.8	959	4.9929109	7	Singlet	Endothelial_cells
SAT_26M	1218.9	719	7.6601385	7	Singlet	Endothelial_cells
SAT_26M	1211.2	895	0.9980231	1	Singlet	Immune_cells
SAT_26M	2217	1316	3.8261241	8	Singlet	Mesothelial
SAT_26M	1399.3	873	5.6183082	7	Singlet	Endothelial_cells
SAT_26M	2286.5	1316	1.4571861	0	Singlet	Adipocyte
SAT_26M	1031.5	725	0.0435915	8	Singlet	Mesothelial
SAT_26M	1096.4	608	1.1323981	5	Singlet	Immune_cells
SAT_26M	2601.5	1396	2.015947	0	Singlet	Adipocyte
SAT_26M	1020.2	681	4.3589191	7	Singlet	Endothelial_cells
SAT_26M	1093	738	0.9771763	0	Singlet	Adipocyte
SAT_26M	1355.9	902	1.5569769	0	Singlet	Adipocyte
SAT_26M	1320.7	840	5.4161512	7	Singlet	Endothelial_cells
SAT_26M	1083.1	860	1.7831963	11	Singlet	Smooth_muscle_cells

SAT_26M	1964	1149	1.6354177	0	Singlet	Adipocyte
SAT_26M	1861.7	1174	0.9037911	0	Singlet	Adipocyte
SAT_26M	2356.5	1277	0	0	Singlet	Adipocyte
SAT_26M	1237.2	835	1.8468311	6	Singlet	Neurons
SAT_26M	2499.1	1458	4.147077	0	Singlet	Adipocyte
SAT_26M	3520.9	1915	11.14847	9	Singlet	Mesothelial
SAT_26M	1233.6	871	0.0060916	0	Singlet	Adipocyte
SAT_26M	2854.6	1412	1.3939951	10	Singlet	Immune_cells
SAT_26M	1204.7	846	1.1745696	7	Singlet	Endothelial_cells
SAT_26M	1868.7	1206	1.0036422	5	Singlet	Immune_cells
SAT_26M	3179.3	1889	1.1082897	8	Singlet	Mesothelial
SAT_26M	2008.1	1080	1.0211107	10	Singlet	Immune_cells
SAT_26M	2798.6	1764	0.4593621	8	Singlet	Mesothelial
SAT_26M	1590.5	1079	2.0011583	0	Singlet	Adipocyte
SAT_26M	2507.1	1435	0.0508822	0	Singlet	Adipocyte
SAT_26M	3727.3	1989	3.9439849	0	Singlet	Adipocyte
SAT_26M	1135.5	572	6.0296597	0	Singlet	Adipocyte
SAT_26M	1684.6	1017	9.7988284	3	Singlet	FAPs
SAT_26M	2679.1	1438	12.208658	7	Singlet	Endothelial_cells
SAT_26M	1349.9	964	0.9088163	11	Singlet	Smooth_muscle_cells
SAT_26M	1673.1	1140	2.1967123	11	Singlet	Smooth_muscle_cells
SAT_26M	2232.8	1295	0.2504517	5	Singlet	Immune_cells
SAT_26M	1546.6	730	8.1549744	0	Singlet	Adipocyte
SAT_26M	1177	901	0.2274749	6	Singlet	Neurons
SAT_26M	1246.4	770	2.4692287	0	Singlet	Adipocyte
SAT_26M	1366.8	859	0.072108	0	Singlet	Adipocyte
SAT_26M	2997.2	1752	0.2633259	8	Singlet	Mesothelial
SAT_26M	1259	897	1.3758155	5	Singlet	Immune_cells
SAT_26M	1951.5	1159	0.0154025	0	Singlet	Adipocyte
SAT_26M	1489.9	978	2.8168096	6	Singlet	Neurons
SAT_26M	1127.5	707	3.3555414	7	Singlet	Endothelial_cells
SAT_26M	1620.3	930	4.0130957	5	Singlet	Immune_cells
SAT_26M	1424.4	951	4.4785086	0	Singlet	Adipocyte
SAT_26M	1346.8	909	3.5378995	4	Singlet	Immune_cells
SAT_26M	5253.1	2472	6.8483375	0	Singlet	Adipocyte
SAT_26M	1125.1	849	0.1488195	4	Singlet	Immune_cells
SAT_26M	1228.7	752	7.6055549	7	Singlet	Endothelial_cells
SAT_26M	5518.4	2513	0.1888081	0	Singlet	Adipocyte
SAT_26M	1497.2	928	0.0984942	0	Singlet	Adipocyte
SAT_26M	1007.1	662	2.2090597	0	Singlet	Adipocyte
SAT_26M	2078.7	1029	9.7838502	0	Singlet	Adipocyte
SAT_26M	1276.7	612	3.8904336	10	Singlet	Immune_cells
SAT_26M	1713.4	1068	0.0173764	8	Singlet	Mesothelial
SAT_26M	1639.6	1140	0.9664931	7	Singlet	Endothelial_cells
SAT_26M	1661.5	1099	0.1992207	0	Singlet	Adipocyte
SAT_26M	1014.2	639	3.385213	7	Singlet	Endothelial_cells
SAT_26M	1008.5	649	7.7861008	4	Singlet	Immune_cells
SAT_26M	1156.2	762	0.3811661	0	Singlet	Adipocyte
SAT_26M	1150.6	718	1.5385118	5	Singlet	Immune_cells
SAT_26M	1451	969	0	0	Singlet	Adipocyte
SAT_26M	1180.8	809	0.2874905	5	Singlet	Immune_cells

SAT_26M	3424.1	1847	3.4368432	0	Singlet	Adipocyte
SAT_26M	1291.3	781	14.416056	0	Singlet	Adipocyte
SAT_26M	1940.7	1127	0.0941696	0	Singlet	Adipocyte
SAT_26M	1056.8	761	2.4826999	4	Singlet	Immune_cells
SAT_26M	2028.5	1223	3.0114342	7	Singlet	Endothelial_cells
SAT_26M	1659.9	989	10.409531	5	Singlet	Immune_cells
SAT_26M	1145.2	827	1.5199233	8	Singlet	Mesothelial
SAT_26M	1795.9	1115	0.1154403	0	Singlet	Adipocyte
SAT_26M	1370.2	947	0.0251969	0	Singlet	Adipocyte
SAT_26M	1581.6	1035	2.6350595	0	Singlet	Adipocyte
SAT_26M	1326.8	835	5.7348253	0	Singlet	Adipocyte
SAT_26M	1417.4	889	0.5554859	0	Singlet	Adipocyte
SAT_26M	1459.7	922	1.2493466	0	Singlet	Adipocyte
SAT_26M	3137.7	1674	0.5703275	0	Singlet	Adipocyte
SAT_26M	2357.4	1185	3.2361546	5	Singlet	Immune_cells
SAT_26M	2670.8	1638	0.9570824	5	Singlet	Immune_cells
SAT_26M	1778.3	1092	0.6260123	0	Singlet	Adipocyte
SAT_26M	1323.1	758	3.6265778	5	Singlet	Immune_cells
SAT_26M	1149.8	626	14.039411	0	Singlet	Adipocyte
SAT_26M	2685.5	1624	1.6704364	0	Singlet	Adipocyte
SAT_26M	1025.4	771	0.1473269	8	Singlet	Mesothelial
SAT_26M	1569.3	904	11.81519	0	Singlet	Adipocyte
SAT_26M	1296.5	876	0	8	Singlet	Mesothelial
SAT_26M	1563.4	1004	1.2525478	0	Singlet	Adipocyte
SAT_26M	4509.8	1909	3.6773705	10	Singlet	Immune_cells
SAT_26M	1083	674	7.4982609	8	Singlet	Mesothelial
SAT_26M	5460.6	2830	2.5542444	8	Singlet	Mesothelial
SAT_26M	4358.1	2211	1.4470313	0	Singlet	Adipocyte
SAT_26M	2050.7	1391	0.5939514	0	Singlet	Adipocyte
SAT_26M	2378.9	1331	4.043251	7	Singlet	Endothelial_cells
SAT_26M	1711	1011	0.0108511	0	Singlet	Adipocyte
SAT_26M	2682.7	1336	9.3995233	0	Singlet	Adipocyte
SAT_26M	1692.1	1085	2.8860263	0	Singlet	Adipocyte
SAT_26M	3903.9	1907	0.6096732	0	Singlet	Adipocyte
SAT_26M	5666.5	2602	0	0	Singlet	Adipocyte
SAT_26M	1264.4	910	4.4177716	4	Singlet	Immune_cells
SAT_26M	1245.4	740	4.5042591	7	Singlet	Endothelial_cells
SAT_26M	1726.7	1055	1.8358762	7	Singlet	Endothelial_cells
SAT_26M	2766.1	1478	0.4932247	0	Singlet	Adipocyte
SAT_26M	1750.3	898	7.1956289	0	Singlet	Adipocyte
SAT_26M	1996.5	1193	0.1648025	0	Singlet	Adipocyte
SAT_26M	3403.7	1947	0.705918	1	Singlet	Immune_cells
SAT_26M	1736.4	1118	4.0470024	5	Singlet	Immune_cells
SAT_26M	2178.3	1043	12.23396	0	Singlet	Adipocyte
SAT_26M	1127.1	829	0.0129965	8	Singlet	Mesothelial
SAT_26M	1959	1116	6.3336169	7	Singlet	Endothelial_cells
SAT_26M	1081.3	684	0.3346935	0	Singlet	Adipocyte
SAT_26M	2548.4	1439	0.2814851	0	Singlet	Adipocyte
SAT_26M	2157.4	1261	1.1551847	0	Singlet	Adipocyte
SAT_26M	2474.3	1329	5.403938	0	Singlet	Adipocyte
SAT_26M	3422.8	1751	5.4273536	0	Singlet	Adipocyte

SAT_26M	1815.2	1072	2.7651374	7	Singlet	Endothelial_cells
SAT_26M	6625	3125	4.6136916	9	Singlet	Mesothelial
SAT_26M	1344.2	927	0.1363464	5	Singlet	Immune_cells
SAT_26M	2454.6	1431	2.3131941	7	Singlet	Endothelial_cells
SAT_26M	1208.8	915	2.7241845	1	Singlet	Immune_cells
SAT_26M	1511.1	917	5.6724605	0	Singlet	Adipocyte
SAT_26M	1810.6	1150	0.2411617	5	Singlet	Immune_cells
SAT_26M	1098.9	655	14.32085	7	Singlet	Endothelial_cells
SAT_26M	1957.3	1063	0	6	Singlet	Neurons
SAT_26M	1107.4	736	7.0893874	0	Singlet	Adipocyte
SAT_26M	2462.1	1142	12.34777	0	Singlet	Adipocyte
SAT_26M	1423.9	961	1.0119044	1	Singlet	Immune_cells
SAT_26M	1699.6	939	5.244308	0	Singlet	Adipocyte
SAT_26M	1237.6	823	8.664966	3	Singlet	FAPs
SAT_26M	2128.7	1146	1.0507104	10	Singlet	Immune_cells
SAT_26M	2335.4	1394	0.1273548	0	Singlet	Adipocyte
SAT_26M	2652.1	1469	2.1978333	0	Singlet	Adipocyte
SAT_26M	3303.5	1700	5.8766187	0	Singlet	Adipocyte
SAT_26M	2214	1196	5.8989073	0	Singlet	Adipocyte
SAT_26M	1224.3	794	3.1447611	0	Singlet	Adipocyte
SAT_26M	3274.4	1709	5.8003548	0	Singlet	Adipocyte
SAT_26M	3411.3	1855	0.2385009	0	Singlet	Adipocyte
SAT_26M	1008.5	717	2.1137628	6	Singlet	Neurons
SAT_26M	1595.8	1097	2.8054875	1	Singlet	Immune_cells
SAT_26M	1503.5	887	0.0549156	0	Singlet	Adipocyte
SAT_26M	1294.4	936	4.7147042	1	Singlet	Immune_cells
SAT_26M	1308.3	883	1.3248607	0	Singlet	Adipocyte
SAT_26M	2835.4	1572	0.189312	0	Singlet	Adipocyte
SAT_26M	1164.4	746	3.9259193	0	Singlet	Adipocyte
SAT_26M	2198.3	1172	7.8389992	7	Singlet	Endothelial_cells
SAT_26M	1745.2	1069	0.8912576	0	Singlet	Adipocyte
SAT_26M	1267.9	836	5.327888	5	Singlet	Immune_cells
SAT_26M	1621.6	865	9.8730676	7	Singlet	Endothelial_cells
SAT_26M	1275.2	878	0	0	Singlet	Adipocyte
SAT_26M	1874.7	1238	2.3351349	4	Singlet	Immune_cells
SAT_26M	1606.1	886	9.4501774	7	Singlet	Endothelial_cells
SAT_26M	1971.1	1167	0.4699005	5	Singlet	Immune_cells
SAT_26M	3640.3	1808	0.895308	0	Singlet	Adipocyte
SAT_26M	1003.7	729	4.2700063	8	Singlet	Mesothelial
SAT_26M	1484.3	872	5.8405717	7	Singlet	Endothelial_cells
SAT_26M	1764.9	952	7.5830432	10	Singlet	Immune_cells
SAT_26M	2102.4	1359	4.1061903	7	Singlet	Endothelial_cells
SAT_26M	1985.2	1113	1.0248539	10	Singlet	Immune_cells
SAT_26M	1393.1	871	4.8033011	8	Singlet	Mesothelial
SAT_26M	1539.4	935	9.5695546	7	Singlet	Endothelial_cells
SAT_26M	1432.7	883	0.5200752	0	Singlet	Adipocyte
SAT_26M	1209	837	1.3818001	5	Singlet	Immune_cells
SAT_26M	2112.1	1341	0.5782657	7	Singlet	Endothelial_cells
SAT_26M	1882.9	1131	3.7512061	0	Singlet	Adipocyte
SAT_26M	1291.4	864	0.0159665	0	Singlet	Adipocyte
SAT_26M	3087.2	1627	0.1922497	0	Singlet	Adipocyte

SAT_26M	1510.7	791	9.2082863	0	Singlet	Adipocyte
SAT_26M	1482.1	978	4.8026446	0	Singlet	Adipocyte
SAT_26M	1306.1	981	0.1343301	1	Singlet	Immune_cells
SAT_26M	1388.2	908	6.3395353	4	Singlet	Immune_cells
SAT_26M	1961.8	904	11.664554	0	Singlet	Adipocyte
SAT_26M	1755.4	1107	0.0057578	0	Singlet	Adipocyte
SAT_26M	1117.3	740	8.0858698	8	Singlet	Mesothelial
SAT_26M	1155.6	845	0.1779637	3	Singlet	FAPs
SAT_26M	1539.1	941	8.8061142	0	Singlet	Adipocyte
SAT_26M	3193.4	1434	10.329581	5	Singlet	Immune_cells
SAT_26M	3942.8	2073	0	0	Singlet	Adipocyte
SAT_26M	1128	779	4.1464547	3	Singlet	FAPs
SAT_26M	1115.8	604	3.8623488	0	Singlet	Adipocyte
SAT_26M	1511.2	962	0.0544956	0	Singlet	Adipocyte
SAT_26M	3099.7	1971	1.7697315	8	Singlet	Mesothelial
SAT_26M	3943.5	2077	1.8353986	0	Singlet	Adipocyte
SAT_26M	1041.4	745	0.0426252	8	Singlet	Mesothelial
SAT_26M	1651.7	900	8.055414	0	Singlet	Adipocyte
SAT_26M	1089.2	777	3.5126735	3	Singlet	FAPs
SAT_26M	2138.6	1178	2.4445973	5	Singlet	Immune_cells
SAT_26M	2908	1734	5.1805078	8	Singlet	Mesothelial
SAT_26M	3624.5	1926	0.4709176	0	Singlet	Adipocyte
SAT_26M	2510.2	1425	4.159081	7	Singlet	Endothelial_cells
SAT_26M	1391.3	945	1.9204295	5	Singlet	Immune_cells
SAT_26M	1696.4	1084	5.3726855	0	Singlet	Adipocyte
SAT_26M	1136.4	735	2.1834509	0	Singlet	Adipocyte
SAT_26M	1160.2	583	11.011482	5	Singlet	Immune_cells
SAT_26M	1835.4	1244	0.8834843	8	Singlet	Mesothelial
SAT_26M	1194.3	871	0.0470463	5	Singlet	Immune_cells
SAT_26M	1492.2	917	7.0278417	9	Singlet	Mesothelial
SAT_26M	2649.8	1431	0.1958798	0	Singlet	Adipocyte
SAT_26M	1116.4	635	2.4710669	0	Singlet	Adipocyte
SAT_26M	3275.6	1648	5.9275768	0	Singlet	Adipocyte
SAT_26M	5238.5	2310	6.5627091	0	Singlet	Adipocyte
SAT_26M	1335.6	870	1.2744402	0	Singlet	Adipocyte
SAT_26M	2027.7	1057	11.889036	7	Singlet	Endothelial_cells
SAT_26M	1017.4	643	0.1100111	3	Singlet	FAPs
SAT_26M	2405.2	1424	0.6034876	0	Singlet	Adipocyte
SAT_26M	2456	1563	0	8	Singlet	Mesothelial
SAT_26M	2267.7	1399	6.3740473	7	Singlet	Endothelial_cells
SAT_26M	1739.8	1134	0.0386388	0	Singlet	Adipocyte
SAT_26M	2719.2	1459	2.9044642	0	Singlet	Adipocyte
SAT_26M	2782.6	1512	0.5425019	0	Singlet	Adipocyte
SAT_26M	3618.7	1765	8.6963774	0	Singlet	Adipocyte
SAT_26M	2765.2	1547	0.2121564	0	Singlet	Adipocyte
SAT_26M	1614.7	1010	0.1067712	5	Singlet	Immune_cells
SAT_26M	1079	706	3.1445207	7	Singlet	Endothelial_cells
SAT_26M	1953.6	1105	8.8203193	0	Singlet	Adipocyte
SAT_26M	1843.1	1094	1.5917829	0	Singlet	Adipocyte
SAT_26M	2341.6	1379	0.0359069	0	Singlet	Adipocyte
SAT_26M	3860.6	1902	0.2920235	0	Singlet	Adipocyte

SAT_26M	1660.1	979	5.5542737	0	Singlet	Adipocyte
SAT_26M	1113.9	781	0.3146028	6	Singlet	Neurons
SAT_26M	2268	1232	7.8314689	7	Singlet	Endothelial_cells
SAT_26M	1614.8	1023	4.6534844	3	Singlet	FAPs
SAT_26M	1500.2	858	7.1004506	0	Singlet	Adipocyte
SAT_26M	2835.1	1700	2.5434221	5	Singlet	Immune_cells
SAT_26M	2541.2	1466	0.0572005	0	Singlet	Adipocyte
SAT_26M	1946	1160	13.947715	9	Singlet	Mesothelial
SAT_26M	1886.5	1204	0.2825156	0	Singlet	Adipocyte
SAT_26M	3343.8	1832	2.7445204	0	Singlet	Adipocyte
SAT_26M	1129	799	0	3	Singlet	FAPs
SAT_26M	1722.5	1098	4.2638791	7	Singlet	Endothelial_cells
SAT_26M	1542.3	921	0.5633288	0	Singlet	Adipocyte
SAT_26M	1138.7	799	0.1428973	0	Singlet	Adipocyte
SAT_26M	1806.3	1091	2.1553352	10	Singlet	Immune_cells
SAT_26M	1507.5	750	12.51927	7	Singlet	Endothelial_cells
SAT_26M	3173.3	1752	9.0615968	9	Singlet	Mesothelial
SAT_26M	2212.9	1212	1.0511444	0	Singlet	Adipocyte
SAT_26M	1227	706	11.361676	7	Singlet	Endothelial_cells
SAT_26M	5412.5	2584	4.6896456	9	Singlet	Mesothelial
SAT_26M	2981.5	1659	0.5480977	0	Singlet	Adipocyte
SAT_26M	2071.9	1173	3.0876287	0	Singlet	Adipocyte
SAT_26M	1208	741	9.6522951	4	Singlet	Immune_cells
SAT_26M	4541.4	2163	10.210096	9	Singlet	Mesothelial
SAT_26M	1416.9	904	0.3048332	5	Singlet	Immune_cells
SAT_26M	3299	1696	3.1622819	0	Singlet	Adipocyte
SAT_26M	1834.9	1103	8.693361	9	Singlet	Mesothelial
SAT_26M	1543.5	1098	1.3895161	8	Singlet	Mesothelial
SAT_26M	1525.4	962	0.0421998	7	Singlet	Endothelial_cells
SAT_26M	1210.6	791	0.4046724	0	Singlet	Adipocyte
SAT_26M	1409.7	931	10.189066	9	Singlet	Mesothelial
SAT_26M	1891.6	1137	1.9253712	0	Singlet	Adipocyte
SAT_26M	2130.7	1324	1.6984938	3	Singlet	FAPs
SAT_26M	1458.2	937	0	5	Singlet	Immune_cells
SAT_26M	1206.1	771	1.0422487	0	Singlet	Adipocyte
SAT_26M	1830.9	1080	1.7412932	0	Singlet	Adipocyte
SAT_26M	1675.5	1172	1.6975882	5	Singlet	Immune_cells
SAT_26M	1747.5	1176	0	0	Singlet	Adipocyte
SAT_26M	4513.9	2198	3.2251424	0	Singlet	Adipocyte
SAT_26M	1178.7	731	2.4499207	7	Singlet	Endothelial_cells
SAT_26M	1685.7	1070	0.6989494	0	Singlet	Adipocyte
SAT_26M	1149.4	771	0.2476955	7	Singlet	Endothelial_cells
SAT_26M	2581.8	1416	0.6818261	0	Singlet	Adipocyte
SAT_26M	1656.7	1061	7.0538273	4	Singlet	Immune_cells
SAT_26M	1780	956	1.6911302	10	Singlet	Immune_cells
SAT_26M	1800.5	916	7.9882266	0	Singlet	Adipocyte
SAT_26M	1962.7	1142	3.0741999	0	Singlet	Adipocyte
SAT_26M	1232.8	884	0.6154746	0	Singlet	Adipocyte
SAT_26M	2098.8	1264	0.7135486	0	Singlet	Adipocyte
SAT_26M	1842.2	1176	0.0088947	8	Singlet	Mesothelial
SAT_26M	1198.3	765	11.426266	4	Singlet	Immune_cells

SAT_26M	2242.3	1200	9.1613099	0	Singlet	Adipocyte
SAT_26M	1727.6	1065	0.0431992	0	Singlet	Adipocyte
SAT_26M	1497.9	983	0.426678	0	Singlet	Adipocyte
SAT_26M	1833.2	1197	1.0200062	3	Singlet	FAPs
SAT_26M	2056.5	1333	0.030081	0	Singlet	Adipocyte
SAT_26M	3283	1683	4.3213066	3	Singlet	FAPs
SAT_26M	2096	1067	6.9129172	10	Singlet	Immune_cells
SAT_26M	1517.5	936	1.6083327	0	Singlet	Adipocyte
SAT_26M	2187	990	13.964427	0	Singlet	Adipocyte
SAT_26M	1409.6	941	5.5843303	8	Singlet	Mesothelial
SAT_26M	1063.1	719	0.8416726	7	Singlet	Endothelial_cells
SAT_26M	3989.9	1960	0.1928713	0	Singlet	Adipocyte
SAT_26M	1061	781	0.3591596	0	Singlet	Adipocyte
SAT_26M	3327.2	1929	1.9069934	9	Singlet	Mesothelial
SAT_26M	2358.7	1463	0.54257	0	Singlet	Adipocyte
SAT_26M	1750.9	1020	8.3946863	7	Singlet	Endothelial_cells
SAT_26M	1255.9	857	1.0043798	0	Singlet	Adipocyte
SAT_26M	2436.2	1450	0.046929	0	Singlet	Adipocyte
SAT_26M	1603.5	1023	4.8992862	9	Singlet	Mesothelial
SAT_26M	1448.4	851	0.1859316	0	Singlet	Adipocyte
SAT_26M	1038.3	675	0.0728824	6	Singlet	Neurons
SAT_26M	3603.9	1897	0.65594	0	Singlet	Adipocyte
SAT_26M	1034.7	598	6.4704177	0	Singlet	Adipocyte
SAT_26M	1530	1060	0.5597657	3	Singlet	FAPs
SAT_26M	1061	607	11.160673	7	Singlet	Endothelial_cells
SAT_26M	1527.1	1012	0	8	Singlet	Mesothelial
SAT_26M	1068	693	0.9622463	5	Singlet	Immune_cells
SAT_26M	1048.3	704	1.7233453	0	Singlet	Adipocyte
SAT_26M	1572	941	1.6005716	0	Singlet	Adipocyte
SAT_26M	1832.9	1132	0.8315544	0	Singlet	Adipocyte
SAT_26M	1443.3	948	0	0	Singlet	Adipocyte
SAT_26M	1078.4	736	0.0842914	8	Singlet	Mesothelial
SAT_26M	1911.4	999	14.66592	7	Singlet	Endothelial_cells
SAT_26M	1090.1	778	0.2408347	8	Singlet	Mesothelial
SAT_26M	3764.2	1858	3.0264517	0	Singlet	Adipocyte
SAT_26M	1217.1	930	1.3148972	4	Singlet	Immune_cells
SAT_26M	2728.3	1552	2.9373947	0	Singlet	Adipocyte
SAT_26M	2074.3	1191	9.7424293	9	Singlet	Mesothelial
SAT_26M	1018.7	683	0.5216237	0	Singlet	Adipocyte
SAT_26M	1071.5	796	0.3946095	3	Singlet	FAPs
SAT_26M	2421.4	1451	0.8715533	0	Singlet	Adipocyte
SAT_26M	1358.4	933	0.0734699	0	Singlet	Adipocyte
SAT_26M	1885.4	1235	4.1376531	9	Singlet	Mesothelial
SAT_26M	5752	2597	9.9699702	0	Singlet	Adipocyte
SAT_26M	2596	1453	0.0288218	0	Singlet	Adipocyte
SAT_26M	1051.5	743	12.854719	1	Singlet	Immune_cells
SAT_26M	1114.9	728	1.1249568	7	Singlet	Endothelial_cells
SAT_26M	1200.6	832	0	8	Singlet	Mesothelial
SAT_26M	1077.6	656	5.0281228	5	Singlet	Immune_cells
SAT_26M	1152.2	673	0	6	Singlet	Neurons
SAT_26M	1391.3	770	14.606884	5	Singlet	Immune_cells

SAT_26M	3620.6	2188	6.0575853	8	Singlet	Mesothelial
SAT_26M	1143.5	793	0.2282845	8	Singlet	Mesothelial
SAT_26M	1033.3	641	3.7773317	0	Singlet	Adipocyte
SAT_26M	1338.4	972	0.9428545	0	Singlet	Adipocyte
SAT_26M	1850.2	1156	0.1035316	0	Singlet	Adipocyte
SAT_26M	1375.3	807	13.168572	3	Singlet	FAPs
SAT_26M	2367.3	1362	0.026197	7	Singlet	Endothelial_cells
SAT_26M	1578.7	1060	2.8057342	1	Singlet	Immune_cells
SAT_26M	1113	802	0.2782693	8	Singlet	Mesothelial
SAT_26M	1122.4	708	5.9728221	0	Singlet	Adipocyte
SAT_26M	1250.3	817	0.0087546	6	Singlet	Neurons
SAT_26M	2490.7	1460	1.5062569	7	Singlet	Endothelial_cells
SAT_26M	1337.5	892	2.2172624	9	Singlet	Mesothelial
SAT_26M	1072.1	753	0.9932258	0	Singlet	Adipocyte
SAT_26M	1139.7	822	0.6368755	3	Singlet	FAPs
SAT_26M	1573.8	1038	0	0	Singlet	Adipocyte
SAT_26M	2826.8	1407	11.628383	7	Singlet	Endothelial_cells
SAT_26M	1015.6	653	6.6655158	3	Singlet	FAPs
SAT_26M	1899.6	1217	0.3068304	0	Singlet	Adipocyte
SAT_26M	3685.8	1650	8.6677064	0	Singlet	Adipocyte
SAT_26M	2758.6	1540	1.8765389	0	Singlet	Adipocyte
SAT_26M	2271.5	1305	0.2421758	0	Singlet	Adipocyte
SAT_26M	2312.1	1306	3.3292329	0	Singlet	Adipocyte
SAT_26M	2734.9	1532	0.034525	8	Singlet	Mesothelial
SAT_26M	1040.8	683	5.4740516	0	Singlet	Adipocyte
SAT_26M	1113.1	731	0	0	Singlet	Adipocyte
SAT_26M	2363.1	1488	0.3197725	1	Singlet	Immune_cells
SAT_26M	1294.1	859	0.0795245	0	Singlet	Adipocyte
SAT_26M	1098.7	738	1.6315075	0	Singlet	Adipocyte
SAT_26M	1695.1	924	6.5579178	0	Singlet	Adipocyte
SAT_26M	1340.8	945	1.180107	1	Singlet	Immune_cells
SAT_26M	1353.5	949	0	0	Singlet	Adipocyte
SAT_26M	1507.7	907	7.7305904	0	Singlet	Adipocyte
SAT_26M	1537.6	888	3.9495848	0	Singlet	Adipocyte
SAT_26M	1054.5	775	0.9088398	0	Singlet	Adipocyte
SAT_26M	2256.1	1175	12.779113	0	Singlet	Adipocyte
SAT_26M	1662.1	1179	2.6358316	1	Singlet	Immune_cells
SAT_26M	1237.2	845	0.0164327	0	Singlet	Adipocyte
SAT_26M	2101.7	1004	3.7615579	10	Singlet	Immune_cells
SAT_26M	1358.2	878	9.0036962	8	Singlet	Mesothelial
SAT_26M	2569.3	1428	0.0082595	0	Singlet	Adipocyte
SAT_26M	1124.7	808	0.2075981	8	Singlet	Mesothelial
SAT_26M	1353.7	903	8.3795115	4	Singlet	Immune_cells
SAT_26M	2036.3	1356	0.6318088	0	Singlet	Adipocyte
SAT_26M	2718.2	1698	0	8	Singlet	Mesothelial
SAT_26M	1265.3	847	3.5340699	8	Singlet	Mesothelial
SAT_26M	3814.1	2038	0.7595757	0	Singlet	Adipocyte
SAT_26M	1329.6	859	0.0082905	8	Singlet	Mesothelial
SAT_26M	1169.2	621	12.35742	7	Singlet	Endothelial_cells
SAT_26M	2669.1	1618	2.0546215	7	Singlet	Endothelial_cells
SAT_26M	1230.3	839	0	0	Singlet	Adipocyte

SAT_26M	1263.7	939	4.2589659	1 Singlet	Immune_cells
SAT_26M	3422	1947	3.9819089	9 Singlet	Mesothelial
SAT_26M	1196.7	781	0.7749763	0 Singlet	Adipocyte
SAT_26M	2418.8	1435	5.0327437	7 Singlet	Endothelial_cells
SAT_26M	1744.8	1101	6.0631535	8 Singlet	Mesothelial
SAT_26M	3725.2	1593	0.4136636	10 Singlet	Immune_cells
SAT_26M	4166.5	2057	12.64724	9 Singlet	Mesothelial
SAT_26M	2638.2	1420	1.2709316	0 Singlet	Adipocyte
SAT_26M	1450.6	826	7.4301414	0 Singlet	Adipocyte
SAT_26M	1058	670	9.5797618	9 Singlet	Mesothelial
SAT_26M	2180.5	1125	2.7528449	7 Singlet	Endothelial_cells
SAT_26M	1394.5	937	0.2299789	0 Singlet	Adipocyte
SAT_26M	2076.1	1164	2.9330092	0 Singlet	Adipocyte
SAT_26M	4927.6	2292	0.3069921	0 Singlet	Adipocyte
SAT_26M	1515.6	1058	0.7135644	4 Singlet	Immune_cells
SAT_26M	4683.8	2310	0.0317713	0 Singlet	Adipocyte
SAT_26M	1057.4	741	0	0 Singlet	Adipocyte
SAT_26M	1225	582	10.4696	0 Singlet	Adipocyte
SAT_26M	1021.3	709	0.1282491	8 Singlet	Mesothelial
SAT_26M	1454.4	918	0.6549888	0 Singlet	Adipocyte
SAT_26M	2436.6	1403	7.3713061	0 Singlet	Adipocyte
SAT_26M	2651.5	1774	2.2230359	4 Singlet	Immune_cells
SAT_26M	2399	1352	0	8 Singlet	Mesothelial
SAT_26M	2321.4	1278	10.359747	7 Singlet	Endothelial_cells
SAT_26M	2509.3	1340	6.0461748	0 Singlet	Adipocyte
SAT_26M	1179.9	746	3.52827	0 Singlet	Adipocyte
SAT_26M	1241.4	890	0	8 Singlet	Mesothelial
SAT_26M	1442.6	901	3.9230972	0 Singlet	Adipocyte
SAT_26M	2006.2	1191	4.5302164	7 Singlet	Endothelial_cells
SAT_26M	1078	701	1.0990046	0 Singlet	Adipocyte
SAT_26M	1492.8	965	2.4888305	5 Singlet	Immune_cells
SAT_26M	4372.8	2381	3.2475937	8 Singlet	Mesothelial
SAT_26M	2739.6	1377	8.6108429	7 Singlet	Endothelial_cells
SAT_26M	1746.4	1102	0.1245796	0 Singlet	Adipocyte
SAT_26M	1067.9	741	0.2463793	0 Singlet	Adipocyte
SAT_26M	4507.8	2228	1.6495785	0 Singlet	Adipocyte
SAT_26M	6666.1	2997	1.4430116	0 Singlet	Adipocyte
SAT_26M	1427.8	891	6.0576211	7 Singlet	Endothelial_cells
SAT_26M	1406.4	991	0	4 Singlet	Immune_cells
SAT_26M	1284.7	906	0.6845251	0 Singlet	Adipocyte
SAT_26M	1282.3	808	0.2553412	5 Singlet	Immune_cells
SAT_26M	1138.3	814	0	8 Singlet	Mesothelial
SAT_26M	1573.6	1156	0.5606447	5 Singlet	Immune_cells
SAT_26M	1473.3	916	2.3559286	0 Singlet	Adipocyte
SAT_26M	1953.3	1286	1.6356073	8 Singlet	Mesothelial
SAT_26M	1827	1102	0.4736871	0 Singlet	Adipocyte
SAT_26M	1017.1	634	3.5898967	5 Singlet	Immune_cells
SAT_26M	1462	1087	0.443963	1 Singlet	Immune_cells
SAT_26M	1977.6	1053	6.2732343	7 Singlet	Endothelial_cells
SAT_26M	3676.7	1494	12.103594	0 Singlet	Adipocyte
SAT_26M	1059.1	712	0.4689874	0 Singlet	Adipocyte

SAT_26M	1453.2	947	2.3932671	2	Singlet	FAPs
SAT_26M	2667.1	1375	0.8397382	10	Singlet	Immune_cells
SAT_26M	2203	1401	1.54499	5	Singlet	Immune_cells
SAT_26M	1369.5	851	11.450927	7	Singlet	Endothelial_cells
SAT_26M	1232	803	1.7167306	0	Singlet	Adipocyte
SAT_26M	1165.5	809	0.8126925	0	Singlet	Adipocyte
SAT_26M	1332.1	860	0.113662	0	Singlet	Adipocyte
SAT_26M	1321.7	863	0.3587271	0	Singlet	Adipocyte
SAT_26M	1067.4	744	10.334486	4	Singlet	Immune_cells
SAT_26M	3412.7	1764	0.0572579	0	Singlet	Adipocyte
SAT_26M	3322.3	1765	10.049492	9	Singlet	Mesothelial
SAT_26M	2984.3	1439	9.7471178	0	Singlet	Adipocyte
SAT_26M	3423.3	1846	1.9295861	7	Singlet	Endothelial_cells
SAT_26M	1021.5	618	10.563205	5	Singlet	Immune_cells
SAT_26M	2319.8	1402	3.4454904	0	Singlet	Adipocyte
SAT_26M	3246.8	1689	0.108936	0	Singlet	Adipocyte
SAT_26M	1153.5	818	0.335916	0	Singlet	Adipocyte
SAT_26M	1052.5	690	4.8553986	0	Singlet	Adipocyte
SAT_26M	6730.6	2872	3.2444395	0	Singlet	Adipocyte
SAT_26M	1463.3	790	6.1343293	0	Singlet	Adipocyte
SAT_26M	3808.2	1758	11.589663	9	Singlet	Mesothelial
VAT_2M	4283.6	2043	1.4372919	0	Singlet	Adipocyte
VAT_2M	5674.4	2205	0	0	Singlet	Adipocyte
VAT_2M	4005.6	1818	1.1536038	0	Singlet	Adipocyte
VAT_2M	1990.7	1257	1.2660856	2	Singlet	FAPs
VAT_2M	1179.9	966	0.0058415	3	Singlet	FAPs
VAT_2M	2489.5	998	12.244657	2	Singlet	FAPs
VAT_2M	1048.1	530	12.774838	6	Singlet	Neurons
VAT_2M	1984.5	1236	0.194229	3	Singlet	FAPs
VAT_2M	6889.3	2679	3.4161099	0	Singlet	Adipocyte
VAT_2M	3116	1605	0	0	Singlet	Adipocyte
VAT_2M	1966.6	1251	0	0	Singlet	Adipocyte
VAT_2M	1755	903	12.478018	3	Singlet	FAPs
VAT_2M	1842.2	1298	14.599738	2	Singlet	FAPs
VAT_2M	1710.6	874	7.1696317	0	Singlet	Adipocyte
VAT_2M	8842.5	2883	13.099659	0	Singlet	Adipocyte
VAT_2M	1848.8	963	9.9872461	0	Singlet	Adipocyte
VAT_2M	6015	2564	3.4750349	0	Singlet	Adipocyte
VAT_2M	1174.5	878	0.0789406	0	Singlet	Adipocyte
VAT_2M	4823.3	2095	0.2806164	0	Singlet	Adipocyte
VAT_2M	2291	1390	1.3052465	0	Singlet	Adipocyte
VAT_2M	1733.4	1043	0.4773212	0	Singlet	Adipocyte
VAT_2M	2038	1142	8.8828272	0	Singlet	Adipocyte
VAT_2M	1013.8	706	9.9535806	9	Singlet	Mesothelial
VAT_2M	8019.6	2933	0	0	Singlet	Adipocyte
VAT_2M	3938.3	1778	13.777396	3	Singlet	FAPs
VAT_2M	4092.7	1832	0.4728298	0	Singlet	Adipocyte
VAT_2M	2240.3	1227	0.2404742	0	Singlet	Adipocyte
VAT_2M	1000.8	646	10.672822	0	Singlet	Adipocyte
VAT_2M	2357.8	1285	2.1792135	0	Singlet	Adipocyte
VAT_2M	1891.1	956	10.078137	5	Singlet	Immune_cells

VAT_2M	1422.8	903	0.0316282	0 Singlet	Adipocyte
VAT_2M	1022.2	667	10.50115	0 Singlet	Adipocyte
VAT_2M	2833	1449	0.0302846	0 Singlet	Adipocyte
VAT_2M	3402.5	1499	12.176964	0 Singlet	Adipocyte
VAT_2M	1115.6	646	8.5443668	2 Singlet	FAPs
VAT_2M	3072.2	1509	0	0 Singlet	Adipocyte
VAT_2M	3621.4	1780	2.8200715	0 Singlet	Adipocyte
VAT_2M	1388.5	902	2.4689664	0 Singlet	Adipocyte
VAT_2M	3759.1	1859	6.6261496	2 Singlet	FAPs
VAT_2M	4189.4	1931	2.4155722	0 Singlet	Adipocyte
VAT_2M	1124.5	769	0.2397484	2 Singlet	FAPs
VAT_2M	2807.3	1379	7.4729003	0 Singlet	Adipocyte
VAT_2M	1466.7	941	4.9440471	2 Singlet	FAPs
VAT_2M	1035	645	4.8447662	3 Singlet	FAPs
VAT_2M	1792.5	870	9.6628241	0 Singlet	Adipocyte
VAT_2M	1052.4	651	13.817281	5 Singlet	Immune_cells
VAT_2M	1036.1	755	0.2137343	0 Singlet	Adipocyte
VAT_2M	1305.5	820	9.3642895	0 Singlet	Adipocyte
VAT_2M	1024.3	886	0.123591	5 Singlet	Immune_cells
VAT_2M	1297.7	760	0.5323334	0 Singlet	Adipocyte
VAT_2M	2302.6	1251	0.233806	0 Singlet	Adipocyte
VAT_2M	4596	2210	5.4088067	0 Singlet	Adipocyte
VAT_2M	3375.1	1687	7.4235787	0 Singlet	Adipocyte
VAT_2M	1570.2	1004	0.1359979	0 Singlet	Adipocyte
VAT_2M	1520	942	2.2982301	0 Singlet	Adipocyte
VAT_2M	1946.4	1075	10.683302	0 Singlet	Adipocyte
VAT_2M	1093.4	821	13.060516	4 Singlet	Immune_cells
VAT_2M	2031.3	1161	1.0940176	0 Singlet	Adipocyte
VAT_2M	1066.4	740	7.3100334	5 Singlet	Immune_cells
VAT_2M	1602.9	1218	0.8754878	6 Singlet	Neurons
VAT_2M	3389.5	1576	8.9380805	0 Singlet	Adipocyte
VAT_2M	2047.7	1106	8.0672558	3 Singlet	FAPs
VAT_2M	2355.4	1376	0.310621	0 Singlet	Adipocyte
VAT_2M	1324	860	0.0591332	3 Singlet	FAPs
VAT_2M	1929.8	1307	2.755405	5 Singlet	Immune_cells
VAT_2M	1504.8	1010	0.7546308	5 Singlet	Immune_cells
VAT_2M	1124.9	664	0.3012102	0 Singlet	Adipocyte
VAT_2M	1123.2	545	14.338258	9 Singlet	Mesothelial
VAT_2M	2602.1	1444	4.873963	0 Singlet	Adipocyte
VAT_2M	1261.3	794	0.3005346	0 Singlet	Adipocyte
VAT_2M	3737.4	1780	5.0410127	0 Singlet	Adipocyte
VAT_2M	2634	1341	0.051419	0 Singlet	Adipocyte
VAT_2M	3732.7	1846	0.0862023	0 Singlet	Adipocyte
VAT_2M	4561.8	2194	0.0511435	0 Singlet	Adipocyte
VAT_2M	3320.1	1733	2.2399636	0 Singlet	Adipocyte
VAT_2M	1314.2	849	3.0434	6 Singlet	Neurons
VAT_2M	3024	1510	0.0188544	0 Singlet	Adipocyte
VAT_2M	2324.4	1328	0.1486236	0 Singlet	Adipocyte
VAT_2M	1378.8	941	1.3427414	5 Singlet	Immune_cells
VAT_2M	2531.7	1404	0.0778422	0 Singlet	Adipocyte
VAT_2M	1191.4	748	11.854482	0 Singlet	Adipocyte

VAT_2M	1297.1	923	0.1305425	5	Singlet	Immune_cells
VAT_2M	2605.7	1471	0.6830732	0	Singlet	Adipocyte
VAT_2M	2190.8	1239	12.512452	0	Singlet	Adipocyte
VAT_2M	7676.9	3002	3.5591126	0	Singlet	Adipocyte
VAT_2M	3674.9	1576	5.2048831	0	Singlet	Adipocyte
VAT_2M	3960.6	1767	0.1497699	0	Singlet	Adipocyte
VAT_2M	1368	904	7.0449607	5	Singlet	Immune_cells
VAT_2M	6854.1	2792	6.4233695	0	Singlet	Adipocyte
VAT_2M	2173.9	1063	10.066628	5	Singlet	Immune_cells
VAT_2M	1216.9	922	0.5572752	0	Singlet	Adipocyte
VAT_2M	1587.8	991	2.6903851	0	Singlet	Adipocyte
VAT_2M	1768.3	1012	0	0	Singlet	Adipocyte
VAT_2M	1063.8	889	4.6923884	6	Singlet	Neurons
VAT_2M	4405.1	2083	0.0100465	0	Singlet	Adipocyte
VAT_2M	2235.4	1521	5.4774576	2	Singlet	FAPs
VAT_2M	3292.8	1652	10.970721	0	Singlet	Adipocyte
VAT_2M	1454.7	1000	0.1332793	7	Singlet	Endothelial_cells
VAT_2M	3244.5	1644	3.8506271	0	Singlet	Adipocyte
VAT_2M	1205.7	815	0.1901514	6	Singlet	Neurons
VAT_2M	1214	866	0	0	Singlet	Adipocyte
VAT_2M	2794.9	1393	7.0294008	0	Singlet	Adipocyte
VAT_2M	1176.3	765	0.1990179	0	Singlet	Adipocyte
VAT_2M	1560.9	919	4.5392708	0	Singlet	Adipocyte
VAT_2M	1540.1	972	4.6490103	0	Singlet	Adipocyte
VAT_2M	1188.1	977	5.6941228	6	Singlet	Neurons
VAT_2M	1061.8	684	0.1394725	5	Singlet	Immune_cells
VAT_2M	1988.4	1094	8.6746823	3	Singlet	FAPs
VAT_2M	1289.8	1136	0.032055	6	Singlet	Neurons
VAT_2M	3510.2	1700	0.1135792	0	Singlet	Adipocyte
VAT_2M	2335.7	1425	0	0	Singlet	Adipocyte
VAT_2M	1520.7	957	9.2318775	2	Singlet	FAPs
VAT_2M	3885.3	1924	0	0	Singlet	Adipocyte
VAT_2M	1518.2	905	2.4967443	0	Singlet	Adipocyte
VAT_2M	1782.8	1027	14.668237	0	Singlet	Adipocyte
VAT_2M	1495.9	930	5.1948244	0	Singlet	Adipocyte
VAT_2M	1286.5	926	8.2842614	2	Singlet	FAPs
VAT_2M	3078.1	1512	0.0865014	0	Singlet	Adipocyte
VAT_2M	5419.4	2233	6.2029161	0	Singlet	Adipocyte
VAT_2M	4107	2067	0	0	Singlet	Adipocyte
VAT_2M	1396.7	825	0.343187	0	Singlet	Adipocyte
VAT_2M	2462.7	1309	0	0	Singlet	Adipocyte
VAT_2M	2666.6	1503	1.2028268	0	Singlet	Adipocyte
VAT_2M	1485	1064	8.3436843	5	Singlet	Immune_cells
VAT_2M	1121.9	682	9.8442284	0	Singlet	Adipocyte
VAT_2M	2042.7	1166	0.3307931	0	Singlet	Adipocyte
VAT_2M	1467.4	868	6.3910084	0	Singlet	Adipocyte
VAT_2M	1269.8	1060	5.106351	6	Singlet	Neurons
VAT_2M	1547.9	895	8.0810663	0	Singlet	Adipocyte
VAT_2M	1649.2	1010	5.5809267	3	Singlet	FAPs
VAT_2M	3533.2	1801	4.9260707	0	Singlet	Adipocyte
VAT_2M	2059.4	1205	1.3444689	0	Singlet	Adipocyte

VAT_2M	6315.9	2446	5.6894358	0	Singlet	Adipocyte
VAT_2M	3161.2	1554	0.1392345	0	Singlet	Adipocyte
VAT_2M	4604.1	2043	8.0903132	0	Singlet	Adipocyte
VAT_2M	2054.8	1212	1.8136417	0	Singlet	Adipocyte
VAT_2M	1849.6	1126	12.879877	2	Singlet	FAPs
VAT_2M	1516.9	979	4.6526889	0	Singlet	Adipocyte
VAT_2M	1809	1142	0.9655347	0	Singlet	Adipocyte
VAT_2M	1541.4	938	0.4711227	0	Singlet	Adipocyte
VAT_2M	2051.2	1125	0.0510928	0	Singlet	Adipocyte
VAT_2M	1202.2	884	0	3	Singlet	FAPs
VAT_2M	1428.3	855	13.974938	0	Singlet	Adipocyte
VAT_2M	2151.6	1229	0.1994206	0	Singlet	Adipocyte
VAT_2M	1121.9	921	0.0374547	6	Singlet	Neurons
VAT_2M	3486.3	1833	0.0751375	0	Singlet	Adipocyte
VAT_2M	1744.3	945	11.206547	5	Singlet	Immune_cells
VAT_2M	1347.3	782	0.2729221	0	Singlet	Adipocyte
VAT_2M	2699.3	1334	8.2628706	0	Singlet	Adipocyte
VAT_2M	2102.2	1158	14.907793	2	Singlet	FAPs
VAT_2M	3196.5	1770	0.2693293	0	Singlet	Adipocyte
VAT_2M	1203.2	791	9.8486021	3	Singlet	FAPs
VAT_2M	2809.5	1371	3.5693427	0	Singlet	Adipocyte
VAT_2M	3210.4	1756	0.2693305	0	Singlet	Adipocyte
VAT_2M	2211	1251	0.0943419	0	Singlet	Adipocyte
VAT_2M	4418.4	2373	1.4795549	2	Singlet	FAPs
VAT_2M	1120.7	710	0.1315186	0	Singlet	Adipocyte
VAT_2M	1247.7	727	0.0198655	0	Singlet	Adipocyte
VAT_2M	1686.8	1134	6.7787347	1	Singlet	Immune_cells
VAT_2M	1341.6	788	14.725452	0	Singlet	Adipocyte
VAT_2M	2706.1	1419	5.8413304	0	Singlet	Adipocyte
VAT_2M	1727.8	1258	3.1530491	3	Singlet	FAPs
VAT_2M	1348.3	933	0.5583771	5	Singlet	Immune_cells
VAT_2M	1405.3	975	2.8108041	0	Singlet	Adipocyte
VAT_2M	2315.9	1245	10.567595	0	Singlet	Adipocyte
VAT_2M	1544.8	967	0.2041856	0	Singlet	Adipocyte
VAT_2M	1485.2	898	13.293858	0	Singlet	Adipocyte
VAT_2M	1140.8	681	12.701306	3	Singlet	FAPs
VAT_2M	3717.4	1781	0	0	Singlet	Adipocyte
VAT_2M	1297.3	931	4.1049635	0	Singlet	Adipocyte
VAT_2M	1706.8	1013	0	0	Singlet	Adipocyte
VAT_2M	1060.5	654	12.159425	0	Singlet	Adipocyte
VAT_2M	1442.2	1084	0	0	Singlet	Adipocyte
VAT_2M	1781.5	994	0.2104762	0	Singlet	Adipocyte
VAT_2M	1150	822	6.0552686	6	Singlet	Neurons
VAT_2M	3407.5	1627	0.0153739	0	Singlet	Adipocyte
VAT_2M	2595.7	1321	5.8608885	0	Singlet	Adipocyte
VAT_2M	1580.7	925	0	0	Singlet	Adipocyte
VAT_2M	1454.7	1056	0	3	Singlet	FAPs
VAT_2M	1877.9	1079	0	0	Singlet	Adipocyte
VAT_2M	1568.7	1080	0.4249897	2	Singlet	FAPs
VAT_2M	2215.4	1271	0.0140651	0	Singlet	Adipocyte
VAT_2M	1970	1292	0.9585272	1	Singlet	Immune_cells

VAT_2M	4383.6	2018	1.1548828	0	Singlet	Adipocyte
VAT_2M	1024.7	640	6.9226722	3	Singlet	FAPs
VAT_2M	2140	1260	1.0749842	0	Singlet	Adipocyte
VAT_2M	1826.4	1109	5.9128117	0	Singlet	Adipocyte
VAT_2M	1717.4	853	13.929253	0	Singlet	Adipocyte
VAT_2M	1516.8	1141	3.0961934	2	Singlet	FAPs
VAT_2M	2437.1	1332	0.4556722	0	Singlet	Adipocyte
VAT_2M	5282.9	2307	1.0818419	0	Singlet	Adipocyte
VAT_2M	1869.5	1089	0.0394175	0	Singlet	Adipocyte
VAT_2M	2841.3	1459	8.3456559	5	Singlet	Immune_cells
VAT_2M	4997.4	2259	3.3400025	0	Singlet	Adipocyte
VAT_2M	3249.4	1699	0	0	Singlet	Adipocyte
VAT_2M	2198.1	1471	6.8468808	5	Singlet	Immune_cells
VAT_2M	1609.1	964	2.1002477	0	Singlet	Adipocyte
VAT_2M	4630.9	2151	0.4488049	0	Singlet	Adipocyte
VAT_2M	2321.3	1387	0.0670478	0	Singlet	Adipocyte
VAT_2M	2796.6	1626	1.2082791	0	Singlet	Adipocyte
VAT_2M	1210.6	791	8.0958712	0	Singlet	Adipocyte
VAT_2M	1824.4	1191	0.0670955	2	Singlet	FAPs
VAT_2M	1142.5	774	0.1910059	0	Singlet	Adipocyte
VAT_2M	1055.2	751	2.3978615	0	Singlet	Adipocyte
VAT_2M	1916.7	1084	6.8595951	0	Singlet	Adipocyte
VAT_2M	2953.1	1445	3.312332	0	Singlet	Adipocyte
VAT_2M	3738.2	1838	0.3113659	0	Singlet	Adipocyte
VAT_2M	1493.6	1039	0.245175	3	Singlet	FAPs
VAT_2M	4865.6	1969	14.461594	0	Singlet	Adipocyte
VAT_2M	1103.5	764	2.3222926	0	Singlet	Adipocyte
VAT_2M	3910.8	1882	0.0809139	0	Singlet	Adipocyte
VAT_2M	5480.3	2174	7.5542964	0	Singlet	Adipocyte
VAT_2M	1086.3	908	0.4599495	3	Singlet	FAPs
VAT_2M	4372.3	1779	1.4924899	0	Singlet	Adipocyte
VAT_2M	1374.2	1063	6.0373177	5	Singlet	Immune_cells
VAT_2M	2643.4	1376	0.0070912	0	Singlet	Adipocyte
VAT_2M	1256.8	756	7.5707234	0	Singlet	Adipocyte
VAT_2M	2294.5	1263	0.8657775	0	Singlet	Adipocyte
VAT_2M	2794	1361	4.7310091	0	Singlet	Adipocyte
VAT_2M	2628.7	1401	0	0	Singlet	Adipocyte
VAT_2M	4519.6	2145	0.1145223	0	Singlet	Adipocyte
VAT_2M	1735.4	955	11.742627	5	Singlet	Immune_cells
VAT_2M	1096.5	737	10.954907	3	Singlet	FAPs
VAT_2M	1623.2	1015	0.1275232	3	Singlet	FAPs
VAT_2M	2095.1	1143	0.6351956	0	Singlet	Adipocyte
VAT_2M	1368.4	883	0.0054912	0	Singlet	Adipocyte
VAT_2M	1496.5	831	11.022951	0	Singlet	Adipocyte
VAT_2M	1750.6	1081	0.0648683	7	Singlet	Endothelial_cells
VAT_2M	1907.6	1029	0.2908578	0	Singlet	Adipocyte
VAT_2M	1747.4	1202	8.9756465	5	Singlet	Immune_cells
VAT_2M	3642.5	1843	4.9358262	0	Singlet	Adipocyte
VAT_2M	2634.4	1373	0.1009906	0	Singlet	Adipocyte
VAT_2M	9650.2	3243	8.3815099	0	Singlet	Adipocyte
VAT_2M	1581.9	1001	0.0092646	0	Singlet	Adipocyte

VAT_2M	1329.7	856	4.8979405	0	Singlet	Adipocyte
VAT_2M	3911.1	1860	9.748301	0	Singlet	Adipocyte
VAT_2M	6846.7	2654	3.2057164	0	Singlet	Adipocyte
VAT_2M	1701.5	1001	0.0450205	0	Singlet	Adipocyte
VAT_2M	5389.8	2182	5.6689733	0	Singlet	Adipocyte
VAT_2M	1808.4	1004	11.772085	0	Singlet	Adipocyte
VAT_2M	1777.6	1010	2.0853708	0	Singlet	Adipocyte
VAT_2M	2069.4	1201	0	0	Singlet	Adipocyte
VAT_2M	1013.2	763	0.0528659	0	Singlet	Adipocyte
VAT_2M	1868.2	1171	1.6207061	0	Singlet	Adipocyte
VAT_2M	2555.4	1401	1.713325	0	Singlet	Adipocyte
VAT_2M	1160.2	921	2.8222546	1	Singlet	Immune_cells
VAT_2M	1158.7	656	11.86757	2	Singlet	FAPs
VAT_2M	1734.5	1037	6.3756732	2	Singlet	FAPs
VAT_2M	1792.8	1068	9.5916615	2	Singlet	FAPs
VAT_2M	2022.7	1161	0.0231906	0	Singlet	Adipocyte
VAT_2M	1303.3	787	14.091591	5	Singlet	Immune_cells
VAT_2M	1103.4	729	2.1907153	0	Singlet	Adipocyte
VAT_2M	1020.4	707	7.0602024	0	Singlet	Adipocyte
VAT_2M	1248.4	852	0.035407	0	Singlet	Adipocyte
VAT_2M	1378.5	890	5.5183863	0	Singlet	Adipocyte
VAT_2M	4258.2	1943	0.0721149	0	Singlet	Adipocyte
VAT_2M	1363.4	857	0.7743284	5	Singlet	Immune_cells
VAT_2M	2338	1391	1.3964789	3	Singlet	FAPs
VAT_2M	1292.7	779	12.762885	5	Singlet	Immune_cells
VAT_2M	4272.3	2052	5.032999	0	Singlet	Adipocyte
VAT_2M	1528.4	961	3.0495919	5	Singlet	Immune_cells
VAT_2M	1037.1	813	0.0244689	0	Singlet	Adipocyte
VAT_2M	1611.7	975	2.3189167	5	Singlet	Immune_cells
VAT_2M	1024.3	754	5.5736746	2	Singlet	FAPs
VAT_2M	1673	1051	1.6887924	5	Singlet	Immune_cells
VAT_2M	2089.7	1208	9.0450424	1	Singlet	Immune_cells
VAT_2M	1127.8	690	3.5760162	5	Singlet	Immune_cells
VAT_2M	1841.7	1158	5.2358599	3	Singlet	FAPs
VAT_2M	2717	1744	0.0464127	5	Singlet	Immune_cells
VAT_2M	3241.4	1643	5.391494	0	Singlet	Adipocyte
VAT_2M	1201.4	1037	0	6	Singlet	Neurons
VAT_2M	1327.5	958	0.0352503	0	Singlet	Adipocyte
VAT_2M	4033.9	1954	0.1087469	0	Singlet	Adipocyte
VAT_2M	2286.5	1460	1.0403535	5	Singlet	Immune_cells
VAT_2M	1398.4	1030	2.7828721	3	Singlet	FAPs
VAT_2M	6447.4	2666	0	0	Singlet	Adipocyte
VAT_2M	1119.6	764	0.0609127	7	Singlet	Endothelial_cells
VAT_2M	7063	2635	0	0	Singlet	Adipocyte
VAT_2M	1159.6	977	3.7587193	6	Singlet	Neurons
VAT_2M	1836.6	1237	0.2378404	3	Singlet	FAPs
VAT_2M	1866.6	1093	2.1699577	0	Singlet	Adipocyte
VAT_2M	1617.5	1157	0	6	Singlet	Neurons
VAT_2M	1205.2	892	2.4825062	0	Singlet	Adipocyte
VAT_2M	3180.4	1616	0	0	Singlet	Adipocyte
VAT_2M	7833.3	2743	0.4504848	0	Singlet	Adipocyte

VAT_2M	1395.1	888	0.0876589	0	Singlet	Adipocyte
VAT_2M	3268.1	1550	0	0	Singlet	Adipocyte
VAT_2M	1227.9	992	0.452771	2	Singlet	FAPs
VAT_2M	1301	951	7.459254	0	Singlet	Adipocyte
VAT_2M	2102.4	1265	3.7082348	0	Singlet	Adipocyte
VAT_2M	8635.1	3109	1.3436674	0	Singlet	Adipocyte
VAT_2M	1725.3	1111	5.4692715	2	Singlet	FAPs
VAT_2M	3577.1	1664	11.997562	0	Singlet	Adipocyte
VAT_2M	1067.9	786	0.1268565	5	Singlet	Immune_cells
VAT_2M	1200.9	808	0	0	Singlet	Adipocyte
VAT_2M	1400.9	975	1.1353994	0	Singlet	Adipocyte
VAT_2M	2720.6	1245	13.456226	0	Singlet	Adipocyte
VAT_2M	3608.7	1653	9.0685669	0	Singlet	Adipocyte
VAT_2M	1865	1039	2.0043731	0	Singlet	Adipocyte
VAT_2M	3124.2	1732	0	0	Singlet	Adipocyte
VAT_2M	2154.1	1231	0.1999934	0	Singlet	Adipocyte
VAT_2M	3076.2	1657	6.066773	0	Singlet	Adipocyte
VAT_2M	1142.4	731	0.4629075	0	Singlet	Adipocyte
VAT_2M	1950.2	996	14.058971	0	Singlet	Adipocyte
VAT_2M	3083.6	1635	10.99261	2	Singlet	FAPs
VAT_2M	1692.8	1104	7.4603751	2	Singlet	FAPs
VAT_2M	1002	917	3.8127543	6	Singlet	Neurons
VAT_2M	1018.3	705	8.4892563	2	Singlet	FAPs
VAT_2M	4372.4	2059	0.1356927	0	Singlet	Adipocyte
VAT_2M	1967.6	1099	0.9271677	0	Singlet	Adipocyte
VAT_2M	1767.1	1002	0.0394465	0	Singlet	Adipocyte
VAT_2M	5517.1	2245	12.068515	0	Singlet	Adipocyte
VAT_2M	1554.3	973	0.5967581	0	Singlet	Adipocyte
VAT_2M	2092.2	1139	0.314174	0	Singlet	Adipocyte
VAT_2M	5635.3	2345	0.0031011	0	Singlet	Adipocyte
VAT_2M	1752.5	949	0.1021172	0	Singlet	Adipocyte
VAT_2M	5951.7	2453	0.4718288	3	Singlet	FAPs
VAT_2M	1876.7	1149	0.1192308	0	Singlet	Adipocyte
VAT_2M	2295.4	1377	9.097318	2	Singlet	FAPs
VAT_2M	1324	804	3.4940639	0	Singlet	Adipocyte
VAT_2M	3017.2	1530	0.2895939	0	Singlet	Adipocyte
VAT_2M	1797.5	1423	2.1508695	6	Singlet	Neurons
VAT_2M	4006.4	2028	0.0534748	0	Singlet	Adipocyte
VAT_2M	4594.5	1981	1.6903769	0	Singlet	Adipocyte
VAT_2M	2388.6	1221	7.6606458	0	Singlet	Adipocyte
VAT_2M	1306.6	892	0	3	Singlet	FAPs
VAT_2M	1461.8	942	14.251934	0	Singlet	Adipocyte
VAT_2M	1166.8	803	1.441973	3	Singlet	FAPs
VAT_2M	1073.8	716	12.366583	5	Singlet	Immune_cells
VAT_2M	1215.1	818	12.133716	0	Singlet	Adipocyte
VAT_2M	2224	1445	2.7238583	3	Singlet	FAPs
VAT_2M	3722.2	1815	0.0660578	0	Singlet	Adipocyte
VAT_2M	7560.8	2961	1.8800688	0	Singlet	Adipocyte
VAT_2M	1172	536	10.986446	5	Singlet	Immune_cells
VAT_2M	2528.4	1431	2.1109864	0	Singlet	Adipocyte
VAT_2M	2726.1	1390	7.7706552	0	Singlet	Adipocyte

VAT_2M	1575.9	1011	1.4813188	0	Singlet	Adipocyte
VAT_2M	1655.6	858	5.3392008	0	Singlet	Adipocyte
VAT_2M	4019.9	1857	1.3422773	0	Singlet	Adipocyte
VAT_2M	1113.3	871	4.1179951	5	Singlet	Immune_cells
VAT_2M	1245	893	0	0	Singlet	Adipocyte
VAT_2M	2774.1	1531	0.0151615	0	Singlet	Adipocyte
VAT_2M	4685.2	2037	5.4853021	0	Singlet	Adipocyte
VAT_2M	1087.2	890	5.9310772	6	Singlet	Neurons
VAT_2M	1183.4	813	3.7884069	5	Singlet	Immune_cells
VAT_2M	3927.1	1803	0.1953376	0	Singlet	Adipocyte
VAT_2M	2003.1	1108	0.5416089	0	Singlet	Adipocyte
VAT_2M	1239.8	743	2.6041634	0	Singlet	Adipocyte
VAT_2M	1864	986	11.301787	3	Singlet	FAPs
VAT_2M	1221.4	819	0	0	Singlet	Adipocyte
VAT_2M	1906	1056	11.040285	3	Singlet	FAPs
VAT_2M	1060.5	674	6.9352588	0	Singlet	Adipocyte
VAT_2M	4012.4	1727	10.978138	0	Singlet	Adipocyte
VAT_2M	2581.6	1506	0	3	Singlet	FAPs
VAT_2M	1310.7	771	1.0274601	0	Singlet	Adipocyte
VAT_2M	2717.5	1505	0.5541365	0	Singlet	Adipocyte
VAT_2M	2177.7	1267	1.08476	0	Singlet	Adipocyte
VAT_2M	1815.7	1239	0.7900329	0	Singlet	Adipocyte
VAT_2M	1320.4	871	0.1325019	0	Singlet	Adipocyte
VAT_2M	2258.6	1472	3.2348238	7	Singlet	Endothelial_cells
VAT_2M	1502.3	957	0.2558926	0	Singlet	Adipocyte
VAT_2M	1884	1171	0.9586302	3	Singlet	FAPs
VAT_2M	1046.7	775	2.0188866	5	Singlet	Immune_cells
VAT_2M	3914	2149	0.4639149	0	Singlet	Adipocyte
VAT_2M	1240.9	716	14.897574	0	Singlet	Adipocyte
VAT_2M	1316.2	872	0.3594285	0	Singlet	Adipocyte
VAT_2M	1275.9	581	4.8320644	6	Singlet	Neurons
VAT_2M	2148.7	1289	0.6694395	0	Singlet	Adipocyte
VAT_2M	1459.3	857	0.0288613	0	Singlet	Adipocyte
VAT_2M	1434.9	1044	5.1591193	0	Singlet	Adipocyte
VAT_2M	1533.1	920	5.9119358	0	Singlet	Adipocyte
VAT_2M	3726.4	1769	0.0127267	0	Singlet	Adipocyte
VAT_2M	1551.4	862	9.2931872	5	Singlet	Immune_cells
VAT_2M	6272	2127	6.9856205	0	Singlet	Adipocyte
VAT_2M	1910.5	1085	1.126452	0	Singlet	Adipocyte
VAT_2M	1866.7	1268	1.0936766	7	Singlet	Endothelial_cells
VAT_2M	1209	719	9.4287741	0	Singlet	Adipocyte
VAT_2M	1014.8	792	0.237821	5	Singlet	Immune_cells
VAT_2M	1081.8	670	0.2055184	0	Singlet	Adipocyte
VAT_2M	2820.5	1329	0	0	Singlet	Adipocyte
VAT_2M	1344.7	1021	11.692534	6	Singlet	Neurons
VAT_2M	1103.2	838	0.0875916	6	Singlet	Neurons
VAT_2M	2014.6	1194	0.0062597	0	Singlet	Adipocyte
VAT_2M	1698.6	1114	0.1105394	0	Singlet	Adipocyte
VAT_2M	1051.7	809	0.0821112	3	Singlet	FAPs
VAT_2M	7422.1	2669	0.4212622	0	Singlet	Adipocyte
VAT_2M	2640	1337	7.8360961	0	Singlet	Adipocyte

VAT_2M	3350.7	1698	1.7628776	0	Singlet	Adipocyte
VAT_2M	1341.3	1004	0	0	Singlet	Adipocyte
VAT_2M	2897.8	1488	0.3023751	0	Singlet	Adipocyte
VAT_2M	1119.9	721	0.1564967	0	Singlet	Adipocyte
VAT_2M	1199.4	773	0.0025669	0	Singlet	Adipocyte
VAT_2M	6357	2452	0.0410917	0	Singlet	Adipocyte
VAT_2M	1267.3	802	0.186719	0	Singlet	Adipocyte
VAT_2M	3401.9	1693	8.1618951	0	Singlet	Adipocyte
VAT_2M	2585.1	1460	2.2642313	0	Singlet	Adipocyte
VAT_2M	1757.8	1055	6.4963896	0	Singlet	Adipocyte
VAT_2M	5992.2	2406	5.9445855	0	Singlet	Adipocyte
VAT_2M	2985.7	1799	8.3415762	2	Singlet	FAPs
VAT_2M	4360.2	1626	12.637825	0	Singlet	Adipocyte
VAT_2M	5696.3	2483	0.1212679	0	Singlet	Adipocyte
VAT_2M	1176.1	751	4.2041616	3	Singlet	FAPs
VAT_2M	1084.3	664	9.1899171	3	Singlet	FAPs
VAT_2M	1363.1	746	14.434949	7	Singlet	Endothelial_cells
VAT_2M	1817.9	1041	7.5500588	0	Singlet	Adipocyte
VAT_2M	3354.6	1636	1.8518596	0	Singlet	Adipocyte
VAT_2M	1710.6	1116	0.0849186	3	Singlet	FAPs
VAT_2M	2342.2	1156	12.77545	0	Singlet	Adipocyte
VAT_2M	1526.6	942	2.7557904	0	Singlet	Adipocyte
VAT_2M	1282.7	651	12.949477	0	Singlet	Adipocyte
VAT_2M	2273.2	1256	3.3814408	5	Singlet	Immune_cells
VAT_2M	1684.7	997	1.3325886	0	Singlet	Adipocyte
VAT_2M	1912.8	1046	0	0	Singlet	Adipocyte
VAT_2M	3248	1765	0.2870503	0	Singlet	Adipocyte
VAT_2M	2159.4	1255	0	0	Singlet	Adipocyte
VAT_2M	3158.4	1714	0.6001761	0	Singlet	Adipocyte
VAT_2M	1751.1	1018	3.6324903	0	Singlet	Adipocyte
VAT_2M	2087	1101	1.5565794	0	Singlet	Adipocyte
VAT_2M	4219.9	1989	0.0291505	0	Singlet	Adipocyte
VAT_2M	2738.8	1472	0.0696073	0	Singlet	Adipocyte
VAT_2M	1262.4	853	0	3	Singlet	FAPs
VAT_2M	1653.1	909	0.0048821	0	Singlet	Adipocyte
VAT_2M	1127.3	734	2.9879883	0	Singlet	Adipocyte
VAT_2M	1372.5	926	4.5600998	3	Singlet	FAPs
VAT_2M	3901.7	1946	2.0568991	0	Singlet	Adipocyte
VAT_2M	1804.6	979	0.2642374	0	Singlet	Adipocyte
VAT_2M	3209.9	1509	14.37351	0	Singlet	Adipocyte
VAT_2M	1186.4	856	1.2491409	3	Singlet	FAPs
VAT_2M	1527.6	1045	0	0	Singlet	Adipocyte
VAT_2M	1170.1	795	2.3461542	7	Singlet	Endothelial_cells
VAT_2M	1077	731	3.231201	0	Singlet	Adipocyte
VAT_2M	1349.2	814	9.0241645	7	Singlet	Endothelial_cells
VAT_2M	2284.9	1330	2.4305892	0	Singlet	Adipocyte
VAT_2M	1265.8	955	2.3000758	6	Singlet	Neurons
VAT_2M	1577.9	950	1.6626319	0	Singlet	Adipocyte
VAT_2M	1534.5	891	0.0983695	0	Singlet	Adipocyte
VAT_2M	3004.6	1595	0	0	Singlet	Adipocyte
VAT_2M	3664.1	1770	1.9590802	0	Singlet	Adipocyte

VAT_2M	1023.3	749	0.9706897	3	Singlet	FAPs
VAT_2M	1056.8	781	10.431057	5	Singlet	Immune_cells
VAT_2M	2590.1	1398	0.0006071	0	Singlet	Adipocyte
VAT_2M	1808.8	1132	0	3	Singlet	FAPs
VAT_2M	1368.6	829	12.192513	2	Singlet	FAPs
VAT_2M	2034.5	1146	0.1285039	0	Singlet	Adipocyte
VAT_2M	1543.8	1077	0.8156591	3	Singlet	FAPs
VAT_2M	2132.7	1264	9.4112076	0	Singlet	Adipocyte
VAT_2M	1053.7	733	0.7109221	3	Singlet	FAPs
VAT_2M	3582.7	1702	1.8553387	0	Singlet	Adipocyte
VAT_2M	2976.5	1632	2.0205751	0	Singlet	Adipocyte
VAT_2M	1879.4	1098	0	0	Singlet	Adipocyte
VAT_2M	2364.4	1485	0.0747555	7	Singlet	Endothelial_cells
VAT_2M	2274.3	1311	0.5884795	0	Singlet	Adipocyte
VAT_2M	4489.2	2024	10.990605	0	Singlet	Adipocyte
VAT_2M	4784.2	2135	9.5624366	0	Singlet	Adipocyte
VAT_2M	4105.2	1838	1.4159368	0	Singlet	Adipocyte
VAT_2M	2048.5	1150	6.1609065	0	Singlet	Adipocyte
VAT_2M	4233.1	1697	12.938765	0	Singlet	Adipocyte
VAT_2M	2709.4	1502	0	0	Singlet	Adipocyte
VAT_2M	1455.7	911	0.939003	0	Singlet	Adipocyte
VAT_2M	13942	3839	14.15244	0	Singlet	Adipocyte
VAT_2M	5484.2	2258	4.6166355	0	Singlet	Adipocyte
VAT_2M	5571.7	2231	2.5516732	0	Singlet	Adipocyte
VAT_2M	1000.8	744	0	0	Singlet	Adipocyte
VAT_2M	2798.5	1714	1.0641737	2	Singlet	FAPs
VAT_2M	1527.2	960	1.4685408	0	Singlet	Adipocyte
VAT_2M	2259.1	1209	0	0	Singlet	Adipocyte
VAT_2M	1925	1193	4.4908937	0	Singlet	Adipocyte
VAT_2M	7326.6	2597	4.1450366	0	Singlet	Adipocyte
VAT_2M	2658.4	1365	0	0	Singlet	Adipocyte
VAT_2M	3507.2	1504	0.0133251	0	Singlet	Adipocyte
VAT_2M	1474.1	947	2.2327819	2	Singlet	FAPs
VAT_2M	1789.5	1242	10.723438	5	Singlet	Immune_cells
VAT_2M	2301	1109	0	0	Singlet	Adipocyte
VAT_2M	2158.1	1185	0.159419	0	Singlet	Adipocyte
VAT_2M	1304.5	969	0.4110113	3	Singlet	FAPs
VAT_2M	1519.1	957	10.92627	2	Singlet	FAPs
VAT_2M	2528	1378	1.6511466	0	Singlet	Adipocyte
VAT_2M	1870.4	1054	4.3456279	2	Singlet	FAPs
VAT_2M	1367.5	822	0	6	Singlet	Neurons
VAT_2M	1678.1	818	4.1442095	3	Singlet	FAPs
VAT_2M	3040.5	1643	1.0535652	0	Singlet	Adipocyte
VAT_2M	1106.8	740	9.466239	9	Singlet	Mesothelial
VAT_2M	6251.6	1526	12.501562	0	Singlet	Adipocyte
VAT_2M	1034.7	641	3.1381329	0	Singlet	Adipocyte
VAT_2M	1209.1	910	4.5386879	0	Singlet	Adipocyte
VAT_2M	2990	1494	9.8825952	0	Singlet	Adipocyte
VAT_2M	1779.2	1005	0.0711213	0	Singlet	Adipocyte
VAT_2M	1841.8	1133	4.5824172	2	Singlet	FAPs
VAT_2M	1015.2	677	10.880142	0	Singlet	Adipocyte

VAT_2M	1770.2	1238	1.3021053	3	Singlet	FAPs
VAT_2M	2971.8	1152	0.3972142	0	Singlet	Adipocyte
VAT_2M	4180.6	1841	6.2988634	0	Singlet	Adipocyte
VAT_2M	1860.7	1194	0	3	Singlet	FAPs
VAT_2M	1213.6	833	9.0076466	0	Singlet	Adipocyte
VAT_2M	1205.5	813	0.3288885	0	Singlet	Adipocyte
VAT_2M	1816.4	1129	0.1895579	3	Singlet	FAPs
VAT_2M	1647.8	1122	0	5	Singlet	Immune_cells
VAT_2M	1769.6	1073	0.0587465	0	Singlet	Adipocyte
VAT_2M	1321.3	881	0.2697444	0	Singlet	Adipocyte
VAT_2M	3146.2	1606	0	0	Singlet	Adipocyte
VAT_2M	1336.9	1034	0.3994114	6	Singlet	Neurons
VAT_2M	3839.2	1895	0.0233984	0	Singlet	Adipocyte
VAT_2M	3712.8	1683	11.555791	0	Singlet	Adipocyte
VAT_2M	2231.6	1165	11.670502	2	Singlet	FAPs
VAT_2M	5404.9	2306	3.6560387	0	Singlet	Adipocyte
VAT_2M	2214.6	1468	2.7752187	5	Singlet	Immune_cells
VAT_2M	1634	948	0.0469546	0	Singlet	Adipocyte
VAT_2M	1146.9	614	12.977579	0	Singlet	Adipocyte
VAT_2M	1593.3	1400	0.6288648	11	Singlet	Smooth_muscle_cells
VAT_2M	3629.2	1888	0	0	Singlet	Adipocyte
VAT_2M	1256.3	1214	0.1867708	6	Singlet	Neurons
VAT_2M	2881.9	1524	0.2578102	0	Singlet	Adipocyte
VAT_2M	3222.2	1699	1.1903312	0	Singlet	Adipocyte
VAT_2M	1113.7	663	6.4375201	0	Singlet	Adipocyte
VAT_2M	6873.1	2874	0.0192914	0	Singlet	Adipocyte
VAT_2M	2321.6	1281	0.9193489	0	Singlet	Adipocyte
VAT_2M	1182.1	790	5.9025462	5	Singlet	Immune_cells
VAT_2M	2265.3	1266	12.960628	0	Singlet	Adipocyte
VAT_2M	4951.1	2192	1.075882	0	Singlet	Adipocyte
VAT_2M	1823.8	1025	0.7356092	0	Singlet	Adipocyte
VAT_2M	1739.1	975	3.7642386	5	Singlet	Immune_cells
VAT_2M	1850	1085	0.0950842	0	Singlet	Adipocyte
VAT_2M	5523.1	2301	6.7312575	0	Singlet	Adipocyte
VAT_2M	1304.1	820	13.225376	2	Singlet	FAPs
VAT_2M	1421.9	907	2.0473358	0	Singlet	Adipocyte
VAT_2M	1926.2	1238	4.6247345	5	Singlet	Immune_cells
VAT_2M	3302.2	1597	0.0543158	0	Singlet	Adipocyte
VAT_2M	1089.9	864	2.3308847	2	Singlet	FAPs
VAT_2M	4789.4	2176	7.798418	0	Singlet	Adipocyte
VAT_2M	1500.2	1041	0	0	Singlet	Adipocyte
VAT_2M	1193	738	0	4	Singlet	Immune_cells
VAT_2M	1086.9	799	0	0	Singlet	Adipocyte
VAT_2M	1619.1	1492	0	6	Singlet	Neurons
VAT_2M	1056.1	821	0.0883878	0	Singlet	Adipocyte
VAT_2M	2350.8	1522	1.166681	0	Singlet	Adipocyte
VAT_2M	2608.5	1456	0.8591028	7	Singlet	Endothelial_cells
VAT_2M	2217.7	1336	1.2933584	0	Singlet	Adipocyte
VAT_2M	3716.7	2081	0	5	Singlet	Immune_cells
VAT_2M	1038.8	645	4.7306584	3	Singlet	FAPs
VAT_2M	3216.6	1710	0.7861503	0	Singlet	Adipocyte

VAT_2M	1712.3	1086	0	0	Singlet	Adipocyte
VAT_2M	1165.6	840	0	0	Singlet	Adipocyte
VAT_2M	3680.8	1860	0.0245807	0	Singlet	Adipocyte
VAT_2M	4947.9	2168	14.346325	7	Singlet	Endothelial_cells
VAT_2M	1808.3	1089	9.1125455	3	Singlet	FAPs
VAT_2M	1595.9	978	0.0686892	0	Singlet	Adipocyte
VAT_2M	1549.2	853	14.875851	0	Singlet	Adipocyte
VAT_2M	1816	1049	0.6066314	0	Singlet	Adipocyte
VAT_2M	1239.1	811	0	0	Singlet	Adipocyte
VAT_2M	2564.6	1448	0.0158754	0	Singlet	Adipocyte
VAT_2M	1309.6	938	4.6881876	2	Singlet	FAPs
VAT_2M	2141.4	1304	1.1790456	0	Singlet	Adipocyte
VAT_2M	1125.2	699	11.35506	0	Singlet	Adipocyte
VAT_2M	1097.4	667	0.4526315	0	Singlet	Adipocyte
VAT_2M	1088.9	915	0.1326393	7	Singlet	Endothelial_cells
VAT_2M	1027.7	773	9.5100598	3	Singlet	FAPs
VAT_2M	1523.1	921	12.531183	0	Singlet	Adipocyte
VAT_2M	3095	1822	0.9789206	0	Singlet	Adipocyte
VAT_2M	2190.9	1220	0.2549448	0	Singlet	Adipocyte
VAT_2M	1341.6	880	0.3185729	0	Singlet	Adipocyte
VAT_2M	2071	1186	9.3249357	0	Singlet	Adipocyte
VAT_2M	1360	1268	1.7993784	6	Singlet	Neurons
VAT_2M	3949.2	2003	0.4888413	0	Singlet	Adipocyte
VAT_2M	3295.6	1735	5.359347	0	Singlet	Adipocyte
VAT_2M	1936.8	1091	0.6431271	0	Singlet	Adipocyte
VAT_2M	1437	887	0.6089168	0	Singlet	Adipocyte
VAT_2M	2105.7	1164	6.5575606	0	Singlet	Adipocyte
VAT_2M	1834.7	1133	7.9392407	0	Singlet	Adipocyte
VAT_2M	4981.2	2319	0.0054459	0	Singlet	Adipocyte
VAT_2M	5159.3	2278	0.4037267	0	Singlet	Adipocyte
VAT_2M	1208.6	862	0.6592792	0	Singlet	Adipocyte
VAT_2M	2259.4	1231	0	0	Singlet	Adipocyte
VAT_2M	1649.9	1191	1.0986847	0	Singlet	Adipocyte
VAT_2M	2143.4	1343	0.0735564	0	Singlet	Adipocyte
VAT_2M	3170.2	1778	0	3	Singlet	FAPs
VAT_2M	2443.3	1257	0	0	Singlet	Adipocyte
VAT_2M	1050.6	790	0	6	Singlet	Neurons
VAT_2M	2064.8	1242	1.1130565	0	Singlet	Adipocyte
VAT_2M	1917.5	951	12.095185	0	Singlet	Adipocyte
VAT_2M	6404.1	2689	0.0904347	0	Singlet	Adipocyte
VAT_2M	2551.5	1355	1.0487953	0	Singlet	Adipocyte
VAT_2M	1296.4	734	5.0046452	0	Singlet	Adipocyte
VAT_2M	3449.1	1755	0	0	Singlet	Adipocyte
VAT_2M	1020.5	710	0.7080874	5	Singlet	Immune_cells
VAT_2M	1451.4	816	0.4507403	5	Singlet	Immune_cells
VAT_2M	1065.5	776	13.836933	6	Singlet	Neurons
VAT_2M	4426.6	1852	13.792667	0	Singlet	Adipocyte
VAT_2M	1236.9	767	12.063491	0	Singlet	Adipocyte
VAT_2M	2122.6	1261	0.3962143	0	Singlet	Adipocyte
VAT_2M	2363.7	1585	0.0292912	3	Singlet	FAPs
VAT_2M	5344	2221	0.090942	0	Singlet	Adipocyte

VAT_2M	2751	1377	10.896129	0	Singlet	Adipocyte
VAT_2M	2551.6	1495	0	0	Singlet	Adipocyte
VAT_2M	1699	1273	0.2739743	3	Singlet	FAPs
VAT_2M	3443.8	1790	3.1509468	0	Singlet	Adipocyte
VAT_2M	1236	769	6.3665964	2	Singlet	FAPs
VAT_2M	1096.8	661	7.0620843	0	Singlet	Adipocyte
VAT_2M	2435.7	1855	0	6	Singlet	Neurons
VAT_2M	1778.1	1113	0.7981824	0	Singlet	Adipocyte
VAT_2M	1308.5	764	8.0479704	0	Singlet	Adipocyte
VAT_2M	1725.3	1054	0	0	Singlet	Adipocyte
VAT_2M	1239.3	711	14.43028	0	Singlet	Adipocyte
VAT_2M	1710	832	11.721561	5	Singlet	Immune_cells
VAT_2M	4775.8	2106	0.0864879	0	Singlet	Adipocyte
VAT_2M	1595.8	943	3.8300625	2	Singlet	FAPs
VAT_2M	1578.1	969	13.498003	3	Singlet	FAPs
VAT_2M	1109.3	735	1.149341	0	Singlet	Adipocyte
VAT_2M	3306.9	1522	14.366808	0	Singlet	Adipocyte
VAT_2M	2970.6	1554	0.2358533	0	Singlet	Adipocyte
VAT_2M	4810.5	2049	7.2285944	0	Singlet	Adipocyte
VAT_2M	2515	1441	0.0257429	0	Singlet	Adipocyte
VAT_2M	1756.1	1138	0	0	Singlet	Adipocyte
VAT_2M	2987.4	1591	0.2425239	0	Singlet	Adipocyte
VAT_2M	1597.2	1048	2.5155213	5	Singlet	Immune_cells
VAT_2M	2233.5	1319	0.0575365	0	Singlet	Adipocyte
VAT_2M	1530.8	926	0.6778505	0	Singlet	Adipocyte
VAT_2M	4634.6	2109	0.1604259	0	Singlet	Adipocyte
VAT_2M	2000.5	1227	0	0	Singlet	Adipocyte
VAT_2M	6201.5	2578	2.1979066	0	Singlet	Adipocyte
VAT_2M	2070.6	1100	0.0173949	0	Singlet	Adipocyte
VAT_2M	1097.3	869	6.7943788	6	Singlet	Neurons
VAT_2M	3618.9	1661	0	0	Singlet	Adipocyte
VAT_2M	1218.6	889	1.0030077	5	Singlet	Immune_cells
VAT_2M	1565.4	1139	0	0	Singlet	Adipocyte
VAT_2M	1752	921	11.188218	0	Singlet	Adipocyte
VAT_2M	2034.8	1176	2.8261593	0	Singlet	Adipocyte
VAT_2M	3553	1696	11.944187	0	Singlet	Adipocyte
VAT_2M	1640.4	1017	0.1198492	0	Singlet	Adipocyte
VAT_2M	2527.4	1319	0.0096105	0	Singlet	Adipocyte
VAT_2M	2742.8	1333	6.6535484	0	Singlet	Adipocyte
VAT_2M	2390.6	1399	0.0579825	3	Singlet	FAPs
VAT_2M	1410.9	849	11.435419	5	Singlet	Immune_cells
VAT_2M	5157	2251	0.0047723	0	Singlet	Adipocyte
VAT_2M	1038.4	672	0.0697064	0	Singlet	Adipocyte
VAT_2M	1401.9	945	4.411419	0	Singlet	Adipocyte
VAT_2M	3928	1841	11.110402	0	Singlet	Adipocyte
VAT_2M	2246.7	1251	0	0	Singlet	Adipocyte
VAT_2M	1453.9	1003	0.1424494	2	Singlet	FAPs
VAT_2M	2330.4	1234	1.474263	0	Singlet	Adipocyte
VAT_2M	2157.1	1135	7.9758307	0	Singlet	Adipocyte
VAT_2M	1572.6	1160	1.2552386	6	Singlet	Neurons
VAT_2M	1218.5	775	5.4965923	5	Singlet	Immune_cells

VAT_2M	5816.8	2281	12.391964	0	Singlet	Adipocyte
VAT_2M	3014.8	1583	0.08301	0	Singlet	Adipocyte
VAT_2M	3106.2	1585	0.0160296	0	Singlet	Adipocyte
VAT_2M	1395.6	860	2.8296183	7	Singlet	Endothelial_cells
VAT_2M	2577.1	1288	1.3036993	0	Singlet	Adipocyte
VAT_2M	3980.8	1933	8.4254265	0	Singlet	Adipocyte
VAT_2M	1133.8	726	3.5808743	2	Singlet	FAPs
VAT_2M	4805.1	2110	4.7682522	0	Singlet	Adipocyte
VAT_2M	1915.7	1198	8.1413101	2	Singlet	FAPs
VAT_2M	1169.6	780	9.3465435	3	Singlet	FAPs
VAT_2M	1284.8	935	7.3552125	6	Singlet	Neurons
VAT_2M	1055.4	843	5.0712792	0	Singlet	Adipocyte
VAT_2M	2921.1	1680	0.020032	0	Singlet	Adipocyte
VAT_2M	2116.5	1209	3.1614016	0	Singlet	Adipocyte
VAT_2M	3133.8	1526	3.1616152	0	Singlet	Adipocyte
VAT_2M	1353.9	982	2.2105809	0	Singlet	Adipocyte
VAT_2M	1875.3	1046	7.7551034	0	Singlet	Adipocyte
VAT_2M	1436.7	955	0	0	Singlet	Adipocyte
VAT_2M	5172.3	2263	0	0	Singlet	Adipocyte
VAT_2M	1118.6	697	0.620359	0	Singlet	Adipocyte
VAT_2M	3454.7	1673	0	0	Singlet	Adipocyte
VAT_2M	2736.8	1445	2.1909704	0	Singlet	Adipocyte
VAT_2M	1208.1	817	5.8507858	2	Singlet	FAPs
VAT_2M	2202.6	1469	6.2053882	5	Singlet	Immune_cells
VAT_2M	2822.2	1456	12.281157	0	Singlet	Adipocyte
VAT_2M	1200	829	4.179048	5	Singlet	Immune_cells
VAT_2M	4908.8	2248	0	0	Singlet	Adipocyte
VAT_2M	7224.2	2610	9.9428931	0	Singlet	Adipocyte
VAT_2M	1973.5	1112	0.0439365	0	Singlet	Adipocyte
VAT_2M	1090.1	865	0.0863339	3	Singlet	FAPs
VAT_2M	1017.6	752	2.4803434	0	Singlet	Adipocyte
VAT_2M	1113.3	811	0	0	Singlet	Adipocyte
VAT_2M	1157.8	1028	2.8144359	6	Singlet	Neurons
VAT_2M	2260.3	1181	0.074591	0	Singlet	Adipocyte
VAT_2M	1036.3	877	0.6314849	6	Singlet	Neurons
VAT_2M	1268	1105	1.3811963	9	Singlet	Mesothelial
VAT_2M	2455.2	1521	1.149114	2	Singlet	FAPs
VAT_2M	3845.1	1829	0.7300221	0	Singlet	Adipocyte
VAT_2M	1205.7	785	5.4031925	0	Singlet	Adipocyte
VAT_2M	3968.6	1768	0.9369898	0	Singlet	Adipocyte
VAT_2M	1122.6	632	12.375911	2	Singlet	FAPs
VAT_2M	2003	1164	1.8643241	0	Singlet	Adipocyte
VAT_2M	2125.8	1282	0.3881611	5	Singlet	Immune_cells
VAT_2M	2697.3	1448	0	0	Singlet	Adipocyte
VAT_2M	1337.6	820	0.0601174	0	Singlet	Adipocyte
VAT_2M	1137.2	779	0.1169654	0	Singlet	Adipocyte
VAT_2M	1667.5	981	1.0913895	0	Singlet	Adipocyte
VAT_2M	3157.7	1637	6.1773015	0	Singlet	Adipocyte
VAT_2M	2741.6	1566	1.8884357	0	Singlet	Adipocyte
VAT_2M	2926.3	1535	0	0	Singlet	Adipocyte
VAT_2M	1942	1312	2.5344367	2	Singlet	FAPs

VAT_2M	1382.7	829	0.0051499	0	Singlet	Adipocyte
VAT_2M	4689.1	2093	3.2945405	0	Singlet	Adipocyte
VAT_2M	2543	1381	0.1867679	0	Singlet	Adipocyte
VAT_2M	2053.6	1130	3.3426578	0	Singlet	Adipocyte
VAT_2M	4973	2172	4.5517785	0	Singlet	Adipocyte
VAT_2M	3103.3	1401	9.1462432	0	Singlet	Adipocyte
VAT_2M	1466.2	886	6.3949538	0	Singlet	Adipocyte
VAT_2M	4404.1	1966	0.3393275	0	Singlet	Adipocyte
VAT_2M	2268.8	1363	0	0	Singlet	Adipocyte
VAT_2M	1117.2	763	5.6485754	2	Singlet	FAPs
VAT_2M	2564.6	1417	10.662069	0	Singlet	Adipocyte
VAT_2M	4634.1	2250	0.4015523	0	Singlet	Adipocyte
VAT_2M	1437.1	925	4.4713838	0	Singlet	Adipocyte
VAT_2M	3442.4	1693	0.014643	0	Singlet	Adipocyte
VAT_2M	1002.9	704	14.947667	3	Singlet	FAPs
VAT_2M	1018.3	598	14.190185	2	Singlet	FAPs
VAT_2M	1330.9	749	9.5550323	0	Singlet	Adipocyte
VAT_2M	3296.2	1624	4.9530114	0	Singlet	Adipocyte
VAT_2M	1803.8	1075	3.9344691	0	Singlet	Adipocyte
VAT_2M	2049.6	1204	0.3192856	3	Singlet	FAPs
VAT_2M	4555.1	2050	4.1735241	0	Singlet	Adipocyte
VAT_2M	3558	1756	9.3800636	0	Singlet	Adipocyte
VAT_2M	3482.3	1853	0.0599102	0	Singlet	Adipocyte
VAT_2M	1871.5	1019	4.829003	0	Singlet	Adipocyte
VAT_2M	1949.2	1207	0.4524431	0	Singlet	Adipocyte
VAT_2M	6641.1	2967	11.990055	0	Singlet	Adipocyte
VAT_2M	1276.5	792	4.9407042	0	Singlet	Adipocyte
VAT_2M	1144.8	756	2.5147031	0	Singlet	Adipocyte
VAT_2M	3084	1565	2.1857107	0	Singlet	Adipocyte
VAT_2M	1123.6	623	10.477762	0	Singlet	Adipocyte
VAT_2M	2078.2	1113	0.4735674	0	Singlet	Adipocyte
VAT_2M	4724.9	1908	11.733351	0	Singlet	Adipocyte
VAT_2M	2621.1	1339	3.4514081	0	Singlet	Adipocyte
VAT_2M	1860.3	1127	0.2848279	0	Singlet	Adipocyte
VAT_2M	2010.8	1152	0	0	Singlet	Adipocyte
VAT_2M	2873.4	1413	7.4414336	0	Singlet	Adipocyte
VAT_2M	1072.2	746	12.361264	6	Singlet	Neurons
VAT_2M	2391.3	1295	6.7073724	0	Singlet	Adipocyte
VAT_2M	1042.8	641	1.7728303	0	Singlet	Adipocyte
VAT_2M	2047	1105	0.2909613	0	Singlet	Adipocyte
VAT_2M	1394.5	873	10.438854	0	Singlet	Adipocyte
VAT_2M	3299.8	1481	11.198929	0	Singlet	Adipocyte
VAT_2M	3258.7	1664	6.8279277	2	Singlet	FAPs
VAT_2M	1564.5	999	5.8123857	7	Singlet	Endothelial_cells
VAT_2M	2194.7	1387	2.3729834	3	Singlet	FAPs
VAT_2M	3224.8	1721	0.2716995	0	Singlet	Adipocyte
VAT_2M	1213.3	894	5.5751533	11	Singlet	Smooth_muscle_cells
VAT_2M	2160.8	1225	0.3645457	0	Singlet	Adipocyte
VAT_2M	5786.9	2501	3.1210966	0	Singlet	Adipocyte
VAT_2M	2155.1	1266	0.0133825	0	Singlet	Adipocyte
VAT_2M	6719.4	2773	0.071265	0	Singlet	Adipocyte

VAT_2M	5228.9	2283	0.0735609	0	Singlet	Adipocyte
VAT_2M	4040.2	2069	3.9539797	5	Singlet	Immune_cells
VAT_2M	1305.4	1027	1.4581049	6	Singlet	Neurons
VAT_2M	3320.9	1491	11.20289	2	Singlet	FAPs
VAT_2M	1774.4	1131	0.0780184	0	Singlet	Adipocyte
VAT_2M	2516.9	1519	2.5154361	3	Singlet	FAPs
VAT_2M	2191.8	1120	13.188696	0	Singlet	Adipocyte
VAT_2M	1064.3	646	4.8414953	0	Singlet	Adipocyte
VAT_2M	4692.7	2148	3.8179992	0	Singlet	Adipocyte
VAT_2M	4868.4	2192	12.043	0	Singlet	Adipocyte
VAT_2M	1287.9	939	0.1149531	5	Singlet	Immune_cells
VAT_2M	4800.5	2371	4.133055	0	Singlet	Adipocyte
VAT_2M	2403.7	1403	2.4574113	0	Singlet	Adipocyte
VAT_2M	1996.4	1070	8.2381481	0	Singlet	Adipocyte
VAT_2M	2674.9	1386	7.9201706	0	Singlet	Adipocyte
VAT_2M	3425.1	1674	0.4881089	0	Singlet	Adipocyte
VAT_2M	2805.8	1666	7.245872	9	Singlet	Mesothelial
VAT_2M	1755.9	1390	0.0326339	11	Singlet	Smooth_muscle_cells
VAT_2M	1351.2	802	7.5765289	0	Singlet	Adipocyte
VAT_2M	1099	835	3.7796279	6	Singlet	Neurons
VAT_2M	1908.7	1151	1.5680964	0	Singlet	Adipocyte
VAT_2M	2605.2	1368	0.8959961	0	Singlet	Adipocyte
VAT_2M	1695.9	1030	9.3464439	5	Singlet	Immune_cells
VAT_2M	2245	1281	1.4888317	3	Singlet	FAPs
VAT_2M	2004.6	1107	3.4631225	0	Singlet	Adipocyte
VAT_2M	1775.3	957	9.7262348	0	Singlet	Adipocyte
VAT_2M	2127.3	1222	0	0	Singlet	Adipocyte
VAT_2M	2772.4	1520	6.8204302	0	Singlet	Adipocyte
VAT_2M	2683.7	1414	0.0336539	0	Singlet	Adipocyte
VAT_2M	8412.4	2952	5.7203257	0	Singlet	Adipocyte
VAT_2M	2888.5	1474	7.4420544	0	Singlet	Adipocyte
VAT_2M	3664.2	1890	2.0468967	0	Singlet	Adipocyte
VAT_2M	3557.2	1765	7.1588874	0	Singlet	Adipocyte
VAT_2M	1769.3	1035	13.802075	5	Singlet	Immune_cells
VAT_2M	1965.4	1229	0.4711022	0	Singlet	Adipocyte
VAT_2M	1003.2	752	0.0235866	3	Singlet	FAPs
VAT_2M	2374.9	1145	3.9629076	0	Singlet	Adipocyte
VAT_2M	1392	935	0	0	Singlet	Adipocyte
VAT_2M	1067.4	827	0.6839814	6	Singlet	Neurons
VAT_2M	2408.9	1242	3.3987265	0	Singlet	Adipocyte
VAT_2M	1083.3	687	4.8428867	3	Singlet	FAPs
VAT_2M	3614.4	1803	2.3406449	0	Singlet	Adipocyte
VAT_2M	3074	1535	2.9169799	0	Singlet	Adipocyte
VAT_2M	2915.2	1517	1.8375014	3	Singlet	FAPs
VAT_2M	1574.3	1041	5.2903071	2	Singlet	FAPs
VAT_2M	1514.1	787	10.041255	0	Singlet	Adipocyte
VAT_2M	2806.2	1611	0.635618	0	Singlet	Adipocyte
VAT_2M	1571.8	907	1.6617457	0	Singlet	Adipocyte
VAT_2M	2480.9	1244	9.9185988	2	Singlet	FAPs
VAT_2M	1787.3	1073	0.2984793	2	Singlet	FAPs
VAT_2M	2741.5	1346	14.434557	0	Singlet	Adipocyte

VAT_2M	3772.4	1739	10.679626	0	Singlet	Adipocyte
VAT_2M	2263.3	1312	4.7276557	0	Singlet	Adipocyte
VAT_2M	1974.1	1155	0.1123072	0	Singlet	Adipocyte
VAT_2M	1311.9	788	0.1908811	0	Singlet	Adipocyte
VAT_2M	2759.4	1588	5.5439689	2	Singlet	FAPs
VAT_2M	2279.6	1286	2.9696844	0	Singlet	Adipocyte
VAT_2M	1483.4	1076	4.3937768	0	Singlet	Adipocyte
VAT_2M	1442.5	905	13.725254	6	Singlet	Neurons
VAT_2M	3711.5	1852	1.4538649	0	Singlet	Adipocyte
VAT_2M	2444.7	1303	0	0	Singlet	Adipocyte
VAT_2M	3848.8	1902	0	0	Singlet	Adipocyte
VAT_2M	5322.7	2413	0.0893467	0	Singlet	Adipocyte
VAT_2M	1960.1	1208	0.2673667	0	Singlet	Adipocyte
VAT_2M	1108.1	737	2.9606265	0	Singlet	Adipocyte
VAT_2M	2643.4	1502	0	3	Singlet	FAPs
VAT_2M	1357.7	993	1.4386691	6	Singlet	Neurons
VAT_2M	1790.5	1013	0.0049726	0	Singlet	Adipocyte
VAT_2M	5816.5	2440	8.1803078	0	Singlet	Adipocyte
VAT_2M	1433.2	1025	10.829136	11	Singlet	Smooth_muscle_cells
VAT_2M	2790.4	1594	0.4480018	0	Singlet	Adipocyte
VAT_2M	5114.1	2325	0.9565359	0	Singlet	Adipocyte
VAT_2M	6486.9	2546	1.1061551	0	Singlet	Adipocyte
VAT_2M	1604.9	1039	0	3	Singlet	FAPs
VAT_2M	3506.3	1539	11.140259	0	Singlet	Adipocyte
VAT_2M	1123.6	780	3.1222828	3	Singlet	FAPs
VAT_2M	1519.7	1016	0	0	Singlet	Adipocyte
VAT_2M	7817.2	3006	0.1420442	0	Singlet	Adipocyte
VAT_2M	2136.3	1239	0.1372825	0	Singlet	Adipocyte
VAT_2M	1496.8	880	3.8993772	0	Singlet	Adipocyte
VAT_2M	1803.1	944	14.512156	5	Singlet	Immune_cells
VAT_2M	1680	1019	4.2679789	5	Singlet	Immune_cells
VAT_2M	1690.4	1082	0.0455846	0	Singlet	Adipocyte
VAT_2M	1461.2	1039	6.2828348	2	Singlet	FAPs
VAT_2M	1895.3	1056	0.1344106	0	Singlet	Adipocyte
VAT_2M	2143.6	1123	0.0965837	0	Singlet	Adipocyte
VAT_2M	2951.3	1308	8.7080189	0	Singlet	Adipocyte
VAT_2M	3378.7	1685	9.3373747	0	Singlet	Adipocyte
VAT_2M	4628.5	2174	4.9598243	0	Singlet	Adipocyte
VAT_2M	2800.1	1434	0.0922025	0	Singlet	Adipocyte
VAT_2M	2404.3	1287	10.166354	0	Singlet	Adipocyte
VAT_2M	1462.2	862	0.0119995	0	Singlet	Adipocyte
VAT_2M	2727	1342	6.2900349	0	Singlet	Adipocyte
VAT_2M	1865.1	1097	4.6169562	0	Singlet	Adipocyte
VAT_2M	2264.5	1181	4.2510814	0	Singlet	Adipocyte
VAT_2M	2266.1	1483	0.5425571	3	Singlet	FAPs
VAT_2M	1006.6	811	5.3484985	5	Singlet	Immune_cells
VAT_2M	1106.3	725	0.0775842	0	Singlet	Adipocyte
VAT_2M	1911.4	617	12.82529	3	Singlet	FAPs
VAT_2M	1672.8	1160	7.2825591	2	Singlet	FAPs
VAT_2M	2153.2	1194	0.4496434	0	Singlet	Adipocyte
VAT_2M	2302	1412	0.1417299	3	Singlet	FAPs

VAT_2M	1329.6	870	3.8532167	3	Singlet	FAPs
VAT_2M	3077.9	1615	0	0	Singlet	Adipocyte
VAT_2M	1443.9	1129	0	0	Singlet	Adipocyte
VAT_2M	5018	2250	2.8666831	2	Singlet	FAPs
VAT_2M	1047.3	808	0.1432162	6	Singlet	Neurons
VAT_2M	3728.3	1836	10.185294	0	Singlet	Adipocyte
VAT_2M	3602.9	1652	12.174053	0	Singlet	Adipocyte
VAT_2M	1694.8	1088	1.2184607	5	Singlet	Immune_cells
VAT_2M	4745.2	2103	0	0	Singlet	Adipocyte
VAT_2M	5128.2	2417	0	0	Singlet	Adipocyte
VAT_2M	1684.5	1033	3.2902681	3	Singlet	FAPs
VAT_2M	2593.7	1378	1.795519	0	Singlet	Adipocyte
VAT_2M	2632	1460	0	0	Singlet	Adipocyte
VAT_2M	1795.5	1612	1.214663	6	Singlet	Neurons
VAT_2M	1678	995	0.207662	0	Singlet	Adipocyte
VAT_2M	4015.7	1965	0.0261115	0	Singlet	Adipocyte
VAT_2M	2987.1	1592	0.8912868	0	Singlet	Adipocyte
VAT_2M	1182.7	674	4.0999455	0	Singlet	Adipocyte
VAT_2M	1002.7	752	1.025645	0	Singlet	Adipocyte
VAT_2M	2779.1	1433	3.5233221	0	Singlet	Adipocyte
VAT_2M	2307.2	1183	9.525792	0	Singlet	Adipocyte
VAT_2M	4588	2098	3.8895945	0	Singlet	Adipocyte
VAT_2M	1685	915	4.2000585	0	Singlet	Adipocyte
VAT_2M	1312	822	0.1564716	0	Singlet	Adipocyte
VAT_2M	2383.8	1249	3.5994633	0	Singlet	Adipocyte
VAT_2M	5375.4	2226	5.200885	0	Singlet	Adipocyte
VAT_2M	2304.1	1308	3.3163918	2	Singlet	FAPs
VAT_2M	1626.8	1011	14.440526	6	Singlet	Neurons
VAT_2M	5116.9	2256	7.2318918	0	Singlet	Adipocyte
VAT_2M	1081.1	741	0	0	Singlet	Adipocyte
VAT_2M	1578.9	837	13.086664	0	Singlet	Adipocyte
VAT_2M	2190	1191	0.0160392	0	Singlet	Adipocyte
VAT_2M	1912.3	1054	4.9084696	0	Singlet	Adipocyte
VAT_2M	2722.6	1398	0	0	Singlet	Adipocyte
VAT_2M	4356.5	2100	0	0	Singlet	Adipocyte
VAT_2M	2633	1399	4.2002626	0	Singlet	Adipocyte
VAT_2M	2406.4	1318	2.2371699	0	Singlet	Adipocyte
VAT_2M	3192.7	1781	11.4338	0	Singlet	Adipocyte
VAT_2M	1038.8	828	9.4103747	8	Singlet	Mesothelial
VAT_2M	3292.3	1735	0	0	Singlet	Adipocyte
VAT_2M	2763.4	1524	2.0605512	0	Singlet	Adipocyte
VAT_2M	4229.9	1919	0.34672	0	Singlet	Adipocyte
VAT_2M	3436.8	1679	4.1720172	0	Singlet	Adipocyte
VAT_2M	1326.3	1154	2.1947883	8	Singlet	Mesothelial
VAT_2M	9670.9	3373	7.267793	0	Singlet	Adipocyte
VAT_2M	1984.2	1141	0	0	Singlet	Adipocyte
VAT_2M	3737.8	1753	0.2407187	0	Singlet	Adipocyte
VAT_2M	1979.3	1105	5.3227189	0	Singlet	Adipocyte
VAT_2M	2211.4	1214	14.578625	3	Singlet	FAPs
VAT_2M	5407.2	2351	0.4400803	0	Singlet	Adipocyte
VAT_2M	1276.3	711	5.1807827	0	Singlet	Adipocyte

VAT_2M	2029.4	923	8.5089804	0	Singlet	Adipocyte
VAT_2M	7584.1	2968	7.3261304	0	Singlet	Adipocyte
VAT_2M	1225.6	917	0.5645235	0	Singlet	Adipocyte
VAT_2M	1422.8	1051	0	0	Singlet	Adipocyte
VAT_2M	2037.6	1116	13.72349	5	Singlet	Immune_cells
VAT_2M	1605.9	924	0.2217477	0	Singlet	Adipocyte
VAT_2M	1600.9	1021	0.7025217	0	Singlet	Adipocyte
VAT_2M	5685.3	2347	0.2339038	0	Singlet	Adipocyte
VAT_2M	4932	2147	0.3002746	0	Singlet	Adipocyte
VAT_2M	1064.2	763	0	0	Singlet	Adipocyte
VAT_2M	4308.4	2132	0.1120773	0	Singlet	Adipocyte
VAT_2M	3069.1	1670	10.202056	5	Singlet	Immune_cells
VAT_2M	2769	1388	8.4661399	0	Singlet	Adipocyte
VAT_2M	1120.3	704	3.7686489	3	Singlet	FAPs
VAT_2M	4300.9	1928	0.4948959	0	Singlet	Adipocyte
VAT_2M	1012.5	679	2.3566017	7	Singlet	Endothelial_cells
VAT_2M	1400.5	819	8.7209653	7	Singlet	Endothelial_cells
VAT_2M	3412	1697	0.1063118	0	Singlet	Adipocyte
VAT_2M	1649.6	1035	0.0271721	0	Singlet	Adipocyte
VAT_2M	2107.3	1187	0.3060957	0	Singlet	Adipocyte
VAT_2M	3264.6	1678	0.4721842	0	Singlet	Adipocyte
VAT_2M	2050.5	1293	0.2736683	0	Singlet	Adipocyte
VAT_2M	4474.8	2015	5.8076263	0	Singlet	Adipocyte
VAT_2M	3398.4	1656	3.0507151	0	Singlet	Adipocyte
VAT_2M	1687	974	2.8640037	0	Singlet	Adipocyte
VAT_2M	4321.8	2061	0.3488032	0	Singlet	Adipocyte
VAT_2M	5367.1	2410	0	0	Singlet	Adipocyte
VAT_2M	2868.6	1419	0.0261975	0	Singlet	Adipocyte
VAT_2M	3379	1753	11.909266	2	Singlet	FAPs
VAT_2M	1965.3	1166	1.7058267	2	Singlet	FAPs
VAT_2M	1678.7	1021	4.8906554	0	Singlet	Adipocyte
VAT_2M	4535.3	2019	11.355405	0	Singlet	Adipocyte
VAT_2M	1541.8	896	6.9847516	5	Singlet	Immune_cells
VAT_2M	2532.4	1491	1.5822943	5	Singlet	Immune_cells
VAT_2M	1363.9	868	5.5914116	5	Singlet	Immune_cells
VAT_2M	3810.5	1850	3.2787959	0	Singlet	Adipocyte
VAT_2M	1315.4	787	9.0160812	2	Singlet	FAPs
VAT_2M	3063.5	1573	0.0422075	0	Singlet	Adipocyte
VAT_2M	1478.9	988	13.575291	6	Singlet	Neurons
VAT_2M	3916.9	1874	1.2496171	0	Singlet	Adipocyte
VAT_2M	2001.9	1058	8.4227815	0	Singlet	Adipocyte
VAT_2M	1330.8	983	5.6472607	2	Singlet	FAPs
VAT_2M	1868.4	1144	0.6368674	0	Singlet	Adipocyte
VAT_2M	1048.3	769	0.2521873	3	Singlet	FAPs
VAT_2M	1417.9	1002	10.676149	0	Singlet	Adipocyte
VAT_2M	4759.2	2199	10.536096	0	Singlet	Adipocyte
VAT_2M	6217	2474	6.749284	0	Singlet	Adipocyte
VAT_2M	1738.3	1136	6.2545283	2	Singlet	FAPs
VAT_2M	1528.9	976	4.6242797	3	Singlet	FAPs
VAT_2M	2323.3	1329	0.1137591	0	Singlet	Adipocyte
VAT_2M	2925.5	1489	0.3659507	0	Singlet	Adipocyte

VAT_2M	7072	2782	0	0	Singlet	Adipocyte
VAT_2M	5111.4	2254	0.1381139	0	Singlet	Adipocyte
VAT_2M	1485.9	1041	11.39306	6	Singlet	Neurons
VAT_2M	1012.3	637	6.6622796	0	Singlet	Adipocyte
VAT_2M	1805.9	1044	1.1350933	0	Singlet	Adipocyte
VAT_2M	2359.1	1346	3.4669188	0	Singlet	Adipocyte
VAT_2M	1717	1032	0.2906897	0	Singlet	Adipocyte
VAT_2M	1501.8	988	9.2500356	2	Singlet	FAPs
VAT_2M	1469.9	880	0.1633321	0	Singlet	Adipocyte
VAT_2M	6741.8	2467	9.9271744	0	Singlet	Adipocyte
VAT_2M	1641	929	10.957836	0	Singlet	Adipocyte
VAT_2M	2166	1403	6.8855476	0	Singlet	Adipocyte
VAT_2M	2573.2	1436	0.0412574	0	Singlet	Adipocyte
VAT_2M	1106.6	644	6.2390454	3	Singlet	FAPs
VAT_2M	3711.1	1702	6.0320209	0	Singlet	Adipocyte
VAT_2M	1356.1	1208	3.1828304	6	Singlet	Neurons
VAT_2M	2187.3	1255	8.2744505	5	Singlet	Immune_cells
VAT_2M	3820.8	1932	1.2164086	0	Singlet	Adipocyte
VAT_2M	2081.3	1230	0.9611119	0	Singlet	Adipocyte
VAT_2M	3565.6	1697	0.2606061	0	Singlet	Adipocyte
VAT_2M	2526.2	1364	8.0539617	0	Singlet	Adipocyte
VAT_2M	1023.4	856	6.8459754	6	Singlet	Neurons
VAT_2M	1960.1	1128	9.1227415	3	Singlet	FAPs
VAT_2M	1144.9	865	0.231006	0	Singlet	Adipocyte
VAT_2M	2143.6	1207	3.8377345	0	Singlet	Adipocyte
VAT_2M	1232.9	823	6.0248442	2	Singlet	FAPs
VAT_2M	1113.4	796	4.3680319	0	Singlet	Adipocyte
VAT_2M	4584.5	1902	11.721709	0	Singlet	Adipocyte
VAT_2M	1883.1	1265	6.1399876	2	Singlet	FAPs
VAT_2M	2712.9	1557	0.2275799	0	Singlet	Adipocyte
VAT_2M	1663.3	976	5.3300866	0	Singlet	Adipocyte
VAT_2M	1005.1	849	1.038249	7	Singlet	Endothelial_cells
VAT_2M	1294.5	801	1.6618914	0	Singlet	Adipocyte
VAT_2M	2154.7	1208	0	0	Singlet	Adipocyte
VAT_2M	1272.7	1015	0.5385167	6	Singlet	Neurons
VAT_2M	1407.8	802	0.9856555	0	Singlet	Adipocyte
VAT_2M	1040.8	760	13.627252	6	Singlet	Neurons
VAT_2M	2268.9	1342	0.759288	0	Singlet	Adipocyte
VAT_2M	1429.6	860	1.7033938	0	Singlet	Adipocyte
VAT_2M	1187.7	969	5.4802813	11	Singlet	Smooth_muscle_cells
VAT_2M	1225	742	1.9455986	0	Singlet	Adipocyte
VAT_2M	5597.3	2240	9.5896283	0	Singlet	Adipocyte
VAT_2M	2690.2	1437	0	0	Singlet	Adipocyte
VAT_2M	1910.7	1065	0	0	Singlet	Adipocyte
VAT_2M	1389.1	977	2.0276151	2	Singlet	FAPs
VAT_2M	1858.3	1096	0.4777148	0	Singlet	Adipocyte
VAT_2M	1581.5	994	0.1603656	3	Singlet	FAPs
VAT_2M	1709.5	1159	1.5605179	3	Singlet	FAPs
VAT_2M	3388.6	1526	10.490442	0	Singlet	Adipocyte
VAT_2M	1761.8	1323	4.2816529	11	Singlet	Smooth_muscle_cells
VAT_2M	3888.2	1957	1.1832445	0	Singlet	Adipocyte

VAT_2M	6340.2	2708	0.0320494	0	Singlet	Adipocyte
VAT_2M	3970.7	1881	0.0951657	0	Singlet	Adipocyte
VAT_2M	1323.4	867	5.9120717	2	Singlet	FAPs
VAT_2M	1198	726	5.0440282	0	Singlet	Adipocyte
VAT_2M	1289.9	796	10.400471	6	Singlet	Neurons
VAT_2M	1380.3	864	13.577816	7	Singlet	Endothelial_cells
VAT_2M	2227.4	1214	6.8093629	0	Singlet	Adipocyte
VAT_2M	2377.9	1321	13.468123	2	Singlet	FAPs
VAT_2M	3003.1	1638	4.9202092	3	Singlet	FAPs
VAT_2M	9256	3263	1.9360409	0	Singlet	Adipocyte
VAT_2M	1338.5	955	0.1770266	3	Singlet	FAPs
VAT_2M	5395.9	2307	2.5208762	0	Singlet	Adipocyte
VAT_2M	1918.1	1114	9.0078121	0	Singlet	Adipocyte
VAT_2M	1116.4	714	10.805444	0	Singlet	Adipocyte
VAT_2M	2322.5	1348	10.382374	0	Singlet	Adipocyte
VAT_2M	3733.1	1734	3.8275711	0	Singlet	Adipocyte
VAT_2M	4136.5	1918	2.0419498	0	Singlet	Adipocyte
VAT_2M	1556.9	934	2.0838401	7	Singlet	Endothelial_cells
VAT_2M	1341.9	960	0.2055349	7	Singlet	Endothelial_cells
VAT_2M	2073.9	1124	0.2383662	0	Singlet	Adipocyte
VAT_2M	1145.9	807	3.2772817	7	Singlet	Endothelial_cells
VAT_2M	1044.1	734	3.9228683	6	Singlet	Neurons
VAT_2M	4398.5	1919	11.504906	0	Singlet	Adipocyte
VAT_2M	8519.3	3068	9.0284045	0	Singlet	Adipocyte
VAT_2M	3821.3	1594	12.013118	7	Singlet	Endothelial_cells
VAT_2M	1294.8	987	0	6	Singlet	Neurons
VAT_2M	1043.5	670	1.3577742	0	Singlet	Adipocyte
VAT_2M	1171.4	795	8.2529393	6	Singlet	Neurons
VAT_2M	1447.1	1001	13.581273	2	Singlet	FAPs
VAT_2M	7328.7	2685	8.0018833	0	Singlet	Adipocyte
VAT_2M	2634.1	1343	1.0449118	0	Singlet	Adipocyte
VAT_2M	2595.7	1530	0.0242165	0	Singlet	Adipocyte
VAT_2M	1101.2	784	8.6329206	6	Singlet	Neurons
VAT_2M	4500.6	2141	1.3178239	0	Singlet	Adipocyte
VAT_2M	7084	2819	0	0	Singlet	Adipocyte
VAT_2M	1608.3	1254	11.962662	6	Singlet	Neurons
VAT_2M	1309.3	860	3.0850285	0	Singlet	Adipocyte
VAT_2M	1106.1	768	0	0	Singlet	Adipocyte
VAT_2M	1746.6	1004	8.3840117	0	Singlet	Adipocyte
VAT_2M	1924.6	1469	5.1576552	0	Singlet	Adipocyte
VAT_2M	3141.6	1565	0.8469313	0	Singlet	Adipocyte
VAT_2M	1471.1	914	6.8652386	5	Singlet	Immune_cells
VAT_2M	1345.5	676	10.719397	3	Singlet	FAPs
VAT_2M	3033.8	1481	13.88759	0	Singlet	Adipocyte
VAT_2M	1009.6	718	5.0802116	4	Singlet	Immune_cells
VAT_2M	2034.4	1168	0.0053995	0	Singlet	Adipocyte
VAT_2M	1433.2	1258	0.0530527	6	Singlet	Neurons
VAT_2M	6155	2507	1.2966841	0	Singlet	Adipocyte
VAT_2M	2999.1	1530	0.242399	0	Singlet	Adipocyte
VAT_2M	1900.7	1073	6.0829129	0	Singlet	Adipocyte
VAT_2M	2398.4	1368	0.0120153	0	Singlet	Adipocyte

VAT_2M	1692.5	1018	8.0608801	3	Singlet	FAPs
VAT_2M	7288.3	2669	10.814626	0	Singlet	Adipocyte
VAT_2M	3286.1	1542	1.1889267	0	Singlet	Adipocyte
VAT_2M	1166.1	728	11.967204	0	Singlet	Adipocyte
VAT_2M	1648.9	1190	10.908548	6	Singlet	Neurons
VAT_2M	1513.2	858	0.1182405	0	Singlet	Adipocyte
VAT_2M	1024.6	614	10.611504	7	Singlet	Endothelial_cells
VAT_2M	5796.7	2403	2.3158331	0	Singlet	Adipocyte
VAT_2M	1806.6	1065	7.8901193	2	Singlet	FAPs
VAT_2M	3186	1507	12.231095	0	Singlet	Adipocyte
VAT_2M	1341	892	2.8004539	2	Singlet	FAPs
VAT_2M	1529	882	8.1755796	0	Singlet	Adipocyte
VAT_2M	2917.7	1476	12.118756	0	Singlet	Adipocyte
VAT_2M	8570.1	3074	3.4117643	0	Singlet	Adipocyte
VAT_2M	1056	776	0	0	Singlet	Adipocyte
VAT_2M	1453.3	953	7.8498059	3	Singlet	FAPs
VAT_2M	4542.3	2219	12.422219	2	Singlet	FAPs
VAT_2M	2056.5	1026	12.559185	0	Singlet	Adipocyte
VAT_2M	3370.2	1831	3.6838172	0	Singlet	Adipocyte
VAT_2M	2901.8	1545	0.3883512	0	Singlet	Adipocyte
VAT_2M	2046.9	1208	0.5384549	0	Singlet	Adipocyte
VAT_2M	1817.5	1072	3.2373558	0	Singlet	Adipocyte
VAT_2M	1406.1	913	0.7660741	0	Singlet	Adipocyte
VAT_2M	5121.5	2427	1.304349	0	Singlet	Adipocyte
VAT_2M	4347.7	1971	10.53967	0	Singlet	Adipocyte
VAT_2M	1710.7	983	1.8633507	0	Singlet	Adipocyte
VAT_2M	1920.8	1147	1.1901172	0	Singlet	Adipocyte
VAT_2M	2205.5	1092	8.8413505	0	Singlet	Adipocyte
VAT_2M	1319	771	1.5267177	0	Singlet	Adipocyte
VAT_2M	1007.3	710	0.029787	0	Singlet	Adipocyte
VAT_2M	2383.9	1344	0.9532441	0	Singlet	Adipocyte
VAT_2M	2074.2	1152	0.7027129	0	Singlet	Adipocyte
VAT_2M	1104.4	763	0.0409153	0	Singlet	Adipocyte
VAT_2M	4616.1	2295	0.0121896	0	Singlet	Adipocyte
VAT_2M	1169.4	702	3.8064137	0	Singlet	Adipocyte
VAT_2M	2163	1260	0.9861799	3	Singlet	FAPs
VAT_2M	2904.1	1604	0.2833039	0	Singlet	Adipocyte
VAT_2M	1797.3	973	0.0028535	0	Singlet	Adipocyte
VAT_2M	2248.2	1244	3.588939	5	Singlet	Immune_cells
VAT_2M	2560.3	1487	0.0401239	7	Singlet	Endothelial_cells
VAT_2M	1383.2	774	0.7268119	0	Singlet	Adipocyte
VAT_2M	4413.1	2092	2.043895	0	Singlet	Adipocyte
VAT_2M	1076.4	774	10.895025	11	Singlet	Smooth_muscle_cells
VAT_2M	1065	701	2.5679566	0	Singlet	Adipocyte
VAT_2M	1200.7	821	0.0759531	6	Singlet	Neurons
VAT_2M	1677.8	995	2.0957265	0	Singlet	Adipocyte
VAT_2M	1037.8	731	5.4428252	0	Singlet	Adipocyte
VAT_2M	5621.4	2277	7.8307986	0	Singlet	Adipocyte
VAT_2M	3551.8	2088	0.1457866	3	Singlet	FAPs
VAT_2M	1753.8	1159	0.3221207	3	Singlet	FAPs
VAT_2M	1193.6	880	6.9522141	4	Singlet	Immune_cells

VAT_2M	7025	2818	4.1606097	0	Singlet	Adipocyte
VAT_2M	3700.3	1784	0.0513642	0	Singlet	Adipocyte
VAT_2M	2136.7	1329	0.3335722	0	Singlet	Adipocyte
VAT_2M	1467.8	996	0.0293967	0	Singlet	Adipocyte
VAT_2M	1989.2	1351	0.061292	5	Singlet	Immune_cells
VAT_2M	4118.6	2057	1.2547086	0	Singlet	Adipocyte
VAT_2M	1833.2	1324	1.0817729	6	Singlet	Neurons
VAT_2M	1417.9	777	11.393257	3	Singlet	FAPs
VAT_2M	1118	812	0	0	Singlet	Adipocyte
VAT_2M	1002.1	618	14.113902	2	Singlet	FAPs
VAT_2M	4674.1	2136	3.4646801	0	Singlet	Adipocyte
VAT_2M	1100.1	751	6.9650527	9	Singlet	Mesothelial
VAT_2M	1309.6	697	12.483784	9	Singlet	Mesothelial
VAT_2M	1558.8	1059	6.9831871	2	Singlet	FAPs
VAT_2M	3977.6	1872	10.842523	0	Singlet	Adipocyte
VAT_2M	1509.6	931	6.8622931	0	Singlet	Adipocyte
VAT_2M	2188.5	1303	1.2420994	0	Singlet	Adipocyte
VAT_2M	2709.9	1568	1.5364623	3	Singlet	FAPs
VAT_2M	1035.4	723	4.174063	0	Singlet	Adipocyte
VAT_2M	1295.5	929	4.6814529	2	Singlet	FAPs
VAT_2M	1438.1	1092	0	0	Singlet	Adipocyte
VAT_2M	4723.2	1894	14.555769	0	Singlet	Adipocyte
VAT_2M	1052.6	942	0.5813675	3	Singlet	FAPs
VAT_2M	1130	801	2.0151416	6	Singlet	Neurons
VAT_2M	1760.6	1080	0	0	Singlet	Adipocyte
VAT_2M	4283.2	1667	12.026884	0	Singlet	Adipocyte
VAT_2M	4273.1	2148	0.0126578	0	Singlet	Adipocyte
VAT_2M	1681.2	976	4.3190285	0	Singlet	Adipocyte
VAT_2M	1276.2	903	1.0776123	3	Singlet	FAPs
VAT_2M	2742.2	1462	8.1294855	0	Singlet	Adipocyte
VAT_2M	1399.3	1209	2.6322484	6	Singlet	Neurons
VAT_2M	5022.8	2167	12.298657	0	Singlet	Adipocyte
VAT_2M	1526.1	903	0.423978	0	Singlet	Adipocyte
VAT_2M	1860.1	1100	0.0929546	0	Singlet	Adipocyte
VAT_2M	1198.9	721	0.9680352	0	Singlet	Adipocyte
VAT_2M	1462.5	985	0.2218543	3	Singlet	FAPs
VAT_2M	1015.3	645	8.6552693	2	Singlet	FAPs
VAT_2M	1886.5	1096	0.2520348	0	Singlet	Adipocyte
VAT_2M	2036.6	1283	0.6729644	5	Singlet	Immune_cells
VAT_2M	3349.5	1628	0.3862553	0	Singlet	Adipocyte
VAT_2M	1220.7	943	0.0963774	7	Singlet	Endothelial_cells
VAT_2M	1788.2	1086	1.5378215	0	Singlet	Adipocyte
VAT_2M	1224.6	793	0.2811764	0	Singlet	Adipocyte
VAT_2M	2320.2	1429	4.667158	2	Singlet	FAPs
VAT_2M	3287.9	1995	0	3	Singlet	FAPs
VAT_2M	2523.8	1253	0.0588791	0	Singlet	Adipocyte
VAT_2M	4861.1	2247	1.193898	0	Singlet	Adipocyte
VAT_2M	1027.3	656	0.9809767	0	Singlet	Adipocyte
VAT_2M	1886.4	1099	0	0	Singlet	Adipocyte
VAT_2M	4789.7	2065	0	0	Singlet	Adipocyte
VAT_2M	1458.3	880	3.0068577	0	Singlet	Adipocyte

VAT_2M	2256.2	1314	1.7643689	0	Singlet	Adipocyte
VAT_2M	1053.3	663	4.5109722	2	Singlet	FAPs
VAT_2M	1954	1138	3.5825242	0	Singlet	Adipocyte
VAT_2M	1782.6	1245	1.7424737	0	Singlet	Adipocyte
VAT_2M	6573.6	2515	2.781141	0	Singlet	Adipocyte
VAT_2M	1211.6	952	11.133116	5	Singlet	Immune_cells
VAT_2M	6686.7	2508	0	0	Singlet	Adipocyte
VAT_2M	2701.5	1425	1.5675008	0	Singlet	Adipocyte
VAT_2M	1509.7	923	8.9936874	3	Singlet	FAPs
VAT_2M	1290.8	896	0.9972486	0	Singlet	Adipocyte
VAT_2M	1174.1	702	0.1108621	0	Singlet	Adipocyte
VAT_2M	4870.2	2275	0.9689703	0	Singlet	Adipocyte
VAT_2M	2516.6	1040	10.288191	5	Singlet	Immune_cells
VAT_2M	1912.1	1154	0.061964	0	Singlet	Adipocyte
VAT_2M	2411.3	1406	4.4563552	0	Singlet	Adipocyte
VAT_2M	1717.2	931	10.637423	0	Singlet	Adipocyte
VAT_2M	1084.7	866	3.1965372	5	Singlet	Immune_cells
VAT_2M	1504.8	1042	2.975051	0	Singlet	Adipocyte
VAT_2M	2143.3	1216	0.200447	0	Singlet	Adipocyte
VAT_2M	1363.4	903	1.4051997	3	Singlet	FAPs
VAT_2M	3538.7	1852	0.2322466	0	Singlet	Adipocyte
VAT_2M	1097.9	602	0.5440043	0	Singlet	Adipocyte
VAT_2M	1394.8	748	10.134545	5	Singlet	Immune_cells
VAT_2M	2744.2	1436	3.0463263	0	Singlet	Adipocyte
VAT_2M	1039	794	0.0982593	3	Singlet	FAPs
VAT_2M	1637.1	1045	3.5841712	0	Singlet	Adipocyte
VAT_2M	1471.1	953	6.6400478	2	Singlet	FAPs
VAT_2M	1375.7	797	11.727667	0	Singlet	Adipocyte
VAT_2M	2576.7	1457	2.295953	3	Singlet	FAPs
VAT_2M	1480.3	864	3.7616462	3	Singlet	FAPs
VAT_2M	4570.2	2158	2.673866	0	Singlet	Adipocyte
VAT_2M	1068.2	559	5.4557942	3	Singlet	FAPs
VAT_2M	1080.9	672	1.382321	0	Singlet	Adipocyte
VAT_2M	1045.6	708	13.129085	3	Singlet	FAPs
VAT_2M	3434.8	1530	10.755667	2	Singlet	FAPs
VAT_2M	2065.1	1193	13.242427	9	Singlet	Mesothelial
VAT_2M	1312.1	795	0.3041007	0	Singlet	Adipocyte
VAT_2M	1084.5	617	6.4262281	11	Singlet	Smooth_muscle_cells
VAT_2M	5924.3	2328	0.0021791	0	Singlet	Adipocyte
VAT_2M	9205.2	3321	5.0469587	0	Singlet	Adipocyte
VAT_2M	1006.4	593	9.2711508	5	Singlet	Immune_cells
VAT_2M	1074.9	742	8.4579407	3	Singlet	FAPs
VAT_2M	4126.5	2003	0	0	Singlet	Adipocyte
VAT_2M	4243.9	1891	9.3250018	0	Singlet	Adipocyte
VAT_2M	2779.9	1753	0.5911312	2	Singlet	FAPs
VAT_2M	1969.5	1050	2.6436157	7	Singlet	Endothelial_cells
VAT_2M	3243.8	1780	0	3	Singlet	FAPs
VAT_2M	4017.4	1852	10.44018	0	Singlet	Adipocyte
VAT_2M	1565	1030	0	7	Singlet	Endothelial_cells
VAT_2M	1293.7	814	1.5143507	3	Singlet	FAPs
VAT_2M	1063.5	800	5.9941991	2	Singlet	FAPs

VAT_2M	1135.5	772	0	3	Singlet	FAPs
VAT_2M	1627.6	995	3.5462216	0	Singlet	Adipocyte
VAT_2M	1352.9	1014	3.6028177	5	Singlet	Immune_cells
VAT_2M	2158.5	1188	0	0	Singlet	Adipocyte
VAT_2M	1490.5	1043	0.3355676	0	Singlet	Adipocyte
VAT_2M	1250.3	823	0.6502929	0	Singlet	Adipocyte
VAT_2M	2182.2	1165	7.1380127	0	Singlet	Adipocyte
VAT_2M	1658.3	886	7.6220654	0	Singlet	Adipocyte
VAT_2M	1818.1	1089	6.0432355	7	Singlet	Endothelial_cells
VAT_2M	8114.9	2998	6.1170033	0	Singlet	Adipocyte
VAT_2M	1639.4	1031	0.1642048	0	Singlet	Adipocyte
VAT_2M	4639	2085	6.9790403	0	Singlet	Adipocyte
VAT_2M	1050.1	683	0.3945836	0	Singlet	Adipocyte
VAT_2M	3837.2	1823	0	0	Singlet	Adipocyte
VAT_2M	1683.6	938	1.8605883	0	Singlet	Adipocyte
VAT_2M	1191.8	749	0.2230224	0	Singlet	Adipocyte
VAT_2M	1280.9	817	0.0942511	3	Singlet	FAPs
VAT_2M	1555	954	0.0520579	0	Singlet	Adipocyte
VAT_2M	3636.5	1671	14.048259	5	Singlet	Immune_cells
VAT_2M	1107.5	763	0.5763546	0	Singlet	Adipocyte
VAT_2M	4361.9	1966	4.5553308	0	Singlet	Adipocyte
VAT_2M	1128.7	926	0.0625331	6	Singlet	Neurons
VAT_2M	1835.9	1018	1.8007407	0	Singlet	Adipocyte
VAT_2M	2953	1564	2.1613055	0	Singlet	Adipocyte
VAT_2M	1382.7	946	12.429317	6	Singlet	Neurons
VAT_2M	2598.1	1335	0.1229484	0	Singlet	Adipocyte
VAT_2M	4838.2	2267	1.1722658	0	Singlet	Adipocyte
VAT_2M	4738.5	2161	5.9907505	0	Singlet	Adipocyte
VAT_2M	4070.2	1715	0	0	Singlet	Adipocyte
VAT_2M	4393.2	2101	2.2659275	0	Singlet	Adipocyte
VAT_2M	1040.7	736	9.9935014	7	Singlet	Endothelial_cells
VAT_2M	1144	760	11.54213	2	Singlet	FAPs
VAT_2M	1445.2	1116	3.6577402	11	Singlet	Smooth_muscle_cells
VAT_2M	3521.5	1759	0.0035022	0	Singlet	Adipocyte
VAT_2M	2028.4	1062	0.789959	0	Singlet	Adipocyte
VAT_2M	3554.8	1649	0.3063062	0	Singlet	Adipocyte
VAT_2M	4228.5	2002	0.0092647	0	Singlet	Adipocyte
VAT_2M	1256.2	820	6.1850024	0	Singlet	Adipocyte
VAT_2M	1552.9	777	11.474271	0	Singlet	Adipocyte
VAT_2M	4755.8	2055	0.039278	0	Singlet	Adipocyte
VAT_2M	2157	1090	7.5975392	7	Singlet	Endothelial_cells
VAT_2M	1544	1024	12.637704	5	Singlet	Immune_cells
VAT_2M	1049.9	742	6.3102948	5	Singlet	Immune_cells
VAT_2M	3992.6	1738	12.682617	0	Singlet	Adipocyte
VAT_2M	2455.1	1186	12.439291	0	Singlet	Adipocyte
VAT_2M	2091.9	1252	0.2898197	0	Singlet	Adipocyte
VAT_2M	1784.5	1113	0	3	Singlet	FAPs
VAT_2M	2244.8	1259	0.4037478	0	Singlet	Adipocyte
VAT_2M	2913.5	1546	2.6995639	0	Singlet	Adipocyte
VAT_2M	3131.3	1508	12.316955	0	Singlet	Adipocyte
VAT_2M	2961.5	1569	6.7821302	0	Singlet	Adipocyte

VAT_2M	5878.9	2467	0.1084598	0	Singlet	Adipocyte
VAT_2M	1725.1	1110	3.5756107	0	Singlet	Adipocyte
VAT_2M	1321.3	727	10.106532	0	Singlet	Adipocyte
VAT_2M	1174.5	787	9.1613095	2	Singlet	FAPs
VAT_2M	1660.5	986	1.4182283	0	Singlet	Adipocyte
VAT_2M	2316.2	1310	0	0	Singlet	Adipocyte
VAT_2M	1742.4	832	7.3661554	0	Singlet	Adipocyte
VAT_2M	1820.9	1068	0.2956727	0	Singlet	Adipocyte
VAT_2M	1702.9	1305	0.0352186	6	Singlet	Neurons
VAT_2M	1295.3	1170	0	8	Singlet	Mesothelial
VAT_2M	2584.8	1362	4.6812473	0	Singlet	Adipocyte
VAT_2M	2357.4	1389	2.288937	0	Singlet	Adipocyte
VAT_2M	1641.1	877	11.604851	5	Singlet	Immune_cells
VAT_2M	1469.3	906	0	0	Singlet	Adipocyte
VAT_2M	1143.1	746	0.0754964	0	Singlet	Adipocyte
VAT_2M	2851.6	1377	6.7808719	5	Singlet	Immune_cells
VAT_2M	4475	1848	2.6682532	0	Singlet	Adipocyte
VAT_2M	5621.7	2274	0.8702322	0	Singlet	Adipocyte
VAT_2M	4232.3	1967	5.961891	0	Singlet	Adipocyte
VAT_2M	1012.9	565	6.1542175	1	Singlet	Immune_cells
VAT_2M	1153.4	806	5.5291607	0	Singlet	Adipocyte
VAT_2M	1446.6	817	9.2114674	0	Singlet	Adipocyte
VAT_2M	6613	2548	0.1977071	0	Singlet	Adipocyte
VAT_2M	1011.6	904	6.3368994	8	Singlet	Mesothelial
VAT_2M	1009	598	11.152842	2	Singlet	FAPs
VAT_2M	5861.2	2399	0.3411723	0	Singlet	Adipocyte
VAT_2M	1212.3	808	1.4601384	5	Singlet	Immune_cells
VAT_2M	5021.1	2480	0.6453728	0	Singlet	Adipocyte
VAT_2M	1282.4	897	0.6690849	3	Singlet	FAPs
VAT_2M	1086.9	709	6.4015122	4	Singlet	Immune_cells
VAT_2M	6335.2	2124	13.745635	5	Singlet	Immune_cells
VAT_2M	4153.1	1869	1.8505705	0	Singlet	Adipocyte
VAT_2M	1117.8	754	0.0137138	5	Singlet	Immune_cells
VAT_2M	1171.8	919	1.246707	3	Singlet	FAPs
VAT_2M	1572.6	960	1.3992289	0	Singlet	Adipocyte
VAT_2M	2749.3	1543	5.9713629	2	Singlet	FAPs
VAT_2M	1242.1	1043	0.235499	5	Singlet	Immune_cells
VAT_2M	1873.4	1232	1.9190776	3	Singlet	FAPs
VAT_2M	2096.2	1215	13.521466	2	Singlet	FAPs
VAT_2M	1077.5	756	5.3538291	2	Singlet	FAPs
VAT_2M	2103.3	1196	8.5458897	0	Singlet	Adipocyte
VAT_2M	5091.4	2055	5.1405525	0	Singlet	Adipocyte
VAT_2M	5990.2	2359	12.569887	0	Singlet	Adipocyte
VAT_2M	1285.3	874	0.0074171	3	Singlet	FAPs
VAT_2M	1403.8	922	0.0380598	0	Singlet	Adipocyte
VAT_2M	1219.1	746	11.275642	5	Singlet	Immune_cells
VAT_2M	1456.5	1083	0.0324255	0	Singlet	Adipocyte
VAT_2M	2842.1	1568	0.0428707	0	Singlet	Adipocyte
VAT_2M	1299.7	740	8.8674035	5	Singlet	Immune_cells
VAT_2M	1060.1	909	0.3058409	6	Singlet	Neurons
VAT_2M	1050	825	8.41934	3	Singlet	FAPs

VAT_2M	4340.2	2020	10.26928	0	Singlet	Adipocyte
VAT_2M	1298.9	955	0.1872408	0	Singlet	Adipocyte
VAT_2M	2004.4	1093	1.7942531	0	Singlet	Adipocyte
VAT_2M	3988.5	1888	5.6139524	0	Singlet	Adipocyte
VAT_2M	4463.7	2047	6.4470035	0	Singlet	Adipocyte
VAT_2M	1818.5	1138	8.5473387	0	Singlet	Adipocyte
VAT_2M	1019.2	643	12.583169	5	Singlet	Immune_cells
VAT_2M	3584.9	1778	3.8674775	0	Singlet	Adipocyte
VAT_2M	2425.8	1286	10.913452	0	Singlet	Adipocyte
VAT_2M	5281.2	2206	6.1564977	0	Singlet	Adipocyte
VAT_2M	1433.5	929	0	6	Singlet	Neurons
VAT_2M	3091	1602	0.049054	0	Singlet	Adipocyte
VAT_2M	1298.7	845	0.4887245	0	Singlet	Adipocyte
VAT_2M	4179.7	2348	7.2322525	2	Singlet	FAPs
VAT_2M	2269.2	1149	12.627092	0	Singlet	Adipocyte
VAT_2M	3318.9	1714	3.7741318	0	Singlet	Adipocyte
VAT_2M	1907.9	1130	0.0633509	0	Singlet	Adipocyte
VAT_2M	2143.7	1219	0.4282237	0	Singlet	Adipocyte
VAT_2M	3524.5	1923	1.3527456	5	Singlet	Immune_cells
VAT_2M	1801.5	912	11.359385	0	Singlet	Adipocyte
VAT_2M	1591.9	932	13.160103	2	Singlet	FAPs
VAT_2M	1159.9	731	9.1542119	1	Singlet	Immune_cells
VAT_2M	3529.8	1787	0	3	Singlet	FAPs
VAT_2M	1082.8	960	1.097516	6	Singlet	Neurons
VAT_2M	1223.7	826	7.2989441	5	Singlet	Immune_cells
VAT_2M	1778.7	1054	0	0	Singlet	Adipocyte
VAT_2M	1180.6	824	3.8536665	5	Singlet	Immune_cells
VAT_2M	3026.1	1438	10.04855	0	Singlet	Adipocyte
VAT_2M	1999.3	1081	4.4916736	0	Singlet	Adipocyte
VAT_2M	1031.6	810	7.1157872	6	Singlet	Neurons
VAT_2M	2035.6	1160	0.1327281	0	Singlet	Adipocyte
VAT_2M	2141.9	1207	2.8774731	0	Singlet	Adipocyte
VAT_2M	1218.8	798	0.0345732	0	Singlet	Adipocyte
VAT_2M	5603.4	2405	1.0182245	0	Singlet	Adipocyte
VAT_2M	1733.8	1165	0	0	Singlet	Adipocyte
VAT_2M	1002.2	849	4.0762202	8	Singlet	Mesothelial
VAT_2M	1588	996	0.1017024	0	Singlet	Adipocyte
VAT_2M	1408.7	757	9.3733373	2	Singlet	FAPs
VAT_2M	1550.6	864	0.1848813	0	Singlet	Adipocyte
VAT_2M	1704.5	940	12.016175	5	Singlet	Immune_cells
VAT_2M	2301	1348	1.0835947	3	Singlet	FAPs
VAT_2M	1089.8	836	2.1226065	4	Singlet	Immune_cells
VAT_2M	1095.9	807	14.596239	6	Singlet	Neurons
VAT_2M	3379.9	1692	0.9903701	0	Singlet	Adipocyte
VAT_2M	6001.1	2542	0.8566024	0	Singlet	Adipocyte
VAT_2M	1523.3	1030	9.1410782	5	Singlet	Immune_cells
VAT_2M	1206.2	832	0.063586	7	Singlet	Endothelial_cells
VAT_2M	2983.7	1443	6.7549183	0	Singlet	Adipocyte
VAT_2M	3474.4	1778	5.7191074	5	Singlet	Immune_cells
VAT_2M	1008.5	762	9.2430511	2	Singlet	FAPs
VAT_2M	3909.8	1723	0.0573508	0	Singlet	Adipocyte

VAT_2M	5322.3	2295	2.5394134	0	Singlet	Adipocyte
VAT_2M	2603.5	1414	0	0	Singlet	Adipocyte
VAT_2M	2591.7	1428	0.0408528	0	Singlet	Adipocyte
VAT_2M	6132.5	2430	4.7684859	0	Singlet	Adipocyte
VAT_2M	2698.9	1418	4.7036465	0	Singlet	Adipocyte
VAT_2M	2129.9	1148	13.651922	3	Singlet	FAPs
VAT_2M	1159.7	792	9.3716614	0	Singlet	Adipocyte
VAT_2M	2486.7	1272	1.4195977	0	Singlet	Adipocyte
VAT_2M	1229.8	751	9.0565897	5	Singlet	Immune_cells
VAT_2M	1910.9	1035	0.6788204	0	Singlet	Adipocyte
VAT_2M	2707.7	1243	13.958847	0	Singlet	Adipocyte
VAT_2M	2820	1568	0	3	Singlet	FAPs
VAT_2M	1764.8	1015	0.1192232	0	Singlet	Adipocyte
VAT_2M	1792.5	988	0.4769383	0	Singlet	Adipocyte
VAT_2M	1165.9	780	1.1286949	0	Singlet	Adipocyte
VAT_2M	3146.4	1487	8.3728608	0	Singlet	Adipocyte
VAT_2M	5137.1	2153	0.1804992	0	Singlet	Adipocyte
VAT_2M	2574.5	1369	0	0	Singlet	Adipocyte
VAT_2M	1105.9	720	10.918269	2	Singlet	FAPs
VAT_2M	1996.3	1185	0.1978168	0	Singlet	Adipocyte
VAT_2M	3269.1	1769	1.4848294	0	Singlet	Adipocyte
VAT_2M	3283.3	1611	5.8210148	0	Singlet	Adipocyte
VAT_2M	4866.5	2166	0	0	Singlet	Adipocyte
VAT_2M	2674.3	1430	0	0	Singlet	Adipocyte
VAT_2M	1987.8	1175	0.0389761	0	Singlet	Adipocyte
VAT_2M	1005.8	726	13.777846	6	Singlet	Neurons
VAT_2M	1197.3	769	0.8833463	0	Singlet	Adipocyte
VAT_2M	4222	1982	0.029306	0	Singlet	Adipocyte
VAT_2M	1038.1	748	4.611979	5	Singlet	Immune_cells
VAT_2M	1746.4	1031	0.1758297	0	Singlet	Adipocyte
VAT_2M	1811	1031	3.1456906	0	Singlet	Adipocyte
VAT_2M	1823	1089	4.165428	0	Singlet	Adipocyte
VAT_2M	1014.2	631	0.0129759	0	Singlet	Adipocyte
VAT_2M	3157	1628	7.0916694	0	Singlet	Adipocyte
VAT_2M	1596.6	1144	12.012379	2	Singlet	FAPs
VAT_2M	1961.6	1526	0.047917	6	Singlet	Neurons
VAT_2M	1362.7	778	13.702925	0	Singlet	Adipocyte
VAT_2M	1155.2	754	7.0344374	5	Singlet	Immune_cells
VAT_2M	2529.9	1435	2.1270972	0	Singlet	Adipocyte
VAT_2M	3703.9	1769	4.405349	0	Singlet	Adipocyte
VAT_2M	1951.5	969	10.025564	0	Singlet	Adipocyte
VAT_2M	2728	1512	5.1778379	0	Singlet	Adipocyte
VAT_2M	3385.8	1704	3.9903138	0	Singlet	Adipocyte
VAT_2M	3719.9	1533	0.1014569	0	Singlet	Adipocyte
VAT_2M	1586	1501	0.3304657	6	Singlet	Neurons
VAT_2M	6227.7	2544	6.4243749	0	Singlet	Adipocyte
VAT_2M	4942.9	2218	0.8943707	0	Singlet	Adipocyte
VAT_2M	2684.1	1352	7.7989312	3	Singlet	FAPs
VAT_2M	3175.1	1515	12.422614	0	Singlet	Adipocyte
VAT_2M	4242.1	2090	1.7417323	0	Singlet	Adipocyte
VAT_2M	7937.7	2978	0.302031	0	Singlet	Adipocyte

VAT_2M	3112.3	1683	0	0	Singlet	Adipocyte
VAT_2M	6274.3	2445	0.0042458	0	Singlet	Adipocyte
VAT_2M	1921.6	997	8.111756	2	Singlet	FAPs
VAT_2M	2283.2	1216	12.694008	2	Singlet	FAPs
VAT_2M	1097.2	761	0	7	Singlet	Endothelial_cells
VAT_2M	1263.9	623	14.405202	5	Singlet	Immune_cells
VAT_2M	2114.9	1209	0.0274872	7	Singlet	Endothelial_cells
VAT_2M	2714.2	1775	2.8117901	5	Singlet	Immune_cells
VAT_2M	1299.3	849	4.7572989	0	Singlet	Adipocyte
VAT_2M	2302.7	1231	0.0466983	0	Singlet	Adipocyte
VAT_2M	2363.6	1345	1.5624469	0	Singlet	Adipocyte
VAT_2M	2076.1	1134	9.7019611	0	Singlet	Adipocyte
VAT_2M	2855.6	1455	0.2683787	0	Singlet	Adipocyte
VAT_2M	1133.1	760	7.8032803	2	Singlet	FAPs
VAT_2M	1801.1	996	4.8288722	2	Singlet	FAPs
VAT_2M	3672.7	1765	0.1925562	0	Singlet	Adipocyte
VAT_2M	3928.8	1917	4.6119508	0	Singlet	Adipocyte
VAT_2M	1174	956	8.8958022	5	Singlet	Immune_cells
VAT_2M	4077.9	2002	9.3353738	0	Singlet	Adipocyte
VAT_2M	1679.1	1111	3.3673477	5	Singlet	Immune_cells
VAT_2M	3767.7	2107	1.7609139	5	Singlet	Immune_cells
VAT_2M	3114.7	1797	1.5301232	0	Singlet	Adipocyte
VAT_2M	2767.3	1445	0.8107982	0	Singlet	Adipocyte
VAT_2M	2469.9	1258	0.5054796	0	Singlet	Adipocyte
VAT_2M	2040.7	1100	0.5292321	0	Singlet	Adipocyte
VAT_2M	1463	896	1.1921892	5	Singlet	Immune_cells
VAT_2M	2804.3	1450	1.1296939	0	Singlet	Adipocyte
VAT_2M	1412.6	940	13.569556	0	Singlet	Adipocyte
VAT_2M	3754.8	1944	4.64916	3	Singlet	FAPs
VAT_2M	1255.5	980	4.082289	2	Singlet	FAPs
VAT_2M	1228.5	825	0.0609071	0	Singlet	Adipocyte
VAT_2M	3912.3	1979	1.7844812	0	Singlet	Adipocyte
VAT_2M	3435.7	1612	7.7805972	0	Singlet	Adipocyte
VAT_2M	5260.8	2137	10.839903	0	Singlet	Adipocyte
VAT_2M	1663.9	1026	4.1635324	0	Singlet	Adipocyte
VAT_2M	5296	2100	9.9864281	0	Singlet	Adipocyte
VAT_2M	2570.8	1339	0.312296	0	Singlet	Adipocyte
VAT_2M	1048.4	628	11.434483	3	Singlet	FAPs
VAT_2M	1300.3	964	1.5971138	1	Singlet	Immune_cells
VAT_2M	1709.7	1223	2.6881226	0	Singlet	Adipocyte
VAT_2M	1952.8	1134	7.6377347	2	Singlet	FAPs
VAT_2M	2394.2	1248	8.8386295	0	Singlet	Adipocyte
VAT_2M	1043	798	0.0348892	3	Singlet	FAPs
VAT_2M	1835.5	1017	0.0016883	0	Singlet	Adipocyte
VAT_2M	7821.7	2743	6.0344764	7	Singlet	Endothelial_cells
VAT_2M	1598.6	942	0.1166833	0	Singlet	Adipocyte
VAT_2M	1971.1	1097	4.9119122	0	Singlet	Adipocyte
VAT_2M	1459.7	1047	3.9552737	6	Singlet	Neurons
VAT_2M	1652.8	1093	7.0302225	2	Singlet	FAPs
VAT_2M	11530	3730	4.3504597	0	Singlet	Adipocyte
VAT_2M	1491.2	846	5.3488496	0	Singlet	Adipocyte

VAT_2M	1133.5	810	0.252855	0	Singlet	Adipocyte
VAT_2M	2008.1	1197	0.8524208	0	Singlet	Adipocyte
VAT_2M	2426.4	1320	3.6762631	0	Singlet	Adipocyte
VAT_2M	1209.1	741	6.6533176	0	Singlet	Adipocyte
VAT_2M	3040.6	1387	12.007452	0	Singlet	Adipocyte
VAT_2M	2067.2	1146	0.1600598	0	Singlet	Adipocyte
VAT_2M	1743.5	1197	0	0	Singlet	Adipocyte
VAT_2M	1824.2	938	14.649657	5	Singlet	Immune_cells
VAT_2M	5285.1	2422	6.7233561	0	Singlet	Adipocyte
VAT_2M	5677.9	2338	8.3076415	0	Singlet	Adipocyte
VAT_2M	4385.2	1873	13.703626	0	Singlet	Adipocyte
VAT_2M	3526.6	1697	1.9362351	0	Singlet	Adipocyte
VAT_2M	1867.5	1122	3.7942116	0	Singlet	Adipocyte
VAT_2M	1362.3	916	0.0320303	0	Singlet	Adipocyte
VAT_2M	5439.2	2311	4.6918859	0	Singlet	Adipocyte
VAT_2M	3344.3	1659	13.051065	0	Singlet	Adipocyte
VAT_2M	1644.9	1002	5.3462476	0	Singlet	Adipocyte
VAT_2M	1199.8	1120	0.0249381	6	Singlet	Neurons
VAT_2M	2164.8	1253	0	0	Singlet	Adipocyte
VAT_2M	3532.2	1754	3.3333567	0	Singlet	Adipocyte
VAT_2M	5433.9	2025	7.5046514	0	Singlet	Adipocyte
VAT_2M	5547.9	2342	0.0210006	0	Singlet	Adipocyte
VAT_2M	2277	1293	3.8300046	0	Singlet	Adipocyte
VAT_2M	2940.3	1548	1.9609469	0	Singlet	Adipocyte
VAT_2M	1386.5	834	13.874294	5	Singlet	Immune_cells
VAT_2M	4447.9	1930	4.4252935	0	Singlet	Adipocyte
VAT_2M	1641.8	1038	0.0192726	0	Singlet	Adipocyte
VAT_2M	1992.1	1102	13.57168	0	Singlet	Adipocyte
VAT_2M	1699.5	912	10.181608	0	Singlet	Adipocyte
VAT_2M	4137.6	1929	0.389012	0	Singlet	Adipocyte
VAT_2M	5524.7	2150	0	0	Singlet	Adipocyte
VAT_2M	1003.1	665	6.0757949	3	Singlet	FAPs
VAT_2M	1856.3	1112	0.1213861	0	Singlet	Adipocyte
VAT_2M	1357.7	838	0.7763848	0	Singlet	Adipocyte
VAT_2M	1200	789	2.3453939	7	Singlet	Endothelial_cells
VAT_2M	1881.9	1070	4.0770548	0	Singlet	Adipocyte
VAT_2M	1749.5	1049	1.1061153	0	Singlet	Adipocyte
VAT_2M	1399.8	876	8.7626564	3	Singlet	FAPs
VAT_2M	2549.9	1405	4.0117842	0	Singlet	Adipocyte
VAT_2M	1131.8	607	0.3990155	0	Singlet	Adipocyte
VAT_2M	2884.7	1502	7.3987437	0	Singlet	Adipocyte
VAT_2M	3473.2	1845	5.0521412	0	Singlet	Adipocyte
VAT_2M	2436.9	1361	8.1578969	2	Singlet	FAPs
VAT_2M	3079.8	1520	0.8286589	0	Singlet	Adipocyte
VAT_2M	8558.1	2890	5.2961525	0	Singlet	Adipocyte
VAT_2M	1409.7	1177	0.3463796	6	Singlet	Neurons
VAT_2M	2654.2	1406	0.3561466	0	Singlet	Adipocyte
VAT_2M	2722.7	1428	0	0	Singlet	Adipocyte
VAT_2M	1767.5	1035	6.7274201	0	Singlet	Adipocyte
VAT_2M	3025.6	1827	2.1565731	2	Singlet	FAPs
VAT_2M	1684.8	1178	2.5031793	5	Singlet	Immune_cells

VAT_2M	1697.6	982	13.019802	3	Singlet	FAPs
VAT_2M	2362	1301	2.4957447	5	Singlet	Immune_cells
VAT_2M	5059.1	2241	0.0417156	0	Singlet	Adipocyte
VAT_2M	1389.6	872	9.3202485	2	Singlet	FAPs
VAT_2M	2379.9	1425	11.179949	2	Singlet	FAPs
VAT_2M	1769.7	897	13.708888	5	Singlet	Immune_cells
VAT_2M	2290.1	1198	12.869442	5	Singlet	Immune_cells
VAT_2M	1417.2	802	10.099144	0	Singlet	Adipocyte
VAT_2M	3946.2	1892	0.8859622	0	Singlet	Adipocyte
VAT_2M	1952.6	1328	14.808031	2	Singlet	FAPs
VAT_2M	1281.1	886	7.4506042	0	Singlet	Adipocyte
VAT_2M	2325.2	1310	0	0	Singlet	Adipocyte
VAT_2M	1494.8	910	5.8775222	0	Singlet	Adipocyte
VAT_2M	1804.5	1060	7.296313	5	Singlet	Immune_cells
VAT_2M	1352.8	1302	0.01115	4	Singlet	Immune_cells
VAT_2M	1928.9	1115	1.418317	0	Singlet	Adipocyte
VAT_2M	1959.1	1370	0.2108296	0	Singlet	Adipocyte
VAT_2M	5566.9	2271	2.459589	0	Singlet	Adipocyte
VAT_2M	3696.2	2211	1.9667841	2	Singlet	FAPs
VAT_2M	3926.2	1919	0.1057033	0	Singlet	Adipocyte
VAT_2M	1676.4	1010	3.4700749	2	Singlet	FAPs
VAT_2M	1089.4	980	0.856604	6	Singlet	Neurons
VAT_2M	1206.2	1082	7.6902101	6	Singlet	Neurons
VAT_2M	7254.4	2707	0.2281598	0	Singlet	Adipocyte
VAT_2M	1020	856	7.2213715	6	Singlet	Neurons
VAT_2M	1152	798	0.1126951	5	Singlet	Immune_cells
VAT_2M	1387.1	860	3.7794974	0	Singlet	Adipocyte
VAT_2M	1474.6	1082	1.0280926	0	Singlet	Adipocyte
VAT_2M	1924.1	1147	4.3637494	0	Singlet	Adipocyte
VAT_2M	2879.8	1591	2.849192	0	Singlet	Adipocyte
VAT_2M	1333.9	926	8.1421099	0	Singlet	Adipocyte
VAT_2M	2644.6	1328	0.0239514	0	Singlet	Adipocyte
VAT_2M	1381.4	846	4.106854	0	Singlet	Adipocyte
VAT_2M	4216.7	2005	3.6027665	0	Singlet	Adipocyte
VAT_2M	3671.5	1781	0.5216815	0	Singlet	Adipocyte
VAT_2M	5583.1	2371	2.5737682	0	Singlet	Adipocyte
VAT_2M	3574.9	1791	4.1467911	5	Singlet	Immune_cells
VAT_2M	1893.5	1134	1.7563303	0	Singlet	Adipocyte
VAT_2M	2019.5	1352	11.097851	5	Singlet	Immune_cells
VAT_2M	1626.3	979	10.648554	0	Singlet	Adipocyte
VAT_2M	1309.5	872	5.1876487	0	Singlet	Adipocyte
VAT_2M	2241.5	1167	0.1111727	0	Singlet	Adipocyte
VAT_2M	1697	1023	0.0238867	0	Singlet	Adipocyte
VAT_2M	3605.1	1884	0.2307055	0	Singlet	Adipocyte
VAT_2M	2321.2	1366	2.4954764	0	Singlet	Adipocyte
VAT_2M	1198.6	886	0.0242124	3	Singlet	FAPs
VAT_2M	1668.1	1120	2.3024672	11	Singlet	Smooth_muscle_cells
VAT_2M	1886.6	1110	3.2508399	0	Singlet	Adipocyte
VAT_2M	2072.5	1172	4.2519457	0	Singlet	Adipocyte
VAT_2M	2302	1270	14.533733	0	Singlet	Adipocyte
VAT_2M	3367.4	1616	12.483363	0	Singlet	Adipocyte

VAT_2M	3561.2	1903	5.0661711	0	Singlet	Adipocyte
VAT_2M	2216.8	1238	0.4839201	0	Singlet	Adipocyte
VAT_2M	1596.4	993	0.2658811	0	Singlet	Adipocyte
VAT_2M	5806.3	2338	1.1188413	0	Singlet	Adipocyte
VAT_2M	2116	1616	5.8133699	2	Singlet	FAPs
VAT_2M	9698.3	3223	4.0311465	0	Singlet	Adipocyte
VAT_2M	1872.1	1082	6.4131025	3	Singlet	FAPs
VAT_2M	3504.1	1589	11.947251	3	Singlet	FAPs
VAT_2M	5575.9	2451	3.9517005	0	Singlet	Adipocyte
VAT_2M	3806.3	1749	10.124417	0	Singlet	Adipocyte
VAT_2M	1426.9	1024	1.2620616	0	Singlet	Adipocyte
VAT_2M	1357.4	826	0	0	Singlet	Adipocyte
VAT_2M	2284.5	1286	3.8770605	3	Singlet	FAPs
VAT_2M	1581.4	986	12.356904	5	Singlet	Immune_cells
VAT_2M	5404.2	2236	12.075524	0	Singlet	Adipocyte
VAT_2M	1646.7	1141	2.8522004	5	Singlet	Immune_cells
VAT_2M	1918.6	1086	13.696437	2	Singlet	FAPs
VAT_2M	1695.2	987	0.0475848	7	Singlet	Endothelial_cells
VAT_2M	1625.3	1014	2.2879578	3	Singlet	FAPs
VAT_2M	2684.5	1430	0.3948161	0	Singlet	Adipocyte
VAT_2M	1810.9	1243	0.0741769	3	Singlet	FAPs
VAT_2M	3788.3	1755	0.0122651	0	Singlet	Adipocyte
VAT_2M	4625.2	2012	0.5273792	0	Singlet	Adipocyte
VAT_2M	2263.6	1233	14.543498	0	Singlet	Adipocyte
VAT_2M	1211.3	1073	2.8600118	6	Singlet	Neurons
VAT_2M	1735.5	963	1.4892711	0	Singlet	Adipocyte
VAT_2M	1056.7	905	10.849661	1	Singlet	Immune_cells
VAT_2M	1549.3	992	1.2337022	3	Singlet	FAPs
VAT_2M	1676.9	998	2.413	0	Singlet	Adipocyte
VAT_2M	2740.4	1508	4.0019526	2	Singlet	FAPs
VAT_2M	2244.6	1407	2.4428554	2	Singlet	FAPs
VAT_2M	2570.2	1411	0	0	Singlet	Adipocyte
VAT_2M	1183.4	776	5.9210627	2	Singlet	FAPs
VAT_2M	1349.4	888	11.475554	0	Singlet	Adipocyte
VAT_2M	1305.5	975	0	3	Singlet	FAPs
VAT_2M	2627.8	1523	0.7679609	0	Singlet	Adipocyte
VAT_2M	1746.3	902	0.0011786	0	Singlet	Adipocyte
VAT_2M	2646.8	1509	10.765559	0	Singlet	Adipocyte
VAT_2M	7942.8	3047	3.1199775	0	Singlet	Adipocyte
VAT_2M	2528	1464	0.0993294	0	Singlet	Adipocyte
VAT_2M	4384.2	2051	1.2549287	0	Singlet	Adipocyte
VAT_2M	3653.5	2077	1.5392505	7	Singlet	Endothelial_cells
VAT_2M	5054.5	2254	2.107981	0	Singlet	Adipocyte
VAT_2M	2382.3	1520	3.4686403	2	Singlet	FAPs
VAT_2M	1834.4	1101	1.2036534	0	Singlet	Adipocyte
VAT_2M	2795.8	1551	3.7210656	5	Singlet	Immune_cells
VAT_2M	4655.3	2106	11.659708	0	Singlet	Adipocyte
VAT_2M	1012.4	649	0.0387467	0	Singlet	Adipocyte
VAT_2M	1643.1	1015	0.8460115	0	Singlet	Adipocyte
VAT_2M	1200.2	941	5.3072366	0	Singlet	Adipocyte
VAT_2M	1637.1	981	4.6938657	0	Singlet	Adipocyte

VAT_2M	2673.6	1461	1.8779446	0	Singlet	Adipocyte
VAT_2M	1417.1	927	3.7726383	3	Singlet	FAPs
VAT_2M	1051.9	685	14.000076	9	Singlet	Mesothelial
VAT_2M	5856.7	2541	8.787316	0	Singlet	Adipocyte
VAT_2M	1146.9	720	12.018745	2	Singlet	FAPs
VAT_2M	1837.2	995	11.687567	7	Singlet	Endothelial_cells
VAT_2M	1196.9	966	0.0652303	3	Singlet	FAPs
VAT_2M	2154.4	1157	5.4542515	0	Singlet	Adipocyte
VAT_2M	4649.7	2071	12.072362	0	Singlet	Adipocyte
VAT_2M	3120.6	1736	0.3072387	5	Singlet	Immune_cells
VAT_2M	5820.7	2443	3.5775151	0	Singlet	Adipocyte
VAT_2M	9676.1	3145	7.4785121	0	Singlet	Adipocyte
VAT_2M	1661.8	1027	0.6449152	0	Singlet	Adipocyte
VAT_2M	2778.7	1386	13.384726	0	Singlet	Adipocyte
VAT_2M	1823.2	1026	13.748171	3	Singlet	FAPs
VAT_2M	1298.4	1134	1.6475141	6	Singlet	Neurons
VAT_2M	1141.1	780	5.0614731	5	Singlet	Immune_cells
VAT_2M	5338.9	2317	7.6761397	0	Singlet	Adipocyte
VAT_2M	1946.4	1048	4.8109701	0	Singlet	Adipocyte
VAT_2M	1479.8	845	0	0	Singlet	Adipocyte
VAT_2M	2884.1	1412	14.677815	0	Singlet	Adipocyte
VAT_2M	1416.9	895	0.0851896	7	Singlet	Endothelial_cells
VAT_2M	3280.3	1753	0.3071573	0	Singlet	Adipocyte
VAT_2M	2887.4	1441	5.9190923	0	Singlet	Adipocyte
VAT_2M	3978.4	1969	0	0	Singlet	Adipocyte
VAT_2M	1385.1	871	7.7180362	5	Singlet	Immune_cells
VAT_2M	4200.7	1877	4.3765314	0	Singlet	Adipocyte
VAT_2M	2264.6	1165	0.1394085	0	Singlet	Adipocyte
VAT_2M	1061.3	624	13.647845	4	Singlet	Immune_cells
VAT_2M	1000.3	763	5.9522124	6	Singlet	Neurons
VAT_2M	4550.3	2045	0	0	Singlet	Adipocyte
VAT_2M	2282.4	1285	1.8182673	0	Singlet	Adipocyte
VAT_2M	1911.7	1197	0.4495627	0	Singlet	Adipocyte
VAT_2M	1483.1	857	8.518474	0	Singlet	Adipocyte
VAT_2M	3972.3	1896	1.5942624	0	Singlet	Adipocyte
VAT_2M	4962	2206	0.1642495	0	Singlet	Adipocyte
VAT_2M	1668.9	978	0.129122	0	Singlet	Adipocyte
VAT_2M	3313.7	1611	7.9401652	0	Singlet	Adipocyte
VAT_2M	1251.7	917	3.0496955	2	Singlet	FAPs
VAT_2M	2986.6	1526	5.2645208	0	Singlet	Adipocyte
VAT_2M	1162.7	710	7.3126627	2	Singlet	FAPs
VAT_2M	1189.2	771	0.8892521	3	Singlet	FAPs
VAT_2M	1629.3	766	12.501747	5	Singlet	Immune_cells
VAT_2M	1540.6	996	8.0074875	5	Singlet	Immune_cells
VAT_2M	1008.8	638	14.975501	3	Singlet	FAPs
VAT_2M	2877.6	1329	13.790571	0	Singlet	Adipocyte
VAT_2M	1347.9	871	0.1971019	0	Singlet	Adipocyte
VAT_2M	1044.1	717	4.0373084	5	Singlet	Immune_cells
VAT_2M	1146.8	782	14.411575	0	Singlet	Adipocyte
VAT_2M	2039.1	1066	2.5292003	0	Singlet	Adipocyte
VAT_2M	1920.7	1112	0.0628377	0	Singlet	Adipocyte

VAT_2M	3208.8	1688	0	0	Singlet	Adipocyte
VAT_2M	1069.4	695	1.320541	0	Singlet	Adipocyte
VAT_2M	2020.5	1143	0.6622877	0	Singlet	Adipocyte
VAT_2M	2431.9	1633	3.5226366	2	Singlet	FAPs
VAT_2M	1364.8	911	0	0	Singlet	Adipocyte
VAT_2M	2591.5	1419	1.6882595	3	Singlet	FAPs
VAT_2M	1266.5	791	8.4813921	5	Singlet	Immune_cells
VAT_2M	1717.3	1190	7.6528638	0	Singlet	Adipocyte
VAT_2M	4642.2	2153	2.0467095	0	Singlet	Adipocyte
VAT_2M	1073.1	708	3.5042821	5	Singlet	Immune_cells
VAT_2M	1814.5	977	12.535026	2	Singlet	FAPs
VAT_2M	4537.2	2139	2.5844295	0	Singlet	Adipocyte
VAT_2M	1076.7	639	9.3980718	3	Singlet	FAPs
VAT_2M	1034.7	665	0	0	Singlet	Adipocyte
VAT_2M	1036.2	809	10.820749	6	Singlet	Neurons
VAT_2M	1246.4	781	0.5069491	0	Singlet	Adipocyte
VAT_2M	1136	590	7.3334285	0	Singlet	Adipocyte
VAT_2M	1008.2	702	0.3882351	3	Singlet	FAPs
VAT_2M	2127.2	1172	0.6962175	0	Singlet	Adipocyte
VAT_2M	1274.8	888	11.982413	3	Singlet	FAPs
VAT_2M	2313.9	1318	0.0786177	0	Singlet	Adipocyte
VAT_2M	2733.9	1552	0.819186	0	Singlet	Adipocyte
VAT_2M	1046.8	678	0.4172303	0	Singlet	Adipocyte
VAT_2M	2997.6	1378	0.2923204	0	Singlet	Adipocyte
VAT_2M	2607.1	1330	7.8677407	0	Singlet	Adipocyte
VAT_2M	1394.1	712	1.8715	0	Singlet	Adipocyte
VAT_2M	3042.8	2033	5.5868594	9	Singlet	Mesothelial
VAT_2M	1563.6	1070	0.3273508	2	Singlet	FAPs
VAT_2M	1068.2	728	2.2433254	3	Singlet	FAPs
VAT_2M	2653.8	1577	0.1893509	5	Singlet	Immune_cells
VAT_2M	2294	1450	0.0889874	2	Singlet	FAPs
VAT_2M	3732.3	1793	0.0123027	0	Singlet	Adipocyte
VAT_2M	1826.2	1074	3.7673881	0	Singlet	Adipocyte
VAT_2M	4627.9	2087	0.1779243	0	Singlet	Adipocyte
VAT_2M	4161.8	1920	0	0	Singlet	Adipocyte
VAT_2M	2961.2	1625	4.9184138	0	Singlet	Adipocyte
VAT_2M	2835.3	1486	7.461265	3	Singlet	FAPs
VAT_2M	1996.2	1241	3.280778	0	Singlet	Adipocyte
VAT_2M	1892.1	1104	0.0659716	0	Singlet	Adipocyte
VAT_2M	4192.7	1972	0.0436275	0	Singlet	Adipocyte
VAT_2M	2395.6	1193	14.593139	0	Singlet	Adipocyte
VAT_2M	1877.3	1009	0.3049263	0	Singlet	Adipocyte
VAT_2M	3282.7	1668	0.0157671	0	Singlet	Adipocyte
VAT_2M	1319.1	659	9.1033843	3	Singlet	FAPs
VAT_2M	4786.1	2113	6.9716308	0	Singlet	Adipocyte
VAT_2M	1187.4	924	13.711854	6	Singlet	Neurons
VAT_2M	3537.5	1812	0.0171833	0	Singlet	Adipocyte
VAT_2M	3294.7	1636	0.4919538	0	Singlet	Adipocyte
VAT_2M	1161.6	846	1.56074	6	Singlet	Neurons
VAT_2M	3581.2	1726	0.3515703	0	Singlet	Adipocyte
VAT_2M	1458.7	1029	0	5	Singlet	Immune_cells

VAT_2M	2767.9	1601	0.2174993	0	Singlet	Adipocyte
VAT_2M	1609.5	1041	1.2170113	0	Singlet	Adipocyte
VAT_2M	1694.9	937	8.7670582	0	Singlet	Adipocyte
VAT_2M	1097.6	714	7.0145147	0	Singlet	Adipocyte
VAT_2M	5553.5	2490	1.9277089	0	Singlet	Adipocyte
VAT_2M	2070	1306	2.9331472	0	Singlet	Adipocyte
VAT_2M	1629.5	1036	0.0912994	3	Singlet	FAPs
VAT_2M	4460.4	2801	3.7748224	2	Singlet	FAPs
VAT_2M	8630.8	3242	4.995577	0	Singlet	Adipocyte
VAT_2M	2756.4	1537	0.1663999	0	Singlet	Adipocyte
VAT_2M	5353.1	2469	4.2464862	0	Singlet	Adipocyte
VAT_2M	3106.2	1583	13.246175	0	Singlet	Adipocyte
VAT_2M	1407.9	758	12.447104	0	Singlet	Adipocyte
VAT_2M	2671.9	1467	0	0	Singlet	Adipocyte
VAT_2M	1182.9	854	0.1227407	0	Singlet	Adipocyte
VAT_2M	5774.8	2389	2.67926	0	Singlet	Adipocyte
VAT_2M	1209.8	894	12.709366	8	Singlet	Mesothelial
VAT_2M	2164.3	1225	0	0	Singlet	Adipocyte
VAT_2M	2548.5	1376	0.0549399	0	Singlet	Adipocyte
VAT_2M	3927.9	2040	0	0	Singlet	Adipocyte
VAT_2M	2286.9	1212	3.3149638	0	Singlet	Adipocyte
VAT_2M	4540	2014	0	0	Singlet	Adipocyte
VAT_2M	5481.7	2574	0.2335352	3	Singlet	FAPs
VAT_2M	3052	1397	0.1457416	0	Singlet	Adipocyte
VAT_2M	1018	914	0.0481273	6	Singlet	Neurons
VAT_2M	1546.3	884	0.6796098	0	Singlet	Adipocyte
VAT_2M	2907.7	1577	1.1324304	0	Singlet	Adipocyte
VAT_2M	4641	2243	0.6274439	0	Singlet	Adipocyte
VAT_2M	1441.1	1112	0.2351329	0	Singlet	Adipocyte
VAT_2M	5623.6	2358	7.2394378	0	Singlet	Adipocyte
VAT_2M	1646.6	1022	4.6425005	0	Singlet	Adipocyte
VAT_2M	2118	1273	4.8406242	2	Singlet	FAPs
VAT_2M	3132.9	1669	1.0604416	0	Singlet	Adipocyte
VAT_2M	1585.6	860	5.9704246	0	Singlet	Adipocyte
VAT_2M	3847.9	2141	10.677297	2	Singlet	FAPs
VAT_2M	6233	2609	0.4078437	0	Singlet	Adipocyte
VAT_2M	1225.6	906	2.0850442	0	Singlet	Adipocyte
VAT_2M	2329.1	1307	13.055142	0	Singlet	Adipocyte
VAT_2M	2444.2	1305	0.2119371	0	Singlet	Adipocyte
VAT_2M	1952.2	1103	0	0	Singlet	Adipocyte
VAT_2M	1414.8	871	0.402862	0	Singlet	Adipocyte
VAT_2M	1237.2	950	11.007023	2	Singlet	FAPs
VAT_2M	6798	2617	9.4965358	0	Singlet	Adipocyte
VAT_2M	3611.8	1823	1.4230055	0	Singlet	Adipocyte
VAT_2M	3962.2	1819	7.6597446	0	Singlet	Adipocyte
VAT_2M	2047.4	1178	5.8056202	0	Singlet	Adipocyte
VAT_2M	1190.4	671	12.268732	6	Singlet	Neurons
VAT_2M	1527.7	966	12.44228	2	Singlet	FAPs
VAT_2M	1169	969	0	6	Singlet	Neurons
VAT_2M	2349.3	1379	1.3455865	0	Singlet	Adipocyte
VAT_2M	6612	2519	5.5452435	0	Singlet	Adipocyte

VAT_2M	7414	2795	0.0120147	0	Singlet	Adipocyte
VAT_2M	2057	1349	4.8353908	5	Singlet	Immune_cells
VAT_2M	3614.4	1849	0	0	Singlet	Adipocyte
VAT_2M	1864.5	1011	3.7115813	0	Singlet	Adipocyte
VAT_2M	2432.3	1295	9.9429476	0	Singlet	Adipocyte
VAT_2M	2172.2	1196	6.2447373	0	Singlet	Adipocyte
VAT_2M	3982.3	1877	2.8347856	0	Singlet	Adipocyte
VAT_2M	3179.2	1707	3.6923436	0	Singlet	Adipocyte
VAT_2M	2503.4	1462	3.0748158	0	Singlet	Adipocyte
VAT_2M	3853.7	1723	10.803334	0	Singlet	Adipocyte
VAT_2M	3780	1877	5.0129621	0	Singlet	Adipocyte
VAT_2M	1319	1099	2.3561486	11	Singlet	Smooth_muscle_cells
VAT_2M	1191.6	747	9.375945	2	Singlet	FAPs
VAT_2M	1893.1	1112	0.9054102	0	Singlet	Adipocyte
VAT_2M	1221.1	983	0	6	Singlet	Neurons
VAT_2M	3865.4	2063	0.2413128	0	Singlet	Adipocyte
VAT_2M	2843.3	1533	0.4006486	0	Singlet	Adipocyte
VAT_2M	5218.6	2281	0.1919316	0	Singlet	Adipocyte
VAT_2M	2932.7	1530	3.7540601	0	Singlet	Adipocyte
VAT_2M	2624.6	1549	3.9396416	2	Singlet	FAPs
VAT_2M	3476.9	1558	9.6664561	5	Singlet	Immune_cells
VAT_2M	6327.2	2608	6.3598122	0	Singlet	Adipocyte
VAT_2M	1055.5	718	0.8604251	3	Singlet	FAPs
VAT_2M	1481.8	866	0.3604237	0	Singlet	Adipocyte
VAT_2M	1410.1	831	0.9518485	0	Singlet	Adipocyte
VAT_2M	1667.2	1012	1.6627665	3	Singlet	FAPs
VAT_2M	1058.9	675	5.889921	0	Singlet	Adipocyte
VAT_2M	1178.8	697	9.4584236	0	Singlet	Adipocyte
VAT_2M	7540.4	2797	6.7961968	0	Singlet	Adipocyte
VAT_2M	2904.4	1583	0.3455716	0	Singlet	Adipocyte
VAT_2M	3481	1630	0.977473	0	Singlet	Adipocyte
VAT_2M	3234.7	1579	3.9788454	0	Singlet	Adipocyte
VAT_2M	4514.1	2027	12.434006	0	Singlet	Adipocyte
VAT_2M	1722.6	1071	0	3	Singlet	FAPs
VAT_2M	5897.3	2554	7.9715878	0	Singlet	Adipocyte
VAT_2M	1242	759	0.1282681	0	Singlet	Adipocyte
VAT_2M	1520.2	1050	3.809708	2	Singlet	FAPs
VAT_2M	1441.6	856	6.0646228	0	Singlet	Adipocyte
VAT_2M	1533	913	0	0	Singlet	Adipocyte
VAT_2M	7806.7	2850	0	0	Singlet	Adipocyte
VAT_2M	1204.9	844	0.2094563	3	Singlet	FAPs
VAT_2M	1254.4	778	0	0	Singlet	Adipocyte
VAT_2M	3010.6	1672	0.1832645	0	Singlet	Adipocyte
VAT_2M	5271	2522	0.0231528	0	Singlet	Adipocyte
VAT_2M	3712	1658	14.750538	0	Singlet	Adipocyte
VAT_2M	6621.3	2683	3.9693709	0	Singlet	Adipocyte
VAT_2M	2552.6	1368	0.0736565	0	Singlet	Adipocyte
VAT_2M	2413.1	1323	7.9157984	7	Singlet	Endothelial_cells
VAT_2M	1830.2	1177	0.4794339	3	Singlet	FAPs
VAT_2M	2698.3	1688	1.114222	9	Singlet	Mesothelial
VAT_2M	1479.4	814	0.0405508	0	Singlet	Adipocyte

VAT_2M	1925.5	1226	0.0156493	0	Singlet	Adipocyte
VAT_2M	1070.4	662	13.005068	6	Singlet	Neurons
VAT_2M	1271.7	719	9.8665117	5	Singlet	Immune_cells
VAT_2M	3282.6	1540	11.554182	0	Singlet	Adipocyte
VAT_2M	3909	1840	0.0368501	0	Singlet	Adipocyte
VAT_2M	1766.2	1013	0.0444926	0	Singlet	Adipocyte
VAT_2M	1199.7	803	7.1309894	2	Singlet	FAPs
VAT_2M	3535.4	1914	4.4579478	3	Singlet	FAPs
VAT_2M	1108.8	798	0	3	Singlet	FAPs
VAT_2M	1187.6	941	3.1083596	11	Singlet	Smooth_muscle_cells
VAT_2M	1165.3	827	1.0009159	0	Singlet	Adipocyte
VAT_2M	1729.6	895	14.481283	5	Singlet	Immune_cells
VAT_2M	1030.8	841	0.085137	3	Singlet	FAPs
VAT_2M	2793.6	1335	9.6646682	7	Singlet	Endothelial_cells
VAT_2M	1433.2	917	0.0598164	0	Singlet	Adipocyte
VAT_2M	2938.9	1565	0	0	Singlet	Adipocyte
VAT_2M	1575	1121	3.2160659	0	Singlet	Adipocyte
VAT_2M	1300.3	732	9.8759661	0	Singlet	Adipocyte
VAT_2M	5282.4	2291	0.0390431	0	Singlet	Adipocyte
VAT_2M	2032.1	1267	5.2602884	3	Singlet	FAPs
VAT_2M	1160.2	1027	0.0675447	5	Singlet	Immune_cells
VAT_2M	2707.1	1455	3.0495629	0	Singlet	Adipocyte
VAT_2M	1858.8	1042	5.8823445	0	Singlet	Adipocyte
VAT_2M	1965.6	1107	2.0052768	0	Singlet	Adipocyte
VAT_2M	1408.9	901	1.0704004	5	Singlet	Immune_cells
VAT_2M	3022.9	1475	10.982641	0	Singlet	Adipocyte
VAT_2M	8814.6	3264	9.4157793	0	Singlet	Adipocyte
VAT_2M	1206.5	1053	0.630882	6	Singlet	Neurons
VAT_2M	3342.3	1576	0.6215198	0	Singlet	Adipocyte
VAT_2M	1411	897	0.4319176	0	Singlet	Adipocyte
VAT_2M	9524.1	3114	12.449835	0	Singlet	Adipocyte
VAT_2M	1848.8	1091	0	0	Singlet	Adipocyte
VAT_2M	1988.3	1109	0.6361372	0	Singlet	Adipocyte
VAT_2M	1154.6	733	12.033116	0	Singlet	Adipocyte
VAT_2M	1957	1231	0.8472967	0	Singlet	Adipocyte
VAT_2M	3624.3	1930	0.4772288	0	Singlet	Adipocyte
VAT_2M	5743.7	2817	6.5519467	5	Singlet	Immune_cells
VAT_2M	3200.8	1591	0.0171469	0	Singlet	Adipocyte
VAT_2M	3497.3	1795	0.0604695	0	Singlet	Adipocyte
VAT_2M	1358.1	1296	0.2726968	6	Singlet	Neurons
VAT_2M	2170.1	1287	0	0	Singlet	Adipocyte
VAT_2M	1110	913	6.2357303	8	Singlet	Mesothelial
VAT_2M	1217.1	776	7.4977155	2	Singlet	FAPs
VAT_2M	1873.6	1281	0.1268064	3	Singlet	FAPs
VAT_2M	2123.9	1196	0.4228909	0	Singlet	Adipocyte
VAT_2M	3216	1824	0.3017499	0	Singlet	Adipocyte
VAT_2M	1376.4	888	13.492901	5	Singlet	Immune_cells
VAT_2M	1932.4	1204	2.7079555	0	Singlet	Adipocyte
VAT_2M	1695.4	1123	1.7383942	3	Singlet	FAPs
VAT_2M	1599.7	972	0.9201696	0	Singlet	Adipocyte
VAT_2M	2583.1	1451	0.1585682	0	Singlet	Adipocyte

VAT_2M	4665	2086	7.3381746	0	Singlet	Adipocyte
VAT_2M	1368.9	1046	10.513677	6	Singlet	Neurons
VAT_2M	2600.3	1190	5.9595368	5	Singlet	Immune_cells
VAT_2M	4666.6	2026	13.924942	0	Singlet	Adipocyte
VAT_2M	1653.8	928	7.1373225	0	Singlet	Adipocyte
VAT_2M	1632.3	989	0.1540838	0	Singlet	Adipocyte
VAT_2M	1473.5	971	1.402732	6	Singlet	Neurons
VAT_2M	1057.3	780	4.6480711	2	Singlet	FAPs
VAT_2M	1113.2	672	12.339359	0	Singlet	Adipocyte
VAT_2M	3637.4	2108	7.0903378	2	Singlet	FAPs
VAT_2M	10146	3578	9.4953554	0	Singlet	Adipocyte
VAT_2M	2347.1	1190	14.341903	0	Singlet	Adipocyte
VAT_2M	1093.8	673	4.7520818	0	Singlet	Adipocyte
VAT_2M	1872.9	1116	6.2943872	3	Singlet	FAPs
VAT_2M	2045.8	1146	5.9801542	0	Singlet	Adipocyte
VAT_2M	1986.8	1160	0.541674	0	Singlet	Adipocyte
VAT_2M	1707.6	885	12.593447	2	Singlet	FAPs
VAT_2M	1194.5	844	0.0427598	3	Singlet	FAPs
VAT_2M	2261.6	1274	0	0	Singlet	Adipocyte
VAT_2M	1295.1	967	0.7771674	3	Singlet	FAPs
VAT_2M	1576.9	1081	1.1266257	3	Singlet	FAPs
VAT_2M	1382.2	910	14.778308	5	Singlet	Immune_cells
VAT_2M	2950.2	1575	0.2594032	0	Singlet	Adipocyte
VAT_2M	1889.9	1176	2.1780833	0	Singlet	Adipocyte
VAT_2M	3732.8	1726	1.8203611	0	Singlet	Adipocyte
VAT_2M	1413.2	969	2.5471262	3	Singlet	FAPs
VAT_2M	6296.9	2531	0.5710495	0	Singlet	Adipocyte
VAT_2M	2176.3	1469	5.7931353	2	Singlet	FAPs
VAT_2M	3834.2	1690	5.6650885	0	Singlet	Adipocyte
VAT_2M	1066.5	794	6.1595498	6	Singlet	Neurons
VAT_2M	1762.1	1015	2.7697063	0	Singlet	Adipocyte
VAT_2M	2093.5	1247	4.2945382	3	Singlet	FAPs
VAT_2M	1287.2	882	5.0519427	3	Singlet	FAPs
VAT_2M	1138.9	859	0.1237322	0	Singlet	Adipocyte
VAT_2M	2740.4	1380	6.8887531	3	Singlet	FAPs
VAT_2M	2318.8	1394	4.4785427	0	Singlet	Adipocyte
VAT_2M	1153.3	1001	8.0146155	6	Singlet	Neurons
VAT_2M	1659.8	1092	0.6648872	0	Singlet	Adipocyte
VAT_2M	1189.8	1034	0.0862603	6	Singlet	Neurons
VAT_2M	4188.3	2021	0.9854657	0	Singlet	Adipocyte
VAT_2M	4734.9	2191	4.6288385	0	Singlet	Adipocyte
VAT_2M	3084.3	1563	0.110409	0	Singlet	Adipocyte
VAT_2M	2053.9	1332	0.3044562	7	Singlet	Endothelial_cells
VAT_2M	2177.4	1431	6.9395424	0	Singlet	Adipocyte
VAT_2M	3088.5	1543	11.260499	0	Singlet	Adipocyte
VAT_2M	1713.8	1447	1.7605373	6	Singlet	Neurons
VAT_2M	2385.1	1385	2.4720153	0	Singlet	Adipocyte
VAT_2M	1893.5	1131	2.8190106	0	Singlet	Adipocyte
VAT_2M	3877.8	1746	10.655118	0	Singlet	Adipocyte
VAT_2M	1631	914	7.0712368	0	Singlet	Adipocyte
VAT_2M	3063.1	1629	0.7260299	0	Singlet	Adipocyte

VAT_2M	2344.4	1368	10.846454	2	Singlet	FAPs
VAT_2M	2991.2	1558	3.8180837	0	Singlet	Adipocyte
VAT_2M	1479.9	956	0	0	Singlet	Adipocyte
VAT_2M	1855.1	1130	1.6269482	0	Singlet	Adipocyte
VAT_2M	1191.6	750	4.9241092	3	Singlet	FAPs
VAT_2M	4809.3	2131	8.3748963	0	Singlet	Adipocyte
VAT_2M	1682	890	11.459042	2	Singlet	FAPs
VAT_2M	2050.7	1162	8.7161021	2	Singlet	FAPs
VAT_2M	1832.9	1014	6.2137131	0	Singlet	Adipocyte
VAT_2M	3961.6	1991	2.5643304	0	Singlet	Adipocyte
VAT_2M	1758.1	966	10.785594	0	Singlet	Adipocyte
VAT_2M	1185.8	734	0.0061614	0	Singlet	Adipocyte
VAT_2M	4776.7	2168	4.2349858	0	Singlet	Adipocyte
VAT_2M	1852.4	1082	13.580714	9	Singlet	Mesothelial
VAT_2M	3795.7	1804	8.0275376	0	Singlet	Adipocyte
VAT_2M	3680.9	1838	1.6989982	0	Singlet	Adipocyte
VAT_2M	4148.3	2021	6.9803603	0	Singlet	Adipocyte
VAT_2M	5044.8	2309	0.8132572	0	Singlet	Adipocyte
VAT_2M	1325.9	793	6.0268775	3	Singlet	FAPs
VAT_2M	3575	1726	8.7366445	0	Singlet	Adipocyte
VAT_2M	1042.4	865	0.7535325	5	Singlet	Immune_cells
VAT_2M	1228.3	788	0.5195251	0	Singlet	Adipocyte
VAT_2M	1832.2	1007	10.817208	7	Singlet	Endothelial_cells
VAT_2M	1136.1	789	0.2320138	5	Singlet	Immune_cells
VAT_2M	1618.3	877	1.1331865	0	Singlet	Adipocyte
VAT_2M	3672.2	1930	0.403226	0	Singlet	Adipocyte
VAT_2M	2055.1	1242	0	0	Singlet	Adipocyte
VAT_2M	4174.5	1987	0.3375027	0	Singlet	Adipocyte
VAT_2M	1431.4	911	4.9182312	0	Singlet	Adipocyte
VAT_2M	1247.5	763	14.892901	0	Singlet	Adipocyte
VAT_2M	5868.2	2516	6.6872189	0	Singlet	Adipocyte
VAT_2M	1170.6	823	6.7756014	2	Singlet	FAPs
VAT_2M	2846.4	1599	1.400535	0	Singlet	Adipocyte
VAT_2M	1920	1097	4.8541098	0	Singlet	Adipocyte
VAT_2M	1202.4	815	0.1027116	0	Singlet	Adipocyte
VAT_2M	1006.6	788	0.0330024	6	Singlet	Neurons
VAT_2M	4206.2	1781	0.7840281	0	Singlet	Adipocyte
VAT_2M	2892.2	1549	0.1867882	0	Singlet	Adipocyte
VAT_2M	1886.8	1101	2.5533696	0	Singlet	Adipocyte
VAT_2M	1190.8	812	4.219721	0	Singlet	Adipocyte
VAT_2M	1247.3	980	7.5151265	3	Singlet	FAPs
VAT_2M	1751	1078	0.047077	0	Singlet	Adipocyte
VAT_2M	1811.8	839	13.030556	0	Singlet	Adipocyte
VAT_2M	1559	913	9.3927566	0	Singlet	Adipocyte
VAT_2M	1292.6	972	0.0574937	3	Singlet	FAPs
VAT_2M	2014.3	1064	10.804211	0	Singlet	Adipocyte
VAT_2M	1626.8	937	13.983375	6	Singlet	Neurons
VAT_2M	2677.2	1441	0	0	Singlet	Adipocyte
VAT_2M	3536.9	1703	0.0140091	0	Singlet	Adipocyte
VAT_2M	2611.5	1501	0.4872927	0	Singlet	Adipocyte
VAT_2M	3523.4	1844	0	0	Singlet	Adipocyte

VAT_2M	2988.8	1531	0.3399406	0	Singlet	Adipocyte
VAT_2M	1136.5	756	0.4518915	2	Singlet	FAPs
VAT_2M	2431.9	1377	9.2913138	0	Singlet	Adipocyte
VAT_2M	1875.1	1132	13.510891	5	Singlet	Immune_cells
VAT_2M	2606.9	1443	0.0241605	0	Singlet	Adipocyte
VAT_2M	1028.5	814	4.7481908	5	Singlet	Immune_cells
VAT_2M	3833.2	1809	9.4196534	5	Singlet	Immune_cells
VAT_2M	1094.8	750	0.1703712	0	Singlet	Adipocyte
VAT_2M	2338.7	1207	13.791706	0	Singlet	Adipocyte
VAT_2M	1884.7	1044	1.8568539	0	Singlet	Adipocyte
VAT_2M	1233.5	777	11.997037	6	Singlet	Neurons
VAT_2M	3052.4	1667	0.080917	0	Singlet	Adipocyte
VAT_2M	1603.6	1064	1.981026	0	Singlet	Adipocyte
VAT_2M	1398	951	0	6	Singlet	Neurons
VAT_2M	3534.3	1752	0.5618813	0	Singlet	Adipocyte
VAT_2M	2442.9	1196	10.505391	0	Singlet	Adipocyte
VAT_2M	1106.4	713	0	0	Singlet	Adipocyte
VAT_2M	1675.3	1093	4.6991359	6	Singlet	Neurons
VAT_2M	4974.5	1999	0.0130881	0	Singlet	Adipocyte
VAT_2M	1718.8	1077	0	3	Singlet	FAPs
VAT_2M	10475	3219	14.997764	0	Singlet	Adipocyte
VAT_2M	2645.3	1447	0.0412387	0	Singlet	Adipocyte
VAT_2M	2834.7	1375	11.688866	0	Singlet	Adipocyte
VAT_2M	1592.9	1053	1.9393571	7	Singlet	Endothelial_cells
VAT_2M	5827.8	2641	5.704524	0	Singlet	Adipocyte
VAT_2M	1343.6	862	8.2555917	0	Singlet	Adipocyte
VAT_2M	2350.6	1315	0.1394045	10	Singlet	Immune_cells
VAT_2M	2142.3	1196	8.8932227	3	Singlet	FAPs
VAT_2M	1787.5	1073	0.1560444	0	Singlet	Adipocyte
VAT_2M	2432.9	1368	0	0	Singlet	Adipocyte
VAT_2M	8005.7	2818	0.1869213	0	Singlet	Adipocyte
VAT_2M	2322.6	1337	0.9198855	0	Singlet	Adipocyte
VAT_2M	4712.9	1823	14.062623	0	Singlet	Adipocyte
VAT_2M	1805.1	1601	3.1787303	6	Singlet	Neurons
VAT_2M	1429.5	973	1.979293	5	Singlet	Immune_cells
VAT_2M	1644.8	977	0.164705	0	Singlet	Adipocyte
VAT_2M	3463.6	1561	0.4575502	0	Singlet	Adipocyte
VAT_2M	3444.7	1807	0.1586018	0	Singlet	Adipocyte
VAT_2M	2284.5	1380	0.4465137	3	Singlet	FAPs
VAT_2M	4343.3	2117	0.0798112	0	Singlet	Adipocyte
VAT_2M	1277.1	707	8.1351861	9	Singlet	Mesothelial
VAT_2M	1120.7	1027	0.4007599	8	Singlet	Mesothelial
VAT_2M	1570.1	884	13.976807	3	Singlet	FAPs
VAT_2M	1449.5	1033	5.4181798	6	Singlet	Neurons
VAT_2M	2764.7	1434	0	0	Singlet	Adipocyte
VAT_2M	1756.2	1076	0.8273238	6	Singlet	Neurons
VAT_2M	1450.3	906	10.601178	0	Singlet	Adipocyte
VAT_2M	1528.6	827	10.803971	0	Singlet	Adipocyte
VAT_2M	3076.6	1505	0.0829975	0	Singlet	Adipocyte
VAT_2M	4929.6	2328	1.7264441	0	Singlet	Adipocyte
VAT_2M	6764.1	2655	9.9813213	0	Singlet	Adipocyte

VAT_2M	2708.4	1409	14.008561	0	Singlet	Adipocyte
VAT_2M	1905	1036	0.5458321	0	Singlet	Adipocyte
VAT_2M	7129.1	2920	0.3793655	0	Singlet	Adipocyte
VAT_2M	2083.2	1184	0	0	Singlet	Adipocyte
VAT_2M	1357.9	1257	0.1036061	8	Singlet	Mesothelial
VAT_2M	6646.3	2460	0	0	Singlet	Adipocyte
VAT_2M	2613.7	1440	0.5716676	0	Singlet	Adipocyte
VAT_2M	1545.8	890	0	0	Singlet	Adipocyte
VAT_2M	3855.5	1911	0.5881145	0	Singlet	Adipocyte
VAT_2M	1953.8	1039	9.3570352	0	Singlet	Adipocyte
VAT_2M	3849.8	1974	3.4771199	0	Singlet	Adipocyte
VAT_2M	1289.7	977	2.78442	0	Singlet	Adipocyte
VAT_2M	3310.3	1658	0.0159601	0	Singlet	Adipocyte
VAT_2M	1707.3	980	4.6895635	0	Singlet	Adipocyte
VAT_2M	1276.8	1026	10.018722	5	Singlet	Immune_cells
VAT_2M	1258	783	0.7807821	0	Singlet	Adipocyte
VAT_2M	7147.3	2811	5.0488593	0	Singlet	Adipocyte
VAT_2M	4378.9	1840	14.568317	0	Singlet	Adipocyte
VAT_2M	4678	2107	0	0	Singlet	Adipocyte
VAT_2M	4674	2437	9.0296097	5	Singlet	Immune_cells
VAT_2M	1491	902	0.9769583	5	Singlet	Immune_cells
VAT_2M	2546	1580	7.3041228	2	Singlet	FAPs
VAT_2M	1078	787	0.1853439	0	Singlet	Adipocyte
VAT_2M	1004.6	706	1.9135424	0	Singlet	Adipocyte
VAT_2M	1155.8	680	11.263082	2	Singlet	FAPs
VAT_2M	1994.2	1220	0.4688296	0	Singlet	Adipocyte
VAT_2M	2133	1242	1.5090687	3	Singlet	FAPs
VAT_2M	2020.2	1113	0.4692315	0	Singlet	Adipocyte
VAT_2M	1093.5	712	14.750975	5	Singlet	Immune_cells
VAT_2M	1210.7	755	13.277891	0	Singlet	Adipocyte
VAT_2M	1278.6	876	2.0221975	2	Singlet	FAPs
VAT_2M	5000	2137	14.733893	0	Singlet	Adipocyte
VAT_2M	3947.1	1732	13.731249	0	Singlet	Adipocyte
VAT_2M	1372.7	788	12.36052	3	Singlet	FAPs
VAT_2M	3707.7	1664	13.319423	0	Singlet	Adipocyte
VAT_2M	2611	1386	1.9116081	0	Singlet	Adipocyte
VAT_2M	1238.4	721	7.1243175	5	Singlet	Immune_cells
VAT_2M	1141.5	674	14.157243	0	Singlet	Adipocyte
VAT_2M	1777.9	1235	2.8994144	2	Singlet	FAPs
VAT_2M	1037.6	617	12.352603	0	Singlet	Adipocyte
VAT_2M	1916.7	961	0	0	Singlet	Adipocyte
VAT_2M	3419.5	1677	0.048172	0	Singlet	Adipocyte
VAT_2M	4025.4	1927	0.1035854	0	Singlet	Adipocyte
VAT_2M	1499.4	763	7.3187213	0	Singlet	Adipocyte
VAT_2M	2753.5	1386	13.744191	0	Singlet	Adipocyte
VAT_2M	1563.2	847	11.750746	9	Singlet	Mesothelial
VAT_2M	1501	1004	1.3845888	3	Singlet	FAPs
VAT_2M	1315	733	5.8245058	3	Singlet	FAPs
VAT_2M	1542.5	968	0.2288363	0	Singlet	Adipocyte
VAT_2M	1140.5	745	0.1227072	3	Singlet	FAPs
VAT_2M	1882.1	1171	0.2349704	0	Singlet	Adipocyte

VAT_2M	5096	2212	3.2995074	0	Singlet	Adipocyte
VAT_2M	4092	2004	3.1956864	0	Singlet	Adipocyte
VAT_2M	2478.1	1403	0.4031941	0	Singlet	Adipocyte
VAT_2M	1494.4	949	0	0	Singlet	Adipocyte
VAT_2M	5018.2	2236	0.0130048	0	Singlet	Adipocyte
VAT_2M	2090.6	1144	0.9513819	0	Singlet	Adipocyte
VAT_2M	2509	1392	0.4139768	0	Singlet	Adipocyte
VAT_2M	4769.2	2118	0.006569	0	Singlet	Adipocyte
VAT_2M	1817.7	863	7.2778843	5	Singlet	Immune_cells
VAT_2M	3206.5	1742	0.634363	0	Singlet	Adipocyte
VAT_2M	3033.4	1540	0	0	Singlet	Adipocyte
VAT_2M	3012.2	1494	0.0187984	0	Singlet	Adipocyte
VAT_2M	2761.4	1482	0.011942	0	Singlet	Adipocyte
VAT_2M	1503.7	888	4.9183546	0	Singlet	Adipocyte
VAT_2M	2475.9	1446	1.9802402	0	Singlet	Adipocyte
VAT_2M	2919.7	1403	11.485916	0	Singlet	Adipocyte
VAT_2M	1372.7	987	0	0	Singlet	Adipocyte
VAT_2M	2576.3	1138	2.8548138	0	Singlet	Adipocyte
VAT_2M	2453.3	1347	14.107102	0	Singlet	Adipocyte
VAT_2M	1586.1	1053	2.5998172	6	Singlet	Neurons
VAT_2M	1587.4	1079	4.3189659	0	Singlet	Adipocyte
VAT_2M	5727.2	2325	0	0	Singlet	Adipocyte
VAT_2M	2283.4	1203	13.607739	5	Singlet	Immune_cells
VAT_2M	3886.4	1769	5.9571142	0	Singlet	Adipocyte
VAT_2M	1031	767	2.8609849	0	Singlet	Adipocyte
VAT_2M	3410.2	1712	0.5737033	0	Singlet	Adipocyte
VAT_2M	2108.7	933	10.94725	0	Singlet	Adipocyte
VAT_2M	2150.9	1231	0.5348571	0	Singlet	Adipocyte
VAT_2M	1200	721	13.492546	3	Singlet	FAPs
VAT_2M	1387	866	8.4218767	6	Singlet	Neurons
VAT_2M	2272.5	1229	1.1549554	0	Singlet	Adipocyte
VAT_2M	3270.7	1689	0.0641003	0	Singlet	Adipocyte
VAT_2M	3419.7	1620	3.8852944	0	Singlet	Adipocyte
VAT_2M	1378.4	1051	0	6	Singlet	Neurons
VAT_2M	3240.3	1602	14.386125	0	Singlet	Adipocyte
VAT_2M	3075.5	1802	8.7376133	2	Singlet	FAPs
VAT_2M	3992.3	1734	0.0106511	0	Singlet	Adipocyte
VAT_2M	8089.4	2980	0	0	Singlet	Adipocyte
VAT_2M	1169.9	755	0.2991759	0	Singlet	Adipocyte
VAT_2M	2416.8	1235	13.332278	0	Singlet	Adipocyte
VAT_2M	1345.4	875	6.1483603	5	Singlet	Immune_cells
VAT_2M	2404.5	1278	0.114348	0	Singlet	Adipocyte
VAT_2M	1901.9	1248	2.9074018	7	Singlet	Endothelial_cells
VAT_2M	1187.6	816	2.2836536	7	Singlet	Endothelial_cells
VAT_2M	1344.2	631	10.868542	3	Singlet	FAPs
VAT_2M	3895.9	1821	3.1149186	0	Singlet	Adipocyte
VAT_2M	3017	1411	10.181073	0	Singlet	Adipocyte
VAT_2M	1127.3	605	10.85992	3	Singlet	FAPs
VAT_2M	1645.1	1036	6.3786199	2	Singlet	FAPs
VAT_2M	3830.6	1599	10.019715	0	Singlet	Adipocyte
VAT_2M	1418.7	833	11.126195	2	Singlet	FAPs

VAT_2M	2135.7	1280	0.3641547	0	Singlet	Adipocyte
VAT_2M	2556.5	1325	3.7112313	5	Singlet	Immune_cells
VAT_2M	2729.6	1501	11.473552	2	Singlet	FAPs
VAT_2M	3114.7	1624	2.5812141	0	Singlet	Adipocyte
VAT_2M	4026.4	1818	0	0	Singlet	Adipocyte
VAT_2M	4575.7	2107	2.6225491	0	Singlet	Adipocyte
VAT_2M	1106.2	801	5.055863	2	Singlet	FAPs
VAT_2M	4519	2131	7.8647237	2	Singlet	FAPs
VAT_2M	2014.2	1156	5.1738426	5	Singlet	Immune_cells
VAT_2M	2563.5	1448	0.2198773	0	Singlet	Adipocyte
VAT_2M	4768.3	2215	2.0989918	0	Singlet	Adipocyte
VAT_2M	1952.1	934	11.009531	0	Singlet	Adipocyte
VAT_2M	3158.4	1773	0.4082102	3	Singlet	FAPs
VAT_2M	2198.5	1371	2.2152938	5	Singlet	Immune_cells
VAT_2M	1330.4	963	2.7795681	2	Singlet	FAPs
VAT_2M	2899.6	1588	1.5137402	0	Singlet	Adipocyte
VAT_2M	1030.8	753	8.2226576	2	Singlet	FAPs
VAT_2M	3356.2	1777	2.066542	0	Singlet	Adipocyte
VAT_2M	1499	884	2.1190642	5	Singlet	Immune_cells
VAT_2M	1702.2	907	7.5072466	5	Singlet	Immune_cells
VAT_2M	3505.9	1630	12.04354	0	Singlet	Adipocyte
VAT_2M	1970.8	1111	0.0240028	0	Singlet	Adipocyte
VAT_2M	2280.5	1193	5.6917328	0	Singlet	Adipocyte
VAT_2M	2518.2	1401	10.512103	0	Singlet	Adipocyte
VAT_2M	3002.9	1413	7.2879761	0	Singlet	Adipocyte
VAT_2M	2083.5	1150	5.6829645	0	Singlet	Adipocyte
VAT_2M	1009.5	757	4.4783197	3	Singlet	FAPs
VAT_2M	1630	1111	3.0635346	5	Singlet	Immune_cells
VAT_2M	1869.7	1168	8.9098411	0	Singlet	Adipocyte
VAT_2M	1230.2	731	14.659403	7	Singlet	Endothelial_cells
VAT_2M	1049.5	757	0.209537	6	Singlet	Neurons
VAT_2M	3214.4	1503	8.6317005	0	Singlet	Adipocyte
VAT_2M	1238.9	989	5.087621	6	Singlet	Neurons
VAT_2M	2934	1485	5.7055183	0	Singlet	Adipocyte
VAT_2M	4819.4	2085	0.9499714	0	Singlet	Adipocyte
VAT_2M	3096.3	1642	1.8391024	0	Singlet	Adipocyte
VAT_2M	3771.1	1683	4.0725247	0	Singlet	Adipocyte
VAT_2M	1423.9	1098	3.5668915	5	Singlet	Immune_cells
VAT_2M	1454.3	1033	0.2166953	3	Singlet	FAPs
VAT_2M	5146.2	2321	6.4915464	0	Singlet	Adipocyte
VAT_2M	6155.6	2529	5.5896603	0	Singlet	Adipocyte
VAT_2M	1699.7	1379	6.361999	6	Singlet	Neurons
VAT_2M	1307.4	937	2.0616771	0	Singlet	Adipocyte
VAT_2M	1303.8	852	0	0	Singlet	Adipocyte
VAT_2M	1167.5	831	6.3071291	0	Singlet	Adipocyte
VAT_2M	1891.9	1048	4.8462044	0	Singlet	Adipocyte
VAT_2M	4533.7	2091	0.5388553	0	Singlet	Adipocyte
VAT_2M	2610.6	1407	1.7655352	0	Singlet	Adipocyte
VAT_2M	7931.7	2949	0.2437701	0	Singlet	Adipocyte
VAT_2M	3268.7	1582	12.52571	0	Singlet	Adipocyte
VAT_2M	1072.3	842	9.2978514	6	Singlet	Neurons

VAT_2M	3515.6	1879	0.3038926	0	Singlet	Adipocyte
VAT_2M	2676.3	1458	1.7420142	0	Singlet	Adipocyte
VAT_2M	1081.2	763	11.327486	11	Singlet	Smooth_muscle_cells
VAT_2M	5644.8	2413	0.1068779	0	Singlet	Adipocyte
VAT_2M	1315.5	811	3.6080127	0	Singlet	Adipocyte
VAT_2M	4866.7	2229	0.2745983	0	Singlet	Adipocyte
VAT_2M	1971.9	1172	0	7	Singlet	Endothelial_cells
VAT_2M	1625.4	1132	10.595932	0	Singlet	Adipocyte
VAT_2M	1694.6	1024	0.100843	0	Singlet	Adipocyte
VAT_2M	3117.1	1542	0	0	Singlet	Adipocyte
VAT_2M	2070.7	1125	0.0695904	0	Singlet	Adipocyte
VAT_2M	1048.9	687	2.353729	5	Singlet	Immune_cells
VAT_2M	2267.2	1352	3.5180874	0	Singlet	Adipocyte
VAT_2M	1300.8	869	7.006222	5	Singlet	Immune_cells
VAT_2M	1444.1	991	0.6823811	0	Singlet	Adipocyte
VAT_2M	1968.8	1144	14.795559	0	Singlet	Adipocyte
VAT_2M	2136.5	1154	0.5868293	0	Singlet	Adipocyte
VAT_2M	4017.9	1931	0	0	Singlet	Adipocyte
VAT_2M	1479.2	893	1.1232776	0	Singlet	Adipocyte
VAT_2M	2183.6	1252	0.7469778	0	Singlet	Adipocyte
VAT_2M	3416.8	2005	0.0475444	3	Singlet	FAPs
VAT_2M	3851.8	1877	10.043979	2	Singlet	FAPs
VAT_2M	2909.7	1414	0.1372257	0	Singlet	Adipocyte
VAT_2M	1638.8	942	5.8917956	3	Singlet	FAPs
VAT_2M	2420.8	1369	11.892271	0	Singlet	Adipocyte
VAT_2M	2755.4	1391	13.862161	0	Singlet	Adipocyte
VAT_2M	1021.5	642	11.931417	0	Singlet	Adipocyte
VAT_2M	1404.1	1169	1.9916719	3	Singlet	FAPs
VAT_2M	3421	1788	0	0	Singlet	Adipocyte
VAT_2M	3524.7	1810	4.660284	0	Singlet	Adipocyte
VAT_2M	1938.7	1165	0.1383433	0	Singlet	Adipocyte
VAT_2M	2337.7	1387	4.9696799	0	Singlet	Adipocyte
VAT_2M	3606.5	1814	0.190814	0	Singlet	Adipocyte
VAT_2M	1236.9	752	13.886305	0	Singlet	Adipocyte
VAT_2M	1994.9	1423	0	0	Singlet	Adipocyte
VAT_2M	1267	849	13.384664	3	Singlet	FAPs
VAT_2M	1551.9	1081	4.6422894	5	Singlet	Immune_cells
VAT_2M	2775.9	1357	0.033453	0	Singlet	Adipocyte
VAT_2M	1482.3	911	0.0770412	0	Singlet	Adipocyte
VAT_2M	3904.1	1846	3.2902391	0	Singlet	Adipocyte
VAT_2M	1732.2	1001	6.1463076	0	Singlet	Adipocyte
VAT_2M	4287.6	1980	5.7167938	0	Singlet	Adipocyte
VAT_2M	1126.4	713	3.529169	0	Singlet	Adipocyte
VAT_2M	1289.8	972	0.0251038	0	Singlet	Adipocyte
VAT_2M	1371.4	1000	10.259198	3	Singlet	FAPs
VAT_2M	5870	2510	0.0021229	0	Singlet	Adipocyte
VAT_2M	1499.8	973	10.213865	0	Singlet	Adipocyte
VAT_2M	2308.1	1392	2.2737102	7	Singlet	Endothelial_cells
VAT_2M	2597.7	1459	1.6835822	0	Singlet	Adipocyte
VAT_2M	1296.3	894	8.447886	2	Singlet	FAPs
VAT_2M	3555.8	1608	8.686775	0	Singlet	Adipocyte

VAT_2M	2772.3	1510	13.496813	2	Singlet	FAPs
VAT_2M	4203.5	1987	8.5769753	0	Singlet	Adipocyte
VAT_2M	2958.8	1543	3.3089296	5	Singlet	Immune_cells
VAT_2M	1261.7	814	3.564482	0	Singlet	Adipocyte
VAT_2M	1331	949	1.1464814	2	Singlet	FAPs
VAT_2M	1119.7	785	4.2314651	0	Singlet	Adipocyte
VAT_2M	2127.3	1208	3.0335963	0	Singlet	Adipocyte
VAT_2M	1161.7	710	14.606884	2	Singlet	FAPs
VAT_2M	1148.6	895	1.7064736	5	Singlet	Immune_cells
VAT_2M	1346.9	902	2.4361017	3	Singlet	FAPs
VAT_2M	1359	1183	4.1948886	6	Singlet	Neurons
VAT_2M	4254.8	1981	0.0108102	0	Singlet	Adipocyte
VAT_2M	1416.1	1130	11.759854	6	Singlet	Neurons
VAT_2M	2354.3	1201	5.9153743	0	Singlet	Adipocyte
VAT_2M	6739	2657	0	0	Singlet	Adipocyte
VAT_2M	1502.7	1080	4.4929714	3	Singlet	FAPs
VAT_2M	1805.2	1078	3.9858338	0	Singlet	Adipocyte
VAT_2M	1119.7	618	12.623779	7	Singlet	Endothelial_cells
VAT_2M	1623.8	977	3.1479318	0	Singlet	Adipocyte
VAT_2M	4468.7	2436	11.066734	2	Singlet	FAPs
VAT_2M	4762.6	2165	5.6386147	0	Singlet	Adipocyte
VAT_2M	1170.5	743	8.9291044	0	Singlet	Adipocyte
VAT_2M	3693.3	1878	6.5025786	0	Singlet	Adipocyte
VAT_2M	1410.3	945	0.2298397	3	Singlet	FAPs
VAT_2M	2202.8	1120	9.0929149	0	Singlet	Adipocyte
VAT_2M	1999.1	1267	5.2126231	0	Singlet	Adipocyte
VAT_2M	2018.1	1191	0.4578307	0	Singlet	Adipocyte
VAT_2M	2396.6	1184	7.6974313	0	Singlet	Adipocyte
VAT_2M	1933.9	1060	10.04843	0	Singlet	Adipocyte
VAT_2M	1870	1088	8.9408669	0	Singlet	Adipocyte
VAT_2M	2702.5	1406	8.746129	0	Singlet	Adipocyte
VAT_2M	1688.9	940	5.9453819	3	Singlet	FAPs
VAT_2M	2771.1	1447	7.485156	0	Singlet	Adipocyte
VAT_2M	2520.3	1363	2.8771293	0	Singlet	Adipocyte
VAT_2M	4165.6	1929	3.5764905	0	Singlet	Adipocyte
VAT_2M	1084.5	757	2.1305839	3	Singlet	FAPs
VAT_2M	2261.3	1257	8.9909567	0	Singlet	Adipocyte
VAT_2M	1184.8	925	0.0838723	5	Singlet	Immune_cells
VAT_2M	1052.6	793	12.816625	4	Singlet	Immune_cells
VAT_2M	2943.7	1637	3.6012031	0	Singlet	Adipocyte
VAT_2M	3192.7	1689	1.484591	0	Singlet	Adipocyte
VAT_2M	1557.3	1005	0.2942712	0	Singlet	Adipocyte
VAT_2M	2107.7	1098	9.4111594	0	Singlet	Adipocyte
VAT_2M	3396.3	1672	1.1831003	0	Singlet	Adipocyte
VAT_2M	2682.7	1446	2.2510267	0	Singlet	Adipocyte
VAT_2M	1198.3	864	0.1325166	5	Singlet	Immune_cells
VAT_2M	1624	1069	1.3387361	3	Singlet	FAPs
VAT_2M	6041	2512	2.1923578	0	Singlet	Adipocyte
VAT_2M	1337.4	771	0.4134568	0	Singlet	Adipocyte
VAT_2M	3245.9	1524	6.9073657	0	Singlet	Adipocyte
VAT_2M	2516.4	1259	0.0447554	0	Singlet	Adipocyte

VAT_2M	1590.8	962	9.2008889	0	Singlet	Adipocyte
VAT_2M	2776.5	1564	4.9877259	0	Singlet	Adipocyte
VAT_2M	1053.2	808	1.5076572	0	Singlet	Adipocyte
VAT_2M	2400.6	1269	0.0473061	0	Singlet	Adipocyte
VAT_2M	1584.2	1101	1.6538904	3	Singlet	FAPs
VAT_2M	1512.8	833	12.221985	5	Singlet	Immune_cells
VAT_2M	1783.6	1059	0.0029168	0	Singlet	Adipocyte
VAT_2M	2472.9	1393	1.7562711	0	Singlet	Adipocyte
VAT_2M	1158.9	763	0.9589604	3	Singlet	FAPs
VAT_2M	1113.4	869	0.573054	0	Singlet	Adipocyte
VAT_2M	6593.5	2577	0.0308988	0	Singlet	Adipocyte
VAT_2M	1508.7	1120	10.462226	0	Singlet	Adipocyte
VAT_2M	4151.9	1826	3.6473858	0	Singlet	Adipocyte
VAT_2M	1809.7	1055	8.6195542	5	Singlet	Immune_cells
VAT_2M	1562.3	989	0	0	Singlet	Adipocyte
VAT_2M	3647.4	1742	10.72755	0	Singlet	Adipocyte
VAT_2M	1081.4	766	2.8726584	2	Singlet	FAPs
VAT_2M	2192.3	1517	5.2677592	5	Singlet	Immune_cells
VAT_2M	1155.7	818	0.4056199	3	Singlet	FAPs
VAT_2M	3380.8	1728	12.494574	0	Singlet	Adipocyte
VAT_2M	1162.5	791	0.4375571	0	Singlet	Adipocyte
VAT_2M	1213.3	971	0.1657109	5	Singlet	Immune_cells
VAT_2M	4254.8	2117	0.0096467	0	Singlet	Adipocyte
VAT_2M	1656.2	1114	12.253756	0	Singlet	Adipocyte
VAT_2M	2685.4	1354	3.103299	0	Singlet	Adipocyte
VAT_2M	1842.5	1219	4.3667887	3	Singlet	FAPs
VAT_2M	1226.5	865	1.7066572	3	Singlet	FAPs
VAT_2M	2460	1305	0.0269225	0	Singlet	Adipocyte
VAT_2M	1084.6	816	0.0704333	0	Singlet	Adipocyte
VAT_2M	1663.7	952	3.2491869	0	Singlet	Adipocyte
VAT_2M	4641.1	2133	8.9668378	0	Singlet	Adipocyte
VAT_2M	7920.8	2977	9.3848898	0	Singlet	Adipocyte
VAT_2M	3776.4	1932	0.0119352	0	Singlet	Adipocyte
VAT_2M	1223.1	842	0	0	Singlet	Adipocyte
VAT_2M	2762.1	1710	8.4182861	2	Singlet	FAPs
VAT_2M	1715.2	986	8.3392976	0	Singlet	Adipocyte
VAT_2M	1501.1	880	10.375143	0	Singlet	Adipocyte
VAT_2M	1717.1	1176	0.448202	3	Singlet	FAPs
VAT_2M	2209.2	1329	9.3715502	0	Singlet	Adipocyte
VAT_2M	2034.1	1033	11.445561	11	Singlet	Smooth_muscle_cells
VAT_2M	1580.3	907	0.1674245	0	Singlet	Adipocyte
VAT_2M	3726	1826	0.7579311	0	Singlet	Adipocyte
VAT_2M	1476.4	973	8.7488686	2	Singlet	FAPs
VAT_2M	1973.6	1194	0.0676463	0	Singlet	Adipocyte
VAT_2M	2367.8	1439	8.583781	0	Singlet	Adipocyte
VAT_2M	2116.5	1178	0	0	Singlet	Adipocyte
VAT_2M	1221.5	714	13.104046	0	Singlet	Adipocyte
VAT_2M	1178.9	931	8.2675288	6	Singlet	Neurons
VAT_2M	3922.4	2047	0.0448807	3	Singlet	FAPs
VAT_2M	1056.1	731	0.7137371	0	Singlet	Adipocyte
VAT_2M	2359.7	1370	2.4762061	0	Singlet	Adipocyte

VAT_2M	1766.9	1001	0.9477829	0 Singlet	Adipocyte
VAT_2M	5687.9	2422	0.6223191	0 Singlet	Adipocyte
VAT_2M	2326.1	1245	2.2380143	0 Singlet	Adipocyte
VAT_2M	9109.7	3218	6.8620929	0 Singlet	Adipocyte
VAT_2M	1322.4	895	7.2263501	2 Singlet	FAPs
VAT_2M	6401.5	2411	13.411741	0 Singlet	Adipocyte
VAT_2M	7856.1	2716	12.719293	2 Singlet	FAPs
VAT_2M	1001.9	653	13.993543	6 Singlet	Neurons
VAT_2M	5369.9	2160	6.4393084	0 Singlet	Adipocyte
VAT_2M	2144.5	1176	7.7837538	3 Singlet	FAPs
VAT_2M	2659.2	1453	5.2111622	0 Singlet	Adipocyte
VAT_2M	2977.7	1503	9.8012646	0 Singlet	Adipocyte
VAT_2M	1585.5	940	4.6079965	0 Singlet	Adipocyte
VAT_2M	1116.1	714	13.014102	0 Singlet	Adipocyte
VAT_2M	1177.9	855	5.3158541	2 Singlet	FAPs
VAT_2M	3111.8	2025	3.4764823	3 Singlet	FAPs
VAT_2M	1114.1	668	14.760074	2 Singlet	FAPs
VAT_2M	2830.8	1422	0	0 Singlet	Adipocyte
VAT_2M	2122.7	1214	2.4093828	0 Singlet	Adipocyte
VAT_2M	1097.1	785	0.0501551	0 Singlet	Adipocyte
VAT_2M	2920.9	1572	2.0225171	0 Singlet	Adipocyte
VAT_2M	2877.1	1493	2.8057237	0 Singlet	Adipocyte
VAT_2M	1859.4	1232	0.1561334	3 Singlet	FAPs
VAT_2M	1523.3	956	3.736016	0 Singlet	Adipocyte
VAT_2M	5814.7	2295	11.507438	0 Singlet	Adipocyte
VAT_2M	2214.4	1111	14.687472	0 Singlet	Adipocyte
VAT_2M	1251.4	1010	0.0421272	6 Singlet	Neurons
VAT_2M	1664.6	981	0.7571127	0 Singlet	Adipocyte
VAT_2M	1808.4	1049	2.4771232	0 Singlet	Adipocyte
VAT_2M	1560.4	936	1.095684	0 Singlet	Adipocyte
VAT_2M	1716.9	1052	0.2175077	0 Singlet	Adipocyte
VAT_2M	1387.2	789	0.1394406	0 Singlet	Adipocyte
VAT_2M	3155.1	1437	10.911559	0 Singlet	Adipocyte
VAT_2M	3448.8	1719	0.7200487	0 Singlet	Adipocyte
VAT_2M	1408.5	981	0.1223105	0 Singlet	Adipocyte
VAT_2M	1141.2	765	0	3 Singlet	FAPs
VAT_2M	1675.2	1051	8.5187034	5 Singlet	Immune_cells
VAT_2M	1966	1214	3.1957765	0 Singlet	Adipocyte
VAT_2M	2653.6	1349	11.996292	0 Singlet	Adipocyte
VAT_2M	1260.2	584	13.482214	0 Singlet	Adipocyte
VAT_2M	1527.1	956	0.0576094	0 Singlet	Adipocyte
VAT_2M	5582	2377	12.347261	0 Singlet	Adipocyte
VAT_2M	1569.2	1031	0	0 Singlet	Adipocyte
VAT_2M	3334.7	1718	0.6955993	0 Singlet	Adipocyte
VAT_2M	2108.4	1233	5.9045685	0 Singlet	Adipocyte
VAT_2M	2111.3	1221	0.0447486	0 Singlet	Adipocyte
VAT_2M	1719.5	929	1.9016499	0 Singlet	Adipocyte
VAT_2M	1310.2	870	2.9168707	2 Singlet	FAPs
VAT_2M	1004.9	558	11.052892	5 Singlet	Immune_cells
VAT_2M	3777.7	1970	0	0 Singlet	Adipocyte
VAT_2M	1095.5	876	9.9387139	8 Singlet	Mesothelial

VAT_2M	1450.9	980	0	0	Singlet	Adipocyte
VAT_2M	2134.6	1329	4.5634981	7	Singlet	Endothelial_cells
VAT_2M	1818.6	1088	0.1827781	0	Singlet	Adipocyte
VAT_2M	1025.2	666	11.189152	6	Singlet	Neurons
VAT_2M	1302.9	620	11.715957	9	Singlet	Mesothelial
VAT_2M	1893.2	1122	5.7850589	0	Singlet	Adipocyte
VAT_2M	3776	1802	8.5000569	0	Singlet	Adipocyte
VAT_2M	2854.2	1447	0.0203046	0	Singlet	Adipocyte
VAT_2M	2472.2	1373	0.9096225	0	Singlet	Adipocyte
VAT_2M	1722.3	1105	5.0059284	2	Singlet	FAPs
VAT_2M	1174.9	786	2.9713141	0	Singlet	Adipocyte
VAT_2M	2743.9	1391	6.9848548	0	Singlet	Adipocyte
VAT_2M	1660.5	1179	0.0724014	0	Singlet	Adipocyte
VAT_2M	1264.5	652	10.536396	0	Singlet	Adipocyte
VAT_2M	1686.8	1012	0.0899249	0	Singlet	Adipocyte
VAT_2M	1443	791	10.977248	0	Singlet	Adipocyte
VAT_2M	1472.1	1104	0	0	Singlet	Adipocyte
VAT_2M	2367.5	1388	0.3672221	5	Singlet	Immune_cells
VAT_2M	1085.3	806	0.477546	0	Singlet	Adipocyte
VAT_2M	2891.7	1831	2.6978285	2	Singlet	FAPs
VAT_2M	2660.9	1357	0.0185174	0	Singlet	Adipocyte
VAT_2M	1366.4	839	0.7316102	0	Singlet	Adipocyte
VAT_2M	1802.7	1073	12.073273	0	Singlet	Adipocyte
VAT_2M	2835.3	1357	0.1944111	0	Singlet	Adipocyte
VAT_2M	1264.1	1056	0	3	Singlet	FAPs
VAT_2M	1092.7	664	1.8760744	0	Singlet	Adipocyte
VAT_2M	2078.9	1141	0.9795697	0	Singlet	Adipocyte
VAT_2M	2337.2	1365	2.5514039	0	Singlet	Adipocyte
VAT_2M	1178.7	831	8.4161441	0	Singlet	Adipocyte
VAT_2M	1663.6	1055	0.7884056	7	Singlet	Endothelial_cells
VAT_2M	1374.2	736	10.793432	5	Singlet	Immune_cells
VAT_2M	1403.6	867	10.470601	7	Singlet	Endothelial_cells
VAT_2M	3180.9	853	14.543282	2	Singlet	FAPs
VAT_2M	1000.5	732	0.3881978	0	Singlet	Adipocyte
VAT_2M	2716.7	1353	3.2482753	0	Singlet	Adipocyte
VAT_2M	1833.7	1195	9.9060793	0	Singlet	Adipocyte
VAT_2M	4613.6	1953	4.9461129	0	Singlet	Adipocyte
VAT_2M	1705.6	1097	0	3	Singlet	FAPs
VAT_2M	2324.2	1199	7.49429	0	Singlet	Adipocyte
VAT_2M	4457.3	1968	0	0	Singlet	Adipocyte
VAT_2M	3164	1787	0.0172213	0	Singlet	Adipocyte
VAT_2M	1998.9	1217	0.1901567	0	Singlet	Adipocyte
VAT_2M	1780.7	1068	2.0494179	0	Singlet	Adipocyte
VAT_2M	2446.2	1352	4.4411114	0	Singlet	Adipocyte
VAT_2M	4278.2	1788	12.183054	0	Singlet	Adipocyte
VAT_2M	1415.8	852	5.3046354	0	Singlet	Adipocyte
VAT_2M	2992.7	1570	3.0159037	0	Singlet	Adipocyte
VAT_2M	1618.1	961	5.5893466	9	Singlet	Mesothelial
VAT_2M	1589	1005	0.883802	3	Singlet	FAPs
VAT_2M	1268.2	710	4.726937	0	Singlet	Adipocyte
VAT_2M	1076.6	677	6.9433613	2	Singlet	FAPs

VAT_2M	2112.9	1340	0.3308823	0	Singlet	Adipocyte
VAT_2M	4096.9	1948	2.8822498	0	Singlet	Adipocyte
VAT_2M	1443.9	937	11.994517	0	Singlet	Adipocyte
VAT_2M	1068	1004	0.8914799	6	Singlet	Neurons
VAT_2M	2775.4	1311	0.056439	0	Singlet	Adipocyte
VAT_2M	1190.4	690	10.991558	5	Singlet	Immune_cells
VAT_2M	1402.7	969	6.4849038	5	Singlet	Immune_cells
VAT_2M	1551	1053	0.060556	0	Singlet	Adipocyte
VAT_2M	1428.3	1169	5.9739835	6	Singlet	Neurons
VAT_2M	1112.1	912	5.878458	9	Singlet	Mesothelial
VAT_2M	2142	1317	0.278567	0	Singlet	Adipocyte
VAT_2M	1712.6	990	1.505506	7	Singlet	Endothelial_cells
VAT_2M	1097	1014	1.6432153	6	Singlet	Neurons
VAT_2M	1666.7	1127	0.0330587	3	Singlet	FAPs
VAT_2M	6823.8	2647	0.1931984	0	Singlet	Adipocyte
VAT_2M	6003	2464	12.886239	0	Singlet	Adipocyte
VAT_2M	1603.1	955	5.2583333	0	Singlet	Adipocyte
VAT_2M	1712.6	1018	0	0	Singlet	Adipocyte
VAT_2M	3302.9	1628	13.009297	0	Singlet	Adipocyte
VAT_2M	2232.7	1336	9.6642168	3	Singlet	FAPs
VAT_2M	1058.7	786	4.2825839	3	Singlet	FAPs
VAT_2M	3319.7	1706	0.5237087	0	Singlet	Adipocyte
VAT_2M	3058.1	1552	9.4567218	0	Singlet	Adipocyte
VAT_2M	2919.3	1530	7.8318429	0	Singlet	Adipocyte
VAT_2M	2013	1199	0.8633998	0	Singlet	Adipocyte
VAT_2M	1985.9	1170	0	0	Singlet	Adipocyte
VAT_2M	2893.7	1569	0.4737225	0	Singlet	Adipocyte
VAT_2M	2537.2	1341	5.5636707	0	Singlet	Adipocyte
VAT_2M	1528.2	973	1.041616	5	Singlet	Immune_cells
VAT_2M	1425.2	953	0.0653932	0	Singlet	Adipocyte
VAT_2M	4207.7	1945	5.8916137	0	Singlet	Adipocyte
VAT_2M	5630.9	2114	12.776666	0	Singlet	Adipocyte
VAT_2M	5226.3	2221	2.8536023	0	Singlet	Adipocyte
VAT_2M	1302.9	1001	0	0	Singlet	Adipocyte
VAT_2M	5018	2343	4.1503516	0	Singlet	Adipocyte
VAT_2M	4484.8	2050	0	0	Singlet	Adipocyte
VAT_2M	2392.2	1309	0.1943039	0	Singlet	Adipocyte
VAT_2M	1359.2	988	5.2240247	0	Singlet	Adipocyte
VAT_2M	1016.3	809	6.894106	11	Singlet	Smooth_muscle_cells
VAT_2M	2430.5	1416	4.1852261	0	Singlet	Adipocyte
VAT_2M	1523.2	1001	1.8756578	0	Singlet	Adipocyte
VAT_2M	3111.4	1643	0.0823267	0	Singlet	Adipocyte
VAT_2M	3570.3	1660	0.0290999	0	Singlet	Adipocyte
VAT_2M	2697.7	1367	7.5494053	0	Singlet	Adipocyte
VAT_2M	2092	1048	6.9214176	3	Singlet	FAPs
VAT_2M	1838.4	1218	0	3	Singlet	FAPs
VAT_2M	1105.8	664	3.5944107	0	Singlet	Adipocyte
VAT_2M	2024.2	890	14.638683	5	Singlet	Immune_cells
VAT_2M	1021.8	682	7.6297061	0	Singlet	Adipocyte
VAT_2M	1303.7	985	0	0	Singlet	Adipocyte
VAT_2M	1792.8	1047	3.9340867	0	Singlet	Adipocyte

VAT_2M	3020.5	1481	0	0	Singlet	Adipocyte
VAT_2M	3884.5	1860	5.2491448	0	Singlet	Adipocyte
VAT_2M	2476.3	1506	0.5570911	0	Singlet	Adipocyte
VAT_2M	3521.3	1817	0	0	Singlet	Adipocyte
VAT_2M	1148.2	678	0.2271027	0	Singlet	Adipocyte
VAT_2M	3290.4	1615	3.4963285	0	Singlet	Adipocyte
VAT_2M	4676.6	1979	0	0	Singlet	Adipocyte
VAT_2M	2503.8	1398	0.2998032	0	Singlet	Adipocyte
VAT_2M	2508.1	1336	7.6626035	0	Singlet	Adipocyte
VAT_2M	1756.9	1004	4.5118467	0	Singlet	Adipocyte
VAT_2M	1296.6	914	0.0012378	3	Singlet	FAPs
VAT_2M	1480.9	805	8.0661632	0	Singlet	Adipocyte
VAT_2M	1210.3	790	1.3195169	0	Singlet	Adipocyte
VAT_2M	4188.7	2043	6.5478317	0	Singlet	Adipocyte
VAT_2M	5425.9	2296	3.1314916	0	Singlet	Adipocyte
VAT_2M	7946.9	2839	4.6086008	0	Singlet	Adipocyte
VAT_2M	3602.7	1739	0	0	Singlet	Adipocyte
VAT_2M	4294.7	2208	0.0095581	0	Singlet	Adipocyte
VAT_2M	1410.8	827	11.262243	5	Singlet	Immune_cells
VAT_2M	3987.7	1890	0.010926	0	Singlet	Adipocyte
VAT_2M	1486.1	1000	0.7623851	3	Singlet	FAPs
VAT_2M	2508.4	1350	0.157988	0	Singlet	Adipocyte
VAT_2M	1469.6	1068	1.23071	5	Singlet	Immune_cells
VAT_2M	2384.3	1364	0.0331755	0	Singlet	Adipocyte
VAT_2M	1987.4	1109	0.3380478	0	Singlet	Adipocyte
VAT_2M	1443	1000	0.4447097	6	Singlet	Neurons
VAT_2M	3045.9	1482	12.069071	0	Singlet	Adipocyte
VAT_2M	2655.3	1691	10.64252	5	Singlet	Immune_cells
VAT_2M	2370	1364	7.1267202	0	Singlet	Adipocyte
VAT_2M	2551.3	1501	6.3160696	2	Singlet	FAPs
VAT_2M	1598.8	921	0.9093816	0	Singlet	Adipocyte
VAT_2M	1350.6	967	10.395695	4	Singlet	Immune_cells
VAT_2M	3587.3	1909	0.5545413	0	Singlet	Adipocyte
VAT_2M	3604.1	1806	7.5910972	0	Singlet	Adipocyte
VAT_2M	1763.8	1234	1.2785314	0	Singlet	Adipocyte
VAT_2M	2017.4	1184	2.2419052	3	Singlet	FAPs
VAT_2M	3518.1	1586	14.627101	0	Singlet	Adipocyte
VAT_2M	2758.2	1769	0.4380064	3	Singlet	FAPs
VAT_2M	4387	1984	4.3630638	0	Singlet	Adipocyte
VAT_2M	1027.5	730	11.59396	5	Singlet	Immune_cells
VAT_2M	1169.7	992	3.3182187	6	Singlet	Neurons
VAT_2M	1012.1	743	0.0269523	0	Singlet	Adipocyte
VAT_2M	2349.3	1356	1.665447	0	Singlet	Adipocyte
VAT_2M	1329.3	842	13.225629	0	Singlet	Adipocyte
VAT_2M	7784.6	2676	12.163495	0	Singlet	Adipocyte
VAT_2M	1887.4	1106	0.558646	0	Singlet	Adipocyte
VAT_2M	2176	1305	3.936834	0	Singlet	Adipocyte
VAT_2M	1098.7	711	0.0376584	0	Singlet	Adipocyte
VAT_2M	1099.8	880	0	6	Singlet	Neurons
VAT_2M	1910.9	1194	12.353959	2	Singlet	FAPs
VAT_2M	3000.7	1470	10.878778	0	Singlet	Adipocyte

VAT_2M	1906.4	1351	0.0555668	0	Singlet	Adipocyte
VAT_2M	2685.6	1498	0	0	Singlet	Adipocyte
VAT_2M	3280.7	1509	0	0	Singlet	Adipocyte
VAT_2M	1906.3	1198	0.0429996	5	Singlet	Immune_cells
VAT_2M	1337.7	762	4.7014717	0	Singlet	Adipocyte
VAT_2M	1211.3	825	3.371178	0	Singlet	Adipocyte
VAT_2M	2254	1170	5.3855186	0	Singlet	Adipocyte
VAT_2M	3714.5	1624	12.69014	0	Singlet	Adipocyte
VAT_2M	4810	2282	2.2160543	0	Singlet	Adipocyte
VAT_2M	3928	2167	5.368619	2	Singlet	FAPs
VAT_2M	2339.3	1389	0.0467254	0	Singlet	Adipocyte
VAT_2M	1131.4	806	0	6	Singlet	Neurons
VAT_2M	2088.8	1219	0.2631273	0	Singlet	Adipocyte
VAT_2M	1101.1	798	2.4611112	0	Singlet	Adipocyte
VAT_2M	4290.1	1903	1.9881768	0	Singlet	Adipocyte
VAT_2M	1135.6	820	0.7907002	6	Singlet	Neurons
VAT_2M	9443.6	3197	2.1695902	0	Singlet	Adipocyte
VAT_2M	1063.3	868	3.2914558	8	Singlet	Mesothelial
VAT_2M	2090.4	1175	0.2078455	0	Singlet	Adipocyte
VAT_2M	3357.2	1779	14.485902	5	Singlet	Immune_cells
VAT_2M	2085.7	1239	0	0	Singlet	Adipocyte
VAT_2M	1692.4	1375	3.149569	11	Singlet	Smooth_muscle_cells
VAT_2M	1083.1	692	0.6765769	0	Singlet	Adipocyte
VAT_2M	3763.8	1805	2.0140861	0	Singlet	Adipocyte
VAT_2M	6573.9	2536	8.7453677	0	Singlet	Adipocyte
VAT_2M	1899.2	958	11.472758	0	Singlet	Adipocyte
VAT_2M	1098.7	714	3.2799377	3	Singlet	FAPs
VAT_2M	1074.7	826	0.2907863	0	Singlet	Adipocyte
VAT_2M	2545	1438	0	0	Singlet	Adipocyte
VAT_2M	1614.1	946	13.940266	0	Singlet	Adipocyte
VAT_2M	1855.3	1138	0	0	Singlet	Adipocyte
VAT_2M	1075.2	671	0	0	Singlet	Adipocyte
VAT_2M	4175.6	1884	0.1427863	0	Singlet	Adipocyte
VAT_2M	2397.7	1150	12.132485	0	Singlet	Adipocyte
VAT_2M	1401.5	870	4.1365475	0	Singlet	Adipocyte
VAT_2M	3981.9	1858	0	0	Singlet	Adipocyte
VAT_2M	2607.3	1497	0	0	Singlet	Adipocyte
VAT_2M	4035.5	1837	2.5499367	0	Singlet	Adipocyte
VAT_2M	4100.8	1868	9.4037239	0	Singlet	Adipocyte
VAT_2M	1074.4	722	0.0837836	0	Singlet	Adipocyte
VAT_2M	1278.7	1078	0.0180014	6	Singlet	Neurons
VAT_2M	5915.3	2504	0.1161467	0	Singlet	Adipocyte
VAT_2M	4816.4	2180	0	0	Singlet	Adipocyte
VAT_2M	8824.2	3117	0.034598	0	Singlet	Adipocyte
VAT_2M	3763.6	1881	3.0099813	5	Singlet	Immune_cells
VAT_2M	6832.2	2721	0.0089213	0	Singlet	Adipocyte
VAT_2M	1642.3	983	0.4485693	0	Singlet	Adipocyte
VAT_2M	2490.9	1499	0.1934169	6	Singlet	Neurons
VAT_2M	2083.7	1278	1.0722166	2	Singlet	FAPs
VAT_2M	13093	3997	0.182489	0	Singlet	Adipocyte
VAT_2M	1834.3	1180	0.5781294	0	Singlet	Adipocyte

VAT_2M	2531.8	1545	0.2439475	0	Singlet	Adipocyte
VAT_2M	7295.2	2967	0.0153248	0	Singlet	Adipocyte
VAT_2M	1716.9	1379	0.3998532	8	Singlet	Mesothelial
VAT_2M	1033.5	769	0.9907662	5	Singlet	Immune_cells
VAT_2M	10083	3329	0.2000904	0	Singlet	Adipocyte
VAT_2M	2060	1301	3.2805906	2	Singlet	FAPs
VAT_2M	2354.5	1347	1.1974343	0	Singlet	Adipocyte
VAT_2M	1293.8	953	3.9716851	5	Singlet	Immune_cells
VAT_2M	4401.9	2090	0	0	Singlet	Adipocyte
VAT_2M	10541	3477	0.1138328	0	Singlet	Adipocyte
VAT_2M	1234.3	945	1.2616031	2	Singlet	FAPs
VAT_2M	1506.5	1043	2.3353797	5	Singlet	Immune_cells
VAT_2M	1782.5	857	8.0353335	8	Singlet	Mesothelial
VAT_2M	4237.2	2040	0.5369236	0	Singlet	Adipocyte
VAT_2M	3571.5	1971	0.1859752	0	Singlet	Adipocyte
VAT_2M	1202.2	943	0.9022466	11	Singlet	Smooth_muscle_cells
VAT_2M	1254	811	1.5018216	7	Singlet	Endothelial_cells
VAT_2M	4417.1	2027	0.0225697	0	Singlet	Adipocyte
VAT_2M	1877.3	1259	0.9215408	0	Singlet	Adipocyte
VAT_2M	1575.6	1130	5.117424	1	Singlet	Immune_cells
VAT_2M	2692.3	1450	0.3339249	0	Singlet	Adipocyte
VAT_2M	1458.9	1086	2.8558469	2	Singlet	FAPs
VAT_2M	2509.8	1559	0.0942947	3	Singlet	FAPs
VAT_2M	1143	823	2.1706339	10	Singlet	Immune_cells
VAT_2M	3013.9	1596	0.07542	0	Singlet	Adipocyte
VAT_2M	6540.4	2694	0.9386199	0	Singlet	Adipocyte
VAT_2M	2141.8	1277	0.2881595	2	Singlet	FAPs
VAT_2M	4516.2	2124	0.0106651	0	Singlet	Adipocyte
VAT_2M	1895.1	1165	1.4270642	0	Singlet	Adipocyte
VAT_2M	6915.5	2888	0.0004486	0	Singlet	Adipocyte
VAT_2M	7261.2	2849	0.0158252	0	Singlet	Adipocyte
VAT_2M	3326.3	1795	0.3398172	0	Singlet	Adipocyte
VAT_2M	2765.4	1572	0	6	Singlet	Neurons
VAT_2M	6026.3	2417	0.0826564	0	Singlet	Adipocyte
VAT_2M	2445.2	1452	3.3299504	2	Singlet	FAPs
VAT_2M	2782.5	1720	0.5709512	2	Singlet	FAPs
VAT_2M	2626.6	1674	0.0607738	7	Singlet	Endothelial_cells
VAT_2M	7347.8	2855	2.3904083	0	Singlet	Adipocyte
VAT_2M	1811.1	1261	1.9755283	3	Singlet	FAPs
VAT_2M	2254.5	1274	0	0	Singlet	Adipocyte
VAT_2M	4962.3	2348	0.0542572	0	Singlet	Adipocyte
VAT_2M	2024.2	1286	1.5629262	3	Singlet	FAPs
VAT_2M	1422.3	1176	0.4595445	5	Singlet	Immune_cells
VAT_2M	1465.2	1079	5.3680536	2	Singlet	FAPs
VAT_2M	2345.9	1237	5.8150914	7	Singlet	Endothelial_cells
VAT_2M	1273.9	1075	1.6901938	9	Singlet	Mesothelial
VAT_2M	1407.2	962	0.0298344	5	Singlet	Immune_cells
VAT_2M	1302.9	999	5.7923545	6	Singlet	Neurons
VAT_2M	1605	987	0	0	Singlet	Adipocyte
VAT_2M	2384.6	1661	0.3603537	2	Singlet	FAPs
VAT_2M	6190.7	2607	0	0	Singlet	Adipocyte

VAT_2M	1862.5	1292	2.4602683	2	Singlet	FAPs
VAT_2M	1897.8	1210	0.3146981	6	Singlet	Neurons
VAT_2M	3959.7	1985	0.0528795	0	Singlet	Adipocyte
VAT_2M	2140.3	1198	14.348714	7	Singlet	Endothelial_cells
VAT_2M	2537.5	1477	0.5545238	3	Singlet	FAPs
VAT_2M	7346.4	2952	0.05899	0	Singlet	Adipocyte
VAT_2M	2123.2	1277	5.8211879	7	Singlet	Endothelial_cells
VAT_2M	2992.6	1638	0.3142254	0	Singlet	Adipocyte
VAT_2M	6134.6	2494	0	0	Singlet	Adipocyte
VAT_2M	7170.3	2842	0.0195213	0	Singlet	Adipocyte
VAT_2M	2659.2	1642	0.8996144	5	Singlet	Immune_cells
VAT_2M	7173.8	2866	0.9559005	0	Singlet	Adipocyte
VAT_2M	2422.7	1330	1.2005372	0	Singlet	Adipocyte
VAT_2M	1810.4	1094	0.5305093	0	Singlet	Adipocyte
VAT_2M	1968.2	1318	2.8269599	5	Singlet	Immune_cells
VAT_2M	1727.3	1155	0.4077651	6	Singlet	Neurons
VAT_2M	5015.9	2222	0.0286617	0	Singlet	Adipocyte
VAT_2M	2510.2	1501	0.0261636	0	Singlet	Adipocyte
VAT_2M	1249.2	927	0.2521623	6	Singlet	Neurons
VAT_2M	2155.2	1416	2.5707658	2	Singlet	FAPs
VAT_2M	1040.6	811	3.6549505	10	Singlet	Immune_cells
VAT_2M	5747.8	2498	0.0950535	0	Singlet	Adipocyte
VAT_2M	4162.9	1959	0.0774729	0	Singlet	Adipocyte
VAT_2M	1185.2	909	0.1581564	5	Singlet	Immune_cells
VAT_2M	1586.5	1160	0.3090602	3	Singlet	FAPs
VAT_2M	11017	3519	0.0117312	0	Singlet	Adipocyte
VAT_2M	2964	1630	1.1106724	6	Singlet	Neurons
VAT_2M	1993.7	1178	0.5892908	7	Singlet	Endothelial_cells
VAT_2M	5110.6	2321	0.1716881	0	Singlet	Adipocyte
VAT_2M	7585.2	2914	0.0152349	0	Singlet	Adipocyte
VAT_2M	1227.8	944	3.3707986	2	Singlet	FAPs
VAT_2M	1552.8	1090	0.343994	0	Singlet	Adipocyte
VAT_2M	1258.9	952	0.4196279	2	Singlet	FAPs
VAT_2M	1115.9	829	0.8942755	3	Singlet	FAPs
VAT_2M	3317.7	1762	0.4661553	0	Singlet	Adipocyte
VAT_2M	1855.4	1324	1.2870358	5	Singlet	Immune_cells
VAT_2M	5045.5	2235	0.1554326	0	Singlet	Adipocyte
VAT_2M	1598.3	1085	1.9103343	3	Singlet	FAPs
VAT_2M	3880.8	2028	0.0120601	0	Singlet	Adipocyte
VAT_2M	1751.8	1330	1.4354618	6	Singlet	Neurons
VAT_2M	3763.8	1907	1.2778624	0	Singlet	Adipocyte
VAT_2M	1877.9	1009	10.876216	0	Singlet	Adipocyte
VAT_2M	3927.4	1943	0.0784477	0	Singlet	Adipocyte
VAT_2M	3147	1693	0.0941019	0	Singlet	Adipocyte
VAT_2M	2492.9	1370	6.3698151	0	Singlet	Adipocyte
VAT_2M	1915.6	1254	0.1506682	0	Singlet	Adipocyte
VAT_2M	3163.2	1880	0.3396129	3	Singlet	FAPs
VAT_2M	3949.6	1935	0.2064882	0	Singlet	Adipocyte
VAT_2M	1775.4	1197	0.212256	0	Singlet	Adipocyte
VAT_2M	1631.5	1001	0.2185264	0	Singlet	Adipocyte
VAT_2M	1426.9	1045	2.8605239	1	Singlet	Immune_cells

VAT_2M	6368.4	2645	0.5284462	0	Singlet	Adipocyte
VAT_2M	2449.2	1384	0.1167506	0	Singlet	Adipocyte
VAT_2M	1123.7	927	0.0289855	6	Singlet	Neurons
VAT_2M	1120.1	891	0.6827035	7	Singlet	Endothelial_cells
VAT_2M	2080.8	1268	0.0842396	0	Singlet	Adipocyte
VAT_2M	6887.7	2735	0.0065843	0	Singlet	Adipocyte
VAT_2M	1574.5	990	0.1082899	0	Singlet	Adipocyte
VAT_2M	4001.6	2032	0.8679161	7	Singlet	Endothelial_cells
VAT_2M	2727.3	1577	0.6452682	3	Singlet	FAPs
VAT_2M	3499.6	1804	0.034977	0	Singlet	Adipocyte
VAT_2M	1961.7	1341	0.9099126	3	Singlet	FAPs
VAT_2M	1704.2	985	3.4847403	9	Singlet	Mesothelial
VAT_2M	3638.4	1995	1.1990671	0	Singlet	Adipocyte
VAT_2M	2693.4	1635	0.6477423	11	Singlet	Smooth_muscle_cells
VAT_2M	1822.5	1148	1.9699622	0	Singlet	Adipocyte
VAT_2M	1760.1	1255	0.6572238	6	Singlet	Neurons
VAT_2M	4480.5	2185	0.0138727	0	Singlet	Adipocyte
VAT_2M	1646	1132	0.7169066	3	Singlet	FAPs
VAT_2M	6429	2697	0.1246889	0	Singlet	Adipocyte
VAT_2M	5768.1	2374	0.0190354	0	Singlet	Adipocyte
VAT_2M	1258	896	2.5696886	9	Singlet	Mesothelial
VAT_2M	1746.5	1328	1.0741234	11	Singlet	Smooth_muscle_cells
VAT_2M	1890.4	1331	0.4100519	7	Singlet	Endothelial_cells
VAT_2M	2287.8	1445	0.3627846	0	Singlet	Adipocyte
VAT_2M	6325.1	2551	0.0382057	0	Singlet	Adipocyte
VAT_2M	1187.9	983	0.9315611	11	Singlet	Smooth_muscle_cells
VAT_2M	4806.7	2239	0	0	Singlet	Adipocyte
VAT_2M	6405.5	2605	0.1132644	0	Singlet	Adipocyte
VAT_2M	1393.2	996	0.6182142	3	Singlet	FAPs
VAT_2M	7092.6	2739	0.0201046	0	Singlet	Adipocyte
VAT_2M	8547.5	3210	0.0028308	0	Singlet	Adipocyte
VAT_2M	4650.5	2195	0.8856621	0	Singlet	Adipocyte
VAT_2M	1882.4	1460	2.3861712	7	Singlet	Endothelial_cells
VAT_2M	4927.7	2206	0.0228028	0	Singlet	Adipocyte
VAT_2M	4555.1	2163	0.3065835	0	Singlet	Adipocyte
VAT_2M	9023.8	3155	0.0751579	0	Singlet	Adipocyte
VAT_2M	4832	2227	0	0	Singlet	Adipocyte
VAT_2M	2049.8	1268	0.2936161	0	Singlet	Adipocyte
VAT_2M	2186.5	1173	0.3246986	0	Singlet	Adipocyte
VAT_2M	2036.7	1310	0	0	Singlet	Adipocyte
VAT_2M	1268.5	1073	1.0103091	9	Singlet	Mesothelial
VAT_2M	7933.6	2871	0	0	Singlet	Adipocyte
VAT_2M	1371.1	963	1.2647553	2	Singlet	FAPs
VAT_2M	6262.2	2683	0.2446178	0	Singlet	Adipocyte
VAT_2M	1126.6	785	3.4416314	8	Singlet	Mesothelial
VAT_2M	1356.6	1054	1.1286657	2	Singlet	FAPs
VAT_2M	4666.6	2197	0.0538206	0	Singlet	Adipocyte
VAT_2M	4275.1	1995	0.2389607	0	Singlet	Adipocyte
VAT_2M	1633.2	1133	6.467356	9	Singlet	Mesothelial
VAT_2M	1274.8	921	0.626813	6	Singlet	Neurons
VAT_2M	1360.6	1046	2.0084492	6	Singlet	Neurons

VAT_2M	2422.6	1407	0.0205018	0	Singlet	Adipocyte
VAT_2M	4628.7	2087	0.1025052	0	Singlet	Adipocyte
VAT_2M	5954.1	2625	0.0584054	0	Singlet	Adipocyte
VAT_2M	3250.7	1843	0.272537	0	Singlet	Adipocyte
VAT_2M	1659.3	1079	1.192673	3	Singlet	FAPs
VAT_2M	1132.2	849	0.936452	6	Singlet	Neurons
VAT_2M	1744.7	1223	0	7	Singlet	Endothelial_cells
VAT_2M	1620.3	1082	3.192722	5	Singlet	Immune_cells
VAT_2M	1239.5	898	1.0226298	3	Singlet	FAPs
VAT_2M	4300.5	1983	0.3937675	0	Singlet	Adipocyte
VAT_2M	2717.8	1699	0.2684196	6	Singlet	Neurons
VAT_2M	1143.7	932	1.2633454	7	Singlet	Endothelial_cells
VAT_2M	1992	1371	1.2967759	2	Singlet	FAPs
VAT_2M	1132	913	0.7793042	7	Singlet	Endothelial_cells
VAT_2M	1751.8	1367	1.0731001	3	Singlet	FAPs
VAT_2M	2588.3	1498	0.8081545	2	Singlet	FAPs
VAT_2M	1651	1348	1.5338147	3	Singlet	FAPs
VAT_2M	5583.2	2416	0.0335264	0	Singlet	Adipocyte
VAT_2M	7629.4	2942	0.0589634	0	Singlet	Adipocyte
VAT_2M	2675.4	1596	0.9426053	3	Singlet	FAPs
VAT_2M	6008.7	2594	0.1620121	0	Singlet	Adipocyte
VAT_2M	6300.7	2494	0.0046394	0	Singlet	Adipocyte
VAT_2M	7229.9	2780	0.0803051	0	Singlet	Adipocyte
VAT_2M	1059.6	847	1.9846258	4	Singlet	Immune_cells
VAT_2M	1085	767	0.4712263	2	Singlet	FAPs
VAT_2M	1363.5	963	0.9284983	0	Singlet	Adipocyte
VAT_2M	1043.2	819	1.5527432	2	Singlet	FAPs
VAT_2M	8670.7	3117	0.3007865	0	Singlet	Adipocyte
VAT_2M	1083.9	856	1.8429915	5	Singlet	Immune_cells
VAT_2M	1563.6	1064	0.4178022	0	Singlet	Adipocyte
VAT_2M	1035.7	680	0.0035598	0	Singlet	Adipocyte
VAT_2M	9265	3061	0.0397202	0	Singlet	Adipocyte
VAT_2M	5714.3	2509	0.0242958	0	Singlet	Adipocyte
VAT_2M	2174.4	1439	0.0466357	0	Singlet	Adipocyte
VAT_2M	1748.2	1104	0.2241888	0	Singlet	Adipocyte
VAT_2M	3085.5	1791	0.3926862	0	Singlet	Adipocyte
VAT_2M	1552.5	1072	1.4726755	2	Singlet	FAPs
VAT_2M	2193.6	843	1.8516311	1	Singlet	Immune_cells
VAT_2M	1669.2	1110	1.3990022	0	Singlet	Adipocyte
VAT_2M	1881.5	1235	1.1294321	2	Singlet	FAPs
VAT_2M	1235.1	929	0.6621751	0	Singlet	Adipocyte
VAT_2M	4705.9	2248	0.1875635	0	Singlet	Adipocyte
VAT_2M	1105.4	939	0.7637551	6	Singlet	Neurons
VAT_2M	1609.2	1189	0.3687796	7	Singlet	Endothelial_cells
VAT_2M	1319.3	968	2.6623341	3	Singlet	FAPs
VAT_2M	1994.2	1370	2.576549	2	Singlet	FAPs
VAT_2M	1586.2	1153	0.2569759	3	Singlet	FAPs
VAT_2M	2319.9	1576	1.0732241	2	Singlet	FAPs
VAT_2M	3307.8	1939	0.4964389	2	Singlet	FAPs
VAT_2M	2102.8	1347	0.4943177	0	Singlet	Adipocyte
VAT_2M	3746.1	1930	0.136054	0	Singlet	Adipocyte

VAT_2M	2176.4	1571	1.3320106	2	Singlet	FAPs
VAT_2M	6936.1	2752	0.0520345	0	Singlet	Adipocyte
VAT_2M	4914.1	2163	0.1618461	0	Singlet	Adipocyte
VAT_2M	7806.8	2882	0.0338417	0	Singlet	Adipocyte
VAT_2M	1447	1040	1.0394599	2	Singlet	FAPs
VAT_2M	2849.1	1601	0.3167327	0	Singlet	Adipocyte
VAT_2M	4216.4	2103	0	0	Singlet	Adipocyte
VAT_2M	1388	1079	3.8969583	6	Singlet	Neurons
VAT_2M	1900.1	1229	1.2704631	0	Singlet	Adipocyte
VAT_2M	1056.8	802	0.1996226	2	Singlet	FAPs
VAT_2M	8547.9	3044	0.0021155	0	Singlet	Adipocyte
VAT_2M	1210.5	963	2.2402895	6	Singlet	Neurons
VAT_2M	1675.1	1085	0.70148	0	Singlet	Adipocyte
VAT_2M	2017.3	1363	0.8920865	7	Singlet	Endothelial_cells
VAT_2M	3092.8	1597	0.1501785	0	Singlet	Adipocyte
VAT_2M	1585.1	879	8.6190362	9	Singlet	Mesothelial
VAT_2M	1955.3	1326	1.5270803	2	Singlet	FAPs
VAT_2M	3794.9	1918	0.0231716	0	Singlet	Adipocyte
VAT_2M	3100.6	1598	0.1092912	0	Singlet	Adipocyte
VAT_2M	5831.6	2528	0.4788594	0	Singlet	Adipocyte
VAT_2M	2678.8	1564	0.4212899	0	Singlet	Adipocyte
VAT_2M	9771.4	3395	1.2840982	0	Singlet	Adipocyte
VAT_2M	3290.6	1949	0.5973275	2	Singlet	FAPs
VAT_2M	3516.3	1936	0.0447445	0	Singlet	Adipocyte
VAT_2M	2114.7	1326	0.0358632	0	Singlet	Adipocyte
VAT_2M	9025.1	3149	0.0014929	0	Singlet	Adipocyte
VAT_2M	1454.1	1148	1.3715399	6	Singlet	Neurons
VAT_2M	1382.3	1043	1.2363695	2	Singlet	FAPs
VAT_2M	12035	3650	0.4399098	0	Singlet	Adipocyte
VAT_2M	2343	1529	0.7439563	5	Singlet	Immune_cells
VAT_2M	1033	835	0.6642096	6	Singlet	Neurons
VAT_2M	4146.5	2345	1.5329043	2	Singlet	FAPs
VAT_2M	1426.7	1050	1.4538533	3	Singlet	FAPs
VAT_2M	1597.7	1172	3.0934136	3	Singlet	FAPs
VAT_2M	2406.5	1626	1.5837346	2	Singlet	FAPs
VAT_2M	6936	2667	0.0065508	0	Singlet	Adipocyte
VAT_2M	3671.3	1920	0.1428341	0	Singlet	Adipocyte
VAT_2M	4868.6	2535	0.0108332	0	Singlet	Adipocyte
VAT_2M	3110	1762	0.0669645	0	Singlet	Adipocyte
VAT_2M	2917.2	1887	1.1101388	2	Singlet	FAPs
VAT_2M	1756.1	1169	0.0712146	0	Singlet	Adipocyte
VAT_2M	1871.1	1187	0.2045653	3	Singlet	FAPs
VAT_2M	2212.4	1299	0.6038218	6	Singlet	Neurons
VAT_2M	1276.5	930	1.2925994	11	Singlet	Smooth_muscle_cells
VAT_2M	1144.3	881	2.1401878	9	Singlet	Mesothelial
VAT_2M	2213	1645	0.5732166	2	Singlet	FAPs
VAT_2M	4945.2	2186	0.024586	0	Singlet	Adipocyte
VAT_2M	1003.4	771	1.8415175	6	Singlet	Neurons
VAT_2M	2555.3	1765	0.845752	2	Singlet	FAPs
VAT_2M	1902	1266	2.8138483	3	Singlet	FAPs
VAT_2M	1419.7	1120	2.6470956	2	Singlet	FAPs

VAT_2M	3263.6	1644	0.0196192	0	Singlet	Adipocyte
VAT_2M	1142	848	1.0420213	3	Singlet	FAPs
VAT_2M	1717.3	1056	0.1238912	0	Singlet	Adipocyte
VAT_2M	2581.9	1445	0.1927825	0	Singlet	Adipocyte
VAT_2M	1630.3	1152	1.1001509	0	Singlet	Adipocyte
VAT_2M	7757.2	2993	0.0337529	0	Singlet	Adipocyte
VAT_2M	1723.3	1173	0.6770696	3	Singlet	FAPs
VAT_2M	4203.7	2027	0.0364142	0	Singlet	Adipocyte
VAT_2M	4556.5	2202	0.3331677	0	Singlet	Adipocyte
VAT_2M	2564	1695	0.384527	2	Singlet	FAPs
VAT_2M	1513.8	1041	1.6628627	5	Singlet	Immune_cells
VAT_2M	4294	1983	0.0132929	0	Singlet	Adipocyte
VAT_2M	1066.8	805	2.6923025	3	Singlet	FAPs
VAT_2M	2925.7	1674	0.0763189	0	Singlet	Adipocyte
VAT_2M	6256.3	2609	0.0225048	0	Singlet	Adipocyte
VAT_2M	2793.5	1501	0.3033249	0	Singlet	Adipocyte
VAT_2M	1720.7	1279	1.8298313	6	Singlet	Neurons
VAT_2M	7567.3	2893	0.2935731	0	Singlet	Adipocyte
VAT_2M	3761.5	1814	0.1223434	0	Singlet	Adipocyte
VAT_2M	2333.4	1511	0.005955	0	Singlet	Adipocyte
VAT_2M	5022.4	2341	0.1106411	0	Singlet	Adipocyte
VAT_2M	2259.4	1482	0.9475785	2	Singlet	FAPs
VAT_2M	3108.2	1791	1.0878589	3	Singlet	FAPs
VAT_2M	5155.9	2281	0	0	Singlet	Adipocyte
VAT_2M	2528.1	1507	1.365285	3	Singlet	FAPs
VAT_2M	4957.5	2331	0.289307	0	Singlet	Adipocyte
VAT_2M	1018.3	858	3.0110183	9	Singlet	Mesothelial
VAT_2M	1136.2	933	4.0385508	6	Singlet	Neurons
VAT_2M	1636.5	1187	2.5290756	2	Singlet	FAPs
VAT_2M	1057.4	841	0.2987415	6	Singlet	Neurons
VAT_2M	5368.7	2578	0.1757279	0	Singlet	Adipocyte
VAT_2M	1297.9	1028	2.6602412	9	Singlet	Mesothelial
VAT_2M	6234.7	2543	0.007547	0	Singlet	Adipocyte
VAT_2M	4195.2	2154	0	0	Singlet	Adipocyte
VAT_2M	9752.4	3164	0.0086383	0	Singlet	Adipocyte
VAT_2M	2925.6	1584	0.4586487	0	Singlet	Adipocyte
VAT_2M	6863.2	2674	0.0542321	0	Singlet	Adipocyte
VAT_2M	9389.6	3282	0.0020314	0	Singlet	Adipocyte
VAT_2M	6707.8	2651	0.1732657	0	Singlet	Adipocyte
VAT_2M	2830.8	1679	1.2345985	2	Singlet	FAPs
VAT_2M	2553.6	1451	1.1526507	5	Singlet	Immune_cells
VAT_2M	5989.6	2527	0.0064752	0	Singlet	Adipocyte
VAT_2M	2394.1	1341	0.1876649	0	Singlet	Adipocyte
VAT_2M	1470.4	1254	0.3606552	6	Singlet	Neurons
VAT_2M	5694.6	2404	0.3114236	0	Singlet	Adipocyte
VAT_2M	2910.9	1607	0.2684218	0	Singlet	Adipocyte
VAT_2M	7565.3	2776	0.6036797	0	Singlet	Adipocyte
VAT_2M	2218	1312	0.5350871	0	Singlet	Adipocyte
VAT_2M	4960.2	2340	0	0	Singlet	Adipocyte
VAT_2M	1628.8	956	2.7353084	5	Singlet	Immune_cells
VAT_2M	2911.1	1884	0.6656733	2	Singlet	FAPs

VAT_2M	5587.1	2441	0	0	Singlet	Adipocyte
VAT_2M	9176.3	3253	0.3110236	0	Singlet	Adipocyte
VAT_2M	2505.1	1559	0.337184	7	Singlet	Endothelial_cells
VAT_2M	2085.1	1236	0.0382917	0	Singlet	Adipocyte
VAT_2M	5985.8	2600	0.0634097	0	Singlet	Adipocyte
VAT_2M	1066.8	891	1.2958816	6	Singlet	Neurons
VAT_2M	1038.4	763	0.6567785	3	Singlet	FAPs
VAT_2M	3258.4	1756	0.0186704	0	Singlet	Adipocyte
VAT_2M	1338.2	972	0.2901119	10	Singlet	Immune_cells
VAT_2M	6552.7	2570	0.4639561	0	Singlet	Adipocyte
VAT_2M	7377.8	2696	0.0662005	0	Singlet	Adipocyte
VAT_2M	1445.5	1020	0.2385059	2	Singlet	FAPs
VAT_2M	5537.2	2415	0.0682943	0	Singlet	Adipocyte
VAT_2M	9378.1	3349	0	0	Singlet	Adipocyte
VAT_2M	1575.6	1208	1.7019102	2	Singlet	FAPs
VAT_2M	1055	885	0.7743791	8	Singlet	Mesothelial
VAT_2M	7401.3	2803	1.4028341	0	Singlet	Adipocyte
VAT_2M	2265.4	1437	0.6721765	3	Singlet	FAPs
VAT_2M	1245.5	854	5.6080435	8	Singlet	Mesothelial
VAT_2M	2612.3	1491	2.2517769	3	Singlet	FAPs
VAT_2M	3547.9	1933	0.0342438	0	Singlet	Adipocyte
VAT_2M	2581.8	1495	0.1437256	0	Singlet	Adipocyte
VAT_2M	3539.7	1906	0.3399546	0	Singlet	Adipocyte
VAT_2M	6138.7	2607	0.0320035	0	Singlet	Adipocyte
VAT_2M	1757.8	1126	0.5848248	0	Singlet	Adipocyte
VAT_2M	4189.6	2049	0.5097473	0	Singlet	Adipocyte
VAT_2M	9446.3	3379	0.1003236	0	Singlet	Adipocyte
VAT_2M	2525	1657	2.3433935	2	Singlet	FAPs
VAT_2M	2414.5	1577	0.4699533	3	Singlet	FAPs
VAT_2M	3873.2	2064	0.5281263	0	Singlet	Adipocyte
VAT_2M	3644.4	2141	0.8402206	2	Singlet	FAPs
VAT_2M	3700.6	1999	0.922859	5	Singlet	Immune_cells
VAT_2M	1934.9	1158	14.965128	3	Singlet	FAPs
VAT_2M	6886	2754	0.0021319	0	Singlet	Adipocyte
VAT_2M	2570.1	1659	0.1592891	2	Singlet	FAPs
VAT_2M	1866.6	1497	0.6686517	11	Singlet	Smooth_muscle_cells
VAT_2M	2241.5	1463	0.9361421	8	Singlet	Mesothelial
VAT_2M	2239.8	1468	1.3373099	0	Singlet	Adipocyte
VAT_2M	2815	1648	0.4177356	0	Singlet	Adipocyte
VAT_2M	3323.4	1739	0.7835111	0	Singlet	Adipocyte
VAT_2M	7784.7	2951	0.020192	0	Singlet	Adipocyte
VAT_2M	3497.2	1791	6.629561	7	Singlet	Endothelial_cells
VAT_2M	1076.8	795	0.8474909	2	Singlet	FAPs
VAT_2M	4382.6	2134	0.0816321	0	Singlet	Adipocyte
VAT_2M	9226.2	3237	0.1103968	0	Singlet	Adipocyte
VAT_2M	3203.7	1621	0.481764	0	Singlet	Adipocyte
VAT_2M	8576	3144	0.252698	0	Singlet	Adipocyte
VAT_2M	3339.5	1832	0.1978091	0	Singlet	Adipocyte
VAT_2M	1122.5	886	3.5319226	9	Singlet	Mesothelial
VAT_2M	1340.7	977	1.3420306	7	Singlet	Endothelial_cells
VAT_2M	1270.3	1034	1.8294616	3	Singlet	FAPs

VAT_2M	4077.1	1942	1.1353009	0	Singlet	Adipocyte
VAT_2M	2559.9	1619	0.1518664	0	Singlet	Adipocyte
VAT_2M	6891	2813	0.0082967	0	Singlet	Adipocyte
VAT_2M	1500.8	1122	0.5808508	6	Singlet	Neurons
VAT_2M	3051	1717	0.1295754	0	Singlet	Adipocyte
VAT_2M	1865.1	1099	0.3046409	0	Singlet	Adipocyte
VAT_2M	12432	3755	0.0672384	0	Singlet	Adipocyte
VAT_2M	1014.8	860	1.2909109	6	Singlet	Neurons
VAT_2M	1114.3	834	0.7185594	11	Singlet	Smooth_muscle_cells
VAT_2M	3790.9	1821	0.1071747	0	Singlet	Adipocyte
VAT_2M	4801.9	2151	0.0215252	0	Singlet	Adipocyte
VAT_2M	2480.9	1474	0.4488133	7	Singlet	Endothelial_cells
VAT_2M	1315.2	1041	2.4624482	3	Singlet	FAPs
VAT_2M	8709.3	3263	0.0580637	0	Singlet	Adipocyte
VAT_2M	7030.8	2864	0.0098202	0	Singlet	Adipocyte
VAT_2M	1160.1	949	1.9467312	5	Singlet	Immune_cells
VAT_2M	1298	1071	0.4279935	3	Singlet	FAPs
VAT_2M	3421.8	1957	0.249952	2	Singlet	FAPs
VAT_2M	8619.4	3273	0.2710895	0	Singlet	Adipocyte
VAT_2M	1435.5	1058	2.1532416	2	Singlet	FAPs
VAT_2M	2384.8	1491	0.3389874	0	Singlet	Adipocyte
VAT_2M	1757.1	1169	0.3771995	0	Singlet	Adipocyte
VAT_2M	1053.7	830	1.0497478	4	Singlet	Immune_cells
VAT_2M	9266.7	3159	0	0	Singlet	Adipocyte
VAT_2M	4382.6	2020	0.0905377	0	Singlet	Adipocyte
VAT_2M	4583.1	2205	0.1563383	0	Singlet	Adipocyte
VAT_2M	11173	3255	0.0283071	0	Singlet	Adipocyte
VAT_2M	3716.2	1854	0.016232	0	Singlet	Adipocyte
VAT_2M	8944.5	3048	0.0213093	0	Singlet	Adipocyte
VAT_2M	3639.2	1866	0.0428382	0	Singlet	Adipocyte
VAT_2M	5513.4	2443	0.0555583	0	Singlet	Adipocyte
VAT_2M	3622	1954	0.9084455	5	Singlet	Immune_cells
VAT_2M	4521.6	2378	0.5037266	3	Singlet	FAPs
VAT_2M	2248.7	1271	0.0576809	0	Singlet	Adipocyte
VAT_2M	1136.2	838	0.696565	3	Singlet	FAPs
VAT_2M	1499	1154	1.6706535	5	Singlet	Immune_cells
VAT_2M	2098.2	1465	2.0528238	0	Singlet	Adipocyte
VAT_2M	2483	1769	1.7311202	2	Singlet	FAPs
VAT_2M	3130	1713	0.5379948	0	Singlet	Adipocyte
VAT_2M	3227.6	1788	5.5405078	0	Singlet	Adipocyte
VAT_2M	9983.6	3418	0	0	Singlet	Adipocyte
VAT_2M	1224.6	896	0.2045344	5	Singlet	Immune_cells
VAT_2M	9395.5	3287	0.0541778	0	Singlet	Adipocyte
VAT_2M	2753.6	1631	0.399987	0	Singlet	Adipocyte
VAT_2M	2568.5	1735	1.1334804	2	Singlet	FAPs
VAT_2M	3464.9	1816	0.1264579	0	Singlet	Adipocyte
VAT_2M	3036.2	1557	0.0764321	0	Singlet	Adipocyte
VAT_2M	1737.3	1232	0.6816786	5	Singlet	Immune_cells
VAT_2M	1191.7	1014	2.2100282	8	Singlet	Mesothelial
VAT_2M	6017.1	2571	0.0150861	0	Singlet	Adipocyte
VAT_2M	2727.6	1592	0.7093953	0	Singlet	Adipocyte

VAT_2M	5227.6	2488	0.029061	0	Singlet	Adipocyte
VAT_2M	4600.8	2182	0.1007469	0	Singlet	Adipocyte
VAT_2M	2027.7	1225	0.6069259	0	Singlet	Adipocyte
VAT_2M	1674.3	1089	0.6385235	7	Singlet	Endothelial_cells
VAT_2M	7914.4	3026	0.017771	0	Singlet	Adipocyte
VAT_2M	6879.8	2712	0.0318622	0	Singlet	Adipocyte
VAT_2M	1435.1	1085	0.5153827	2	Singlet	FAPs
VAT_2M	1953.9	1280	0.0391948	0	Singlet	Adipocyte
VAT_2M	3012.3	1825	0.0679032	6	Singlet	Neurons
VAT_2M	1318.7	1044	2.3932328	6	Singlet	Neurons
VAT_2M	2154.6	1216	0.2864675	0	Singlet	Adipocyte
VAT_2M	1602.1	1046	1.32426	0	Singlet	Adipocyte
VAT_2M	1435.4	1002	0.8012268	9	Singlet	Mesothelial
VAT_2M	10674	3512	0.0004524	0	Singlet	Adipocyte
VAT_2M	1040.4	788	1.6231059	4	Singlet	Immune_cells
VAT_2M	1898.5	1229	1.0420903	7	Singlet	Endothelial_cells
VAT_2M	6357	2634	0.0899034	0	Singlet	Adipocyte
VAT_2M	5237.5	2534	0.0257749	0	Singlet	Adipocyte
VAT_2M	1306.1	964	2.8422113	2	Singlet	FAPs
VAT_2M	3992.7	2074	0.7526861	3	Singlet	FAPs
VAT_2M	3540.1	1796	0.0776014	0	Singlet	Adipocyte
VAT_2M	8900.8	3229	0.0747091	0	Singlet	Adipocyte
VAT_2M	1804.6	1203	0.5886336	3	Singlet	FAPs
VAT_2M	1329	979	0.3584739	2	Singlet	FAPs
VAT_2M	2236.9	1438	0.2683325	2	Singlet	FAPs
VAT_2M	2262.7	1326	0.00985	0	Singlet	Adipocyte
VAT_2M	3243.3	1841	0.4965447	2	Singlet	FAPs
VAT_2M	1467.2	1015	1.1653701	2	Singlet	FAPs
VAT_2M	1607.2	1177	1.5963478	2	Singlet	FAPs
VAT_2M	2262.5	1452	0.4285027	0	Singlet	Adipocyte
VAT_2M	1675.3	1179	0.6698713	0	Singlet	Adipocyte
VAT_2M	1058.3	810	1.0401591	5	Singlet	Immune_cells
VAT_2M	9409	3378	5.0666079	0	Singlet	Adipocyte
VAT_2M	1522.4	1104	3.2644465	5	Singlet	Immune_cells
VAT_2M	1224.1	980	2.3224288	6	Singlet	Neurons
VAT_2M	1397.6	1025	3.0451139	2	Singlet	FAPs
VAT_2M	1466.8	1189	0.6975505	11	Singlet	Smooth_muscle_cells
VAT_2M	3941.4	1871	0.1604747	0	Singlet	Adipocyte
VAT_2M	6229.5	2602	0.0143454	0	Singlet	Adipocyte
VAT_2M	1077.4	856	1.7454481	8	Singlet	Mesothelial
VAT_2M	1994.3	1423	0.2675454	2	Singlet	FAPs
VAT_2M	4414.5	2280	0.0918143	0	Singlet	Adipocyte
VAT_2M	11931	3487	1.1869361	0	Singlet	Adipocyte
VAT_2M	1926.5	1197	0.053199	0	Singlet	Adipocyte
VAT_2M	1218	1042	0.5280725	6	Singlet	Neurons
VAT_2M	7544.5	2856	0.2199475	0	Singlet	Adipocyte
VAT_2M	5174.5	2290	0.0738712	0	Singlet	Adipocyte
VAT_2M	3162.2	1600	0	0	Singlet	Adipocyte
VAT_2M	1238.4	884	0.1638644	6	Singlet	Neurons
VAT_2M	5042.9	2372	0.0387365	0	Singlet	Adipocyte
VAT_2M	1439.9	994	0.3511356	2	Singlet	FAPs

VAT_2M	1047.3	783	3.4268192	1	Singlet	Immune_cells
VAT_2M	2746.7	1562	0.6591788	0	Singlet	Adipocyte
VAT_2M	1000.8	853	1.8620459	6	Singlet	Neurons
VAT_2M	4870.8	2361	0	0	Singlet	Adipocyte
VAT_2M	1821	1139	0.0403528	0	Singlet	Adipocyte
VAT_2M	1052	738	0.2326596	11	Singlet	Smooth_muscle_cells
VAT_2M	2259.9	1615	0.7810523	2	Singlet	FAPs
VAT_2M	1823.6	1224	0.0453467	3	Singlet	FAPs
VAT_2M	3120.6	1923	0.6400572	2	Singlet	FAPs
VAT_2M	2253.9	1438	0.3836045	0	Singlet	Adipocyte
VAT_2M	2147.2	1440	1.8366887	2	Singlet	FAPs
VAT_2M	6260.4	2732	0.0185845	0	Singlet	Adipocyte
VAT_2M	1722	1075	0.666803	3	Singlet	FAPs
VAT_2M	1813.2	1267	1.4721066	5	Singlet	Immune_cells
VAT_2M	1091.2	777	0.6089301	7	Singlet	Endothelial_cells
VAT_2M	2618.4	1544	0.1258445	0	Singlet	Adipocyte
VAT_2M	6772.2	2720	0.1224595	0	Singlet	Adipocyte
VAT_2M	6769.6	2714	0.0853399	0	Singlet	Adipocyte
VAT_2M	3680.7	1858	0.1992056	0	Singlet	Adipocyte
VAT_2M	1618.3	1016	0.3291752	0	Singlet	Adipocyte
VAT_2M	1079	871	1.6669914	4	Singlet	Immune_cells
VAT_2M	7908.1	3004	0.2859123	0	Singlet	Adipocyte
VAT_2M	9704.1	3458	0.0536744	0	Singlet	Adipocyte
VAT_2M	1776.1	1169	1.2043525	3	Singlet	FAPs
VAT_2M	3935.8	2196	0.4411988	0	Singlet	Adipocyte
VAT_2M	7682.3	2914	0.0260109	0	Singlet	Adipocyte
VAT_2M	1210.2	881	0.3162043	5	Singlet	Immune_cells
VAT_2M	1598.6	973	1.2219904	0	Singlet	Adipocyte
VAT_2M	6836.6	2690	0	0	Singlet	Adipocyte
VAT_2M	1435.4	1026	1.1998306	7	Singlet	Endothelial_cells
VAT_2M	6204.6	2594	0.0463705	0	Singlet	Adipocyte
VAT_2M	1409.2	1061	0.8631543	2	Singlet	FAPs
VAT_2M	1209.4	995	0.1305427	6	Singlet	Neurons
VAT_2M	1185.9	832	0.1309721	3	Singlet	FAPs
VAT_2M	1746.8	1217	10.542957	4	Singlet	Immune_cells
VAT_2M	1849.6	1118	0.0160397	0	Singlet	Adipocyte
VAT_2M	4773.7	2346	0.6734604	0	Singlet	Adipocyte
VAT_2M	1203.5	959	1.6315473	8	Singlet	Mesothelial
VAT_2M	3031.2	1550	0.0592646	0	Singlet	Adipocyte
VAT_2M	1876.8	1254	0.2981658	2	Singlet	FAPs
VAT_2M	1386.4	977	1.1058427	2	Singlet	FAPs
VAT_2M	1078.5	753	2.7565987	9	Singlet	Mesothelial
VAT_2M	4145.8	2056	0.1292468	0	Singlet	Adipocyte
VAT_2M	7556.5	2745	0.0227374	0	Singlet	Adipocyte
VAT_2M	1394.1	1071	0.6724704	3	Singlet	FAPs
VAT_2M	1446	983	1.5423526	2	Singlet	FAPs
VAT_2M	1848.2	1190	0.3067451	3	Singlet	FAPs
VAT_2M	1365.5	984	3.359458	3	Singlet	FAPs
VAT_2M	1766.7	1077	0.0461058	0	Singlet	Adipocyte
VAT_2M	4913.2	2248	0.114793	0	Singlet	Adipocyte
VAT_2M	8197.6	2894	0.0193362	0	Singlet	Adipocyte

VAT_2M	1601.2	1148	0.8567368	5	Singlet	Immune_cells
VAT_2M	2885	1530	1.0695969	0	Singlet	Adipocyte
VAT_2M	7252.5	2952	0.0996837	0	Singlet	Adipocyte
VAT_2M	2315.6	1611	0.4802306	2	Singlet	FAPs
VAT_2M	1074.9	822	5.3710284	11	Singlet	Smooth_muscle_cells
VAT_2M	1023.6	823	0.5677742	3	Singlet	FAPs
VAT_2M	13283	3923	0.0216166	0	Singlet	Adipocyte
VAT_2M	7354	2746	0.0073987	0	Singlet	Adipocyte
VAT_2M	1971.8	1293	3.725921	5	Singlet	Immune_cells
VAT_2M	6871.2	2889	0.1851869	0	Singlet	Adipocyte
VAT_2M	9273.2	3089	0.0077111	0	Singlet	Adipocyte
VAT_2M	3037.3	1674	4.4787992	5	Singlet	Immune_cells
VAT_2M	1690.8	1143	0.914814	0	Singlet	Adipocyte
VAT_2M	5943	2514	0.0105492	0	Singlet	Adipocyte
VAT_2M	1562	1120	0.5286176	5	Singlet	Immune_cells
VAT_2M	1125.5	972	0.3536515	6	Singlet	Neurons
VAT_2M	1758.2	1306	1.2052073	7	Singlet	Endothelial_cells
VAT_2M	2864.7	1505	0	0	Singlet	Adipocyte
VAT_2M	6134.1	2604	0.146932	0	Singlet	Adipocyte
VAT_2M	1636	1050	2.7318118	3	Singlet	FAPs
VAT_2M	5105.2	2312	0.1208506	0	Singlet	Adipocyte
VAT_2M	1837	1215	0.7014442	5	Singlet	Immune_cells
VAT_2M	1892.5	1376	1.4115714	6	Singlet	Neurons
VAT_2M	1720.6	1153	4.7736865	9	Singlet	Mesothelial
VAT_2M	2300.5	1529	2.4250933	3	Singlet	FAPs
VAT_2M	3921.1	1987	0.1389176	0	Singlet	Adipocyte
VAT_2M	1378.9	983	2.5228352	3	Singlet	FAPs
VAT_2M	1835.7	1145	1.3319481	0	Singlet	Adipocyte
VAT_2M	2519.1	1444	0.1207072	0	Singlet	Adipocyte
VAT_2M	1660.4	1366	1.0520045	11	Singlet	Smooth_muscle_cells
VAT_2M	3394.5	1792	0	0	Singlet	Adipocyte
VAT_2M	1722.2	1251	0.048571	3	Singlet	FAPs
VAT_2M	7874.9	3059	0.0217743	0	Singlet	Adipocyte
VAT_2M	2675.1	1529	0.6019736	2	Singlet	FAPs
VAT_2M	1464.2	1104	0.5705222	2	Singlet	FAPs
VAT_2M	2783.4	1471	0.1551815	0	Singlet	Adipocyte
VAT_2M	3170.2	1778	0.0207102	0	Singlet	Adipocyte
VAT_2M	1011.5	775	1.603442	3	Singlet	FAPs
VAT_2M	1432.3	1075	2.0255909	11	Singlet	Smooth_muscle_cells
VAT_2M	2476.5	1397	0.1534177	6	Singlet	Neurons
VAT_2M	4447.3	2119	0.0202247	0	Singlet	Adipocyte
VAT_2M	1710.8	1197	2.8392622	5	Singlet	Immune_cells
VAT_2M	6366.8	2623	0.0399167	0	Singlet	Adipocyte
VAT_2M	2908.9	1609	0.5119916	0	Singlet	Adipocyte
VAT_2M	5893.4	2531	0.0970935	0	Singlet	Adipocyte
VAT_2M	1111.9	957	2.7504386	8	Singlet	Mesothelial
VAT_2M	7960.3	2872	0.0275564	0	Singlet	Adipocyte
VAT_2M	1036.5	821	0.979349	4	Singlet	Immune_cells
VAT_2M	1248.2	858	2.5863483	2	Singlet	FAPs
VAT_2M	1693.8	948	2.7325546	0	Singlet	Adipocyte
VAT_2M	5166	2439	0	0	Singlet	Adipocyte

VAT_2M	2052.1	1205	0.283937	0	Singlet	Adipocyte
VAT_2M	10365	3451	0.0074636	0	Singlet	Adipocyte
VAT_2M	2789.3	1537	4.4061513	5	Singlet	Immune_cells
VAT_2M	4337.6	1937	0.5500293	0	Singlet	Adipocyte
VAT_2M	1935.7	1393	1.0689697	2	Singlet	FAPs
VAT_2M	1511.6	1094	0.6844144	6	Singlet	Neurons
VAT_2M	4596.8	2233	0.009643	0	Singlet	Adipocyte
VAT_2M	5134.9	2357	0.1428715	0	Singlet	Adipocyte
VAT_2M	1077.1	778	3.3695152	6	Singlet	Neurons
VAT_2M	3636.4	1909	0.2077363	0	Singlet	Adipocyte
VAT_2M	1526.7	1028	0.0647874	3	Singlet	FAPs
VAT_2M	1740.7	1239	0.0006243	3	Singlet	FAPs
VAT_2M	7093.8	2892	0.0932456	0	Singlet	Adipocyte
VAT_2M	1696.9	1330	0.0039995	2	Singlet	FAPs
VAT_2M	2051.5	1422	1.2453305	2	Singlet	FAPs
VAT_2M	1442.1	1006	5.3298185	2	Singlet	FAPs
VAT_2M	1018.9	824	0.2388656	0	Singlet	Adipocyte
VAT_2M	7891.4	2823	0.0858769	0	Singlet	Adipocyte
VAT_2M	3464.3	1941	1.1399976	5	Singlet	Immune_cells
VAT_2M	2386.5	1501	1.3835249	3	Singlet	FAPs
VAT_2M	1394.7	1020	1.4415198	2	Singlet	FAPs
VAT_2M	2570.7	1596	1.3699138	0	Singlet	Adipocyte
VAT_2M	6060.6	2603	0	0	Singlet	Adipocyte
VAT_2M	1664	1268	0.955549	6	Singlet	Neurons
VAT_2M	1402	904	0.1155108	3	Singlet	FAPs
VAT_2M	1648.8	1226	0.7171032	5	Singlet	Immune_cells
VAT_2M	2167.7	1434	0.2535599	6	Singlet	Neurons
VAT_2M	1130.5	841	2.0284642	5	Singlet	Immune_cells
VAT_2M	4909.9	2311	0.279815	0	Singlet	Adipocyte
VAT_2M	1266.2	1042	0.8525777	8	Singlet	Mesothelial
VAT_2M	1880.9	1233	1.0831975	2	Singlet	FAPs
VAT_2M	5215.4	2376	0.00791	0	Singlet	Adipocyte
VAT_2M	1627	1301	2.7619456	5	Singlet	Immune_cells
VAT_2M	1114.3	889	1.1037175	6	Singlet	Neurons
VAT_2M	1072.8	850	2.0386414	1	Singlet	Immune_cells
VAT_2M	1120.7	855	0.8057514	7	Singlet	Endothelial_cells
VAT_2M	4483.9	1972	0	0	Singlet	Adipocyte
VAT_2M	2911.5	1610	0.0778809	0	Singlet	Adipocyte
VAT_2M	2306.8	1400	0.3039591	0	Singlet	Adipocyte
VAT_2M	3147.6	1800	0.4298058	3	Singlet	FAPs
VAT_2M	1254.3	1027	1.0123756	3	Singlet	FAPs
VAT_2M	1590.2	1145	0.9768827	2	Singlet	FAPs
VAT_2M	2623.5	1592	0.9095615	5	Singlet	Immune_cells
VAT_2M	9341.5	3206	0.2391658	0	Singlet	Adipocyte
VAT_2M	2109.4	1292	0.2050038	3	Singlet	FAPs
VAT_2M	3003.3	1608	0.4837806	0	Singlet	Adipocyte
VAT_2M	4806.3	2237	0.0108131	0	Singlet	Adipocyte
VAT_2M	1357.6	1003	3.898245	9	Singlet	Mesothelial
VAT_2M	2678.3	1522	0.2776526	0	Singlet	Adipocyte
VAT_2M	1275.4	1022	1.7824161	1	Singlet	Immune_cells
VAT_2M	8938.5	3101	0.0011099	0	Singlet	Adipocyte

VAT_2M	1151.5	897	2.3398204	6	Singlet	Neurons
VAT_2M	1998.6	1332	2.7074474	2	Singlet	FAPs
VAT_2M	6928.8	2863	0.3069689	0	Singlet	Adipocyte
VAT_2M	6048	2499	0	0	Singlet	Adipocyte
VAT_2M	1909.6	1453	1.1044791	6	Singlet	Neurons
VAT_2M	1747.5	1113	0.32156	0	Singlet	Adipocyte
VAT_2M	3482.9	1862	0.2760334	0	Singlet	Adipocyte
VAT_2M	1311.8	975	0.3662612	2	Singlet	FAPs
VAT_2M	9270.4	3124	0	0	Singlet	Adipocyte
VAT_2M	2357.9	1542	0.3355238	5	Singlet	Immune_cells
VAT_2M	9650.4	3277	0.4909393	0	Singlet	Adipocyte
VAT_2M	7610.6	2950	0.0111939	0	Singlet	Adipocyte
VAT_2M	9213.8	3277	0.0561122	0	Singlet	Adipocyte
VAT_2M	2158.8	1437	1.8601854	2	Singlet	FAPs
VAT_2M	6003.4	2455	0.1612036	0	Singlet	Adipocyte
VAT_2M	2304.9	1523	2.2775939	2	Singlet	FAPs
VAT_2M	1474.2	1144	1.985858	6	Singlet	Neurons
VAT_2M	6918.4	2859	0.0823781	0	Singlet	Adipocyte
VAT_2M	2990.3	1497	2.5523427	0	Singlet	Adipocyte
VAT_2M	2343.8	1316	1.2988983	0	Singlet	Adipocyte
VAT_2M	3010.2	1844	2.2322113	3	Singlet	FAPs
VAT_2M	2043.9	1120	0.0358416	0	Singlet	Adipocyte
VAT_2M	1051.4	848	4.4237419	9	Singlet	Mesothelial
VAT_2M	1899.1	1376	1.0452272	7	Singlet	Endothelial_cells
VAT_2M	5087.7	2142	0.4668501	0	Singlet	Adipocyte
VAT_2M	3610.5	1888	0.1931324	0	Singlet	Adipocyte
VAT_2M	7546.6	2910	0.065704	0	Singlet	Adipocyte
VAT_2M	1259.9	875	2.0445907	5	Singlet	Immune_cells
VAT_2M	1197.5	915	3.3058619	6	Singlet	Neurons
VAT_2M	1177.2	902	9.461318	6	Singlet	Neurons
VAT_2M	3528	2121	1.2338537	3	Singlet	FAPs
VAT_2M	2694.7	1540	0.6842048	0	Singlet	Adipocyte
VAT_2M	1464	1102	0.9088563	3	Singlet	FAPs
VAT_2M	1366.2	849	9.5091073	0	Singlet	Adipocyte
VAT_2M	3703.5	2011	0.0518154	0	Singlet	Adipocyte
VAT_2M	1119	967	0.4462355	8	Singlet	Mesothelial
VAT_2M	2234.3	1344	0.1650928	0	Singlet	Adipocyte
VAT_2M	1228.2	888	1.1119344	2	Singlet	FAPs
VAT_2M	2645.8	1479	0.096852	0	Singlet	Adipocyte
VAT_2M	1496.5	991	1.4220681	2	Singlet	FAPs
VAT_2M	6237.7	2775	0.045247	0	Singlet	Adipocyte
VAT_2M	1730.7	1057	2.4744474	5	Singlet	Immune_cells
VAT_2M	2062	1179	5.2324458	2	Singlet	FAPs
VAT_2M	2611.6	1497	0.7347268	0	Singlet	Adipocyte
VAT_2M	2586.7	1538	1.0646666	2	Singlet	FAPs
VAT_2M	1743.1	1253	2.247804	2	Singlet	FAPs
VAT_2M	5204.6	2157	0.0722563	0	Singlet	Adipocyte
VAT_2M	4865.6	2259	0.2909068	0	Singlet	Adipocyte
VAT_2M	1185.9	952	0.8157559	2	Singlet	FAPs
VAT_2M	1120.9	869	0.4538697	2	Singlet	FAPs
VAT_2M	5822.5	2428	0.4210219	0	Singlet	Adipocyte

VAT_2M	1563.5	1032	0.1711733	3	Singlet	FAPs
VAT_2M	1103.7	862	1.1899903	7	Singlet	Endothelial_cells
VAT_2M	2047.3	1375	0.4525324	5	Singlet	Immune_cells
VAT_2M	10868	3446	0.0263621	0	Singlet	Adipocyte
VAT_2M	2367.6	1449	2.3302415	7	Singlet	Endothelial_cells
VAT_2M	5933.2	2473	0.9580873	0	Singlet	Adipocyte
VAT_2M	7851.5	2897	0.0076205	0	Singlet	Adipocyte
VAT_2M	1601.2	1090	0.8632547	2	Singlet	FAPs
VAT_2M	1949.9	1237	0.1254677	0	Singlet	Adipocyte
VAT_2M	1442.8	960	0.362555	5	Singlet	Immune_cells
VAT_2M	1678.7	1162	0.6867255	3	Singlet	FAPs
VAT_2M	4519.8	2248	0.1809976	0	Singlet	Adipocyte
VAT_2M	3519.3	2095	3.1541358	2	Singlet	FAPs
VAT_2M	7514.9	2756	0.1135092	0	Singlet	Adipocyte
VAT_2M	1629	1152	0.5565485	2	Singlet	FAPs
VAT_2M	1739.1	1205	0.8839214	2	Singlet	FAPs
VAT_2M	3892.7	2027	0.0550039	0	Singlet	Adipocyte
VAT_2M	1185.2	908	0.0283782	3	Singlet	FAPs
VAT_2M	1007	771	1.4025325	2	Singlet	FAPs
VAT_2M	1164.4	830	1.6143221	2	Singlet	FAPs
VAT_2M	1741.4	1267	0.4996746	2	Singlet	FAPs
VAT_2M	1041.8	786	0.6489174	2	Singlet	FAPs
VAT_2M	2355	1623	0.1856061	2	Singlet	FAPs
VAT_2M	6294.6	2635	0.4782318	0	Singlet	Adipocyte
VAT_2M	1722.6	1118	1.0452858	6	Singlet	Neurons
VAT_2M	5090.9	2425	0.1812601	0	Singlet	Adipocyte
VAT_2M	2137.9	1214	0.1364369	0	Singlet	Adipocyte
VAT_2M	1169.2	937	0.4807916	7	Singlet	Endothelial_cells
VAT_2M	1854.7	1017	0.2220026	0	Singlet	Adipocyte
VAT_2M	4649.4	2412	1.9504953	2	Singlet	FAPs
VAT_2M	2117.8	1282	0.7837706	0	Singlet	Adipocyte
VAT_2M	5379.8	2262	0.0306575	0	Singlet	Adipocyte
VAT_2M	3387.6	1935	0.3761015	2	Singlet	FAPs
VAT_2M	1025.6	810	0.8076445	6	Singlet	Neurons
VAT_2M	1896.4	1239	3.5781001	5	Singlet	Immune_cells
VAT_2M	5488.8	2586	0.0262864	0	Singlet	Adipocyte
VAT_2M	1669.3	1113	0.7706084	5	Singlet	Immune_cells
VAT_2M	6917.7	2834	0.0918646	0	Singlet	Adipocyte
VAT_2M	11129	3434	0.0154174	0	Singlet	Adipocyte
VAT_2M	3616.9	1899	0.6648447	3	Singlet	FAPs
VAT_2M	2541.1	1572	1.2263123	0	Singlet	Adipocyte
VAT_2M	7531	2922	0.0562966	0	Singlet	Adipocyte
VAT_2M	5145.9	2236	0.0082863	0	Singlet	Adipocyte
VAT_2M	2910.6	1774	0.1714412	0	Singlet	Adipocyte
VAT_2M	4188.5	2098	0.1604247	0	Singlet	Adipocyte
VAT_2M	1899	1259	0.2722703	0	Singlet	Adipocyte
VAT_2M	1904.2	1401	2.8191867	11	Singlet	Smooth_muscle_cells
VAT_2M	1849.3	1299	0.649726	4	Singlet	Immune_cells
VAT_2M	5374.1	2402	0.189673	0	Singlet	Adipocyte
VAT_2M	1300.5	946	0.1021837	7	Singlet	Endothelial_cells
VAT_2M	1611.4	1148	0.9302504	5	Singlet	Immune_cells

VAT_2M	5903.5	2585	0.0310115	0	Singlet	Adipocyte
VAT_2M	5871.2	2573	0.0691539	0	Singlet	Adipocyte
VAT_2M	2976.9	1907	1.7049726	3	Singlet	FAPs
VAT_2M	2334.8	1530	0.9517059	6	Singlet	Neurons
VAT_2M	7399.7	2821	0.0449093	0	Singlet	Adipocyte
VAT_2M	1938.8	1271	1.9519378	9	Singlet	Mesothelial
VAT_2M	1537.8	838	1.115992	5	Singlet	Immune_cells
VAT_2M	3000	1678	0.0743913	0	Singlet	Adipocyte
VAT_2M	2301.4	1646	0.7534367	3	Singlet	FAPs
VAT_2M	4560.6	2182	0.4590346	0	Singlet	Adipocyte
VAT_2M	1309	905	3.3098591	5	Singlet	Immune_cells
VAT_2M	10410	3276	0.0172556	0	Singlet	Adipocyte
VAT_2M	1131.1	809	0.6436873	7	Singlet	Endothelial_cells
VAT_2M	6328.8	2702	0.5247715	0	Singlet	Adipocyte
VAT_2M	1278.8	1054	2.161878	8	Singlet	Mesothelial
VAT_2M	2177.4	1355	0.9069532	0	Singlet	Adipocyte
VAT_2M	4763.2	2176	0.0780908	0	Singlet	Adipocyte
VAT_2M	5089.1	2167	0.0648678	0	Singlet	Adipocyte
VAT_2M	1063.7	849	2.9649469	2	Singlet	FAPs
VAT_2M	2002.7	1247	0.1617396	0	Singlet	Adipocyte
VAT_2M	1466.9	920	0.8470323	2	Singlet	FAPs
VAT_2M	1163.2	955	0.1428385	3	Singlet	FAPs
VAT_2M	1116.2	832	1.5864244	11	Singlet	Smooth_muscle_cells
VAT_2M	1711.9	1149	0.3141794	2	Singlet	FAPs
VAT_2M	2360.1	1346	1.316298	5	Singlet	Immune_cells
VAT_2M	5363.3	2774	0.1336626	2	Singlet	FAPs
VAT_2M	2006.2	1273	1.0823039	6	Singlet	Neurons
VAT_2M	1261.4	903	0.1497535	5	Singlet	Immune_cells
VAT_2M	6820.9	2615	0.1284243	0	Singlet	Adipocyte
VAT_2M	6218.2	2535	0.0724253	0	Singlet	Adipocyte
VAT_2M	5790.4	2526	0.0884275	0	Singlet	Adipocyte
VAT_2M	1070.7	811	2.2395465	2	Singlet	FAPs
VAT_2M	3412.2	1811	0.9718429	0	Singlet	Adipocyte
VAT_2M	5465.1	2350	0.0117682	0	Singlet	Adipocyte
VAT_2M	5393.9	2323	0.0239874	0	Singlet	Adipocyte
VAT_2M	5390.9	2428	0.0250171	0	Singlet	Adipocyte
VAT_2M	1920.6	1186	2.1648873	3	Singlet	FAPs
VAT_2M	3082.7	1610	0.6032845	0	Singlet	Adipocyte
VAT_2M	1031.8	813	1.0811988	9	Singlet	Mesothelial
VAT_2M	1386.8	1014	0.2493508	3	Singlet	FAPs
VAT_2M	1474.9	973	1.8353325	2	Singlet	FAPs
VAT_2M	1123.8	919	1.1301023	8	Singlet	Mesothelial
VAT_2M	3564.6	1833	0.0263244	0	Singlet	Adipocyte
VAT_2M	6843.2	2750	0.0251571	0	Singlet	Adipocyte
VAT_2M	3061.1	1553	0	0	Singlet	Adipocyte
VAT_2M	2341.3	1586	1.4397854	2	Singlet	FAPs
VAT_2M	5909.8	2793	1.2033231	3	Singlet	FAPs
VAT_2M	1878.7	1325	0.2400064	0	Singlet	Adipocyte
VAT_2M	1285.2	895	2.8885301	2	Singlet	FAPs
VAT_2M	1347.5	1062	2.4515789	11	Singlet	Smooth_muscle_cells
VAT_2M	1601.7	1233	3.243452	1	Singlet	Immune_cells

VAT_2M	2959.2	1271	3.2797334	2	Singlet	FAPs
VAT_2M	3028.1	1568	0.0871633	0	Singlet	Adipocyte
VAT_2M	8130.2	2899	0.0422712	0	Singlet	Adipocyte
VAT_2M	6275.1	2655	0.0202527	0	Singlet	Adipocyte
VAT_2M	1564.4	1115	1.222306	6	Singlet	Neurons
VAT_2M	1840	1265	0.3286101	7	Singlet	Endothelial_cells
VAT_2M	2063.3	906	3.0112288	6	Singlet	Neurons
VAT_2M	2468.5	1457	0.7937104	3	Singlet	FAPs
VAT_2M	2336.1	1488	0.4475891	2	Singlet	FAPs
VAT_2M	1013.2	842	3.0298016	2	Singlet	FAPs
VAT_2M	3405.2	1799	0.0839184	0	Singlet	Adipocyte
VAT_2M	2513.3	1382	2.1299477	2	Singlet	FAPs
VAT_2M	1550	1271	1.1676591	11	Singlet	Smooth_muscle_cells
VAT_2M	11113	3545	0	0	Singlet	Adipocyte
VAT_2M	1684.2	1123	1.247694	6	Singlet	Neurons
VAT_2M	2485.4	1444	0.1434535	0	Singlet	Adipocyte
VAT_2M	1391.5	962	0.2570289	7	Singlet	Endothelial_cells
VAT_2M	9260.2	3254	0.0519122	0	Singlet	Adipocyte
VAT_2M	3945.3	1983	0.3571122	0	Singlet	Adipocyte
VAT_2M	2022	1201	3.5948916	3	Singlet	FAPs
VAT_2M	1025.2	819	2.1669838	8	Singlet	Mesothelial
VAT_2M	2727.1	1814	0.6185688	2	Singlet	FAPs
VAT_2M	3248.7	1704	0.3222242	2	Singlet	FAPs
VAT_2M	7853.5	3082	0.0334106	0	Singlet	Adipocyte
VAT_2M	1131	930	1.3779697	2	Singlet	FAPs
VAT_2M	2108.3	1243	1.1770378	0	Singlet	Adipocyte
VAT_2M	2564.3	1470	0.2297363	0	Singlet	Adipocyte
VAT_2M	6488.1	2622	0.0057919	0	Singlet	Adipocyte
VAT_2M	2623.8	1441	0.5072257	0	Singlet	Adipocyte
VAT_2M	7343.4	2885	0.3716426	0	Singlet	Adipocyte
VAT_2M	1291.9	1106	1.121472	8	Singlet	Mesothelial
VAT_2M	1843.3	1289	1.592525	1	Singlet	Immune_cells
VAT_2M	1948.8	1426	3.6090947	2	Singlet	FAPs
VAT_2M	2544.3	1620	1.1912354	2	Singlet	FAPs
VAT_2M	6030.8	2556	0.0120904	0	Singlet	Adipocyte
VAT_2M	1831.7	1177	0.089147	0	Singlet	Adipocyte
VAT_2M	6107.6	2496	0.1462504	0	Singlet	Adipocyte
VAT_2M	1688.7	1079	0.2225242	0	Singlet	Adipocyte
VAT_2M	3896.7	1870	0.1835203	0	Singlet	Adipocyte
VAT_2M	1097.4	823	0.448818	7	Singlet	Endothelial_cells
VAT_2M	3670.1	1942	0.0776213	0	Singlet	Adipocyte
VAT_2M	2970	1773	0.2774673	2	Singlet	FAPs
VAT_2M	8296.4	2927	0.0278679	0	Singlet	Adipocyte
VAT_2M	2667.3	1665	3.1507087	2	Singlet	FAPs
VAT_2M	1436.5	1048	0.5366671	6	Singlet	Neurons
VAT_2M	4124.8	2105	0.0770328	0	Singlet	Adipocyte
VAT_2M	20715	4737	0.0625395	0	Singlet	Adipocyte
VAT_2M	8998.9	3166	0.006773	0	Singlet	Adipocyte
VAT_2M	1041.5	771	1.4422641	3	Singlet	FAPs
VAT_2M	1134.8	927	0.5360145	6	Singlet	Neurons
VAT_2M	8683	3037	0.1341473	0	Singlet	Adipocyte

VAT_2M	6885.1	2755	0.0118656	0	Singlet	Adipocyte
VAT_2M	2110.3	1443	0.5105445	2	Singlet	FAPs
VAT_2M	9259.7	3286	0.0042028	0	Singlet	Adipocyte
VAT_2M	1453	1212	1.157659	8	Singlet	Mesothelial
VAT_2M	3524.1	1768	0.0408832	0	Singlet	Adipocyte
VAT_2M	1229.3	1006	0.9005219	6	Singlet	Neurons
VAT_2M	1149.3	891	0.10846	3	Singlet	FAPs
VAT_2M	3529.2	1831	0.0763418	0	Singlet	Adipocyte
VAT_2M	5897.3	2441	0.0821164	0	Singlet	Adipocyte
VAT_2M	1376.4	1087	0.2430363	5	Singlet	Immune_cells
VAT_2M	1043.5	863	2.1067241	2	Singlet	FAPs
VAT_2M	5960.6	2695	0.1137099	0	Singlet	Adipocyte
VAT_2M	2851.5	1496	7.0225733	7	Singlet	Endothelial_cells
VAT_2M	1791.4	1258	0.4739619	3	Singlet	FAPs
VAT_2M	1506.7	1089	4.4855682	2	Singlet	FAPs
VAT_2M	1113.1	732	2.2175407	9	Singlet	Mesothelial
VAT_2M	4296.4	2112	0.1761087	0	Singlet	Adipocyte
VAT_2M	5597.8	2429	0.0342164	0	Singlet	Adipocyte
VAT_2M	2418.4	1398	0.0480121	3	Singlet	FAPs
VAT_2M	8225.8	3006	0.0154976	0	Singlet	Adipocyte
VAT_2M	2684.9	1496	0.4143242	0	Singlet	Adipocyte
VAT_2M	1737.5	1207	0.3854529	2	Singlet	FAPs
VAT_2M	7214	2832	0.0657931	0	Singlet	Adipocyte
VAT_2M	1000.7	782	2.5304814	2	Singlet	FAPs
VAT_2M	6974.3	2935	0.0918073	0	Singlet	Adipocyte
VAT_2M	8294.6	2969	0.0075864	0	Singlet	Adipocyte
VAT_2M	1795.3	1217	0.1011971	5	Singlet	Immune_cells
VAT_2M	1041.4	773	0.49764	2	Singlet	FAPs
VAT_2M	1521.5	1056	0.2526218	3	Singlet	FAPs
VAT_2M	2251.5	1383	0.0615689	2	Singlet	FAPs
VAT_2M	6119	2493	0	0	Singlet	Adipocyte
VAT_2M	5562.7	2413	0.4558227	0	Singlet	Adipocyte
VAT_2M	2306.3	1485	2.5382176	3	Singlet	FAPs
VAT_2M	1786.4	1191	0.3370679	0	Singlet	Adipocyte
VAT_2M	10143	3404	0.0116637	0	Singlet	Adipocyte
VAT_2M	1441.5	975	0.3757586	3	Singlet	FAPs
VAT_2M	6119.2	2570	0.0425484	0	Singlet	Adipocyte
VAT_2M	1608.4	1169	0.7230447	2	Singlet	FAPs
VAT_2M	2124.3	1333	1.323623	0	Singlet	Adipocyte
VAT_2M	3981.2	1970	0.0115706	0	Singlet	Adipocyte
VAT_2M	1824	1105	0.0442413	0	Singlet	Adipocyte
VAT_2M	3064.6	1626	0.0243028	0	Singlet	Adipocyte
VAT_2M	1064.3	797	1.9716835	3	Singlet	FAPs
VAT_2M	4306.1	1898	7.2927348	7	Singlet	Endothelial_cells
VAT_2M	2191.2	1383	0.3224582	3	Singlet	FAPs
VAT_2M	6205.4	2688	0.0098833	0	Singlet	Adipocyte
VAT_2M	2601.1	1598	0.0669751	2	Singlet	FAPs
VAT_2M	3527.4	2078	0.5206719	5	Singlet	Immune_cells
VAT_2M	3887	2044	0.0379161	0	Singlet	Adipocyte
VAT_2M	1898.6	1242	1.9524413	0	Singlet	Adipocyte
VAT_2M	1000.9	844	0.8542607	6	Singlet	Neurons

VAT_2M	4159.5	2041	0.1721701	0	Singlet	Adipocyte
VAT_2M	7771.9	2860	0.0047425	0	Singlet	Adipocyte
VAT_2M	7066.9	2696	0.0713808	0	Singlet	Adipocyte
VAT_2M	1080.3	759	3.5207288	6	Singlet	Neurons
VAT_2M	5680.3	2285	0.0989657	0	Singlet	Adipocyte
VAT_2M	3961.8	1927	0.2435178	0	Singlet	Adipocyte
VAT_2M	4882.3	2294	0.0416407	0	Singlet	Adipocyte
VAT_2M	8647.5	3073	0.0720498	0	Singlet	Adipocyte
VAT_2M	5515.9	2519	0.2129976	0	Singlet	Adipocyte
VAT_2M	1829.3	1364	1.2808146	1	Singlet	Immune_cells
VAT_2M	1255.2	947	2.0152734	3	Singlet	FAPs
VAT_2M	5120.3	2205	0.0561335	0	Singlet	Adipocyte
VAT_2M	4098.5	1882	1.080731	0	Singlet	Adipocyte
VAT_2M	1611.8	1043	0.8565892	2	Singlet	FAPs
VAT_2M	1648.7	1191	0.9229416	3	Singlet	FAPs
VAT_2M	1898.5	1275	0.7764339	2	Singlet	FAPs
VAT_2M	4896.8	2092	0.076562	0	Singlet	Adipocyte
VAT_2M	1312.2	1078	0.6967089	6	Singlet	Neurons
VAT_2M	1176.3	1008	1.1460855	6	Singlet	Neurons
VAT_2M	1125	825	3.4357041	9	Singlet	Mesothelial
VAT_2M	1559.5	1106	2.1010622	6	Singlet	Neurons
VAT_2M	3207.4	1987	0.6927909	5	Singlet	Immune_cells
VAT_2M	1912.8	1191	0.8005821	3	Singlet	FAPs
VAT_2M	2699.1	1752	1.9576792	5	Singlet	Immune_cells
VAT_2M	1372.8	1037	0.3934246	7	Singlet	Endothelial_cells
VAT_2M	7741.4	2970	0.3509997	0	Singlet	Adipocyte
VAT_2M	4671.5	2209	0.2039743	3	Singlet	FAPs
VAT_2M	1945.9	1240	1.0228892	3	Singlet	FAPs
VAT_2M	1367	989	2.3541654	2	Singlet	FAPs
VAT_2M	1587.9	1074	2.922043	2	Singlet	FAPs
VAT_2M	3222.5	1655	0.1523705	0	Singlet	Adipocyte
VAT_2M	9040.4	2983	0.0124274	0	Singlet	Adipocyte
VAT_2M	5126.8	2499	0.3213028	0	Singlet	Adipocyte
VAT_2M	2105.8	1228	0.2041877	0	Singlet	Adipocyte
VAT_2M	13240	3577	0.0004109	0	Singlet	Adipocyte
VAT_2M	1035.9	724	0.7880656	2	Singlet	FAPs
VAT_2M	4176	2036	0.1321737	0	Singlet	Adipocyte
VAT_2M	7118.3	2773	0.0335297	0	Singlet	Adipocyte
VAT_2M	2148.9	1323	0.7798356	0	Singlet	Adipocyte
VAT_2M	6471.5	2781	0.0684404	0	Singlet	Adipocyte
VAT_2M	8363.7	3041	1.2365084	0	Singlet	Adipocyte
VAT_2M	2992.7	1689	0.073236	0	Singlet	Adipocyte
VAT_2M	1081.5	729	2.1137721	3	Singlet	FAPs
VAT_2M	2405.2	1576	3.6020278	2	Singlet	FAPs
VAT_2M	1718.1	1045	0	0	Singlet	Adipocyte
VAT_2M	2639.7	1477	0	0	Singlet	Adipocyte
VAT_2M	1764.8	1142	0	0	Singlet	Adipocyte
VAT_2M	3489.9	1886	0.0197884	0	Singlet	Adipocyte
VAT_2M	1333.4	1173	1.3512432	6	Singlet	Neurons
VAT_2M	1704.7	1242	0.5115464	2	Singlet	FAPs
VAT_2M	1001.9	773	1.7207142	9	Singlet	Mesothelial

VAT_2M	2406.3	1477	0.4678624	0	Singlet	Adipocyte
VAT_2M	2632.5	1612	1.609859	3	Singlet	FAPs
VAT_2M	1814.9	1209	0	3	Singlet	FAPs
VAT_2M	9429.1	3150	0	0	Singlet	Adipocyte
VAT_2M	4994.2	2301	0.0855786	0	Singlet	Adipocyte
VAT_2M	2721.5	1594	0	0	Singlet	Adipocyte
VAT_2M	1060.3	758	1.4010877	9	Singlet	Mesothelial
VAT_2M	5746.4	2622	0.0231418	0	Singlet	Adipocyte
VAT_2M	8394.4	3014	0.0294424	0	Singlet	Adipocyte
VAT_2M	2703.8	1635	1.1523867	5	Singlet	Immune_cells
VAT_2M	1401.7	1039	1.8626651	11	Singlet	Smooth_muscle_cells
VAT_2M	3267.2	1737	0.1947566	0	Singlet	Adipocyte
VAT_2M	2954.7	1643	0.1385695	0	Singlet	Adipocyte
VAT_2M	1548.1	1054	5.915943	0	Singlet	Adipocyte
VAT_2M	1149.4	901	2.1629803	5	Singlet	Immune_cells
VAT_2M	1594.4	1113	4.9277853	9	Singlet	Mesothelial
VAT_2M	1597	1155	0.438142	1	Singlet	Immune_cells
VAT_2M	2043.4	1314	6.7989331	7	Singlet	Endothelial_cells
VAT_2M	2310.7	1414	0.7245249	0	Singlet	Adipocyte
VAT_2M	2255.7	1378	0.1448647	0	Singlet	Adipocyte
VAT_2M	9949.5	3579	0.033462	0	Singlet	Adipocyte
VAT_2M	1918	1288	0.9771614	3	Singlet	FAPs
VAT_2M	2558.2	1599	0.4976751	6	Singlet	Neurons
VAT_2M	2910.6	1646	0.0693426	0	Singlet	Adipocyte
VAT_2M	1031.4	833	1.9893819	7	Singlet	Endothelial_cells
VAT_2M	2106.5	1267	0.2533299	0	Singlet	Adipocyte
VAT_2M	1493.3	1208	0.5589947	8	Singlet	Mesothelial
VAT_2M	1025.6	827	0.6107541	4	Singlet	Immune_cells
VAT_2M	2533.6	1136	2.1391831	3	Singlet	FAPs
VAT_2M	1462.8	1094	0.6317276	3	Singlet	FAPs
VAT_2M	1158.3	908	1.5648763	7	Singlet	Endothelial_cells
VAT_2M	12943	3767	0.0051873	0	Singlet	Adipocyte
VAT_2M	2974.3	1615	0.1139376	0	Singlet	Adipocyte
VAT_2M	2052.3	1386	0.0767487	3	Singlet	FAPs
VAT_2M	1114.5	947	0.7410149	8	Singlet	Mesothelial
VAT_2M	4184	2015	0	0	Singlet	Adipocyte
VAT_2M	1017.7	728	1.7439254	7	Singlet	Endothelial_cells
VAT_2M	2065.5	1495	0.8436495	3	Singlet	FAPs
VAT_2M	2153.9	1650	1.1928204	6	Singlet	Neurons
VAT_2M	1119.9	954	1.3250634	1	Singlet	Immune_cells
VAT_2M	2533	1437	0.080238	0	Singlet	Adipocyte
VAT_2M	2140	1148	11.104439	0	Singlet	Adipocyte
VAT_2M	4657.8	2249	1.7967322	3	Singlet	FAPs
VAT_2M	1395.8	960	1.21795	3	Singlet	FAPs
VAT_2M	5384.6	2413	0.2018967	0	Singlet	Adipocyte
VAT_2M	1302.5	976	1.5179676	6	Singlet	Neurons
VAT_2M	2459.3	1551	1.5603485	6	Singlet	Neurons
VAT_2M	3602.6	1964	0.0483065	0	Singlet	Adipocyte
VAT_2M	2006	1262	0.0876429	0	Singlet	Adipocyte
VAT_2M	5234	2375	0.5982026	5	Singlet	Immune_cells
VAT_2M	5357.2	2485	0.3525104	0	Singlet	Adipocyte

VAT_2M	4223.8	2100	0.2401828	0	Singlet	Adipocyte
VAT_2M	1536.1	1034	0.1535158	3	Singlet	FAPs
VAT_2M	3639.2	1940	0.1001711	0	Singlet	Adipocyte
VAT_2M	3909.2	2109	0	0	Singlet	Adipocyte
VAT_2M	7756.4	3045	0.0188389	0	Singlet	Adipocyte
VAT_2M	1130.2	881	5.0460089	0	Singlet	Adipocyte
VAT_2M	2215.8	1326	0.011229	0	Singlet	Adipocyte
VAT_2M	10576	3391	0.0439454	0	Singlet	Adipocyte
VAT_2M	3007.9	1651	0.0580298	2	Singlet	FAPs
VAT_2M	8979.7	3122	0	0	Singlet	Adipocyte
VAT_2M	2886.3	1757	0.1727369	7	Singlet	Endothelial_cells
VAT_2M	2095	1221	0.6593936	0	Singlet	Adipocyte
VAT_2M	2185.1	1451	0.8385997	2	Singlet	FAPs
VAT_2M	1209.2	903	0.1607897	3	Singlet	FAPs
VAT_2M	6892.9	2669	0.3926678	0	Singlet	Adipocyte
VAT_2M	1163.7	981	1.7298433	8	Singlet	Mesothelial
VAT_2M	1266.1	966	0.090079	9	Singlet	Mesothelial
VAT_2M	1474.7	1059	0.4328609	2	Singlet	FAPs
VAT_2M	1834.4	1393	0.8929261	7	Singlet	Endothelial_cells
VAT_2M	3735.4	2074	0.2747216	2	Singlet	FAPs
VAT_2M	7505.4	2795	0.1172554	0	Singlet	Adipocyte
VAT_2M	1510.6	1188	1.0415661	8	Singlet	Mesothelial
VAT_2M	1904.8	1155	0.2173928	0	Singlet	Adipocyte
VAT_2M	1296.4	867	1.2608852	7	Singlet	Endothelial_cells
VAT_2M	6125.9	2718	0.0343116	0	Singlet	Adipocyte
VAT_2M	1403.2	1053	1.1600132	2	Singlet	FAPs
VAT_2M	1028.9	773	3.5800265	5	Singlet	Immune_cells
VAT_2M	5189	2266	0.0952992	0	Singlet	Adipocyte
VAT_2M	2464.2	1630	0.1567972	2	Singlet	FAPs
VAT_2M	1359.4	1174	1.5153204	6	Singlet	Neurons
VAT_2M	1807.9	1043	3.3159168	0	Singlet	Adipocyte
VAT_2M	1187.1	845	3.3258104	9	Singlet	Mesothelial
VAT_2M	1110.2	905	1.0880607	8	Singlet	Mesothelial
VAT_2M	1372	1117	0.1166008	6	Singlet	Neurons
VAT_2M	2001.1	1308	0.4011397	0	Singlet	Adipocyte
VAT_2M	2234.9	1287	0.9294647	0	Singlet	Adipocyte
VAT_2M	3228.2	2008	0.3265023	2	Singlet	FAPs
VAT_2M	1175	864	0.583175	2	Singlet	FAPs
VAT_2M	1724.7	1040	0.403652	0	Singlet	Adipocyte
VAT_2M	3016.3	1743	0.1451591	7	Singlet	Endothelial_cells
VAT_2M	3293.9	1994	0.9515869	2	Singlet	FAPs
VAT_2M	2253.3	1514	2.1955735	6	Singlet	Neurons
VAT_2M	1949.8	1304	2.190391	2	Singlet	FAPs
VAT_2M	2107.4	1519	2.3797274	2	Singlet	FAPs
VAT_2M	4319.1	1992	0.037725	0	Singlet	Adipocyte
VAT_2M	1203.8	1073	1.178921	6	Singlet	Neurons
VAT_2M	6766.1	2883	0.0528139	0	Singlet	Adipocyte
VAT_2M	3666.1	2077	2.1675414	5	Singlet	Immune_cells
VAT_2M	1661.5	1088	1.0776555	6	Singlet	Neurons
VAT_2M	7331.9	2779	0.0022709	0	Singlet	Adipocyte
VAT_2M	3936.6	2008	0.1173095	0	Singlet	Adipocyte

VAT_2M	3509.6	1903	0.846479	0 Singlet	Adipocyte
VAT_2M	1864.1	1198	1.3009708	5 Singlet	Immune_cells
VAT_2M	3392.5	2054	1.0757553	2 Singlet	FAPs
VAT_2M	1879.9	1324	1.6447795	6 Singlet	Neurons
VAT_2M	1857.6	1184	0.2995851	0 Singlet	Adipocyte
VAT_2M	3183.1	1741	0.0719256	0 Singlet	Adipocyte
VAT_2M	1558.3	1060	2.4248113	9 Singlet	Mesothelial
VAT_2M	10999	3540	0	0 Singlet	Adipocyte
VAT_2M	1483.3	1135	2.0293183	2 Singlet	FAPs
VAT_2M	1237.7	880	0.1759392	4 Singlet	Immune_cells
VAT_2M	1386.9	974	0.4203295	3 Singlet	FAPs
VAT_2M	4598.2	2468	1.0855344	2 Singlet	FAPs
VAT_2M	1147.4	870	3.4464933	2 Singlet	FAPs
VAT_2M	2007.5	1252	0.2610204	3 Singlet	FAPs
VAT_2M	1191.4	897	0.9205405	5 Singlet	Immune_cells
VAT_2M	3673.2	1905	0.0182185	0 Singlet	Adipocyte
VAT_2M	3687.8	1937	0.4759324	5 Singlet	Immune_cells
VAT_2M	5829.5	2475	0.0485826	0 Singlet	Adipocyte
VAT_2M	3023.9	1915	0.119352	2 Singlet	FAPs
VAT_2M	5276.8	2541	0.0784073	0 Singlet	Adipocyte
VAT_2M	1969.1	1310	0.2204702	3 Singlet	FAPs
VAT_2M	1153.1	854	2.3840124	4 Singlet	Immune_cells
VAT_2M	7009.9	2679	0.0251488	0 Singlet	Adipocyte
VAT_2M	3657.4	2076	4.1506705	0 Singlet	Adipocyte
VAT_2M	1270.4	978	2.0102635	2 Singlet	FAPs
VAT_2M	8341.4	3016	0.0664206	0 Singlet	Adipocyte
VAT_2M	1352.3	1106	4.7155443	6 Singlet	Neurons
VAT_2M	1223.4	980	0.7795467	2 Singlet	FAPs
VAT_2M	1516.9	1166	0.4707795	2 Singlet	FAPs
VAT_2M	3733.3	1817	0.1813512	0 Singlet	Adipocyte
VAT_2M	5449.6	2413	2.5112073	0 Singlet	Adipocyte
VAT_2M	1522.9	1099	0.6765287	5 Singlet	Immune_cells
VAT_2M	11794	3617	0.0043725	0 Singlet	Adipocyte
VAT_2M	2291.4	1559	0.9962101	2 Singlet	FAPs
VAT_2M	2327.3	1537	0.4279351	6 Singlet	Neurons
VAT_2M	1093.7	814	0.4665636	2 Singlet	FAPs
VAT_2M	4034.8	2051	0.3639094	0 Singlet	Adipocyte
VAT_2M	2562.6	1486	0.5803241	0 Singlet	Adipocyte
VAT_2M	2172.9	1365	0.1965705	0 Singlet	Adipocyte
VAT_2M	3594	1856	0.1583813	0 Singlet	Adipocyte
VAT_2M	1609.9	1193	0.4717211	3 Singlet	FAPs
VAT_2M	1433.8	1101	0.4756628	6 Singlet	Neurons
VAT_2M	2500.6	1564	0.4493221	0 Singlet	Adipocyte
VAT_2M	8583.8	3065	0.0968029	0 Singlet	Adipocyte
VAT_2M	2517.3	1634	0.2636082	2 Singlet	FAPs
VAT_2M	1755.9	1285	0.6594928	2 Singlet	FAPs
VAT_2M	1442.6	1079	3.3771606	3 Singlet	FAPs
VAT_2M	1220.1	931	0.9368838	3 Singlet	FAPs
VAT_2M	4733.5	2319	0.3394697	0 Singlet	Adipocyte
VAT_2M	1541	1320	1.2304588	6 Singlet	Neurons
VAT_2M	1693.5	1172	3.4982769	9 Singlet	Mesothelial

VAT_2M	9931.8	3495	0.0155333	0	Singlet	Adipocyte
VAT_2M	1612.9	1031	1.7700705	0	Singlet	Adipocyte
VAT_2M	2639	1544	0.093869	0	Singlet	Adipocyte
VAT_2M	3038.2	1901	0.5567862	2	Singlet	FAPs
VAT_2M	5447.2	2403	0.0632759	0	Singlet	Adipocyte
VAT_2M	5647.5	2449	0.0040277	0	Singlet	Adipocyte
VAT_2M	1956.8	1342	0.5760728	2	Singlet	FAPs
VAT_2M	3120.9	1576	8.3516059	7	Singlet	Endothelial_cells
VAT_2M	4583.4	2108	0.0106942	0	Singlet	Adipocyte
VAT_2M	3227.7	1528	0.1242558	0	Singlet	Adipocyte
VAT_2M	6168.2	2436	0	0	Singlet	Adipocyte
VAT_2M	3738.9	1893	0.1641368	0	Singlet	Adipocyte
VAT_2M	2734.1	1468	0.7339707	0	Singlet	Adipocyte
VAT_2M	9244.4	3085	0.1707152	0	Singlet	Adipocyte
VAT_2M	3698.1	2095	1.8439768	2	Singlet	FAPs
VAT_2M	1920.9	1210	0.218968	7	Singlet	Endothelial_cells
VAT_2M	1104.9	795	0.5310008	3	Singlet	FAPs
VAT_2M	1793.9	1363	1.0209144	2	Singlet	FAPs
VAT_2M	6124.6	2713	0.0840892	0	Singlet	Adipocyte
VAT_2M	4763.5	2192	0.021558	0	Singlet	Adipocyte
VAT_2M	5031.9	2298	0.0992286	0	Singlet	Adipocyte
VAT_2M	1374.2	1007	0.9715775	2	Singlet	FAPs
VAT_2M	1494	1222	1.9033863	2	Singlet	FAPs
VAT_2M	1337.6	966	3.6689846	5	Singlet	Immune_cells
VAT_2M	4411	2100	0.0114315	0	Singlet	Adipocyte
VAT_2M	1148.6	804	0.8204379	0	Singlet	Adipocyte
VAT_2M	1332.2	961	0.1643966	3	Singlet	FAPs
VAT_2M	2656.9	1484	0.196224	0	Singlet	Adipocyte
VAT_2M	2020.8	1216	6.6745629	7	Singlet	Endothelial_cells
VAT_2M	1979.9	1369	0.8475306	2	Singlet	FAPs
VAT_2M	3186.3	1794	0.2251035	0	Singlet	Adipocyte
VAT_2M	3259.5	1844	0.2769031	2	Singlet	FAPs
VAT_2M	4642.3	2341	0.0986532	0	Singlet	Adipocyte
VAT_2M	1733.5	1167	0.0974704	2	Singlet	FAPs
VAT_2M	5624.7	2323	0	0	Singlet	Adipocyte
VAT_2M	1920.7	1213	0.8323661	0	Singlet	Adipocyte
VAT_2M	3852.1	1951	0.3247156	0	Singlet	Adipocyte
VAT_2M	1217.1	1007	1.7491423	7	Singlet	Endothelial_cells
VAT_2M	1169.9	945	0.6835685	11	Singlet	Smooth_muscle_cells
VAT_2M	1607.6	1152	1.0457329	2	Singlet	FAPs
VAT_2M	1807.7	1233	2.1974654	3	Singlet	FAPs
VAT_2M	1331.3	1047	2.7204209	8	Singlet	Mesothelial
VAT_2M	1127.2	1022	0.9885217	8	Singlet	Mesothelial
VAT_2M	1381.5	1173	1.6162567	6	Singlet	Neurons
VAT_2M	8174.4	2993	0.0164352	0	Singlet	Adipocyte
VAT_2M	1264.8	1081	0.5736664	6	Singlet	Neurons
VAT_2M	3952.5	2068	0.3629917	0	Singlet	Adipocyte
VAT_2M	4862.1	2296	0.2728718	0	Singlet	Adipocyte
VAT_2M	2925.9	1456	0.3845008	0	Singlet	Adipocyte
VAT_2M	1219	915	0.7873481	7	Singlet	Endothelial_cells
VAT_2M	4621.2	2250	0	0	Singlet	Adipocyte

VAT_2M	1097.3	849	0.2434261	2	Singlet	FAPs
VAT_2M	2216.4	1607	0.1419468	2	Singlet	FAPs
VAT_2M	1134	935	1.028332	6	Singlet	Neurons
VAT_2M	6335.7	2663	0.0329081	0	Singlet	Adipocyte
VAT_2M	1257.4	1061	0.2747724	6	Singlet	Neurons
VAT_2M	7366.4	2713	0.0056686	0	Singlet	Adipocyte
VAT_2M	7163.6	2917	0.0285515	0	Singlet	Adipocyte
VAT_2M	4627.3	2257	0.0836208	0	Singlet	Adipocyte
VAT_2M	2809	1525	0.0201961	11	Singlet	Smooth_muscle_cells
VAT_2M	1568	1125	0.9341087	7	Singlet	Endothelial_cells
VAT_2M	1407.3	959	0.9103279	7	Singlet	Endothelial_cells
VAT_2M	1992.3	1493	2.0053713	7	Singlet	Endothelial_cells
VAT_2M	3196.1	1676	0.057917	0	Singlet	Adipocyte
VAT_2M	4968.8	2293	0.0085745	0	Singlet	Adipocyte
VAT_2M	1563.9	1005	1.5916934	0	Singlet	Adipocyte
VAT_2M	1331.6	977	0.9265719	5	Singlet	Immune_cells
VAT_2M	1028.8	684	3.6749204	2	Singlet	FAPs
VAT_2M	1805.6	1503	1.1058241	6	Singlet	Neurons
VAT_2M	6487.3	2660	0.0647663	0	Singlet	Adipocyte
VAT_2M	5989.7	2558	0.1179718	0	Singlet	Adipocyte
VAT_2M	7340.6	2868	0.029755	0	Singlet	Adipocyte
VAT_2M	2724	1497	0.0248273	0	Singlet	Adipocyte
VAT_2M	1470.5	1123	0.2345533	3	Singlet	FAPs
VAT_2M	6595.2	2647	0.0209419	0	Singlet	Adipocyte
VAT_2M	6257.1	2697	0.0434843	0	Singlet	Adipocyte
VAT_2M	3180.6	1658	0.359283	0	Singlet	Adipocyte
VAT_2M	1444	1003	0.4518888	3	Singlet	FAPs
VAT_2M	1981	1244	1.4743869	0	Singlet	Adipocyte
VAT_2M	1176.7	827	2.2500458	3	Singlet	FAPs
VAT_2M	1416.8	980	3.4049733	7	Singlet	Endothelial_cells
VAT_2M	1631.6	1032	0.3374701	0	Singlet	Adipocyte
VAT_2M	10002	3430	0.0740741	0	Singlet	Adipocyte
VAT_2M	1273.8	919	2.6291508	2	Singlet	FAPs
VAT_2M	2134.6	1387	1.6471165	0	Singlet	Adipocyte
VAT_2M	2233.5	1561	0.7747555	6	Singlet	Neurons
VAT_2M	1089.6	829	0.7328286	2	Singlet	FAPs
VAT_2M	2152.3	1318	0.4618737	2	Singlet	FAPs
VAT_2M	4862.5	2202	0.0130871	0	Singlet	Adipocyte
VAT_2M	1311.5	1053	2.9076316	6	Singlet	Neurons
VAT_2M	1088.2	891	0.7218477	7	Singlet	Endothelial_cells
VAT_2M	2458.3	1540	0.0702733	0	Singlet	Adipocyte
VAT_2M	4427.2	2133	0.0103999	0	Singlet	Adipocyte
VAT_2M	1991.9	1279	0.5285386	0	Singlet	Adipocyte
VAT_2M	1588.3	968	0.1801481	0	Singlet	Adipocyte
VAT_2M	3759.8	1876	0.1055535	0	Singlet	Adipocyte
VAT_2M	2197.1	1355	0.6694578	6	Singlet	Neurons
VAT_2M	7029.4	2980	0.0124465	0	Singlet	Adipocyte
VAT_2M	3133.6	1877	0.0276421	11	Singlet	Smooth_muscle_cells
VAT_2M	5697.6	2514	0.0148791	0	Singlet	Adipocyte
VAT_2M	1272.3	984	0.2781665	2	Singlet	FAPs
VAT_2M	4774.3	2325	0.7164676	0	Singlet	Adipocyte

VAT_2M	7388.5	2868	0.0045962	0 Singlet	Adipocyte
VAT_2M	2473.5	1551	0.1911044	2 Singlet	FAPs
VAT_2M	1811.7	1157	0.2145186	6 Singlet	Neurons
VAT_2M	2020.3	1301	0.5436954	0 Singlet	Adipocyte
VAT_2M	1405.8	1184	2.0724032	6 Singlet	Neurons
VAT_2M	1428.1	1056	1.3200221	5 Singlet	Immune_cells
VAT_2M	1208.2	852	1.8846795	2 Singlet	FAPs
VAT_2M	1326.4	1059	0.2590201	0 Singlet	Adipocyte
VAT_2M	3511.6	1722	0.2677569	0 Singlet	Adipocyte
VAT_2M	5932	2705	0.0540728	0 Singlet	Adipocyte
VAT_2M	3191.3	1720	0.0491952	0 Singlet	Adipocyte
VAT_2M	2658.6	1562	0.2005644	0 Singlet	Adipocyte
VAT_2M	3205.7	1719	0.1429118	0 Singlet	Adipocyte
VAT_2M	11558	3641	0.0082276	0 Singlet	Adipocyte
VAT_2M	2264.2	1275	0.2211241	0 Singlet	Adipocyte
VAT_2M	1415.5	926	2.3941048	2 Singlet	FAPs
VAT_2M	6274.2	2712	0	0 Singlet	Adipocyte
VAT_2M	1970.5	1206	0.2297649	0 Singlet	Adipocyte
VAT_2M	1624.5	1172	5.4577614	3 Singlet	FAPs
VAT_2M	4987.1	2296	0.4420321	0 Singlet	Adipocyte
VAT_2M	1661.1	1262	0.2921382	2 Singlet	FAPs
VAT_2M	5945.1	2533	0.0074232	0 Singlet	Adipocyte
VAT_2M	1630.7	1040	0.1959385	0 Singlet	Adipocyte
VAT_2M	1076.6	893	1.6682447	6 Singlet	Neurons
VAT_2M	2678.9	1429	0.0848322	0 Singlet	Adipocyte
VAT_2M	2524	1528	0.0676119	0 Singlet	Adipocyte
VAT_2M	2980.6	1804	0.924481	0 Singlet	Adipocyte
VAT_2M	3786.9	2077	0.6248425	2 Singlet	FAPs
VAT_2M	1060.7	781	1.7519496	7 Singlet	Endothelial_cells
VAT_2M	1476.4	1082	0.3020327	6 Singlet	Neurons
VAT_2M	5539.9	2613	0.0950261	0 Singlet	Adipocyte
VAT_2M	2472.9	1445	8.2660912	5 Singlet	Immune_cells
VAT_2M	2417.9	1643	1.85746	2 Singlet	FAPs
VAT_2M	1007	865	0.7090937	6 Singlet	Neurons
VAT_2M	1660.5	1097	0.7902319	7 Singlet	Endothelial_cells
VAT_2M	2743.6	1675	0.179854	0 Singlet	Adipocyte
VAT_2M	8745.8	2985	0.0023185	0 Singlet	Adipocyte
VAT_2M	1454.2	1008	2.1243321	2 Singlet	FAPs
VAT_2M	7061.2	2719	0.0518168	0 Singlet	Adipocyte
VAT_2M	1065.3	832	2.1428452	6 Singlet	Neurons
VAT_2M	1283.4	1011	0.3367913	7 Singlet	Endothelial_cells
VAT_2M	1402.2	1038	0.3067545	5 Singlet	Immune_cells
VAT_2M	2365.4	1467	1.397295	2 Singlet	FAPs
VAT_2M	1113.5	909	3.209797	6 Singlet	Neurons
VAT_2M	1603.9	1388	1.266333	6 Singlet	Neurons
VAT_2M	10124	3258	0.0098746	0 Singlet	Adipocyte
VAT_2M	1498.4	1081	2.3145401	3 Singlet	FAPs
VAT_2M	1050.5	838	1.6035443	8 Singlet	Mesothelial
VAT_2M	2000.4	1407	0.6368597	6 Singlet	Neurons
VAT_2M	3803.5	1978	0.0363636	0 Singlet	Adipocyte
VAT_2M	6036.3	2953	1.0496516	2 Singlet	FAPs

VAT_2M	1743	1147	0.7252964	0	Singlet	Adipocyte
VAT_2M	1368.6	1069	3.3321805	1	Singlet	Immune_cells
VAT_2M	6981.8	2802	0.0335958	0	Singlet	Adipocyte
VAT_2M	5589.6	2414	0.0893005	0	Singlet	Adipocyte
VAT_2M	2259.3	1291	2.0197824	0	Singlet	Adipocyte
VAT_2M	7869.8	3031	0.020159	0	Singlet	Adipocyte
VAT_2M	1989.1	1311	1.0252234	2	Singlet	FAPs
VAT_2M	5338.2	2350	0.0690766	0	Singlet	Adipocyte
VAT_2M	2126.6	1370	0.1328719	5	Singlet	Immune_cells
VAT_2M	1687.9	1070	0.2662462	0	Singlet	Adipocyte
VAT_2M	11420	3615	0	0	Singlet	Adipocyte
VAT_2M	1079.8	813	0.4470586	7	Singlet	Endothelial_cells
VAT_2M	1969.3	1329	0.3568673	6	Singlet	Neurons
VAT_2M	1457.3	1089	1.0911708	3	Singlet	FAPs
VAT_2M	2306.3	1381	0.5676134	0	Singlet	Adipocyte
VAT_2M	1717.6	1168	0.8091913	2	Singlet	FAPs
VAT_2M	4399.9	2077	0.0235479	0	Singlet	Adipocyte
VAT_2M	2536.3	1572	1.9891153	6	Singlet	Neurons
VAT_2M	5666.4	2413	0.0761131	0	Singlet	Adipocyte
VAT_2M	2065.9	1362	2.2883501	5	Singlet	Immune_cells
VAT_2M	7263.5	2756	0.8053074	0	Singlet	Adipocyte
VAT_2M	5606.5	2521	0.28063	0	Singlet	Adipocyte
VAT_2M	1283.9	1099	1.2981373	6	Singlet	Neurons
VAT_2M	3571.4	1706	0.0354259	0	Singlet	Adipocyte
VAT_2M	1661	1071	0	7	Singlet	Endothelial_cells
VAT_2M	1435.5	1120	1.1267051	2	Singlet	FAPs
VAT_2M	4978	2338	0.5906606	0	Singlet	Adipocyte
VAT_2M	6175	2798	0.019816	0	Singlet	Adipocyte
VAT_2M	6505.3	2487	0.085179	0	Singlet	Adipocyte
VAT_2M	2447.6	1507	0.059663	0	Singlet	Adipocyte
VAT_2M	2945.5	1797	0.1412832	2	Singlet	FAPs
VAT_2M	4896.5	2433	0.02685	0	Singlet	Adipocyte
VAT_2M	4608.9	2185	0.0153912	0	Singlet	Adipocyte
VAT_2M	7466.1	2792	0.1352815	0	Singlet	Adipocyte
VAT_2M	1837.6	1144	0.2996991	0	Singlet	Adipocyte
VAT_2M	2769.8	1564	0.2778307	0	Singlet	Adipocyte
VAT_2M	1372.5	1010	4.0153824	2	Singlet	FAPs
VAT_2M	1011.8	832	2.0728044	6	Singlet	Neurons
VAT_2M	1245.7	958	1.1811116	6	Singlet	Neurons
VAT_2M	6311.4	2967	6.8947548	7	Singlet	Endothelial_cells
VAT_2M	8695.2	3217	0.0030221	0	Singlet	Adipocyte
VAT_2M	2691.1	1502	0.478668	0	Singlet	Adipocyte
VAT_2M	1331	920	2.9330966	3	Singlet	FAPs
VAT_2M	1626.4	1335	0.7957051	6	Singlet	Neurons
VAT_2M	1099.8	896	1.4508368	9	Singlet	Mesothelial
VAT_2M	2109	1454	0.7462748	2	Singlet	FAPs
VAT_2M	3447.6	1961	0.0887569	2	Singlet	FAPs
VAT_2M	1763.3	1186	0.044846	0	Singlet	Adipocyte
VAT_2M	2426	1519	0.0296081	0	Singlet	Adipocyte
VAT_2M	1671.4	1262	0.5265101	3	Singlet	FAPs
VAT_2M	14930	4261	0.0087201	0	Singlet	Adipocyte

VAT_2M	1626.5	1156	1.3273055	3	Singlet	FAPs
VAT_2M	4668.5	2226	0.0105511	0	Singlet	Adipocyte
VAT_2M	1458.4	1051	2.4463874	9	Singlet	Mesothelial
VAT_2M	5682.9	2532	0.4244821	0	Singlet	Adipocyte
VAT_2M	3611.1	1941	0.4141661	0	Singlet	Adipocyte
VAT_2M	7081.2	2916	0.0740069	0	Singlet	Adipocyte
VAT_2M	6033.5	2575	0	0	Singlet	Adipocyte
VAT_2M	2033.6	1416	1.5079691	6	Singlet	Neurons
VAT_2M	2228.4	1409	0.1666805	0	Singlet	Adipocyte
VAT_2M	2023	1115	5.121964	7	Singlet	Endothelial_cells
VAT_2M	2397.2	1601	0.1547413	3	Singlet	FAPs
VAT_2M	3577.8	1818	0.6464513	0	Singlet	Adipocyte
VAT_2M	4689.4	2214	0.639164	0	Singlet	Adipocyte
VAT_2M	1009.3	784	0.2261972	3	Singlet	FAPs
VAT_2M	7434.4	2780	0.3030342	0	Singlet	Adipocyte
VAT_2M	1780.2	1210	2.951608	11	Singlet	Smooth_muscle_cells
VAT_2M	1631.4	1204	0.9960444	2	Singlet	FAPs
VAT_2M	6279.9	2529	0.3659669	0	Singlet	Adipocyte
VAT_2M	3262.1	1910	0.9940214	0	Singlet	Adipocyte
VAT_2M	6426.7	2667	0.0537441	0	Singlet	Adipocyte
VAT_2M	3171.5	1634	0.0766904	0	Singlet	Adipocyte
VAT_2M	4960.3	2131	0.0358878	0	Singlet	Adipocyte
VAT_2M	1945.7	1514	0.5867999	11	Singlet	Smooth_muscle_cells
VAT_2M	3598.8	1734	0.0734739	0	Singlet	Adipocyte
VAT_2M	1622.8	1015	0.32874	6	Singlet	Neurons
VAT_2M	2656.1	1473	0.14483	0	Singlet	Adipocyte
VAT_2M	1848.6	1180	0	3	Singlet	FAPs
VAT_2M	1293.5	1031	0.2179016	5	Singlet	Immune_cells
VAT_2M	2066.3	1453	0.3848455	2	Singlet	FAPs
VAT_2M	1336.8	1033	1.2349485	2	Singlet	FAPs
VAT_2M	5606.9	2278	0.0426422	0	Singlet	Adipocyte
VAT_2M	4465.9	2211	0.1035261	0	Singlet	Adipocyte
VAT_2M	2797.6	1747	0.1580621	0	Singlet	Adipocyte
VAT_2M	1050.9	818	0.998636	2	Singlet	FAPs
VAT_2M	3571.6	1919	0.0350677	3	Singlet	FAPs
VAT_2M	1888.3	1669	0.8395492	6	Singlet	Neurons
VAT_2M	2156.4	1415	0.1100523	7	Singlet	Endothelial_cells
VAT_2M	1349.6	1025	2.1451505	2	Singlet	FAPs
VAT_2M	3357.6	1995	1.0585397	2	Singlet	FAPs
VAT_2M	1553.8	1108	0.466199	2	Singlet	FAPs
VAT_2M	1364.9	937	2.0470794	11	Singlet	Smooth_muscle_cells
VAT_2M	8216	3011	0.0996112	0	Singlet	Adipocyte
VAT_2M	1512.6	1064	0.5693928	1	Singlet	Immune_cells
VAT_2M	1397.2	1040	0.599081	3	Singlet	FAPs
VAT_2M	1133.7	1014	2.1085504	6	Singlet	Neurons
VAT_2M	5673.3	2318	0.0106011	0	Singlet	Adipocyte
VAT_2M	4705.1	2354	0.9454697	0	Singlet	Adipocyte
VAT_2M	4620.3	2181	0.1673336	0	Singlet	Adipocyte
VAT_2M	2494.3	1548	0.8367746	2	Singlet	FAPs
VAT_2M	2311.7	1474	1.3420914	6	Singlet	Neurons
VAT_2M	1183.4	947	0.5584326	1	Singlet	Immune_cells

VAT_2M	1001	846	1.4499623	6	Singlet	Neurons
VAT_2M	1022.2	872	1.2486986	1	Singlet	Immune_cells
VAT_2M	1057.4	803	0.5144317	11	Singlet	Smooth_muscle_cells
VAT_2M	3730.8	2246	0.8929296	3	Singlet	FAPs
VAT_2M	2477.1	1442	0.0610763	0	Singlet	Adipocyte
VAT_2M	1010.8	828	0.0394454	6	Singlet	Neurons
VAT_2M	9226.3	3408	0.2238976	0	Singlet	Adipocyte
VAT_2M	2058	1069	10.103686	7	Singlet	Endothelial_cells
VAT_2M	1628.7	1060	0.0954889	0	Singlet	Adipocyte
VAT_2M	2732.2	1543	0.3707364	0	Singlet	Adipocyte
VAT_2M	6481.5	2577	0.0254277	0	Singlet	Adipocyte
VAT_2M	6682	2751	0.018993	0	Singlet	Adipocyte
VAT_2M	4979.9	2400	0.5183426	0	Singlet	Adipocyte
VAT_2M	1274.5	907	3.2670891	5	Singlet	Immune_cells
VAT_2M	1420.8	963	2.8221087	5	Singlet	Immune_cells
VAT_2M	6380.9	2626	0.0527438	0	Singlet	Adipocyte
VAT_2M	2001.9	1203	0.1284881	0	Singlet	Adipocyte
VAT_2M	1959.4	1283	0.5285182	0	Singlet	Adipocyte
VAT_2M	9745.5	3418	0.0101667	0	Singlet	Adipocyte
VAT_2M	1157.5	927	1.7677624	2	Singlet	FAPs
VAT_2M	1528.2	1083	0.9167404	0	Singlet	Adipocyte
VAT_2M	1833.4	1280	0.414341	2	Singlet	FAPs
VAT_2M	7978.2	3116	0.0999392	0	Singlet	Adipocyte
VAT_2M	1708.7	1239	1.6815411	5	Singlet	Immune_cells
VAT_2M	8887.3	3208	0	0	Singlet	Adipocyte
VAT_2M	3361.8	1812	0.7209076	0	Singlet	Adipocyte
VAT_2M	9426	3310	0.0112293	0	Singlet	Adipocyte
VAT_2M	4789.5	2057	0	0	Singlet	Adipocyte
VAT_2M	1872.1	1271	1.6636693	0	Singlet	Adipocyte
VAT_2M	4154.2	1981	0.0179974	0	Singlet	Adipocyte
VAT_2M	3444.4	1945	0.3048729	6	Singlet	Neurons
VAT_2M	2916.8	1736	0.5098953	0	Singlet	Adipocyte
VAT_2M	2239	1478	0.2713571	3	Singlet	FAPs
VAT_2M	5758.1	2571	0.1914564	0	Singlet	Adipocyte
VAT_2M	1582.9	1126	1.4199421	6	Singlet	Neurons
VAT_2M	7522.7	2843	0.0195778	0	Singlet	Adipocyte
VAT_2M	2103.1	1399	0.7743561	5	Singlet	Immune_cells
VAT_2M	2113.7	1296	0.1004236	0	Singlet	Adipocyte
VAT_2M	2446.4	1293	1.4799406	0	Singlet	Adipocyte
VAT_2M	6745.2	2859	0.0459098	0	Singlet	Adipocyte
VAT_2M	1992.3	1421	1.0576672	2	Singlet	FAPs
VAT_2M	1539.8	1051	0.5105561	7	Singlet	Endothelial_cells
VAT_2M	1165.5	918	6.3135677	9	Singlet	Mesothelial
VAT_2M	7854.5	3196	0.1350943	0	Singlet	Adipocyte
VAT_2M	2832.3	1534	7.319712	7	Singlet	Endothelial_cells
VAT_2M	2432.6	1510	0	3	Singlet	FAPs
VAT_2M	8993.7	3230	0.0111487	0	Singlet	Adipocyte
VAT_2M	2726.9	1752	0.5433515	6	Singlet	Neurons
VAT_2M	3190.6	1781	0.1135072	0	Singlet	Adipocyte
VAT_2M	2131.9	1341	0.8016605	0	Singlet	Adipocyte
VAT_2M	5102	2292	0.0857053	0	Singlet	Adipocyte

VAT_2M	1936	1438	2.1579596	5	Singlet	Immune_cells
VAT_2M	2742.4	1502	0	0	Singlet	Adipocyte
VAT_2M	10961	3760	0.0657623	0	Singlet	Adipocyte
VAT_2M	1127.8	819	1.3891115	9	Singlet	Mesothelial
VAT_2M	1826	1225	0.6851594	5	Singlet	Immune_cells
VAT_2M	4651	2294	0.0807263	0	Singlet	Adipocyte
VAT_2M	9658.1	3263	0.0495725	0	Singlet	Adipocyte
VAT_2M	1196.2	967	1.6286504	11	Singlet	Smooth_muscle_cells
VAT_2M	2551.6	1446	0.1183558	0	Singlet	Adipocyte
VAT_2M	7709.4	3033	0.0454729	0	Singlet	Adipocyte
VAT_2M	4228.3	2208	1.2993989	0	Singlet	Adipocyte
VAT_2M	1981.1	1225	0.1419124	2	Singlet	FAPs
VAT_2M	1703.4	1145	0.6258192	3	Singlet	FAPs
VAT_2M	1029.9	802	2.5427699	5	Singlet	Immune_cells
VAT_2M	7828.1	2935	0.1698131	0	Singlet	Adipocyte
VAT_2M	1154.3	836	1.3619452	3	Singlet	FAPs
VAT_2M	1451.5	1027	0.2084551	5	Singlet	Immune_cells
VAT_2M	2116.6	1253	0.3403749	0	Singlet	Adipocyte
VAT_2M	1747.7	1279	1.4246158	6	Singlet	Neurons
VAT_2M	1934.2	1356	0.8452705	2	Singlet	FAPs
VAT_2M	4806.8	2450	0.0649776	0	Singlet	Adipocyte
VAT_2M	2046.8	1383	0.3538349	3	Singlet	FAPs
VAT_2M	2380.7	1586	0.1542309	2	Singlet	FAPs
VAT_2M	2667.7	1698	1.0670172	2	Singlet	FAPs
VAT_2M	1242.3	851	1.6500536	3	Singlet	FAPs
VAT_2M	10681	3541	0.0030816	0	Singlet	Adipocyte
VAT_2M	6756.8	2743	0.1587655	0	Singlet	Adipocyte
VAT_2M	3194.5	1922	0.4107528	6	Singlet	Neurons
VAT_2M	1887	1322	0.2248686	3	Singlet	FAPs
VAT_2M	3037.8	1657	0.0223003	0	Singlet	Adipocyte
VAT_2M	1890.1	1237	1.6440472	7	Singlet	Endothelial_cells
VAT_2M	3577.5	1989	0.7646264	3	Singlet	FAPs
VAT_2M	1195.8	915	3.0407312	2	Singlet	FAPs
VAT_2M	1696.2	1485	0.4029917	6	Singlet	Neurons
VAT_2M	5133.8	2407	0.1609302	0	Singlet	Adipocyte
VAT_2M	4340.2	2185	0.1884526	0	Singlet	Adipocyte
VAT_2M	1722.4	1282	0.1562145	7	Singlet	Endothelial_cells
VAT_2M	1230.5	1043	0.2704481	11	Singlet	Smooth_muscle_cells
VAT_2M	6757	2849	0.0971835	0	Singlet	Adipocyte
VAT_2M	1941.4	1274	1.6762431	0	Singlet	Adipocyte
VAT_2M	1230.7	937	0.0999323	8	Singlet	Mesothelial
VAT_2M	1038.3	768	1.6917016	2	Singlet	FAPs
VAT_2M	4534.3	2081	0.0636315	0	Singlet	Adipocyte
VAT_2M	3678.7	2027	0.1292503	0	Singlet	Adipocyte
VAT_2M	2329.7	1306	0.1740301	0	Singlet	Adipocyte
VAT_2M	1615.7	1170	2.7992173	5	Singlet	Immune_cells
VAT_2M	3487.7	2042	1.4503215	3	Singlet	FAPs
VAT_2M	2532.7	1507	0.106931	11	Singlet	Smooth_muscle_cells
VAT_2M	1805.3	1126	1.2883543	5	Singlet	Immune_cells
VAT_2M	1647.5	1085	1.2798306	6	Singlet	Neurons
VAT_2M	1131.9	922	0.5215231	4	Singlet	Immune_cells

VAT_2M	3030.3	1821	1.2092932	6	Singlet	Neurons
VAT_2M	3573.7	1895	0.6065087	0	Singlet	Adipocyte
VAT_2M	1704.4	1244	1.0952021	6	Singlet	Neurons
VAT_2M	5574.3	2517	0.3453268	0	Singlet	Adipocyte
VAT_2M	2286.4	1454	0.1278293	5	Singlet	Immune_cells
VAT_2M	3841.5	2135	1.3772155	2	Singlet	FAPs
VAT_2M	1208.9	855	3.5895114	3	Singlet	FAPs
VAT_2M	6504.7	2581	0.04244	0	Singlet	Adipocyte
VAT_2M	11402	3508	0.6212298	0	Singlet	Adipocyte
VAT_2M	4092.8	2370	1.2581174	5	Singlet	Immune_cells
VAT_2M	2346.3	1257	0.273052	0	Singlet	Adipocyte
VAT_2M	2069.5	1306	0.2874302	3	Singlet	FAPs
VAT_2M	2044.1	1202	1.074255	0	Singlet	Adipocyte
VAT_2M	1559.3	1146	0.394796	2	Singlet	FAPs
VAT_2M	4165	2143	0.5556369	2	Singlet	FAPs
VAT_2M	2558.1	1483	0.0388846	3	Singlet	FAPs
VAT_2M	7381.4	2708	0.0032508	0	Singlet	Adipocyte
VAT_2M	1596.2	1236	0.1987719	3	Singlet	FAPs
VAT_2M	1497.8	1189	1.1767425	7	Singlet	Endothelial_cells
VAT_2M	6912.6	2712	0.0063653	0	Singlet	Adipocyte
VAT_2M	4498.5	2167	0.0160317	0	Singlet	Adipocyte
VAT_2M	1656.1	1129	2.1481874	2	Singlet	FAPs
VAT_2M	6806.9	2676	0.2854778	0	Singlet	Adipocyte
VAT_2M	7072.6	2728	0.0561914	0	Singlet	Adipocyte
VAT_2M	7008.4	2795	0.0345297	0	Singlet	Adipocyte
VAT_2M	6214.3	2597	0.0701844	0	Singlet	Adipocyte
VAT_2M	6384.8	2825	0.0734666	0	Singlet	Adipocyte
VAT_2M	1055.7	856	0.6791762	2	Singlet	FAPs
VAT_2M	1138.7	908	3.1114279	2	Singlet	FAPs
VAT_2M	1269.8	973	0.560293	4	Singlet	Immune_cells
VAT_2M	5536.8	2422	0.2925321	0	Singlet	Adipocyte
VAT_2M	4496.6	2288	0.1704728	0	Singlet	Adipocyte
VAT_2M	1421.9	1090	0.4339315	2	Singlet	FAPs
VAT_2M	1258.8	974	1.5604263	6	Singlet	Neurons
VAT_2M	2015.4	1367	2.6746369	2	Singlet	FAPs
VAT_2M	5186.6	2337	0.0117776	0	Singlet	Adipocyte
VAT_2M	5496.8	2317	0.240544	0	Singlet	Adipocyte
VAT_2M	1190.8	896	3.5628169	2	Singlet	FAPs
VAT_2M	1674.8	1191	2.1549023	3	Singlet	FAPs
VAT_2M	2240.2	1357	0.6916436	2	Singlet	FAPs
VAT_2M	1851.3	1536	1.4523757	11	Singlet	Smooth_muscle_cells
VAT_2M	3495.5	1840	5.8321501	7	Singlet	Endothelial_cells
VAT_2M	2370.6	1571	0.4706369	3	Singlet	FAPs
VAT_2M	8092.5	2949	0.0009788	0	Singlet	Adipocyte
VAT_2M	2110.9	1433	1.4339796	3	Singlet	FAPs
VAT_2M	5190.7	2347	0.0354728	0	Singlet	Adipocyte
VAT_2M	1011.4	750	0.2825377	7	Singlet	Endothelial_cells
VAT_2M	1038.5	770	2.9846199	2	Singlet	FAPs
VAT_2M	8295.9	2908	0.0261727	0	Singlet	Adipocyte
VAT_2M	1737.9	1335	1.1582755	2	Singlet	FAPs
VAT_2M	2221.5	1403	0.2306013	3	Singlet	FAPs

VAT_2M	7882.8	2948	0.0151251	0	Singlet	Adipocyte
VAT_2M	1650.1	1255	0.2531668	5	Singlet	Immune_cells
VAT_2M	4110.1	2011	0.2881042	0	Singlet	Adipocyte
VAT_2M	4854.4	2702	4.6273142	7	Singlet	Endothelial_cells
VAT_2M	6074	2394	0.0028617	0	Singlet	Adipocyte
VAT_2M	1194.2	914	2.2250216	9	Singlet	Mesothelial
VAT_2M	1412.2	978	1.6473314	2	Singlet	FAPs
VAT_2M	9036	3213	0.0009331	0	Singlet	Adipocyte
VAT_2M	2461.6	1338	0.0310546	0	Singlet	Adipocyte
VAT_2M	4439.3	2203	4.3871106	7	Singlet	Endothelial_cells
VAT_2M	6873.7	2599	0.0235127	0	Singlet	Adipocyte
VAT_2M	2380.3	1456	1.3143345	6	Singlet	Neurons
VAT_2M	5686.9	2428	0.0130717	0	Singlet	Adipocyte
VAT_2M	6942.4	2662	0.0346596	0	Singlet	Adipocyte
VAT_2M	3348.8	1717	0.0878484	0	Singlet	Adipocyte
VAT_2M	5110.4	2349	0.0116839	0	Singlet	Adipocyte
VAT_2M	7283.1	2964	0.0022549	0	Singlet	Adipocyte
VAT_2M	7363.3	2898	0	0	Singlet	Adipocyte
VAT_2M	3460	1917	0.3609875	0	Singlet	Adipocyte
VAT_2M	1460.9	1092	1.8604638	2	Singlet	FAPs
VAT_2M	4538.8	2255	0.2485592	0	Singlet	Adipocyte
VAT_2M	1435.3	1059	0.6505681	3	Singlet	FAPs
VAT_2M	1624.2	1093	0.0071945	5	Singlet	Immune_cells
VAT_2M	2155.9	1356	1.6183297	6	Singlet	Neurons
VAT_2M	1909.3	1447	0.9539504	5	Singlet	Immune_cells
VAT_2M	5255.7	2276	0.0631979	0	Singlet	Adipocyte
VAT_2M	2138.1	1314	0.3687193	6	Singlet	Neurons
VAT_2M	6989.9	3131	0.0343561	0	Singlet	Adipocyte
VAT_2M	1680.7	1064	0.3109797	0	Singlet	Adipocyte
VAT_2M	3235	1737	0.2683505	0	Singlet	Adipocyte
VAT_2M	5981.1	2535	0.0423203	0	Singlet	Adipocyte
VAT_2M	1363.4	1018	0.1972757	2	Singlet	FAPs
VAT_2M	4927	2719	1.2915132	3	Singlet	FAPs
VAT_2M	1485	1092	0.5519062	5	Singlet	Immune_cells
VAT_2M	4901.5	2458	0.1051598	0	Singlet	Adipocyte
VAT_2M	3336.1	1721	0.0778973	0	Singlet	Adipocyte
VAT_2M	2259.5	1391	1.2889154	5	Singlet	Immune_cells
VAT_2M	2465.9	1353	0.1318918	0	Singlet	Adipocyte
VAT_2M	3484.2	1853	0.4437979	0	Singlet	Adipocyte
VAT_2M	1836.6	1223	2.0868962	2	Singlet	FAPs
VAT_2M	1848.5	1128	0.3870929	0	Singlet	Adipocyte
VAT_2M	5059.9	2307	0	0	Singlet	Adipocyte
VAT_2M	1256.4	1030	0.9732221	7	Singlet	Endothelial_cells
VAT_2M	7332.6	2915	0.018208	0	Singlet	Adipocyte
VAT_2M	1954	1453	0.5856119	2	Singlet	FAPs
VAT_2M	1488.4	1046	2.6559321	3	Singlet	FAPs
VAT_2M	1212.4	907	0.2321095	3	Singlet	FAPs
VAT_2M	2072.1	1304	0.1522661	0	Singlet	Adipocyte
VAT_2M	1862.3	1168	0.1717101	0	Singlet	Adipocyte
VAT_2M	4729.9	2052	0.0258494	0	Singlet	Adipocyte
VAT_2M	2256.6	1473	0.0536279	0	Singlet	Adipocyte

VAT_2M	8553.3	3045	0.0597969	0	Singlet	Adipocyte
VAT_2M	5031.1	2314	0.1879328	0	Singlet	Adipocyte
VAT_2M	3037.2	1724	0.5234841	0	Singlet	Adipocyte
VAT_2M	1952.8	1158	0.8706193	3	Singlet	FAPs
VAT_2M	1312.4	839	4.142067	5	Singlet	Immune_cells
VAT_2M	4972.7	2319	0.078327	0	Singlet	Adipocyte
VAT_2M	2399	1492	0.7919573	6	Singlet	Neurons
VAT_2M	1001	746	2.5093281	9	Singlet	Mesothelial
VAT_2M	1261.7	875	1.2526057	7	Singlet	Endothelial_cells
VAT_2M	2426.8	1316	0.7811734	0	Singlet	Adipocyte
VAT_2M	8748.7	3270	0.4336177	0	Singlet	Adipocyte
VAT_2M	4489.8	2207	0.0806735	0	Singlet	Adipocyte
VAT_2M	2499.9	1520	0.0117579	11	Singlet	Smooth_muscle_cells
VAT_2M	1606.9	1265	0.289925	3	Singlet	FAPs
VAT_2M	1930.4	1452	1.55134	2	Singlet	FAPs
VAT_2M	1004.5	791	1.8761814	6	Singlet	Neurons
VAT_2M	1052.7	877	3.424034	9	Singlet	Mesothelial
VAT_2M	1230.5	976	2.1602575	11	Singlet	Smooth_muscle_cells
VAT_2M	2159.5	1247	3.3515996	9	Singlet	Mesothelial
VAT_2M	8128.2	3031	0.0749703	0	Singlet	Adipocyte
VAT_2M	1903.1	1292	0.7463862	2	Singlet	FAPs
VAT_2M	1575.7	987	2.5804959	2	Singlet	FAPs
VAT_2M	2379.7	1504	3.9314149	5	Singlet	Immune_cells
VAT_2M	1457.3	1244	0.0245521	6	Singlet	Neurons
VAT_2M	2466.4	1392	0.6604278	0	Singlet	Adipocyte
VAT_2M	1037.5	713	5.7927643	3	Singlet	FAPs
VAT_2M	2017	1340	2.2063557	2	Singlet	FAPs
VAT_2M	4316.4	2024	0.1777639	0	Singlet	Adipocyte
VAT_2M	1415.5	1011	0.2897958	7	Singlet	Endothelial_cells
VAT_2M	1042.1	776	0.965018	2	Singlet	FAPs
VAT_2M	1200	910	1.791708	3	Singlet	FAPs
VAT_2M	2552.4	1663	0.8044632	2	Singlet	FAPs
VAT_2M	6254.6	2569	0.064376	0	Singlet	Adipocyte
VAT_2M	1245.3	944	0.5656408	9	Singlet	Mesothelial
VAT_2M	2902.4	1533	0.8774257	0	Singlet	Adipocyte
VAT_2M	1098.7	877	0.87539	8	Singlet	Mesothelial
VAT_2M	1868.3	1189	0.1243713	0	Singlet	Adipocyte
VAT_2M	2479.9	1342	0.4299238	0	Singlet	Adipocyte
VAT_2M	6556.3	2611	0.0430189	0	Singlet	Adipocyte
VAT_2M	2892.7	1738	0.5327402	0	Singlet	Adipocyte
VAT_2M	2506.2	1401	0.9070704	0	Singlet	Adipocyte
VAT_2M	2646.9	1451	0.0539401	0	Singlet	Adipocyte
VAT_2M	2087.7	1429	4.5804148	7	Singlet	Endothelial_cells
VAT_2M	1416.9	958	1.9709639	2	Singlet	FAPs
VAT_2M	1430.6	1063	2.4197913	7	Singlet	Endothelial_cells
VAT_2M	1250.3	1014	0.7709851	6	Singlet	Neurons
VAT_2M	3733.4	2048	1.1610123	3	Singlet	FAPs
VAT_2M	5020.9	2107	0.0615973	0	Singlet	Adipocyte
VAT_2M	4251.1	1920	0.3606906	0	Singlet	Adipocyte
VAT_2M	4969.4	2112	0.0535661	0	Singlet	Adipocyte
VAT_2M	1732.8	1234	0.5213627	7	Singlet	Endothelial_cells

VAT_2M	1598.8	1119	0.0742568	1	Singlet	Immune_cells
VAT_2M	2910.2	1653	0.0877491	3	Singlet	FAPs
VAT_2M	8401.7	3025	0.2193644	0	Singlet	Adipocyte
VAT_2M	1175.8	988	0.6784974	4	Singlet	Immune_cells
VAT_2M	1299.2	922	0.0065853	8	Singlet	Mesothelial
VAT_2M	3232.8	1557	0.0628233	0	Singlet	Adipocyte
VAT_2M	3255.5	1846	1.5166373	5	Singlet	Immune_cells
VAT_2M	4751.4	2531	1.0314349	3	Singlet	FAPs
VAT_2M	1124.8	846	0.9436903	6	Singlet	Neurons
VAT_2M	1318.3	967	0.337637	1	Singlet	Immune_cells
VAT_2M	1362.9	999	0.1708707	2	Singlet	FAPs
VAT_2M	1675.5	869	2.6054665	5	Singlet	Immune_cells
VAT_2M	2151.1	1182	2.6294574	1	Singlet	Immune_cells
VAT_2M	1880.6	1144	0.4514278	6	Singlet	Neurons
VAT_2M	8485.3	2730	0.0731389	0	Singlet	Adipocyte
VAT_2M	1121.8	850	1.767428	2	Singlet	FAPs
VAT_2M	2760.3	1443	0.3257088	6	Singlet	Neurons
VAT_2M	1666.2	1002	0.8198007	3	Singlet	FAPs
VAT_2M	5253.5	2174	0.0566288	0	Singlet	Adipocyte
VAT_2M	1151.8	1026	0.6836566	1	Singlet	Immune_cells
VAT_2M	7541.6	2792	0.0126917	0	Singlet	Adipocyte
VAT_2M	1705.2	982	4.4565634	7	Singlet	Endothelial_cells
VAT_2M	2457.1	1489	1.4720097	0	Singlet	Adipocyte
VAT_2M	10678	3493	0.2996516	0	Singlet	Adipocyte
VAT_2M	1127.8	848	0.8794229	5	Singlet	Immune_cells
VAT_2M	1774.4	1042	1.1545957	0	Singlet	Adipocyte
VAT_2M	4398.4	1752	0.0127845	0	Singlet	Adipocyte
VAT_2M	3892.3	1909	0	0	Singlet	Adipocyte
VAT_2M	6159.3	2403	0.0683421	0	Singlet	Adipocyte
VAT_2M	1895	1464	1.9128307	11	Singlet	Smooth_muscle_cells
VAT_2M	1051.2	803	3.3816538	2	Singlet	FAPs
VAT_2M	5363.1	2362	0.043318	0	Singlet	Adipocyte
VAT_2M	1174.3	872	2.0239133	2	Singlet	FAPs
VAT_2M	6005	2372	0.0153275	0	Singlet	Adipocyte
VAT_2M	1781.8	1337	2.0716401	5	Singlet	Immune_cells
VAT_2M	7983.2	2576	0.3088168	0	Singlet	Adipocyte
VAT_2M	2725.1	1483	0.4100049	0	Singlet	Adipocyte
VAT_2M	3132.8	1862	0.4322336	0	Singlet	Adipocyte
VAT_2M	5530.3	2474	0.9915981	0	Singlet	Adipocyte
VAT_2M	4508.3	2087	0.096942	0	Singlet	Adipocyte
VAT_2M	1634.9	1217	0.9005947	2	Singlet	FAPs
VAT_2M	2036.6	1457	1.1708534	5	Singlet	Immune_cells
VAT_2M	2036.8	1058	0.1273401	0	Singlet	Adipocyte
VAT_2M	6241.6	2246	0	0	Singlet	Adipocyte
VAT_2M	1099.3	802	0.4632147	5	Singlet	Immune_cells
VAT_2M	5743.5	2189	0.2199099	0	Singlet	Adipocyte
VAT_2M	1053.9	881	2.9005549	1	Singlet	Immune_cells
VAT_2M	3230.8	2027	1.5236396	5	Singlet	Immune_cells
VAT_2M	3301.6	1791	1.100583	0	Singlet	Adipocyte
VAT_2M	5662.1	2596	0.1507772	0	Singlet	Adipocyte
VAT_2M	5788.8	2625	0.4294816	0	Singlet	Adipocyte

VAT_2M	1837.7	1221	0.1144314	0 Singlet	Adipocyte
VAT_2M	1611	965	2.9570262	2 Singlet	FAPs
VAT_2M	1580.8	1021	1.6591634	4 Singlet	Immune_cells
VAT_2M	1587.1	1061	0.768734	7 Singlet	Endothelial_cells
VAT_2M	1641.9	1337	0.8813274	8 Singlet	Mesothelial
VAT_2M	6661.8	2889	0.0386046	0 Singlet	Adipocyte
VAT_2M	10388	3475	0.8190863	0 Singlet	Adipocyte
VAT_2M	1686.7	1107	1.2487525	7 Singlet	Endothelial_cells
VAT_2M	1189.1	862	3.8150192	3 Singlet	FAPs
VAT_2M	1671.7	1199	2.8835949	2 Singlet	FAPs
VAT_2M	10899	3351	0.1047699	0 Singlet	Adipocyte
VAT_2M	6947.2	2780	0.0495679	0 Singlet	Adipocyte
VAT_2M	3335.4	1762	0	0 Singlet	Adipocyte
VAT_2M	8825.7	3271	0.2003287	0 Singlet	Adipocyte
VAT_2M	3705.1	1865	0	0 Singlet	Adipocyte
VAT_2M	1018.4	812	1.9443402	3 Singlet	FAPs
VAT_2M	1379.9	886	1.1604241	3 Singlet	FAPs
VAT_2M	2705.8	1520	0.3153734	0 Singlet	Adipocyte
VAT_2M	4111.7	2214	2.1149346	3 Singlet	FAPs
VAT_2M	9736	3044	0.03079	0 Singlet	Adipocyte
VAT_2M	8780.7	2923	0.0907749	0 Singlet	Adipocyte
VAT_2M	1575.5	914	0.5246824	0 Singlet	Adipocyte
VAT_2M	1082	901	2.2925242	5 Singlet	Immune_cells
VAT_2M	1745.8	1078	1.7375486	6 Singlet	Neurons
VAT_2M	8912.5	3246	0.0198306	0 Singlet	Adipocyte
VAT_2M	1691.7	1025	3.2880546	3 Singlet	FAPs
VAT_2M	6935.3	2605	0.1534936	0 Singlet	Adipocyte
VAT_2M	2155.5	1335	0	0 Singlet	Adipocyte
VAT_2M	3314	1801	0.3650283	0 Singlet	Adipocyte
VAT_2M	3359.9	1904	1.0876393	5 Singlet	Immune_cells
VAT_2M	1106	819	0.1761009	0 Singlet	Adipocyte
VAT_2M	1238.8	913	9.4791335	2 Singlet	FAPs
VAT_2M	8986	3227	0.8186581	0 Singlet	Adipocyte
VAT_2M	3054.7	1903	0.4003116	3 Singlet	FAPs
VAT_2M	2019	1502	0.3516182	3 Singlet	FAPs
VAT_2M	1315.7	997	1.3911168	2 Singlet	FAPs
VAT_2M	1264.7	858	1.9400479	6 Singlet	Neurons
VAT_2M	2637.3	1546	0.0712948	2 Singlet	FAPs
VAT_2M	1367.7	1040	0.7528934	7 Singlet	Endothelial_cells
VAT_2M	1123.7	807	0.3007926	3 Singlet	FAPs
VAT_2M	3192.8	1463	0.1186674	0 Singlet	Adipocyte
VAT_2M	1418.3	1101	1.4106934	2 Singlet	FAPs
VAT_2M	2523.1	1514	0.2954606	6 Singlet	Neurons
VAT_2M	2277.4	1223	0.7396959	3 Singlet	FAPs
VAT_2M	1618.5	1080	0.2472491	3 Singlet	FAPs
VAT_2M	10460	3074	0.1507955	0 Singlet	Adipocyte
VAT_2M	1847.1	1135	0	0 Singlet	Adipocyte
VAT_2M	11815	3617	0.0053535	0 Singlet	Adipocyte
VAT_2M	5563.8	2413	0.1008737	0 Singlet	Adipocyte
VAT_2M	1241.6	1021	3.4702721	6 Singlet	Neurons
VAT_2M	5819.3	2373	0.0056318	0 Singlet	Adipocyte

VAT_2M	2095.7	1144	0.3725805	0	Singlet	Adipocyte
VAT_2M	1195	907	2.6318606	7	Singlet	Endothelial_cells
VAT_2M	1695	1109	0.0469933	6	Singlet	Neurons
VAT_2M	2381.9	1461	1.9573867	3	Singlet	FAPs
VAT_2M	3707.1	2018	0.7135211	0	Singlet	Adipocyte
VAT_2M	1870.1	1183	5.8763059	7	Singlet	Endothelial_cells
VAT_2M	2144	1481	0.7225298	5	Singlet	Immune_cells
VAT_2M	3919	1892	0.3474942	0	Singlet	Adipocyte
VAT_2M	5098.9	2356	0.0562631	0	Singlet	Adipocyte
VAT_2M	4639.2	2230	0.1388868	0	Singlet	Adipocyte
VAT_2M	1705.4	1279	0.7260264	2	Singlet	FAPs
VAT_2M	1076.7	855	1.9891299	6	Singlet	Neurons
VAT_2M	1177.6	1009	0.249349	6	Singlet	Neurons
VAT_2M	1913.2	1222	0.5416703	3	Singlet	FAPs
VAT_2M	4857.6	2242	0.0165503	0	Singlet	Adipocyte
VAT_2M	9309.4	3115	0.0205311	0	Singlet	Adipocyte
VAT_2M	2907.8	1356	0.1801904	0	Singlet	Adipocyte
VAT_2M	1588.4	978	0.1570035	0	Singlet	Adipocyte
VAT_2M	3435.3	1834	0	0	Singlet	Adipocyte
VAT_2M	3710.7	1981	0	0	Singlet	Adipocyte
VAT_2M	1419.5	1050	0.7336414	2	Singlet	FAPs
VAT_2M	1239.3	890	1.2493923	6	Singlet	Neurons
VAT_2M	5690.1	2365	0.0518607	0	Singlet	Adipocyte
VAT_2M	2609.1	1769	2.9492242	2	Singlet	FAPs
VAT_2M	1379	969	1.6023974	3	Singlet	FAPs
VAT_2M	1520.9	788	1.2407748	8	Singlet	Mesothelial
VAT_2M	6482.1	2750	0.0056383	0	Singlet	Adipocyte
VAT_2M	1436.5	1228	0.7898287	6	Singlet	Neurons
VAT_2M	5512.2	2390	0	0	Singlet	Adipocyte
VAT_2M	3816.2	1967	0.4454659	0	Singlet	Adipocyte
VAT_2M	1314.7	985	3.2176511	9	Singlet	Mesothelial
VAT_2M	1789.3	1352	1.5673122	2	Singlet	FAPs
VAT_2M	1661.7	1083	0.0519849	2	Singlet	FAPs
VAT_2M	1087.6	844	1.2138172	8	Singlet	Mesothelial
VAT_2M	9763.5	3363	0.2199218	0	Singlet	Adipocyte
VAT_2M	2131.3	1299	0.2861421	3	Singlet	FAPs
VAT_2M	2520.2	1514	0.0005529	0	Singlet	Adipocyte
VAT_2M	1113.5	989	0.0295768	9	Singlet	Mesothelial
VAT_2M	1072.5	929	0.8402519	6	Singlet	Neurons
VAT_2M	8808.4	3142	0	0	Singlet	Adipocyte
VAT_2M	1470.5	1181	0.634999	2	Singlet	FAPs
VAT_2M	1167.5	883	0.255393	2	Singlet	FAPs
VAT_2M	7390.9	2775	0.0189244	0	Singlet	Adipocyte
VAT_2M	4076.8	1992	0.0827843	0	Singlet	Adipocyte
VAT_2M	7295	3075	0.9355424	0	Singlet	Adipocyte
VAT_2M	1048.8	860	1.9216055	6	Singlet	Neurons
VAT_2M	6497.5	2781	0.0067477	0	Singlet	Adipocyte
VAT_2M	1101.6	873	9.1272889	6	Singlet	Neurons
VAT_2M	6291	2624	0.3320187	0	Singlet	Adipocyte
VAT_2M	1005.2	811	0.8020239	3	Singlet	FAPs
VAT_2M	6834.7	2753	0.0212568	0	Singlet	Adipocyte

VAT_2M	3322.6	1718	0.1214017	3	Singlet	FAPs
VAT_2M	1330.6	1103	2.1720251	8	Singlet	Mesothelial
VAT_2M	5152	2178	0.0546655	0	Singlet	Adipocyte
VAT_2M	3212.1	1681	0.1158697	0	Singlet	Adipocyte
VAT_2M	7069.3	2880	0.0319458	0	Singlet	Adipocyte
VAT_2M	2181	1493	1.0213906	3	Singlet	FAPs
VAT_2M	8224.5	3009	1.2829647	0	Singlet	Adipocyte
VAT_2M	1634.6	997	1.0360032	0	Singlet	Adipocyte
VAT_2M	6931.7	2967	0.0251021	0	Singlet	Adipocyte
VAT_2M	1029.8	827	0.0809997	6	Singlet	Neurons
VAT_2M	2129.4	1371	0.2225752	0	Singlet	Adipocyte
VAT_2M	3283	1867	0.7803602	0	Singlet	Adipocyte
VAT_2M	2308.9	1467	0.9680534	2	Singlet	FAPs
VAT_2M	6684.5	2739	0.0204571	0	Singlet	Adipocyte
VAT_2M	4606.9	2047	0.1387461	0	Singlet	Adipocyte
VAT_2M	5119.8	2566	0.4352657	2	Singlet	FAPs
VAT_2M	1934.9	1014	1.010065	2	Singlet	FAPs
VAT_2M	2418.1	1626	0.6027459	2	Singlet	FAPs
VAT_2M	1706.5	1257	0.0496761	3	Singlet	FAPs
VAT_2M	7442.3	2911	0.0300076	0	Singlet	Adipocyte
VAT_2M	2183.2	1371	0.5751216	3	Singlet	FAPs
VAT_2M	9811.2	3305	0.0174364	0	Singlet	Adipocyte
VAT_2M	1410.8	1100	0.8137326	9	Singlet	Mesothelial
VAT_2M	5945.3	2554	0.1858799	0	Singlet	Adipocyte
VAT_2M	1224	882	1.0996094	11	Singlet	Smooth_muscle_cells
VAT_2M	1257	927	1.0777965	2	Singlet	FAPs
VAT_2M	1536.7	1107	1.3214668	0	Singlet	Adipocyte
VAT_2M	1855.9	1271	2.7403926	6	Singlet	Neurons
VAT_2M	1944.1	1146	0.6451005	0	Singlet	Adipocyte
VAT_2M	1943.1	1301	0.2377234	0	Singlet	Adipocyte
VAT_2M	9905	3165	0.3053702	0	Singlet	Adipocyte
VAT_2M	3510.6	1809	1.9339207	0	Singlet	Adipocyte
VAT_2M	8454.2	2997	0	0	Singlet	Adipocyte
VAT_2M	8398.4	2963	0	0	Singlet	Adipocyte
VAT_2M	1661.3	1250	1.3649197	2	Singlet	FAPs
VAT_2M	2795.1	1670	2.0409544	0	Singlet	Adipocyte
VAT_2M	4921	2310	0.1815273	0	Singlet	Adipocyte
VAT_2M	2837.4	1543	0.4912099	0	Singlet	Adipocyte
VAT_2M	6847.9	2596	0	0	Singlet	Adipocyte
VAT_2M	1197.3	863	2.3981097	2	Singlet	FAPs
VAT_2M	8350.2	3055	0.0159665	0	Singlet	Adipocyte
VAT_2M	2944.5	1842	0.3944598	5	Singlet	Immune_cells
VAT_2M	6869.1	2689	0.0902845	0	Singlet	Adipocyte
VAT_2M	10212	3245	0.0562603	0	Singlet	Adipocyte
VAT_2M	3129.4	1723	4.1572339	5	Singlet	Immune_cells
VAT_2M	1622.3	1066	0.9821745	2	Singlet	FAPs
VAT_2M	4230.3	2070	0.273436	0	Singlet	Adipocyte
VAT_2M	7586.5	2843	0.6676582	0	Singlet	Adipocyte
VAT_2M	1005.8	779	0.8567307	6	Singlet	Neurons
VAT_2M	5907.7	2488	0.0164794	0	Singlet	Adipocyte
VAT_2M	2733.6	1519	0.1445807	0	Singlet	Adipocyte

VAT_2M	2654.2	1577	0.984935	5	Singlet	Immune_cells
VAT_2M	1646.3	1292	0.4309182	11	Singlet	Smooth_muscle_cells
VAT_2M	1419.5	876	1.005272	2	Singlet	FAPs
VAT_2M	7482.2	2791	0.0016907	0	Singlet	Adipocyte
VAT_2M	1799	1141	0.7664301	5	Singlet	Immune_cells
VAT_2M	2153.1	1341	0.0231583	2	Singlet	FAPs
VAT_2M	2764.6	1658	0.7904787	2	Singlet	FAPs
VAT_2M	1535.5	1156	0.4525931	2	Singlet	FAPs
VAT_2M	1689	1063	4.1661133	5	Singlet	Immune_cells
VAT_2M	3760.5	2178	1.4907745	2	Singlet	FAPs
VAT_2M	2012.3	1297	0.4670737	6	Singlet	Neurons
VAT_2M	4839.8	2203	0.169361	0	Singlet	Adipocyte
VAT_2M	3849.4	2078	1.0983201	3	Singlet	FAPs
VAT_2M	3294.8	1703	0.579442	0	Singlet	Adipocyte
VAT_2M	2729.1	1554	0.9092893	2	Singlet	FAPs
VAT_2M	2435.6	1369	0.7914142	0	Singlet	Adipocyte
VAT_2M	7653.7	2926	0.0424123	0	Singlet	Adipocyte
VAT_2M	6766.1	2621	0.0363042	0	Singlet	Adipocyte
VAT_2M	10993	3557	0.0021438	0	Singlet	Adipocyte
VAT_2M	2065.2	1196	0.5334985	0	Singlet	Adipocyte
VAT_2M	1027.4	879	0.4984862	8	Singlet	Mesothelial
VAT_2M	6116.8	2620	0.8824458	0	Singlet	Adipocyte
VAT_2M	1191.5	956	0.8825711	6	Singlet	Neurons
VAT_2M	8313.7	2996	1.6878875	0	Singlet	Adipocyte
VAT_2M	1396.4	971	1.1511255	3	Singlet	FAPs
VAT_2M	1045.7	783	3.2423797	7	Singlet	Endothelial_cells
VAT_2M	4054.8	1915	0	0	Singlet	Adipocyte
VAT_2M	8327.5	2950	0.0178511	0	Singlet	Adipocyte
VAT_2M	7075.4	2878	1.1964151	0	Singlet	Adipocyte
VAT_2M	1344.3	1160	0.5316906	6	Singlet	Neurons
VAT_2M	3723.4	1990	0.2130114	0	Singlet	Adipocyte
VAT_2M	2115.8	1337	0.9037585	6	Singlet	Neurons
VAT_2M	7093.5	2794	0.0962285	0	Singlet	Adipocyte
VAT_2M	8222.9	3077	0.1528078	0	Singlet	Adipocyte
VAT_2M	1381.1	1046	2.3966638	2	Singlet	FAPs
VAT_2M	3906.7	1891	0.1000472	0	Singlet	Adipocyte
VAT_2M	1361.2	1044	1.2483744	7	Singlet	Endothelial_cells
VAT_2M	7983.2	3112	0.1928297	0	Singlet	Adipocyte
VAT_2M	1143.2	839	2.127726	2	Singlet	FAPs
VAT_2M	2849.5	1791	1.2226067	0	Singlet	Adipocyte
VAT_2M	1087.9	796	1.3134004	0	Singlet	Adipocyte
VAT_2M	1756.6	1166	0.1752693	6	Singlet	Neurons
VAT_2M	1736.3	1159	0.0248443	0	Singlet	Adipocyte
VAT_2M	2574.8	1554	1.9854969	5	Singlet	Immune_cells
VAT_2M	4734.4	2216	0.007566	0	Singlet	Adipocyte
VAT_2M	3492.3	1984	0.319496	0	Singlet	Adipocyte
VAT_2M	4949.6	2194	0.0486869	0	Singlet	Adipocyte
VAT_2M	1474	1121	0.4885105	5	Singlet	Immune_cells
VAT_2M	3648.5	2185	1.2451509	3	Singlet	FAPs
VAT_2M	1177.6	1001	2.7606486	6	Singlet	Neurons
VAT_2M	7653.1	2651	0.0291517	0	Singlet	Adipocyte

VAT_2M	1120.4	865	1.0715156	2	Singlet	FAPs
VAT_2M	7511.6	2887	0.1280356	0	Singlet	Adipocyte
VAT_2M	4521.4	2160	0.0136424	0	Singlet	Adipocyte
VAT_2M	3896.3	1965	0.0614095	0	Singlet	Adipocyte
VAT_2M	8613.1	3251	0.0259046	0	Singlet	Adipocyte
VAT_2M	2257.4	1151	5.9578359	0	Singlet	Adipocyte
VAT_2M	1571.3	1100	0.2938562	3	Singlet	FAPs
VAT_2M	1721.8	1246	1.4774333	11	Singlet	Smooth_muscle_cells
VAT_2M	1282.2	930	2.0064375	9	Singlet	Mesothelial
VAT_2M	2336.6	1329	0.0307061	5	Singlet	Immune_cells
VAT_2M	2838.3	1725	1.0039133	2	Singlet	FAPs
VAT_2M	1410.2	1074	0.1669008	5	Singlet	Immune_cells
VAT_2M	1558.2	1277	0.7752441	11	Singlet	Smooth_muscle_cells
VAT_2M	3598	1841	0.770888	0	Singlet	Adipocyte
VAT_2M	1462	1118	0.9685209	5	Singlet	Immune_cells
VAT_2M	10157	3345	0.0083146	0	Singlet	Adipocyte
VAT_2M	1320	962	1.7446721	3	Singlet	FAPs
VAT_2M	5368.2	2293	0.0734865	0	Singlet	Adipocyte
VAT_2M	1861.6	1179	0.2773528	0	Singlet	Adipocyte
VAT_2M	1318.4	1035	1.9830803	2	Singlet	FAPs
VAT_2M	3220.8	1750	0.3206414	0	Singlet	Adipocyte
VAT_2M	2629.3	1616	0.3032218	3	Singlet	FAPs
VAT_2M	7013	2915	0.0374818	0	Singlet	Adipocyte
VAT_2M	1599.6	1033	3.7267254	2	Singlet	FAPs
VAT_2M	8168.4	3052	0.0267504	0	Singlet	Adipocyte
VAT_2M	3621	1836	0.1635647	0	Singlet	Adipocyte
VAT_2M	1075.1	763	2.209585	1	Singlet	Immune_cells
VAT_2M	1984.4	1355	0.6061654	2	Singlet	FAPs
VAT_2M	3372.9	1811	0.1927785	0	Singlet	Adipocyte
VAT_2M	5477.7	2348	0.092208	0	Singlet	Adipocyte
VAT_2M	3743.2	1999	0.4151594	0	Singlet	Adipocyte
VAT_2M	1707.9	1150	0.3488173	2	Singlet	FAPs
VAT_2M	6161.9	2643	0.0525617	0	Singlet	Adipocyte
VAT_2M	1199.2	833	2.6151599	5	Singlet	Immune_cells
VAT_2M	5792.5	2536	0.0658786	0	Singlet	Adipocyte
VAT_2M	6495.7	2634	0.0289112	0	Singlet	Adipocyte
VAT_2M	5457.7	2429	0.2262733	0	Singlet	Adipocyte
VAT_2M	2699.2	1517	1.3461106	0	Singlet	Adipocyte
VAT_2M	4424	2205	0	0	Singlet	Adipocyte
VAT_2M	4387	2125	0.7506244	0	Singlet	Adipocyte
VAT_2M	1317.2	920	3.670436	2	Singlet	FAPs
VAT_2M	5101.7	2455	0.009396	0	Singlet	Adipocyte
VAT_2M	4711.8	2299	1.3628604	0	Singlet	Adipocyte
VAT_2M	2640.1	1634	0.845612	6	Singlet	Neurons
VAT_2M	1095.9	837	0.8376149	2	Singlet	FAPs
VAT_2M	1629.5	1281	1.7251336	6	Singlet	Neurons
VAT_2M	1617.9	1183	0.2514286	5	Singlet	Immune_cells
VAT_2M	8573.9	3003	0.1067564	0	Singlet	Adipocyte
VAT_2M	3955.5	1939	0.0560353	0	Singlet	Adipocyte
VAT_2M	3409.2	1851	0	0	Singlet	Adipocyte
VAT_2M	4025.9	2010	1.2871408	0	Singlet	Adipocyte

VAT_2M	5188	2286	0.1099806	0 Singlet	Adipocyte
VAT_2M	1996.4	1404	0.5716032	7 Singlet	Endothelial_cells
VAT_2M	1795	1190	0.5221054	0 Singlet	Adipocyte
VAT_2M	3967.7	2095	1.5733819	0 Singlet	Adipocyte
VAT_2M	2669	1576	1.4074521	2 Singlet	FAPs
VAT_2M	5305.9	2229	6.5491548	0 Singlet	Adipocyte
VAT_2M	4921.8	2222	0.0303707	0 Singlet	Adipocyte
VAT_2M	1465	1128	0.7420039	2 Singlet	FAPs
VAT_2M	3398.1	1722	0.0488715	0 Singlet	Adipocyte
VAT_2M	5345.1	2336	0.0791337	0 Singlet	Adipocyte
VAT_2M	1338.5	886	0.5379893	3 Singlet	FAPs
VAT_2M	1775.9	1080	0.0926143	0 Singlet	Adipocyte
VAT_2M	7306.6	2936	0.0749423	0 Singlet	Adipocyte
VAT_2M	2141.6	1343	0.1905378	0 Singlet	Adipocyte
VAT_2M	1015.9	807	0.9110438	3 Singlet	FAPs
VAT_2M	2070.2	1281	1.3221413	3 Singlet	FAPs
VAT_2M	7487.9	2986	0.224679	0 Singlet	Adipocyte
VAT_2M	8137.6	2860	0	0 Singlet	Adipocyte
VAT_2M	3604.5	1885	0.2697702	0 Singlet	Adipocyte
VAT_2M	4058.9	2047	0.0111003	0 Singlet	Adipocyte
VAT_2M	2324.4	1398	0	0 Singlet	Adipocyte
VAT_2M	3401.3	1738	0.2251586	0 Singlet	Adipocyte
VAT_2M	1142	975	0.080672	6 Singlet	Neurons
VAT_2M	6691.9	2698	0.0036604	0 Singlet	Adipocyte
VAT_2M	3175.7	1700	0.354695	0 Singlet	Adipocyte
VAT_2M	1653.8	1168	0.9196756	3 Singlet	FAPs
VAT_2M	8339	3093	0.0005641	0 Singlet	Adipocyte
VAT_2M	4706.7	2091	0.0501018	0 Singlet	Adipocyte
VAT_2M	6845.7	2636	0.1247633	0 Singlet	Adipocyte
VAT_2M	1411.3	818	12.618866	0 Singlet	Adipocyte
VAT_2M	2078.9	1380	0.1615145	3 Singlet	FAPs
VAT_2M	1215	736	0.6938964	7 Singlet	Endothelial_cells
VAT_2M	1082.5	934	1.5415357	6 Singlet	Neurons
VAT_2M	2657.2	1551	0.3862554	3 Singlet	FAPs
VAT_2M	4071.7	2081	0.035569	0 Singlet	Adipocyte
VAT_2M	1070.4	771	3.3133038	3 Singlet	FAPs
VAT_2M	1647.7	1052	0.0758338	0 Singlet	Adipocyte
VAT_2M	5021.3	2231	0.0772498	0 Singlet	Adipocyte
VAT_2M	5193.1	2254	0.0920802	0 Singlet	Adipocyte
VAT_2M	5433.1	2412	0.3649809	0 Singlet	Adipocyte
VAT_2M	1466.2	977	0.7724997	5 Singlet	Immune_cells
VAT_2M	1828.9	1238	0.5807539	2 Singlet	FAPs
VAT_2M	2174.6	1269	1.1525597	0 Singlet	Adipocyte
VAT_2M	2923.3	1567	0.0799599	0 Singlet	Adipocyte
VAT_2M	1306.6	1029	0.6595611	4 Singlet	Immune_cells
VAT_2M	4462.4	2437	0.3015212	0 Singlet	Adipocyte
VAT_2M	2222.4	1339	0.067699	0 Singlet	Adipocyte
VAT_2M	1379.3	986	2.7041835	2 Singlet	FAPs
VAT_2M	1981.8	1316	1.1530508	2 Singlet	FAPs
VAT_2M	4072.9	2166	0.2541062	0 Singlet	Adipocyte
VAT_2M	1396.6	1014	3.496531	3 Singlet	FAPs

VAT_2M	3920.2	2034	0	0 Singlet	Adipocyte
VAT_2M	2163.4	1285	0.479991	0 Singlet	Adipocyte
VAT_2M	2740.7	1356	4.251048	7 Singlet	Endothelial_cells
VAT_2M	2083	1481	0.5150321	2 Singlet	FAPs
VAT_2M	3879.7	1789	0.9952365	0 Singlet	Adipocyte
VAT_2M	1269.5	1086	0.9149565	6 Singlet	Neurons
VAT_2M	1597.9	1054	1.6769356	2 Singlet	FAPs
VAT_2M	1790.7	1290	0.4429226	2 Singlet	FAPs
VAT_2M	2063.6	1327	0.8268437	0 Singlet	Adipocyte
VAT_2M	7575	2995	0.0490922	0 Singlet	Adipocyte
VAT_2M	1560.6	1046	0.7879301	6 Singlet	Neurons
VAT_2M	1814.5	1229	0.0351176	7 Singlet	Endothelial_cells
VAT_2M	9203.1	3189	0.06154	0 Singlet	Adipocyte
VAT_2M	1106.6	819	0.3100812	0 Singlet	Adipocyte
VAT_2M	1200.3	923	1.1525788	11 Singlet	Smooth_muscle_cells
VAT_2M	2035.9	1292	0.7901168	2 Singlet	FAPs
VAT_2M	2324.4	1388	0.095166	0 Singlet	Adipocyte
VAT_2M	1506.2	1040	4.0470727	2 Singlet	FAPs
VAT_2M	2770.4	1488	0.14134	0 Singlet	Adipocyte
VAT_2M	2094.5	1389	0.4628428	3 Singlet	FAPs
VAT_2M	2648.2	1762	2.1475298	3 Singlet	FAPs
VAT_2M	2241.9	1395	2.9389041	9 Singlet	Mesothelial
VAT_2M	2182.1	1364	0.730272	0 Singlet	Adipocyte
VAT_2M	4202.3	2139	0.4042323	0 Singlet	Adipocyte
VAT_2M	1142.5	977	0.9289705	6 Singlet	Neurons
VAT_2M	2010.3	1238	0.2740459	3 Singlet	FAPs
VAT_2M	2179.8	1427	0	3 Singlet	FAPs
VAT_2M	2908.6	1722	1.1613083	2 Singlet	FAPs
VAT_2M	2597.5	1524	0.5105923	0 Singlet	Adipocyte
VAT_2M	2090.9	1316	1.2623154	9 Singlet	Mesothelial
VAT_2M	1751.1	1159	1.7184783	7 Singlet	Endothelial_cells
VAT_2M	1728.7	1309	1.0372798	3 Singlet	FAPs
VAT_2M	1346.8	1079	0.7891394	5 Singlet	Immune_cells
VAT_2M	7053.5	2654	0.0032119	0 Singlet	Adipocyte
VAT_2M	1584.6	1011	0.6378184	0 Singlet	Adipocyte
VAT_2M	1320.4	920	0.2757199	3 Singlet	FAPs
VAT_2M	4623.7	2180	0.0320359	0 Singlet	Adipocyte
VAT_2M	1208.7	987	1.9387587	8 Singlet	Mesothelial
VAT_2M	2119.4	1607	2.7886611	2 Singlet	FAPs
VAT_2M	2391.8	1434	0.1396638	0 Singlet	Adipocyte
VAT_2M	6903.8	2616	0.0293236	0 Singlet	Adipocyte
VAT_2M	3783.6	1858	0.0617915	0 Singlet	Adipocyte
VAT_2M	2367.8	1666	0.905542	2 Singlet	FAPs
VAT_2M	1826.8	1200	0.9357191	0 Singlet	Adipocyte
VAT_2M	2956.8	1718	1.9709218	3 Singlet	FAPs
VAT_2M	1833.4	1299	0.904713	2 Singlet	FAPs
VAT_2M	2320.1	1364	0.1730833	3 Singlet	FAPs
VAT_2M	3912.9	1995	0.4148961	0 Singlet	Adipocyte
VAT_2M	1430.3	1005	2.9868731	2 Singlet	FAPs
VAT_2M	2718.6	1566	0.0267912	0 Singlet	Adipocyte
VAT_2M	1207.3	909	0.5325959	2 Singlet	FAPs

VAT_2M	8452.2	3114	0.0112293	0	Singlet	Adipocyte
VAT_2M	3338.2	2084	0.2416783	3	Singlet	FAPs
VAT_2M	1980.2	1281	2.2689536	2	Singlet	FAPs
VAT_2M	7241.4	2780	0	0	Singlet	Adipocyte
VAT_2M	1554.3	1086	0.8474052	3	Singlet	FAPs
VAT_2M	4710.3	2271	0	0	Singlet	Adipocyte
VAT_2M	7194.5	2879	0.0511674	0	Singlet	Adipocyte
VAT_2M	6509.4	2626	0.0342546	0	Singlet	Adipocyte
VAT_2M	1694.5	1114	0.1147506	0	Singlet	Adipocyte
VAT_2M	1721.1	1101	0.0920501	0	Singlet	Adipocyte
VAT_2M	1011.7	824	0.5570192	7	Singlet	Endothelial_cells
VAT_2M	1156.3	868	0.8428994	3	Singlet	FAPs
VAT_2M	13064	3553	1.663601	0	Singlet	Adipocyte
VAT_2M	5376.5	2328	0.0144723	0	Singlet	Adipocyte
VAT_2M	6577.8	2688	0.0141791	0	Singlet	Adipocyte
VAT_2M	9986	3296	0.0369207	0	Singlet	Adipocyte
VAT_2M	5802.1	2262	0.0060284	0	Singlet	Adipocyte
VAT_2M	2444.2	1438	1.0589247	5	Singlet	Immune_cells
VAT_2M	2133.5	1402	0.9591996	2	Singlet	FAPs
VAT_2M	2237.8	1482	0.9077031	2	Singlet	FAPs
VAT_2M	1071.5	874	1.6601708	7	Singlet	Endothelial_cells
VAT_2M	1485.4	943	0.370352	0	Singlet	Adipocyte
VAT_2M	7310.5	2611	0.0317279	0	Singlet	Adipocyte
VAT_2M	8057.4	2782	0.0007586	0	Singlet	Adipocyte
VAT_2M	3484.7	1998	2.0796431	3	Singlet	FAPs
VAT_2M	5386.7	2318	0.0235112	0	Singlet	Adipocyte
VAT_2M	4022	1883	0.1110935	0	Singlet	Adipocyte
VAT_2M	1033.9	828	2.70478	1	Singlet	Immune_cells
VAT_2M	3652.9	2123	0.0331446	2	Singlet	FAPs
VAT_2M	2954.2	1404	1.0622652	0	Singlet	Adipocyte
VAT_2M	6537.5	2727	0.0567239	0	Singlet	Adipocyte
VAT_2M	1253.9	1057	0.8704255	4	Singlet	Immune_cells
VAT_2M	1183.7	847	0.5740422	7	Singlet	Endothelial_cells
VAT_2M	3120.6	1656	0.1499471	0	Singlet	Adipocyte
VAT_2M	1674.8	1262	0.327906	3	Singlet	FAPs
VAT_2M	3088.1	1873	0.9655114	3	Singlet	FAPs
VAT_2M	6712.2	2818	0.0053722	0	Singlet	Adipocyte
VAT_2M	4365.7	1979	0.0457848	0	Singlet	Adipocyte
VAT_2M	8672.6	3107	0.1458314	0	Singlet	Adipocyte
VAT_2M	3573	2081	2.2012882	3	Singlet	FAPs
VAT_2M	1164.1	931	0.658963	6	Singlet	Neurons
VAT_2M	1644.1	1147	0.1791444	3	Singlet	FAPs
VAT_2M	3032.6	1550	0	0	Singlet	Adipocyte
VAT_2M	2787.9	1774	0.4025996	5	Singlet	Immune_cells
VAT_2M	7790.8	3125	0.0721792	0	Singlet	Adipocyte
VAT_2M	4961.4	2146	0.264024	0	Singlet	Adipocyte
VAT_2M	3537.1	1945	0.5679804	0	Singlet	Adipocyte
VAT_2M	1961.3	1337	1.8056648	6	Singlet	Neurons
VAT_2M	4979.2	2213	0.1065529	0	Singlet	Adipocyte
VAT_2M	1336.4	1027	1.298938	6	Singlet	Neurons
VAT_2M	9315.2	3148	0.0127331	0	Singlet	Adipocyte

VAT_2M	1234.4	917	0.0278639	2	Singlet	FAPs
VAT_2M	10236	3110	0.0202322	0	Singlet	Adipocyte
VAT_2M	3943.9	1793	0.3845691	3	Singlet	FAPs
VAT_2M	1564.7	1160	1.0937244	3	Singlet	FAPs
VAT_2M	2234.3	1314	1.8642108	2	Singlet	FAPs
VAT_2M	1805.3	1168	2.388204	0	Singlet	Adipocyte
VAT_2M	7848.8	2979	0.201445	0	Singlet	Adipocyte
VAT_2M	1163.7	891	1.2917907	2	Singlet	FAPs
VAT_2M	2904.3	1747	0.6008382	11	Singlet	Smooth_muscle_cells
VAT_2M	2630.9	1593	1.2621948	3	Singlet	FAPs
VAT_2M	1469.7	1056	0.8736947	7	Singlet	Endothelial_cells
VAT_2M	7670.2	2812	0.4095216	0	Singlet	Adipocyte
VAT_2M	4517.9	1881	0.0309025	0	Singlet	Adipocyte
VAT_2M	3632.3	1754	1.9797835	0	Singlet	Adipocyte
VAT_2M	8390.6	3066	0.0073795	0	Singlet	Adipocyte
VAT_2M	2071.1	1483	0.4918538	5	Singlet	Immune_cells
VAT_2M	6146.4	2573	0.0617979	0	Singlet	Adipocyte
VAT_2M	6638.1	2543	0.0994281	0	Singlet	Adipocyte
VAT_2M	4339.4	1840	0.9274244	0	Singlet	Adipocyte
VAT_2M	2335.2	1545	0.1990679	3	Singlet	FAPs
VAT_2M	1310.5	937	2.4585265	2	Singlet	FAPs
VAT_2M	4061.8	1778	0.0153693	0	Singlet	Adipocyte
VAT_2M	3533.3	1930	0.9532255	2	Singlet	FAPs
VAT_2M	1225.8	1039	0.6745204	6	Singlet	Neurons
VAT_2M	1534.6	973	2.1305327	5	Singlet	Immune_cells
VAT_2M	3268.7	1944	1.1463404	2	Singlet	FAPs
VAT_2M	2375.4	1368	7.7084587	2	Singlet	FAPs
VAT_2M	1926.5	1219	0.9245682	2	Singlet	FAPs
VAT_2M	3421.5	1540	0.2985941	0	Singlet	Adipocyte
VAT_2M	2027.4	1266	0.2153771	11	Singlet	Smooth_muscle_cells
VAT_2M	3719.5	1886	0.8359645	0	Singlet	Adipocyte
VAT_2M	4726	2323	0.0408391	0	Singlet	Adipocyte
VAT_2M	1962.5	962	0.1837418	0	Singlet	Adipocyte
VAT_2M	1088.8	644	0.1867767	6	Singlet	Neurons
VAT_2M	1363.1	912	1.8448807	3	Singlet	FAPs
VAT_2M	1300.7	920	1.017227	7	Singlet	Endothelial_cells
VAT_2M	7246.5	2847	0.0093986	0	Singlet	Adipocyte
VAT_2M	1517.5	871	5.5874105	9	Singlet	Mesothelial
VAT_2M	5060.5	2367	0.0936843	0	Singlet	Adipocyte
VAT_2M	2269.3	1552	1.065509	2	Singlet	FAPs
VAT_2M	1037.7	693	0.2975346	4	Singlet	Immune_cells
VAT_2M	3510.8	1948	0.8728583	0	Singlet	Adipocyte
VAT_2M	1137.1	779	0.1740617	3	Singlet	FAPs
VAT_2M	1527.1	901	5.3550571	5	Singlet	Immune_cells
VAT_2M	4746.4	2390	0.2393249	0	Singlet	Adipocyte
VAT_2M	1026.9	785	1.01982	0	Singlet	Adipocyte
VAT_2M	6776.5	2546	0.0644506	0	Singlet	Adipocyte
VAT_2M	1594.9	1078	2.7451068	9	Singlet	Mesothelial
VAT_2M	4302.9	1987	0.0382785	0	Singlet	Adipocyte
VAT_2M	1696.6	1085	0.2164748	3	Singlet	FAPs
VAT_2M	5355.4	2180	14.208865	7	Singlet	Endothelial_cells

VAT_2M	1047.2	894	2.0652724	6	Singlet	Neurons
VAT_2M	1772.1	1511	1.9697597	6	Singlet	Neurons
VAT_2M	1813.4	1410	2.6530622	3	Singlet	FAPs
VAT_2M	2021.3	1351	1.0597648	2	Singlet	FAPs
VAT_2M	3056.2	1713	0.5578424	0	Singlet	Adipocyte
VAT_2M	1229.8	710	2.1538117	7	Singlet	Endothelial_cells
VAT_2M	1152.3	906	1.1555384	3	Singlet	FAPs
VAT_2M	1837.2	942	2.3620407	0	Singlet	Adipocyte
VAT_2M	5343.3	2546	0.0110619	0	Singlet	Adipocyte
VAT_2M	4766.1	2151	0.1989494	0	Singlet	Adipocyte
VAT_2M	1122	877	2.0472924	5	Singlet	Immune_cells
VAT_2M	2260.8	1536	1.7030198	2	Singlet	FAPs
VAT_2M	4652.8	2144	0	0	Singlet	Adipocyte
VAT_2M	3890.5	1843	0.039772	0	Singlet	Adipocyte
VAT_2M	1877.6	1303	4.167051	6	Singlet	Neurons
VAT_2M	2436.7	1471	0.2105423	0	Singlet	Adipocyte
VAT_2M	1223.9	863	0.3020294	7	Singlet	Endothelial_cells
VAT_2M	6018.9	2515	0.1056823	0	Singlet	Adipocyte
VAT_2M	3336.5	1752	0.3945115	0	Singlet	Adipocyte
VAT_2M	1139.5	866	0.5035399	2	Singlet	FAPs
VAT_2M	1160.3	890	1.9394189	2	Singlet	FAPs
VAT_2M	2555.6	1560	0.6989708	5	Singlet	Immune_cells
VAT_2M	5403.9	2361	0.1306408	0	Singlet	Adipocyte
VAT_2M	2048	1299	1.2571118	7	Singlet	Endothelial_cells
VAT_2M	2903.4	1865	0.9028719	2	Singlet	FAPs
VAT_2M	1290.5	932	0.8184373	2	Singlet	FAPs
VAT_2M	1764.7	1083	0.2629125	0	Singlet	Adipocyte
VAT_2M	3629.8	1809	0.1537012	0	Singlet	Adipocyte
VAT_2M	1326.9	942	0.8810666	3	Singlet	FAPs
VAT_2M	6176.6	2479	0.047028	0	Singlet	Adipocyte
VAT_2M	1064	758	3.7115459	7	Singlet	Endothelial_cells
VAT_2M	6415	2594	0.4554552	0	Singlet	Adipocyte
VAT_2M	1735.3	1277	2.3764226	9	Singlet	Mesothelial
VAT_2M	3092.9	1783	0.2305873	0	Singlet	Adipocyte
VAT_2M	1738.4	1375	0.054007	3	Singlet	FAPs
VAT_2M	1673.1	1232	0.4261453	2	Singlet	FAPs
VAT_2M	2394.8	1564	0.1852797	2	Singlet	FAPs
VAT_2M	1705.5	1202	0.8733819	6	Singlet	Neurons
VAT_2M	2568.7	1701	3.7196998	0	Singlet	Adipocyte
VAT_2M	1059.2	879	0.2664668	8	Singlet	Mesothelial
VAT_2M	4523.6	2146	0.0105573	0	Singlet	Adipocyte
VAT_2M	2045.8	1195	1.2706433	7	Singlet	Endothelial_cells
VAT_2M	8054.9	2945	0.0039007	0	Singlet	Adipocyte
VAT_2M	4299.1	2086	0.6416153	0	Singlet	Adipocyte
VAT_2M	2200.8	1507	2.594883	2	Singlet	FAPs
VAT_2M	2452.8	1490	0.0867385	0	Singlet	Adipocyte
VAT_2M	2513.3	1669	1.1868229	5	Singlet	Immune_cells
VAT_2M	1005	808	3.7172703	6	Singlet	Neurons
VAT_2M	2038.1	1376	1.1415169	3	Singlet	FAPs
VAT_2M	2713	1742	0.2445193	2	Singlet	FAPs
VAT_2M	7943.2	2957	0.048396	0	Singlet	Adipocyte

VAT_2M	1274.4	1086	0.7297635	8	Singlet	Mesothelial
VAT_2M	6943.6	2810	0.1646932	0	Singlet	Adipocyte
VAT_2M	1652.7	1050	5.5743968	2	Singlet	FAPs
VAT_2M	1425.1	999	0.8536386	3	Singlet	FAPs
VAT_2M	1199.5	995	0.9704137	8	Singlet	Mesothelial
VAT_2M	1803.4	1253	1.0861377	2	Singlet	FAPs
VAT_2M	1070.8	863	2.8900931	8	Singlet	Mesothelial
VAT_2M	3696.6	1915	0.2006389	0	Singlet	Adipocyte
VAT_2M	2371.4	1373	0.2196544	0	Singlet	Adipocyte
VAT_2M	2143.3	1335	0.8287418	3	Singlet	FAPs
VAT_2M	6365.9	2504	0.0334918	0	Singlet	Adipocyte
VAT_2M	9683.4	3462	0.0183022	0	Singlet	Adipocyte
VAT_2M	7189	2820	0.1115716	0	Singlet	Adipocyte
VAT_2M	4187.2	2045	0.0604864	0	Singlet	Adipocyte
VAT_2M	1544.1	1045	3.6728067	2	Singlet	FAPs
VAT_2M	1406.3	1010	2.1056716	6	Singlet	Neurons
VAT_2M	2213.5	1356	1.2078359	2	Singlet	FAPs
VAT_2M	1577.7	1082	1.9388891	2	Singlet	FAPs
VAT_2M	6675.8	2683	0.0238974	0	Singlet	Adipocyte
VAT_2M	1154.5	827	1.470779	3	Singlet	FAPs
VAT_2M	1258	963	0.2576805	3	Singlet	FAPs
VAT_2M	1835.2	1166	8.0466885	5	Singlet	Immune_cells
VAT_2M	1554.5	1356	0.6133664	6	Singlet	Neurons
VAT_2M	5294.5	2480	0.0147454	0	Singlet	Adipocyte
VAT_2M	4911.1	2234	0.0522197	0	Singlet	Adipocyte
VAT_2M	9268.5	2951	0.024396	0	Singlet	Adipocyte
VAT_2M	4769	2281	0.0087931	0	Singlet	Adipocyte
VAT_2M	7488.8	2802	0.0754915	0	Singlet	Adipocyte
VAT_2M	3645	1838	0.1500254	0	Singlet	Adipocyte
VAT_2M	2765.3	1526	3.0977982	5	Singlet	Immune_cells
VAT_2M	1187.7	848	7.3205449	5	Singlet	Immune_cells
VAT_2M	3685.4	1903	0.06987	3	Singlet	FAPs
VAT_2M	4415.1	2181	0.0684216	0	Singlet	Adipocyte
VAT_2M	1311.2	932	0.8104623	3	Singlet	FAPs
VAT_2M	5471.3	2377	0.5426972	0	Singlet	Adipocyte
VAT_2M	2691.2	1526	0.0257844	0	Singlet	Adipocyte
VAT_2M	1203.7	875	0.434251	6	Singlet	Neurons
VAT_2M	1592.4	1214	0.9443979	2	Singlet	FAPs
VAT_2M	2262.6	1599	0.994112	3	Singlet	FAPs
VAT_2M	1776	1147	6.2787453	7	Singlet	Endothelial_cells
VAT_2M	2975.9	1855	0.9783949	2	Singlet	FAPs
VAT_2M	3611.8	1966	0.3379978	2	Singlet	FAPs
VAT_2M	2303.3	1184	9.7808652	7	Singlet	Endothelial_cells
VAT_2M	5711.3	2450	0.0294875	0	Singlet	Adipocyte
VAT_2M	1110.3	874	4.9486464	0	Singlet	Adipocyte
VAT_2M	3849.6	1998	0	0	Singlet	Adipocyte
VAT_2M	1374.6	1001	1.7445301	3	Singlet	FAPs
VAT_2M	3063.5	1786	0.9273499	5	Singlet	Immune_cells
VAT_2M	9855.3	3412	0.0106366	0	Singlet	Adipocyte
VAT_2M	3082.2	1883	2.6845264	2	Singlet	FAPs
VAT_2M	1294.8	953	0.3951307	6	Singlet	Neurons

VAT_2M	5103.7	2409	0.1460746	0	Singlet	Adipocyte
VAT_2M	1111	866	2.6921438	6	Singlet	Neurons
VAT_2M	1575	972	0.8722701	0	Singlet	Adipocyte
VAT_2M	11966	3694	0.0757393	0	Singlet	Adipocyte
VAT_2M	1557.4	1207	0	5	Singlet	Immune_cells
VAT_2M	4984.9	2152	0.0954879	0	Singlet	Adipocyte
VAT_2M	2630.6	1512	0.8209742	0	Singlet	Adipocyte
VAT_2M	1549.4	1106	1.1482563	2	Singlet	FAPs
VAT_2M	1662.9	1190	0	6	Singlet	Neurons
VAT_2M	3303.6	2025	0.9394927	3	Singlet	FAPs
VAT_2M	8190	2968	0.1468559	0	Singlet	Adipocyte
VAT_2M	2653.3	1695	0.0395949	0	Singlet	Adipocyte
VAT_2M	1425.3	992	0.1799929	3	Singlet	FAPs
VAT_2M	1800.4	1365	1.3272016	9	Singlet	Mesothelial
VAT_2M	1151.5	931	3.2744998	5	Singlet	Immune_cells
VAT_2M	2827.7	1654	1.0541247	0	Singlet	Adipocyte
VAT_2M	3495.5	1972	0.4143596	2	Singlet	FAPs
VAT_2M	6808.2	2562	0.0827158	0	Singlet	Adipocyte
VAT_2M	7458.4	2927	0.1413486	0	Singlet	Adipocyte
VAT_2M	6866.4	2656	0.0279629	0	Singlet	Adipocyte
VAT_2M	4131	2010	0.0275049	0	Singlet	Adipocyte
VAT_2M	2498.6	1400	0.1931305	0	Singlet	Adipocyte
VAT_2M	2568.6	1364	0.1923233	0	Singlet	Adipocyte
VAT_2M	1244.3	954	0	8	Singlet	Mesothelial
VAT_2M	1140.1	820	3.9160185	0	Singlet	Adipocyte
VAT_2M	5967.2	2525	0.0840387	0	Singlet	Adipocyte
VAT_2M	1751.3	1109	0.155124	5	Singlet	Immune_cells
VAT_2M	4137.5	2191	1.1532855	5	Singlet	Immune_cells
VAT_2M	2070.8	1256	0.4491893	0	Singlet	Adipocyte
VAT_2M	2803.9	1768	0.935678	2	Singlet	FAPs
VAT_2M	4346.8	2158	0.3011144	0	Singlet	Adipocyte
VAT_2M	2044.6	1378	0.3168993	2	Singlet	FAPs
VAT_2M	2154.1	1266	0.0334403	0	Singlet	Adipocyte
VAT_2M	1974.1	1253	0.5729544	3	Singlet	FAPs
VAT_2M	10307	3281	1.2131808	0	Singlet	Adipocyte
VAT_2M	3240.2	1756	0.0368115	0	Singlet	Adipocyte
VAT_2M	3665.9	1858	0.1052611	0	Singlet	Adipocyte
VAT_2M	1688.3	1059	3.8073721	2	Singlet	FAPs
VAT_2M	6709.6	2734	0	0	Singlet	Adipocyte
VAT_2M	1284.1	878	1.7325832	2	Singlet	FAPs
VAT_2M	1585.1	1144	1.3101257	2	Singlet	FAPs
VAT_2M	1743	1200	1.9847409	3	Singlet	FAPs
VAT_2M	1133.5	865	0.4305148	0	Singlet	Adipocyte
VAT_2M	4813	2354	0.0302251	0	Singlet	Adipocyte
VAT_2M	3188.4	1795	0.0506707	0	Singlet	Adipocyte
VAT_2M	2378.1	1492	0.4425909	3	Singlet	FAPs
VAT_2M	4488.6	2468	1.7694867	5	Singlet	Immune_cells
VAT_2M	2199.8	1429	0.257835	3	Singlet	FAPs
VAT_2M	2525.8	1680	1.7148156	2	Singlet	FAPs
VAT_2M	6110.2	2434	0.8278353	0	Singlet	Adipocyte
VAT_2M	4235.8	2145	0.0763509	0	Singlet	Adipocyte

VAT_2M	1051.6	795	0.5845187	0	Singlet	Adipocyte
VAT_2M	7034.4	2787	0.0996738	0	Singlet	Adipocyte
VAT_2M	6228.8	2598	0	0	Singlet	Adipocyte
VAT_2M	1048.2	772	1.4315443	3	Singlet	FAPs
VAT_2M	2107.2	1241	0.6461653	0	Singlet	Adipocyte
VAT_2M	1957.6	1280	2.6290356	0	Singlet	Adipocyte
VAT_2M	1457.9	1187	0.4017673	6	Singlet	Neurons
VAT_2M	5274	2454	1.3085407	5	Singlet	Immune_cells
VAT_2M	1349	1034	0.4112325	5	Singlet	Immune_cells
VAT_2M	5729.1	2552	0.0089773	0	Singlet	Adipocyte
VAT_2M	1933.7	1292	0.2843227	3	Singlet	FAPs
VAT_2M	5371	2353	0.0401862	0	Singlet	Adipocyte
VAT_2M	1925.5	1306	1.5936432	3	Singlet	FAPs
VAT_2M	1014.6	746	0.2773866	3	Singlet	FAPs
VAT_2M	1042.2	756	1.4413355	2	Singlet	FAPs
VAT_2M	1521.7	1053	1.249801	6	Singlet	Neurons
VAT_2M	3630.4	1772	0.0652477	0	Singlet	Adipocyte
VAT_2M	1805.8	1195	0.3387176	6	Singlet	Neurons
VAT_2M	6572	2662	0.1209111	0	Singlet	Adipocyte
VAT_2M	1033.1	754	0.9921521	5	Singlet	Immune_cells
VAT_2M	4733.3	1987	0.02735	0	Singlet	Adipocyte
VAT_2M	7215.2	2793	0.070404	0	Singlet	Adipocyte
VAT_2M	3573.2	1797	0	0	Singlet	Adipocyte
VAT_2M	6079.3	2670	0.0191271	0	Singlet	Adipocyte
VAT_2M	1944.3	1450	1.4752837	2	Singlet	FAPs
VAT_2M	2210.2	1412	0.5174249	3	Singlet	FAPs
VAT_2M	1154.4	816	0.1464847	3	Singlet	FAPs
VAT_2M	1775.3	1199	1.817614	2	Singlet	FAPs
VAT_2M	5097.1	2219	0.1112405	0	Singlet	Adipocyte
VAT_2M	3564.9	1942	0.4416093	0	Singlet	Adipocyte
VAT_2M	1029	857	2.3568258	6	Singlet	Neurons
VAT_2M	5268.3	2304	0.1930551	0	Singlet	Adipocyte
VAT_2M	1124.5	910	1.3073644	2	Singlet	FAPs
VAT_2M	7245.4	2890	0.105555	0	Singlet	Adipocyte
VAT_2M	1166.7	877	1.0047497	2	Singlet	FAPs
VAT_2M	6863.1	2725	0.0516374	0	Singlet	Adipocyte
VAT_2M	3659.9	2059	2.2629486	2	Singlet	FAPs
VAT_2M	4294.7	2088	0.0596718	0	Singlet	Adipocyte
VAT_2M	1045.3	698	11.01819	9	Singlet	Mesothelial
VAT_2M	1101.6	799	1.1432704	7	Singlet	Endothelial_cells
VAT_2M	1074.1	793	0.3834209	2	Singlet	FAPs
VAT_2M	3921.6	2011	8.7903649	7	Singlet	Endothelial_cells
VAT_2M	2205.1	1348	0.6684646	0	Singlet	Adipocyte
VAT_2M	4531.7	2137	0.3983927	0	Singlet	Adipocyte
VAT_2M	1438.8	1043	0.7149263	2	Singlet	FAPs
VAT_2M	5231.3	2386	0.0281291	0	Singlet	Adipocyte
VAT_2M	1890.3	1312	0.7858567	5	Singlet	Immune_cells
VAT_2M	5140.2	2368	0.1081412	0	Singlet	Adipocyte
VAT_2M	3940	1998	0.2490573	0	Singlet	Adipocyte
VAT_2M	2914.9	1770	0.509073	5	Singlet	Immune_cells
VAT_2M	1580.2	1107	0.3843085	5	Singlet	Immune_cells

VAT_2M	1026.9	861	2.0542266	6	Singlet	Neurons
VAT_2M	8567.3	3018	0.0123054	0	Singlet	Adipocyte
VAT_2M	4513.4	2125	0	0	Singlet	Adipocyte
VAT_2M	4295.9	2010	0.2339487	0	Singlet	Adipocyte
VAT_2M	3155.1	1663	0.1997054	0	Singlet	Adipocyte
VAT_2M	9133.2	3199	0.2520985	0	Singlet	Adipocyte
VAT_2M	1691.6	1250	2.6084321	2	Singlet	FAPs
VAT_2M	1167	856	1.1083865	5	Singlet	Immune_cells
VAT_2M	1951.5	1152	0.677657	0	Singlet	Adipocyte
VAT_2M	3291	1695	0.735442	0	Singlet	Adipocyte
VAT_2M	2913.6	1732	0.120438	5	Singlet	Immune_cells
VAT_2M	1421.9	1047	1.4289639	4	Singlet	Immune_cells
VAT_2M	3654.7	1941	0.2514161	0	Singlet	Adipocyte
VAT_2M	2829	1834	0.0206789	3	Singlet	FAPs
VAT_2M	7419.7	2913	0.1073494	0	Singlet	Adipocyte
VAT_2M	1581.5	1072	0.8154555	2	Singlet	FAPs
VAT_2M	2174.1	1772	0.3037228	6	Singlet	Neurons
VAT_2M	1290.4	886	0.4176395	7	Singlet	Endothelial_cells
VAT_2M	1690.8	1278	0.7341877	2	Singlet	FAPs
VAT_2M	7575.4	3042	0.2184383	0	Singlet	Adipocyte
VAT_2M	1813.1	1374	0.9487897	5	Singlet	Immune_cells
VAT_2M	1001.9	683	6.4936613	5	Singlet	Immune_cells
VAT_2M	1176	975	2.8086816	6	Singlet	Neurons
VAT_2M	1512.2	1077	1.4459404	5	Singlet	Immune_cells
VAT_2M	6329.7	2743	0.043598	0	Singlet	Adipocyte
VAT_2M	1200.3	913	1.383518	3	Singlet	FAPs
VAT_2M	1028.5	775	2.2161259	5	Singlet	Immune_cells
VAT_2M	2389.8	1463	1.6858125	0	Singlet	Adipocyte
VAT_2M	2819.3	1479	0.4843482	3	Singlet	FAPs
VAT_2M	4147.4	2055	0.5547821	0	Singlet	Adipocyte
VAT_2M	6681.2	2761	0.0256109	0	Singlet	Adipocyte
VAT_2M	1880.7	1254	2.6878335	7	Singlet	Endothelial_cells
VAT_2M	1562.4	1160	1.1811575	3	Singlet	FAPs
VAT_2M	1096.8	915	0.6419954	7	Singlet	Endothelial_cells
VAT_2M	3788.9	2121	0.0994967	0	Singlet	Adipocyte
VAT_2M	3472.8	1867	0.0742518	0	Singlet	Adipocyte
VAT_2M	2508	1569	0.2096988	2	Singlet	FAPs
VAT_2M	3442.2	1854	0.2918026	0	Singlet	Adipocyte
VAT_2M	2830.2	1557	0.829624	0	Singlet	Adipocyte
VAT_2M	3830.9	1976	0.0322618	0	Singlet	Adipocyte
VAT_2M	1551.2	1077	0.8122823	3	Singlet	FAPs
VAT_2M	4052.3	2030	0.02254	0	Singlet	Adipocyte
VAT_2M	3048.6	1811	0.0555084	0	Singlet	Adipocyte
VAT_2M	2516	1464	0.8580817	0	Singlet	Adipocyte
VAT_2M	1232.5	847	1.1762835	7	Singlet	Endothelial_cells
VAT_2M	2887.6	1651	0.3701656	0	Singlet	Adipocyte
VAT_2M	2234.8	1611	0.969077	5	Singlet	Immune_cells
VAT_2M	1436	995	0.8752192	2	Singlet	FAPs
VAT_2M	1803.9	1145	0.1644937	0	Singlet	Adipocyte
VAT_2M	2627.4	1659	1.4161576	2	Singlet	FAPs
VAT_2M	2687.1	1591	0.2670192	3	Singlet	FAPs

VAT_2M	2776.3	1827	0.8565454	2	Singlet	FAPs
VAT_2M	1078.6	920	1.6289365	6	Singlet	Neurons
VAT_2M	1162.9	813	0.3772682	7	Singlet	Endothelial_cells
VAT_2M	1194	971	0.1701399	7	Singlet	Endothelial_cells
VAT_2M	2080.1	1263	1.807953	0	Singlet	Adipocyte
VAT_2M	1019.2	861	0.4461862	1	Singlet	Immune_cells
VAT_2M	1260	969	2.950613	6	Singlet	Neurons
VAT_2M	1189.7	922	3.3301185	1	Singlet	Immune_cells
VAT_2M	2360.6	1387	0.5150747	0	Singlet	Adipocyte
VAT_2M	5525.2	2387	0.0408351	0	Singlet	Adipocyte
VAT_2M	3755	1930	0.1567233	0	Singlet	Adipocyte
VAT_2M	3740.1	1868	0.0853262	0	Singlet	Adipocyte
VAT_2M	1727.4	1214	0.1102254	2	Singlet	FAPs
VAT_2M	1066.3	947	0.8869318	6	Singlet	Neurons
VAT_2M	3349.5	1894	4.4756975	5	Singlet	Immune_cells
VAT_2M	9362.7	3285	0.4283379	0	Singlet	Adipocyte
VAT_2M	3971.8	2094	0.0355027	0	Singlet	Adipocyte
VAT_2M	4892.4	2329	0.0109269	0	Singlet	Adipocyte
VAT_2M	2726.4	1623	0.1703058	0	Singlet	Adipocyte
VAT_2M	3591	1813	0.2014074	0	Singlet	Adipocyte
VAT_2M	3616.3	1846	1.2797929	0	Singlet	Adipocyte
VAT_2M	5589.8	2580	0.2311277	0	Singlet	Adipocyte
VAT_2M	1554.7	974	1.4842008	7	Singlet	Endothelial_cells
VAT_2M	6323.2	2633	0.0723941	0	Singlet	Adipocyte
VAT_2M	1040.9	889	1.5487181	4	Singlet	Immune_cells
VAT_2M	2048.2	1281	1.9272347	5	Singlet	Immune_cells
VAT_2M	2178	1544	1.358806	2	Singlet	FAPs
VAT_2M	9826.8	3285	0	0	Singlet	Adipocyte
VAT_2M	6807.9	2497	0.0865606	0	Singlet	Adipocyte
VAT_2M	1900.1	1223	0.5983403	0	Singlet	Adipocyte
VAT_2M	3193.9	1785	0.17811	2	Singlet	FAPs
VAT_2M	3854.8	2035	0.1833235	0	Singlet	Adipocyte
VAT_2M	2275	1628	1.4869918	2	Singlet	FAPs
VAT_2M	1741.9	1070	2.766239	3	Singlet	FAPs
VAT_2M	4714.1	2109	0.1339604	0	Singlet	Adipocyte
VAT_2M	1976.1	1154	0.2662495	0	Singlet	Adipocyte
VAT_2M	1555.5	1332	1.3196355	6	Singlet	Neurons
VAT_2M	9542.5	3303	1.0554698	0	Singlet	Adipocyte
VAT_2M	1845.4	1398	0.1585424	7	Singlet	Endothelial_cells
VAT_2M	2001.9	1242	0.142058	0	Singlet	Adipocyte
VAT_2M	3349.3	1788	0.1718416	0	Singlet	Adipocyte
VAT_2M	1352	965	8.3459585	2	Singlet	FAPs
VAT_2M	1120.2	892	0.9055791	0	Singlet	Adipocyte
VAT_2M	4450.9	2212	0.1090007	0	Singlet	Adipocyte
VAT_2M	1665.8	1234	2.0097078	2	Singlet	FAPs
VAT_2M	2489.4	1452	0.2536734	0	Singlet	Adipocyte
VAT_2M	1383	1023	1.3174863	2	Singlet	FAPs
VAT_2M	2094.1	1377	2.6876856	2	Singlet	FAPs
VAT_2M	1666	1136	1.8225275	3	Singlet	FAPs
VAT_2M	1462	1024	1.4000321	5	Singlet	Immune_cells
VAT_2M	1835.2	1195	0.2554796	5	Singlet	Immune_cells

VAT_2M	6827.2	2954	0.0368549	0	Singlet	Adipocyte
VAT_2M	2950.4	1610	0.1796873	0	Singlet	Adipocyte
VAT_2M	1230.5	1020	0.733292	6	Singlet	Neurons
VAT_2M	5390	2360	0.1620362	0	Singlet	Adipocyte
VAT_2M	1675.2	1093	0.6575477	0	Singlet	Adipocyte
VAT_2M	1036.6	851	0.3066418	6	Singlet	Neurons
VAT_2M	6490.4	2670	0	0	Singlet	Adipocyte
VAT_2M	3070.2	1690	0.7417182	7	Singlet	Endothelial_cells
VAT_2M	7158.8	2884	1.0144338	0	Singlet	Adipocyte
VAT_2M	1537.9	1254	1.0530961	4	Singlet	Immune_cells
VAT_2M	4873.3	2202	0.0494076	0	Singlet	Adipocyte
VAT_2M	1299.8	941	1.4126437	2	Singlet	FAPs
VAT_2M	2234.5	1407	1.1940614	2	Singlet	FAPs
VAT_2M	1314.6	907	2.1624012	9	Singlet	Mesothelial
VAT_2M	1846	1138	0.0583686	0	Singlet	Adipocyte
VAT_2M	1608.7	1209	0.2528738	11	Singlet	Smooth_muscle_cells
VAT_2M	1457.4	1080	0.6351276	2	Singlet	FAPs
VAT_2M	4515	2199	0.0341691	0	Singlet	Adipocyte
VAT_2M	1368.4	1003	0.255118	2	Singlet	FAPs
VAT_2M	1090.4	861	0.0333514	7	Singlet	Endothelial_cells
VAT_2M	2131.4	1428	1.2399271	0	Singlet	Adipocyte
VAT_2M	1622.8	1201	0.1038082	5	Singlet	Immune_cells
VAT_2M	7985	2944	0.0149182	0	Singlet	Adipocyte
VAT_2M	1028	793	0.2379305	5	Singlet	Immune_cells
VAT_2M	7841.7	2985	0.1957641	0	Singlet	Adipocyte
VAT_2M	6621.7	2722	0.0029238	0	Singlet	Adipocyte
VAT_2M	2175.7	1651	4.475534	2	Singlet	FAPs
VAT_2M	7058.4	2913	0.037702	0	Singlet	Adipocyte
VAT_2M	5045.3	2375	4.1528105	7	Singlet	Endothelial_cells
VAT_2M	3436	1913	0.6134476	0	Singlet	Adipocyte
VAT_2M	6403.7	2532	0.0328701	0	Singlet	Adipocyte
VAT_2M	5017.8	2189	0.0548032	0	Singlet	Adipocyte
VAT_2M	1029.9	843	1.1709925	8	Singlet	Mesothelial
VAT_2M	3356.7	1707	0	0	Singlet	Adipocyte
VAT_2M	6286	2689	0	0	Singlet	Adipocyte
VAT_2M	3212.9	1722	1.0946989	0	Singlet	Adipocyte
VAT_2M	6956.2	2933	0.0910997	0	Singlet	Adipocyte
VAT_2M	1554.9	1217	1.9022841	6	Singlet	Neurons
VAT_2M	1107	774	1.5108887	11	Singlet	Smooth_muscle_cells
VAT_2M	1896.2	1445	1.5853083	2	Singlet	FAPs
VAT_2M	1009.9	718	3.3621553	2	Singlet	FAPs
VAT_2M	1410.7	1080	3.1088243	2	Singlet	FAPs
VAT_2M	1729.1	1368	0.712959	2	Singlet	FAPs
VAT_2M	1051.2	873	0.731093	6	Singlet	Neurons
VAT_2M	8363.9	3058	0.0849779	0	Singlet	Adipocyte
VAT_2M	11130	3596	0.0438884	0	Singlet	Adipocyte
VAT_2M	1345.8	1033	0.2632569	3	Singlet	FAPs
VAT_2M	2583.7	1420	0.3117414	0	Singlet	Adipocyte
VAT_2M	2219.6	1425	0.1067706	7	Singlet	Endothelial_cells
VAT_2M	12996	3772	0.0418873	0	Singlet	Adipocyte
VAT_2M	8568	3074	0.0708303	0	Singlet	Adipocyte

VAT_2M	2214.6	1376	0.405363	0	Singlet	Adipocyte
VAT_2M	8613.7	3109	0.0942019	0	Singlet	Adipocyte
VAT_2M	2210.5	1366	1.7474093	3	Singlet	FAPs
VAT_2M	2243.6	1367	0.2997115	0	Singlet	Adipocyte
VAT_2M	1700.6	1188	0.0079374	2	Singlet	FAPs
VAT_2M	3233.2	1792	0.7512641	0	Singlet	Adipocyte
VAT_2M	2292.9	1385	0.1389262	3	Singlet	FAPs
VAT_2M	2687.9	1526	0.173813	0	Singlet	Adipocyte
VAT_2M	1064.3	902	1.6664875	1	Singlet	Immune_cells
VAT_2M	1282.3	1031	4.0510186	2	Singlet	FAPs
VAT_2M	9531.8	3123	0.0098089	0	Singlet	Adipocyte
VAT_2M	1107.5	867	2.7025619	4	Singlet	Immune_cells
VAT_2M	1866.1	1305	1.171351	3	Singlet	FAPs
VAT_2M	4625	2167	0.8519919	0	Singlet	Adipocyte
VAT_2M	1517.9	1041	0.7289349	2	Singlet	FAPs
VAT_2M	1763.2	1174	0.1699754	6	Singlet	Neurons
VAT_2M	1667.8	1273	2.8596784	2	Singlet	FAPs
VAT_2M	3754.6	1918	1.7612562	0	Singlet	Adipocyte
VAT_2M	2261.9	1256	0	0	Singlet	Adipocyte
VAT_2M	6531.2	2822	0.0500843	0	Singlet	Adipocyte
VAT_2M	3662.5	1993	0.6781852	5	Singlet	Immune_cells
VAT_2M	1111.2	774	2.0644747	2	Singlet	FAPs
VAT_2M	6139.9	2583	0	0	Singlet	Adipocyte
VAT_2M	3145.9	1839	0	0	Singlet	Adipocyte
VAT_2M	1289.4	1075	0.6898633	6	Singlet	Neurons
VAT_2M	3445.1	1809	0.0287605	0	Singlet	Adipocyte
VAT_2M	1108.6	865	1.7907472	2	Singlet	FAPs
VAT_2M	1883.8	1160	0.9773747	2	Singlet	FAPs
VAT_2M	1802.4	1343	0.7345199	3	Singlet	FAPs
VAT_2M	3072.6	1687	0.1309993	0	Singlet	Adipocyte
VAT_2M	6534.6	2552	0.0075347	0	Singlet	Adipocyte
VAT_2M	4392.1	2057	0.0119811	0	Singlet	Adipocyte
VAT_2M	1685.9	998	0.1922495	0	Singlet	Adipocyte
VAT_2M	3251.1	1906	0.5136134	2	Singlet	FAPs
VAT_2M	1272.9	921	0.6173821	5	Singlet	Immune_cells
VAT_2M	1795.2	1320	1.1990497	5	Singlet	Immune_cells
VAT_2M	1971.3	1427	4.6278533	5	Singlet	Immune_cells
VAT_2M	8462.9	3134	0.9372289	0	Singlet	Adipocyte
VAT_2M	1248.1	924	1.9508964	9	Singlet	Mesothelial
VAT_2M	2069.4	1467	0.6053657	2	Singlet	FAPs
VAT_2M	1566	1043	0.963811	0	Singlet	Adipocyte
VAT_2M	1353.2	978	1.1567801	5	Singlet	Immune_cells
VAT_2M	4327	2087	0.0286639	0	Singlet	Adipocyte
VAT_2M	1482.3	1072	1.6224311	1	Singlet	Immune_cells
VAT_2M	2736.3	1595	0.0432799	7	Singlet	Endothelial_cells
VAT_2M	6960.2	2846	0.0957993	0	Singlet	Adipocyte
VAT_2M	1886.8	1201	0.0810632	0	Singlet	Adipocyte
VAT_2M	1205.9	930	3.5004156	3	Singlet	FAPs
VAT_2M	3980	2003	1.5124747	0	Singlet	Adipocyte
VAT_2M	6375.2	2724	0.0085617	0	Singlet	Adipocyte
VAT_2M	4799.9	2133	0.1548262	0	Singlet	Adipocyte

VAT_2M	1004.4	761	0.5765492	2	Singlet	FAPs
VAT_2M	5537.6	2913	0.6993667	2	Singlet	FAPs
VAT_2M	6319.8	2735	0.0861331	0	Singlet	Adipocyte
VAT_2M	6026.6	2501	0.2448153	0	Singlet	Adipocyte
VAT_2M	2606.4	1667	2.0885452	5	Singlet	Immune_cells
VAT_2M	1625.6	1189	0.3757616	5	Singlet	Immune_cells
VAT_2M	3012	1674	0.1484463	0	Singlet	Adipocyte
VAT_2M	3403.9	1807	0.0186805	0	Singlet	Adipocyte
VAT_2M	4381	2130	0.0917616	0	Singlet	Adipocyte
VAT_2M	3288.6	1693	1.2566651	0	Singlet	Adipocyte
VAT_2M	6897.9	2857	0.2063209	0	Singlet	Adipocyte
VAT_2M	1118.5	932	0.6813454	6	Singlet	Neurons
VAT_2M	7849.5	3014	0.0073246	0	Singlet	Adipocyte
VAT_2M	1221.2	833	1.8568898	9	Singlet	Mesothelial
VAT_2M	3326	1906	0.0406219	0	Singlet	Adipocyte
VAT_2M	1843.3	1152	0.3713635	0	Singlet	Adipocyte
VAT_2M	2080.3	1278	0.4030827	3	Singlet	FAPs
VAT_2M	1810.6	1137	0.2761025	0	Singlet	Adipocyte
VAT_2M	1565.9	999	2.3420568	5	Singlet	Immune_cells
VAT_2M	7309.2	2779	0.0092516	0	Singlet	Adipocyte
VAT_2M	5890.9	2653	0.0221785	0	Singlet	Adipocyte
VAT_2M	2813.2	1541	0.0100778	0	Singlet	Adipocyte
VAT_2M	3027.5	1641	0.068655	0	Singlet	Adipocyte
VAT_2M	1691.2	1100	0.0041671	3	Singlet	FAPs
VAT_2M	2450.3	1496	0.6384511	3	Singlet	FAPs
VAT_2M	4937.4	2185	0.0182068	0	Singlet	Adipocyte
VAT_2M	1084.6	887	1.0105218	3	Singlet	FAPs
VAT_2M	2098.5	1270	0.3575972	0	Singlet	Adipocyte
VAT_2M	10649	3389	0.0423872	0	Singlet	Adipocyte
VAT_2M	1016.3	767	0.0837492	3	Singlet	FAPs
VAT_2M	2586.2	1528	0.4934699	2	Singlet	FAPs
VAT_2M	2758.2	1621	0.2652407	5	Singlet	Immune_cells
VAT_2M	4721.5	2451	0.0867853	0	Singlet	Adipocyte
VAT_2M	3983.6	1969	0.3938883	0	Singlet	Adipocyte
VAT_2M	1391.5	1074	0.677616	3	Singlet	FAPs
VAT_2M	1224.3	873	0.3887159	3	Singlet	FAPs
VAT_2M	4920.9	2341	0.0509554	0	Singlet	Adipocyte
VAT_2M	2393.4	1015	10.664349	0	Singlet	Adipocyte
VAT_2M	1150.6	821	0.1149179	1	Singlet	Immune_cells
VAT_2M	8712.2	3200	0.0485093	0	Singlet	Adipocyte
VAT_2M	4799	2166	0.0189221	0	Singlet	Adipocyte
VAT_2M	1140.1	838	0.9434212	2	Singlet	FAPs
VAT_2M	1729.1	1192	0.9579041	3	Singlet	FAPs
VAT_2M	1887.7	1327	1.5818517	9	Singlet	Mesothelial
VAT_2M	1331.5	1062	1.4409439	3	Singlet	FAPs
VAT_2M	1400	963	0.5099298	5	Singlet	Immune_cells
VAT_2M	1416.6	976	0.1053016	3	Singlet	FAPs
VAT_2M	1068	869	4.1876933	6	Singlet	Neurons
VAT_2M	1417.2	977	0.286992	2	Singlet	FAPs
VAT_2M	1675.6	1147	0.7649533	5	Singlet	Immune_cells
VAT_2M	1547.5	1059	0.321468	7	Singlet	Endothelial_cells

VAT_2M	7289.7	2876	0.1350427	0	Singlet	Adipocyte
VAT_2M	3502.5	2064	0.0979983	3	Singlet	FAPs
VAT_2M	1021.7	754	0.2373222	1	Singlet	Immune_cells
VAT_2M	1339.4	1048	1.021623	2	Singlet	FAPs
VAT_2M	1022.1	838	0.5969677	11	Singlet	Smooth_muscle_cells
VAT_2M	1191.1	898	0.282087	3	Singlet	FAPs
VAT_2M	8173.6	2963	0.0139603	0	Singlet	Adipocyte
VAT_2M	1195.1	911	0.5612907	7	Singlet	Endothelial_cells
VAT_2M	1116	879	1.5720101	3	Singlet	FAPs
VAT_2M	1468.4	1004	1.0082391	7	Singlet	Endothelial_cells
VAT_2M	4656.5	2305	0.0218299	0	Singlet	Adipocyte
VAT_2M	1792.1	1642	2.9248203	6	Singlet	Neurons
VAT_2M	2472.2	1543	0.4699671	0	Singlet	Adipocyte
VAT_2M	1290.2	954	0.7346602	7	Singlet	Endothelial_cells
VAT_2M	8636.9	3145	0.0140595	0	Singlet	Adipocyte
VAT_2M	2897.9	1707	14.265592	6	Singlet	Neurons
VAT_2M	2339.6	1379	0.0660473	0	Singlet	Adipocyte
VAT_2M	2561.5	1621	0.3915214	2	Singlet	FAPs
VAT_2M	6706.8	2784	0.046736	0	Singlet	Adipocyte
VAT_2M	8759.8	3048	0.0492703	0	Singlet	Adipocyte
VAT_2M	3124.5	1880	0.5530816	2	Singlet	FAPs
VAT_2M	5893.9	2657	0.7846933	0	Singlet	Adipocyte
VAT_2M	3097.7	1655	0.0420503	0	Singlet	Adipocyte
VAT_2M	2039.2	1409	3.4504083	2	Singlet	FAPs
VAT_2M	1443.8	1173	0.55071	7	Singlet	Endothelial_cells
VAT_2M	10608	3484	0.2217598	0	Singlet	Adipocyte
VAT_2M	3191.5	1845	0.090703	0	Singlet	Adipocyte
VAT_2M	1021.5	841	1.473601	11	Singlet	Smooth_muscle_cells
VAT_2M	1014.7	804	3.1633378	5	Singlet	Immune_cells
VAT_2M	2149.9	1495	0.8153546	2	Singlet	FAPs
VAT_2M	6842.9	2646	0.0578386	0	Singlet	Adipocyte
VAT_2M	2126.1	1313	0.7176829	7	Singlet	Endothelial_cells
VAT_2M	3497.3	1813	0.6153059	0	Singlet	Adipocyte
VAT_2M	2509.2	1390	0.8195401	0	Singlet	Adipocyte
VAT_2M	2293.3	1540	0.2699132	6	Singlet	Neurons
VAT_2M	1773.1	1226	0.1103953	2	Singlet	FAPs
VAT_2M	1673.9	1078	0.9780507	0	Singlet	Adipocyte
VAT_2M	8360	2965	0.0232307	0	Singlet	Adipocyte
VAT_2M	1249	893	0.6932178	7	Singlet	Endothelial_cells
VAT_2M	1449.6	1124	0.6111009	2	Singlet	FAPs
VAT_2M	2441.5	1378	2.9060563	0	Singlet	Adipocyte
VAT_2M	2219.3	1431	0.3562864	0	Singlet	Adipocyte
VAT_2M	2797.4	1774	1.5107089	3	Singlet	FAPs
VAT_2M	5051.6	2413	0.2339471	0	Singlet	Adipocyte
VAT_2M	4243.9	2032	0	0	Singlet	Adipocyte
VAT_2M	5240	2444	0.0803636	0	Singlet	Adipocyte
VAT_2M	3766.4	2054	0.6388265	0	Singlet	Adipocyte
VAT_2M	1040.2	833	0.7810948	2	Singlet	FAPs
VAT_2M	2598.1	1420	0.3309374	0	Singlet	Adipocyte
VAT_2M	1966.2	1394	0.9876321	2	Singlet	FAPs
VAT_2M	6968.6	2725	0.0392424	0	Singlet	Adipocyte

VAT_2M	1160	983	0.6611301	8 Singlet	Mesothelial
VAT_2M	1199.2	921	1.5616275	2 Singlet	FAPs
VAT_2M	3800	2043	0.5269593	0 Singlet	Adipocyte
VAT_2M	1388.8	1080	1.3058175	4 Singlet	Immune_cells
VAT_2M	2765.7	1432	0.7741726	0 Singlet	Adipocyte
VAT_2M	1417.8	948	2.4059762	2 Singlet	FAPs
VAT_2M	1080.3	914	0.7880351	8 Singlet	Mesothelial
VAT_2M	1911.7	1330	0.2403807	2 Singlet	FAPs
VAT_2M	2370.4	1401	0.9004848	0 Singlet	Adipocyte
VAT_2M	1872.9	1292	0.9165604	2 Singlet	FAPs
VAT_2M	1808.1	1115	0.2999179	0 Singlet	Adipocyte
VAT_2M	2242.9	1311	0.0328277	0 Singlet	Adipocyte
VAT_2M	1525.5	1107	0.4699189	2 Singlet	FAPs
VAT_2M	5933.8	2522	0.0314136	0 Singlet	Adipocyte
VAT_2M	1667.7	1204	1.4007461	3 Singlet	FAPs
VAT_2M	6397.9	2551	0.0039833	0 Singlet	Adipocyte
VAT_2M	1198.3	853	1.0255496	8 Singlet	Mesothelial
VAT_2M	1389.2	1039	1.1606263	2 Singlet	FAPs
VAT_2M	1040.8	726	0.8918676	5 Singlet	Immune_cells
VAT_2M	8504.7	3069	0.147116	0 Singlet	Adipocyte
VAT_2M	1300.1	929	0.508659	2 Singlet	FAPs
VAT_2M	1220.4	805	7.0929671	7 Singlet	Endothelial_cells
VAT_2M	1993.7	1417	0.7020949	5 Singlet	Immune_cells
VAT_2M	5626.8	2498	0.0781801	0 Singlet	Adipocyte
VAT_2M	1385.8	1001	0.3542037	3 Singlet	FAPs
VAT_2M	1545.8	1046	3.4004623	9 Singlet	Mesothelial
VAT_2M	4219.7	1901	0.1987161	0 Singlet	Adipocyte
VAT_2M	1650.2	1152	1.5440487	3 Singlet	FAPs
VAT_2M	1744.3	1139	0.8930035	5 Singlet	Immune_cells
VAT_2M	3394.8	1517	10.071942	7 Singlet	Endothelial_cells
VAT_2M	1342.9	1081	1.2990363	8 Singlet	Mesothelial
VAT_2M	3041.6	1834	0.7012302	2 Singlet	FAPs
VAT_2M	2045	1402	0.1584623	2 Singlet	FAPs
VAT_2M	1779.9	1253	0.788226	3 Singlet	FAPs
VAT_2M	2105.8	1429	1.0193627	0 Singlet	Adipocyte
VAT_2M	1149.4	780	2.8292502	7 Singlet	Endothelial_cells
VAT_2M	2182.3	1266	1.9637659	0 Singlet	Adipocyte
VAT_2M	1700.9	1181	2.8914688	2 Singlet	FAPs
VAT_2M	4013.6	1887	0.235672	0 Singlet	Adipocyte
VAT_2M	1063	805	0.229046	2 Singlet	FAPs
VAT_2M	1356.7	1105	1.4757761	1 Singlet	Immune_cells
VAT_2M	1021.5	886	1.2836389	6 Singlet	Neurons
VAT_2M	1155.7	954	2.6526164	0 Singlet	Adipocyte
VAT_2M	1599.2	1330	2.8333765	2 Singlet	FAPs
VAT_2M	1228.1	952	1.6849869	2 Singlet	FAPs
VAT_2M	3727.9	2074	0.0420563	0 Singlet	Adipocyte
VAT_2M	1748.4	1253	0.2407267	6 Singlet	Neurons
VAT_2M	7576	3069	0.0785591	0 Singlet	Adipocyte
VAT_2M	1745.7	1213	0.1753715	2 Singlet	FAPs
VAT_2M	7566.7	2980	0.025199	0 Singlet	Adipocyte
VAT_2M	1643.9	1272	0.1716282	5 Singlet	Immune_cells

VAT_2M	1625	992	0.2068493	0	Singlet	Adipocyte
VAT_2M	1820.9	1058	0.1610551	0	Singlet	Adipocyte
VAT_2M	2178.8	1368	1.0000733	0	Singlet	Adipocyte
VAT_2M	2612.3	1538	0.0267555	0	Singlet	Adipocyte
VAT_2M	11095	3604	1.7589013	0	Singlet	Adipocyte
VAT_2M	6601.6	2587	0.0410609	0	Singlet	Adipocyte
VAT_2M	2137.3	1274	0.2851224	3	Singlet	FAPs
VAT_2M	1322.6	974	1.825628	2	Singlet	FAPs
VAT_2M	1055.6	811	2.0843704	11	Singlet	Smooth_muscle_cells
VAT_2M	1399	1066	4.3404871	2	Singlet	FAPs
VAT_2M	2314.2	1361	0.4439372	0	Singlet	Adipocyte
VAT_2M	5110.1	2134	0.1723267	0	Singlet	Adipocyte
VAT_2M	1839.7	1155	1.9101675	2	Singlet	FAPs
VAT_2M	1406.8	1052	0.577529	3	Singlet	FAPs
VAT_2M	1740.2	1410	1.5784548	6	Singlet	Neurons
VAT_2M	6648.5	2708	0.0950956	0	Singlet	Adipocyte
VAT_2M	1459.8	1125	0.6419234	7	Singlet	Endothelial_cells
VAT_2M	6240	2484	0.1090577	0	Singlet	Adipocyte
VAT_2M	8570.1	3105	0.2559596	0	Singlet	Adipocyte
VAT_2M	2035.7	1343	0.202883	6	Singlet	Neurons
VAT_2M	1835.1	1458	1.514134	4	Singlet	Immune_cells
VAT_2M	2172.9	1338	0.0130025	0	Singlet	Adipocyte
VAT_2M	5612.2	2554	0.2133912	0	Singlet	Adipocyte
VAT_2M	5784.5	2413	0.171585	0	Singlet	Adipocyte
VAT_2M	2002.8	1272	0.1995167	0	Singlet	Adipocyte
VAT_2M	1192.2	799	0.3266935	2	Singlet	FAPs
VAT_2M	11433	3796	0.0253399	0	Singlet	Adipocyte
VAT_2M	1130.4	1004	1.5201634	1	Singlet	Immune_cells
VAT_2M	7789.7	2913	0.0823032	0	Singlet	Adipocyte
VAT_2M	2497.1	1762	0.4173666	2	Singlet	FAPs
VAT_2M	2337.3	1583	0.1697163	2	Singlet	FAPs
VAT_2M	7217.1	2763	0.4783893	0	Singlet	Adipocyte
VAT_2M	8170.5	3069	0.0033708	0	Singlet	Adipocyte
VAT_2M	6861.8	2886	0.0817523	0	Singlet	Adipocyte
VAT_2M	8534.3	3036	0	0	Singlet	Adipocyte
VAT_2M	1407.1	1008	1.8561069	3	Singlet	FAPs
VAT_2M	1378.1	1001	2.9052571	3	Singlet	FAPs
VAT_2M	3686.4	1879	1.3464388	0	Singlet	Adipocyte
VAT_2M	1040.1	788	2.4973609	2	Singlet	FAPs
VAT_2M	1226.9	924	0.2875657	5	Singlet	Immune_cells
VAT_2M	1457.8	1064	0.1303794	3	Singlet	FAPs
VAT_2M	7755.4	2972	0.0162455	0	Singlet	Adipocyte
VAT_2M	5097.6	2220	0.0723305	0	Singlet	Adipocyte
VAT_2M	1187.3	856	1.1189752	0	Singlet	Adipocyte
VAT_2M	10948	3475	0.4342664	0	Singlet	Adipocyte
VAT_2M	2050.7	1422	0.9601946	2	Singlet	FAPs
VAT_2M	1706.5	1162	1.3627007	9	Singlet	Mesothelial
VAT_2M	3455	1786	0.0175466	0	Singlet	Adipocyte
VAT_2M	8618.3	2836	0.1336416	0	Singlet	Adipocyte
VAT_2M	1464.9	1007	4.6448274	11	Singlet	Smooth_muscle_cells
VAT_2M	1591	1000	2.2588369	5	Singlet	Immune_cells

VAT_2M	1681.1	1430	1.6054728	6	Singlet	Neurons
VAT_2M	1886.9	1223	1.4587338	6	Singlet	Neurons
VAT_2M	6974.1	2838	0.00584	0	Singlet	Adipocyte
VAT_2M	10786	3312	0.0189375	0	Singlet	Adipocyte
VAT_2M	2028.8	1272	0.4059775	3	Singlet	FAPs
VAT_2M	4144.7	2022	0	0	Singlet	Adipocyte
VAT_2M	1005.3	885	1.0834463	8	Singlet	Mesothelial
VAT_2M	1462.3	1085	0.9509942	2	Singlet	FAPs
VAT_2M	2277.7	1543	0.3974371	2	Singlet	FAPs
VAT_2M	5780.9	2402	0.0088753	0	Singlet	Adipocyte
VAT_2M	1132.4	902	1.3781267	6	Singlet	Neurons
VAT_2M	1185.9	886	2.6526446	2	Singlet	FAPs
VAT_2M	1374	1012	3.9982037	2	Singlet	FAPs
VAT_2M	2776.8	1648	2.5027078	5	Singlet	Immune_cells
VAT_2M	1021.6	603	4.2126644	5	Singlet	Immune_cells
VAT_2M	3902.8	1830	0.5638234	0	Singlet	Adipocyte
VAT_2M	3223.6	1681	0.025291	0	Singlet	Adipocyte
VAT_2M	1037.1	766	0.7906276	2	Singlet	FAPs
VAT_2M	4986.7	2258	0.1015996	0	Singlet	Adipocyte
VAT_2M	3629.5	2123	1.8289819	3	Singlet	FAPs
VAT_2M	2611.8	1529	0.3687029	0	Singlet	Adipocyte
VAT_2M	1085.8	868	1.1047732	3	Singlet	FAPs
VAT_2M	1806.5	1075	7.3314812	7	Singlet	Endothelial_cells
VAT_2M	1056.2	802	4.3610296	5	Singlet	Immune_cells
VAT_2M	1544.9	1057	1.7886711	2	Singlet	FAPs
VAT_2M	1359	1126	0.6970915	11	Singlet	Smooth_muscle_cells
VAT_2M	3209.2	1663	0.0721644	0	Singlet	Adipocyte
VAT_2M	4491.6	2226	0.0446034	0	Singlet	Adipocyte
VAT_2M	2187.1	1485	1.5828098	6	Singlet	Neurons
VAT_2M	8345.5	3054	0.0280741	0	Singlet	Adipocyte
VAT_2M	1421.7	1032	1.2881537	6	Singlet	Neurons
VAT_2M	2020	1416	1.3064245	2	Singlet	FAPs
VAT_2M	1434.8	1025	0.5258293	6	Singlet	Neurons
VAT_2M	1597.7	1107	0.6250062	2	Singlet	FAPs
VAT_2M	1038.8	877	1.2290576	7	Singlet	Endothelial_cells
VAT_2M	2917	1788	1.1479645	0	Singlet	Adipocyte
VAT_2M	1291.1	953	3.1605173	6	Singlet	Neurons
VAT_2M	6161.4	2507	0.0121991	0	Singlet	Adipocyte
VAT_2M	1690.7	1339	1.0306659	4	Singlet	Immune_cells
VAT_2M	3567.4	1870	0.568452	0	Singlet	Adipocyte
VAT_2M	1170.3	863	0.3334091	3	Singlet	FAPs
VAT_2M	1997.6	1349	1.0457944	3	Singlet	FAPs
VAT_2M	1392.9	990	0.2439848	3	Singlet	FAPs
VAT_2M	3279.1	1855	0.561665	0	Singlet	Adipocyte
VAT_2M	9961.8	3493	0.0095506	0	Singlet	Adipocyte
VAT_2M	1242.8	866	1.7371931	2	Singlet	FAPs
VAT_2M	1110	815	0.9389578	9	Singlet	Mesothelial
VAT_2M	1881.8	1263	0.2844687	0	Singlet	Adipocyte
VAT_2M	2796.4	1803	1.7405407	2	Singlet	FAPs
VAT_2M	1610.4	1113	0.0952953	0	Singlet	Adipocyte
VAT_2M	1328.2	1045	0.0753097	7	Singlet	Endothelial_cells

VAT_2M	1355.6	939	1.3055113	0	Singlet	Adipocyte
VAT_2M	1721.5	1072	0.3457318	0	Singlet	Adipocyte
VAT_2M	1926.9	1203	0	3	Singlet	FAPs
VAT_2M	5489.8	2364	0.0088329	0	Singlet	Adipocyte
VAT_2M	6344.8	2596	0.0260542	0	Singlet	Adipocyte
VAT_2M	1578.2	1077	1.090745	6	Singlet	Neurons
VAT_2M	7092.7	2872	0.6302846	0	Singlet	Adipocyte
VAT_2M	3684.4	1902	1.2377436	5	Singlet	Immune_cells
VAT_2M	2623.1	1748	0.1491041	2	Singlet	FAPs
VAT_2M	1377.7	994	1.3151265	2	Singlet	FAPs
VAT_2M	6402.8	2667	0.3287578	0	Singlet	Adipocyte
VAT_2M	3950	2003	0.2699606	2	Singlet	FAPs
VAT_2M	7164.3	2762	0.0048515	0	Singlet	Adipocyte
VAT_2M	2874.9	1653	0.0885576	0	Singlet	Adipocyte
VAT_2M	2692.3	1697	0.8940789	2	Singlet	FAPs
VAT_2M	3771.7	1945	0.0617194	0	Singlet	Adipocyte
VAT_2M	2134.3	1228	0	0	Singlet	Adipocyte
VAT_2M	2077.3	1190	0.3734793	0	Singlet	Adipocyte
VAT_2M	1082.8	887	2.7972122	6	Singlet	Neurons
VAT_2M	9338.1	3196	0.0181815	0	Singlet	Adipocyte
VAT_2M	4274	2036	0.6766332	0	Singlet	Adipocyte
VAT_2M	1053.7	752	1.4672548	3	Singlet	FAPs
VAT_2M	1018.6	682	0.6920532	6	Singlet	Neurons
VAT_2M	1214.7	922	1.0350599	8	Singlet	Mesothelial
VAT_2M	1235	951	2.2476078	6	Singlet	Neurons
VAT_2M	1271.6	1010	0.9834984	6	Singlet	Neurons
VAT_2M	3854.9	2212	0.9169437	3	Singlet	FAPs
VAT_2M	4769.3	2268	0.2501245	0	Singlet	Adipocyte
VAT_2M	2692.3	1579	0.0522798	0	Singlet	Adipocyte
VAT_2M	2387.1	1565	1.3697702	2	Singlet	FAPs
VAT_2M	1999.3	1332	0.700774	0	Singlet	Adipocyte
VAT_2M	2510.2	1517	0.1692632	0	Singlet	Adipocyte
VAT_2M	1178.3	937	1.2405991	6	Singlet	Neurons
VAT_2M	1687.7	1051	1.3373479	0	Singlet	Adipocyte
VAT_2M	1247.8	1072	0.7801428	6	Singlet	Neurons
VAT_2M	1094.1	887	0.03752	5	Singlet	Immune_cells
VAT_2M	3611.1	1799	0.4470381	0	Singlet	Adipocyte
VAT_2M	6814.9	2611	0.0093346	0	Singlet	Adipocyte
VAT_2M	3140.2	1559	0.8154557	11	Singlet	Smooth_muscle_cells
VAT_2M	10987	3496	0.0138316	0	Singlet	Adipocyte
VAT_2M	10650	3439	0.4900269	0	Singlet	Adipocyte
VAT_2M	6812.7	2696	0.021547	0	Singlet	Adipocyte
VAT_2M	2511	1556	0.6262003	5	Singlet	Immune_cells
VAT_2M	1274.5	897	1.3019225	3	Singlet	FAPs
VAT_2M	7792.1	2918	0.0674306	0	Singlet	Adipocyte
VAT_2M	5383	2350	0.0309312	0	Singlet	Adipocyte
VAT_2M	2229	1329	5.7362378	2	Singlet	FAPs
VAT_2M	3052.1	1484	2.848191	2	Singlet	FAPs
VAT_2M	3009.5	1660	5.3322872	0	Singlet	Adipocyte
VAT_2M	1246.1	1072	0.3950012	6	Singlet	Neurons
VAT_2M	8816.7	3259	0.0144602	0	Singlet	Adipocyte

VAT_2M	5557.1	2340	0	0	Singlet	Adipocyte
VAT_2M	1882.2	1226	2.5922593	5	Singlet	Immune_cells
VAT_2M	2440.4	1363	0	0	Singlet	Adipocyte
VAT_2M	1108.8	722	0.4614992	3	Singlet	FAPs
VAT_2M	5179.8	2562	0.8584968	0	Singlet	Adipocyte
VAT_2M	4599.7	2192	0.1479561	0	Singlet	Adipocyte
VAT_2M	1795	1224	0.076484	7	Singlet	Endothelial_cells
VAT_2M	2682.1	1497	0.1671178	0	Singlet	Adipocyte
VAT_2M	2967.4	1553	0.8342189	0	Singlet	Adipocyte
VAT_2M	2137.2	1391	0.8060844	0	Singlet	Adipocyte
VAT_2M	1445.2	1040	0.2017293	3	Singlet	FAPs
VAT_2M	1747.5	1140	2.9411327	11	Singlet	Smooth_muscle_cells
VAT_2M	1312	981	0.8039125	2	Singlet	FAPs
VAT_2M	4406.5	2201	0.1872933	0	Singlet	Adipocyte
VAT_2M	6107.3	2488	0.0225491	0	Singlet	Adipocyte
VAT_2M	4768.2	2329	0.5820846	0	Singlet	Adipocyte
VAT_2M	1537.1	1078	1.4080962	2	Singlet	FAPs
VAT_2M	2083.1	1385	0.4678186	0	Singlet	Adipocyte
VAT_2M	1468	1012	0.0124262	3	Singlet	FAPs
VAT_2M	2726.1	1664	0.2843972	2	Singlet	FAPs
VAT_2M	2036.1	1384	0.7646167	3	Singlet	FAPs
VAT_2M	1643	1292	2.325261	2	Singlet	FAPs
VAT_2M	1959.2	1293	0.1632985	6	Singlet	Neurons
VAT_2M	6681.8	2695	0.0136457	0	Singlet	Adipocyte
VAT_2M	1738.2	1240	1.9629087	2	Singlet	FAPs
VAT_2M	3020.8	1902	0.2572894	2	Singlet	FAPs
VAT_2M	1881.4	1254	3.1267052	6	Singlet	Neurons
VAT_2M	1632.6	987	5.1676286	0	Singlet	Adipocyte
VAT_2M	1576.4	1052	3.2335639	2	Singlet	FAPs
VAT_2M	1180.3	888	3.5911959	9	Singlet	Mesothelial
VAT_2M	5855.6	2563	0.0134521	0	Singlet	Adipocyte
VAT_2M	5108.3	2260	0.031236	0	Singlet	Adipocyte
VAT_2M	6579.3	2508	0.0448332	0	Singlet	Adipocyte
VAT_2M	3226.1	1759	0.2981754	0	Singlet	Adipocyte
VAT_2M	1012	817	3.7358222	1	Singlet	Immune_cells
VAT_2M	1291.7	1001	1.042255	11	Singlet	Smooth_muscle_cells
VAT_2M	3190.7	1790	1.149673	5	Singlet	Immune_cells
VAT_2M	3950.9	1941	0.3524211	0	Singlet	Adipocyte
VAT_2M	2420.4	1419	0.9667416	3	Singlet	FAPs
VAT_2M	2010.9	1385	0.4809169	6	Singlet	Neurons
VAT_2M	1794.5	1167	0.7691223	2	Singlet	FAPs
VAT_2M	8246.6	3091	0.0038627	0	Singlet	Adipocyte
VAT_2M	2886.9	1835	2.4876609	3	Singlet	FAPs
VAT_2M	1244.4	955	0.1167095	3	Singlet	FAPs
VAT_2M	2148.8	1300	0.4069201	0	Singlet	Adipocyte
VAT_2M	1164.1	797	0.1141384	6	Singlet	Neurons
VAT_2M	4095.3	2040	0.0702706	0	Singlet	Adipocyte
VAT_2M	1479.8	1069	1.965277	0	Singlet	Adipocyte
VAT_2M	1272.1	1034	2.1300012	4	Singlet	Immune_cells
VAT_2M	3853	1962	0.0915047	0	Singlet	Adipocyte
VAT_2M	6767.8	2786	0.4399975	0	Singlet	Adipocyte

VAT_2M	3693.1	1873	0.0613115	0	Singlet	Adipocyte
VAT_2M	1506.5	1067	0.4605727	2	Singlet	FAPs
VAT_2M	2263.9	1435	0.6094063	0	Singlet	Adipocyte
VAT_2M	1410.2	1153	2.1541162	9	Singlet	Mesothelial
VAT_2M	1936.9	1199	0.2413432	0	Singlet	Adipocyte
VAT_2M	1623.2	1135	0.0067874	3	Singlet	FAPs
VAT_2M	1636.1	1211	0.1754722	2	Singlet	FAPs
VAT_2M	3313.1	1618	1.5894022	0	Singlet	Adipocyte
VAT_2M	1243.6	736	1.7371483	9	Singlet	Mesothelial
VAT_2M	5091.4	2229	0.0060199	0	Singlet	Adipocyte
VAT_2M	1150.8	867	1.5500708	2	Singlet	FAPs
VAT_2M	7750.5	2918	0.1337417	0	Singlet	Adipocyte
VAT_2M	2946.8	1828	4.9542347	7	Singlet	Endothelial_cells
VAT_2M	13737	3877	0.021826	0	Singlet	Adipocyte
VAT_2M	1196.3	878	0.9984261	2	Singlet	FAPs
VAT_2M	8290.9	2964	0.0903083	0	Singlet	Adipocyte
VAT_2M	7685.6	3072	0.0434027	0	Singlet	Adipocyte
VAT_2M	7217.9	2912	0.056027	0	Singlet	Adipocyte
VAT_2M	5962.2	2562	0.1743188	0	Singlet	Adipocyte
VAT_2M	5778.3	2453	0.0294041	0	Singlet	Adipocyte
VAT_2M	4308.9	2131	0.4762164	0	Singlet	Adipocyte
VAT_2M	2739	1578	0.4452916	3	Singlet	FAPs
VAT_2M	3080.7	1967	0	2	Singlet	FAPs
VAT_2M	1784.7	1134	0.6475198	0	Singlet	Adipocyte
VAT_2M	3365.3	1773	0.1273888	0	Singlet	Adipocyte
VAT_2M	2944.6	1689	1.5491912	0	Singlet	Adipocyte
VAT_2M	3266	1680	0.7535403	0	Singlet	Adipocyte
VAT_2M	1441.7	1010	0.5897721	5	Singlet	Immune_cells
VAT_2M	1524	1049	0.2670361	5	Singlet	Immune_cells
VAT_2M	1076.6	853	0.7377235	1	Singlet	Immune_cells
VAT_2M	1338.8	952	1.4238856	9	Singlet	Mesothelial
VAT_2M	2371.4	1396	0.2958799	3	Singlet	FAPs
VAT_2M	3925.9	2194	1.3885866	3	Singlet	FAPs
VAT_2M	5939.5	2643	0.1625616	0	Singlet	Adipocyte
VAT_2M	6517.6	2724	0.0296373	0	Singlet	Adipocyte
VAT_2M	1549.9	1042	0.5129595	7	Singlet	Endothelial_cells
VAT_2M	5815.2	2414	0.1103383	0	Singlet	Adipocyte
VAT_2M	2805.3	1513	0.2788182	0	Singlet	Adipocyte
VAT_2M	1778	1147	1.0664008	2	Singlet	FAPs
VAT_2M	1519.8	1052	2.0374598	6	Singlet	Neurons
VAT_2M	1959.6	1378	1.6736438	9	Singlet	Mesothelial
VAT_2M	4311.3	2107	0.1640529	0	Singlet	Adipocyte
VAT_2M	1834.3	1292	0.5292848	2	Singlet	FAPs
VAT_2M	6848.8	2949	0.055651	0	Singlet	Adipocyte
VAT_2M	1222.3	903	0.9454739	3	Singlet	FAPs
VAT_2M	1851.2	1122	3.4203093	2	Singlet	FAPs
VAT_2M	2460.6	1272	2.1307335	2	Singlet	FAPs
VAT_2M	1576	1135	1.8869593	5	Singlet	Immune_cells
VAT_2M	1542.7	990	0.8742391	0	Singlet	Adipocyte
VAT_2M	2340.5	1489	0.1357818	0	Singlet	Adipocyte
VAT_2M	4077.2	2150	0.6154357	0	Singlet	Adipocyte

VAT_2M	4372.4	2065	0.060867	0	Singlet	Adipocyte
VAT_2M	2574.3	1417	0.181966	0	Singlet	Adipocyte
VAT_2M	11341	3528	0.0293124	0	Singlet	Adipocyte
VAT_2M	1539.9	1179	0.5133371	2	Singlet	FAPs
VAT_2M	1113.3	805	0.7213196	2	Singlet	FAPs
VAT_2M	1286.4	1113	0.4374843	6	Singlet	Neurons
VAT_2M	1314.1	919	0.7393689	7	Singlet	Endothelial_cells
VAT_2M	1343.1	928	0.5760426	6	Singlet	Neurons
VAT_2M	2040.8	1278	0.4736705	0	Singlet	Adipocyte
VAT_2M	1675.6	1079	3.9577697	0	Singlet	Adipocyte
VAT_2M	5310.5	2400	0.1090274	0	Singlet	Adipocyte
VAT_2M	1898.9	1318	1.7849514	6	Singlet	Neurons
VAT_2M	1518.3	1050	1.2453989	3	Singlet	FAPs
VAT_2M	4525.1	2201	0.3013423	0	Singlet	Adipocyte
VAT_2M	1925.8	1115	0.1296251	3	Singlet	FAPs
VAT_2M	1464	1013	1.631455	2	Singlet	FAPs
VAT_2M	5816.4	2573	0.1317377	0	Singlet	Adipocyte
VAT_2M	4753.1	2262	0.0327476	0	Singlet	Adipocyte
VAT_2M	1012.3	843	0.8089617	6	Singlet	Neurons
VAT_2M	3727.6	1772	0.1875023	0	Singlet	Adipocyte
VAT_2M	5067.8	2388	0.0095105	0	Singlet	Adipocyte
VAT_2M	2914	1790	0.3444501	2	Singlet	FAPs
VAT_2M	2262.1	1467	1.2067825	2	Singlet	FAPs
VAT_2M	1128.1	871	1.5811097	6	Singlet	Neurons
VAT_2M	2576.4	1478	1.0501113	2	Singlet	FAPs
VAT_2M	2048.1	1192	1.5815555	0	Singlet	Adipocyte
VAT_2M	5246.9	2323	0.0054814	0	Singlet	Adipocyte
VAT_2M	2720.8	1575	0.1024722	2	Singlet	FAPs
VAT_2M	5296.2	2496	0.2147574	0	Singlet	Adipocyte
VAT_2M	1971.1	1312	0.6524602	7	Singlet	Endothelial_cells
VAT_2M	2057.6	1378	0.14594	6	Singlet	Neurons
VAT_2M	2093.2	1397	0.7515436	6	Singlet	Neurons
VAT_2M	1079.2	905	0.2726183	7	Singlet	Endothelial_cells
VAT_2M	6866.5	2955	0.2087661	0	Singlet	Adipocyte
VAT_2M	1220.5	845	0.8379073	6	Singlet	Neurons
VAT_2M	1879.5	1348	0.341489	0	Singlet	Adipocyte
VAT_2M	5134.3	2339	0.0057378	0	Singlet	Adipocyte
VAT_2M	7836.2	3007	0	0	Singlet	Adipocyte
VAT_2M	1631.4	1201	0.2959143	7	Singlet	Endothelial_cells
VAT_2M	1172.4	976	1.993605	1	Singlet	Immune_cells
VAT_2M	4073.3	1883	0.4884706	0	Singlet	Adipocyte
VAT_2M	1997.2	1230	2.874859	3	Singlet	FAPs
VAT_2M	2101	1302	0.1555201	2	Singlet	FAPs
VAT_2M	1081.4	801	0.7877407	6	Singlet	Neurons
VAT_2M	1630.5	1164	0.9108074	2	Singlet	FAPs
VAT_2M	2033.5	1433	0.4380388	0	Singlet	Adipocyte
VAT_2M	6301.4	2555	0.0382308	0	Singlet	Adipocyte
VAT_2M	5512.9	2471	0.3577671	0	Singlet	Adipocyte
VAT_2M	1027.3	875	2.2437443	1	Singlet	Immune_cells
VAT_2M	1551.3	1120	0.845611	3	Singlet	FAPs
VAT_2M	3397.7	1758	0.0639474	0	Singlet	Adipocyte

VAT_2M	3770.7	1987	0.1032914	0	Singlet	Adipocyte
VAT_2M	1196	864	2.5173452	6	Singlet	Neurons
VAT_2M	3775.9	2050	0.6503521	3	Singlet	FAPs
VAT_2M	1812.3	1319	0.2603407	2	Singlet	FAPs
VAT_2M	1119.7	769	0.4559339	3	Singlet	FAPs
VAT_2M	6619.7	2682	0.0653196	0	Singlet	Adipocyte
VAT_2M	1333.8	1075	0.550216	2	Singlet	FAPs
VAT_2M	4082.9	1910	0	0	Singlet	Adipocyte
VAT_2M	1995.4	1283	0.5469575	3	Singlet	FAPs
VAT_2M	3448.3	1701	0.1067255	0	Singlet	Adipocyte
VAT_2M	8260.1	3009	0.1352881	0	Singlet	Adipocyte
VAT_2M	2659.2	1462	1.396768	0	Singlet	Adipocyte
VAT_2M	6182.1	2678	0.0048166	0	Singlet	Adipocyte
VAT_2M	1822	1233	0.1546516	6	Singlet	Neurons
VAT_2M	6885.8	2746	0.3797596	0	Singlet	Adipocyte
VAT_2M	1129.8	874	0.5129579	9	Singlet	Mesothelial
VAT_2M	1496.1	1048	0.8799018	2	Singlet	FAPs
VAT_2M	3125	1744	0.0845907	0	Singlet	Adipocyte
VAT_2M	1263.2	1023	1.0890882	7	Singlet	Endothelial_cells
VAT_2M	11317	3615	9.08E-05	0	Singlet	Adipocyte
VAT_2M	2574.4	1456	1.5147639	0	Singlet	Adipocyte
VAT_2M	4778.3	2200	0.0196151	0	Singlet	Adipocyte
VAT_2M	1330.3	936	3.6004368	3	Singlet	FAPs
VAT_2M	6449.5	2829	0.0885448	0	Singlet	Adipocyte
VAT_2M	3054	1569	0.0214303	0	Singlet	Adipocyte
VAT_2M	4054.7	2283	0.3672294	2	Singlet	FAPs
VAT_2M	1667.6	1066	0.1265573	2	Singlet	FAPs
VAT_2M	6795.8	2692	0.0518146	0	Singlet	Adipocyte
VAT_2M	1859.5	1055	1.8216124	0	Singlet	Adipocyte
VAT_2M	2383.9	1384	0.0905145	0	Singlet	Adipocyte
VAT_2M	1179.2	846	0.6835741	3	Singlet	FAPs
VAT_2M	8531.7	3101	0.0379758	0	Singlet	Adipocyte
VAT_2M	3009.1	1533	0.1435649	0	Singlet	Adipocyte
VAT_2M	2104.5	1376	1.0619062	3	Singlet	FAPs
VAT_2M	2207.9	1494	3.6497305	5	Singlet	Immune_cells
VAT_2M	8883.7	3117	0.0457643	0	Singlet	Adipocyte
VAT_2M	1350.3	1006	3.316564	3	Singlet	FAPs
VAT_2M	1982.7	1145	0.1078418	0	Singlet	Adipocyte
VAT_2M	1301.5	989	0.2112214	3	Singlet	FAPs
VAT_2M	1749	1144	1.0015778	6	Singlet	Neurons
VAT_2M	2080.8	1335	0.8097955	5	Singlet	Immune_cells
VAT_2M	1160.8	954	2.9007446	2	Singlet	FAPs
VAT_2M	4321.7	2176	0.1905639	0	Singlet	Adipocyte
VAT_2M	8873.4	3171	0.1100885	0	Singlet	Adipocyte
VAT_2M	2627.5	1577	0.8230808	0	Singlet	Adipocyte
VAT_2M	6772.2	2636	0.1555322	0	Singlet	Adipocyte
VAT_2M	8225.4	2970	0.0109534	0	Singlet	Adipocyte
VAT_2M	8321.1	2967	0	0	Singlet	Adipocyte
VAT_2M	2094.9	1349	0.9826324	0	Singlet	Adipocyte
VAT_2M	5825.7	2433	0.0132852	0	Singlet	Adipocyte
VAT_2M	1910.8	1632	0.1372708	6	Singlet	Neurons

VAT_2M	1790.3	1052	0.6616014	0	Singlet	Adipocyte
VAT_2M	3065.7	1768	0.3342631	5	Singlet	Immune_cells
VAT_2M	6233.3	2467	1.9136015	0	Singlet	Adipocyte
VAT_2M	4196.9	2141	0.1189601	0	Singlet	Adipocyte
VAT_2M	3683.5	2005	0.1072484	0	Singlet	Adipocyte
VAT_2M	1212.9	929	0.4047058	3	Singlet	FAPs
VAT_2M	11810	3622	0.0269222	0	Singlet	Adipocyte
VAT_2M	3878.5	2002	1.0842222	0	Singlet	Adipocyte
VAT_2M	4823.1	2267	0.0097165	0	Singlet	Adipocyte
VAT_2M	1071.3	823	0.483967	3	Singlet	FAPs
VAT_2M	1790.3	1136	0.0661936	0	Singlet	Adipocyte
VAT_2M	1160.9	813	0.2557761	5	Singlet	Immune_cells
VAT_2M	3126.7	1847	1.428364	2	Singlet	FAPs
VAT_2M	4466.5	2344	0.4069814	5	Singlet	Immune_cells
VAT_2M	1845.4	1207	0.2932371	0	Singlet	Adipocyte
VAT_2M	3819.1	1858	0.1642949	0	Singlet	Adipocyte
VAT_2M	7800.5	3035	0.7273595	0	Singlet	Adipocyte
VAT_2M	3043.6	1667	0.7943948	3	Singlet	FAPs
VAT_2M	2113.8	1333	1.6770062	0	Singlet	Adipocyte
VAT_2M	8111.9	3265	0.1850379	0	Singlet	Adipocyte
VAT_2M	1155.7	865	2.1224123	9	Singlet	Mesothelial
VAT_2M	7021.9	2744	0.0105282	0	Singlet	Adipocyte
VAT_2M	4805	2327	0	0	Singlet	Adipocyte
VAT_2M	7922.7	3021	0.0574914	0	Singlet	Adipocyte
VAT_2M	1461.5	1110	0.2414988	5	Singlet	Immune_cells
VAT_2M	2295.3	1289	0.4914283	0	Singlet	Adipocyte
VAT_2M	1432.3	1021	2.3048147	2	Singlet	FAPs
VAT_2M	7651.2	2968	0.1549582	0	Singlet	Adipocyte
VAT_2M	1485.3	1186	0.8810164	2	Singlet	FAPs
VAT_2M	1541.2	1144	0.4367243	2	Singlet	FAPs
VAT_2M	2211.1	1539	1.1116717	6	Singlet	Neurons
VAT_2M	8349.2	2876	0.1379567	0	Singlet	Adipocyte
VAT_2M	1143	938	0.8634141	6	Singlet	Neurons
VAT_2M	4604.9	2149	0.1187359	0	Singlet	Adipocyte
VAT_2M	2783.8	1756	1.4628861	3	Singlet	FAPs
VAT_2M	3795.3	2008	0.0306162	0	Singlet	Adipocyte
VAT_2M	1996.4	1241	0.9113289	0	Singlet	Adipocyte
VAT_2M	5518.1	2790	4.2629621	7	Singlet	Endothelial_cells
VAT_2M	4602.1	2187	0.1218548	0	Singlet	Adipocyte
VAT_2M	1603.7	1154	1.5082082	7	Singlet	Endothelial_cells
VAT_2M	5682.9	2480	0.0893599	0	Singlet	Adipocyte
VAT_2M	1484.2	1140	1.1423646	6	Singlet	Neurons
VAT_2M	7557.5	2947	0.0663886	0	Singlet	Adipocyte
VAT_2M	2796.1	1748	2.0250819	3	Singlet	FAPs
VAT_2M	2643.4	1504	0.0025573	0	Singlet	Adipocyte
VAT_2M	1714.1	1487	0.7863621	6	Singlet	Neurons
VAT_2M	2244.5	1401	0.341414	5	Singlet	Immune_cells
VAT_2M	3272.8	1726	1.8520622	0	Singlet	Adipocyte
VAT_2M	1574.1	1121	2.4429277	9	Singlet	Mesothelial
VAT_2M	6535.7	2615	0.0039711	0	Singlet	Adipocyte
VAT_2M	6194.5	2599	0.0590916	0	Singlet	Adipocyte

VAT_2M	2233.5	1299	0.1777544	0	Singlet	Adipocyte
VAT_2M	1304.5	906	3.0887334	1	Singlet	Immune_cells
VAT_2M	1346.1	962	0.1426782	8	Singlet	Mesothelial
VAT_2M	3013.9	1593	0.8223676	0	Singlet	Adipocyte
VAT_2M	2959.5	1651	0.507215	0	Singlet	Adipocyte
VAT_2M	1017.3	760	0.1288797	5	Singlet	Immune_cells
VAT_2M	3907	2076	0.043337	0	Singlet	Adipocyte
VAT_2M	1132.2	854	0.1873265	5	Singlet	Immune_cells
VAT_2M	7718.9	2935	0.0582704	0	Singlet	Adipocyte
VAT_2M	8315.4	3128	0.3066555	0	Singlet	Adipocyte
VAT_2M	2191.8	1348	0.4026875	7	Singlet	Endothelial_cells
VAT_2M	9481.8	3069	0.0244806	0	Singlet	Adipocyte
VAT_2M	1683.1	1150	0.755111	5	Singlet	Immune_cells
VAT_2M	1198	866	1.2305158	3	Singlet	FAPs
VAT_2M	2976.7	1654	0.1405621	0	Singlet	Adipocyte
VAT_2M	1005.5	811	2.5840719	4	Singlet	Immune_cells
VAT_2M	1529.9	1096	2.1616624	3	Singlet	FAPs
VAT_2M	7699.6	2957	0.0416818	0	Singlet	Adipocyte
VAT_2M	2288.1	1568	0.2390092	0	Singlet	Adipocyte
VAT_2M	7562.7	2878	0.2305275	0	Singlet	Adipocyte
VAT_2M	2866.9	1594	0.2139061	0	Singlet	Adipocyte
VAT_2M	3826.9	1910	0.0384008	0	Singlet	Adipocyte
VAT_2M	2183.2	1452	0.0714831	0	Singlet	Adipocyte
VAT_2M	2573.2	1538	0.7275986	0	Singlet	Adipocyte
VAT_2M	8229.2	2985	0.0044715	0	Singlet	Adipocyte
VAT_2M	3670.5	2022	0.8073435	7	Singlet	Endothelial_cells
VAT_2M	2361.1	1472	2.2163235	0	Singlet	Adipocyte
VAT_2M	3231.2	1716	0.1536732	0	Singlet	Adipocyte
VAT_2M	5473.1	2466	0.0818007	0	Singlet	Adipocyte
VAT_2M	4877.6	2202	0	0	Singlet	Adipocyte
VAT_2M	1466	1042	0.6319835	3	Singlet	FAPs
VAT_2M	8075.2	3221	0.3072903	0	Singlet	Adipocyte
VAT_2M	1789.4	1351	2.9060403	6	Singlet	Neurons
VAT_2M	1482.6	1142	0.4847158	2	Singlet	FAPs
VAT_2M	5925.6	2508	0.0084992	0	Singlet	Adipocyte
VAT_2M	8572.1	3057	0.0031194	0	Singlet	Adipocyte
VAT_2M	5821.8	2495	0.0555457	0	Singlet	Adipocyte
VAT_2M	1013.3	780	6.4318602	6	Singlet	Neurons
VAT_2M	2852	1601	0.0846326	0	Singlet	Adipocyte
VAT_2M	1678.5	1234	1.9759822	5	Singlet	Immune_cells
VAT_2M	1639.4	1254	1.8596312	2	Singlet	FAPs
VAT_2M	1636.5	1108	0.5440598	6	Singlet	Neurons
VAT_2M	9536.9	3166	0.0176102	0	Singlet	Adipocyte
VAT_2M	5700.6	2540	0.0620912	0	Singlet	Adipocyte
VAT_2M	3981.9	1935	0.1212889	0	Singlet	Adipocyte
VAT_2M	9124.2	2990	0.1301157	0	Singlet	Adipocyte
VAT_2M	2960.8	1602	0.2312073	0	Singlet	Adipocyte
VAT_2M	1593.1	1122	1.2439521	2	Singlet	FAPs
VAT_2M	4906.7	2295	0.2297631	0	Singlet	Adipocyte
VAT_2M	2068.7	1301	10.79631	9	Singlet	Mesothelial
VAT_2M	2468.7	1490	0.3111808	0	Singlet	Adipocyte

VAT_2M	5947.6	2924	2.0672488	3	Singlet	FAPs
VAT_2M	7079.2	2832	0.4661895	0	Singlet	Adipocyte
VAT_2M	3614.2	2150	0.6859264	2	Singlet	FAPs
VAT_2M	1368	992	5.0876645	8	Singlet	Mesothelial
VAT_2M	2698.8	1555	0.7659348	0	Singlet	Adipocyte
VAT_2M	1565.8	1191	2.1476925	6	Singlet	Neurons
VAT_2M	1139.2	910	2.7680226	6	Singlet	Neurons
VAT_2M	2799.8	1514	0	0	Singlet	Adipocyte
VAT_2M	1690.1	1179	4.2031651	6	Singlet	Neurons
VAT_2M	1074.9	888	2.3188442	6	Singlet	Neurons
VAT_2M	1304	967	1.0345868	2	Singlet	FAPs
VAT_2M	3640.8	1906	0	0	Singlet	Adipocyte
VAT_2M	1494.1	1196	1.4458808	5	Singlet	Immune_cells
VAT_2M	9418.6	3028	0.0031088	0	Singlet	Adipocyte
VAT_2M	4976.3	2201	0.017072	0	Singlet	Adipocyte
VAT_2M	10928	3456	0.1259121	0	Singlet	Adipocyte
VAT_2M	1760.2	1218	0.5962655	3	Singlet	FAPs
VAT_2M	3856.9	1855	0.400641	0	Singlet	Adipocyte
VAT_2M	1148.5	862	1.2864449	9	Singlet	Mesothelial
VAT_2M	1903.4	1104	0.187459	0	Singlet	Adipocyte
VAT_2M	1065.2	799	7.7299208	6	Singlet	Neurons
VAT_2M	3207.6	1816	0.0596913	0	Singlet	Adipocyte
VAT_2M	7282.4	2615	0.3370998	0	Singlet	Adipocyte
VAT_2M	10076	3239	0	0	Singlet	Adipocyte
VAT_2M	8717.3	3039	0	0	Singlet	Adipocyte
VAT_2M	2436.6	1478	0.6831396	3	Singlet	FAPs
VAT_2M	1781.9	693	14.439735	0	Singlet	Adipocyte
VAT_2M	2252.9	1436	1.1342053	3	Singlet	FAPs
VAT_2M	9731.8	3216	0.0183553	0	Singlet	Adipocyte
VAT_2M	1558.8	1278	0.0035083	7	Singlet	Endothelial_cells
VAT_2M	6788.9	2840	0.0375811	0	Singlet	Adipocyte
VAT_2M	1724	965	9.0133056	0	Singlet	Adipocyte
VAT_2M	1075.3	798	0.3508365	3	Singlet	FAPs
VAT_2M	2479	1612	0.8605001	5	Singlet	Immune_cells
VAT_2M	5145.6	2291	0.0085448	0	Singlet	Adipocyte
VAT_2M	2514.9	1585	0.2415482	2	Singlet	FAPs
VAT_2M	1209.3	852	1.4352908	7	Singlet	Endothelial_cells
VAT_2M	2927.1	1651	0.8624535	0	Singlet	Adipocyte
VAT_2M	1379.6	1127	0.6115001	6	Singlet	Neurons
VAT_2M	4715.7	2265	0.1089911	0	Singlet	Adipocyte
VAT_2M	4213.3	2042	0.0299128	0	Singlet	Adipocyte
VAT_2M	5125.2	2497	0.045517	0	Singlet	Adipocyte
VAT_2M	2355.8	1577	1.1257452	5	Singlet	Immune_cells
VAT_2M	4847.4	2233	0.0284472	0	Singlet	Adipocyte
VAT_2M	1034.6	867	1.0134583	6	Singlet	Neurons
VAT_2M	3105.2	1694	0.1143844	0	Singlet	Adipocyte
VAT_2M	1271.4	1004	1.7566764	5	Singlet	Immune_cells
VAT_2M	3922.4	1991	0.2330226	0	Singlet	Adipocyte
VAT_2M	3965.5	2001	0.1823431	0	Singlet	Adipocyte
VAT_2M	2063.5	1292	0.1538988	3	Singlet	FAPs
VAT_2M	1756.8	1262	0.5793868	6	Singlet	Neurons

VAT_2M	1119	778	2.3353929	2	Singlet	FAPs
VAT_2M	7804	2995	0.6622709	0	Singlet	Adipocyte
VAT_2M	5342.4	2303	0.0446052	0	Singlet	Adipocyte
VAT_2M	1894.4	1255	1.4662063	5	Singlet	Immune_cells
VAT_2M	2388.8	1494	0.0572392	7	Singlet	Endothelial_cells
VAT_2M	2155.3	1432	2.2193779	5	Singlet	Immune_cells
VAT_2M	2283.9	1453	0.4066015	6	Singlet	Neurons
VAT_2M	2330.8	1455	0.0900898	3	Singlet	FAPs
VAT_2M	5784.8	2533	0	0	Singlet	Adipocyte
VAT_2M	1371.4	966	1.1022177	2	Singlet	FAPs
VAT_2M	2110.7	1430	1.5996593	6	Singlet	Neurons
VAT_2M	1582.7	1146	0.2641782	2	Singlet	FAPs
VAT_2M	2562.3	1536	0.3754463	0	Singlet	Adipocyte
VAT_2M	1159.4	919	0.9548638	0	Singlet	Adipocyte
VAT_2M	3033.9	1592	0.1574668	0	Singlet	Adipocyte
VAT_2M	8391	3211	0.0128657	0	Singlet	Adipocyte
VAT_2M	6234.6	2701	0.208274	0	Singlet	Adipocyte
VAT_2M	2547.1	1641	0.0783219	2	Singlet	FAPs
VAT_2M	1016.7	829	1.2977531	8	Singlet	Mesothelial
VAT_2M	3926.5	1921	0.5227142	0	Singlet	Adipocyte
VAT_2M	3649.4	2023	0.8612633	2	Singlet	FAPs
VAT_2M	3951.5	2030	0	0	Singlet	Adipocyte
VAT_2M	4188.9	2040	0.6379225	0	Singlet	Adipocyte
VAT_2M	2384.2	1596	0.1290712	6	Singlet	Neurons
VAT_2M	4204.2	2097	0.1261318	0	Singlet	Adipocyte
VAT_2M	2370.5	1340	4.5557003	0	Singlet	Adipocyte
VAT_2M	6216.4	2675	0.0048023	0	Singlet	Adipocyte
VAT_2M	1393.7	1030	1.3156909	3	Singlet	FAPs
VAT_2M	8307.3	2949	0.0020125	0	Singlet	Adipocyte
VAT_2M	1594.2	1259	1.4605005	9	Singlet	Mesothelial
VAT_2M	6996	2800	0.3531803	0	Singlet	Adipocyte
VAT_2M	1777.6	1266	1.0365934	2	Singlet	FAPs
VAT_2M	4038.3	2225	2.5649685	2	Singlet	FAPs
VAT_2M	1572.7	983	0.0803059	7	Singlet	Endothelial_cells
VAT_2M	3358.8	1805	0.4290306	2	Singlet	FAPs
VAT_2M	2150.8	1379	1.180832	7	Singlet	Endothelial_cells
VAT_2M	1617.7	1146	2.8189371	3	Singlet	FAPs
VAT_2M	1297.9	1056	1.7337325	11	Singlet	Smooth_muscle_cells
VAT_2M	8202.1	2866	0.0627857	0	Singlet	Adipocyte
VAT_2M	1222.6	904	2.6416916	6	Singlet	Neurons
VAT_2M	2324	1486	0.9089144	5	Singlet	Immune_cells
VAT_2M	6462.8	2901	0.0548521	0	Singlet	Adipocyte
VAT_2M	2874.8	1461	1.5584721	0	Singlet	Adipocyte
VAT_2M	3781.9	1957	0.5141208	0	Singlet	Adipocyte
VAT_2M	2804.6	1627	0.0247601	0	Singlet	Adipocyte
VAT_2M	3393.8	1814	0.0679176	0	Singlet	Adipocyte
VAT_2M	1279	887	0.5278971	2	Singlet	FAPs
VAT_2M	5333.1	2491	0	0	Singlet	Adipocyte
VAT_2M	6940.6	2807	0.1520398	0	Singlet	Adipocyte
VAT_2M	8055.2	3081	0.0159465	0	Singlet	Adipocyte
VAT_2M	2670	1516	4.8456308	0	Singlet	Adipocyte

VAT_2M	1359	1003	2.1675065	6	Singlet	Neurons
VAT_2M	2964.7	1627	0.1624876	0	Singlet	Adipocyte
VAT_2M	6823.2	2648	0.0045339	0	Singlet	Adipocyte
VAT_2M	1371.9	1024	0.7549164	2	Singlet	FAPs
VAT_2M	1923.1	1608	1.2214345	6	Singlet	Neurons
VAT_2M	5423.5	2221	0.1816096	0	Singlet	Adipocyte
VAT_2M	1863.4	1014	0.0978578	0	Singlet	Adipocyte
VAT_2M	1681.7	1130	1.3525613	2	Singlet	FAPs
VAT_2M	3695.3	1919	0.4780381	0	Singlet	Adipocyte
VAT_2M	2920.4	1681	0.3709351	0	Singlet	Adipocyte
VAT_2M	5411.5	2424	0.2024295	0	Singlet	Adipocyte
VAT_2M	1570.9	1000	0.5834353	6	Singlet	Neurons
VAT_2M	2036.6	1331	0.0896952	0	Singlet	Adipocyte
VAT_2M	1524.8	1072	2.2675366	2	Singlet	FAPs
VAT_2M	4941.1	2301	0.0314493	0	Singlet	Adipocyte
VAT_2M	1320.4	950	0.5726698	3	Singlet	FAPs
VAT_2M	1285.7	960	0.2926092	3	Singlet	FAPs
VAT_2M	4808.3	2248	0.3572124	0	Singlet	Adipocyte
VAT_2M	3002.7	1769	0.2826267	0	Singlet	Adipocyte
VAT_2M	1630.5	1172	0.5449551	2	Singlet	FAPs
VAT_2M	1753	1261	1.4435135	2	Singlet	FAPs
VAT_2M	1720.6	1199	0.5832613	6	Singlet	Neurons
VAT_2M	8341	3058	0.0669979	0	Singlet	Adipocyte
VAT_2M	5306.7	2447	0.5480098	0	Singlet	Adipocyte
VAT_2M	2150.8	1374	0.0943687	3	Singlet	FAPs
VAT_2M	1358.5	995	1.7205477	2	Singlet	FAPs
VAT_2M	6887.6	2750	0	0	Singlet	Adipocyte
VAT_2M	1606.3	1138	0.1794493	3	Singlet	FAPs
VAT_2M	5753.6	2413	0.0673645	0	Singlet	Adipocyte
VAT_2M	4330.3	2143	0.2289726	0	Singlet	Adipocyte
VAT_2M	1887.5	1158	0.1347866	0	Singlet	Adipocyte
VAT_2M	2000.8	1204	0.5154952	0	Singlet	Adipocyte
VAT_2M	2204.7	1418	0.3152781	6	Singlet	Neurons
VAT_2M	5920.8	2426	0.1134331	0	Singlet	Adipocyte
VAT_2M	4729.1	2238	0	0	Singlet	Adipocyte
VAT_2M	1598.6	1119	1.334607	7	Singlet	Endothelial_cells
VAT_2M	3150.3	1903	1.3470133	5	Singlet	Immune_cells
VAT_2M	2123.2	1548	1.0950149	3	Singlet	FAPs
VAT_2M	1176.2	836	0.6276871	6	Singlet	Neurons
VAT_2M	5817.9	2292	0.0296108	0	Singlet	Adipocyte
VAT_2M	2256	1428	0.572659	0	Singlet	Adipocyte
VAT_2M	3050.8	1735	0.2371152	0	Singlet	Adipocyte
VAT_2M	1904.4	1291	1.1930789	3	Singlet	FAPs
VAT_2M	6085.8	2653	0.0214468	0	Singlet	Adipocyte
VAT_2M	1316	1094	0.7948373	2	Singlet	FAPs
VAT_2M	1784	943	2.5439502	11	Singlet	Smooth_muscle_cells
VAT_2M	3007.5	1591	0.1042799	0	Singlet	Adipocyte
VAT_2M	1758.7	1291	1.0323014	2	Singlet	FAPs
VAT_2M	1302.4	977	2.3179143	9	Singlet	Mesothelial
VAT_2M	1242.5	923	1.4281447	3	Singlet	FAPs
VAT_2M	1260.8	952	1.7794573	3	Singlet	FAPs

VAT_2M	1115.2	922	0.9556816	6	Singlet	Neurons
VAT_2M	5192.2	2156	0.0568483	0	Singlet	Adipocyte
VAT_2M	4199.2	2171	0.0567815	0	Singlet	Adipocyte
VAT_2M	3068.3	1915	1.0791556	5	Singlet	Immune_cells
VAT_2M	6355.1	2713	0.0968364	0	Singlet	Adipocyte
VAT_2M	3948.9	1964	0	0	Singlet	Adipocyte
VAT_2M	3836.6	1977	0.3892873	0	Singlet	Adipocyte
VAT_2M	4777.4	2159	0	0	Singlet	Adipocyte
VAT_2M	7401.7	2772	0.1548249	0	Singlet	Adipocyte
VAT_2M	3498.5	1113	5.5702047	7	Singlet	Endothelial_cells
VAT_2M	1935.1	1066	4.1573658	0	Singlet	Adipocyte
VAT_2M	1801.8	940	5.5051101	0	Singlet	Adipocyte
VAT_2M	6129.8	2503	0.5897541	0	Singlet	Adipocyte
VAT_2M	8206.1	3052	0.0131202	0	Singlet	Adipocyte
VAT_2M	9556	3175	0.0203225	0	Singlet	Adipocyte
VAT_2M	1358.1	957	2.6518736	5	Singlet	Immune_cells
VAT_2M	8258.9	3089	0.0018334	0	Singlet	Adipocyte
VAT_2M	5581.9	2350	0.6789869	0	Singlet	Adipocyte
VAT_2M	1120.9	920	2.0501048	1	Singlet	Immune_cells
VAT_2M	5878.7	2621	0.013694	0	Singlet	Adipocyte
VAT_2M	7755.6	2833	0.0090827	0	Singlet	Adipocyte
VAT_2M	2877.2	1589	0.5823477	0	Singlet	Adipocyte
VAT_2M	3408.5	1669	0.0160636	0	Singlet	Adipocyte
VAT_2M	2244.4	1554	0.744858	6	Singlet	Neurons
VAT_2M	1402.9	1174	2.2088981	6	Singlet	Neurons
VAT_2M	6658.7	2803	0.05274	0	Singlet	Adipocyte
VAT_2M	1158.1	860	0.5165466	7	Singlet	Endothelial_cells
VAT_2M	1006	683	11.789969	2	Singlet	FAPs
VAT_2M	1201.2	885	2.7775956	4	Singlet	Immune_cells
VAT_2M	1938.1	1323	0.6893337	2	Singlet	FAPs
VAT_2M	1027.6	885	1.2741601	6	Singlet	Neurons
VAT_2M	1566.6	1094	0.3315891	5	Singlet	Immune_cells
VAT_2M	2056	1340	0.5821601	3	Singlet	FAPs
VAT_2M	2879	1566	0.4382862	0	Singlet	Adipocyte
VAT_2M	9340.2	3076	0.0282673	0	Singlet	Adipocyte
VAT_2M	6238.6	2819	0.3381258	0	Singlet	Adipocyte
VAT_2M	5672.3	2460	0.0380603	0	Singlet	Adipocyte
VAT_2M	7666.3	2929	0.8045164	0	Singlet	Adipocyte
VAT_2M	8224.6	3104	0.2329954	0	Singlet	Adipocyte
VAT_2M	3937.2	1841	0.1462299	0	Singlet	Adipocyte
VAT_2M	1085	832	1.0788729	1	Singlet	Immune_cells
VAT_2M	5110.3	2392	0.055581	0	Singlet	Adipocyte
VAT_2M	2214.9	1529	0.899737	3	Singlet	FAPs
VAT_2M	2822.8	1620	3.9399558	5	Singlet	Immune_cells
VAT_2M	8411.2	3137	0.0015094	0	Singlet	Adipocyte
VAT_2M	7373.6	2831	0.0617486	0	Singlet	Adipocyte
VAT_2M	3626	2061	1.842408	7	Singlet	Endothelial_cells
VAT_2M	1685.7	1228	0.1244801	7	Singlet	Endothelial_cells
VAT_2M	8742.3	3217	0.0230207	0	Singlet	Adipocyte
VAT_2M	1499.7	1212	1.7838747	11	Singlet	Smooth_muscle_cells
VAT_2M	1281.5	853	1.8277137	2	Singlet	FAPs

VAT_2M	3054.9	1651	1.1999139	3	Singlet	FAPs
VAT_2M	1188.2	924	0.8499444	7	Singlet	Endothelial_cells
VAT_2M	1866.2	977	8.1255141	0	Singlet	Adipocyte
VAT_2M	1549.8	1036	2.3640881	2	Singlet	FAPs
VAT_2M	1015.9	793	0.6510594	7	Singlet	Endothelial_cells
VAT_2M	3273.9	1854	0.0588662	0	Singlet	Adipocyte
VAT_2M	3031.6	1664	0	0	Singlet	Adipocyte
VAT_2M	2393.7	1426	6.0861082	5	Singlet	Immune_cells
VAT_2M	3065.3	1912	0.9255374	2	Singlet	FAPs
VAT_2M	9808.3	3280	0.0088992	0	Singlet	Adipocyte
VAT_2M	1499.4	1074	2.327594	5	Singlet	Immune_cells
VAT_2M	2883.2	1503	0.2815701	0	Singlet	Adipocyte
VAT_2M	1909.3	1288	8.8477356	4	Singlet	Immune_cells
VAT_2M	1387	1046	1.1155481	11	Singlet	Smooth_muscle_cells
VAT_2M	6326.1	2585	0.002014	0	Singlet	Adipocyte
VAT_2M	7003.6	2821	0.0204079	0	Singlet	Adipocyte
VAT_2M	2521.6	1453	0.4244596	3	Singlet	FAPs
VAT_2M	1933.8	1061	0.8778709	0	Singlet	Adipocyte
VAT_2M	1744.6	1217	1.0227464	6	Singlet	Neurons
VAT_2M	2191.8	1487	0.5692277	6	Singlet	Neurons
VAT_2M	2047.5	1208	0.7509964	0	Singlet	Adipocyte
VAT_2M	1959.3	1128	0.2994137	0	Singlet	Adipocyte
VAT_2M	5121.9	2366	0	0	Singlet	Adipocyte
VAT_2M	1362.8	792	8.9381728	5	Singlet	Immune_cells
VAT_2M	2170.9	1663	5.2109849	11	Singlet	Smooth_muscle_cells
VAT_2M	10827	3486	0.0779176	0	Singlet	Adipocyte
VAT_2M	7442	2949	0.0168461	0	Singlet	Adipocyte
VAT_2M	1068	907	0.1957163	6	Singlet	Neurons
VAT_2M	3911.5	2085	0.2099683	0	Singlet	Adipocyte
VAT_2M	7113.7	2801	0.0348555	0	Singlet	Adipocyte
VAT_2M	2691.4	1576	0.3354623	0	Singlet	Adipocyte
VAT_2M	4786.7	2323	0.0893871	0	Singlet	Adipocyte
VAT_2M	2466.4	1433	0.0463239	0	Singlet	Adipocyte
VAT_2M	8493.6	3066	1.6275204	0	Singlet	Adipocyte
VAT_2M	2352.2	1350	0.033517	0	Singlet	Adipocyte
VAT_2M	6865.3	2681	0.3221957	0	Singlet	Adipocyte
VAT_2M	1337	1028	2.1923123	8	Singlet	Mesothelial
VAT_2M	1452.9	984	1.3613708	2	Singlet	FAPs
VAT_2M	6963.5	2864	0.3760295	0	Singlet	Adipocyte
VAT_2M	4222.8	2314	4.9387893	2	Singlet	FAPs
VAT_2M	2770.9	1583	0.0596233	11	Singlet	Smooth_muscle_cells
VAT_2M	2257.9	1418	0.9866334	6	Singlet	Neurons
VAT_2M	1789.7	1228	0.8671466	2	Singlet	FAPs
VAT_2M	3103.8	1663	0.0661197	0	Singlet	Adipocyte
VAT_2M	2288.6	1485	1.5271291	5	Singlet	Immune_cells
VAT_2M	1095.9	903	2.1301206	8	Singlet	Mesothelial
VAT_2M	3341.7	1819	0.4657634	0	Singlet	Adipocyte
VAT_2M	2098.6	1274	0.2552601	3	Singlet	FAPs
VAT_2M	1551.5	978	1.0377255	3	Singlet	FAPs
VAT_2M	4712.9	2196	0.1045698	0	Singlet	Adipocyte
VAT_2M	8518.8	2942	0.0621486	0	Singlet	Adipocyte

VAT_2M	6030.9	2558	0.0222803	0	Singlet	Adipocyte
VAT_2M	4769.8	2397	0.2042355	0	Singlet	Adipocyte
VAT_2M	5936.8	2501	0	0	Singlet	Adipocyte
VAT_2M	2370.7	1456	1.0305697	0	Singlet	Adipocyte
VAT_2M	5369.8	2460	0.0084425	0	Singlet	Adipocyte
VAT_2M	1660.2	976	2.6899515	0	Singlet	Adipocyte
VAT_2M	3476.5	1903	0.8588023	2	Singlet	FAPs
VAT_2M	6194.1	2544	0.0973296	0	Singlet	Adipocyte
VAT_2M	3066.7	1676	0.2276102	0	Singlet	Adipocyte
VAT_2M	1133.9	827	0.3279365	5	Singlet	Immune_cells
VAT_2M	1150.3	830	0.1949614	7	Singlet	Endothelial_cells
VAT_2M	2309.4	1330	0.402014	0	Singlet	Adipocyte
VAT_2M	8865.6	3044	0	0	Singlet	Adipocyte
VAT_2M	1699.2	1229	0.1183377	3	Singlet	FAPs
VAT_2M	1449.5	1076	1.6034497	9	Singlet	Mesothelial
VAT_2M	2581.5	1473	1.0665062	0	Singlet	Adipocyte
VAT_2M	1102.7	882	0.183885	2	Singlet	FAPs
VAT_2M	5863.3	2471	0.0612503	0	Singlet	Adipocyte
VAT_2M	3468.1	1984	0.2010918	0	Singlet	Adipocyte
VAT_2M	3334.3	1818	0.0357676	0	Singlet	Adipocyte
VAT_2M	1654.6	1234	0.6564002	8	Singlet	Mesothelial
VAT_2M	6341.8	2737	0.3211905	0	Singlet	Adipocyte
VAT_2M	1060.1	908	1.0717484	6	Singlet	Neurons
VAT_2M	1364.9	974	1.3320076	7	Singlet	Endothelial_cells
VAT_2M	2507.9	1498	0.6796142	0	Singlet	Adipocyte
VAT_2M	4035.2	2080	0.1530259	0	Singlet	Adipocyte
VAT_2M	9838.9	3327	0.0272752	0	Singlet	Adipocyte
VAT_2M	3889.2	1986	0.0658984	0	Singlet	Adipocyte
VAT_2M	1061.4	832	0.4853807	6	Singlet	Neurons
VAT_2M	6839.9	2746	0.0046664	0	Singlet	Adipocyte
VAT_2M	1994.6	1246	0.1074766	0	Singlet	Adipocyte
VAT_2M	1323.9	1082	1.171918	10	Singlet	Immune_cells
VAT_2M	1912.6	1121	0	0	Singlet	Adipocyte
VAT_2M	1664.7	1179	0.43077	5	Singlet	Immune_cells
VAT_2M	8392.9	3167	1.53E-05	0	Singlet	Adipocyte
VAT_2M	4661.6	2118	0.1251359	0	Singlet	Adipocyte
VAT_2M	2102.6	1316	0.1657308	0	Singlet	Adipocyte
VAT_2M	3902.3	2015	0.1807509	0	Singlet	Adipocyte
VAT_2M	3081.7	1716	0.1745177	0	Singlet	Adipocyte
VAT_2M	2066.6	1275	0.2633576	0	Singlet	Adipocyte
VAT_2M	3716	2110	1.1060183	0	Singlet	Adipocyte
VAT_2M	3802.4	1995	0.0537307	0	Singlet	Adipocyte
VAT_2M	1478.6	1163	1.4385361	6	Singlet	Neurons
VAT_2M	3105.8	1706	0.0299783	0	Singlet	Adipocyte
VAT_2M	1525.4	1081	1.3535407	7	Singlet	Endothelial_cells
VAT_2M	1976.5	1247	0.2973903	0	Singlet	Adipocyte
VAT_2M	1823	1201	0	0	Singlet	Adipocyte
VAT_2M	2207.5	1281	0.5505722	0	Singlet	Adipocyte
VAT_2M	4705.3	2250	0.2041884	0	Singlet	Adipocyte
VAT_2M	6834.3	2824	0.095387	0	Singlet	Adipocyte
VAT_2M	5583.8	2552	0	0	Singlet	Adipocyte

VAT_2M	3474.4	1810	0.057337	0	Singlet	Adipocyte
VAT_2M	5264.1	2389	0	0	Singlet	Adipocyte
VAT_2M	1045.4	790	1.8193256	9	Singlet	Mesothelial
VAT_2M	1896.5	1339	1.6186678	9	Singlet	Mesothelial
VAT_2M	7325.1	2892	0.0885602	0	Singlet	Adipocyte
VAT_2M	1056.3	798	4.8821274	3	Singlet	FAPs
VAT_2M	4842.8	2361	0.10821	0	Singlet	Adipocyte
VAT_2M	1823.1	1329	0.6427346	2	Singlet	FAPs
VAT_2M	4739.4	2112	0.130073	0	Singlet	Adipocyte
VAT_2M	9066.2	3296	0.1569851	0	Singlet	Adipocyte
VAT_2M	1228.5	898	0.5550914	5	Singlet	Immune_cells
VAT_2M	1062.4	828	1.7784276	5	Singlet	Immune_cells
VAT_2M	2999.7	1579	0.1350536	0	Singlet	Adipocyte
VAT_2M	4278.8	2134	0	0	Singlet	Adipocyte
VAT_2M	6644.5	2605	0.0281342	0	Singlet	Adipocyte
VAT_2M	1253.8	915	0.2047361	7	Singlet	Endothelial_cells
VAT_2M	2424.8	1749	1.2209815	2	Singlet	FAPs
VAT_2M	1692.4	1107	0.0051016	5	Singlet	Immune_cells
VAT_2M	2884.5	1634	0.0426105	0	Singlet	Adipocyte
VAT_2M	2437.5	1496	0.3055109	0	Singlet	Adipocyte
VAT_2M	1653.8	1142	0.0832474	0	Singlet	Adipocyte
VAT_2M	1175.3	883	0.3367011	5	Singlet	Immune_cells
VAT_2M	8228.5	2973	0.0018944	0	Singlet	Adipocyte
VAT_2M	4027.6	1864	0.0528353	0	Singlet	Adipocyte
VAT_2M	1011.4	823	0.1411307	3	Singlet	FAPs
VAT_2M	4346	2201	0.5446944	0	Singlet	Adipocyte
VAT_2M	1025.2	711	0.9031073	7	Singlet	Endothelial_cells
VAT_2M	1923.1	1196	0.1838213	0	Singlet	Adipocyte
VAT_2M	5987.9	2516	0.4261333	0	Singlet	Adipocyte
VAT_2M	6195.1	2500	0.0672328	0	Singlet	Adipocyte
VAT_2M	1193.4	961	0.0844591	5	Singlet	Immune_cells
VAT_2M	1897.7	1336	3.0335605	3	Singlet	FAPs
VAT_2M	2445.3	1470	0.3562316	3	Singlet	FAPs
VAT_2M	5985.2	2652	0.0122183	0	Singlet	Adipocyte
VAT_2M	3393.4	1774	0.3867477	0	Singlet	Adipocyte
VAT_2M	1393.2	895	12.262085	9	Singlet	Mesothelial
VAT_2M	1135.2	905	0.3606494	2	Singlet	FAPs
VAT_2M	1587.9	1124	0.5577407	3	Singlet	FAPs
VAT_2M	2368.1	1356	0.1294257	0	Singlet	Adipocyte
VAT_2M	3317.1	1742	0.0194668	0	Singlet	Adipocyte
VAT_2M	1789.4	1262	0.3363725	2	Singlet	FAPs
VAT_2M	1020.1	756	1.3678395	2	Singlet	FAPs
VAT_2M	2416.4	1331	0.2580696	0	Singlet	Adipocyte
VAT_2M	1346.3	1117	0.1254022	4	Singlet	Immune_cells
VAT_2M	1245.1	1005	2.1601662	6	Singlet	Neurons
VAT_2M	1435.4	1028	0.0888341	2	Singlet	FAPs
VAT_2M	9354.2	3274	0.0444508	0	Singlet	Adipocyte
VAT_2M	4132.6	2142	0.1478707	0	Singlet	Adipocyte
VAT_2M	1162.5	882	0.3443051	2	Singlet	FAPs
VAT_2M	7796.5	2965	0.0112881	0	Singlet	Adipocyte
VAT_2M	1074.6	765	1.4665187	5	Singlet	Immune_cells

VAT_2M	1385.7	1044	0.2554208	5	Singlet	Immune_cells
VAT_2M	2043.6	1408	0.6812412	5	Singlet	Immune_cells
VAT_2M	9489.9	3230	0.0700126	0	Singlet	Adipocyte
VAT_2M	3518.3	1826	0.0936789	0	Singlet	Adipocyte
VAT_2M	1961.9	1403	0.6543354	0	Singlet	Adipocyte
VAT_2M	5017.3	2306	0	0	Singlet	Adipocyte
VAT_2M	1559.5	1128	0.7179383	9	Singlet	Mesothelial
VAT_2M	4534.3	1996	0.0223067	0	Singlet	Adipocyte
VAT_2M	2646.1	1519	1.5654286	2	Singlet	FAPs
VAT_2M	3743.5	1925	0	0	Singlet	Adipocyte
VAT_2M	9332.8	3236	0.0020947	0	Singlet	Adipocyte
VAT_2M	6356.4	2672	0.0484017	0	Singlet	Adipocyte
VAT_2M	6607	2735	0.0423689	0	Singlet	Adipocyte
VAT_2M	5386.8	2279	0.0321126	0	Singlet	Adipocyte
VAT_2M	1371.6	970	0.8465659	7	Singlet	Endothelial_cells
VAT_2M	8740.5	3132	0.0024654	0	Singlet	Adipocyte
VAT_2M	1620.5	1222	0.7835303	2	Singlet	FAPs
VAT_2M	4352	2237	0.0511781	0	Singlet	Adipocyte
VAT_2M	1832.3	1211	0.3762462	5	Singlet	Immune_cells
VAT_2M	9014.8	3250	0.0103697	0	Singlet	Adipocyte
VAT_2M	1037.4	731	0.3935939	7	Singlet	Endothelial_cells
VAT_2M	1396.2	1025	2.5080881	2	Singlet	FAPs
VAT_2M	2145.6	1611	6.3171923	2	Singlet	FAPs
VAT_2M	2725.4	1535	0.0675205	0	Singlet	Adipocyte
VAT_2M	1603.9	1069	0.0470815	0	Singlet	Adipocyte
VAT_2M	1482	990	8.930992	4	Singlet	Immune_cells
VAT_2M	2972.5	1548	1.2222417	0	Singlet	Adipocyte
VAT_2M	2311.6	1314	0.0235103	0	Singlet	Adipocyte
VAT_2M	1817.2	1249	2.5822463	2	Singlet	FAPs
VAT_2M	2284.9	1479	0.0472968	0	Singlet	Adipocyte
VAT_2M	2629.4	1677	0.2405103	3	Singlet	FAPs
VAT_2M	1000.8	899	0.5773409	1	Singlet	Immune_cells
VAT_2M	3241.8	1691	0.0708233	0	Singlet	Adipocyte
VAT_2M	2114.9	1369	1.6194317	0	Singlet	Adipocyte
VAT_2M	2559.7	1524	0.3227704	2	Singlet	FAPs
VAT_2M	1747.7	1220	0.8456005	6	Singlet	Neurons
VAT_2M	1131.4	833	0	2	Singlet	FAPs
VAT_2M	3118.7	1738	1.9145529	2	Singlet	FAPs
VAT_2M	7844.3	3152	0.4300672	0	Singlet	Adipocyte
VAT_2M	1103.1	890	3.0327609	1	Singlet	Immune_cells
VAT_2M	3286.2	1727	0.0169884	0	Singlet	Adipocyte
VAT_2M	1596	1146	1.1642112	2	Singlet	FAPs
VAT_2M	1719.8	1085	0	7	Singlet	Endothelial_cells
VAT_2M	1474.1	977	1.8946674	6	Singlet	Neurons
VAT_2M	1236.1	913	1.3550679	3	Singlet	FAPs
VAT_2M	2141	1309	0.0984425	0	Singlet	Adipocyte
VAT_2M	1121.8	789	0.8143771	2	Singlet	FAPs
VAT_2M	13428	3826	0.1129185	0	Singlet	Adipocyte
VAT_2M	5465.2	2509	0.1422599	0	Singlet	Adipocyte
VAT_2M	4736.9	2315	0.008754	0	Singlet	Adipocyte
VAT_2M	3784.1	1999	0.0617255	0	Singlet	Adipocyte

VAT_2M	3586.7	1786	0.0202511	0	Singlet	Adipocyte
VAT_2M	2673.8	1482	0.5614521	0	Singlet	Adipocyte
VAT_2M	11279	3603	0.6505866	0	Singlet	Adipocyte
VAT_2M	2388.4	1512	0.9274797	3	Singlet	FAPs
VAT_2M	2496.3	1386	0.0960031	0	Singlet	Adipocyte
VAT_2M	3272.8	1815	0.2370322	0	Singlet	Adipocyte
VAT_2M	1129.7	848	0.6916621	2	Singlet	FAPs
VAT_2M	1569.6	1004	0.7025762	0	Singlet	Adipocyte
VAT_2M	2104.8	1383	0.6136292	3	Singlet	FAPs
VAT_2M	2998.5	1548	5.0524684	5	Singlet	Immune_cells
VAT_2M	3081.9	1673	0.0751644	0	Singlet	Adipocyte
VAT_2M	3743.1	2080	0.5569159	3	Singlet	FAPs
VAT_2M	4666	2204	0.009899	0	Singlet	Adipocyte
VAT_2M	1271.8	1011	0.9872683	5	Singlet	Immune_cells
VAT_2M	2182.7	1410	0.6151409	0	Singlet	Adipocyte
VAT_2M	4958.8	2275	0.0941467	0	Singlet	Adipocyte
VAT_2M	2340.8	1582	4.4733029	9	Singlet	Mesothelial
VAT_2M	4123.8	1992	3.910783	0	Singlet	Adipocyte
VAT_2M	6568.7	2645	0.6896406	0	Singlet	Adipocyte
VAT_2M	6416.2	2657	0.0552708	0	Singlet	Adipocyte
VAT_2M	2763.1	1570	0.0624977	0	Singlet	Adipocyte
VAT_2M	1549	1164	0.6039746	11	Singlet	Smooth_muscle_cells
VAT_2M	1229.6	934	0.7966561	5	Singlet	Immune_cells
VAT_2M	7925.6	2853	0	0	Singlet	Adipocyte
VAT_2M	2830.6	1563	0.2711728	0	Singlet	Adipocyte
VAT_2M	1602.5	1064	5.9039138	0	Singlet	Adipocyte
VAT_2M	1767.4	1134	0.1843475	11	Singlet	Smooth_muscle_cells
VAT_2M	2729.1	1536	0.4079445	0	Singlet	Adipocyte
VAT_2M	3321.5	1821	0.0681253	0	Singlet	Adipocyte
VAT_2M	3106	1687	0.0276957	0	Singlet	Adipocyte
VAT_2M	7603.8	2971	0.0009472	0	Singlet	Adipocyte
VAT_2M	1818.7	1172	0.8661626	0	Singlet	Adipocyte
VAT_2M	1909.5	1281	3.4307856	0	Singlet	Adipocyte
VAT_2M	2166.5	1225	4.4637432	0	Singlet	Adipocyte
VAT_2M	1847.6	1158	1.0985323	0	Singlet	Adipocyte
VAT_2M	6850	2613	0.0208367	0	Singlet	Adipocyte
VAT_2M	1113.5	848	1.4303008	5	Singlet	Immune_cells
VAT_2M	1747	926	1.4360865	0	Singlet	Adipocyte
VAT_2M	6469.9	2543	0.1028298	0	Singlet	Adipocyte
VAT_2M	7899.6	3031	0.0024019	0	Singlet	Adipocyte
VAT_2M	3109.7	1690	0.2240573	0	Singlet	Adipocyte
VAT_2M	2462.5	1425	1.184738	0	Singlet	Adipocyte
VAT_2M	6400.9	2455	0.0424501	0	Singlet	Adipocyte
VAT_2M	8444.4	2877	0.0415969	0	Singlet	Adipocyte
VAT_2M	1816.3	1034	4.4126698	8	Singlet	Mesothelial
VAT_2M	2564.9	1634	0.0821472	0	Singlet	Adipocyte
VAT_2M	6595.7	2680	0.6968105	0	Singlet	Adipocyte
VAT_2M	6688.5	2732	0.0048025	0	Singlet	Adipocyte
VAT_2M	7126.8	2965	0.072631	0	Singlet	Adipocyte
VAT_2M	2192.2	1404	1.8358708	6	Singlet	Neurons
VAT_2M	2810.7	1702	0.3578958	0	Singlet	Adipocyte

VAT_2M	1729.6	1202	0.4581557	2	Singlet	FAPs
VAT_2M	5166.8	2435	0.4991536	0	Singlet	Adipocyte
VAT_2M	9161.6	3163	0.0752491	0	Singlet	Adipocyte
VAT_2M	1645	1109	0.472113	0	Singlet	Adipocyte
VAT_2M	1763.4	1293	0.4855446	2	Singlet	FAPs
VAT_2M	2349.3	1453	0.1107592	0	Singlet	Adipocyte
VAT_2M	1774.8	1268	2.6969805	7	Singlet	Endothelial_cells
VAT_2M	1792.3	1093	3.7441111	5	Singlet	Immune_cells
VAT_2M	2323.5	1342	0.4067907	0	Singlet	Adipocyte
VAT_2M	9140.9	3403	0.0456656	0	Singlet	Adipocyte
VAT_2M	1626.3	1076	0.1435301	0	Singlet	Adipocyte
VAT_2M	1133.9	932	1.2135787	6	Singlet	Neurons
VAT_2M	2068.4	1211	0.5252414	0	Singlet	Adipocyte
VAT_2M	5480	2479	0	0	Singlet	Adipocyte
VAT_2M	4350.3	2142	0.279387	0	Singlet	Adipocyte
VAT_2M	1287.3	964	4.4138941	2	Singlet	FAPs
VAT_2M	1039.9	799	0.1176352	3	Singlet	FAPs
VAT_2M	4059.2	1909	0.0297725	0	Singlet	Adipocyte
VAT_2M	7971	2971	0.2843178	0	Singlet	Adipocyte
VAT_2M	2335.3	1520	0.9779514	5	Singlet	Immune_cells
VAT_2M	1350	966	0.3568266	6	Singlet	Neurons
VAT_2M	2002.2	1281	0.6683973	0	Singlet	Adipocyte
VAT_2M	1471	1054	2.2302041	2	Singlet	FAPs
VAT_2M	1272.4	1003	2.2608407	5	Singlet	Immune_cells
VAT_2M	14491	3868	0.0013464	0	Singlet	Adipocyte
VAT_2M	1922.2	1171	0.4494461	0	Singlet	Adipocyte
VAT_2M	4517.6	2187	0.4356384	3	Singlet	FAPs
VAT_2M	1854.3	1100	0.1429849	0	Singlet	Adipocyte
VAT_2M	1036	720	1.7840172	2	Singlet	FAPs
VAT_2M	1955.3	1248	0.4300432	7	Singlet	Endothelial_cells
VAT_2M	1096.5	856	0.9108018	4	Singlet	Immune_cells
VAT_2M	4327.9	2047	0	0	Singlet	Adipocyte
VAT_2M	1290.1	1009	0.4417746	5	Singlet	Immune_cells
VAT_2M	1502.2	1058	0.7331465	2	Singlet	FAPs
VAT_2M	2475.2	1485	0.9058333	11	Singlet	Smooth_muscle_cells
VAT_2M	10295	3422	0.0225501	0	Singlet	Adipocyte
VAT_2M	2970.3	1625	0.6570272	0	Singlet	Adipocyte
VAT_2M	1382	911	0.0065782	7	Singlet	Endothelial_cells
VAT_2M	1659.6	974	6.9039603	7	Singlet	Endothelial_cells
VAT_2M	1215.9	937	1.9274141	2	Singlet	FAPs
VAT_2M	4710.5	2360	2.0168903	0	Singlet	Adipocyte
VAT_2M	3039.8	1746	0.2154874	3	Singlet	FAPs
VAT_2M	1905.8	1293	1.813478	3	Singlet	FAPs
VAT_2M	1527.2	1060	4.5015925	5	Singlet	Immune_cells
VAT_2M	1850.1	1527	3.5453796	6	Singlet	Neurons
VAT_2M	2999.5	1770	2.5272803	3	Singlet	FAPs
VAT_2M	1024.7	835	1.0610142	8	Singlet	Mesothelial
VAT_2M	5866.3	2557	0.2105302	0	Singlet	Adipocyte
VAT_2M	1258	977	2.2415348	3	Singlet	FAPs
VAT_2M	1161.1	922	1.6473019	6	Singlet	Neurons
VAT_2M	2351.1	1298	3.1596149	0	Singlet	Adipocyte

VAT_2M	3424.2	1917	0.9124931	0	Singlet	Adipocyte
VAT_2M	2856.1	1622	0	0	Singlet	Adipocyte
VAT_2M	1831.5	1218	1.5519916	7	Singlet	Endothelial_cells
VAT_2M	2539.1	1691	1.4206549	5	Singlet	Immune_cells
VAT_2M	5954.9	2540	0.0065389	0	Singlet	Adipocyte
VAT_2M	9220.5	3265	0.0032903	0	Singlet	Adipocyte
VAT_2M	2939.5	1834	0.0937555	3	Singlet	FAPs
VAT_2M	4983.6	2210	0.0243212	0	Singlet	Adipocyte
VAT_2M	1127.1	892	2.2725648	7	Singlet	Endothelial_cells
VAT_2M	1544.3	1109	1.150387	2	Singlet	FAPs
VAT_2M	4556.9	2419	0.1144956	0	Singlet	Adipocyte
VAT_2M	2573.4	1572	0.0677333	2	Singlet	FAPs
VAT_2M	12806	3830	0.1800719	0	Singlet	Adipocyte
VAT_2M	1021.5	736	2.1665667	6	Singlet	Neurons
VAT_2M	1026	760	0.2226366	5	Singlet	Immune_cells
VAT_2M	1789.7	1171	0.172395	0	Singlet	Adipocyte
VAT_2M	3333.4	1897	0.290391	0	Singlet	Adipocyte
VAT_2M	2814.7	1578	0	0	Singlet	Adipocyte
VAT_2M	3848.9	1948	1.3877585	0	Singlet	Adipocyte
VAT_2M	1561.5	909	3.0764936	6	Singlet	Neurons
VAT_2M	5799.1	2606	0.0151304	0	Singlet	Adipocyte
VAT_2M	9953.7	3469	0.0694546	0	Singlet	Adipocyte
VAT_2M	2951.3	1782	0.5878392	3	Singlet	FAPs
VAT_2M	2347.8	1559	0.1628464	11	Singlet	Smooth_muscle_cells
VAT_2M	1584.5	982	0	0	Singlet	Adipocyte
VAT_2M	12564	3758	0.0215832	0	Singlet	Adipocyte
VAT_2M	1396.5	1066	1.8087058	6	Singlet	Neurons
VAT_2M	4807.1	2170	0.0408119	0	Singlet	Adipocyte
VAT_2M	2167.8	1335	0.3451486	0	Singlet	Adipocyte
VAT_2M	4958.7	2410	0.9954023	0	Singlet	Adipocyte
VAT_2M	1209.2	895	2.7569137	5	Singlet	Immune_cells
VAT_2M	9505.4	3347	0.0026142	0	Singlet	Adipocyte
VAT_2M	1879.5	1334	1.5351751	6	Singlet	Neurons
VAT_2M	5745.7	2509	0.1618491	0	Singlet	Adipocyte
VAT_2M	2056.8	1265	0.4445126	0	Singlet	Adipocyte
VAT_2M	1591	1039	0.2748433	6	Singlet	Neurons
VAT_2M	2174.9	1481	1.6439093	2	Singlet	FAPs
VAT_2M	3791.2	1939	0.0723918	0	Singlet	Adipocyte
VAT_2M	2047.3	1251	1.0100069	2	Singlet	FAPs
VAT_2M	1170.8	1002	1.3862769	6	Singlet	Neurons
VAT_2M	6836	2762	0.0606249	0	Singlet	Adipocyte
VAT_2M	10544	3569	0.0171915	0	Singlet	Adipocyte
VAT_2M	3830.5	1962	0.0713043	0	Singlet	Adipocyte
VAT_2M	7516.5	2911	1.0667982	0	Singlet	Adipocyte
VAT_2M	1078.8	880	2.1309885	4	Singlet	Immune_cells
VAT_2M	3105.6	1763	0.6321048	3	Singlet	FAPs
VAT_2M	1113.5	789	1.6030752	2	Singlet	FAPs
VAT_2M	1340.7	1057	0.8540826	2	Singlet	FAPs
VAT_2M	1453.4	932	0.464403	0	Singlet	Adipocyte
VAT_2M	1485.1	1082	2.2585707	2	Singlet	FAPs
VAT_2M	3874.5	1993	0.0199414	0	Singlet	Adipocyte

VAT_2M	1169.6	816	0.8340916	5	Singlet	Immune_cells
VAT_2M	1900.7	1140	0	0	Singlet	Adipocyte
VAT_2M	1222.2	904	2.1972434	2	Singlet	FAPs
VAT_2M	3438.5	1826	0.6318057	0	Singlet	Adipocyte
VAT_2M	1895.8	1393	0.8736922	2	Singlet	FAPs
VAT_2M	6121.2	2532	0.25294	0	Singlet	Adipocyte
VAT_2M	2087	1295	2.6646259	0	Singlet	Adipocyte
VAT_2M	5937.9	2658	0.3727487	0	Singlet	Adipocyte
VAT_2M	7353.2	2926	0.0445875	0	Singlet	Adipocyte
VAT_2M	1010.7	791	1.1962671	7	Singlet	Endothelial_cells
VAT_2M	6574.4	2641	0.0206915	0	Singlet	Adipocyte
VAT_2M	2152.8	1438	0.4278917	5	Singlet	Immune_cells
VAT_2M	4998	2438	0.0345544	0	Singlet	Adipocyte
VAT_2M	4731.2	2375	0.4911881	0	Singlet	Adipocyte
VAT_2M	2359.4	1488	0.4062059	0	Singlet	Adipocyte
VAT_2M	2870.8	1601	0.8964553	0	Singlet	Adipocyte
VAT_2M	1858.6	1238	0.2762815	7	Singlet	Endothelial_cells
VAT_2M	1042.6	847	1.4485814	6	Singlet	Neurons
VAT_2M	1347.9	957	0.7127401	5	Singlet	Immune_cells
VAT_2M	2865	1604	0.2467991	0	Singlet	Adipocyte
VAT_2M	3305.4	1630	0.1240859	0	Singlet	Adipocyte
VAT_2M	4818.5	2080	0.0731307	0	Singlet	Adipocyte
VAT_2M	1933.8	1171	0.4752032	0	Singlet	Adipocyte
VAT_2M	3337.4	1751	0.2141738	0	Singlet	Adipocyte
VAT_2M	1283.3	938	4.8423091	2	Singlet	FAPs
VAT_2M	1701.1	1214	1.3594223	9	Singlet	Mesothelial
VAT_2M	1336.8	1045	1.5005404	2	Singlet	FAPs
VAT_2M	1051.6	814	0.5864397	5	Singlet	Immune_cells
VAT_2M	2965.2	1919	0.3311415	2	Singlet	FAPs
VAT_2M	5253.4	2458	0.4333441	0	Singlet	Adipocyte
VAT_2M	4773	2347	0.1786779	0	Singlet	Adipocyte
VAT_2M	1095	804	0.5503002	3	Singlet	FAPs
VAT_2M	1952.1	1321	0.7412286	6	Singlet	Neurons
VAT_2M	3302.6	1784	0.0731614	0	Singlet	Adipocyte
VAT_2M	1104.4	801	0.8482611	3	Singlet	FAPs
VAT_2M	6456.5	2753	2.3060056	0	Singlet	Adipocyte
VAT_2M	1210.7	897	0.3536824	0	Singlet	Adipocyte
VAT_2M	12024	3592	0	0	Singlet	Adipocyte
VAT_2M	2839.5	1721	2.1822776	0	Singlet	Adipocyte
VAT_2M	1156.5	860	0.4815843	7	Singlet	Endothelial_cells
VAT_2M	2153.7	1401	1.3831725	3	Singlet	FAPs
VAT_2M	1190.2	1013	0.9087818	1	Singlet	Immune_cells
VAT_2M	2104.5	1251	2.8030668	0	Singlet	Adipocyte
VAT_2M	1987.2	1365	0.3502186	2	Singlet	FAPs
VAT_2M	2066.5	1142	14.127615	7	Singlet	Endothelial_cells
VAT_2M	1043.7	704	0.9646578	1	Singlet	Immune_cells
VAT_2M	8530.9	3279	0.0029375	0	Singlet	Adipocyte
VAT_2M	1971.2	1224	0.710592	3	Singlet	FAPs
VAT_2M	2013.7	1332	0.0789233	3	Singlet	FAPs
VAT_2M	1238.6	832	0.2892265	0	Singlet	Adipocyte
VAT_2M	3184.8	1691	0.4803071	0	Singlet	Adipocyte

VAT_2M	8040.7	2919	0.019184	0	Singlet	Adipocyte
VAT_2M	2556.4	1514	0.1731848	3	Singlet	FAPs
VAT_2M	2238.5	1365	1.5688389	3	Singlet	FAPs
VAT_2M	1392.6	1007	1.1703117	3	Singlet	FAPs
VAT_2M	3520.9	2093	0.399627	2	Singlet	FAPs
VAT_2M	1840.4	1283	0.1980892	5	Singlet	Immune_cells
VAT_2M	1872.8	1325	1.9627388	9	Singlet	Mesothelial
VAT_2M	1613.2	1120	0.243565	2	Singlet	FAPs
VAT_2M	2488.5	1467	0.0741357	3	Singlet	FAPs
VAT_2M	8076	2834	0.0137699	0	Singlet	Adipocyte
VAT_2M	3953.5	2008	0.1688602	0	Singlet	Adipocyte
VAT_2M	3389	1813	0.1800659	0	Singlet	Adipocyte
VAT_2M	3469.4	2026	0	0	Singlet	Adipocyte
VAT_2M	1859.1	1165	0.573284	2	Singlet	FAPs
VAT_2M	7420.2	3114	0.1302	0	Singlet	Adipocyte
VAT_2M	1875	1175	1.2388831	6	Singlet	Neurons
VAT_2M	1077.3	901	1.875662	7	Singlet	Endothelial_cells
VAT_2M	2043	1357	0.9469216	3	Singlet	FAPs
VAT_2M	11104	3662	0.0589154	0	Singlet	Adipocyte
VAT_2M	3134.5	1662	0.0495254	0	Singlet	Adipocyte
VAT_2M	1037.7	743	2.2198766	2	Singlet	FAPs
VAT_2M	2768.2	1699	0.0588103	0	Singlet	Adipocyte
VAT_2M	5743.4	2530	0.4529373	0	Singlet	Adipocyte
VAT_2M	1578.6	1071	0.9590697	0	Singlet	Adipocyte
VAT_2M	4319.5	2360	0.4635301	2	Singlet	FAPs
VAT_2M	5105.4	2194	0.0641995	0	Singlet	Adipocyte
VAT_2M	1135.5	812	0.872009	2	Singlet	FAPs
VAT_2M	8016	3150	0.0499246	0	Singlet	Adipocyte
VAT_2M	1365.5	941	0.0320772	3	Singlet	FAPs
VAT_2M	1112.9	930	1.0125988	8	Singlet	Mesothelial
VAT_2M	1064.4	916	0.8863628	6	Singlet	Neurons
VAT_2M	4700.8	2221	0.0861408	0	Singlet	Adipocyte
VAT_2M	2383.8	1348	0.9647277	3	Singlet	FAPs
VAT_2M	6355.4	2694	0.096895	0	Singlet	Adipocyte
VAT_2M	1770.1	1281	1.1019637	2	Singlet	FAPs
VAT_2M	2427.6	1447	0.1780376	0	Singlet	Adipocyte
VAT_2M	2062.7	1395	0.1289687	2	Singlet	FAPs
VAT_2M	2015.6	1277	0.1741349	3	Singlet	FAPs
VAT_2M	1237.1	894	1.114999	6	Singlet	Neurons
VAT_2M	1484.6	1087	0.8282575	5	Singlet	Immune_cells
VAT_2M	2715.6	1507	0	0	Singlet	Adipocyte
VAT_2M	3582.5	1950	0	0	Singlet	Adipocyte
VAT_2M	4283.2	2152	0.0247677	0	Singlet	Adipocyte
VAT_2M	2115.9	1497	0.6468947	5	Singlet	Immune_cells
VAT_2M	9365.4	3326	0.012207	0	Singlet	Adipocyte
VAT_2M	1819.1	1164	0.5728539	0	Singlet	Adipocyte
VAT_2M	1558.2	1084	0.8277738	2	Singlet	FAPs
VAT_2M	6501.9	2744	0.0190708	0	Singlet	Adipocyte
VAT_2M	8591.9	3062	0.0145663	0	Singlet	Adipocyte
VAT_2M	8388.2	2981	0.0948582	0	Singlet	Adipocyte
VAT_2M	5415.5	2461	0	0	Singlet	Adipocyte

VAT_2M	2414.2	1750	0.2535304	2	Singlet	FAPs
VAT_2M	2246.9	1569	1.3135279	5	Singlet	Immune_cells
VAT_2M	2504	1440	0.8187801	0	Singlet	Adipocyte
VAT_2M	5565.2	2369	0.124037	0	Singlet	Adipocyte
VAT_2M	6544.2	2686	0.2328095	0	Singlet	Adipocyte
VAT_2M	1033.6	784	1.441701	3	Singlet	FAPs
VAT_2M	1516.5	1227	0.1849301	6	Singlet	Neurons
VAT_2M	8314	3112	0.0226756	0	Singlet	Adipocyte
VAT_2M	1839.3	1202	1.6997643	2	Singlet	FAPs
VAT_2M	1031.1	782	1.5122621	4	Singlet	Immune_cells
VAT_2M	4942.1	2378	0.0389336	0	Singlet	Adipocyte
VAT_2M	3698.9	2089	0.3956455	2	Singlet	FAPs
VAT_2M	2340.7	1522	0.0410135	3	Singlet	FAPs
VAT_2M	7523.8	2855	0.0019733	0	Singlet	Adipocyte
VAT_2M	3899.3	2070	0.0134784	0	Singlet	Adipocyte
VAT_2M	2782.7	1626	0.4318256	0	Singlet	Adipocyte
VAT_2M	3824.8	1838	0.0409352	0	Singlet	Adipocyte
VAT_2M	1905.4	1430	1.46889	2	Singlet	FAPs
VAT_2M	1315	900	2.8399349	2	Singlet	FAPs
VAT_2M	1394.2	1047	0.4488753	2	Singlet	FAPs
VAT_2M	2223.4	1373	0.0779488	0	Singlet	Adipocyte
VAT_2M	8771.3	3180	0.5550447	0	Singlet	Adipocyte
VAT_2M	3537.6	1995	1.2997474	0	Singlet	Adipocyte
VAT_2M	3967.7	1987	0.1700106	0	Singlet	Adipocyte
VAT_2M	9822	2830	0.0062874	0	Singlet	Adipocyte
VAT_2M	3966.3	1888	0.1955228	0	Singlet	Adipocyte
VAT_2M	1910.5	1254	3.6485289	9	Singlet	Mesothelial
VAT_2M	2343.3	1431	0.2974018	2	Singlet	FAPs
VAT_2M	8075.8	3153	0.0648825	0	Singlet	Adipocyte
VAT_2M	7908.4	2915	0.0417856	0	Singlet	Adipocyte
VAT_2M	7146.4	2627	0	0	Singlet	Adipocyte
VAT_2M	2477.3	1493	0.8259618	3	Singlet	FAPs
VAT_2M	4309.5	2153	4.9873739	7	Singlet	Endothelial_cells
VAT_2M	8581.1	3027	0.0211656	0	Singlet	Adipocyte
VAT_2M	2191.6	1501	0.283005	2	Singlet	FAPs
VAT_2M	2010.2	1447	0.6935301	2	Singlet	FAPs
VAT_2M	6952.9	2768	0.1279761	0	Singlet	Adipocyte
VAT_2M	5092.1	2369	0.3331418	0	Singlet	Adipocyte
VAT_2M	5790.9	2376	0.0034673	0	Singlet	Adipocyte
VAT_2M	5886	2580	0.0683522	0	Singlet	Adipocyte
VAT_2M	1209.1	831	2.8194436	2	Singlet	FAPs
VAT_2M	1750.6	1249	2.574597	2	Singlet	FAPs
VAT_2M	4478.8	2208	0.035012	0	Singlet	Adipocyte
VAT_2M	1890.5	1165	3.1326566	2	Singlet	FAPs
VAT_2M	1193.9	951	0.6596695	2	Singlet	FAPs
VAT_2M	1757.9	1202	5.8801059	3	Singlet	FAPs
VAT_2M	4968.7	2386	0.0923816	0	Singlet	Adipocyte
VAT_2M	1003.9	769	1.5854151	2	Singlet	FAPs
VAT_2M	6355.8	2837	0.0713428	0	Singlet	Adipocyte
VAT_2M	1609.7	984	1.8703748	2	Singlet	FAPs
VAT_2M	2464.9	1431	0.2331935	0	Singlet	Adipocyte

VAT_2M	3609	2200	0.6303408	2	Singlet	FAPs
VAT_2M	1257.4	891	0.2196135	3	Singlet	FAPs
VAT_2M	4878	2575	4.3790589	7	Singlet	Endothelial_cells
VAT_2M	1466.3	1062	0.2378141	3	Singlet	FAPs
VAT_2M	1856	1286	1.6622404	5	Singlet	Immune_cells
VAT_2M	7601.4	3052	0.0826651	0	Singlet	Adipocyte
VAT_2M	1316.5	1051	0.8769439	10	Singlet	Immune_cells
VAT_2M	3682.6	2029	0.7897551	3	Singlet	FAPs
VAT_2M	4094.4	1906	0	0	Singlet	Adipocyte
VAT_2M	1832.2	1173	0.7943709	2	Singlet	FAPs
VAT_2M	1596.9	1096	0.6065156	3	Singlet	FAPs
VAT_2M	1550.7	1025	0.0848626	7	Singlet	Endothelial_cells
VAT_2M	3682.5	1686	0.7781036	0	Singlet	Adipocyte
VAT_2M	4352.7	2188	0.259431	3	Singlet	FAPs
VAT_2M	1002.6	872	3.3771018	6	Singlet	Neurons
VAT_2M	4096.8	2132	0.0122346	0	Singlet	Adipocyte
VAT_2M	7860	2863	0.019747	0	Singlet	Adipocyte
VAT_2M	1357.9	952	0.4655743	3	Singlet	FAPs
VAT_2M	2164.1	1323	1.9205096	2	Singlet	FAPs
VAT_2M	6747.1	2765	3.4254741	0	Singlet	Adipocyte
VAT_2M	2148.3	1393	0.8559911	3	Singlet	FAPs
VAT_2M	1458.2	1048	2.4890913	10	Singlet	Immune_cells
VAT_2M	5480.7	2503	0	0	Singlet	Adipocyte
VAT_2M	2541.7	1440	0.084957	0	Singlet	Adipocyte
VAT_2M	2479.4	1518	1.000768	2	Singlet	FAPs
VAT_2M	1133.8	850	3.587956	3	Singlet	FAPs
VAT_2M	2838.9	1492	2.4129764	0	Singlet	Adipocyte
VAT_2M	2479.7	1615	0.5895863	3	Singlet	FAPs
VAT_2M	4728.9	2288	0.0297125	0	Singlet	Adipocyte
VAT_2M	2094.7	1131	0.8537148	2	Singlet	FAPs
VAT_2M	1651.7	1301	4.5578247	6	Singlet	Neurons
VAT_2M	1326.7	984	1.0536697	7	Singlet	Endothelial_cells
VAT_2M	2142	1428	0.3767053	2	Singlet	FAPs
VAT_2M	3101.9	1686	0.2280235	0	Singlet	Adipocyte
VAT_2M	2735.4	1557	0.4238217	0	Singlet	Adipocyte
VAT_2M	2447	1375	0.0324607	0	Singlet	Adipocyte
VAT_2M	3519.2	1884	0.319195	0	Singlet	Adipocyte
VAT_2M	1385.1	998	0.4667782	3	Singlet	FAPs
VAT_2M	7175	2831	2.4363475	0	Singlet	Adipocyte
VAT_2M	2942.2	1624	0.5261045	0	Singlet	Adipocyte
VAT_2M	4457.9	2163	0.0139504	0	Singlet	Adipocyte
VAT_2M	1044.4	748	3.2397974	4	Singlet	Immune_cells
VAT_2M	1484.6	1027	1.999557	3	Singlet	FAPs
VAT_2M	7671.3	2936	0.298663	0	Singlet	Adipocyte
VAT_2M	4101.9	1878	0.0133382	0	Singlet	Adipocyte
VAT_2M	2207	1303	0.1131657	0	Singlet	Adipocyte
VAT_2M	1156.9	1039	1.5224528	8	Singlet	Mesothelial
VAT_2M	3346.3	1709	0.3190428	0	Singlet	Adipocyte
VAT_2M	3008.3	1669	0.0466941	0	Singlet	Adipocyte
VAT_2M	1084.7	850	0	3	Singlet	FAPs
VAT_2M	3245.6	1686	0	0	Singlet	Adipocyte

VAT_2M	1674.7	1094	4.0697585	7	Singlet	Endothelial_cells
VAT_2M	3070.7	1742	0.3179845	3	Singlet	FAPs
VAT_2M	2567.1	1655	0.7228686	6	Singlet	Neurons
VAT_2M	1706.5	1137	0.690199	2	Singlet	FAPs
VAT_2M	1106.6	797	1.3092423	7	Singlet	Endothelial_cells
VAT_2M	2144.6	1316	0.4880181	0	Singlet	Adipocyte
VAT_2M	6310.7	2565	0	0	Singlet	Adipocyte
VAT_2M	7445.2	2694	0.0071026	0	Singlet	Adipocyte
VAT_2M	5997.9	2440	0.0306106	0	Singlet	Adipocyte
VAT_2M	8348.5	3161	0	0	Singlet	Adipocyte
VAT_2M	1065.7	771	4.5959315	9	Singlet	Mesothelial
VAT_2M	2006.6	973	10.051717	5	Singlet	Immune_cells
VAT_2M	1635.8	1254	2.4208141	6	Singlet	Neurons
VAT_2M	1282.6	816	1.6903249	6	Singlet	Neurons
VAT_2M	4873.3	2175	0.1096363	0	Singlet	Adipocyte
VAT_2M	5104.9	2372	1.5452268	0	Singlet	Adipocyte
VAT_2M	2358.2	1483	0.2163998	0	Singlet	Adipocyte
VAT_2M	5342.4	2354	0.0793842	0	Singlet	Adipocyte
VAT_2M	4632.1	2266	0.3205853	0	Singlet	Adipocyte
VAT_2M	1135.9	909	1.5531692	3	Singlet	FAPs
VAT_2M	3428.2	1928	1.4101136	3	Singlet	FAPs
VAT_2M	1972.1	1355	0.2618924	3	Singlet	FAPs
VAT_2M	1518	997	0.5369977	2	Singlet	FAPs
VAT_2M	1219	968	0.567936	11	Singlet	Smooth_muscle_cells
VAT_2M	3732.8	2047	2.7010115	3	Singlet	FAPs
VAT_2M	6490.9	2641	0.0076605	0	Singlet	Adipocyte
VAT_2M	3338.7	1786	0.3700072	0	Singlet	Adipocyte
VAT_2M	7558.7	2994	0.0027491	0	Singlet	Adipocyte
VAT_2M	4833.6	2311	0.0699326	0	Singlet	Adipocyte
VAT_2M	1984.4	1254	0.8067332	6	Singlet	Neurons
VAT_2M	1320.2	981	1.8968122	7	Singlet	Endothelial_cells
VAT_2M	2919.7	1539	0.1088161	3	Singlet	FAPs
VAT_2M	9272	3205	0.333749	0	Singlet	Adipocyte
VAT_2M	3440.8	1824	0	0	Singlet	Adipocyte
VAT_2M	3683.9	1871	0.0389106	11	Singlet	Smooth_muscle_cells
VAT_2M	2931.8	1578	0.0677904	0	Singlet	Adipocyte
VAT_2M	1625	1058	0.4146615	0	Singlet	Adipocyte
VAT_2M	2664.7	1659	0.0553714	0	Singlet	Adipocyte
VAT_2M	5381	2455	0.0587372	0	Singlet	Adipocyte
VAT_2M	1403.2	959	0.5900451	2	Singlet	FAPs
VAT_2M	4747.8	2213	0.1316694	0	Singlet	Adipocyte
VAT_2M	2360.5	1461	0.1806586	0	Singlet	Adipocyte
VAT_2M	7513.2	2852	0.0971825	0	Singlet	Adipocyte
VAT_2M	3186.9	1768	0.0206252	0	Singlet	Adipocyte
VAT_2M	8508.2	3011	0.0139343	0	Singlet	Adipocyte
VAT_2M	1871.3	1277	2.8120423	3	Singlet	FAPs
VAT_2M	7129.9	2890	0.0070745	0	Singlet	Adipocyte
VAT_2M	3821.7	1869	0.1944872	0	Singlet	Adipocyte
VAT_2M	3807.2	1868	0.030552	0	Singlet	Adipocyte
VAT_2M	1868.1	1151	0.353577	0	Singlet	Adipocyte
VAT_2M	3536	1992	0	0	Singlet	Adipocyte

VAT_2M	1970.2	1384	0.065269	6	Singlet	Neurons
VAT_2M	2096	1240	0.8996582	3	Singlet	FAPs
VAT_2M	1332.3	1030	1.5136904	8	Singlet	Mesothelial
VAT_2M	4570.7	2229	0.0853835	0	Singlet	Adipocyte
VAT_2M	7728	2853	0	0	Singlet	Adipocyte
VAT_2M	2300.2	1454	0.6261155	2	Singlet	FAPs
VAT_2M	3065.4	1833	0.8180977	3	Singlet	FAPs
VAT_2M	3499.9	1735	0.0171472	0	Singlet	Adipocyte
VAT_2M	4532.1	2229	1.0417505	0	Singlet	Adipocyte
VAT_2M	6309.4	2337	0.0255505	0	Singlet	Adipocyte
VAT_2M	2156.3	1350	0.0559876	3	Singlet	FAPs
VAT_2M	1026.5	809	1.6647788	8	Singlet	Mesothelial
VAT_2M	2624.8	1730	0.2129715	2	Singlet	FAPs
VAT_2M	6483.8	2664	0.0053984	0	Singlet	Adipocyte
VAT_2M	4133.3	2229	0.1240775	0	Singlet	Adipocyte
VAT_2M	2084.5	1260	0.1902473	0	Singlet	Adipocyte
VAT_2M	1219.5	892	1.2523485	7	Singlet	Endothelial_cells
VAT_2M	1493.8	1010	1.9304708	2	Singlet	FAPs
VAT_2M	3746	1824	3.1036514	0	Singlet	Adipocyte
VAT_2M	1068.2	801	2.1415382	7	Singlet	Endothelial_cells
VAT_2M	5021.2	2293	0.2100693	0	Singlet	Adipocyte
VAT_2M	10829	3628	0.0274805	0	Singlet	Adipocyte
VAT_2M	2533.2	1513	0.3952126	2	Singlet	FAPs
VAT_2M	7185	2893	0.0496379	0	Singlet	Adipocyte
VAT_2M	1969	1344	0.8248782	9	Singlet	Mesothelial
VAT_2M	6494	2508	0.0053065	0	Singlet	Adipocyte
VAT_2M	1191	860	1.2326158	5	Singlet	Immune_cells
VAT_2M	1412.2	982	0.2938461	7	Singlet	Endothelial_cells
VAT_2M	4045.8	1999	0.0126037	0	Singlet	Adipocyte
VAT_2M	2857.5	1561	0.0604112	0	Singlet	Adipocyte
VAT_2M	7251.3	2786	0.1993399	0	Singlet	Adipocyte
VAT_2M	3751.9	1935	0.0959216	3	Singlet	FAPs
VAT_2M	2439.4	1473	1.7169672	2	Singlet	FAPs
VAT_2M	2398.2	1655	2.7655016	2	Singlet	FAPs
VAT_2M	1680	1117	0.0879419	0	Singlet	Adipocyte
VAT_2M	1284.3	923	2.7387579	2	Singlet	FAPs
VAT_2M	2279.2	1276	1.1061687	6	Singlet	Neurons
VAT_2M	3031.6	1633	0.2906051	0	Singlet	Adipocyte
VAT_2M	1301.6	945	0.4262955	5	Singlet	Immune_cells
VAT_2M	1692.7	1263	2.3779993	2	Singlet	FAPs
VAT_2M	12505	3756	0.0112043	0	Singlet	Adipocyte
VAT_2M	3880.7	1930	0.728752	0	Singlet	Adipocyte
VAT_2M	2745.9	1561	0.6483226	0	Singlet	Adipocyte
VAT_2M	4497.3	2314	1.4446886	3	Singlet	FAPs
VAT_2M	3143.2	1897	0.2849045	2	Singlet	FAPs
VAT_2M	1583	916	9.3130436	3	Singlet	FAPs
VAT_2M	1238.3	932	0.1273407	7	Singlet	Endothelial_cells
VAT_2M	5849.6	2518	0.003475	0	Singlet	Adipocyte
VAT_2M	1048.3	871	1.5175195	2	Singlet	FAPs
VAT_2M	1236.4	974	0.427431	3	Singlet	FAPs
VAT_2M	2687.1	1472	0.3000246	0	Singlet	Adipocyte

VAT_2M	2284.3	1340	1.629174	0	Singlet	Adipocyte
VAT_2M	2054.5	1193	0.6135931	0	Singlet	Adipocyte
VAT_2M	1433.5	1135	1.5341113	2	Singlet	FAPs
VAT_2M	12266	3808	0.2017523	0	Singlet	Adipocyte
VAT_2M	1427.6	1042	2.1170564	2	Singlet	FAPs
VAT_2M	1955.6	1207	2.0945475	2	Singlet	FAPs
VAT_2M	1046.1	750	1.4318886	5	Singlet	Immune_cells
VAT_2M	2977.1	1716	0.2487299	0	Singlet	Adipocyte
VAT_2M	9186.7	3159	0.0340512	0	Singlet	Adipocyte
VAT_2M	8181.2	3039	1.3966949	0	Singlet	Adipocyte
VAT_2M	2554.9	1410	0.3980638	0	Singlet	Adipocyte
VAT_2M	2416.8	1587	0.4196351	7	Singlet	Endothelial_cells
VAT_2M	1187.5	883	1.6605769	6	Singlet	Neurons
VAT_2M	1641.7	1182	1.1923196	3	Singlet	FAPs
VAT_2M	5240.3	2444	0.0922579	0	Singlet	Adipocyte
VAT_2M	5142.4	2500	0.0428225	0	Singlet	Adipocyte
VAT_2M	1077	971	1.9422447	6	Singlet	Neurons
VAT_2M	1810.6	1216	0.7373583	2	Singlet	FAPs
VAT_2M	3405.2	1730	8.625741	5	Singlet	Immune_cells
VAT_2M	1644.3	1047	2.6453515	6	Singlet	Neurons
VAT_2M	3164.4	1658	0.2853478	0	Singlet	Adipocyte
VAT_2M	5554.2	2468	0	0	Singlet	Adipocyte
VAT_2M	10469	3507	1.0252832	0	Singlet	Adipocyte
VAT_2M	1466.3	1035	0.3040563	3	Singlet	FAPs
VAT_2M	1123.5	867	2.5665163	5	Singlet	Immune_cells
VAT_2M	1659.6	1216	2.5551507	5	Singlet	Immune_cells
VAT_2M	6994.5	2781	1.5194685	0	Singlet	Adipocyte
VAT_2M	6526	2603	0.2355784	0	Singlet	Adipocyte
VAT_2M	2714.5	1696	0.0638249	2	Singlet	FAPs
VAT_2M	3639.9	1790	0.3930563	0	Singlet	Adipocyte
VAT_2M	1520	1022	2.4032394	3	Singlet	FAPs
VAT_2M	1435.5	1162	2.1328211	6	Singlet	Neurons
VAT_2M	6618.3	2765	0.0177383	0	Singlet	Adipocyte
VAT_2M	1194.3	852	3.6518465	2	Singlet	FAPs
VAT_2M	2981	1847	0.2808558	2	Singlet	FAPs
VAT_2M	11856	3778	2.3148313	0	Singlet	Adipocyte
VAT_2M	1236.6	992	2.304761	5	Singlet	Immune_cells
VAT_2M	1071.9	842	5.2041929	6	Singlet	Neurons
VAT_2M	2490.2	1417	0.1741888	0	Singlet	Adipocyte
VAT_2M	1292.4	951	0.5033658	2	Singlet	FAPs
VAT_2M	1490.1	1111	1.8675848	3	Singlet	FAPs
VAT_2M	11821	3543	0	0	Singlet	Adipocyte
VAT_2M	3895.4	2034	0.0386635	2	Singlet	FAPs
VAT_2M	2346.9	1381	0.8506382	0	Singlet	Adipocyte
VAT_2M	7714.4	2947	0.0860888	0	Singlet	Adipocyte
VAT_2M	3862.8	2273	1.0634352	3	Singlet	FAPs
VAT_2M	1141.8	863	1.7425246	9	Singlet	Mesothelial
VAT_2M	1264.2	855	2.1245667	5	Singlet	Immune_cells
VAT_2M	1619.3	1256	0.7949798	4	Singlet	Immune_cells
VAT_2M	2974.5	1830	1.7181391	2	Singlet	FAPs
VAT_2M	6029.7	2576	0.5268402	0	Singlet	Adipocyte

VAT_2M	1965.3	1220	2.367172	5	Singlet	Immune_cells
VAT_2M	1315.4	1060	3.5136384	8	Singlet	Mesothelial
VAT_2M	2453.6	1499	2.537433	3	Singlet	FAPs
VAT_2M	5451.7	2461	0.1143281	0	Singlet	Adipocyte
VAT_2M	1403.9	1037	0.1762082	5	Singlet	Immune_cells
VAT_2M	1274.8	1045	1.3442123	8	Singlet	Mesothelial
VAT_2M	1067.9	840	1.2340591	2	Singlet	FAPs
VAT_2M	2509	1638	1.1251022	2	Singlet	FAPs
VAT_2M	1932.6	1086	0.508336	0	Singlet	Adipocyte
VAT_2M	1550.8	1132	0.6475474	11	Singlet	Smooth_muscle_cells
VAT_2M	1262.4	978	0.701496	6	Singlet	Neurons
VAT_2M	1722.9	1353	2.5920959	4	Singlet	Immune_cells
VAT_2M	9543.7	3369	0.004225	0	Singlet	Adipocyte
VAT_2M	1375.4	1007	0.6897344	6	Singlet	Neurons
VAT_2M	2776.6	1705	0	0	Singlet	Adipocyte
VAT_2M	5396.8	2330	0.114807	0	Singlet	Adipocyte
VAT_2M	2363.1	1404	0.2795806	0	Singlet	Adipocyte
VAT_2M	1445.8	1131	1.8907956	2	Singlet	FAPs
VAT_2M	2074.5	1428	1.0951156	2	Singlet	FAPs
VAT_2M	7032.6	2851	0	0	Singlet	Adipocyte
VAT_2M	6359.5	2526	0.0047888	0	Singlet	Adipocyte
VAT_2M	8033.5	3062	0.0010419	0	Singlet	Adipocyte
VAT_2M	1346.6	1024	1.3823138	6	Singlet	Neurons
VAT_2M	1006.8	770	1.9867609	2	Singlet	FAPs
VAT_2M	1215.8	867	3.1552501	6	Singlet	Neurons
VAT_2M	2004.4	1447	0.1160464	7	Singlet	Endothelial_cells
VAT_2M	1604.4	1290	0.3626277	7	Singlet	Endothelial_cells
VAT_2M	1028	866	1.0708379	7	Singlet	Endothelial_cells
VAT_2M	2317.4	1587	1.9258632	2	Singlet	FAPs
VAT_2M	1664.4	1217	1.3535368	5	Singlet	Immune_cells
VAT_2M	1886.9	1314	1.3860866	2	Singlet	FAPs
VAT_2M	1665.1	1230	0.7852903	11	Singlet	Smooth_muscle_cells
VAT_2M	4687.6	2056	0	0	Singlet	Adipocyte
VAT_2M	1880.4	1270	1.1258652	1	Singlet	Immune_cells
VAT_2M	7486	2958	0.1043322	0	Singlet	Adipocyte
VAT_2M	5227.8	2407	0.0488144	0	Singlet	Adipocyte
VAT_2M	1008.1	776	0.5257115	5	Singlet	Immune_cells
VAT_2M	2384.4	1567	0.3531634	2	Singlet	FAPs
VAT_2M	1153.7	778	0.864704	2	Singlet	FAPs
VAT_2M	2101.7	1309	0.3655658	0	Singlet	Adipocyte
VAT_2M	6802.4	2720	0.0030437	0	Singlet	Adipocyte
VAT_2M	6384.8	2776	0.0109542	0	Singlet	Adipocyte
VAT_2M	1223.4	1000	3.6428698	6	Singlet	Neurons
VAT_2M	7189.6	2706	0.0036294	0	Singlet	Adipocyte
VAT_2M	2869.5	1710	0.9942408	0	Singlet	Adipocyte
VAT_2M	7162.5	2831	0.0335469	0	Singlet	Adipocyte
VAT_2M	4775.7	2092	0.0200891	0	Singlet	Adipocyte
VAT_2M	6006.4	2657	0.4941452	0	Singlet	Adipocyte
VAT_2M	1932.9	1304	4.3286284	9	Singlet	Mesothelial
VAT_2M	8236.3	2948	0.0077437	0	Singlet	Adipocyte
VAT_2M	2916.6	1792	0.0722683	6	Singlet	Neurons

VAT_2M	3475.9	2023	0.7200754	2	Singlet	FAPs
VAT_2M	1081.5	878	1.4421724	9	Singlet	Mesothelial
VAT_2M	3100	1871	1.1484077	6	Singlet	Neurons
VAT_2M	1716.5	1014	12.33177	7	Singlet	Endothelial_cells
VAT_2M	13356	3681	0.059164	0	Singlet	Adipocyte
VAT_2M	1690	1155	1.6632006	6	Singlet	Neurons
VAT_2M	3268.2	1736	0.0496157	0	Singlet	Adipocyte
VAT_2M	1265.1	1065	0.5130925	6	Singlet	Neurons
VAT_2M	4140.9	2425	1.3314142	3	Singlet	FAPs
VAT_2M	7125.3	2464	1.6256323	0	Singlet	Adipocyte
VAT_2M	3902.2	1919	0.0392605	0	Singlet	Adipocyte
VAT_2M	2191	1317	2.5678876	5	Singlet	Immune_cells
VAT_2M	8357.6	3041	0	0	Singlet	Adipocyte
VAT_2M	1299.6	1005	2.0962976	4	Singlet	Immune_cells
VAT_2M	1633.5	1137	0.6392815	2	Singlet	FAPs
VAT_2M	1041.3	812	0.7044577	3	Singlet	FAPs
VAT_2M	10293	3553	0.0105761	0	Singlet	Adipocyte
VAT_2M	2047.9	1215	3.6138297	0	Singlet	Adipocyte
VAT_2M	1042.5	847	0.0759674	7	Singlet	Endothelial_cells
VAT_2M	4169	2063	0.2201851	2	Singlet	FAPs
VAT_2M	1192.7	881	1.5427493	6	Singlet	Neurons
VAT_2M	7435.5	2873	0	0	Singlet	Adipocyte
VAT_2M	2039.9	1433	1.3295513	2	Singlet	FAPs
VAT_2M	3045.2	1783	0.1054299	2	Singlet	FAPs
VAT_2M	2352.7	1533	0.1258988	5	Singlet	Immune_cells
VAT_2M	2009.9	1220	0.0391141	0	Singlet	Adipocyte
VAT_2M	1888.8	1279	5.9088873	3	Singlet	FAPs
VAT_2M	5899.9	2532	0	0	Singlet	Adipocyte
VAT_2M	1830.3	1316	1.6647676	5	Singlet	Immune_cells
VAT_2M	3788.6	1903	0.083611	0	Singlet	Adipocyte
VAT_2M	2522.7	1671	0	2	Singlet	FAPs
VAT_2M	1120	761	0.2150834	0	Singlet	Adipocyte
VAT_2M	3027.3	1634	3.8424636	0	Singlet	Adipocyte
VAT_2M	6885.4	2839	0.1394903	0	Singlet	Adipocyte
VAT_2M	1339.2	1062	3.0661262	4	Singlet	Immune_cells
VAT_2M	1008.6	888	2.1039245	9	Singlet	Mesothelial
VAT_2M	7808.6	3149	0.254151	0	Singlet	Adipocyte
VAT_2M	2272.4	1356	1.2864184	7	Singlet	Endothelial_cells
VAT_2M	1752.3	1256	0.492817	5	Singlet	Immune_cells
VAT_2M	1660.5	1149	0.7804812	3	Singlet	FAPs
VAT_2M	2004.6	1585	3.8070712	2	Singlet	FAPs
VAT_2M	2718.7	1681	1.8589757	3	Singlet	FAPs
VAT_2M	1970.6	1481	1.5445573	2	Singlet	FAPs
VAT_2M	1277.3	938	1.5906131	2	Singlet	FAPs
VAT_2M	5475.8	2440	0.1438622	0	Singlet	Adipocyte
VAT_2M	2776.8	1635	0.3518925	0	Singlet	Adipocyte
VAT_2M	2803.2	1379	12.769914	5	Singlet	Immune_cells
VAT_2M	2862.4	1735	0.5652703	3	Singlet	FAPs
VAT_2M	1234.3	1024	0.3601497	5	Singlet	Immune_cells
VAT_2M	2273.5	1415	5.174536	3	Singlet	FAPs
VAT_2M	3243.1	1715	0.0883054	0	Singlet	Adipocyte

VAT_2M	1417.1	1065	0.9368413	1	Singlet	Immune_cells
VAT_2M	6787.6	2860	0.4609734	0	Singlet	Adipocyte
VAT_2M	1534.1	1090	0.7018227	3	Singlet	FAPs
VAT_2M	8389	3118	0.0685126	0	Singlet	Adipocyte
VAT_2M	2576.5	1574	3.3037524	2	Singlet	FAPs
VAT_2M	1138.1	865	3.6232377	8	Singlet	Mesothelial
VAT_2M	4759.8	2407	4.9805742	7	Singlet	Endothelial_cells
VAT_2M	3695.8	1934	0.2093041	0	Singlet	Adipocyte
VAT_2M	3406.5	1791	0	0	Singlet	Adipocyte
VAT_2M	4514.2	2009	0.3026411	0	Singlet	Adipocyte
VAT_2M	5400.6	2371	0.0839059	0	Singlet	Adipocyte
VAT_2M	4024.9	2083	0.0276295	0	Singlet	Adipocyte
VAT_2M	1830	1358	2.1993314	2	Singlet	FAPs
VAT_2M	2005.5	1267	0.6425998	3	Singlet	FAPs
VAT_2M	1922.7	1255	0.4302582	0	Singlet	Adipocyte
VAT_2M	1950.5	1212	0.2235588	0	Singlet	Adipocyte
VAT_2M	1477.6	1010	2.2023803	3	Singlet	FAPs
VAT_2M	1056.4	855	1.706685	6	Singlet	Neurons
VAT_2M	4683.2	2353	0.570879	0	Singlet	Adipocyte
VAT_2M	3449.1	1758	0.0384457	0	Singlet	Adipocyte
VAT_2M	2923.7	1747	0.9650795	6	Singlet	Neurons
VAT_2M	1331.1	1158	0.2987163	6	Singlet	Neurons
VAT_2M	2863	1652	3.1666226	5	Singlet	Immune_cells
VAT_2M	3740.2	2009	1.2361723	2	Singlet	FAPs
VAT_2M	1464.6	1103	0.3264683	7	Singlet	Endothelial_cells
VAT_2M	1083.6	905	0.9641333	1	Singlet	Immune_cells
VAT_2M	1800	1263	0.9779091	2	Singlet	FAPs
VAT_2M	5436.8	2194	0.4997992	10	Singlet	Immune_cells
VAT_2M	2078.5	1543	0.8623872	2	Singlet	FAPs
VAT_2M	7901.7	3063	0.0408164	0	Singlet	Adipocyte
VAT_2M	3579.2	1772	0.1540485	0	Singlet	Adipocyte
VAT_2M	2484.4	1497	0.1649076	2	Singlet	FAPs
VAT_2M	1618.2	1134	3.9745969	2	Singlet	FAPs
VAT_2M	7483.1	2950	0	0	Singlet	Adipocyte
VAT_2M	9278.5	3144	0.012622	0	Singlet	Adipocyte
VAT_2M	2398	1446	0.1245807	0	Singlet	Adipocyte
VAT_2M	2686.3	1687	0.5832918	3	Singlet	FAPs
VAT_2M	2392.2	1610	0.561112	2	Singlet	FAPs
VAT_2M	1838.7	1420	1.2438217	7	Singlet	Endothelial_cells
VAT_2M	6199.7	2629	0.1870678	0	Singlet	Adipocyte
VAT_2M	1045.1	833	0.7743281	6	Singlet	Neurons
VAT_2M	1681	984	0.8838021	0	Singlet	Adipocyte
VAT_2M	4115.7	2148	0.5930277	2	Singlet	FAPs
VAT_2M	1349.8	1064	1.2060275	3	Singlet	FAPs
VAT_2M	1853.4	1240	2.7954066	6	Singlet	Neurons
VAT_2M	5982.7	2543	0.0255499	0	Singlet	Adipocyte
VAT_2M	10018	3384	0.0289592	0	Singlet	Adipocyte
VAT_2M	7437.9	2830	0.0077358	0	Singlet	Adipocyte
VAT_2M	12510	3585	0.193721	0	Singlet	Adipocyte
VAT_2M	4854	2130	0.2732624	0	Singlet	Adipocyte
VAT_2M	2937.6	1599	0.080812	0	Singlet	Adipocyte

VAT_2M	2440.5	1506	0.3786979	3	Singlet	FAPs
VAT_2M	3078.4	1862	1.3915805	2	Singlet	FAPs
VAT_2M	9100.4	3166	0.009617	0	Singlet	Adipocyte
VAT_2M	3908.4	1781	0.2018861	0	Singlet	Adipocyte
VAT_2M	5874.9	2486	0.1649732	0	Singlet	Adipocyte
VAT_2M	7476.5	2884	0.0953604	0	Singlet	Adipocyte
VAT_2M	6800.7	2744	0.0581828	0	Singlet	Adipocyte
VAT_2M	2918.2	1672	1.180833	2	Singlet	FAPs
VAT_2M	4275.1	2192	0.2035265	0	Singlet	Adipocyte
VAT_2M	5002.6	2211	0.1249426	0	Singlet	Adipocyte
VAT_2M	1501.8	1086	0.0983854	5	Singlet	Immune_cells
VAT_2M	4368.2	2053	0.2355593	0	Singlet	Adipocyte
VAT_2M	1822.3	1383	3.2112496	6	Singlet	Neurons
VAT_2M	1001.9	718	4.4555045	5	Singlet	Immune_cells
VAT_2M	2654.9	1541	5.8542332	7	Singlet	Endothelial_cells
VAT_2M	1304.8	992	0.5709174	7	Singlet	Endothelial_cells
VAT_2M	7402.5	2895	0.0110898	0	Singlet	Adipocyte
VAT_2M	1352.1	1033	3.5676637	9	Singlet	Mesothelial
VAT_2M	1470.6	1084	0.6088829	7	Singlet	Endothelial_cells
VAT_2M	3057.1	1754	2.9088903	5	Singlet	Immune_cells
VAT_2M	1050.2	773	1.5987604	7	Singlet	Endothelial_cells
VAT_2M	2117.4	1148	5.3436916	0	Singlet	Adipocyte
VAT_2M	5495.5	2303	0	0	Singlet	Adipocyte
VAT_2M	1292.1	965	3.411976	2	Singlet	FAPs
VAT_2M	1551.1	1183	1.4642264	5	Singlet	Immune_cells
VAT_2M	2668.7	1407	0.2021544	0	Singlet	Adipocyte
VAT_2M	1521.7	1073	1.2485045	2	Singlet	FAPs
VAT_2M	4593.4	2349	0.0668529	0	Singlet	Adipocyte
VAT_2M	6370.1	2744	0.0862802	0	Singlet	Adipocyte
VAT_2M	1497.8	1094	0.6795239	2	Singlet	FAPs
VAT_2M	3414.9	1763	0.0179246	0	Singlet	Adipocyte
VAT_2M	1600.5	1071	1.072281	0	Singlet	Adipocyte
VAT_2M	1744.9	1269	0.2293501	3	Singlet	FAPs
VAT_2M	7548.2	2969	0.4979045	5	Singlet	Immune_cells
VAT_2M	5250.1	2367	0.0378974	0	Singlet	Adipocyte
VAT_2M	7488.3	2823	0.0459462	0	Singlet	Adipocyte
VAT_2M	14512	4192	0.6984892	0	Singlet	Adipocyte
VAT_2M	5885.6	2513	0	0	Singlet	Adipocyte
VAT_2M	12547	3653	0	0	Singlet	Adipocyte
VAT_2M	7188.1	2761	0.1846854	0	Singlet	Adipocyte
VAT_2M	1779.7	1138	1.8748842	2	Singlet	FAPs
VAT_2M	1072.8	853	1.2156802	6	Singlet	Neurons
VAT_2M	1202.1	709	12.251026	7	Singlet	Endothelial_cells
VAT_2M	1346	982	1.9443422	2	Singlet	FAPs
VAT_2M	1273.8	1023	0.1220086	5	Singlet	Immune_cells
VAT_2M	9492.8	3203	0.1340779	0	Singlet	Adipocyte
VAT_2M	1081.3	856	1.5692657	6	Singlet	Neurons
VAT_2M	1165.2	952	1.6623581	8	Singlet	Mesothelial
VAT_2M	1306.3	956	2.3264438	2	Singlet	FAPs
VAT_2M	4673	2139	0.0903446	0	Singlet	Adipocyte
VAT_2M	3135.9	1892	0.5153231	2	Singlet	FAPs

VAT_2M	2538.3	1655	0.5924233	5	Singlet	Immune_cells
VAT_2M	1379	1114	0.8303396	6	Singlet	Neurons
VAT_2M	8652.2	3038	0.563613	0	Singlet	Adipocyte
VAT_2M	1239.5	926	2.2791341	2	Singlet	FAPs
VAT_2M	2575.4	1451	0.218472	0	Singlet	Adipocyte
VAT_2M	2726.9	1809	0.3088267	3	Singlet	FAPs
VAT_2M	1102.1	809	1.9915019	6	Singlet	Neurons
VAT_2M	4124.8	1949	0.0263999	0	Singlet	Adipocyte
VAT_2M	2795.9	1654	0.3045971	3	Singlet	FAPs
VAT_2M	6844.9	2604	0.0372088	0	Singlet	Adipocyte
VAT_2M	1600.4	1123	0.5128783	0	Singlet	Adipocyte
VAT_2M	1629.4	1094	0.0964149	6	Singlet	Neurons
VAT_2M	3541.1	1780	0.1541891	0	Singlet	Adipocyte
VAT_2M	5267.2	2328	0.0268599	0	Singlet	Adipocyte
VAT_2M	3748	1979	0.56629	0	Singlet	Adipocyte
VAT_2M	3044.2	1595	0.0159328	0	Singlet	Adipocyte
VAT_2M	3588.9	2091	0.7269234	7	Singlet	Endothelial_cells
VAT_2M	1104.5	800	1.249648	5	Singlet	Immune_cells
VAT_2M	2731.4	1830	0.7333084	2	Singlet	FAPs
VAT_2M	1023.6	791	1.1742462	3	Singlet	FAPs
VAT_2M	3185.3	1742	0.5751111	0	Singlet	Adipocyte
VAT_2M	7749.8	2877	4.2010616	0	Singlet	Adipocyte
VAT_2M	1068.8	820	8.2506408	11	Singlet	Smooth_muscle_cells
VAT_2M	1092.8	979	0.5510161	6	Singlet	Neurons
VAT_2M	1602.6	1083	2.5751032	2	Singlet	FAPs
VAT_2M	7079.3	2920	0	0	Singlet	Adipocyte
VAT_2M	1510.1	1071	3.4873236	5	Singlet	Immune_cells
VAT_2M	2117.3	1522	1.3776679	6	Singlet	Neurons
VAT_2M	1905.3	1205	0.4007638	0	Singlet	Adipocyte
VAT_2M	2266.5	1513	0.6369555	3	Singlet	FAPs
VAT_2M	1708.1	1009	1.9589368	6	Singlet	Neurons
VAT_2M	2170.7	1294	0.0353453	0	Singlet	Adipocyte
VAT_2M	1171.6	797	1.5910819	4	Singlet	Immune_cells
VAT_2M	3581.2	2161	0.1538254	3	Singlet	FAPs
VAT_2M	5358.7	2425	0.3260242	0	Singlet	Adipocyte
VAT_2M	5048.7	2291	0.0174823	0	Singlet	Adipocyte
VAT_2M	1057.5	797	7.17151	9	Singlet	Mesothelial
VAT_2M	1864.3	1302	0.1904013	6	Singlet	Neurons
VAT_2M	6047.4	2535	0	0	Singlet	Adipocyte
VAT_2M	1173.5	863	0.1739273	5	Singlet	Immune_cells
VAT_2M	3215.9	1821	0.0462494	0	Singlet	Adipocyte
VAT_2M	2059.2	1262	0.5419577	0	Singlet	Adipocyte
VAT_2M	1490.7	1111	0.2891004	2	Singlet	FAPs
VAT_2M	2092.4	1170	0.4573687	0	Singlet	Adipocyte
VAT_2M	1081.3	786	1.2777159	2	Singlet	FAPs
VAT_2M	1235.3	974	1.4142531	1	Singlet	Immune_cells
VAT_2M	1657.4	1112	3.903868	5	Singlet	Immune_cells
VAT_2M	4299.3	2255	0.0995867	0	Singlet	Adipocyte
VAT_2M	2896.6	1758	0.0226405	0	Singlet	Adipocyte
VAT_2M	1076.5	795	0.2634242	7	Singlet	Endothelial_cells
VAT_2M	2052	1197	1.2678681	0	Singlet	Adipocyte

VAT_2M	2659.9	1751	1.7234448	3	Singlet	FAPs
VAT_2M	1502.7	1127	1.0076943	6	Singlet	Neurons
VAT_2M	6616.3	2703	0.1416767	0	Singlet	Adipocyte
VAT_2M	3051.7	1697	0.6383446	0	Singlet	Adipocyte
VAT_2M	1781.4	1306	1.9046684	3	Singlet	FAPs
VAT_2M	1254.8	961	1.3293784	8	Singlet	Mesothelial
VAT_2M	2305.1	1464	1.5098182	3	Singlet	FAPs
VAT_2M	1005.5	814	0.3697295	5	Singlet	Immune_cells
VAT_2M	3275.7	1662	0.0433765	0	Singlet	Adipocyte
VAT_2M	2510.6	1498	0.2874966	0	Singlet	Adipocyte
VAT_2M	1601.5	1120	0.0806269	0	Singlet	Adipocyte
VAT_2M	1826.3	1261	0.3612572	3	Singlet	FAPs
VAT_2M	3062.4	1747	0.3958608	0	Singlet	Adipocyte
VAT_2M	1837.5	1180	0.9579031	6	Singlet	Neurons
VAT_2M	2379.4	1336	1.0998294	0	Singlet	Adipocyte
VAT_2M	2923.1	1649	0.0752645	0	Singlet	Adipocyte
VAT_2M	1399.7	1121	0.9412264	6	Singlet	Neurons
VAT_2M	9373.9	3287	0.0290023	0	Singlet	Adipocyte
VAT_2M	6163.2	2392	0.0593436	0	Singlet	Adipocyte
VAT_2M	6301.8	2647	0	0	Singlet	Adipocyte
VAT_2M	2456.6	1403	0.7574021	0	Singlet	Adipocyte
VAT_2M	1868.4	1108	6.5111527	2	Singlet	FAPs
VAT_2M	1269.1	932	0.5490892	6	Singlet	Neurons
VAT_2M	1300.3	1017	1.2728467	6	Singlet	Neurons
VAT_2M	3926.9	2031	0.2323851	0	Singlet	Adipocyte
VAT_2M	3553.9	1689	0.091355	0	Singlet	Adipocyte
VAT_2M	8224.1	3107	0.0017526	0	Singlet	Adipocyte
VAT_2M	7847.4	3013	0.0540578	0	Singlet	Adipocyte
VAT_2M	4976.6	2406	0.0311325	0	Singlet	Adipocyte
VAT_2M	1275.4	926	1.8192394	5	Singlet	Immune_cells
VAT_2M	1045.9	851	2.4737946	6	Singlet	Neurons
VAT_2M	2416.7	1314	0.2029864	0	Singlet	Adipocyte
VAT_2M	2287.1	1405	2.0638688	2	Singlet	FAPs
VAT_2M	2883.2	1707	0.04646	0	Singlet	Adipocyte
VAT_2M	5198.9	2292	0.1559116	0	Singlet	Adipocyte
VAT_2M	1095.5	886	3.6112111	6	Singlet	Neurons
VAT_2M	1306.2	946	5.1498944	6	Singlet	Neurons
VAT_2M	1285.6	1149	0.3602971	6	Singlet	Neurons
VAT_2M	5143.5	2568	0.5728292	3	Singlet	FAPs
VAT_2M	4613.7	2222	0.008061	0	Singlet	Adipocyte
VAT_2M	7192.2	2625	0.0028931	0	Singlet	Adipocyte
VAT_2M	4236.8	2438	1.6246888	2	Singlet	FAPs
VAT_2M	5396.7	2321	0.0118781	0	Singlet	Adipocyte
VAT_2M	1813.2	1190	0.1474766	3	Singlet	FAPs
VAT_2M	6963.8	2865	0.1038082	0	Singlet	Adipocyte
VAT_2M	1111.9	793	0.6073606	2	Singlet	FAPs
VAT_2M	2259.5	1445	3.0285837	0	Singlet	Adipocyte
VAT_2M	1403.6	994	0.2665412	2	Singlet	FAPs
VAT_2M	2249	1318	1.809765	0	Singlet	Adipocyte
VAT_2M	2374.9	1545	0.077557	6	Singlet	Neurons
VAT_2M	7467	2901	0.0088353	0	Singlet	Adipocyte

VAT_2M	1371.3	1061	1.0396956	11	Singlet	Smooth_muscle_cells
VAT_2M	3713.3	1837	0.051178	0	Singlet	Adipocyte
VAT_2M	3620.8	1722	0.1102548	0	Singlet	Adipocyte
VAT_2M	2025	1317	0	2	Singlet	FAPs
VAT_2M	5998.1	2573	0.0749142	0	Singlet	Adipocyte
VAT_2M	1629.3	1370	2.4957505	6	Singlet	Neurons
VAT_2M	1543.8	1125	1.1666369	2	Singlet	FAPs
VAT_2M	4334.4	2042	0.064102	0	Singlet	Adipocyte
VAT_2M	2417.9	1397	0.1734507	0	Singlet	Adipocyte
VAT_2M	1512.6	1090	2.4296744	10	Singlet	Immune_cells
VAT_2M	1038.8	846	1.6285421	7	Singlet	Endothelial_cells
VAT_2M	1893.9	1262	0.2490714	3	Singlet	FAPs
VAT_2M	4522.3	2224	0.923611	0	Singlet	Adipocyte
VAT_2M	3415.4	1891	1.6727874	0	Singlet	Adipocyte
VAT_2M	8984.4	3036	0.0306851	0	Singlet	Adipocyte
VAT_2M	2622.1	1422	0.2659779	0	Singlet	Adipocyte
VAT_2M	1687.1	1081	0.121545	3	Singlet	FAPs
VAT_2M	3923.9	2035	0.2011386	0	Singlet	Adipocyte
VAT_2M	2073.3	1418	1.1392405	5	Singlet	Immune_cells
VAT_2M	3951.9	1984	0.0144491	0	Singlet	Adipocyte
VAT_2M	2497.6	1584	1.743596	3	Singlet	FAPs
VAT_2M	4256.5	1981	0.1813856	0	Singlet	Adipocyte
VAT_2M	7157.1	2915	0.0217743	0	Singlet	Adipocyte
VAT_2M	1708.4	1320	0.1114288	5	Singlet	Immune_cells
VAT_2M	1102.3	888	0.6286354	6	Singlet	Neurons
VAT_2M	1210.6	866	1.0368413	9	Singlet	Mesothelial
VAT_2M	5405.3	2438	0.0100997	0	Singlet	Adipocyte
VAT_2M	1734.7	1353	5.4369695	6	Singlet	Neurons
VAT_2M	2140.4	1445	0.6733673	2	Singlet	FAPs
VAT_2M	3476.4	1823	0.1736361	0	Singlet	Adipocyte
VAT_2M	2606.6	1574	0.0286266	0	Singlet	Adipocyte
VAT_2M	6169.8	2430	0.0122804	0	Singlet	Adipocyte
VAT_2M	1872.9	1197	0.0502403	3	Singlet	FAPs
VAT_2M	2906.6	1796	0.1156514	0	Singlet	Adipocyte
VAT_2M	5224.5	2481	0.0092074	0	Singlet	Adipocyte
VAT_2M	1236.6	976	0.2335239	2	Singlet	FAPs
VAT_2M	1417.7	962	0.4026011	0	Singlet	Adipocyte
VAT_2M	5834.3	2636	0.0137677	0	Singlet	Adipocyte
VAT_2M	1241.8	898	0.4670502	2	Singlet	FAPs
VAT_2M	6242.7	2646	1.1317837	0	Singlet	Adipocyte
VAT_2M	5949.4	2436	0.0478962	0	Singlet	Adipocyte
VAT_2M	1021.8	815	1.4655731	6	Singlet	Neurons
VAT_2M	1262.4	922	0.0231559	3	Singlet	FAPs
VAT_2M	4944.3	2319	0	0	Singlet	Adipocyte
VAT_2M	3847.8	2057	1.5024838	0	Singlet	Adipocyte
VAT_2M	3372.3	1944	0.0456498	0	Singlet	Adipocyte
VAT_2M	1327.6	954	0.8702229	2	Singlet	FAPs
VAT_2M	8415	3011	0.0030739	0	Singlet	Adipocyte
VAT_2M	3516.1	1710	0.1457488	0	Singlet	Adipocyte
VAT_2M	5579.2	2632	0.028362	0	Singlet	Adipocyte
VAT_2M	5047	2355	0.5874939	0	Singlet	Adipocyte

VAT_2M	2056	1369	0.5251179	2	Singlet	FAPs
VAT_2M	6797.8	2735	0.0047192	0	Singlet	Adipocyte
VAT_2M	2548.8	1609	0.8753076	2	Singlet	FAPs
VAT_2M	1938.1	1248	1.7856585	0	Singlet	Adipocyte
VAT_2M	2048.3	1279	0.329286	0	Singlet	Adipocyte
VAT_2M	2198.1	1467	3.0422017	6	Singlet	Neurons
VAT_2M	8386.6	3033	0.0151575	0	Singlet	Adipocyte
VAT_2M	12181	3847	0.8608508	0	Singlet	Adipocyte
VAT_2M	1329.4	1026	0.4321189	11	Singlet	Smooth_muscle_cells
VAT_2M	1496.1	1011	0.2331967	2	Singlet	FAPs
VAT_2M	2316.7	1417	0.1980002	0	Singlet	Adipocyte
VAT_2M	1394	1145	1.7171209	6	Singlet	Neurons
VAT_2M	1744.7	1221	0.7444226	3	Singlet	FAPs
VAT_2M	4756.2	2223	0.0283049	0	Singlet	Adipocyte
VAT_2M	1784.1	1234	1.9215744	3	Singlet	FAPs
VAT_2M	1051.5	887	0.5491538	6	Singlet	Neurons
VAT_2M	2093.6	1292	0.3360169	0	Singlet	Adipocyte
VAT_2M	6192.6	2528	0.0758361	0	Singlet	Adipocyte
VAT_2M	3579.5	1911	0.1261065	0	Singlet	Adipocyte
VAT_2M	1388.1	988	1.1641218	9	Singlet	Mesothelial
VAT_2M	6411.1	2525	0.0217303	0	Singlet	Adipocyte
VAT_2M	6200.3	2686	0.0502302	0	Singlet	Adipocyte
VAT_2M	7998.2	3112	0.1738222	0	Singlet	Adipocyte
VAT_2M	2137	1265	0.8407871	7	Singlet	Endothelial_cells
VAT_2M	1597.4	1098	4.8359758	3	Singlet	FAPs
VAT_2M	8651.6	3005	0.0671165	0	Singlet	Adipocyte
VAT_2M	8226.1	3187	0.0806428	0	Singlet	Adipocyte
VAT_2M	1070.1	806	0.9361997	1	Singlet	Immune_cells
VAT_2M	2839.4	1590	0.1428155	3	Singlet	FAPs
VAT_2M	1973.6	1362	0.2370743	6	Singlet	Neurons
VAT_2M	2634.6	1687	1.0261402	6	Singlet	Neurons
VAT_2M	2597.9	1499	0.6047436	6	Singlet	Neurons
VAT_2M	9954	3472	0.0617882	0	Singlet	Adipocyte
VAT_2M	2287.6	1580	2.1705077	2	Singlet	FAPs
VAT_2M	2231	1337	1.0261068	7	Singlet	Endothelial_cells
VAT_2M	12978	3831	0.4964885	0	Singlet	Adipocyte
VAT_2M	7601.8	2843	0.0547925	0	Singlet	Adipocyte
VAT_2M	6492.3	2536	0.0647644	0	Singlet	Adipocyte
VAT_2M	4447.7	2189	0.1718019	0	Singlet	Adipocyte
VAT_2M	7290.5	2881	0.0596844	0	Singlet	Adipocyte
VAT_2M	1064.4	773	1.7897566	2	Singlet	FAPs
VAT_2M	1434.1	1058	0.5143581	3	Singlet	FAPs
VAT_2M	2649.9	1739	1.6698747	5	Singlet	Immune_cells
VAT_2M	2748.1	1545	4.1430075	9	Singlet	Mesothelial
VAT_2M	1839.3	1445	1.3608431	11	Singlet	Smooth_muscle_cells
VAT_2M	6215	2670	0.0095515	0	Singlet	Adipocyte
VAT_2M	5034.9	2192	0.074269	0	Singlet	Adipocyte
VAT_2M	7714.9	2913	0.0862573	0	Singlet	Adipocyte
VAT_2M	4732.1	2143	0.133047	0	Singlet	Adipocyte
VAT_2M	1564.1	1216	1.1836172	6	Singlet	Neurons
VAT_2M	2078.2	1300	0.4162292	0	Singlet	Adipocyte

VAT_2M	5575.4	2583	0.0517219	0	Singlet	Adipocyte
VAT_2M	4549.1	2340	1.2549925	3	Singlet	FAPs
VAT_2M	1638.8	1153	0.6598217	3	Singlet	FAPs
VAT_2M	2367.7	1455	0.4345805	0	Singlet	Adipocyte
VAT_2M	13469	3993	0.2824922	0	Singlet	Adipocyte
VAT_2M	1130.3	885	1.4933406	6	Singlet	Neurons
VAT_2M	6708.1	2755	0.0223483	0	Singlet	Adipocyte
VAT_2M	1831.3	1365	0.7986175	5	Singlet	Immune_cells
VAT_2M	1650.5	1363	0.3292525	6	Singlet	Neurons
VAT_2M	2291.2	1311	0.1210968	0	Singlet	Adipocyte
VAT_2M	2249	1519	1.6882696	2	Singlet	FAPs
VAT_2M	1711.6	1186	0.9771699	2	Singlet	FAPs
VAT_2M	1500.7	1057	3.8565321	9	Singlet	Mesothelial
VAT_2M	5395.9	2447	0.0310133	0	Singlet	Adipocyte
VAT_2M	11775	3699	0.0366779	0	Singlet	Adipocyte
VAT_2M	1956.8	1315	0.971467	5	Singlet	Immune_cells
VAT_2M	1931.1	1303	1.7622991	2	Singlet	FAPs
VAT_2M	3495.9	1923	0.2554636	0	Singlet	Adipocyte
VAT_2M	1150.3	842	9.9365803	6	Singlet	Neurons
VAT_2M	1564.8	1032	0	3	Singlet	FAPs
VAT_2M	2750.5	1700	0	6	Singlet	Neurons
VAT_2M	1169.4	927	1.0279723	2	Singlet	FAPs
VAT_2M	6062.8	2563	0.0438355	0	Singlet	Adipocyte
VAT_2M	2668.2	1426	1.5818099	5	Singlet	Immune_cells
VAT_2M	1834.5	1170	6.4799588	5	Singlet	Immune_cells
VAT_2M	5964.7	2592	0.0136464	0	Singlet	Adipocyte
VAT_2M	2829.9	1541	0.5037937	0	Singlet	Adipocyte
VAT_2M	4205.6	2142	0.5474955	0	Singlet	Adipocyte
VAT_2M	3488	1634	0.1355099	0	Singlet	Adipocyte
VAT_2M	1290.5	1002	1.7257882	2	Singlet	FAPs
VAT_2M	1249	979	0.5367255	6	Singlet	Neurons
VAT_2M	2252	1548	0.6001496	2	Singlet	FAPs
VAT_2M	4657.8	2090	0.358058	0	Singlet	Adipocyte
VAT_2M	2014.9	1383	0.2875877	0	Singlet	Adipocyte
VAT_2M	4455.7	1990	0.1131255	0	Singlet	Adipocyte
VAT_2M	6521.9	2511	0	0	Singlet	Adipocyte
VAT_2M	1870.7	1091	0.5885897	0	Singlet	Adipocyte
VAT_2M	1634.4	1095	0.3125967	0	Singlet	Adipocyte
VAT_2M	1879.7	1226	2.0182996	2	Singlet	FAPs
VAT_2M	1226	930	0.3746552	3	Singlet	FAPs
VAT_2M	2730.1	1604	1.2815566	5	Singlet	Immune_cells
VAT_2M	3740.9	2242	1.0915582	5	Singlet	Immune_cells
VAT_2M	2386.5	1560	0.6371753	5	Singlet	Immune_cells
VAT_2M	3580.1	1911	0.3107702	5	Singlet	Immune_cells
VAT_2M	1599.4	1181	0.0182624	10	Singlet	Immune_cells
VAT_2M	1100.5	820	0.4091261	2	Singlet	FAPs
VAT_2M	1386.5	1040	1.0106161	6	Singlet	Neurons
VAT_2M	6885.1	2694	0.0036083	0	Singlet	Adipocyte
VAT_2M	1053.9	847	0.4027627	11	Singlet	Smooth_muscle_cells
VAT_2M	1759.2	1085	0.97923	0	Singlet	Adipocyte
VAT_2M	6257	2648	0.2464453	0	Singlet	Adipocyte

VAT_2M	1453	1094	2.4091029	1	Singlet	Immune_cells
VAT_2M	5576	2516	0.0978213	0	Singlet	Adipocyte
VAT_2M	1419.4	1072	0.7487773	8	Singlet	Mesothelial
VAT_2M	3016.4	1792	0.3365912	0	Singlet	Adipocyte
VAT_2M	1379.3	1069	1.4793294	2	Singlet	FAPs
VAT_2M	2353.8	1450	1.3485697	0	Singlet	Adipocyte
VAT_2M	5268.3	2314	2.5549855	0	Singlet	Adipocyte
VAT_2M	6069.5	2592	0.0219448	0	Singlet	Adipocyte
VAT_2M	1219.7	919	0.0509961	2	Singlet	FAPs
VAT_2M	5671.7	2569	0	0	Singlet	Adipocyte
VAT_2M	4112.9	1927	0	0	Singlet	Adipocyte
VAT_2M	1860.4	1085	1.131151	0	Singlet	Adipocyte
VAT_2M	1809.5	1187	0.632239	3	Singlet	FAPs
VAT_2M	1171.5	879	3.8928542	4	Singlet	Immune_cells
VAT_2M	2284	1576	0.8545607	6	Singlet	Neurons
VAT_2M	2294.2	1413	0.8209289	0	Singlet	Adipocyte
VAT_2M	1608.3	1072	0.2537079	5	Singlet	Immune_cells
VAT_2M	2401.6	1445	0.0047345	0	Singlet	Adipocyte
VAT_2M	1043.4	843	3.0292088	3	Singlet	FAPs
VAT_2M	10093	3190	0.0425092	0	Singlet	Adipocyte
VAT_2M	3191.9	1659	0.1235304	0	Singlet	Adipocyte
VAT_2M	6176.3	2429	0.0094478	0	Singlet	Adipocyte
VAT_2M	1926.9	1401	0.8937616	0	Singlet	Adipocyte
VAT_2M	1407.2	1001	1.55327	3	Singlet	FAPs
VAT_2M	6386.4	2612	0.0463968	0	Singlet	Adipocyte
VAT_2M	3386.4	1946	0.122987	0	Singlet	Adipocyte
VAT_2M	1069.3	854	0.650789	3	Singlet	FAPs
VAT_2M	1566.5	1004	2.0434468	0	Singlet	Adipocyte
VAT_2M	3501.5	1835	0.0345824	0	Singlet	Adipocyte
VAT_2M	3054.5	1693	1.6839113	0	Singlet	Adipocyte
VAT_2M	1204.7	967	2.1978503	3	Singlet	FAPs
VAT_2M	1972.2	1214	0.4618662	0	Singlet	Adipocyte
VAT_2M	2627.7	1440	0.1324674	0	Singlet	Adipocyte
VAT_2M	1550.2	1139	1.0778913	3	Singlet	FAPs
VAT_2M	1236.3	851	4.2643863	5	Singlet	Immune_cells
VAT_2M	1379.3	1093	1.5520984	2	Singlet	FAPs
VAT_2M	7801.4	2915	0.0028055	0	Singlet	Adipocyte
VAT_2M	1979.8	1147	0.3999662	0	Singlet	Adipocyte
VAT_2M	6452.5	2679	0.1458244	0	Singlet	Adipocyte
VAT_2M	2952	1667	0.5984007	0	Singlet	Adipocyte
VAT_2M	1292.6	936	2.1946247	3	Singlet	FAPs
VAT_2M	3570.6	1972	0.1644004	0	Singlet	Adipocyte
VAT_2M	5331	2388	0.2398933	0	Singlet	Adipocyte
VAT_2M	1984.4	1212	0.2999028	0	Singlet	Adipocyte
VAT_2M	2561.6	1437	0.0762056	0	Singlet	Adipocyte
VAT_2M	1380.4	1117	0.0916542	7	Singlet	Endothelial_cells
VAT_2M	2997.8	1756	0.5859098	0	Singlet	Adipocyte
VAT_2M	1787.3	1182	0.7595678	6	Singlet	Neurons
VAT_2M	1019.1	799	1.9669906	7	Singlet	Endothelial_cells
VAT_2M	1361.1	973	0.6425219	6	Singlet	Neurons
VAT_2M	6528.2	3331	0.4649003	2	Singlet	FAPs

VAT_2M	1885.9	1644	0.4217969	11	Singlet	Smooth_muscle_cells
VAT_2M	1070.4	821	1.9336847	9	Singlet	Mesothelial
VAT_2M	1326.5	999	0.4143724	2	Singlet	FAPs
VAT_2M	1393	979	0.3282096	2	Singlet	FAPs
VAT_2M	2637.4	1735	0.697467	2	Singlet	FAPs
VAT_2M	1213.3	1008	5.3637587	6	Singlet	Neurons
VAT_2M	1757.7	1166	6.7752111	5	Singlet	Immune_cells
VAT_2M	2686.4	1480	0.0900231	0	Singlet	Adipocyte
VAT_2M	5998	2668	0.0745392	0	Singlet	Adipocyte
VAT_2M	3664.4	2064	0.7580771	3	Singlet	FAPs
VAT_2M	1410	1073	1.2167471	1	Singlet	Immune_cells
VAT_2M	2541.5	1535	0.6856257	0	Singlet	Adipocyte
VAT_2M	2455.3	1441	0.1165238	0	Singlet	Adipocyte
VAT_2M	9108.6	3222	0	0	Singlet	Adipocyte
VAT_2M	1574.9	879	4.5444415	9	Singlet	Mesothelial
VAT_2M	4972.6	2348	0.339321	0	Singlet	Adipocyte
VAT_2M	1606	1234	1.3549102	6	Singlet	Neurons
VAT_2M	1193.5	845	4.8136057	2	Singlet	FAPs
VAT_2M	1199.9	973	0.3259438	3	Singlet	FAPs
VAT_2M	1259.2	1028	3.1829582	9	Singlet	Mesothelial
VAT_2M	8415.5	3080	0.0053798	0	Singlet	Adipocyte
VAT_2M	3479.6	1801	0.2052356	0	Singlet	Adipocyte
VAT_2M	5051.1	2304	0.0469097	0	Singlet	Adipocyte
VAT_2M	2064.7	1177	0.2598182	0	Singlet	Adipocyte
VAT_2M	5591	2494	0.025781	0	Singlet	Adipocyte
VAT_2M	1282	986	2.1410969	3	Singlet	FAPs
VAT_2M	1615	1212	0.9128457	2	Singlet	FAPs
VAT_2M	6562	2749	0.2007737	0	Singlet	Adipocyte
VAT_2M	2823.2	1640	0	0	Singlet	Adipocyte
VAT_2M	7681.8	2965	0.1059793	0	Singlet	Adipocyte
VAT_2M	3914.9	1962	0.1690539	0	Singlet	Adipocyte
VAT_2M	1035.3	775	1.7478723	2	Singlet	FAPs
VAT_2M	2099.4	1233	3.633855	3	Singlet	FAPs
VAT_2M	2855.8	1486	0.2935361	3	Singlet	FAPs
VAT_2M	6492.9	2630	0.0417346	0	Singlet	Adipocyte
VAT_2M	8153.8	2859	1.5076189	0	Singlet	Adipocyte
VAT_2M	1495.2	1184	1.6026664	6	Singlet	Neurons
VAT_2M	4919.1	2299	0.0677777	0	Singlet	Adipocyte
VAT_2M	8629	3085	0.0720107	0	Singlet	Adipocyte
VAT_2M	2012.2	1334	2.0101689	3	Singlet	FAPs
VAT_2M	3198.5	1704	0.120212	0	Singlet	Adipocyte
VAT_2M	1946.3	1253	1.0743783	0	Singlet	Adipocyte
VAT_2M	1165.3	932	3.6030895	1	Singlet	Immune_cells
VAT_2M	8168.1	3124	0.8472408	0	Singlet	Adipocyte
VAT_2M	1203.2	911	2.3646622	2	Singlet	FAPs
VAT_2M	1131.9	911	3.5137056	9	Singlet	Mesothelial
VAT_2M	3465.8	1747	0.1624001	0	Singlet	Adipocyte
VAT_2M	1390.5	1108	1.231731	3	Singlet	FAPs
VAT_2M	1725.9	1148	4.25036	0	Singlet	Adipocyte
VAT_2M	2936.6	1697	1.4286087	0	Singlet	Adipocyte
VAT_2M	1565.9	1111	0.6899773	2	Singlet	FAPs

VAT_2M	1351.6	1031	0.5637149	11	Singlet	Smooth_muscle_cells
VAT_2M	2090.5	1480	2.9707511	2	Singlet	FAPs
VAT_2M	2565.3	1476	0.6440221	0	Singlet	Adipocyte
VAT_2M	3731.9	1878	0.0170107	0	Singlet	Adipocyte
VAT_2M	1943.7	1148	0.43664	0	Singlet	Adipocyte
VAT_2M	4395.3	2040	0.1460762	0	Singlet	Adipocyte
VAT_2M	1324.7	902	2.3018984	8	Singlet	Mesothelial
VAT_2M	4960.2	2286	0.0584482	0	Singlet	Adipocyte
VAT_2M	1633.8	1056	0.4190797	3	Singlet	FAPs
VAT_2M	1099.9	898	0.9943787	6	Singlet	Neurons
VAT_2M	4063.9	2045	0.1716155	0	Singlet	Adipocyte
VAT_2M	12199	3743	3.4149515	0	Singlet	Adipocyte
VAT_2M	1948.8	1356	0.0672364	6	Singlet	Neurons
VAT_2M	1165.7	840	2.1680588	7	Singlet	Endothelial_cells
VAT_2M	1238.9	946	2.6112065	2	Singlet	FAPs
VAT_2M	6232.9	2691	0.0258355	0	Singlet	Adipocyte
VAT_2M	1188.7	830	2.4844576	3	Singlet	FAPs
VAT_2M	2479.5	1394	0.2565393	0	Singlet	Adipocyte
VAT_2M	1040.5	807	1.2276834	9	Singlet	Mesothelial
VAT_2M	1113.9	913	2.1240659	5	Singlet	Immune_cells
VAT_2M	3205.2	1603	0.0713149	0	Singlet	Adipocyte
VAT_2M	2971.1	1677	0.0887463	0	Singlet	Adipocyte
VAT_2M	1777.4	1179	0.2701956	0	Singlet	Adipocyte
VAT_2M	1154.9	874	1.2006115	2	Singlet	FAPs
VAT_2M	4102.2	2047	0.1820386	0	Singlet	Adipocyte
VAT_2M	2334.5	1445	2.0721163	6	Singlet	Neurons
VAT_2M	1068.6	833	2.784834	4	Singlet	Immune_cells
VAT_2M	3894.1	1930	0.0692266	0	Singlet	Adipocyte
VAT_2M	1288.9	1021	0.2874481	6	Singlet	Neurons
VAT_2M	8805.1	3227	0.0478824	0	Singlet	Adipocyte
VAT_2M	2684.6	1546	0.0625939	0	Singlet	Adipocyte
VAT_2M	4040.9	1924	0	0	Singlet	Adipocyte
VAT_2M	4956.8	2115	0.1655552	0	Singlet	Adipocyte
VAT_2M	3972.9	2029	1.2497442	2	Singlet	FAPs
VAT_2M	1608.1	1123	1.0513251	2	Singlet	FAPs
VAT_2M	1254.3	1008	3.7030549	5	Singlet	Immune_cells
VAT_2M	1732.1	1179	1.2036122	0	Singlet	Adipocyte
VAT_2M	3093.6	1585	0.0955071	0	Singlet	Adipocyte
VAT_2M	5109.3	2515	0.2615637	0	Singlet	Adipocyte
VAT_2M	1833.6	1117	1.8971167	0	Singlet	Adipocyte
VAT_2M	1262.9	932	0.8127464	7	Singlet	Endothelial_cells
VAT_2M	1677.2	1149	1.2907272	6	Singlet	Neurons
VAT_2M	2034.6	1178	1.0051931	0	Singlet	Adipocyte
VAT_2M	2152.7	1452	1.7043531	6	Singlet	Neurons
VAT_2M	1019.6	790	0.3300902	2	Singlet	FAPs
VAT_2M	1698.4	1321	1.8055827	6	Singlet	Neurons
VAT_2M	2427.9	1405	0.1818229	0	Singlet	Adipocyte
VAT_2M	3856.3	2081	0.1905049	0	Singlet	Adipocyte
VAT_2M	1150.4	864	2.4176702	6	Singlet	Neurons
VAT_2M	2070.3	1363	0.6583022	2	Singlet	FAPs
VAT_2M	12046	3519	0.0482047	0	Singlet	Adipocyte

VAT_2M	1520.8	1056	0.7936458	3	Singlet	FAPs
VAT_2M	2099.5	1316	4.6589524	7	Singlet	Endothelial_cells
VAT_2M	1023.7	878	0.8879018	4	Singlet	Immune_cells
VAT_2M	4761.1	2233	0.3781462	0	Singlet	Adipocyte
VAT_2M	1333.4	986	1.5296709	2	Singlet	FAPs
VAT_2M	12942	3757	0.0179642	0	Singlet	Adipocyte
VAT_2M	4856.3	2166	1.3872536	0	Singlet	Adipocyte
VAT_2M	4870.5	2031	0.202706	0	Singlet	Adipocyte
VAT_2M	3730.9	2187	0.8217624	2	Singlet	FAPs
VAT_2M	3649.4	1837	1.586159	0	Singlet	Adipocyte
VAT_2M	7247.4	2996	0	0	Singlet	Adipocyte
VAT_2M	6517.4	2687	0.0544365	0	Singlet	Adipocyte
VAT_2M	8344.3	2956	0	0	Singlet	Adipocyte
VAT_2M	2591.6	1740	1.1094398	5	Singlet	Immune_cells
VAT_2M	2002.9	1262	0.8478032	0	Singlet	Adipocyte
VAT_2M	2861.6	1683	2.3228138	9	Singlet	Mesothelial
VAT_2M	2221.6	1569	1.8153965	2	Singlet	FAPs
VAT_2M	7326.9	3004	0.0033462	0	Singlet	Adipocyte
VAT_2M	3315.2	1995	1.7546467	2	Singlet	FAPs
VAT_2M	1456.7	1115	1.2694054	1	Singlet	Immune_cells
VAT_2M	8380.5	3154	0.0132865	0	Singlet	Adipocyte
VAT_2M	4920.9	2317	1.7432196	0	Singlet	Adipocyte
VAT_2M	1932.6	1363	1.6437293	2	Singlet	FAPs
VAT_2M	1020.7	875	0.210599	4	Singlet	Immune_cells
VAT_2M	1147.3	753	8.5743916	4	Singlet	Immune_cells
VAT_2M	1055.9	832	1.4154099	2	Singlet	FAPs
VAT_2M	8098.7	3038	0.000806	0	Singlet	Adipocyte
VAT_2M	1883.1	1378	0.6218759	2	Singlet	FAPs
VAT_2M	2954.7	1696	0.6939669	0	Singlet	Adipocyte
VAT_2M	1435.5	1034	3.4504532	7	Singlet	Endothelial_cells
VAT_2M	1355.3	1004	0.2466563	0	Singlet	Adipocyte
VAT_2M	1793.9	1291	1.7857661	2	Singlet	FAPs
VAT_2M	10233	3488	0.0139891	0	Singlet	Adipocyte
VAT_2M	3332.2	1812	0.0189269	0	Singlet	Adipocyte
VAT_2M	1635.4	1206	0.9016526	2	Singlet	FAPs
VAT_2M	1901.2	1290	0.8242561	0	Singlet	Adipocyte
VAT_2M	4108.9	2217	4.638094	5	Singlet	Immune_cells
VAT_2M	1137	869	0.2364391	7	Singlet	Endothelial_cells
VAT_2M	5662.1	2523	0.0481415	0	Singlet	Adipocyte
VAT_2M	1146.5	836	1.0254329	5	Singlet	Immune_cells
VAT_2M	4502.7	2092	0.1578904	0	Singlet	Adipocyte
VAT_2M	1715.4	1263	2.5823617	5	Singlet	Immune_cells
VAT_2M	1799.7	1288	0.5139259	8	Singlet	Mesothelial
VAT_2M	11023	3438	0.0677105	0	Singlet	Adipocyte
VAT_2M	1774.4	1114	0.1388989	0	Singlet	Adipocyte
VAT_2M	1909.1	1132	1.0749775	0	Singlet	Adipocyte
VAT_2M	6258.6	2603	0.0574268	0	Singlet	Adipocyte
VAT_2M	5618.6	2436	0	0	Singlet	Adipocyte
VAT_2M	2732.4	1609	0.2021981	0	Singlet	Adipocyte
VAT_2M	9190.6	3258	0.0489985	0	Singlet	Adipocyte
VAT_2M	5369.9	2394	0.0268298	0	Singlet	Adipocyte

VAT_2M	1660.3	1342	1.0731803	6	Singlet	Neurons
VAT_2M	2049.6	1485	1.5295914	6	Singlet	Neurons
VAT_2M	1276.7	895	5.1085532	2	Singlet	FAPs
VAT_2M	1548.4	1087	1.0128632	11	Singlet	Smooth_muscle_cells
VAT_2M	8564.7	3204	0.8429481	0	Singlet	Adipocyte
VAT_2M	2635.1	1534	0.9136559	0	Singlet	Adipocyte
VAT_2M	1664.1	1315	2.5320683	5	Singlet	Immune_cells
VAT_2M	1308.4	964	0.8151149	5	Singlet	Immune_cells
VAT_2M	6621	2738	0.1382574	0	Singlet	Adipocyte
VAT_2M	1272.8	968	2.0935931	5	Singlet	Immune_cells
VAT_2M	10897	3444	0	0	Singlet	Adipocyte
VAT_2M	1076.1	787	2.1353496	6	Singlet	Neurons
VAT_2M	1179.9	942	1.928964	8	Singlet	Mesothelial
VAT_2M	1165.5	909	3.4324224	7	Singlet	Endothelial_cells
VAT_2M	7299.2	2657	0.0273696	0	Singlet	Adipocyte
VAT_2M	1606.6	1215	1.1728719	2	Singlet	FAPs
VAT_2M	2507.3	1349	2.0100008	3	Singlet	FAPs
VAT_2M	1894.6	1311	0.4288606	2	Singlet	FAPs
VAT_2M	3032.3	1734	1.3515454	3	Singlet	FAPs
VAT_2M	1614.9	990	0.384256	0	Singlet	Adipocyte
VAT_2M	1267.5	954	0	5	Singlet	Immune_cells
VAT_2M	1105.4	870	0.9914626	3	Singlet	FAPs
VAT_2M	5066.8	2338	0.0169975	0	Singlet	Adipocyte
VAT_2M	5162.3	2373	0.1115574	0	Singlet	Adipocyte
VAT_2M	4125.3	2123	0.1194838	0	Singlet	Adipocyte
VAT_2M	1793	1219	1.2720898	2	Singlet	FAPs
VAT_2M	10413	3510	0.1635903	0	Singlet	Adipocyte
VAT_2M	3264.6	1755	0.0658182	0	Singlet	Adipocyte
VAT_2M	4874.6	2298	0	0	Singlet	Adipocyte
VAT_2M	5844.5	2402	0.0847946	0	Singlet	Adipocyte
VAT_2M	12580	3656	0.008668	0	Singlet	Adipocyte
VAT_2M	1163.1	840	0.2218265	5	Singlet	Immune_cells
VAT_2M	1064	875	0.3838612	9	Singlet	Mesothelial
VAT_2M	4298.3	2144	0.1434355	0	Singlet	Adipocyte
VAT_2M	4491.2	2117	0.0082536	0	Singlet	Adipocyte
VAT_2M	1800.3	1270	8.3625781	3	Singlet	FAPs
VAT_2M	5361.8	2322	0.0195399	0	Singlet	Adipocyte
VAT_2M	3202.7	1627	0.6036752	0	Singlet	Adipocyte
VAT_2M	4355.4	2002	0.0701762	0	Singlet	Adipocyte
VAT_2M	2836.8	1654	0.3075878	11	Singlet	Smooth_muscle_cells
VAT_2M	1198.8	1003	0.9480733	6	Singlet	Neurons
VAT_2M	1427.4	1001	0.6703212	2	Singlet	FAPs
VAT_2M	5021.3	2260	0.0290322	0	Singlet	Adipocyte
VAT_2M	4216.3	2267	0.3227409	0	Singlet	Adipocyte
VAT_2M	2842.2	1546	0.4030127	0	Singlet	Adipocyte
VAT_2M	1562.9	1008	0.7897962	0	Singlet	Adipocyte
VAT_2M	1379	974	1.0972229	2	Singlet	FAPs
VAT_2M	3329.3	1719	0.0727306	7	Singlet	Endothelial_cells
VAT_2M	2235.5	1368	0.0802094	5	Singlet	Immune_cells
VAT_2M	1063.9	845	0.3018627	5	Singlet	Immune_cells
VAT_2M	1228.7	926	0.9490544	3	Singlet	FAPs

VAT_2M	2489.7	1449	0.0291887	0 Singlet	Adipocyte
VAT_2M	2205.2	1411	2.2572733	6 Singlet	Neurons
VAT_2M	2856.9	1511	0.0216485	0 Singlet	Adipocyte
VAT_2M	4480.9	2307	0.1774695	0 Singlet	Adipocyte
VAT_2M	2746.2	1619	1.175749	0 Singlet	Adipocyte
VAT_2M	1745.4	1178	3.5489378	2 Singlet	FAPs
VAT_2M	1478.7	1101	2.6214053	9 Singlet	Mesothelial
VAT_2M	1259.4	1032	3.3341923	6 Singlet	Neurons
VAT_2M	1054	859	2.4397195	8 Singlet	Mesothelial
VAT_2M	1957.8	1387	1.5054533	3 Singlet	FAPs
VAT_2M	2741.2	1496	0.1540178	0 Singlet	Adipocyte
VAT_2M	2583.6	1557	0.2070198	3 Singlet	FAPs
VAT_2M	1130.3	894	2.432866	7 Singlet	Endothelial_cells
VAT_2M	6499	2707	0.0239568	0 Singlet	Adipocyte
VAT_2M	5395.5	2582	1.7683935	2 Singlet	FAPs
VAT_2M	7201.6	2711	0.0024969	0 Singlet	Adipocyte
VAT_2M	3736.4	1983	0.3928629	0 Singlet	Adipocyte
VAT_2M	4227.4	2052	0.1131904	0 Singlet	Adipocyte
VAT_2M	7564	3005	0.0070278	0 Singlet	Adipocyte
VAT_2M	2453.7	1525	1.5310274	2 Singlet	FAPs
VAT_2M	1682.3	1024	1.618383	3 Singlet	FAPs
VAT_2M	1888.3	1323	0.950302	6 Singlet	Neurons
VAT_2M	6912.9	2706	0.0373506	0 Singlet	Adipocyte
VAT_2M	2035.6	1127	0.9811712	0 Singlet	Adipocyte
VAT_2M	2991.4	1767	1.8568844	2 Singlet	FAPs
VAT_2M	2847.5	1662	0.1833093	0 Singlet	Adipocyte
VAT_2M	1716.5	1125	3.6261157	1 Singlet	Immune_cells
VAT_2M	2344.7	1498	0.5157085	6 Singlet	Neurons
VAT_2M	1568.3	1088	3.7426176	1 Singlet	Immune_cells
VAT_2M	8993.2	3136	0.0183852	0 Singlet	Adipocyte
VAT_2M	2863.9	1668	3.8865335	3 Singlet	FAPs
VAT_2M	8763.8	3337	0.0107964	0 Singlet	Adipocyte
VAT_2M	2159.1	1338	2.5093188	3 Singlet	FAPs
VAT_2M	4582.5	2150	0	0 Singlet	Adipocyte
VAT_2M	1065.4	710	4.1034735	6 Singlet	Neurons
VAT_2M	1394.8	1093	0.6508783	9 Singlet	Mesothelial
VAT_2M	2340.1	1351	1.7732097	5 Singlet	Immune_cells
VAT_2M	3321.5	1849	0.9846208	0 Singlet	Adipocyte
VAT_2M	5702.4	2317	0.3114708	0 Singlet	Adipocyte
VAT_2M	3063.2	1801	1.1638749	5 Singlet	Immune_cells
VAT_2M	1857.2	1190	2.2953931	3 Singlet	FAPs
VAT_2M	1002.7	749	1.1226864	7 Singlet	Endothelial_cells
VAT_2M	1162.2	920	0.9997682	6 Singlet	Neurons
VAT_2M	2613.4	1561	2.0230296	6 Singlet	Neurons
VAT_2M	6886.1	2697	0.9316553	0 Singlet	Adipocyte
VAT_2M	2672.2	1550	0.9060542	3 Singlet	FAPs
VAT_2M	3513	1861	0.2773708	0 Singlet	Adipocyte
VAT_2M	1702	1221	0.8660172	6 Singlet	Neurons
VAT_2M	1435.2	999	0.3806991	2 Singlet	FAPs
VAT_2M	3952.2	1900	0.2225455	0 Singlet	Adipocyte
VAT_2M	4001.1	2029	1.3281254	3 Singlet	FAPs

VAT_2M	5214.8	2336	0.102628	0	Singlet	Adipocyte
VAT_2M	2042.2	1293	2.9518403	3	Singlet	FAPs
VAT_2M	1031.7	915	2.4026747	6	Singlet	Neurons
VAT_2M	1525.5	1151	1.4883386	3	Singlet	FAPs
VAT_2M	9795.6	3187	0.0678042	0	Singlet	Adipocyte
VAT_2M	1117.3	935	1.9361166	2	Singlet	FAPs
VAT_2M	3792.8	1979	0.0563966	0	Singlet	Adipocyte
VAT_2M	6534	2835	1.4790808	0	Singlet	Adipocyte
VAT_2M	11323	3497	0.061203	0	Singlet	Adipocyte
VAT_2M	1681.5	1140	1.0317107	0	Singlet	Adipocyte
VAT_2M	2392.8	1502	0.1476887	3	Singlet	FAPs
VAT_2M	1731.5	1261	0.8954277	6	Singlet	Neurons
VAT_2M	1017.5	800	0.6106362	7	Singlet	Endothelial_cells
VAT_2M	2524.8	1584	0.7608123	0	Singlet	Adipocyte
VAT_2M	2915.4	1623	0.0259285	0	Singlet	Adipocyte
VAT_2M	2639.4	1456	0.2487557	0	Singlet	Adipocyte
VAT_2M	2705.4	1651	0.8643671	2	Singlet	FAPs
VAT_2M	2106.2	1219	1.9334733	5	Singlet	Immune_cells
VAT_2M	3334.1	1693	0.2835872	0	Singlet	Adipocyte
VAT_2M	6794.2	2715	0.0376449	0	Singlet	Adipocyte
VAT_2M	5083.8	2320	0.4341145	0	Singlet	Adipocyte
VAT_2M	4441.6	2017	0.0721478	0	Singlet	Adipocyte
VAT_2M	5328.8	2193	0.0087535	0	Singlet	Adipocyte
VAT_2M	4222	2162	0.8740102	0	Singlet	Adipocyte
VAT_2M	1777.5	1122	1.0906385	0	Singlet	Adipocyte
VAT_2M	1031.9	876	1.8505544	0	Singlet	Adipocyte
VAT_2M	1144	850	1.0620922	6	Singlet	Neurons
VAT_2M	7141.4	2842	0.0076807	0	Singlet	Adipocyte
VAT_2M	2341.9	1868	4.802253	6	Singlet	Neurons
VAT_2M	1234.8	958	0.6267946	2	Singlet	FAPs
VAT_2M	14179	3828	0	0	Singlet	Adipocyte
VAT_2M	9697.2	3218	0	0	Singlet	Adipocyte
VAT_2M	5304	2458	0.2115485	0	Singlet	Adipocyte
VAT_2M	3103.2	1730	0.0557068	0	Singlet	Adipocyte
VAT_2M	8202.1	3096	0.001821	0	Singlet	Adipocyte
VAT_2M	1730.1	1192	1.0482116	1	Singlet	Immune_cells
VAT_2M	2797.6	1521	0.773283	2	Singlet	FAPs
VAT_2M	1765.8	969	2.4530918	5	Singlet	Immune_cells
VAT_2M	2595.3	1529	0.3029902	0	Singlet	Adipocyte
VAT_2M	1519.6	1081	0.7015801	2	Singlet	FAPs
VAT_2M	1762.7	1244	2.2908184	5	Singlet	Immune_cells
VAT_2M	1096.2	798	0.8623014	0	Singlet	Adipocyte
VAT_2M	7758	3105	0	0	Singlet	Adipocyte
VAT_2M	4254.4	2238	1.1487027	2	Singlet	FAPs
VAT_2M	2272.3	1474	0.2232857	0	Singlet	Adipocyte
VAT_2M	1056.3	841	2.5498712	5	Singlet	Immune_cells
VAT_2M	1940	1397	11.928149	6	Singlet	Neurons
VAT_2M	1862.3	1237	0.2464201	3	Singlet	FAPs
VAT_2M	2086.5	1429	1.8421013	3	Singlet	FAPs
VAT_2M	8664.6	3118	0	0	Singlet	Adipocyte
VAT_2M	1877.1	1164	0.4628728	7	Singlet	Endothelial_cells

VAT_2M	1109.8	947	1.950411	6 Singlet	Neurons
VAT_2M	2367.4	1684	0.1038412	3 Singlet	FAPs
VAT_2M	6540.9	2747	0.0173973	0 Singlet	Adipocyte
VAT_2M	2681.1	1442	1.249322	2 Singlet	FAPs
VAT_2M	1295.8	890	7.5792015	2 Singlet	FAPs
VAT_2M	1275.4	1072	1.041097	8 Singlet	Mesothelial
VAT_2M	2037.4	1315	0.3884571	5 Singlet	Immune_cells
VAT_2M	1614	1040	1.8340546	3 Singlet	FAPs
VAT_2M	4885.2	2278	0.061882	0 Singlet	Adipocyte
VAT_2M	12565	3616	0.0211488	0 Singlet	Adipocyte
VAT_2M	1896.5	1182	0.5404592	0 Singlet	Adipocyte
VAT_2M	5386.2	2509	0.8237276	0 Singlet	Adipocyte
VAT_2M	3714.2	1857	0.0169021	0 Singlet	Adipocyte
VAT_2M	7676.6	2888	0.0015703	0 Singlet	Adipocyte
VAT_2M	2543.2	1471	0.1708283	0 Singlet	Adipocyte
VAT_2M	3093	1682	0.8146311	0 Singlet	Adipocyte
VAT_2M	5231.5	2380	0.1795843	0 Singlet	Adipocyte
VAT_2M	1581.1	1003	0.9496569	7 Singlet	Endothelial_cells
VAT_2M	1177.1	862	0.10408	7 Singlet	Endothelial_cells
VAT_2M	1470.8	956	0.1457842	7 Singlet	Endothelial_cells
VAT_2M	8384.1	3123	0.0505167	0 Singlet	Adipocyte
VAT_2M	5104.7	2449	0.5154449	0 Singlet	Adipocyte
VAT_2M	2704.4	1127	13.441329	7 Singlet	Endothelial_cells
VAT_2M	1875.6	1182	0.5498537	7 Singlet	Endothelial_cells
VAT_2M	6693.7	2848	0.0093326	0 Singlet	Adipocyte
VAT_2M	1181.7	968	0.9153991	8 Singlet	Mesothelial
VAT_2M	6302.7	2473	0.0183141	0 Singlet	Adipocyte
VAT_2M	4697.6	2622	0.2047468	2 Singlet	FAPs
VAT_2M	4206.8	2031	0.0301246	0 Singlet	Adipocyte
VAT_2M	4820.1	2405	0.0305394	0 Singlet	Adipocyte
VAT_2M	1626.3	1229	2.656692	2 Singlet	FAPs
VAT_2M	11436	3534	0	0 Singlet	Adipocyte
VAT_2M	1571.5	1124	0.8334551	6 Singlet	Neurons
VAT_2M	7103.9	2909	0.0264642	0 Singlet	Adipocyte
VAT_2M	8767.4	3076	0.0844227	0 Singlet	Adipocyte
VAT_2M	4533.1	2072	0.511256	0 Singlet	Adipocyte
VAT_2M	2711.7	1548	0.0268671	0 Singlet	Adipocyte
VAT_2M	1099.6	889	1.2445647	2 Singlet	FAPs
VAT_2M	2537.8	1494	0.1220906	0 Singlet	Adipocyte
VAT_2M	1187.8	814	7.3529401	5 Singlet	Immune_cells
VAT_2M	1858.6	1167	3.107945	6 Singlet	Neurons
VAT_2M	3300.6	1937	0.5578467	3 Singlet	FAPs
VAT_2M	2243.9	1466	0.634408	2 Singlet	FAPs
VAT_2M	1032.6	880	0	8 Singlet	Mesothelial
VAT_2M	2111.2	1238	0.2261455	0 Singlet	Adipocyte
VAT_2M	2010.1	1443	2.2417042	0 Singlet	Adipocyte
VAT_2M	5823.3	2480	0.4490999	0 Singlet	Adipocyte
VAT_2M	11252	3467	0.0355381	0 Singlet	Adipocyte
VAT_2M	3227.9	1744	7.3372823	5 Singlet	Immune_cells
VAT_2M	2193.2	1265	0	0 Singlet	Adipocyte
VAT_2M	1881.4	1157	0.3292007	0 Singlet	Adipocyte

VAT_2M	1031	780	1.1362461	9	Singlet	Mesothelial
VAT_2M	1458.1	1016	4.6130789	6	Singlet	Neurons
VAT_2M	2442.5	1665	0.2159991	3	Singlet	FAPs
VAT_2M	1105.5	928	0.1761497	9	Singlet	Mesothelial
VAT_2M	5556.7	2365	0.0703712	0	Singlet	Adipocyte
VAT_2M	4594.5	2199	0.5719337	0	Singlet	Adipocyte
VAT_2M	3649.8	1907	0.85758	0	Singlet	Adipocyte
VAT_2M	6608	2625	0.0356205	0	Singlet	Adipocyte
VAT_2M	2055.7	1433	0.3878206	6	Singlet	Neurons
VAT_2M	2141.1	1314	1.2069947	7	Singlet	Endothelial_cells
VAT_2M	4144	1942	0.1335243	0	Singlet	Adipocyte
VAT_2M	4558	2203	0.1744509	0	Singlet	Adipocyte
VAT_2M	2618.5	1491	0.5143567	0	Singlet	Adipocyte
VAT_2M	6447.8	2598	0.3856463	0	Singlet	Adipocyte
VAT_2M	3340.2	1701	0.0515714	0	Singlet	Adipocyte
VAT_2M	1034.3	876	1.3632234	6	Singlet	Neurons
VAT_2M	7803	3051	0.0333536	0	Singlet	Adipocyte
VAT_2M	1188.9	875	2.1478938	2	Singlet	FAPs
VAT_2M	1105.2	744	5.4636199	4	Singlet	Immune_cells
VAT_2M	1066.8	827	0.4834199	7	Singlet	Endothelial_cells
VAT_2M	1395.9	1101	0.3764649	4	Singlet	Immune_cells
VAT_2M	1952.9	1343	1.6633387	8	Singlet	Mesothelial
VAT_2M	2736.2	1593	1.436106	0	Singlet	Adipocyte
VAT_2M	3772.8	1891	0.0607936	0	Singlet	Adipocyte
VAT_2M	1733	1162	0.7433368	0	Singlet	Adipocyte
VAT_2M	1538.1	1153	0.3279624	2	Singlet	FAPs
VAT_2M	1026.5	828	0.9012612	5	Singlet	Immune_cells
VAT_2M	1240.8	1000	0.5535335	11	Singlet	Smooth_muscle_cells
VAT_2M	1367.5	1107	1.3160426	11	Singlet	Smooth_muscle_cells
VAT_2M	5014.8	2328	0.0940861	0	Singlet	Adipocyte
VAT_2M	1033.8	822	2.1000237	5	Singlet	Immune_cells
VAT_2M	1152.5	820	3.6267385	2	Singlet	FAPs
VAT_2M	9201.1	3353	0.5290152	0	Singlet	Adipocyte
VAT_2M	1947.4	1305	0.6012909	7	Singlet	Endothelial_cells
VAT_2M	1955.1	1070	0.0893582	0	Singlet	Adipocyte
VAT_2M	1643.1	1175	1.1502235	8	Singlet	Mesothelial
VAT_2M	1486.1	1220	0.889879	5	Singlet	Immune_cells
VAT_2M	6091.4	2483	0.0740412	0	Singlet	Adipocyte
VAT_2M	6333.6	2679	0.0041423	0	Singlet	Adipocyte
VAT_2M	1762.2	1208	0.0578577	2	Singlet	FAPs
VAT_2M	1930.7	1494	0.616895	3	Singlet	FAPs
VAT_2M	4011.4	2076	0.1229929	0	Singlet	Adipocyte
VAT_2M	1050.1	736	0.2674774	3	Singlet	FAPs
VAT_2M	2241.9	1537	1.1104667	3	Singlet	FAPs
VAT_2M	1284.1	875	3.5069877	2	Singlet	FAPs
VAT_2M	9606.4	3282	0.1462846	0	Singlet	Adipocyte
VAT_2M	2360.3	1364	5.4310636	0	Singlet	Adipocyte
VAT_2M	4955.6	2083	0.0087484	0	Singlet	Adipocyte
VAT_2M	1198.2	831	2.3382244	2	Singlet	FAPs
VAT_2M	9422.6	3276	0.0019775	0	Singlet	Adipocyte
VAT_2M	2360.8	1638	2.896705	2	Singlet	FAPs

VAT_2M	3129.4	1803	0.714135	0	Singlet	Adipocyte
VAT_2M	2115.2	1258	2.2123736	7	Singlet	Endothelial_cells
VAT_2M	2205.9	1421	1.9126324	3	Singlet	FAPs
VAT_2M	1009.4	822	4.6536275	4	Singlet	Immune_cells
VAT_2M	1512.3	980	1.2570793	2	Singlet	FAPs
VAT_2M	1839.2	1121	0.893325	3	Singlet	FAPs
VAT_2M	1600.9	1070	1.6011033	3	Singlet	FAPs
VAT_2M	2339.9	1674	0.3125979	3	Singlet	FAPs
VAT_2M	4614.9	2294	0.0759512	0	Singlet	Adipocyte
VAT_2M	9880.1	3352	0.0012175	0	Singlet	Adipocyte
VAT_2M	2899.1	1556	0.1609007	0	Singlet	Adipocyte
VAT_2M	1629.7	1206	1.467875	6	Singlet	Neurons
VAT_2M	1647.3	1264	1.0814435	8	Singlet	Mesothelial
VAT_2M	4035.1	2338	0.4735812	5	Singlet	Immune_cells
VAT_2M	4013.9	2183	0.4569869	3	Singlet	FAPs
VAT_2M	3773.5	1972	0.1926039	2	Singlet	FAPs
VAT_2M	1033.4	843	1.6918106	6	Singlet	Neurons
VAT_2M	1136.5	912	3.6521396	6	Singlet	Neurons
VAT_2M	1651.6	1125	0.469036	5	Singlet	Immune_cells
VAT_2M	9645	3400	0.0102611	0	Singlet	Adipocyte
VAT_2M	1817.6	1267	1.9840894	3	Singlet	FAPs
VAT_2M	2807	1813	4.1592256	3	Singlet	FAPs
VAT_2M	1185.3	845	1.2384668	5	Singlet	Immune_cells
VAT_2M	4052.7	2132	0.0161431	0	Singlet	Adipocyte
VAT_2M	2483.2	1369	0.1316616	0	Singlet	Adipocyte
VAT_2M	4406.8	2210	0.0467816	0	Singlet	Adipocyte
VAT_2M	2267.8	1495	0.0848039	5	Singlet	Immune_cells
VAT_2M	1184.6	943	1.7287995	11	Singlet	Smooth_muscle_cells
VAT_2M	1726.8	1085	0.0577892	7	Singlet	Endothelial_cells
VAT_2M	4759.9	2109	0.1591786	0	Singlet	Adipocyte
VAT_2M	3704.6	1871	1.5290582	0	Singlet	Adipocyte
VAT_2M	6335.5	2760	0.5194168	0	Singlet	Adipocyte
VAT_2M	6498	2580	0.0414888	0	Singlet	Adipocyte
VAT_2M	5855.5	2610	0.0162946	0	Singlet	Adipocyte
VAT_2M	5356.3	2342	0.0087969	0	Singlet	Adipocyte
VAT_2M	2321.2	1321	0.4231759	0	Singlet	Adipocyte
VAT_2M	5890.7	2405	0	0	Singlet	Adipocyte
VAT_2M	1975.2	1280	6.4820982	6	Singlet	Neurons
VAT_2M	2660.4	1542	3.1185963	2	Singlet	FAPs
VAT_2M	2718.1	1665	0.6802594	7	Singlet	Endothelial_cells
VAT_2M	1568.7	1003	0.4681687	2	Singlet	FAPs
VAT_2M	2080.9	1371	2.9166577	2	Singlet	FAPs
VAT_2M	2309.2	1314	0.1211337	0	Singlet	Adipocyte
VAT_2M	1569.1	1099	0.7110865	0	Singlet	Adipocyte
VAT_2M	1297.4	925	8.5783711	1	Singlet	Immune_cells
VAT_2M	1681.7	1080	0.7838466	0	Singlet	Adipocyte
VAT_2M	7108.9	2767	0.0030799	0	Singlet	Adipocyte
VAT_2M	1196.4	880	2.2149473	3	Singlet	FAPs
VAT_2M	1517.1	1042	3.9128729	1	Singlet	Immune_cells
VAT_2M	2547.7	1494	0.6793861	0	Singlet	Adipocyte
VAT_2M	1704.1	1100	1.3854623	3	Singlet	FAPs

VAT_2M	3022	1661	0.0380698	0	Singlet	Adipocyte
VAT_2M	8784.6	3149	0.2475582	0	Singlet	Adipocyte
VAT_2M	1105.6	916	0.7942244	7	Singlet	Endothelial_cells
VAT_2M	2379.6	1385	0.0705073	0	Singlet	Adipocyte
VAT_2M	6238.4	2539	0.0325286	0	Singlet	Adipocyte
VAT_2M	1198.4	917	1.9785606	5	Singlet	Immune_cells
VAT_2M	1519	1056	0.2954088	0	Singlet	Adipocyte
VAT_2M	6385.6	2703	0.1314521	0	Singlet	Adipocyte
VAT_2M	8662.4	3132	0.032989	0	Singlet	Adipocyte
VAT_2M	1944.5	1423	2.0973879	5	Singlet	Immune_cells
VAT_2M	1719.7	1262	0.9210484	2	Singlet	FAPs
VAT_2M	2111.3	1307	0.9202537	3	Singlet	FAPs
VAT_2M	4810.3	2238	0.0578482	0	Singlet	Adipocyte
VAT_2M	4619	2056	0.1385742	0	Singlet	Adipocyte
VAT_2M	7052.6	2859	0.0004054	0	Singlet	Adipocyte
VAT_2M	1401.7	981	0.250918	2	Singlet	FAPs
VAT_2M	6444.3	2720	0.0342265	0	Singlet	Adipocyte
VAT_2M	2047.4	1396	0.7707932	2	Singlet	FAPs
VAT_2M	2283	1410	1.6480136	0	Singlet	Adipocyte
VAT_2M	7399.6	2861	0.1096136	0	Singlet	Adipocyte
VAT_2M	5195.9	2351	0.0107817	0	Singlet	Adipocyte
VAT_2M	1478.8	1211	1.4755331	6	Singlet	Neurons
VAT_2M	1567.1	1069	2.0919737	3	Singlet	FAPs
VAT_2M	10800	3167	0.0903626	0	Singlet	Adipocyte
VAT_2M	4675.4	2204	0.366359	0	Singlet	Adipocyte
VAT_2M	5641.5	2545	0	0	Singlet	Adipocyte
VAT_2M	1471.3	1045	0.8847991	7	Singlet	Endothelial_cells
VAT_2M	1779	1093	0.2884281	0	Singlet	Adipocyte
VAT_2M	2993.9	1880	1.1790302	5	Singlet	Immune_cells
VAT_2M	1104.6	843	0.8980232	2	Singlet	FAPs
VAT_2M	3800	1879	0.6714945	0	Singlet	Adipocyte
VAT_2M	1565.6	1134	0.9043496	3	Singlet	FAPs
VAT_2M	2346.3	1555	4.214297	5	Singlet	Immune_cells
VAT_2M	8052.4	3004	0.1088292	0	Singlet	Adipocyte
VAT_2M	1685.9	1129	1.3569292	2	Singlet	FAPs
VAT_2M	3089.3	1844	0.1177349	3	Singlet	FAPs
VAT_2M	4953	2287	0	0	Singlet	Adipocyte
VAT_2M	6648.5	2780	0.0281887	0	Singlet	Adipocyte
VAT_2M	1118.1	865	1.8612787	11	Singlet	Smooth_muscle_cells
VAT_2M	1534.3	1190	2.1807936	10	Singlet	Immune_cells
VAT_2M	1113.9	872	1.2455115	7	Singlet	Endothelial_cells
VAT_2M	6562.3	2728	0.0757809	0	Singlet	Adipocyte
VAT_2M	1689.5	853	2.4514956	2	Singlet	FAPs
VAT_2M	5328.7	2638	1.9642853	3	Singlet	FAPs
VAT_2M	1242.4	972	3.1003441	11	Singlet	Smooth_muscle_cells
VAT_2M	6900.5	2729	0.0435448	0	Singlet	Adipocyte
VAT_2M	5080.2	2261	0.0317788	0	Singlet	Adipocyte
VAT_2M	4691.7	2186	0.0219494	0	Singlet	Adipocyte
VAT_2M	7865.4	2942	0.0136917	0	Singlet	Adipocyte
VAT_2M	1275.5	923	1.0658131	3	Singlet	FAPs
VAT_2M	1231	873	2.2229542	9	Singlet	Mesothelial

VAT_2M	2987.8	1764	0.2999986	3	Singlet	FAPs
VAT_2M	4314.5	2181	0.2704558	0	Singlet	Adipocyte
VAT_2M	1492.4	1089	0.5012316	5	Singlet	Immune_cells
VAT_2M	2327	1488	0.2727158	0	Singlet	Adipocyte
VAT_2M	8204.8	3082	0.046082	0	Singlet	Adipocyte
VAT_2M	2649.1	1604	0.9725944	5	Singlet	Immune_cells
VAT_2M	1105.2	920	1.347101	1	Singlet	Immune_cells
VAT_2M	1471.8	1070	1.1619555	2	Singlet	FAPs
VAT_2M	10645	3283	0.0112437	0	Singlet	Adipocyte
VAT_2M	4472.6	2338	0.9701477	3	Singlet	FAPs
VAT_2M	1458.7	1065	0.6373432	3	Singlet	FAPs
VAT_2M	9109.3	3104	0.0817824	0	Singlet	Adipocyte
VAT_2M	2106	1347	1.3219216	2	Singlet	FAPs
VAT_2M	9306.2	3194	0.3233081	0	Singlet	Adipocyte
VAT_2M	1984	1385	0.9030167	2	Singlet	FAPs
VAT_2M	2477.4	1494	0.4911877	5	Singlet	Immune_cells
VAT_2M	2476.8	1656	2.9510679	5	Singlet	Immune_cells
VAT_2M	1143.8	922	0.9406861	6	Singlet	Neurons
VAT_2M	2624.8	1333	0.4159001	0	Singlet	Adipocyte
VAT_2M	1633.9	1235	0.6036652	5	Singlet	Immune_cells
VAT_2M	2397.8	1177	0.7689428	7	Singlet	Endothelial_cells
VAT_2M	1719.2	1040	0.1841378	0	Singlet	Adipocyte
VAT_2M	4597.9	2093	0.1067639	0	Singlet	Adipocyte
VAT_2M	1340.8	975	1.802149	1	Singlet	Immune_cells
VAT_2M	1793	1303	0.5231414	6	Singlet	Neurons
VAT_2M	1318.5	1144	0.1300702	5	Singlet	Immune_cells
VAT_2M	2050.9	1487	0.5147819	6	Singlet	Neurons
VAT_2M	1909.1	1332	0.8492622	5	Singlet	Immune_cells
VAT_2M	1711.6	1052	4.6906987	3	Singlet	FAPs
VAT_2M	1945.9	1319	4.5833561	3	Singlet	FAPs
VAT_2M	2368.7	1619	0.671243	3	Singlet	FAPs
VAT_2M	3250.3	1777	0.1038246	0	Singlet	Adipocyte
VAT_2M	6388.2	2681	0.144052	0	Singlet	Adipocyte
VAT_2M	2802	1535	4.9579507	0	Singlet	Adipocyte
VAT_2M	4152.1	2158	0.3557528	0	Singlet	Adipocyte
VAT_2M	1485.1	1090	0.234808	2	Singlet	FAPs
VAT_2M	1977.2	1161	0.0397836	0	Singlet	Adipocyte
VAT_2M	1658.5	1098	1.4550938	3	Singlet	FAPs
VAT_2M	1641.4	1119	2.2858092	0	Singlet	Adipocyte
VAT_2M	6584.3	2700	0.0079914	0	Singlet	Adipocyte
VAT_2M	4342.5	2144	0.093172	0	Singlet	Adipocyte
VAT_2M	1268.5	955	0.5094676	0	Singlet	Adipocyte
VAT_2M	6702	2720	0.0196531	0	Singlet	Adipocyte
VAT_2M	3435.2	1810	0.0276679	0	Singlet	Adipocyte
VAT_2M	3112	1749	0.1303913	0	Singlet	Adipocyte
VAT_2M	1054.7	862	2.1720091	8	Singlet	Mesothelial
VAT_2M	2059.7	1350	3.2765175	2	Singlet	FAPs
VAT_2M	1725.3	1205	2.2171406	5	Singlet	Immune_cells
VAT_2M	8128.5	2998	0.0125607	0	Singlet	Adipocyte
VAT_2M	6803.1	2829	0.0317025	0	Singlet	Adipocyte
VAT_2M	5403.7	2313	0.0083603	0	Singlet	Adipocyte

VAT_2M	1036	821	0.391132	6	Singlet	Neurons
VAT_2M	14007	3769	0.3553346	0	Singlet	Adipocyte
VAT_2M	1439.1	1147	1.7382008	6	Singlet	Neurons
VAT_2M	2089.9	1235	0.115044	0	Singlet	Adipocyte
VAT_2M	1598.6	986	0.2653789	7	Singlet	Endothelial_cells
VAT_2M	2353.5	1499	0.2011789	2	Singlet	FAPs
VAT_2M	5936.5	2355	0.0185735	0	Singlet	Adipocyte
VAT_2M	4289.6	2101	0.10977	0	Singlet	Adipocyte
VAT_2M	1398.4	943	3.808435	4	Singlet	Immune_cells
VAT_2M	2688.7	1477	0.0323455	0	Singlet	Adipocyte
VAT_2M	3529.9	1846	0.4124357	0	Singlet	Adipocyte
VAT_2M	8533.7	3131	0.0657032	0	Singlet	Adipocyte
VAT_2M	5597.6	2407	0.1997766	0	Singlet	Adipocyte
VAT_2M	2846	1503	0.2021626	0	Singlet	Adipocyte
VAT_2M	5490.4	2277	0.0481013	0	Singlet	Adipocyte
VAT_2M	2237.7	1606	3.8540838	6	Singlet	Neurons
VAT_2M	2736.8	1410	0.2676737	0	Singlet	Adipocyte
VAT_2M	5572.5	2469	0.0675733	0	Singlet	Adipocyte
VAT_2M	1119.2	830	0.4592158	5	Singlet	Immune_cells
VAT_2M	1730.4	1128	2.2185957	5	Singlet	Immune_cells
VAT_2M	2014.9	1222	0.2489175	0	Singlet	Adipocyte
VAT_2M	5166.7	2414	0.1000542	0	Singlet	Adipocyte
VAT_2M	10767	3408	0	0	Singlet	Adipocyte
VAT_2M	1269.1	1021	1.8153457	11	Singlet	Smooth_muscle_cells
VAT_2M	5869.3	2569	0.1301995	0	Singlet	Adipocyte
VAT_2M	1856.3	1297	0.6643606	0	Singlet	Adipocyte
VAT_2M	1781.6	1123	0.7798106	7	Singlet	Endothelial_cells
VAT_2M	1262.7	1028	2.5001605	2	Singlet	FAPs
VAT_2M	1059.3	849	0.714599	7	Singlet	Endothelial_cells
VAT_2M	1056.9	908	0.8226538	8	Singlet	Mesothelial
VAT_2M	2433	1307	0.4137412	0	Singlet	Adipocyte
VAT_2M	5161.4	2208	0.1288389	0	Singlet	Adipocyte
VAT_2M	2455.2	1354	0.1769584	0	Singlet	Adipocyte
VAT_2M	7396.6	2821	0	0	Singlet	Adipocyte
VAT_2M	5529.3	2442	0.0079423	0	Singlet	Adipocyte
VAT_2M	6151.7	2547	0.0602222	0	Singlet	Adipocyte
VAT_2M	2124.1	1280	0.3980588	0	Singlet	Adipocyte
VAT_2M	1989.8	1256	0.9281391	0	Singlet	Adipocyte
VAT_2M	2947.6	1445	3.3770547	2	Singlet	FAPs
VAT_2M	1044.4	825	0.7613971	11	Singlet	Smooth_muscle_cells
VAT_2M	1611.7	1167	0.9157682	7	Singlet	Endothelial_cells
VAT_2M	2910.7	1504	0.1168098	0	Singlet	Adipocyte
VAT_2M	1890.8	1409	2.2200379	9	Singlet	Mesothelial
VAT_2M	1617.5	1263	0.1946477	3	Singlet	FAPs
VAT_2M	6739.7	2674	0.0263101	0	Singlet	Adipocyte
VAT_2M	2756.5	1601	0.028718	0	Singlet	Adipocyte
VAT_2M	1656.6	1142	0.8487366	0	Singlet	Adipocyte
VAT_2M	3084.9	1610	0.0976293	0	Singlet	Adipocyte
VAT_2M	1987.8	1383	1.3795188	6	Singlet	Neurons
VAT_2M	2285.8	1459	0.1565749	0	Singlet	Adipocyte
VAT_2M	2627.5	1460	0.3849571	3	Singlet	FAPs

VAT_2M	2055.3	1445	0.5416865	2	Singlet	FAPs
VAT_2M	1693.5	1073	1.964909	0	Singlet	Adipocyte
VAT_2M	2081.5	1545	0.0103571	7	Singlet	Endothelial_cells
VAT_2M	2792.6	1662	0.8601284	2	Singlet	FAPs
VAT_2M	2511.1	1732	1.6226603	11	Singlet	Smooth_muscle_cells
VAT_2M	10507	3594	0.0070276	0	Singlet	Adipocyte
VAT_2M	2795.7	1863	0.4167618	3	Singlet	FAPs
VAT_2M	1666.1	1474	1.4545196	6	Singlet	Neurons
VAT_2M	2221.9	1353	0.3748819	0	Singlet	Adipocyte
VAT_2M	9603.1	3226	0.011285	0	Singlet	Adipocyte
VAT_2M	4358.5	2061	1.4987185	0	Singlet	Adipocyte
VAT_2M	1699.1	1126	0.2039865	0	Singlet	Adipocyte
VAT_2M	1557.9	1111	0.5783349	7	Singlet	Endothelial_cells
VAT_2M	1038.3	778	1.8273123	3	Singlet	FAPs
VAT_2M	6577.3	2628	0.007656	0	Singlet	Adipocyte
VAT_2M	8063.6	2958	0.0126385	0	Singlet	Adipocyte
VAT_2M	1243.1	1012	0.8662058	7	Singlet	Endothelial_cells
VAT_2M	2938.2	1637	0.073351	0	Singlet	Adipocyte
VAT_2M	4112.9	2095	0.3773195	0	Singlet	Adipocyte
VAT_2M	1972.8	1596	2.49908	2	Singlet	FAPs
VAT_2M	2683.1	1517	0.8423986	0	Singlet	Adipocyte
VAT_2M	1895.7	1174	0.1748529	0	Singlet	Adipocyte
VAT_2M	1486.5	1045	1.8790844	5	Singlet	Immune_cells
VAT_2M	1233.1	997	1.098504	2	Singlet	FAPs
VAT_2M	3045.5	1655	0.2648217	0	Singlet	Adipocyte
VAT_2M	1780.7	1202	1.927865	0	Singlet	Adipocyte
VAT_2M	2841.3	1730	2.2261861	3	Singlet	FAPs
VAT_2M	1689.9	1137	1.5136856	6	Singlet	Neurons
VAT_2M	2334.9	1204	3.5906235	5	Singlet	Immune_cells
VAT_2M	1251.4	970	0.6747723	2	Singlet	FAPs
VAT_2M	3582.8	1859	0	0	Singlet	Adipocyte
VAT_2M	4544.5	2116	0.0507205	0	Singlet	Adipocyte
VAT_2M	1903.8	1376	1.2037434	3	Singlet	FAPs
VAT_2M	6328.5	2685	0.0406169	0	Singlet	Adipocyte
VAT_2M	9362	3201	0.1794521	0	Singlet	Adipocyte
VAT_2M	1522.9	1225	3.1249921	8	Singlet	Mesothelial
VAT_2M	1401.8	1106	3.7387849	6	Singlet	Neurons
VAT_2M	4029.7	2060	0.1885236	0	Singlet	Adipocyte
VAT_2M	11495	3735	0.0318133	0	Singlet	Adipocyte
VAT_2M	1834.1	1094	0.1414354	0	Singlet	Adipocyte
VAT_2M	1199	905	0.5716955	5	Singlet	Immune_cells
VAT_2M	1541.4	1232	0.5878204	6	Singlet	Neurons
VAT_2M	1586.7	1023	0.0909486	0	Singlet	Adipocyte
VAT_2M	1331.2	1136	2.2184734	6	Singlet	Neurons
VAT_2M	4727.3	2220	0.8068603	0	Singlet	Adipocyte
VAT_2M	3447.5	1812	0.1024032	0	Singlet	Adipocyte
VAT_2M	1581.2	1069	0.3344846	3	Singlet	FAPs
VAT_2M	4438	2039	0.0322693	0	Singlet	Adipocyte
VAT_2M	6333.3	2666	0.02519	0	Singlet	Adipocyte
VAT_2M	1913.1	1186	0.0427944	0	Singlet	Adipocyte
VAT_2M	1791.8	1336	2.6142085	2	Singlet	FAPs

VAT_2M	9607.8	3356	0	0	Singlet	Adipocyte
VAT_2M	1345.2	904	0.4634319	7	Singlet	Endothelial_cells
VAT_2M	1912.1	1296	1.1438802	2	Singlet	FAPs
VAT_2M	1121.9	901	0.7035217	2	Singlet	FAPs
VAT_2M	1842.7	1239	1.198401	2	Singlet	FAPs
VAT_2M	1517	1104	0.7378228	7	Singlet	Endothelial_cells
VAT_2M	1150.4	849	0.6039907	5	Singlet	Immune_cells
VAT_2M	4220.9	2055	0.7957061	0	Singlet	Adipocyte
VAT_2M	4968.1	2352	0.057899	0	Singlet	Adipocyte
VAT_2M	6822.6	2624	0.2426775	0	Singlet	Adipocyte
VAT_2M	3851.3	1890	0.2087895	0	Singlet	Adipocyte
VAT_2M	3152.8	1808	0.642838	3	Singlet	FAPs
VAT_2M	9805.5	3516	0.0030837	0	Singlet	Adipocyte
VAT_2M	3749.7	2262	0.8635954	2	Singlet	FAPs
VAT_2M	1232.3	901	1.6669242	3	Singlet	FAPs
VAT_2M	10380	3482	0.0367264	0	Singlet	Adipocyte
VAT_2M	2755.7	1701	0.1195037	0	Singlet	Adipocyte
VAT_2M	8336.9	3071	0.2786962	0	Singlet	Adipocyte
VAT_2M	1242.5	1073	1.3022675	6	Singlet	Neurons
VAT_2M	1064.2	778	0.0410469	2	Singlet	FAPs
VAT_2M	1313.4	975	3.5812374	2	Singlet	FAPs
VAT_2M	2195.5	1369	0.6107248	6	Singlet	Neurons
VAT_2M	1638	1056	1.0474015	0	Singlet	Adipocyte
VAT_2M	7542.1	2775	0.2250807	0	Singlet	Adipocyte
VAT_2M	1854.5	1357	0.6412132	3	Singlet	FAPs
VAT_2M	6977.2	2803	0.0347109	0	Singlet	Adipocyte
VAT_2M	2374	1593	2.0255447	5	Singlet	Immune_cells
VAT_2M	6427.2	2607	0.0033224	0	Singlet	Adipocyte
VAT_2M	1473	1142	3.4276649	5	Singlet	Immune_cells
VAT_2M	1658.1	1089	0.872092	0	Singlet	Adipocyte
VAT_2M	1499.5	1185	2.5392299	5	Singlet	Immune_cells
VAT_2M	1408.2	1012	0.6269769	2	Singlet	FAPs
VAT_2M	2584.9	1505	0.6305163	0	Singlet	Adipocyte
VAT_2M	1196.1	856	0.4385503	2	Singlet	FAPs
VAT_2M	1027	764	1.0812696	3	Singlet	FAPs
VAT_2M	1663.1	1253	0.9217284	7	Singlet	Endothelial_cells
VAT_2M	1637.6	1154	0.7341252	2	Singlet	FAPs
VAT_2M	8025.2	3100	0.1403874	0	Singlet	Adipocyte
VAT_2M	2720.8	1486	0.5415688	0	Singlet	Adipocyte
VAT_2M	3457.7	1833	0	0	Singlet	Adipocyte
VAT_2M	3472.1	1976	0.1194817	0	Singlet	Adipocyte
VAT_2M	2010.8	1356	1.4180808	3	Singlet	FAPs
VAT_2M	4685.2	2161	0.0437427	0	Singlet	Adipocyte
VAT_2M	5513.3	2473	0.0229975	0	Singlet	Adipocyte
VAT_2M	4965.2	2168	0.0604021	0	Singlet	Adipocyte
VAT_2M	1678.7	1134	0.7631477	0	Singlet	Adipocyte
VAT_2M	8847.4	3102	1.3378068	0	Singlet	Adipocyte
VAT_2M	6238.4	2632	0.0442945	0	Singlet	Adipocyte
VAT_2M	1302.3	875	0.0078351	0	Singlet	Adipocyte
VAT_2M	10307	3303	0	0	Singlet	Adipocyte
VAT_2M	6817.9	2679	0.0261378	0	Singlet	Adipocyte

VAT_2M	1447.8	1010	2.2825542	2	Singlet	FAPs
VAT_2M	1431	994	0.0422436	2	Singlet	FAPs
VAT_2M	3713.5	1833	0.0709349	0	Singlet	Adipocyte
VAT_2M	1238.2	939	1.0920021	2	Singlet	FAPs
VAT_2M	2443.9	1359	1.129485	2	Singlet	FAPs
VAT_2M	6776.7	2606	0.0776394	0	Singlet	Adipocyte
VAT_2M	6881.4	2940	0.0731245	0	Singlet	Adipocyte
VAT_2M	1256.2	939	1.9139996	7	Singlet	Endothelial_cells
VAT_2M	1523.3	1016	0.3710612	3	Singlet	FAPs
VAT_2M	6012.9	2495	0.2263577	0	Singlet	Adipocyte
VAT_2M	1024	866	2.9173399	1	Singlet	Immune_cells
VAT_2M	1008	815	2.5847772	6	Singlet	Neurons
VAT_2M	5640.4	2301	0.3247358	0	Singlet	Adipocyte
VAT_2M	6263	2632	0.3141083	0	Singlet	Adipocyte
VAT_2M	4366.8	2535	2.4684134	7	Singlet	Endothelial_cells
VAT_2M	7715	2881	0.1636405	0	Singlet	Adipocyte
VAT_2M	1024.2	749	1.6721864	7	Singlet	Endothelial_cells
VAT_2M	1150.2	885	1.1800659	3	Singlet	FAPs
VAT_2M	8418.1	3091	0.0286715	0	Singlet	Adipocyte
VAT_2M	3068.5	1780	0.4670598	2	Singlet	FAPs
VAT_2M	1249.7	913	0.0920717	6	Singlet	Neurons
VAT_2M	7136.9	2950	0.3906839	0	Singlet	Adipocyte
VAT_2M	4474.4	2080	0.3644322	0	Singlet	Adipocyte
VAT_2M	4421.1	2092	2.2423031	5	Singlet	Immune_cells
VAT_2M	4089.1	1969	0.2577922	0	Singlet	Adipocyte
VAT_2M	4422.1	2082	0.0397662	0	Singlet	Adipocyte
VAT_2M	3967.3	2303	1.6526103	3	Singlet	FAPs
VAT_2M	1069.8	711	2.3218885	8	Singlet	Mesothelial
VAT_2M	1838.1	1128	0.0885902	0	Singlet	Adipocyte
VAT_2M	1374	961	3.4339407	1	Singlet	Immune_cells
VAT_2M	2637.5	1510	0.5973078	0	Singlet	Adipocyte
VAT_2M	1599.8	1132	1.1194901	7	Singlet	Endothelial_cells
VAT_2M	1580.1	1165	1.6105875	9	Singlet	Mesothelial
VAT_2M	8388.5	3017	0.0507494	0	Singlet	Adipocyte
VAT_2M	1634.4	896	0.9189304	0	Singlet	Adipocyte
VAT_2M	1812.5	1074	1.5622037	3	Singlet	FAPs
VAT_2M	2937.9	1650	0.3572848	0	Singlet	Adipocyte
VAT_2M	4753.2	2177	1.159685	0	Singlet	Adipocyte
VAT_2M	3901.4	1927	0.0927172	0	Singlet	Adipocyte
VAT_2M	6545	2759	0.0056407	0	Singlet	Adipocyte
VAT_2M	8397.4	3056	0.0263444	0	Singlet	Adipocyte
VAT_2M	1213.1	883	3.2457945	9	Singlet	Mesothelial
VAT_2M	3716.4	1790	0.4298466	0	Singlet	Adipocyte
VAT_2M	5271.6	2289	0.1300927	0	Singlet	Adipocyte
VAT_2M	3032.2	1613	1.3297124	0	Singlet	Adipocyte
VAT_2M	2639.5	1509	0.1800727	0	Singlet	Adipocyte
VAT_2M	7750.4	2878	0.244264	0	Singlet	Adipocyte
VAT_2M	1386.6	1077	0.9641211	2	Singlet	FAPs
VAT_2M	4566.4	2227	0.0137348	0	Singlet	Adipocyte
VAT_2M	2280	1419	0.2819506	0	Singlet	Adipocyte
VAT_2M	1543.1	1057	0.5850658	7	Singlet	Endothelial_cells

VAT_2M	9667.5	3400	0.0662857	0	Singlet	Adipocyte
VAT_2M	2117.2	1384	0.5264891	7	Singlet	Endothelial_cells
VAT_2M	9560.1	3498	0.1325673	0	Singlet	Adipocyte
VAT_2M	1320.5	883	4.3103427	9	Singlet	Mesothelial
VAT_2M	2015.4	1300	1.0154337	0	Singlet	Adipocyte
VAT_2M	1827.9	1269	2.1330857	6	Singlet	Neurons
VAT_2M	6492.7	2712	0.0668799	0	Singlet	Adipocyte
VAT_2M	8666	2837	0.0350656	0	Singlet	Adipocyte
VAT_2M	1300.2	975	0.037628	3	Singlet	FAPs
VAT_2M	7612.1	2920	0.030185	0	Singlet	Adipocyte
VAT_2M	2352.6	1479	4.7302636	7	Singlet	Endothelial_cells
VAT_2M	2019.3	1505	0.2797124	5	Singlet	Immune_cells
VAT_2M	1758.3	1374	2.2147	2	Singlet	FAPs
VAT_2M	2148.2	1223	0.1643924	0	Singlet	Adipocyte
VAT_2M	3897.7	1933	0	11	Singlet	Smooth_muscle_cells
VAT_2M	3587	1948	0.9617572	2	Singlet	FAPs
VAT_2M	2188.3	1314	1.6617983	2	Singlet	FAPs
VAT_2M	8181.2	3120	0.0869175	0	Singlet	Adipocyte
VAT_2M	1077	905	2.6745646	6	Singlet	Neurons
VAT_2M	4535.9	2036	0.0086198	0	Singlet	Adipocyte
VAT_2M	6163.3	2600	0.2703688	0	Singlet	Adipocyte
VAT_2M	2863	1760	0.5121707	0	Singlet	Adipocyte
VAT_2M	6727.2	2618	0.0035814	0	Singlet	Adipocyte
VAT_2M	1003.1	819	1.2202214	1	Singlet	Immune_cells
VAT_2M	6611.2	2548	0.0692997	0	Singlet	Adipocyte
VAT_2M	1301.3	919	2.8658608	2	Singlet	FAPs
VAT_2M	2393.2	1574	0.4889436	6	Singlet	Neurons
VAT_2M	5193	2390	0.011322	0	Singlet	Adipocyte
VAT_2M	3715	1853	0	0	Singlet	Adipocyte
VAT_2M	2355.8	1466	0	11	Singlet	Smooth_muscle_cells
VAT_2M	3467.1	1977	0.5188379	0	Singlet	Adipocyte
VAT_2M	1574.1	1059	0.7505178	3	Singlet	FAPs
VAT_2M	3744.3	1985	1.7112081	0	Singlet	Adipocyte
VAT_2M	1997.9	1541	1.1037921	11	Singlet	Smooth_muscle_cells
VAT_2M	3587.6	1906	0.2711513	0	Singlet	Adipocyte
VAT_2M	3157.5	1837	0.6161469	3	Singlet	FAPs
VAT_2M	2843.9	1535	0.1185075	0	Singlet	Adipocyte
VAT_2M	13737	3863	0.0009457	0	Singlet	Adipocyte
VAT_2M	7499.5	2847	0.0129499	0	Singlet	Adipocyte
VAT_2M	1363.1	949	3.1743865	9	Singlet	Mesothelial
VAT_2M	1750	1532	0.5411567	6	Singlet	Neurons
VAT_2M	2029	1517	0.9607432	2	Singlet	FAPs
VAT_2M	2656.3	1602	0.1658839	0	Singlet	Adipocyte
VAT_2M	7561.9	2980	0.0597223	0	Singlet	Adipocyte
VAT_2M	4625.8	2173	0.01075	0	Singlet	Adipocyte
VAT_2M	1263.3	1029	1.4026647	6	Singlet	Neurons
VAT_2M	8439.6	3077	0.0251519	0	Singlet	Adipocyte
VAT_2M	1050.2	782	3.0184249	9	Singlet	Mesothelial
VAT_2M	4943.4	2292	0.0207237	0	Singlet	Adipocyte
VAT_2M	1083.5	883	1.258132	8	Singlet	Mesothelial
VAT_2M	1736.7	1265	1.1813592	2	Singlet	FAPs

VAT_2M	4475.3	2091	0.5744993	0 Singlet	Adipocyte
VAT_2M	2285.2	1251	0.0091505	0 Singlet	Adipocyte
VAT_2M	3450.7	1843	0.4939292	0 Singlet	Adipocyte
VAT_2M	5711.5	2361	0.0148504	0 Singlet	Adipocyte
VAT_2M	6752.7	2950	0.0363223	0 Singlet	Adipocyte
VAT_2M	4161.7	2044	0.1501656	0 Singlet	Adipocyte
VAT_2M	1197.9	929	2.7981597	6 Singlet	Neurons
VAT_2M	3233.7	1740	0.0467626	0 Singlet	Adipocyte
VAT_2M	1872.6	1259	0.8277409	0 Singlet	Adipocyte
VAT_2M	1027	746	4.7261598	2 Singlet	FAPs
VAT_2M	1502	1083	7.1812386	6 Singlet	Neurons
VAT_2M	1375.8	972	0.9456132	3 Singlet	FAPs
VAT_2M	8173.9	2796	0.0208354	0 Singlet	Adipocyte
VAT_2M	4211.8	2060	0.0124735	0 Singlet	Adipocyte
VAT_2M	1909.6	1339	1.6140063	5 Singlet	Immune_cells
VAT_2M	5877.2	2547	0.0574391	0 Singlet	Adipocyte
VAT_2M	4018.9	1838	0.8673792	2 Singlet	FAPs
VAT_2M	1806.8	1245	0.2697591	0 Singlet	Adipocyte
VAT_2M	1252.6	1032	0.9028267	6 Singlet	Neurons
VAT_2M	2378.8	1509	1.6753478	3 Singlet	FAPs
VAT_2M	7162.8	2872	0.0091441	0 Singlet	Adipocyte
VAT_2M	3823.8	1952	0.0177676	0 Singlet	Adipocyte
VAT_2M	1348.1	1036	0.6522067	1 Singlet	Immune_cells
VAT_2M	2590.5	1572	2.2121708	5 Singlet	Immune_cells
VAT_2M	6622	2435	0.0014534	0 Singlet	Adipocyte
VAT_2M	8529.5	3187	0.0120895	0 Singlet	Adipocyte
VAT_2M	1039.1	713	3.5642035	5 Singlet	Immune_cells
VAT_2M	3607.8	1719	0.0340529	0 Singlet	Adipocyte
VAT_2M	1430.7	995	1.6841683	3 Singlet	FAPs
VAT_2M	5562.6	2402	0.0078652	0 Singlet	Adipocyte
VAT_2M	2057.5	1280	0.7135538	0 Singlet	Adipocyte
VAT_2M	7637.5	2983	0.0612842	0 Singlet	Adipocyte
VAT_2M	1483.4	1069	0.2774006	3 Singlet	FAPs
VAT_2M	2225.3	1403	0.2579077	0 Singlet	Adipocyte
VAT_2M	5997.3	2457	0.4379272	0 Singlet	Adipocyte
VAT_2M	2754.2	1726	0.026128	0 Singlet	Adipocyte
VAT_2M	1116.9	826	2.0511266	8 Singlet	Mesothelial
VAT_2M	5194	2316	0.029928	0 Singlet	Adipocyte
VAT_2M	2983.9	1790	0.0128481	3 Singlet	FAPs
VAT_2M	7000.9	2720	0.0857617	0 Singlet	Adipocyte
VAT_2M	3947.1	2150	0	0 Singlet	Adipocyte
VAT_2M	3347.1	1866	0.8723195	0 Singlet	Adipocyte
VAT_2M	1058.2	806	0.2657063	5 Singlet	Immune_cells
VAT_2M	2992.2	1735	0.0108895	2 Singlet	FAPs
VAT_2M	1126.5	866	1.4923011	3 Singlet	FAPs
VAT_2M	1501	1029	11.407519	2 Singlet	FAPs
VAT_2M	5980	2388	0.062674	0 Singlet	Adipocyte
VAT_2M	1727.1	1243	1.2343914	9 Singlet	Mesothelial
VAT_2M	4299.1	2017	0.0573174	0 Singlet	Adipocyte
VAT_2M	2071.2	1384	0.4443959	6 Singlet	Neurons
VAT_2M	1956.9	1354	0.1745899	5 Singlet	Immune_cells

VAT_2M	2860.6	1644	0.3769869	2	Singlet	FAPs
VAT_2M	1360.3	1025	1.1347343	5	Singlet	Immune_cells
VAT_2M	1720.3	1272	0.8449473	11	Singlet	Smooth_muscle_cells
VAT_2M	1126.4	817	2.0124115	9	Singlet	Mesothelial
VAT_2M	1907.8	1168	1.1038966	0	Singlet	Adipocyte
VAT_2M	3377.2	1827	0.0162324	0	Singlet	Adipocyte
VAT_2M	1138.6	930	1.8345134	10	Singlet	Immune_cells
VAT_2M	1371.2	1026	2.5199068	2	Singlet	FAPs
VAT_2M	4233.3	1951	0.0744423	0	Singlet	Adipocyte
VAT_2M	1124.6	890	0.9690644	9	Singlet	Mesothelial
VAT_2M	1731.8	1050	0.171363	0	Singlet	Adipocyte
VAT_2M	6193.7	2502	0.0702087	0	Singlet	Adipocyte
VAT_2M	7977.4	2979	0.0194965	0	Singlet	Adipocyte
VAT_2M	4625.9	2213	0.2808472	0	Singlet	Adipocyte
VAT_2M	1660.9	1227	1.4034471	2	Singlet	FAPs
VAT_2M	1854.5	1080	0.0391226	7	Singlet	Endothelial_cells
VAT_2M	4508.5	2121	0.0333547	0	Singlet	Adipocyte
VAT_2M	2091.5	1390	1.3174059	0	Singlet	Adipocyte
VAT_2M	5571.8	2443	0.0071287	0	Singlet	Adipocyte
VAT_2M	2379.5	1641	2.8451496	2	Singlet	FAPs
VAT_2M	1008.5	800	3.6416402	2	Singlet	FAPs
VAT_2M	1597.5	1248	2.2305516	3	Singlet	FAPs
VAT_2M	1774.7	1167	1.0954899	0	Singlet	Adipocyte
VAT_2M	2935	1723	0.857551	5	Singlet	Immune_cells
VAT_2M	1846.5	1285	1.5216491	3	Singlet	FAPs
VAT_2M	3974.6	2161	0.2067551	0	Singlet	Adipocyte
VAT_2M	3974.3	2012	1.1209206	0	Singlet	Adipocyte
VAT_2M	1078.1	843	0.3762298	8	Singlet	Mesothelial
VAT_2M	2248.7	1454	1.2066884	0	Singlet	Adipocyte
VAT_2M	1713.7	1140	0.6954798	6	Singlet	Neurons
VAT_2M	4217.2	2080	0.0279197	0	Singlet	Adipocyte
VAT_2M	3678	1825	1.1020961	0	Singlet	Adipocyte
VAT_2M	2132.1	1453	1.3787128	5	Singlet	Immune_cells
VAT_2M	6538	2635	0.2583416	0	Singlet	Adipocyte
VAT_2M	2103.9	1461	0.3457274	3	Singlet	FAPs
VAT_2M	1338	972	0.9984802	2	Singlet	FAPs
VAT_2M	1107.3	856	7.885215	6	Singlet	Neurons
VAT_2M	2452.7	1757	1.3489755	3	Singlet	FAPs
VAT_2M	2955.7	1906	1.6600538	2	Singlet	FAPs
VAT_2M	3036.8	1659	0.0663785	0	Singlet	Adipocyte
VAT_2M	1913.4	1169	0.1830004	0	Singlet	Adipocyte
VAT_2M	2094.4	1350	0.3875759	3	Singlet	FAPs
VAT_2M	6089.7	2698	0.021435	0	Singlet	Adipocyte
VAT_2M	1391.3	1063	2.2963257	2	Singlet	FAPs
VAT_2M	1186.9	933	1.4790552	5	Singlet	Immune_cells
VAT_2M	3477	2071	1.1085442	5	Singlet	Immune_cells
VAT_2M	1679.1	1155	5.1896576	9	Singlet	Mesothelial
VAT_2M	1689.6	994	2.171261	0	Singlet	Adipocyte
VAT_2M	2015.8	1301	0.0477354	0	Singlet	Adipocyte
VAT_2M	4752.1	2182	0.1421104	0	Singlet	Adipocyte
VAT_2M	1201.6	877	0.4908171	5	Singlet	Immune_cells

VAT_2M	2685.3	1578	0.0260038	0	Singlet	Adipocyte
VAT_2M	1190.9	810	0.5669001	3	Singlet	FAPs
VAT_2M	1156.9	920	0.060342	5	Singlet	Immune_cells
VAT_2M	3146.9	1782	0.5041268	0	Singlet	Adipocyte
VAT_2M	6032.4	2436	0.023109	0	Singlet	Adipocyte
VAT_2M	1660.4	1094	0.580772	0	Singlet	Adipocyte
VAT_2M	9006.3	3218	0.0407544	0	Singlet	Adipocyte
VAT_2M	1078.8	772	1.8496511	7	Singlet	Endothelial_cells
VAT_2M	1184.3	981	0.3194078	6	Singlet	Neurons
VAT_2M	4085.8	2349	1.0279077	5	Singlet	Immune_cells
VAT_2M	1549	1035	0.9672795	0	Singlet	Adipocyte
VAT_2M	3607.7	1860	0.8925443	0	Singlet	Adipocyte
VAT_2M	3456.9	1931	0.2798125	0	Singlet	Adipocyte
VAT_2M	1792	989	0.3357446	0	Singlet	Adipocyte
VAT_2M	3910.3	2091	1.6114477	2	Singlet	FAPs
VAT_2M	4705.7	2249	0.2426772	0	Singlet	Adipocyte
VAT_2M	2768.7	1519	0	11	Singlet	Smooth_muscle_cells
VAT_2M	1421.7	924	5.2020656	5	Singlet	Immune_cells
VAT_2M	1129.6	846	3.1594705	2	Singlet	FAPs
VAT_2M	1655.6	1133	1.1298836	3	Singlet	FAPs
VAT_2M	5546.2	2400	0.1577405	0	Singlet	Adipocyte
VAT_2M	2519.4	1433	2.7033531	3	Singlet	FAPs
VAT_2M	2288.6	1344	0.2240994	2	Singlet	FAPs
VAT_2M	2070.8	1233	0.2144972	3	Singlet	FAPs
VAT_2M	2699.8	1450	0.078878	0	Singlet	Adipocyte
VAT_2M	1910	1337	0.0253931	7	Singlet	Endothelial_cells
VAT_2M	5305.1	2305	0.0157457	0	Singlet	Adipocyte
VAT_2M	3184.3	1687	0.0845533	0	Singlet	Adipocyte
VAT_2M	2909	1702	1.7591565	7	Singlet	Endothelial_cells
VAT_2M	7250.1	2871	0.0725174	0	Singlet	Adipocyte
VAT_2M	2285.9	1380	0.1567015	0	Singlet	Adipocyte
VAT_2M	5231.2	2389	0.6409818	0	Singlet	Adipocyte
VAT_2M	6634.3	2445	0.0069527	0	Singlet	Adipocyte
VAT_2M	12471	3857	0.0192177	0	Singlet	Adipocyte
VAT_2M	3233	1871	6.1286217	6	Singlet	Neurons
VAT_2M	2910.6	1616	2.2841078	5	Singlet	Immune_cells
VAT_2M	2257.4	1544	0.5080028	2	Singlet	FAPs
VAT_2M	1505.7	1052	2.5281424	7	Singlet	Endothelial_cells
VAT_2M	5408.3	2417	0.0434304	0	Singlet	Adipocyte
VAT_2M	7414.4	2971	0.0270322	0	Singlet	Adipocyte
VAT_2M	1034.2	846	0.4485092	7	Singlet	Endothelial_cells
VAT_2M	1308	918	0.1504757	0	Singlet	Adipocyte
VAT_2M	2157.7	1719	4.0479769	9	Singlet	Mesothelial
VAT_2M	2919	1646	0.024408	0	Singlet	Adipocyte
VAT_2M	1644.1	1131	2.0493025	5	Singlet	Immune_cells
VAT_2M	1998.8	1327	5.596333	0	Singlet	Adipocyte
VAT_2M	2082.2	1193	1.7033693	5	Singlet	Immune_cells
VAT_2M	4640.2	2184	0.0773197	0	Singlet	Adipocyte
VAT_2M	6450.3	2693	0.1662839	0	Singlet	Adipocyte
VAT_2M	10267	3552	0.0089037	0	Singlet	Adipocyte
VAT_2M	2879.8	1438	0.1470831	0	Singlet	Adipocyte

VAT_2M	4699.8	2547	1.6108089	3	Singlet	FAPs
VAT_2M	3933.2	1954	0.373185	0	Singlet	Adipocyte
VAT_2M	1505.2	1056	0.4889178	5	Singlet	Immune_cells
VAT_2M	3439.8	1847	2.1515461	2	Singlet	FAPs
VAT_2M	4746.6	2219	0.0102037	0	Singlet	Adipocyte
VAT_2M	8607.1	3023	0.0006017	0	Singlet	Adipocyte
VAT_2M	8434.1	3228	0	0	Singlet	Adipocyte
VAT_2M	1401.6	1070	0.5088575	0	Singlet	Adipocyte
VAT_2M	1097.7	902	0.9120707	5	Singlet	Immune_cells
VAT_2M	1277.3	981	1.1232419	2	Singlet	FAPs
VAT_2M	8735.2	3390	0.0224366	0	Singlet	Adipocyte
VAT_2M	2706	1697	0.2388525	2	Singlet	FAPs
VAT_2M	4764.8	2268	0.2918743	0	Singlet	Adipocyte
VAT_2M	6860.2	2802	0	0	Singlet	Adipocyte
VAT_2M	1965.3	1310	0.2786785	2	Singlet	FAPs
VAT_2M	8329.8	3137	0.0159271	0	Singlet	Adipocyte
VAT_2M	2246.4	1116	0.2535197	0	Singlet	Adipocyte
VAT_2M	2563.5	1354	0.3031116	0	Singlet	Adipocyte
VAT_2M	1443.5	1014	4.264038	2	Singlet	FAPs
VAT_2M	1361.8	1025	3.6628781	3	Singlet	FAPs
VAT_2M	5758.3	2635	0.022888	0	Singlet	Adipocyte
VAT_2M	4780.1	2202	0.0115878	0	Singlet	Adipocyte
VAT_2M	1223	854	3.2111741	3	Singlet	FAPs
VAT_2M	6085.2	2651	0.0977761	0	Singlet	Adipocyte
VAT_2M	2652	1479	0.3457351	0	Singlet	Adipocyte
VAT_2M	8938.4	3307	0.3497735	0	Singlet	Adipocyte
VAT_2M	1708.8	1202	0.2310714	7	Singlet	Endothelial_cells
VAT_2M	1640.5	1125	1.2015233	5	Singlet	Immune_cells
VAT_2M	8545.3	3103	0.0759784	0	Singlet	Adipocyte
VAT_2M	1953	1284	0.925203	5	Singlet	Immune_cells
VAT_2M	8989.9	3088	0	0	Singlet	Adipocyte
VAT_2M	1520.9	1120	0.5812929	2	Singlet	FAPs
VAT_2M	2488.2	1616	0.6019234	2	Singlet	FAPs
VAT_2M	2232.4	1521	2.7831221	5	Singlet	Immune_cells
VAT_2M	3495.8	2018	0.8859295	0	Singlet	Adipocyte
VAT_2M	1683.9	1170	5.001445	9	Singlet	Mesothelial
VAT_2M	1429.9	976	1.0151025	0	Singlet	Adipocyte
VAT_2M	1813.9	1256	0.8319155	2	Singlet	FAPs
VAT_2M	4343.7	2039	0.0888521	0	Singlet	Adipocyte
VAT_2M	2625.6	1417	0.2163642	0	Singlet	Adipocyte
VAT_2M	1366.5	1091	0.8513003	5	Singlet	Immune_cells
VAT_2M	6434.4	2729	0.0259617	0	Singlet	Adipocyte
VAT_2M	1454.3	1254	1.114117	6	Singlet	Neurons
VAT_2M	1022.3	834	1.5588349	7	Singlet	Endothelial_cells
VAT_2M	3445.2	1764	0.0201531	0	Singlet	Adipocyte
VAT_2M	2684.3	1759	2.0543129	2	Singlet	FAPs
VAT_2M	3226.8	1697	0.3769598	0	Singlet	Adipocyte
VAT_2M	3551	1806	0.03476	0	Singlet	Adipocyte
VAT_2M	8066	3131	1.2162938	0	Singlet	Adipocyte
VAT_2M	8634.9	3004	0.1039984	0	Singlet	Adipocyte
VAT_2M	1761.8	1193	0.1662478	3	Singlet	FAPs

VAT_2M	6148.6	2464	0.0189678	0	Singlet	Adipocyte
VAT_2M	7652.5	2747	0.0035636	0	Singlet	Adipocyte
VAT_2M	1140.2	841	0.1482927	7	Singlet	Endothelial_cells
VAT_2M	7724.2	2993	0.0690271	0	Singlet	Adipocyte
VAT_2M	1072.8	842	0.2893105	7	Singlet	Endothelial_cells
VAT_2M	3227.8	2047	1.5153419	5	Singlet	Immune_cells
VAT_2M	2120.1	1297	1.144374	0	Singlet	Adipocyte
VAT_2M	1501.7	1023	1.5336003	0	Singlet	Adipocyte
VAT_2M	4041.8	2014	0.036236	0	Singlet	Adipocyte
VAT_2M	2260.6	1275	0.1289887	3	Singlet	FAPs
VAT_2M	3573.5	2125	0.083301	0	Singlet	Adipocyte
VAT_2M	1108.8	791	0.0778365	7	Singlet	Endothelial_cells
VAT_2M	4874.4	2261	0	0	Singlet	Adipocyte
VAT_2M	1088.8	924	0.9786857	6	Singlet	Neurons
VAT_2M	1465.1	1198	1.1467919	7	Singlet	Endothelial_cells
VAT_2M	1194.9	886	0.8292048	2	Singlet	FAPs
VAT_2M	9564.5	3403	0.0030664	0	Singlet	Adipocyte
VAT_2M	1218	974	2.440836	4	Singlet	Immune_cells
VAT_2M	1170.6	972	1.5053425	3	Singlet	FAPs
VAT_2M	5232.6	2418	0.0270697	0	Singlet	Adipocyte
VAT_2M	8742.3	3021	0.0160332	0	Singlet	Adipocyte
VAT_2M	1717.9	1340	0.2301724	8	Singlet	Mesothelial
VAT_2M	1693.1	1158	0.5698166	2	Singlet	FAPs
VAT_2M	3079.9	1703	0	0	Singlet	Adipocyte
VAT_2M	8367.4	3061	0.1903541	0	Singlet	Adipocyte
VAT_2M	5451.8	2420	0.325849	0	Singlet	Adipocyte
VAT_2M	4645	2308	0.1760422	0	Singlet	Adipocyte
VAT_2M	1726.7	1135	0.2839017	0	Singlet	Adipocyte
VAT_2M	3396.9	1816	0.0005658	3	Singlet	FAPs
VAT_2M	1392.7	1104	0.4710075	3	Singlet	FAPs
VAT_2M	1111.3	889	2.3041115	4	Singlet	Immune_cells
VAT_2M	1653.1	1080	0.1110922	6	Singlet	Neurons
VAT_2M	3036.8	1798	2.1089225	0	Singlet	Adipocyte
VAT_2M	1610.3	1185	1.780915	2	Singlet	FAPs
VAT_2M	5436.4	2404	0	0	Singlet	Adipocyte
VAT_2M	5628	2408	0.0303458	0	Singlet	Adipocyte
VAT_2M	2392.2	1449	1.5747337	0	Singlet	Adipocyte
VAT_2M	6504.4	2584	0.0477849	0	Singlet	Adipocyte
VAT_2M	7727.4	3098	0.0490644	0	Singlet	Adipocyte
VAT_2M	3022.8	1757	0.1020003	0	Singlet	Adipocyte
VAT_2M	1386.8	1028	2.3735503	3	Singlet	FAPs
VAT_2M	3356.3	1833	0.4578503	0	Singlet	Adipocyte
VAT_2M	5815.5	2697	0.0168415	0	Singlet	Adipocyte
VAT_2M	8525.8	3236	0.0401683	0	Singlet	Adipocyte
VAT_2M	2782.4	1631	0	0	Singlet	Adipocyte
VAT_2M	5043.9	2135	0.058896	0	Singlet	Adipocyte
VAT_2M	3440.3	1800	0.1289382	0	Singlet	Adipocyte
VAT_2M	1100.7	807	0.8112406	7	Singlet	Endothelial_cells
VAT_2M	7111.8	2744	0.0121995	0	Singlet	Adipocyte
VAT_2M	4218	2003	0.2477973	0	Singlet	Adipocyte
VAT_2M	3904.6	2014	1.7548402	0	Singlet	Adipocyte

VAT_2M	1346.9	920	2.2448156	2	Singlet	FAPs
VAT_2M	1147.9	914	3.1215572	4	Singlet	Immune_cells
VAT_2M	3811.1	1867	0.1815144	0	Singlet	Adipocyte
VAT_2M	2261.1	1671	0.5665485	5	Singlet	Immune_cells
VAT_2M	1156.9	923	1.6986135	8	Singlet	Mesothelial
VAT_2M	7976.2	3004	0.0021355	0	Singlet	Adipocyte
VAT_2M	2268.6	1411	1.9488025	2	Singlet	FAPs
VAT_2M	5446	2487	0.2367526	0	Singlet	Adipocyte
VAT_2M	6704	2708	0.0144967	0	Singlet	Adipocyte
VAT_2M	6031.5	2834	1.7217122	3	Singlet	FAPs
VAT_2M	4209.4	2137	0.1759764	0	Singlet	Adipocyte
VAT_2M	6704.9	2633	0.0496419	0	Singlet	Adipocyte
VAT_2M	5491.9	2471	0.077219	0	Singlet	Adipocyte
VAT_2M	2017.3	1129	2.8975815	2	Singlet	FAPs
VAT_2M	1870.7	1287	0.5473399	2	Singlet	FAPs
VAT_2M	4672.1	2143	0.2669123	0	Singlet	Adipocyte
VAT_2M	2381.1	1674	1.0813965	3	Singlet	FAPs
VAT_2M	5470.8	2432	0.0136857	0	Singlet	Adipocyte
VAT_2M	2735.8	1622	1.4679039	2	Singlet	FAPs
VAT_2M	3584.2	1897	0.1837676	0	Singlet	Adipocyte
VAT_2M	7156.8	3012	0.0167322	0	Singlet	Adipocyte
VAT_2M	5161.9	2168	0.0144868	0	Singlet	Adipocyte
VAT_2M	1876.6	1207	2.7993669	0	Singlet	Adipocyte
VAT_2M	1169.1	941	2.3601476	2	Singlet	FAPs
VAT_2M	4539.7	2080	0.0867377	0	Singlet	Adipocyte
VAT_2M	1199	1033	0.5639101	6	Singlet	Neurons
VAT_2M	1306.5	1080	1.2568366	2	Singlet	FAPs
VAT_2M	1593.4	1057	3.3094012	8	Singlet	Mesothelial
VAT_2M	3089.7	1932	0.4913754	2	Singlet	FAPs
VAT_2M	6537.8	2746	0.1498262	0	Singlet	Adipocyte
VAT_2M	8237.9	3006	0.0011412	0	Singlet	Adipocyte
VAT_2M	7203.8	2939	0.0230161	0	Singlet	Adipocyte
VAT_2M	1844.8	1289	0.4109698	6	Singlet	Neurons
VAT_2M	1940.1	1276	0.1160926	0	Singlet	Adipocyte
VAT_2M	4306.8	2206	0.0338344	0	Singlet	Adipocyte
VAT_2M	4528.6	2067	0.2846083	0	Singlet	Adipocyte
VAT_2M	1144	940	2.792275	6	Singlet	Neurons
VAT_2M	9651.6	3314	0	0	Singlet	Adipocyte
VAT_2M	5569.7	2297	0.013571	0	Singlet	Adipocyte
VAT_2M	1112.2	929	0.804452	6	Singlet	Neurons
VAT_2M	1768.8	1205	3.167024	5	Singlet	Immune_cells
VAT_2M	1165.8	785	1.6171042	2	Singlet	FAPs
VAT_2M	2439.9	1379	0.7177335	0	Singlet	Adipocyte
VAT_2M	2391.7	1443	1.6805595	5	Singlet	Immune_cells
VAT_2M	1133.3	933	0.7017417	7	Singlet	Endothelial_cells
VAT_2M	3029.9	1855	1.5794516	2	Singlet	FAPs
VAT_2M	2261.1	1393	0.1253771	0	Singlet	Adipocyte
VAT_2M	2661.2	1586	0.1247203	3	Singlet	FAPs
VAT_2M	9313.7	3174	0.225919	0	Singlet	Adipocyte
VAT_2M	7004	2740	0.002956	0	Singlet	Adipocyte
VAT_2M	1713	1219	0.122445	2	Singlet	FAPs

VAT_2M	1029.2	903	0.1712469	9	Singlet	Mesothelial
VAT_2M	1158.7	978	0.5407245	9	Singlet	Mesothelial
VAT_2M	3251.1	1727	0.1482663	0	Singlet	Adipocyte
VAT_2M	9790	3447	0.0328874	0	Singlet	Adipocyte
VAT_2M	2102.3	1394	0.8073855	6	Singlet	Neurons
VAT_2M	2843	1607	0.0240307	0	Singlet	Adipocyte
VAT_2M	1344.8	1037	0.914943	2	Singlet	FAPs
VAT_2M	2246	1406	0.8771926	2	Singlet	FAPs
VAT_2M	5371.5	2557	0.0673898	0	Singlet	Adipocyte
VAT_2M	8893	3177	0.2459966	0	Singlet	Adipocyte
VAT_2M	4053.5	2029	0.2045049	0	Singlet	Adipocyte
VAT_2M	1066.4	882	0.3785465	6	Singlet	Neurons
VAT_2M	1634.5	1192	0.5473646	2	Singlet	FAPs
VAT_2M	1647.7	1148	0.4898515	0	Singlet	Adipocyte
VAT_2M	1373.2	1040	1.1239937	3	Singlet	FAPs
VAT_2M	3666.9	1849	0.2872348	0	Singlet	Adipocyte
VAT_2M	3586.7	2047	2.2558556	2	Singlet	FAPs
VAT_2M	1133.2	859	3.1271503	3	Singlet	FAPs
VAT_2M	2991	1647	0.0199688	0	Singlet	Adipocyte
VAT_2M	2010.5	1368	0.5060319	6	Singlet	Neurons
VAT_2M	1708.2	1321	0.52924	2	Singlet	FAPs
VAT_2M	6928.2	2649	0.0695381	0	Singlet	Adipocyte
VAT_2M	1039.2	821	0.9742959	11	Singlet	Smooth_muscle_cells
VAT_2M	6342.8	2660	0.200039	0	Singlet	Adipocyte
VAT_2M	1685.1	1163	1.271637	2	Singlet	FAPs
VAT_2M	6341.7	2613	0.0836156	0	Singlet	Adipocyte
VAT_2M	5821.3	2406	0.198196	0	Singlet	Adipocyte
VAT_2M	2636.8	1514	0.1040573	2	Singlet	FAPs
VAT_2M	1326	1097	2.4438555	6	Singlet	Neurons
VAT_2M	3345	1846	0.2195685	0	Singlet	Adipocyte
VAT_2M	3107.8	1671	0.0223332	0	Singlet	Adipocyte
VAT_2M	1889	1235	0.9182734	0	Singlet	Adipocyte
VAT_2M	2687.4	1540	0	0	Singlet	Adipocyte
VAT_2M	5897.2	2520	0.0619103	0	Singlet	Adipocyte
VAT_2M	1867.1	1143	3.5667468	0	Singlet	Adipocyte
VAT_2M	7326.3	2878	0.0035909	0	Singlet	Adipocyte
VAT_2M	8623.1	3211	0.0785282	0	Singlet	Adipocyte
VAT_2M	1174.9	915	3.775832	5	Singlet	Immune_cells
VAT_2M	1627.8	1029	3.1050755	0	Singlet	Adipocyte
VAT_2M	1405.7	977	1.7814596	2	Singlet	FAPs
VAT_2M	7224.6	2775	0.0214918	0	Singlet	Adipocyte
VAT_2M	2428.2	1512	0.3341939	2	Singlet	FAPs
VAT_2M	4729	2244	0.101726	0	Singlet	Adipocyte
VAT_2M	1731.1	1151	4.8002064	2	Singlet	FAPs
VAT_2M	6443.5	2645	0.0041699	0	Singlet	Adipocyte
VAT_2M	1208.6	921	1.7882708	11	Singlet	Smooth_muscle_cells
VAT_2M	2895.6	1726	0.0706782	0	Singlet	Adipocyte
VAT_2M	2082.5	1398	0.1218175	0	Singlet	Adipocyte
VAT_2M	5660.9	2429	0.0064357	0	Singlet	Adipocyte
VAT_2M	1961.2	1175	0.2082365	0	Singlet	Adipocyte
VAT_2M	5553	2475	0.0224206	0	Singlet	Adipocyte

VAT_2M	1046.1	847	0.7763495	2	Singlet	FAPs
VAT_2M	4701.7	2173	0.195257	0	Singlet	Adipocyte
VAT_2M	4179.7	2096	0	0	Singlet	Adipocyte
VAT_2M	7210.6	3004	0.0830365	0	Singlet	Adipocyte
VAT_2M	1222.8	1032	1.8453668	4	Singlet	Immune_cells
VAT_2M	2262.8	1477	1.0316774	6	Singlet	Neurons
VAT_2M	1210.9	927	0.3236489	3	Singlet	FAPs
VAT_2M	1208	923	1.7618833	6	Singlet	Neurons
VAT_2M	1736.9	1133	0.1356205	0	Singlet	Adipocyte
VAT_2M	2215.5	1344	4.8589514	0	Singlet	Adipocyte
VAT_2M	6244	2604	0.0071114	0	Singlet	Adipocyte
VAT_2M	11478	3625	0.0125881	0	Singlet	Adipocyte
VAT_2M	6176.3	2749	0.0770105	0	Singlet	Adipocyte
VAT_2M	1487.2	1024	2.015517	3	Singlet	FAPs
VAT_2M	1269.1	1089	0.4846813	1	Singlet	Immune_cells
VAT_2M	9088.1	3087	0.0102696	0	Singlet	Adipocyte
VAT_2M	3099.1	1797	1.9449778	5	Singlet	Immune_cells
VAT_2M	1955.1	1308	0.2382994	5	Singlet	Immune_cells
VAT_2M	2461.9	1487	0.761193	0	Singlet	Adipocyte
VAT_2M	9886.6	3239	0	0	Singlet	Adipocyte
VAT_2M	2333.8	1511	2.6621945	6	Singlet	Neurons
VAT_2M	1589.3	1362	1.4767936	6	Singlet	Neurons
VAT_2M	7689.6	2746	0.0384119	0	Singlet	Adipocyte
VAT_2M	3339	1835	0.3069883	0	Singlet	Adipocyte
VAT_2M	1084.5	836	1.1219558	3	Singlet	FAPs
VAT_2M	1944.8	1361	1.9104543	6	Singlet	Neurons
VAT_2M	1827.1	1133	2.888782	0	Singlet	Adipocyte
VAT_2M	2991.9	1607	0.1768166	0	Singlet	Adipocyte
VAT_2M	4282.8	2169	0.2380832	0	Singlet	Adipocyte
VAT_2M	3047.8	1762	0.6205113	3	Singlet	FAPs
VAT_2M	4176.5	2212	0.5089462	0	Singlet	Adipocyte
VAT_2M	1584.5	833	12.026516	0	Singlet	Adipocyte
VAT_2M	3232.6	1973	3.9446033	2	Singlet	FAPs
VAT_2M	6895.4	2858	0.0738115	0	Singlet	Adipocyte
VAT_2M	2391	1484	0.1363691	0	Singlet	Adipocyte
VAT_2M	1894.7	1213	1.1580667	2	Singlet	FAPs
VAT_2M	1949.4	1160	0.4587156	0	Singlet	Adipocyte
VAT_2M	1162.4	901	0.4986692	2	Singlet	FAPs
VAT_2M	1111	832	2.5125902	5	Singlet	Immune_cells
VAT_2M	1036.6	807	0.2147512	3	Singlet	FAPs
VAT_2M	5982.1	2643	0.108991	0	Singlet	Adipocyte
VAT_2M	1616.4	1098	0.2224959	6	Singlet	Neurons
VAT_2M	1272.1	1067	0.898201	6	Singlet	Neurons
VAT_2M	1153	918	0.6017111	4	Singlet	Immune_cells
VAT_2M	4481.9	2162	0.0476243	0	Singlet	Adipocyte
VAT_2M	5133	2254	0.0079528	0	Singlet	Adipocyte
VAT_2M	2929.9	1616	0.2056381	0	Singlet	Adipocyte
VAT_2M	4018.9	2023	0.1039346	0	Singlet	Adipocyte
VAT_2M	2506.4	1647	2.2113382	3	Singlet	FAPs
VAT_2M	5518.4	2428	0.0322782	0	Singlet	Adipocyte
VAT_2M	9467.5	3289	0.1328462	0	Singlet	Adipocyte

VAT_2M	1184.1	905	0.7571463	2 Singlet	FAPs
VAT_2M	3726.3	1893	0.5530641	0 Singlet	Adipocyte
VAT_2M	1097.2	876	4.2801122	2 Singlet	FAPs
VAT_2M	2765.9	1549	1.4886301	0 Singlet	Adipocyte
VAT_2M	1449.7	1221	1.2165089	8 Singlet	Mesothelial
VAT_2M	9367.8	3118	0.0719459	0 Singlet	Adipocyte
VAT_2M	2933.3	1697	2.2158936	2 Singlet	FAPs
VAT_2M	4588.7	2085	0.0133497	0 Singlet	Adipocyte
VAT_2M	2015.4	1253	0.321026	5 Singlet	Immune_cells
VAT_2M	8332.1	3095	0.0229496	0 Singlet	Adipocyte
VAT_2M	2135.9	1370	0	0 Singlet	Adipocyte
VAT_2M	1460.7	1141	0.4898556	3 Singlet	FAPs
VAT_2M	1407.5	1031	1.9235164	2 Singlet	FAPs
VAT_2M	2202.8	1345	0.588948	3 Singlet	FAPs
VAT_2M	2046.5	1243	1.9897287	5 Singlet	Immune_cells
VAT_2M	1115	887	1.6843329	2 Singlet	FAPs
VAT_2M	3064.5	1620	4.7233668	5 Singlet	Immune_cells
VAT_2M	1915.2	1418	0.9804609	5 Singlet	Immune_cells
VAT_2M	1186.8	1013	0.8214729	6 Singlet	Neurons
VAT_2M	1747.3	1088	1.3869365	3 Singlet	FAPs
VAT_2M	6444.3	2700	0.2076022	0 Singlet	Adipocyte
VAT_2M	1654.9	1232	0.8535466	6 Singlet	Neurons
VAT_2M	4697.3	2134	0.0641074	0 Singlet	Adipocyte
VAT_2M	1843.1	1107	4.5008238	4 Singlet	Immune_cells
VAT_2M	1190.1	938	0.8265788	3 Singlet	FAPs
VAT_2M	2757	1416	1.5626497	0 Singlet	Adipocyte
VAT_2M	9748.8	3286	0	0 Singlet	Adipocyte
VAT_2M	1608.7	1059	0.7397773	5 Singlet	Immune_cells
VAT_2M	3908.2	2016	0.0389771	0 Singlet	Adipocyte
VAT_2M	2297.6	1472	0.2333783	0 Singlet	Adipocyte
VAT_2M	4717	2253	0.0414166	0 Singlet	Adipocyte
VAT_2M	2637	1469	1.2896901	3 Singlet	FAPs
VAT_2M	6457.8	2662	0.1177878	0 Singlet	Adipocyte
VAT_2M	4850.3	2291	0.0069339	0 Singlet	Adipocyte
VAT_2M	3177.4	1903	0.0377626	0 Singlet	Adipocyte
VAT_2M	1476.9	1029	0.1562852	3 Singlet	FAPs
VAT_2M	1615.4	965	12.7956	0 Singlet	Adipocyte
VAT_2M	1176.3	868	1.4661304	6 Singlet	Neurons
VAT_2M	1702.9	1132	0.4070064	2 Singlet	FAPs
VAT_2M	2598.4	1547	0	0 Singlet	Adipocyte
VAT_2M	2182.2	1430	1.0454744	2 Singlet	FAPs
VAT_2M	1610.9	1051	0.3121155	0 Singlet	Adipocyte
VAT_2M	4522	2087	0.00214	0 Singlet	Adipocyte
VAT_2M	1041.2	838	1.3460646	6 Singlet	Neurons
VAT_2M	11740	3664	0.000676	0 Singlet	Adipocyte
VAT_2M	4365.5	2032	0.1688036	0 Singlet	Adipocyte
VAT_2M	4454.5	2116	0.2221918	0 Singlet	Adipocyte
VAT_2M	2125.4	1409	1.2979295	5 Singlet	Immune_cells
VAT_2M	2042.6	1363	0.0919596	2 Singlet	FAPs
VAT_2M	1023.8	834	0.9914689	6 Singlet	Neurons
VAT_2M	1943.3	1232	0.3790143	0 Singlet	Adipocyte

VAT_2M	4310.2	2384	0.3875373	3	Singlet	FAPs
VAT_2M	1322.3	1038	1.1570051	6	Singlet	Neurons
VAT_2M	5719.6	2413	0	0	Singlet	Adipocyte
VAT_2M	1645.4	1286	3.0207134	10	Singlet	Immune_cells
VAT_2M	3278.5	1840	0.0554164	0	Singlet	Adipocyte
VAT_2M	1719.8	1115	1.7101733	6	Singlet	Neurons
VAT_2M	6732.7	2672	0.0324836	0	Singlet	Adipocyte
VAT_2M	7038.5	2844	0.8252235	0	Singlet	Adipocyte
VAT_2M	1193.5	1003	1.3063512	6	Singlet	Neurons
VAT_2M	1649.5	1102	2.0508446	11	Singlet	Smooth_muscle_cells
VAT_2M	1669.8	1209	1.8872095	3	Singlet	FAPs
VAT_2M	6860.6	2801	0.2381408	0	Singlet	Adipocyte
VAT_2M	1148.3	846	1.2268102	2	Singlet	FAPs
VAT_2M	1737.9	1124	1.5452633	7	Singlet	Endothelial_cells
VAT_2M	1249.3	1036	1.0979864	6	Singlet	Neurons
VAT_2M	7138.7	2981	0.3734467	0	Singlet	Adipocyte
VAT_2M	5887.9	2455	0.9344361	0	Singlet	Adipocyte
VAT_2M	1567.7	1048	3.0912597	3	Singlet	FAPs
VAT_2M	1162.1	866	0.1336762	2	Singlet	FAPs
VAT_2M	2236	1355	0.1970747	0	Singlet	Adipocyte
VAT_2M	3483.3	1894	0.5479889	0	Singlet	Adipocyte
VAT_2M	1613.9	1008	1.5559976	2	Singlet	FAPs
VAT_2M	1228.1	920	1.5502948	1	Singlet	Immune_cells
VAT_2M	1687.6	1173	0.0498686	3	Singlet	FAPs
VAT_2M	1534.7	1214	1.2971498	3	Singlet	FAPs
VAT_2M	7542.9	2851	0.0258947	0	Singlet	Adipocyte
VAT_2M	9646.9	3202	0.0199008	0	Singlet	Adipocyte
VAT_2M	1511.4	1095	0.1612618	5	Singlet	Immune_cells
VAT_2M	2589.6	1380	13.53442	7	Singlet	Endothelial_cells
VAT_2M	2475.3	1501	0.0514823	6	Singlet	Neurons
VAT_2M	3390.6	1827	0	0	Singlet	Adipocyte
VAT_2M	2193	1301	0.177499	0	Singlet	Adipocyte
VAT_2M	3657.4	1950	0.0980528	0	Singlet	Adipocyte
VAT_2M	5256.7	2643	0.1822434	2	Singlet	FAPs
VAT_2M	1167.9	897	10.650025	8	Singlet	Mesothelial
VAT_2M	5365.2	2274	0.0308541	0	Singlet	Adipocyte
VAT_2M	2892.5	1681	0.0470697	0	Singlet	Adipocyte
VAT_2M	1953.8	1273	0.0765844	3	Singlet	FAPs
VAT_2M	4225.3	1937	0.1803147	0	Singlet	Adipocyte
VAT_2M	7974	3000	0.0274528	0	Singlet	Adipocyte
VAT_2M	1798.2	1220	0.1086452	2	Singlet	FAPs
VAT_2M	3124.5	1675	0.0411227	0	Singlet	Adipocyte
VAT_2M	1418.1	1003	3.7647541	7	Singlet	Endothelial_cells
VAT_2M	1961.3	1434	0.7501034	2	Singlet	FAPs
VAT_2M	3499.8	1826	0.5292305	0	Singlet	Adipocyte
VAT_2M	1733.3	1202	0.1774697	5	Singlet	Immune_cells
VAT_2M	1459.2	989	0.1160146	6	Singlet	Neurons
VAT_2M	1560.7	1180	0.4450502	11	Singlet	Smooth_muscle_cells
VAT_2M	2670	1600	2.128801	5	Singlet	Immune_cells
VAT_2M	1870	1287	0.2950621	0	Singlet	Adipocyte
VAT_2M	6191.3	2669	0.0125412	0	Singlet	Adipocyte

VAT_2M	1282.6	971	0.5979665	6 Singlet	Neurons
VAT_2M	1086.4	822	2.8113803	3 Singlet	FAPs
VAT_2M	4461.5	2167	0.0148132	0 Singlet	Adipocyte
VAT_2M	7141.5	2708	2.3942999	0 Singlet	Adipocyte
VAT_2M	1639.5	1026	0.3344388	0 Singlet	Adipocyte
VAT_2M	6160.5	2455	0.3070619	0 Singlet	Adipocyte
VAT_2M	9382.6	3329	0.0160051	0 Singlet	Adipocyte
VAT_2M	6149.5	2542	0.009287	0 Singlet	Adipocyte
VAT_2M	1673.7	1188	0.2892474	3 Singlet	FAPs
VAT_2M	1659.8	1239	0.3570238	3 Singlet	FAPs
VAT_2M	3482.4	2009	0.8020308	3 Singlet	FAPs
VAT_2M	1412.9	1004	0.0731796	3 Singlet	FAPs
VAT_2M	6283.8	2622	0.1435697	0 Singlet	Adipocyte
VAT_2M	1625.6	1163	0.9067781	2 Singlet	FAPs
VAT_2M	1257.8	864	1.2467213	6 Singlet	Neurons
VAT_2M	2872.6	1489	0.9086945	0 Singlet	Adipocyte
VAT_2M	10364	3397	0	0 Singlet	Adipocyte
VAT_2M	2712.1	1874	1.8843843	5 Singlet	Immune_cells
VAT_2M	1031.1	881	1.5333741	6 Singlet	Neurons
VAT_2M	3374.6	2110	1.1213269	2 Singlet	FAPs
VAT_2M	1076.6	790	0.2089694	5 Singlet	Immune_cells
VAT_2M	4714.7	2150	0.0392959	0 Singlet	Adipocyte
VAT_2M	4172.2	2103	0.4022685	0 Singlet	Adipocyte
VAT_2M	2620	1654	0.3964219	2 Singlet	FAPs
VAT_2M	3864.7	2070	0.2325753	0 Singlet	Adipocyte
VAT_2M	1630.8	1247	5.415138	2 Singlet	FAPs
VAT_2M	2363.4	1496	1.2057701	2 Singlet	FAPs
VAT_2M	2787.5	1625	1.6455856	5 Singlet	Immune_cells
VAT_2M	3261.1	1836	2.1547415	2 Singlet	FAPs
VAT_2M	4292	2051	0	0 Singlet	Adipocyte
VAT_2M	1006.6	759	2.1917682	5 Singlet	Immune_cells
VAT_2M	2728.9	1694	1.1250981	7 Singlet	Endothelial_cells
VAT_2M	3163.4	1632	0.1310056	0 Singlet	Adipocyte
VAT_2M	1853.4	1261	0.9254972	2 Singlet	FAPs
VAT_2M	4185.7	2000	0.0777535	0 Singlet	Adipocyte
VAT_2M	1425.1	993	2.6687202	5 Singlet	Immune_cells
VAT_2M	1067.9	823	1.4741572	2 Singlet	FAPs
VAT_2M	4643.8	2085	0.0737326	0 Singlet	Adipocyte
VAT_2M	1866.8	1398	0.804687	3 Singlet	FAPs
VAT_2M	4345.2	2235	2.1009071	3 Singlet	FAPs
VAT_2M	1046.7	789	0.1695782	4 Singlet	Immune_cells
VAT_2M	4529.6	2070	0.2407038	0 Singlet	Adipocyte
VAT_2M	1134.9	801	1.0849119	0 Singlet	Adipocyte
VAT_2M	4387.6	2158	0.0113379	0 Singlet	Adipocyte
VAT_2M	2769.1	1704	1.0452867	0 Singlet	Adipocyte
VAT_2M	2427.7	1409	0.3077346	0 Singlet	Adipocyte
VAT_2M	1033.8	884	1.5494498	6 Singlet	Neurons
VAT_2M	1858.2	1064	9.8585285	7 Singlet	Endothelial_cells
VAT_2M	1472.5	980	0.5024567	5 Singlet	Immune_cells
VAT_2M	2063.7	1334	0.5340094	2 Singlet	FAPs
VAT_2M	6851.6	2802	0.0434459	0 Singlet	Adipocyte

VAT_2M	1967.7	1423	2.2303469	6	Singlet	Neurons
VAT_2M	1321.8	971	4.8402122	3	Singlet	FAPs
VAT_2M	2221.4	1388	0	2	Singlet	FAPs
VAT_2M	4213.6	2414	0.0729693	2	Singlet	FAPs
VAT_2M	5298.7	2262	0	0	Singlet	Adipocyte
VAT_2M	6272.6	2634	0.0094313	0	Singlet	Adipocyte
VAT_2M	12436	3905	0.7854167	0	Singlet	Adipocyte
VAT_2M	5420.7	2329	0.3719981	0	Singlet	Adipocyte
VAT_2M	4256.8	2046	0.336782	0	Singlet	Adipocyte
VAT_2M	1913.7	1361	0.342426	6	Singlet	Neurons
VAT_2M	2260.8	1331	0.4285154	5	Singlet	Immune_cells
VAT_2M	1287.7	1010	0.2870695	2	Singlet	FAPs
VAT_2M	2761	1661	0.9845164	2	Singlet	FAPs
VAT_2M	1201.6	957	11.534298	4	Singlet	Immune_cells
VAT_2M	4701.8	2278	0.113858	0	Singlet	Adipocyte
VAT_2M	1311.1	943	1.6348484	5	Singlet	Immune_cells
VAT_2M	9181	3291	0.0012909	0	Singlet	Adipocyte
VAT_2M	4056.1	2019	0.0243642	0	Singlet	Adipocyte
VAT_2M	1465.8	1154	0.3976191	7	Singlet	Endothelial_cells
VAT_2M	6495.4	2579	0.0053623	0	Singlet	Adipocyte
VAT_2M	1168.5	932	0.564112	7	Singlet	Endothelial_cells
VAT_2M	4006.3	1905	0.0240544	0	Singlet	Adipocyte
VAT_2M	7785.3	2923	0.0592345	0	Singlet	Adipocyte
VAT_2M	8496.7	3193	0.106198	0	Singlet	Adipocyte
VAT_2M	1948.4	1427	0.1825987	2	Singlet	FAPs
VAT_2M	1217.8	987	1.1997394	6	Singlet	Neurons
VAT_2M	3027.7	1635	0.1667496	0	Singlet	Adipocyte
VAT_2M	2880.9	1503	1.625171	0	Singlet	Adipocyte
VAT_2M	1637.6	1031	4.2549168	3	Singlet	FAPs
VAT_2M	1729.7	1290	0.3360024	6	Singlet	Neurons
VAT_2M	6605.8	2750	0.0034128	0	Singlet	Adipocyte
VAT_2M	1147.1	867	1.4392458	1	Singlet	Immune_cells
VAT_2M	1098	880	1.175734	7	Singlet	Endothelial_cells
VAT_2M	1910.3	1268	2.2476038	6	Singlet	Neurons
VAT_2M	1712.5	1016	0.9227604	2	Singlet	FAPs
VAT_2M	2314.5	1428	2.4165532	2	Singlet	FAPs
VAT_2M	1384.1	1106	2.3507268	6	Singlet	Neurons
VAT_2M	2057.5	1297	0.1708898	2	Singlet	FAPs
VAT_2M	10462	3273	0.036524	0	Singlet	Adipocyte
VAT_2M	5121.8	2261	0.0427411	0	Singlet	Adipocyte
VAT_2M	1759.3	1181	0.9472882	0	Singlet	Adipocyte
VAT_2M	4714.3	2305	0.4431057	0	Singlet	Adipocyte
VAT_2M	3879.2	1884	0	0	Singlet	Adipocyte
VAT_2M	2652.8	1588	0.6010907	5	Singlet	Immune_cells
VAT_2M	2094.3	1224	0.4508296	7	Singlet	Endothelial_cells
VAT_2M	1585.2	1018	4.4600173	5	Singlet	Immune_cells
VAT_2M	5314	2550	0.1820065	0	Singlet	Adipocyte
VAT_2M	3507.7	1794	0.1216456	0	Singlet	Adipocyte
VAT_2M	3520.4	1878	3.20909	5	Singlet	Immune_cells
VAT_2M	7480.3	2721	0.0301591	0	Singlet	Adipocyte
VAT_2M	7093.1	2964	0.0050488	0	Singlet	Adipocyte

VAT_2M	1827.5	1236	0.1676163	0	Singlet	Adipocyte
VAT_2M	6012	2517	0.040464	0	Singlet	Adipocyte
VAT_2M	1730.2	1309	1.7635733	3	Singlet	FAPs
VAT_2M	2183.1	1309	0.455346	3	Singlet	FAPs
VAT_2M	5486.6	2694	0.0042028	0	Singlet	Adipocyte
VAT_2M	2293.7	1556	0.7599598	5	Singlet	Immune_cells
VAT_2M	1262.6	965	1.1780763	7	Singlet	Endothelial_cells
VAT_2M	4436.7	2352	0.0086094	0	Singlet	Adipocyte
VAT_2M	11959	3783	0.6558616	0	Singlet	Adipocyte
VAT_2M	6301.9	2615	0.0457013	0	Singlet	Adipocyte
VAT_2M	9425.7	3363	1.1951219	0	Singlet	Adipocyte
VAT_2M	11096	3562	0.0219849	0	Singlet	Adipocyte
VAT_2M	8836.7	3061	0.0087588	0	Singlet	Adipocyte
VAT_2M	1211.5	854	0.0242088	7	Singlet	Endothelial_cells
VAT_2M	6282.7	2814	0.0133721	0	Singlet	Adipocyte
VAT_2M	3940.1	2018	0.0761038	0	Singlet	Adipocyte
VAT_2M	1827.9	1220	1.4495406	2	Singlet	FAPs
VAT_2M	6874.3	2615	0.0300195	0	Singlet	Adipocyte
VAT_2M	1887.4	1185	2.2305574	0	Singlet	Adipocyte
VAT_2M	4400.6	2190	0.0749677	0	Singlet	Adipocyte
VAT_2M	3619.3	1874	0.2158869	0	Singlet	Adipocyte
VAT_2M	7703.2	2987	0.1936621	0	Singlet	Adipocyte
VAT_2M	2189.6	1312	1.5664175	0	Singlet	Adipocyte
VAT_2M	1994.9	986	1.9782016	7	Singlet	Endothelial_cells
VAT_2M	11147	3565	0	0	Singlet	Adipocyte
VAT_2M	9387.8	3290	2.3833601	0	Singlet	Adipocyte
VAT_2M	3519.1	1977	0.6278114	3	Singlet	FAPs
VAT_2M	2308.3	1430	0.3730843	0	Singlet	Adipocyte
VAT_2M	1552.9	1115	0.9142125	7	Singlet	Endothelial_cells
VAT_2M	1056.7	940	1.4509968	6	Singlet	Neurons
VAT_2M	1230.2	878	1.5892907	9	Singlet	Mesothelial
VAT_2M	1556.3	1146	0.3195372	7	Singlet	Endothelial_cells
VAT_2M	3968.8	1994	1.4004114	0	Singlet	Adipocyte
VAT_2M	7219.4	2669	3.5324718	0	Singlet	Adipocyte
VAT_2M	1145.4	867	2.4259156	3	Singlet	FAPs
VAT_2M	4066.2	2312	0.4751013	2	Singlet	FAPs
VAT_2M	7424.2	2998	0.0699162	0	Singlet	Adipocyte
VAT_2M	1057.7	820	1.6732602	6	Singlet	Neurons
VAT_2M	1949.6	1155	0.2677508	0	Singlet	Adipocyte
VAT_2M	1567.1	1188	3.0972398	2	Singlet	FAPs
VAT_2M	1499.8	1137	1.4314297	1	Singlet	Immune_cells
VAT_2M	1145.3	860	0.9675878	2	Singlet	FAPs
VAT_2M	2066.7	1285	1.2398744	0	Singlet	Adipocyte
VAT_2M	1779.9	1122	0.1601561	2	Singlet	FAPs
VAT_2M	2516.1	1614	0.9408629	3	Singlet	FAPs
VAT_2M	3103.6	1818	1.3815611	0	Singlet	Adipocyte
VAT_2M	4810.3	2295	0.0132823	0	Singlet	Adipocyte
VAT_2M	4765.6	2184	0.0752935	0	Singlet	Adipocyte
VAT_2M	5181.8	2236	0.0109014	0	Singlet	Adipocyte
VAT_2M	7100.5	2738	0.0246261	0	Singlet	Adipocyte
VAT_2M	6581.2	2638	1.211499	0	Singlet	Adipocyte

VAT_2M	1632.9	1002	0.0522674	0	Singlet	Adipocyte
VAT_2M	1432.2	1026	0.8515828	6	Singlet	Neurons
VAT_2M	2029.1	1399	0.4072187	5	Singlet	Immune_cells
VAT_2M	2852.7	1596	0.3886668	0	Singlet	Adipocyte
VAT_2M	6413.3	2637	0.6601679	0	Singlet	Adipocyte
VAT_2M	1723.5	1297	1.7592565	9	Singlet	Mesothelial
VAT_2M	2251.5	1530	1.3365529	2	Singlet	FAPs
VAT_2M	1658.5	1144	0.7207676	3	Singlet	FAPs
VAT_2M	2439.3	1347	0.3603507	0	Singlet	Adipocyte
VAT_2M	1346.4	953	3.4306259	2	Singlet	FAPs
VAT_2M	2277.9	1523	1.2290911	2	Singlet	FAPs
VAT_2M	2711	1441	0.8984674	0	Singlet	Adipocyte
VAT_2M	1013.9	757	1.1874099	2	Singlet	FAPs
VAT_2M	1313	976	0.6498989	0	Singlet	Adipocyte
VAT_2M	1456.4	1063	1.8563886	5	Singlet	Immune_cells
VAT_2M	4832.7	2249	0.0899811	0	Singlet	Adipocyte
VAT_2M	1832.5	1245	1.1921094	3	Singlet	FAPs
VAT_2M	1894.7	1383	0	7	Singlet	Endothelial_cells
VAT_2M	1689.8	1268	1.6081589	6	Singlet	Neurons
VAT_2M	1888.5	1466	1.8938061	6	Singlet	Neurons
VAT_2M	2588.7	1542	3.6665954	5	Singlet	Immune_cells
VAT_2M	6079.1	2630	0.010629	0	Singlet	Adipocyte
VAT_2M	12852	3856	0.0128769	0	Singlet	Adipocyte
VAT_2M	3633.1	1904	0.0159699	0	Singlet	Adipocyte
VAT_2M	1129	919	1.7311139	6	Singlet	Neurons
VAT_2M	6756.5	2734	0.0186002	0	Singlet	Adipocyte
VAT_2M	2192	1233	3.3407231	2	Singlet	FAPs
VAT_2M	6456.1	2675	0.0150088	0	Singlet	Adipocyte
VAT_2M	2297.8	1595	0.3630113	2	Singlet	FAPs
VAT_2M	3729.1	1724	0.6168658	0	Singlet	Adipocyte
VAT_2M	3567.9	1751	0.004669	0	Singlet	Adipocyte
VAT_2M	4308.4	2173	0.2195677	0	Singlet	Adipocyte
VAT_2M	2025	1342	2.0052591	3	Singlet	FAPs
VAT_2M	2019.5	1246	1.4944142	0	Singlet	Adipocyte
VAT_2M	1734.1	1346	1.1931344	7	Singlet	Endothelial_cells
VAT_2M	5916.6	2587	0.0539937	0	Singlet	Adipocyte
VAT_2M	1883	1248	0.3827182	3	Singlet	FAPs
VAT_2M	2107.9	1273	2.0320177	0	Singlet	Adipocyte
VAT_2M	3297.7	1888	2.0581278	3	Singlet	FAPs
VAT_2M	5452.9	2591	0.0357695	0	Singlet	Adipocyte
VAT_2M	7008.7	2665	0.0674877	0	Singlet	Adipocyte
VAT_2M	7076.5	2990	0.0361824	0	Singlet	Adipocyte
VAT_2M	1793.4	1279	1.7902293	2	Singlet	FAPs
VAT_2M	3441.3	1880	0.0940923	0	Singlet	Adipocyte
VAT_2M	1590.5	1287	1.4061697	6	Singlet	Neurons
VAT_2M	2968	1705	0.1828627	0	Singlet	Adipocyte
VAT_2M	4336.3	2182	0.0797637	0	Singlet	Adipocyte
VAT_2M	3966.9	1922	0.0152027	0	Singlet	Adipocyte
VAT_2M	3777.6	2258	1.5833905	2	Singlet	FAPs
VAT_2M	2725.1	1620	0.1612911	2	Singlet	FAPs
VAT_2M	6552.9	2663	0.0863933	0	Singlet	Adipocyte

VAT_2M	1078.8	761	3.6005835	9 Singlet	Mesothelial
VAT_2M	1822.2	1215	0.4392513	7 Singlet	Endothelial_cells
VAT_2M	10590	3474	0.4958352	0 Singlet	Adipocyte
VAT_2M	1463.8	1083	0.9371397	2 Singlet	FAPs
VAT_2M	1523.1	1069	3.8473202	9 Singlet	Mesothelial
VAT_2M	1307.6	1066	1.4921963	6 Singlet	Neurons
VAT_2M	4697.9	2221	0.0875495	0 Singlet	Adipocyte
VAT_2M	1569.4	1068	0.3275396	3 Singlet	FAPs
VAT_2M	7518.9	2958	0.0041301	0 Singlet	Adipocyte
VAT_2M	1255.8	937	0.9263623	3 Singlet	FAPs
VAT_2M	1509.2	1137	1.6387338	2 Singlet	FAPs
VAT_2M	1638.6	1063	7.7936545	7 Singlet	Endothelial_cells
VAT_2M	2971.1	1609	3.4468397	0 Singlet	Adipocyte
VAT_2M	10175	3410	0.1002662	0 Singlet	Adipocyte
VAT_2M	1417.2	1074	0.5372862	2 Singlet	FAPs
VAT_2M	1675.3	1159	1.5903061	5 Singlet	Immune_cells
VAT_2M	9440.5	3188	1.0808176	0 Singlet	Adipocyte
VAT_2M	1502.7	1194	1.5223364	8 Singlet	Mesothelial
VAT_2M	1537	1207	3.3042787	7 Singlet	Endothelial_cells
VAT_2M	1145.4	928	2.0821911	3 Singlet	FAPs
VAT_2M	1752.1	1113	0.3166752	0 Singlet	Adipocyte
VAT_2M	2271.4	1424	0.6016269	2 Singlet	FAPs
VAT_2M	6158.9	2674	0.0687361	0 Singlet	Adipocyte
VAT_2M	1716.3	1229	0.5311025	7 Singlet	Endothelial_cells
VAT_2M	3906.7	2016	0.1421738	0 Singlet	Adipocyte
VAT_2M	4289.3	1846	0.105528	0 Singlet	Adipocyte
VAT_2M	2434.9	1633	2.0551466	2 Singlet	FAPs
VAT_2M	1705.9	1287	2.232461	2 Singlet	FAPs
VAT_2M	2103	1500	1.4630646	5 Singlet	Immune_cells
VAT_2M	1693.1	1234	1.309162	2 Singlet	FAPs
VAT_2M	1676.7	1193	0.6360697	0 Singlet	Adipocyte
VAT_2M	4759.4	2132	0.0198912	0 Singlet	Adipocyte
VAT_2M	1882.1	1387	0.6580119	6 Singlet	Neurons
VAT_2M	11081	3693	0.0102471	0 Singlet	Adipocyte
VAT_2M	1316.5	1033	3.1241706	2 Singlet	FAPs
VAT_2M	2259.8	1416	0.2572403	0 Singlet	Adipocyte
VAT_2M	1412.8	1111	0.3775044	2 Singlet	FAPs
VAT_2M	5886.6	2526	0.1220855	0 Singlet	Adipocyte
VAT_2M	9653.5	3367	0	0 Singlet	Adipocyte
VAT_2M	2714.8	1650	0.3319799	0 Singlet	Adipocyte
VAT_2M	1471.8	1230	0.6900373	6 Singlet	Neurons
VAT_2M	1766.3	1101	0.6598122	0 Singlet	Adipocyte
VAT_2M	1567.4	1119	0.7820731	8 Singlet	Mesothelial
VAT_2M	2079.8	1515	0.9920444	6 Singlet	Neurons
VAT_2M	4919.4	2174	0.012536	0 Singlet	Adipocyte
VAT_2M	3219.2	1724	0.3729353	7 Singlet	Endothelial_cells
VAT_2M	2075.4	1216	6.6587295	5 Singlet	Immune_cells
VAT_2M	6579.6	3156	0.744835	2 Singlet	FAPs
VAT_2M	7925.7	2866	0.2858666	0 Singlet	Adipocyte
VAT_2M	2968.2	1494	0.1390857	0 Singlet	Adipocyte
VAT_2M	1059.6	857	0.790719	8 Singlet	Mesothelial

VAT_2M	1442.6	1157	0.2877408	4	Singlet	Immune_cells
VAT_2M	1262	960	0.9838867	2	Singlet	FAPs
VAT_2M	4056.6	2163	0.4919357	0	Singlet	Adipocyte
VAT_2M	3727.4	2061	0.3557279	0	Singlet	Adipocyte
VAT_2M	1736.2	1196	3.5052562	3	Singlet	FAPs
VAT_2M	7352	2801	0.0181213	0	Singlet	Adipocyte
VAT_2M	1673.4	1114	0.5143159	3	Singlet	FAPs
VAT_2M	1076.4	848	0.4613799	1	Singlet	Immune_cells
VAT_2M	4860.2	2250	0.9112776	0	Singlet	Adipocyte
VAT_2M	2512	1479	0.4589888	0	Singlet	Adipocyte
VAT_2M	2222	1418	0.4327544	6	Singlet	Neurons
VAT_2M	1393.4	1022	0.3225592	2	Singlet	FAPs
VAT_2M	2788.4	1670	1.0436214	0	Singlet	Adipocyte
VAT_2M	7090.1	2805	0.7181807	0	Singlet	Adipocyte
VAT_2M	7617.8	2923	0.045627	0	Singlet	Adipocyte
VAT_2M	4153.4	2145	0.0124441	0	Singlet	Adipocyte
VAT_2M	2235	1376	0.2588718	0	Singlet	Adipocyte
VAT_2M	2146.1	1342	0.0796289	0	Singlet	Adipocyte
VAT_2M	1362.6	1027	2.8756587	6	Singlet	Neurons
VAT_2M	1974	1353	0.0637886	6	Singlet	Neurons
VAT_2M	1763.1	1093	0	0	Singlet	Adipocyte
VAT_2M	2219.9	1363	5.33878	2	Singlet	FAPs
VAT_2M	12738	3791	0.0074988	0	Singlet	Adipocyte
VAT_2M	8037.9	3110	0.888539	0	Singlet	Adipocyte
VAT_2M	1485.5	1096	1.1422545	2	Singlet	FAPs
VAT_2M	1970.1	1357	0.5040897	0	Singlet	Adipocyte
VAT_2M	8109.6	2957	0.2915463	0	Singlet	Adipocyte
VAT_2M	1683.9	1258	2.6801316	5	Singlet	Immune_cells
VAT_2M	3529.3	1882	0.5321989	0	Singlet	Adipocyte
VAT_2M	3179.3	1972	0.8396947	2	Singlet	FAPs
VAT_2M	2087	1426	0.7908775	3	Singlet	FAPs
VAT_2M	1403.9	907	0.5328551	3	Singlet	FAPs
VAT_2M	1980.9	1267	0.7146678	2	Singlet	FAPs
VAT_2M	1240.4	896	0.9490552	9	Singlet	Mesothelial
VAT_2M	6463.6	2848	0.1156309	0	Singlet	Adipocyte
VAT_2M	1803.1	1247	0.4830896	6	Singlet	Neurons
VAT_2M	2886.4	1713	0.447374	0	Singlet	Adipocyte
VAT_2M	2692.8	1691	8.9728816	3	Singlet	FAPs
VAT_2M	3679.7	2100	0.9583193	2	Singlet	FAPs
VAT_2M	6097.2	2494	0.2555398	0	Singlet	Adipocyte
VAT_2M	2206	1225	0.2257247	0	Singlet	Adipocyte
VAT_2M	3444.6	1694	0.0937533	0	Singlet	Adipocyte
VAT_2M	4979	2212	0.2434711	0	Singlet	Adipocyte
VAT_2M	1139	881	4.0941235	6	Singlet	Neurons
VAT_2M	2165.8	1251	3.7593923	0	Singlet	Adipocyte
VAT_2M	1311.6	936	0.7962675	7	Singlet	Endothelial_cells
VAT_2M	2088.9	1476	2.3025606	3	Singlet	FAPs
VAT_2M	1240.2	892	4.3849415	6	Singlet	Neurons
VAT_2M	6812.7	2848	0.1128333	0	Singlet	Adipocyte
VAT_2M	1600.1	1167	3.3312681	5	Singlet	Immune_cells
VAT_2M	4440.2	2169	2.6916252	0	Singlet	Adipocyte

VAT_2M	6995.2	2753	0.3448657	0	Singlet	Adipocyte
VAT_2M	1422.8	1013	0.8517872	2	Singlet	FAPs
VAT_2M	4960.2	2299	0.1069986	0	Singlet	Adipocyte
VAT_2M	1581.5	1151	0.4895076	3	Singlet	FAPs
VAT_2M	3641.1	1734	0.0357409	0	Singlet	Adipocyte
VAT_2M	1526.8	1068	1.6273198	3	Singlet	FAPs
VAT_2M	1403.7	1189	0.3268546	5	Singlet	Immune_cells
VAT_2M	1137.1	831	0.8720418	2	Singlet	FAPs
VAT_2M	4552.2	2071	0.0560787	0	Singlet	Adipocyte
VAT_2M	1688.3	1131	0.2982222	0	Singlet	Adipocyte
VAT_2M	6314.9	2691	0.0984268	0	Singlet	Adipocyte
VAT_2M	1229.9	842	0.8206491	0	Singlet	Adipocyte
VAT_2M	2582.5	1699	0.212533	2	Singlet	FAPs
VAT_2M	7286.7	2835	0.0156591	0	Singlet	Adipocyte
VAT_2M	4626.6	2196	0.0725756	0	Singlet	Adipocyte
VAT_2M	3613.8	1996	0.5462903	0	Singlet	Adipocyte
VAT_2M	1223.9	947	1.2548843	6	Singlet	Neurons
VAT_2M	3155.9	1641	0.089599	0	Singlet	Adipocyte
VAT_2M	5306.4	2324	0	0	Singlet	Adipocyte
VAT_2M	1269.1	907	1.7572143	3	Singlet	FAPs
VAT_2M	1110.4	819	0.0121949	6	Singlet	Neurons
VAT_2M	1467	1075	0.9553714	2	Singlet	FAPs
VAT_2M	2024.6	1213	0.2013835	2	Singlet	FAPs
VAT_2M	5513.5	2409	0.0114857	0	Singlet	Adipocyte
VAT_2M	2849.4	1735	0.570425	5	Singlet	Immune_cells
VAT_2M	2107.8	1379	2.1311494	0	Singlet	Adipocyte
VAT_2M	1612	1137	6.7129193	2	Singlet	FAPs
VAT_2M	1007.3	809	3.8328227	8	Singlet	Mesothelial
VAT_2M	1169.5	966	0.7265314	6	Singlet	Neurons
VAT_2M	7478	2993	0.3239965	0	Singlet	Adipocyte
VAT_2M	9044.2	3216	0.2948653	0	Singlet	Adipocyte
VAT_2M	8429.9	3111	0.0279563	0	Singlet	Adipocyte
VAT_2M	1272.4	976	3.6402002	8	Singlet	Mesothelial
VAT_2M	1760.9	1122	12.596156	7	Singlet	Endothelial_cells
VAT_2M	1019.2	808	2.460451	6	Singlet	Neurons
VAT_2M	6331.2	2444	0.0549502	0	Singlet	Adipocyte
VAT_2M	8375.5	3104	0.0600497	0	Singlet	Adipocyte
VAT_2M	2200.5	1373	0.7029707	6	Singlet	Neurons
VAT_2M	1920.3	1064	0.5979222	0	Singlet	Adipocyte
VAT_2M	1445	1197	0.8513768	6	Singlet	Neurons
VAT_2M	1218.3	866	4.5994526	1	Singlet	Immune_cells
VAT_2M	6016	2606	0	0	Singlet	Adipocyte
VAT_2M	2683.7	1594	0.5535209	0	Singlet	Adipocyte
VAT_2M	5350.7	2331	0.0364403	0	Singlet	Adipocyte
VAT_2M	1157.1	938	0.2510104	8	Singlet	Mesothelial
VAT_2M	6145.5	2556	0.0053708	0	Singlet	Adipocyte
VAT_2M	1004.2	700	8.1415096	6	Singlet	Neurons
VAT_2M	6726.6	2619	0.0031618	0	Singlet	Adipocyte
VAT_2M	1842.4	1195	0.9787344	5	Singlet	Immune_cells
VAT_2M	1276.2	1088	0.7894308	7	Singlet	Endothelial_cells
VAT_2M	2781	1478	8.4984235	7	Singlet	Endothelial_cells

VAT_2M	1110.4	848	3.5715663	2	Singlet	FAPs
VAT_2M	2369.8	1594	0.5030525	5	Singlet	Immune_cells
VAT_2M	1026.6	831	0.5421394	11	Singlet	Smooth_muscle_cells
VAT_2M	2051.3	1349	0.7204393	3	Singlet	FAPs
VAT_2M	1389.3	1026	0.3582577	3	Singlet	FAPs
VAT_2M	1574.1	956	1.1523313	6	Singlet	Neurons
VAT_2M	8923.8	3183	0.0042743	0	Singlet	Adipocyte
VAT_2M	1322.5	1070	4.8490368	6	Singlet	Neurons
VAT_2M	5817.3	2465	0.0048927	0	Singlet	Adipocyte
VAT_2M	1922.9	1256	0.5820136	5	Singlet	Immune_cells
VAT_2M	3343.5	1815	0.1681107	0	Singlet	Adipocyte
VAT_2M	2379.3	1399	0.283341	0	Singlet	Adipocyte
VAT_2M	4417.8	2363	1.8104854	3	Singlet	FAPs
VAT_2M	7865.8	2932	0.044015	0	Singlet	Adipocyte
VAT_2M	5508.1	2494	0.0079873	0	Singlet	Adipocyte
VAT_2M	5174.3	2388	0.0290315	0	Singlet	Adipocyte
VAT_2M	1281.4	1079	1.1266321	7	Singlet	Endothelial_cells
VAT_2M	1347.5	1162	0.3734062	6	Singlet	Neurons
VAT_2M	3136.4	1879	1.7129226	2	Singlet	FAPs
VAT_2M	2005.5	1365	2.5167065	3	Singlet	FAPs
VAT_2M	1250	925	2.3573374	9	Singlet	Mesothelial
VAT_2M	1332	1006	1.2301018	0	Singlet	Adipocyte
VAT_2M	6072.9	2641	0.2044279	0	Singlet	Adipocyte
VAT_2M	4405.5	2173	0.4502108	0	Singlet	Adipocyte
VAT_2M	3083.4	1936	1.7498032	3	Singlet	FAPs
VAT_2M	1217.9	976	1.4641455	5	Singlet	Immune_cells
VAT_2M	1867	1372	1.062974	3	Singlet	FAPs
VAT_2M	3144.5	1926	1.3165146	3	Singlet	FAPs
VAT_2M	3308.8	1774	0.8037799	7	Singlet	Endothelial_cells
VAT_2M	2419.7	1472	1.0475338	0	Singlet	Adipocyte
VAT_2M	1579.1	986	0.5034384	0	Singlet	Adipocyte
VAT_2M	1567.5	1078	0.2402786	6	Singlet	Neurons
VAT_2M	6703.4	2834	0.0141405	0	Singlet	Adipocyte
VAT_2M	3704.6	1761	0.802769	0	Singlet	Adipocyte
VAT_2M	3077.9	1877	0.5618574	0	Singlet	Adipocyte
VAT_2M	2743.6	1579	0.202667	0	Singlet	Adipocyte
VAT_2M	9599.7	3411	0.802802	0	Singlet	Adipocyte
VAT_2M	6735.1	2732	0.0060445	0	Singlet	Adipocyte
VAT_2M	1081.1	776	2.5809888	5	Singlet	Immune_cells
VAT_2M	5235.6	2305	0.018218	0	Singlet	Adipocyte
VAT_2M	1003.9	767	1.7069672	2	Singlet	FAPs
VAT_2M	8874.7	3250	0.7370114	0	Singlet	Adipocyte
VAT_2M	6058.1	2637	1.5641632	0	Singlet	Adipocyte
VAT_2M	2617.8	1692	3.0272164	5	Singlet	Immune_cells
VAT_2M	10731	3264	0	0	Singlet	Adipocyte
VAT_2M	1204.5	930	2.2790046	2	Singlet	FAPs
VAT_2M	3442.9	1724	0.0720816	0	Singlet	Adipocyte
VAT_2M	6996.6	2836	0	0	Singlet	Adipocyte
VAT_2M	6380.5	2629	0.0455306	0	Singlet	Adipocyte
VAT_2M	2677.4	1368	0.2283756	0	Singlet	Adipocyte
VAT_2M	1311	952	0.9750397	6	Singlet	Neurons

VAT_2M	3607.6	1790	0.1443793	0	Singlet	Adipocyte
VAT_2M	6503.5	2760	0.0051658	0	Singlet	Adipocyte
VAT_2M	1582.8	964	0.758201	0	Singlet	Adipocyte
VAT_2M	6637.9	2791	0	0	Singlet	Adipocyte
VAT_2M	5323.2	2293	0.084527	0	Singlet	Adipocyte
VAT_2M	3931.6	1875	0.1174582	0	Singlet	Adipocyte
VAT_2M	4769.9	2410	0.0280692	0	Singlet	Adipocyte
VAT_2M	2986	1609	2.1312697	0	Singlet	Adipocyte
VAT_2M	2484.3	1461	0.1129892	0	Singlet	Adipocyte
VAT_2M	1605.2	1216	1.035716	5	Singlet	Immune_cells
VAT_2M	3634.8	1897	0.2180187	0	Singlet	Adipocyte
VAT_2M	2411.7	1430	0.5544713	3	Singlet	FAPs
VAT_2M	1323.2	1099	0.9359281	4	Singlet	Immune_cells
VAT_2M	1543	1100	0.9684639	2	Singlet	FAPs
VAT_2M	10578	3438	0	0	Singlet	Adipocyte
VAT_2M	1748.9	1161	0.1167892	0	Singlet	Adipocyte
VAT_2M	1885.1	1104	0.4349642	0	Singlet	Adipocyte
VAT_2M	1230.1	915	1.3447139	2	Singlet	FAPs
VAT_2M	2086	1367	0.4203947	0	Singlet	Adipocyte
VAT_2M	1928.7	1134	0	0	Singlet	Adipocyte
VAT_2M	6341.6	2522	0.0483959	0	Singlet	Adipocyte
VAT_2M	2895.3	1638	0.0735152	0	Singlet	Adipocyte
VAT_2M	2380.1	1420	0.028722	0	Singlet	Adipocyte
VAT_2M	1393.7	982	0.3961048	5	Singlet	Immune_cells
VAT_2M	1195.1	913	1.7959861	3	Singlet	FAPs
VAT_2M	1624.9	1109	2.97371	9	Singlet	Mesothelial
VAT_2M	4793.9	2098	0.079606	0	Singlet	Adipocyte
VAT_2M	2144.7	1394	0.2409067	0	Singlet	Adipocyte
VAT_2M	7341.5	2920	0.1816129	0	Singlet	Adipocyte
VAT_2M	6837.2	2640	0.0402888	0	Singlet	Adipocyte
VAT_2M	4241.9	1948	0.0671811	0	Singlet	Adipocyte
VAT_2M	2507.8	1579	0.1754507	2	Singlet	FAPs
VAT_2M	6929.5	2736	0.137702	0	Singlet	Adipocyte
VAT_2M	6433.9	2681	0.0530587	0	Singlet	Adipocyte
VAT_2M	6467.1	2605	0.0045196	0	Singlet	Adipocyte
VAT_2M	3273.3	1567	0.7556137	0	Singlet	Adipocyte
VAT_2M	2641.8	1590	0.302869	0	Singlet	Adipocyte
VAT_2M	5663.4	2393	0.306423	0	Singlet	Adipocyte
VAT_2M	3432.4	1852	0.4252445	0	Singlet	Adipocyte
VAT_2M	6920.4	2679	0.0641804	0	Singlet	Adipocyte
VAT_2M	1181.4	894	3.5043028	2	Singlet	FAPs
VAT_2M	1049.8	832	1.7034456	9	Singlet	Mesothelial
VAT_2M	1518.1	1289	3.5468273	6	Singlet	Neurons
VAT_2M	4970.7	2423	0.0348699	0	Singlet	Adipocyte
VAT_2M	1859	1106	2.2078635	5	Singlet	Immune_cells
VAT_2M	8125.7	2992	0.6998444	0	Singlet	Adipocyte
VAT_2M	3193.5	1889	0.8076622	3	Singlet	FAPs
VAT_2M	1796.1	1588	1.0859252	8	Singlet	Mesothelial
VAT_2M	1387.7	931	3.8931923	2	Singlet	FAPs
VAT_2M	9107.7	3116	1.365598	0	Singlet	Adipocyte
VAT_2M	5836.9	2716	0.2080414	0	Singlet	Adipocyte

VAT_2M	5449	2584	1.8845608	2	Singlet	FAPs
VAT_2M	1457.3	1045	1.9238431	9	Singlet	Mesothelial
VAT_2M	1110	917	0.1235524	3	Singlet	FAPs
VAT_2M	1376.6	954	2.6299412	2	Singlet	FAPs
VAT_2M	2143	1314	0.2387235	0	Singlet	Adipocyte
VAT_2M	2295.3	1358	0.4701168	0	Singlet	Adipocyte
VAT_2M	2742.5	1746	0.3327996	0	Singlet	Adipocyte
VAT_2M	1320.6	961	5.0596683	3	Singlet	FAPs
VAT_2M	4216.9	2076	0.1027271	0	Singlet	Adipocyte
VAT_2M	9634	3178	1.5910541	0	Singlet	Adipocyte
VAT_2M	8420	2959	0.0268213	0	Singlet	Adipocyte
VAT_2M	4307.8	2203	0.0588331	0	Singlet	Adipocyte
VAT_2M	6182	2640	0.5392511	0	Singlet	Adipocyte
VAT_2M	2599.7	1510	1.8308566	5	Singlet	Immune_cells
VAT_2M	1822.7	1479	1.0861429	6	Singlet	Neurons
VAT_2M	2107.6	1451	0.3387542	2	Singlet	FAPs
VAT_2M	9292.5	3124	0.0404034	0	Singlet	Adipocyte
VAT_2M	3046.7	1584	0.6460822	0	Singlet	Adipocyte
VAT_2M	3611.9	1959	0.6307752	5	Singlet	Immune_cells
VAT_2M	2538	1599	2.6950447	2	Singlet	FAPs
VAT_2M	9283.8	2916	0.0077755	0	Singlet	Adipocyte
VAT_2M	4338.7	2054	0.4219599	0	Singlet	Adipocyte
VAT_2M	3695.4	1836	0.3727329	0	Singlet	Adipocyte
VAT_2M	2084.4	1328	0.9941259	0	Singlet	Adipocyte
VAT_2M	3591.5	2063	1.1815113	2	Singlet	FAPs
VAT_2M	6754.7	2643	1.5831435	0	Singlet	Adipocyte
VAT_2M	3700.1	1835	0.1912963	0	Singlet	Adipocyte
VAT_2M	2619.6	1385	0.2990076	0	Singlet	Adipocyte
VAT_2M	8731.7	3096	0	0	Singlet	Adipocyte
VAT_2M	6570.7	2704	0.0228767	0	Singlet	Adipocyte
VAT_2M	3180.3	1813	0.814335	0	Singlet	Adipocyte
VAT_2M	4685.5	2112	0.3352961	0	Singlet	Adipocyte
VAT_2M	2309.3	1385	0.1533617	0	Singlet	Adipocyte
VAT_2M	4685.7	2010	0.6380354	0	Singlet	Adipocyte
VAT_2M	3752.2	1827	0.0468652	0	Singlet	Adipocyte
VAT_2M	8327.4	3256	0.2206173	0	Singlet	Adipocyte
VAT_2M	7851.7	2963	0.0281868	0	Singlet	Adipocyte
VAT_2M	1281	1013	0.2605431	7	Singlet	Endothelial_cells
VAT_2M	8924.3	2935	0.1707226	0	Singlet	Adipocyte
VAT_2M	1808.5	1090	2.0060377	5	Singlet	Immune_cells
VAT_2M	8746.6	3112	0	0	Singlet	Adipocyte
VAT_2M	3120.3	1862	0.6079858	2	Singlet	FAPs
VAT_2M	2036.8	1101	3.8119476	6	Singlet	Neurons
VAT_2M	1455.5	1121	1.432825	3	Singlet	FAPs
VAT_2M	1718.8	1258	1.3604065	3	Singlet	FAPs
VAT_2M	1599.8	1091	5.4490276	9	Singlet	Mesothelial
VAT_2M	2608.7	1647	0.0566597	3	Singlet	FAPs
VAT_2M	2005.3	1277	0.5799626	7	Singlet	Endothelial_cells
VAT_2M	10374	3415	0.0099157	0	Singlet	Adipocyte
VAT_2M	4767.4	2353	0.6265726	0	Singlet	Adipocyte
VAT_2M	1732.8	1021	0	0	Singlet	Adipocyte

VAT_2M	3309.6	2046	1.1291333	2	Singlet	FAPs
VAT_2M	1357.3	999	1.1913475	2	Singlet	FAPs
VAT_2M	1500	1057	1.9658605	3	Singlet	FAPs
VAT_2M	2689.7	1614	0.8224974	3	Singlet	FAPs
VAT_2M	8612.4	3247	0.0321094	0	Singlet	Adipocyte
VAT_2M	1041.8	775	0.0778179	7	Singlet	Endothelial_cells
VAT_2M	1870.5	1256	0.7613144	5	Singlet	Immune_cells
VAT_2M	2310.8	1362	3.592509	2	Singlet	FAPs
VAT_2M	2577.8	1555	0.4932217	7	Singlet	Endothelial_cells
VAT_2M	5630.7	2443	0.0536844	0	Singlet	Adipocyte
VAT_2M	2044.2	1342	0	3	Singlet	FAPs
VAT_2M	5680.7	2458	0.0250066	0	Singlet	Adipocyte
VAT_2M	3377.5	1939	1.8948214	2	Singlet	FAPs
VAT_2M	3306.6	1916	1.2327089	3	Singlet	FAPs
VAT_2M	7897	3127	0.1159701	0	Singlet	Adipocyte
VAT_2M	7600.5	2839	0.0038634	0	Singlet	Adipocyte
VAT_2M	1265.7	822	4.1589881	5	Singlet	Immune_cells
VAT_2M	1141.1	943	0.3421911	9	Singlet	Mesothelial
VAT_2M	2875.4	1479	0.0242328	0	Singlet	Adipocyte
VAT_2M	2545.3	1438	0.039421	0	Singlet	Adipocyte
VAT_2M	1260.8	1012	0.8380654	6	Singlet	Neurons
VAT_2M	2518.6	1517	3.1140361	0	Singlet	Adipocyte
VAT_2M	3085.3	1619	0.2669898	0	Singlet	Adipocyte
VAT_2M	3315	1696	0.0774437	0	Singlet	Adipocyte
VAT_2M	1141.1	970	1.7755513	1	Singlet	Immune_cells
VAT_2M	2566.1	1502	0.9267752	0	Singlet	Adipocyte
VAT_2M	4554.4	2315	0.0642827	0	Singlet	Adipocyte
VAT_2M	1594.8	1056	1.1289297	2	Singlet	FAPs
VAT_2M	5162.6	2630	0.884818	3	Singlet	FAPs
VAT_2M	2985.5	1733	0.17765	0	Singlet	Adipocyte
VAT_2M	1536.6	1132	0.0111902	2	Singlet	FAPs
VAT_2M	3147.1	1829	0.3310071	0	Singlet	Adipocyte
VAT_2M	4810.6	2333	0.5013656	0	Singlet	Adipocyte
VAT_2M	1298.1	993	1.2610015	5	Singlet	Immune_cells
VAT_2M	7257.2	2937	0.4208636	0	Singlet	Adipocyte
VAT_2M	6401.5	2617	0.262308	0	Singlet	Adipocyte
VAT_2M	2767.6	1476	0.0229318	0	Singlet	Adipocyte
VAT_2M	4392.9	2129	0.6503617	0	Singlet	Adipocyte
VAT_2M	2516.2	1541	1.0896075	2	Singlet	FAPs
VAT_2M	1377.5	982	0.0189571	2	Singlet	FAPs
VAT_2M	1901.7	1338	1.4636303	2	Singlet	FAPs
VAT_2M	1205.8	935	0.7078588	2	Singlet	FAPs
VAT_2M	6589.6	2584	0.1479931	0	Singlet	Adipocyte
VAT_2M	1405.8	1133	1.4264196	6	Singlet	Neurons
VAT_2M	1108.4	811	3.8619462	2	Singlet	FAPs
VAT_2M	1171.5	905	1.9594645	8	Singlet	Mesothelial
VAT_2M	1116.3	839	1.4836198	2	Singlet	FAPs
VAT_2M	1947.7	1390	0.3139141	2	Singlet	FAPs
VAT_2M	3810.4	1814	1.1043864	0	Singlet	Adipocyte
VAT_2M	5789.5	2508	0.0087673	0	Singlet	Adipocyte
VAT_2M	8218.5	3111	0.1254165	0	Singlet	Adipocyte

VAT_2M	3809.5	1957	0.0141675	0	Singlet	Adipocyte
VAT_2M	3307.9	1764	0	0	Singlet	Adipocyte
VAT_2M	4760.3	2185	0.0101764	0	Singlet	Adipocyte
VAT_2M	1116.7	980	0.0562968	11	Singlet	Smooth_muscle_cells
VAT_2M	3043.7	1703	2.1133195	5	Singlet	Immune_cells
VAT_2M	1372.6	1065	1.9580238	8	Singlet	Mesothelial
VAT_2M	1391.1	1008	2.3867718	6	Singlet	Neurons
VAT_2M	1718.6	1174	0.9086224	2	Singlet	FAPs
VAT_2M	7688.3	2939	0.1390985	0	Singlet	Adipocyte
VAT_2M	2245.4	1365	0.0484971	3	Singlet	FAPs
VAT_2M	1643.6	1177	0.595444	2	Singlet	FAPs
VAT_2M	3533.2	1855	0.0163511	0	Singlet	Adipocyte
VAT_2M	1188.4	946	1.4703794	5	Singlet	Immune_cells
VAT_2M	2555.9	1531	1.050136	0	Singlet	Adipocyte
VAT_2M	1853.9	1338	3.0865169	2	Singlet	FAPs
VAT_2M	3238.8	1691	0.2452389	0	Singlet	Adipocyte
VAT_2M	6591.9	2648	0.0090207	0	Singlet	Adipocyte
VAT_2M	1816.6	1114	0.3326261	0	Singlet	Adipocyte
VAT_2M	6574	2743	0.08069	0	Singlet	Adipocyte
VAT_2M	2323.8	1332	1.427553	0	Singlet	Adipocyte
VAT_2M	1963.7	1567	0.7708757	11	Singlet	Smooth_muscle_cells
VAT_2M	3791.4	1808	0.6624154	0	Singlet	Adipocyte
VAT_2M	1380.2	785	13.786475	7	Singlet	Endothelial_cells
VAT_2M	3232.4	1794	1.2906004	0	Singlet	Adipocyte
VAT_2M	2162.9	1323	0.7721877	6	Singlet	Neurons
VAT_2M	5314.7	2330	0.0334471	0	Singlet	Adipocyte
VAT_2M	2612.7	1426	0	0	Singlet	Adipocyte
VAT_2M	1347.8	1002	1.6391638	1	Singlet	Immune_cells
VAT_2M	3659.1	1871	0.086659	0	Singlet	Adipocyte
VAT_2M	4187.5	1849	0.161781	0	Singlet	Adipocyte
VAT_2M	10592	3623	0.0099497	0	Singlet	Adipocyte
VAT_2M	1760.2	1256	0.9687285	7	Singlet	Endothelial_cells
VAT_2M	3543.2	1916	0.2746678	0	Singlet	Adipocyte
VAT_2M	2511.6	1511	0.3615317	0	Singlet	Adipocyte
VAT_2M	1049.5	820	0.425419	6	Singlet	Neurons
VAT_2M	1449.2	1101	0.8068579	6	Singlet	Neurons
VAT_2M	2262.6	1346	5.6745641	0	Singlet	Adipocyte
VAT_2M	12587	4064	0.2845839	0	Singlet	Adipocyte
VAT_2M	1697.8	1242	2.1436644	5	Singlet	Immune_cells
VAT_2M	3539.8	1829	0.3970415	0	Singlet	Adipocyte
VAT_2M	4553.3	2143	0.2941031	0	Singlet	Adipocyte
VAT_2M	1952.5	1286	1.071602	2	Singlet	FAPs
VAT_2M	3174.3	1698	0.4438436	0	Singlet	Adipocyte
VAT_2M	2460.2	1425	0.425372	3	Singlet	FAPs
VAT_2M	1171.8	827	0.5150685	3	Singlet	FAPs
VAT_2M	2084.9	1336	0.1470245	0	Singlet	Adipocyte
VAT_2M	4168.7	2150	1.2270595	0	Singlet	Adipocyte
VAT_2M	2411.6	1541	0.3050875	0	Singlet	Adipocyte
VAT_2M	1727.1	1203	5.0446929	2	Singlet	FAPs
VAT_2M	2306.5	1533	1.6326985	5	Singlet	Immune_cells
VAT_2M	1620.8	1058	0.2824939	0	Singlet	Adipocyte

VAT_2M	4012.6	1992	1.3951956	0	Singlet	Adipocyte
VAT_2M	1411	1056	0.4783417	6	Singlet	Neurons
VAT_2M	2149.1	1366	0.9053622	0	Singlet	Adipocyte
VAT_2M	1693.1	1021	4.6255228	0	Singlet	Adipocyte
VAT_2M	6207.5	2438	0.0052814	0	Singlet	Adipocyte
VAT_2M	2044.2	1394	1.9749148	3	Singlet	FAPs
VAT_2M	2514.2	1528	0.6147647	0	Singlet	Adipocyte
VAT_2M	10545	3351	0.163126	0	Singlet	Adipocyte
VAT_2M	2126.3	1194	0.1937595	0	Singlet	Adipocyte
VAT_2M	2884.7	1596	0.0560317	0	Singlet	Adipocyte
VAT_2M	1739.5	1250	0.2322381	2	Singlet	FAPs
VAT_2M	1745.6	1290	0.1719117	5	Singlet	Immune_cells
VAT_2M	3578.5	1788	0.5778807	0	Singlet	Adipocyte
VAT_2M	1116.5	794	0.3485572	3	Singlet	FAPs
VAT_2M	8710.1	3100	0.026736	0	Singlet	Adipocyte
VAT_2M	2615.5	1436	0.603098	0	Singlet	Adipocyte
VAT_2M	1433.8	1072	3.0884394	5	Singlet	Immune_cells
VAT_2M	6692.8	2708	0.0040151	0	Singlet	Adipocyte
VAT_2M	11182	3527	0	0	Singlet	Adipocyte
VAT_2M	1296.9	959	0.6227702	6	Singlet	Neurons
VAT_2M	1411.5	1259	0.1370601	6	Singlet	Neurons
VAT_2M	1179.4	839	1.1728523	2	Singlet	FAPs
VAT_2M	4172.7	2143	0.0737793	0	Singlet	Adipocyte
VAT_2M	2907.6	1599	2.9782418	2	Singlet	FAPs
VAT_2M	1558.1	1084	3.2504529	9	Singlet	Mesothelial
VAT_2M	4988	2228	0	0	Singlet	Adipocyte
VAT_2M	7762.6	3030	0.0470239	0	Singlet	Adipocyte
VAT_2M	1112.7	889	1.6068896	5	Singlet	Immune_cells
VAT_2M	2863.8	1478	3.6102852	5	Singlet	Immune_cells
VAT_2M	1571.1	1290	1.1289604	2	Singlet	FAPs
VAT_2M	1264.1	951	0.3007197	7	Singlet	Endothelial_cells
VAT_2M	5634	2477	0.2498071	0	Singlet	Adipocyte
VAT_2M	5172.8	2265	0.0987299	0	Singlet	Adipocyte
VAT_2M	6965.4	2730	1.4052775	0	Singlet	Adipocyte
VAT_2M	1655.3	1190	0.5145775	3	Singlet	FAPs
VAT_2M	1029.1	847	1.4071804	6	Singlet	Neurons
VAT_2M	2336.8	1368	9.1264691	7	Singlet	Endothelial_cells
VAT_2M	1290.2	1177	1.3296056	8	Singlet	Mesothelial
VAT_2M	5612.7	2447	0.2387173	0	Singlet	Adipocyte
VAT_2M	2330	1463	0.1923901	0	Singlet	Adipocyte
VAT_2M	1689.4	1122	2.0563833	2	Singlet	FAPs
VAT_2M	3124.4	1646	0.0709558	0	Singlet	Adipocyte
VAT_2M	1887.9	1156	3.4272172	5	Singlet	Immune_cells
VAT_2M	8598.9	3151	0.0053844	0	Singlet	Adipocyte
VAT_2M	3126.2	1833	0.0407422	0	Singlet	Adipocyte
VAT_2M	9958.3	3422	0.0897826	0	Singlet	Adipocyte
VAT_2M	7121.3	2727	0.1253685	0	Singlet	Adipocyte
VAT_2M	11028	3615	0	0	Singlet	Adipocyte
VAT_2M	5563.5	2426	0.0539585	0	Singlet	Adipocyte
VAT_2M	9636.5	3189	0.1711419	0	Singlet	Adipocyte
VAT_2M	2616.1	1576	0.8411978	6	Singlet	Neurons

VAT_2M	10493	3523	0.0008041	0	Singlet	Adipocyte
VAT_2M	1362.3	1138	1.729719	9	Singlet	Mesothelial
VAT_2M	1134.8	917	0.1217864	2	Singlet	FAPs
VAT_2M	1638	1144	0.5354771	3	Singlet	FAPs
VAT_2M	4897.1	2254	0.0401619	0	Singlet	Adipocyte
VAT_2M	7434	2812	0.0286259	0	Singlet	Adipocyte
VAT_2M	1730.9	1102	0.3966222	0	Singlet	Adipocyte
VAT_2M	6127.1	2657	0.531093	0	Singlet	Adipocyte
VAT_2M	5017.7	2250	0.0753321	0	Singlet	Adipocyte
VAT_2M	1040.5	874	1.040622	6	Singlet	Neurons
VAT_2M	9197.2	3292	0.0080523	0	Singlet	Adipocyte
VAT_2M	2046.5	1366	2.1187329	2	Singlet	FAPs
VAT_2M	2966.8	1777	2.3546969	3	Singlet	FAPs
VAT_2M	2475.7	1658	0.4129095	2	Singlet	FAPs
VAT_2M	1623.1	1095	3.6228365	7	Singlet	Endothelial_cells
VAT_2M	2298	1281	0.1107812	0	Singlet	Adipocyte
VAT_2M	3728.5	1915	0.9546938	0	Singlet	Adipocyte
VAT_2M	3723.6	1961	0.2602297	0	Singlet	Adipocyte
VAT_2M	1472.1	1199	0.3010397	5	Singlet	Immune_cells
VAT_2M	2575.6	1427	0.1826373	0	Singlet	Adipocyte
VAT_2M	1199.3	940	2.1623494	5	Singlet	Immune_cells
VAT_2M	1531.4	1216	0.2800951	3	Singlet	FAPs
VAT_2M	3304.3	1722	0.2010385	0	Singlet	Adipocyte
VAT_2M	1118.9	808	1.5680231	7	Singlet	Endothelial_cells
VAT_2M	1865.6	1269	0.1437157	5	Singlet	Immune_cells
VAT_2M	2745.6	1634	0.845001	3	Singlet	FAPs
VAT_2M	1942.5	1205	0.9740805	6	Singlet	Neurons
VAT_2M	1315.1	942	2.3858637	5	Singlet	Immune_cells
VAT_2M	1533.4	1174	1.5446086	4	Singlet	Immune_cells
VAT_2M	1048.4	877	1.9525913	1	Singlet	Immune_cells
VAT_2M	1169.1	905	0.2517082	2	Singlet	FAPs
VAT_2M	1849.4	1285	1.7176246	2	Singlet	FAPs
VAT_2M	1465	1033	0.6274958	5	Singlet	Immune_cells
VAT_2M	5221.1	2397	0.596849	0	Singlet	Adipocyte
VAT_2M	4065.3	2050	1.3562801	0	Singlet	Adipocyte
VAT_2M	1293.6	919	0.7530365	7	Singlet	Endothelial_cells
VAT_2M	5695.1	2541	0.0173887	0	Singlet	Adipocyte
VAT_2M	4962.2	2382	0.0603346	0	Singlet	Adipocyte
VAT_2M	5153.5	2256	0.1571931	0	Singlet	Adipocyte
VAT_2M	2034.6	1213	0.1257273	0	Singlet	Adipocyte
VAT_2M	1649.9	1084	1.4243731	3	Singlet	FAPs
VAT_2M	6391.9	2629	0.0801064	0	Singlet	Adipocyte
VAT_2M	1798.8	1265	0.1462189	3	Singlet	FAPs
VAT_2M	7733.5	2963	0	0	Singlet	Adipocyte
VAT_2M	1268.5	1045	1.8574894	4	Singlet	Immune_cells
VAT_2M	7693.8	3023	0.0026526	0	Singlet	Adipocyte
VAT_2M	2242.4	1286	0.0680423	0	Singlet	Adipocyte
VAT_2M	1085.1	736	2.2927479	9	Singlet	Mesothelial
VAT_2M	1293.6	1107	1.9205855	6	Singlet	Neurons
VAT_2M	3184.8	1637	0.0221778	0	Singlet	Adipocyte
VAT_2M	1105	911	0.4747854	6	Singlet	Neurons

VAT_2M	11352	3226	0.008994	0	Singlet	Adipocyte
VAT_2M	1468.1	934	4.7190687	5	Singlet	Immune_cells
VAT_2M	1160.2	851	0.0597601	5	Singlet	Immune_cells
VAT_2M	3215.4	1689	0.2684392	0	Singlet	Adipocyte
VAT_2M	4395	2133	0.0149441	0	Singlet	Adipocyte
VAT_2M	1401.2	1074	2.9200598	6	Singlet	Neurons
VAT_2M	4108.6	2175	0.0981998	2	Singlet	FAPs
VAT_2M	2071.1	1354	0.7220696	3	Singlet	FAPs
VAT_2M	1294.6	955	1.2757163	5	Singlet	Immune_cells
VAT_2M	1781.2	1402	0.851426	3	Singlet	FAPs
VAT_2M	2719.9	1506	0.3971139	0	Singlet	Adipocyte
VAT_2M	1867.9	1272	0.2446136	3	Singlet	FAPs
VAT_2M	3917.2	2250	1.2418261	2	Singlet	FAPs
VAT_2M	1685.4	1420	4.3494144	11	Singlet	Smooth_muscle_cells
VAT_2M	1925.5	1267	0.1350311	6	Singlet	Neurons
VAT_2M	5739.2	2396	0.1260149	0	Singlet	Adipocyte
VAT_2M	1437	998	0.5269329	6	Singlet	Neurons
VAT_2M	7700.3	2933	0.0110391	0	Singlet	Adipocyte
VAT_2M	2360.8	1706	1.3763879	2	Singlet	FAPs
VAT_2M	1210.7	896	1.361039	2	Singlet	FAPs
VAT_2M	1797	1246	0.2097615	2	Singlet	FAPs
VAT_2M	2665.8	1350	3.0109068	2	Singlet	FAPs
VAT_2M	2392.3	1446	3.152445	5	Singlet	Immune_cells
VAT_2M	1210.6	981	1.202732	5	Singlet	Immune_cells
VAT_2M	2057.1	1280	0.0573112	0	Singlet	Adipocyte
VAT_2M	1930	1318	0.2670154	2	Singlet	FAPs
VAT_2M	2690.5	1511	0.0678647	0	Singlet	Adipocyte
VAT_2M	1654.7	1066	3.0547571	0	Singlet	Adipocyte
VAT_2M	1293.5	937	14.07488	6	Singlet	Neurons
VAT_2M	7361	3030	0.0427091	0	Singlet	Adipocyte
VAT_2M	4778.1	2395	0	0	Singlet	Adipocyte
VAT_2M	2289.7	1446	1.2612841	0	Singlet	Adipocyte
VAT_2M	2187.3	1230	0.1315169	0	Singlet	Adipocyte
VAT_2M	1624.2	1090	2.0073269	0	Singlet	Adipocyte
VAT_2M	1248.3	1123	0.1383924	6	Singlet	Neurons
VAT_2M	1023.6	829	0.1365328	7	Singlet	Endothelial_cells
VAT_2M	1614.5	1251	0.7950969	2	Singlet	FAPs
VAT_2M	1194.7	982	0.9105773	1	Singlet	Immune_cells
VAT_2M	1098.9	882	1.9560166	1	Singlet	Immune_cells
VAT_2M	1210.5	865	2.8608622	2	Singlet	FAPs
VAT_2M	8235.3	3011	0	0	Singlet	Adipocyte
VAT_2M	1566.6	1256	0.3020417	6	Singlet	Neurons
VAT_2M	8214.5	2993	0.0245623	0	Singlet	Adipocyte
VAT_2M	2320.5	1593	0.727096	2	Singlet	FAPs
VAT_2M	1475.1	1023	0.1719876	5	Singlet	Immune_cells
VAT_2M	2387.1	1602	2.4068482	2	Singlet	FAPs
VAT_2M	4202.4	1936	0.0497165	0	Singlet	Adipocyte
VAT_2M	2587.7	1609	0.6100662	0	Singlet	Adipocyte
VAT_2M	2548	1539	0.211164	0	Singlet	Adipocyte
VAT_2M	8000.2	2948	0.1189507	0	Singlet	Adipocyte
VAT_2M	2059.1	1310	0.5116138	3	Singlet	FAPs

VAT_2M	1512	1022	0.0836389	3	Singlet	FAPs
VAT_2M	2057.8	1296	9.1327831	9	Singlet	Mesothelial
VAT_2M	1242.8	1027	0.5909987	6	Singlet	Neurons
VAT_2M	3294.8	1752	0.0691969	0	Singlet	Adipocyte
VAT_2M	1139.5	958	0.3715001	6	Singlet	Neurons
VAT_2M	2627.2	1514	1.1165371	3	Singlet	FAPs
VAT_2M	1722.2	1034	1.4590715	0	Singlet	Adipocyte
VAT_2M	2407	1418	0.028143	0	Singlet	Adipocyte
VAT_2M	1627.9	1124	0.8905642	9	Singlet	Mesothelial
VAT_2M	1718.6	1394	1.7202201	6	Singlet	Neurons
VAT_2M	8676	3203	0.0986489	0	Singlet	Adipocyte
VAT_2M	1706.1	1248	1.1978961	2	Singlet	FAPs
VAT_2M	2025.8	1185	0.1502198	0	Singlet	Adipocyte
VAT_2M	12011	3560	0.0373724	0	Singlet	Adipocyte
VAT_2M	2065.7	1236	7.6919112	7	Singlet	Endothelial_cells
VAT_2M	3150.4	1602	1.060554	0	Singlet	Adipocyte
VAT_2M	2806.6	1776	0.7805194	2	Singlet	FAPs
VAT_2M	2909.1	1744	0.0769314	0	Singlet	Adipocyte
VAT_2M	1801.2	1316	0.2149913	2	Singlet	FAPs
VAT_2M	9728.4	3224	0.0747207	0	Singlet	Adipocyte
VAT_2M	1516.2	1051	6.4380534	9	Singlet	Mesothelial
VAT_2M	1593.7	1038	2.1842111	0	Singlet	Adipocyte
VAT_2M	3454	1869	0.0485385	0	Singlet	Adipocyte
VAT_2M	1660.5	1220	0.777315	2	Singlet	FAPs
VAT_2M	1576	1103	1.5020842	5	Singlet	Immune_cells
VAT_2M	4130.2	1952	0.0605233	0	Singlet	Adipocyte
VAT_2M	3462.6	1885	0.1044907	0	Singlet	Adipocyte
VAT_2M	1600.3	1177	0.5705898	0	Singlet	Adipocyte
VAT_2M	5568.7	2297	0	0	Singlet	Adipocyte
VAT_2M	4988.5	2275	0.4590537	0	Singlet	Adipocyte
VAT_2M	2007.9	1278	0.3004663	0	Singlet	Adipocyte
VAT_2M	2190	1317	2.2435709	2	Singlet	FAPs
VAT_2M	1696	1095	0.5169291	5	Singlet	Immune_cells
VAT_2M	1011.2	787	1.0015628	6	Singlet	Neurons
VAT_2M	2664.6	1563	0.3516724	2	Singlet	FAPs
VAT_2M	2537.6	1588	0.6227279	0	Singlet	Adipocyte
VAT_2M	6916.4	2782	0.0065721	0	Singlet	Adipocyte
VAT_2M	1001	718	6.0616805	6	Singlet	Neurons
VAT_2M	1122.8	796	3.4582527	6	Singlet	Neurons
VAT_2M	2111.3	1309	1.8241609	0	Singlet	Adipocyte
VAT_2M	2793.1	1576	0.1495666	0	Singlet	Adipocyte
VAT_2M	2665.2	1563	0.6930188	0	Singlet	Adipocyte
VAT_2M	5891.1	2502	0.0343032	0	Singlet	Adipocyte
VAT_2M	1444.1	1203	0.3653382	1	Singlet	Immune_cells
VAT_2M	4219.9	1939	0.2624972	0	Singlet	Adipocyte
VAT_2M	6629.2	2851	0.1104438	0	Singlet	Adipocyte
VAT_2M	1021.7	817	2.7382636	10	Singlet	Immune_cells
VAT_2M	5979.1	2560	0.5682457	0	Singlet	Adipocyte
VAT_2M	7589.7	2935	0.0277538	0	Singlet	Adipocyte
VAT_2M	9865.2	3182	0	0	Singlet	Adipocyte
VAT_2M	1337.6	1040	0.9459473	4	Singlet	Immune_cells

VAT_2M	1286.6	845	1.7764377	2	Singlet	FAPs
VAT_2M	1339.6	1009	1.8960732	2	Singlet	FAPs
VAT_2M	2743.9	1471	0.1527123	0	Singlet	Adipocyte
VAT_2M	1046.6	900	0.2588635	10	Singlet	Immune_cells
VAT_2M	6534.3	2752	0.636345	0	Singlet	Adipocyte
VAT_2M	8031.7	2929	0.0023942	0	Singlet	Adipocyte
VAT_2M	1769.9	1309	1.3835078	2	Singlet	FAPs
VAT_2M	1258.7	845	5.4275905	5	Singlet	Immune_cells
VAT_2M	12291	3751	0.012997	0	Singlet	Adipocyte
VAT_2M	4286.2	2413	1.0383385	5	Singlet	Immune_cells
VAT_2M	6561.5	2583	0.02339	0	Singlet	Adipocyte
VAT_2M	7601.9	2866	0.0037347	0	Singlet	Adipocyte
VAT_2M	8354.2	2986	2.0650689	0	Singlet	Adipocyte
VAT_2M	6790.5	2771	0.003043	0	Singlet	Adipocyte
VAT_2M	1252.7	899	2.3590215	2	Singlet	FAPs
VAT_2M	1679	1092	0.8839399	3	Singlet	FAPs
VAT_2M	1666.1	1325	0.2163139	6	Singlet	Neurons
VAT_2M	3718.4	1970	0.1081644	0	Singlet	Adipocyte
VAT_2M	4614.1	2090	0.0115901	0	Singlet	Adipocyte
VAT_2M	8491.1	3061	0.0818568	0	Singlet	Adipocyte
VAT_2M	8106.8	3225	0.0025214	0	Singlet	Adipocyte
VAT_2M	1660	979	0.2069288	0	Singlet	Adipocyte
VAT_2M	6038.1	2532	0	0	Singlet	Adipocyte
VAT_2M	1576.7	1195	0.080066	3	Singlet	FAPs
VAT_2M	1333.4	949	0.4628779	7	Singlet	Endothelial_cells
VAT_2M	1325.7	950	0.7129681	3	Singlet	FAPs
VAT_2M	1187.6	831	1.4334116	6	Singlet	Neurons
VAT_2M	1236.5	880	0.1651563	6	Singlet	Neurons
VAT_2M	1027.2	825	0.6924728	4	Singlet	Immune_cells
VAT_2M	9185	3183	2.383175	0	Singlet	Adipocyte
VAT_2M	1038.4	788	1.5442606	11	Singlet	Smooth_muscle_cells
VAT_2M	1513.1	1132	0.8055038	3	Singlet	FAPs
VAT_2M	5589.7	2348	0.1004782	0	Singlet	Adipocyte
VAT_2M	1014.7	787	2.3381719	2	Singlet	FAPs
VAT_2M	2502.8	1569	0	6	Singlet	Neurons
VAT_2M	6239.2	2486	0.0219919	0	Singlet	Adipocyte
VAT_2M	1381	1080	0.6266282	2	Singlet	FAPs
VAT_2M	4723.8	2381	0.0037976	11	Singlet	Smooth_muscle_cells
VAT_2M	1060.3	800	0.1692104	0	Singlet	Adipocyte
VAT_2M	7886.4	2935	0.0024773	0	Singlet	Adipocyte
VAT_2M	1903.4	1401	3.5385764	2	Singlet	FAPs
VAT_2M	3268.4	1710	0.1066364	0	Singlet	Adipocyte
VAT_2M	4239.9	2064	0.0439697	0	Singlet	Adipocyte
VAT_2M	1667.7	1078	0.9196956	1	Singlet	Immune_cells
VAT_2M	1185.7	895	3.0900329	5	Singlet	Immune_cells
VAT_2M	1708.8	1067	0.8026343	2	Singlet	FAPs
VAT_2M	2867.1	1689	4.8159536	7	Singlet	Endothelial_cells
VAT_2M	1054.5	828	2.3508122	2	Singlet	FAPs
VAT_2M	15284	4008	2.0720991	0	Singlet	Adipocyte
VAT_2M	4272.8	2089	0.0640459	0	Singlet	Adipocyte
VAT_2M	10285	3550	0.0341468	0	Singlet	Adipocyte

VAT_2M	3200.7	1652	0.244416	0	Singlet	Adipocyte
VAT_2M	1075.7	869	2.4917119	2	Singlet	FAPs
VAT_2M	2608.2	1397	0.3537881	0	Singlet	Adipocyte
VAT_2M	6584.2	2654	0.033524	0	Singlet	Adipocyte
VAT_2M	5123.9	2252	2.5201091	0	Singlet	Adipocyte
VAT_2M	1004.8	770	1.4892836	2	Singlet	FAPs
VAT_2M	1052.4	752	1.1994059	2	Singlet	FAPs
VAT_2M	1853.2	1062	0.0422915	0	Singlet	Adipocyte
VAT_2M	3160.5	1744	0.0616139	0	Singlet	Adipocyte
VAT_2M	7355.8	2904	0.0352747	0	Singlet	Adipocyte
VAT_2M	9867.4	3202	0.018719	0	Singlet	Adipocyte
VAT_2M	2543.7	1468	0.0477212	0	Singlet	Adipocyte
VAT_2M	1766.2	1073	0.3994992	0	Singlet	Adipocyte
VAT_2M	1051.9	829	0.4876042	6	Singlet	Neurons
VAT_2M	9370.2	3124	0.0116401	0	Singlet	Adipocyte
VAT_2M	3357.1	1844	0.8995147	2	Singlet	FAPs
VAT_2M	4818.6	2285	0.3926133	0	Singlet	Adipocyte
VAT_2M	4231.1	2041	0	0	Singlet	Adipocyte
VAT_2M	7447.8	2815	0.7983191	0	Singlet	Adipocyte
VAT_2M	1764.4	1175	0.2023249	0	Singlet	Adipocyte
VAT_2M	3998.7	2033	0.2476425	0	Singlet	Adipocyte
VAT_2M	1489.2	1066	0.3431046	3	Singlet	FAPs
VAT_2M	2781.1	1706	0.0159634	2	Singlet	FAPs
VAT_2M	2145.4	1375	0.2821361	3	Singlet	FAPs
VAT_2M	2305.3	1367	1.2189003	0	Singlet	Adipocyte
VAT_2M	2332.4	1571	1.665076	2	Singlet	FAPs
VAT_2M	1337.5	941	2.3351178	2	Singlet	FAPs
VAT_2M	1472.8	1046	0.6564625	8	Singlet	Mesothelial
VAT_2M	2598.4	1526	0.1892469	0	Singlet	Adipocyte
VAT_2M	3764.8	2048	1.6543423	3	Singlet	FAPs
VAT_2M	1941.6	1271	1.1589947	7	Singlet	Endothelial_cells
VAT_2M	4856.3	2253	0.0494393	0	Singlet	Adipocyte
VAT_2M	4161.6	2047	0	0	Singlet	Adipocyte
VAT_2M	3587.9	1840	0.1095	0	Singlet	Adipocyte
VAT_2M	1672.9	1042	0.7965664	0	Singlet	Adipocyte
VAT_2M	2122.1	1302	0.6668491	0	Singlet	Adipocyte
VAT_2M	1048.2	847	0.483388	9	Singlet	Mesothelial
VAT_2M	3523.2	1811	1.2767686	0	Singlet	Adipocyte
VAT_2M	2925.6	1768	0.2240519	0	Singlet	Adipocyte
VAT_2M	1275.9	1111	0.6013499	6	Singlet	Neurons
VAT_2M	1426	941	1.285319	5	Singlet	Immune_cells
VAT_2M	1334.9	1035	1.7333427	2	Singlet	FAPs
VAT_2M	6984.9	2714	0.1298787	0	Singlet	Adipocyte
VAT_2M	3308	1611	4.1286662	2	Singlet	FAPs
VAT_2M	1779.5	1085	1.0331181	6	Singlet	Neurons
VAT_2M	4944.6	2463	0.1275718	0	Singlet	Adipocyte
VAT_2M	2699.3	1519	0.1040767	3	Singlet	FAPs
VAT_2M	1739.8	1297	2.5181027	1	Singlet	Immune_cells
VAT_2M	1696.3	1034	0.6487783	6	Singlet	Neurons
VAT_2M	3584.6	1722	2.1934313	0	Singlet	Adipocyte
VAT_2M	6597	2716	0.0345296	0	Singlet	Adipocyte

VAT_2M	5526.8	2374	0.0140634	0	Singlet	Adipocyte
VAT_2M	1394.4	998	5.1967988	9	Singlet	Mesothelial
VAT_2M	7489.3	2834	0.0451284	0	Singlet	Adipocyte
VAT_2M	1480.8	1177	0.352995	6	Singlet	Neurons
VAT_2M	6130.4	2706	0.1532538	0	Singlet	Adipocyte
VAT_2M	1245	915	0.3159294	2	Singlet	FAPs
VAT_2M	3839.8	1781	0.0823748	0	Singlet	Adipocyte
VAT_2M	2536.8	1583	1.3239935	7	Singlet	Endothelial_cells
VAT_2M	2969.1	1578	0.6307255	0	Singlet	Adipocyte
VAT_2M	3962.6	1905	0.1413191	0	Singlet	Adipocyte
VAT_2M	1531.3	1078	1.1121899	3	Singlet	FAPs
VAT_2M	6536	2642	0	0	Singlet	Adipocyte
VAT_2M	2996.1	1805	0.2940899	0	Singlet	Adipocyte
VAT_2M	6949.2	2594	0.093182	0	Singlet	Adipocyte
VAT_2M	1994.6	1206	3.0518338	6	Singlet	Neurons
VAT_2M	4054.9	1942	0.4146852	0	Singlet	Adipocyte
VAT_2M	9242.3	3309	0.1856804	0	Singlet	Adipocyte
VAT_2M	2108.7	1353	1.69261	6	Singlet	Neurons
VAT_2M	7242	3006	0.0205414	0	Singlet	Adipocyte
VAT_2M	2746.5	1704	2.2805864	5	Singlet	Immune_cells
VAT_2M	6104.4	2424	0	0	Singlet	Adipocyte
VAT_2M	1613.8	1018	0.8052647	5	Singlet	Immune_cells
VAT_2M	3899.3	2101	0.4008743	0	Singlet	Adipocyte
VAT_2M	6675.4	2771	0.9932322	0	Singlet	Adipocyte
VAT_2M	8124.9	2925	0.0062564	0	Singlet	Adipocyte
VAT_2M	1834.8	1340	0.8188741	3	Singlet	FAPs
VAT_2M	1188.7	890	0.8176747	3	Singlet	FAPs
VAT_2M	1784.9	1252	1.1549532	2	Singlet	FAPs
VAT_2M	2560.6	1625	1.3233496	3	Singlet	FAPs
VAT_2M	3647.7	1847	0.0328759	0	Singlet	Adipocyte
VAT_2M	8220.2	2865	0.0139983	0	Singlet	Adipocyte
VAT_2M	11056	3532	0.0298986	0	Singlet	Adipocyte
VAT_2M	1655.6	1178	1.0398596	6	Singlet	Neurons
VAT_2M	2808	1493	0.085206	0	Singlet	Adipocyte
VAT_2M	3031.6	1794	0.5430637	0	Singlet	Adipocyte
VAT_2M	4574.3	2677	1.7243081	2	Singlet	FAPs
VAT_2M	5763.2	2512	0.101553	0	Singlet	Adipocyte
VAT_2M	1055.1	760	2.8305206	2	Singlet	FAPs
VAT_2M	8041.1	3001	0.0504912	0	Singlet	Adipocyte
VAT_2M	1060.5	808	0.2100736	5	Singlet	Immune_cells
VAT_2M	3289.3	1783	1.0620004	0	Singlet	Adipocyte
VAT_2M	2745.3	1514	0.6859739	0	Singlet	Adipocyte
VAT_2M	3448.8	1831	0.0716512	0	Singlet	Adipocyte
VAT_2M	1013.2	854	0.7111446	3	Singlet	FAPs
VAT_2M	6330.1	2539	0.0428884	0	Singlet	Adipocyte
VAT_2M	2140.7	1242	0.1595823	0	Singlet	Adipocyte
VAT_2M	1236.8	916	0.0682356	3	Singlet	FAPs
VAT_2M	7712.4	2971	0.1523792	0	Singlet	Adipocyte
VAT_2M	5493.9	2426	0.1330135	0	Singlet	Adipocyte
VAT_2M	4561.7	2381	7.6773673	7	Singlet	Endothelial_cells
VAT_2M	8588.5	3145	0.023448	0	Singlet	Adipocyte

VAT_2M	5165.2	2368	0.1328192	0	Singlet	Adipocyte
VAT_2M	1263.9	914	1.4533519	2	Singlet	FAPs
VAT_2M	3355.7	2056	1.7983681	5	Singlet	Immune_cells
VAT_2M	1257.9	949	2.0343925	3	Singlet	FAPs
VAT_2M	1896.2	1227	0.5879624	0	Singlet	Adipocyte
VAT_2M	3284.9	1733	0.1453747	0	Singlet	Adipocyte
VAT_2M	1715.3	1228	0.1760622	3	Singlet	FAPs
VAT_2M	1156.3	951	0.5597381	6	Singlet	Neurons
VAT_2M	4992.4	2287	0.0078676	0	Singlet	Adipocyte
VAT_2M	1952.6	1279	0.1829261	2	Singlet	FAPs
VAT_2M	7607.4	2983	0.0293716	0	Singlet	Adipocyte
VAT_2M	5237.5	2398	0.032681	0	Singlet	Adipocyte
VAT_2M	1200.4	1035	1.06291	6	Singlet	Neurons
VAT_2M	5512.3	2492	0.0245662	0	Singlet	Adipocyte
VAT_2M	1825.3	1300	0.1560958	2	Singlet	FAPs
VAT_2M	7091	2735	0.0077873	0	Singlet	Adipocyte
VAT_2M	7109	2677	0.011865	0	Singlet	Adipocyte
VAT_2M	2426	1420	0.1173837	0	Singlet	Adipocyte
VAT_2M	1368.5	1001	2.7118011	2	Singlet	FAPs
VAT_2M	2018.5	1132	4.1867418	3	Singlet	FAPs
VAT_2M	5190.5	2153	0.0189663	0	Singlet	Adipocyte
VAT_2M	6204	2631	0.0394458	0	Singlet	Adipocyte
VAT_2M	1731.8	1090	0.7737044	0	Singlet	Adipocyte
VAT_2M	8405.5	3100	0.1924542	0	Singlet	Adipocyte
VAT_2M	1989.2	1358	1.7949021	5	Singlet	Immune_cells
VAT_2M	1110.1	896	2.0522509	8	Singlet	Mesothelial
VAT_2M	2681.8	1581	0.1998265	0	Singlet	Adipocyte
VAT_2M	4169.8	2089	0.2568468	0	Singlet	Adipocyte
VAT_2M	3150.7	1698	0.6238144	0	Singlet	Adipocyte
VAT_2M	1942.7	1248	0.6771478	6	Singlet	Neurons
VAT_2M	2346.4	1414	0.9152753	0	Singlet	Adipocyte
VAT_2M	1163.8	817	0.1653548	2	Singlet	FAPs
VAT_2M	1025.7	730	4.8644776	8	Singlet	Mesothelial
VAT_2M	1102.2	839	0.3877094	6	Singlet	Neurons
VAT_2M	1574.2	1093	0.2579384	5	Singlet	Immune_cells
VAT_2M	3318.1	1754	0.0790089	0	Singlet	Adipocyte
VAT_2M	4404.8	2099	0.198462	0	Singlet	Adipocyte
VAT_2M	1202.3	974	0.1292691	6	Singlet	Neurons
VAT_2M	4516.3	2066	0.0327989	0	Singlet	Adipocyte
VAT_2M	1270.3	943	0.4354211	3	Singlet	FAPs
VAT_2M	5441.8	2297	0.0406134	0	Singlet	Adipocyte
VAT_2M	1391.3	1034	1.5744541	6	Singlet	Neurons
VAT_2M	6139.7	2763	0.0215304	0	Singlet	Adipocyte
VAT_2M	2538.5	1389	0.0288881	0	Singlet	Adipocyte
VAT_2M	7311.1	2893	0.0945204	0	Singlet	Adipocyte
VAT_2M	1261.4	926	3.3448063	2	Singlet	FAPs
VAT_2M	4614.4	2282	0.0211841	0	Singlet	Adipocyte
VAT_2M	4920.4	2294	0.0181004	0	Singlet	Adipocyte
VAT_2M	1123.2	882	1.4117194	11	Singlet	Smooth_muscle_cells
VAT_2M	3473.7	2015	1.470894	3	Singlet	FAPs
VAT_2M	2768.6	1596	0.1484676	0	Singlet	Adipocyte

VAT_2M	1870.7	1106	0.0438527	0	Singlet	Adipocyte
VAT_2M	1132.2	857	1.387264	2	Singlet	FAPs
VAT_2M	2091.6	1428	1.8476994	2	Singlet	FAPs
VAT_2M	1509.2	1096	1.7871042	2	Singlet	FAPs
VAT_2M	1055.4	894	0.3890272	6	Singlet	Neurons
VAT_2M	5614.5	2377	0.0604513	0	Singlet	Adipocyte
VAT_2M	2231.8	1396	1.485406	6	Singlet	Neurons
VAT_2M	1072.7	753	4.1845749	3	Singlet	FAPs
VAT_2M	4106.7	2078	0.1625494	0	Singlet	Adipocyte
VAT_2M	7990.9	2769	0	0	Singlet	Adipocyte
VAT_2M	1677.9	1199	1.0699411	5	Singlet	Immune_cells
VAT_2M	3042.6	1630	0.0461359	0	Singlet	Adipocyte
VAT_2M	2287.1	1340	1.918961	0	Singlet	Adipocyte
VAT_2M	2278.7	1570	0.4470083	2	Singlet	FAPs
VAT_2M	2067.3	1349	0.9985586	2	Singlet	FAPs
VAT_2M	5160.9	2362	0.2382918	0	Singlet	Adipocyte
VAT_2M	2257.1	1402	0.7425757	5	Singlet	Immune_cells
VAT_2M	1064.6	900	1.6625118	6	Singlet	Neurons
VAT_2M	1110.8	848	0.263943	0	Singlet	Adipocyte
VAT_2M	8152	3037	0.0200797	0	Singlet	Adipocyte
VAT_2M	1954.2	1182	0	0	Singlet	Adipocyte
VAT_2M	1844.1	1533	1.3087995	2	Singlet	FAPs
VAT_2M	2292.6	1436	1.3373486	0	Singlet	Adipocyte
VAT_2M	1360.2	1018	4.4733271	2	Singlet	FAPs
VAT_2M	1141.5	874	1.0498638	2	Singlet	FAPs
VAT_2M	2158.2	1294	0.2461868	0	Singlet	Adipocyte
VAT_2M	2196.7	1346	0.1157615	0	Singlet	Adipocyte
VAT_2M	3263	1776	0.1404697	7	Singlet	Endothelial_cells
VAT_2M	2063.3	1329	0.1367425	3	Singlet	FAPs
VAT_2M	1055.5	955	0.5772454	9	Singlet	Mesothelial
VAT_2M	6605.1	2619	0.0030835	0	Singlet	Adipocyte
VAT_2M	5019.2	2332	0.1424958	0	Singlet	Adipocyte
VAT_2M	1804.2	1327	1.0525951	2	Singlet	FAPs
VAT_2M	1371	996	2.0664544	2	Singlet	FAPs
VAT_2M	1522.3	1073	1.954308	3	Singlet	FAPs
VAT_2M	6755.1	2833	0.1485472	0	Singlet	Adipocyte
VAT_2M	9350	3277	0.0509876	0	Singlet	Adipocyte
VAT_2M	3995.6	2153	0.2192328	0	Singlet	Adipocyte
VAT_2M	2850.6	1698	0.3732149	0	Singlet	Adipocyte
VAT_2M	2455.4	1546	0.1019647	0	Singlet	Adipocyte
VAT_2M	4191.3	2067	0.1885862	0	Singlet	Adipocyte
VAT_2M	2872.1	1641	0.243279	0	Singlet	Adipocyte
VAT_2M	1186.7	916	0.6745022	5	Singlet	Immune_cells
VAT_2M	1092.1	924	1.0014624	6	Singlet	Neurons
VAT_2M	1035.1	874	0.531413	6	Singlet	Neurons
VAT_2M	1778	1078	0.1560992	0	Singlet	Adipocyte
VAT_2M	2006.7	1299	1.3872313	2	Singlet	FAPs
VAT_2M	1043.4	827	0.4537176	6	Singlet	Neurons
VAT_2M	8892.4	3289	0.029666	0	Singlet	Adipocyte
VAT_2M	2387.7	1463	0.10163	0	Singlet	Adipocyte
VAT_2M	2132.1	1312	0.4758839	3	Singlet	FAPs

VAT_2M	4704.6	2084	0.1593426	0	Singlet	Adipocyte
VAT_2M	1522.7	1343	0.4363451	6	Singlet	Neurons
VAT_2M	7270	2790	0.0020315	0	Singlet	Adipocyte
VAT_2M	6898	3020	0.0871244	0	Singlet	Adipocyte
VAT_2M	2807.4	1663	1.1985942	6	Singlet	Neurons
VAT_2M	2356.1	1426	0.5040933	0	Singlet	Adipocyte
VAT_2M	2287.3	1609	0.0570566	2	Singlet	FAPs
VAT_2M	1513.6	1081	0.8064056	2	Singlet	FAPs
VAT_2M	1203.2	892	0.6421397	1	Singlet	Immune_cells
VAT_2M	1318.7	966	0.8752367	2	Singlet	FAPs
VAT_2M	1735.6	1054	0	0	Singlet	Adipocyte
VAT_2M	3956.1	1976	1.2321237	2	Singlet	FAPs
VAT_2M	1084.8	766	0.6484989	3	Singlet	FAPs
VAT_2M	1609.9	1064	0.0670099	3	Singlet	FAPs
VAT_2M	2058.5	1372	0.1342808	7	Singlet	Endothelial_cells
VAT_2M	2813.9	1587	0.3599729	0	Singlet	Adipocyte
VAT_2M	7290.4	3043	0.0435058	0	Singlet	Adipocyte
VAT_2M	1917.1	1221	1.3272158	0	Singlet	Adipocyte
VAT_2M	3052.2	1696	1.4626033	0	Singlet	Adipocyte
VAT_2M	1164.6	934	3.3215436	6	Singlet	Neurons
VAT_2M	1219	868	1.5549386	5	Singlet	Immune_cells
VAT_2M	2715.7	1508	7.6409404	7	Singlet	Endothelial_cells
VAT_2M	5416.3	2355	0.0189749	0	Singlet	Adipocyte
VAT_2M	1755.3	1207	0.3969026	6	Singlet	Neurons
VAT_2M	1373.1	1031	1.9093592	2	Singlet	FAPs
VAT_2M	3860.8	2010	1.0203568	0	Singlet	Adipocyte
VAT_2M	2579.1	1484	3.7842165	0	Singlet	Adipocyte
VAT_2M	2078.7	1353	0.2668802	2	Singlet	FAPs
VAT_2M	2541.9	1492	0.323176	0	Singlet	Adipocyte
VAT_2M	2450.1	1444	0.1018294	0	Singlet	Adipocyte
VAT_2M	1581	1111	0.2277327	6	Singlet	Neurons
VAT_2M	3495	1988	2.1661768	5	Singlet	Immune_cells
VAT_2M	2909.7	1713	0.1149265	3	Singlet	FAPs
VAT_2M	1806	1123	2.7679418	2	Singlet	FAPs
VAT_2M	1433.5	1114	0.3218185	7	Singlet	Endothelial_cells
VAT_2M	1103.5	838	3.7729031	2	Singlet	FAPs
VAT_2M	1431.2	1204	1.3459958	6	Singlet	Neurons
VAT_2M	2079.6	1298	0.7033284	0	Singlet	Adipocyte
VAT_2M	2044.6	1306	0.9053406	3	Singlet	FAPs
VAT_2M	4300.7	2305	0.5826696	2	Singlet	FAPs
VAT_2M	5275.7	2265	0.1782064	0	Singlet	Adipocyte
VAT_2M	8295.9	3099	0.0198938	0	Singlet	Adipocyte
VAT_2M	1047.7	833	0.5857612	7	Singlet	Endothelial_cells
VAT_2M	5254.5	2294	0.0936796	0	Singlet	Adipocyte
VAT_2M	5184.1	2346	0.0356646	0	Singlet	Adipocyte
VAT_2M	4138.4	2006	0.0502632	0	Singlet	Adipocyte
VAT_2M	1778.2	1127	2.948396	0	Singlet	Adipocyte
VAT_2M	2009.7	1277	0.1519644	0	Singlet	Adipocyte
VAT_2M	1326	1003	0.862293	2	Singlet	FAPs
VAT_2M	1378.2	1182	1.9852869	6	Singlet	Neurons
VAT_2M	1933.5	1201	0.2058061	8	Singlet	Mesothelial

VAT_2M	1212.1	976	1.7999766	6	Singlet	Neurons
VAT_2M	1405.3	1022	1.4454923	2	Singlet	FAPs
VAT_2M	1213.4	1051	1.5149539	6	Singlet	Neurons
VAT_2M	5123.7	2319	0	0	Singlet	Adipocyte
VAT_2M	3644.9	1764	0.0275565	11	Singlet	Smooth_muscle_cells
VAT_2M	2800.7	1521	0.1272312	0	Singlet	Adipocyte
VAT_2M	3274.9	1617	0.2929924	0	Singlet	Adipocyte
VAT_2M	1746.3	1126	0.5709684	6	Singlet	Neurons
VAT_2M	1627.4	1010	0.3282446	0	Singlet	Adipocyte
VAT_2M	1185.5	963	2.7283037	11	Singlet	Smooth_muscle_cells
VAT_2M	2161.8	1315	0.1427942	0	Singlet	Adipocyte
VAT_2M	7388.1	2872	0.0855416	0	Singlet	Adipocyte
VAT_2M	1335.4	949	1.9578724	2	Singlet	FAPs
VAT_2M	4305.5	2133	0.0940927	0	Singlet	Adipocyte
VAT_2M	2198.7	1312	0.5085555	0	Singlet	Adipocyte
VAT_2M	4321.5	2074	0.0264353	0	Singlet	Adipocyte
VAT_2M	1942.4	1158	0.5988899	0	Singlet	Adipocyte
VAT_2M	3193.4	1630	0.3441122	0	Singlet	Adipocyte
VAT_2M	4533.5	2110	0.3662576	0	Singlet	Adipocyte
VAT_2M	1108.6	884	4.08112	6	Singlet	Neurons
VAT_2M	11415	3676	0.1859695	0	Singlet	Adipocyte
VAT_2M	11630	3501	0.0397762	0	Singlet	Adipocyte
VAT_2M	8112	3130	0.1333939	0	Singlet	Adipocyte
VAT_2M	1181.9	883	0.3259123	2	Singlet	FAPs
VAT_2M	2598.7	1494	0.3336783	3	Singlet	FAPs
VAT_2M	1404	1049	0.2552189	2	Singlet	FAPs
VAT_2M	1897.2	1193	0.7026952	0	Singlet	Adipocyte
VAT_2M	2521.3	1441	1.2967775	2	Singlet	FAPs
VAT_2M	3867	2032	0.2066936	0	Singlet	Adipocyte
VAT_2M	3030.3	1716	0.0522558	0	Singlet	Adipocyte
VAT_2M	1029.5	819	0.205826	3	Singlet	FAPs
VAT_2M	5311.2	2714	0.7619975	2	Singlet	FAPs
VAT_2M	6370.4	2456	0.0560402	0	Singlet	Adipocyte
VAT_2M	4347.1	2188	1.9688615	2	Singlet	FAPs
VAT_2M	11195	3352	0	0	Singlet	Adipocyte
VAT_2M	10358	3463	0.4938621	0	Singlet	Adipocyte
VAT_2M	2023.8	1412	0.5396638	2	Singlet	FAPs
VAT_2M	7310.7	2692	0.0202741	0	Singlet	Adipocyte
VAT_2M	2077.8	1427	2.7015127	2	Singlet	FAPs
VAT_2M	1172.5	912	1.8077838	3	Singlet	FAPs
VAT_2M	9226.6	3400	0.0169489	0	Singlet	Adipocyte
VAT_2M	2139.3	1378	2.0760815	2	Singlet	FAPs
VAT_2M	2302.4	1339	9.5221017	6	Singlet	Neurons
VAT_2M	2302.9	1590	3.0164044	3	Singlet	FAPs
VAT_2M	1116.7	861	1.6975031	6	Singlet	Neurons
VAT_2M	2149.4	1337	0.1444773	0	Singlet	Adipocyte
VAT_2M	6849.5	2787	0.0760934	0	Singlet	Adipocyte
VAT_2M	7615.6	3101	0.3187352	0	Singlet	Adipocyte
VAT_2M	1165.5	837	1.2525269	3	Singlet	FAPs
VAT_2M	2275.3	1500	0.7900705	2	Singlet	FAPs
VAT_2M	2364.6	1546	0.6697247	5	Singlet	Immune_cells

VAT_2M	3924.6	1933	0.7345452	0	Singlet	Adipocyte
VAT_2M	2214.5	1184	0	0	Singlet	Adipocyte
VAT_2M	1316.2	945	1.6939874	6	Singlet	Neurons
VAT_2M	1086.5	862	0.7950916	6	Singlet	Neurons
VAT_2M	2494.9	1478	0.588828	6	Singlet	Neurons
VAT_2M	1363.8	972	1.1399474	2	Singlet	FAPs
VAT_2M	1112.6	822	3.5998694	0	Singlet	Adipocyte
VAT_2M	1140.2	883	3.823373	2	Singlet	FAPs
VAT_2M	5090.4	2167	0.0399145	0	Singlet	Adipocyte
VAT_2M	1123.6	963	2.8010204	6	Singlet	Neurons
VAT_2M	1431.5	982	0.2389924	3	Singlet	FAPs
VAT_2M	2726.5	1506	0	0	Singlet	Adipocyte
VAT_2M	1156.5	818	2.386503	2	Singlet	FAPs
VAT_2M	2070.1	1303	0.1320396	0	Singlet	Adipocyte
VAT_2M	1123.6	931	1.0621164	6	Singlet	Neurons
VAT_2M	2018.4	1245	0.2723438	3	Singlet	FAPs
VAT_2M	2658.6	1843	1.2677398	2	Singlet	FAPs
VAT_2M	1398	1062	0.5281961	2	Singlet	FAPs
VAT_2M	2852.5	1613	0.0935498	0	Singlet	Adipocyte
VAT_2M	3540.2	2082	0.2091267	2	Singlet	FAPs
VAT_2M	8741.2	3002	0.4593913	0	Singlet	Adipocyte
VAT_2M	1033.5	886	0.9380817	0	Singlet	Adipocyte
VAT_2M	2929.8	1711	0.0726167	0	Singlet	Adipocyte
VAT_2M	1475.3	1065	0.954494	6	Singlet	Neurons
VAT_2M	5926.1	2484	0.0948086	0	Singlet	Adipocyte
VAT_2M	5509.2	2458	1.3679833	0	Singlet	Adipocyte
VAT_2M	1558.8	1033	0.4060673	3	Singlet	FAPs
VAT_2M	5366	2341	0.0989117	0	Singlet	Adipocyte
VAT_2M	4504.2	2232	0.1717515	0	Singlet	Adipocyte
VAT_2M	1340.5	1154	0.9265609	6	Singlet	Neurons
VAT_2M	1756.4	1333	1.1472678	11	Singlet	Smooth_muscle_cells
VAT_2M	1747.9	1185	1.2393976	3	Singlet	FAPs
VAT_2M	1409	1083	0.8730742	2	Singlet	FAPs
VAT_2M	4170.3	2155	0.0805128	0	Singlet	Adipocyte
VAT_2M	4246.3	2171	0.3309957	0	Singlet	Adipocyte
VAT_2M	2062.2	1407	0.3876634	2	Singlet	FAPs
VAT_2M	6159.3	2612	0.3466852	0	Singlet	Adipocyte
VAT_2M	1027.9	792	3.9618052	9	Singlet	Mesothelial
VAT_2M	1039.7	909	0.2021407	6	Singlet	Neurons
VAT_2M	5080.8	2358	0	0	Singlet	Adipocyte
VAT_2M	2190.1	1461	1.13695	6	Singlet	Neurons
VAT_2M	3882.1	1965	0.2400039	0	Singlet	Adipocyte
VAT_2M	4826.6	2346	0.0392992	0	Singlet	Adipocyte
VAT_2M	1179.5	938	0.2565514	8	Singlet	Mesothelial
VAT_2M	1864.6	1362	0.652546	3	Singlet	FAPs
VAT_2M	1357.7	899	5.2573188	7	Singlet	Endothelial_cells
VAT_2M	4709.4	2161	0.2996	0	Singlet	Adipocyte
VAT_2M	4657.3	2178	0	0	Singlet	Adipocyte
VAT_2M	1585.1	1211	1.3675906	2	Singlet	FAPs
VAT_2M	8719.4	2813	0.001497	0	Singlet	Adipocyte
VAT_2M	4617.3	2209	0.0534461	0	Singlet	Adipocyte

VAT_2M	1088.1	907	0.2469118	6	Singlet	Neurons
VAT_2M	1554.9	1212	3.6366176	2	Singlet	FAPs
VAT_2M	3685.1	1950	0.2767934	0	Singlet	Adipocyte
VAT_2M	6429.5	2725	0.0234519	0	Singlet	Adipocyte
VAT_2M	3877.2	2186	1.4146596	3	Singlet	FAPs
VAT_2M	1917.6	1321	0.8133165	2	Singlet	FAPs
VAT_2M	3038.6	1814	0.1413646	3	Singlet	FAPs
VAT_2M	7891.5	2890	0.0310388	0	Singlet	Adipocyte
VAT_2M	1020	796	1.8221169	3	Singlet	FAPs
VAT_2M	4631.1	2269	0.1613876	0	Singlet	Adipocyte
VAT_2M	1551.9	1200	1.9839684	5	Singlet	Immune_cells
VAT_2M	3021.5	1609	0.0718483	0	Singlet	Adipocyte
VAT_2M	1020.1	857	0.4086624	6	Singlet	Neurons
VAT_2M	1105.2	842	0.3700933	3	Singlet	FAPs
VAT_2M	2994.3	1738	0.0223117	0	Singlet	Adipocyte
VAT_2M	2591.3	1453	0.1623997	0	Singlet	Adipocyte
VAT_2M	1880.7	1232	0.4018106	0	Singlet	Adipocyte
VAT_2M	2200.1	1370	0.138556	2	Singlet	FAPs
VAT_2M	1370.8	1072	1.7195909	6	Singlet	Neurons
VAT_2M	1711.6	1151	0.1533086	7	Singlet	Endothelial_cells
VAT_2M	2632.9	1630	0.7579797	2	Singlet	FAPs
VAT_2M	6005.1	2583	0.0411634	0	Singlet	Adipocyte
VAT_2M	6774.9	2713	0.0115142	0	Singlet	Adipocyte
VAT_2M	8638.8	3181	0.0400887	0	Singlet	Adipocyte
VAT_2M	1404.9	1090	0.5731978	6	Singlet	Neurons
VAT_2M	1999.7	1288	2.3264497	5	Singlet	Immune_cells
VAT_2M	9595.4	3220	0.0315617	0	Singlet	Adipocyte
VAT_2M	7382.9	2824	0.0361018	0	Singlet	Adipocyte
VAT_2M	1255.4	849	0.3120036	5	Singlet	Immune_cells
VAT_2M	2497.6	1596	0.6664808	2	Singlet	FAPs
VAT_2M	2756.2	1644	0.3798128	0	Singlet	Adipocyte
VAT_2M	8372.1	3068	0.0974931	0	Singlet	Adipocyte
VAT_2M	3125	1644	0.2813186	0	Singlet	Adipocyte
VAT_2M	6823.2	2730	0.8731766	0	Singlet	Adipocyte
VAT_2M	2205.8	1298	2.2369908	11	Singlet	Smooth_muscle_cells
VAT_2M	1366.5	995	0.923332	6	Singlet	Neurons
VAT_2M	3155.9	1838	2.4181723	3	Singlet	FAPs
VAT_2M	1624	1045	4.8217885	5	Singlet	Immune_cells
VAT_2M	4188.6	2065	0.0769324	0	Singlet	Adipocyte
VAT_2M	1408.7	925	0.0132061	7	Singlet	Endothelial_cells
VAT_2M	2349.6	1437	0.0787989	0	Singlet	Adipocyte
VAT_2M	8610.5	2979	0.0146578	0	Singlet	Adipocyte
VAT_2M	2147.7	1313	1.6394232	0	Singlet	Adipocyte
VAT_2M	3080.2	1589	0.0225982	0	Singlet	Adipocyte
VAT_2M	7235.6	2923	0.0020199	0	Singlet	Adipocyte
VAT_2M	6017.9	2704	0.0084039	0	Singlet	Adipocyte
VAT_2M	1737.6	1217	0.61588	2	Singlet	FAPs
VAT_2M	2251.2	1431	4.0337793	5	Singlet	Immune_cells
VAT_2M	3267.3	1857	2.3411114	5	Singlet	Immune_cells
VAT_2M	2125.1	1248	0.2398131	0	Singlet	Adipocyte
VAT_2M	2436.2	1440	0.4614257	0	Singlet	Adipocyte

VAT_2M	2021.3	1314	0.6337726	0	Singlet	Adipocyte
VAT_2M	1480.9	1141	0.8979377	3	Singlet	FAPs
VAT_2M	5007.8	2302	0.0097904	0	Singlet	Adipocyte
VAT_2M	4096.3	2067	0.2659317	0	Singlet	Adipocyte
VAT_2M	1016.1	745	4.3824262	7	Singlet	Endothelial_cells
VAT_2M	5984	2527	0.0651347	0	Singlet	Adipocyte
VAT_2M	5819.1	2717	6.0594675	7	Singlet	Endothelial_cells
VAT_2M	1400.3	1106	1.2190103	11	Singlet	Smooth_muscle_cells
VAT_2M	1000.8	741	2.1025507	2	Singlet	FAPs
VAT_2M	2474.3	1549	0.0139086	0	Singlet	Adipocyte
VAT_2M	1266.7	947	0.443162	0	Singlet	Adipocyte
VAT_2M	1229.8	1000	0.2370075	5	Singlet	Immune_cells
VAT_2M	2715	1460	0.0282743	0	Singlet	Adipocyte
VAT_2M	2143	1379	0.3110373	6	Singlet	Neurons
VAT_2M	1614.4	1052	0.6768745	3	Singlet	FAPs
VAT_2M	4219.1	2096	0	0	Singlet	Adipocyte
VAT_2M	1071.5	818	0.6577934	9	Singlet	Mesothelial
VAT_2M	2062	1464	1.0892134	2	Singlet	FAPs
VAT_2M	5222.8	2236	0.0498927	0	Singlet	Adipocyte
VAT_2M	1191.1	1006	0.0724929	6	Singlet	Neurons
VAT_2M	1517.5	1153	1.4959877	2	Singlet	FAPs
VAT_2M	6109.1	2729	0.0966073	0	Singlet	Adipocyte
VAT_2M	3526.9	1866	0.106756	0	Singlet	Adipocyte
VAT_2M	1839.6	1448	1.6618729	7	Singlet	Endothelial_cells
VAT_2M	4436	2216	0.0568272	0	Singlet	Adipocyte
VAT_2M	2400.9	1571	0.9758154	5	Singlet	Immune_cells
VAT_2M	6948.1	2810	0.0055796	0	Singlet	Adipocyte
VAT_2M	2453.3	1646	0.2447278	2	Singlet	FAPs
VAT_2M	4478.4	2289	0.2208553	2	Singlet	FAPs
VAT_2M	1704.5	1132	2.1198657	3	Singlet	FAPs
VAT_2M	2756.9	1720	0.682603	5	Singlet	Immune_cells
VAT_2M	4126.2	1916	0.014334	0	Singlet	Adipocyte
VAT_2M	2412.2	1478	0.7225808	2	Singlet	FAPs
VAT_2M	2189.6	1397	0.6421681	6	Singlet	Neurons
VAT_2M	6954	2586	0.0112272	0	Singlet	Adipocyte
VAT_2M	1858.2	1351	0.4899006	6	Singlet	Neurons
VAT_2M	1578.7	1153	1.0730949	6	Singlet	Neurons
VAT_2M	3800.5	1986	0.0600765	0	Singlet	Adipocyte
VAT_2M	2885.2	1639	0.1685507	0	Singlet	Adipocyte
VAT_2M	1541.6	1076	1.947221	5	Singlet	Immune_cells
VAT_2M	1787.7	1128	0.0775379	0	Singlet	Adipocyte
VAT_2M	6195.9	2715	0.2026104	0	Singlet	Adipocyte
VAT_2M	1604.2	1216	1.8451508	2	Singlet	FAPs
VAT_2M	3496.2	1827	0.6349437	0	Singlet	Adipocyte
VAT_2M	1138.5	823	0.9676252	5	Singlet	Immune_cells
VAT_2M	2170.6	1397	2.9666863	2	Singlet	FAPs
VAT_2M	2308.1	1430	0.0915542	5	Singlet	Immune_cells
VAT_2M	2814.3	1602	1.283518	0	Singlet	Adipocyte
VAT_2M	1375.7	1180	0.56013	6	Singlet	Neurons
VAT_2M	1148.8	989	0.5080904	6	Singlet	Neurons
VAT_2M	8446.6	3115	0.9303537	0	Singlet	Adipocyte

VAT_2M	4779.7	2212	0.0515852	0	Singlet	Adipocyte
VAT_2M	6279.5	2636	0.0687374	0	Singlet	Adipocyte
VAT_2M	1587.4	966	2.0194873	0	Singlet	Adipocyte
VAT_2M	5746.4	2628	0.3000047	0	Singlet	Adipocyte
VAT_2M	1375.5	1128	0.4638324	8	Singlet	Mesothelial
VAT_2M	1587.4	1044	0.2946024	11	Singlet	Smooth_muscle_cells
VAT_2M	3920.3	2000	3.2119085	5	Singlet	Immune_cells
VAT_2M	1435	1024	0.6350637	2	Singlet	FAPs
VAT_2M	4373.8	1954	0.4935938	0	Singlet	Adipocyte
VAT_2M	2763.9	1643	2.7056626	2	Singlet	FAPs
VAT_2M	1070.6	872	2.5390407	6	Singlet	Neurons
VAT_2M	5447.9	2565	0.1291666	0	Singlet	Adipocyte
VAT_2M	4989.6	2398	0.7641073	3	Singlet	FAPs
VAT_2M	6056.7	2643	0.3213164	0	Singlet	Adipocyte
VAT_2M	4612.9	2153	0.6294277	0	Singlet	Adipocyte
VAT_2M	1144.3	890	0.5273982	6	Singlet	Neurons
VAT_2M	1609.2	1113	0.6170812	3	Singlet	FAPs
VAT_2M	1087.2	817	1.4233645	6	Singlet	Neurons
VAT_2M	1269.4	944	1.0712002	2	Singlet	FAPs
VAT_2M	1935.9	1208	0.8639588	3	Singlet	FAPs
VAT_2M	6530.8	2538	0.0463722	0	Singlet	Adipocyte
VAT_2M	5487.3	2330	0.2652257	0	Singlet	Adipocyte
VAT_2M	1217.1	960	1.3799315	4	Singlet	Immune_cells
VAT_2M	1024.2	819	2.4458499	3	Singlet	FAPs
VAT_2M	2904.2	1674	0.1602293	0	Singlet	Adipocyte
VAT_2M	1647.3	1244	2.1397845	6	Singlet	Neurons
VAT_2M	2334.2	1332	0.1363846	0	Singlet	Adipocyte
VAT_2M	5916.9	2442	0.0331122	0	Singlet	Adipocyte
VAT_2M	1866.7	1466	0.7040351	5	Singlet	Immune_cells
VAT_2M	1956.2	1210	1.0326618	0	Singlet	Adipocyte
VAT_2M	1642.5	1005	0.1402754	0	Singlet	Adipocyte
VAT_2M	1886.8	1213	3.9709756	9	Singlet	Mesothelial
VAT_2M	1813.8	1228	2.7445026	1	Singlet	Immune_cells
VAT_2M	5371.7	2310	0.0337112	0	Singlet	Adipocyte
VAT_2M	1049.7	644	3.9021127	5	Singlet	Immune_cells
VAT_2M	1197.8	863	0.8309719	5	Singlet	Immune_cells
VAT_2M	4438.5	1818	4.648945	0	Singlet	Adipocyte
VAT_2M	10112	3351	0.1224944	0	Singlet	Adipocyte
VAT_2M	6955.1	2586	0.0021427	0	Singlet	Adipocyte
VAT_2M	13192	3949	0.0070897	0	Singlet	Adipocyte
VAT_2M	9593.8	3257	0	0	Singlet	Adipocyte
VAT_2M	7399.4	2888	0.0493143	0	Singlet	Adipocyte
VAT_2M	2853.9	1535	0.0808614	0	Singlet	Adipocyte
VAT_2M	5926.6	2560	0.0534996	0	Singlet	Adipocyte
VAT_2M	1657	1154	1.1653882	3	Singlet	FAPs
VAT_2M	1489.5	1092	0.9650509	3	Singlet	FAPs
VAT_2M	2424.5	1242	2.3781909	2	Singlet	FAPs
VAT_2M	2299.7	1528	1.0486629	5	Singlet	Immune_cells
VAT_2M	1046.8	795	1.9201508	2	Singlet	FAPs
VAT_2M	1064.4	824	0.2171852	3	Singlet	FAPs
VAT_2M	1978.4	1227	0	0	Singlet	Adipocyte

VAT_2M	1625	1181	0.8849894	7	Singlet	Endothelial_cells
VAT_2M	2347.2	1499	1.4520052	2	Singlet	FAPs
VAT_2M	2104.6	1240	0.7031831	7	Singlet	Endothelial_cells
VAT_2M	4023.7	2033	1.1800013	11	Singlet	Smooth_muscle_cells
VAT_2M	1107.3	844	0.8169241	4	Singlet	Immune_cells
VAT_2M	3514	1810	0.0654895	0	Singlet	Adipocyte
VAT_2M	2308.3	1522	0.9075263	2	Singlet	FAPs
VAT_2M	6280.3	2780	0.0056912	0	Singlet	Adipocyte
VAT_2M	2172.6	1360	0.7226784	3	Singlet	FAPs
VAT_2M	2233.9	1362	0.1383489	0	Singlet	Adipocyte
VAT_2M	2214.1	1385	2.7154321	5	Singlet	Immune_cells
VAT_2M	7878.4	2942	0.0078993	0	Singlet	Adipocyte
VAT_2M	1180.3	1029	0.2415612	6	Singlet	Neurons
VAT_2M	1419.8	1049	1.8281837	2	Singlet	FAPs
VAT_2M	3545	1963	0	0	Singlet	Adipocyte
VAT_2M	1762.2	1131	0.8883073	0	Singlet	Adipocyte
VAT_2M	3356.6	1919	1.0026788	3	Singlet	FAPs
VAT_2M	2651.8	1753	0.718154	2	Singlet	FAPs
VAT_2M	3458.4	1700	0.329493	0	Singlet	Adipocyte
VAT_2M	1243.2	985	0.9297538	4	Singlet	Immune_cells
VAT_2M	8248.7	3004	0.0297796	0	Singlet	Adipocyte
VAT_2M	4494.6	2029	0.0111167	0	Singlet	Adipocyte
VAT_2M	3790.5	2160	0.1641737	0	Singlet	Adipocyte
VAT_2M	3483.4	1853	0.6626309	0	Singlet	Adipocyte
VAT_2M	1507.4	1097	2.3262121	3	Singlet	FAPs
VAT_2M	3392.2	1876	0.3495843	0	Singlet	Adipocyte
VAT_2M	4061.7	2006	0.2429349	0	Singlet	Adipocyte
VAT_2M	2798.7	1341	3.707229	5	Singlet	Immune_cells
VAT_2M	2327.4	1280	0.0074006	0	Singlet	Adipocyte
VAT_2M	3083.7	1940	0.484874	6	Singlet	Neurons
VAT_2M	3316	1670	0.0182937	0	Singlet	Adipocyte
VAT_2M	6172.7	2664	0.0375506	0	Singlet	Adipocyte
VAT_2M	3221	1353	6.1241522	3	Singlet	FAPs
VAT_2M	3235.2	1733	0.2104108	0	Singlet	Adipocyte
VAT_2M	1058.8	673	10.872662	9	Singlet	Mesothelial
VAT_2M	2116.2	1248	0.2961047	0	Singlet	Adipocyte
VAT_2M	2488.5	1677	0.8940033	3	Singlet	FAPs
VAT_2M	1713.5	1140	0.1735579	5	Singlet	Immune_cells
VAT_2M	5869.8	2506	0.0549756	0	Singlet	Adipocyte
VAT_2M	1587.2	1147	0.5281246	6	Singlet	Neurons
VAT_2M	7677.3	2844	0.0122643	0	Singlet	Adipocyte
VAT_2M	1299.3	948	0.0070206	6	Singlet	Neurons
VAT_2M	6557.2	2548	0.0016461	0	Singlet	Adipocyte
VAT_2M	1805.1	1117	1.8477583	5	Singlet	Immune_cells
VAT_2M	2146.6	1405	0.3582179	0	Singlet	Adipocyte
VAT_2M	3395.1	1973	0.7047806	0	Singlet	Adipocyte
VAT_2M	4138.8	2049	0.037452	0	Singlet	Adipocyte
VAT_2M	1632.6	1131	0.1901661	3	Singlet	FAPs
VAT_2M	1204.5	802	2.6045002	5	Singlet	Immune_cells
VAT_2M	1593.3	1007	0.0104282	7	Singlet	Endothelial_cells
VAT_2M	6114.3	2509	0.3959872	0	Singlet	Adipocyte

VAT_2M	2945	1817	0.2813397	2	Singlet	FAPs
VAT_2M	1314.7	931	0.4253342	3	Singlet	FAPs
VAT_2M	7226	2914	0.4330641	0	Singlet	Adipocyte
VAT_2M	6138.6	2505	0.0209969	0	Singlet	Adipocyte
VAT_2M	3101	1595	0	0	Singlet	Adipocyte
VAT_2M	11546	3717	2.6589517	0	Singlet	Adipocyte
VAT_2M	1626	1096	0.0268253	6	Singlet	Neurons
VAT_2M	2811.2	1683	0.0625399	0	Singlet	Adipocyte
VAT_2M	2536	1485	0.0988403	0	Singlet	Adipocyte
VAT_2M	1193.6	1000	0.3233107	6	Singlet	Neurons
VAT_2M	7214.1	2834	0.0292482	0	Singlet	Adipocyte
VAT_2M	1090.9	814	3.8323883	1	Singlet	Immune_cells
VAT_2M	1506.3	1166	0.9347213	7	Singlet	Endothelial_cells
VAT_2M	2194.3	1421	0.3255981	3	Singlet	FAPs
VAT_2M	9641.1	3345	0.3627893	0	Singlet	Adipocyte
VAT_2M	5383.2	2404	0.1166113	0	Singlet	Adipocyte
VAT_2M	2982.2	1715	0.3264951	3	Singlet	FAPs
VAT_2M	1883.6	1273	0.1803063	3	Singlet	FAPs
VAT_2M	1030.8	777	0.4592472	2	Singlet	FAPs
VAT_2M	3099.2	1772	0.042556	2	Singlet	FAPs
VAT_2M	2598	1674	0.3378755	0	Singlet	Adipocyte
VAT_2M	1021.2	814	3.7278311	2	Singlet	FAPs
VAT_2M	3414	1749	0	0	Singlet	Adipocyte
VAT_2M	3382.5	1782	0.7989945	0	Singlet	Adipocyte
VAT_2M	5004.9	2314	0.3554656	0	Singlet	Adipocyte
VAT_2M	2187.8	1374	1.9755169	2	Singlet	FAPs
VAT_2M	4242.1	2102	0.1568075	0	Singlet	Adipocyte
VAT_2M	7761.6	2998	0.0028132	0	Singlet	Adipocyte
VAT_2M	2289.1	1437	2.2635027	3	Singlet	FAPs
VAT_2M	6462.7	2710	0.0475835	0	Singlet	Adipocyte
VAT_2M	1263.1	872	1.014744	2	Singlet	FAPs
VAT_2M	1023.2	811	0.4168822	6	Singlet	Neurons
VAT_2M	1929	1258	1.6120637	2	Singlet	FAPs
VAT_2M	1060.4	852	2.2751504	5	Singlet	Immune_cells
VAT_2M	3768.1	2017	1.4416286	3	Singlet	FAPs
VAT_2M	3021.3	1582	0.1359799	0	Singlet	Adipocyte
VAT_2M	2100.9	1459	2.4411149	2	Singlet	FAPs
VAT_2M	1610.8	1115	0.1384153	3	Singlet	FAPs
VAT_2M	3211.3	1832	0.1086355	3	Singlet	FAPs
VAT_2M	2172	1315	1.0518382	0	Singlet	Adipocyte
VAT_2M	7546.4	2821	0.0549427	0	Singlet	Adipocyte
VAT_2M	2534.5	1719	0.7224385	2	Singlet	FAPs
VAT_2M	2138.5	1356	0.276354	3	Singlet	FAPs
VAT_2M	1718.4	1124	0	3	Singlet	FAPs
VAT_2M	2976.8	1645	1.1767521	5	Singlet	Immune_cells
VAT_2M	1283.8	916	0.565661	7	Singlet	Endothelial_cells
VAT_2M	1437.9	1022	1.6146989	5	Singlet	Immune_cells
VAT_2M	1124	935	0.377774	3	Singlet	FAPs
VAT_2M	1414	1136	0.5167335	4	Singlet	Immune_cells
VAT_2M	7916.7	2938	0.6181176	0	Singlet	Adipocyte
VAT_2M	3146.7	1741	1.3980807	0	Singlet	Adipocyte

VAT_2M	8520.7	3082	0.0016942	0	Singlet	Adipocyte
VAT_2M	3174.1	1726	1.55958	0	Singlet	Adipocyte
VAT_2M	1923.4	1249	2.1813738	6	Singlet	Neurons
VAT_2M	5982.9	2849	0.8746378	3	Singlet	FAPs
VAT_2M	10737	3486	0.1560305	0	Singlet	Adipocyte
VAT_2M	1471.4	1167	2.1714609	2	Singlet	FAPs
VAT_2M	3534.2	2059	1.3713687	3	Singlet	FAPs
VAT_2M	4232.4	2050	0.9324208	0	Singlet	Adipocyte
VAT_2M	1762.2	1176	1.3251602	6	Singlet	Neurons
VAT_2M	3706.6	1803	0.0295095	0	Singlet	Adipocyte
VAT_2M	2328	1470	0.9040497	2	Singlet	FAPs
VAT_2M	1162.4	849	2.0527143	6	Singlet	Neurons
VAT_2M	13336	3855	0.0439975	0	Singlet	Adipocyte
VAT_2M	1789.9	1169	2.8187898	2	Singlet	FAPs
VAT_2M	8746.5	3114	0	0	Singlet	Adipocyte
VAT_2M	10218	3410	0.0110496	0	Singlet	Adipocyte
VAT_2M	4610	2358	0.0238489	0	Singlet	Adipocyte
VAT_2M	2849.6	1714	0.7293608	0	Singlet	Adipocyte
VAT_2M	3156	1720	0.0426387	0	Singlet	Adipocyte
VAT_2M	8035.7	3149	0.0259877	0	Singlet	Adipocyte
VAT_2M	2805.4	1634	1.8901562	0	Singlet	Adipocyte
VAT_2M	2128.5	1319	0.1546564	0	Singlet	Adipocyte
VAT_2M	2962	1567	1.411195	0	Singlet	Adipocyte
VAT_2M	1148.1	832	1.7017021	7	Singlet	Endothelial_cells
VAT_2M	1417.9	1165	0.688961	6	Singlet	Neurons
VAT_2M	5854.9	2428	0	0	Singlet	Adipocyte
VAT_2M	1273.5	1000	1.9717116	6	Singlet	Neurons
VAT_2M	2445.5	1402	0.2479855	0	Singlet	Adipocyte
VAT_2M	1575.7	1206	2.3518048	2	Singlet	FAPs
VAT_2M	2950.8	1660	0.0232606	0	Singlet	Adipocyte
VAT_2M	1756.5	1175	0.0157293	0	Singlet	Adipocyte
VAT_2M	5712.2	2443	0.0692037	0	Singlet	Adipocyte
VAT_2M	1236	896	0.3835364	3	Singlet	FAPs
VAT_2M	2331	1577	0.325677	2	Singlet	FAPs
VAT_2M	2624.4	1845	0.8474282	2	Singlet	FAPs
VAT_2M	3323.5	1765	0.4420278	0	Singlet	Adipocyte
VAT_2M	7624.9	2968	0.0189798	0	Singlet	Adipocyte
VAT_2M	1473.5	1045	0.8947221	2	Singlet	FAPs
VAT_2M	1045	844	0.686527	3	Singlet	FAPs
VAT_2M	6293.1	2831	0.0886194	0	Singlet	Adipocyte
VAT_2M	1972.1	1224	0.309665	0	Singlet	Adipocyte
VAT_2M	1734.3	1247	0.1600476	7	Singlet	Endothelial_cells
VAT_2M	7790.8	3151	0.3144134	0	Singlet	Adipocyte
VAT_2M	1862.8	1372	0.4444688	3	Singlet	FAPs
VAT_2M	2761.6	1787	2.6936826	5	Singlet	Immune_cells
VAT_2M	4540.1	2042	0.044651	0	Singlet	Adipocyte
VAT_2M	6741.9	2711	0.0351035	0	Singlet	Adipocyte
VAT_2M	1860.8	1156	1.5625681	0	Singlet	Adipocyte
VAT_2M	2138.1	1485	0.9611738	2	Singlet	FAPs
VAT_2M	5126.2	2356	0.0289824	0	Singlet	Adipocyte
VAT_2M	1075.7	754	0.1653337	3	Singlet	FAPs

VAT_2M	1572	1039	0.1009794	0	Singlet	Adipocyte
VAT_2M	3546.3	1761	0.1395006	0	Singlet	Adipocyte
VAT_2M	2093.7	1209	0.091488	0	Singlet	Adipocyte
VAT_2M	2039.2	1252	0.5866792	0	Singlet	Adipocyte
VAT_2M	1318.7	1040	0.9351979	3	Singlet	FAPs
VAT_2M	6557.4	2713	0.07332	0	Singlet	Adipocyte
VAT_2M	5075.8	2280	0	0	Singlet	Adipocyte
VAT_2M	3337.6	1824	0.361207	0	Singlet	Adipocyte
VAT_2M	3107	1693	2.9317688	2	Singlet	FAPs
VAT_2M	3629	2093	0.0843506	0	Singlet	Adipocyte
VAT_2M	1883.9	1543	0.7744602	6	Singlet	Neurons
VAT_2M	1906.6	1423	2.574992	4	Singlet	Immune_cells
VAT_2M	2363	1555	0.157746	7	Singlet	Endothelial_cells
VAT_2M	1967.7	1431	0.7331266	10	Singlet	Immune_cells
VAT_2M	2936.9	1630	0.2586557	0	Singlet	Adipocyte
VAT_2M	1994.4	1271	0.9963283	2	Singlet	FAPs
VAT_2M	1823.9	1149	1.3729803	5	Singlet	Immune_cells
VAT_2M	1195.4	847	0.2852024	2	Singlet	FAPs
VAT_2M	3393.2	1955	0.0609759	0	Singlet	Adipocyte
VAT_2M	2958.4	1641	0.1260596	0	Singlet	Adipocyte
VAT_2M	1270.7	972	0.6757752	5	Singlet	Immune_cells
VAT_2M	2123	1468	1.9641928	2	Singlet	FAPs
VAT_2M	3257.5	1692	0.0374128	0	Singlet	Adipocyte
VAT_2M	1174	974	1.7538161	6	Singlet	Neurons
VAT_2M	1102.5	945	0.6024668	10	Singlet	Immune_cells
VAT_2M	1932.9	1224	0.1939148	0	Singlet	Adipocyte
VAT_2M	5199.4	2368	0.0443784	0	Singlet	Adipocyte
VAT_2M	2784.6	1700	0.0563003	0	Singlet	Adipocyte
VAT_2M	1491.6	934	2.8027589	2	Singlet	FAPs
VAT_2M	2386.1	1721	0.5027141	3	Singlet	FAPs
VAT_2M	1885	1334	3.7969663	5	Singlet	Immune_cells
VAT_2M	1651.7	1182	4.0712983	9	Singlet	Mesothelial
VAT_2M	5284.5	2317	0.9897507	0	Singlet	Adipocyte
VAT_2M	5351.2	2279	0.832471	0	Singlet	Adipocyte
VAT_2M	8947.8	3285	0.1190402	0	Singlet	Adipocyte
VAT_2M	1160	988	3.8714657	6	Singlet	Neurons
VAT_2M	1490.4	1161	4.4939685	5	Singlet	Immune_cells
VAT_2M	1873.9	1207	4.0965157	3	Singlet	FAPs
VAT_2M	2740.9	1746	0.4428118	3	Singlet	FAPs
VAT_2M	8832.5	3170	0	0	Singlet	Adipocyte
VAT_2M	6375.8	2554	0	0	Singlet	Adipocyte
VAT_2M	1087.5	816	1.9035119	3	Singlet	FAPs
VAT_2M	6171	2715	0.4189264	0	Singlet	Adipocyte
VAT_2M	1339.3	918	0.4197054	7	Singlet	Endothelial_cells
VAT_2M	1385.8	972	0.4764899	3	Singlet	FAPs
VAT_2M	12359	3483	0.0088149	0	Singlet	Adipocyte
VAT_2M	1822.4	1212	1.6767158	8	Singlet	Mesothelial
VAT_2M	8554.8	2956	0	0	Singlet	Adipocyte
VAT_2M	2225	1479	1.3538354	3	Singlet	FAPs
VAT_2M	4176.7	2118	0.0922311	0	Singlet	Adipocyte
VAT_2M	1753	1213	0.5808048	7	Singlet	Endothelial_cells

VAT_2M	1030.1	801	7.6401632	9	Singlet	Mesothelial
VAT_2M	1931.8	1325	0.0296156	6	Singlet	Neurons
VAT_2M	2670.8	1394	0.2666699	0	Singlet	Adipocyte
VAT_2M	5811.3	2528	0.0363416	0	Singlet	Adipocyte
VAT_2M	5671.5	2458	0.2238015	0	Singlet	Adipocyte
VAT_2M	1529.8	1103	1.3575427	3	Singlet	FAPs
VAT_2M	1007.4	843	0.4150387	7	Singlet	Endothelial_cells
VAT_2M	3378.8	1695	0.216583	0	Singlet	Adipocyte
VAT_2M	3953.5	1986	0.030792	0	Singlet	Adipocyte
VAT_2M	1690.9	1196	0.8023832	2	Singlet	FAPs
VAT_2M	1572.4	1132	1.6313674	9	Singlet	Mesothelial
VAT_2M	2042.2	1182	0.0381304	0	Singlet	Adipocyte
VAT_2M	7835.9	2906	0.0500008	0	Singlet	Adipocyte
VAT_2M	2099.4	1341	1.362972	3	Singlet	FAPs
VAT_2M	3621.9	1790	0.0958803	0	Singlet	Adipocyte
VAT_2M	6522.3	2589	0.0170311	0	Singlet	Adipocyte
VAT_2M	1245.8	998	4.6135667	2	Singlet	FAPs
VAT_2M	2557.2	1462	0.6781703	0	Singlet	Adipocyte
VAT_2M	1054.9	834	0.6631571	1	Singlet	Immune_cells
VAT_2M	2444.3	1509	0.0255226	11	Singlet	Smooth_muscle_cells
VAT_2M	7143.5	2781	0.0169809	0	Singlet	Adipocyte
VAT_2M	1808.8	1243	0.5807333	3	Singlet	FAPs
VAT_2M	1271	867	1.0021188	2	Singlet	FAPs
VAT_2M	3382.4	1775	0.0161152	0	Singlet	Adipocyte
VAT_2M	6657.5	2656	0.521542	0	Singlet	Adipocyte
VAT_2M	1802.8	1237	1.1447903	5	Singlet	Immune_cells
VAT_2M	1053.4	914	1.3951139	4	Singlet	Immune_cells
VAT_2M	1614.6	1126	0.9364509	6	Singlet	Neurons
VAT_2M	7852.3	3015	0.1838098	0	Singlet	Adipocyte
VAT_2M	1583.9	1081	3.7245098	5	Singlet	Immune_cells
VAT_2M	3967.7	1985	0.1441706	0	Singlet	Adipocyte
VAT_2M	1793.4	1185	0.3716668	2	Singlet	FAPs
VAT_2M	2648.8	1396	0.2086924	0	Singlet	Adipocyte
VAT_2M	1380.1	1003	3.1131667	2	Singlet	FAPs
VAT_2M	1098	829	3.4416399	6	Singlet	Neurons
VAT_2M	7071	2983	0.0902994	0	Singlet	Adipocyte
VAT_2M	6371.2	2621	0	0	Singlet	Adipocyte
VAT_2M	6550	2822	0.0392367	0	Singlet	Adipocyte
VAT_2M	4574	2252	0.0352452	0	Singlet	Adipocyte
VAT_2M	5391.3	2381	0.246908	0	Singlet	Adipocyte
VAT_2M	1681.3	1110	0.2355717	3	Singlet	FAPs
VAT_2M	2309.4	1379	0.6416444	3	Singlet	FAPs
VAT_2M	3479.6	1956	0.7007482	11	Singlet	Smooth_muscle_cells
VAT_2M	6049	2560	0	0	Singlet	Adipocyte
VAT_2M	7308.3	2870	0.0023277	0	Singlet	Adipocyte
VAT_2M	1120.9	891	1.3226791	5	Singlet	Immune_cells
VAT_2M	1527.7	1072	0.965146	6	Singlet	Neurons
VAT_2M	1426.5	977	0.0863166	5	Singlet	Immune_cells
VAT_2M	1300.9	891	2.8901031	5	Singlet	Immune_cells
VAT_2M	2653.2	1591	0.8780264	3	Singlet	FAPs
VAT_2M	1159.1	922	0.3967467	6	Singlet	Neurons

VAT_2M	4463.6	2359	5.2108973	7	Singlet	Endothelial_cells
VAT_2M	1994.8	1303	1.1341738	0	Singlet	Adipocyte
VAT_2M	3603.2	1855	8.6766775	7	Singlet	Endothelial_cells
VAT_2M	2026.9	1168	0.1944974	0	Singlet	Adipocyte
VAT_2M	1057.9	928	0.5787479	6	Singlet	Neurons
VAT_2M	1080.8	750	3.221024	2	Singlet	FAPs
VAT_2M	5396	2513	0.2585203	0	Singlet	Adipocyte
VAT_2M	2437.8	1603	1.2205346	5	Singlet	Immune_cells
VAT_2M	1947.2	1355	2.1041034	7	Singlet	Endothelial_cells
VAT_2M	2674.6	1748	3.7678725	2	Singlet	FAPs
VAT_2M	2109.6	1307	0.9329619	3	Singlet	FAPs
VAT_2M	3393.8	1723	0	0	Singlet	Adipocyte
VAT_2M	2906.9	1662	0.0216612	3	Singlet	FAPs
VAT_2M	1665.5	1326	1.4192047	2	Singlet	FAPs
VAT_2M	3113.1	1786	0.4173072	0	Singlet	Adipocyte
VAT_2M	2038.8	1360	1.2564735	2	Singlet	FAPs
VAT_2M	1403.1	1098	0.1315683	3	Singlet	FAPs
VAT_2M	1845.3	1252	1.7340054	2	Singlet	FAPs
VAT_2M	1229.4	932	0.5548358	4	Singlet	Immune_cells
VAT_2M	1212.6	986	2.1128044	11	Singlet	Smooth_muscle_cells
VAT_2M	2648.9	1545	0.2388859	0	Singlet	Adipocyte
VAT_2M	1451.8	879	4.8313396	0	Singlet	Adipocyte
VAT_2M	1075.8	788	1.7510738	5	Singlet	Immune_cells
VAT_2M	3192.3	1681	0.186688	0	Singlet	Adipocyte
VAT_2M	1133.9	977	1.0455594	6	Singlet	Neurons
VAT_2M	1205.3	979	1.5324338	5	Singlet	Immune_cells
VAT_2M	1841.9	1396	2.0091469	2	Singlet	FAPs
VAT_2M	1209.1	919	0.3142549	3	Singlet	FAPs
VAT_2M	2004.2	1296	2.4313228	2	Singlet	FAPs
VAT_2M	3658.8	1912	0.4386705	0	Singlet	Adipocyte
VAT_2M	2076.4	1529	1.8370921	2	Singlet	FAPs
VAT_2M	1955.8	1168	0.271878	0	Singlet	Adipocyte
VAT_2M	2653	1713	0.0947984	2	Singlet	FAPs
VAT_2M	3383.6	1652	0.5762352	0	Singlet	Adipocyte
VAT_2M	2045.6	1356	0.6214384	6	Singlet	Neurons
VAT_2M	1240.2	924	1.4307911	2	Singlet	FAPs
VAT_2M	6619.4	2648	0.2077596	0	Singlet	Adipocyte
VAT_2M	5051.4	2174	0.0260792	0	Singlet	Adipocyte
VAT_2M	2419.4	1438	0.0466208	0	Singlet	Adipocyte
VAT_2M	1381.5	1143	0.565535	2	Singlet	FAPs
VAT_2M	1862.3	1311	0.3541135	3	Singlet	FAPs
VAT_2M	2818.5	1654	0.0506755	0	Singlet	Adipocyte
VAT_2M	1229.8	930	2.1565094	3	Singlet	FAPs
VAT_2M	1563.1	1118	1.3357383	2	Singlet	FAPs
VAT_2M	2255.9	1343	0.3477415	0	Singlet	Adipocyte
VAT_2M	5797.5	2563	0.0409619	0	Singlet	Adipocyte
VAT_2M	1106.4	777	0.6031564	3	Singlet	FAPs
VAT_2M	1153.4	965	1.709193	1	Singlet	Immune_cells
VAT_2M	3255.5	1767	0.1678646	0	Singlet	Adipocyte
VAT_2M	1176.7	800	1.7260746	7	Singlet	Endothelial_cells
VAT_2M	1149.5	947	2.0438656	6	Singlet	Neurons

VAT_2M	5231.7	2419	0.2529914	0	Singlet	Adipocyte
VAT_2M	1465	906	0.1832678	3	Singlet	FAPs
VAT_2M	2855.4	1604	0.8429667	0	Singlet	Adipocyte
VAT_2M	1208.5	886	0.7409843	5	Singlet	Immune_cells
VAT_2M	1656.6	1174	0.1236825	3	Singlet	FAPs
VAT_2M	1357.3	1209	0.1985001	6	Singlet	Neurons
VAT_2M	4831.7	2264	0.0734245	0	Singlet	Adipocyte
VAT_2M	5904.3	2528	0.0032574	0	Singlet	Adipocyte
VAT_2M	1062.6	920	2.4616534	1	Singlet	Immune_cells
VAT_2M	1766.6	1443	1.4086069	2	Singlet	FAPs
VAT_2M	1267.8	995	4.5877338	9	Singlet	Mesothelial
VAT_2M	2018.5	1511	1.1151683	2	Singlet	FAPs
VAT_2M	1239.7	939	2.1380625	5	Singlet	Immune_cells
VAT_2M	1064.8	827	1.689896	2	Singlet	FAPs
VAT_2M	2133.6	1434	0.5347439	6	Singlet	Neurons
VAT_2M	6402.4	2648	0.2043708	0	Singlet	Adipocyte
VAT_2M	11530	3679	0.0060889	0	Singlet	Adipocyte
VAT_2M	4360.1	2182	0	0	Singlet	Adipocyte
VAT_2M	9133	3099	0.2341087	0	Singlet	Adipocyte
VAT_2M	2750.8	1560	0.2978476	0	Singlet	Adipocyte
VAT_2M	1182.3	978	0.9910908	6	Singlet	Neurons
VAT_2M	3060	1649	0.2181259	0	Singlet	Adipocyte
VAT_2M	6091.1	2648	0.0062952	0	Singlet	Adipocyte
VAT_2M	3889.4	1954	0.0771191	0	Singlet	Adipocyte
VAT_2M	5039.5	2460	0.1755125	0	Singlet	Adipocyte
VAT_2M	3245.8	1749	0.2369288	0	Singlet	Adipocyte
VAT_2M	1733.5	1305	1.0756871	2	Singlet	FAPs
VAT_2M	1105.5	879	1.1731503	11	Singlet	Smooth_muscle_cells
VAT_2M	3982.6	1897	0.0397312	0	Singlet	Adipocyte
VAT_2M	3820.7	1973	0.8656004	0	Singlet	Adipocyte
VAT_2M	1246.6	929	0.6180968	6	Singlet	Neurons
VAT_2M	6882.2	2690	0.0357765	0	Singlet	Adipocyte
VAT_2M	3829.9	2234	0.1794697	3	Singlet	FAPs
VAT_2M	2989.1	1611	0.618237	0	Singlet	Adipocyte
VAT_2M	3269.1	1743	1.1711865	3	Singlet	FAPs
VAT_2M	2604.3	1707	0.0420226	3	Singlet	FAPs
VAT_2M	3576.1	1781	0.0640297	0	Singlet	Adipocyte
VAT_2M	2804.6	1583	0.0295505	0	Singlet	Adipocyte
VAT_2M	10955	3632	0.3144883	0	Singlet	Adipocyte
VAT_2M	3562.9	2138	1.7280825	2	Singlet	FAPs
VAT_2M	1146.7	985	0.6182558	6	Singlet	Neurons
VAT_2M	1012.1	789	3.467943	10	Singlet	Immune_cells
VAT_2M	7321.6	3008	0.0059588	0	Singlet	Adipocyte
VAT_2M	7542.1	2899	0.0304255	0	Singlet	Adipocyte
VAT_2M	1946.6	1172	1.0188203	0	Singlet	Adipocyte
VAT_2M	3391.4	1707	0.3866103	0	Singlet	Adipocyte
VAT_2M	2129.5	1272	0.3422206	0	Singlet	Adipocyte
VAT_2M	6364.4	2274	0.0035806	0	Singlet	Adipocyte
VAT_2M	8020.6	3301	0.2461907	0	Singlet	Adipocyte
VAT_2M	3023.5	1627	2.0441603	0	Singlet	Adipocyte
VAT_2M	2373.3	1449	0.3748477	7	Singlet	Endothelial_cells

VAT_2M	1338.7	1012	0.2922878	0 Singlet	Adipocyte
VAT_2M	1649.4	1164	1.4206115	2 Singlet	FAPs
VAT_2M	1161	971	2.0515245	5 Singlet	Immune_cells
VAT_2M	2908.7	1712	0.2724833	0 Singlet	Adipocyte
VAT_2M	1286.5	1055	2.2803879	5 Singlet	Immune_cells
VAT_2M	2397.9	1632	1.208369	2 Singlet	FAPs
VAT_2M	2584.9	1622	0.5157236	0 Singlet	Adipocyte
VAT_2M	1271	945	0.9834267	3 Singlet	FAPs
VAT_2M	2100.5	1543	0.653686	2 Singlet	FAPs
VAT_2M	2088.1	1391	0.9963443	3 Singlet	FAPs
VAT_2M	1819.7	1315	2.222635	5 Singlet	Immune_cells
VAT_2M	2057.6	1393	0.9671135	5 Singlet	Immune_cells
VAT_2M	2956.1	1837	1.1606039	2 Singlet	FAPs
VAT_2M	2413.8	1555	0.2095253	0 Singlet	Adipocyte
VAT_2M	5417.3	2318	0.5127306	0 Singlet	Adipocyte
VAT_2M	5450.3	2472	0.0803136	0 Singlet	Adipocyte
VAT_2M	2251.9	1283	2.0956511	5 Singlet	Immune_cells
VAT_2M	2212.9	1601	1.904886	2 Singlet	FAPs
VAT_2M	1560.3	1206	1.9010746	2 Singlet	FAPs
VAT_2M	1255.8	986	0.8291304	5 Singlet	Immune_cells
VAT_2M	1259.2	896	2.0866193	2 Singlet	FAPs
VAT_2M	11660	3487	0.0198631	0 Singlet	Adipocyte
VAT_2M	5874.5	2664	0.2421622	0 Singlet	Adipocyte
VAT_2M	11249	3550	0.0324506	0 Singlet	Adipocyte
VAT_2M	2704.6	1864	8.1344867	2 Singlet	FAPs
VAT_2M	2032.2	1290	0.6371256	2 Singlet	FAPs
VAT_2M	3410.5	2018	2.4022112	5 Singlet	Immune_cells
VAT_2M	2184.3	1443	0.6359403	3 Singlet	FAPs
VAT_2M	1254.7	926	1.7174325	6 Singlet	Neurons
VAT_2M	2400.7	1633	0.3052036	6 Singlet	Neurons
VAT_2M	1126.9	793	2.6137678	6 Singlet	Neurons
VAT_2M	1811.1	1037	3.360338	0 Singlet	Adipocyte
VAT_2M	1648.7	1163	2.697313	5 Singlet	Immune_cells
VAT_2M	5518	2385	0.0275722	0 Singlet	Adipocyte
VAT_2M	1658.8	1283	0.4100531	11 Singlet	Smooth_muscle_cells
VAT_2M	6393.4	2639	0.3351326	0 Singlet	Adipocyte
VAT_2M	1368.4	1064	1.3442012	6 Singlet	Neurons
VAT_2M	1898	1380	0.5610889	0 Singlet	Adipocyte
VAT_2M	2641.7	1698	0.111654	3 Singlet	FAPs
VAT_2M	1895	1326	0.8979149	2 Singlet	FAPs
VAT_2M	6831.5	2793	0.0023162	0 Singlet	Adipocyte
VAT_2M	1677.9	1102	3.5780279	3 Singlet	FAPs
VAT_2M	7035.4	2870	0.0146186	0 Singlet	Adipocyte
VAT_2M	1781.5	1094	0.1393122	0 Singlet	Adipocyte
VAT_2M	2389.8	1639	1.5370974	6 Singlet	Neurons
VAT_2M	6841.5	2851	0.0909126	0 Singlet	Adipocyte
VAT_2M	2387.8	1515	1.7924245	5 Singlet	Immune_cells
VAT_2M	2437.3	1634	1.4654262	2 Singlet	FAPs
VAT_2M	6754.2	2813	0.0482302	0 Singlet	Adipocyte
VAT_2M	1183.7	933	4.685446	2 Singlet	FAPs
VAT_2M	2208.6	1432	0.0674881	0 Singlet	Adipocyte

VAT_2M	2131.1	1391	1.3221792	2	Singlet	FAPs
VAT_2M	1074.7	798	1.0317369	3	Singlet	FAPs
VAT_2M	5654.3	2477	0.188911	0	Singlet	Adipocyte
VAT_2M	1648.5	998	2.1473983	0	Singlet	Adipocyte
VAT_2M	2015.6	1163	0.2444712	0	Singlet	Adipocyte
VAT_2M	1880.2	1299	2.1286531	7	Singlet	Endothelial_cells
VAT_2M	1822.2	1225	2.51761	3	Singlet	FAPs
VAT_2M	1694.4	1366	0.6794518	7	Singlet	Endothelial_cells
VAT_2M	2000.8	1392	0.5186351	2	Singlet	FAPs
VAT_2M	1970.1	1259	0.2992845	0	Singlet	Adipocyte
VAT_2M	1861.7	1101	0.6740778	3	Singlet	FAPs
VAT_2M	1241.2	941	0.3259412	4	Singlet	Immune_cells
VAT_2M	1028.7	825	2.7320333	6	Singlet	Neurons
VAT_2M	7124.4	2822	0	0	Singlet	Adipocyte
VAT_2M	7543.6	2896	0.0146866	0	Singlet	Adipocyte
VAT_2M	3619.9	1813	0.0141853	0	Singlet	Adipocyte
VAT_2M	2956.5	1820	0.4935317	2	Singlet	FAPs
VAT_2M	1623.5	1024	5.8148969	0	Singlet	Adipocyte
VAT_2M	11373	3501	0.1796514	0	Singlet	Adipocyte
VAT_2M	3689	1768	0.0324211	0	Singlet	Adipocyte
VAT_2M	6900	2546	0.0500252	0	Singlet	Adipocyte
VAT_2M	4677.9	2108	0.1569242	0	Singlet	Adipocyte
VAT_2M	3354.9	1608	0.2708621	0	Singlet	Adipocyte
VAT_2M	4388.9	2240	0.1903327	0	Singlet	Adipocyte
VAT_2M	1458.3	988	1.2423441	3	Singlet	FAPs
VAT_2M	2163.6	1355	1.7639196	2	Singlet	FAPs
VAT_2M	9321.3	3245	0.0308024	0	Singlet	Adipocyte
VAT_2M	2193.1	1447	0.5418642	3	Singlet	FAPs
VAT_2M	1760.6	1203	1.1234639	6	Singlet	Neurons
VAT_2M	10130	3486	0	0	Singlet	Adipocyte
VAT_2M	3368.2	1857	0.1800053	0	Singlet	Adipocyte
VAT_2M	1057.1	750	4.6182265	3	Singlet	FAPs
VAT_2M	2321.2	1499	3.6764498	3	Singlet	FAPs
VAT_2M	1268.8	947	3.0186491	0	Singlet	Adipocyte
VAT_2M	6156.1	2709	0.2688855	0	Singlet	Adipocyte
VAT_2M	4096.8	2070	0.1293029	0	Singlet	Adipocyte
VAT_2M	4341.1	2199	0.1037621	0	Singlet	Adipocyte
VAT_2M	2688	1497	0.319893	0	Singlet	Adipocyte
VAT_2M	5590.9	2358	0.0362943	0	Singlet	Adipocyte
VAT_2M	3117.8	1623	0.0222721	0	Singlet	Adipocyte
VAT_2M	1853.7	1117	0.4415889	0	Singlet	Adipocyte
VAT_2M	6228.4	2834	0.2108943	0	Singlet	Adipocyte
VAT_2M	7920.2	2980	0.0002505	0	Singlet	Adipocyte
VAT_2M	4569	2230	1.5639739	0	Singlet	Adipocyte
VAT_2M	1707.1	1350	0	7	Singlet	Endothelial_cells
VAT_2M	1407	1064	2.3437917	5	Singlet	Immune_cells
VAT_2M	1855.8	1346	0.8766003	3	Singlet	FAPs
VAT_2M	3262.1	1927	0.1982733	3	Singlet	FAPs
VAT_2M	1910.9	1096	0.078913	0	Singlet	Adipocyte
VAT_2M	6764.8	3302	0.4698879	2	Singlet	FAPs
VAT_2M	4027.9	2090	0.1027613	0	Singlet	Adipocyte

VAT_2M	2230.1	1271	1.7872123	0	Singlet	Adipocyte
VAT_2M	4178.1	1938	0.2203536	0	Singlet	Adipocyte
VAT_2M	2187.1	1291	1.0491839	2	Singlet	FAPs
VAT_2M	5187.4	2462	0	0	Singlet	Adipocyte
VAT_2M	3058.2	1650	0.0784741	0	Singlet	Adipocyte
VAT_2M	1647.8	1292	0.3575103	3	Singlet	FAPs
VAT_2M	1438.2	1184	2.6202976	2	Singlet	FAPs
VAT_2M	4542.5	2242	0.7864709	0	Singlet	Adipocyte
VAT_2M	1798.2	1369	1.0266225	3	Singlet	FAPs
VAT_2M	1900.8	1068	0.5891833	0	Singlet	Adipocyte
VAT_2M	1509.7	1051	3.0282275	3	Singlet	FAPs
VAT_2M	4007.2	1852	0.0138004	0	Singlet	Adipocyte
VAT_2M	4812.5	2392	0.1007948	0	Singlet	Adipocyte
VAT_2M	3925.9	2043	0.0858715	0	Singlet	Adipocyte
VAT_2M	3442.7	1857	1.772945	0	Singlet	Adipocyte
VAT_2M	1186.9	737	11.537236	4	Singlet	Immune_cells
VAT_2M	1813.1	1089	0.6299499	2	Singlet	FAPs
VAT_2M	1049.7	789	0.9644603	6	Singlet	Neurons
VAT_2M	5149.6	2264	0.1091909	0	Singlet	Adipocyte
VAT_2M	3721.3	2088	0.3599162	2	Singlet	FAPs
VAT_2M	1018.4	775	6.083176	2	Singlet	FAPs
VAT_2M	8459.3	2998	0.0717492	0	Singlet	Adipocyte
VAT_2M	1095.9	794	1.2750259	3	Singlet	FAPs
VAT_2M	3415.2	1802	0.0180923	0	Singlet	Adipocyte
VAT_2M	3717.7	1823	0.0879114	0	Singlet	Adipocyte
VAT_2M	2764.1	1723	0.0489823	5	Singlet	Immune_cells
VAT_2M	2012	1311	1.8792697	2	Singlet	FAPs
VAT_2M	1213.3	945	0.7379506	2	Singlet	FAPs
VAT_2M	2631.5	1541	1.0604674	0	Singlet	Adipocyte
VAT_2M	5605.8	2507	0.1287131	0	Singlet	Adipocyte
VAT_2M	1673.8	1115	0.4491912	0	Singlet	Adipocyte
VAT_2M	2336.8	1297	0.2220704	0	Singlet	Adipocyte
VAT_2M	1568.6	1020	0.5935518	0	Singlet	Adipocyte
VAT_2M	2751	1593	0	0	Singlet	Adipocyte
VAT_2M	1158.5	869	2.7272409	3	Singlet	FAPs
VAT_2M	6409.1	2822	0.0164723	0	Singlet	Adipocyte
VAT_2M	2363.1	1600	3.5764816	9	Singlet	Mesothelial
VAT_2M	4948.8	2419	0.6110426	0	Singlet	Adipocyte
VAT_2M	5933.4	2653	0.0046351	0	Singlet	Adipocyte
VAT_2M	4554.5	2129	0.9671856	0	Singlet	Adipocyte
VAT_2M	1714.2	1163	0.1200784	3	Singlet	FAPs
VAT_2M	1126.6	855	1.2444939	7	Singlet	Endothelial_cells
VAT_2M	5994.3	2558	0	0	Singlet	Adipocyte
VAT_2M	1776	1173	0.1459864	3	Singlet	FAPs
VAT_2M	1095.6	903	1.7942379	9	Singlet	Mesothelial
VAT_2M	2479.4	1364	0.4383666	0	Singlet	Adipocyte
VAT_2M	11482	3707	0.0494454	0	Singlet	Adipocyte
VAT_2M	1213	862	0.5551322	7	Singlet	Endothelial_cells
VAT_2M	1850.7	1259	2.7562592	2	Singlet	FAPs
VAT_2M	1861.8	1247	1.4473412	4	Singlet	Immune_cells
VAT_2M	1198.4	1025	1.3476079	1	Singlet	Immune_cells

VAT_2M	1032	897	2.1118629	4	Singlet	Immune_cells
VAT_2M	8288.2	2990	0.0479384	0	Singlet	Adipocyte
VAT_2M	2063.5	1266	0.4307003	0	Singlet	Adipocyte
VAT_2M	2852.1	1750	0.3221515	11	Singlet	Smooth_muscle_cells
VAT_2M	2103	1411	0.7025528	2	Singlet	FAPs
VAT_2M	3581.3	1811	0.0459563	0	Singlet	Adipocyte
VAT_2M	3016.1	1699	0.0486387	0	Singlet	Adipocyte
VAT_2M	1300	839	0.4386721	2	Singlet	FAPs
VAT_2M	6939.6	2745	0.0990049	0	Singlet	Adipocyte
VAT_2M	9712.3	3338	0.0232379	0	Singlet	Adipocyte
VAT_2M	3147	2032	0.8555159	5	Singlet	Immune_cells
VAT_2M	1186.4	885	0.7558337	0	Singlet	Adipocyte
VAT_2M	1120.6	871	0.726748	2	Singlet	FAPs
VAT_2M	1461.9	1062	0.9036661	5	Singlet	Immune_cells
VAT_2M	3335.3	1741	0.0781048	0	Singlet	Adipocyte
VAT_2M	1037.4	817	0.0378671	7	Singlet	Endothelial_cells
VAT_2M	6495	2737	0.0152178	0	Singlet	Adipocyte
VAT_2M	5323.5	2365	0.0388659	0	Singlet	Adipocyte
VAT_2M	2072.3	1583	1.9237923	3	Singlet	FAPs
VAT_2M	5097.9	2286	0.2354221	0	Singlet	Adipocyte
VAT_2M	6269.9	2519	0.5917376	0	Singlet	Adipocyte
VAT_2M	6725.6	2698	0.0047187	0	Singlet	Adipocyte
VAT_2M	2010.6	1420	0.7068119	2	Singlet	FAPs
VAT_2M	1004.4	829	0.1849258	4	Singlet	Immune_cells
VAT_2M	3316.3	1862	0.8278749	3	Singlet	FAPs
VAT_2M	1947.5	1365	5.0036931	9	Singlet	Mesothelial
VAT_2M	1471.3	1162	1.8887104	3	Singlet	FAPs
VAT_2M	1417.2	1116	1.0166915	6	Singlet	Neurons
VAT_2M	3959.4	2087	0.8507722	3	Singlet	FAPs
VAT_2M	9083.9	3265	0.0743184	0	Singlet	Adipocyte
VAT_2M	5286.7	2283	0.1868048	0	Singlet	Adipocyte
VAT_2M	1939.7	1188	0.3489975	0	Singlet	Adipocyte
VAT_2M	3194.7	1823	0.0995954	6	Singlet	Neurons
VAT_2M	1200	979	2.5979521	6	Singlet	Neurons
VAT_2M	2447.8	1548	3.0974431	9	Singlet	Mesothelial
VAT_2M	1851.3	1278	2.9555507	5	Singlet	Immune_cells
VAT_2M	6320.8	2594	0.8288772	0	Singlet	Adipocyte
VAT_2M	1805.8	1262	0.1099845	6	Singlet	Neurons
VAT_2M	1642	1230	0.4402392	6	Singlet	Neurons
VAT_2M	1071	798	1.8200258	3	Singlet	FAPs
VAT_2M	7172.8	2951	0.1208865	0	Singlet	Adipocyte
VAT_2M	1035.9	881	2.698664	6	Singlet	Neurons
VAT_2M	2412.4	1449	2.6403582	3	Singlet	FAPs
VAT_2M	1192.5	931	2.2609014	5	Singlet	Immune_cells
VAT_2M	3741	1793	1.3424225	0	Singlet	Adipocyte
VAT_2M	1918.6	1226	1.2440958	0	Singlet	Adipocyte
VAT_2M	5661.8	2367	0	0	Singlet	Adipocyte
VAT_2M	2571	1642	0.7990528	5	Singlet	Immune_cells
VAT_2M	1537.6	1161	3.2119085	2	Singlet	FAPs
VAT_2M	1016.7	796	1.6875022	5	Singlet	Immune_cells
VAT_2M	3941.1	2330	1.6644471	2	Singlet	FAPs

VAT_2M	1032.7	796	2.4016739	7	Singlet	Endothelial_cells
VAT_2M	5379.4	2272	0.0773712	0	Singlet	Adipocyte
VAT_2M	3724.4	1836	0	0	Singlet	Adipocyte
VAT_2M	1008	837	0.2384429	4	Singlet	Immune_cells
VAT_2M	2392.4	1519	0.9222271	0	Singlet	Adipocyte
VAT_2M	2394.1	1389	0.1920843	0	Singlet	Adipocyte
VAT_2M	1901.1	1297	1.1173755	2	Singlet	FAPs
VAT_2M	1525.6	1073	0.5861708	2	Singlet	FAPs
VAT_2M	1642.9	1147	0.6571888	2	Singlet	FAPs
VAT_2M	5932.5	2483	0.0051462	0	Singlet	Adipocyte
VAT_2M	8417.3	3086	0	0	Singlet	Adipocyte
VAT_2M	5069.5	2727	1.4662517	2	Singlet	FAPs
VAT_2M	5421.6	2378	0	0	Singlet	Adipocyte
VAT_2M	2951.2	1743	0.5417141	3	Singlet	FAPs
VAT_2M	2102.2	1419	0.2796394	3	Singlet	FAPs
VAT_2M	2138.8	1504	0.7721974	2	Singlet	FAPs
VAT_2M	1023	815	0.7693481	8	Singlet	Mesothelial
VAT_2M	3146.5	1750	0.5533737	3	Singlet	FAPs
VAT_2M	1024.1	838	1.6824448	8	Singlet	Mesothelial
VAT_2M	1292.1	941	0	6	Singlet	Neurons
VAT_2M	1033	790	2.9134921	5	Singlet	Immune_cells
VAT_2M	5979.5	2658	0.0108886	0	Singlet	Adipocyte
VAT_2M	1083	885	1.3767093	1	Singlet	Immune_cells
VAT_2M	4202.2	2237	0.4709471	0	Singlet	Adipocyte
VAT_2M	2842	1904	0.2216782	3	Singlet	FAPs
VAT_2M	1419.2	1009	0.4453358	5	Singlet	Immune_cells
VAT_2M	2480.6	1395	0.1113221	2	Singlet	FAPs
VAT_2M	4634.3	2258	0.4695185	0	Singlet	Adipocyte
VAT_2M	1648.9	1056	0.3097025	2	Singlet	FAPs
VAT_2M	6332.7	2535	0.7164877	0	Singlet	Adipocyte
VAT_2M	2737.5	1709	2.600184	5	Singlet	Immune_cells
VAT_2M	4953.4	2159	0.0419567	0	Singlet	Adipocyte
VAT_2M	1657.4	1099	0.9480851	0	Singlet	Adipocyte
VAT_2M	7761	2970	0.0026794	0	Singlet	Adipocyte
VAT_2M	6494.3	2663	0.1246771	0	Singlet	Adipocyte
VAT_2M	1460.8	1072	1.9774917	7	Singlet	Endothelial_cells
VAT_2M	2516.6	1546	0.2313003	0	Singlet	Adipocyte
VAT_2M	6693.5	2600	0.0698327	0	Singlet	Adipocyte
VAT_2M	1067.9	795	2.1710456	2	Singlet	FAPs
VAT_2M	2919.5	1527	0.5917082	0	Singlet	Adipocyte
VAT_2M	1035.8	849	1.621242	6	Singlet	Neurons
VAT_2M	1112.4	930	0.5461239	7	Singlet	Endothelial_cells
VAT_2M	7966.8	2948	0.0332871	0	Singlet	Adipocyte
VAT_2M	7230.3	2737	0.0876201	0	Singlet	Adipocyte
VAT_2M	2217.2	1381	0.9074136	0	Singlet	Adipocyte
VAT_2M	2346.9	1471	0.7904269	0	Singlet	Adipocyte
VAT_2M	1461.3	1023	1.4693689	11	Singlet	Smooth_muscle_cells
VAT_2M	3557.6	1723	0.0825232	0	Singlet	Adipocyte
VAT_2M	2325	1407	0.3644461	0	Singlet	Adipocyte
VAT_2M	1580.5	1101	1.5666144	6	Singlet	Neurons
VAT_2M	2674.1	1604	0.2190483	0	Singlet	Adipocyte

VAT_2M	1674.2	1187	4.1974263	2	Singlet	FAPs
VAT_2M	2345	1527	0.7933982	2	Singlet	FAPs
VAT_2M	5877.1	2479	0.0317078	0	Singlet	Adipocyte
VAT_2M	4183.3	1882	0.0344301	0	Singlet	Adipocyte
VAT_2M	7373.1	2809	0.0035919	0	Singlet	Adipocyte
VAT_2M	1021.1	842	1.3584131	6	Singlet	Neurons
VAT_2M	3108.5	1655	0.4132146	0	Singlet	Adipocyte
VAT_2M	1320	977	1.4256885	6	Singlet	Neurons
VAT_2M	1264.8	936	0.6481638	2	Singlet	FAPs
VAT_2M	2106.8	1195	2.6966314	0	Singlet	Adipocyte
VAT_2M	3147	1767	0.0243011	0	Singlet	Adipocyte
VAT_2M	2378.5	1438	0.0293904	3	Singlet	FAPs
VAT_2M	1175.9	899	0.3520415	6	Singlet	Neurons
VAT_2M	1063.4	779	1.3170737	5	Singlet	Immune_cells
VAT_2M	2803.9	1765	2.5811458	3	Singlet	FAPs
VAT_2M	2128.3	1448	0.167488	2	Singlet	FAPs
VAT_2M	4549.5	1894	0.6147175	0	Singlet	Adipocyte
VAT_2M	1460.4	1052	0.5507595	3	Singlet	FAPs
VAT_2M	7772.8	2975	0.0430006	0	Singlet	Adipocyte
VAT_2M	2325.8	1510	0.6270439	11	Singlet	Smooth_muscle_cells
VAT_2M	2777.3	1542	0.1657036	0	Singlet	Adipocyte
VAT_2M	1980	1460	0.4757111	5	Singlet	Immune_cells
VAT_2M	1337.9	1093	1.2330999	6	Singlet	Neurons
VAT_2M	4837	2254	0.0085913	0	Singlet	Adipocyte
VAT_2M	1042.5	817	1.2380191	3	Singlet	FAPs
VAT_2M	1346.5	1001	3.4188702	7	Singlet	Endothelial_cells
VAT_2M	2385.8	1444	0.5144755	0	Singlet	Adipocyte
VAT_2M	3849.7	1852	0.0275675	0	Singlet	Adipocyte
VAT_2M	6615.3	2726	0.1217799	0	Singlet	Adipocyte
VAT_2M	6624	2823	1.0664431	0	Singlet	Adipocyte
VAT_2M	1818.2	1311	2.2274876	2	Singlet	FAPs
VAT_2M	1655.3	1135	0.1606461	0	Singlet	Adipocyte
VAT_2M	2377.3	1554	3.1614196	7	Singlet	Endothelial_cells
VAT_2M	1753.3	1204	0.7536776	5	Singlet	Immune_cells
VAT_2M	1664.8	1283	0.6984944	5	Singlet	Immune_cells
VAT_2M	4655.4	2185	0.0078463	0	Singlet	Adipocyte
VAT_2M	1864.3	1222	0.3710305	0	Singlet	Adipocyte
VAT_2M	1083.6	877	1.3369565	6	Singlet	Neurons
VAT_2M	2226.1	1397	0.4461579	0	Singlet	Adipocyte
VAT_2M	2990.7	1759	0.3693532	0	Singlet	Adipocyte
VAT_2M	1920.8	1286	0.4808029	2	Singlet	FAPs
VAT_2M	2306.1	1384	0.1508045	0	Singlet	Adipocyte
VAT_2M	1051.6	834	2.0716634	6	Singlet	Neurons
VAT_2M	1846.6	1455	1.168909	2	Singlet	FAPs
VAT_2M	1389.7	1098	1.0409577	6	Singlet	Neurons
VAT_2M	4453.5	2180	0.3303396	0	Singlet	Adipocyte
VAT_2M	1493	1178	0.4515147	6	Singlet	Neurons
VAT_2M	3234.6	1778	8.8561384	7	Singlet	Endothelial_cells
VAT_2M	1932.2	1345	1.2794729	2	Singlet	FAPs
VAT_2M	1029.4	772	0.1840261	5	Singlet	Immune_cells
VAT_2M	1609.7	1044	0.1050148	0	Singlet	Adipocyte

VAT_2M	2624.5	1503	0.088566	0	Singlet	Adipocyte
VAT_2M	7136.2	2768	0.0335501	0	Singlet	Adipocyte
VAT_2M	1997.3	1368	1.5332345	3	Singlet	FAPs
VAT_2M	1020.5	787	0.8037649	6	Singlet	Neurons
VAT_2M	1761.9	1129	0.4125789	0	Singlet	Adipocyte
VAT_2M	7130.9	2725	0.0183045	0	Singlet	Adipocyte
VAT_2M	2089.5	1214	0.7034615	0	Singlet	Adipocyte
VAT_2M	1010.6	839	0.6805636	6	Singlet	Neurons
VAT_2M	4923.1	2192	0.0831865	0	Singlet	Adipocyte
VAT_2M	7051.3	2776	0.0039106	0	Singlet	Adipocyte
VAT_2M	6962.6	2637	0.0643422	0	Singlet	Adipocyte
VAT_2M	1234.3	895	0.4643597	0	Singlet	Adipocyte
VAT_2M	1982.4	1258	0.0406239	0	Singlet	Adipocyte
VAT_2M	3502.1	2022	0.1137385	2	Singlet	FAPs
VAT_2M	3477.2	1809	2.9830579	0	Singlet	Adipocyte
VAT_2M	1201.1	882	1.2645265	0	Singlet	Adipocyte
VAT_2M	3118.9	1594	0.2186636	0	Singlet	Adipocyte
VAT_2M	5921.4	2683	0.0475704	0	Singlet	Adipocyte
VAT_2M	3991.1	2092	0.0941085	0	Singlet	Adipocyte
VAT_2M	6435.3	2534	0.125115	0	Singlet	Adipocyte
VAT_2M	2939.4	1776	0.2363699	0	Singlet	Adipocyte
VAT_2M	1373.9	1043	1.9743714	2	Singlet	FAPs
VAT_2M	2592.1	1542	0.7537934	3	Singlet	FAPs
VAT_2M	1922.9	1233	1.5275997	11	Singlet	Smooth_muscle_cells
VAT_2M	7640.8	2951	0.0317815	0	Singlet	Adipocyte
VAT_2M	2959.3	1918	0.9515061	5	Singlet	Immune_cells
VAT_2M	1402.6	976	0.2530046	7	Singlet	Endothelial_cells
VAT_2M	4906.5	2328	0.0517462	0	Singlet	Adipocyte
VAT_2M	2354.7	1451	0.0880519	0	Singlet	Adipocyte
VAT_2M	1746.4	1323	1.3246706	9	Singlet	Mesothelial
VAT_2M	1269.9	989	0.2238437	5	Singlet	Immune_cells
VAT_2M	2871.7	1637	2.1090538	3	Singlet	FAPs
VAT_2M	6438	2748	0.0059123	0	Singlet	Adipocyte
VAT_2M	1323	949	0	3	Singlet	FAPs
VAT_2M	8646.4	3305	0.0035213	0	Singlet	Adipocyte
VAT_2M	2079.2	1243	0.1683129	0	Singlet	Adipocyte
VAT_2M	5501.2	2603	0	0	Singlet	Adipocyte
VAT_2M	1043.9	738	1.0809511	2	Singlet	FAPs
VAT_2M	2388.2	1366	0.8017733	0	Singlet	Adipocyte
VAT_2M	1184.8	981	0.5664902	11	Singlet	Smooth_muscle_cells
VAT_2M	2906.3	1734	4.3054494	7	Singlet	Endothelial_cells
VAT_2M	1346.9	917	0.1825189	3	Singlet	FAPs
VAT_2M	3776.8	1829	0.0686868	0	Singlet	Adipocyte
VAT_2M	7991.6	2858	0.004011	0	Singlet	Adipocyte
VAT_2M	1371.2	965	0.0186341	5	Singlet	Immune_cells
VAT_2M	8059.9	3145	0.0006557	0	Singlet	Adipocyte
VAT_2M	1548.3	1117	0.5337738	3	Singlet	FAPs
VAT_2M	5919.5	2496	0.0201628	0	Singlet	Adipocyte
VAT_2M	3477.6	1986	0.6489038	5	Singlet	Immune_cells
VAT_2M	1806.5	1125	0.1301011	0	Singlet	Adipocyte
VAT_2M	1073.6	776	2.2030736	5	Singlet	Immune_cells

VAT_2M	7738.5	2993	0.043448	0 Singlet	Adipocyte
VAT_2M	5263	2364	0.0616106	0 Singlet	Adipocyte
VAT_2M	8349.2	3057	0.0496369	0 Singlet	Adipocyte
VAT_2M	4808.1	2384	0.0653535	0 Singlet	Adipocyte
VAT_2M	1675.6	1131	0.8273467	6 Singlet	Neurons
VAT_2M	3086.9	1960	1.2938999	2 Singlet	FAPs
VAT_2M	1637.7	1156	0.5407691	9 Singlet	Mesothelial
VAT_2M	1286.2	893	3.5206329	7 Singlet	Endothelial_cells
VAT_2M	2403.1	1586	1.3158711	2 Singlet	FAPs
VAT_2M	1096.5	857	0.0698635	2 Singlet	FAPs
VAT_2M	7987.3	2956	0.0026562	0 Singlet	Adipocyte
VAT_2M	2457.4	1327	0.0274636	0 Singlet	Adipocyte
VAT_2M	1553.1	1086	1.4318529	5 Singlet	Immune_cells
VAT_2M	2855.6	1627	0.2922636	0 Singlet	Adipocyte
VAT_2M	3023.4	1633	0.1005509	0 Singlet	Adipocyte
VAT_2M	1123.2	834	1.8503617	9 Singlet	Mesothelial
VAT_2M	1975.9	1233	0.3406754	5 Singlet	Immune_cells
VAT_2M	1816.8	1177	1.2572614	6 Singlet	Neurons
VAT_2M	4095.9	2033	0.974857	0 Singlet	Adipocyte
VAT_2M	2296.5	1344	0.2392832	3 Singlet	FAPs
VAT_2M	2248.7	1400	2.7776645	2 Singlet	FAPs
VAT_2M	1306.2	1001	0.1664074	9 Singlet	Mesothelial
VAT_2M	1956.4	1249	0.0987661	5 Singlet	Immune_cells
VAT_2M	8048.8	3047	0.0525129	0 Singlet	Adipocyte
VAT_2M	1401.3	1030	3.0637771	2 Singlet	FAPs
VAT_2M	5383.4	2308	0.2683567	0 Singlet	Adipocyte
VAT_2M	9516.9	3350	0.0290181	0 Singlet	Adipocyte
VAT_2M	1170.1	910	0.1511019	6 Singlet	Neurons
VAT_2M	2193.7	1422	1.0833623	3 Singlet	FAPs
VAT_2M	4672.9	2171	0.1471066	0 Singlet	Adipocyte
VAT_2M	6599.9	2677	0.1467594	0 Singlet	Adipocyte
VAT_2M	2147.6	1331	0.0754663	7 Singlet	Endothelial_cells
VAT_2M	1802.7	1114	0.2179316	0 Singlet	Adipocyte
VAT_2M	1648.7	984	0.1045651	0 Singlet	Adipocyte
VAT_2M	1296.7	1079	0.8195556	6 Singlet	Neurons
VAT_2M	1246	1041	0.2992994	8 Singlet	Mesothelial
VAT_2M	6762.6	2713	0.0619969	0 Singlet	Adipocyte
VAT_2M	4180	2015	0.0232215	0 Singlet	Adipocyte
VAT_2M	1850.7	1290	0.3433805	3 Singlet	FAPs
VAT_2M	6222.1	2638	0.3338144	0 Singlet	Adipocyte
VAT_2M	2258.2	1306	2.2199923	0 Singlet	Adipocyte
VAT_2M	4402.7	2020	0	0 Singlet	Adipocyte
VAT_2M	8221.9	3114	0.8068571	0 Singlet	Adipocyte
VAT_2M	2559.9	1508	0.4584487	0 Singlet	Adipocyte
VAT_2M	3168.2	1622	0.1636773	0 Singlet	Adipocyte
VAT_2M	1324	1005	1.2162343	7 Singlet	Endothelial_cells
VAT_2M	1299.2	929	1.1831566	2 Singlet	FAPs
VAT_2M	5063.5	2400	2.3663748	2 Singlet	FAPs
VAT_2M	6570.9	2589	0	0 Singlet	Adipocyte
VAT_2M	1140.6	815	0.1015927	7 Singlet	Endothelial_cells
VAT_2M	2344.7	1405	1.1302423	0 Singlet	Adipocyte

VAT_2M	1218.2	936	0.4814849	3	Singlet	FAPs
VAT_2M	1252.4	803	14.009711	5	Singlet	Immune_cells
VAT_2M	3879.9	1801	0.0399847	0	Singlet	Adipocyte
VAT_2M	1834.3	1335	1.4945606	3	Singlet	FAPs
VAT_2M	8092.4	3060	0.0186159	0	Singlet	Adipocyte
VAT_2M	6756.6	2580	0.0143461	0	Singlet	Adipocyte
VAT_2M	9691.7	3371	0.0306513	0	Singlet	Adipocyte
VAT_2M	4015.7	2071	1.3547369	0	Singlet	Adipocyte
VAT_2M	7355	2956	0.0017438	0	Singlet	Adipocyte
VAT_2M	2823.4	1667	2.2995906	2	Singlet	FAPs
VAT_2M	4529	2182	0.0109796	0	Singlet	Adipocyte
VAT_2M	3406	1772	1.3363506	3	Singlet	FAPs
VAT_2M	1914.4	1100	1.4045019	0	Singlet	Adipocyte
VAT_2M	4023.9	2233	0	0	Singlet	Adipocyte
VAT_2M	5758.7	2374	0.012407	0	Singlet	Adipocyte
VAT_2M	5692.3	2545	0.0574662	0	Singlet	Adipocyte
VAT_2M	7084.4	2926	0.2583514	0	Singlet	Adipocyte
VAT_2M	2228.2	1347	0.3037004	0	Singlet	Adipocyte
VAT_2M	1315.3	1055	1.3219398	5	Singlet	Immune_cells
VAT_2M	1163.9	929	0.1749933	6	Singlet	Neurons
VAT_2M	3878	1903	0.1532172	0	Singlet	Adipocyte
VAT_2M	5291.9	2455	0.0336279	0	Singlet	Adipocyte
VAT_2M	2134.8	1353	1.3281575	2	Singlet	FAPs
VAT_2M	1185.1	899	2.2232759	6	Singlet	Neurons
VAT_2M	2184.1	1438	0.7737636	6	Singlet	Neurons
VAT_2M	1944.6	1347	1.6770402	3	Singlet	FAPs
VAT_2M	2834.9	1469	0.2234579	0	Singlet	Adipocyte
VAT_2M	1052	871	0.257476	4	Singlet	Immune_cells
VAT_2M	2174.2	1419	0.2359846	2	Singlet	FAPs
VAT_2M	11352	3630	0.0517331	0	Singlet	Adipocyte
VAT_2M	8824.5	3202	0.1010141	0	Singlet	Adipocyte
VAT_2M	3694.4	1805	0.2504483	0	Singlet	Adipocyte
VAT_2M	8271.5	2964	0	0	Singlet	Adipocyte
VAT_2M	1806.8	1283	0.7364511	6	Singlet	Neurons
VAT_2M	8427.3	3075	0.1065779	0	Singlet	Adipocyte
VAT_2M	7319.9	2912	0.0219096	0	Singlet	Adipocyte
VAT_2M	1609.2	1111	0.3912104	2	Singlet	FAPs
VAT_2M	1632.5	1145	1.6317457	3	Singlet	FAPs
VAT_2M	2025.9	1411	3.7551343	7	Singlet	Endothelial_cells
VAT_2M	1956.1	1138	0.3701515	0	Singlet	Adipocyte
VAT_2M	4412.2	2218	0.0921794	0	Singlet	Adipocyte
VAT_2M	1893.2	1269	0.2891974	3	Singlet	FAPs
VAT_2M	8959.4	3539	0.2028934	0	Singlet	Adipocyte
VAT_2M	1842.7	1227	1.5307197	0	Singlet	Adipocyte
VAT_2M	1305	1010	0.3029623	8	Singlet	Mesothelial
VAT_2M	1308.2	913	1.5386126	2	Singlet	FAPs
VAT_2M	4958.9	2257	0	0	Singlet	Adipocyte
VAT_2M	4465.4	2344	1.1147975	0	Singlet	Adipocyte
VAT_2M	4185.1	2188	0.0590894	0	Singlet	Adipocyte
VAT_2M	2515.1	1565	0.3412898	3	Singlet	FAPs
VAT_2M	6037.7	2435	0.036938	0	Singlet	Adipocyte

VAT_2M	3326.9	1871	0.3326273	3 Singlet	FAPs
VAT_2M	1812.7	1384	3.9635424	2 Singlet	FAPs
VAT_2M	1959.3	1228	1.9362757	6 Singlet	Neurons
VAT_2M	8928.2	3372	0.091395	0 Singlet	Adipocyte
VAT_2M	1480.4	1133	3.9082769	6 Singlet	Neurons
VAT_2M	1131.7	834	0.6249999	3 Singlet	FAPs
VAT_2M	1326.3	939	3.3403384	5 Singlet	Immune_cells
VAT_2M	2154	1416	0.2460805	2 Singlet	FAPs
VAT_2M	2112.1	1211	0.2077682	0 Singlet	Adipocyte
VAT_2M	2608.9	1641	2.4531401	2 Singlet	FAPs
VAT_2M	9196.3	3196	0.0097658	0 Singlet	Adipocyte
VAT_2M	1305.8	911	0.217559	2 Singlet	FAPs
VAT_2M	2787.9	1659	0	0 Singlet	Adipocyte
VAT_2M	7453.1	2917	0.1685691	0 Singlet	Adipocyte
VAT_2M	8204.5	2975	0.0031208	0 Singlet	Adipocyte
VAT_2M	1084.7	836	0.1622321	3 Singlet	FAPs
VAT_2M	1698.7	1261	0.5723285	3 Singlet	FAPs
VAT_2M	2623.2	1625	0.2790734	7 Singlet	Endothelial_cells
VAT_2M	1122.1	822	0.5295456	2 Singlet	FAPs
VAT_2M	6231.3	2716	0	0 Singlet	Adipocyte
VAT_2M	3586.3	1829	0.0034565	0 Singlet	Adipocyte
VAT_2M	1068.9	741	7.33616	5 Singlet	Immune_cells
VAT_2M	1068.4	852	1.4029678	1 Singlet	Immune_cells
VAT_2M	1121.1	860	0.8595589	6 Singlet	Neurons
VAT_2M	3291.2	1819	0.0723503	0 Singlet	Adipocyte
VAT_2M	1439.3	1058	1.682618	2 Singlet	FAPs
VAT_2M	3936.1	1964	0.1011809	0 Singlet	Adipocyte
VAT_2M	1005.9	889	0.4826081	6 Singlet	Neurons
VAT_2M	5291.4	2309	0.648629	0 Singlet	Adipocyte
VAT_2M	1019.3	815	3.5287052	8 Singlet	Mesothelial
VAT_2M	7631.4	3007	0.1350656	0 Singlet	Adipocyte
VAT_2M	2097.3	1427	1.1958301	6 Singlet	Neurons
VAT_2M	1776.2	1237	1.8111579	2 Singlet	FAPs
VAT_2M	1217.6	1044	1.1922577	6 Singlet	Neurons
VAT_2M	9215.6	2997	2.8748293	0 Singlet	Adipocyte
VAT_2M	2874.8	1623	0.2430677	0 Singlet	Adipocyte
VAT_2M	9418.7	3225	0.1001302	0 Singlet	Adipocyte
VAT_2M	1110.1	916	0.4496066	2 Singlet	FAPs
VAT_2M	6980.4	2646	0.0136516	0 Singlet	Adipocyte
VAT_2M	5501	2322	0.3439743	0 Singlet	Adipocyte
VAT_2M	2853.3	1636	0.5014156	0 Singlet	Adipocyte
VAT_2M	2313.5	1536	0.7448972	2 Singlet	FAPs
VAT_2M	2987.1	1743	0.9131349	5 Singlet	Immune_cells
VAT_2M	1846.3	1611	1.1360577	6 Singlet	Neurons
VAT_2M	2813.7	1568	10.528718	7 Singlet	Endothelial_cells
VAT_2M	1580.1	1019	0.9489999	7 Singlet	Endothelial_cells
VAT_2M	7566.1	2891	0.0028853	0 Singlet	Adipocyte
VAT_2M	5984.8	2695	0.0715597	0 Singlet	Adipocyte
VAT_2M	5138.2	2265	0.3583445	0 Singlet	Adipocyte
VAT_2M	1538.3	1111	1.208605	3 Singlet	FAPs
VAT_2M	2112	1308	0.0806439	0 Singlet	Adipocyte

VAT_2M	5322.8	2264	0.0328653	0	Singlet	Adipocyte
VAT_2M	2026.6	1256	0.1355888	0	Singlet	Adipocyte
VAT_2M	1075.7	866	3.1180994	6	Singlet	Neurons
VAT_2M	1522.2	1125	1.0485278	6	Singlet	Neurons
VAT_2M	1203.4	890	0.3937251	7	Singlet	Endothelial_cells
VAT_2M	1044.8	817	0.1784173	6	Singlet	Neurons
VAT_2M	2610	1786	1.0960135	2	Singlet	FAPs
VAT_2M	4026	2021	0.4344096	0	Singlet	Adipocyte
VAT_2M	1719.5	1133	7.6403039	5	Singlet	Immune_cells
VAT_2M	2240.3	1254	4.4043983	5	Singlet	Immune_cells
VAT_2M	7649.9	2886	0	0	Singlet	Adipocyte
VAT_2M	9068.3	2919	0.0762217	0	Singlet	Adipocyte
VAT_2M	1954.2	1466	1.6771623	2	Singlet	FAPs
VAT_2M	6695.2	2640	0.1010848	0	Singlet	Adipocyte
VAT_2M	1484.2	1124	1.671494	0	Singlet	Adipocyte
VAT_2M	1733.2	1312	3.0080476	11	Singlet	Smooth_muscle_cells
VAT_2M	1138.9	901	0.7066267	11	Singlet	Smooth_muscle_cells
VAT_2M	6333.2	2635	0.3959959	0	Singlet	Adipocyte
VAT_2M	2573.9	1555	0.1496745	0	Singlet	Adipocyte
VAT_2M	13752	3959	0.0372283	0	Singlet	Adipocyte
VAT_2M	7646.3	2867	0.0037674	0	Singlet	Adipocyte
VAT_2M	1126.9	913	1.6720984	6	Singlet	Neurons
VAT_2M	1270.7	922	2.1437994	3	Singlet	FAPs
VAT_2M	8336.2	2966	0.0245986	0	Singlet	Adipocyte
VAT_2M	10780	3318	0.0526605	0	Singlet	Adipocyte
VAT_2M	2580.9	1598	1.7539067	5	Singlet	Immune_cells
VAT_2M	1241.8	929	0.6215259	7	Singlet	Endothelial_cells
VAT_2M	2178.1	1475	0.6451321	7	Singlet	Endothelial_cells
VAT_2M	4466.9	2105	1.5796936	0	Singlet	Adipocyte
VAT_2M	4468.4	2079	0.0382186	0	Singlet	Adipocyte
VAT_2M	2109.1	1496	0.3655768	6	Singlet	Neurons
VAT_2M	1363.5	959	2.651622	3	Singlet	FAPs
VAT_2M	1900.4	1399	0.1446096	2	Singlet	FAPs
VAT_2M	1055.9	748	5.1915542	9	Singlet	Mesothelial
VAT_2M	1897.1	1108	3.6969565	2	Singlet	FAPs
VAT_2M	1901.2	1204	0.2974593	0	Singlet	Adipocyte
VAT_2M	2009.8	1330	0.7268792	0	Singlet	Adipocyte
VAT_2M	1359.8	1045	3.1665315	8	Singlet	Mesothelial
VAT_2M	2621.5	1436	0.2246147	0	Singlet	Adipocyte
VAT_2M	1934.7	1322	0.8843857	2	Singlet	FAPs
VAT_2M	8898.6	3202	0.0146583	0	Singlet	Adipocyte
VAT_2M	3027.7	1900	0.9645193	5	Singlet	Immune_cells
VAT_2M	2873.4	1637	0.3228971	5	Singlet	Immune_cells
VAT_2M	1843.6	1317	1.3408754	7	Singlet	Endothelial_cells
VAT_2M	7190.1	2808	0.3441276	0	Singlet	Adipocyte
VAT_2M	1190.6	927	1.7245006	2	Singlet	FAPs
VAT_2M	4843	2380	0.3045249	0	Singlet	Adipocyte
VAT_2M	2535.6	1491	2.0292415	5	Singlet	Immune_cells
VAT_2M	6234.4	2468	0.0066262	0	Singlet	Adipocyte
VAT_2M	2426.7	1550	1.9280459	2	Singlet	FAPs
VAT_2M	1728.7	1184	0.0011353	3	Singlet	FAPs

VAT_2M	1941.1	1058	0.1252666	0	Singlet	Adipocyte
VAT_2M	3938.8	2216	0.193446	0	Singlet	Adipocyte
VAT_2M	1857.5	1222	2.5186102	0	Singlet	Adipocyte
VAT_2M	2699.6	1489	0.2895889	0	Singlet	Adipocyte
VAT_2M	1625.6	975	0	0	Singlet	Adipocyte
VAT_2M	2523.4	1656	0.2608193	6	Singlet	Neurons
VAT_2M	1876	1265	0.9759954	3	Singlet	FAPs
VAT_2M	3028.5	1653	2.0643231	3	Singlet	FAPs
VAT_2M	1499.3	1075	2.0515489	5	Singlet	Immune_cells
VAT_2M	1382.2	959	1.0008398	9	Singlet	Mesothelial
VAT_2M	3946.3	1983	0.0659111	0	Singlet	Adipocyte
VAT_2M	1975	1281	1.1093698	3	Singlet	FAPs
VAT_2M	1902.9	1250	2.179262	2	Singlet	FAPs
VAT_2M	5034.4	2449	0.9185267	0	Singlet	Adipocyte
VAT_2M	1350.7	906	1.4750436	7	Singlet	Endothelial_cells
VAT_2M	7997.5	3040	0.0322154	0	Singlet	Adipocyte
VAT_2M	1992.7	1479	2.048946	6	Singlet	Neurons
VAT_2M	1990.3	1200	0.8505372	0	Singlet	Adipocyte
VAT_2M	1876.8	1118	0.3480653	3	Singlet	FAPs
VAT_2M	3652.2	2027	0.1986801	0	Singlet	Adipocyte
VAT_2M	1894.2	1401	1.2723159	5	Singlet	Immune_cells
VAT_2M	2098.7	1286	0.3597005	2	Singlet	FAPs
VAT_2M	2874.4	1649	0.3478151	3	Singlet	FAPs
VAT_2M	1011.8	759	1.6945205	9	Singlet	Mesothelial
VAT_2M	1605	1144	1.6556779	2	Singlet	FAPs
VAT_2M	1560.1	1292	1.3803764	9	Singlet	Mesothelial
VAT_2M	6855.1	2679	0.678075	0	Singlet	Adipocyte
VAT_2M	1276.6	1028	0	10	Singlet	Immune_cells
VAT_2M	1229	841	3.2879032	5	Singlet	Immune_cells
VAT_2M	5904.7	2485	0.2054448	0	Singlet	Adipocyte
VAT_2M	4445.1	1974	0.0259014	0	Singlet	Adipocyte
VAT_2M	1265.2	882	2.4571338	9	Singlet	Mesothelial
VAT_2M	4153.4	2116	0.2767716	0	Singlet	Adipocyte
VAT_2M	5249.5	2431	0.0178501	0	Singlet	Adipocyte
VAT_2M	2293.3	1275	0.1323715	0	Singlet	Adipocyte
VAT_2M	1402.2	1050	0.7347654	7	Singlet	Endothelial_cells
VAT_2M	4133.6	1888	0	0	Singlet	Adipocyte
VAT_2M	3123	1836	0.132144	0	Singlet	Adipocyte
VAT_2M	3047.9	1888	0.1745265	5	Singlet	Immune_cells
VAT_2M	2465.1	1398	1.1036933	0	Singlet	Adipocyte
VAT_2M	4143.4	2077	0.0323188	0	Singlet	Adipocyte
VAT_2M	5122.6	2372	0.0159191	0	Singlet	Adipocyte
VAT_2M	1029	787	1.465697	5	Singlet	Immune_cells
VAT_2M	2589.1	1627	2.5826133	3	Singlet	FAPs
VAT_2M	2047.6	1313	0.152919	6	Singlet	Neurons
VAT_2M	1837.8	1339	0.2414558	3	Singlet	FAPs
VAT_2M	1215.4	872	2.5832702	3	Singlet	FAPs
VAT_2M	4473.9	2223	0.0446568	0	Singlet	Adipocyte
VAT_2M	2274	1371	0.0333075	2	Singlet	FAPs
VAT_2M	1294	1018	1.8043598	5	Singlet	Immune_cells
VAT_2M	2037.1	1384	0.8439801	2	Singlet	FAPs

VAT_2M	1713.8	1252	0.1088812	2	Singlet	FAPs
VAT_2M	5118.7	2429	0	0	Singlet	Adipocyte
VAT_2M	1962.2	1362	0.5883561	3	Singlet	FAPs
VAT_2M	2825.1	1693	0.2854687	2	Singlet	FAPs
VAT_2M	2409.6	1464	1.3682154	5	Singlet	Immune_cells
VAT_2M	3111.4	1738	0	0	Singlet	Adipocyte
VAT_2M	4901.6	2268	1.3045889	0	Singlet	Adipocyte
VAT_2M	1126.9	826	2.0027837	5	Singlet	Immune_cells
VAT_2M	1854.1	1317	3.6957929	3	Singlet	FAPs
VAT_2M	4367.8	2140	1.2053827	0	Singlet	Adipocyte
VAT_2M	2768.5	1575	0.4353326	7	Singlet	Endothelial_cells
VAT_2M	2068.2	1398	1.7142646	3	Singlet	FAPs
VAT_2M	1377.1	897	0.1643666	5	Singlet	Immune_cells
VAT_2M	8586.1	3265	0	0	Singlet	Adipocyte
VAT_2M	4010.8	2003	0.1733776	2	Singlet	FAPs
VAT_2M	2003.1	1381	1.5337836	2	Singlet	FAPs
VAT_2M	1731.3	1315	2.6898463	1	Singlet	Immune_cells
VAT_2M	5187	2310	0.0463257	0	Singlet	Adipocyte
VAT_2M	2905.2	1574	1.7745616	0	Singlet	Adipocyte
VAT_2M	2397.7	1503	0.8832371	5	Singlet	Immune_cells
VAT_2M	1667.6	1175	2.3556887	9	Singlet	Mesothelial
VAT_2M	5999.5	2632	0.0420402	0	Singlet	Adipocyte
VAT_2M	1533.9	1158	1.4210902	3	Singlet	FAPs
VAT_2M	1911.1	1417	3.1911072	6	Singlet	Neurons
VAT_2M	1081.5	785	1.9239235	3	Singlet	FAPs
VAT_2M	9128.7	3226	0.0028906	0	Singlet	Adipocyte
VAT_2M	10153	3374	0.021211	0	Singlet	Adipocyte
VAT_2M	11251	3606	1.8496316	0	Singlet	Adipocyte
VAT_2M	2238.3	1442	0.9894032	0	Singlet	Adipocyte
VAT_2M	1360.4	902	1.627601	3	Singlet	FAPs
VAT_2M	2139.7	1413	1.0483911	4	Singlet	Immune_cells
VAT_2M	10787	3494	0.2106889	0	Singlet	Adipocyte
VAT_2M	2673.7	1538	0.0240746	0	Singlet	Adipocyte
VAT_2M	2624.3	1430	0.1832666	0	Singlet	Adipocyte
VAT_2M	3802.6	1725	0.3448388	0	Singlet	Adipocyte
VAT_2M	1196.1	814	1.2417253	11	Singlet	Smooth_muscle_cells
VAT_2M	1711.3	1186	1.0399285	2	Singlet	FAPs
VAT_2M	6886.6	2694	0.1003801	0	Singlet	Adipocyte
VAT_2M	1412	975	6.6391749	6	Singlet	Neurons
VAT_2M	5444.5	2170	3.3499955	0	Singlet	Adipocyte
VAT_2M	7346.1	2834	0.021958	0	Singlet	Adipocyte
VAT_2M	9205.3	3328	0.0015068	0	Singlet	Adipocyte
VAT_2M	2420.4	1500	0.1398581	6	Singlet	Neurons
VAT_2M	2783	1670	0.8793351	3	Singlet	FAPs
VAT_2M	2066.2	1400	0.8019738	6	Singlet	Neurons
VAT_2M	3695.7	1944	0.3550205	6	Singlet	Neurons
VAT_2M	3011.6	1852	0.036943	6	Singlet	Neurons
VAT_2M	1474.6	1288	1.3969093	6	Singlet	Neurons
VAT_2M	7166.4	2875	0.0689084	0	Singlet	Adipocyte
VAT_2M	2178	1347	0.2341948	0	Singlet	Adipocyte
VAT_2M	1048.2	746	0.1973193	7	Singlet	Endothelial_cells

VAT_2M	1960.2	1156	0.4298084	0	Singlet	Adipocyte
VAT_2M	2138.4	1338	0.4028488	0	Singlet	Adipocyte
VAT_2M	2162.5	1385	1.1232561	0	Singlet	Adipocyte
VAT_2M	1263.3	951	0.0434641	3	Singlet	FAPs
VAT_2M	1303.4	943	0.6752707	2	Singlet	FAPs
VAT_2M	6520.8	2425	2.3671998	0	Singlet	Adipocyte
VAT_2M	12624	3544	0.0439969	0	Singlet	Adipocyte
VAT_2M	7138.7	2724	0.0225324	0	Singlet	Adipocyte
VAT_2M	1299.4	1103	0.7436403	8	Singlet	Mesothelial
VAT_2M	10167	3440	0.0259148	0	Singlet	Adipocyte
VAT_2M	1648.4	1040	2.939912	5	Singlet	Immune_cells
VAT_2M	4091.2	2034	0.0159743	0	Singlet	Adipocyte
VAT_2M	1721.3	1289	0.5571663	6	Singlet	Neurons
VAT_2M	2685.8	1712	0.7438702	5	Singlet	Immune_cells
VAT_2M	6030.9	2533	0.0433282	0	Singlet	Adipocyte
VAT_2M	5913.1	2383	0.4956171	0	Singlet	Adipocyte
VAT_2M	1876.9	910	7.7129326	3	Singlet	FAPs
VAT_2M	7808.4	2755	0.0307326	0	Singlet	Adipocyte
VAT_2M	3612.5	1625	1.5062422	0	Singlet	Adipocyte
VAT_2M	3203.7	1747	1.1313938	3	Singlet	FAPs
VAT_2M	1797.1	1425	1.0459838	5	Singlet	Immune_cells
VAT_2M	1490.5	1148	1.8828348	5	Singlet	Immune_cells
VAT_2M	1011.3	739	2.2686842	5	Singlet	Immune_cells
VAT_2M	5496.9	2504	0.104917	0	Singlet	Adipocyte
VAT_2M	6744.1	2666	0.177168	0	Singlet	Adipocyte
VAT_2M	7161.2	2892	1.5867766	0	Singlet	Adipocyte
VAT_2M	2189.2	1416	0.4781582	2	Singlet	FAPs
VAT_2M	1474.4	1012	1.0189484	3	Singlet	FAPs
VAT_2M	3859.1	1924	0.2599935	0	Singlet	Adipocyte
VAT_2M	2095.8	1398	1.0688136	6	Singlet	Neurons
VAT_2M	5232.7	2371	0.2356186	0	Singlet	Adipocyte
VAT_2M	2641.1	1559	2.3948181	0	Singlet	Adipocyte
VAT_2M	1693.7	1129	0.0706991	0	Singlet	Adipocyte
VAT_2M	2027.8	1384	0.1418142	11	Singlet	Smooth_muscle_cells
VAT_2M	3602.3	1818	1.9399102	0	Singlet	Adipocyte
VAT_2M	2679.6	1676	0.4614726	0	Singlet	Adipocyte
VAT_2M	1774.9	1230	2.8423931	5	Singlet	Immune_cells
VAT_2M	4925.6	2208	0.0101701	0	Singlet	Adipocyte
VAT_2M	9609.7	3322	0.5141297	0	Singlet	Adipocyte
VAT_2M	1569.8	1041	0.147726	3	Singlet	FAPs
VAT_2M	3738.3	1893	0.1403896	0	Singlet	Adipocyte
VAT_2M	2142.9	1679	5.0417935	11	Singlet	Smooth_muscle_cells
VAT_2M	2930.1	1787	3.7613934	6	Singlet	Neurons
VAT_2M	1018.8	825	0.803028	2	Singlet	FAPs
VAT_2M	4604.4	2308	0.0127195	0	Singlet	Adipocyte
VAT_2M	2479.1	1448	0.5044002	0	Singlet	Adipocyte
VAT_2M	2180.5	1393	0.1229131	7	Singlet	Endothelial_cells
VAT_2M	6317.8	2727	0.0710449	0	Singlet	Adipocyte
VAT_2M	5419	2611	7.0895777	3	Singlet	FAPs
VAT_2M	5989.7	2558	0.3403589	0	Singlet	Adipocyte
VAT_2M	1838.6	1236	0.5612671	2	Singlet	FAPs

VAT_2M	7786.2	2960	0	0 Singlet	Adipocyte
VAT_2M	1047.1	701	4.7427069	6 Singlet	Neurons
VAT_2M	1123.9	866	1.6594137	3 Singlet	FAPs
VAT_2M	3867.9	1961	0.5523006	0 Singlet	Adipocyte
VAT_2M	2860.5	1598	0.450639	5 Singlet	Immune_cells
VAT_2M	2292.6	1399	0.0232042	0 Singlet	Adipocyte
VAT_2M	5528.3	2421	0.0632901	0 Singlet	Adipocyte
VAT_2M	1058.2	870	2.636115	6 Singlet	Neurons
VAT_2M	5148.7	2484	0.1004266	0 Singlet	Adipocyte
VAT_2M	1250.7	717	1.0780952	3 Singlet	FAPs
VAT_2M	1384.5	1013	2.0483987	2 Singlet	FAPs
VAT_2M	8920.2	3060	0.0104003	0 Singlet	Adipocyte
VAT_2M	2556.9	1682	0.7807231	6 Singlet	Neurons
VAT_2M	1012.8	914	0.839166	6 Singlet	Neurons
VAT_2M	4634.6	2223	0.1287607	0 Singlet	Adipocyte
VAT_2M	3034.8	1926	0.6028787	3 Singlet	FAPs
VAT_2M	7109.3	2947	1.0809625	0 Singlet	Adipocyte
VAT_2M	3619.3	1756	0.1753254	0 Singlet	Adipocyte
VAT_2M	2957.7	1529	0.3717903	0 Singlet	Adipocyte
VAT_2M	2411.8	1397	0.0849296	0 Singlet	Adipocyte
VAT_2M	1398	974	0.9433546	3 Singlet	FAPs
VAT_2M	8536	3007	0.4587935	0 Singlet	Adipocyte
VAT_2M	1355.9	1026	1.0025271	7 Singlet	Endothelial_cells
VAT_2M	1163.5	919	3.4372694	5 Singlet	Immune_cells
VAT_2M	2124.2	1352	0.0348189	2 Singlet	FAPs
VAT_2M	1074.7	746	3.1349536	3 Singlet	FAPs
VAT_2M	12928	4018	0.0245679	0 Singlet	Adipocyte
VAT_2M	7983.4	3016	0.0564974	0 Singlet	Adipocyte
VAT_2M	1608.5	1095	0	3 Singlet	FAPs
VAT_2M	4686.4	2095	0.011542	0 Singlet	Adipocyte
VAT_2M	1849.7	1268	1.9575428	9 Singlet	Mesothelial
VAT_2M	1750.6	1139	0.5093238	7 Singlet	Endothelial_cells
VAT_2M	2196.9	1369	0.9492952	0 Singlet	Adipocyte
VAT_2M	4573.6	2163	0.0851346	0 Singlet	Adipocyte
VAT_2M	8740.7	3271	0.0033194	0 Singlet	Adipocyte
VAT_2M	1450.4	1057	0.21861	5 Singlet	Immune_cells
VAT_2M	3385.5	1766	0.0187551	0 Singlet	Adipocyte
VAT_2M	5105.9	2428	0.0919865	0 Singlet	Adipocyte
VAT_2M	3406.6	1902	4.4360942	0 Singlet	Adipocyte
VAT_2M	2973.5	1570	0.0795821	0 Singlet	Adipocyte
VAT_2M	7248.8	2873	0.0173671	0 Singlet	Adipocyte
VAT_2M	6088.9	2453	0.0055746	0 Singlet	Adipocyte
VAT_2M	7443	2910	0.076903	0 Singlet	Adipocyte
VAT_2M	1133.3	871	0.5055376	6 Singlet	Neurons
VAT_2M	4241	2015	0.1599659	0 Singlet	Adipocyte
VAT_2M	2372.8	1509	0.8576077	6 Singlet	Neurons
VAT_2M	3385.5	1780	0.945097	0 Singlet	Adipocyte
VAT_2M	3709	1800	0.0443197	0 Singlet	Adipocyte
VAT_2M	1426.4	993	0.0160282	5 Singlet	Immune_cells
VAT_2M	2139.6	1389	0.3573099	0 Singlet	Adipocyte
VAT_2M	5934.7	2396	0.0215214	0 Singlet	Adipocyte

VAT_2M	2630.6	1343	0.7225193	0	Singlet	Adipocyte
VAT_2M	1012.2	900	0.8771472	6	Singlet	Neurons
VAT_2M	6364.4	2542	0.0721123	0	Singlet	Adipocyte
VAT_2M	2732.1	1692	0.6108086	0	Singlet	Adipocyte
VAT_2M	6305.2	2676	0.2932068	0	Singlet	Adipocyte
VAT_2M	1024.8	757	2.0551817	2	Singlet	FAPs
VAT_2M	2572.2	1571	0.4486	0	Singlet	Adipocyte
VAT_2M	1057.1	912	1.7356373	9	Singlet	Mesothelial
VAT_2M	4847.3	2102	0.7555746	0	Singlet	Adipocyte
VAT_2M	3550.2	2004	0	0	Singlet	Adipocyte
VAT_2M	7886.2	3038	0.0008544	0	Singlet	Adipocyte
VAT_2M	6055.9	2367	0.2021344	0	Singlet	Adipocyte
VAT_2M	5753.7	2543	0.0472048	0	Singlet	Adipocyte
VAT_2M	1649.5	1076	0.5994979	6	Singlet	Neurons
VAT_2M	7919.3	3078	0.0740559	0	Singlet	Adipocyte
VAT_2M	3087.6	1574	0	0	Singlet	Adipocyte
VAT_2M	2104	1599	2.4487714	7	Singlet	Endothelial_cells
VAT_2M	2101.8	1263	0.1790494	0	Singlet	Adipocyte
VAT_2M	1624	1330	3.6419201	8	Singlet	Mesothelial
VAT_2M	2017.8	1219	0.946135	3	Singlet	FAPs
VAT_2M	1748.4	1417	0.3115084	3	Singlet	FAPs
VAT_2M	1715.6	1200	0.9766979	6	Singlet	Neurons
VAT_2M	3125.8	1654	0.670168	0	Singlet	Adipocyte
VAT_2M	1656.5	1275	2.8973331	1	Singlet	Immune_cells
VAT_2M	2310.9	1567	0.634553	5	Singlet	Immune_cells
VAT_2M	1470.3	1233	1.3651196	6	Singlet	Neurons
VAT_2M	1878.8	1209	2.1760044	2	Singlet	FAPs
VAT_2M	6358.6	2719	0.0285153	0	Singlet	Adipocyte
VAT_2M	5559.5	2586	0.0236349	0	Singlet	Adipocyte
VAT_2M	3401.8	1926	0.2620667	0	Singlet	Adipocyte
VAT_2M	1483.9	1019	2.0711531	9	Singlet	Mesothelial
VAT_2M	3284	1765	0.0509138	0	Singlet	Adipocyte
VAT_2M	1069.9	839	0.4650999	7	Singlet	Endothelial_cells
VAT_2M	1316	1055	2.081129	2	Singlet	FAPs
VAT_2M	7832.1	2991	0.0285615	0	Singlet	Adipocyte
VAT_2M	9094.3	3092	0.0254552	0	Singlet	Adipocyte
VAT_2M	1051.3	827	2.3164956	6	Singlet	Neurons
VAT_2M	2480.3	1682	0.6624402	7	Singlet	Endothelial_cells
VAT_2M	1314.6	1107	1.2105113	6	Singlet	Neurons
VAT_2M	2078.8	1173	1.1384665	0	Singlet	Adipocyte
VAT_2M	12729	3767	0.0432146	0	Singlet	Adipocyte
VAT_2M	1271	870	1.1423018	11	Singlet	Smooth_muscle_cells
VAT_2M	1618.5	1178	0.4993387	2	Singlet	FAPs
VAT_2M	1172.5	943	1.1908272	8	Singlet	Mesothelial
VAT_2M	8778.7	3197	0.0074243	0	Singlet	Adipocyte
VAT_2M	1140.9	938	0.6571504	1	Singlet	Immune_cells
VAT_2M	5082.5	2600	0.4815742	2	Singlet	FAPs
VAT_2M	1131.3	885	0.6100579	6	Singlet	Neurons
VAT_2M	1566.9	1216	0.5158686	2	Singlet	FAPs
VAT_2M	4158.4	2018	0.3868925	0	Singlet	Adipocyte
VAT_2M	1219	1007	0.2730009	11	Singlet	Smooth_muscle_cells

VAT_2M	4508.8	2080	0.0208313	0	Singlet	Adipocyte
VAT_2M	1301	994	3.5102507	6	Singlet	Neurons
VAT_2M	2558.3	1426	0.0978737	0	Singlet	Adipocyte
VAT_2M	1966.4	1292	0.80211	3	Singlet	FAPs
VAT_2M	1932.7	1183	0.88098	2	Singlet	FAPs
VAT_2M	1770.2	1124	2.4815043	6	Singlet	Neurons
VAT_2M	1072.7	735	1.1952081	3	Singlet	FAPs
VAT_2M	5611.4	2435	0.0108446	0	Singlet	Adipocyte
VAT_2M	2120.6	1519	2.4503917	2	Singlet	FAPs
VAT_2M	12167	3443	0.1445847	0	Singlet	Adipocyte
VAT_2M	1529.4	1099	2.7203328	3	Singlet	FAPs
VAT_2M	4481.6	1954	0.0449172	0	Singlet	Adipocyte
VAT_2M	9193.6	3052	0.0537028	0	Singlet	Adipocyte
VAT_2M	3147.6	1668	0.5432391	0	Singlet	Adipocyte
VAT_2M	1419.1	910	3.4358454	6	Singlet	Neurons
VAT_2M	6536	2802	0.4519121	2	Singlet	FAPs
VAT_2M	4641.8	2191	0.140045	0	Singlet	Adipocyte
VAT_2M	2234.5	1391	1.881889	5	Singlet	Immune_cells
VAT_2M	9266.6	3353	0.3828718	0	Singlet	Adipocyte
VAT_2M	1550.4	1066	1.0349343	5	Singlet	Immune_cells
VAT_2M	6828.8	2936	0.2229235	0	Singlet	Adipocyte
VAT_2M	2006.2	1192	0.2724427	0	Singlet	Adipocyte
VAT_2M	7580	2722	0.0409503	0	Singlet	Adipocyte
VAT_2M	1656.6	1157	1.4862711	9	Singlet	Mesothelial
VAT_2M	2259.2	1456	2.7847966	2	Singlet	FAPs
VAT_2M	1436.8	1088	0.784061	6	Singlet	Neurons
VAT_2M	2297.6	1373	1.2520423	0	Singlet	Adipocyte
VAT_2M	2933.8	1770	0.4217116	3	Singlet	FAPs
VAT_2M	1389	1019	1.1835376	3	Singlet	FAPs
VAT_2M	1231.7	966	1.6642948	1	Singlet	Immune_cells
VAT_2M	2234.4	1360	0.9774895	5	Singlet	Immune_cells
VAT_2M	1892.3	1103	0.4373235	6	Singlet	Neurons
VAT_2M	7397	2962	0.0324712	0	Singlet	Adipocyte
VAT_2M	1836.4	1283	3.1000893	5	Singlet	Immune_cells
VAT_2M	5472.3	2591	0.0230532	0	Singlet	Adipocyte
VAT_2M	1430.1	1022	0.7091533	3	Singlet	FAPs
VAT_2M	1567.5	1001	0.3147214	0	Singlet	Adipocyte
VAT_2M	13153	3441	0.0254799	0	Singlet	Adipocyte
VAT_2M	6276	2559	0.0710324	0	Singlet	Adipocyte
VAT_2M	4602.4	2139	2.9915182	0	Singlet	Adipocyte
VAT_2M	7274.4	2811	0.0489098	0	Singlet	Adipocyte
VAT_2M	2206.5	1354	0.5186356	7	Singlet	Endothelial_cells
VAT_2M	1381	1113	2.1412082	6	Singlet	Neurons
VAT_2M	2322.9	1325	0.2233417	0	Singlet	Adipocyte
VAT_2M	1767.5	1017	1.1790767	3	Singlet	FAPs
VAT_2M	1956.2	1331	2.1772413	5	Singlet	Immune_cells
VAT_2M	8317.6	2954	0.0515027	0	Singlet	Adipocyte
VAT_2M	1547.2	1117	4.2408648	2	Singlet	FAPs
VAT_2M	1331.6	972	0.2607993	3	Singlet	FAPs
VAT_2M	2460.8	1618	0.6528899	2	Singlet	FAPs
VAT_2M	2834.3	1656	1.0533388	0	Singlet	Adipocyte

VAT_2M	5675.9	2392	0.0594006	0	Singlet	Adipocyte
VAT_2M	1758.5	1177	1.900323	2	Singlet	FAPs
VAT_2M	7596.5	2892	0.0574686	0	Singlet	Adipocyte
VAT_2M	1584.5	1019	0.6306225	7	Singlet	Endothelial_cells
VAT_2M	1375.2	929	0.0880598	7	Singlet	Endothelial_cells
VAT_2M	1339.1	979	1.3160978	2	Singlet	FAPs
VAT_2M	11648	3685	0.0007602	0	Singlet	Adipocyte
VAT_2M	1073.3	851	1.4947761	6	Singlet	Neurons
VAT_2M	9310.7	3081	0.0357132	0	Singlet	Adipocyte
VAT_2M	1383.9	931	2.1105535	6	Singlet	Neurons
VAT_2M	5365.2	2438	0.6425838	0	Singlet	Adipocyte
VAT_2M	7837	2996	0	0	Singlet	Adipocyte
VAT_2M	7706.9	3086	0.065051	0	Singlet	Adipocyte
VAT_2M	5653.7	2469	0.0054444	0	Singlet	Adipocyte
VAT_2M	8868.9	3356	0.2679868	0	Singlet	Adipocyte
VAT_2M	3521.2	1890	0.1453067	0	Singlet	Adipocyte
VAT_2M	1804.8	1254	0.8998071	3	Singlet	FAPs
VAT_2M	1741.8	1146	1.348104	8	Singlet	Mesothelial
VAT_2M	1998.2	1112	0.5632308	0	Singlet	Adipocyte
VAT_2M	3014.3	1666	4.570493	3	Singlet	FAPs
VAT_2M	1831.4	1149	0.0161001	0	Singlet	Adipocyte
VAT_2M	4627.9	2350	0.6894699	3	Singlet	FAPs
VAT_2M	4136.5	1964	0.6054193	0	Singlet	Adipocyte
VAT_2M	7690.1	2850	0.2845204	0	Singlet	Adipocyte
VAT_2M	4407.1	2175	0.3113448	0	Singlet	Adipocyte
VAT_2M	2371.5	1523	0.3101575	2	Singlet	FAPs
VAT_2M	6061.4	2839	0.164592	2	Singlet	FAPs
VAT_2M	2785.8	1759	0.4469233	6	Singlet	Neurons
VAT_2M	4996.4	2211	0.0098771	0	Singlet	Adipocyte
VAT_2M	3292.3	1722	7.5712055	7	Singlet	Endothelial_cells
VAT_2M	3808.4	2149	0.9441034	3	Singlet	FAPs
VAT_2M	1758.7	1120	3.0826998	3	Singlet	FAPs
VAT_2M	5519.8	2579	0.0265229	0	Singlet	Adipocyte
VAT_2M	2158	1184	4.0290426	0	Singlet	Adipocyte
VAT_2M	1385.1	1009	0.1030843	2	Singlet	FAPs
VAT_2M	1384.5	1121	2.671804	2	Singlet	FAPs
VAT_2M	2148.4	1324	1.5070697	2	Singlet	FAPs
VAT_2M	3452	1998	2.8055676	2	Singlet	FAPs
VAT_2M	4302.6	2081	0.1200665	0	Singlet	Adipocyte
VAT_2M	10294	3394	0	0	Singlet	Adipocyte
VAT_2M	5789.4	2821	1.1882176	2	Singlet	FAPs
VAT_2M	2793.4	1697	1.6775035	0	Singlet	Adipocyte
VAT_2M	1067.8	824	1.2746539	0	Singlet	Adipocyte
VAT_2M	1005.8	731	0.1732571	6	Singlet	Neurons
VAT_2M	2511.2	1457	0.3631571	0	Singlet	Adipocyte
VAT_2M	9514.2	3220	0.0042627	0	Singlet	Adipocyte
VAT_2M	4334.3	2371	1.0342319	3	Singlet	FAPs
VAT_2M	1819.4	1322	2.3111892	2	Singlet	FAPs
VAT_2M	2718.1	1707	0.8752757	3	Singlet	FAPs
VAT_2M	1238.7	828	2.4432654	1	Singlet	Immune_cells
VAT_2M	2927.4	1881	1.4085298	3	Singlet	FAPs

VAT_2M	1044.7	815	5.9790715	6	Singlet	Neurons
VAT_2M	2305.6	1313	0.1836729	0	Singlet	Adipocyte
VAT_2M	4932.9	2154	0.0387833	0	Singlet	Adipocyte
VAT_2M	1024.4	847	0.5939431	6	Singlet	Neurons
VAT_2M	1406.5	1058	0.6646223	3	Singlet	FAPs
VAT_2M	2461.8	1619	3.7530721	5	Singlet	Immune_cells
VAT_2M	3140.7	1630	0.3782227	0	Singlet	Adipocyte
VAT_2M	9759.9	3332	0.0315257	0	Singlet	Adipocyte
VAT_2M	4033.7	1837	0.0217602	0	Singlet	Adipocyte
VAT_2M	1722.3	1188	0.9011321	5	Singlet	Immune_cells
VAT_2M	1845.4	1148	0.7095061	0	Singlet	Adipocyte
VAT_2M	1401.3	987	0.4027666	5	Singlet	Immune_cells
VAT_2M	1100.2	963	1.2084426	6	Singlet	Neurons
VAT_2M	3223.2	1659	0.9433089	0	Singlet	Adipocyte
VAT_2M	4678.5	2268	0.4845047	0	Singlet	Adipocyte
VAT_2M	1456.5	1025	0.4844932	5	Singlet	Immune_cells
VAT_2M	3800	1986	0.3062655	0	Singlet	Adipocyte
VAT_2M	8547.5	3103	0.2861348	0	Singlet	Adipocyte
VAT_2M	7736	2991	0.0239904	0	Singlet	Adipocyte
VAT_2M	4337.7	2106	0	0	Singlet	Adipocyte
VAT_2M	1116	803	4.3688744	9	Singlet	Mesothelial
VAT_2M	3149.4	1756	0.15062	0	Singlet	Adipocyte
VAT_2M	1158.6	890	0.6817206	3	Singlet	FAPs
VAT_2M	1586.7	1053	1.0583042	3	Singlet	FAPs
VAT_2M	5273.7	2344	0.0099651	0	Singlet	Adipocyte
VAT_2M	5127.5	2549	2.9102118	2	Singlet	FAPs
VAT_2M	8165.6	3099	0.0590091	0	Singlet	Adipocyte
VAT_2M	1058.3	800	3.3076876	2	Singlet	FAPs
VAT_2M	12341	3776	0	0	Singlet	Adipocyte
VAT_2M	10804	3412	0.0142741	0	Singlet	Adipocyte
VAT_2M	3886.4	1918	0.20941	0	Singlet	Adipocyte
VAT_2M	1795.2	1146	0.0018661	2	Singlet	FAPs
VAT_2M	1204.5	838	2.4066294	0	Singlet	Adipocyte
VAT_2M	2977.2	1784	0.091721	0	Singlet	Adipocyte
VAT_2M	4233.6	2072	0.5677815	0	Singlet	Adipocyte
VAT_2M	9186.7	3272	0.116713	0	Singlet	Adipocyte
VAT_2M	2750.9	1795	1.9989149	0	Singlet	Adipocyte
VAT_2M	1877.1	1221	1.3472869	6	Singlet	Neurons
VAT_2M	1182.4	970	0	6	Singlet	Neurons
VAT_2M	4219.4	2421	2.4989958	2	Singlet	FAPs
VAT_2M	1016.1	644	10.628465	7	Singlet	Endothelial_cells
VAT_2M	2491.8	1608	0.1202582	0	Singlet	Adipocyte
VAT_2M	1181.3	939	1.4884817	2	Singlet	FAPs
VAT_2M	2935.9	1808	0.6011872	3	Singlet	FAPs
VAT_2M	1195.6	996	0.4980612	5	Singlet	Immune_cells
VAT_2M	1524.9	1140	0.284522	3	Singlet	FAPs
VAT_2M	1578	1106	4.0189621	2	Singlet	FAPs
VAT_2M	1801.7	1219	1.1166831	0	Singlet	Adipocyte
VAT_2M	2432.7	1609	1.2453605	9	Singlet	Mesothelial
VAT_2M	6442.3	2617	0.3690788	0	Singlet	Adipocyte
VAT_2M	1503.2	1103	0.4087856	2	Singlet	FAPs

VAT_2M	7281	2899	0.0731236	0	Singlet	Adipocyte
VAT_2M	4869.2	2304	0.0182858	0	Singlet	Adipocyte
VAT_2M	2572.1	1393	1.2165414	0	Singlet	Adipocyte
VAT_2M	1166.9	954	1.485021	6	Singlet	Neurons
VAT_2M	1494.5	1180	0.7801087	7	Singlet	Endothelial_cells
VAT_2M	2954	1703	0.1615217	3	Singlet	FAPs
VAT_2M	5143.3	2369	0.4706581	0	Singlet	Adipocyte
VAT_2M	11133	3368	0.0164079	0	Singlet	Adipocyte
VAT_2M	7497	2962	0.0472281	0	Singlet	Adipocyte
VAT_2M	1881.3	1390	1.2658877	2	Singlet	FAPs
VAT_2M	3383.3	1686	0.0519818	0	Singlet	Adipocyte
VAT_2M	1661.7	1077	3.6271023	0	Singlet	Adipocyte
VAT_2M	1605.2	1049	2.7066814	3	Singlet	FAPs
VAT_2M	5738.3	2428	0.2608495	0	Singlet	Adipocyte
VAT_2M	2207.1	1472	2.2045045	1	Singlet	Immune_cells
VAT_2M	1328.2	832	12.838242	1	Singlet	Immune_cells
VAT_2M	13574	3762	0	0	Singlet	Adipocyte
VAT_2M	2608	1447	1.1439178	0	Singlet	Adipocyte
VAT_2M	12107	3491	0.0182656	0	Singlet	Adipocyte
VAT_2M	2397.4	1315	2.9660656	0	Singlet	Adipocyte
VAT_2M	3210.1	1673	0	0	Singlet	Adipocyte
VAT_2M	1345.8	879	1.6552059	3	Singlet	FAPs
VAT_2M	5833.2	2467	1.6292013	0	Singlet	Adipocyte
VAT_2M	8396.5	3107	0.0487143	0	Singlet	Adipocyte
VAT_2M	1559.5	1026	2.331475	2	Singlet	FAPs
VAT_2M	1827.7	1272	0.8281275	2	Singlet	FAPs
VAT_2M	7582.4	2826	0.0592267	0	Singlet	Adipocyte
VAT_2M	1549.9	1087	0.8416311	1	Singlet	Immune_cells
VAT_2M	4951.3	2390	0.5649694	0	Singlet	Adipocyte
VAT_2M	4405.9	2129	0.1248008	0	Singlet	Adipocyte
VAT_2M	2280.8	1508	0.4239663	2	Singlet	FAPs
VAT_2M	2840.3	1823	3.5641466	7	Singlet	Endothelial_cells
VAT_2M	2889.1	1695	1.1145131	5	Singlet	Immune_cells
VAT_2M	9058	3153	0.1044963	0	Singlet	Adipocyte
VAT_2M	1142.6	941	0.7770572	9	Singlet	Mesothelial
VAT_2M	1461.7	1024	0.6524713	9	Singlet	Mesothelial
VAT_2M	2076.4	1288	1.9841572	0	Singlet	Adipocyte
VAT_2M	7609.3	2860	0.023163	0	Singlet	Adipocyte
VAT_2M	3536	2008	0.4235949	0	Singlet	Adipocyte
VAT_2M	1084.7	913	3.2302945	6	Singlet	Neurons
VAT_2M	3174.4	1687	0.2404275	0	Singlet	Adipocyte
VAT_2M	4421.6	2103	0.0448506	0	Singlet	Adipocyte
VAT_2M	3142.7	1669	0.1944689	2	Singlet	FAPs
VAT_2M	9023.7	2979	0.014776	0	Singlet	Adipocyte
VAT_2M	3781	1915	0.0520525	0	Singlet	Adipocyte
VAT_2M	3158.9	1655	0.164463	0	Singlet	Adipocyte
VAT_2M	7679	2665	0	0	Singlet	Adipocyte
VAT_2M	3429.2	2008	0.3795869	0	Singlet	Adipocyte
VAT_2M	1431.1	1035	1.6728558	3	Singlet	FAPs
VAT_2M	1415	1081	2.5338831	2	Singlet	FAPs
VAT_2M	1037.2	905	0.5614329	4	Singlet	Immune_cells

VAT_2M	3182.3	2085	1.8648156	2	Singlet	FAPs
VAT_2M	1502.7	1209	0.4258736	4	Singlet	Immune_cells
VAT_2M	2316.4	1525	0.3701549	6	Singlet	Neurons
VAT_2M	1565.3	1020	2.7339033	6	Singlet	Neurons
VAT_2M	4736.7	2208	0	0	Singlet	Adipocyte
VAT_2M	1648.8	1024	0.3537021	0	Singlet	Adipocyte
VAT_2M	5535.7	2327	0.0072216	0	Singlet	Adipocyte
VAT_2M	1368.3	830	0.0533716	0	Singlet	Adipocyte
VAT_2M	2166.4	1253	3.6458287	2	Singlet	FAPs
VAT_2M	7657.3	2825	0.0196892	0	Singlet	Adipocyte
VAT_2M	10221	3157	0	0	Singlet	Adipocyte
VAT_2M	2563.1	1493	0.6125385	0	Singlet	Adipocyte
VAT_2M	1770.6	1327	3.218988	10	Singlet	Immune_cells
VAT_2M	7410.7	2844	0.2107196	0	Singlet	Adipocyte
VAT_2M	2519.3	1625	0.3213454	5	Singlet	Immune_cells
VAT_2M	1174.4	978	1.6856562	2	Singlet	FAPs
VAT_2M	2443.3	1643	3.6922192	2	Singlet	FAPs
VAT_2M	2666.9	1516	0.0601392	0	Singlet	Adipocyte
VAT_2M	1099.8	801	2.6897333	2	Singlet	FAPs
VAT_2M	3786.3	1955	0.0868281	0	Singlet	Adipocyte
VAT_2M	5566.6	2571	0.2011266	0	Singlet	Adipocyte
VAT_2M	1175.5	873	2.2014332	8	Singlet	Mesothelial
VAT_2M	2284.3	1455	0.0336346	6	Singlet	Neurons
VAT_2M	1232.6	893	1.0998944	5	Singlet	Immune_cells
VAT_2M	1991.6	1148	0.4128514	0	Singlet	Adipocyte
VAT_2M	1639.8	1214	2.1393206	7	Singlet	Endothelial_cells
VAT_2M	1519.4	1117	1.9602846	5	Singlet	Immune_cells
VAT_2M	1592.4	1282	1.7305861	6	Singlet	Neurons
VAT_2M	1041.6	824	1.208003	7	Singlet	Endothelial_cells
VAT_2M	4272.8	2097	0.8029054	0	Singlet	Adipocyte
VAT_2M	8028.9	3101	0.1470819	0	Singlet	Adipocyte
VAT_2M	4056.2	2336	0.3493057	2	Singlet	FAPs
VAT_2M	1912.9	1095	0.2262051	0	Singlet	Adipocyte
VAT_2M	8026.1	2838	0.0014025	0	Singlet	Adipocyte
VAT_2M	1201.4	859	1.3087375	2	Singlet	FAPs
VAT_2M	2115.1	1330	0.1672107	0	Singlet	Adipocyte
VAT_2M	1497.6	1147	0.4304335	5	Singlet	Immune_cells
VAT_2M	1188.1	903	0.7748784	1	Singlet	Immune_cells
VAT_2M	4967.4	2261	0.2076363	0	Singlet	Adipocyte
VAT_2M	1523.9	1098	0.5947924	4	Singlet	Immune_cells
VAT_2M	1581.5	774	5.1152743	4	Singlet	Immune_cells
VAT_2M	1263.7	975	0.103694	5	Singlet	Immune_cells
VAT_2M	5904	2348	0.1052671	0	Singlet	Adipocyte
VAT_2M	1148.1	794	2.9371438	2	Singlet	FAPs
VAT_2M	1371.1	914	2.7950433	9	Singlet	Mesothelial
VAT_2M	1791.1	1286	2.5992128	2	Singlet	FAPs
VAT_2M	2697.7	1529	2.0842954	0	Singlet	Adipocyte
VAT_2M	2789	1837	3.2837842	3	Singlet	FAPs
VAT_2M	2218.3	1383	0.3518697	5	Singlet	Immune_cells
VAT_2M	2691	1685	0.8515977	3	Singlet	FAPs
VAT_2M	2149.4	1417	1.7947102	0	Singlet	Adipocyte

VAT_2M	6210	2644	0.0085241	0	Singlet	Adipocyte
VAT_2M	8369.5	2933	0.0134706	0	Singlet	Adipocyte
VAT_2M	2923.8	1694	1.9562916	5	Singlet	Immune_cells
VAT_2M	1075.5	836	2.2405946	2	Singlet	FAPs
VAT_2M	4947.8	2432	0.0784415	0	Singlet	Adipocyte
VAT_2M	1337.7	1070	0.5450602	3	Singlet	FAPs
VAT_2M	1033.1	769	1.8440238	2	Singlet	FAPs
VAT_2M	8103.2	2948	1.0500051	0	Singlet	Adipocyte
VAT_2M	1601.2	1287	2.2374407	7	Singlet	Endothelial_cells
VAT_2M	5115.2	2456	4.0719964	7	Singlet	Endothelial_cells
VAT_2M	1593.8	1326	1.3092666	6	Singlet	Neurons
VAT_2M	1430.6	1109	1.4647562	2	Singlet	FAPs
VAT_2M	1387.3	999	3.5998651	2	Singlet	FAPs
VAT_2M	2307.4	1572	0.2041422	5	Singlet	Immune_cells
VAT_2M	3072.3	1657	0.1174838	0	Singlet	Adipocyte
VAT_2M	1869.9	1275	1.2650782	2	Singlet	FAPs
VAT_2M	1641.5	1212	1.5552478	5	Singlet	Immune_cells
VAT_2M	2610	1461	0.5749963	0	Singlet	Adipocyte
VAT_2M	4130	2054	0.0154993	0	Singlet	Adipocyte
VAT_2M	1623.6	1212	0.370151	2	Singlet	FAPs
VAT_2M	1093.1	757	7.3887705	4	Singlet	Immune_cells
VAT_2M	1160.6	845	1.2019391	5	Singlet	Immune_cells
VAT_2M	1675	1166	1.4012732	0	Singlet	Adipocyte
VAT_2M	2003	1213	0.074325	0	Singlet	Adipocyte
VAT_2M	2727.6	1618	0.3419648	6	Singlet	Neurons
VAT_2M	1040.4	726	1.0669273	3	Singlet	FAPs
VAT_2M	1002.3	781	0.8032493	7	Singlet	Endothelial_cells
VAT_2M	4760.1	2241	0.2076405	0	Singlet	Adipocyte
VAT_2M	6345.1	2581	0.0134084	0	Singlet	Adipocyte
VAT_2M	2030.3	1301	0.0807593	3	Singlet	FAPs
VAT_2M	2879.8	1697	0.4348056	0	Singlet	Adipocyte
VAT_2M	2032.5	1404	0.6425809	3	Singlet	FAPs
VAT_2M	1091.2	922	0.5506292	8	Singlet	Mesothelial
VAT_2M	5416.4	2535	0	0	Singlet	Adipocyte
VAT_2M	7506.5	3066	0.013313	0	Singlet	Adipocyte
VAT_2M	6788	2708	0.0098455	0	Singlet	Adipocyte
VAT_2M	4730.7	2284	0.0098423	0	Singlet	Adipocyte
VAT_2M	2099.6	1167	1.1352384	0	Singlet	Adipocyte
VAT_2M	2354.7	1549	0.6653364	3	Singlet	FAPs
VAT_2M	1790.9	1167	0.3902718	0	Singlet	Adipocyte
VAT_2M	5367.5	2357	1.8171485	0	Singlet	Adipocyte
VAT_2M	4385.7	2103	0.0303792	0	Singlet	Adipocyte
VAT_2M	4723.1	2361	0.3165729	0	Singlet	Adipocyte
VAT_2M	2287.1	1388	0.7104517	0	Singlet	Adipocyte
VAT_2M	8578.8	3141	0.2061122	0	Singlet	Adipocyte
VAT_2M	1350.1	1046	1.7955317	2	Singlet	FAPs
VAT_2M	1532	1209	2.4551128	3	Singlet	FAPs
VAT_2M	3700.7	1639	0.1871778	0	Singlet	Adipocyte
VAT_2M	1270	864	0.0699487	5	Singlet	Immune_cells
VAT_2M	1453.3	1113	2.1185919	2	Singlet	FAPs
VAT_2M	3338.6	1943	2.634042	3	Singlet	FAPs

VAT_2M	1757.3	1194	0.335583	6	Singlet	Neurons
VAT_2M	11204	3490	0.0535255	0	Singlet	Adipocyte
VAT_2M	3744.9	1873	0.0153478	0	Singlet	Adipocyte
VAT_2M	1712.9	1155	0.5550251	0	Singlet	Adipocyte
VAT_2M	5793.6	2583	0.0288983	0	Singlet	Adipocyte
VAT_2M	1973.5	1121	0.2439304	0	Singlet	Adipocyte
VAT_2M	8052.6	3065	0.0191688	0	Singlet	Adipocyte
VAT_2M	1865	1114	0.9773482	0	Singlet	Adipocyte
VAT_2M	1763.7	1173	1.8345005	2	Singlet	FAPs
VAT_2M	9080.8	3260	0.0039453	0	Singlet	Adipocyte
VAT_2M	1678.5	1151	0.7533044	2	Singlet	FAPs
VAT_2M	3981.4	1983	0.0883202	0	Singlet	Adipocyte
VAT_2M	2197.6	1204	0.0695384	0	Singlet	Adipocyte
VAT_2M	8794.3	2960	0.0335137	0	Singlet	Adipocyte
VAT_2M	1113.8	959	1.1692172	8	Singlet	Mesothelial
VAT_2M	6924.3	2779	0	0	Singlet	Adipocyte
VAT_2M	1331.5	1063	0.6220585	6	Singlet	Neurons
VAT_2M	1745.3	1149	0.7755857	0	Singlet	Adipocyte
VAT_2M	1035.8	727	6.0736699	6	Singlet	Neurons
VAT_2M	8298.8	3244	0.2171382	0	Singlet	Adipocyte
VAT_2M	1242.7	1065	1.0006394	1	Singlet	Immune_cells
VAT_2M	2435.3	1388	1.7426481	0	Singlet	Adipocyte
VAT_2M	7399.8	2736	0.0135811	0	Singlet	Adipocyte
VAT_2M	1422	1013	0.9483719	0	Singlet	Adipocyte
VAT_2M	8644.7	3276	0.2421044	0	Singlet	Adipocyte
VAT_2M	1318.3	1008	0.6471028	2	Singlet	FAPs
VAT_2M	3822.3	1865	0.0611966	0	Singlet	Adipocyte
VAT_2M	3726.5	2010	1.3758603	0	Singlet	Adipocyte
VAT_2M	2064.2	1214	3.7544584	6	Singlet	Neurons
VAT_2M	4395.9	2226	0.4476456	5	Singlet	Immune_cells
VAT_2M	2022	1220	0.0404302	0	Singlet	Adipocyte
VAT_2M	4382.2	2076	0.0150308	0	Singlet	Adipocyte
VAT_2M	1275	991	2.4462924	2	Singlet	FAPs
VAT_2M	4770	2354	0.0506888	0	Singlet	Adipocyte
VAT_2M	1924.1	1293	0.3396288	6	Singlet	Neurons
VAT_2M	1002.3	846	1.677992	1	Singlet	Immune_cells
VAT_2M	2777.8	1475	0.0502539	0	Singlet	Adipocyte
VAT_2M	1754.3	1184	1.3163902	7	Singlet	Endothelial_cells
VAT_2M	1942.7	1121	0.4597016	0	Singlet	Adipocyte
VAT_2M	1827.6	1261	1.5943404	2	Singlet	FAPs
VAT_2M	10576	3497	0.0186753	0	Singlet	Adipocyte
VAT_2M	5734.6	2330	0.0090333	0	Singlet	Adipocyte
VAT_2M	4886.5	2329	0.041386	0	Singlet	Adipocyte
VAT_2M	1225.1	912	1.9890502	3	Singlet	FAPs
VAT_2M	8079.3	3062	0.0191362	0	Singlet	Adipocyte
VAT_2M	6397.3	2528	0.1163147	0	Singlet	Adipocyte
VAT_2M	4207.2	2322	1.385004	2	Singlet	FAPs
VAT_2M	2375.7	1498	0.1949566	0	Singlet	Adipocyte
VAT_2M	1756.8	1084	0.0557642	0	Singlet	Adipocyte
VAT_2M	3299.9	1850	0.1632365	0	Singlet	Adipocyte
VAT_2M	2183.7	1562	1.7561592	2	Singlet	FAPs

VAT_2M	3194	1550	0.0226666	0	Singlet	Adipocyte
VAT_2M	1820.6	1258	0.2579892	2	Singlet	FAPs
VAT_2M	3369.3	1657	0.1156314	0	Singlet	Adipocyte
VAT_2M	1622.1	1032	0.4421974	3	Singlet	FAPs
VAT_2M	1035.4	814	2.7860501	4	Singlet	Immune_cells
VAT_2M	1043.4	844	2.8811436	8	Singlet	Mesothelial
VAT_2M	6715	2550	0.0054918	0	Singlet	Adipocyte
VAT_2M	6772.2	2637	0.0093692	0	Singlet	Adipocyte
VAT_2M	1103.5	927	1.0260659	6	Singlet	Neurons
VAT_2M	1246.7	883	2.1445969	2	Singlet	FAPs
VAT_2M	2306.8	1328	0	0	Singlet	Adipocyte
VAT_2M	2359.6	1525	1.9724767	2	Singlet	FAPs
VAT_2M	1423	1039	0.0897288	5	Singlet	Immune_cells
VAT_2M	3703.5	1790	0.0994827	0	Singlet	Adipocyte
VAT_2M	3992.1	2121	0	0	Singlet	Adipocyte
VAT_2M	4272.3	2129	0.2533621	0	Singlet	Adipocyte
VAT_2M	5977.6	2557	0.0072323	0	Singlet	Adipocyte
VAT_2M	1265.7	870	2.3236824	9	Singlet	Mesothelial
VAT_2M	1645.8	967	1.3821793	0	Singlet	Adipocyte
VAT_2M	4121.5	2468	0.4905549	2	Singlet	FAPs
VAT_2M	1681.8	1043	2.2231821	0	Singlet	Adipocyte
VAT_2M	1042.2	735	0.5977323	7	Singlet	Endothelial_cells
VAT_2M	3194.8	1844	0.1145732	3	Singlet	FAPs
VAT_2M	1044.5	788	0.7890049	3	Singlet	FAPs
VAT_2M	7604.5	2848	0.8892858	0	Singlet	Adipocyte
VAT_2M	4774.1	2163	0.0074145	0	Singlet	Adipocyte
VAT_2M	1880.6	1227	1.7451563	3	Singlet	FAPs
VAT_2M	6322.4	2929	1.3016445	2	Singlet	FAPs
VAT_2M	6393.5	2697	0.2146433	0	Singlet	Adipocyte
VAT_2M	1042.1	757	6.7818615	5	Singlet	Immune_cells
VAT_2M	5468.8	2458	0.0139042	0	Singlet	Adipocyte
VAT_2M	1915.9	1144	0.0267401	0	Singlet	Adipocyte
VAT_2M	2843.5	1648	0.1961104	3	Singlet	FAPs
VAT_2M	2267.7	1437	0	9	Singlet	Mesothelial
VAT_2M	3790.5	1913	0.0827905	0	Singlet	Adipocyte
VAT_2M	1625.4	1188	0.5665308	5	Singlet	Immune_cells
VAT_2M	9627.4	3258	0.0615393	0	Singlet	Adipocyte
VAT_2M	1197.9	944	1.5619652	2	Singlet	FAPs
VAT_2M	9078.6	3491	0.8130871	0	Singlet	Adipocyte
VAT_2M	2632.8	1596	0.048697	2	Singlet	FAPs
VAT_2M	9869.3	3522	0.2789211	0	Singlet	Adipocyte
VAT_2M	2206.2	1361	0.6357874	0	Singlet	Adipocyte
VAT_2M	3182.4	1607	1.5169751	0	Singlet	Adipocyte
VAT_2M	6190.4	2598	0.0397274	0	Singlet	Adipocyte
VAT_2M	11376	3455	1.2127621	0	Singlet	Adipocyte
VAT_2M	6514.6	2653	0.0882983	0	Singlet	Adipocyte
VAT_2M	6658.8	2873	0.2767568	0	Singlet	Adipocyte
VAT_2M	2369.9	1378	0.1878283	0	Singlet	Adipocyte
VAT_2M	1392.3	964	0.4215059	6	Singlet	Neurons
VAT_2M	1261.2	1146	0.1289889	6	Singlet	Neurons
VAT_2M	1860.3	1210	1.0568887	7	Singlet	Endothelial_cells

VAT_2M	1839.1	1226	0.4211972	2	Singlet	FAPs
VAT_2M	1089.8	879	0.4734731	2	Singlet	FAPs
VAT_2M	2137.7	1221	0.4758531	0	Singlet	Adipocyte
VAT_2M	2708.7	1476	0.3092281	0	Singlet	Adipocyte
VAT_2M	3067.2	1754	0.3202973	0	Singlet	Adipocyte
VAT_2M	7968.4	3080	0.1168977	0	Singlet	Adipocyte
VAT_2M	2148.4	1467	0.0706956	5	Singlet	Immune_cells
VAT_2M	3047.9	1581	0.6734005	0	Singlet	Adipocyte
VAT_2M	4283.9	2124	0.0097327	0	Singlet	Adipocyte
VAT_2M	3192.3	1775	0	0	Singlet	Adipocyte
VAT_2M	1004.1	821	0.3188024	4	Singlet	Immune_cells
VAT_2M	1613.9	1071	0.195531	0	Singlet	Adipocyte
VAT_2M	2451.1	1582	2.659471	2	Singlet	FAPs
VAT_2M	1215.9	984	1.3415425	11	Singlet	Smooth_muscle_cells
VAT_2M	6277	2695	0	0	Singlet	Adipocyte
VAT_2M	5325.4	2477	0.0051562	0	Singlet	Adipocyte
VAT_2M	1676	1221	0.6937364	2	Singlet	FAPs
VAT_2M	1974.3	1352	1.9448722	6	Singlet	Neurons
VAT_2M	5227.1	2425	0.0221388	0	Singlet	Adipocyte
VAT_2M	1666.2	1229	0.7409594	1	Singlet	Immune_cells
VAT_2M	2723.5	1592	2.7437307	3	Singlet	FAPs
VAT_2M	1302.9	1057	1.4766222	6	Singlet	Neurons
VAT_2M	2233.9	1197	0.6033537	0	Singlet	Adipocyte
VAT_2M	2066.9	1335	0.1575217	3	Singlet	FAPs
VAT_2M	3737.5	1882	1.5122426	0	Singlet	Adipocyte
VAT_2M	1402.9	1039	0.899483	5	Singlet	Immune_cells
VAT_2M	2206.3	1325	0.080835	0	Singlet	Adipocyte
VAT_2M	1587.8	1131	3.4250725	9	Singlet	Mesothelial
VAT_2M	1069.6	850	0.6592503	5	Singlet	Immune_cells
VAT_2M	1340.9	874	1.0386968	7	Singlet	Endothelial_cells
VAT_2M	3560.9	1834	0.0179804	0	Singlet	Adipocyte
VAT_2M	7877.4	3032	0.0610459	0	Singlet	Adipocyte
VAT_2M	4754.9	2202	0.0311388	0	Singlet	Adipocyte
VAT_2M	1084.6	841	0.4471634	9	Singlet	Mesothelial
VAT_2M	1277.7	984	0.951365	1	Singlet	Immune_cells
VAT_2M	3240.6	1902	2.3147846	5	Singlet	Immune_cells
VAT_2M	11161	3584	0.0052494	0	Singlet	Adipocyte
VAT_2M	2027.8	1394	1.5988811	0	Singlet	Adipocyte
VAT_2M	8196.1	3164	0.555551	0	Singlet	Adipocyte
VAT_2M	1474.1	965	4.9989744	2	Singlet	FAPs
VAT_2M	2627.5	1525	0.0626111	0	Singlet	Adipocyte
VAT_2M	3372	1633	0.0237389	0	Singlet	Adipocyte
VAT_2M	8624.9	3146	0.1446578	0	Singlet	Adipocyte
VAT_2M	3075.6	1720	0.1586679	0	Singlet	Adipocyte
VAT_2M	2966	1564	0	0	Singlet	Adipocyte
VAT_2M	1469.4	1095	0.1310051	3	Singlet	FAPs
VAT_2M	1414.7	1026	1.2047395	2	Singlet	FAPs
VAT_2M	9350.9	3236	0.1555427	0	Singlet	Adipocyte
VAT_2M	1308.1	889	3.0408141	6	Singlet	Neurons
VAT_2M	2778.5	1505	0.0665846	0	Singlet	Adipocyte
VAT_2M	4614.3	2193	0.1264494	0	Singlet	Adipocyte

VAT_2M	7530.6	2833	0.0700672	0	Singlet	Adipocyte
VAT_2M	1921.6	1139	1.3656426	0	Singlet	Adipocyte
VAT_2M	10720	3529	0.068315	0	Singlet	Adipocyte
VAT_2M	10104	3264	0.0404909	0	Singlet	Adipocyte
VAT_2M	1328.9	1096	0.3935241	8	Singlet	Mesothelial
VAT_2M	6999.3	2665	0.0536551	0	Singlet	Adipocyte
VAT_2M	8934.5	3356	0.0327555	0	Singlet	Adipocyte
VAT_2M	2518.7	1633	3.2252924	3	Singlet	FAPs
VAT_2M	1740.1	1123	0.9071041	2	Singlet	FAPs
VAT_2M	2566.1	1784	2.4617449	3	Singlet	FAPs
VAT_2M	3738	1769	0.0218927	0	Singlet	Adipocyte
VAT_2M	1097.3	916	0.4564362	6	Singlet	Neurons
VAT_2M	1262.6	969	0.2013103	5	Singlet	Immune_cells
VAT_2M	4979.4	2320	1.0510341	0	Singlet	Adipocyte
VAT_2M	2863	1692	0.6358714	0	Singlet	Adipocyte
VAT_2M	1592	1107	0.8045671	2	Singlet	FAPs
VAT_2M	1638.3	1066	2.1003348	2	Singlet	FAPs
VAT_2M	3740.1	2082	1.1576487	0	Singlet	Adipocyte
VAT_2M	2179	1398	1.9251328	3	Singlet	FAPs
VAT_2M	7729	2750	0.0473782	0	Singlet	Adipocyte
VAT_2M	2568.6	1692	0	5	Singlet	Immune_cells
VAT_2M	1704.5	1098	8.0046058	6	Singlet	Neurons
VAT_2M	1493.5	993	6.3965966	11	Singlet	Smooth_muscle_cells
VAT_2M	2227.9	1390	0.4584479	0	Singlet	Adipocyte
VAT_2M	1970.5	1236	0.3695792	2	Singlet	FAPs
VAT_2M	6708.8	2752	0	0	Singlet	Adipocyte
VAT_2M	2832.2	1763	0.1661439	6	Singlet	Neurons
VAT_2M	1111.9	791	0.8096576	1	Singlet	Immune_cells
VAT_2M	5582	2494	0.0817784	0	Singlet	Adipocyte
VAT_2M	1206.6	948	1.9629171	8	Singlet	Mesothelial
VAT_2M	8277.9	3163	0.0375895	0	Singlet	Adipocyte
VAT_2M	2386.9	1515	0.5575761	2	Singlet	FAPs
VAT_2M	1666.1	1197	0.8264659	7	Singlet	Endothelial_cells
VAT_2M	1752.4	1266	0.9436296	9	Singlet	Mesothelial
VAT_2M	1183.1	887	0.3851224	7	Singlet	Endothelial_cells
VAT_2M	1486.3	1033	2.201189	2	Singlet	FAPs
VAT_2M	1144.2	901	1.1078223	2	Singlet	FAPs
VAT_2M	2270.6	1454	0.2493486	3	Singlet	FAPs
VAT_2M	1018.1	850	1.4931406	6	Singlet	Neurons
VAT_2M	5992.4	2578	0.0273047	0	Singlet	Adipocyte
VAT_2M	1767.2	1186	4.9743453	2	Singlet	FAPs
VAT_2M	2067.6	1270	0.0005779	0	Singlet	Adipocyte
VAT_2M	1396.7	942	1.3790956	2	Singlet	FAPs
VAT_2M	3071.3	1770	0.1330693	6	Singlet	Neurons
VAT_2M	4422.9	2195	0.0433088	0	Singlet	Adipocyte
VAT_2M	6048.7	2495	0.0652907	0	Singlet	Adipocyte
VAT_2M	1454.3	1085	0.0586661	8	Singlet	Mesothelial
VAT_2M	1454.1	1073	1.6100961	1	Singlet	Immune_cells
VAT_2M	11127	3483	0.2432114	0	Singlet	Adipocyte
VAT_2M	5958.3	2508	0.0606797	0	Singlet	Adipocyte
VAT_2M	2385.3	1445	0.3491635	3	Singlet	FAPs

VAT_2M	7293.5	2875	0.043868	0	Singlet	Adipocyte
VAT_2M	1780.8	1116	0.6276298	0	Singlet	Adipocyte
VAT_2M	1412.2	1005	1.7918926	2	Singlet	FAPs
VAT_2M	8755.7	3099	0.0390349	0	Singlet	Adipocyte
VAT_2M	1173.7	940	1.5034105	7	Singlet	Endothelial_cells
VAT_2M	7823.2	2846	0.0010845	0	Singlet	Adipocyte
VAT_2M	1812.8	1217	4.3493543	5	Singlet	Immune_cells
VAT_2M	1326.8	974	1.9809587	2	Singlet	FAPs
VAT_2M	9609.7	3403	0.0631299	0	Singlet	Adipocyte
VAT_2M	2971.2	1805	0	0	Singlet	Adipocyte
VAT_2M	1676.8	1178	0.3444061	2	Singlet	FAPs
VAT_2M	1612.5	1110	4.419066	5	Singlet	Immune_cells
VAT_2M	1218.9	977	5.8687427	6	Singlet	Neurons
VAT_2M	1867.1	1256	2.8816848	3	Singlet	FAPs
VAT_2M	1248.8	944	0.7684406	2	Singlet	FAPs
VAT_2M	2730	1539	0.727171	2	Singlet	FAPs
VAT_2M	1067.2	797	1.7613273	2	Singlet	FAPs
VAT_2M	1531.2	1295	0.8586654	11	Singlet	Smooth_muscle_cells
VAT_2M	9406.3	3304	0.0813502	0	Singlet	Adipocyte
VAT_2M	6576.7	2661	0.0852244	0	Singlet	Adipocyte
VAT_2M	8725	3273	0.1423313	0	Singlet	Adipocyte
VAT_2M	1637.1	1243	2.4827835	8	Singlet	Mesothelial
VAT_2M	3719.9	2074	0.9814687	3	Singlet	FAPs
VAT_2M	5280.2	2291	0.1580891	0	Singlet	Adipocyte
VAT_2M	2120.5	1199	0.0542676	0	Singlet	Adipocyte
VAT_2M	3458.8	1860	2.2535011	5	Singlet	Immune_cells
VAT_2M	9790.5	3313	0.010285	0	Singlet	Adipocyte
VAT_2M	2736.1	1613	0.8238712	0	Singlet	Adipocyte
VAT_2M	5735.1	2506	0.1498792	0	Singlet	Adipocyte
VAT_2M	5869.6	2686	0.0185438	0	Singlet	Adipocyte
VAT_2M	5194.5	2387	0.0083528	0	Singlet	Adipocyte
VAT_2M	6536.5	2716	0.0517312	0	Singlet	Adipocyte
VAT_2M	8548.9	3014	0.0732597	0	Singlet	Adipocyte
VAT_2M	5963.2	2419	0.0557127	0	Singlet	Adipocyte
VAT_2M	5921.3	2462	0.1009493	0	Singlet	Adipocyte
VAT_2M	1277.7	942	0.5874383	7	Singlet	Endothelial_cells
VAT_2M	1012	829	2.1503383	6	Singlet	Neurons
VAT_2M	5276.1	2300	0.3183552	0	Singlet	Adipocyte
VAT_2M	1336.9	1016	0.572188	5	Singlet	Immune_cells
VAT_2M	3024.8	1935	1.7479462	2	Singlet	FAPs
VAT_2M	6191.6	2508	0.5800652	0	Singlet	Adipocyte
VAT_2M	11088	3245	0.000404	0	Singlet	Adipocyte
VAT_2M	13026	3582	0.1286844	0	Singlet	Adipocyte
VAT_2M	2613.6	1573	0.2578393	0	Singlet	Adipocyte
VAT_2M	3600.6	1900	0.2812606	0	Singlet	Adipocyte
VAT_2M	1414.8	898	1.428222	2	Singlet	FAPs
VAT_2M	1162.1	909	1.9659034	8	Singlet	Mesothelial
VAT_2M	1330.6	1096	1.0886843	6	Singlet	Neurons
VAT_2M	1215.8	901	0.9727684	5	Singlet	Immune_cells
VAT_2M	3303.5	1683	0.6284325	0	Singlet	Adipocyte
VAT_2M	1408	954	1.2380037	2	Singlet	FAPs

VAT_2M	7106.2	2714	0.0179541	0	Singlet	Adipocyte
VAT_2M	1986.4	1286	1.3737627	2	Singlet	FAPs
VAT_2M	1195.6	854	0.0259128	7	Singlet	Endothelial_cells
VAT_2M	4779.7	2208	0.0808724	0	Singlet	Adipocyte
VAT_2M	2324.7	1453	0.3740245	3	Singlet	FAPs
VAT_2M	2129.5	1252	0.3440954	0	Singlet	Adipocyte
VAT_2M	1644.4	882	0.3808569	0	Singlet	Adipocyte
VAT_2M	6659.5	2758	0.0970684	0	Singlet	Adipocyte
VAT_2M	2307.6	1623	1.4601769	3	Singlet	FAPs
VAT_2M	1027.7	845	2.6338511	2	Singlet	FAPs
VAT_2M	1495.4	1087	0.412373	2	Singlet	FAPs
VAT_2M	9145.2	3322	0.0859073	0	Singlet	Adipocyte
VAT_2M	3814.1	2031	0.1375247	0	Singlet	Adipocyte
VAT_2M	2979.7	1611	0.0366199	0	Singlet	Adipocyte
VAT_2M	7465.5	2846	0.0146091	0	Singlet	Adipocyte
VAT_2M	1201.2	1042	0.5300953	8	Singlet	Mesothelial
VAT_2M	1842.3	1176	0.5121303	3	Singlet	FAPs
VAT_2M	2533.8	1489	0.0663267	0	Singlet	Adipocyte
VAT_2M	7422.2	2890	0.0509205	0	Singlet	Adipocyte
VAT_2M	5849.1	2575	0.4984898	0	Singlet	Adipocyte
VAT_2M	3818.6	1981	0.5326914	0	Singlet	Adipocyte
VAT_2M	1100.4	704	2.8065661	9	Singlet	Mesothelial
VAT_2M	2574.9	1646	1.0215606	3	Singlet	FAPs
VAT_2M	1082.4	815	1.2683562	2	Singlet	FAPs
VAT_2M	2529.7	1749	0.434942	2	Singlet	FAPs
VAT_2M	4571.7	2180	0.0993469	0	Singlet	Adipocyte
VAT_2M	4712.7	2231	0.0668143	0	Singlet	Adipocyte
VAT_2M	1270.2	980	2.0599868	5	Singlet	Immune_cells
VAT_2M	1384.2	1055	0.3271456	2	Singlet	FAPs
VAT_2M	2452.6	1442	0.9107753	6	Singlet	Neurons
VAT_2M	2598.5	1595	0.9801918	5	Singlet	Immune_cells
VAT_2M	1675.2	1055	0.1657312	0	Singlet	Adipocyte
VAT_2M	2587.3	1639	1.0161105	2	Singlet	FAPs
VAT_2M	3387	1763	0.2476832	0	Singlet	Adipocyte
VAT_2M	1848.7	1262	0.7933664	2	Singlet	FAPs
VAT_2M	1334.5	1023	0.7538324	3	Singlet	FAPs
VAT_2M	11713	3912	0.0818835	0	Singlet	Adipocyte
VAT_2M	8100.9	3111	0.4134121	0	Singlet	Adipocyte
VAT_2M	8727.1	3233	0.0059618	0	Singlet	Adipocyte
VAT_2M	3911.3	2065	0	0	Singlet	Adipocyte
VAT_2M	2018.1	1308	1.3585415	0	Singlet	Adipocyte
VAT_2M	1703.6	1510	1.9268484	6	Singlet	Neurons
VAT_2M	1527.7	1248	0.5228367	3	Singlet	FAPs
VAT_2M	1367.3	1182	4.2692605	6	Singlet	Neurons
VAT_2M	6964.1	2822	0.0842032	0	Singlet	Adipocyte
VAT_2M	1415.3	1134	0.8557342	2	Singlet	FAPs
VAT_2M	4348.7	2271	0.7665507	0	Singlet	Adipocyte
VAT_2M	6046	2576	0.0230747	0	Singlet	Adipocyte
VAT_2M	6287	2770	0.005874	0	Singlet	Adipocyte
VAT_2M	2822.9	1626	0.3521932	0	Singlet	Adipocyte
VAT_2M	1761.3	1254	0	12	Singlet	Adipocyte

VAT_2M	3948.2	1946	0.0487564	0	Singlet	Adipocyte
VAT_2M	2958.4	1605	0.5254612	0	Singlet	Adipocyte
VAT_2M	10342	3475	0.0679512	0	Singlet	Adipocyte
VAT_2M	2538.3	1606	0.5910618	5	Singlet	Immune_cells
VAT_2M	2545.2	1669	0.8946915	3	Singlet	FAPs
VAT_2M	1249.4	1050	1.3822499	6	Singlet	Neurons
VAT_2M	1288.5	1066	0.5887013	1	Singlet	Immune_cells
VAT_2M	1574.3	1121	0.4487929	2	Singlet	FAPs
VAT_2M	10241	3417	2.2338392	0	Singlet	Adipocyte
VAT_2M	7366.6	3093	0.0038052	0	Singlet	Adipocyte
VAT_2M	1889.7	1314	1.1430284	0	Singlet	Adipocyte
VAT_2M	5619.1	2442	0.2880152	0	Singlet	Adipocyte
VAT_2M	7131.4	2908	0.067215	0	Singlet	Adipocyte
VAT_2M	9462.5	3286	0.0122378	0	Singlet	Adipocyte
VAT_2M	1541.2	842	7.4491865	0	Singlet	Adipocyte
VAT_2M	1211.9	902	0.3200784	2	Singlet	FAPs
VAT_2M	5165.7	2349	0	0	Singlet	Adipocyte
VAT_2M	1323.6	1120	1.0094345	6	Singlet	Neurons
VAT_2M	1399.9	1120	1.2340427	4	Singlet	Immune_cells
VAT_2M	1979	1404	1.2448152	3	Singlet	FAPs
VAT_2M	7626.7	2992	0.3351435	0	Singlet	Adipocyte
VAT_2M	9438.2	3156	0.0009731	0	Singlet	Adipocyte
VAT_2M	1373.3	1021	2.5569803	2	Singlet	FAPs
VAT_2M	1400.1	1041	1.5045706	2	Singlet	FAPs
VAT_2M	3579.2	2024	1.4179702	3	Singlet	FAPs
VAT_2M	2556.9	1660	0.2557787	2	Singlet	FAPs
VAT_2M	1872.7	1318	1.8758102	2	Singlet	FAPs
VAT_2M	1569.1	1052	0.590089	6	Singlet	Neurons
VAT_2M	2300.4	1357	2.862337	3	Singlet	FAPs
VAT_2M	1142.7	836	0.8309021	7	Singlet	Endothelial_cells
VAT_2M	4604	2118	0.1181152	0	Singlet	Adipocyte
VAT_2M	2799.8	1524	0.2675019	0	Singlet	Adipocyte
VAT_2M	1086.3	947	0.218782	4	Singlet	Immune_cells
VAT_2M	3512.3	1993	0.0514194	0	Singlet	Adipocyte
VAT_2M	1450.6	943	0.057055	0	Singlet	Adipocyte
VAT_2M	3331.6	1910	0.6758504	3	Singlet	FAPs
VAT_2M	1155.3	892	1.3466893	3	Singlet	FAPs
VAT_2M	1305	946	1.7270044	7	Singlet	Endothelial_cells
VAT_2M	1035.8	898	0.9525191	6	Singlet	Neurons
VAT_2M	1155.8	850	0.3674875	2	Singlet	FAPs
VAT_2M	1068.8	827	1.2147657	5	Singlet	Immune_cells
VAT_2M	1141.8	868	0.2233023	2	Singlet	FAPs
VAT_2M	2260.8	1373	0.4041537	2	Singlet	FAPs
VAT_2M	1038.6	874	1.2301963	6	Singlet	Neurons
VAT_2M	3379.4	1764	0.0580763	0	Singlet	Adipocyte
VAT_2M	11241	3566	0.0693312	0	Singlet	Adipocyte
VAT_2M	1940.1	1235	2.7763452	2	Singlet	FAPs
VAT_2M	1964.8	1282	0.5696844	3	Singlet	FAPs
VAT_2M	2217.7	1495	0.9098289	6	Singlet	Neurons
VAT_2M	2023.5	1051	0.4655488	0	Singlet	Adipocyte
VAT_2M	9929.3	3507	0.2251275	0	Singlet	Adipocyte

VAT_2M	2124.7	1468	1.529175	5	Singlet	Immune_cells
VAT_2M	4763	2373	0	0	Singlet	Adipocyte
VAT_2M	2385.8	1409	0.8635276	0	Singlet	Adipocyte
VAT_2M	5378.8	2576	0.0379951	0	Singlet	Adipocyte
VAT_2M	1309.7	951	1.8703698	9	Singlet	Mesothelial
VAT_2M	2008.4	1271	0.7359284	0	Singlet	Adipocyte
VAT_2M	7572.4	2739	0.0824324	0	Singlet	Adipocyte
VAT_2M	2087	1286	0.9570764	5	Singlet	Immune_cells
VAT_2M	2391.6	1350	0.1276242	0	Singlet	Adipocyte
VAT_2M	1697.6	1132	0.2209864	3	Singlet	FAPs
VAT_2M	4928.4	2313	0.0567516	0	Singlet	Adipocyte
VAT_2M	7896.2	3027	0.0022991	0	Singlet	Adipocyte
VAT_2M	1881.1	1407	1.8657355	2	Singlet	FAPs
VAT_2M	4445.7	2097	0.0803052	0	Singlet	Adipocyte
VAT_2M	2201.1	1417	4.898181	3	Singlet	FAPs
VAT_2M	2307.6	1374	0.0539831	7	Singlet	Endothelial_cells
VAT_2M	1232.1	955	1.6664447	5	Singlet	Immune_cells
VAT_2M	1150.5	962	2.1990801	6	Singlet	Neurons
VAT_2M	1061.9	782	0.6693817	2	Singlet	FAPs
VAT_2M	9358.3	3269	0.0529743	0	Singlet	Adipocyte
VAT_2M	6405.8	2659	0.0637862	0	Singlet	Adipocyte
VAT_2M	2216.8	1429	0.7307239	5	Singlet	Immune_cells
VAT_2M	7267.1	2738	0.019103	0	Singlet	Adipocyte
VAT_2M	1015.9	764	1.2351981	3	Singlet	FAPs
VAT_2M	5585.5	2419	0	0	Singlet	Adipocyte
VAT_2M	2596.1	1641	2.1468485	3	Singlet	FAPs
VAT_2M	7050.1	2857	0.974412	0	Singlet	Adipocyte
VAT_2M	1880.7	1296	0.2084809	3	Singlet	FAPs
VAT_2M	1056	820	0	2	Singlet	FAPs
VAT_2M	2876.8	1892	0.1272924	6	Singlet	Neurons
VAT_2M	2181.6	1547	1.259637	5	Singlet	Immune_cells
VAT_2M	1899.8	1330	0.4690592	6	Singlet	Neurons
VAT_2M	1435	1059	0.4378684	7	Singlet	Endothelial_cells
VAT_2M	7908.1	3150	0.9319467	0	Singlet	Adipocyte
VAT_2M	2248.4	1280	0.3243719	0	Singlet	Adipocyte
VAT_2M	1016.8	798	1.9791787	2	Singlet	FAPs
VAT_2M	1636.1	1295	2.3427977	6	Singlet	Neurons
VAT_2M	1766.7	1228	0.4443083	5	Singlet	Immune_cells
VAT_2M	1960.9	1299	0.8829844	5	Singlet	Immune_cells
VAT_2M	6438.1	2687	0.0218345	0	Singlet	Adipocyte
VAT_2M	3633.7	1947	0.0414439	0	Singlet	Adipocyte
VAT_2M	1042.2	821	5.2866549	9	Singlet	Mesothelial
VAT_2M	2029.8	1298	0.3913697	2	Singlet	FAPs
VAT_2M	7837.6	3126	0.4923247	0	Singlet	Adipocyte
VAT_2M	1285.1	934	0	2	Singlet	FAPs
VAT_2M	5214.8	2217	0	0	Singlet	Adipocyte
VAT_2M	6701.4	2710	0.0644463	0	Singlet	Adipocyte
VAT_2M	2067.4	1275	0.6025497	0	Singlet	Adipocyte
VAT_2M	1633.7	1017	0.0383724	0	Singlet	Adipocyte
VAT_2M	8493.4	3065	0.0003983	0	Singlet	Adipocyte
VAT_2M	4955.4	2320	0.1359668	0	Singlet	Adipocyte

VAT_2M	5326.4	2289	0.0746004	0	Singlet	Adipocyte
VAT_2M	1590.7	1135	0.3691412	6	Singlet	Neurons
VAT_2M	3841.5	2018	0.5384018	0	Singlet	Adipocyte
VAT_2M	8649.6	3019	0.1101672	0	Singlet	Adipocyte
VAT_2M	6836.2	2770	0.1646911	0	Singlet	Adipocyte
VAT_2M	3749.7	1781	0.4536245	0	Singlet	Adipocyte
VAT_2M	7309.1	3056	0.112567	0	Singlet	Adipocyte
VAT_2M	3654.6	1800	1.4742743	0	Singlet	Adipocyte
VAT_2M	2498.6	1399	0.1807307	0	Singlet	Adipocyte
VAT_2M	6317.1	2711	0.6609258	0	Singlet	Adipocyte
VAT_2M	1265.9	961	2.2255264	1	Singlet	Immune_cells
VAT_2M	1313.9	1097	0.6636386	9	Singlet	Mesothelial
VAT_2M	5626.4	2457	0.226575	0	Singlet	Adipocyte
VAT_2M	1235.7	928	1.8919351	3	Singlet	FAPs
VAT_2M	1640.9	1062	2.6993127	3	Singlet	FAPs
VAT_2M	10390	3470	0.019674	0	Singlet	Adipocyte
VAT_2M	3806.6	1926	0.0420221	0	Singlet	Adipocyte
VAT_2M	3300.4	1937	2.2481157	0	Singlet	Adipocyte
VAT_2M	1646.6	1114	0.2931585	6	Singlet	Neurons
VAT_2M	4172.9	2061	0.1599301	0	Singlet	Adipocyte
VAT_2M	1330.3	958	0.0995356	3	Singlet	FAPs
VAT_2M	4147.6	2110	0	0	Singlet	Adipocyte
VAT_2M	6006.8	2500	0	0	Singlet	Adipocyte
VAT_2M	1683.1	1206	0.5920443	2	Singlet	FAPs
VAT_2M	1943.2	1135	12.107581	2	Singlet	FAPs
VAT_2M	1884.3	1272	0.5971802	2	Singlet	FAPs
VAT_2M	2856.9	1650	1.7243015	0	Singlet	Adipocyte
VAT_2M	2009.5	1320	0.2651921	3	Singlet	FAPs
VAT_2M	4389	2078	0	0	Singlet	Adipocyte
VAT_2M	2818.9	1741	1.2532235	3	Singlet	FAPs
VAT_2M	1696.8	1237	1.0351854	2	Singlet	FAPs
VAT_2M	1911.9	1116	0.0525085	0	Singlet	Adipocyte
VAT_2M	1488.9	1006	1.4672684	3	Singlet	FAPs
VAT_2M	3759.7	1941	0.7247618	0	Singlet	Adipocyte
VAT_2M	6623.3	2562	0.0817238	0	Singlet	Adipocyte
VAT_2M	1143.5	810	0.9501964	3	Singlet	FAPs
VAT_2M	7924.1	3016	0.6766351	0	Singlet	Adipocyte
VAT_2M	4082	1911	0.0469927	0	Singlet	Adipocyte
VAT_2M	14879	4071	0.0667781	0	Singlet	Adipocyte
VAT_2M	1197.7	879	6.4922992	4	Singlet	Immune_cells
VAT_2M	2018.5	1333	1.4752291	2	Singlet	FAPs
VAT_2M	1358.7	1000	0.1054728	3	Singlet	FAPs
VAT_2M	3832	1944	0.0444178	0	Singlet	Adipocyte
VAT_2M	3701.1	1861	0.0605466	0	Singlet	Adipocyte
VAT_2M	1291	951	0.712797	3	Singlet	FAPs
VAT_2M	4125.8	1766	0.0729127	0	Singlet	Adipocyte
VAT_2M	3668.6	2052	0.938025	3	Singlet	FAPs
VAT_2M	6364.6	2606	0.0083939	0	Singlet	Adipocyte
VAT_2M	1407.5	1137	0.6998987	6	Singlet	Neurons
VAT_2M	9286.2	3311	0.0766003	0	Singlet	Adipocyte
VAT_2M	6905.6	2693	0.1022404	0	Singlet	Adipocyte

VAT_2M	4345.3	2053	0.3401068	0	Singlet	Adipocyte
VAT_2M	2351.7	1503	1.4516258	2	Singlet	FAPs
VAT_2M	1860.8	1192	0.9428616	7	Singlet	Endothelial_cells
VAT_2M	7761.7	2870	0	0	Singlet	Adipocyte
VAT_2M	2184.2	1281	0.2649715	0	Singlet	Adipocyte
VAT_2M	2346.4	1484	0.7473013	2	Singlet	FAPs
VAT_2M	1809.1	1331	1.9288519	3	Singlet	FAPs
VAT_2M	7781.8	2888	0.0203959	0	Singlet	Adipocyte
VAT_2M	3762.2	2032	1.8111293	2	Singlet	FAPs
VAT_2M	2200.2	1400	0.0241838	7	Singlet	Endothelial_cells
VAT_2M	2724.9	1780	0.8056263	2	Singlet	FAPs
VAT_2M	10509	3382	0.0402855	0	Singlet	Adipocyte
VAT_2M	2303.4	1470	0.4694984	0	Singlet	Adipocyte
VAT_2M	2136.3	1488	0.0714708	3	Singlet	FAPs
VAT_2M	1706.2	1072	9.2319798	7	Singlet	Endothelial_cells
VAT_2M	3833	1927	0.0793166	0	Singlet	Adipocyte
VAT_2M	2633.2	1398	1.7913729	0	Singlet	Adipocyte
VAT_2M	1255.9	808	0.1261218	7	Singlet	Endothelial_cells
VAT_2M	1030.6	835	0.6652256	6	Singlet	Neurons
VAT_2M	6829.7	2704	0.0094254	0	Singlet	Adipocyte
VAT_2M	1006.6	841	0.3484223	6	Singlet	Neurons
VAT_2M	3464.2	1769	0.8617708	0	Singlet	Adipocyte
VAT_2M	4873.3	2488	1.9086156	3	Singlet	FAPs
VAT_2M	3048.3	1778	0.4135354	0	Singlet	Adipocyte
VAT_2M	1826.5	1130	0.3749519	0	Singlet	Adipocyte
VAT_2M	1461.5	958	1.9462034	7	Singlet	Endothelial_cells
VAT_2M	1457.7	1160	0.9156363	5	Singlet	Immune_cells
VAT_2M	3020.7	1734	0.9554863	6	Singlet	Neurons
VAT_2M	3189.5	1625	0.181778	0	Singlet	Adipocyte
VAT_2M	2007	1349	0.1224486	2	Singlet	FAPs
VAT_2M	1727.6	1338	1.3615586	2	Singlet	FAPs
VAT_2M	4798.9	2174	0.0426812	0	Singlet	Adipocyte
VAT_2M	1624.9	1112	0.1498293	6	Singlet	Neurons
VAT_2M	2026.9	1496	1.132976	6	Singlet	Neurons
VAT_2M	4275.1	1977	0.0144491	0	Singlet	Adipocyte
VAT_2M	2949	1703	0.4434685	0	Singlet	Adipocyte
VAT_2M	1524	1106	1.7837552	7	Singlet	Endothelial_cells
VAT_2M	2714.9	1579	0.0404998	0	Singlet	Adipocyte
VAT_2M	1121.3	891	0.7199036	2	Singlet	FAPs
VAT_2M	1985.5	1191	0.2195869	0	Singlet	Adipocyte
VAT_2M	3492.5	1814	0.2409791	0	Singlet	Adipocyte
VAT_2M	1151.3	961	1.9375784	10	Singlet	Immune_cells
VAT_2M	1599.2	1110	0.3349207	0	Singlet	Adipocyte
VAT_2M	4416.7	2172	0.523429	0	Singlet	Adipocyte
VAT_2M	1425.6	998	5.1859877	5	Singlet	Immune_cells
VAT_2M	1218.2	869	0.6930408	7	Singlet	Endothelial_cells
VAT_2M	1083.7	769	1.099654	9	Singlet	Mesothelial
VAT_2M	5134	2355	0.01826	0	Singlet	Adipocyte
VAT_2M	1586.4	1035	1.2471759	0	Singlet	Adipocyte
VAT_2M	1279.1	963	2.4474217	4	Singlet	Immune_cells
VAT_2M	2999.4	1561	0.7996128	0	Singlet	Adipocyte

VAT_2M	5355.1	2277	0.0154033	0	Singlet	Adipocyte
VAT_2M	1468.8	1253	0.6613403	6	Singlet	Neurons
VAT_2M	1089.5	861	0.3721056	9	Singlet	Mesothelial
VAT_2M	5496.3	2282	0.0088548	0	Singlet	Adipocyte
VAT_2M	2533.9	1607	0.3023746	0	Singlet	Adipocyte
VAT_2M	2994	1886	0.0485247	2	Singlet	FAPs
VAT_2M	2745.5	1502	0.5482112	0	Singlet	Adipocyte
VAT_2M	1040	882	1.1130412	6	Singlet	Neurons
VAT_2M	1169.3	924	0.5853065	6	Singlet	Neurons
VAT_2M	3717.1	1998	2.7468167	5	Singlet	Immune_cells
VAT_2M	3387.9	1701	0.2360781	0	Singlet	Adipocyte
VAT_2M	3551.7	1852	0	0	Singlet	Adipocyte
VAT_2M	12237	3790	0.0002876	0	Singlet	Adipocyte
VAT_2M	10138	3393	0	0	Singlet	Adipocyte
VAT_2M	6905	2827	0.00948	0	Singlet	Adipocyte
VAT_2M	4785.4	2174	0.151305	0	Singlet	Adipocyte
VAT_2M	4369	2149	0.1921423	0	Singlet	Adipocyte
VAT_2M	1862.4	1277	0.200812	3	Singlet	FAPs
VAT_2M	4325.9	2438	1.8885098	2	Singlet	FAPs
VAT_2M	9844.2	3215	0	0	Singlet	Adipocyte
VAT_2M	1909.9	1277	2.9061083	3	Singlet	FAPs
VAT_2M	5558.4	2318	0.2434151	0	Singlet	Adipocyte
VAT_2M	5315.6	2228	0.0573062	0	Singlet	Adipocyte
VAT_2M	1873.4	1637	3.996005	6	Singlet	Neurons
VAT_2M	1495.7	1214	0.7730885	9	Singlet	Mesothelial
VAT_2M	1420.2	963	7.0190252	6	Singlet	Neurons
VAT_2M	2582.6	1475	0.3278324	0	Singlet	Adipocyte
VAT_2M	8571.9	3172	0.0130669	0	Singlet	Adipocyte
VAT_2M	1157.1	871	0	2	Singlet	FAPs
VAT_2M	1651.5	1016	0.1909251	0	Singlet	Adipocyte
VAT_2M	2182.5	1468	0.4977156	2	Singlet	FAPs
VAT_2M	3756.7	1889	0.1322285	0	Singlet	Adipocyte
VAT_2M	1026.8	819	1.5584772	7	Singlet	Endothelial_cells
VAT_2M	8990.5	3234	0.172844	0	Singlet	Adipocyte
VAT_2M	3481.3	1974	0.2479716	0	Singlet	Adipocyte
VAT_2M	2994.5	1859	1.3652267	3	Singlet	FAPs
VAT_2M	1019	850	0.9750308	10	Singlet	Immune_cells
VAT_2M	5829.4	2317	0.0087595	0	Singlet	Adipocyte
VAT_2M	1279.9	968	0.5766	3	Singlet	FAPs
VAT_2M	7895	3015	0.0925933	0	Singlet	Adipocyte
VAT_2M	7538.9	2688	0	0	Singlet	Adipocyte
VAT_2M	3806.3	1963	0	2	Singlet	FAPs
VAT_2M	3887.6	2129	0.1371907	0	Singlet	Adipocyte
VAT_2M	2651.8	1701	3.3509123	9	Singlet	Mesothelial
VAT_2M	3436.1	1956	1.5560992	2	Singlet	FAPs
VAT_2M	5795.7	2332	0.0203164	0	Singlet	Adipocyte
VAT_2M	1090.2	822	0.2655938	7	Singlet	Endothelial_cells
VAT_2M	1284.7	953	5.3793871	6	Singlet	Neurons
VAT_2M	6639.7	2809	0.3445856	0	Singlet	Adipocyte
VAT_2M	2584.9	1448	0.6219559	0	Singlet	Adipocyte
VAT_2M	1600.2	1143	0.9801994	2	Singlet	FAPs

VAT_2M	1569.5	1008	3.2842768	3	Singlet	FAPs
VAT_2M	1529.8	1052	0.6570478	3	Singlet	FAPs
VAT_2M	6143.9	2601	0.0292831	0	Singlet	Adipocyte
VAT_2M	2682.1	930	12.916323	0	Singlet	Adipocyte
VAT_2M	1559.4	1307	0.2029224	3	Singlet	FAPs
VAT_2M	3561.5	1965	0.1157874	0	Singlet	Adipocyte
VAT_2M	6294.6	2611	0.2260413	0	Singlet	Adipocyte
VAT_2M	1768.8	1137	1.9391122	5	Singlet	Immune_cells
VAT_2M	1909.3	1262	2.054419	2	Singlet	FAPs
VAT_2M	2608.3	1449	0.0277953	0	Singlet	Adipocyte
VAT_2M	1796.2	1112	5.4583906	8	Singlet	Mesothelial
VAT_2M	9122.9	3173	0.3719343	0	Singlet	Adipocyte
VAT_2M	2498.9	1574	1.0184344	5	Singlet	Immune_cells
VAT_2M	6475.6	2760	0.8468379	0	Singlet	Adipocyte
VAT_2M	9548.4	3131	0.0071151	0	Singlet	Adipocyte
VAT_2M	1632.2	1095	0.252499	0	Singlet	Adipocyte
VAT_2M	5382	2335	0.1715538	0	Singlet	Adipocyte
VAT_2M	2317.7	1588	0.221377	3	Singlet	FAPs
VAT_2M	1770.8	1324	0.5260636	2	Singlet	FAPs
VAT_2M	2933.6	1546	0.0569175	0	Singlet	Adipocyte
VAT_2M	4710.5	2277	0.1050005	0	Singlet	Adipocyte
VAT_2M	1959.8	1522	0.4849211	6	Singlet	Neurons
VAT_2M	1803.1	1144	0.4327233	0	Singlet	Adipocyte
VAT_2M	1342	953	2.0291981	5	Singlet	Immune_cells
VAT_2M	3697.7	1972	0.0135042	0	Singlet	Adipocyte
VAT_2M	1200.2	873	0.1103687	3	Singlet	FAPs
VAT_2M	1443.2	1047	0.8349515	2	Singlet	FAPs
VAT_2M	7628	2847	0.0743432	0	Singlet	Adipocyte
VAT_2M	4197.3	2006	0	0	Singlet	Adipocyte
VAT_2M	5302.6	2458	0.0151933	0	Singlet	Adipocyte
VAT_2M	3343.8	2162	4.0373564	9	Singlet	Mesothelial
VAT_2M	1091.7	779	1.8973726	3	Singlet	FAPs
VAT_2M	1428.3	1010	0.1089144	3	Singlet	FAPs
VAT_2M	5559.5	2408	0	0	Singlet	Adipocyte
VAT_2M	1293.7	1029	0.895542	3	Singlet	FAPs
VAT_2M	4140.3	2114	0.3482442	0	Singlet	Adipocyte
VAT_2M	2723.4	1515	0.4242704	0	Singlet	Adipocyte
VAT_2M	1011	726	2.7606528	2	Singlet	FAPs
VAT_2M	4020.2	2043	0	0	Singlet	Adipocyte
VAT_2M	5631.3	2483	0.0686248	0	Singlet	Adipocyte
VAT_2M	1252.6	898	3.6825148	2	Singlet	FAPs
VAT_2M	6966.7	2802	0.0083679	0	Singlet	Adipocyte
VAT_2M	2010.8	1324	0.0378116	0	Singlet	Adipocyte
VAT_2M	5840.6	2605	0	0	Singlet	Adipocyte
VAT_2M	3787.5	1832	0.0658575	0	Singlet	Adipocyte
VAT_2M	1659.5	1136	1.3050926	5	Singlet	Immune_cells
VAT_2M	1866.6	1201	0.5580119	5	Singlet	Immune_cells
VAT_2M	11031	3661	0.0557779	0	Singlet	Adipocyte
VAT_2M	1679	1098	0.3420029	3	Singlet	FAPs
VAT_2M	8551.3	3164	0.0238237	0	Singlet	Adipocyte
VAT_2M	2424.9	1502	1.7735012	2	Singlet	FAPs

VAT_2M	1340.4	1004	2.3833164	2	Singlet	FAPs
VAT_2M	3104.9	1780	0.3799775	0	Singlet	Adipocyte
VAT_2M	4830.8	2291	0.414942	0	Singlet	Adipocyte
VAT_2M	1703.4	949	0.2966037	0	Singlet	Adipocyte
VAT_2M	4461.4	2424	1.1867741	3	Singlet	FAPs
VAT_2M	2475.1	1419	0.3988643	0	Singlet	Adipocyte
VAT_2M	6981.5	2801	0.8299716	0	Singlet	Adipocyte
VAT_2M	1365.5	1017	0.4001753	3	Singlet	FAPs
VAT_2M	3545.1	1847	0.147787	0	Singlet	Adipocyte
VAT_2M	6913.9	2675	0.0407291	0	Singlet	Adipocyte
VAT_2M	1997.4	1411	0.6702659	3	Singlet	FAPs
VAT_2M	4049.5	2073	0.4564963	0	Singlet	Adipocyte
VAT_2M	1090.2	809	1.8271915	5	Singlet	Immune_cells
VAT_2M	3430.1	2166	0.6771707	2	Singlet	FAPs
VAT_2M	9053.4	3146	0.0110058	0	Singlet	Adipocyte
VAT_2M	2647.2	1737	0.9398922	5	Singlet	Immune_cells
VAT_2M	3835.6	2014	0.215812	0	Singlet	Adipocyte
VAT_2M	1261.4	977	3.9170714	11	Singlet	Smooth_muscle_cells
VAT_2M	3278.2	1762	0.0320362	0	Singlet	Adipocyte
VAT_2M	5799.3	2533	0.1212241	0	Singlet	Adipocyte
VAT_2M	13460	3680	0	0	Singlet	Adipocyte
VAT_2M	1282.7	1028	0.4446349	2	Singlet	FAPs
VAT_2M	3870.5	1920	0.0806079	0	Singlet	Adipocyte
VAT_2M	1512.3	999	0.6550657	7	Singlet	Endothelial_cells
VAT_2M	1984	1236	0.9642205	7	Singlet	Endothelial_cells
VAT_2M	2538.5	1397	2.950959	5	Singlet	Immune_cells
VAT_2M	5086	2203	0.0103069	0	Singlet	Adipocyte
VAT_2M	3818.2	1844	0.1873094	0	Singlet	Adipocyte
VAT_2M	3402	1750	0.3203321	0	Singlet	Adipocyte
VAT_2M	6078.3	2586	0.0269272	0	Singlet	Adipocyte
VAT_2M	1736.2	1098	0.5442825	0	Singlet	Adipocyte
VAT_2M	3021	1570	0.4975494	0	Singlet	Adipocyte
VAT_2M	6866	2791	0.0315087	0	Singlet	Adipocyte
VAT_2M	1678.4	1084	7.7780268	7	Singlet	Endothelial_cells
VAT_2M	6018.9	2526	0.0494143	0	Singlet	Adipocyte
VAT_2M	5063.2	2236	0.1464547	0	Singlet	Adipocyte
VAT_2M	3812.5	1808	0.0578602	0	Singlet	Adipocyte
VAT_2M	6205.1	2600	0.0693036	0	Singlet	Adipocyte
VAT_2M	5136.7	2378	0.0140919	0	Singlet	Adipocyte
VAT_2M	1178.4	896	2.1310046	9	Singlet	Mesothelial
VAT_2M	1179.2	887	1.3421737	9	Singlet	Mesothelial
VAT_2M	1108.6	850	0.7175622	3	Singlet	FAPs
VAT_2M	2032.8	1257	0.0397034	0	Singlet	Adipocyte
VAT_2M	7294.7	2895	0.2840785	0	Singlet	Adipocyte
VAT_2M	7498.7	2971	0.028087	0	Singlet	Adipocyte
VAT_2M	3323.8	1718	1.2668706	0	Singlet	Adipocyte
VAT_2M	9279.5	3455	0.3363638	0	Singlet	Adipocyte
VAT_2M	5464.1	2402	0.0061438	0	Singlet	Adipocyte
VAT_2M	3471.8	1880	0.0815576	0	Singlet	Adipocyte
VAT_2M	1505	1119	1.4622785	2	Singlet	FAPs
VAT_2M	1661.6	985	0.2579145	0	Singlet	Adipocyte

VAT_2M	3832.8	1776	1.9313431	2	Singlet	FAPs
VAT_2M	10967	3572	0	0	Singlet	Adipocyte
VAT_2M	4313.9	2032	0	0	Singlet	Adipocyte
VAT_2M	1365.7	1018	2.0716104	5	Singlet	Immune_cells
VAT_2M	5127.6	2378	0.3209649	0	Singlet	Adipocyte
VAT_2M	1652.3	1207	0.9375387	0	Singlet	Adipocyte
VAT_2M	3275	1713	0.0563985	0	Singlet	Adipocyte
VAT_2M	6891	2786	0.0396774	0	Singlet	Adipocyte
VAT_2M	2376.1	1468	0.2235698	0	Singlet	Adipocyte
VAT_2M	1076.6	864	0.5226462	7	Singlet	Endothelial_cells
VAT_2M	1253.1	998	1.0690656	2	Singlet	FAPs
VAT_2M	3269.8	1802	1.0063415	0	Singlet	Adipocyte
VAT_2M	10169	3371	0.0080553	0	Singlet	Adipocyte
VAT_2M	5820.9	2480	0.0329849	0	Singlet	Adipocyte
VAT_2M	4152.6	1894	0.0644975	0	Singlet	Adipocyte
VAT_2M	4594.1	2142	0.8038793	0	Singlet	Adipocyte
VAT_2M	1633	1151	1.6435189	3	Singlet	FAPs
VAT_2M	4136.2	2210	0.2093475	2	Singlet	FAPs
VAT_2M	6672.6	2848	0	0	Singlet	Adipocyte
VAT_2M	1025.4	880	1.8599978	4	Singlet	Immune_cells
VAT_2M	1931.5	1352	1.1860601	3	Singlet	FAPs
VAT_2M	1177.3	1019	2.2762094	6	Singlet	Neurons
VAT_2M	4794	2214	0.0904931	0	Singlet	Adipocyte
VAT_2M	1812.7	1226	0.2605905	3	Singlet	FAPs
VAT_2M	6241.5	2531	0.0583211	0	Singlet	Adipocyte
VAT_2M	1537.6	1045	0.0143804	3	Singlet	FAPs
VAT_2M	4016.6	2049	0.9375907	0	Singlet	Adipocyte
VAT_2M	1262.3	950	3.0416313	6	Singlet	Neurons
VAT_2M	2978.5	1637	5.0162017	0	Singlet	Adipocyte
VAT_2M	1407	1084	1.2053241	11	Singlet	Smooth_muscle_cells
VAT_2M	2395.8	1185	1.1959156	0	Singlet	Adipocyte
VAT_2M	1155.7	882	1.0155711	3	Singlet	FAPs
VAT_2M	1606	938	12.610615	7	Singlet	Endothelial_cells
VAT_2M	6307.8	2657	0.1027111	0	Singlet	Adipocyte
VAT_2M	1780.3	1262	2.2066737	0	Singlet	Adipocyte
VAT_2M	1088.5	834	0.8304975	5	Singlet	Immune_cells
VAT_2M	6478.7	2774	0.1577645	0	Singlet	Adipocyte
VAT_2M	1030.4	908	1.7396737	1	Singlet	Immune_cells
VAT_2M	1140.8	908	1.9682831	11	Singlet	Smooth_muscle_cells
VAT_2M	1604.6	1175	0.1064921	8	Singlet	Mesothelial
VAT_2M	2540.5	1291	0.1511175	0	Singlet	Adipocyte
VAT_2M	5915.4	2494	0.3395203	0	Singlet	Adipocyte
VAT_2M	4966.2	2098	0.5251692	0	Singlet	Adipocyte
VAT_2M	2085.1	1400	0.434964	2	Singlet	FAPs
VAT_2M	6515.7	2718	0.0794293	0	Singlet	Adipocyte
VAT_2M	7279.7	2714	0.0364894	0	Singlet	Adipocyte
VAT_2M	1322.3	968	0.9290842	2	Singlet	FAPs
VAT_2M	4492	1889	10.263453	0	Singlet	Adipocyte
VAT_2M	5169.1	2319	0.0465249	0	Singlet	Adipocyte
VAT_2M	4342	2124	0.0672805	0	Singlet	Adipocyte
VAT_2M	1777.3	1063	0.0080909	0	Singlet	Adipocyte

VAT_2M	1204	963	0.8873995	7	Singlet	Endothelial_cells
VAT_2M	7695.3	3101	0.0015114	0	Singlet	Adipocyte
VAT_2M	3920.9	1853	0.0775581	0	Singlet	Adipocyte
VAT_2M	1916.7	1359	0.1867055	6	Singlet	Neurons
VAT_2M	1150.1	683	10.676431	0	Singlet	Adipocyte
VAT_2M	1303.4	1079	2.4551011	6	Singlet	Neurons
VAT_2M	6475.8	2638	0.0247405	0	Singlet	Adipocyte
VAT_2M	1710.8	1367	2.3615016	4	Singlet	Immune_cells
VAT_2M	1327.8	898	0.695964	3	Singlet	FAPs
VAT_2M	1047.7	802	0.5461103	5	Singlet	Immune_cells
VAT_2M	3562.9	1882	0	0	Singlet	Adipocyte
VAT_2M	2646.5	1726	0.8473118	6	Singlet	Neurons
VAT_2M	4298.7	2138	0.4091873	0	Singlet	Adipocyte
VAT_2M	4749	2331	0.1471836	0	Singlet	Adipocyte
VAT_2M	1733.2	1151	1.1936069	0	Singlet	Adipocyte
VAT_2M	4076.5	1986	1.393924	0	Singlet	Adipocyte
VAT_2M	1547	1305	0.6125369	6	Singlet	Neurons
VAT_2M	1346	976	0.2200839	6	Singlet	Neurons
VAT_2M	7038.2	2825	0.0173784	0	Singlet	Adipocyte
VAT_2M	2801.5	1536	0.6364222	0	Singlet	Adipocyte
VAT_2M	2740.2	1766	1.2166241	2	Singlet	FAPs
VAT_2M	3940.8	2148	0.4275664	0	Singlet	Adipocyte
VAT_2M	3098.9	1758	0.2977291	0	Singlet	Adipocyte
VAT_2M	1351.2	1070	0.1554237	2	Singlet	FAPs
VAT_2M	2253.7	1284	0.8972626	0	Singlet	Adipocyte
VAT_2M	2054.8	1276	0.7028568	3	Singlet	FAPs
VAT_2M	1903	1315	1.0260977	5	Singlet	Immune_cells
VAT_2M	6446	2688	0.0315184	0	Singlet	Adipocyte
VAT_2M	6093.8	2571	0.3853833	0	Singlet	Adipocyte
VAT_2M	3019.3	1642	0.3711841	0	Singlet	Adipocyte
VAT_2M	7881.5	3071	0.0279434	0	Singlet	Adipocyte
VAT_2M	1904.9	1571	2.3553007	6	Singlet	Neurons
VAT_2M	1020.9	759	3.9665291	6	Singlet	Neurons
VAT_2M	3665.1	1693	5.578255	2	Singlet	FAPs
VAT_2M	3191.8	1741	1.7157069	0	Singlet	Adipocyte
VAT_2M	2482	1541	2.3669152	0	Singlet	Adipocyte
VAT_2M	9037.4	3198	0.0454282	0	Singlet	Adipocyte
VAT_2M	4767.3	2165	0	0	Singlet	Adipocyte
VAT_2M	3007.4	1719	0.3212973	0	Singlet	Adipocyte
VAT_2M	1666.6	1189	1.3089721	3	Singlet	FAPs
VAT_2M	1782.7	1245	2.1395919	5	Singlet	Immune_cells
VAT_2M	4153.2	1877	0.3262733	0	Singlet	Adipocyte
VAT_2M	7891.9	2864	0.1017007	0	Singlet	Adipocyte
VAT_2M	4738.3	2224	0.0917687	0	Singlet	Adipocyte
VAT_2M	1009.1	849	0.5324671	6	Singlet	Neurons
VAT_2M	4581	2106	0.1323143	0	Singlet	Adipocyte
VAT_2M	2332.2	1346	1.9509407	7	Singlet	Endothelial_cells
VAT_2M	2509.6	1515	12.385848	11	Singlet	Smooth_muscle_cells
VAT_2M	8077.2	3096	2.8477586	0	Singlet	Adipocyte
VAT_2M	8085.4	3074	0	0	Singlet	Adipocyte
VAT_2M	3233.8	1795	0.3797256	0	Singlet	Adipocyte

VAT_2M	2542.6	1402	0.1340528	3	Singlet	FAPs
VAT_2M	7291.4	2799	0.0164373	0	Singlet	Adipocyte
VAT_2M	1050.5	789	0.3149929	5	Singlet	Immune_cells
VAT_2M	1910.3	1125	0.0406088	0	Singlet	Adipocyte
VAT_2M	2479.3	1526	1.9885365	3	Singlet	FAPs
VAT_2M	1564.7	1139	2.1427336	3	Singlet	FAPs
VAT_2M	2043.4	1141	0.0141001	0	Singlet	Adipocyte
VAT_2M	1597.1	1143	0.3223486	5	Singlet	Immune_cells
VAT_2M	1983.9	1331	0.2667293	5	Singlet	Immune_cells
VAT_2M	1409.9	921	4.3342807	7	Singlet	Endothelial_cells
VAT_2M	1540.9	1042	0.4574152	2	Singlet	FAPs
VAT_2M	1120.5	812	0.1424785	7	Singlet	Endothelial_cells
VAT_2M	3410.7	1761	0.4357412	0	Singlet	Adipocyte
VAT_2M	2876.4	1769	5.7779161	7	Singlet	Endothelial_cells
VAT_2M	1660.8	1317	0.8883323	7	Singlet	Endothelial_cells
VAT_2M	1727.4	1067	1.7300761	2	Singlet	FAPs
VAT_2M	8647.7	3044	0	0	Singlet	Adipocyte
VAT_2M	6045.5	2462	0.0316988	0	Singlet	Adipocyte
VAT_2M	5803.5	2342	0.1511074	0	Singlet	Adipocyte
VAT_2M	3471.5	1795	0.0720603	0	Singlet	Adipocyte
VAT_2M	2354.9	1586	0.827206	2	Singlet	FAPs
VAT_2M	2001.7	1357	0.2660361	2	Singlet	FAPs
VAT_2M	4998	2227	0.0065036	0	Singlet	Adipocyte
VAT_2M	2655.3	1676	0.2699785	3	Singlet	FAPs
VAT_2M	8287.1	3103	0.0585782	0	Singlet	Adipocyte
VAT_2M	6480.3	2788	0.0063545	0	Singlet	Adipocyte
VAT_2M	4871.9	2679	4.0346665	7	Singlet	Endothelial_cells
VAT_2M	2037.4	1281	0.0391722	0	Singlet	Adipocyte
VAT_2M	2962.4	1699	0.0747589	0	Singlet	Adipocyte
VAT_2M	1832	1188	1.7050043	2	Singlet	FAPs
VAT_2M	5423.5	2412	0.7218706	0	Singlet	Adipocyte
VAT_2M	2542.3	1596	0.2867845	6	Singlet	Neurons
VAT_2M	1799.7	1181	0.5426843	5	Singlet	Immune_cells
VAT_2M	8238.8	2940	0.0151812	0	Singlet	Adipocyte
VAT_2M	1009.2	853	1.1762008	6	Singlet	Neurons
VAT_2M	9988.4	3264	1.3373044	0	Singlet	Adipocyte
VAT_2M	3189.2	1966	1.3659644	2	Singlet	FAPs
VAT_2M	2209.3	1528	1.4959862	6	Singlet	Neurons
VAT_2M	4578.9	2208	0.0532671	0	Singlet	Adipocyte
VAT_2M	1028.8	842	0.1079551	10	Singlet	Immune_cells
VAT_2M	3672.7	1796	0.4603744	0	Singlet	Adipocyte
VAT_2M	7403.8	2860	0.1628311	0	Singlet	Adipocyte
VAT_2M	9272.4	3093	0.0826097	0	Singlet	Adipocyte
VAT_2M	1628.9	1051	1.5155294	0	Singlet	Adipocyte
VAT_2M	4388.3	1940	0.5947598	0	Singlet	Adipocyte
VAT_2M	1795.7	1418	0.5268032	6	Singlet	Neurons
VAT_2M	5547.2	2386	0.0181473	0	Singlet	Adipocyte
VAT_2M	7581.1	3103	0	0	Singlet	Adipocyte
VAT_2M	5090.1	2176	0.0166457	0	Singlet	Adipocyte
VAT_2M	1192	892	1.5591664	3	Singlet	FAPs
VAT_2M	1544.2	1247	1.6691653	11	Singlet	Smooth_muscle_cells

VAT_2M	2109.2	1268	0.4047951	0	Singlet	Adipocyte
VAT_2M	1015.6	833	0.7989494	6	Singlet	Neurons
VAT_2M	1398.9	1110	0.3776774	1	Singlet	Immune_cells
VAT_2M	1942.9	1351	0.2857423	0	Singlet	Adipocyte
VAT_2M	3486.9	1748	0.0175365	0	Singlet	Adipocyte
VAT_2M	1223.4	1017	2.1611643	6	Singlet	Neurons
VAT_2M	6385.9	2740	0.0511811	0	Singlet	Adipocyte
VAT_2M	1302.9	956	0.3594607	10	Singlet	Immune_cells
VAT_2M	4809.8	2141	0.0801498	0	Singlet	Adipocyte
VAT_2M	4838.3	2178	0.0508847	0	Singlet	Adipocyte
VAT_2M	9146.1	3281	0.003629	0	Singlet	Adipocyte
VAT_2M	2034.4	1184	0.3146857	0	Singlet	Adipocyte
VAT_2M	1312.3	1031	0.8628726	2	Singlet	FAPs
VAT_2M	2478	1591	3.0541879	6	Singlet	Neurons
VAT_2M	2134.7	1418	0.117806	2	Singlet	FAPs
VAT_2M	2484.5	1532	0.5365866	2	Singlet	FAPs
VAT_2M	2445.8	1603	0.9026704	3	Singlet	FAPs
VAT_2M	2259.9	1381	2.3543831	5	Singlet	Immune_cells
VAT_2M	2616.3	1502	0.151232	0	Singlet	Adipocyte
VAT_2M	6471.4	2642	0.0038245	0	Singlet	Adipocyte
VAT_2M	9391.3	3189	0.0581654	0	Singlet	Adipocyte
VAT_2M	1351.2	1000	0.8920721	7	Singlet	Endothelial_cells
VAT_2M	1031.9	780	1.7322667	2	Singlet	FAPs
VAT_2M	3038.6	1524	11.081742	7	Singlet	Endothelial_cells
VAT_2M	3276	1808	0.0220398	0	Singlet	Adipocyte
VAT_2M	1007.9	826	1.8453661	7	Singlet	Endothelial_cells
VAT_2M	7872.6	2862	0.0140386	0	Singlet	Adipocyte
VAT_2M	1540.4	1066	0.856954	5	Singlet	Immune_cells
VAT_2M	1071.6	801	1.8560037	2	Singlet	FAPs
VAT_2M	7313.8	2888	0.034344	0	Singlet	Adipocyte
VAT_2M	1805.3	1187	4.6926748	3	Singlet	FAPs
VAT_2M	5270.4	2310	0.2293504	0	Singlet	Adipocyte
VAT_2M	2968.8	1874	1.0925742	5	Singlet	Immune_cells
VAT_2M	5137	2305	0.0786457	0	Singlet	Adipocyte
VAT_2M	2032.5	1445	2.7221157	2	Singlet	FAPs
VAT_2M	1002.3	785	0.2512607	1	Singlet	Immune_cells
VAT_2M	1473.9	1126	1.8946407	2	Singlet	FAPs
VAT_2M	3087.3	1764	1.5195054	5	Singlet	Immune_cells
VAT_2M	2385.3	1407	0.0494048	3	Singlet	FAPs
VAT_2M	4060.2	2024	0.2906086	0	Singlet	Adipocyte
VAT_2M	1053.8	772	0.076362	3	Singlet	FAPs
VAT_2M	1824.2	1281	0.5722234	6	Singlet	Neurons
VAT_2M	1762.3	1213	0	7	Singlet	Endothelial_cells
VAT_2M	8535.8	3203	0.0150219	0	Singlet	Adipocyte
VAT_2M	2078.6	1428	0.1496117	3	Singlet	FAPs
VAT_2M	1106.5	781	0.1233961	7	Singlet	Endothelial_cells
VAT_2M	1233.3	875	0.4601608	7	Singlet	Endothelial_cells
VAT_2M	1012	807	1.9009753	2	Singlet	FAPs
VAT_2M	1074.6	813	1.2166943	6	Singlet	Neurons
VAT_2M	8942.9	3183	0.000129	0	Singlet	Adipocyte
VAT_2M	2130.5	1435	2.5158365	5	Singlet	Immune_cells

VAT_2M	1418.4	992	7.3992282	5	Singlet	Immune_cells
VAT_2M	1156.4	964	1.6998814	6	Singlet	Neurons
VAT_2M	1131.4	756	1.1341052	5	Singlet	Immune_cells
VAT_2M	3957	2035	0.0151714	0	Singlet	Adipocyte
VAT_2M	2011	1667	0.0507874	6	Singlet	Neurons
VAT_2M	1048.4	798	2.246934	0	Singlet	Adipocyte
VAT_2M	1118.2	869	0.8946763	11	Singlet	Smooth_muscle_cells
VAT_2M	11107	3584	0.0127724	0	Singlet	Adipocyte
VAT_2M	1074	900	4.2438126	6	Singlet	Neurons
VAT_2M	3280.5	1812	0.9956406	3	Singlet	FAPs
VAT_2M	1792.1	1245	0.2659886	5	Singlet	Immune_cells
VAT_2M	1239.1	983	2.1562925	5	Singlet	Immune_cells
VAT_2M	3084.6	1571	3.0237697	2	Singlet	FAPs
VAT_2M	2283.1	1636	1.0505019	2	Singlet	FAPs
VAT_2M	2982.9	1789	2.3634222	0	Singlet	Adipocyte
VAT_2M	1337.9	984	0.1517569	3	Singlet	FAPs
VAT_2M	2065.9	1520	1.3028608	2	Singlet	FAPs
VAT_2M	2657.2	1657	3.110888	3	Singlet	FAPs
VAT_2M	1338	1055	1.8186656	6	Singlet	Neurons
VAT_2M	1505.2	1113	0.8160162	2	Singlet	FAPs
VAT_2M	2776.6	1583	0.503308	0	Singlet	Adipocyte
VAT_2M	4458.6	2037	0.0679869	0	Singlet	Adipocyte
VAT_2M	1486.8	1049	4.031025	5	Singlet	Immune_cells
VAT_2M	1198.7	951	0.7417347	6	Singlet	Neurons
VAT_2M	1916.5	1248	0.4871932	0	Singlet	Adipocyte
VAT_2M	2376.7	1378	0.0603808	0	Singlet	Adipocyte
VAT_2M	10227	3262	0.1129474	0	Singlet	Adipocyte
VAT_2M	4142.2	2139	0.1025776	0	Singlet	Adipocyte
VAT_2M	10217	3320	0	0	Singlet	Adipocyte
VAT_2M	1756.2	1320	0.0997843	6	Singlet	Neurons
VAT_2M	1773	1301	0.3839379	7	Singlet	Endothelial_cells
VAT_2M	6971	2853	0.0834291	0	Singlet	Adipocyte
VAT_2M	3477.6	1880	0	0	Singlet	Adipocyte
VAT_2M	1248.1	871	0.3360109	6	Singlet	Neurons
VAT_2M	1132.7	898	1.2863062	11	Singlet	Smooth_muscle_cells
VAT_2M	3462.9	1737	0.2390183	0	Singlet	Adipocyte
VAT_2M	3561.1	1795	3.2484241	0	Singlet	Adipocyte
VAT_2M	2417.4	1476	0.7022952	2	Singlet	FAPs
VAT_2M	1661.9	1158	1.0704928	3	Singlet	FAPs
VAT_2M	1107.2	842	0.6104447	2	Singlet	FAPs
VAT_2M	1747.7	1182	0.1662543	0	Singlet	Adipocyte
VAT_2M	1401.4	1077	0.5294379	2	Singlet	FAPs
VAT_2M	8144.5	3112	0.1247112	0	Singlet	Adipocyte
VAT_2M	7318.5	2984	0.0268113	0	Singlet	Adipocyte
VAT_2M	2474.8	1636	0.2789466	3	Singlet	FAPs
VAT_2M	1024	820	0.28677	6	Singlet	Neurons
VAT_2M	3107.6	1606	0.7019996	0	Singlet	Adipocyte
VAT_2M	4178.3	1937	0.0300736	0	Singlet	Adipocyte
VAT_2M	3292.4	1787	2.0131636	0	Singlet	Adipocyte
VAT_2M	5924.1	2461	0.0106806	0	Singlet	Adipocyte
VAT_2M	13045	3769	0	0	Singlet	Adipocyte

VAT_2M	1562.6	1302	0.8657458	6	Singlet	Neurons
VAT_2M	2861.5	1555	0.1682135	0	Singlet	Adipocyte
VAT_2M	7071.2	2878	0.0824888	0	Singlet	Adipocyte
VAT_2M	1583.2	1073	0.5587649	2	Singlet	FAPs
VAT_2M	3491.7	1920	0.4723896	0	Singlet	Adipocyte
VAT_2M	1341.2	877	3.2862786	9	Singlet	Mesothelial
VAT_2M	1067.9	896	0.644217	6	Singlet	Neurons
VAT_2M	5133.2	2341	0.1650636	0	Singlet	Adipocyte
VAT_2M	9211.9	3302	0.0505767	0	Singlet	Adipocyte
VAT_2M	11035	3714	0	0	Singlet	Adipocyte
VAT_2M	9606.8	3299	0.041922	0	Singlet	Adipocyte
VAT_2M	4035.7	2105	0.0294203	0	Singlet	Adipocyte
VAT_2M	1319.2	975	2.2305277	2	Singlet	FAPs
VAT_2M	1975.8	1238	0.4603794	0	Singlet	Adipocyte
VAT_2M	4849.8	2214	0.0580009	0	Singlet	Adipocyte
VAT_2M	2097.5	1431	1.1729762	2	Singlet	FAPs
VAT_2M	7805.7	3061	0.0765427	0	Singlet	Adipocyte
VAT_2M	1403.9	972	0.2638888	0	Singlet	Adipocyte
VAT_2M	1715.1	1115	2.7268505	7	Singlet	Endothelial_cells
VAT_2M	9054.3	3084	0.0389434	0	Singlet	Adipocyte
VAT_2M	4226.5	2041	0.0123215	0	Singlet	Adipocyte
VAT_2M	1654.1	1195	2.5782972	5	Singlet	Immune_cells
VAT_2M	6493.5	2791	0.0159983	0	Singlet	Adipocyte
VAT_2M	1681	1152	2.1700342	2	Singlet	FAPs
VAT_2M	1792.8	1283	1.7246298	2	Singlet	FAPs
VAT_2M	1577.9	1030	1.411461	0	Singlet	Adipocyte
VAT_2M	1815.9	1274	4.186301	6	Singlet	Neurons
VAT_2M	2702.1	1566	0.5274431	0	Singlet	Adipocyte
VAT_2M	1893.6	1177	3.0559717	2	Singlet	FAPs
VAT_2M	1497.9	1093	1.5585104	0	Singlet	Adipocyte
VAT_2M	11229	3699	0.0958807	0	Singlet	Adipocyte
VAT_2M	1187.4	840	5.2591995	2	Singlet	FAPs
VAT_2M	6189	2623	0.2319999	0	Singlet	Adipocyte
VAT_2M	1883.4	1170	0.268249	0	Singlet	Adipocyte
VAT_2M	1140.8	975	2.2607125	6	Singlet	Neurons
VAT_2M	1537.3	1204	0.6330957	11	Singlet	Smooth_muscle_cells
VAT_2M	1825.2	1216	0.375443	3	Singlet	FAPs
VAT_2M	3878.9	1996	0.0163502	0	Singlet	Adipocyte
VAT_2M	1822.3	1340	0.4671371	2	Singlet	FAPs
VAT_2M	6027.7	2490	0.8784849	0	Singlet	Adipocyte
VAT_2M	1362.7	730	14.545933	5	Singlet	Immune_cells
VAT_2M	1236.7	892	2.6976564	9	Singlet	Mesothelial
VAT_2M	3266.9	1764	0.0885204	0	Singlet	Adipocyte
VAT_2M	5349.8	2198	0.0577611	0	Singlet	Adipocyte
VAT_2M	3365.8	1856	0.2096093	2	Singlet	FAPs
VAT_2M	6813.3	2802	0.0393338	0	Singlet	Adipocyte
VAT_2M	2242	1372	0.4692838	5	Singlet	Immune_cells
VAT_2M	10680	3447	0	0	Singlet	Adipocyte
VAT_2M	4441.6	2109	0.3788169	0	Singlet	Adipocyte
VAT_2M	5525.8	2673	1.2146241	5	Singlet	Immune_cells
VAT_2M	5250.1	2444	0	0	Singlet	Adipocyte

VAT_2M	1840.7	1036	0.1930876	0	Singlet	Adipocyte
VAT_2M	2716.6	1513	0	0	Singlet	Adipocyte
VAT_2M	1368.9	1019	1.0023983	6	Singlet	Neurons
VAT_2M	5066.3	2410	0.174834	0	Singlet	Adipocyte
VAT_2M	2137.8	1276	0.8837509	0	Singlet	Adipocyte
VAT_2M	1494.5	1131	0.6685985	2	Singlet	FAPs
VAT_2M	4293.2	2314	0.2092241	2	Singlet	FAPs
VAT_2M	3428.7	2125	0.3238545	2	Singlet	FAPs
VAT_2M	4376.2	2106	0.0150184	0	Singlet	Adipocyte
VAT_2M	3752.4	1886	0.0867438	0	Singlet	Adipocyte
VAT_2M	3572	2107	1.4774041	5	Singlet	Immune_cells
VAT_2M	1240.4	864	2.7795696	9	Singlet	Mesothelial
VAT_2M	3655	1907	0.0370247	0	Singlet	Adipocyte
VAT_2M	2004.5	1341	0.591467	0	Singlet	Adipocyte
VAT_2M	5094.2	2393	0.1953192	0	Singlet	Adipocyte
VAT_2M	6954.2	2993	0.0966143	0	Singlet	Adipocyte
VAT_2M	6232	2556	0.0230255	0	Singlet	Adipocyte
VAT_2M	6314.3	2448	0.213191	0	Singlet	Adipocyte
VAT_2M	1504.8	1189	0.7986752	1	Singlet	Immune_cells
VAT_2M	1787.6	1342	0.66155	1	Singlet	Immune_cells
VAT_2M	1164	836	1.7816532	6	Singlet	Neurons
VAT_2M	2272.8	1469	1.0751642	2	Singlet	FAPs
VAT_2M	1581.6	1126	2.9794838	6	Singlet	Neurons
VAT_2M	6732.3	2786	0.0194337	0	Singlet	Adipocyte
VAT_2M	3746.3	1945	1.2890847	0	Singlet	Adipocyte
VAT_2M	1043.6	835	3.4385543	9	Singlet	Mesothelial
VAT_2M	7946.9	2912	0.0151742	0	Singlet	Adipocyte
VAT_2M	7144.1	2707	0.017068	0	Singlet	Adipocyte
VAT_2M	6341.5	2665	0.1444984	0	Singlet	Adipocyte
VAT_2M	1045.3	810	1.1451864	2	Singlet	FAPs
VAT_2M	3626.5	2180	0.231859	2	Singlet	FAPs
VAT_2M	7660.9	2970	0.0122673	0	Singlet	Adipocyte
VAT_2M	1172.9	850	0.6852049	9	Singlet	Mesothelial
VAT_2M	1038.9	891	0.8747055	6	Singlet	Neurons
VAT_2M	1212.5	1033	2.9841465	6	Singlet	Neurons
VAT_2M	1153.9	952	1.0275643	9	Singlet	Mesothelial
VAT_2M	1941.3	1267	0.5765661	3	Singlet	FAPs
VAT_2M	6830.9	2611	0.2565781	0	Singlet	Adipocyte
VAT_2M	1153	838	0.8148406	6	Singlet	Neurons
VAT_2M	6649.9	2556	1.3519046	0	Singlet	Adipocyte
VAT_2M	2137.2	1313	1.827688	6	Singlet	Neurons
VAT_2M	7106.3	2628	0	0	Singlet	Adipocyte
VAT_2M	7252.7	2961	0.0485133	0	Singlet	Adipocyte
VAT_2M	1471.6	1095	1.434027	5	Singlet	Immune_cells
VAT_2M	3181.9	1698	0.0452945	0	Singlet	Adipocyte
VAT_2M	1736	1141	0.4230057	7	Singlet	Endothelial_cells
VAT_2M	1439.1	945	0.2011235	3	Singlet	FAPs
VAT_2M	2346.6	1524	0.4019462	7	Singlet	Endothelial_cells
VAT_2M	1000.6	769	1.2912432	2	Singlet	FAPs
VAT_2M	6401.4	2695	0.0187857	0	Singlet	Adipocyte
VAT_2M	3317.9	1972	1.6025875	2	Singlet	FAPs

VAT_2M	1191.7	960	0.6240718	6	Singlet	Neurons
VAT_2M	1376.4	967	1.5354102	5	Singlet	Immune_cells
VAT_2M	2656.3	1544	0	0	Singlet	Adipocyte
VAT_2M	7080.3	2840	0.0596191	0	Singlet	Adipocyte
VAT_2M	1001.7	817	1.0056568	6	Singlet	Neurons
VAT_2M	1062.8	858	1.3617685	6	Singlet	Neurons
VAT_2M	1117.9	858	1.4386894	2	Singlet	FAPs
VAT_2M	3051.4	1962	0.0657317	5	Singlet	Immune_cells
VAT_2M	3200.5	1642	0	0	Singlet	Adipocyte
VAT_2M	9499.6	3347	0.0218168	0	Singlet	Adipocyte
VAT_2M	3306.1	1873	0.1961233	3	Singlet	FAPs
VAT_2M	2574.7	1694	0.0301859	3	Singlet	FAPs
VAT_2M	2149.5	1470	1.5802264	3	Singlet	FAPs
VAT_2M	1786	1181	0.4268426	7	Singlet	Endothelial_cells
VAT_2M	1708.5	1189	1.3327236	6	Singlet	Neurons
VAT_2M	7857.4	2984	0.0304177	0	Singlet	Adipocyte
VAT_2M	8672	3157	0.0350016	0	Singlet	Adipocyte
VAT_2M	4582.9	2263	0.0890098	0	Singlet	Adipocyte
VAT_2M	6376.1	2623	0.0359871	0	Singlet	Adipocyte
VAT_2M	7044	2914	0.0405472	0	Singlet	Adipocyte
VAT_2M	1801.8	906	8.4493274	7	Singlet	Endothelial_cells
VAT_2M	5681.5	2508	0.0514297	0	Singlet	Adipocyte
VAT_2M	1892.6	1210	2.736833	3	Singlet	FAPs
VAT_2M	1598.9	1067	0.8669678	7	Singlet	Endothelial_cells
VAT_2M	2233.3	1306	1.7506528	0	Singlet	Adipocyte
VAT_2M	1039.1	842	0	7	Singlet	Endothelial_cells
VAT_2M	5221.5	2370	0	0	Singlet	Adipocyte
VAT_2M	2373.9	1476	0.0831529	0	Singlet	Adipocyte
VAT_2M	2443.1	1394	0.9331734	0	Singlet	Adipocyte
VAT_2M	4774.1	2160	0.1301592	0	Singlet	Adipocyte
VAT_2M	1263.3	947	0.9128006	3	Singlet	FAPs
VAT_2M	9949.9	2908	0.0092836	0	Singlet	Adipocyte
VAT_2M	1389.8	1044	0.6851619	2	Singlet	FAPs
VAT_2M	2108.3	1440	0.6178269	2	Singlet	FAPs
VAT_2M	1276.1	1012	1.1973436	7	Singlet	Endothelial_cells
VAT_2M	1118.8	903	0.6219339	6	Singlet	Neurons
VAT_2M	1718	1204	0.8549411	2	Singlet	FAPs
VAT_2M	1358	984	2.8228508	5	Singlet	Immune_cells
VAT_2M	3865.1	1921	0.1238254	0	Singlet	Adipocyte
VAT_2M	5984.2	2640	0.2186114	0	Singlet	Adipocyte
VAT_2M	1049.5	830	1.9771436	3	Singlet	FAPs
VAT_2M	1749.4	1247	0.355427	7	Singlet	Endothelial_cells
VAT_2M	2224.7	1317	2.8836262	5	Singlet	Immune_cells
VAT_2M	1896.4	1185	0.6837618	0	Singlet	Adipocyte
VAT_2M	3760.5	1915	0.028672	0	Singlet	Adipocyte
VAT_2M	1913.1	1353	0.6973781	2	Singlet	FAPs
VAT_2M	1616.6	1095	2.3548309	2	Singlet	FAPs
VAT_2M	6523	2543	1.592568	0	Singlet	Adipocyte
VAT_2M	1369.9	1015	0.2054007	6	Singlet	Neurons
VAT_2M	1172.3	851	0.6035251	6	Singlet	Neurons
VAT_2M	4439.5	2174	0.0647836	0	Singlet	Adipocyte

VAT_2M	5651.2	2645	0.0858553	0	Singlet	Adipocyte
VAT_2M	3272.9	1774	1.5765354	0	Singlet	Adipocyte
VAT_2M	2528.1	1566	0.4726522	0	Singlet	Adipocyte
VAT_2M	2707.4	1548	0.0954216	0	Singlet	Adipocyte
VAT_2M	2680.2	1513	0	0	Singlet	Adipocyte
VAT_2M	8915.6	3288	0.0468541	0	Singlet	Adipocyte
VAT_2M	2534.6	1493	0.0184996	7	Singlet	Endothelial_cells
VAT_2M	1354.8	952	2.2282323	2	Singlet	FAPs
VAT_2M	1737.3	1161	2.2472224	3	Singlet	FAPs
VAT_2M	1343.2	1009	2.1856939	5	Singlet	Immune_cells
VAT_2M	1179.7	842	1.4961302	5	Singlet	Immune_cells
VAT_2M	6863.7	2701	0.0115708	0	Singlet	Adipocyte
VAT_2M	2949.7	1673	0	0	Singlet	Adipocyte
VAT_2M	1348.6	1103	2.8130137	6	Singlet	Neurons
VAT_2M	1590.3	1065	1.1744317	2	Singlet	FAPs
VAT_2M	3639.2	1819	0.8367946	0	Singlet	Adipocyte
VAT_2M	4222.1	2171	0.6769069	3	Singlet	FAPs
VAT_2M	1954.9	1256	0.1762562	2	Singlet	FAPs
VAT_2M	2365.5	1435	0.6808384	0	Singlet	Adipocyte
VAT_2M	1379.3	989	0.4568305	2	Singlet	FAPs
VAT_2M	2010.3	1320	1.8313168	5	Singlet	Immune_cells
VAT_2M	1123.2	858	3.9716901	9	Singlet	Mesothelial
VAT_2M	1511.1	984	2.887755	5	Singlet	Immune_cells
VAT_2M	1997	1416	0.3233673	3	Singlet	FAPs
VAT_2M	1098.9	837	2.448025	5	Singlet	Immune_cells
VAT_2M	4816.8	2186	0.0095968	0	Singlet	Adipocyte
VAT_2M	1611.2	1125	0.9245431	3	Singlet	FAPs
VAT_2M	1615.7	1093	1.306558	7	Singlet	Endothelial_cells
VAT_2M	7019.1	2665	0.1171887	0	Singlet	Adipocyte
VAT_2M	2202.5	1343	0.5707987	0	Singlet	Adipocyte
VAT_2M	1187.9	909	2.3031912	9	Singlet	Mesothelial
VAT_2M	3306.7	2021	0.3400321	7	Singlet	Endothelial_cells
VAT_2M	7349.8	2892	0.0723881	0	Singlet	Adipocyte
VAT_2M	2049.1	1125	0	0	Singlet	Adipocyte
VAT_2M	7116.3	2883	0.1892433	0	Singlet	Adipocyte
VAT_2M	4897.9	2322	0.0773444	0	Singlet	Adipocyte
VAT_2M	3001.4	1950	0.47267	7	Singlet	Endothelial_cells
VAT_2M	2039.9	1307	1.0245379	0	Singlet	Adipocyte
VAT_2M	7790.9	3076	0.1057112	0	Singlet	Adipocyte
VAT_2M	1772.6	1484	0.4382748	6	Singlet	Neurons
VAT_2M	2598.7	1640	0.881838	3	Singlet	FAPs
VAT_2M	1642.7	1140	0.0707709	3	Singlet	FAPs
VAT_2M	5154.5	2241	0.0611899	0	Singlet	Adipocyte
VAT_2M	7202.5	2776	0.0065828	0	Singlet	Adipocyte
VAT_2M	1691.2	1067	0.9342349	0	Singlet	Adipocyte
VAT_2M	5806.3	2576	0.0206667	0	Singlet	Adipocyte
VAT_2M	1914.3	1324	0.6232031	1	Singlet	Immune_cells
VAT_2M	2552.1	1670	0.1266152	2	Singlet	FAPs
VAT_2M	2160.3	1491	1.8993731	3	Singlet	FAPs
VAT_2M	7609.3	2949	0.044727	0	Singlet	Adipocyte
VAT_2M	8891.8	3218	0.0868667	0	Singlet	Adipocyte

VAT_2M	1208.9	979	1.9752121	6	Singlet	Neurons
VAT_2M	6087	2737	0.0282527	0	Singlet	Adipocyte
VAT_2M	8343.5	3076	0.0463583	0	Singlet	Adipocyte
VAT_2M	3699.2	1868	0.0382889	0	Singlet	Adipocyte
VAT_2M	6026.6	3019	5.6753247	7	Singlet	Endothelial_cells
VAT_2M	1054.9	914	2.8267578	6	Singlet	Neurons
VAT_2M	1092.1	858	2.1709146	2	Singlet	FAPs
VAT_2M	5648.2	2271	0.3104653	0	Singlet	Adipocyte
VAT_2M	1564.9	1176	0.5755898	2	Singlet	FAPs
VAT_2M	1131.9	915	0.9549059	11	Singlet	Smooth_muscle_cells
VAT_2M	2800.7	1491	0.0204372	11	Singlet	Smooth_muscle_cells
VAT_2M	2666.5	1786	0.9854144	3	Singlet	FAPs
VAT_2M	1758.9	1149	1.115787	6	Singlet	Neurons
VAT_2M	8734.7	2967	0	0	Singlet	Adipocyte
VAT_2M	1964.1	1347	1.5640677	2	Singlet	FAPs
VAT_2M	3213.6	1735	1.5307712	0	Singlet	Adipocyte
VAT_2M	4314.7	2154	0.6188346	0	Singlet	Adipocyte
VAT_2M	2679.7	1493	0.2517865	0	Singlet	Adipocyte
VAT_2M	1650.5	1060	4.4369712	3	Singlet	FAPs
VAT_2M	1583.1	1010	0.9661136	0	Singlet	Adipocyte
VAT_2M	6424.3	2703	0.0665132	0	Singlet	Adipocyte
VAT_2M	1201.3	947	1.6849179	2	Singlet	FAPs
VAT_2M	7366.7	2787	0.0202572	0	Singlet	Adipocyte
VAT_2M	7278.9	3027	0.0036273	0	Singlet	Adipocyte
VAT_2M	1751	1121	0.552982	7	Singlet	Endothelial_cells
VAT_2M	2818.8	1617	0.2790598	7	Singlet	Endothelial_cells
VAT_2M	2420	1474	0.7245344	0	Singlet	Adipocyte
VAT_2M	1135.5	898	1.7281712	2	Singlet	FAPs
VAT_2M	4796.6	2086	0.0606258	0	Singlet	Adipocyte
VAT_2M	2782.5	1717	3.0896807	3	Singlet	FAPs
VAT_2M	2047.7	1298	0.071465	3	Singlet	FAPs
VAT_2M	2451.5	1454	0.2606356	0	Singlet	Adipocyte
VAT_2M	4359.9	2176	0.3356499	0	Singlet	Adipocyte
VAT_2M	3424.6	1831	0.0385573	0	Singlet	Adipocyte
VAT_2M	1142.7	839	3.0344585	5	Singlet	Immune_cells
VAT_2M	5235.2	2334	0.3001517	0	Singlet	Adipocyte
VAT_2M	1230.4	894	0.3153252	2	Singlet	FAPs
VAT_2M	2334.6	1610	1.8716695	2	Singlet	FAPs
VAT_2M	1574.5	1021	0.260606	0	Singlet	Adipocyte
VAT_2M	1614.8	1123	0.9310575	6	Singlet	Neurons
VAT_2M	1401.4	1059	6.4416609	11	Singlet	Smooth_muscle_cells
VAT_2M	7695.9	2978	0.0016156	0	Singlet	Adipocyte
VAT_2M	7346.1	2846	0.0162064	0	Singlet	Adipocyte
VAT_2M	1772	1284	0.8125566	5	Singlet	Immune_cells
VAT_2M	2294.5	1570	0.4881012	6	Singlet	Neurons
VAT_2M	1512.8	1092	2.1831999	5	Singlet	Immune_cells
VAT_2M	3078.6	1559	0.0553184	0	Singlet	Adipocyte
VAT_2M	2625.7	1546	1.5025336	0	Singlet	Adipocyte
VAT_2M	3703.5	2055	3.0290103	2	Singlet	FAPs
VAT_2M	1365.9	950	3.0144256	9	Singlet	Mesothelial
VAT_2M	5163	2323	0.0187349	0	Singlet	Adipocyte

VAT_2M	1614.9	1120	2.3906366	2	Singlet	FAPs
VAT_2M	1857.6	1251	1.1052367	2	Singlet	FAPs
VAT_2M	1215.6	1001	1.5259194	6	Singlet	Neurons
VAT_2M	3801.8	2164	1.4821542	2	Singlet	FAPs
VAT_2M	3869.4	2030	0.0465659	0	Singlet	Adipocyte
VAT_2M	3897	1903	0.1418353	0	Singlet	Adipocyte
VAT_2M	1816.4	1310	1.0728097	6	Singlet	Neurons
VAT_2M	2250	1502	0.383928	3	Singlet	FAPs
VAT_2M	3250.5	1845	0.2229278	0	Singlet	Adipocyte
VAT_2M	1959.1	1238	0.0393949	5	Singlet	Immune_cells
VAT_2M	4346.9	2267	0.6572244	0	Singlet	Adipocyte
VAT_2M	2559.2	1581	0.2584837	0	Singlet	Adipocyte
VAT_2M	6825.8	2758	0.2345954	0	Singlet	Adipocyte
VAT_2M	3355.2	1861	0	0	Singlet	Adipocyte
VAT_2M	7464.8	2994	1.1114751	0	Singlet	Adipocyte
VAT_2M	1405.4	1101	1.6411811	1	Singlet	Immune_cells
VAT_2M	7102.6	2706	0.0056744	0	Singlet	Adipocyte
VAT_2M	7493.6	2959	0	0	Singlet	Adipocyte
VAT_2M	1288.5	1050	2.5597674	8	Singlet	Mesothelial
VAT_2M	10251	3563	0.127767	0	Singlet	Adipocyte
VAT_2M	2113.4	1266	3.1818444	5	Singlet	Immune_cells
VAT_2M	1289	952	0.2818904	3	Singlet	FAPs
VAT_2M	1122.2	997	2.3899651	8	Singlet	Mesothelial
VAT_2M	1877.7	1212	0.5655481	6	Singlet	Neurons
VAT_2M	3436.1	1816	5.1808246	0	Singlet	Adipocyte
VAT_2M	4660.6	2217	0.0542451	0	Singlet	Adipocyte
VAT_2M	7047.9	2861	0.0201281	0	Singlet	Adipocyte
VAT_2M	2472.8	1456	0.0698508	0	Singlet	Adipocyte
VAT_2M	8092.3	3018	0.0842545	0	Singlet	Adipocyte
VAT_2M	3817.6	1823	0.1128824	0	Singlet	Adipocyte
VAT_2M	4590.7	2276	1.3392274	0	Singlet	Adipocyte
VAT_2M	6550.7	2561	0.1382815	0	Singlet	Adipocyte
VAT_2M	1929.8	1263	0.210526	7	Singlet	Endothelial_cells
VAT_2M	3329.7	1761	0.4921006	0	Singlet	Adipocyte
VAT_2M	2389.2	1403	0.019623	0	Singlet	Adipocyte
VAT_2M	8786.5	3164	0.4348877	0	Singlet	Adipocyte
VAT_2M	1638.3	1153	0.7692193	2	Singlet	FAPs
VAT_2M	3115.7	1860	0.0693786	2	Singlet	FAPs
VAT_2M	4011.3	1943	0.1072002	0	Singlet	Adipocyte
VAT_2M	3473.8	1917	0.0175748	0	Singlet	Adipocyte
VAT_2M	1115.5	831	1.577151	2	Singlet	FAPs
VAT_2M	2026.1	1320	0.1260335	5	Singlet	Immune_cells
VAT_2M	1703.1	1131	1.7211119	3	Singlet	FAPs
VAT_2M	1480.2	1242	1.7083693	1	Singlet	Immune_cells
VAT_2M	1356.8	895	0.4611036	7	Singlet	Endothelial_cells
VAT_2M	1034.2	829	1.9352671	3	Singlet	FAPs
VAT_2M	1265	935	0.540558	3	Singlet	FAPs
VAT_2M	1752.9	1260	0.0916257	6	Singlet	Neurons
VAT_2M	2773.9	1625	0.2202636	0	Singlet	Adipocyte
VAT_2M	6345.8	2617	0.0070131	0	Singlet	Adipocyte
VAT_2M	1798.4	1312	0.9703555	2	Singlet	FAPs

VAT_2M	8103	2895	0.1196847	0	Singlet	Adipocyte
VAT_2M	1113	828	0.4440977	9	Singlet	Mesothelial
VAT_2M	2688.6	1691	0.0250837	0	Singlet	Adipocyte
VAT_2M	2356	1321	0.7898354	0	Singlet	Adipocyte
VAT_2M	8476.6	3293	0.4222522	0	Singlet	Adipocyte
VAT_2M	6585.5	2729	0.189309	0	Singlet	Adipocyte
VAT_2M	2632.8	1552	0.0693157	0	Singlet	Adipocyte
VAT_2M	2549.7	1505	7.5059151	5	Singlet	Immune_cells
VAT_2M	3129.9	1810	0.1026837	0	Singlet	Adipocyte
VAT_2M	1219.9	1042	1.985359	8	Singlet	Mesothelial
VAT_2M	6270.9	2727	0.0046226	0	Singlet	Adipocyte
VAT_2M	1692.6	1311	0.2312503	7	Singlet	Endothelial_cells
VAT_2M	1112	854	0.7957351	1	Singlet	Immune_cells
VAT_2M	2317.9	1427	1.5401539	2	Singlet	FAPs
VAT_2M	1946.8	1168	0.4268444	0	Singlet	Adipocyte
VAT_2M	1215.9	794	3.9061489	9	Singlet	Mesothelial
VAT_2M	2451.8	1419	1.9967232	0	Singlet	Adipocyte
VAT_2M	2703.6	1782	0.9666487	2	Singlet	FAPs
VAT_2M	3522.7	1868	0.9300576	0	Singlet	Adipocyte
VAT_2M	2184.3	1486	0.1846007	2	Singlet	FAPs
VAT_2M	1501.9	1006	0.5300334	3	Singlet	FAPs
VAT_2M	2245.5	1461	0.3488095	3	Singlet	FAPs
VAT_2M	1468.8	1064	0.7634842	2	Singlet	FAPs
VAT_2M	1630.1	1086	0.3000907	3	Singlet	FAPs
VAT_2M	1033.7	750	5.038297	8	Singlet	Mesothelial
VAT_2M	4672.1	2157	0.0102652	0	Singlet	Adipocyte
VAT_2M	2839.3	1846	1.8084515	2	Singlet	FAPs
VAT_2M	1757.5	1092	0.2608568	0	Singlet	Adipocyte
VAT_2M	1298	985	1.1338778	2	Singlet	FAPs
VAT_2M	1744	1280	0.5042476	5	Singlet	Immune_cells
VAT_2M	2028	1333	0.2100192	6	Singlet	Neurons
VAT_2M	1111.1	890	1.1699194	6	Singlet	Neurons
VAT_2M	1022.8	841	2.0989884	10	Singlet	Immune_cells
VAT_2M	1723.6	1231	1.1225544	6	Singlet	Neurons
VAT_2M	1234	938	0.3195952	2	Singlet	FAPs
VAT_2M	1271.6	971	0.992648	2	Singlet	FAPs
VAT_2M	7822.8	2805	0.0266499	0	Singlet	Adipocyte
VAT_2M	4952.3	2425	0.2486589	0	Singlet	Adipocyte
VAT_2M	7430.1	2922	0.0099975	0	Singlet	Adipocyte
VAT_2M	1291.6	966	0.9878474	5	Singlet	Immune_cells
VAT_2M	2847.6	1578	0.2087851	2	Singlet	FAPs
VAT_2M	1342.4	1027	2.4763687	2	Singlet	FAPs
VAT_2M	2213.5	1481	0.6249275	0	Singlet	Adipocyte
VAT_2M	2378.2	1637	3.7940435	2	Singlet	FAPs
VAT_2M	7209.5	2908	0.0441923	0	Singlet	Adipocyte
VAT_2M	2794.3	1648	0.1770582	0	Singlet	Adipocyte
VAT_2M	1094.2	853	0.9112257	3	Singlet	FAPs
VAT_2M	1722.3	1228	1.9308648	2	Singlet	FAPs
VAT_2M	1069.9	747	0.0401586	5	Singlet	Immune_cells
VAT_2M	1991.3	1297	0.312797	2	Singlet	FAPs
VAT_2M	3084.2	1771	0.1462186	0	Singlet	Adipocyte

VAT_2M	5235.5	2512	0.1547124	0	Singlet	Adipocyte
VAT_2M	1555.2	987	0.5808854	7	Singlet	Endothelial_cells
VAT_2M	2315.1	1321	0.3899344	6	Singlet	Neurons
VAT_2M	7350	2765	0.1205901	0	Singlet	Adipocyte
VAT_2M	12324	3803	1.3099488	0	Singlet	Adipocyte
VAT_2M	4502.1	2170	0.002259	0	Singlet	Adipocyte
VAT_2M	1793.7	1032	7.7735491	7	Singlet	Endothelial_cells
VAT_2M	1946.3	1297	0.2663182	3	Singlet	FAPs
VAT_2M	1602.2	1208	0.2757844	6	Singlet	Neurons
VAT_2M	1467.9	1205	1.0196535	5	Singlet	Immune_cells
VAT_2M	1160.2	904	1.4929688	6	Singlet	Neurons
VAT_2M	1489.2	1030	1.1833207	7	Singlet	Endothelial_cells
VAT_2M	4737.6	2015	0.0274505	0	Singlet	Adipocyte
VAT_2M	1849.8	1282	1.3152536	6	Singlet	Neurons
VAT_2M	4917.3	2289	0.1622225	0	Singlet	Adipocyte
VAT_2M	5987.8	2576	0.0846684	0	Singlet	Adipocyte
VAT_2M	4530.4	2214	0.2051945	0	Singlet	Adipocyte
VAT_2M	1476.3	999	1.0373386	3	Singlet	FAPs
VAT_2M	7275.2	2839	0.002653	0	Singlet	Adipocyte
VAT_2M	1497.6	1070	0.6696639	2	Singlet	FAPs
VAT_2M	1379.1	1084	1.3695177	6	Singlet	Neurons
VAT_2M	3410.1	2160	0.5069808	2	Singlet	FAPs
VAT_2M	2704.5	1774	0.970704	2	Singlet	FAPs
VAT_2M	1703	1238	2.1165774	9	Singlet	Mesothelial
VAT_2M	1049	780	0.4938391	5	Singlet	Immune_cells
VAT_2M	7279.8	2892	0.0320823	0	Singlet	Adipocyte
VAT_2M	1882.9	1132	1.3315841	2	Singlet	FAPs
VAT_2M	8610.2	3234	0.3942761	0	Singlet	Adipocyte
VAT_2M	1917.2	1268	0.7521984	3	Singlet	FAPs
VAT_2M	13971	4174	0.0123233	0	Singlet	Adipocyte
VAT_2M	2047.7	1317	2.137817	5	Singlet	Immune_cells
VAT_2M	2345	1481	0.7361347	3	Singlet	FAPs
VAT_2M	5615.3	2647	0.0904641	0	Singlet	Adipocyte
VAT_2M	8425.5	3042	0.2372971	0	Singlet	Adipocyte
VAT_2M	1632.1	857	7.4508505	0	Singlet	Adipocyte
VAT_2M	1428.7	1039	1.3353695	3	Singlet	FAPs
VAT_2M	6763.6	2589	0.1990736	0	Singlet	Adipocyte
VAT_2M	3719.4	1854	0.1305609	0	Singlet	Adipocyte
VAT_2M	1573	1113	1.8769373	9	Singlet	Mesothelial
VAT_2M	5218.4	2452	0.0129295	0	Singlet	Adipocyte
VAT_2M	1408.5	826	5.0943274	5	Singlet	Immune_cells
VAT_2M	4740.3	2117	0.5001696	0	Singlet	Adipocyte
VAT_2M	2413.1	1417	0.6213838	0	Singlet	Adipocyte
VAT_2M	1206.1	874	2.6036253	2	Singlet	FAPs
VAT_2M	3259	1972	3.2892003	5	Singlet	Immune_cells
VAT_2M	10092	3450	0.0044677	0	Singlet	Adipocyte
VAT_2M	2707.3	1605	0.097995	3	Singlet	FAPs
VAT_2M	1865.8	1183	1.6662886	5	Singlet	Immune_cells
VAT_2M	1026.7	824	2.2352585	11	Singlet	Smooth_muscle_cells
VAT_2M	1711	1157	0.2709271	6	Singlet	Neurons
VAT_2M	4243.2	2016	0.2225515	0	Singlet	Adipocyte

VAT_2M	1490.8	1248	2.9913824	6	Singlet	Neurons
VAT_2M	1310.3	976	2.898769	1	Singlet	Immune_cells
VAT_2M	9106.2	3198	0.0649954	0	Singlet	Adipocyte
VAT_2M	1367.2	1002	2.7767396	3	Singlet	FAPs
VAT_2M	2230.1	1323	0.498323	2	Singlet	FAPs
VAT_2M	1873.2	1270	0.556459	2	Singlet	FAPs
VAT_2M	1723.4	1293	1.362425	2	Singlet	FAPs
VAT_2M	1569.3	1060	1.6016903	0	Singlet	Adipocyte
VAT_2M	1606.9	1046	0.0611038	7	Singlet	Endothelial_cells
VAT_2M	2988.3	1963	2.3763844	5	Singlet	Immune_cells
VAT_2M	1264.5	936	2.5541927	2	Singlet	FAPs
VAT_2M	6315.8	2715	0.0490412	0	Singlet	Adipocyte
VAT_2M	1436.8	1219	0.1646707	6	Singlet	Neurons
VAT_2M	1486.4	1175	0	7	Singlet	Endothelial_cells
VAT_2M	3265.8	1956	1.6475362	2	Singlet	FAPs
VAT_2M	1883	1346	0.2307288	3	Singlet	FAPs
VAT_2M	1389	1050	1.5290739	5	Singlet	Immune_cells
VAT_2M	5212.2	2481	1.5884892	0	Singlet	Adipocyte
VAT_2M	10413	3518	0	0	Singlet	Adipocyte
VAT_2M	3863.8	1947	0.1117123	0	Singlet	Adipocyte
VAT_2M	1055.9	869	0.2107422	5	Singlet	Immune_cells
VAT_2M	4085.1	2063	1.1152328	5	Singlet	Immune_cells
VAT_2M	1395.9	1005	2.9994024	4	Singlet	Immune_cells
VAT_2M	2050.2	1320	0.6784643	0	Singlet	Adipocyte
VAT_2M	3502.9	1772	0	0	Singlet	Adipocyte
VAT_2M	1298.1	948	0.3655361	7	Singlet	Endothelial_cells
VAT_2M	1260.4	878	1.3251414	2	Singlet	FAPs
VAT_2M	5861.5	2538	0.0257963	0	Singlet	Adipocyte
VAT_2M	1747.6	1139	0.3463966	2	Singlet	FAPs
VAT_2M	7188.8	2939	0.0310977	0	Singlet	Adipocyte
VAT_2M	1551.9	1022	2.0458447	5	Singlet	Immune_cells
VAT_2M	9280.9	3202	0.0962289	0	Singlet	Adipocyte
VAT_2M	1668.7	1125	0.3370348	0	Singlet	Adipocyte
VAT_2M	9602.9	3433	0.0501935	0	Singlet	Adipocyte
VAT_2M	9967.8	3314	0.0664588	0	Singlet	Adipocyte
VAT_2M	4513.1	2108	0.2297078	0	Singlet	Adipocyte
VAT_2M	1836.5	1370	0.1527631	5	Singlet	Immune_cells
VAT_2M	1951.3	1339	1.3206051	2	Singlet	FAPs
VAT_2M	1018	804	1.0984434	3	Singlet	FAPs
VAT_2M	4754.6	2376	0.1572649	0	Singlet	Adipocyte
VAT_2M	7417.6	2995	0.1073117	0	Singlet	Adipocyte
VAT_2M	2789	1514	0.3419444	0	Singlet	Adipocyte
VAT_2M	1581.5	975	7.6417456	7	Singlet	Endothelial_cells
VAT_2M	1235.3	875	2.1594627	5	Singlet	Immune_cells
VAT_2M	2686.7	1429	0.3715356	0	Singlet	Adipocyte
VAT_2M	1336.5	909	0.4923954	3	Singlet	FAPs
VAT_2M	2042.6	1444	0.2102729	5	Singlet	Immune_cells
VAT_2M	1141.5	962	0.399935	8	Singlet	Mesothelial
VAT_2M	3634.4	1949	0.0171256	0	Singlet	Adipocyte
VAT_2M	7195.5	2854	0	0	Singlet	Adipocyte
VAT_2M	1012.8	733	0.6047084	7	Singlet	Endothelial_cells

VAT_2M	1871.8	1240	1.3329139	2	Singlet	FAPs
VAT_2M	5527.8	2345	0	0	Singlet	Adipocyte
VAT_2M	8859.5	2983	0.0093472	0	Singlet	Adipocyte
VAT_2M	8894.7	3266	0.1171838	0	Singlet	Adipocyte
VAT_2M	1637.7	1186	0.960108	5	Singlet	Immune_cells
VAT_2M	2369.2	1474	0.2131025	0	Singlet	Adipocyte
VAT_2M	1172.2	906	3.6255566	8	Singlet	Mesothelial
VAT_2M	2752	1558	0.0595111	0	Singlet	Adipocyte
VAT_2M	6322.1	2565	0.1457781	0	Singlet	Adipocyte
VAT_2M	1252.5	1021	0.8006131	6	Singlet	Neurons
VAT_2M	2018	1249	0.7429536	0	Singlet	Adipocyte
VAT_2M	2084.4	1384	0.1437807	6	Singlet	Neurons
VAT_2M	1951.1	1262	1.8362551	1	Singlet	Immune_cells
VAT_2M	1829.2	1300	0.0953189	2	Singlet	FAPs
VAT_2M	3921.3	1973	0.0446562	0	Singlet	Adipocyte
VAT_2M	3140.8	1870	0.2238786	3	Singlet	FAPs
VAT_2M	1689.3	1173	0.1155652	0	Singlet	Adipocyte
VAT_2M	3046.4	1779	0.3848559	2	Singlet	FAPs
VAT_2M	2090.1	1626	0.4803238	6	Singlet	Neurons
VAT_2M	4198.9	2162	3.668121	5	Singlet	Immune_cells
VAT_2M	1887.6	1188	0.6406201	2	Singlet	FAPs
VAT_2M	1346.9	988	1.9417853	7	Singlet	Endothelial_cells
VAT_2M	7285.7	2825	0	0	Singlet	Adipocyte
VAT_2M	7347.7	3013	0.0229809	0	Singlet	Adipocyte
VAT_2M	2941.2	1421	13.360139	0	Singlet	Adipocyte
VAT_2M	1232	918	1.2644896	2	Singlet	FAPs
VAT_2M	1379.8	994	1.1696856	2	Singlet	FAPs
VAT_2M	6321	2562	0.0667144	0	Singlet	Adipocyte
VAT_2M	1349.6	996	0.5399314	3	Singlet	FAPs
VAT_2M	1644.1	1142	0.5520883	6	Singlet	Neurons
VAT_2M	3045.9	1690	0.4430607	0	Singlet	Adipocyte
VAT_2M	1493.9	1291	5.0376775	6	Singlet	Neurons
VAT_2M	1334.2	973	2.8084275	6	Singlet	Neurons
VAT_2M	1427.8	1063	1.0621531	3	Singlet	FAPs
VAT_2M	10887	3551	0.5752754	0	Singlet	Adipocyte
VAT_2M	1754.8	1081	0.9789988	0	Singlet	Adipocyte
VAT_2M	4387.5	2223	0.9395816	3	Singlet	FAPs
VAT_2M	6207.7	2548	0.0488301	0	Singlet	Adipocyte
VAT_2M	1206.3	967	0.4764524	11	Singlet	Smooth_muscle_cells
VAT_2M	1380.4	974	0.7542692	2	Singlet	FAPs
VAT_2M	4990.9	2356	0.1374598	0	Singlet	Adipocyte
VAT_2M	4722.1	2207	0.0480378	0	Singlet	Adipocyte
VAT_2M	1546.7	1358	0.2171331	6	Singlet	Neurons
VAT_2M	2438.4	1387	3.7175407	5	Singlet	Immune_cells
VAT_2M	3461.9	1795	0.1398028	0	Singlet	Adipocyte
VAT_2M	6063.1	2575	0.0098698	0	Singlet	Adipocyte
VAT_2M	2713.1	1463	1.2469173	0	Singlet	Adipocyte
VAT_2M	7040.4	2761	0	0	Singlet	Adipocyte
VAT_2M	2798.4	1742	0.4009581	7	Singlet	Endothelial_cells
VAT_2M	1711.9	1094	0.7752883	0	Singlet	Adipocyte
VAT_2M	3443.8	1697	0.0180058	0	Singlet	Adipocyte

VAT_2M	1819.1	1072	0.2144943	0	Singlet	Adipocyte
VAT_2M	2725.6	1676	0.6976532	3	Singlet	FAPs
VAT_2M	9112.7	3188	0.1258173	0	Singlet	Adipocyte
VAT_2M	11762	3883	0.4832644	0	Singlet	Adipocyte
VAT_2M	1499.5	1137	0.1339003	3	Singlet	FAPs
VAT_2M	1470.5	1004	4.1763954	3	Singlet	FAPs
VAT_2M	1182	876	1.6705463	3	Singlet	FAPs
VAT_2M	6573.9	2834	0.0429546	0	Singlet	Adipocyte
VAT_2M	5890.6	2506	0.0285517	0	Singlet	Adipocyte
VAT_2M	2055.1	998	9.1834463	7	Singlet	Endothelial_cells
VAT_2M	4441.6	2116	0.6129714	0	Singlet	Adipocyte
VAT_2M	1776.8	1272	0	11	Singlet	Smooth_muscle_cells
VAT_2M	1557.7	1184	1.9636057	9	Singlet	Mesothelial
VAT_2M	1302.2	987	1.3421865	2	Singlet	FAPs
VAT_2M	1721	1161	4.3407784	7	Singlet	Endothelial_cells
VAT_2M	5347.4	2399	0	0	Singlet	Adipocyte
VAT_2M	9961.4	3096	0.0545149	0	Singlet	Adipocyte
VAT_2M	5552.3	2552	0	0	Singlet	Adipocyte
VAT_2M	1325.2	1004	2.4931772	3	Singlet	FAPs
VAT_2M	1416.3	1009	0.0163226	5	Singlet	Immune_cells
VAT_2M	1642.3	1139	1.4855212	3	Singlet	FAPs
VAT_2M	2955.2	1807	0.6905718	0	Singlet	Adipocyte
VAT_2M	1041.7	827	0.6868205	2	Singlet	FAPs
VAT_2M	1074.7	802	0.8565811	5	Singlet	Immune_cells
VAT_2M	4214.2	2116	0.1034585	0	Singlet	Adipocyte
VAT_2M	1279	1037	0.5544895	6	Singlet	Neurons
VAT_2M	3066.4	1841	0.02118	0	Singlet	Adipocyte
VAT_2M	3702.6	1815	0.2127897	0	Singlet	Adipocyte
VAT_2M	5408.5	2172	0.6558628	0	Singlet	Adipocyte
VAT_2M	1421.7	1182	0.5071751	6	Singlet	Neurons
VAT_2M	4402.9	2216	0.0245961	0	Singlet	Adipocyte
VAT_2M	1212.7	818	0.397585	3	Singlet	FAPs
VAT_2M	1242	913	1.1613536	2	Singlet	FAPs
VAT_2M	18239	4658	0.0049454	0	Singlet	Adipocyte
VAT_2M	3543.7	2125	0.080949	2	Singlet	FAPs
VAT_2M	3039.2	1652	1.0890685	5	Singlet	Immune_cells
VAT_2M	1577.3	995	3.2188242	9	Singlet	Mesothelial
VAT_2M	2248.8	1378	0.1810239	3	Singlet	FAPs
VAT_2M	1275.5	1080	0.7114855	6	Singlet	Neurons
VAT_2M	8629.3	3187	0.0261717	0	Singlet	Adipocyte
VAT_2M	6417.9	2791	0.0286305	0	Singlet	Adipocyte
VAT_2M	1326.4	1070	0.7645252	6	Singlet	Neurons
VAT_2M	2319.1	1589	0.2966559	11	Singlet	Smooth_muscle_cells
VAT_2M	1000.4	829	0.7146684	9	Singlet	Mesothelial
VAT_2M	1074.6	780	0.8413054	1	Singlet	Immune_cells
VAT_2M	1918.6	1327	2.2165941	5	Singlet	Immune_cells
VAT_2M	2064.3	1413	0.1563826	5	Singlet	Immune_cells
VAT_2M	2007.9	1402	4.1012011	5	Singlet	Immune_cells
VAT_2M	7841	2976	0.0024278	0	Singlet	Adipocyte
VAT_2M	2815.2	1849	0.2587554	5	Singlet	Immune_cells
VAT_2M	1461.5	1162	0.444813	2	Singlet	FAPs

VAT_2M	1105.4	718	11.644833	0	Singlet	Adipocyte
VAT_2M	4635.6	2331	0.0115864	0	Singlet	Adipocyte
VAT_2M	10731	3695	0.1402772	0	Singlet	Adipocyte
VAT_2M	2667.1	1512	0.3092121	0	Singlet	Adipocyte
VAT_2M	2088.9	1277	0.293666	0	Singlet	Adipocyte
VAT_2M	3641.6	1715	0.0450359	0	Singlet	Adipocyte
VAT_2M	9249.4	3189	0	0	Singlet	Adipocyte
VAT_2M	1131.4	867	0.6220043	3	Singlet	FAPs
VAT_2M	1799.7	1355	0.0459101	3	Singlet	FAPs
VAT_2M	1753.5	1175	0.3484047	0	Singlet	Adipocyte
VAT_2M	6459.1	2633	2.3368283	0	Singlet	Adipocyte
VAT_2M	2632.5	1485	0.1251291	0	Singlet	Adipocyte
VAT_2M	4391.8	2017	2.0338189	0	Singlet	Adipocyte
VAT_2M	11822	3651	0.186322	0	Singlet	Adipocyte
VAT_2M	2719.8	1734	0.4789856	3	Singlet	FAPs
VAT_2M	4917.8	2323	0.1279144	0	Singlet	Adipocyte
VAT_2M	7098.3	2880	0.4692892	0	Singlet	Adipocyte
VAT_2M	2061.1	1167	2.4368282	0	Singlet	Adipocyte
VAT_2M	6951.5	2778	0.0669156	0	Singlet	Adipocyte
VAT_2M	1110.5	855	3.1820119	6	Singlet	Neurons
VAT_2M	1260.9	967	0.9377648	6	Singlet	Neurons
VAT_2M	1479.2	1089	0.1490557	2	Singlet	FAPs
VAT_2M	1994	1198	0.0411136	0	Singlet	Adipocyte
VAT_2M	1466.3	1137	2.0517861	4	Singlet	Immune_cells
VAT_2M	6887.6	2708	0.0255635	0	Singlet	Adipocyte
VAT_2M	1142.1	803	2.8465231	5	Singlet	Immune_cells
VAT_2M	1393.7	926	1.51626	2	Singlet	FAPs
VAT_2M	2397.6	1426	0.3329919	0	Singlet	Adipocyte
VAT_2M	3354.7	1686	2.8762474	4	Singlet	Immune_cells
VAT_2M	2218.6	1367	0.2760189	0	Singlet	Adipocyte
VAT_2M	1173.3	857	0.253069	7	Singlet	Endothelial_cells
VAT_2M	1655.1	1107	0.3786128	0	Singlet	Adipocyte
VAT_2M	1158.1	934	0.6012052	8	Singlet	Mesothelial
VAT_2M	6388.8	2711	0.0202671	0	Singlet	Adipocyte
VAT_2M	1273.1	961	0.5732921	5	Singlet	Immune_cells
VAT_2M	1792.3	1160	1.5045757	0	Singlet	Adipocyte
VAT_2M	6703.1	2672	0.0596663	0	Singlet	Adipocyte
VAT_2M	1037.7	820	3.8986909	7	Singlet	Endothelial_cells
VAT_2M	9976.3	3233	0.0391406	0	Singlet	Adipocyte
VAT_2M	2580.9	1636	1.5324273	5	Singlet	Immune_cells
VAT_2M	3542.5	1827	0.0905685	0	Singlet	Adipocyte
VAT_2M	7675.6	2972	0.0213278	0	Singlet	Adipocyte
VAT_2M	6216.5	2567	0.0642968	0	Singlet	Adipocyte
VAT_2M	8001.1	3019	0.0314151	0	Singlet	Adipocyte
VAT_2M	1919	1321	0.4165491	7	Singlet	Endothelial_cells
VAT_2M	7779	2919	0.0158617	0	Singlet	Adipocyte
VAT_2M	1998.3	1570	1.2268036	5	Singlet	Immune_cells
VAT_2M	1588.4	1108	2.2393838	3	Singlet	FAPs
VAT_2M	1731.1	1156	0.054211	5	Singlet	Immune_cells
VAT_2M	1651.2	1177	0.0538125	5	Singlet	Immune_cells
VAT_2M	2324.7	1431	0.6948316	0	Singlet	Adipocyte

VAT_2M	9207.3	3304	0.0255329	0	Singlet	Adipocyte
VAT_2M	1924.6	1248	3.301962	5	Singlet	Immune_cells
VAT_2M	9088.4	3215	0.0383713	0	Singlet	Adipocyte
VAT_2M	1575.4	1257	1.2068091	6	Singlet	Neurons
VAT_2M	1214.6	827	4.3883025	3	Singlet	FAPs
VAT_2M	2045.3	1227	2.0178127	0	Singlet	Adipocyte
VAT_2M	1748.6	1123	0.8773221	6	Singlet	Neurons
VAT_2M	2892.1	1524	0.0844222	0	Singlet	Adipocyte
VAT_2M	1669.8	1203	0.5049448	2	Singlet	FAPs
VAT_2M	4667.4	2465	0.5785891	2	Singlet	FAPs
VAT_2M	7520.6	3052	0.0564855	0	Singlet	Adipocyte
VAT_2M	2029	1537	0.7294031	3	Singlet	FAPs
VAT_2M	4375.9	2038	0.2347467	0	Singlet	Adipocyte
VAT_2M	3837	1983	0.2462765	0	Singlet	Adipocyte
VAT_2M	3936.2	2180	1.4809225	3	Singlet	FAPs
VAT_2M	7703.8	2939	0.0271591	0	Singlet	Adipocyte
VAT_2M	1678.5	1231	1.0901069	5	Singlet	Immune_cells
VAT_2M	5150.7	2367	0.0350105	0	Singlet	Adipocyte
VAT_2M	2012.9	1297	1.9651897	2	Singlet	FAPs
VAT_2M	7671.7	2855	0.1887247	0	Singlet	Adipocyte
VAT_2M	1925.9	1151	3.5223477	5	Singlet	Immune_cells
VAT_2M	6083.3	2648	0.017748	0	Singlet	Adipocyte
VAT_2M	2849.8	1878	2.2857952	3	Singlet	FAPs
VAT_2M	2251.4	1632	1.2152844	3	Singlet	FAPs
VAT_2M	1298.6	965	0.2160937	0	Singlet	Adipocyte
VAT_2M	3561.4	1821	0.1590087	0	Singlet	Adipocyte
VAT_2M	1080	932	1.9466896	9	Singlet	Mesothelial
VAT_2M	9089.3	3150	0.0246302	0	Singlet	Adipocyte
VAT_2M	2138.1	1263	3.0386645	5	Singlet	Immune_cells
VAT_2M	1607.6	1142	3.0618588	2	Singlet	FAPs
VAT_2M	1722.9	1236	0.210426	3	Singlet	FAPs
VAT_2M	5044.5	2280	0.1788753	0	Singlet	Adipocyte
VAT_2M	3849.6	1952	0.5814899	0	Singlet	Adipocyte
VAT_2M	1728.7	1100	0.9321286	0	Singlet	Adipocyte
VAT_2M	6732.2	2717	0	0	Singlet	Adipocyte
VAT_2M	1889.9	1149	1.17213	0	Singlet	Adipocyte
VAT_2M	1630.4	1146	0.4409405	7	Singlet	Endothelial_cells
VAT_2M	1309.8	989	0.9244239	4	Singlet	Immune_cells
VAT_2M	1235.7	964	0.0487303	2	Singlet	FAPs
VAT_2M	3832.1	2113	0.2049518	0	Singlet	Adipocyte
VAT_2M	7695.4	2900	0.0307108	0	Singlet	Adipocyte
VAT_2M	7959.4	2859	0.0146359	0	Singlet	Adipocyte
VAT_2M	1711.4	1169	0.2776676	0	Singlet	Adipocyte
VAT_2M	1090.8	774	0.6389738	7	Singlet	Endothelial_cells
VAT_2M	1233	881	3.3040418	5	Singlet	Immune_cells
VAT_2M	1008.8	807	1.5995976	6	Singlet	Neurons
VAT_2M	3893.8	1890	0.1389278	0	Singlet	Adipocyte
VAT_2M	1477.9	1150	2.6668716	3	Singlet	FAPs
VAT_2M	5817.1	2620	0.0750031	0	Singlet	Adipocyte
VAT_2M	1282.8	973	0.2156091	7	Singlet	Endothelial_cells
VAT_2M	1293.8	908	2.4176309	2	Singlet	FAPs

VAT_2M	1903	1192	0.2984951	0	Singlet	Adipocyte
VAT_2M	3482.3	1890	0.4584437	0	Singlet	Adipocyte
VAT_2M	4026.8	1873	0.0275766	0	Singlet	Adipocyte
VAT_2M	1185.7	842	0.356744	0	Singlet	Adipocyte
VAT_2M	10478	3438	0	0	Singlet	Adipocyte
VAT_2M	1800.3	1222	3.9787065	5	Singlet	Immune_cells
VAT_2M	6373.9	2529	0.1254686	0	Singlet	Adipocyte
VAT_2M	1693.5	1226	1.9019408	2	Singlet	FAPs
VAT_2M	3442.4	1746	0.1122146	0	Singlet	Adipocyte
VAT_2M	1170.6	813	0	5	Singlet	Immune_cells
VAT_2M	1587.8	1125	0.4959065	3	Singlet	FAPs
VAT_2M	1405.9	1007	0.7361997	2	Singlet	FAPs
VAT_2M	1156	979	0.8690194	6	Singlet	Neurons
VAT_2M	1482.1	1137	0.257602	2	Singlet	FAPs
VAT_2M	1380.6	1063	0.6010888	2	Singlet	FAPs
VAT_2M	6233.9	2682	1.8201196	3	Singlet	FAPs
VAT_2M	7036	2703	0.0018884	0	Singlet	Adipocyte
VAT_2M	1181.6	1013	2.1759686	6	Singlet	Neurons
VAT_2M	6387.3	2815	0.2784964	0	Singlet	Adipocyte
VAT_2M	3617.9	2046	0.2190153	0	Singlet	Adipocyte
VAT_2M	1233.1	854	0.1989699	7	Singlet	Endothelial_cells
VAT_2M	4466.9	2120	0.1813534	0	Singlet	Adipocyte
VAT_2M	1422.4	998	8.2488681	5	Singlet	Immune_cells
VAT_2M	3076.6	1822	1.2367356	2	Singlet	FAPs
VAT_2M	1108.8	910	1.5152796	6	Singlet	Neurons
VAT_2M	7115.2	3019	0.1708782	0	Singlet	Adipocyte
VAT_2M	2163.7	1400	0.1201142	0	Singlet	Adipocyte
VAT_2M	2394.1	1471	0.7803438	2	Singlet	FAPs
VAT_2M	1169.4	980	1.261951	7	Singlet	Endothelial_cells
VAT_2M	2824.7	1643	0.5489775	0	Singlet	Adipocyte
VAT_2M	1464	1072	0.0557775	3	Singlet	FAPs
VAT_2M	4647.4	2342	0.2350281	0	Singlet	Adipocyte
VAT_2M	2459.4	1310	0.0930792	0	Singlet	Adipocyte
VAT_2M	9628.4	3285	7.99E-05	0	Singlet	Adipocyte
VAT_2M	2715.2	1605	0.5132576	0	Singlet	Adipocyte
VAT_2M	2629.4	1661	0.3690735	0	Singlet	Adipocyte
VAT_2M	3263.7	1611	0.1794151	0	Singlet	Adipocyte
VAT_2M	8016.2	2957	0.0260348	0	Singlet	Adipocyte
VAT_2M	6218.8	2608	0.0115959	0	Singlet	Adipocyte
VAT_2M	2658	1596	0.4526793	3	Singlet	FAPs
VAT_2M	5717.7	2374	0.0198719	0	Singlet	Adipocyte
VAT_2M	1916.2	1372	1.1453054	3	Singlet	FAPs
VAT_2M	1649.3	1120	3.8434839	5	Singlet	Immune_cells
VAT_2M	1280.5	991	0.1662866	2	Singlet	FAPs
VAT_2M	4311.5	2114	0.1407498	0	Singlet	Adipocyte
VAT_2M	2215.6	1411	0.1685285	0	Singlet	Adipocyte
VAT_2M	6757.9	2745	0.0929407	0	Singlet	Adipocyte
VAT_2M	1524.9	897	2.9571433	7	Singlet	Endothelial_cells
VAT_2M	1212.4	982	1.1670074	7	Singlet	Endothelial_cells
VAT_2M	4661.1	2134	0.3154257	0	Singlet	Adipocyte
VAT_2M	1702	1061	1.4498407	0	Singlet	Adipocyte

VAT_2M	2769.5	1553	0.518375	0	Singlet	Adipocyte
VAT_2M	4631.8	2160	0.2037967	0	Singlet	Adipocyte
VAT_2M	1937.1	1279	1.7581977	6	Singlet	Neurons
VAT_2M	1109.8	821	0.3599154	2	Singlet	FAPs
VAT_2M	4185.1	1942	0.0894829	0	Singlet	Adipocyte
VAT_2M	2254.7	1372	3.8539463	9	Singlet	Mesothelial
VAT_2M	1523.7	1097	1.0318743	2	Singlet	FAPs
VAT_2M	5734.5	2474	1.0000558	0	Singlet	Adipocyte
VAT_2M	3836.3	2183	4.7423694	2	Singlet	FAPs
VAT_2M	1732.8	1214	0.6200194	2	Singlet	FAPs
VAT_2M	1366.8	1001	0.6821062	2	Singlet	FAPs
VAT_2M	5160	2503	0.0630119	0	Singlet	Adipocyte
VAT_2M	1895.3	1389	0.3766064	2	Singlet	FAPs
VAT_2M	2086.5	1339	0.8862916	5	Singlet	Immune_cells
VAT_2M	1788.5	1319	1.8408927	2	Singlet	FAPs
VAT_2M	1138.8	839	1.7798027	2	Singlet	FAPs
VAT_2M	3641.7	1796	0.0630922	0	Singlet	Adipocyte
VAT_2M	2943.8	1700	0.6467051	0	Singlet	Adipocyte
VAT_2M	2235.5	1260	0.2552858	0	Singlet	Adipocyte
VAT_2M	2244.1	1322	0.6074549	7	Singlet	Endothelial_cells
VAT_2M	2086.8	1504	0.8982175	5	Singlet	Immune_cells
VAT_2M	1606.1	1113	1.2246778	2	Singlet	FAPs
VAT_2M	1376.8	1021	0.9476311	2	Singlet	FAPs
VAT_2M	5369.9	2296	0.0152736	0	Singlet	Adipocyte
VAT_2M	1087.6	800	1.0755237	3	Singlet	FAPs
VAT_2M	1644	1185	0.723156	6	Singlet	Neurons
VAT_2M	3224.8	1692	0.1960553	0	Singlet	Adipocyte
VAT_2M	2666.2	1942	1.3256033	2	Singlet	FAPs
VAT_2M	7618.6	2863	0.0715767	0	Singlet	Adipocyte
VAT_2M	1891.3	1118	0	0	Singlet	Adipocyte
VAT_2M	2271.9	1319	0.8318293	0	Singlet	Adipocyte
VAT_2M	2221.2	1256	1.3196495	3	Singlet	FAPs
VAT_2M	5576.1	2467	0.2111922	0	Singlet	Adipocyte
VAT_2M	3262.9	1850	0.0021906	0	Singlet	Adipocyte
VAT_2M	3677	1942	0.1633742	0	Singlet	Adipocyte
VAT_2M	8607.8	3251	0	0	Singlet	Adipocyte
VAT_2M	1213.4	881	1.6437993	4	Singlet	Immune_cells
VAT_2M	5427.1	2545	0.0555192	0	Singlet	Adipocyte
VAT_2M	1257	1002	5.3779365	1	Singlet	Immune_cells
VAT_2M	1124.4	862	1.8680052	6	Singlet	Neurons
VAT_2M	2574	1401	0.1053332	0	Singlet	Adipocyte
VAT_2M	6452.6	2653	0.0045027	0	Singlet	Adipocyte
VAT_2M	1419.8	1189	0.0513627	6	Singlet	Neurons
VAT_2M	1836.3	1380	2.8103614	3	Singlet	FAPs
VAT_2M	1275.8	1096	0.8471267	8	Singlet	Mesothelial
VAT_2M	1029	854	0.8589474	6	Singlet	Neurons
VAT_2M	5090.9	2418	0.0444121	0	Singlet	Adipocyte
VAT_2M	2550.8	1492	0.1947305	0	Singlet	Adipocyte
VAT_2M	3096.6	1790	0.0229469	0	Singlet	Adipocyte
VAT_2M	2067.5	1358	1.3242428	6	Singlet	Neurons
VAT_2M	2528	1605	0.0570668	0	Singlet	Adipocyte

VAT_2M	1275.3	946	0.244479	0	Singlet	Adipocyte
VAT_2M	3073.9	1668	0.1081369	0	Singlet	Adipocyte
VAT_2M	2544.3	1497	0.0567486	0	Singlet	Adipocyte
VAT_2M	2690.3	1602	0.0283441	0	Singlet	Adipocyte
VAT_2M	1693.3	1339	3.7746678	11	Singlet	Smooth_muscle_cells
VAT_2M	1139.4	842	0.4575122	6	Singlet	Neurons
VAT_2M	2384.1	1749	1.7002951	2	Singlet	FAPs
VAT_2M	6137.5	2673	0	0	Singlet	Adipocyte
VAT_2M	4518.6	2077	0.1812164	0	Singlet	Adipocyte
VAT_2M	2305.5	1384	0.515068	0	Singlet	Adipocyte
VAT_2M	4587.2	2158	1.9654292	0	Singlet	Adipocyte
VAT_2M	6024.4	2486	0.1103832	0	Singlet	Adipocyte
VAT_2M	3148.9	1705	0.084103	0	Singlet	Adipocyte
VAT_2M	10941	3516	0	0	Singlet	Adipocyte
VAT_2M	3073.4	1613	0.0454187	0	Singlet	Adipocyte
VAT_2M	2691.6	1493	0.0615456	0	Singlet	Adipocyte
VAT_2M	5516.5	2396	0.5297695	0	Singlet	Adipocyte
VAT_2M	1477.3	1184	2.1438096	11	Singlet	Smooth_muscle_cells
VAT_2M	5013.3	2267	0.0086957	0	Singlet	Adipocyte
VAT_2M	1023.3	868	3.0722754	11	Singlet	Smooth_muscle_cells
VAT_2M	9680.8	3295	0	0	Singlet	Adipocyte
VAT_2M	2566.6	1676	0.2638147	2	Singlet	FAPs
VAT_2M	5308.5	2510	0.0152467	0	Singlet	Adipocyte
VAT_2M	1675.3	1176	5.325136	2	Singlet	FAPs
VAT_2M	2925	1537	0	0	Singlet	Adipocyte
VAT_2M	9132.5	3210	0.0195251	0	Singlet	Adipocyte
VAT_2M	1590.6	1148	1.970104	3	Singlet	FAPs
VAT_2M	5422.1	2217	0.0295165	0	Singlet	Adipocyte
VAT_2M	3591.4	1846	0.0748262	0	Singlet	Adipocyte
VAT_2M	6804.1	2929	0.1715718	0	Singlet	Adipocyte
VAT_2M	6644.4	2888	0.1575329	0	Singlet	Adipocyte
VAT_2M	3540.1	1833	0.124931	0	Singlet	Adipocyte
VAT_2M	5900	2492	0.9253238	0	Singlet	Adipocyte
VAT_2M	6049.9	2617	0.0375478	0	Singlet	Adipocyte
VAT_2M	2069.9	1365	0.2940976	5	Singlet	Immune_cells
VAT_2M	2204	1406	2.3136726	2	Singlet	FAPs
VAT_2M	2299	1510	1.0906467	6	Singlet	Neurons
VAT_2M	1215.9	855	1.3722412	3	Singlet	FAPs
VAT_2M	1262.8	994	1.677708	6	Singlet	Neurons
VAT_2M	1797.8	1313	3.5152593	5	Singlet	Immune_cells
VAT_2M	6643.9	2634	0	0	Singlet	Adipocyte
VAT_2M	5666.6	2429	0.0801576	0	Singlet	Adipocyte
VAT_2M	1110.5	864	1.0750223	8	Singlet	Mesothelial
VAT_2M	2110.2	1348	0.5487732	0	Singlet	Adipocyte
VAT_2M	3618.1	1892	0.0785564	0	Singlet	Adipocyte
VAT_2M	14983	4057	0.0030049	0	Singlet	Adipocyte
VAT_2M	1856.8	1137	1.0532665	6	Singlet	Neurons
VAT_2M	1907.9	1278	2.719121	2	Singlet	FAPs
VAT_2M	1284	865	0.2077809	6	Singlet	Neurons
VAT_2M	2609.8	1499	5.6168314	7	Singlet	Endothelial_cells
VAT_2M	2497.8	1312	1.7839346	7	Singlet	Endothelial_cells

VAT_2M	2298.8	1451	0.3270764	3	Singlet	FAPs
VAT_2M	1676.5	1212	6.0926264	2	Singlet	FAPs
VAT_2M	1168.1	831	7.470353	1	Singlet	Immune_cells
VAT_2M	1576.2	1097	6.1191857	5	Singlet	Immune_cells
VAT_2M	3789.9	1951	0.5116803	0	Singlet	Adipocyte
VAT_2M	6321.3	2727	0.0224802	0	Singlet	Adipocyte
VAT_2M	1613.4	1399	1.4416072	6	Singlet	Neurons
VAT_2M	1375.6	1002	1.7598757	5	Singlet	Immune_cells
VAT_2M	1557.4	1045	3.5297866	9	Singlet	Mesothelial
VAT_2M	1289.5	926	0.9199164	2	Singlet	FAPs
VAT_2M	2100	1293	0.0166719	0	Singlet	Adipocyte
VAT_2M	1020.2	754	0.888268	7	Singlet	Endothelial_cells
VAT_2M	3541	1756	0.1120013	0	Singlet	Adipocyte
VAT_2M	3753.8	2023	0.4966941	0	Singlet	Adipocyte
VAT_2M	1254.2	895	0.4668177	3	Singlet	FAPs
VAT_2M	1515.5	804	12.863199	0	Singlet	Adipocyte
VAT_2M	11350	3531	0.0434896	0	Singlet	Adipocyte
VAT_2M	6091.9	2529	0.0106982	0	Singlet	Adipocyte
VAT_2M	2647.5	1604	0.644693	0	Singlet	Adipocyte
VAT_2M	7086.6	2882	0.0035322	0	Singlet	Adipocyte
VAT_2M	7810.6	2945	0.0053021	0	Singlet	Adipocyte
VAT_2M	1660.6	1005	0.5479413	0	Singlet	Adipocyte
VAT_2M	1983.1	1399	1.2496828	2	Singlet	FAPs
VAT_2M	5457.5	2405	0.0559851	0	Singlet	Adipocyte
VAT_2M	1846.5	1138	3.5212913	0	Singlet	Adipocyte
VAT_2M	3460.5	1799	0.2194528	0	Singlet	Adipocyte
VAT_2M	1703.2	1104	0.2775626	7	Singlet	Endothelial_cells
VAT_2M	1323.5	995	0.5433166	5	Singlet	Immune_cells
VAT_2M	1296.5	918	5.1770276	11	Singlet	Smooth_muscle_cells
VAT_2M	5427.6	2474	0.0168169	0	Singlet	Adipocyte
VAT_2M	2445.5	1572	1.4625166	2	Singlet	FAPs
VAT_2M	1666.6	1195	0.7812163	8	Singlet	Mesothelial
VAT_2M	4559.1	2265	0.0322839	0	Singlet	Adipocyte
VAT_2M	1531.1	1109	3.884326	9	Singlet	Mesothelial
VAT_2M	2342.2	1450	0.6815457	6	Singlet	Neurons
VAT_2M	1364.3	904	0.3043528	11	Singlet	Smooth_muscle_cells
VAT_2M	8179.9	2937	0.0646521	0	Singlet	Adipocyte
VAT_2M	1450.5	962	0.6214981	3	Singlet	FAPs
VAT_2M	5204.1	2431	0.3020289	0	Singlet	Adipocyte
VAT_2M	7313.4	3032	0.1074247	0	Singlet	Adipocyte
VAT_2M	2290	1433	1.4228264	7	Singlet	Endothelial_cells
VAT_2M	3098.2	1746	0.1013585	0	Singlet	Adipocyte
VAT_2M	1949.9	1215	0.2760507	0	Singlet	Adipocyte
VAT_2M	1430.5	1037	0.1809735	5	Singlet	Immune_cells
VAT_2M	3821	1826	0.1404233	0	Singlet	Adipocyte
VAT_2M	1108.6	822	2.1347519	2	Singlet	FAPs
VAT_2M	3240.2	1737	0.1185441	0	Singlet	Adipocyte
VAT_2M	2179.2	1309	0.6973795	0	Singlet	Adipocyte
VAT_2M	11106	3716	0.8423415	0	Singlet	Adipocyte
VAT_2M	2334.6	1363	0.0963117	0	Singlet	Adipocyte
VAT_2M	1892.8	1253	0.5844128	6	Singlet	Neurons

VAT_2M	3730.9	1964	0.0264158	0	Singlet	Adipocyte
VAT_2M	7900.6	3032	1.0857361	0	Singlet	Adipocyte
VAT_2M	1940.9	1215	1.957457	0	Singlet	Adipocyte
VAT_2M	1262.2	896	3.9903396	9	Singlet	Mesothelial
VAT_2M	4759.8	2268	0.0633231	0	Singlet	Adipocyte
VAT_2M	1125.1	853	0.859943	3	Singlet	FAPs
VAT_2M	2144.2	1285	1.1016762	2	Singlet	FAPs
VAT_2M	9044.7	3318	0.0440682	0	Singlet	Adipocyte
VAT_2M	5232.6	2256	0.3450914	0	Singlet	Adipocyte
VAT_2M	1664.5	1377	0.5920046	5	Singlet	Immune_cells
VAT_2M	3729.6	1949	1.6399656	0	Singlet	Adipocyte
VAT_2M	2201.6	1350	2.6056143	5	Singlet	Immune_cells
VAT_2M	1003.9	804	0.8219871	3	Singlet	FAPs
VAT_2M	1815.4	1146	1.3330912	3	Singlet	FAPs
VAT_2M	1213.8	1041	0.3904732	9	Singlet	Mesothelial
VAT_2M	3632.5	1851	0.0341755	0	Singlet	Adipocyte
VAT_2M	1338.8	992	0.2219422	6	Singlet	Neurons
VAT_2M	2100.5	1295	0.0789874	0	Singlet	Adipocyte
VAT_2M	1132.6	896	1.5669641	2	Singlet	FAPs
VAT_2M	4409.7	2077	0.012912	0	Singlet	Adipocyte
VAT_2M	4443.3	1940	0.0111172	0	Singlet	Adipocyte
VAT_2M	9377.9	3183	0.0110352	0	Singlet	Adipocyte
VAT_2M	2928.6	1783	0.1036532	6	Singlet	Neurons
VAT_2M	1923.4	1267	0.0392649	3	Singlet	FAPs
VAT_2M	1320.2	1057	0.4751654	3	Singlet	FAPs
VAT_2M	2237.4	1453	0.5232112	0	Singlet	Adipocyte
VAT_2M	1060.9	942	1.2500067	11	Singlet	Smooth_muscle_cells
VAT_2M	7031.1	2829	0.021281	0	Singlet	Adipocyte
VAT_2M	6533.2	2551	0.4404835	0	Singlet	Adipocyte
VAT_2M	1231.1	857	1.5965275	3	Singlet	FAPs
VAT_2M	1215.9	878	0.1632822	5	Singlet	Immune_cells
VAT_2M	1603	1111	2.3330663	3	Singlet	FAPs
VAT_2M	6413.8	2547	0.0815165	0	Singlet	Adipocyte
VAT_2M	1036.7	804	0.5360974	8	Singlet	Mesothelial
VAT_2M	7749.7	3157	0.0079861	0	Singlet	Adipocyte
VAT_2M	1726.7	1217	0.3461659	2	Singlet	FAPs
VAT_2M	2352.1	1605	0.1944126	3	Singlet	FAPs
VAT_2M	2240.9	1450	0.9582205	2	Singlet	FAPs
VAT_2M	5135.1	2370	0.0695788	0	Singlet	Adipocyte
VAT_2M	3650.3	2115	0.2326247	2	Singlet	FAPs
VAT_2M	1920	1281	0.0245136	11	Singlet	Smooth_muscle_cells
VAT_2M	1785.1	1160	0.2681253	0	Singlet	Adipocyte
VAT_2M	1530.4	1200	0	9	Singlet	Mesothelial
VAT_2M	8415.9	3105	0.0374351	0	Singlet	Adipocyte
VAT_2M	5982.2	2387	0	0	Singlet	Adipocyte
VAT_2M	10562	3472	0.1345486	0	Singlet	Adipocyte
VAT_2M	7628.7	2864	0.0241678	0	Singlet	Adipocyte
VAT_2M	1235.3	997	0.8315532	6	Singlet	Neurons
VAT_2M	4319.1	2120	0.0492864	0	Singlet	Adipocyte
VAT_2M	2721	1631	0.2323294	0	Singlet	Adipocyte
VAT_2M	1208.7	917	0.3519134	10	Singlet	Immune_cells

VAT_2M	2233.6	1399	0.8314953	3	Singlet	FAPs
VAT_2M	1318.7	903	0.0227509	2	Singlet	FAPs
VAT_2M	1920.5	1424	0.4883009	5	Singlet	Immune_cells
VAT_2M	7538.6	2945	0.0487215	0	Singlet	Adipocyte
VAT_2M	9742.4	3134	0.0023069	0	Singlet	Adipocyte
VAT_2M	5670.3	2332	0.0237558	0	Singlet	Adipocyte
VAT_2M	1978.1	1231	0.7167894	0	Singlet	Adipocyte
VAT_2M	5682.9	2415	0.0214147	0	Singlet	Adipocyte
VAT_2M	1328	899	1.5947902	2	Singlet	FAPs
VAT_2M	2587.7	1505	0.025177	0	Singlet	Adipocyte
VAT_2M	7443.3	2962	0.2651465	0	Singlet	Adipocyte
VAT_2M	5408.6	2456	0.2493156	0	Singlet	Adipocyte
VAT_2M	1212.6	948	3.2619146	2	Singlet	FAPs
VAT_2M	3057.8	1783	0.2609941	0	Singlet	Adipocyte
VAT_2M	7230.4	2820	0.907077	0	Singlet	Adipocyte
VAT_2M	1839.7	1151	0.5662315	2	Singlet	FAPs
VAT_2M	4972.6	2233	0.0123016	0	Singlet	Adipocyte
VAT_2M	1003.2	847	0.4954255	8	Singlet	Mesothelial
VAT_2M	1705.3	1035	3.5287555	1	Singlet	Immune_cells
VAT_2M	1195.8	817	0.2466896	7	Singlet	Endothelial_cells
VAT_2M	2724.4	1721	0.7845347	2	Singlet	FAPs
VAT_2M	1147.3	872	0.6997865	1	Singlet	Immune_cells
VAT_2M	1637	1147	1.8398914	7	Singlet	Endothelial_cells
VAT_2M	3084	1862	0.3717884	7	Singlet	Endothelial_cells
VAT_2M	1812.3	1180	2.626906	6	Singlet	Neurons
VAT_2M	3676.5	1940	0	0	Singlet	Adipocyte
VAT_2M	2762	1536	0.4682759	3	Singlet	FAPs
VAT_2M	2754	1632	0.5327744	2	Singlet	FAPs
VAT_2M	5831.3	2494	0.092274	0	Singlet	Adipocyte
VAT_2M	2625	1582	0.6401087	3	Singlet	FAPs
VAT_2M	1130.4	922	0.6169139	6	Singlet	Neurons
VAT_2M	1867.4	1481	0.3479456	2	Singlet	FAPs
VAT_2M	2350.2	1494	1.4333387	0	Singlet	Adipocyte
VAT_2M	1275.9	829	0.5229909	7	Singlet	Endothelial_cells
VAT_2M	1997.3	1124	0.0224381	0	Singlet	Adipocyte
VAT_2M	1022	863	0.8950888	6	Singlet	Neurons
VAT_2M	11452	3814	0.1541491	0	Singlet	Adipocyte
VAT_2M	10408	3396	0.0742218	0	Singlet	Adipocyte
VAT_2M	1352.6	957	0.9983864	5	Singlet	Immune_cells
VAT_2M	1807	1358	2.3413571	3	Singlet	FAPs
VAT_2M	1877.4	1323	0.2563028	5	Singlet	Immune_cells
VAT_2M	1346	926	0.2787869	5	Singlet	Immune_cells
VAT_2M	7549.4	3015	0.7888861	0	Singlet	Adipocyte
VAT_2M	1125.7	837	0.8852663	3	Singlet	FAPs
VAT_2M	1141.6	851	0.6824294	7	Singlet	Endothelial_cells
VAT_2M	6256	2562	0.0823763	0	Singlet	Adipocyte
VAT_2M	1126.6	920	2.7383557	10	Singlet	Immune_cells
VAT_2M	1188	958	0.9130636	11	Singlet	Smooth_muscle_cells
VAT_2M	4322.3	2284	0.3942121	0	Singlet	Adipocyte
VAT_2M	1060.4	816	6.9270128	4	Singlet	Immune_cells
VAT_2M	1686.1	1121	0.8943293	7	Singlet	Endothelial_cells

VAT_2M	2525.4	1446	0.1867986	0	Singlet	Adipocyte
VAT_2M	2279.7	1583	0.8011375	3	Singlet	FAPs
VAT_2M	1315.3	906	0.8088065	5	Singlet	Immune_cells
VAT_2M	4780.8	2283	0.0716317	0	Singlet	Adipocyte
VAT_2M	1102.9	816	1.6969955	3	Singlet	FAPs
VAT_2M	5425.4	2262	0.0189488	0	Singlet	Adipocyte
VAT_2M	1906.9	1328	0.8422447	2	Singlet	FAPs
VAT_2M	3267.1	1823	0.9652987	3	Singlet	FAPs
VAT_2M	1244.7	1032	0.8489572	6	Singlet	Neurons
VAT_2M	6600.7	2596	0.0305905	0	Singlet	Adipocyte
VAT_2M	4146.2	2012	0.0133006	0	Singlet	Adipocyte
VAT_2M	4446.9	2253	0.0603219	0	Singlet	Adipocyte
VAT_2M	2385.1	1509	1.8312464	3	Singlet	FAPs
VAT_2M	6686.8	2711	0.181328	0	Singlet	Adipocyte
VAT_2M	4263.7	2065	0	0	Singlet	Adipocyte
VAT_2M	8194.2	3022	0.0148457	0	Singlet	Adipocyte
VAT_2M	3978.5	2127	0.2296935	0	Singlet	Adipocyte
VAT_2M	5577.2	2478	0.0398812	0	Singlet	Adipocyte
VAT_2M	1847.4	1222	0.809777	3	Singlet	FAPs
VAT_26M	2197.4	1294	1.1033651	0	Singlet	Adipocyte
VAT_26M	1437.2	945	0.6614528	3	Singlet	FAPs
VAT_26M	1971.6	1227	2.2127872	3	Singlet	FAPs
VAT_26M	1843.4	1054	0	0	Singlet	Adipocyte
VAT_26M	2242.1	1258	0.0094893	0	Singlet	Adipocyte
VAT_26M	1871	1080	0.7187662	0	Singlet	Adipocyte
VAT_26M	1604.4	949	0.2212709	0	Singlet	Adipocyte
VAT_26M	2293.6	1294	0.0530867	0	Singlet	Adipocyte
VAT_26M	1164	762	0.3099153	0	Singlet	Adipocyte
VAT_26M	2227.2	1171	0.1169417	0	Singlet	Adipocyte
VAT_26M	2229.7	1269	3.3523575	0	Singlet	Adipocyte
VAT_26M	3987.7	1981	0.0150956	0	Singlet	Adipocyte
VAT_26M	1469.9	905	1.232285	3	Singlet	FAPs
VAT_26M	3323.8	1765	0	3	Singlet	FAPs
VAT_26M	1706.3	1037	0.0834723	0	Singlet	Adipocyte
VAT_26M	1433.5	998	2.206157	3	Singlet	FAPs
VAT_26M	1382	788	0.8061023	0	Singlet	Adipocyte
VAT_26M	1161.4	743	2.7298373	3	Singlet	FAPs
VAT_26M	3034	1519	0.109472	0	Singlet	Adipocyte
VAT_26M	1861.6	1166	0.0230988	0	Singlet	Adipocyte
VAT_26M	1622.7	1031	0	0	Singlet	Adipocyte
VAT_26M	3003.2	1548	0	0	Singlet	Adipocyte
VAT_26M	3066.6	1668	0.3775232	0	Singlet	Adipocyte
VAT_26M	3125.8	1537	0.3267098	0	Singlet	Adipocyte
VAT_26M	1641	1048	0.0259425	0	Singlet	Adipocyte
VAT_26M	1721.4	1001	0.8448291	0	Singlet	Adipocyte
VAT_26M	2323.8	1360	0	0	Singlet	Adipocyte
VAT_26M	1849	1188	5.2886279	2	Singlet	FAPs
VAT_26M	1073.3	712	0.060817	0	Singlet	Adipocyte
VAT_26M	1759.4	1080	0.0522709	0	Singlet	Adipocyte
VAT_26M	1607.4	940	0.051491	0	Singlet	Adipocyte
VAT_26M	1087	693	0	6	Singlet	Neurons

VAT_26M	5669.1	2490	7.52E-05	0	Singlet	Adipocyte
VAT_26M	2444.1	1352	3.8700713	5	Singlet	Immune_cells
VAT_26M	1088.6	710	0	8	Singlet	Mesothelial
VAT_26M	1160.2	814	5.1348751	7	Singlet	Endothelial_cells
VAT_26M	3359.1	1767	0.3349851	0	Singlet	Adipocyte
VAT_26M	1928.1	1162	0	0	Singlet	Adipocyte
VAT_26M	1372.6	848	0.7880765	0	Singlet	Adipocyte
VAT_26M	1104.6	691	0.2257827	0	Singlet	Adipocyte
VAT_26M	5083.2	2340	0.005205	0	Singlet	Adipocyte
VAT_26M	1563.7	928	0.4443091	0	Singlet	Adipocyte
VAT_26M	1210.8	752	0	0	Singlet	Adipocyte
VAT_26M	2949.2	1626	0.0007195	0	Singlet	Adipocyte
VAT_26M	1707.4	1109	2.2525248	7	Singlet	Endothelial_cells
VAT_26M	4276.8	2108	0	0	Singlet	Adipocyte
VAT_26M	1738.7	1090	0.1351527	0	Singlet	Adipocyte
VAT_26M	1180.8	796	0.4717184	2	Singlet	FAPs
VAT_26M	1600.4	1069	3.5978141	3	Singlet	FAPs
VAT_26M	1194.1	810	0.8779781	2	Singlet	FAPs
VAT_26M	1254.4	849	4.1927702	7	Singlet	Endothelial_cells
VAT_26M	3685.1	1605	0.0281884	0	Singlet	Adipocyte
VAT_26M	3120.2	1562	0.020587	0	Singlet	Adipocyte
VAT_26M	1032.9	629	6.2173062	0	Singlet	Adipocyte
VAT_26M	2444.1	1471	0.0808413	2	Singlet	FAPs
VAT_26M	1110.7	708	0.2942129	0	Singlet	Adipocyte
VAT_26M	1260.4	830	0	0	Singlet	Adipocyte
VAT_26M	2520.9	1374	1.8643293	0	Singlet	Adipocyte
VAT_26M	1793.3	1167	0.6273534	0	Singlet	Adipocyte
VAT_26M	3912	1886	0.001745	0	Singlet	Adipocyte
VAT_26M	3259.7	1712	0.291028	0	Singlet	Adipocyte
VAT_26M	1139.5	749	0.0544753	3	Singlet	FAPs
VAT_26M	2397.1	1309	0.544541	0	Singlet	Adipocyte
VAT_26M	2554.2	1404	0	0	Singlet	Adipocyte
VAT_26M	1125.8	745	0.6625402	3	Singlet	FAPs
VAT_26M	5145.4	2252	0.2660197	0	Singlet	Adipocyte
VAT_26M	1280	781	5.3599598	3	Singlet	FAPs
VAT_26M	2697.7	1399	0	0	Singlet	Adipocyte
VAT_26M	4188.4	1961	0.0454173	0	Singlet	Adipocyte
VAT_26M	1671	1041	0	0	Singlet	Adipocyte
VAT_26M	1932.4	1086	0.0680287	0	Singlet	Adipocyte
VAT_26M	1823.3	1129	3.0328404	2	Singlet	FAPs
VAT_26M	2595.3	1405	0.0100799	0	Singlet	Adipocyte
VAT_26M	2680.5	1453	0.2633726	0	Singlet	Adipocyte
VAT_26M	4077.6	1918	1.3110826	0	Singlet	Adipocyte
VAT_26M	2719.2	1388	0.0284196	0	Singlet	Adipocyte
VAT_26M	3262.1	1664	0.0254186	0	Singlet	Adipocyte
VAT_26M	2161.8	1217	0.5995979	0	Singlet	Adipocyte
VAT_26M	2675.4	1389	1.3303359	0	Singlet	Adipocyte
VAT_26M	1877.4	1082	0	0	Singlet	Adipocyte
VAT_26M	1080.9	746	0.003774	11	Singlet	Smooth_muscle_cells
VAT_26M	2101.1	1240	0.5233513	0	Singlet	Adipocyte
VAT_26M	3077.6	1568	0	0	Singlet	Adipocyte

VAT_26M	1012.3	683	0.3208545	0	Singlet	Adipocyte
VAT_26M	1818.3	1057	0.2214967	0	Singlet	Adipocyte
VAT_26M	1148.1	746	1.7248938	3	Singlet	FAPs
VAT_26M	1991.2	1028	12.288923	5	Singlet	Immune_cells
VAT_26M	1320.4	880	0.001327	0	Singlet	Adipocyte
VAT_26M	1156.3	814	1.1288251	5	Singlet	Immune_cells
VAT_26M	1279	808	2.4164259	0	Singlet	Adipocyte
VAT_26M	5336.2	2346	0.0216639	0	Singlet	Adipocyte
VAT_26M	1278.9	837	0	0	Singlet	Adipocyte
VAT_26M	3547.5	1630	0	0	Singlet	Adipocyte
VAT_26M	3137.4	1519	0.0149749	0	Singlet	Adipocyte
VAT_26M	1086.7	786	1.2955306	7	Singlet	Endothelial_cells
VAT_26M	2015.8	1231	0.3428873	0	Singlet	Adipocyte
VAT_26M	3202	1864	0.0893604	2	Singlet	FAPs
VAT_26M	3494.7	1696	0	0	Singlet	Adipocyte
VAT_26M	1373.2	901	0.7464196	3	Singlet	FAPs
VAT_26M	1459.3	860	0.1297939	0	Singlet	Adipocyte
VAT_26M	5317	2233	0.0111304	0	Singlet	Adipocyte
VAT_26M	2507	1354	0.0105403	0	Singlet	Adipocyte
VAT_26M	1800.9	1194	0.5942355	3	Singlet	FAPs
VAT_26M	1100.9	801	0.949134	4	Singlet	Immune_cells
VAT_26M	1672.3	936	0.2042243	0	Singlet	Adipocyte
VAT_26M	1415.2	853	0.2432809	0	Singlet	Adipocyte
VAT_26M	1032.2	760	1.4431172	3	Singlet	FAPs
VAT_26M	5052	2275	0	0	Singlet	Adipocyte
VAT_26M	2014.5	1221	1.0190531	0	Singlet	Adipocyte
VAT_26M	1385.8	876	0.2788249	0	Singlet	Adipocyte
VAT_26M	1215.4	846	0	2	Singlet	FAPs
VAT_26M	2471.6	1265	1.0174514	0	Singlet	Adipocyte
VAT_26M	1008.8	694	7.1652451	9	Singlet	Mesothelial
VAT_26M	4153.8	1939	0	0	Singlet	Adipocyte
VAT_26M	1167.4	806	0.3877352	6	Singlet	Neurons
VAT_26M	1718.5	1108	0.16545	0	Singlet	Adipocyte
VAT_26M	1398.2	830	5.5866858	0	Singlet	Adipocyte
VAT_26M	1489.9	871	0.1624117	0	Singlet	Adipocyte
VAT_26M	1251.1	856	0.0449799	0	Singlet	Adipocyte
VAT_26M	1590.4	946	0.3488493	0	Singlet	Adipocyte
VAT_26M	1599.6	963	0.0291474	0	Singlet	Adipocyte
VAT_26M	2424	1254	0.0061803	0	Singlet	Adipocyte
VAT_26M	1891.5	1113	0.0728696	0	Singlet	Adipocyte
VAT_26M	1809.5	1082	0.0769373	0	Singlet	Adipocyte
VAT_26M	1312	873	1.1436345	0	Singlet	Adipocyte
VAT_26M	1263.2	850	0.0461348	0	Singlet	Adipocyte
VAT_26M	1210	863	0.1447625	0	Singlet	Adipocyte
VAT_26M	1627.3	1067	4.6527861	2	Singlet	FAPs
VAT_26M	1829.1	1084	0.3938323	0	Singlet	Adipocyte
VAT_26M	1089.1	809	0.4448445	5	Singlet	Immune_cells
VAT_26M	1157.5	750	0.1393851	0	Singlet	Adipocyte
VAT_26M	1275.1	810	0.0819305	0	Singlet	Adipocyte
VAT_26M	1505.5	981	0.0215023	0	Singlet	Adipocyte
VAT_26M	1130.1	748	2.2022028	7	Singlet	Endothelial_cells

VAT_26M	1954.1	1215	4.6094561	2	Singlet	FAPs
VAT_26M	1709.5	1013	1.5446401	0	Singlet	Adipocyte
VAT_26M	1289.6	823	0.6908265	0	Singlet	Adipocyte
VAT_26M	1307.2	772	0	0	Singlet	Adipocyte
VAT_26M	1466	827	2.0048384	0	Singlet	Adipocyte
VAT_26M	1625.1	1071	0.0137604	0	Singlet	Adipocyte
VAT_26M	1360	842	2.5270672	0	Singlet	Adipocyte
VAT_26M	1085.3	684	0.0831097	0	Singlet	Adipocyte
VAT_26M	1820.3	1049	0	0	Singlet	Adipocyte
VAT_26M	2416.9	1349	10.9651	5	Singlet	Immune_cells
VAT_26M	4126.4	2025	0	0	Singlet	Adipocyte
VAT_26M	1055.8	727	0.514102	0	Singlet	Adipocyte
VAT_26M	1038.3	695	1.7435387	2	Singlet	FAPs
VAT_26M	1392	917	7.9703564	7	Singlet	Endothelial_cells
VAT_26M	2204.9	1245	0.4529045	0	Singlet	Adipocyte
VAT_26M	4431.7	2077	0.212251	0	Singlet	Adipocyte
VAT_26M	2373.4	1338	1.574255	0	Singlet	Adipocyte
VAT_26M	2801.4	1526	0	0	Singlet	Adipocyte
VAT_26M	1623.7	970	5.0981288	0	Singlet	Adipocyte
VAT_26M	2274.8	1276	0.0350124	0	Singlet	Adipocyte
VAT_26M	3070.6	1568	0.0871306	0	Singlet	Adipocyte
VAT_26M	1504.7	925	0.0143127	0	Singlet	Adipocyte
VAT_26M	2744	1497	1.5826289	0	Singlet	Adipocyte
VAT_26M	1088.3	733	1.267882	3	Singlet	FAPs
VAT_26M	1672	1040	1.8621538	5	Singlet	Immune_cells
VAT_26M	1003.5	730	1.1427801	5	Singlet	Immune_cells
VAT_26M	1045.1	801	1.388583	8	Singlet	Mesothelial
VAT_26M	2044.9	1246	0.0527822	0	Singlet	Adipocyte
VAT_26M	2000.4	1150	0.0241908	0	Singlet	Adipocyte
VAT_26M	1296.6	890	0.26443	0	Singlet	Adipocyte
VAT_26M	1195.3	844	0.5201115	0	Singlet	Adipocyte
VAT_26M	1311.1	803	9.0265806	3	Singlet	FAPs
VAT_26M	1543.9	977	0.1020379	0	Singlet	Adipocyte
VAT_26M	2489.2	1369	0.0863091	0	Singlet	Adipocyte
VAT_26M	1321.4	840	0.2237283	0	Singlet	Adipocyte
VAT_26M	5496.8	2366	0.6318996	0	Singlet	Adipocyte
VAT_26M	2346.1	1302	0.3224036	0	Singlet	Adipocyte
VAT_26M	3206.4	1660	1.6541883	0	Singlet	Adipocyte
VAT_26M	2183.8	1232	0.0489033	0	Singlet	Adipocyte
VAT_26M	1509.3	920	1.259751	3	Singlet	FAPs
VAT_26M	1025.9	706	0.0726365	6	Singlet	Neurons
VAT_26M	1610.6	970	0.1657627	0	Singlet	Adipocyte
VAT_26M	1029.6	677	0.2296876	3	Singlet	FAPs
VAT_26M	1221.1	834	0.4461245	2	Singlet	FAPs
VAT_26M	1458.2	839	12.47542	3	Singlet	FAPs
VAT_26M	4107.9	2049	0.0476196	0	Singlet	Adipocyte
VAT_26M	2271.7	1273	0.4314185	0	Singlet	Adipocyte
VAT_26M	2569.3	1393	0.9045044	0	Singlet	Adipocyte
VAT_26M	1365.6	831	0.361041	0	Singlet	Adipocyte
VAT_26M	2698.7	1536	2.9219321	3	Singlet	FAPs
VAT_26M	3696.8	1866	1.2308156	0	Singlet	Adipocyte

VAT_26M	1354.5	908	1.0839993	3	Singlet	FAPs
VAT_26M	2130.5	1227	0.4292911	0	Singlet	Adipocyte
VAT_26M	1560.1	989	0.3744745	0	Singlet	Adipocyte
VAT_26M	2787.5	1356	0.0655897	0	Singlet	Adipocyte
VAT_26M	2381.2	1233	0.1769735	0	Singlet	Adipocyte
VAT_26M	2068.7	1179	0	0	Singlet	Adipocyte
VAT_26M	3401.5	1605	0.1139286	0	Singlet	Adipocyte
VAT_26M	2562.7	1442	0	0	Singlet	Adipocyte
VAT_26M	1471.4	953	2.9254827	3	Singlet	FAPs
VAT_26M	2058.9	1160	0.1160746	0	Singlet	Adipocyte
VAT_26M	1450.3	817	13.151646	5	Singlet	Immune_cells
VAT_26M	1270.6	865	1.0129842	3	Singlet	FAPs
VAT_26M	1971.3	1189	0.0680673	0	Singlet	Adipocyte
VAT_26M	2052.7	1182	0	0	Singlet	Adipocyte
VAT_26M	1327.2	860	0	0	Singlet	Adipocyte
VAT_26M	2130	1257	2.0471903	0	Singlet	Adipocyte
VAT_26M	2579.5	1422	0.9784863	0	Singlet	Adipocyte
VAT_26M	3458	1757	0.074885	0	Singlet	Adipocyte
VAT_26M	1074.4	711	0.4521904	0	Singlet	Adipocyte
VAT_26M	1995.8	1167	0.4689726	0	Singlet	Adipocyte
VAT_26M	3522.5	1711	0.1453916	0	Singlet	Adipocyte
VAT_26M	2461.3	1412	0.0473826	0	Singlet	Adipocyte
VAT_26M	1208.6	788	0.1297188	0	Singlet	Adipocyte
VAT_26M	1613.2	895	0.03273	0	Singlet	Adipocyte
VAT_26M	5761.6	2415	0	0	Singlet	Adipocyte
VAT_26M	1329.6	840	0.4103671	0	Singlet	Adipocyte
VAT_26M	1431.1	867	0.4980855	0	Singlet	Adipocyte
VAT_26M	2924.6	1586	0.1296888	0	Singlet	Adipocyte
VAT_26M	4563.1	2206	0.2051604	0	Singlet	Adipocyte
VAT_26M	2490.8	1336	0.3502463	0	Singlet	Adipocyte
VAT_26M	1887.1	1053	0.4644168	0	Singlet	Adipocyte
VAT_26M	1078	726	1.3773165	0	Singlet	Adipocyte
VAT_26M	4659.1	2179	0.0321081	0	Singlet	Adipocyte
VAT_26M	1031.8	719	0.0522623	0	Singlet	Adipocyte
VAT_26M	2597.2	1392	0.0090602	0	Singlet	Adipocyte
VAT_26M	1396.3	866	0.4604025	0	Singlet	Adipocyte
VAT_26M	1829.8	1054	1.4547625	0	Singlet	Adipocyte
VAT_26M	1790	1041	7.5244984	7	Singlet	Endothelial_cells
VAT_26M	2272.2	1266	0.1003868	0	Singlet	Adipocyte
VAT_26M	1083.9	723	0.6288132	6	Singlet	Neurons
VAT_26M	1509.6	958	0.0941737	0	Singlet	Adipocyte
VAT_26M	3809.6	1944	0.1649354	0	Singlet	Adipocyte
VAT_26M	1245	825	4.3243242	2	Singlet	FAPs
VAT_26M	2984	1635	0.0539171	0	Singlet	Adipocyte
VAT_26M	1442.4	883	0.2122194	0	Singlet	Adipocyte
VAT_26M	1431.7	1011	2.7760568	1	Singlet	Immune_cells
VAT_26M	2427.3	1359	0.0078713	0	Singlet	Adipocyte
VAT_26M	1832.6	1089	0.3285308	0	Singlet	Adipocyte
VAT_26M	4395.9	2088	0.0657087	0	Singlet	Adipocyte
VAT_26M	2869.3	1678	1.6163361	3	Singlet	FAPs
VAT_26M	6196.2	2540	0.094949	0	Singlet	Adipocyte

VAT_26M	4462.2	1928	0	0	Singlet	Adipocyte
VAT_26M	2222.4	1243	0.3783826	0	Singlet	Adipocyte
VAT_26M	2618.9	1429	0.8599638	0	Singlet	Adipocyte
VAT_26M	1393.2	884	4.2602363	3	Singlet	FAPs
VAT_26M	4020.2	1878	1.9008577	0	Singlet	Adipocyte
VAT_26M	3802.3	1877	0.0167779	0	Singlet	Adipocyte
VAT_26M	1601.2	1028	0.0475557	0	Singlet	Adipocyte
VAT_26M	2113.3	1242	0.0804909	0	Singlet	Adipocyte
VAT_26M	1505	951	5.9688313	7	Singlet	Endothelial_cells
VAT_26M	1643.4	1070	0.8361616	0	Singlet	Adipocyte
VAT_26M	2157.7	1211	0	0	Singlet	Adipocyte
VAT_26M	2683.8	1536	5.195399	2	Singlet	FAPs
VAT_26M	2760.5	1481	0.0024846	0	Singlet	Adipocyte
VAT_26M	1414.5	943	0.1222259	5	Singlet	Immune_cells
VAT_26M	2278	1371	0.5292518	0	Singlet	Adipocyte
VAT_26M	1042.7	695	0.7125025	0	Singlet	Adipocyte
VAT_26M	2409.5	1252	0.122235	0	Singlet	Adipocyte
VAT_26M	2807	1601	0.0262361	0	Singlet	Adipocyte
VAT_26M	3444.8	1714	3.4113174	0	Singlet	Adipocyte
VAT_26M	1469.9	932	0.2691808	0	Singlet	Adipocyte
VAT_26M	3420.5	1681	0.0228775	0	Singlet	Adipocyte
VAT_26M	1076.4	669	0.4521696	3	Singlet	FAPs
VAT_26M	2163.2	1203	0	0	Singlet	Adipocyte
VAT_26M	2116.9	1242	1.1190098	0	Singlet	Adipocyte
VAT_26M	1674	995	0.1038282	0	Singlet	Adipocyte
VAT_26M	1942.1	1182	3.0387432	3	Singlet	FAPs
VAT_26M	1850	989	0	0	Singlet	Adipocyte
VAT_26M	1513.4	912	0.0607393	0	Singlet	Adipocyte
VAT_26M	2199.4	1362	0.1008975	0	Singlet	Adipocyte
VAT_26M	1317.8	807	0.0025919	0	Singlet	Adipocyte
VAT_26M	3015.1	1619	1.9101739	0	Singlet	Adipocyte
VAT_26M	1173.3	823	1.6319077	7	Singlet	Endothelial_cells
VAT_26M	1263.1	834	2.3886382	3	Singlet	FAPs
VAT_26M	1365	776	0.0301929	0	Singlet	Adipocyte
VAT_26M	3245.4	1670	0.0386936	0	Singlet	Adipocyte
VAT_26M	1386.4	884	0.3112617	0	Singlet	Adipocyte
VAT_26M	1356.7	839	1.2179768	0	Singlet	Adipocyte
VAT_26M	5550.7	2346	0	0	Singlet	Adipocyte
VAT_26M	1340.5	824	0.1160149	0	Singlet	Adipocyte
VAT_26M	1058.6	701	0	0	Singlet	Adipocyte
VAT_26M	1147.8	745	9.7831224	3	Singlet	FAPs
VAT_26M	1093.8	738	2.0093741	3	Singlet	FAPs
VAT_26M	1117.3	712	0.0446378	0	Singlet	Adipocyte
VAT_26M	4489.2	2401	1.0063559	3	Singlet	FAPs
VAT_26M	1807.2	1135	0.3115542	0	Singlet	Adipocyte
VAT_26M	2353.9	1344	0.074116	0	Singlet	Adipocyte
VAT_26M	1209	772	0.350551	0	Singlet	Adipocyte
VAT_26M	2646.8	1404	0.0276374	0	Singlet	Adipocyte
VAT_26M	4402.2	1976	0	0	Singlet	Adipocyte
VAT_26M	1984	1160	0.0246633	0	Singlet	Adipocyte
VAT_26M	1239	817	1.0076681	5	Singlet	Immune_cells

VAT_26M	1404.2	891	0.8391074	0	Singlet	Adipocyte
VAT_26M	2040.5	1125	0.0656075	0	Singlet	Adipocyte
VAT_26M	1050.4	706	0.2307922	0	Singlet	Adipocyte
VAT_26M	2632.2	1396	0.040359	0	Singlet	Adipocyte
VAT_26M	1504.5	872	0.155401	0	Singlet	Adipocyte
VAT_26M	1409	865	0	0	Singlet	Adipocyte
VAT_26M	1828.1	1098	0	0	Singlet	Adipocyte
VAT_26M	2938.9	1621	0.0485132	0	Singlet	Adipocyte
VAT_26M	1279.9	783	0.0981241	0	Singlet	Adipocyte
VAT_26M	3100.8	1546	0.0612713	0	Singlet	Adipocyte
VAT_26M	2133.3	1242	0.3816771	0	Singlet	Adipocyte
VAT_26M	1766	1130	10.112719	3	Singlet	FAPs
VAT_26M	5034.6	2325	0	0	Singlet	Adipocyte
VAT_26M	2881.2	1499	0.0167251	0	Singlet	Adipocyte
VAT_26M	1319.5	830	13.157877	3	Singlet	FAPs
VAT_26M	1028.5	719	3.2211586	0	Singlet	Adipocyte
VAT_26M	1136.9	731	0.2175474	6	Singlet	Neurons
VAT_26M	4211.7	2064	0	0	Singlet	Adipocyte
VAT_26M	2912	1538	0.0164193	0	Singlet	Adipocyte
VAT_26M	1045.3	702	0.1267189	0	Singlet	Adipocyte
VAT_26M	2346.3	1332	0	0	Singlet	Adipocyte
VAT_26M	3565.5	1824	0	0	Singlet	Adipocyte
VAT_26M	1075.6	766	0.4445935	5	Singlet	Immune_cells
VAT_26M	1293	817	7.9675633	3	Singlet	FAPs
VAT_26M	2547.4	1317	0.2821294	0	Singlet	Adipocyte
VAT_26M	2022.1	1179	0	0	Singlet	Adipocyte
VAT_26M	1661.1	1054	0.0726859	3	Singlet	FAPs
VAT_26M	2463.5	1393	0.1181427	0	Singlet	Adipocyte
VAT_26M	1043.9	701	0.0299991	3	Singlet	FAPs
VAT_26M	1983.3	1146	5.3795106	0	Singlet	Adipocyte
VAT_26M	4523.8	2078	0.0811053	0	Singlet	Adipocyte
VAT_26M	1597.8	1010	0	0	Singlet	Adipocyte
VAT_26M	4744.8	2009	0	0	Singlet	Adipocyte
VAT_26M	3706.6	1757	0.1692049	0	Singlet	Adipocyte
VAT_26M	2547.7	1437	0.0056701	0	Singlet	Adipocyte
VAT_26M	3867	1992	0.0166016	0	Singlet	Adipocyte
VAT_26M	1572.6	998	0.3826813	0	Singlet	Adipocyte
VAT_26M	1002.3	717	2.5998312	5	Singlet	Immune_cells
VAT_26M	1280	801	0	0	Singlet	Adipocyte
VAT_26M	1057.6	634	0.4059094	0	Singlet	Adipocyte
VAT_26M	1567.7	942	13.863696	3	Singlet	FAPs
VAT_26M	3963.8	1844	0	0	Singlet	Adipocyte
VAT_26M	1418	878	3.3290804	0	Singlet	Adipocyte
VAT_26M	1212.3	839	0	0	Singlet	Adipocyte
VAT_26M	3324.4	1731	0.2755905	0	Singlet	Adipocyte
VAT_26M	1353.2	849	0.113713	6	Singlet	Neurons
VAT_26M	1057.7	658	0.1615992	0	Singlet	Adipocyte
VAT_26M	1437.9	871	0	0	Singlet	Adipocyte
VAT_26M	1566.7	980	5.9192325	3	Singlet	FAPs
VAT_26M	1258.6	871	0.5930522	3	Singlet	FAPs
VAT_26M	3083.2	1624	0.383744	0	Singlet	Adipocyte

VAT_26M	1987.1	1095	0	0	Singlet	Adipocyte
VAT_26M	4873.1	2321	0.0257955	0	Singlet	Adipocyte
VAT_26M	1208.8	744	4.8090586	3	Singlet	FAPs
VAT_26M	4360.4	2134	0.0472338	0	Singlet	Adipocyte
VAT_26M	1246.4	798	0.1279679	0	Singlet	Adipocyte
VAT_26M	3384.7	1778	0.1466746	0	Singlet	Adipocyte
VAT_26M	1048.2	694	0.0828206	0	Singlet	Adipocyte
VAT_26M	3874.2	1772	0.4231206	0	Singlet	Adipocyte
VAT_26M	6962.2	2584	0	0	Singlet	Adipocyte
VAT_26M	4431.2	2021	0.3868495	0	Singlet	Adipocyte
VAT_26M	1321	867	0.0007499	0	Singlet	Adipocyte
VAT_26M	3267.8	1623	0.7386679	0	Singlet	Adipocyte
VAT_26M	1652.7	951	0.8921024	7	Singlet	Endothelial_cells
VAT_26M	1018.3	612	3.8184926	7	Singlet	Endothelial_cells
VAT_26M	1520.3	890	0	0	Singlet	Adipocyte
VAT_26M	2295.3	1300	0.1410414	0	Singlet	Adipocyte
VAT_26M	2183.1	1251	0	0	Singlet	Adipocyte
VAT_26M	2939.3	1528	0.0493367	0	Singlet	Adipocyte
VAT_26M	1069.6	768	0.8822061	5	Singlet	Immune_cells
VAT_26M	3368.8	1682	0	0	Singlet	Adipocyte
VAT_26M	1707.9	1065	0.4529008	2	Singlet	FAPs
VAT_26M	2926.4	1585	0.0923906	0	Singlet	Adipocyte
VAT_26M	1442.4	928	0.2119348	5	Singlet	Immune_cells
VAT_26M	2383.2	1280	3.7577385	0	Singlet	Adipocyte
VAT_26M	2877.9	1489	0.0009997	0	Singlet	Adipocyte
VAT_26M	2383.4	1216	0.7356804	0	Singlet	Adipocyte
VAT_26M	1101.1	702	3.6260109	3	Singlet	FAPs
VAT_26M	2705.2	1588	10.179832	2	Singlet	FAPs
VAT_26M	1408.1	878	0.0538794	0	Singlet	Adipocyte
VAT_26M	2429.2	1410	7.8716138	5	Singlet	Immune_cells
VAT_26M	1123.6	778	0.7612296	5	Singlet	Immune_cells
VAT_26M	1135	680	0.054825	0	Singlet	Adipocyte
VAT_26M	7038.8	2680	0	0	Singlet	Adipocyte
VAT_26M	2690.2	1502	0.3846896	0	Singlet	Adipocyte
VAT_26M	2950.1	1732	2.9458747	3	Singlet	FAPs
VAT_26M	2958.6	1572	0	0	Singlet	Adipocyte
VAT_26M	1074.1	705	1.8466499	0	Singlet	Adipocyte
VAT_26M	2273.7	1331	0.1211381	0	Singlet	Adipocyte
VAT_26M	2128	1172	0.0162477	0	Singlet	Adipocyte
VAT_26M	3802.6	1950	0.4960992	0	Singlet	Adipocyte
VAT_26M	2646.8	1470	6.4052041	3	Singlet	FAPs
VAT_26M	2121.3	1131	0	0	Singlet	Adipocyte
VAT_26M	4708	2251	0	0	Singlet	Adipocyte
VAT_26M	2570.8	1497	0.6321542	3	Singlet	FAPs
VAT_26M	1126.5	777	0	3	Singlet	FAPs
VAT_26M	3077.6	1552	0	0	Singlet	Adipocyte
VAT_26M	1567.8	1003	1.1968109	0	Singlet	Adipocyte
VAT_26M	1446.8	868	1.1914197	0	Singlet	Adipocyte
VAT_26M	2017.2	1192	0.0367457	0	Singlet	Adipocyte
VAT_26M	3453.5	1856	0.0507127	0	Singlet	Adipocyte
VAT_26M	1045.7	675	7.8984508	3	Singlet	FAPs

VAT_26M	2034.5	1159	0.6744305	0	Singlet	Adipocyte
VAT_26M	4550.8	2028	4.224538	0	Singlet	Adipocyte
VAT_26M	1218.3	852	0.0767512	5	Singlet	Immune_cells
VAT_26M	1235.4	904	0	5	Singlet	Immune_cells
VAT_26M	1906.3	1233	4.0127833	2	Singlet	FAPs
VAT_26M	1308	803	0.3053608	0	Singlet	Adipocyte
VAT_26M	3315.7	1774	0.0402163	0	Singlet	Adipocyte
VAT_26M	5922.8	2608	0.3568658	0	Singlet	Adipocyte
VAT_26M	1086.2	691	7.6210492	0	Singlet	Adipocyte
VAT_26M	1298.7	796	4.1901168	0	Singlet	Adipocyte
VAT_26M	3250.7	1697	0	0	Singlet	Adipocyte
VAT_26M	6987.9	2881	0.0047335	0	Singlet	Adipocyte
VAT_26M	1367.4	875	0.2589013	0	Singlet	Adipocyte
VAT_26M	2759.5	1410	0.5795934	0	Singlet	Adipocyte
VAT_26M	1001.3	665	0.0661785	0	Singlet	Adipocyte
VAT_26M	1405.8	895	0.0262263	0	Singlet	Adipocyte
VAT_26M	1419.5	915	0.4534988	3	Singlet	FAPs
VAT_26M	2507.8	1354	0.1351901	0	Singlet	Adipocyte
VAT_26M	1238.5	683	2.9159866	3	Singlet	FAPs
VAT_26M	1041	688	0.0518302	0	Singlet	Adipocyte
VAT_26M	2145.6	1194	0.2044652	0	Singlet	Adipocyte
VAT_26M	1340.5	879	2.9008331	7	Singlet	Endothelial_cells
VAT_26M	1715.6	955	12.206024	7	Singlet	Endothelial_cells
VAT_26M	3540.1	1721	0	0	Singlet	Adipocyte
VAT_26M	3679.1	1709	0.0866457	0	Singlet	Adipocyte
VAT_26M	1098.3	745	0.4962355	0	Singlet	Adipocyte
VAT_26M	1968.4	1106	0.0173182	0	Singlet	Adipocyte
VAT_26M	2370.9	1412	0.0092364	0	Singlet	Adipocyte
VAT_26M	1001.9	625	2.9351158	7	Singlet	Endothelial_cells
VAT_26M	1129.1	750	0.0134832	0	Singlet	Adipocyte
VAT_26M	1112.1	780	0.0587639	3	Singlet	FAPs
VAT_26M	1044.2	711	0.1127409	0	Singlet	Adipocyte
VAT_26M	1295.2	923	3.2201887	5	Singlet	Immune_cells
VAT_26M	1443.3	1008	2.3562301	3	Singlet	FAPs
VAT_26M	2518.6	1493	0.0805672	0	Singlet	Adipocyte
VAT_26M	2500.8	1412	1.448109	0	Singlet	Adipocyte
VAT_26M	1445	1015	0.0027069	5	Singlet	Immune_cells
VAT_26M	1556.2	965	1.3469025	0	Singlet	Adipocyte
VAT_26M	1229.3	837	0.2635215	0	Singlet	Adipocyte
VAT_26M	1044.1	631	0.1822513	6	Singlet	Neurons
VAT_26M	1688.6	1121	1.321918	0	Singlet	Adipocyte
VAT_26M	1579.9	944	1.3477629	0	Singlet	Adipocyte
VAT_26M	1793.6	1040	0.0800657	0	Singlet	Adipocyte
VAT_26M	2480	1286	0.3082516	0	Singlet	Adipocyte
VAT_26M	1631.6	1019	1.7677111	7	Singlet	Endothelial_cells
VAT_26M	1162.1	798	0.6392857	2	Singlet	FAPs
VAT_26M	1522	864	0.1178004	0	Singlet	Adipocyte
VAT_26M	1875.7	1134	0.8395929	0	Singlet	Adipocyte
VAT_26M	2951.1	1521	1.1148109	0	Singlet	Adipocyte
VAT_26M	4657.1	2171	0.0586548	0	Singlet	Adipocyte
VAT_26M	2336.5	1325	7.752668	7	Singlet	Endothelial_cells

VAT_26M	2126.1	1221	0.0648089	0	Singlet	Adipocyte
VAT_26M	2112.4	1265	1.3526257	0	Singlet	Adipocyte
VAT_26M	2091.3	1265	0.0137059	0	Singlet	Adipocyte
VAT_26M	1185.9	750	0.0550514	0	Singlet	Adipocyte
VAT_26M	2320	1280	0.0516236	0	Singlet	Adipocyte
VAT_26M	2090.1	1104	0.63024	0	Singlet	Adipocyte
VAT_26M	2208.1	1261	0.3485841	0	Singlet	Adipocyte
VAT_26M	2593.8	1544	0.4648376	3	Singlet	FAPs
VAT_26M	1534	910	0.0201181	0	Singlet	Adipocyte
VAT_26M	1593.8	950	0.4435886	0	Singlet	Adipocyte
VAT_26M	2228.7	1272	0.3610351	0	Singlet	Adipocyte
VAT_26M	2120.6	1189	5.1160309	0	Singlet	Adipocyte
VAT_26M	3342.5	1516	0.0980667	0	Singlet	Adipocyte
VAT_26M	1652.5	942	0.2510213	0	Singlet	Adipocyte
VAT_26M	3822.6	1881	0	0	Singlet	Adipocyte
VAT_26M	1628.8	963	0	0	Singlet	Adipocyte
VAT_26M	1754.8	1075	0	0	Singlet	Adipocyte
VAT_26M	1064.5	647	0.1326869	0	Singlet	Adipocyte
VAT_26M	1306.8	818	1.0749086	0	Singlet	Adipocyte
VAT_26M	2374.7	1245	0.132435	0	Singlet	Adipocyte
VAT_26M	1547.6	921	0	0	Singlet	Adipocyte
VAT_26M	1991.5	1183	0	0	Singlet	Adipocyte
VAT_26M	3608.9	1796	2.263405	0	Singlet	Adipocyte
VAT_26M	1066.9	692	0.4935178	5	Singlet	Immune_cells
VAT_26M	4339.1	2088	0.0136419	0	Singlet	Adipocyte
VAT_26M	3703.8	1848	2.4404565	0	Singlet	Adipocyte
VAT_26M	1758.1	1105	1.27176	5	Singlet	Immune_cells
VAT_26M	1701.4	1014	0.0386471	0	Singlet	Adipocyte
VAT_26M	1277.3	836	9.5188537	2	Singlet	FAPs
VAT_26M	2405.6	1233	4.5692843	0	Singlet	Adipocyte
VAT_26M	3672.3	1881	0.0049839	0	Singlet	Adipocyte
VAT_26M	1134.3	747	2.4421654	0	Singlet	Adipocyte
VAT_26M	2011.5	1107	0.2217553	0	Singlet	Adipocyte
VAT_26M	2178	1163	7.048543	0	Singlet	Adipocyte
VAT_26M	1344	880	3.0784242	5	Singlet	Immune_cells
VAT_26M	5515.3	2441	0.6648643	0	Singlet	Adipocyte
VAT_26M	1627.1	1008	3.2302082	0	Singlet	Adipocyte
VAT_26M	1365.1	855	0.0286324	0	Singlet	Adipocyte
VAT_26M	2358.9	1414	0	0	Singlet	Adipocyte
VAT_26M	3965	1956	0.0593747	0	Singlet	Adipocyte
VAT_26M	1085.4	708	0.1190006	0	Singlet	Adipocyte
VAT_26M	1820.1	1165	4.706193	7	Singlet	Endothelial_cells
VAT_26M	1258.2	767	0.0522374	0	Singlet	Adipocyte
VAT_26M	1269.6	757	0.0270066	0	Singlet	Adipocyte
VAT_26M	3985	1878	4.428629	0	Singlet	Adipocyte
VAT_26M	1928.9	1103	0.0085346	0	Singlet	Adipocyte
VAT_26M	1939.7	1143	1.3339626	0	Singlet	Adipocyte
VAT_26M	2550.4	1333	0.2090495	0	Singlet	Adipocyte
VAT_26M	1509.8	897	0.0546601	0	Singlet	Adipocyte
VAT_26M	1750.8	1176	1.3212681	7	Singlet	Endothelial_cells
VAT_26M	1045.1	728	2.6412853	3	Singlet	FAPs

VAT_26M	2357.7	1304	0.406474	0	Singlet	Adipocyte
VAT_26M	1087.4	680	0.0191425	0	Singlet	Adipocyte
VAT_26M	2362.2	1348	5.2840585	2	Singlet	FAPs
VAT_26M	1328.8	871	0	0	Singlet	Adipocyte
VAT_26M	1959.8	1168	0	0	Singlet	Adipocyte
VAT_26M	1293.6	785	0.5500262	0	Singlet	Adipocyte
VAT_26M	1025.7	789	0.217002	4	Singlet	Immune_cells
VAT_26M	1790.3	1060	11.254686	9	Singlet	Mesothelial
VAT_26M	1369.5	827	0.6533925	0	Singlet	Adipocyte
VAT_26M	5257.2	2259	2.1732859	0	Singlet	Adipocyte
VAT_26M	2705.3	1427	4.4945524	0	Singlet	Adipocyte
VAT_26M	3320.7	1667	0	0	Singlet	Adipocyte
VAT_26M	1271.7	811	0.4011362	0	Singlet	Adipocyte
VAT_26M	2499.2	1387	4.5365691	2	Singlet	FAPs
VAT_26M	1740.6	992	0.0620027	0	Singlet	Adipocyte
VAT_26M	5390.7	2351	0.2595487	0	Singlet	Adipocyte
VAT_26M	2473.7	1407	0.0825251	0	Singlet	Adipocyte
VAT_26M	1252.7	804	1.8078636	3	Singlet	FAPs
VAT_26M	1232.6	730	8.8998119	3	Singlet	FAPs
VAT_26M	2419.7	1353	0.6493702	0	Singlet	Adipocyte
VAT_26M	1615	925	6.0121215	5	Singlet	Immune_cells
VAT_26M	1991.7	1256	8.0652427	7	Singlet	Endothelial_cells
VAT_26M	2768.1	1709	1.4298544	7	Singlet	Endothelial_cells
VAT_26M	2109.4	1322	2.9968648	7	Singlet	Endothelial_cells
VAT_26M	2308	1314	0.0224608	0	Singlet	Adipocyte
VAT_26M	1032.3	696	0.693538	0	Singlet	Adipocyte
VAT_26M	2223.5	1202	0	0	Singlet	Adipocyte
VAT_26M	3887	1854	0.0157284	0	Singlet	Adipocyte
VAT_26M	1289.7	799	2.3350726	3	Singlet	FAPs
VAT_26M	1713.7	999	0.0405361	0	Singlet	Adipocyte
VAT_26M	2005.4	1215	0.7650414	0	Singlet	Adipocyte
VAT_26M	4926	2145	0.0933695	0	Singlet	Adipocyte
VAT_26M	3177.4	1605	1.7548231	0	Singlet	Adipocyte
VAT_26M	2592.4	1484	0.237848	0	Singlet	Adipocyte
VAT_26M	3212.3	1622	0.2464933	0	Singlet	Adipocyte
VAT_26M	1034.9	692	1.4030043	0	Singlet	Adipocyte
VAT_26M	5373.5	2371	0.3699493	0	Singlet	Adipocyte
VAT_26M	4364	1931	0	0	Singlet	Adipocyte
VAT_26M	1600.5	941	3.428481	0	Singlet	Adipocyte
VAT_26M	3248.6	1611	0.0368999	0	Singlet	Adipocyte
VAT_26M	1790.8	1139	0.61779	2	Singlet	FAPs
VAT_26M	2119.9	1353	5.045682	3	Singlet	FAPs
VAT_26M	3133.7	1582	0.230304	0	Singlet	Adipocyte
VAT_26M	3973	1872	0.8561267	0	Singlet	Adipocyte
VAT_26M	2550.9	1388	0	0	Singlet	Adipocyte
VAT_26M	2402.5	1195	0.0810718	0	Singlet	Adipocyte
VAT_26M	1156.2	752	5.1741759	7	Singlet	Endothelial_cells
VAT_26M	1100.9	747	5.5831801	2	Singlet	FAPs
VAT_26M	2536.4	1428	0.5162147	0	Singlet	Adipocyte
VAT_26M	1087.3	706	5.1893697	7	Singlet	Endothelial_cells
VAT_26M	1151.5	834	0.5600692	3	Singlet	FAPs

VAT_26M	1037	743	5.9457519	5	Singlet	Immune_cells
VAT_26M	1816.4	1079	0.1433175	0	Singlet	Adipocyte
VAT_26M	3438.3	1757	1.0642645	0	Singlet	Adipocyte
VAT_26M	2382.3	1257	1.5851694	0	Singlet	Adipocyte
VAT_26M	3578.3	1900	5.4400569	7	Singlet	Endothelial_cells
VAT_26M	3218.9	1522	3.3692373	0	Singlet	Adipocyte
VAT_26M	1220.8	777	2.737787	3	Singlet	FAPs
VAT_26M	5884.1	2499	0.0660923	0	Singlet	Adipocyte
VAT_26M	1899.9	1089	0.4081571	0	Singlet	Adipocyte
VAT_26M	1394.4	874	0	0	Singlet	Adipocyte
VAT_26M	2417.2	1354	0.9668253	0	Singlet	Adipocyte
VAT_26M	2015.1	1215	0.2698114	0	Singlet	Adipocyte
VAT_26M	2743.7	1488	0.7192275	0	Singlet	Adipocyte
VAT_26M	1031.8	683	3.2965401	7	Singlet	Endothelial_cells
VAT_26M	2548.9	1404	0.1699568	0	Singlet	Adipocyte
VAT_26M	2797	1570	0.8124385	0	Singlet	Adipocyte
VAT_26M	3893.7	1911	0	0	Singlet	Adipocyte
VAT_26M	1025.2	619	0.1221277	0	Singlet	Adipocyte
VAT_26M	4028.6	1938	0.0105175	0	Singlet	Adipocyte
VAT_26M	1589.6	1017	0.1315957	0	Singlet	Adipocyte
VAT_26M	3162.2	1546	0	0	Singlet	Adipocyte
VAT_26M	2220.6	1354	0.2665987	0	Singlet	Adipocyte
VAT_26M	1900.1	1094	0.0513366	0	Singlet	Adipocyte
VAT_26M	1119.9	793	0.0443373	5	Singlet	Immune_cells
VAT_26M	1332.1	755	0.0604597	0	Singlet	Adipocyte
VAT_26M	3709.8	1865	1.3384562	0	Singlet	Adipocyte
VAT_26M	1105.9	749	0.1620946	11	Singlet	Smooth_muscle_cells
VAT_26M	2221.7	1271	0.2407358	0	Singlet	Adipocyte
VAT_26M	3681	1837	0	0	Singlet	Adipocyte
VAT_26M	1200.9	808	0.1389529	6	Singlet	Neurons
VAT_26M	1187.1	698	0.9605683	0	Singlet	Adipocyte
VAT_26M	1105.3	731	0.8909746	0	Singlet	Adipocyte
VAT_26M	1154.4	632	1.098718	0	Singlet	Adipocyte
VAT_26M	1218.5	786	0.0384539	0	Singlet	Adipocyte
VAT_26M	2858.7	1218	0.967333	0	Singlet	Adipocyte
VAT_26M	1956.1	1122	0.1701094	0	Singlet	Adipocyte
VAT_26M	1212.7	755	0.2101316	0	Singlet	Adipocyte
VAT_26M	1196.9	757	0	0	Singlet	Adipocyte
VAT_26M	1166.7	743	0.2357962	0	Singlet	Adipocyte
VAT_26M	1396.8	920	3.7473399	2	Singlet	FAPs
VAT_26M	2956	1452	0.0338807	0	Singlet	Adipocyte
VAT_26M	5662.2	2472	0	0	Singlet	Adipocyte
VAT_26M	1935.6	1107	0.1349001	0	Singlet	Adipocyte
VAT_26M	1682.8	985	0.4830288	0	Singlet	Adipocyte
VAT_26M	2186.2	1254	0.1033589	0	Singlet	Adipocyte
VAT_26M	4583.8	1930	0.0085372	0	Singlet	Adipocyte
VAT_26M	1704.7	894	0.0375787	6	Singlet	Neurons
VAT_26M	1032.5	709	6.2238029	0	Singlet	Adipocyte
VAT_26M	1503.2	888	0.0920227	0	Singlet	Adipocyte
VAT_26M	1501.4	985	1.6030136	2	Singlet	FAPs
VAT_26M	1097.6	759	2.4238089	7	Singlet	Endothelial_cells

VAT_26M	1148.2	769	0.1567324	0 Singlet	Adipocyte
VAT_26M	4709.6	2033	10.244087	5 Singlet	Immune_cells
VAT_26M	2101.9	1070	0.1126454	0 Singlet	Adipocyte
VAT_26M	1272.7	629	6.1519716	7 Singlet	Endothelial_cells
VAT_26M	2716.4	1474	0.3672256	0 Singlet	Adipocyte
VAT_26M	2147.3	947	0.0340919	0 Singlet	Adipocyte
VAT_26M	2580.1	1413	9.3858187	0 Singlet	Adipocyte
VAT_26M	1615.4	1007	0.1676824	0 Singlet	Adipocyte
VAT_26M	1122.7	736	0.0692494	0 Singlet	Adipocyte
VAT_26M	1674.4	1040	0.0795666	0 Singlet	Adipocyte
VAT_26M	1218.6	766	0.1294681	0 Singlet	Adipocyte
VAT_26M	3310.5	1682	0.0629001	0 Singlet	Adipocyte
VAT_26M	9260.3	3336	0.0732674	0 Singlet	Adipocyte
VAT_26M	1160.4	621	3.2342695	3 Singlet	FAPs
VAT_26M	1805	1056	0.0851475	0 Singlet	Adipocyte
VAT_26M	1225.3	813	6.0500975	3 Singlet	FAPs
VAT_26M	1143.9	757	2.7798899	0 Singlet	Adipocyte
VAT_26M	2576.8	1472	0.007838	0 Singlet	Adipocyte
VAT_26M	2338.2	1302	0.7927145	3 Singlet	FAPs
VAT_26M	1852.4	920	1.6102941	7 Singlet	Endothelial_cells
VAT_26M	1468.8	923	0.091392	0 Singlet	Adipocyte
VAT_26M	1746.4	1060	0.2077021	0 Singlet	Adipocyte
VAT_26M	3408.2	1756	0.0140007	0 Singlet	Adipocyte
VAT_26M	2054.5	1236	0	0 Singlet	Adipocyte
VAT_26M	1519.8	987	0.5842084	2 Singlet	FAPs
VAT_26M	1116.8	722	0.3740278	0 Singlet	Adipocyte
VAT_26M	1052.3	805	0.8037994	3 Singlet	FAPs
VAT_26M	1884.5	1131	0.3377455	0 Singlet	Adipocyte
VAT_26M	2452.5	1415	0.0882779	0 Singlet	Adipocyte
VAT_26M	1779.7	1101	2.8195509	0 Singlet	Adipocyte
VAT_26M	1009.2	658	0	0 Singlet	Adipocyte
VAT_26M	4481.4	1947	0	0 Singlet	Adipocyte
VAT_26M	1793.3	1186	5.0061687	7 Singlet	Endothelial_cells
VAT_26M	4090	1981	0.7546483	0 Singlet	Adipocyte
VAT_26M	4662.3	2212	3.3870022	0 Singlet	Adipocyte
VAT_26M	1551.5	1036	2.7045532	2 Singlet	FAPs
VAT_26M	1991.7	1142	0.0168781	0 Singlet	Adipocyte
VAT_26M	1251	813	7.172423	7 Singlet	Endothelial_cells
VAT_26M	1005.5	692	4.7178437	3 Singlet	FAPs
VAT_26M	1870.9	1109	1.4604577	0 Singlet	Adipocyte
VAT_26M	1140	736	5.4448736	3 Singlet	FAPs
VAT_26M	1275.6	776	0.0393248	0 Singlet	Adipocyte
VAT_26M	2170	1432	3.9278229	5 Singlet	Immune_cells
VAT_26M	1941.8	1193	0.0241324	0 Singlet	Adipocyte
VAT_26M	3014.3	1628	0.0309344	0 Singlet	Adipocyte
VAT_26M	2006.4	1233	0.0663397	3 Singlet	FAPs
VAT_26M	1073.8	706	2.9004712	0 Singlet	Adipocyte
VAT_26M	3269.6	1728	0.0472459	0 Singlet	Adipocyte
VAT_26M	4191.7	2073	0	0 Singlet	Adipocyte
VAT_26M	6883.2	2805	0.044444	0 Singlet	Adipocyte
VAT_26M	1359.5	835	1.3230633	0 Singlet	Adipocyte

VAT_26M	1305.2	840	1.2480221	0	Singlet	Adipocyte
VAT_26M	3342.3	1663	0.0587497	0	Singlet	Adipocyte
VAT_26M	1952.4	1110	0.6424137	0	Singlet	Adipocyte
VAT_26M	2758.5	1404	0.0025543	0	Singlet	Adipocyte
VAT_26M	1128.3	765	0.7485929	0	Singlet	Adipocyte
VAT_26M	1675.5	1077	2.0719633	2	Singlet	FAPs
VAT_26M	1403.5	830	0	0	Singlet	Adipocyte
VAT_26M	2230.7	1293	0.231691	0	Singlet	Adipocyte
VAT_26M	2040.1	1248	0.0274401	0	Singlet	Adipocyte
VAT_26M	1472.7	972	0.3039068	3	Singlet	FAPs
VAT_26M	1615.2	976	3.9256983	5	Singlet	Immune_cells
VAT_26M	2616.7	1450	0.4629972	0	Singlet	Adipocyte
VAT_26M	3894.5	1815	0	0	Singlet	Adipocyte
VAT_26M	1065.3	717	0.0607254	0	Singlet	Adipocyte
VAT_26M	1097.2	691	0.3100712	0	Singlet	Adipocyte
VAT_26M	1285.5	882	3.1940738	3	Singlet	FAPs
VAT_26M	1018.8	666	0.2723196	6	Singlet	Neurons
VAT_26M	1991.8	1256	1.1368875	0	Singlet	Adipocyte
VAT_26M	2496.3	1384	0.044044	0	Singlet	Adipocyte
VAT_26M	2028.9	1187	0.0145027	0	Singlet	Adipocyte
VAT_26M	3575.7	1778	0	0	Singlet	Adipocyte
VAT_26M	1655.9	968	2.2599342	0	Singlet	Adipocyte
VAT_26M	2991.2	1531	0	0	Singlet	Adipocyte
VAT_26M	1960	1049	0.9143189	0	Singlet	Adipocyte
VAT_26M	1892.5	1213	0.2169084	0	Singlet	Adipocyte
VAT_26M	1466.3	871	0.6134098	0	Singlet	Adipocyte
VAT_26M	2785.7	1372	4.5360316	0	Singlet	Adipocyte
VAT_26M	1880.5	1119	0.4123806	0	Singlet	Adipocyte
VAT_26M	1376.1	917	0.0386022	0	Singlet	Adipocyte
VAT_26M	1945.4	1237	5.5090641	3	Singlet	FAPs
VAT_26M	2187	1256	0.2413833	0	Singlet	Adipocyte
VAT_26M	3540.4	1794	0	0	Singlet	Adipocyte
VAT_26M	1158.4	712	0.2478301	0	Singlet	Adipocyte
VAT_26M	1492.9	892	0.4538921	0	Singlet	Adipocyte
VAT_26M	1594.5	1050	0.0180742	0	Singlet	Adipocyte
VAT_26M	2210.1	1254	0.0113214	0	Singlet	Adipocyte
VAT_26M	1206.2	821	0.430468	3	Singlet	FAPs
VAT_26M	1135.4	700	0.1330147	0	Singlet	Adipocyte
VAT_26M	1324.2	865	2.3694063	3	Singlet	FAPs
VAT_26M	1548.1	913	0.0555287	0	Singlet	Adipocyte
VAT_26M	2371.5	1400	1.8725398	0	Singlet	Adipocyte
VAT_26M	1152	803	0.0123771	0	Singlet	Adipocyte
VAT_26M	3335.1	1704	0.1133399	0	Singlet	Adipocyte
VAT_26M	2137.5	1199	0.0031439	0	Singlet	Adipocyte
VAT_26M	1075.3	784	5.0051788	3	Singlet	FAPs
VAT_26M	4200.6	2155	0	0	Singlet	Adipocyte
VAT_26M	2416.6	1335	0.0076478	0	Singlet	Adipocyte
VAT_26M	3133	1673	0.1642292	0	Singlet	Adipocyte
VAT_26M	3392.9	1707	0.0295913	0	Singlet	Adipocyte
VAT_26M	2799.9	1482	0.0622999	0	Singlet	Adipocyte
VAT_26M	2575.8	1527	0.0532461	2	Singlet	FAPs

VAT_26M	1738.2	1244	0.4836708	3	Singlet	FAPs
VAT_26M	1026.3	651	0.5675869	3	Singlet	FAPs
VAT_26M	1644.5	1046	0.0520323	3	Singlet	FAPs
VAT_26M	1326	899	2.9361746	2	Singlet	FAPs
VAT_26M	6208.8	2540	0.0305551	0	Singlet	Adipocyte
VAT_26M	1014.9	699	0	3	Singlet	FAPs
VAT_26M	1243.1	737	0.0480203	0	Singlet	Adipocyte
VAT_26M	1999.9	1134	0.0261167	0	Singlet	Adipocyte
VAT_26M	1806.4	1082	0.0887532	0	Singlet	Adipocyte
VAT_26M	1150.7	720	0.0675342	0	Singlet	Adipocyte
VAT_26M	1539.2	964	0	0	Singlet	Adipocyte
VAT_26M	1275	737	0.4726903	0	Singlet	Adipocyte
VAT_26M	1157.1	794	1.4649634	0	Singlet	Adipocyte
VAT_26M	1807.4	1030	0.249277	0	Singlet	Adipocyte
VAT_26M	4384.1	2053	12.08004	0	Singlet	Adipocyte
VAT_26M	1385.8	833	0.5660636	0	Singlet	Adipocyte
VAT_26M	3456.9	1704	0.2413151	0	Singlet	Adipocyte
VAT_26M	2879.6	1518	0.1796752	0	Singlet	Adipocyte
VAT_26M	1506.8	931	7.1896113	2	Singlet	FAPs
VAT_26M	2054.7	1178	0.0311989	0	Singlet	Adipocyte
VAT_26M	2557.8	1406	0	0	Singlet	Adipocyte
VAT_26M	1529.8	956	2.5303072	3	Singlet	FAPs
VAT_26M	5555.4	2403	0.907064	0	Singlet	Adipocyte
VAT_26M	1605.1	994	0.5259517	3	Singlet	FAPs
VAT_26M	1284.2	810	7.1696694	3	Singlet	FAPs
VAT_26M	1212.6	738	0.9775485	0	Singlet	Adipocyte
VAT_26M	1092.5	728	0.3096821	0	Singlet	Adipocyte
VAT_26M	1212.1	860	0	5	Singlet	Immune_cells
VAT_26M	3155.3	1686	0.0065984	0	Singlet	Adipocyte
VAT_26M	1041.8	776	3.4321556	3	Singlet	FAPs
VAT_26M	2854.5	1478	0.744824	0	Singlet	Adipocyte
VAT_26M	1140.8	799	0.631235	0	Singlet	Adipocyte
VAT_26M	1407.2	868	3.9858894	0	Singlet	Adipocyte
VAT_26M	2168.4	1259	0.0134422	0	Singlet	Adipocyte
VAT_26M	2281.4	1269	0	0	Singlet	Adipocyte
VAT_26M	1248	603	0.1511948	0	Singlet	Adipocyte
VAT_26M	1372.2	776	1.658415	2	Singlet	FAPs
VAT_26M	2743	1269	0.0947057	0	Singlet	Adipocyte
VAT_26M	1542.8	940	0.7077197	3	Singlet	FAPs
VAT_26M	4671.5	1692	0.0752786	0	Singlet	Adipocyte
VAT_26M	3801.8	1674	0.2645617	0	Singlet	Adipocyte
VAT_26M	2755.9	1524	2.4846991	5	Singlet	Immune_cells
VAT_26M	1345.1	790	9.1181152	7	Singlet	Endothelial_cells
VAT_26M	3733.3	1886	3.1910148	3	Singlet	FAPs
VAT_26M	1383	897	0	11	Singlet	Smooth_muscle_cells
VAT_26M	3577.2	1675	0	0	Singlet	Adipocyte
VAT_26M	1117.9	705	0.7034522	0	Singlet	Adipocyte
VAT_26M	1038	701	2.852275	3	Singlet	FAPs
VAT_26M	2102.8	1151	0.2200362	0	Singlet	Adipocyte
VAT_26M	1021.5	612	0.4778556	2	Singlet	FAPs
VAT_26M	1424.8	949	5.7662819	2	Singlet	FAPs

VAT_26M	2417.8	1328	3.6372334	0	Singlet	Adipocyte
VAT_26M	5502.6	2497	3.5101645	7	Singlet	Endothelial_cells
VAT_26M	2356.4	1377	0.0071636	0	Singlet	Adipocyte
VAT_26M	2905.9	1597	0.0309034	0	Singlet	Adipocyte
VAT_26M	4424.2	2073	0.1441919	0	Singlet	Adipocyte
VAT_26M	3454.7	1579	0	0	Singlet	Adipocyte
VAT_26M	4357.1	2036	0.010948	0	Singlet	Adipocyte
VAT_26M	1290.8	815	0	11	Singlet	Smooth_muscle_cells
VAT_26M	1661.9	1017	0.1507564	0	Singlet	Adipocyte
VAT_26M	1120.6	679	3.7909417	3	Singlet	FAPs
VAT_26M	2097.2	1231	0.3051783	0	Singlet	Adipocyte
VAT_26M	1367.5	876	3.6397794	5	Singlet	Immune_cells
VAT_26M	1724.4	1092	0.6264655	0	Singlet	Adipocyte
VAT_26M	1731.5	896	0.1542805	0	Singlet	Adipocyte
VAT_26M	1098	736	0	3	Singlet	FAPs
VAT_26M	1290.9	710	4.0877633	7	Singlet	Endothelial_cells
VAT_26M	1396.7	901	0.4080269	3	Singlet	FAPs
VAT_26M	1930.4	936	0.1004059	0	Singlet	Adipocyte
VAT_26M	4657.1	1804	0.0073111	0	Singlet	Adipocyte
VAT_26M	1445.1	888	0.5048029	0	Singlet	Adipocyte
VAT_26M	6774.1	2475	2.1932377	0	Singlet	Adipocyte
VAT_26M	1176.3	741	1.3602911	0	Singlet	Adipocyte
VAT_26M	2397.2	1317	0.5974537	0	Singlet	Adipocyte
VAT_26M	2671.5	1170	0.3035269	0	Singlet	Adipocyte
VAT_26M	1687.3	1039	0.7783245	3	Singlet	FAPs
VAT_26M	1435.7	761	0.3189154	0	Singlet	Adipocyte
VAT_26M	1504.6	916	1.0673043	3	Singlet	FAPs
VAT_26M	2804.8	1413	0.2323188	0	Singlet	Adipocyte
VAT_26M	2429.2	1452	2.2724814	9	Singlet	Mesothelial
VAT_26M	2516.2	1412	0.2456415	0	Singlet	Adipocyte
VAT_26M	3534	1744	0	0	Singlet	Adipocyte
VAT_26M	1007.7	639	0.1941763	0	Singlet	Adipocyte
VAT_26M	2931	1478	0.2713044	0	Singlet	Adipocyte
VAT_26M	7130.4	2867	0	0	Singlet	Adipocyte
VAT_26M	2078.1	1180	0.0260048	0	Singlet	Adipocyte
VAT_26M	2024.8	1233	5.5356271	9	Singlet	Mesothelial
VAT_26M	1431.7	892	0.7370947	0	Singlet	Adipocyte
VAT_26M	3988.7	1833	5.0982193	0	Singlet	Adipocyte
VAT_26M	2366	1345	0.9916935	5	Singlet	Immune_cells
VAT_26M	1204.9	772	12.602656	5	Singlet	Immune_cells
VAT_26M	1112.6	710	9.1200359	0	Singlet	Adipocyte
VAT_26M	1848.8	1044	5.51208	3	Singlet	FAPs
VAT_26M	1128.1	710	6.4192535	5	Singlet	Immune_cells
VAT_26M	1042.4	685	0	3	Singlet	FAPs
VAT_26M	2787.3	1484	0.0797317	0	Singlet	Adipocyte
VAT_26M	1417.1	920	0.0670187	0	Singlet	Adipocyte
VAT_26M	2372.5	1321	0.049847	0	Singlet	Adipocyte
VAT_26M	1269.8	825	0.857453	3	Singlet	FAPs
VAT_26M	2062	1099	0.1707867	0	Singlet	Adipocyte
VAT_26M	5309.3	2373	0	0	Singlet	Adipocyte
VAT_26M	1053	678	9.906967	3	Singlet	FAPs

VAT_26M	2108.8	1286	0.0269509	3	Singlet	FAPs
VAT_26M	1987.1	1150	0.0729898	0	Singlet	Adipocyte
VAT_26M	1029.2	712	4.943215	5	Singlet	Immune_cells
VAT_26M	1423.1	914	0.9050048	0	Singlet	Adipocyte
VAT_26M	1746.2	1075	0.064456	10	Singlet	Immune_cells
VAT_26M	1117.9	712	4.2167588	3	Singlet	FAPs
VAT_26M	1860.7	1159	1.113833	0	Singlet	Adipocyte
VAT_26M	1034.3	658	0	0	Singlet	Adipocyte
VAT_26M	1338.4	832	0.4448703	0	Singlet	Adipocyte
VAT_26M	2207.1	1292	0.4493502	0	Singlet	Adipocyte
VAT_26M	2146.2	1278	5.656271	7	Singlet	Endothelial_cells
VAT_26M	3801.2	1881	0.0159793	0	Singlet	Adipocyte
VAT_26M	1691.2	972	2.6841582	0	Singlet	Adipocyte
VAT_26M	4539	2208	0.1304645	0	Singlet	Adipocyte
VAT_26M	2238.8	1262	0.0673312	0	Singlet	Adipocyte
VAT_26M	1676.7	1023	3.4944762	3	Singlet	FAPs
VAT_26M	1323.8	830	0.8110338	0	Singlet	Adipocyte
VAT_26M	1121.5	789	0	0	Singlet	Adipocyte
VAT_26M	3065.7	1564	0.0950149	0	Singlet	Adipocyte
VAT_26M	2316.9	1306	0	0	Singlet	Adipocyte
VAT_26M	1777.1	1063	1.7567346	7	Singlet	Endothelial_cells
VAT_26M	1627.7	1032	0.6003106	3	Singlet	FAPs
VAT_26M	1755.5	1043	1.8966927	0	Singlet	Adipocyte
VAT_26M	2362.5	1346	0	0	Singlet	Adipocyte
VAT_26M	1153.6	823	2.2961701	5	Singlet	Immune_cells
VAT_26M	2044.2	1141	0.614692	0	Singlet	Adipocyte
VAT_26M	4228.7	2050	0.2500682	0	Singlet	Adipocyte
VAT_26M	1115.8	719	5.6165974	5	Singlet	Immune_cells
VAT_26M	1163	733	3.1198439	0	Singlet	Adipocyte
VAT_26M	3647.2	1879	0.0519352	0	Singlet	Adipocyte
VAT_26M	2271	1214	0.0164715	0	Singlet	Adipocyte
VAT_26M	1269.6	987	0.1949263	5	Singlet	Immune_cells
VAT_26M	1816.8	1120	0.0646676	0	Singlet	Adipocyte
VAT_26M	2955.7	1506	4.8700939	2	Singlet	FAPs
VAT_26M	1170.6	760	0.0630772	0	Singlet	Adipocyte
VAT_26M	1228.8	767	3.1615438	3	Singlet	FAPs
VAT_26M	1536	942	0.1073928	0	Singlet	Adipocyte
VAT_26M	1008.6	634	2.1449715	0	Singlet	Adipocyte
VAT_26M	4210.3	1997	0	0	Singlet	Adipocyte
VAT_26M	1111.2	753	1.0005333	7	Singlet	Endothelial_cells
VAT_26M	1263.7	914	1.310815	2	Singlet	FAPs
VAT_26M	1110.7	737	7.6516264	7	Singlet	Endothelial_cells
VAT_26M	2610	1385	1.0393256	0	Singlet	Adipocyte
VAT_26M	1250.8	791	2.9026682	3	Singlet	FAPs
VAT_26M	2827	1648	2.232846	2	Singlet	FAPs
VAT_26M	3350.6	1719	1.0487709	0	Singlet	Adipocyte
VAT_26M	1497.3	851	2.5300153	0	Singlet	Adipocyte
VAT_26M	1100.9	757	0.8937683	3	Singlet	FAPs
VAT_26M	1395.1	828	0.0380278	0	Singlet	Adipocyte
VAT_26M	2107.5	1216	0.5453852	0	Singlet	Adipocyte
VAT_26M	1264.6	872	1.8304685	5	Singlet	Immune_cells

VAT_26M	2355.7	1283	0.2541117	0	Singlet	Adipocyte
VAT_26M	2064.8	1264	1.7974575	7	Singlet	Endothelial_cells
VAT_26M	1183.4	782	0.1340807	0	Singlet	Adipocyte
VAT_26M	1387.4	809	11.004723	7	Singlet	Endothelial_cells
VAT_26M	2408.6	1340	0.0305836	0	Singlet	Adipocyte
VAT_26M	1075.3	730	1.3011958	3	Singlet	FAPs
VAT_26M	1617.7	1109	0.2204324	7	Singlet	Endothelial_cells
VAT_26M	1382.7	855	0.933444	0	Singlet	Adipocyte
VAT_26M	3376.6	1897	0.371955	0	Singlet	Adipocyte
VAT_26M	1369.4	875	0.5772534	0	Singlet	Adipocyte
VAT_26M	5610.1	2508	0.0032581	0	Singlet	Adipocyte
VAT_26M	1138.9	720	0.3447228	0	Singlet	Adipocyte
VAT_26M	1980.2	1169	1.7349262	0	Singlet	Adipocyte
VAT_26M	3620.4	1712	0.047678	0	Singlet	Adipocyte
VAT_26M	1008	710	0.2716544	5	Singlet	Immune_cells
VAT_26M	1105.6	767	0.3321643	2	Singlet	FAPs
VAT_26M	2791.1	1402	0	0	Singlet	Adipocyte
VAT_26M	1706	1033	3.0588083	3	Singlet	FAPs
VAT_26M	1190.3	794	3.885198	3	Singlet	FAPs
VAT_26M	1637.2	1053	1.3539525	2	Singlet	FAPs
VAT_26M	1472.7	927	0.3239403	0	Singlet	Adipocyte
VAT_26M	1463.3	879	3.5328592	3	Singlet	FAPs
VAT_26M	2283.7	1280	0	0	Singlet	Adipocyte
VAT_26M	1854.8	1063	4.4692045	0	Singlet	Adipocyte
VAT_26M	1067.6	812	0.3805262	5	Singlet	Immune_cells
VAT_26M	1921.5	1226	3.2252849	3	Singlet	FAPs
VAT_26M	1132.4	775	2.1230494	2	Singlet	FAPs
VAT_26M	1210.1	818	3.2170595	7	Singlet	Endothelial_cells
VAT_26M	2881.2	1566	4.1639616	0	Singlet	Adipocyte
VAT_26M	1813.8	1076	0.6393138	0	Singlet	Adipocyte
VAT_26M	2605.4	1436	2.987781	0	Singlet	Adipocyte
VAT_26M	2012.8	1252	0.016872	0	Singlet	Adipocyte
VAT_26M	2774.9	1528	0.0023701	0	Singlet	Adipocyte
VAT_26M	1218.2	828	0.455493	0	Singlet	Adipocyte
VAT_26M	1375.6	861	0.7016174	0	Singlet	Adipocyte
VAT_26M	4496.6	1998	0.2219767	0	Singlet	Adipocyte
VAT_26M	1107.3	776	1.5849467	3	Singlet	FAPs
VAT_26M	1715.9	946	0.2533497	0	Singlet	Adipocyte
VAT_26M	1120.3	711	11.529593	5	Singlet	Immune_cells
VAT_26M	1001.5	595	0.0264505	0	Singlet	Adipocyte
VAT_26M	2129.3	1285	0.9396513	0	Singlet	Adipocyte
VAT_26M	1469.8	929	8.1315147	3	Singlet	FAPs
VAT_26M	1505.8	888	0.0542427	0	Singlet	Adipocyte
VAT_26M	5726.6	2346	0.0022008	0	Singlet	Adipocyte
VAT_26M	1037.5	731	2.0647496	1	Singlet	Immune_cells
VAT_26M	2561.9	1355	0.124108	0	Singlet	Adipocyte
VAT_26M	1174.5	695	5.4740149	3	Singlet	FAPs
VAT_26M	1027.5	709	0.6610363	3	Singlet	FAPs
VAT_26M	2333.4	1286	0.1329775	0	Singlet	Adipocyte
VAT_26M	2696.7	1699	3.0155228	2	Singlet	FAPs
VAT_26M	1466	958	0.0823885	0	Singlet	Adipocyte

VAT_26M	3405	1776	0	0	Singlet	Adipocyte
VAT_26M	2585.7	1468	0	0	Singlet	Adipocyte
VAT_26M	6881	2754	0.00322	0	Singlet	Adipocyte
VAT_26M	6272.9	2455	4.1058015	0	Singlet	Adipocyte
VAT_26M	1763	1069	0.035196	0	Singlet	Adipocyte
VAT_26M	1890.4	1172	0.3641317	7	Singlet	Endothelial_cells
VAT_26M	1497.2	926	0.7963553	3	Singlet	FAPs
VAT_26M	1671.3	1005	0.5807672	0	Singlet	Adipocyte
VAT_26M	1294.8	795	0.7473141	0	Singlet	Adipocyte
VAT_26M	1213.7	810	0.6235289	0	Singlet	Adipocyte
VAT_26M	1956.1	1173	0.415602	0	Singlet	Adipocyte
VAT_26M	1056.4	758	0.1468798	11	Singlet	Smooth_muscle_cells
VAT_26M	1680.2	1041	0.270596	0	Singlet	Adipocyte
VAT_26M	1204.2	755	0.088225	6	Singlet	Neurons
VAT_26M	2229.4	1265	0.0150954	0	Singlet	Adipocyte
VAT_26M	1000.6	662	5.713422	5	Singlet	Immune_cells
VAT_26M	1324	850	2.1799327	0	Singlet	Adipocyte
VAT_26M	2674.5	1477	0.7725961	0	Singlet	Adipocyte
VAT_26M	1065.8	816	0.5372661	6	Singlet	Neurons
VAT_26M	1900.8	1088	1.0553685	0	Singlet	Adipocyte
VAT_26M	2372.7	1405	0.515661	3	Singlet	FAPs
VAT_26M	1341.7	813	0.2227428	0	Singlet	Adipocyte
VAT_26M	1503.3	914	3.0496121	0	Singlet	Adipocyte
VAT_26M	2356.6	1261	0.036432	0	Singlet	Adipocyte
VAT_26M	2780.3	1405	0.1430383	0	Singlet	Adipocyte
VAT_26M	2924.3	1501	0.0489565	0	Singlet	Adipocyte
VAT_26M	1612.2	978	0.9647542	0	Singlet	Adipocyte
VAT_26M	1019.9	638	3.6056547	5	Singlet	Immune_cells
VAT_26M	1014	664	0.1649301	0	Singlet	Adipocyte
VAT_26M	2061.7	1157	0.0036059	0	Singlet	Adipocyte
VAT_26M	1521.4	1057	0.7359574	3	Singlet	FAPs
VAT_26M	2182.1	1308	0.1958088	0	Singlet	Adipocyte
VAT_26M	1871	1104	0	0	Singlet	Adipocyte
VAT_26M	2887	1455	1.695066	0	Singlet	Adipocyte
VAT_26M	1323.4	837	0	0	Singlet	Adipocyte
VAT_26M	1283.2	875	7.5527414	3	Singlet	FAPs
VAT_26M	1929.8	1117	0.0066392	0	Singlet	Adipocyte
VAT_26M	2320.9	1403	0.5515091	0	Singlet	Adipocyte
VAT_26M	1142.7	722	0.3271942	0	Singlet	Adipocyte
VAT_26M	1697.2	1015	0.0255967	0	Singlet	Adipocyte
VAT_26M	2082.2	1251	3.3877852	3	Singlet	FAPs
VAT_26M	1025.9	713	0.8173903	3	Singlet	FAPs
VAT_26M	3832.1	1935	1.0541716	0	Singlet	Adipocyte
VAT_26M	1878.9	1154	1.9103231	0	Singlet	Adipocyte
VAT_26M	2385.2	1309	0.0092607	0	Singlet	Adipocyte
VAT_26M	3178	1782	1.7256382	3	Singlet	FAPs
VAT_26M	2266.6	1274	0.0568769	0	Singlet	Adipocyte
VAT_26M	1249.9	874	0.7231311	3	Singlet	FAPs
VAT_26M	2085.3	1154	0.01423	0	Singlet	Adipocyte
VAT_26M	1558.9	944	0.1806058	0	Singlet	Adipocyte
VAT_26M	1666.7	961	1.2735248	0	Singlet	Adipocyte

VAT_26M	2174.9	1245	0.7509683	0	Singlet	Adipocyte
VAT_26M	2014.4	1193	0.0169113	0	Singlet	Adipocyte
VAT_26M	1418.6	843	0.0934719	0	Singlet	Adipocyte
VAT_26M	1323.1	779	4.0342742	5	Singlet	Immune_cells
VAT_26M	1603.9	1029	0.6403254	3	Singlet	FAPs
VAT_26M	2520.1	1413	0.0658817	0	Singlet	Adipocyte
VAT_26M	1162.2	710	11.39743	3	Singlet	FAPs
VAT_26M	1380	872	0.0289495	0	Singlet	Adipocyte
VAT_26M	2406.3	1263	0.6861081	0	Singlet	Adipocyte
VAT_26M	1031.2	649	0.5108303	0	Singlet	Adipocyte
VAT_26M	4979.1	2234	0.0035365	0	Singlet	Adipocyte
VAT_26M	1130.8	739	1.0986856	0	Singlet	Adipocyte
VAT_26M	1279.6	817	0	0	Singlet	Adipocyte
VAT_26M	1130.1	750	0.3805238	0	Singlet	Adipocyte
VAT_26M	3342.7	1741	0.792431	0	Singlet	Adipocyte
VAT_26M	1249.1	806	0.0063484	0	Singlet	Adipocyte
VAT_26M	1017.6	723	0.3213805	5	Singlet	Immune_cells
VAT_26M	1375.9	844	0.7458339	0	Singlet	Adipocyte
VAT_26M	7717.4	2974	0	0	Singlet	Adipocyte
VAT_26M	2704.4	1453	0	0	Singlet	Adipocyte
VAT_26M	2083.7	1222	0	0	Singlet	Adipocyte
VAT_26M	1045.6	718	1.7864109	3	Singlet	FAPs
VAT_26M	1933.7	1233	2.1497967	7	Singlet	Endothelial_cells
VAT_26M	1104.1	723	4.3846695	5	Singlet	Immune_cells
VAT_26M	1024.6	670	0.0638463	0	Singlet	Adipocyte
VAT_26M	1298.6	817	0	0	Singlet	Adipocyte
VAT_26M	1492.4	911	0.0711349	0	Singlet	Adipocyte
VAT_26M	4561.9	2227	0	0	Singlet	Adipocyte
VAT_26M	2460.4	1381	0.4401797	0	Singlet	Adipocyte
VAT_26M	3065	1699	0.0239145	0	Singlet	Adipocyte
VAT_26M	1424.9	877	0.1226129	0	Singlet	Adipocyte
VAT_26M	1629.9	910	0.2776551	0	Singlet	Adipocyte
VAT_26M	1925	1127	0.028405	0	Singlet	Adipocyte
VAT_26M	2490.4	1405	0	0	Singlet	Adipocyte
VAT_26M	2900.5	1652	0.710838	0	Singlet	Adipocyte
VAT_26M	2234.2	1203	0.110937	0	Singlet	Adipocyte
VAT_26M	1118.1	725	0.3857099	0	Singlet	Adipocyte
VAT_26M	2936	1571	0	0	Singlet	Adipocyte
VAT_26M	1281.4	779	0.9759622	0	Singlet	Adipocyte
VAT_26M	1433	921	0.1056155	0	Singlet	Adipocyte
VAT_26M	1011.6	678	0.2386313	0	Singlet	Adipocyte
VAT_26M	2099.3	1246	0.0418768	0	Singlet	Adipocyte
VAT_26M	1184.8	740	0	0	Singlet	Adipocyte
VAT_26M	1327.5	921	1.3382997	2	Singlet	FAPs
VAT_26M	3575.3	1740	0.0220643	0	Singlet	Adipocyte
VAT_26M	1391.5	855	0.208605	0	Singlet	Adipocyte
VAT_26M	2753.1	1493	0.0027221	0	Singlet	Adipocyte
VAT_26M	1306.9	810	0	0	Singlet	Adipocyte
VAT_26M	1598.6	1072	2.9569321	3	Singlet	FAPs
VAT_26M	2273.7	1250	0.2877633	0	Singlet	Adipocyte
VAT_26M	2952.4	1573	0.1913585	0	Singlet	Adipocyte

VAT_26M	1578.1	958	4.569975	0	Singlet	Adipocyte
VAT_26M	2308.3	1326	0.1663212	0	Singlet	Adipocyte
VAT_26M	1557.9	966	0.0614995	0	Singlet	Adipocyte
VAT_26M	1275.1	843	0.4614799	5	Singlet	Immune_cells
VAT_26M	6052.2	2483	0	0	Singlet	Adipocyte
VAT_26M	1361.6	804	0.433452	0	Singlet	Adipocyte
VAT_26M	1855.1	1057	1.4574732	0	Singlet	Adipocyte
VAT_26M	1595.6	929	0.0766421	0	Singlet	Adipocyte
VAT_26M	2577.9	1314	0	0	Singlet	Adipocyte
VAT_26M	3881.6	1983	0.0934999	0	Singlet	Adipocyte
VAT_26M	1040.4	716	0.3720748	0	Singlet	Adipocyte
VAT_26M	3034.8	1511	0.0168912	0	Singlet	Adipocyte
VAT_26M	1509.8	927	6.2180704	3	Singlet	FAPs
VAT_26M	1028.5	749	0.0079923	5	Singlet	Immune_cells
VAT_26M	3255	1696	0.0619635	0	Singlet	Adipocyte
VAT_26M	1414.4	906	0.1211479	0	Singlet	Adipocyte
VAT_26M	1118.8	698	7.0872819	2	Singlet	FAPs
VAT_26M	3092.3	1593	1.0475385	0	Singlet	Adipocyte
VAT_26M	1062.5	750	0.5877578	2	Singlet	FAPs
VAT_26M	1981	1250	3.5121706	3	Singlet	FAPs
VAT_26M	3493.5	1786	0.1372168	0	Singlet	Adipocyte
VAT_26M	5827.6	2414	0.115279	0	Singlet	Adipocyte
VAT_26M	2044	1164	0.1502037	0	Singlet	Adipocyte
VAT_26M	1789	1058	0.1273802	0	Singlet	Adipocyte
VAT_26M	3059.6	1609	1.5243923	0	Singlet	Adipocyte
VAT_26M	1048.1	664	0	0	Singlet	Adipocyte
VAT_26M	9915.7	3472	0	0	Singlet	Adipocyte
VAT_26M	3280.2	1750	0.2938207	0	Singlet	Adipocyte
VAT_26M	1033	646	1.0954254	0	Singlet	Adipocyte
VAT_26M	1936.6	1099	0	0	Singlet	Adipocyte
VAT_26M	1259.8	780	0.1497296	0	Singlet	Adipocyte
VAT_26M	2622.6	1513	0.1222844	0	Singlet	Adipocyte
VAT_26M	1435.7	869	0.167496	0	Singlet	Adipocyte
VAT_26M	1067.1	728	0.454003	2	Singlet	FAPs
VAT_26M	1406.9	839	0.4404411	0	Singlet	Adipocyte
VAT_26M	2416.5	1348	0.3475702	0	Singlet	Adipocyte
VAT_26M	1162.6	750	0.4958834	0	Singlet	Adipocyte
VAT_26M	4780.6	2161	0.2786572	0	Singlet	Adipocyte
VAT_26M	4808.2	2204	0	0	Singlet	Adipocyte
VAT_26M	1718.7	1012	0	0	Singlet	Adipocyte
VAT_26M	3071.6	1587	0.1981517	0	Singlet	Adipocyte
VAT_26M	1023.7	705	0.0637592	6	Singlet	Neurons
VAT_26M	5573.2	2420	0	0	Singlet	Adipocyte
VAT_26M	5407.9	2427	0	0	Singlet	Adipocyte
VAT_26M	1444.5	977	1.9166477	5	Singlet	Immune_cells
VAT_26M	2637.1	1613	0.1600473	3	Singlet	FAPs
VAT_26M	2348.3	1355	0.0410788	0	Singlet	Adipocyte
VAT_26M	5561.3	2471	0.0174876	0	Singlet	Adipocyte
VAT_26M	3636.4	1790	0	0	Singlet	Adipocyte
VAT_26M	1455.3	856	0.0355109	0	Singlet	Adipocyte
VAT_26M	1888.7	1124	0.0192762	0	Singlet	Adipocyte

VAT_26M	2179.5	1353	2.0032762	3	Singlet	FAPs
VAT_26M	1164.3	830	1.8073581	5	Singlet	Immune_cells
VAT_26M	1825.9	1296	0.8055132	4	Singlet	Immune_cells
VAT_26M	1168.4	698	0.624508	0	Singlet	Adipocyte
VAT_26M	1222.6	812	0.0080283	0	Singlet	Adipocyte
VAT_26M	1934.4	1144	2.6336826	3	Singlet	FAPs
VAT_26M	2017.4	1227	0.4791234	0	Singlet	Adipocyte
VAT_26M	1048.1	760	0	6	Singlet	Neurons
VAT_26M	1249.3	765	0.1267463	0	Singlet	Adipocyte
VAT_26M	3196.7	1580	9.4436048	0	Singlet	Adipocyte
VAT_26M	1521.5	936	0	0	Singlet	Adipocyte
VAT_26M	1585.8	927	0.2709919	0	Singlet	Adipocyte
VAT_26M	1247.9	847	2.8903593	2	Singlet	FAPs
VAT_26M	1512.1	916	0.03266	0	Singlet	Adipocyte
VAT_26M	4033.4	1892	3.2504663	0	Singlet	Adipocyte
VAT_26M	1061.8	712	3.0695151	3	Singlet	FAPs
VAT_26M	2367	1220	0.6106566	0	Singlet	Adipocyte
VAT_26M	2448.3	1419	1.1983978	0	Singlet	Adipocyte
VAT_26M	3570.9	1686	0	0	Singlet	Adipocyte
VAT_26M	1251.3	874	1.7467374	2	Singlet	FAPs
VAT_26M	3362.6	1675	0.2785246	0	Singlet	Adipocyte
VAT_26M	2311.6	1215	0	0	Singlet	Adipocyte
VAT_26M	1936.8	1148	0.0372777	0	Singlet	Adipocyte
VAT_26M	1224	733	4.1717571	5	Singlet	Immune_cells
VAT_26M	1083.9	747	2.0616917	5	Singlet	Immune_cells
VAT_26M	3421.5	1854	0.5811765	0	Singlet	Adipocyte
VAT_26M	2301.2	1302	0.4333181	0	Singlet	Adipocyte
VAT_26M	3013.9	1549	0.0366006	0	Singlet	Adipocyte
VAT_26M	1850.1	1155	0.6220246	0	Singlet	Adipocyte
VAT_26M	1575.1	942	0.2452109	0	Singlet	Adipocyte
VAT_26M	4057.5	2018	0.8293498	0	Singlet	Adipocyte
VAT_26M	2010.9	1158	0.1470024	0	Singlet	Adipocyte
VAT_26M	2733.7	1408	0.0013425	0	Singlet	Adipocyte
VAT_26M	1007	694	1.5331132	7	Singlet	Endothelial_cells
VAT_26M	1738.6	1026	0.0139696	0	Singlet	Adipocyte
VAT_26M	2850.8	1440	0	0	Singlet	Adipocyte
VAT_26M	1242.9	764	0.1532293	0	Singlet	Adipocyte
VAT_26M	3117.1	1569	0.0824889	0	Singlet	Adipocyte
VAT_26M	1483.9	905	0.1487138	0	Singlet	Adipocyte
VAT_26M	1092.3	739	0.6685235	0	Singlet	Adipocyte
VAT_26M	1462.4	881	0	0	Singlet	Adipocyte
VAT_26M	5857.3	2665	0.0328202	0	Singlet	Adipocyte
VAT_26M	1772.5	1020	0	0	Singlet	Adipocyte
VAT_26M	2138	1273	0.5237378	3	Singlet	FAPs
VAT_26M	6982.4	2849	0.1182048	0	Singlet	Adipocyte
VAT_26M	1950.7	1085	10.087502	5	Singlet	Immune_cells
VAT_26M	1368.7	914	1.3754916	2	Singlet	FAPs
VAT_26M	1265.6	860	0.287663	3	Singlet	FAPs
VAT_26M	4333.5	1904	3.7558492	0	Singlet	Adipocyte
VAT_26M	1182.3	773	0.2016977	0	Singlet	Adipocyte
VAT_26M	7638.3	2919	0.0159779	0	Singlet	Adipocyte

VAT_26M	2527.6	1279	0	0	Singlet	Adipocyte
VAT_26M	2531.3	1483	3.2149664	3	Singlet	FAPs
VAT_26M	2843.1	1571	0.0650202	0	Singlet	Adipocyte
VAT_26M	2167.7	1190	0.7834454	0	Singlet	Adipocyte
VAT_26M	2133.6	1249	0.1462449	0	Singlet	Adipocyte
VAT_26M	1494.6	929	1.0719606	0	Singlet	Adipocyte
VAT_26M	2418.2	1411	2.6127911	3	Singlet	FAPs
VAT_26M	2393	1289	0.0612254	0	Singlet	Adipocyte
VAT_26M	6582	2602	0.004673	0	Singlet	Adipocyte
VAT_26M	1124.1	707	0.1499897	0	Singlet	Adipocyte
VAT_26M	1750.1	994	0.1380491	0	Singlet	Adipocyte
VAT_26M	1506.6	964	0.2896546	0	Singlet	Adipocyte
VAT_26M	2297.3	1326	0.3850526	0	Singlet	Adipocyte
VAT_26M	3962.6	1923	0.3833255	0	Singlet	Adipocyte
VAT_26M	1281.6	866	0.5592895	7	Singlet	Endothelial_cells
VAT_26M	1070.1	737	3.3577025	2	Singlet	FAPs
VAT_26M	1051.5	642	2.2881148	0	Singlet	Adipocyte
VAT_26M	2661.7	1480	1.1238864	0	Singlet	Adipocyte
VAT_26M	2081.5	1190	0.6654103	0	Singlet	Adipocyte
VAT_26M	1440.3	914	0	0	Singlet	Adipocyte
VAT_26M	1516.5	897	0.0263456	0	Singlet	Adipocyte
VAT_26M	1472.6	1074	4.866492	7	Singlet	Endothelial_cells
VAT_26M	2146.3	1182	1.1445852	0	Singlet	Adipocyte
VAT_26M	1546.3	1024	0.5977713	3	Singlet	FAPs
VAT_26M	2437.1	1270	0.1673092	0	Singlet	Adipocyte
VAT_26M	1804.5	1059	0.0064747	0	Singlet	Adipocyte
VAT_26M	1499.6	995	0.35259	0	Singlet	Adipocyte
VAT_26M	3288.3	1658	1.4691042	0	Singlet	Adipocyte
VAT_26M	1752.5	1093	0.8420231	0	Singlet	Adipocyte
VAT_26M	1302.6	826	1.8576404	0	Singlet	Adipocyte
VAT_26M	2043.7	1178	0.0163449	0	Singlet	Adipocyte
VAT_26M	1252.3	768	7.2930464	7	Singlet	Endothelial_cells
VAT_26M	1445.2	955	0.1658638	0	Singlet	Adipocyte
VAT_26M	1506.4	973	0.0286724	3	Singlet	FAPs
VAT_26M	5534.6	2425	0.7875518	0	Singlet	Adipocyte
VAT_26M	1158.9	721	4.3328704	0	Singlet	Adipocyte
VAT_26M	1390.4	837	0.3817977	0	Singlet	Adipocyte
VAT_26M	2146.3	1167	0.0384684	0	Singlet	Adipocyte
VAT_26M	7406.4	2884	2.3488462	0	Singlet	Adipocyte
VAT_26M	1047.9	700	1.0584675	3	Singlet	FAPs
VAT_26M	5782.2	2611	0	0	Singlet	Adipocyte
VAT_26M	1475	905	0.195123	0	Singlet	Adipocyte
VAT_26M	1375.8	835	11.430595	3	Singlet	FAPs
VAT_26M	1433	1002	1.1551851	5	Singlet	Immune_cells
VAT_26M	1607.2	983	0.1292519	0	Singlet	Adipocyte
VAT_26M	2864.5	1465	0.021009	0	Singlet	Adipocyte
VAT_26M	3800.4	1874	0.7025771	0	Singlet	Adipocyte
VAT_26M	1557.2	997	0.0373358	11	Singlet	Smooth_muscle_cells
VAT_26M	1158.4	769	0.2269439	3	Singlet	FAPs
VAT_26M	2992.7	1607	0.0581616	0	Singlet	Adipocyte
VAT_26M	1202.8	835	6.4316641	5	Singlet	Immune_cells

VAT_26M	3512.2	1693	0.9417218	0	Singlet	Adipocyte
VAT_26M	2546	1468	4.7426281	2	Singlet	FAPs
VAT_26M	1159.3	758	0.4267754	3	Singlet	FAPs
VAT_26M	4198.3	1983	0	0	Singlet	Adipocyte
VAT_26M	1015.7	681	0.0135688	3	Singlet	FAPs
VAT_26M	3117.8	1542	9.3940662	0	Singlet	Adipocyte
VAT_26M	3013.9	1537	0.2896351	0	Singlet	Adipocyte
VAT_26M	3339	1959	0.1316197	0	Singlet	Adipocyte
VAT_26M	5202.2	2222	0	0	Singlet	Adipocyte
VAT_26M	1727.4	1013	0.150487	0	Singlet	Adipocyte
VAT_26M	3278	1734	0.0717784	0	Singlet	Adipocyte
VAT_26M	1043.6	670	0	0	Singlet	Adipocyte
VAT_26M	3875.5	1970	0.4340691	0	Singlet	Adipocyte
VAT_26M	2278	1441	1.2568434	3	Singlet	FAPs
VAT_26M	5129.6	2381	0.0128382	0	Singlet	Adipocyte
VAT_26M	1335.3	950	2.5321471	1	Singlet	Immune_cells
VAT_26M	2608.3	1419	0.0396435	0	Singlet	Adipocyte
VAT_26M	1941.9	1269	2.7071248	2	Singlet	FAPs
VAT_26M	2240.1	1176	0.0207152	0	Singlet	Adipocyte
VAT_26M	1406.6	868	0	0	Singlet	Adipocyte
VAT_26M	1833.3	1141	2.3704394	3	Singlet	FAPs
VAT_26M	1335.5	806	2.1593367	0	Singlet	Adipocyte
VAT_26M	2642.2	1429	0.0227308	0	Singlet	Adipocyte
VAT_26M	1399.6	835	0.163258	0	Singlet	Adipocyte
VAT_26M	1766.5	1078	2.8249405	7	Singlet	Endothelial_cells
VAT_26M	1472.2	978	0.503516	2	Singlet	FAPs
VAT_26M	2343	1377	0.0143632	0	Singlet	Adipocyte
VAT_26M	2515.3	1325	0.0115409	0	Singlet	Adipocyte
VAT_26M	3047.2	1559	0.0866367	0	Singlet	Adipocyte
VAT_26M	2997.7	1538	0.090957	0	Singlet	Adipocyte
VAT_26M	1061.9	704	0.2122785	3	Singlet	FAPs
VAT_26M	2242.4	1271	2.9972105	0	Singlet	Adipocyte
VAT_26M	1656.6	1003	0.1044336	0	Singlet	Adipocyte
VAT_26M	1183.9	746	4.3778692	0	Singlet	Adipocyte
VAT_26M	1533.2	963	4.7216145	3	Singlet	FAPs
VAT_26M	2758.2	1479	2.7360792	0	Singlet	Adipocyte
VAT_26M	1345.4	881	0.1152001	0	Singlet	Adipocyte
VAT_26M	1348.7	841	6.5711036	7	Singlet	Endothelial_cells
VAT_26M	2165.6	1189	0.5692486	0	Singlet	Adipocyte
VAT_26M	2218.3	1263	0.1182736	0	Singlet	Adipocyte
VAT_26M	1725.5	1153	0.4453565	2	Singlet	FAPs
VAT_26M	1446.8	944	3.6718495	9	Singlet	Mesothelial
VAT_26M	1288.6	867	1.6039747	3	Singlet	FAPs
VAT_26M	1060.7	775	0.971947	3	Singlet	FAPs
VAT_26M	1044.2	616	1.1375132	0	Singlet	Adipocyte
VAT_26M	1319.1	899	0.5483721	2	Singlet	FAPs
VAT_26M	1055.6	697	0.2409051	0	Singlet	Adipocyte
VAT_26M	1331.5	830	0	0	Singlet	Adipocyte
VAT_26M	1404.2	885	0	3	Singlet	FAPs
VAT_26M	1856.1	1032	4.63072	3	Singlet	FAPs
VAT_26M	1344.6	789	0.4432139	0	Singlet	Adipocyte

VAT_26M	1154.4	781	1.1685176	0	Singlet	Adipocyte
VAT_26M	2101.9	1222	0	0	Singlet	Adipocyte
VAT_26M	3429.7	1766	0	0	Singlet	Adipocyte
VAT_26M	1558.5	932	1.2795668	0	Singlet	Adipocyte
VAT_26M	1121.9	725	0.3958415	0	Singlet	Adipocyte
VAT_26M	1369.2	844	0.4245732	0	Singlet	Adipocyte
VAT_26M	1439.1	942	0.0366774	0	Singlet	Adipocyte
VAT_26M	1197.7	784	1.2448752	3	Singlet	FAPs
VAT_26M	1871.1	1127	6.0798656	3	Singlet	FAPs
VAT_26M	1335.9	842	12.224759	9	Singlet	Mesothelial
VAT_26M	1396.3	927	1.4686734	0	Singlet	Adipocyte
VAT_26M	1203	833	0.0077499	0	Singlet	Adipocyte
VAT_26M	1395.4	891	0	0	Singlet	Adipocyte
VAT_26M	1115.8	720	0	0	Singlet	Adipocyte
VAT_26M	1014.9	722	0.9257141	2	Singlet	FAPs
VAT_26M	1211.4	708	0	0	Singlet	Adipocyte
VAT_26M	1696.3	1046	1.0881298	0	Singlet	Adipocyte
VAT_26M	3139.2	1580	0.0466331	0	Singlet	Adipocyte
VAT_26M	2038.6	1159	0.0246634	0	Singlet	Adipocyte
VAT_26M	1421.5	925	4.6709205	3	Singlet	FAPs
VAT_26M	2324.6	1257	3.5365365	3	Singlet	FAPs
VAT_26M	1490.9	927	0.5235556	0	Singlet	Adipocyte
VAT_26M	1002.8	658	0.4041682	0	Singlet	Adipocyte
VAT_26M	1791	1056	0.0179384	0	Singlet	Adipocyte
VAT_26M	1408.1	893	0	0	Singlet	Adipocyte
VAT_26M	1062	751	0.8347284	3	Singlet	FAPs
VAT_26M	2034.7	1222	0.0098477	0	Singlet	Adipocyte
VAT_26M	1427.9	872	0.47881	0	Singlet	Adipocyte
VAT_26M	1687.5	1138	0.8383198	0	Singlet	Adipocyte
VAT_26M	1904.2	1139	0.0170762	0	Singlet	Adipocyte
VAT_26M	1095.1	706	0.0777413	0	Singlet	Adipocyte
VAT_26M	1718.2	1011	0.1526532	0	Singlet	Adipocyte
VAT_26M	1803	1086	0.4070914	0	Singlet	Adipocyte
VAT_26M	2637.2	1400	0.0420989	0	Singlet	Adipocyte
VAT_26M	1449	914	0	0	Singlet	Adipocyte
VAT_26M	3138.3	1593	0	0	Singlet	Adipocyte
VAT_26M	1082.3	700	0.3437326	0	Singlet	Adipocyte
VAT_26M	1743.9	1027	0.0238263	0	Singlet	Adipocyte
VAT_26M	1142.3	730	3.2122217	3	Singlet	FAPs
VAT_26M	2699.8	1463	0.1121582	0	Singlet	Adipocyte
VAT_26M	1495.8	1019	0.5225965	0	Singlet	Adipocyte
VAT_26M	1215.7	769	0.9575179	0	Singlet	Adipocyte
VAT_26M	1617.4	1048	2.935602	2	Singlet	FAPs
VAT_26M	2854.1	1483	0.1054849	0	Singlet	Adipocyte
VAT_26M	1169	713	0.0114315	0	Singlet	Adipocyte
VAT_26M	3930.9	1889	1.145202	0	Singlet	Adipocyte
VAT_26M	1265.6	873	3.7864028	5	Singlet	Immune_cells
VAT_26M	2163	1267	0.3481422	0	Singlet	Adipocyte
VAT_26M	1281.1	812	0.0447193	0	Singlet	Adipocyte
VAT_26M	1101	700	0.0741771	0	Singlet	Adipocyte
VAT_26M	4107.1	1891	0.8598782	0	Singlet	Adipocyte

VAT_26M	1191.9	731	0.811271	0	Singlet	Adipocyte
VAT_26M	2301.5	1349	0	0	Singlet	Adipocyte
VAT_26M	1593.3	967	0	0	Singlet	Adipocyte
VAT_26M	1665.5	983	1.855921	3	Singlet	FAPs
VAT_26M	1299.2	814	0.2780133	0	Singlet	Adipocyte
VAT_26M	2426	1365	0.1020689	0	Singlet	Adipocyte
VAT_26M	1195.1	822	1.9575512	3	Singlet	FAPs
VAT_26M	1882.8	1098	0.0892632	0	Singlet	Adipocyte
VAT_26M	2406.2	1334	0.0391428	0	Singlet	Adipocyte
VAT_26M	1341.2	873	1.1276461	7	Singlet	Endothelial_cells
VAT_26M	2729	1575	0.0078146	0	Singlet	Adipocyte
VAT_26M	1273.4	881	0.0689008	0	Singlet	Adipocyte
VAT_26M	1156.7	752	4.6557158	0	Singlet	Adipocyte
VAT_26M	1774.3	1025	1.1701945	0	Singlet	Adipocyte
VAT_26M	1444.8	891	0.1229826	0	Singlet	Adipocyte
VAT_26M	1566.1	1038	1.2211047	0	Singlet	Adipocyte
VAT_26M	1474.5	989	0.1762608	0	Singlet	Adipocyte
VAT_26M	7251.4	3023	0	0	Singlet	Adipocyte
VAT_26M	1134.5	699	0.5349185	0	Singlet	Adipocyte
VAT_26M	3468.1	1747	0.0640199	0	Singlet	Adipocyte
VAT_26M	1226.5	785	0.2217683	0	Singlet	Adipocyte
VAT_26M	1394.6	849	0.6664688	0	Singlet	Adipocyte
VAT_26M	2615.6	1475	0.6934447	0	Singlet	Adipocyte
VAT_26M	1107.5	741	3.3437474	3	Singlet	FAPs
VAT_26M	5702.9	2514	0.2585424	0	Singlet	Adipocyte
VAT_26M	1895.3	1149	1.6811279	0	Singlet	Adipocyte
VAT_26M	1309.5	863	0	0	Singlet	Adipocyte
VAT_26M	1297.1	822	0	0	Singlet	Adipocyte
VAT_26M	2267.1	1309	3.8491351	0	Singlet	Adipocyte
VAT_26M	1136.3	748	0.0146085	0	Singlet	Adipocyte
VAT_26M	1032.2	700	2.4064892	3	Singlet	FAPs
VAT_26M	1039.4	813	0.35828	4	Singlet	Immune_cells
VAT_26M	2100.1	1194	0.2064973	0	Singlet	Adipocyte
VAT_26M	1588.9	895	5.3129011	0	Singlet	Adipocyte
VAT_26M	5321.4	2307	0	0	Singlet	Adipocyte
VAT_26M	5332.9	2392	2.4186232	0	Singlet	Adipocyte
VAT_26M	1648	1106	2.4043865	3	Singlet	FAPs
VAT_26M	1360.2	908	4.5388691	3	Singlet	FAPs
VAT_26M	2050.7	1175	0.1875843	0	Singlet	Adipocyte
VAT_26M	2667.7	1482	0.2366351	0	Singlet	Adipocyte
VAT_26M	2683.4	1421	0.0520171	0	Singlet	Adipocyte
VAT_26M	1582.8	1033	9.3324491	9	Singlet	Mesothelial
VAT_26M	1570.2	918	0.0822278	0	Singlet	Adipocyte
VAT_26M	2061.2	1182	0.0811224	0	Singlet	Adipocyte
VAT_26M	9202.2	3206	0.1805858	0	Singlet	Adipocyte
VAT_26M	1302.9	893	1.2292598	0	Singlet	Adipocyte
VAT_26M	1524.2	1032	0.3340371	0	Singlet	Adipocyte
VAT_26M	1537.9	983	0.1235738	0	Singlet	Adipocyte
VAT_26M	3268.4	1693	0.271698	0	Singlet	Adipocyte
VAT_26M	1857.3	1059	0.0076168	0	Singlet	Adipocyte
VAT_26M	3111.9	1650	0.001981	0	Singlet	Adipocyte

VAT_26M	2050.1	1171	0.4093198	0	Singlet	Adipocyte
VAT_26M	2600	1618	5.6792728	2	Singlet	FAPs
VAT_26M	1297.1	832	1.1520778	0	Singlet	Adipocyte
VAT_26M	3101.5	1638	0.0332815	0	Singlet	Adipocyte
VAT_26M	2344.3	1342	0.535334	0	Singlet	Adipocyte
VAT_26M	2770.5	1561	0	0	Singlet	Adipocyte
VAT_26M	1045.4	701	2.321959	5	Singlet	Immune_cells
VAT_26M	4680.5	2213	0.0575295	0	Singlet	Adipocyte
VAT_26M	1129.6	774	5.9120566	9	Singlet	Mesothelial
VAT_26M	1869.1	1169	3.2480719	0	Singlet	Adipocyte
VAT_26M	1476.4	909	0.5082397	0	Singlet	Adipocyte
VAT_26M	1010.5	679	0.0243567	0	Singlet	Adipocyte
VAT_26M	1025.4	710	0.172547	2	Singlet	FAPs
VAT_26M	1412.7	967	0.6144379	2	Singlet	FAPs
VAT_26M	1827.4	1096	0.2842008	0	Singlet	Adipocyte
VAT_26M	2110.4	1328	3.2128104	2	Singlet	FAPs
VAT_26M	2232.2	1302	0	0	Singlet	Adipocyte
VAT_26M	4737.4	2147	0.0160318	0	Singlet	Adipocyte
VAT_26M	2988.1	1622	14.793926	7	Singlet	Endothelial_cells
VAT_26M	1232.2	756	0	0	Singlet	Adipocyte
VAT_26M	1531.4	990	0.6399834	0	Singlet	Adipocyte
VAT_26M	1267.1	847	0.3228053	0	Singlet	Adipocyte
VAT_26M	2050.4	1131	0.0086624	0	Singlet	Adipocyte
VAT_26M	1803.9	1088	0.2684364	0	Singlet	Adipocyte
VAT_26M	1158.3	783	2.7824122	3	Singlet	FAPs
VAT_26M	2336.8	1363	0.6689526	0	Singlet	Adipocyte
VAT_26M	2092.2	1178	1.1606245	0	Singlet	Adipocyte
VAT_26M	1008.8	711	0.1337936	3	Singlet	FAPs
VAT_26M	1982.9	1090	0	0	Singlet	Adipocyte
VAT_26M	1569.5	914	6.5278537	3	Singlet	FAPs
VAT_26M	1381.3	912	0.0556616	0	Singlet	Adipocyte
VAT_26M	2321	1295	5.4901597	7	Singlet	Endothelial_cells
VAT_26M	2816.7	1496	0.0336188	0	Singlet	Adipocyte
VAT_26M	4971.1	2228	0	0	Singlet	Adipocyte
VAT_26M	1251.7	806	0	0	Singlet	Adipocyte
VAT_26M	1086.5	701	0.4933538	0	Singlet	Adipocyte
VAT_26M	2115.7	1197	0	0	Singlet	Adipocyte
VAT_26M	3773.1	1890	0.0171754	0	Singlet	Adipocyte
VAT_26M	1896.5	1110	1.9187427	3	Singlet	FAPs
VAT_26M	1131.9	753	0.3306795	3	Singlet	FAPs
VAT_26M	2302.3	1331	1.4529993	0	Singlet	Adipocyte
VAT_26M	1391.5	908	0.4624449	0	Singlet	Adipocyte
VAT_26M	4179.5	2009	0	0	Singlet	Adipocyte
VAT_26M	2710.7	1586	0.0096481	0	Singlet	Adipocyte
VAT_26M	5342.1	2356	0.5924949	0	Singlet	Adipocyte
VAT_26M	2391.6	1297	0	0	Singlet	Adipocyte
VAT_26M	3613.7	1846	0	0	Singlet	Adipocyte
VAT_26M	1714.9	1101	1.0903674	2	Singlet	FAPs
VAT_26M	2604.4	1472	0.0065348	0	Singlet	Adipocyte
VAT_26M	1504.6	908	2.4678549	0	Singlet	Adipocyte
VAT_26M	2009.8	1195	0.0223515	0	Singlet	Adipocyte

VAT_26M	1188.5	793	0.114896	3	Singlet	FAPs
VAT_26M	3843.6	1893	0.4217696	0	Singlet	Adipocyte
VAT_26M	1272.7	780	0.4874578	0	Singlet	Adipocyte
VAT_26M	2798.3	1515	0	0	Singlet	Adipocyte
VAT_26M	1298.4	909	1.5860661	2	Singlet	FAPs
VAT_26M	2450.7	1503	6.4363082	2	Singlet	FAPs
VAT_26M	2301.4	1403	0.1902471	0	Singlet	Adipocyte
VAT_26M	1121.4	653	0.1617971	0	Singlet	Adipocyte
VAT_26M	1555.9	889	13.873839	5	Singlet	Immune_cells
VAT_26M	1206.6	749	0.0879077	0	Singlet	Adipocyte
VAT_26M	1399	861	1.4904417	3	Singlet	FAPs
VAT_26M	2340	1289	0.1031144	0	Singlet	Adipocyte
VAT_26M	1199.6	765	3.5513189	3	Singlet	FAPs
VAT_26M	1417.8	928	0.50832	3	Singlet	FAPs
VAT_26M	2399.6	1376	1.6551012	0	Singlet	Adipocyte
VAT_26M	2170.3	1242	0.676975	0	Singlet	Adipocyte
VAT_26M	1062.5	719	0.4883063	2	Singlet	FAPs
VAT_26M	1079.5	676	4.1005272	3	Singlet	FAPs
VAT_26M	1994.6	1144	0.5227396	0	Singlet	Adipocyte
VAT_26M	1183.7	780	0.0112774	0	Singlet	Adipocyte
VAT_26M	1807.3	1049	0.1106916	0	Singlet	Adipocyte
VAT_26M	1412.5	857	0.8726556	0	Singlet	Adipocyte
VAT_26M	1257.4	800	3.7051034	0	Singlet	Adipocyte
VAT_26M	1074.2	767	1.2174223	3	Singlet	FAPs
VAT_26M	1962.1	1181	0.0328031	0	Singlet	Adipocyte
VAT_26M	1301.5	765	2.1956244	0	Singlet	Adipocyte
VAT_26M	2397	1365	0	0	Singlet	Adipocyte
VAT_26M	2418.8	1280	0.0764223	0	Singlet	Adipocyte
VAT_26M	1393.6	893	0.0988417	0	Singlet	Adipocyte
VAT_26M	1532.6	923	2.306652	0	Singlet	Adipocyte
VAT_26M	2493.9	1406	0.3246469	0	Singlet	Adipocyte
VAT_26M	1310.7	822	0.0753757	0	Singlet	Adipocyte
VAT_26M	3411.3	1705	0.207761	0	Singlet	Adipocyte
VAT_26M	1547.7	950	0.8703181	0	Singlet	Adipocyte
VAT_26M	1091	758	4.2354334	7	Singlet	Endothelial_cells
VAT_26M	2756.4	1588	4.1478575	3	Singlet	FAPs
VAT_26M	1282.1	809	0	0	Singlet	Adipocyte
VAT_26M	4314.5	1964	1.3321306	0	Singlet	Adipocyte
VAT_26M	1869.5	1165	0.1695156	0	Singlet	Adipocyte
VAT_26M	1157.6	765	0.3985026	0	Singlet	Adipocyte
VAT_26M	1143.1	700	0.4623734	6	Singlet	Neurons
VAT_26M	1570	985	0.6196162	0	Singlet	Adipocyte
VAT_26M	1557.6	898	0.6941502	0	Singlet	Adipocyte
VAT_26M	1205.4	816	3.8145874	7	Singlet	Endothelial_cells
VAT_26M	4247.2	2044	0	0	Singlet	Adipocyte
VAT_26M	1034.9	702	0.3706158	0	Singlet	Adipocyte
VAT_26M	1139.2	782	6.7066043	9	Singlet	Mesothelial
VAT_26M	1177.1	805	1.2450588	7	Singlet	Endothelial_cells
VAT_26M	2246.9	1313	1.1301262	0	Singlet	Adipocyte
VAT_26M	1506	882	0	0	Singlet	Adipocyte
VAT_26M	1882	1099	0.2508825	0	Singlet	Adipocyte

VAT_26M	1908.4	1096	0.9822545	0	Singlet	Adipocyte
VAT_26M	1182.3	776	0.1783297	0	Singlet	Adipocyte
VAT_26M	2541.6	1327	1.9675788	0	Singlet	Adipocyte
VAT_26M	1761.1	1006	0.0495872	0	Singlet	Adipocyte
VAT_26M	2782.8	1729	2.1262513	2	Singlet	FAPs
VAT_26M	2817	1501	0.0015814	0	Singlet	Adipocyte
VAT_26M	3918.7	1819	0	0	Singlet	Adipocyte
VAT_26M	1504.2	900	0.6833191	0	Singlet	Adipocyte
VAT_26M	2509.7	1315	0	0	Singlet	Adipocyte
VAT_26M	2932.8	1486	3.4742576	0	Singlet	Adipocyte
VAT_26M	1336.7	848	0	0	Singlet	Adipocyte
VAT_26M	1973.5	1235	2.0555408	0	Singlet	Adipocyte
VAT_26M	2985.5	1785	4.5267137	2	Singlet	FAPs
VAT_26M	1283.6	799	14.276306	9	Singlet	Mesothelial
VAT_26M	1955.5	1275	0.0784304	3	Singlet	FAPs
VAT_26M	1107.2	687	0	0	Singlet	Adipocyte
VAT_26M	4483.3	2064	0.077655	0	Singlet	Adipocyte
VAT_26M	2206.1	1324	0.3441015	0	Singlet	Adipocyte
VAT_26M	2747.2	1450	0.8399319	0	Singlet	Adipocyte
VAT_26M	1182.3	808	0.0102204	0	Singlet	Adipocyte
VAT_26M	1944	1117	0.2547812	0	Singlet	Adipocyte
VAT_26M	5329.4	2489	0	0	Singlet	Adipocyte
VAT_26M	4808.9	2171	0.7357597	0	Singlet	Adipocyte
VAT_26M	1083.4	762	0.1613725	2	Singlet	FAPs
VAT_26M	1352.7	855	2.4466624	3	Singlet	FAPs
VAT_26M	1125	786	0.4601167	1	Singlet	Immune_cells
VAT_26M	1010.4	708	2.1158779	2	Singlet	FAPs
VAT_26M	1263.8	771	0.3930685	0	Singlet	Adipocyte
VAT_26M	1412.4	891	0.609026	5	Singlet	Immune_cells
VAT_26M	1687.1	1012	0.1277843	0	Singlet	Adipocyte
VAT_26M	1157	759	4.1241989	3	Singlet	FAPs
VAT_26M	1796.7	1041	0.10421	0	Singlet	Adipocyte
VAT_26M	1963.6	1153	3.534588	3	Singlet	FAPs
VAT_26M	3975.2	2079	0.2122298	0	Singlet	Adipocyte
VAT_26M	3281.7	1694	0.4326391	0	Singlet	Adipocyte
VAT_26M	3021.8	1647	0.1373782	0	Singlet	Adipocyte
VAT_26M	1210.5	787	0.4477459	0	Singlet	Adipocyte
VAT_26M	1215.3	855	0.0386493	5	Singlet	Immune_cells
VAT_26M	1099.7	714	0.2142617	0	Singlet	Adipocyte
VAT_26M	2220.1	1331	0.0160715	0	Singlet	Adipocyte
VAT_26M	1834.7	1026	1.6525959	3	Singlet	FAPs
VAT_26M	1229.3	808	0.9455482	3	Singlet	FAPs
VAT_26M	1003.7	693	0.2870217	5	Singlet	Immune_cells
VAT_26M	4810.6	2147	1.0596496	0	Singlet	Adipocyte
VAT_26M	1761	1047	0.1506593	0	Singlet	Adipocyte
VAT_26M	1212.1	822	3.7783161	5	Singlet	Immune_cells
VAT_26M	2376.1	1284	0	0	Singlet	Adipocyte
VAT_26M	5090.1	2218	0	0	Singlet	Adipocyte
VAT_26M	2345	1391	0	0	Singlet	Adipocyte
VAT_26M	2152.1	1210	0.1213452	0	Singlet	Adipocyte
VAT_26M	2860.2	1475	0	0	Singlet	Adipocyte

VAT_26M	1398	820	0.0368768	0	Singlet	Adipocyte
VAT_26M	1665.4	1013	0.0166957	0	Singlet	Adipocyte
VAT_26M	2012.2	1154	0	0	Singlet	Adipocyte
VAT_26M	2276.3	1254	0.007894	0	Singlet	Adipocyte
VAT_26M	1253.6	792	0.3182438	0	Singlet	Adipocyte
VAT_26M	1118.4	756	0.2164264	5	Singlet	Immune_cells
VAT_26M	1988	1275	0.8487345	2	Singlet	FAPs
VAT_26M	6982	2648	0	0	Singlet	Adipocyte
VAT_26M	3219.6	1524	0.0915147	0	Singlet	Adipocyte
VAT_26M	2359.5	1325	0.2604629	0	Singlet	Adipocyte
VAT_26M	1057.9	698	0.7034658	0	Singlet	Adipocyte
VAT_26M	1038.9	676	0.4582414	0	Singlet	Adipocyte
VAT_26M	1219.3	775	0.0387138	0	Singlet	Adipocyte
VAT_26M	3564.4	1858	0.0068818	0	Singlet	Adipocyte
VAT_26M	4962	2304	0.0751259	0	Singlet	Adipocyte
VAT_26M	4833.5	2217	2.2939763	0	Singlet	Adipocyte
VAT_26M	1169.8	724	0.1506138	3	Singlet	FAPs
VAT_26M	2002.9	1146	0.789786	0	Singlet	Adipocyte
VAT_26M	1283.5	781	0.0457896	0	Singlet	Adipocyte
VAT_26M	1253.5	809	10.249587	3	Singlet	FAPs
VAT_26M	3082.9	1756	0.5747891	0	Singlet	Adipocyte
VAT_26M	1240.5	772	0.21488	0	Singlet	Adipocyte
VAT_26M	2713.4	1369	0.9484354	0	Singlet	Adipocyte
VAT_26M	1059.7	702	0.7171343	7	Singlet	Endothelial_cells
VAT_26M	1821.4	1069	0.3879082	0	Singlet	Adipocyte
VAT_26M	3375.9	1802	0.6411901	0	Singlet	Adipocyte
VAT_26M	1259.7	823	0.2174886	0	Singlet	Adipocyte
VAT_26M	2184.1	1240	0.0074641	0	Singlet	Adipocyte
VAT_26M	1235.9	807	0.3795398	0	Singlet	Adipocyte
VAT_26M	4354.5	2313	0.0249229	2	Singlet	FAPs
VAT_26M	4743	2104	0.3516952	0	Singlet	Adipocyte
VAT_26M	3669	1769	1.7264569	0	Singlet	Adipocyte
VAT_26M	1892.7	1176	0.1420103	0	Singlet	Adipocyte
VAT_26M	1002.7	697	1.4166871	3	Singlet	FAPs
VAT_26M	1223.4	845	0.72659	3	Singlet	FAPs
VAT_26M	1311.2	790	8.0362124	7	Singlet	Endothelial_cells
VAT_26M	1098.8	691	0.6861334	0	Singlet	Adipocyte
VAT_26M	4344	2114	0.1196443	0	Singlet	Adipocyte
VAT_26M	1054.7	667	0.1153167	0	Singlet	Adipocyte
VAT_26M	1362.1	887	0.8325138	0	Singlet	Adipocyte
VAT_26M	1128.6	683	1.2613843	6	Singlet	Neurons
VAT_26M	2344.5	1371	0.4575455	2	Singlet	FAPs
VAT_26M	3680.8	1772	0.0383885	0	Singlet	Adipocyte
VAT_26M	1670.9	969	0.0717112	0	Singlet	Adipocyte
VAT_26M	1199.2	846	0.3114569	0	Singlet	Adipocyte
VAT_26M	1479.1	877	10.293911	3	Singlet	FAPs
VAT_26M	4804.6	2167	0.7587958	0	Singlet	Adipocyte
VAT_26M	1117.1	731	0.0265764	3	Singlet	FAPs
VAT_26M	1205.3	738	0.0387423	0	Singlet	Adipocyte
VAT_26M	1349	898	2.5268393	3	Singlet	FAPs
VAT_26M	1536	940	3.9908717	3	Singlet	FAPs

VAT_26M	1389.1	858	0.2206719	0	Singlet	Adipocyte
VAT_26M	1647	1065	2.4506767	7	Singlet	Endothelial_cells
VAT_26M	1376.4	874	0	0	Singlet	Adipocyte
VAT_26M	1735.8	1084	0.0507687	0	Singlet	Adipocyte
VAT_26M	1490.3	893	0.1006207	0	Singlet	Adipocyte
VAT_26M	1627.6	1055	1.2495233	2	Singlet	FAPs
VAT_26M	3314.4	1768	5.1924105	5	Singlet	Immune_cells
VAT_26M	7568.1	2876	0.0650347	0	Singlet	Adipocyte
VAT_26M	2228.3	1204	0	0	Singlet	Adipocyte
VAT_26M	2633.5	1392	0.0081872	0	Singlet	Adipocyte
VAT_26M	1568.1	910	5.4019687	3	Singlet	FAPs
VAT_26M	4264.7	2089	0.4483811	0	Singlet	Adipocyte
VAT_26M	1887.9	1155	0.0722693	0	Singlet	Adipocyte
VAT_26M	1654.1	1115	1.4232644	2	Singlet	FAPs
VAT_26M	1947.3	1175	0	0	Singlet	Adipocyte
VAT_26M	1432.5	927	0.8142512	3	Singlet	FAPs
VAT_26M	1259.7	787	1.3122386	5	Singlet	Immune_cells
VAT_26M	3796.7	1767	0.0818036	0	Singlet	Adipocyte
VAT_26M	1045.5	691	0	0	Singlet	Adipocyte
VAT_26M	1386.7	931	0.2525251	0	Singlet	Adipocyte
VAT_26M	6427.8	2691	0.9888662	3	Singlet	FAPs
VAT_26M	2177.7	1182	1.2472106	0	Singlet	Adipocyte
VAT_26M	3346.5	1671	0.0267031	0	Singlet	Adipocyte
VAT_26M	1779	1106	0.1507108	0	Singlet	Adipocyte
VAT_26M	5130.4	2419	0	0	Singlet	Adipocyte
VAT_26M	1215.9	783	3.7848625	7	Singlet	Endothelial_cells
VAT_26M	1414.4	849	0.4307189	0	Singlet	Adipocyte
VAT_26M	3237.3	1589	0	0	Singlet	Adipocyte
VAT_26M	2608.8	1488	1.1672966	5	Singlet	Immune_cells
VAT_26M	1820.1	1066	0.9566123	3	Singlet	FAPs
VAT_26M	1146.8	708	4.6418096	3	Singlet	FAPs
VAT_26M	2392.8	1343	0	0	Singlet	Adipocyte
VAT_26M	3380.6	1658	0	0	Singlet	Adipocyte
VAT_26M	2591	1396	0.099927	0	Singlet	Adipocyte
VAT_26M	1784.7	1077	0.0364916	0	Singlet	Adipocyte
VAT_26M	1172.8	721	0.4571671	3	Singlet	FAPs
VAT_26M	2275.4	1268	0.0529836	0	Singlet	Adipocyte
VAT_26M	1758.3	1043	0.5582554	0	Singlet	Adipocyte
VAT_26M	2394.7	1282	0.029651	0	Singlet	Adipocyte
VAT_26M	1357.8	871	7.4843179	3	Singlet	FAPs
VAT_26M	2272.2	1302	0.1317731	0	Singlet	Adipocyte
VAT_26M	6788.9	2718	0.0667266	0	Singlet	Adipocyte
VAT_26M	1640.3	1012	0.0446698	0	Singlet	Adipocyte
VAT_26M	2671	1430	0.004197	0	Singlet	Adipocyte
VAT_26M	3060.6	1660	0.3908153	0	Singlet	Adipocyte
VAT_26M	2526.6	1445	0.5684675	0	Singlet	Adipocyte
VAT_26M	2504.2	1393	13.415877	3	Singlet	FAPs
VAT_26M	1187.5	816	0.406204	3	Singlet	FAPs
VAT_26M	4772.8	2117	1.203584	3	Singlet	FAPs
VAT_26M	1577.9	1056	2.8242547	3	Singlet	FAPs
VAT_26M	1156.8	775	0.1985742	0	Singlet	Adipocyte

VAT_26M	1611.3	1017	2.6803329	3	Singlet	FAPs
VAT_26M	1506.9	768	9.8765635	0	Singlet	Adipocyte
VAT_26M	2197.3	1317	0.3699618	2	Singlet	FAPs
VAT_26M	1315.8	834	6.3945758	3	Singlet	FAPs
VAT_26M	2517.2	1344	0	0	Singlet	Adipocyte
VAT_26M	1067.5	714	0.1144357	0	Singlet	Adipocyte
VAT_26M	2055.8	1202	0.0621437	3	Singlet	FAPs
VAT_26M	2214	1275	0.1585506	0	Singlet	Adipocyte
VAT_26M	2984.1	1507	0	0	Singlet	Adipocyte
VAT_26M	7570.6	2874	1.9670244	0	Singlet	Adipocyte
VAT_26M	2347.8	1366	0.0736038	0	Singlet	Adipocyte
VAT_26M	2831.5	1445	0.1550746	0	Singlet	Adipocyte
VAT_26M	2412	1338	0.0716126	0	Singlet	Adipocyte
VAT_26M	1036.7	666	0	0	Singlet	Adipocyte
VAT_26M	1013.3	716	0.034186	5	Singlet	Immune_cells
VAT_26M	1122.6	673	0.4588449	0	Singlet	Adipocyte
VAT_26M	1874.3	1091	0.2191897	0	Singlet	Adipocyte
VAT_26M	2839.4	1633	0	0	Singlet	Adipocyte
VAT_26M	1210	769	0.2246286	0	Singlet	Adipocyte
VAT_26M	1681.4	970	2.9665947	0	Singlet	Adipocyte
VAT_26M	3269.7	1730	0.0889545	0	Singlet	Adipocyte
VAT_26M	2435.1	1411	0.0484102	0	Singlet	Adipocyte
VAT_26M	1361.9	892	0.5069266	0	Singlet	Adipocyte
VAT_26M	1575.6	993	1.0599981	3	Singlet	FAPs
VAT_26M	1832.8	1097	0.7535339	0	Singlet	Adipocyte
VAT_26M	1145	764	1.5787963	2	Singlet	FAPs
VAT_26M	1145.2	808	2.3408464	5	Singlet	Immune_cells
VAT_26M	1149.7	748	0.013365	0	Singlet	Adipocyte
VAT_26M	2438.7	1370	0.8400935	5	Singlet	Immune_cells
VAT_26M	2746.3	1412	0.1497318	0	Singlet	Adipocyte
VAT_26M	1072.2	734	0.2619011	3	Singlet	FAPs
VAT_26M	2547.2	1420	1.3423833	0	Singlet	Adipocyte
VAT_26M	1060	727	0.1446431	3	Singlet	FAPs
VAT_26M	3799.1	1893	0.0598047	0	Singlet	Adipocyte
VAT_26M	1709.6	1079	6.1062748	3	Singlet	FAPs
VAT_26M	1283.3	798	0.0036821	0	Singlet	Adipocyte
VAT_26M	1115.4	695	3.3712088	3	Singlet	FAPs
VAT_26M	4401.5	2218	0.0904866	0	Singlet	Adipocyte
VAT_26M	1258.3	774	4.8766881	3	Singlet	FAPs
VAT_26M	1494.9	861	0.3495958	0	Singlet	Adipocyte
VAT_26M	1390	878	0.7386491	0	Singlet	Adipocyte
VAT_26M	1097.6	753	2.2649568	2	Singlet	FAPs
VAT_26M	2227.7	1209	6.5682791	0	Singlet	Adipocyte
VAT_26M	2686	1454	0.3630092	0	Singlet	Adipocyte
VAT_26M	3513.4	1766	0.0393901	0	Singlet	Adipocyte
VAT_26M	2860.8	1515	0.0875703	0	Singlet	Adipocyte
VAT_26M	1753.6	1050	0.3094371	0	Singlet	Adipocyte
VAT_26M	1087.4	729	0.9917406	3	Singlet	FAPs
VAT_26M	1313.3	799	10.937242	7	Singlet	Endothelial_cells
VAT_26M	1525	978	3.0766373	3	Singlet	FAPs
VAT_26M	2333.2	1228	0.0418886	0	Singlet	Adipocyte

VAT_26M	1018.2	685	4.085789	3	Singlet	FAPs
VAT_26M	1125.1	791	0.3303188	0	Singlet	Adipocyte
VAT_26M	1106.7	790	0.2701849	5	Singlet	Immune_cells
VAT_26M	2724.3	1424	0.0997865	0	Singlet	Adipocyte
VAT_26M	1598.3	991	2.9084705	3	Singlet	FAPs
VAT_26M	2438.5	1439	0.0062534	4	Singlet	Immune_cells
VAT_26M	3534.2	1758	0.1024536	0	Singlet	Adipocyte
VAT_26M	1732	1125	0.0595289	2	Singlet	FAPs
VAT_26M	1129.1	811	0.0451711	5	Singlet	Immune_cells
VAT_26M	1561.7	980	5.5781488	5	Singlet	Immune_cells
VAT_26M	5518.4	2450	0.1610171	0	Singlet	Adipocyte
VAT_26M	2677.8	1480	0.0044333	0	Singlet	Adipocyte
VAT_26M	1335.8	853	0.0422872	0	Singlet	Adipocyte
VAT_26M	1735.8	1042	0.1325865	3	Singlet	FAPs
VAT_26M	1766.4	1098	1.4679509	3	Singlet	FAPs
VAT_26M	1439.2	881	0.5329475	3	Singlet	FAPs
VAT_26M	1643.4	985	3.6114399	3	Singlet	FAPs
VAT_26M	2659.8	1543	4.1687687	7	Singlet	Endothelial_cells
VAT_26M	2341.5	1325	0.1882135	0	Singlet	Adipocyte
VAT_26M	1440.8	960	0.0573954	0	Singlet	Adipocyte
VAT_26M	1061.3	736	0.0176086	6	Singlet	Neurons
VAT_26M	2143.9	1200	0.9368863	0	Singlet	Adipocyte
VAT_26M	2453.7	1552	3.3285044	2	Singlet	FAPs
VAT_26M	3183.3	1733	0.1593158	0	Singlet	Adipocyte
VAT_26M	2609.5	1621	0.8990912	3	Singlet	FAPs
VAT_26M	1531.5	913	0	0	Singlet	Adipocyte
VAT_26M	2922.4	1470	0.381018	0	Singlet	Adipocyte
VAT_26M	1006.4	696	0.8345836	3	Singlet	FAPs
VAT_26M	1374.5	791	11.518746	5	Singlet	Immune_cells
VAT_26M	1357.6	936	2.7284801	7	Singlet	Endothelial_cells
VAT_26M	1525.3	878	0	0	Singlet	Adipocyte
VAT_26M	1239.3	775	0.0367082	0	Singlet	Adipocyte
VAT_26M	1159.1	755	7.082361	2	Singlet	FAPs
VAT_26M	1316.2	869	0.6890691	0	Singlet	Adipocyte
VAT_26M	1069.9	650	0.0671723	0	Singlet	Adipocyte
VAT_26M	2054.8	1243	2.2234282	3	Singlet	FAPs
VAT_26M	1090.2	797	0.0988042	1	Singlet	Immune_cells
VAT_26M	1795.6	1039	14.694527	3	Singlet	FAPs
VAT_26M	1672.7	1018	0.0416536	0	Singlet	Adipocyte
VAT_26M	1259.9	833	1.2339906	2	Singlet	FAPs
VAT_26M	3446.2	1879	0.1548639	0	Singlet	Adipocyte
VAT_26M	1078.4	730	0.2779435	0	Singlet	Adipocyte
VAT_26M	2178	1290	4.4678628	3	Singlet	FAPs
VAT_26M	1971.4	1165	1.4589933	0	Singlet	Adipocyte
VAT_26M	1524.4	839	0.0170129	0	Singlet	Adipocyte
VAT_26M	1179.5	765	0.0953806	0	Singlet	Adipocyte
VAT_26M	1371.4	831	0.1308851	0	Singlet	Adipocyte
VAT_26M	1298	772	2.2312055	0	Singlet	Adipocyte
VAT_26M	1186.1	744	0.2592254	0	Singlet	Adipocyte
VAT_26M	3568	1857	0	0	Singlet	Adipocyte
VAT_26M	3677.1	1708	0.4342254	0	Singlet	Adipocyte

VAT_26M	1017.4	593	13.806429	3	Singlet	FAPs
VAT_26M	1595.6	1004	1.861359	0	Singlet	Adipocyte
VAT_26M	1510.9	917	0.129146	0	Singlet	Adipocyte
VAT_26M	2140.1	1227	4.5968724	0	Singlet	Adipocyte
VAT_26M	2384.7	1326	0	0	Singlet	Adipocyte
VAT_26M	2336.5	1299	0.0189542	0	Singlet	Adipocyte
VAT_26M	1365.8	892	0	0	Singlet	Adipocyte
VAT_26M	1254.5	823	10.701481	4	Singlet	Immune_cells
VAT_26M	3634.9	1819	0.0135409	0	Singlet	Adipocyte
VAT_26M	2731.7	1423	0	0	Singlet	Adipocyte
VAT_26M	3562	1757	0.017434	0	Singlet	Adipocyte
VAT_26M	1365.1	870	0.0833086	0	Singlet	Adipocyte
VAT_26M	2296	1403	1.678721	7	Singlet	Endothelial_cells
VAT_26M	3172.7	1712	0.0451293	0	Singlet	Adipocyte
VAT_26M	2950.2	1410	0.3586252	0	Singlet	Adipocyte
VAT_26M	1341.8	848	0.1485143	0	Singlet	Adipocyte
VAT_26M	1159.3	688	8.0515038	3	Singlet	FAPs
VAT_26M	1830.3	1158	0.0467237	2	Singlet	FAPs
VAT_26M	1734.3	1024	0	0	Singlet	Adipocyte
VAT_26M	1375.8	913	0.8633265	2	Singlet	FAPs
VAT_26M	1200.3	775	0.3081202	0	Singlet	Adipocyte
VAT_26M	4214.9	1980	0	0	Singlet	Adipocyte
VAT_26M	1041.2	630	0.0631411	0	Singlet	Adipocyte
VAT_26M	3409.9	1644	0.4345253	0	Singlet	Adipocyte
VAT_26M	1408.3	917	0.6005009	0	Singlet	Adipocyte
VAT_26M	2557.5	1462	1.5414688	3	Singlet	FAPs
VAT_26M	4237.2	1974	0.0124639	0	Singlet	Adipocyte
VAT_26M	1750.9	1050	5.325906	0	Singlet	Adipocyte
VAT_26M	2066	1129	0.1204628	0	Singlet	Adipocyte
VAT_26M	1205.6	756	0.0773346	0	Singlet	Adipocyte
VAT_26M	3424.1	1787	2.2400186	0	Singlet	Adipocyte
VAT_26M	1273.2	823	4.8964598	2	Singlet	FAPs
VAT_26M	2578	1437	4.1921647	5	Singlet	Immune_cells
VAT_26M	2570.3	1406	0	0	Singlet	Adipocyte
VAT_26M	2674.3	1468	0.0366484	0	Singlet	Adipocyte
VAT_26M	1397.8	930	0	0	Singlet	Adipocyte
VAT_26M	2448.6	1330	0	0	Singlet	Adipocyte
VAT_26M	1038.7	702	2.6716242	5	Singlet	Immune_cells
VAT_26M	1097.7	710	0.2920795	0	Singlet	Adipocyte
VAT_26M	1269.8	863	0.2924137	5	Singlet	Immune_cells
VAT_26M	2594.5	1390	0.0386978	0	Singlet	Adipocyte
VAT_26M	2574.7	1489	0.0780495	0	Singlet	Adipocyte
VAT_26M	1463.9	907	1.0327566	0	Singlet	Adipocyte
VAT_26M	1011	616	0	0	Singlet	Adipocyte
VAT_26M	2174.9	1263	0.4178352	0	Singlet	Adipocyte
VAT_26M	1556.8	967	0.0305813	3	Singlet	FAPs
VAT_26M	1496.6	879	0.3808819	0	Singlet	Adipocyte
VAT_26M	2117.3	1247	0.0034634	0	Singlet	Adipocyte
VAT_26M	1459.9	888	3.7570582	0	Singlet	Adipocyte
VAT_26M	1194.1	806	7.5234646	3	Singlet	FAPs
VAT_26M	1064.5	744	0.1041216	0	Singlet	Adipocyte

VAT_26M	1027.6	649	0.1198771	6	Singlet	Neurons
VAT_26M	2239.9	1267	0.2649213	0	Singlet	Adipocyte
VAT_26M	1136.8	773	3.1539114	3	Singlet	FAPs
VAT_26M	2192.6	1232	0	0	Singlet	Adipocyte
VAT_26M	2766.6	1502	0.1749245	0	Singlet	Adipocyte
VAT_26M	2104.1	1233	0.5726944	0	Singlet	Adipocyte
VAT_26M	1613.5	991	0	0	Singlet	Adipocyte
VAT_26M	1317.5	836	1.3180408	3	Singlet	FAPs
VAT_26M	1807.7	1099	0.0256221	0	Singlet	Adipocyte
VAT_26M	1244.7	791	0.1195169	0	Singlet	Adipocyte
VAT_26M	1426.7	922	1.4622848	3	Singlet	FAPs
VAT_26M	1551.4	975	1.6122737	2	Singlet	FAPs
VAT_26M	1257.3	848	7.1037006	3	Singlet	FAPs
VAT_26M	1655.8	1013	0.0260719	0	Singlet	Adipocyte
VAT_26M	2874.4	1451	0	0	Singlet	Adipocyte
VAT_26M	1354.7	925	3.0879124	2	Singlet	FAPs
VAT_26M	1236.6	818	0.6662159	0	Singlet	Adipocyte
VAT_26M	2184.2	1258	0.122968	0	Singlet	Adipocyte
VAT_26M	3481.8	1832	0.1886541	0	Singlet	Adipocyte
VAT_26M	3001.4	1627	0.0432907	0	Singlet	Adipocyte
VAT_26M	4073.2	1966	0.0142591	0	Singlet	Adipocyte
VAT_26M	1261.9	865	0.0927313	2	Singlet	FAPs
VAT_26M	1517.3	966	0.6901641	2	Singlet	FAPs
VAT_26M	1922.2	1169	0.0073482	0	Singlet	Adipocyte
VAT_26M	1944.3	1309	0.4309283	0	Singlet	Adipocyte
VAT_26M	4843.4	2144	0	0	Singlet	Adipocyte
VAT_26M	1367.1	886	1.0277154	3	Singlet	FAPs
VAT_26M	2282.2	1265	0.0226909	0	Singlet	Adipocyte
VAT_26M	1703.2	987	3.5527378	0	Singlet	Adipocyte
VAT_26M	1088.6	739	4.636924	9	Singlet	Mesothelial
VAT_26M	1947.7	1079	0	0	Singlet	Adipocyte
VAT_26M	2729.7	1461	0.0036238	0	Singlet	Adipocyte
VAT_26M	1746.8	1052	0.024174	0	Singlet	Adipocyte
VAT_26M	2071	1161	0.0444133	0	Singlet	Adipocyte
VAT_26M	3060.8	1564	0.864929	0	Singlet	Adipocyte
VAT_26M	2755.5	1531	0.0716657	0	Singlet	Adipocyte
VAT_26M	3946.9	1868	0.1056947	0	Singlet	Adipocyte
VAT_26M	1090.8	688	0.1975365	0	Singlet	Adipocyte
VAT_26M	1026.5	678	2.7395364	6	Singlet	Neurons
VAT_26M	1140.8	715	9.8244091	3	Singlet	FAPs
VAT_26M	1563.1	1002	0.0533388	0	Singlet	Adipocyte
VAT_26M	1126.6	776	0.0595725	0	Singlet	Adipocyte
VAT_26M	1436.1	903	0.0226503	6	Singlet	Neurons
VAT_26M	3250.9	1659	0.0770199	0	Singlet	Adipocyte
VAT_26M	1056.3	720	0.0206169	0	Singlet	Adipocyte
VAT_26M	3117.7	1597	0.0195908	0	Singlet	Adipocyte
VAT_26M	1048.6	719	0.0507176	5	Singlet	Immune_cells
VAT_26M	2577	1424	0	0	Singlet	Adipocyte
VAT_26M	1954.1	1232	0.9913169	3	Singlet	FAPs
VAT_26M	1445.3	930	0.2063423	0	Singlet	Adipocyte
VAT_26M	5328.4	2251	0	0	Singlet	Adipocyte

VAT_26M	1476.8	959	0.9891313	0	Singlet	Adipocyte
VAT_26M	1104.2	717	1.81096	0	Singlet	Adipocyte
VAT_26M	1699.8	1004	8.3665194	3	Singlet	FAPs
VAT_26M	1271.1	782	0.7616439	0	Singlet	Adipocyte
VAT_26M	1011.4	749	0.2187129	5	Singlet	Immune_cells
VAT_26M	1197.3	803	0.0544014	3	Singlet	FAPs
VAT_26M	2669.1	1369	6.9793838	0	Singlet	Adipocyte
VAT_26M	1888.5	1103	1.0888491	0	Singlet	Adipocyte
VAT_26M	1890.7	1153	0.012261	0	Singlet	Adipocyte
VAT_26M	1057.8	740	1.6426662	11	Singlet	Smooth_muscle_cells
VAT_26M	3402.3	1707	0	0	Singlet	Adipocyte
VAT_26M	1159.6	820	3.5964918	2	Singlet	FAPs
VAT_26M	3899.6	1873	0	0	Singlet	Adipocyte
VAT_26M	3179.9	1742	0.0498743	0	Singlet	Adipocyte
VAT_26M	1906.7	1042	1.1208202	0	Singlet	Adipocyte
VAT_26M	1236.6	786	13.677962	8	Singlet	Mesothelial
VAT_26M	4778.2	2167	0.023653	0	Singlet	Adipocyte
VAT_26M	4080.1	2053	0	0	Singlet	Adipocyte
VAT_26M	1231.3	782	3.7439499	3	Singlet	FAPs
VAT_26M	2563.9	1382	0	0	Singlet	Adipocyte
VAT_26M	1708.4	1065	0.2572362	0	Singlet	Adipocyte
VAT_26M	1339.4	898	3.3756743	3	Singlet	FAPs
VAT_26M	3357.6	1712	0.0897359	0	Singlet	Adipocyte
VAT_26M	6416.2	2704	0	0	Singlet	Adipocyte
VAT_26M	1591.1	938	0.0391517	0	Singlet	Adipocyte
VAT_26M	1115	772	0.2142994	3	Singlet	FAPs
VAT_26M	5841.9	2271	0.1324934	0	Singlet	Adipocyte
VAT_26M	6558.2	2672	0.002017	0	Singlet	Adipocyte
VAT_26M	1294.4	812	4.1949935	3	Singlet	FAPs
VAT_26M	1236.3	767	0.0076051	0	Singlet	Adipocyte
VAT_26M	3105.7	1675	0.0184088	0	Singlet	Adipocyte
VAT_26M	3160.1	1687	0.1770697	0	Singlet	Adipocyte
VAT_26M	1785.3	1091	0.1018206	0	Singlet	Adipocyte
VAT_26M	2193.1	1326	1.6373129	0	Singlet	Adipocyte
VAT_26M	1043.7	729	1.0496123	0	Singlet	Adipocyte
VAT_26M	2973.8	1646	0.3739958	0	Singlet	Adipocyte
VAT_26M	1694.4	1086	1.7992359	3	Singlet	FAPs
VAT_26M	3160.3	1702	0.0188392	0	Singlet	Adipocyte
VAT_26M	1247.4	1089	0	6	Singlet	Neurons
VAT_26M	4127.9	2015	0.5612199	0	Singlet	Adipocyte
VAT_26M	1044.9	753	0.0038586	5	Singlet	Immune_cells
VAT_26M	1165.1	778	0.1373844	0	Singlet	Adipocyte
VAT_26M	3739.1	1845	0.0183041	0	Singlet	Adipocyte
VAT_26M	1920.1	1072	0.0973682	0	Singlet	Adipocyte
VAT_26M	2787.6	1538	0.0357846	0	Singlet	Adipocyte
VAT_26M	3162.2	1626	0.3083555	0	Singlet	Adipocyte
VAT_26M	2133	1269	0.0189551	0	Singlet	Adipocyte
VAT_26M	2587.2	1413	0.0032087	0	Singlet	Adipocyte
VAT_26M	1935.4	1140	0	0	Singlet	Adipocyte
VAT_26M	3302.1	1667	0.0934154	0	Singlet	Adipocyte
VAT_26M	3223.8	1648	0	0	Singlet	Adipocyte

VAT_26M	1525.1	1005	5.3104307	3	Singlet	FAPs
VAT_26M	1776.2	1070	0.0773562	0	Singlet	Adipocyte
VAT_26M	2797.1	1580	0.2421579	0	Singlet	Adipocyte
VAT_26M	3605.8	1849	0	0	Singlet	Adipocyte
VAT_26M	1049.1	667	0.6115652	0	Singlet	Adipocyte
VAT_26M	2707.4	1496	0.043852	0	Singlet	Adipocyte
VAT_26M	2199.2	1230	0.6808709	0	Singlet	Adipocyte
VAT_26M	1982.9	1127	0.0678128	0	Singlet	Adipocyte
VAT_26M	5270.2	2382	0.2738064	0	Singlet	Adipocyte
VAT_26M	1132.4	749	0	0	Singlet	Adipocyte
VAT_26M	1974.3	1167	0.0904552	0	Singlet	Adipocyte
VAT_26M	3258	1589	0.0261447	0	Singlet	Adipocyte
VAT_26M	1001.7	694	0	0	Singlet	Adipocyte
VAT_26M	2436.9	1392	0.0068613	0	Singlet	Adipocyte
VAT_26M	2020.7	1164	0.2382455	0	Singlet	Adipocyte
VAT_26M	3024.6	1609	0.0276016	0	Singlet	Adipocyte
VAT_26M	3510	1761	0	0	Singlet	Adipocyte
VAT_26M	1272.6	832	5.0706586	7	Singlet	Endothelial_cells
VAT_26M	1674.9	988	0.1001925	0	Singlet	Adipocyte
VAT_26M	2313.4	1365	0.2174855	0	Singlet	Adipocyte
VAT_26M	3736.1	1826	0.0446747	0	Singlet	Adipocyte
VAT_26M	3373.8	1686	4.313591	0	Singlet	Adipocyte
VAT_26M	1149.4	806	0.1807993	2	Singlet	FAPs
VAT_26M	3325	1813	0.1313269	2	Singlet	FAPs
VAT_26M	1305.6	848	2.0833567	5	Singlet	Immune_cells
VAT_26M	2502.4	1491	2.1849189	3	Singlet	FAPs
VAT_26M	2367.9	1352	4.7562889	0	Singlet	Adipocyte
VAT_26M	1907.3	1237	2.5857258	5	Singlet	Immune_cells
VAT_26M	1996.9	1125	0.0257742	0	Singlet	Adipocyte
VAT_26M	1882.1	1091	3.6849224	5	Singlet	Immune_cells
VAT_26M	5543.4	2349	0	0	Singlet	Adipocyte
VAT_26M	4951.6	2174	0	0	Singlet	Adipocyte
VAT_26M	2727	1511	0.8619475	0	Singlet	Adipocyte
VAT_26M	6030.1	2543	0.0067757	0	Singlet	Adipocyte
VAT_26M	2246.5	1288	0.4392091	0	Singlet	Adipocyte
VAT_26M	1023.3	691	0.549649	0	Singlet	Adipocyte
VAT_26M	4104.5	2002	0	0	Singlet	Adipocyte
VAT_26M	1375.7	779	0.4431555	0	Singlet	Adipocyte
VAT_26M	4606.2	2162	0.1795642	0	Singlet	Adipocyte
VAT_26M	3030.6	1625	0.0530382	0	Singlet	Adipocyte
VAT_26M	1123.8	710	0.1583445	0	Singlet	Adipocyte
VAT_26M	1534	948	0.5895791	0	Singlet	Adipocyte
VAT_26M	1368.6	850	0.1130133	0	Singlet	Adipocyte
VAT_26M	1184.7	771	0.6548322	0	Singlet	Adipocyte
VAT_26M	2268.1	1247	0.0591793	0	Singlet	Adipocyte
VAT_26M	1070.2	705	10.35969	5	Singlet	Immune_cells
VAT_26M	5204.2	2807	3.4498593	2	Singlet	FAPs
VAT_26M	3439.9	1637	0.1822314	0	Singlet	Adipocyte
VAT_26M	1088.6	704	6.1110836	7	Singlet	Endothelial_cells
VAT_26M	2000.2	1125	0.0512682	0	Singlet	Adipocyte
VAT_26M	2716.9	1495	0.1406087	0	Singlet	Adipocyte

VAT_26M	1982.6	1106	0.0326123	0	Singlet	Adipocyte
VAT_26M	1550.3	1012	0.9351819	0	Singlet	Adipocyte
VAT_26M	1088.5	850	1.4030354	3	Singlet	FAPs
VAT_26M	3468.7	1714	0.0796415	0	Singlet	Adipocyte
VAT_26M	1578	960	0.1118417	0	Singlet	Adipocyte
VAT_26M	1235.8	849	1.70889	5	Singlet	Immune_cells
VAT_26M	2245.2	1228	0.0094831	0	Singlet	Adipocyte
VAT_26M	2235.3	1255	0.0095701	0	Singlet	Adipocyte
VAT_26M	1616.8	1009	8.026494	2	Singlet	FAPs
VAT_26M	2004.7	1169	0.0164613	0	Singlet	Adipocyte
VAT_26M	1313.3	799	0.4549502	0	Singlet	Adipocyte
VAT_26M	2087.2	1150	0.0185369	0	Singlet	Adipocyte
VAT_26M	1821.5	1145	1.0785044	3	Singlet	FAPs
VAT_26M	1794.8	1107	1.2332947	5	Singlet	Immune_cells
VAT_26M	1241.4	834	0.1787307	3	Singlet	FAPs
VAT_26M	2102.2	1268	0.0741911	0	Singlet	Adipocyte
VAT_26M	3913.6	1957	0.8569572	0	Singlet	Adipocyte
VAT_26M	2159	1262	0.0738285	0	Singlet	Adipocyte
VAT_26M	4627.4	2135	0	0	Singlet	Adipocyte
VAT_26M	2069.9	1368	2.2592383	7	Singlet	Endothelial_cells
VAT_26M	1446.5	859	0.1746143	0	Singlet	Adipocyte
VAT_26M	1630.4	901	1.5211353	0	Singlet	Adipocyte
VAT_26M	1890.8	1180	0.4145885	0	Singlet	Adipocyte
VAT_26M	1822.3	1095	2.5326933	0	Singlet	Adipocyte
VAT_26M	5003	2348	0.2307846	0	Singlet	Adipocyte
VAT_26M	1163.5	731	0.0977337	0	Singlet	Adipocyte
VAT_26M	3769.5	1796	0.1925748	0	Singlet	Adipocyte
VAT_26M	2878.4	1549	1.3303473	0	Singlet	Adipocyte
VAT_26M	1374.1	861	0.1712441	0	Singlet	Adipocyte
VAT_26M	4308.2	2003	0.073397	0	Singlet	Adipocyte
VAT_26M	2781.9	1476	0.0699797	0	Singlet	Adipocyte
VAT_26M	4343.3	2029	0.2497394	0	Singlet	Adipocyte
VAT_26M	1402.2	887	0.4840111	0	Singlet	Adipocyte
VAT_26M	2814.7	1436	0.0410383	0	Singlet	Adipocyte
VAT_26M	1615.5	1091	0.1468152	2	Singlet	FAPs
VAT_26M	1419.1	889	0.0974459	0	Singlet	Adipocyte
VAT_26M	1175.9	731	0.3293084	0	Singlet	Adipocyte
VAT_26M	2519.5	1326	0.5275896	0	Singlet	Adipocyte
VAT_26M	2121.5	1194	0.0158286	0	Singlet	Adipocyte
VAT_26M	1003.7	650	0.7487197	0	Singlet	Adipocyte
VAT_26M	2314.2	1424	3.8205626	11	Singlet	Smooth_muscle_cells
VAT_26M	2792.9	1574	0.0025576	0	Singlet	Adipocyte
VAT_26M	1149.8	746	0.0576243	0	Singlet	Adipocyte
VAT_26M	3619.4	1849	0.1325375	0	Singlet	Adipocyte
VAT_26M	1339.2	866	0.5837652	3	Singlet	FAPs
VAT_26M	1066	723	2.2947516	3	Singlet	FAPs
VAT_26M	1233.4	871	2.5357334	5	Singlet	Immune_cells
VAT_26M	1523.9	954	0.0319018	0	Singlet	Adipocyte
VAT_26M	5088.7	2190	0	0	Singlet	Adipocyte
VAT_26M	2846.8	1532	0.3341757	0	Singlet	Adipocyte
VAT_26M	3969.6	1841	14.638172	0	Singlet	Adipocyte

VAT_26M	1158	727	0.3236822	0	Singlet	Adipocyte
VAT_26M	4721.7	2240	0.0733189	0	Singlet	Adipocyte
VAT_26M	3640.8	1895	0.7672898	0	Singlet	Adipocyte
VAT_26M	1359.5	883	0.0212575	0	Singlet	Adipocyte
VAT_26M	1553.2	904	0.0210486	0	Singlet	Adipocyte
VAT_26M	2606.9	1520	3.1309297	7	Singlet	Endothelial_cells
VAT_26M	1381.9	875	3.7369839	3	Singlet	FAPs
VAT_26M	4184.5	2053	0.5953806	0	Singlet	Adipocyte
VAT_26M	1418.5	887	0.2341066	0	Singlet	Adipocyte
VAT_26M	1912.8	1147	0.1152732	0	Singlet	Adipocyte
VAT_26M	2622.2	1497	5.0018493	7	Singlet	Endothelial_cells
VAT_26M	3203.3	1603	0.1123564	0	Singlet	Adipocyte
VAT_26M	1145.3	757	1.2826921	3	Singlet	FAPs
VAT_26M	1729.3	1089	0.1355751	0	Singlet	Adipocyte
VAT_26M	1456.3	917	2.3067463	0	Singlet	Adipocyte
VAT_26M	2614.6	1371	0.1825079	0	Singlet	Adipocyte
VAT_26M	1101.9	744	0.3981511	5	Singlet	Immune_cells
VAT_26M	3989.1	1961	0	0	Singlet	Adipocyte
VAT_26M	1006.4	726	2.11209	5	Singlet	Immune_cells
VAT_26M	1205.2	802	4.3200515	5	Singlet	Immune_cells
VAT_26M	3051.9	1614	0.5187385	0	Singlet	Adipocyte
VAT_26M	4359.2	2090	0.1746822	0	Singlet	Adipocyte
VAT_26M	1237.6	774	0.0515846	0	Singlet	Adipocyte
VAT_26M	1315.3	821	1.0213447	0	Singlet	Adipocyte
VAT_26M	1542.1	980	0.1045995	3	Singlet	FAPs
VAT_26M	3930.9	1953	0	0	Singlet	Adipocyte
VAT_26M	1101.9	722	0	0	Singlet	Adipocyte
VAT_26M	6819.7	2867	2.2791054	0	Singlet	Adipocyte
VAT_26M	1582	997	0.7310971	0	Singlet	Adipocyte
VAT_26M	1369.3	846	0.0687616	0	Singlet	Adipocyte
VAT_26M	2061.2	1216	0.6911567	0	Singlet	Adipocyte
VAT_26M	2457.1	1323	0.3631178	0	Singlet	Adipocyte
VAT_26M	1114.4	753	0.7088646	5	Singlet	Immune_cells
VAT_26M	1855.1	1094	2.8918986	0	Singlet	Adipocyte
VAT_26M	1332.4	856	1.2873192	11	Singlet	Smooth_muscle_cells
VAT_26M	1582.6	986	0.6148201	0	Singlet	Adipocyte
VAT_26M	1606	963	0.0327332	0	Singlet	Adipocyte
VAT_26M	2489.1	1407	0.0008359	3	Singlet	FAPs
VAT_26M	1526.4	1035	1.9064992	2	Singlet	FAPs
VAT_26M	1165.6	702	0.5787235	0	Singlet	Adipocyte
VAT_26M	1150.6	722	0.1393396	0	Singlet	Adipocyte
VAT_26M	1591.3	984	0.1289738	0	Singlet	Adipocyte
VAT_26M	1580.3	989	0.4596341	0	Singlet	Adipocyte
VAT_26M	1669.3	976	0.3260063	0	Singlet	Adipocyte
VAT_26M	1205.2	819	0.1791608	5	Singlet	Immune_cells
VAT_26M	1204.1	751	0.2808243	0	Singlet	Adipocyte
VAT_26M	2318.6	1320	0.1755016	0	Singlet	Adipocyte
VAT_26M	1529.5	871	3.2709502	0	Singlet	Adipocyte
VAT_26M	5869.5	2466	0.3384161	0	Singlet	Adipocyte
VAT_26M	2477.7	1319	0.154554	0	Singlet	Adipocyte
VAT_26M	1637.4	967	0	0	Singlet	Adipocyte

VAT_26M	1497.8	1002	1.1910821	7	Singlet	Endothelial_cells
VAT_26M	1262.5	761	6.1099027	5	Singlet	Immune_cells
VAT_26M	1137.5	726	0.0564775	0	Singlet	Adipocyte
VAT_26M	2462.2	1321	0.0555096	0	Singlet	Adipocyte
VAT_26M	1100.7	733	8.5902886	3	Singlet	FAPs
VAT_26M	2736.5	1663	1.0675721	2	Singlet	FAPs
VAT_26M	1245.1	841	0.577456	0	Singlet	Adipocyte
VAT_26M	1071.9	662	0.2201548	0	Singlet	Adipocyte
VAT_26M	2152.3	1202	0.0650855	0	Singlet	Adipocyte
VAT_26M	1567.5	983	1.5596195	3	Singlet	FAPs
VAT_26M	1564.7	924	0.0196749	0	Singlet	Adipocyte
VAT_26M	1745.6	1093	0.1567609	0	Singlet	Adipocyte
VAT_26M	2183.3	1262	0.0075166	0	Singlet	Adipocyte
VAT_26M	1939.1	1261	2.5862952	2	Singlet	FAPs
VAT_26M	5944.7	2528	0	0	Singlet	Adipocyte
VAT_26M	1886	1058	0.0586914	0	Singlet	Adipocyte
VAT_26M	2997.4	1594	0	0	Singlet	Adipocyte
VAT_26M	1321.2	765	12.357048	7	Singlet	Endothelial_cells
VAT_26M	3027.8	1535	0.1116953	0	Singlet	Adipocyte
VAT_26M	4414.9	2044	0	0	Singlet	Adipocyte
VAT_26M	2610.3	1411	0.4503605	0	Singlet	Adipocyte
VAT_26M	1937.5	1127	0	0	Singlet	Adipocyte
VAT_26M	1074.2	691	0.767625	0	Singlet	Adipocyte
VAT_26M	2731.4	1409	0	0	Singlet	Adipocyte
VAT_26M	2582.2	1303	0.111863	0	Singlet	Adipocyte
VAT_26M	1971.8	1253	1.5146006	0	Singlet	Adipocyte
VAT_26M	1819.7	1122	0.2773528	0	Singlet	Adipocyte
VAT_26M	1188.4	742	0.1418014	0	Singlet	Adipocyte
VAT_26M	2262.3	1287	0.1564557	0	Singlet	Adipocyte
VAT_26M	4518.7	2035	0	0	Singlet	Adipocyte
VAT_26M	3646.4	1770	0.5673283	0	Singlet	Adipocyte
VAT_26M	1036.2	701	0.1819124	0	Singlet	Adipocyte
VAT_26M	1616.3	1069	0.097155	3	Singlet	FAPs
VAT_26M	2063.1	1254	6.7908585	3	Singlet	FAPs
VAT_26M	1895.5	1140	7.972994	2	Singlet	FAPs
VAT_26M	1617.3	1056	0.9813525	3	Singlet	FAPs
VAT_26M	1165.8	771	0.2885935	0	Singlet	Adipocyte
VAT_26M	2786.8	1524	0.0553214	0	Singlet	Adipocyte
VAT_26M	2606.8	1455	0.1745522	0	Singlet	Adipocyte
VAT_26M	3039	1601	0.0189367	0	Singlet	Adipocyte
VAT_26M	2621.8	1583	1.5459824	3	Singlet	FAPs
VAT_26M	1075.5	735	2.0972845	0	Singlet	Adipocyte
VAT_26M	1499.5	981	1.067393	0	Singlet	Adipocyte
VAT_26M	1800.7	1114	8.1992348	5	Singlet	Immune_cells
VAT_26M	1486.1	905	0	0	Singlet	Adipocyte
VAT_26M	3083.6	1535	0	0	Singlet	Adipocyte
VAT_26M	3300.4	1667	0.0249707	0	Singlet	Adipocyte
VAT_26M	2351.6	1231	0.3798876	0	Singlet	Adipocyte
VAT_26M	2645.7	1465	0.1386491	0	Singlet	Adipocyte
VAT_26M	4857.2	2139	0	0	Singlet	Adipocyte
VAT_26M	1356.6	838	0.029146	0	Singlet	Adipocyte

VAT_26M	3113.3	1617	0.0152029	0	Singlet	Adipocyte
VAT_26M	2471.2	1577	0.0440148	5	Singlet	Immune_cells
VAT_26M	1125.6	743	2.1239659	0	Singlet	Adipocyte
VAT_26M	3935.5	1879	0.1699047	0	Singlet	Adipocyte
VAT_26M	1521.7	910	0.626172	0	Singlet	Adipocyte
VAT_26M	1776.5	1115	0.272401	0	Singlet	Adipocyte
VAT_26M	1896.4	1132	4.4829581	3	Singlet	FAPs
VAT_26M	1847.7	1128	0.0419123	0	Singlet	Adipocyte
VAT_26M	3242.5	1712	0.3907334	0	Singlet	Adipocyte
VAT_26M	2080.5	1214	0.2949796	0	Singlet	Adipocyte
VAT_26M	1139.2	722	0.4647619	5	Singlet	Immune_cells
VAT_26M	4964.9	2276	2.8333358	0	Singlet	Adipocyte
VAT_26M	1522.6	977	0.7751413	5	Singlet	Immune_cells
VAT_26M	1842.2	1023	8.8924165	0	Singlet	Adipocyte
VAT_26M	1110.5	717	2.1877921	3	Singlet	FAPs
VAT_26M	5121.6	2322	0	0	Singlet	Adipocyte
VAT_26M	1381.2	913	0.3157809	0	Singlet	Adipocyte
VAT_26M	1124	689	0.0576197	6	Singlet	Neurons
VAT_26M	1543	965	1.0614686	0	Singlet	Adipocyte
VAT_26M	2916.3	1501	0.2843033	0	Singlet	Adipocyte
VAT_26M	2045	1183	3.3044137	0	Singlet	Adipocyte
VAT_26M	2341.9	1290	0.134267	0	Singlet	Adipocyte
VAT_26M	2827.3	1462	0	0	Singlet	Adipocyte
VAT_26M	1799.9	1063	0	0	Singlet	Adipocyte
VAT_26M	1114.2	825	0.0459478	5	Singlet	Immune_cells
VAT_26M	1411.5	862	0.5265327	0	Singlet	Adipocyte
VAT_26M	1324.1	882	1.0873265	5	Singlet	Immune_cells
VAT_26M	2632.6	1417	0.7992753	0	Singlet	Adipocyte
VAT_26M	1002.1	656	0.7328644	0	Singlet	Adipocyte
VAT_26M	1261.7	837	0.6976488	0	Singlet	Adipocyte
VAT_26M	1614	942	0	0	Singlet	Adipocyte
VAT_26M	4528.5	2120	0.2456021	0	Singlet	Adipocyte
VAT_26M	1249.3	771	0.303915	0	Singlet	Adipocyte
VAT_26M	2313	1285	0	0	Singlet	Adipocyte
VAT_26M	2824.4	1499	1.1151531	0	Singlet	Adipocyte
VAT_26M	1643.8	1018	1.1909337	0	Singlet	Adipocyte
VAT_26M	1064.4	684	0.1730466	0	Singlet	Adipocyte
VAT_26M	1588.3	934	0.0505493	0	Singlet	Adipocyte
VAT_26M	2050.7	1265	0.2597342	0	Singlet	Adipocyte
VAT_26M	1181.2	785	9.4527198	2	Singlet	FAPs
VAT_26M	4088.9	1865	0	0	Singlet	Adipocyte
VAT_26M	1294	827	0	6	Singlet	Neurons
VAT_26M	1669.2	1059	0.1931877	0	Singlet	Adipocyte
VAT_26M	1262	871	0	5	Singlet	Immune_cells
VAT_26M	1820.4	1090	2.1472381	0	Singlet	Adipocyte
VAT_26M	4188.7	1861	0.0951822	0	Singlet	Adipocyte
VAT_26M	1378.1	889	5.689544	6	Singlet	Neurons
VAT_26M	1088.4	631	0.1066178	0	Singlet	Adipocyte
VAT_26M	3716.7	1775	0.0653354	0	Singlet	Adipocyte
VAT_26M	2059.9	1283	2.3021487	3	Singlet	FAPs
VAT_26M	1016.1	581	7.8763888	5	Singlet	Immune_cells

VAT_26M	4238.3	2022	0.0683634	0	Singlet	Adipocyte
VAT_26M	5635.8	2379	0.0164514	0	Singlet	Adipocyte
VAT_26M	1379.4	869	0	2	Singlet	FAPs
VAT_26M	2102.1	1142	0	0	Singlet	Adipocyte
VAT_26M	1133.5	777	0.1556784	0	Singlet	Adipocyte
VAT_26M	2278.4	1423	0.5172318	3	Singlet	FAPs
VAT_26M	3424	1721	0.3420101	0	Singlet	Adipocyte
VAT_26M	6578.8	2607	2.0075657	0	Singlet	Adipocyte
VAT_26M	2214.5	1249	0.0113805	0	Singlet	Adipocyte
VAT_26M	5543.8	2387	0	0	Singlet	Adipocyte
VAT_26M	3235.4	1615	0.0007974	0	Singlet	Adipocyte
VAT_26M	1004	739	2.1439831	4	Singlet	Immune_cells
VAT_26M	1566.7	1002	0.0842716	0	Singlet	Adipocyte
VAT_26M	3339.8	1709	0.1723496	0	Singlet	Adipocyte
VAT_26M	2625.5	1548	2.1952228	7	Singlet	Endothelial_cells
VAT_26M	1574.3	1048	7.7426994	4	Singlet	Immune_cells
VAT_26M	1194.1	738	7.6430729	3	Singlet	FAPs
VAT_26M	2439.4	1342	0.0455358	0	Singlet	Adipocyte
VAT_26M	1934.3	1250	2.3543399	7	Singlet	Endothelial_cells
VAT_26M	1981.3	1154	0.1054611	0	Singlet	Adipocyte
VAT_26M	1369.1	861	4.8591076	3	Singlet	FAPs
VAT_26M	1193	757	1.3930014	3	Singlet	FAPs
VAT_26M	5082.7	2496	3.8171972	3	Singlet	FAPs
VAT_26M	2236.5	1373	0.022194	5	Singlet	Immune_cells
VAT_26M	1001	697	1.1991591	3	Singlet	FAPs
VAT_26M	3611.6	1777	0	0	Singlet	Adipocyte
VAT_26M	1296.1	817	0.6190011	0	Singlet	Adipocyte
VAT_26M	1140.7	712	4.1967178	0	Singlet	Adipocyte
VAT_26M	3466.1	1753	1.6279542	0	Singlet	Adipocyte
VAT_26M	2876.3	1522	0	0	Singlet	Adipocyte
VAT_26M	3700.4	1804	0	0	Singlet	Adipocyte
VAT_26M	1566.9	939	7.9946851	3	Singlet	FAPs
VAT_26M	6086.6	2650	0.0412299	0	Singlet	Adipocyte
VAT_26M	3787	1721	0.9407386	0	Singlet	Adipocyte
VAT_26M	1013.9	706	0.896447	5	Singlet	Immune_cells
VAT_26M	1983.4	1172	1.1980068	0	Singlet	Adipocyte
VAT_26M	3825.8	1840	0.0350999	0	Singlet	Adipocyte
VAT_26M	1030.4	620	0.3236708	0	Singlet	Adipocyte
VAT_26M	1103.3	782	3.0573865	3	Singlet	FAPs
VAT_26M	1530.2	1039	0.5754427	2	Singlet	FAPs
VAT_26M	1824.1	1166	3.5170049	2	Singlet	FAPs
VAT_26M	1442.4	930	2.8760755	3	Singlet	FAPs
VAT_26M	1250	763	0.0479765	0	Singlet	Adipocyte
VAT_26M	1381.3	971	3.5316987	7	Singlet	Endothelial_cells
VAT_26M	1710.6	981	0.1555716	0	Singlet	Adipocyte
VAT_26M	1121.1	726	1.3526963	3	Singlet	FAPs
VAT_26M	1745.9	1021	0.0088466	0	Singlet	Adipocyte
VAT_26M	1742.4	1022	0.6227546	0	Singlet	Adipocyte
VAT_26M	1387.5	885	0.2802549	0	Singlet	Adipocyte
VAT_26M	4068.4	1982	0.1193395	0	Singlet	Adipocyte
VAT_26M	3776.3	1875	0.0190193	0	Singlet	Adipocyte

VAT_26M	1000	737	0.145681	5 Singlet	Immune_cells
VAT_26M	1042.1	669	0.2451891	0 Singlet	Adipocyte
VAT_26M	2069.9	1181	0.3262415	0 Singlet	Adipocyte
VAT_26M	1056.7	719	1.3873982	3 Singlet	FAPs
VAT_26M	1226.4	838	6.3130747	2 Singlet	FAPs
VAT_26M	2800.1	1496	0.6641718	0 Singlet	Adipocyte
VAT_26M	1648.3	974	0.773332	0 Singlet	Adipocyte
VAT_26M	2462.9	1345	2.5288929	0 Singlet	Adipocyte
VAT_26M	2614.7	1419	0	0 Singlet	Adipocyte
VAT_26M	1718.6	1098	0.212529	5 Singlet	Immune_cells
VAT_26M	4746.6	2195	0	0 Singlet	Adipocyte
VAT_26M	1197.2	849	0.0347386	7 Singlet	Endothelial_cells
VAT_26M	1619.5	1139	2.2103981	2 Singlet	FAPs
VAT_26M	1857.8	1153	0.1324008	0 Singlet	Adipocyte
VAT_26M	1971.6	1136	0.0890297	0 Singlet	Adipocyte
VAT_26M	1543.7	897	0	0 Singlet	Adipocyte
VAT_26M	1292.3	859	0.308781	0 Singlet	Adipocyte
VAT_26M	1703.4	1030	1.3677861	0 Singlet	Adipocyte
VAT_26M	1108.9	736	0.3579979	0 Singlet	Adipocyte
VAT_26M	2878.3	1448	0.1223237	0 Singlet	Adipocyte
VAT_26M	1540.9	956	0.0768976	0 Singlet	Adipocyte
VAT_26M	1502	903	0.4753328	0 Singlet	Adipocyte
VAT_26M	2293.7	1374	0.003501	0 Singlet	Adipocyte
VAT_26M	1056.2	683	0.0548056	0 Singlet	Adipocyte
VAT_26M	1684.6	1002	0.0294738	0 Singlet	Adipocyte
VAT_26M	3773	1893	0	0 Singlet	Adipocyte
VAT_26M	1928	1197	1.1141386	3 Singlet	FAPs
VAT_26M	1476.8	1000	2.1160667	5 Singlet	Immune_cells
VAT_26M	1500.8	951	7.6386015	5 Singlet	Immune_cells
VAT_26M	1079.5	682	3.2520454	0 Singlet	Adipocyte
VAT_26M	1032	620	0.0635322	0 Singlet	Adipocyte
VAT_26M	5722.5	2525	0	0 Singlet	Adipocyte
VAT_26M	2555.8	1389	2.4325198	0 Singlet	Adipocyte
VAT_26M	1452.7	926	0.0359465	0 Singlet	Adipocyte
VAT_26M	1157.7	679	0.0416192	0 Singlet	Adipocyte
VAT_26M	4881	2191	3.4436475	0 Singlet	Adipocyte
VAT_26M	3429.8	1693	0.2507164	0 Singlet	Adipocyte
VAT_26M	2032.1	1238	1.6855279	3 Singlet	FAPs
VAT_26M	1397.3	1052	0.0853976	4 Singlet	Immune_cells
VAT_26M	2052.2	1160	0.0039134	0 Singlet	Adipocyte
VAT_26M	1558.7	958	0.5425889	0 Singlet	Adipocyte
VAT_26M	3476.1	1697	0	0 Singlet	Adipocyte
VAT_26M	2308.1	1247	0.3541435	0 Singlet	Adipocyte
VAT_26M	1098.1	724	0.0589154	0 Singlet	Adipocyte
VAT_26M	2177.6	1261	0.337834	0 Singlet	Adipocyte
VAT_26M	1031.3	689	0.8027788	2 Singlet	FAPs
VAT_26M	1930.7	1161	0.0253016	0 Singlet	Adipocyte
VAT_26M	1000.4	675	2.3036459	3 Singlet	FAPs
VAT_26M	1000.9	668	0.0259455	0 Singlet	Adipocyte
VAT_26M	2127.8	1349	0.0335176	0 Singlet	Adipocyte
VAT_26M	2562	1438	0.0715592	0 Singlet	Adipocyte

VAT_26M	1329.8	951	0.234568	3	Singlet	FAPs
VAT_26M	2144	1294	0.6212777	0	Singlet	Adipocyte
VAT_26M	1950.9	1109	1.1407443	0	Singlet	Adipocyte
VAT_26M	2847.2	1489	0.7009186	0	Singlet	Adipocyte
VAT_26M	1925.7	1113	0.0523513	0	Singlet	Adipocyte
VAT_26M	1123.2	718	8.429542	3	Singlet	FAPs
VAT_26M	2447.5	1257	0.0422934	0	Singlet	Adipocyte
VAT_26M	1694.7	989	6.5851943	3	Singlet	FAPs
VAT_26M	2543.5	1315	1.9774732	0	Singlet	Adipocyte
VAT_26M	1789.5	1074	0.2355416	0	Singlet	Adipocyte
VAT_26M	1005.6	679	0.1258554	0	Singlet	Adipocyte
VAT_26M	1917.7	1078	6.2278418	5	Singlet	Immune_cells
VAT_26M	3120	1605	0.0206963	0	Singlet	Adipocyte
VAT_26M	1064.7	708	2.1604047	0	Singlet	Adipocyte
VAT_26M	2176.7	1314	0.166877	0	Singlet	Adipocyte
VAT_26M	1313.5	852	0.1675189	0	Singlet	Adipocyte
VAT_26M	2815.5	1554	0.0765244	0	Singlet	Adipocyte
VAT_26M	2358.5	1251	0.2734736	0	Singlet	Adipocyte
VAT_26M	2400	1259	0.0989677	0	Singlet	Adipocyte
VAT_26M	3104.5	1554	1.179223	0	Singlet	Adipocyte
VAT_26M	1543.6	914	6.0058064	0	Singlet	Adipocyte
VAT_26M	2923.7	1518	0.3948685	0	Singlet	Adipocyte
VAT_26M	1422.6	866	0.1645032	0	Singlet	Adipocyte
VAT_26M	4534.6	2117	0.1746319	0	Singlet	Adipocyte
VAT_26M	1192.6	778	5.3616979	3	Singlet	FAPs
VAT_26M	1334.6	769	10.271759	0	Singlet	Adipocyte
VAT_26M	1576.7	955	0.8144334	0	Singlet	Adipocyte
VAT_26M	1200.1	797	5.3411909	2	Singlet	FAPs
VAT_26M	7221.4	2873	0	0	Singlet	Adipocyte
VAT_26M	2648.6	1472	0	0	Singlet	Adipocyte
VAT_26M	3094	1615	3.5378816	3	Singlet	FAPs
VAT_26M	1219.7	799	0.2203159	0	Singlet	Adipocyte
VAT_26M	1658.6	970	0.7834224	0	Singlet	Adipocyte
VAT_26M	1420.3	922	2.980433	7	Singlet	Endothelial_cells
VAT_26M	1090.3	735	0.8542756	3	Singlet	FAPs
VAT_26M	2449.1	1314	0.0141918	0	Singlet	Adipocyte
VAT_26M	5358.2	2314	0	0	Singlet	Adipocyte
VAT_26M	1554.4	908	0.2760256	0	Singlet	Adipocyte
VAT_26M	2946.6	1616	0.082241	0	Singlet	Adipocyte
VAT_26M	1425.6	878	5.3654218	3	Singlet	FAPs
VAT_26M	2084.9	1191	1.501909	0	Singlet	Adipocyte
VAT_26M	1033.6	687	3.6180583	5	Singlet	Immune_cells
VAT_26M	1252.6	855	0.5235903	0	Singlet	Adipocyte
VAT_26M	1482	951	3.6902259	5	Singlet	Immune_cells
VAT_26M	2260.8	1214	0.0962052	0	Singlet	Adipocyte
VAT_26M	2547.9	1379	0.0679003	0	Singlet	Adipocyte
VAT_26M	2221.8	1305	0.0773245	0	Singlet	Adipocyte
VAT_26M	2288.3	1258	0.1077992	0	Singlet	Adipocyte
VAT_26M	1408	928	0.3726274	0	Singlet	Adipocyte
VAT_26M	1246.9	831	0.5400368	0	Singlet	Adipocyte
VAT_26M	1539.8	881	0	0	Singlet	Adipocyte

VAT_26M	3401.9	1766	0.0286431	0	Singlet	Adipocyte
VAT_26M	1163.4	782	0.046313	0	Singlet	Adipocyte
VAT_26M	1060.6	693	0.0621531	0	Singlet	Adipocyte
VAT_26M	1011.2	720	0.1444648	2	Singlet	FAPs
VAT_26M	1179	743	6.1420779	3	Singlet	FAPs
VAT_26M	2851.2	1466	0	0	Singlet	Adipocyte
VAT_26M	1710.1	994	0.3759701	0	Singlet	Adipocyte
VAT_26M	1135.6	703	0.0546077	0	Singlet	Adipocyte
VAT_26M	2037.1	1203	0.9168484	0	Singlet	Adipocyte
VAT_26M	3749.6	1887	0	0	Singlet	Adipocyte
VAT_26M	1090.4	766	0.0286017	5	Singlet	Immune_cells
VAT_26M	4124.5	2112	0.1121603	0	Singlet	Adipocyte
VAT_26M	1455.9	1008	1.3124815	3	Singlet	FAPs
VAT_26M	1155.9	739	2.0200526	0	Singlet	Adipocyte
VAT_26M	1405	864	0.0273794	0	Singlet	Adipocyte
VAT_26M	4456.4	2086	1.807783	0	Singlet	Adipocyte
VAT_26M	1391.4	834	0.2159257	0	Singlet	Adipocyte
VAT_26M	1581.2	977	5.3047376	2	Singlet	FAPs
VAT_26M	2210.1	1272	3.7226659	3	Singlet	FAPs
VAT_26M	2893.1	1506	0	0	Singlet	Adipocyte
VAT_26M	1024.2	744	0.4531411	5	Singlet	Immune_cells
VAT_26M	1491.3	868	0.0237634	0	Singlet	Adipocyte
VAT_26M	2962.9	1503	0.0825477	0	Singlet	Adipocyte
VAT_26M	2291.1	1239	0.0426534	0	Singlet	Adipocyte
VAT_26M	4411.9	2034	1.8050012	0	Singlet	Adipocyte
VAT_26M	1071.5	735	0	3	Singlet	FAPs
VAT_26M	1424.8	920	0.1610057	0	Singlet	Adipocyte
VAT_26M	1579.7	938	0.0235177	0	Singlet	Adipocyte
VAT_26M	2290.5	1285	0.1065587	0	Singlet	Adipocyte
VAT_26M	1044.2	697	1.6190209	3	Singlet	FAPs
VAT_26M	2300.6	1269	0.0420193	0	Singlet	Adipocyte
VAT_26M	3266.3	1607	0.0255917	0	Singlet	Adipocyte
VAT_26M	2183.1	1188	0.0580471	0	Singlet	Adipocyte
VAT_26M	1645.2	1025	2.7029501	3	Singlet	FAPs
VAT_26M	1504.6	986	0.7958911	3	Singlet	FAPs
VAT_26M	1281.2	861	4.9512824	11	Singlet	Smooth_muscle_cells
VAT_26M	1045.7	717	0.701316	3	Singlet	FAPs
VAT_26M	1070	713	0.0625351	5	Singlet	Immune_cells
VAT_26M	1784.1	1076	0.0679864	0	Singlet	Adipocyte
VAT_26M	2991.7	1533	0	0	Singlet	Adipocyte
VAT_26M	3732.9	1971	7.5543702	9	Singlet	Mesothelial
VAT_26M	1073	775	1.3258102	2	Singlet	FAPs
VAT_26M	1087.2	699	3.5023148	0	Singlet	Adipocyte
VAT_26M	2315.1	1252	4.4462092	0	Singlet	Adipocyte
VAT_26M	3625.1	1779	0.2510891	0	Singlet	Adipocyte
VAT_26M	1221.9	746	0.8771658	0	Singlet	Adipocyte
VAT_26M	2444	1416	0	0	Singlet	Adipocyte
VAT_26M	1391.2	823	5.4237241	0	Singlet	Adipocyte
VAT_26M	1073.7	698	1.4088926	3	Singlet	FAPs
VAT_26M	2440.1	1385	0.0418842	0	Singlet	Adipocyte
VAT_26M	1006.4	710	5.1975059	2	Singlet	FAPs

VAT_26M	1207.9	755	0	0	Singlet	Adipocyte
VAT_26M	1992.1	1204	1.4007871	5	Singlet	Immune_cells
VAT_26M	2353.9	1335	0.0182087	0	Singlet	Adipocyte
VAT_26M	1757.9	1077	0.8611437	0	Singlet	Adipocyte
VAT_26M	1344.2	853	0.0288298	0	Singlet	Adipocyte
VAT_26M	1082.7	672	0.6028018	0	Singlet	Adipocyte
VAT_26M	1101.4	831	0.5552548	5	Singlet	Immune_cells
VAT_26M	2271.1	1277	0	0	Singlet	Adipocyte
VAT_26M	1154.1	779	1.3736358	3	Singlet	FAPs
VAT_26M	1284	888	5.5118188	1	Singlet	Immune_cells
VAT_26M	1160.2	764	6.8689579	11	Singlet	Smooth_muscle_cells
VAT_26M	3867.1	1923	0	0	Singlet	Adipocyte
VAT_26M	1871.5	1126	2.8031697	7	Singlet	Endothelial_cells
VAT_26M	1694.6	965	1.2056704	0	Singlet	Adipocyte
VAT_26M	2455.4	1321	0.0113527	0	Singlet	Adipocyte
VAT_26M	7259.3	2832	1.0844814	0	Singlet	Adipocyte
VAT_26M	1416.4	895	3.1105816	3	Singlet	FAPs
VAT_26M	1641	962	0.366054	0	Singlet	Adipocyte
VAT_26M	1335.1	886	0.8744008	3	Singlet	FAPs
VAT_26M	1180.9	771	0.8643778	0	Singlet	Adipocyte
VAT_26M	1910.9	1148	0	0	Singlet	Adipocyte
VAT_26M	2338.1	1352	0.3182799	0	Singlet	Adipocyte
VAT_26M	2131	1180	0.9892605	0	Singlet	Adipocyte
VAT_26M	1550.3	890	0.4372093	0	Singlet	Adipocyte
VAT_26M	1605.8	970	0	0	Singlet	Adipocyte
VAT_26M	7254.3	3005	0	0	Singlet	Adipocyte
VAT_26M	5556.6	2290	0	0	Singlet	Adipocyte
VAT_26M	1342.4	855	1.5653281	3	Singlet	FAPs
VAT_26M	1364.9	843	0.7468785	3	Singlet	FAPs
VAT_26M	1274.9	854	0.1940629	0	Singlet	Adipocyte
VAT_26M	4354.7	2027	0.0066254	0	Singlet	Adipocyte
VAT_26M	1031.2	713	4.2243188	3	Singlet	FAPs
VAT_26M	1041.1	661	7.5560465	3	Singlet	FAPs
VAT_26M	2933.3	1505	0.0343802	0	Singlet	Adipocyte
VAT_26M	1997	1238	4.495037	5	Singlet	Immune_cells
VAT_26M	1092.8	738	0.5481499	0	Singlet	Adipocyte
VAT_26M	1846.1	1132	0.183566	0	Singlet	Adipocyte
VAT_26M	1179	798	1.3375095	2	Singlet	FAPs
VAT_26M	2458.8	1343	0	0	Singlet	Adipocyte
VAT_26M	1277.4	860	1.8322642	3	Singlet	FAPs
VAT_26M	4302.3	2006	0	0	Singlet	Adipocyte
VAT_26M	1661.6	1032	0.0745	0	Singlet	Adipocyte
VAT_26M	1418.4	1001	0.2457709	2	Singlet	FAPs
VAT_26M	4189.9	1982	0.1312048	0	Singlet	Adipocyte
VAT_26M	1482.7	953	1.7079511	3	Singlet	FAPs
VAT_26M	1264.9	777	0.4455795	0	Singlet	Adipocyte
VAT_26M	1712.2	1082	0.1032441	3	Singlet	FAPs
VAT_26M	5873	2591	0.0168642	0	Singlet	Adipocyte
VAT_26M	4729.1	2122	0.0585005	0	Singlet	Adipocyte
VAT_26M	1086.4	713	1.7522025	2	Singlet	FAPs
VAT_26M	1116.2	744	0.1881223	2	Singlet	FAPs

VAT_26M	2476.6	1403	2.3659979	5	Singlet	Immune_cells
VAT_26M	1664.9	984	0.2074341	0	Singlet	Adipocyte
VAT_26M	1005.4	645	0.7338389	0	Singlet	Adipocyte
VAT_26M	1592.9	1033	2.1819615	3	Singlet	FAPs
VAT_26M	1864.7	1235	0.1017862	5	Singlet	Immune_cells
VAT_26M	1967.1	1171	0.0426308	0	Singlet	Adipocyte
VAT_26M	2978.3	1501	0.0648963	0	Singlet	Adipocyte
VAT_26M	1097.2	772	1.0200534	5	Singlet	Immune_cells
VAT_26M	2725.9	1575	1.2507295	3	Singlet	FAPs
VAT_26M	1935.6	1120	0.0186318	0	Singlet	Adipocyte
VAT_26M	1515.9	972	0.0783796	0	Singlet	Adipocyte
VAT_26M	1226.7	822	1.8494153	0	Singlet	Adipocyte
VAT_26M	1170.9	762	0.1095254	0	Singlet	Adipocyte
VAT_26M	1422.9	930	5.5518214	3	Singlet	FAPs
VAT_26M	1114.7	733	0.2879767	0	Singlet	Adipocyte
VAT_26M	1436.8	925	0.0907089	0	Singlet	Adipocyte
VAT_26M	3890.2	2035	0.2972514	2	Singlet	FAPs
VAT_26M	3989.9	1826	1.6561171	0	Singlet	Adipocyte
VAT_26M	1487	919	0.0949917	0	Singlet	Adipocyte
VAT_26M	1139.6	722	2.109007	0	Singlet	Adipocyte
VAT_26M	1885.8	1161	1.6748971	3	Singlet	FAPs
VAT_26M	3701.2	1942	1.5088491	0	Singlet	Adipocyte
VAT_26M	2381.9	1274	0	0	Singlet	Adipocyte
VAT_26M	1584.8	1022	0.0951684	3	Singlet	FAPs
VAT_26M	2046.1	1329	0.5625859	2	Singlet	FAPs
VAT_26M	3246.8	1709	3.7492199	0	Singlet	Adipocyte
VAT_26M	1764.5	1065	0.9874498	0	Singlet	Adipocyte
VAT_26M	1251.9	789	0.4111512	0	Singlet	Adipocyte
VAT_26M	3080	1521	0	0	Singlet	Adipocyte
VAT_26M	1349.4	834	6.2861635	3	Singlet	FAPs
VAT_26M	2578.6	1408	0.0139647	0	Singlet	Adipocyte
VAT_26M	1528.8	932	0.3681523	0	Singlet	Adipocyte
VAT_26M	1952	1032	0.0179666	0	Singlet	Adipocyte
VAT_26M	1037	735	1.265478	3	Singlet	FAPs
VAT_26M	1214.5	790	0.185848	0	Singlet	Adipocyte
VAT_26M	1177.8	757	0.8363883	3	Singlet	FAPs
VAT_26M	1177.4	803	0	3	Singlet	FAPs
VAT_26M	1571.2	978	0.3994157	0	Singlet	Adipocyte
VAT_26M	1196.5	828	2.7865208	3	Singlet	FAPs
VAT_26M	1043.9	700	10.205492	3	Singlet	FAPs
VAT_26M	1203.5	726	0.6039142	0	Singlet	Adipocyte
VAT_26M	1709.7	972	2.0404336	0	Singlet	Adipocyte
VAT_26M	1069.2	722	0.4936529	1	Singlet	Immune_cells
VAT_26M	3315.2	1664	0.0112006	0	Singlet	Adipocyte
VAT_26M	2006.1	1115	3.4235361	0	Singlet	Adipocyte
VAT_26M	1474	904	0.0639022	0	Singlet	Adipocyte
VAT_26M	1128.7	733	5.7484695	7	Singlet	Endothelial_cells
VAT_26M	1569.2	968	4.4185556	3	Singlet	FAPs
VAT_26M	1328.5	874	0.1988152	0	Singlet	Adipocyte
VAT_26M	1272.4	802	0.0806531	0	Singlet	Adipocyte
VAT_26M	2157.9	1265	0	0	Singlet	Adipocyte

VAT_26M	1744	1078	0.0661271	0	Singlet	Adipocyte
VAT_26M	2461	1289	0.7406778	0	Singlet	Adipocyte
VAT_26M	1630.4	1098	0.4715824	3	Singlet	FAPs
VAT_26M	1199.8	775	4.7342325	0	Singlet	Adipocyte
VAT_26M	1203.8	863	1.5114296	7	Singlet	Endothelial_cells
VAT_26M	2011.3	1195	5.0016716	3	Singlet	FAPs
VAT_26M	1356	812	0.2155662	0	Singlet	Adipocyte
VAT_26M	1332.9	849	10.761887	5	Singlet	Immune_cells
VAT_26M	3000.7	1483	0.0212418	0	Singlet	Adipocyte
VAT_26M	1433.8	872	1.4372439	0	Singlet	Adipocyte
VAT_26M	3305.5	1575	0	0	Singlet	Adipocyte
VAT_26M	2172.8	1286	0.4308363	0	Singlet	Adipocyte
VAT_26M	1610.1	1020	1.6738649	2	Singlet	FAPs
VAT_26M	1723.6	966	0.1082897	0	Singlet	Adipocyte
VAT_26M	2643.9	1477	0.3563048	0	Singlet	Adipocyte
VAT_26M	1808.1	1081	0.1035925	0	Singlet	Adipocyte
VAT_26M	1396.6	906	0.0685441	0	Singlet	Adipocyte
VAT_26M	1859.1	1051	0.4407653	0	Singlet	Adipocyte
VAT_26M	1726.8	1144	2.6095061	4	Singlet	Immune_cells
VAT_26M	1255.3	798	0.7261345	0	Singlet	Adipocyte
VAT_26M	1168.2	805	1.9904244	3	Singlet	FAPs
VAT_26M	1675.1	988	0.8178438	0	Singlet	Adipocyte
VAT_26M	4899.9	2255	0.2086531	0	Singlet	Adipocyte
VAT_26M	1408.6	856	2.2579968	0	Singlet	Adipocyte
VAT_26M	1054.7	729	0.8470224	3	Singlet	FAPs
VAT_26M	2153.4	1234	0.5596257	0	Singlet	Adipocyte
VAT_26M	1865.3	1110	1.0066046	0	Singlet	Adipocyte
VAT_26M	1061.7	682	3.0964189	3	Singlet	FAPs
VAT_26M	1823.4	1025	5.4285963	0	Singlet	Adipocyte
VAT_26M	1795	1083	0.9667891	0	Singlet	Adipocyte
VAT_26M	1011.6	651	2.8814166	0	Singlet	Adipocyte
VAT_26M	1334.2	862	0	3	Singlet	FAPs
VAT_26M	1098.3	697	0.0175878	0	Singlet	Adipocyte
VAT_26M	1763.9	1015	0.0507202	0	Singlet	Adipocyte
VAT_26M	1212.2	823	0.4241192	0	Singlet	Adipocyte
VAT_26M	1533.6	925	1.5475568	0	Singlet	Adipocyte
VAT_26M	1784	1024	0.6025811	0	Singlet	Adipocyte
VAT_26M	1285.3	837	0.3531236	0	Singlet	Adipocyte
VAT_26M	1193.6	704	2.7865536	3	Singlet	FAPs
VAT_26M	1110.9	728	0	0	Singlet	Adipocyte
VAT_26M	1533.4	1007	0.3633719	3	Singlet	FAPs
VAT_26M	8477.1	3174	0	0	Singlet	Adipocyte
VAT_26M	4757.5	2274	0.0133968	0	Singlet	Adipocyte
VAT_26M	1036	718	0.9146913	2	Singlet	FAPs
VAT_26M	1916.5	1133	0	0	Singlet	Adipocyte
VAT_26M	2349.3	1266	0.0514105	0	Singlet	Adipocyte
VAT_26M	1125	783	0.7327929	3	Singlet	FAPs
VAT_26M	1145.5	786	0.9969964	7	Singlet	Endothelial_cells
VAT_26M	1534.2	868	0	0	Singlet	Adipocyte
VAT_26M	1761.8	1011	5.3334823	3	Singlet	FAPs
VAT_26M	1754.5	1055	1.9460613	0	Singlet	Adipocyte

VAT_26M	5957.2	2543	0.0775415	0	Singlet	Adipocyte
VAT_26M	1072.3	710	0.0031064	11	Singlet	Smooth_muscle_cells
VAT_26M	1294.2	901	0.3341259	5	Singlet	Immune_cells
VAT_26M	1849.2	1089	0.2504441	0	Singlet	Adipocyte
VAT_26M	2802.2	1489	0.0373383	0	Singlet	Adipocyte
VAT_26M	1446	830	0.4363762	0	Singlet	Adipocyte
VAT_26M	2721	1523	0.0841513	0	Singlet	Adipocyte
VAT_26M	9442	3200	0.9451669	0	Singlet	Adipocyte
VAT_26M	1251	794	0.169628	0	Singlet	Adipocyte
VAT_26M	2610.1	1468	0.2986973	3	Singlet	FAPs
VAT_26M	1282.7	883	0.2001202	0	Singlet	Adipocyte
VAT_26M	4179.8	1940	3.3718419	0	Singlet	Adipocyte
VAT_26M	1414.3	771	0.0376105	0	Singlet	Adipocyte
VAT_26M	3960	1887	0.1999266	0	Singlet	Adipocyte
VAT_26M	1441.9	966	2.3643	2	Singlet	FAPs
VAT_26M	1720.7	1060	0	0	Singlet	Adipocyte
VAT_26M	1156.9	762	4.0360564	3	Singlet	FAPs
VAT_26M	1348.9	821	0.1157897	0	Singlet	Adipocyte
VAT_26M	1025.3	630	3.2076131	3	Singlet	FAPs
VAT_26M	1123.5	720	0.2775832	0	Singlet	Adipocyte
VAT_26M	1162.7	752	0.3111532	0	Singlet	Adipocyte
VAT_26M	1107.2	701	0	0	Singlet	Adipocyte
VAT_26M	1052.8	706	0.2723277	0	Singlet	Adipocyte
VAT_26M	3002.4	1708	1.3373269	2	Singlet	FAPs
VAT_26M	3057.9	1689	0.2320299	0	Singlet	Adipocyte
VAT_26M	1246.6	794	0.3329573	0	Singlet	Adipocyte
VAT_26M	1301.2	872	0.3090266	3	Singlet	FAPs
VAT_26M	2772.8	1552	0.0284177	0	Singlet	Adipocyte
VAT_26M	2462	1310	0.0051426	0	Singlet	Adipocyte
VAT_26M	1027.5	670	7.270517	3	Singlet	FAPs
VAT_26M	1882.1	1174	0	0	Singlet	Adipocyte
VAT_26M	1725.1	1034	0.0773203	0	Singlet	Adipocyte
VAT_26M	1020.2	659	0.3347035	0	Singlet	Adipocyte
VAT_26M	1527.3	903	0	0	Singlet	Adipocyte
VAT_26M	1640	1007	0.5271645	0	Singlet	Adipocyte
VAT_26M	1039.1	680	0.0443571	0	Singlet	Adipocyte
VAT_26M	2626.7	1498	0.2753395	0	Singlet	Adipocyte
VAT_26M	1152.3	807	2.6876467	3	Singlet	FAPs
VAT_26M	2276.2	1310	0.2243836	0	Singlet	Adipocyte
VAT_26M	2909.4	1448	2.2014334	0	Singlet	Adipocyte
VAT_26M	5055	2317	0.2825235	0	Singlet	Adipocyte
VAT_26M	1275.1	862	2.6335651	0	Singlet	Adipocyte
VAT_26M	1498	940	0	0	Singlet	Adipocyte
VAT_26M	3387.7	1776	0.0708436	0	Singlet	Adipocyte
VAT_26M	1666.2	1094	0.1075166	0	Singlet	Adipocyte
VAT_26M	1619.7	919	0.0967157	0	Singlet	Adipocyte
VAT_26M	1884.5	1140	0.0209741	0	Singlet	Adipocyte
VAT_26M	4053.5	2000	0.3186792	0	Singlet	Adipocyte
VAT_26M	2706.4	1434	0.0840789	0	Singlet	Adipocyte
VAT_26M	1361.3	1050	0.1604537	6	Singlet	Neurons
VAT_26M	1848.4	1117	5.2131031	3	Singlet	FAPs

VAT_26M	2041.3	1207	0.0277544	0	Singlet	Adipocyte
VAT_26M	1606.2	910	0.0289875	0	Singlet	Adipocyte
VAT_26M	4238.4	2012	0.0115422	0	Singlet	Adipocyte
VAT_26M	1221.5	835	1.1130092	3	Singlet	FAPs
VAT_26M	1371.3	871	0	0	Singlet	Adipocyte
VAT_26M	1352.6	895	1.5731473	0	Singlet	Adipocyte
VAT_26M	2394.5	1339	0	0	Singlet	Adipocyte
VAT_26M	3335.8	1766	0.0136479	0	Singlet	Adipocyte
VAT_26M	2605.5	1454	2.5390021	0	Singlet	Adipocyte
VAT_26M	3963.4	2025	0.4200617	2	Singlet	FAPs
VAT_26M	1820.2	1145	0.0551499	0	Singlet	Adipocyte
VAT_26M	2903.1	1676	0.1143452	2	Singlet	FAPs
VAT_26M	1245.6	791	0.5268645	0	Singlet	Adipocyte
VAT_26M	3581.9	1738	0	0	Singlet	Adipocyte
VAT_26M	2105.4	1232	0.0690254	0	Singlet	Adipocyte
VAT_26M	1518.1	997	1.8857907	3	Singlet	FAPs
VAT_26M	1278.3	837	1.4872197	0	Singlet	Adipocyte
VAT_26M	1353.3	830	2.1513076	7	Singlet	Endothelial_cells
VAT_26M	3487.7	1770	0.0626163	0	Singlet	Adipocyte
VAT_26M	4861.4	2180	1.3757569	0	Singlet	Adipocyte
VAT_26M	1106.8	707	0.3005169	0	Singlet	Adipocyte
VAT_26M	1318.3	842	0.3428299	0	Singlet	Adipocyte
VAT_26M	1133.3	705	0.0443546	0	Singlet	Adipocyte
VAT_26M	3038.6	1771	0.1783792	2	Singlet	FAPs
VAT_26M	1242.9	822	3.8242261	2	Singlet	FAPs
VAT_26M	1321.7	830	0.0965609	0	Singlet	Adipocyte
VAT_26M	3671.3	1819	0.1714459	0	Singlet	Adipocyte
VAT_26M	1047.9	672	0.7703707	0	Singlet	Adipocyte
VAT_26M	1216.3	910	0.3329865	5	Singlet	Immune_cells
VAT_26M	1527.3	954	0.0216056	0	Singlet	Adipocyte
VAT_26M	4019.4	1983	0.0458695	0	Singlet	Adipocyte
VAT_26M	2761.6	1610	6.6671229	2	Singlet	FAPs
VAT_26M	1644.7	1000	0.1046402	0	Singlet	Adipocyte
VAT_26M	1828.7	1049	0.1772666	0	Singlet	Adipocyte
VAT_26M	1196.3	742	0.5770119	3	Singlet	FAPs
VAT_26M	1214.9	739	0.4473131	0	Singlet	Adipocyte
VAT_26M	1980.1	1143	2.4285985	0	Singlet	Adipocyte
VAT_26M	1018.2	685	2.4073205	7	Singlet	Endothelial_cells
VAT_26M	3041.8	1511	0.0325161	0	Singlet	Adipocyte
VAT_26M	1484	956	0.6759367	3	Singlet	FAPs
VAT_26M	3970.3	2009	0.2757005	0	Singlet	Adipocyte
VAT_26M	2155.5	1222	0.067549	0	Singlet	Adipocyte
VAT_26M	1135.3	698	1.7978148	0	Singlet	Adipocyte
VAT_26M	5720.1	2561	1.7315744	0	Singlet	Adipocyte
VAT_26M	5688	2526	0.5661082	0	Singlet	Adipocyte
VAT_26M	1200.6	785	8.1118731	8	Singlet	Mesothelial
VAT_26M	2737.8	1526	0.3252074	0	Singlet	Adipocyte
VAT_26M	1995.3	1150	0.1111837	0	Singlet	Adipocyte
VAT_26M	1112.8	721	4.310326	7	Singlet	Endothelial_cells
VAT_26M	1516	961	1.3310599	5	Singlet	Immune_cells
VAT_26M	1153.2	736	1.9489886	0	Singlet	Adipocyte

VAT_26M	1465.1	869	0.2971379	0	Singlet	Adipocyte
VAT_26M	6239.1	2689	0.2753029	0	Singlet	Adipocyte
VAT_26M	3449.3	1785	0.0564733	0	Singlet	Adipocyte
VAT_26M	1143.4	715	0.5974558	0	Singlet	Adipocyte
VAT_26M	3068.6	1755	0.6133793	0	Singlet	Adipocyte
VAT_26M	1099.4	835	1.1120187	4	Singlet	Immune_cells
VAT_26M	2244.7	1296	0	0	Singlet	Adipocyte
VAT_26M	1522.2	1006	3.0002721	3	Singlet	FAPs
VAT_26M	2257	1224	0.1380904	0	Singlet	Adipocyte
VAT_26M	1322.2	919	0.1588339	7	Singlet	Endothelial_cells
VAT_26M	1248.8	754	0	0	Singlet	Adipocyte
VAT_26M	3615.3	1821	0	0	Singlet	Adipocyte
VAT_26M	2948.9	1498	0.0222553	0	Singlet	Adipocyte
VAT_26M	3643.3	1761	0.0316527	0	Singlet	Adipocyte
VAT_26M	4589.9	2038	0.0100779	0	Singlet	Adipocyte
VAT_26M	1357.4	830	8.3255829	7	Singlet	Endothelial_cells
VAT_26M	2316.5	1372	0.3205003	0	Singlet	Adipocyte
VAT_26M	2062.3	1151	0.1493558	0	Singlet	Adipocyte
VAT_26M	1277.7	839	8.49249	5	Singlet	Immune_cells
VAT_26M	2851.8	1539	0.0121415	0	Singlet	Adipocyte
VAT_26M	2503.6	1307	0.0360595	0	Singlet	Adipocyte
VAT_26M	4896.8	2130	0.0282275	0	Singlet	Adipocyte
VAT_26M	1761.8	1103	1.4333298	0	Singlet	Adipocyte
VAT_26M	1507.2	1041	1.8659356	7	Singlet	Endothelial_cells
VAT_26M	3311.8	1754	0.0745459	0	Singlet	Adipocyte
VAT_26M	2115.7	1218	0.1157569	0	Singlet	Adipocyte
VAT_26M	2736.7	1542	7.2878425	5	Singlet	Immune_cells
VAT_26M	1363.6	922	1.348504	0	Singlet	Adipocyte
VAT_26M	6123.7	2375	0	0	Singlet	Adipocyte
VAT_26M	1506.5	987	0.2178089	5	Singlet	Immune_cells
VAT_26M	1595.3	906	1.1536548	0	Singlet	Adipocyte
VAT_26M	1873.1	1101	0.1302035	0	Singlet	Adipocyte
VAT_26M	1491.1	952	9.615792	5	Singlet	Immune_cells
VAT_26M	4333.3	1940	0	0	Singlet	Adipocyte
VAT_26M	2548.3	1405	0.0480199	0	Singlet	Adipocyte
VAT_26M	2974.3	1612	0	0	Singlet	Adipocyte
VAT_26M	1299.7	817	4.9260404	7	Singlet	Endothelial_cells
VAT_26M	2298.4	1268	1.2123754	0	Singlet	Adipocyte
VAT_26M	1013.8	681	4.3207633	3	Singlet	FAPs
VAT_26M	1425.4	875	1.2532458	3	Singlet	FAPs
VAT_26M	1203	737	6.0902573	3	Singlet	FAPs
VAT_26M	1299.6	825	1.508774	0	Singlet	Adipocyte
VAT_26M	7007.1	2976	0.0076347	0	Singlet	Adipocyte
VAT_26M	1198.2	745	6.6909852	3	Singlet	FAPs
VAT_26M	6457.1	2464	0.9828773	0	Singlet	Adipocyte
VAT_26M	1577.5	1046	1.7949238	2	Singlet	FAPs
VAT_26M	1516.7	873	8.7715662	7	Singlet	Endothelial_cells
VAT_26M	1974.5	1212	5.0186326	3	Singlet	FAPs
VAT_26M	1867.5	1179	0.0286839	5	Singlet	Immune_cells
VAT_26M	2317.4	1280	0	0	Singlet	Adipocyte
VAT_26M	1004.3	668	6.4474325	3	Singlet	FAPs

VAT_26M	2233.8	1367	0.3212596	0	Singlet	Adipocyte
VAT_26M	1780.6	1124	0.1043981	0	Singlet	Adipocyte
VAT_26M	1703.5	1035	0.1013626	0	Singlet	Adipocyte
VAT_26M	1553	911	2.0620084	3	Singlet	FAPs
VAT_26M	1425.1	870	0.3867629	0	Singlet	Adipocyte
VAT_26M	1569.9	851	8.5334743	0	Singlet	Adipocyte
VAT_26M	1067.5	682	1.2720094	0	Singlet	Adipocyte
VAT_26M	1921.3	1144	4.581907	3	Singlet	FAPs
VAT_26M	3132	1617	0	0	Singlet	Adipocyte
VAT_26M	1021	673	0.5359149	0	Singlet	Adipocyte
VAT_26M	1021	760	2.6797421	3	Singlet	FAPs
VAT_26M	1895.8	1225	0.3905799	3	Singlet	FAPs
VAT_26M	1727	1027	0.1668919	0	Singlet	Adipocyte
VAT_26M	1045.9	706	9.8246539	7	Singlet	Endothelial_cells
VAT_26M	3016.6	1540	0.0321941	0	Singlet	Adipocyte
VAT_26M	1203	813	0.8935257	3	Singlet	FAPs
VAT_26M	1135.5	704	2.2969164	0	Singlet	Adipocyte
VAT_26M	2308.3	1269	0.0105842	0	Singlet	Adipocyte
VAT_26M	1005.8	655	0	3	Singlet	FAPs
VAT_26M	1803.4	1107	7.0568039	3	Singlet	FAPs
VAT_26M	1509.1	938	0.5237564	2	Singlet	FAPs
VAT_26M	1133.5	777	0.6532487	2	Singlet	FAPs
VAT_26M	1676.7	1078	0.2955916	3	Singlet	FAPs
VAT_26M	2174.6	1223	0.0217431	0	Singlet	Adipocyte
VAT_26M	1240.9	860	0.0603915	3	Singlet	FAPs
VAT_26M	2208.5	1283	3.7100086	0	Singlet	Adipocyte
VAT_26M	1925.8	1064	0.0258827	0	Singlet	Adipocyte
VAT_26M	1595.3	943	0.7635467	0	Singlet	Adipocyte
VAT_26M	1002.3	689	0	5	Singlet	Immune_cells
VAT_26M	1376.4	824	0.7653088	3	Singlet	FAPs
VAT_26M	1395	852	0.1407686	0	Singlet	Adipocyte
VAT_26M	1577.7	917	6.3946925	3	Singlet	FAPs
VAT_26M	1444.3	889	0.4283516	0	Singlet	Adipocyte
VAT_26M	1896.2	1194	0.2588721	2	Singlet	FAPs
VAT_26M	4733.3	2009	0.8190499	0	Singlet	Adipocyte
VAT_26M	1276.5	847	0.4512596	0	Singlet	Adipocyte
VAT_26M	3351.7	1734	0.066581	0	Singlet	Adipocyte
VAT_26M	2121.8	1141	11.898535	5	Singlet	Immune_cells
VAT_26M	1797.5	1094	9.7382763	7	Singlet	Endothelial_cells
VAT_26M	1010.3	734	1.3970652	3	Singlet	FAPs
VAT_26M	2754.5	1436	0.03651	0	Singlet	Adipocyte
VAT_26M	1610.9	1061	0.3306543	2	Singlet	FAPs
VAT_26M	1006	688	0.3315961	0	Singlet	Adipocyte
VAT_26M	1501.7	924	0.4890954	0	Singlet	Adipocyte
VAT_26M	1064.3	703	0.3881326	2	Singlet	FAPs
VAT_26M	1106.3	768	0.3190942	3	Singlet	FAPs
VAT_26M	1489.5	937	0.022226	0	Singlet	Adipocyte
VAT_26M	1196.7	749	0.1447751	0	Singlet	Adipocyte
VAT_26M	5083.5	2337	0.0032865	0	Singlet	Adipocyte
VAT_26M	2173.4	1215	0.107477	0	Singlet	Adipocyte
VAT_26M	3171.7	1580	0	0	Singlet	Adipocyte

VAT_26M	4231.6	2053	0	0	Singlet	Adipocyte
VAT_26M	3297	1690	0.0288896	0	Singlet	Adipocyte
VAT_26M	2227.6	1318	1.174338	0	Singlet	Adipocyte
VAT_26M	1081.7	712	0	0	Singlet	Adipocyte
VAT_26M	2606.7	1424	0.1465289	0	Singlet	Adipocyte
VAT_26M	2074.8	1186	0.0259159	0	Singlet	Adipocyte
VAT_26M	1746.1	1071	0.0755049	0	Singlet	Adipocyte
VAT_26M	1114.1	756	4.7348629	3	Singlet	FAPs
VAT_26M	1194.6	797	0.2219429	0	Singlet	Adipocyte
VAT_26M	1065.1	721	8.7041033	5	Singlet	Immune_cells
VAT_26M	1755.4	1070	0.3579713	0	Singlet	Adipocyte
VAT_26M	1733.8	1099	0.8963084	0	Singlet	Adipocyte
VAT_26M	1556.1	966	0.6066163	0	Singlet	Adipocyte
VAT_26M	6002.8	2570	0.0153861	0	Singlet	Adipocyte
VAT_26M	2044.9	1166	0.070491	0	Singlet	Adipocyte
VAT_26M	2002.9	1156	0.1645647	0	Singlet	Adipocyte
VAT_26M	1929.3	1112	0.0736657	0	Singlet	Adipocyte
VAT_26M	2638.1	1409	0.1490019	0	Singlet	Adipocyte
VAT_26M	2116.7	1129	0.3071529	0	Singlet	Adipocyte
VAT_26M	1307.1	944	0.0403866	5	Singlet	Immune_cells
VAT_26M	1188.2	795	0.0107536	0	Singlet	Adipocyte
VAT_26M	1063.2	722	2.9671849	3	Singlet	FAPs
VAT_26M	1227.2	793	0.1032138	0	Singlet	Adipocyte
VAT_26M	1535.8	956	0.1222191	0	Singlet	Adipocyte
VAT_26M	2295.3	1236	1.68E-05	0	Singlet	Adipocyte
VAT_26M	1068.8	707	0.1045473	0	Singlet	Adipocyte
VAT_26M	1116.3	759	5.4099456	3	Singlet	FAPs
VAT_26M	5027	2174	0	0	Singlet	Adipocyte
VAT_26M	1510.2	956	0.0139629	0	Singlet	Adipocyte
VAT_26M	1219.5	803	0.9106343	6	Singlet	Neurons
VAT_26M	2223.8	1247	0.3521882	0	Singlet	Adipocyte
VAT_26M	1170.1	770	0.0408267	0	Singlet	Adipocyte
VAT_26M	1680.9	1140	0.7289639	5	Singlet	Immune_cells
VAT_26M	1659.8	991	0.243823	0	Singlet	Adipocyte
VAT_26M	1686	1016	0.0311414	0	Singlet	Adipocyte
VAT_26M	1940.8	1124	0.2739546	0	Singlet	Adipocyte
VAT_26M	1352.8	813	0.1441455	0	Singlet	Adipocyte
VAT_26M	1694.9	1030	0.2242024	0	Singlet	Adipocyte
VAT_26M	1292.3	906	2.3442315	0	Singlet	Adipocyte
VAT_26M	1253.2	806	0.0461548	0	Singlet	Adipocyte
VAT_26M	2200.1	1218	0.0935965	0	Singlet	Adipocyte
VAT_26M	2463.6	1336	0.1947519	0	Singlet	Adipocyte
VAT_26M	5279.6	2324	0.0171851	0	Singlet	Adipocyte
VAT_26M	1015.3	673	0.2487307	0	Singlet	Adipocyte
VAT_26M	2748.3	1510	0.1045838	0	Singlet	Adipocyte
VAT_26M	1061.9	700	2.0574463	5	Singlet	Immune_cells
VAT_26M	1358.7	773	0	0	Singlet	Adipocyte
VAT_26M	1410.3	937	2.2547325	5	Singlet	Immune_cells
VAT_26M	4040.5	1853	0.0851934	0	Singlet	Adipocyte
VAT_26M	1014.9	685	1.0622171	3	Singlet	FAPs
VAT_26M	2639.5	1564	0.9375521	0	Singlet	Adipocyte

VAT_26M	2363.7	1338	1.1019844	0	Singlet	Adipocyte
VAT_26M	2000.3	1066	0.0172765	0	Singlet	Adipocyte
VAT_26M	1122.1	769	0.1172808	3	Singlet	FAPs
VAT_26M	1339.4	876	0.4978629	0	Singlet	Adipocyte
VAT_26M	4731	2209	0.2738005	0	Singlet	Adipocyte
VAT_26M	1001.9	687	10.300883	2	Singlet	FAPs
VAT_26M	2246.6	1280	0.4068101	0	Singlet	Adipocyte
VAT_26M	2988.3	1547	0.0052129	0	Singlet	Adipocyte
VAT_26M	1504.8	944	0	0	Singlet	Adipocyte
VAT_26M	1737.6	1040	0.1065312	0	Singlet	Adipocyte
VAT_26M	2320.2	1371	3.0525049	0	Singlet	Adipocyte
VAT_26M	1071.3	690	0.0683137	0	Singlet	Adipocyte
VAT_26M	1763	1031	0.4992655	0	Singlet	Adipocyte
VAT_26M	1286.6	831	0.3454355	0	Singlet	Adipocyte
VAT_26M	4585.3	2452	2.2459449	7	Singlet	Endothelial_cells
VAT_26M	1227.6	768	0.4093125	0	Singlet	Adipocyte
VAT_26M	1870.9	984	0.0846063	0	Singlet	Adipocyte
VAT_26M	1253.1	776	0	0	Singlet	Adipocyte
VAT_26M	1768.5	1123	2.4015818	7	Singlet	Endothelial_cells
VAT_26M	1089.7	693	0	3	Singlet	FAPs
VAT_26M	3932.7	2030	0	0	Singlet	Adipocyte
VAT_26M	3119	1639	1.0827143	0	Singlet	Adipocyte
VAT_26M	1778.6	1060	0.0212198	0	Singlet	Adipocyte
VAT_26M	2997.7	1775	1.1173568	2	Singlet	FAPs
VAT_26M	2089.5	1341	3.8148828	2	Singlet	FAPs
VAT_26M	4089.8	1952	0	0	Singlet	Adipocyte
VAT_26M	1502.1	901	0.7928174	0	Singlet	Adipocyte
VAT_26M	1442.9	968	0.9771639	3	Singlet	FAPs
VAT_26M	1905.3	1112	0	0	Singlet	Adipocyte
VAT_26M	2563.5	1495	0	0	Singlet	Adipocyte
VAT_26M	2608.8	1247	0.1468038	0	Singlet	Adipocyte
VAT_26M	1780.3	1059	6.7243269	5	Singlet	Immune_cells
VAT_26M	2450	1376	0.199679	0	Singlet	Adipocyte
VAT_26M	1220.9	822	2.0482559	10	Singlet	Immune_cells
VAT_26M	2377.1	1287	0.009333	0	Singlet	Adipocyte
VAT_26M	1838.2	1116	1.2018973	0	Singlet	Adipocyte
VAT_26M	1237.8	839	0	2	Singlet	FAPs
VAT_26M	1006.9	648	0.2744541	0	Singlet	Adipocyte
VAT_26M	1335.1	837	4.6354114	3	Singlet	FAPs
VAT_26M	2793.3	1506	0.1317291	0	Singlet	Adipocyte
VAT_26M	1560.6	874	0	0	Singlet	Adipocyte
VAT_26M	4750.7	2125	0.1564607	0	Singlet	Adipocyte
VAT_26M	2019.3	1212	0.3504078	0	Singlet	Adipocyte
VAT_26M	1510.1	926	4.7405725	7	Singlet	Endothelial_cells
VAT_26M	4835.2	2252	0	0	Singlet	Adipocyte
VAT_26M	1896.6	1077	0.2970752	0	Singlet	Adipocyte
VAT_26M	3107.2	1733	2.7878546	0	Singlet	Adipocyte
VAT_26M	1164.3	738	0	0	Singlet	Adipocyte
VAT_26M	3849.5	1925	0.0818934	0	Singlet	Adipocyte
VAT_26M	1090.3	733	2.006597	2	Singlet	FAPs
VAT_26M	2339.2	1310	0	0	Singlet	Adipocyte

VAT_26M	1189.4	782	0.9382473	0	Singlet	Adipocyte
VAT_26M	1299.4	851	0.2003946	0	Singlet	Adipocyte
VAT_26M	2610.4	1496	4.9014234	3	Singlet	FAPs
VAT_26M	1241.5	849	0.1777196	0	Singlet	Adipocyte
VAT_26M	3247.4	1574	0	0	Singlet	Adipocyte
VAT_26M	2086.4	1177	0.0126506	0	Singlet	Adipocyte
VAT_26M	1637.4	999	0.0886959	0	Singlet	Adipocyte
VAT_26M	1128.7	747	0.2047849	7	Singlet	Endothelial_cells
VAT_26M	1765.6	1056	1.1010039	0	Singlet	Adipocyte
VAT_26M	1644.4	1001	0.096005	0	Singlet	Adipocyte
VAT_26M	1219.2	770	1.2851008	0	Singlet	Adipocyte
VAT_26M	1038.7	654	0.1377633	0	Singlet	Adipocyte
VAT_26M	1064	733	3.2024162	2	Singlet	FAPs
VAT_26M	1824.2	1102	0.3345775	0	Singlet	Adipocyte
VAT_26M	1713.1	985	0.3914391	0	Singlet	Adipocyte
VAT_26M	5116.4	2298	0.1095533	0	Singlet	Adipocyte
VAT_26M	4031.4	1853	0.0881714	0	Singlet	Adipocyte
VAT_26M	1191.1	780	0.1007281	0	Singlet	Adipocyte
VAT_26M	4054.6	1849	0.3424653	0	Singlet	Adipocyte
VAT_26M	1123.9	775	0.8929638	2	Singlet	FAPs
VAT_26M	1507.7	918	0.0891929	0	Singlet	Adipocyte
VAT_26M	1862.7	1103	0.0626178	0	Singlet	Adipocyte
VAT_26M	1470.5	980	5.1459399	3	Singlet	FAPs
VAT_26M	1701.8	1028	0.9606281	0	Singlet	Adipocyte
VAT_26M	2401.7	1320	0.0510205	0	Singlet	Adipocyte
VAT_26M	1754.6	1041	0	0	Singlet	Adipocyte
VAT_26M	2720.2	1561	0.0978542	0	Singlet	Adipocyte
VAT_26M	1489.3	1015	1.2939161	3	Singlet	FAPs
VAT_26M	1058	755	0.5591819	0	Singlet	Adipocyte
VAT_26M	1882	1131	0.8441013	0	Singlet	Adipocyte
VAT_26M	1082.8	773	6.0872798	3	Singlet	FAPs
VAT_26M	1968.9	1127	0	0	Singlet	Adipocyte
VAT_26M	1041.1	686	0.1425441	0	Singlet	Adipocyte
VAT_26M	2587.4	1414	0.0287614	0	Singlet	Adipocyte
VAT_26M	1378.9	858	0.9697182	0	Singlet	Adipocyte
VAT_26M	2023.9	1233	2.704239	0	Singlet	Adipocyte
VAT_26M	2423.6	1369	0.0085494	0	Singlet	Adipocyte
VAT_26M	3092.1	1900	2.1387599	3	Singlet	FAPs
VAT_26M	1563.8	990	0.1861617	0	Singlet	Adipocyte
VAT_26M	1156.9	736	0.0530952	0	Singlet	Adipocyte
VAT_26M	1390.8	827	7.5343837	3	Singlet	FAPs
VAT_26M	1391.1	887	6.7280831	3	Singlet	FAPs
VAT_26M	1309.5	907	0.0462564	5	Singlet	Immune_cells
VAT_26M	2380.2	1407	0.3693027	0	Singlet	Adipocyte
VAT_26M	2882.3	1483	0.2850069	0	Singlet	Adipocyte
[truncated: 493,972 more chars]
